# The Abundant Phytocannabinoids in Rheumatoid Arthritis: Therapeutic Targets and Molecular Processes Identified Using Integrated Bioinformatics and Network Pharmacology

**DOI:** 10.3390/life13030700

**Published:** 2023-03-05

**Authors:** Arijit Nandi, Anwesha Das, Yadu Nandan Dey, Kuldeep K. Roy

**Affiliations:** 1 Department of Pharmacology, Dr. B.C. Roy College of Pharmacy and Allied Health Sciences, Durgapur 713206, West Bengal, India; 2 Department of Medicinal Chemistry, National Institute of Pharmaceutical Education and Research, Ahmedabad, Palaj, Gandhinagar 382355, Gujarat, India; 3 Department of Pharmaceutical Sciences, School of Health Sciences and Technology, UPES University, Dehradun 248007, Uttarakhand, India

**Keywords:** cannabis, *Cannabis sativa*, endocannabinoid system, inflammation, network pharmacology

## Abstract

The endocannabinoid system consists of several phytocannabinoids, cannabinoid receptors, and enzymes that aid in numerous steps necessary to manifest any pharmacological activity. It is well known that the endocannabinoid system inhibits the pathogenesis of the inflammatory and autoimmune disease rheumatoid arthritis (RA). To the best of our knowledge, no research has been done that explains the network-pharmacology-based anti-rheumatic processes by focusing on the endocannabinoid system. Therefore, the purpose of this study is to further our understanding of the signaling pathways, associated proteins, and genes underlying RA based on the abundant natural endocannabinoids. The knowledge on how the phytocannabinoids in *Cannabis sativa* affect the endocannabinoid system was gathered from the literature. SwissTarget prediction and BindingDB databases were used to anticipate the targets for the phytocannabinoids. The genes related to RA were retrieved from the DisGeNET and GeneCards databases. Protein–protein interactions (high confidence > 0.7) were carried out with the aid of the string web server and displayed using Cytoscape. The Kyoto Encyclopedia of Genes and Genomes (KEGG) metabolic pathway analysis was used to perform enrichment analyses on the endocannabinoid–RA common targets. ShinyGO 0.76 was used to predict the biological processes listed in the Gene Ontology (GO) classification system. The binding affinity between the ligand and the receptors was precisely understood using molecular docking, induced-fit docking, and a molecular dynamics simulation. The network pharmacology analyses predicted that processes like response to oxygen-containing compounds and peptodyl-amino acid modification are related to the potential mechanisms of treatment for RA. These biological actions are coordinated by cancer, neuroactive ligand–receptor interaction, lipids and atherosclerosis, the calcium signaling pathway, and the Rap1 signaling pathway. According to the results of molecular docking, in the context of RA, phytocannabinoids may bind to important target proteins such PIK3CA, AKT1, MAPK9, PRKCD, BRAF, IGF1R, and NOS3. This entire study predicted the phytocannabinoids’ systemic biological characteristics. Future experimental research is needed, however, to confirm the results so far.

## 1. Introduction

Although inflammation is a typical reaction to tissue damage, it can become out of control and cause further problems. Specific soluble pro-inflammatory mediators, including cytokines or chemokines, prostaglandins, and leukotrienes, are produced as a result of an inflammatory reaction [1]. Many acute and chronic disorders are made worse by persistent inflammation. Rheumatoid arthritis (RA) is just one of the many chronic inflammatory disorders that continues to be a major global health issue. One to two percent of the world’s population suffers from the chronic inflammatory disease known as arthritis. It is characterized by immune-mediated inflammatory sinusitis, which causes hyperplasia, vasculogenesis, pannus formation, cartilage and bone destruction, joint malformation, and functional impairment [2]. Non-steroidal anti-inflammatory drugs (NSAIDs) are frequently used to treat the unpleasant inflammatory diseases, either systemically or by applying topical versions locally. The most prevalent side effects of systemic medications, notwithstanding their effectiveness, are gastrointestinal bleeding and peptic ulcers [3], whereas topical medications simply treat symptoms and do not cure the condition. Therefore, it is necessary to research and validate plant-based/herbal anti-inflammatory drugs from traditional and complementary systems of medicine that have been used clinically for decades and are thought to be safe with few to no side effects.

The endocannabinoid system consists of the numerous phytocannabinoids derived from *Cannabis sativa* (family: Cannabaceae) as well as cannabinoid receptors and enzymes that aid in several steps to exhibit any pharmacological activity [4,5,6]. It is well known that the endocannabinoid system inhibits the pathogenesis of the inflammatory and autoimmune disease rheumatoid arthritis. For ages, people have used *Cannabis sativa* (family: Cannabaceae) as a traditional medication for entheogenic and recreational uses. *Cannabis sativa*, however, may be useful in the treatment of pain, dysmenorrhea, muscle spasms, anorexia, etc., according to earlier research [7,8]. Recently, the tale of these illegal narcotics being used as herbal medicine has grown increasingly intriguing. California and various countries have allowed the use of chemotherapy-diversified phytocannabinoids due to their medical benefits [9]. Furthermore, Canada approved the use of phytocannabinoids for medical and recreational uses in the years 1999 and 2018, respectively [10]. Cannabinoids consist of several phytocannabinoids derived from *Cannabis sativa* [11]. The most prevalent components that have psychoactive properties are tetrahydrocannabinoids (THC) [12]. For a long time, isoprenylated resorcinyl polypeptides known as cannabinoids have been utilized as medicines. Previous research established that various phytocannabinoids target the peroxisome proliferator-activated receptor γ (PPARγ), transient receptor potential ion channels, and protein-coupled cannabinoid receptors (CB1 and CB2). The anti-arthritic properties of phytocannabinoids are due to their effects on the CB1 and CB2 cannabinoid receptors. However, the phytocannabinoids’ therapeutic situation is far more complicated and involves several targets. The presence of a dibenzopyran ring with a hydrophobic alkyl chain is a typical structural characteristic of the identified phytocannabinoids [13,14].

RA is a condition with several diagnoses that is primarily characterized by chronic synovial inflammation. The etiology of this illness involves the infiltration of inflammatory cells, the growth of synoviocytes that resemble fibroblasts, elevated levels of proinflammatory cytokines, and matrix metalloproteinases. These substances encourage angiogenesis and osteoclast activity, which damage joints by altering the development of bone and cartilage [15]. The endocannabinoid system negatively controls immunological reactions and inflammation associated with RA. Negative side effects could result from continued use of these drugs. The endocannabinoid system is a viable pharmacological target for the treatment of RA, hence the treatment with such medications needs to be clarified [16]. The network pharmacology technique offers a platform for predicting the molecular mechanisms of the multi-components present in traditional herbal formulations. Selective molecules may have poorer clinical effectiveness than multi-target medications due to phenotypic resilience and network topology [17]. Many herbal formulations have been analyzed for the probable mechanism in a disease through this approach [18,19,20]. Therefore, using combined bioinformatics and network pharmacology methodologies, the goal of this study is to further understand the signaling pathways, associated proteins, and genes that cause rheumatoid arthritis based on the plentiful naturally occurring phytocannabinoids.

## 2. Materials and Methods

### 2.1. Identification of Bioactive Compounds and Targets

The abundant phytocannabinoids of *Cannabis sativa* used in this investigation were derived according to reports by Morales et al. [14]. In addition, the canonical forms and 2D structures of the phytocannabinoids were retrieved from the PubChem database (https://pubchem.ncbi.nlm.nih.gov/, accessed on 1 December 2022). The targets of the phytocannabinoids were found using the web servers Swiss Target Prediction and BindingDB (https://www.bindingdb.org/bind/index.jsp, accessed on 1 December 2022). In addition, the canonical forms and 2D structures of the phytocannabinoids were retrieved from the PubChem database (https://pubchem.ncbi.nlm.nih.gov, accessed on 1 December 2022). The RA targets were derived from two databases: DisGeNET (www.disgenet.org, accessed on 1 December 2022) and GeneCards (www.genecards.org, accessed on 1 December 2022). The UniProt database (www.uniprot.org, accessed on 1 December 2022) was utilized to standardize the target names into official gene symbols. The keywords “Rheumatoid arthritis” and the organism “Homo sapiens” were used to seek the targets, which are verified by UniProt ID. To obtain the Venn diagram of the shared targets, Venny2.1.0 (https://bioinfogp.cnb.csic.es/tools/venny/index.html, accessed on 1 December 2022) was used.

### 2.2. Collection of Protein–Protein Interaction (PPI) Data

The chosen targets were added to the STRING 11.0 database (available at https://string-db.org, accessed on 1 December 2022) to create a PPI network using “Homo sapiens” as the species and “highest confidence > 0.7” as the minimal interaction threshold. Additionally, the detached nodes were kept a secret. Cytoscape software was used to visualize these networks. To identify PPI subnetworks and clusters with a high degree of interconnection within the PPI network, we employed the Molecular Complex Detection (MCODE) method (version 1.5.1). As threshold settings, we set the Cytoscape plug-in MCODE’s maximum depth to 100, node score at 0.2, and K-score to 2 [21].

### 2.3. GO and KEGG Enrichment Analyses

The KEGG metabolic pathway analysis was used to perform enrichment analyses on *C. sativa* and RA mutual targets, and ShinyGO 0.76 (available at http://bioinformatics.sdstate.edu/go76/, accessed on 1 December 2022) was used to predict Gene Ontology (GO) biological processes. The top 20 biological pathways and processes (False Discovery Rate cut-off of 0.05) were selected based on fold enrichment, and they were then displayed as bubble charts with the adjusted *p*-values, fold enrichment, gene counts, etc. The GO analysis predicted the various biological processes, cellular components, and molecular functions. A network was constructed using the key pathways obtained from the KEGG analysis for the discovery of the important target proteins that play a significant role in the treatment effects of *C. sativa* [22]. For the purpose of predicting the likely mechanism of action of the bioactive components of *C. sativa* in RA, all the information on the important targets and associated pathways was gathered through online software.

### 2.4. Molecular Docking Validation

Molecular docking studies of 13 phytocannabinoids were done using Glide XP (extra-precision) implemented in the Schrödinger suite. The energy-minimized structures of the ligands were docked with the crystal structure of PIK3CA (PDB ID: 4L23, resolution of 2.50 Å), AKT1 ((Gene: Akt1) (AlphFold)), MAPK9 ((Gene: MAPK9) (AlphFold)), PRKCD ((Gene: PRKCD) (AlphFold)), BRAF (PDB ID: 6P3D), IGF1R (PDB ID: 5FXS), and NOS3 (PDB ID: 6NH5) with validated grid parameters [23]. The RCSB protein data bank (PDB) was used to download the X-ray crystal coordinates of different proteins. From the AlphaFold, the predicted structures of the proteins AKT1, MAPK9, and PRKCD were obtained. We used the protein preparation wizard to prepare the proteins. The water molecules that lacked three hydrogen bonds were eliminated. Then, considering the correct ionization states for both basic and acidic amino acid residues, hydrogen bonds corresponding to pH 7 were added. The force field OPLS3 (Optimized Potentials for Liquid Simulations 3) was used to minimize the energy of the protein 3D structure. The optimum protein binding site was identified using Sitemap analysis for proteins predicted by AlphaFold. Additionally, the best binding site’s center was chosen to generate the grid box. The centroid of each of the co-crystallized ligands for PIK3CA, BRAF, IGF1R, and NOS3 (X6K, 0LI, OZN, and KLD, respectively) was chosen to create the grid box. Finally, docking of all low-energy conformations into the Sitemap-predicted site was done using Glide in XP mode in the absence of any constraints.

### 2.5. Molecular Dynamics (MD) Simulation

The Desmond module of the Schrödinger software was used to simulate the docked protein–ligand complexes in an explicit water environment. Using a system builder panel, an orthorhombic box was constructed. The simulation of the bound ligand–protein complex was performed using a simple point charge (SPC) model of water. The MD simulation was performed using a constant-temperature, constant-pressure ensemble (NPT) at 310 K and 1.013 bar as an atmospheric pressure intended to last 100 ns. The Simulation Interactions Diagram Report of the Desmond software was used to thoroughly examine the results of the molecular dynamics research [24,25].

### 2.6. Induced-Fit Docking (IFD)

Induced-fit docking was carried out on the best binding energy-containing bioactives with their respective receptors by employing the IFD panel of the Schrödinger suite [26]. Here, the IFD computations were carried out using the OPLS3 force field and the initial sample methodology, which generated up to 20 docked complexes utilizing automatic docking settings. The best poses produced during the standard Glide XP (extra-precision) docking of bioactive substances with their corresponding receptors were used as the input structures for IFD, and the centroid of the ligands was chosen to generate the grid box. No additional constraints were applied. A ring-conformational sampling of the ligand was done around a 2.5 kcal/mol energy window. Initial-glide docking was performed by setting both the receptor and ligand’s van der Waals (vdW) scaling to 0.5. The amino acid residues were also cleaned up using the Schrödinger Prime module within 5 Å of the ligand. The Prime-refined receptor–ligand complexes within 30 kcal/mol were then redocked using the Glide XP mode. The default settings for other parameters were maintained [27].

### 2.7. Molecular Mechanics-Generalized Born Surface Area (MM-GBSA) Calculation

The binding free energy was calculated from the structural information of the MM-GBSA method. These approaches employ the solvent accessibility method, molecular mechanics, and the generalized Born model to evade the free energy simulations’ convolution [28]. The energy difference between the bound and unbound complex of the protein–ligand complex was calculated with the help of MM-GBSA. The binding free energies were computed by the equation as follows:

MM-GBSA ΔG_Bind_ = G_Complex_ − (G_Receptor_ + G_Ligand_).

Here, the MM-GBSA calculation [29,30] was done by considering the VSGB solvation model with OPLS3 Force Field with a minimized sampling method.

### 2.8. Physicochemical and ADME/T Studies

The QikProp module of Schrödinger suite (QikProp, 2017_2) [31] and SwissADME [32,33] webserver (available at http://www.swissadme.ch/, accessed on 3 December 2022) were used to determine the physicochemical and pharmacokinetic properties of all the phytocannabinoids, which helps to determine the physicochemical significant descriptors and pharmacokinetically important properties of the phytocannabinoids.

## 3. Results

### 3.1. Identification of Bioactive Compounds and Targets

**Figure 1:**
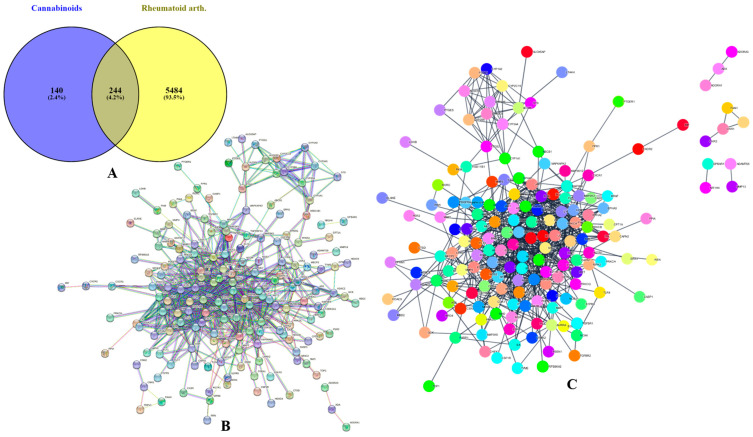
Screening of phytocannabinoids–rheumatoid arthritis common targets and PPI analysis. (**A**) Venn diagram of phytocannabinoids–rheumatoid arthritis common targets. (**B**) PPI network of common targets originated from STRING. (**C**) Visualization of the PPI network in Cytoscape.

A total of 13 phytocannabinoids, found in *Cannabis sativa*, were retrieved from the literature [14]. A total of 140 targets of the various phytocannabinoids were identified using the BindingDB (Supplementary Table S1) and SwissTargetPrediction (Supplementary Table S2) databases. Additionally, it was discovered that there were 2723 and 4881 RA-related targets predicted from two distinct databases, namely the GeneCards and DisGeNET databases (Supplementary Tables S3 and S4). After eliminating the repeating gene symbols, 384 targets linked to phytocannabinoids and 5728 targets associated to RA were obtained from these databases. The intersection of targets linked to phytocannabinoids’ bioactive properties and targets for RA yielded 244 similar targets (Supplementary Table S5) (Figure 1A).

### 3.2. Phytocannabinoids–Rheumatoid Arthritis Target and PPI Network Analysis

**Figure 2:**
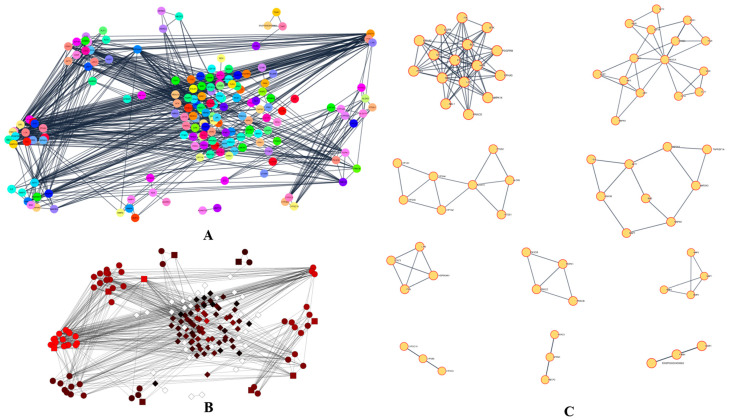
(**A**) PPI clusters visualized in the Cytoscape MCODE plugin. (**B**) Visualization in MCODE style. (**C**) Individual PPI clusters visualized in the Cytoscape MCODE plugin.

**Figure 3:**
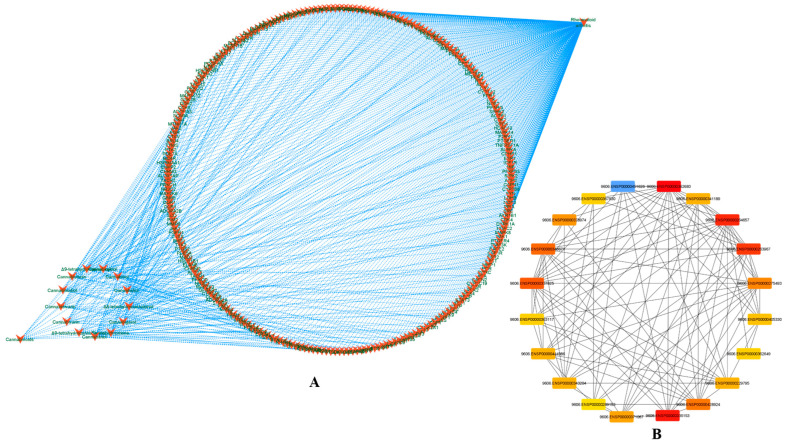
Phytocannabinoids–rheumatoid arthritis target network. (**A**) The 13 phytocannabinoids and the common targets (244 targets). (**B**) The top 20 nodes of the topological network obtained from the Cytohubba plug-in.

The phytocannabinoids and common targets were added to Cytoscape 3.9.1 for the construction of a network (Figure 1B,C). This constructed network demonstrated the existence of many and intricate impacts of phytocannabinoids on RA. Based on the gradation, the utmost common targets are mentioned in the Supplementary Material (Supplementary Table S6). The results predicted that the most prominent RA-associated genes were PIK3CA, PIK3CB, AKT1, AKT2, MAPK8, MAPK9, MAPK10, PRKCB, MAPK14, IGF1R, BRAF, PRKCZ, PRKCA, and NOS3 due to interaction of phytocannabinoids. The results of module analysis of the MCODE also predicted that the involvement of the above genes which supports the present findings (Figure 2A–C). The phytocannabinoids–RA target network constructed in Cytoscape (Figure 3A) predicted that there were 244 common target genes between 13 phytocannabinoids and RA. Further, 20 nodes of the common genes were found to be majorly involved as per the investigation of the topological network in the Cytohubba plug-in (Figure 3B).

### 3.3. GO and KEGG Enrichment Analyses

**Figure 4:**
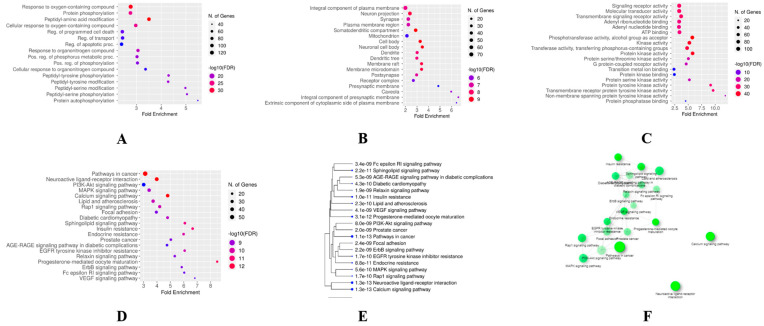
Results of GO analysis. (**A**) Dot plot of the biological process category terms from GO enrichment analysis (the *X*-axis and *Y*-axis show the fold enrichment and full names of the processes, respectively, and the color and size of each bubble represent the gene count and –log_10_FDR, respectively; the subsequent bubble charts are presented similarly). (**B**) Dot plot of the cellular component category terms from GO enrichment analysis. (**C**) Dot plot of the molecular function category terms from GO enrichment analysis. (**D**) Dot plot of pathways highly relevant to the treatment effects of phytocannabinoids. (**E**) Hierarchical clustering tree of the KEGG enrichment analysis. (**F**) Tree tab of the KEGG enrichment analysis.

A total of 4255 terms linked to the therapeutic effects of phytocannabinoids on RA were found as a result of GO enrichment analysis on common phytocannabinoids–RA targets. Figure 4A–C displays bubble charts for the top 20 terms in each of the three categories—biological process, cellular component, and molecular function. The results of the GO analysis showed that phytocannabinoids mainly suppressed cellular oxidation, protein phosphorylation, and changes to peptidyl amino acids. The top 20 pathways, which were ranked by the number of genes engaged in the expressed pathways (Figure 4D–F), were identified by the KEGG pathway enrichment analysis and were found to be involved in the interactions of phytocannabinoids to treat RA (Table S7). The findings indicated that the primary pathways involved in the current study were those that were associated with cancer, neuroactive ligand–receptor interaction, lipids and atherosclerosis, the calcium signaling pathway, and the Rap1 signaling pathway. These pathways are primarily associated with angiogenesis and inflammation, which are the defining characteristics of RA. By selecting the top 10 signaling pathways based on the number of genes, this study was able to identify the main primary targets of phytocannabinoids that are responsible for the pathogenesis of RA. Additionally, PIK3CA, AKT1, MAPK9, PRKCD, BRAF, IGF1R, and NOS3 were among the common target genes that were involved in reducing the severity of RA when different phytocannabinoids were used as treatment. These genes are also known to play a role in the pathogenesis of RA as shown by Cytohubba’s visualization of these genes in Cytoscape.

### 3.4. Molecular Docking Validation

Based on the Glidescores of the phytocannabinoids against PIK3CA (PDB ID: 4L23), the top three phytocannabinoids were discovered to be cannabigerol (−8.309 kcal/mol), Δ^9^-tetrahydrocannabivarin (−7.334 kcal/mol), and cannabichromene (−7.314 kcal/mol) (Supplementary Table S8). The phytocannabinoids cannabigerol and cannabichromene formed H-bonds with amino acid residues, namely Val851 and Tyr836, respectively. Cannabigerol formed a π-π interaction with Tyr836. All three phytocannabinoids developed similar polar interactions with Ser854 and Thr856. From the superimposition of all three phytocannabinoids, it was found that they fitted well into the common hydrophobic binding pocket consisting of Met772, Trp780, Ile800, Tyr836, Ile848, Val850, Val851, Met922, Phe930, and Ile932 (Supplementary Table S9).

In the case of AKT1 (Gene: AKT1), the top three phytocannabinoids were discovered to be cannabitriol (−6.035 kcal/mol), cannabichromene (−4.599 kcal/mol), and cannabielsoin (−4.466 kcal/mol). The phytocannabinoids cannabitriol and cannabichromene formed a common H-bond with Asn279. Cannabitriol formed two additional H-bonds with Lys276 and Asp292. The phytocannabinoid cannabielsoin formed H-bonds with Lys158 and Glu278. All three phytocannabinoids exhibited common polar interactions with Asn279 and Thr291. The superimposition of the three phytocannabinoids predicted that they all easily fit into the Leu156, Val164, Ala177, Met227, Tyr229, Ala230, Met281, and Phe438 residues-based shared hydrophobic binding pocket.

In the case of MAPK9 (Gene: MAPK9), the top three phytocannabinoids were found to be cannabinodiol (−7.664 kcal/mol), cannabichromene (−7.663 kcal/mol), and Δ^8^-tetrahydrocannabinol (−6.751 kcal/mol). Cannabinodiol formed H-bond with Ile32 and Ser155, whereas the phytocannabinoids cannabichromene and Δ^8^-tetrahydrocannabinol formed H-bonds with Glu109 and Met111; and Lys55, respectively. All three phytocannabinoids formed common polar interactions with Ser34, Asn114, and Ser155. Through the superimposition of all three phytocannabinoids, it was revealed that they fitted well into the common hydrophobic binding pocket consisting of Ile32, Val40, Ala53, Ile86, Met108, Leu110, Met111, Val158, and Leu168.

In the case of PRKCD (Gene: PRKCD), the top three phytocannabinoids were found to be cannabitriol (−7.308 kcal/mol), Δ^9^-tetrahydrocannabinol (−5.123 kcal/mol), and Δ^9^-tetrahydrocannabivarin (−5.121 kcal/mol). All three phytocannabinoids formed two common H-bonds with Val174 and Gln284, while cannabitriol formed an additional H-bond with Leu279. All three phytocannabinoids formed four common polar interactions with Ser173, Hie231, Asn283, and Gln284. It was discovered via the superimposition of all three phytocannabinoids that they all fit comfortably into the same hydrophobic binding pocket that is made up of the residues Val174, Cys175, Cys264, Met266, Leu279, Cys280, Ile282, and Leu286.

In the case of BRAF (PDB ID: 6P3D), the top three phytocannabinoids were found to be cannabigerol (−9.484 kcal/mol), cannabinodiol (−8.076 kcal/mol), cannabichromene (−7.522 kcal/mol). The phytocannabinoids cannabigerol and cannabinodiol formed H-bonds and π-π interactions with Cys532 and Phe595, respectively. All three phytocannabinoids formed two common polar interactions with Thr529 and Gln530. It was discovered via the superimposition of all three phytocannabinoids that they all fit comfortably into the same hydrophobic binding pocket made up of the residues Ile463, Val471, Ala481, Val2, Leu514, Trp531, Cys532, Phe583, and Phe595.

In the case of IGF1R (PDB ID: 5FXS), the top three phytocannabinoids were found to be Δ^9^-tetrahydrocannabinol (−8.569 kcal/mol), Δ^9^-tetrahydrocannabivarin (−8.402 kcal/mol), and cannabigerol (−8.190 kcal/mol). All three phytocannabinoids formed an H-bond with Met1082. They also formed one common polar interaction with Thr1083. It was discovered by the superimposition of all three phytocannabinoids that they all fit comfortably into the same hydrophobic binding pocket made up of the residues Leu1005, Phe1010, Val1013, Ala1031, Leu1081, Met1082, Ile1156, and Met1160.

**Figure 5:**
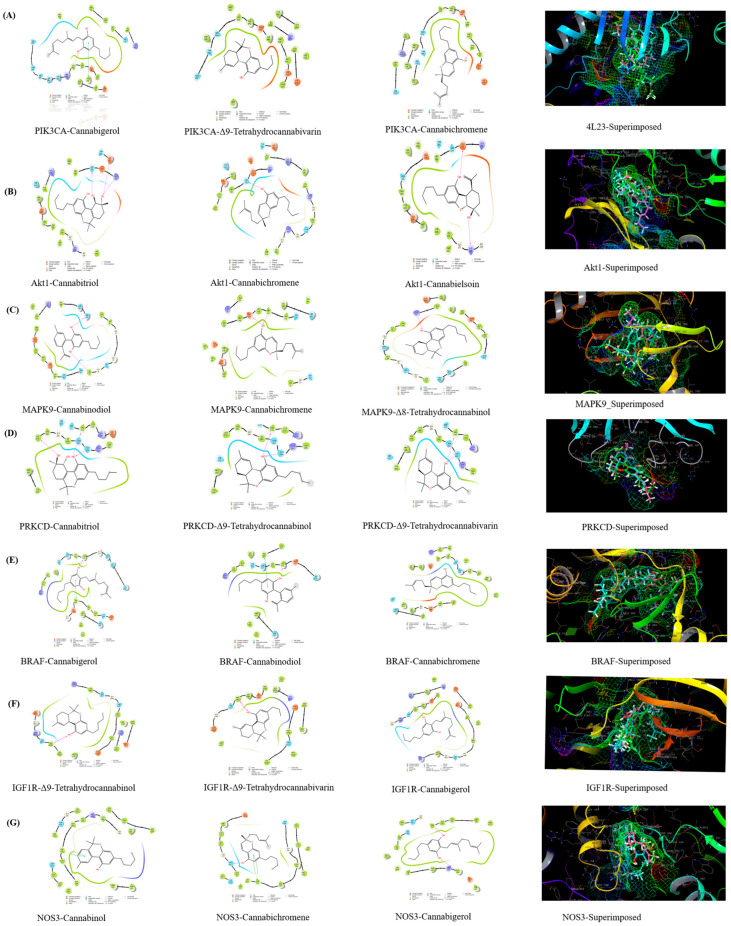
Molecular docking results (2D representation and superimposition) of the best active compounds with key targets, i.e., PIK3CA (**A**), AKT1 (**B**), MAPK9 (**C**), PRKCD (**D**), BRAF (**E**), IGF1R (**F**), and NOS3, (**G**) and phytocannabinoids.

In the case of NOS3 (PDB ID: 6NH5), the top three phytocannabinoids were found to be cannabinol (−8.888 kcal/mol), cannabichromene (−8.152 kcal/mol), and cannabigerol (−8.080 kcal/mol). The phytocannabinoids cannabinol and cannabichromene formed one common π-π interaction with Trp178. Cannabichromene formed one H-bond with Ser354, while cannabigerol formed two H-bonds with Cys184 and Pro334. All three phytocannabinoids formed two common polar interactions with Ser226 and Ser354. It was discovered by the superimposition of all three phytocannabinoids that they all fit comfortably into the same hydrophobic binding pocket that is made up of the residues Trp178, Ala181, Cys184, Leu193, Ala227, Pro334, Val336, Met339, Phe353, and Phe473 (Figure 5 and Figure S1).

### 3.5. Molecular Dynamics

The molecular dynamics simulations of protein–ligand complexes are thought to be the best developed method for assessing the structure-to-function relationships of macromolecules in computer-aided drug design. To clarify the cannabinoid’s potency against the six proteins associated with RA, an impartial molecular dynamics investigation was conducted in the current study. The stability and viability of the best docked ligands against all the target proteins were examined using MD simulation studies, which were run for 100 ns. The root mean square deviation (RMSD), the root mean square fluctuation (RMSF), and protein–ligand interactions were generated with the production of 100 ns MD simulations, and their relevance in the solidity of the protein–ligand complexes was thoroughly examined. Protein–ligand interactions and protein–ligand RMSDs were investigated and visualized during this period.

For the PIK3CA–cannabigerol complex, their RMSD for the first 50 nanoseconds was more than 2.5 Å, and from 50 nanoseconds on, the ligand started to stabilize with the protein at above 3.5 Å. It was discovered through protein–ligand contact analysis that roughly 50% of the amino acids were responsible for the hydrophobic interactions with cannabigerol. The remaining 50% were engaged in either the H-bond interactions or the water bridges. Hydrophobic interactions with cannabigerol were largely caused by the residues Met772, Pro778, Trp780, Ile800, Tyr836, Ile848, Val850, Met922, and Ile932, of which Met772 and Ile932 were the most accountable at 0.15 ns and 0.20 ns fractions of the interaction time, respectively. Glu849 and Val851 were responsible for the highest H-bond contacts at 0.10 ns and 0.25 ns fractions, while Met772, Ser919, and Asn920 were responsible for forming highest water bridge contacts at 0.20 ns and 0.35 ns fractions.

For the AKT1–cannabitriol complex, their RMSD for the first 60 nanoseconds was stabilized at above 4 Å, while for the next 40 nanoseconds, both the ligand and protein RMSDs were slightly fluctuating at 4.8 Å. Through protein–ligand contact analysis, it was discovered that the residues Phe161, Val164, Ala177, Lys179, and Leu181 were highly responsible for forming hydrophobic contact with cannabitriol, among which Val164 and Lys179 were the most accountable ones at 0.3 ns and 0.6 ns fractions of the interaction time, respectively. Glu234 and Asp439 were responsible for the highest water bridge forming contacts at 0.3 ns and 0.5 ns fractions. Lys179 and Asp439 were the H-bonding residues, whereas Glu234 and Asp439 were the residues forming ionic contacts.

The protein–ligand RMSD plots for the MAPK9–cannabinodiol complex showed that RMSD was greater than 4 Å for the first few nanoseconds, increased to 12 Å after a while, and then increased to 16 Å before stabilizing there. Through protein–ligand contact analysis, it was found that the residues Val40, Ala53, Leu57, Ile86, Met108, Leu110, Val158, and Leu168 were responsible for hydrophobic contacts with cannabinodiol, among which Val40, Ala53, Val158, and Leu168 were the most accountable ones at 0.40 ns, 0.30 ns, 0.35 ns, and 0.30 ns fractions of the interaction time, respectively. Ile32 and Lys55 were responsible for the highest water bridging at 0.35 ns, while, on the other hand, Ile32, Lys55, Asn114, Ser155, and Asn156 were responsible for H-bonding contacts.

The RMSD for the PRKCD-cannabitriol complex was greater than 4 Å for the first few nanoseconds before increasing to 8 Å and then stabilizing there. According to the protein–ligand contact study, Ser173, Gln284, and Lys285 in H-bond interactions at 0.8, 1.0, and 0.8 ns of the interaction period, respectively, accounted for the majority of the protein–ligand connections with cannabitriol. The remaining amino acid residues were in charge of either creating water bridges or creating hydrophobic interactions.

The RMSD for the BRAF–cannabigerol complex was greater than 1.6 Å for the first few nanoseconds before increasing to 2.4 Å, then 2.8 Å, and then stabilizing at that value. Trp531, Phe583, and Phe595 were determined to be primarily in charge of hydrophobic interactions with cannabigerol at 0.22 ns, 0.15 ns, and 0.14 ns fractions of the interaction time, respectively, according to the protein–ligand contact study. The majority of the residues either contributed to the formation of water bridges or H-bonds. Among these, Gln461 and Cys532 played a significant role in the formation of H-bonds. Ile463, Glu533, and Gly534 played a significant role in the formation of water bridges.

**Figure 6:**
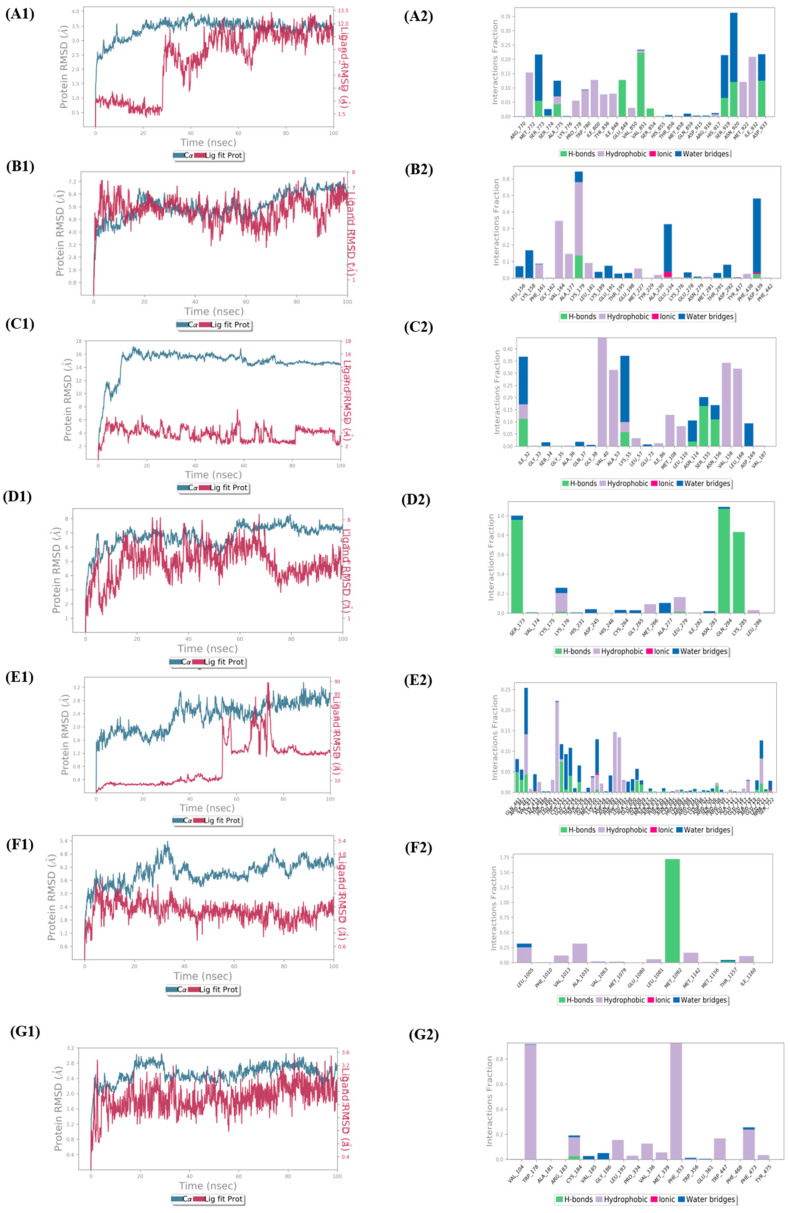
Molecular dynamics results of key target ligands. Two-dimensional representation and protein–ligands contact analysis of PIK3CA–cannabigerol (**A1**,**A2**), Akt1–cannabitriol (**B1**,**B2**), MAPK9–cannabinodiol (**C1**,**C2**), PRKCD–cannabitriol (**D1**,**D2**), BRAF–cannabigerol (**E1**,**E2**), IGF1R-Δ^9^–tetrahydrocannabinol (**F1**,**F2**), and NOS3–cannabinol (**G1**,**G2**) complexes.

The RMSD for the IGF1R-Δ^9^–tetrahydrocannabinol complex was more than 3.0 Å for the first few nanoseconds, but with time, it increased to 4.8 Å, where it stabilized. Based on protein–ligand contact analysis, it was found that Met1082 was responsible for the highest H-bond contact with Δ^9^-tetrahydrocannabinol at the 1.75 ns fraction of the interaction time. The amino acid residues Leu1005, Val1013, Ala1031, Met1142, and Ile1160 were mostly responsible for hydrophobic contacts. Leu1005 and Thr1157 were responsible for forming water bridges (Figure 6 and Figure S2).

The RMSD for the NOS3–cannabinol complex was higher than 2.4 Å for the first few nanoseconds, then it increased to 2.8 Å, and ultimately it decreased to 2.4 Å and stabilized there. The amino acid residues Trp178 and Phe353 were predicted to have the most hydrophobic interactions with cannabinol based on the protein–ligand contact study. Most of the water bridging was done by Gly186 and Val185. It was Cys184 that made the H-bond interaction.

### 3.6. Induced-Fit Docking and MM-GBSA Calculation

IFD (Figure S3) of all the proteins with their best docked pose-forming phytocannabinoids (obtained via standard XP-mode docking) predicted that almost all the proteins had improved Glidescores, with the exception of PIK3CA and PRKCD (Supplementary Table S10). However, for IFD of PIK3CA with cannabigerol, the number of H-bonds increased from one (in the normal XP-mode Glide docking) to two (in the IFD-generated pose). From IFD of MAPK9 with cannabinodiol it was found that the number of H-bonds decreased from two (in the normal XP-mode Glide docking) to one (in the IFD-generated pose). For IFD of PRKCD with cannabitriol, the number of H-bonds increased from three (in the normal XP-mode Glide docking) to five (in the IFD-generated pose). For IFD of BRAF with cannabigerol, the number of H-bonds increased from one (in the normal XP-mode Glide docking) to two (in IFD-generated pose). Similarly, from IFD of IFG1R with Δ^9^-tetrahydrocannabinol it was found that the number of H-bonds increased from one (in the normal XP-mode Glide docking) to two (in IFD-generated pose). In case of IFD of NOS3 with cannabinol, the number of π-π stacking interactions increased from one (in the normal XP-mode Glide docking) to two (in IFD-generated pose). Additionally, one H-bond interaction with Ala177 was observed from the IFD-generated pose which was absent in the normal XP-mode Glide docked pose. Furthermore, the prime MM-GBSA binding free energies of the best five docked complexes were calculated and are given in the Supplementary Material (Table S11).

### 3.7. Physicochemical and ADME/T Studies

Data generated by QikProp predicted that all the expected attributes for phytocannabinoids fell inside the acceptable range. The SwissADME-predicted data showed that the phytocannabinoids had significant values for the studied attributes, and based on their physicochemical features, they demonstrated drug-like qualities. All phytocannabinoids obeyed Veber’s rule. The phytocannabinoids cannabigerol and cannabichromene failed to pass/satisfy the blood–brain barrier (BBB) filter (Figure S4), but other phytocannabinoids, such as cannabinol, cannabivarin, cannadinodiol, and cannabitriol, were discovered to be P-glycoprotein (PGP) substrates. It was projected that the majority of phytocannabinoids, except for cannabielsoin and cannabitriol, would be CYP450 enzyme inhibitors. Both CYP2C9 and CYP2D6 subtypes were found to be inhibited by cannabielsoin; however only CYP2D6 subtypes were found to be inhibited by cannabitriol (Supplementary Table S12).

## 4. Discussion

**Figure 7:**
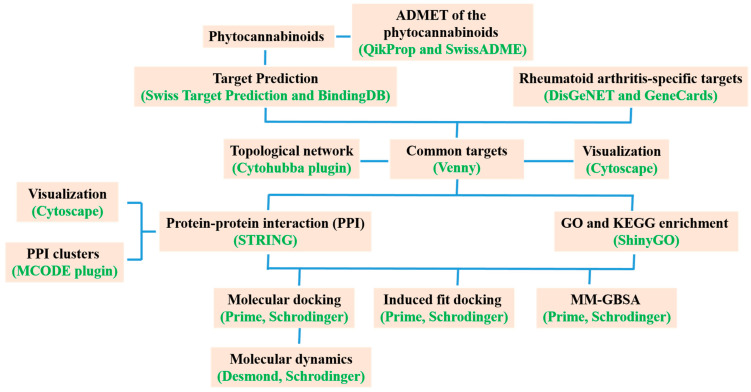
A general workflow scheme of the study.

The chronic inflammatory condition known as arthritis is characterized by immune-mediated inflammatory sinusitis, which causes hyperplasia, vasculogenesis, pannus development, the deterioration of cartilage and bone, joint deformity, and functional impairment. Many phytocannabinoids, cannabinoid receptors, and enzymes that are part of the endocannabinoid system all contribute to the pharmacological activity of cannabis in different ways. It is well known that the endocannabinoid system inhibits the pathogenesis of the inflammatory and autoimmune disease RA. Due to the abundance of natural phytocannabinoids, the goal of this study was to build on the signaling pathways, associated proteins, and genes that cause RA using integrated bioinformatics and network pharmacology. The general workflow scheme of the present study is depicted in Figure 7.

We gathered 2723 and 4881 predicted targets for phytocannabinoids from two distinct databases, namely GeneCards and the DisGeNET database, respectively. Thirteen phytocannabinoids and 244 common targets were obtained by the intersection of RA targets and phytocannabinoids (with the aid of venny 2.1.0). The PPI enrichment analysis results were reported with 244 nodes, 699 edges, highest confidence (0.90), and the PPI enrichment *p*-value of <1.0 × 10^−16^. As would be predicted, the proteins interacted with one another quite a bit. The results of the enrichment analysis showed that the proteins were, collectively, at least loosely physiologically interconnected. For the hub PPI, we chose nodes whose median edge count was greater than double that of all other nodes.

The results of the PPI network and GO analysis predicted that the key proteins involved in the numerous phytocannabinoids–RA common targets were primarily those involved in the pathways for cancer, neuroactive ligand–receptor interaction, lipids and atherosclerosis, calcium signaling, and RAP signaling. In cancer, there is an abnormality in the signaling of PI3K/Akt/mTOR due to which many types of research have been done to find the inhibitors of PI3K/Akt/mTOR signaling which may act as anticancer agents. RA is an autoimmune-mediated inflammatory disease where the abnormal proliferation of immune cells, macrophages, monocytes, dendritic cells, and synovial fibroblasts may significantly overlap with the abnormal growth of cancer cells. The results of some recent studies in arthritis using PI3K signaling inhibitors suggest that small molecule inhibitor strategies directed at PI3K signaling may be useful for immune-mediated arthritis [34]. The present study predicted that PIK3CA and AKT are the most common genes found in the top pathways of phytocannabinoids to combat arthritis. A previous study suggested that phytocannabinoids are responsible for the regulation of PI3K/AKT/GSK-3 in the brain [35]. Hence, PI3K and AKT may be the target of phytocannabinoids in RA. A previous study revealed that MAPK9 causes an increase in cell proliferation, migration, invasion, inflammatory response, facilitates apoptosis in RA patients, and promotes the progression of fibroblast-like synoviocytes [36]. Protein kinase C like PRKCD is known to have a crucial role in the pathogenesis of autoimmune diseases like RA [37]. The current study suggested the involvement of MAPK and the protein kinase C pathway in combating arthritis by phytocannabinoids. Previous in vitro studies in cell lines reveal that phytocannabinoids cause cytotoxicity in leukemic cells through the MAPK pathway [38]. The cannabinoid receptor 2 also has an inhibitory role in synovial fibroblasts in RA through protein kinase [39]. These results support our current findings. The BRAF gene was found to transform the rheumatoid synovial fibroblasts and lead to the destruction of articular cartilage and bone in RA [40]. Previous studies revealed that IGF-1R signaling leads to inflammation in RA due to the formation of various cytokines like IL-6 and activation of T cells [41]. A previous study revealed that cannabinoid receptors promote the chronic intermittent hypoxia-induced breast cancer metastasis via IGF-1R/AKT/GSK-3β. It indicates that phytocannabinoids may ameliorate RA through cannabinoid-receptor-induced IGF-1R and AKT. There is a common link of pathogenesis of RA and atherosclerosis. Patients with RA are at approximately 1.5-fold risk of atherosclerotic cardiovascular disease (CVD) compared with the general population [42]. Pro-inflammatory cytokines such as tumor necrosis factor alpha and interleukin-6, involved in the pathogenesis of RA, are also independently predictive of subsequent cardiovascular disease (CVD). In RA, inflammation alters HDL constituents and the concentration of LDL and HDL, thus facilitating atherosclerosis and CVD events. On the other hand, the increase of oxidative processes, frequently observed in RA, also induces atherosclerosis [43]. Cannabinoids were reported to cause a decrease in inflammation during atherosclerosis [44] which correlates with the current findings. A previous study reported that the alterations in calcium signaling may cause the functional T cell abnormalities associated with RA [45,46]. The RAP signaling pathway is also responsible for the occurrence of RA. RAP1 is deregulated in T lymphocytes, which leads to the overproduction of reactive oxygen species, which promotes the hyporesponsiveness of synovial T lymphocytes, leading to inflammation [47]. A previous study revealed that the cannabinoid receptor (CB1) causes neurite outgrowth through the Rap1 pathway [48]. This indicates that phytocannabinoids may alter the RAP1 pathway through the cannabinoid receptor (CB1). Nitric oxide is produced by nitric oxide synthetase (NOS). Important cytokines produced in RA are IL-1 and TNF-α, which stimulate NOS in inflammatory cells and increase the production of nitric oxides. There is an increase in nitric oxide levels in arthritis, as shown in preclinical and clinical studies [49]. A previous study reported that phytocannabinoids may prevent RA conditions by decreasing nitric oxide levels and cytokines [50] which supports the current findings. The results of the present studies correlate with the results of the previous similar studies that substantiate the present findings [51,52].

In light of the current findings of network pharmacology, PIK3CA, AKT1, MAPK9, PRKCD, BRAF, IGF1R, and NOS3 were chosen as the seven key target proteins closely related to the progression of RA. These proteins were further subjected to molecular docking with phytocannabinoids. Negative binding energy indicates effective binding to the target. Lower binding energy suggests a stable ligand–receptor complex better explained as <−5 kcal/mol called good binding activity and <−7 kcal/mol called strong binding activity. Except for AKT1 and PRKCD, the Glidescores of the top three compounds against the other five proteins were in the range of −8.888 to −6.751 kcal/mol. The top three active ingredients were shown to have a strong binding interaction with their respective proteins. It entails unexpected bias from the materiality, which could hasten the anonymous fallacy in in vitro or in vivo studies. However, the results of the molecular docking experiment suggest the potential therapeutic approaches and could guide trials in animals. Network pharmacology is currently used extensively for various traditional medicines, such as Indian traditional medicine, traditional Chinese medicine, Kampo, traditional Korean medicine, etc. Network pharmacology research is being conducted owing to its robust potential to investigate convoluted relationships among multiple targets. Additionally, network pharmacology accelerates the modernization of the Indian traditional system of medicine by usefully illuminating the synergistic effects of biochemical and systemic biology methodologies. Researchers may identify prospective active ingredients for upcoming in vitro and in vivo studies by using molecular docking to lay out an effective binding mode of the active ingredients with disease-related important target proteins. However, a number of the Ayurvedic formula’s constituents can have antagonistic and synergistic effects on the body while being treated because they target the same protein. It is important to consider these implications and to continue to assess them. The findings of MM-GBSA (binding free energy) and IFD support the molecular dynamics findings that the ligand is stable within the protein. One of the finest molecular dynamics results was obtained with the Akt1-cannabitriol complex, despite its Glidescore of −6.035 kcal/mol. Among the seven proteins, the phytocannabinoids cannabigerol, canabichromene, and Δ^9^-tetrahydrocannabivarin had the highest Glidescores and the most drug-like qualities, as well as acceptable ADME/T profiles.

**Figure 8:**
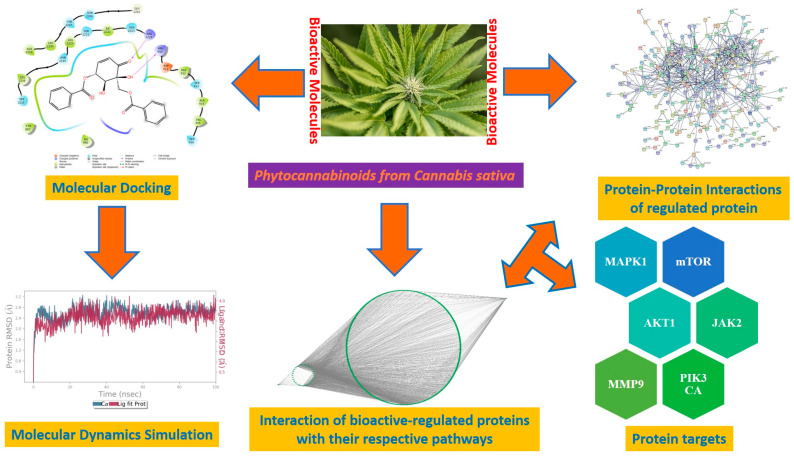
Probable mechanisms of phytocannabinoids in rheumatoid arthritis.

The present study evaluated the integrated bioinformatics and network pharmacology to identify therapeutic targets and molecular processes involving the endocannabinoid system in patients RA. The work used a combined bioinformatics and network pharmacology approach to further examine signaling pathways, associated proteins, and genes that may implicate naturally occurring phytocannabinoids in the pathogenesis of RA. The current research findings suggest that the abundant phytocannabinoids can exert their curative effect through a synergistic effect of multi-components, multi-targets, and multi-pathways in the treatment of RA. However, due to the limitation of network pharmacology and molecular docking, a more complete evaluation of the role of the endocannabinoid system in the immune landscape in patients with RA and a comprehensive analysis of patients with RA and normal controls would be necessary. Hence, further evaluation of in vitro and in vivo studies needs to be carried out to validate the current findings, which is the future scope of the present study. The probable mechanisms of phytocannabinoids in rheumatoid arthritis are depicted in Figure 8.

## 5. Conclusions

The network pharmacology analysis results predicted that the processes such as response to oxygen-containing compounds and peptodyl-amino acid modification are related to the potential treatment mechanisms for rheumatoid arthritis. These biological actions may be coordinated by cancer, neuroactive ligand–receptor interaction, lipids and atherosclerosis, the calcium signaling pathway, and the Rap1 signaling pathway. According to the results of molecular docking, in the context of rheumatoid arthritis, phytocannabinoids may bind to important target proteins such as PIK3CA, AKT1, MAPK9, PRKCD, BRAF, IGF1R, and NOS3. Due to a lack of experimental validation, this study has shortcomings, although it can inspire in vitro or in vivo studies. However, due to the limitations of network pharmacology and molecular docking, a more complete evaluation of the role of the endocannabinoid system in the immune landscape in patients with RA and a comprehensive analysis of patients with RA and normal controls would be necessary. This entire study predicted the phytocannabinoids’ systemic biological characteristics. Future experimental research is still needed to confirm the results so far.

## Data Availability

The datasets supposing the current study are available in public databases from IMPPAT, PUBMED, PubChem, Swiss Target Prediction, BindingDB, DisGeNET, GeneCards, OMIM, UniProt, STRING, ShinyGO, RCSB PDB, and SwissADME. All the analysed data during the current study are available in the manuscript and Supplementary Material.

## Supplementary Materials

The following supporting information can be downloaded at: https://www.mdpi.com/article/10.3390/life13030700/s1, Figure S1: Molecular docking results (3D representation of the best active ingredients) of key targets (PIK3CA, Akt1, MAPK9, PRKCD, BRAF, IGF1R, NOS3) and specific cannabinoids; Figure S2: Molecular dynamics results of key target-ligands. RMSF and Protein-Ligands Contact Analysis of PIK3CA-cannabigerol (A1,B1,C1), Akt1-cannabitriol (A2,B2,C2), MAPK9-cannadinodiol (A3,B3,C3), PRKCD-cannabitriol (A4,B4,C4), BRAF-cannabigerol (A5,B5,C5), IGF1R-Δ9-tetrahydrocannabinol (A6,B6,C6), NOS3-cannabinol (A7,B7,C7) complexes; Figure S3: Induced Fit Docking results of key target-ligands. 2D representation of PIK3CA-cannabigerol, Akt1-cannabitriol, MAPK9-cannadinodiol, PRKCD-cannabitriol, BRAF-cannabigerol, IGF1R-Δ9-tetrahydrocannabinol, NOS3-cannabinol complexes; Figure S4: Egg boil diagram of Cannabigerol and Cannabichromene imported from SwissADME; Table S1: Results of BindingDB Database; Table S2: Results of the SwissTargetPrediction; Table S3: Disgenet-predicted disease targets for rheumatoid arthritis; Table S4: GeneCards predicted disease targets for rheumatoid arthritis; Table S5: List of 244 common genes (data obtained from venny); Table S6: Result of PPI network analysis in String Database; Table S7: Results of KEGG analysis in ShinyGO 0.76 platform; Table S8: Molecular docking results of cannabinoids based on their docking scores; Table S9: Molecular docking results of cannabinoids based on their interactions; Table S10: Results of Induced Fit docking; Table S11: Binding free energies results of cannabinoids the based on MM-GBSA calculations; Table S12: Physico-chemical properties and ADME/T profile of cannabinoids.

## Abbreviations

ADMET	Absorption, distribution, metabolism, excretion, and toxicity
BBB	Blood-brain barrier
CB	Cannabinoid receptor
CVD	Cardiovascular disease
GO	Gene Ontology
IFD	Induced Fit docking
KEGG	Kyoto Encyclopedia of Genes and Genomes
MCODE	Molecular Complex Detection
MM-GBSA	Molecular mechanics-generalized Born surface area
NOS	Nitric oxide synthetase
NSAIDs	Non-steroidal anti-inflammatory drugs
PPI	Protein-protein interaction
PDB	Protein data bank
PGP	P-glycoprotein
RA	Rheumatoid arthritis
RMSD	Root mean square deviation
RMSF	Root mean square fluctuation
SPC	Simple point charge
THC	Tetrahydrocannabinoids
vdW	van der Waals
