# Supplementary material for: The Abundant Phytocannabinoids in Rheumatoid Arthritis: Therapeutic Targets and Molecular Processes Identified Using Integrated Bioinformatics and Network Pharmacology
Source: Life (Basel). 2023 Mar 5;13(3):700. doi: 10.3390/life13030700 (PMC10053995; doi:10.3390/life13030700)
Supplement: Supplementary file 1 [file life-13-00700-s001.zip › life-2143723-supplementary.pdf]

# Supplementary Material

# The abundant phytocannabinoids in rheumatoid arthritis: Therapeutic targets and molecular processes identified using the integrated bioinformatics and network pharmacology

Arijit Nandi[1], Anwesha Das[2], Yadu Nandan Dey[1]*, Kuldeep K. Roy[3]*

[1]Dr. B.C. Roy College of Pharmacy and Allied Health Sciences, Durgapur-713206, West Bengal, India

[2]Department of Medicinal Chemistry, National Institute of Pharmaceutical Education and Research, Ahmedabad, Palaj, Gandhinagar 382355, Gujarat, India

[3]Department of Pharmaceutical Sciences, School of Health Sciences and Technology, UPES University, Dehradun- 248007, Uttarakhand, India

**Corresponding Authors**

*Dr. Yadu Nandan Dey, Associate Professor, Dr. B.C. Roy College of Pharmacy and Allied Health Sciences, Durgapur-713206, West Bengal, India.

E-mail: yadunandandey@gmail.com; Phone: +91-7566232359

*Dr. Kuldeep K. Roy, Associate professor, Department of Pharmaceutical Sciences, School of Health Sciences and Technology, UPES University, Dehradun- 248007, Uttarakhand, India

E-mail: kuldeepkroy@gmail.com, kuldeep.roy@ddn.upes.ac.in

**Supplementary figures.**

**Figure S1.** Molecular docking results (3D representation of the best active ingredients) of key targets (PIK3CA, Akt1, MAPK9, PRKCD, BRAF, IGF1R, NOS3) and specific cannabinoids.

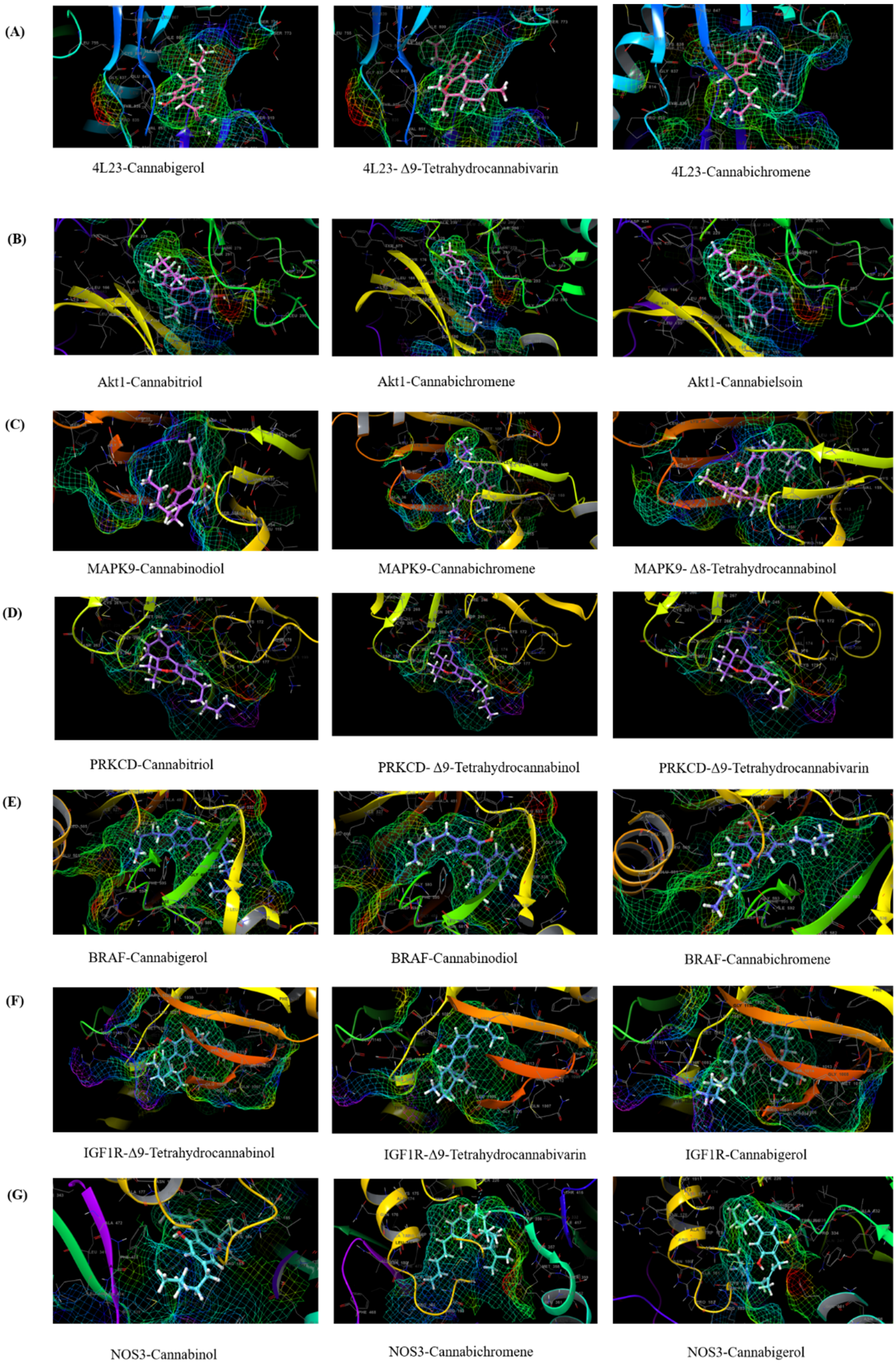


**Figure S2.** Molecular dynamics results of key target-ligands. RMSF and Protein-Ligands Contact Analysis of PIK3CA-cannabigerol (A1, B1, C1), Akt1-cannabitriol (A2,B2,C2), MAPK9-cannadinodiol (A3,B3,C3), PRKCD- cannabitriol (A4,B4,C4), BRAF- cannabigerol (A5,B5,C5), IGF1R- Δ9-tetrahydrocannabinol (A6,B6,C6), NOS3- cannabinol (A7,B7,C7) complexes.

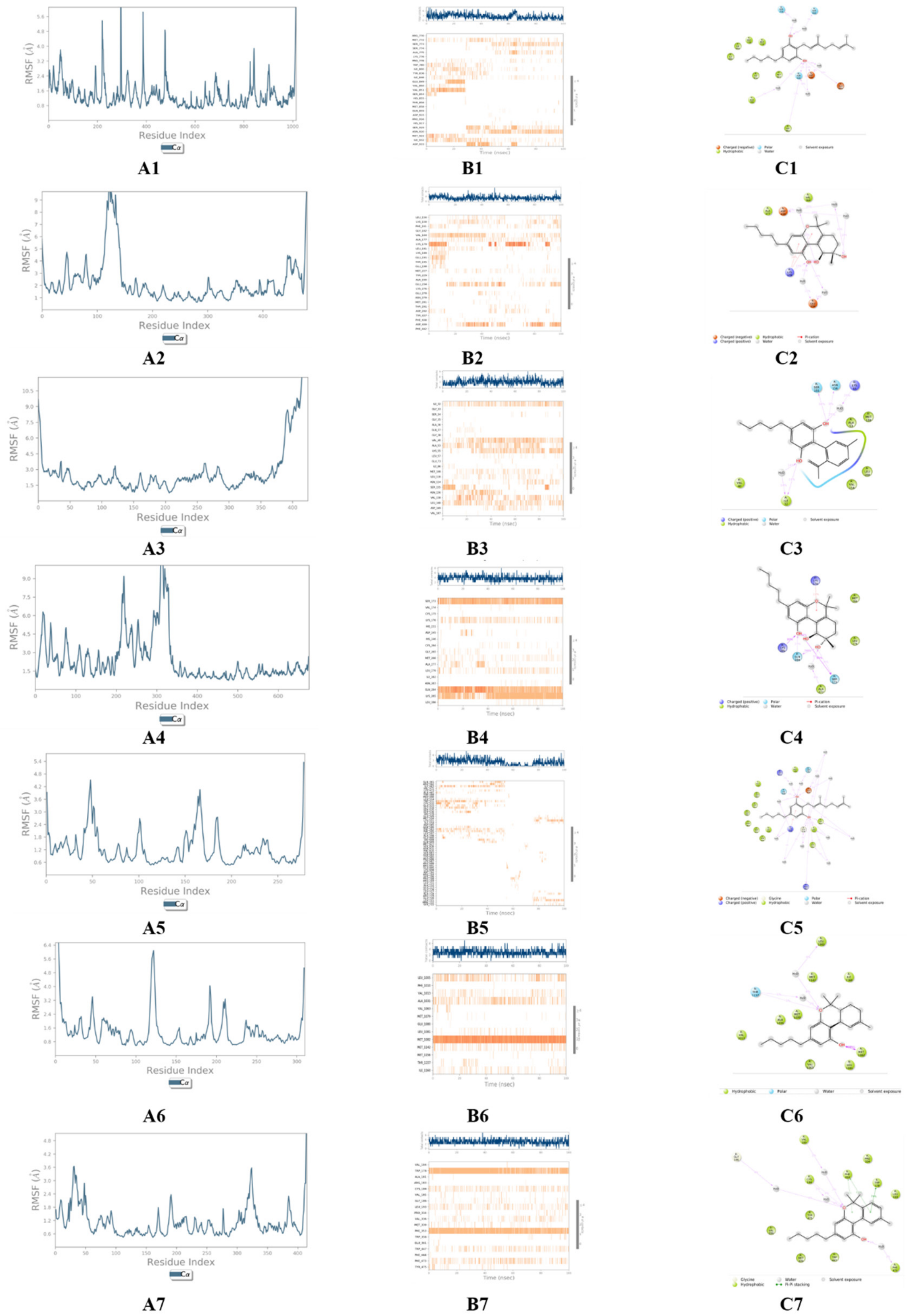


**Figure S3**. Induced Fit Docking results of key target-ligands. 2D representation of PIK3CA-cannabigerol, Akt1-cannabitriol, MAPK9-cannadinodiol, PRKCD-cannabitriol, BRAF- cannabigerol, IGF1R- Δ9-tetrahydrocannabinol, NOS3- cannabinol complexes.

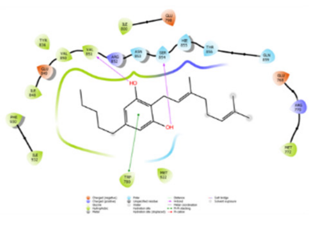
PIK3CA-Cannabigerol

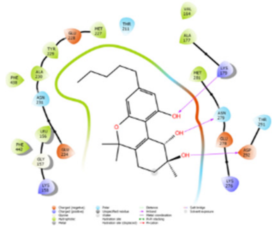
Akt1-Cannabitriol

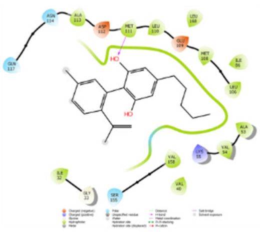
MAPK9-Cannabinodiol

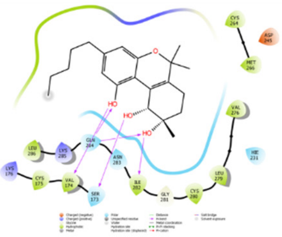
PRKCD-Cannabitriol

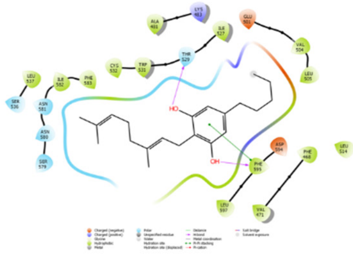
BRAF-Cannabigerol

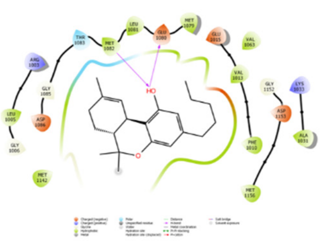
IGF1R-Δ9-Tetrahydrocannabinol

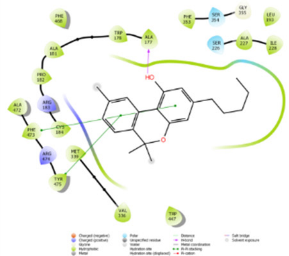
NOS3-Cannabinol

**Figure S4.** Egg boil diagram of Cannabigerol and Cannabichromene imported from SwissADME.

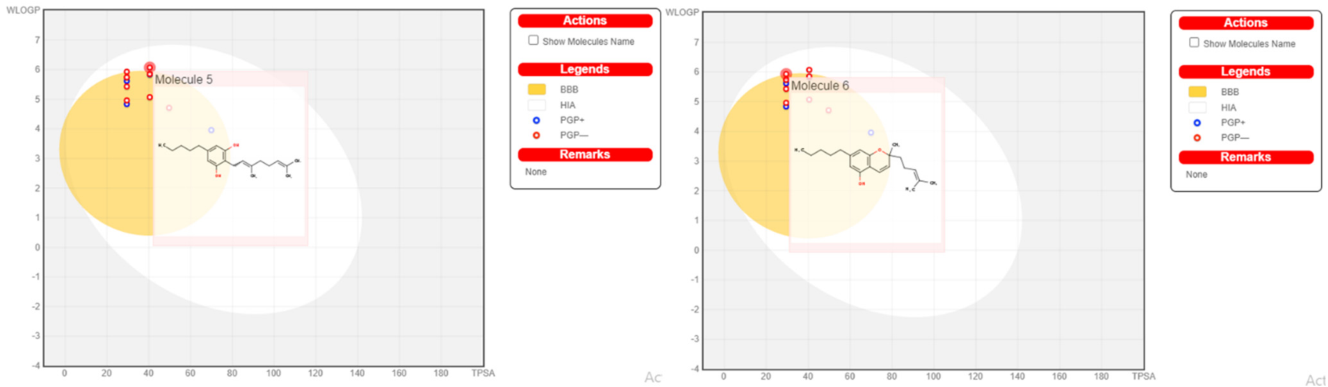


**Supplementary Tables.**

**Table S1. Results of BindingDB Database**

| Sr. No. | Target Name | Uploaded compounds | Max | Hits | Ki | $IC_{50}$ | Kd | $EC_{50}$ | Download | UniProt ID | UniProt Name | Organism | Gene |
|---|---|---|---|---|---|---|---|---|---|---|---|---|---|
| 1 | Cannabinoid receptor 1 | 11 | 1 | 227 | 395 | 12 | 29 | 38 | 2D 3D TSV | P21554 | Cannabinoid receptor 1 | Homo sapiens (Human) | CNR1 (CNR) |
| 2 | Cannabinoid receptor 1/2 | 4 | 1 | 14 | 14 | 5 | 0 | 0 | 2D 3D TSV | P21554 | Cannabinoid receptor 1 | Homo sapiens | CNR1 (CNR) |

| | | | | | | | | | | | | | |
|---|---|---|---|---|---|---|---|---|---|---|---|---|---|
| | | | | | | | | | | | | (Human) | |
| 2 | Cannabinoid receptor 1/2 | 4 | 1 | 14 | 14 | 5 | 0 | 0 | 2D 3D TSV | P34972 | Cannabinoid receptor 2 | Homo sapiens (Human) | CNR2 (CB2A, CB2B) |
| 3 | Cannabinoid receptor 2 | 11 | 1 | 175 | 358 | 13 | 0 | 27 | 2D 3D TSV | P34972 | Cannabinoid receptor 2 | Homo sapiens (Human) | CNR2 (CB2A, CB2B) |
| 4 | Caspase-1 | 2 | 1 | 1 | 0 | 0 | 2 | 0 | 2D 3D TSV | P29466 | Caspase-1 | Homo sapiens (Human) | CASP1 (IL1BC, IL1BCE) |
| 5 | Cholinesterase | 2 | 1 | 1 | 0 | 1 | 0 | 0 | 2D 3D TSV | NOT FOR HUMANS | | | |
| 6 | Cytochrome P450 1A1 | 4 | 1 | 2 | 3 | 0 | 0 | 0 | 2D 3D TSV | P04798 | Cytochrome P450 1A1 | Homo sapiens (Human) | CYP1A1 |
| 7 | Cytochrome P450 1A2 | 2 | 1 | 1 | 1 | 0 | 0 | 0 | 2D 3D TSV | P05177 | Cytochrome P450 1A2 | Homo sapiens (Human) | CYP1A2 |
| 8 | Cytochrome P450 1B1 | 2 | 1 | 1 | 1 | 0 | 0 | 0 | 2D 3D TSV | Q16678 | Cytochrome P450 1B1 | Homo sapiens (Human) | CYP1B1 |
| 9 | Cytochrome P450 2B6 | 2 | 1 | 1 | 1 | 0 | 0 | 0 | 2D 3D TSV | P20813 | Cytochrome P450 2B6 | Homo sapiens (Human) | CYP2B6 |
| 10 | Cytochrome P450 2C19 | 2 | 1 | 1 | 1 | 0 | 0 | 0 | 2D 3D TSV | P33261 | Cytochrome P450 2C19 | Homo sapiens (Human) | CYP2C19 |

| | | | | | | | | | | | | | |
|---|---|---|---|---|---|---|---|---|---|---|---|---|---|
| 11 | Cytochrome P450 3A4 | 2 | 1 | 1 | 1 | 0 | 0 | 0 | 2D 3D TSV | P08684 | Cytochrome P450 3A4 | Homo sapiens (Human) | CYP3A4 (CYP3A3) |
| 12 | Cytochrome P450 3A5 | 2 | 1 | 1 | 1 | 0 | 0 | 0 | 2D 3D TSV | P20815 | Cytochrome P450 3A5 | Homo sapiens (Human) | CYP3A5 |
| 13 | D(3) dopamine receptor | 3 | 0.95 | 4 | 0 | 0 | 0 | 4 | 2D 3D TSV | P35462 | D(3) dopamine receptor | Homo sapiens (Human) | DRD3 |
| 14 | Estrogen receptor | 2 | 0.85 | 4 | 0 | 4 | 0 | 0 | 2D 3D TSV | P03372 | Estrogen receptor | Homo sapiens (Human) | ESR1 (ESR, NR3A1) |
| 15 | Estrogen receptor beta | 2 | 0.85 | 5 | 0 | 5 | 0 | 0 | 2D 3D TSV | Q92731 | Estrogen receptor beta | Homo sapiens (Human) | ESR2 (ESTRB, NR3A2) |
| 16 | Fatty acid-binding protein, liver | 5 | 0.94 | 3 | 3 | 0 | 0 | 0 | 2D 3D TSV | NOT FOR HUMANS | | | |
| 17 | G-protein coupled receptor 55 | 2 | 0.85 | 2 | 0 | 0 | 0 | 2 | 2D 3D TSV | Q9Y2T6 | G-protein coupled receptor 55 | Homo sapiens (Human) | GPR55 |
| 18 | Heat sensitive channel TRPV3 | 2 | 1 | 1 | 0 | 1 | 0 | 1 | 2D 3D TSV | NOT FOR HUMANS | | | |
| 19 | Transient receptor potential cation channel subfamily A | 10 | 1 | 10 | 0 | 4 | 0 | 11 | 2D 3D TSV | NOT FOR HUMANS | | | |

| | | | | | | | | | | | | | |
|---|---|---|---|---|---|---|---|---|---|---|---|---|---|
| | member 1 | | | | | | | | | | | | |
| 20 | Transient receptor potential cation channel subfamily M member 8 | 5 | 1 | 5 | 0 | 5 | 0 | 0 | 2D 3D TSV | NOT FOR HUMANS | | | |
| 21 | Transient receptor potential cation channel subfamily V member 4 | 2 | 1 | 1 | 0 | 0 | 0 | 1 | 2D 3D TSV | NOT FOR HUMANS | | | |
| 22 | Vascular endothelial growth factor receptor 2 | 3 | 0.92 | 7 | 7 | 0 | 0 | 0 | 2D 3D TSV | P35968 | Vascular endothelial growth factor receptor 2 | Homo sapiens (Human) | KDR (FLK1, VEGFR2) |

**Table S2. Results of the SwissTargetPrediction.**

| Target | Common name | Uniprot ID | ChEMBL ID | Target Class | Probability* | Known actives (3D/2D) | Compound name |
|---|---|---|---|---|---|---|---|
| Cannabinoid receptor 1 | CNR1 | P21554 | CHEMBL218 | Family A G protein-coupled receptor | 0.218248 | 979 / 261Â Â Â Â Â | Cannabichromene |
| Cannabinoid receptor 2 | CNR2 | P34972 | CHEMBL253 | Family A G protein-coupled receptor | 0.176594 | 880 / 221Â Â Â Â Â | Cannabichromene |
| Arachidonate 5-lipoxygenase | ALOX5 | P09917 | CHEMBL215 | Oxidoreductase | 0.109946 | 112 / 27Â Â Â Â Â | Cannabichromene |
| 5-lipoxygenase activating protein | ALOX5AP | P20292 | CHEMBL4550 | Other cytosolic protein | 0.101614 | 73 / 0Â Â Â Â Â | Cannabichromene |
| 11-beta-hydroxysteroid dehydrogenase 1 | HSD11B1 | P28845 | CHEMBL4235 | Enzyme | 0.101614 | 240 / 0Â Â Â Â Â | Cannabichromene |
| Vascular endothelial growth factor receptor 2 | KDR | P35968 | CHEMBL279 | Kinase | 0.101614 | 337 / 8Â Â Â Â Â | Cannabichromene |

| | | | | | | | |
|---|---|---|---|---|---|---|---|
| Bile acid receptor FXR | NR1H4 | Q96RI1 | CHEMBL2047 | Nuclear receptor | 0.101614 | 40 / 0Â Â Â Â Â | Cannabichrome ne |
| Cystic fibrosis transmembrane conductance regulator | CFTR | P13569 | CHEMBL4051 | Other ion channel | 0.101614 | 11 / 0Â Â Â Â Â | Cannabichrome ne |
| Leucine-rich repeat serine/threonine-protein kinase 2 | LRRK2 | Q5S007 | CHEMBL1075104 | Kinase | 0.101614 | 46 / 0Â Â Â Â Â | Cannabichrome ne |
| NAD-dependent deacetylase sirtuin 2 | SIRT2 | Q8IXJ6 | CHEMBL4462 | Eraser | 0.101614 | 34 / 0Â Â Â Â Â | Cannabichrome ne |
| Corticotropin releasing factor receptor 1 | CRHR1 | P34998 | CHEMBL1800 | Family B G protein-coupled receptor | 0.101614 | 104 / 0Â Â Â Â Â | Cannabichrome ne |
| Calcium sensing receptor | CASR | P41180 | CHEMBL1878 | Family C G protein-coupled receptor | 0.101614 | 36 / 0Â Â Â Â Â | Cannabichrome ne |
| Phosphodiesterase 7A | PDE7A | Q13946 | CHEMBL3012 | Phosphodies terase | 0.101614 | 28 / 0Â Â Â Â Â | Cannabichrome ne |
| Pyroglutamylated RFamide peptide receptor | QRFPR | Q96P65 | CHEMBL5852 | Family A G protein-coupled receptor | 0.101614 | 9 / 0Â Â Â Â Â | Cannabichrome ne |
| Melatonin receptor 1B | MTNR1 B | P49286 | CHEMBL1946 | Family A G protein-coupled receptor | 0.101614 | 109 / 0Â Â Â Â Â | Cannabichrome ne |
| Kinesin-like protein 1 | KIF11 | P52732 | CHEMBL4581 | Other cytosolic protein | 0.101614 | 42 / 0Â Â Â Â Â | Cannabichrome ne |
| GABA-A receptor; alpha-3/beta-3/gamma-2 | GABRB 3 GABRA 3 GABRG 2 | P28472 P34903 P18507 | CHEMBL2094120 | Ligand-gated ion channel | 0.101614 | 120 / 0Â Â Â Â Â | Cannabichrome ne |
| GABA-A receptor; alpha-1/beta-3/gamma-2 | GABRB 3 GABRG 2 GABRA 1 | P28472 P18507 P14867 | CHEMBL2094121 | Ligand-gated ion channel | 0.101614 | 100 / 0Â Â Â Â Â | Cannabichrome ne |
| GABA-A receptor; alpha-5/beta-3/gamma-2 | GABRB 3 GABRG 2 GABRA 5 | P28472 P18507 P31644 | CHEMBL2094122 | Ligand-gated ion channel | 0.101614 | 92 / 0Â Â Â Â Â | Cannabichrome ne |
| GABA-A receptor; alpha-2/beta-3/gamma-2 | GABRA 2 GABRB 3 GABRG 2 | P47869 P28472 P18507 | CHEMBL2094130 | Ligand-gated ion channel | 0.101614 | 112 / 0Â Â Â Â Â | Cannabichrome ne |
| N-arachidonyl glycine receptor | GPR18 | Q14330 | CHEMBL2384898 | Family A G protein- | 0.101614 | 9 / 1Â Â Â Â Â | Cannabichrome ne |

| | | | | | | | |
|---|---|---|---|---|---|---|---|
| | | | | coupled receptor | | | |
| Glycine receptor subunit alpha-1 | GLRA1 | P23415 | CHEMBL5845 | Ligand-gated ion channel | 0.101614 | 1 / 1Â Â Â Â Â | Cannabichromene |
| Cyclooxygenase-1 | PTGS1 | P23219 | CHEMBL221 | Oxidoreductase | 0.101614 | 46 / 32Â Â Â Â Â | Cannabichromene |
| P2X purinoceptor 3 | P2RX3 | P56373 | CHEMBL2998 | Ligand-gated ion channel | 0.101614 | 38 / 0Â Â Â Â Â | Cannabichromene |
| Glucocorticoid receptor | NR3C1 | P04150 | CHEMBL2034 | Nuclear receptor | 0.101614 | 147 / 0Â Â Â Â Â | Cannabichromene |
| Progesterone receptor | PGR | P06401 | CHEMBL208 | Nuclear receptor | 0.101614 | 65 / 0Â Â Â Â Â | Cannabichromene |
| Tyrosine-protein kinase SRC | SRC | P12931 | CHEMBL267 | Kinase | 0.101614 | 133 / 1Â Â Â Â Â | Cannabichromene |
| Phosphodiesterase 5A | PDE5A | O76074 | CHEMBL1827 | Phosphodiesterase | 0.101614 | 183 / 0Â Â Â Â Â | Cannabichromene |
| Histone deacetylase 2 | HDAC2 | Q92769 | CHEMBL1937 | Eraser | 0.101614 | 36 / 0Â Â Â Â Â | Cannabichromene |
| Histone deacetylase 1 | HDAC1 | Q13547 | CHEMBL325 | Eraser | 0.101614 | 109 / 0Â Â Â Â Â | Cannabichromene |
| G-protein coupled receptor 55 | GPR55 | Q9Y2T6 | CHEMBL1075322 | Family A G protein-coupled receptor | 0.101614 | 15 / 0Â Â Â Â Â | Cannabichromene |
| Adenosine A1 receptor | ADORA1 | P30542 | CHEMBL226 | Family A G protein-coupled receptor | 0.101614 | 367 / 0Â Â Â Â Â | Cannabichromene |
| Adenosine A2a receptor | ADORA2A | P29274 | CHEMBL251 | Family A G protein-coupled receptor | 0.101614 | 284 / 0Â Â Â Â Â | Cannabichromene |
| Adenosine A3 receptor | ADORA3 | P0DMS8 | CHEMBL256 | Family A G protein-coupled receptor | 0.101614 | 265 / 0Â Â Â Â Â | Cannabichromene |
| Cytochrome b-c1 complex subunit 7 | UQCRB | P14927 | CHEMBL1671612 | Transmembrane 1-electron transfer carriers | 0.101614 | 1 / 0Â Â Â Â Â | Cannabichromene |
| ADAMTS5 | ADAMTS5 | Q9UNA0 | CHEMBL2285 | Protease | 0.101614 | 31 / 0Â Â Â Â Â | Cannabichromene |
| Translocator protein (by homology) | TSPO | P30536 | CHEMBL5742 | Membrane receptor | 0.101614 | 267 / 0Â Â Â Â Â | Cannabichromene |
| Serotonin 2c (5-HT2c) receptor | HTR2C | P28335 | CHEMBL225 | Family A G protein-coupled receptor | 0.101614 | 68 / 0Â Â Â Â Â | Cannabichromene |
| Estradiol 17-beta-dehydrogenase 2 | HSD17B2 | P37059 | CHEMBL2789 | Enzyme | 0.101614 | 21 / 0Â Â Â Â Â | Cannabichromene |
| Interleukin-8 receptor A | CXCR1 | P25024 | CHEMBL4029 | Family A G protein-coupled receptor | 0.101614 | 32 / 0Â Â Â Â Â | Cannabichromene |
| Pantothenate kinase 3 | PANK3 | Q9H999 | CHEMBL3407328 | Enzyme | 0.101614 | 7 / 0Â Â Â Â Â | Cannabichromene |

| | | | | | | | |
|---|---|---|---|---|---|---|---|
| Neuropeptide Y receptor type 5 | NPY5R | Q15761 | CHEMBL4561 | Family A G protein-coupled receptor | 0.101614 | 212 / 0Â Â Â Â Â | Cannabichromene |
| Purinergic receptor P2Y1 | P2RY1 | P47900 | CHEMBL4315 | Family A G protein-coupled receptor | 0.101614 | 23 / 0Â Â Â Â Â | Cannabichromene |
| Cathepsin K | CTSK | P43235 | CHEMBL268 | Protease | 0 | 324 / 1Â Â Â Â Â | Cannabichromene |
| Kinesin-1 heavy chain/ Tyrosine-protein kinase receptor RET | RET | P07949 | CHEMBL2041 | Kinase | 0 | 28 / 0Â Â Â Â Â | Cannabichromene |
| Epoxide hydratase | EPHX2 | P34913 | CHEMBL2409 | Protease | 0 | 222 / 0Â Â Â Â Â | Cannabichromene |
| Prenyl protein specific protease | RCE1 | Q9Y256 | CHEMBL3411 | Protease | 0 | 6 / 0Â Â Â Â Â | Cannabichromene |
| Methyl-CpG-binding protein 2 | MECP2 | P51608 | CHEMBL3638346 | Reader | 0 | 1 / 0Â Â Â Â Â | Cannabichromene |
| Methyl-CpG-binding domain protein 2 | MBD2 | Q9UBB5 | CHEMBL3707462 | Reader | 0 | 2 / 0Â Â Â Â Â | Cannabichromene |
| Receptor protein-tyrosine kinase erbB-2 | ERBB2 | P04626 | CHEMBL1824 | Kinase | 0 | 84 / 0Â Â Â Â Â | Cannabichromene |
| Vascular endothelial growth factor receptor 1 (by homology) | FLT1 | P17948 | CHEMBL1868 | Kinase | 0 | 89 / 0Â Â Â Â Â | Cannabichromene |
| Glutamate NMDA receptor; GRIN1/GRIN2B | GRIN1 GRIN2B | Q05586 Q13224 | CHEMBL1907603 | Ligand-gated ion channel | 0 | 3 / 0Â Â Â Â Â | Cannabichromene |
| Cyclophilin A | PPIA | P62937 | CHEMBL1949 | Isomerase | 0 | 39 / 0Â Â Â Â Â | Cannabichromene |
| Hexokinase type IV | GCK | P35557 | CHEMBL3820 | Enzyme | 0 | 96 / 0Â Â Â Â Â | Cannabichromene |
| Phosphodiesterase 10A (by homology) | PDE10A | Q9Y233 | CHEMBL4409 | Phosphodiesterase | 0 | 909 / 0Â Â Â Â Â | Cannabichromene |
| Phosphodiesterase 2A | PDE2A | O00408 | CHEMBL2652 | Phosphodiesterase | 0 | 53 / 0Â Â Â Â Â | Cannabichromene |
| Cholecystokinin B receptor | CCKBR | P32239 | CHEMBL298 | Family A G protein-coupled receptor | 0 | 386 / 0Â Â Â Â Â | Cannabichromene |
| Carnitine O-palmitoyltransferase 1, liver isoform | CPT1A | P50416 | CHEMBL1293194 | Enzyme | 0 | 91 / 0Â Â Â Â Â | Cannabichromene |
| GABA-A receptor; GABA-A site (alpha1/beta2 interface) | GABRA1 GABRB2 | P14867 P47870 | CHEMBL1907597 | Ligand-gated ion channel | 0 | 5 / 0Â Â Â Â Â | Cannabichromene |
| Dopamine D3 receptor | DRD3 | P35462 | CHEMBL234 | Family A G protein-coupled receptor | 0 | 90 / 0Â Â Â Â Â | Cannabichromene |
| LDL-associated phospholipase A2 | PLA2G7 | Q13093 | CHEMBL3514 | Enzyme | 0 | 32 / 0Â Â Â Â Â | Cannabichromene |
| Acyl-CoA desaturase (by homology) | SCD | O00767 | CHEMBL5555 | Enzyme | 0 | 32 / 0Â Â Â Â Â | Cannabichromene |

| | | | | | | | |
|---|---|---|---|---|---|---|---|
| Histone deacetylase 6 | HDAC6 | Q9UBN7 | CHEMBL1865 | Eraser | 0 | 67 / 0Â Â Â Â Â | Cannabichromene |
| Muscarinic acetylcholine receptor M1 (by homology) | CHRM1 | P11229 | CHEMBL216 | Family A G protein-coupled receptor | 0 | 55 / 0Â Â Â Â Â | Cannabichromene |
| Beta secretase 2 | BACE2 | Q9Y5Z0 | CHEMBL2525 | Protease | 0 | 106 / 0Â Â Â Â Â | Cannabichromene |
| Histone deacetylase 8 | HDAC8 | Q9BY41 | CHEMBL3192 | Eraser | 0 | 22 / 0Â Â Â Â Â | Cannabichromene |
| Sphingosine 1-phosphate receptor Edg-3 | S1PR3 | Q99500 | CHEMBL3892 | Family A G protein-coupled receptor | 0 | 91 / 0Â Â Â Â Â | Cannabichromene |
| Orexin receptor 2 | HCRTR2 | O43614 | CHEMBL4792 | Family A G protein-coupled receptor | 0 | 561 / 0Â Â Â Â Â | Cannabichromene |
| Orexin receptor 1 | HCRTR1 | O43613 | CHEMBL5113 | Family A G protein-coupled receptor | 0 | 495 / 0Â Â Â Â Â | Cannabichromene |
| Fibroblast growth factor receptor 1 | FGFR1 | P11362 | CHEMBL3650 | Kinase | 0 | 54 / 1Â Â Â Â Â | Cannabichromene |
| Dihydroorotate dehydrogenase | DHODH | Q02127 | CHEMBL1966 | Oxidoreductase | 0 | 10 / 0Â Â Â Â Â | Cannabichromene |
| Isocitrate dehydrogenase [NADP] cytoplasmic | IDH1 | O75874 | CHEMBL2007625 | Enzyme | 0 | 94 / 0Â Â Â Â Â | Cannabichromene |
| Metabotropic glutamate receptor 5 | GRM5 | P41594 | CHEMBL3227 | Family C G protein-coupled receptor | 0 | 534 / 0Â Â Â Â Â | Cannabichromene |
| Serotonin 5a (5-HT5a) receptor | HTR5A | P47898 | CHEMBL3426 | Family A G protein-coupled receptor | 0 | 8 / 0Â Â Â Â Â | Cannabichromene |
| Neurokinin 2 receptor | TACR2 | P21452 | CHEMBL2327 | Family A G protein-coupled receptor | 0 | 24 / 0Â Â Â Â Â | Cannabichromene |
| Cyclin-dependent kinase 4 | CDK4 | P11802 | CHEMBL331 | Kinase | 0 | 45 / 0Â Â Â Â Â | Cannabichromene |
| Heat shock protein HSP 90-alpha | HSP90AA1 | P07900 | CHEMBL3880 | Other cytosolic protein | 0 | 40 / 0Â Â Â Â Â | Cannabichromene |
| Neurokinin 3 receptor | TACR3 | P29371 | CHEMBL4429 | Family A G protein-coupled receptor | 0 | 134 / 0Â Â Â Â Â | Cannabichromene |
| Tankyrase-2 | TNKS2 | Q9H2K2 | CHEMBL6154 | Enzyme | 0 | 26 / 0Â Â Â Â Â | Cannabichromene |
| Tyrosine-protein kinase ABL | ABL1 | P00519 | CHEMBL1862 | Kinase | 0 | 125 / 0Â Â Â Â Â | Cannabichromene |
| P2X purinoceptor 7 | P2RX7 | Q99572 | CHEMBL4805 | Ligand-gated ion channel | 0 | 136 / 0Â Â Â Â Â | Cannabichromene |
| Hepatocyte growth factor receptor | MET | P08581 | CHEMBL3717 | Kinase | 0 | 209 / 5Â Â Â Â Â | Cannabichromene |

| | | | | | | | |
|---|---|---|---|---|---|---|---|
| FK506-binding protein 1A | FKBP1A | P62942 | CHEMBL1902 | Isomerase | 0 | 82 / 0Â Â Â Â Â | Cannabichromene |
| Phosphodiesterase 4B | PDE4B | Q07343 | CHEMBL275 | Phosphodiesterase | 0 | 82 / 0Â Â Â Â Â | Cannabichromene |
| Cyclin-dependent kinase 2 | CDK2 | P24941 | CHEMBL301 | Kinase | 0 | 92 / 0Â Â Â Â Â | Cannabichromene |
| Cyclin-dependent kinase 1 | CDK1 | P06493 | CHEMBL308 | Kinase | 0 | 60 / 0Â Â Â Â Â | Cannabichromene |
| Glutaminyl-peptide cyclotransferase | QPCT | Q16769 | CHEMBL4508 | Enzyme | 0 | 32 / 0Â Â Â Â Â | Cannabichromene |
| Sodium channel protein type V alpha subunit | SCN5A | Q14524 | CHEMBL1980 | Voltage-gated ion channel | 0 | 23 / 0Â Â Â Â Â | Cannabichromene |
| Anandamide amidohydrolase | FAAH | O00519 | CHEMBL2243 | Enzyme | 0 | 199 / 0Â Â Â Â Â | Cannabichromene |
| Matrix metalloproteinase 3 | MMP3 | P08254 | CHEMBL283 | Protease | 0 | 49 / 0Â Â Â Â Â | Cannabichromene |
| Matrix metalloproteinase 1 | MMP1 | P03956 | CHEMBL332 | Protease | 0 | 57 / 0Â Â Â Â Â | Cannabichromene |
| Sodium channel protein type II alpha subunit | SCN2A | Q99250 | CHEMBL4187 | Voltage-gated ion channel | 0 | 32 / 0Â Â Â Â Â | Cannabichromene |
| Glucagon receptor | GCGR | P47871 | CHEMBL1985 | Family B G protein-coupled receptor | 0 | 43 / 0Â Â Â Â Â | Cannabichromene |
| Gamma-secretase | PSEN2 PSENEN NCSTN APH1A PSEN1 APH1B | P49810 Q9NZ42 Q92542 Q96BI3 P49768 Q8WW43 | CHEMBL2094135 | Protease | 0 | 176 / 0Â Â Â Â Â | Cannabichromene |
| 6-phosphofructo-2-kinase/fructose-2,6-bisphosphatase 3 | PFKFB3 | Q16875 | CHEMBL2331053 | Enzyme | 0 | 90 / 0Â Â Â Â Â | Cannabichromene |
| MAP kinase p38 alpha | MAPK14 | Q16539 | CHEMBL260 | Kinase | 0 | 447 / 0Â Â Â Â Â | Cannabichromene |
| Serotonin 6 (5-HT6) receptor | HTR6 | P50406 | CHEMBL3371 | Family A G protein-coupled receptor | 0 | 118 / 0Â Â Â Â Â | Cannabichromene |
| Bombesin receptor subtype-3 | BRS3 | P32247 | CHEMBL4080 | Family A G protein-coupled receptor | 0 | 5 / 0Â Â Â Â Â | Cannabichromene |
| G-protein coupled bile acid receptor 1 | GPBAR1 | Q8TDU6 | CHEMBL5409 | Family A G protein-coupled receptor | 0 | 35 / 0Â Â Â Â Â | Cannabichromene |
| Elongation of very long chain fatty acids protein 6 | ELOVL6 | Q9H5J4 | CHEMBL5704 | Enzyme | 0 | 17 / 0Â Â Â Â Â | Cannabichromene |
| | | | | | | | |
| Cannabinoid receptor 1 | CNR1 | P21554 | CHEMBL218 | Family A G protein-coupled receptor | 0.759876 | 846 / 386Â Â Â Â Â | Cannabicyclol |

| | | | | | | | |
|---|---|---|---|---|---|---|---|
| Cannabinoid receptor 2 | CNR2 | P34972 | CHEMBL253 | Family A G protein-coupled receptor | 0.759876 | 755 / 379Â Â Â Â Â | Cannabicyclol |
| Vascular endothelial growth factor receptor 2 | KDR | P35968 | CHEMBL279 | Kinase | 0.134939 | 183 / 12Â Â Â Â Â | Cannabicyclol |
| N-arachidonyl glycine receptor | GPR18 | Q14330 | CHEMBL2384898 | Family A G protein-coupled receptor | 0.12661 | 2 / 2Â Â Â Â Â | Cannabicyclol |
| Glycine receptor subunit alpha-1 | GLRA1 | P23415 | CHEMBL5845 | Ligand-gated ion channel | 0.12661 | 1 / 2Â Â Â Â Â | Cannabicyclol |
| PH domain leucine-rich repeat-containing protein phosphatase 1 | PHLPP1 | O60346 | CHEMBL3414405 | Reader | 0.109946 | 0 / 3Â Â Â Â Â | Cannabicyclol |
| Serine/threonine-protein kinase ILK-1 | ILK | Q13418 | CHEMBL5247 | Kinase | 0.109946 | 0 / 2Â Â Â Â Â | Cannabicyclol |
| Serine/threonine-protein kinase AKT | AKT1 | P31749 | CHEMBL4282 | Kinase | 0.101614 | 14 / 19Â Â Â Â Â | Cannabicyclol |
| Arachidonate 5-lipoxygenase | ALOX5 | P09917 | CHEMBL215 | Oxidoreductase | 0.101614 | 51 / 26Â Â Â Â Â | Cannabicyclol |
| Cystic fibrosis transmembrane conductance regulator | CFTR | P13569 | CHEMBL4051 | Other ion channel | 0.101614 | 4 / 0Â Â Â Â Â | Cannabicyclol |
| Estrogen receptor beta | ESR2 | Q92731 | CHEMBL242 | Nuclear receptor | 0.101614 | 19 / 72Â Â Â Â Â | Cannabicyclol |
| Glucagon receptor | GCGR | P47871 | CHEMBL1985 | Family B G protein-coupled receptor | 0.101614 | 30 / 0Â Â Â Â Â | Cannabicyclol |
| Cathepsin D | CTSD | P07339 | CHEMBL2581 | Protease | 0.101614 | 33 / 0Â Â Â Â Â | Cannabicyclol |
| G-protein coupled receptor 55 | GPR55 | Q9Y2T6 | CHEMBL1075322 | Family A G protein-coupled receptor | 0.101614 | 13 / 1Â Â Â Â Â | Cannabicyclol |
| Presenilin 1 | PSEN1 | P49768 | CHEMBL2473 | Other ion channel | 0.101614 | 0 / 7Â Â Â Â Â | Cannabicyclol |
| Estrogen receptor alpha | ESR1 | P03372 | CHEMBL206 | Nuclear receptor | 0.101614 | 22 / 76Â Â Â Â Â | Cannabicyclol |
| NAD-dependent deacetylase sirtuin 2 | SIRT2 | Q8IXJ6 | CHEMBL4462 | Eraser | 0.101614 | 23 / 19Â Â Â Â Â | Cannabicyclol |
| Protein kinase C (PKC) | PRKCZ | Q05513 | CHEMBL3438 | Kinase | 0.101614 | 2 / 0Â Â Â Â Â | Cannabicyclol |
| Nitric oxide synthase, inducible (by homology) | NOS2 | P35228 | CHEMBL4481 | Enzyme | 0.101614 | 80 / 0Â Â Â Â Â | Cannabicyclol |
| Kinesin-like protein 1 | KIF11 | P52732 | CHEMBL4581 | Other cytosolic protein | 0.101614 | 35 / 0Â Â Â Â Â | Cannabicyclol |
| Bile acid receptor FXR | NR1H4 | Q96RI1 | CHEMBL2047 | Nuclear receptor | 0.101614 | 30 / 18Â Â Â Â Â | Cannabicyclol |
| Prostaglandin E synthase | PTGES | O14684 | CHEMBL5658 | Enzyme | 0.101614 | 24 / 8Â Â Â Â Â | Cannabicyclol |

| | | | | | | | |
|---|---|---|---|---|---|---|---|
| Voltage-gated potassium channel, KQT; KCNQ2(Kv7.2)/KCNQ3(Kv7.3) | KCNQ3 KCNQ2 | O43525 O43526 | CHEMBL2221348 | Voltage-gated ion channel | 0.101614 | 8 / 0Â Â Â Â Â | Cannabicyclol |
| Quinone reductase 2 | NQO2 | P16083 | CHEMBL3959 | Enzyme | 0.101614 | 9 / 0Â Â Â Â Â | Cannabicyclol |
| Corticotropin releasing factor receptor 1 | CRHR1 | P34998 | CHEMBL1800 | Family B G protein-coupled receptor | 0.101614 | 107 / 0Â Â Â Â Â | Cannabicyclol |
| Voltage-gated potassium channel subunit Kv1.5 | KCNA5 | P22460 | CHEMBL4306 | Voltage-gated ion channel | 0.101614 | 109 / 0Â Â Â Â Â | Cannabicyclol |
| Beta secretase 2 | BACE2 | Q9Y5Z0 | CHEMBL2525 | Protease | 0.101614 | 50 / 0Â Â Â Â Â | Cannabicyclol |
| Thromboxane A2 receptor | TBXA2R | P21731 | CHEMBL2069 | Family A G protein-coupled receptor | 0.101614 | 6 / 0Â Â Â Â Â | Cannabicyclol |
| Prenyl protein specific protease | RCE1 | Q9Y256 | CHEMBL3411 | Protease | 0.101614 | 6 / 0Â Â Â Â Â | Cannabicyclol |
| Peregrin | BRPF1 | P55201 | CHEMBL3132741 | Reader | 0.101614 | 28 / 0Â Â Â Â Â | Cannabicyclol |
| Hormone sensitive lipase | LIPE | Q05469 | CHEMBL3590 | Enzyme | 0.101614 | 51 / 0Â Â Â Â Â | Cannabicyclol |
| Beta-secretase 1 | BACE1 | P56817 | CHEMBL4822 | Protease | 0.101614 | 202 / 12Â Â Â Â Â | Cannabicyclol |
| Thymidylate synthase | TYMS | P04818 | CHEMBL1952 | Transferase | 0.101614 | 19 / 0Â Â Â Â Â | Cannabicyclol |
| Poly [ADP-ribose] polymerase-1 | PARP1 | P09874 | CHEMBL3105 | Enzyme | 0.101614 | 24 / 0Â Â Â Â Â | Cannabicyclol |
| LDL-associated phospholipase A2 | PLA2G7 | Q13093 | CHEMBL3514 | Enzyme | 0.101614 | 17 / 0Â Â Â Â Â | Cannabicyclol |
| Protoporphyrinogen oxidase | PPOX | P50336 | CHEMBL1926488 | Oxidoreductase | 0.101614 | 2 / 0Â Â Â Â Â | Cannabicyclol |
| ADAMTS5 | ADAMTS5 | Q9UNA0 | CHEMBL2285 | Protease | 0.101614 | 19 / 0Â Â Â Â Â | Cannabicyclol |
| Melanocortin receptor 4 | MC4R | P32245 | CHEMBL259 | Family A G protein-coupled receptor | 0.101614 | 13 / 0Â Â Â Â Â | Cannabicyclol |
| Arachidonate 15-lipoxygenase | ALOX15 | P16050 | CHEMBL2903 | Enzyme | 0.101614 | 19 / 12Â Â Â Â Â | Cannabicyclol |
| Isocitrate dehydrogenase [NADP] cytoplasmic | IDH1 | O75874 | CHEMBL2007625 | Enzyme | 0.101614 | 179 / 0Â Â Â Â Â | Cannabicyclol |
| Neurokinin 2 receptor | TACR2 | P21452 | CHEMBL2327 | Family A G protein-coupled receptor | 0.101614 | 10 / 0Â Â Â Â Â | Cannabicyclol |
| Neurokinin 3 receptor | TACR3 | P29371 | CHEMBL4429 | Family A G protein-coupled receptor | 0.101614 | 110 / 0Â Â Â Â Â | Cannabicyclol |
| Protein-tyrosine phosphatase 1B | PTPN1 | P18031 | CHEMBL335 | Phosphatase | 0.101614 | 7 / 6Â Â Â Â Â | Cannabicyclol |
| Methyl-CpG-binding protein 2 | MECP2 | P51608 | CHEMBL3638346 | Reader | 0 | 1 / 0Â Â Â Â Â | Cannabicyclol |

| | | | | | | | |
|---|---|---|---|---|---|---|---|
| Methyl-CpG-binding domain protein 2 | MBD2 | Q9UBB5 | CHEMBL3707462 | Reader | 0 | 2 / 0Â Â Â Â Â | Cannabicyclol |
| Serine/threonine-protein kinase PAK 1 | PAK1 | Q13153 | CHEMBL4600 | Kinase | 0 | 2 / 0Â Â Â Â Â | Cannabicyclol |
| Vanilloid receptor | TRPV1 | Q8NER1 | CHEMBL4794 | Voltage-gated ion channel | 0 | 145 / 2Â Â Â Â Â | Cannabicyclol |
| Leucine-rich repeat serine/threonine-protein kinase 2 | LRRK2 | Q5S007 | CHEMBL1075104 | Kinase | 0 | 24 / 0Â Â Â Â Â | Cannabicyclol |
| Histone deacetylase 2 | HDAC2 | Q92769 | CHEMBL1937 | Eraser | 0 | 10 / 0Â Â Â Â Â | Cannabicyclol |
| Metabotropic glutamate receptor 2 | GRM2 | Q14416 | CHEMBL5137 | Family C G protein-coupled receptor | 0 | 28 / 63Â Â Â Â Â | Cannabicyclol |
| Adenosine A2a receptor | ADORA2A | P29274 | CHEMBL251 | Family A G protein-coupled receptor | 0 | 113 / 0Â Â Â Â Â | Cannabicyclol |
| Sodium channel protein type II alpha subunit | SCN2A | Q99250 | CHEMBL4187 | Voltage-gated ion channel | 0 | 29 / 0Â Â Â Â Â | Cannabicyclol |
| Translocator protein (by homology) | TSPO | P30536 | CHEMBL5742 | Membrane receptor | 0 | 350 / 0Â Â Â Â Â | Cannabicyclol |
| Cathepsin K | CTSK | P43235 | CHEMBL268 | Protease | 0 | 168 / 0Â Â Â Â Â | Cannabicyclol |
| Serotonin 2a (5-HT2a) receptor (by homology) | HTR2A | P28223 | CHEMBL224 | Family A G protein-coupled receptor | 0 | 40 / 101Â Â Â Â Â | Cannabicyclol |
| Bombesin receptor subtype-3 | BRS3 | P32247 | CHEMBL4080 | Family A G protein-coupled receptor | 0 | 1 / 0Â Â Â Â Â | Cannabicyclol |
| p53-binding protein Mdm-2 | MDM2 | Q00987 | CHEMBL5023 | Other nuclear protein | 0 | 72 / 0Â Â Â Â Â | Cannabicyclol |
| Egl nine homolog 1 | EGLN1 | Q9GZT9 | CHEMBL5697 | Oxidoreductase | 0 | 5 / 0Â Â Â Â Â | Cannabicyclol |
| GABA-A receptor; GABA-A site (alpha1/beta2 interface) | GABRA1 GABRB2 | P14867 P47870 | CHEMBL1907597 | Ligand-gated ion channel | 0 | 4 / 0Â Â Â Â Â | Cannabicyclol |
| Phosphodiesterase 2A | PDE2A | O00408 | CHEMBL2652 | Phosphodiesterase | 0 | 52 / 0Â Â Â Â Â | Cannabicyclol |
| Phosphodiesterase 10A | PDE10A | Q9Y233 | CHEMBL4409 | Phosphodiesterase | 0 | 364 / 0Â Â Â Â Â | Cannabicyclol |
| Anandamide amidohydrolase | FAAH | O00519 | CHEMBL2243 | Enzyme | 0 | 80 / 0Â Â Â Â Â | Cannabicyclol |
| Cyclin-dependent kinase 5/CDK5 activator 1 | CDK5R1 CDK5 | Q15078 Q00535 | CHEMBL1907600 | Kinase | 0 | 74 / 0Â Â Â Â Â | Cannabicyclol |
| Cyclin-dependent kinase 1/cyclin B | CCNB3 CDK1 CCNB1 CCNB2 | Q8WWL7 P06493 P14635 O95067 | CHEMBL2094127 | Other cytosolic protein | 0 | 40 / 0Â Â Â Â Â | Cannabicyclol |

| | | | | | | | |
|---|---|---|---|---|---|---|---|
| Tyrosine-protein kinase JAK3 | JAK3 | P52333 | CHEMBL2148 | Kinase | 0 | 183 / 0Â Â Â Â Â | Cannabicyclol |
| Tyrosine-protein kinase JAK1 | JAK1 | P23458 | CHEMBL2835 | Kinase | 0 | 65 / 0Â Â Â Â Â | Cannabicyclol |
| Tyrosine-protein kinase JAK2 | JAK2 | O60674 | CHEMBL2971 | Kinase | 0 | 147 / 0Â Â Â Â Â | Cannabicyclol |
| JAK3/JAK1 | JAK3 JAK1 | P52333 P23458 | CHEMBL3038491 | Kinase | 0 | 16 / 0Â Â Â Â Â | Cannabicyclol |
| Peroxisome proliferator-activated receptor alpha | PPARA | Q07869 | CHEMBL239 | Nuclear receptor | 0 | 0 / 62Â Â Â Â Â | Cannabicyclol |
| G-protein coupled bile acid receptor 1 | GPBAR1 | Q8TDU 6 | CHEMBL5409 | Family A G protein-coupled receptor | 0 | 30 / 0Â Â Â Â Â | Cannabicyclol |
| Cytochrome b-c1 complex subunit 7 | UQCRB | P14927 | CHEMBL1671612 | Transmembrane 1-electron transfer carriers | 0 | 1 / 0Â Â Â Â Â | Cannabicyclol |
| Phosphodiesterase 7A | PDE7A | Q13946 | CHEMBL3012 | Phosphodiesterase | 0 | 13 / 0Â Â Â Â Â | Cannabicyclol |
| Leukotriene A4 hydrolase | LTA4H | P09960 | CHEMBL4618 | Protease | 0 | 10 / 0Â Â Â Â Â | Cannabicyclol |
| L-lactate dehydrogenase A chain | LDHA | P00338 | CHEMBL4835 | Enzyme | 0 | 7 / 0Â Â Â Â Â | Cannabicyclol |
| Serine/threonine-protein kinase Aurora-B | AURKB | Q96GD 4 | CHEMBL2185 | Kinase | 0 | 21 / 0Â Â Â Â Â | Cannabicyclol |
| Focal adhesion kinase 1 | PTK2 | Q05397 | CHEMBL2695 | Kinase | 0 | 21 / 0Â Â Â Â Â | Cannabicyclol |
| Serine/threonine-protein kinase PLK1 | PLK1 | P53350 | CHEMBL3024 | Kinase | 0 | 15 / 0Â Â Â Â Â | Cannabicyclol |
| Histone deacetylase 4 | HDAC4 | P56524 | CHEMBL3524 | Eraser | 0 | 6 / 0Â Â Â Â Â | Cannabicyclol |
| Histone deacetylase 10 | HDAC10 | Q969S8 | CHEMBL5103 | Eraser | 0 | 4 / 0Â Â Â Â Â | Cannabicyclol |
| GABA-A receptor; alpha-3/beta-3/gamma-2 | GABRB 3 GABRA 3 GABRG 2 | P28472 P34903 P18507 | CHEMBL2094120 | Ligand-gated ion channel | 0 | 71 / 0Â Â Â Â Â | Cannabicyclol |
| GABA-A receptor; alpha-1/beta-3/gamma-2 | GABRB 3 GABRG 2 GABRA 1 | P28472 P18507 P14867 | CHEMBL2094121 | Ligand-gated ion channel | 0 | 57 / 0Â Â Â Â Â | Cannabicyclol |
| GABA-A receptor; alpha-5/beta-3/gamma-2 | GABRB 3 GABRG 2 GABRA 5 | P28472 P18507 P31644 | CHEMBL2094122 | Ligand-gated ion channel | 0 | 57 / 0Â Â Â Â Â | Cannabicyclol |
| GABA-A receptor; alpha-2/beta-3/gamma-2 | GABRA 2 GABRB | P47869 P28472 P18507 | CHEMBL2094130 | Ligand-gated ion channel | 0 | 64 / 0Â Â Â Â Â | Cannabicyclol |

| | | | | | | | |
|---|---|---|---|---|---|---|---|
| | 3 GABRG 2 | | | | | | |
| Cholecystokinin B receptor | CCKBR | P32239 | CHEMBL298 | Family A G protein-coupled receptor | 0 | 244 / 0Â Â Â Â Â | Cannabicyclol |
| Melatonin receptor 1B | MTNR1 B | P49286 | CHEMBL1946 | Family A G protein-coupled receptor | 0 | 86 / 23Â Â Â Â Â | Cannabicyclol |
| Glutamate NMDA receptor; GRIN1/GRIN2B | GRIN1 GRIN2B | Q05586 Q13224 | CHEMBL1907603 | Ligand-gated ion channel | 0 | 2 / 0Â Â Â Â Â | Cannabicyclol |
| Muscarinic acetylcholine receptor M3 | CHRM3 | P20309 | CHEMBL245 | Family A G protein-coupled receptor | 0 | 69 / 0Â Â Â Â Â | Cannabicyclol |
| P2X purinoceptor 3 | P2RX3 | P56373 | CHEMBL2998 | Ligand-gated ion channel | 0 | 47 / 0Â Â Â Â Â | Cannabicyclol |
| Methionine aminopeptidase 2 | METAP 2 | P50579 | CHEMBL3922 | Protease | 0 | 4 / 0Â Â Â Â Â | Cannabicyclol |
| Orexin receptor 1 | HCRTR1 | O43613 | CHEMBL5113 | Family A G protein-coupled receptor | 0 | 246 / 0Â Â Â Â Â | Cannabicyclol |
| Oxytocin receptor (by homology) | OXTR | P30559 | CHEMBL2049 | Family A G protein-coupled receptor | 0 | 51 / 0Â Â Â Â Â | Cannabicyclol |
| Alpha-1,6-mannosyl-glycoprotein 2-beta-N-acetylglucosaminyltransferase | MGAT2 | Q10469 | CHEMBL2321630 | Enzyme | 0 | 22 / 0Â Â Â Â Â | Cannabicyclol |
| Histone deacetylase 6 | HDAC6 | Q9UBN 7 | CHEMBL1865 | Eraser | 0 | 25 / 3Â Â Â Â Â | Cannabicyclol |
| Histone deacetylase 8 | HDAC8 | Q9BY4 1 | CHEMBL3192 | Eraser | 0 | 11 / 3Â Â Â Â Â | Cannabicyclol |
| Dihydroorotate dehydrogenase | DHODH | Q02127 | CHEMBL1966 | Oxidoreductase | 0 | 6 / 0Â Â Â Â Â | Cannabicyclol |
| Thrombin and coagulation factor X | F10 | P00742 | CHEMBL244 | Protease | 0 | 98 / 0Â Â Â Â Â | Cannabicyclol |
| Pyroglutamylated RFamide peptide receptor | QRFPR | Q96P65 | CHEMBL5852 | Family A G protein-coupled receptor | 0 | 8 / 0Â Â Â Â Â | Cannabicyclol |
| Carnitine O-palmitoyltransferase 1, liver isoform | CPT1A | P50416 | CHEMBL1293194 | Enzyme | 0 | 41 / 0Â Â Â Â Â | Cannabicyclol |
| Lysosomal Pro-X carboxypeptidase | PRCP | P42785 | CHEMBL2335 | Protease | 0 | 11 / 0Â Â Â Â Â | Cannabicyclol |
| Carboxypeptidase B | CPB1 | P15086 | CHEMBL2552 | Protease | 0 | 3 / 0Â Â Â Â Â | Cannabicyclol |
| | | | | | | | |
| Cannabinoid receptor 1 | CNR1 | P21554 | CHEMBL218 | Family A G protein- | 0.893165 | 900 / 210Â Â Â Â Â | Cannabidiol |

| | | | | | | | |
|---|---|---|---|---|---|---|---|
| | | | | coupled receptor | | | |
| Cannabinoid receptor 2 | CNR2 | P34972 | CHEMBL253 | Family A G protein-coupled receptor | 0.893165 | 792 / 174Â Â Â Â Â | Cannabidiol |
| G-protein coupled receptor 55 | GPR55 | Q9Y2T6 | CHEMBL1075322 | Family A G protein-coupled receptor | 0.818184 | 14 / 2Â Â Â Â Â | Cannabidiol |
| Arachidonate 5-lipoxygenase | ALOX5 | P09917 | CHEMBL215 | Oxidoreductase | 0.101614 | 63 / 19Â Â Â Â Â | Cannabidiol |
| Cystic fibrosis transmembrane conductance regulator | CFTR | P13569 | CHEMBL4051 | Other ion channel | 0.101614 | 13 / 0Â Â Â Â Â | Cannabidiol |
| Receptor protein-tyrosine kinase erbB-2 | ERBB2 | P04626 | CHEMBL1824 | Kinase | 0.101614 | 48 / 0Â Â Â Â Â | Cannabidiol |
| NAD-dependent deacetylase sirtuin 2 | SIRT2 | Q8IXJ6 | CHEMBL4462 | Eraser | 0.101614 | 27 / 0Â Â Â Â Â | Cannabidiol |
| N-arachidonyl glycine receptor | GPR18 | Q14330 | CHEMBL2384898 | Family A G protein-coupled receptor | 0.101614 | 2 / 2Â Â Â Â Â | Cannabidiol |
| Glycine receptor subunit alpha-1 | GLRA1 | P23415 | CHEMBL5845 | Ligand-gated ion channel | 0.101614 | 1 / 1Â Â Â Â Â | Cannabidiol |
| DNA polymerase beta (by homology) | POLB | P06746 | CHEMBL2392 | Enzyme | 0.101614 | 0 / 3Â Â Â Â Â | Cannabidiol |
| Calcium sensing receptor | CASR | P41180 | CHEMBL1878 | Family C G protein-coupled receptor | 0.101614 | 38 / 0Â Â Â Â Â | Cannabidiol |
| Arachidonate 15-lipoxygenase | ALOX15 | P16050 | CHEMBL2903 | Enzyme | 0.101614 | 26 / 11Â Â Â Â Â | Cannabidiol |
| Cathepsin D | CTSD | P07339 | CHEMBL2581 | Protease | 0.101614 | 34 / 0Â Â Â Â Â | Cannabidiol |
| Beta-secretase 1 | BACE1 | P56817 | CHEMBL4822 | Protease | 0.101614 | 209 / 0Â Â Â Â Â | Cannabidiol |
| Vascular endothelial growth factor receptor 2 | KDR | P35968 | CHEMBL279 | Kinase | 0.101614 | 259 / 12Â Â Â Â Â | Cannabidiol |
| Neurokinin 3 receptor | TACR3 | P29371 | CHEMBL4429 | Family A G protein-coupled receptor | 0.101614 | 116 / 0Â Â Â Â Â | Cannabidiol |
| Cyclooxygenase-2 | PTGS2 | P35354 | CHEMBL230 | Oxidoreductase | 0.101614 | 132 / 3Â Â Â Â Â | Cannabidiol |
| Poly [ADP-ribose] polymerase-1 | PARP1 | P09874 | CHEMBL3105 | Enzyme | 0.101614 | 23 / 0Â Â Â Â Â | Cannabidiol |
| Prenyl protein specific protease | RCE1 | Q9Y256 | CHEMBL3411 | Protease | 0.101614 | 6 / 0Â Â Â Â Â | Cannabidiol |
| Bile acid receptor FXR | NR1H4 | Q96RI1 | CHEMBL2047 | Nuclear receptor | 0.101614 | 30 / 0Â Â Â Â Â | Cannabidiol |
| Histone deacetylase 6 | HDAC6 | Q9UBN7 | CHEMBL1865 | Eraser | 0.101614 | 23 / 0Â Â Â Â Â | Cannabidiol |
| Histone deacetylase 8 | HDAC8 | Q9BY41 | CHEMBL3192 | Eraser | 0.101614 | 10 / 0Â Â Â Â Â | Cannabidiol |

| | | | | | | | |
|---|---|---|---|---|---|---|---|
| Corticotropin releasing factor receptor 1 | CRHR1 | P34998 | CHEMBL1800 | Family B G protein-coupled receptor | 0.101614 | 130 / 0Â Â Â Â Â | Cannabidiol |
| Phosphodiesterase 4B | PDE4B | Q07343 | CHEMBL275 | Phosphodiesterase | 0.101614 | 31 / 0Â Â Â Â Â | Cannabidiol |
| ADAMTS5 | ADAMTS5 | Q9UNA0 | CHEMBL2285 | Protease | 0.101614 | 33 / 0Â Â Â Â Â | Cannabidiol |
| Epoxide hydratase | EPHX2 | P34913 | CHEMBL2409 | Protease | 0.101614 | 129 / 0Â Â Â Â Â | Cannabidiol |
| 11-beta-hydroxysteroid dehydrogenase 1 | HSD11B1 | P28845 | CHEMBL4235 | Enzyme | 0.101614 | 275 / 0Â Â Â Â Â | Cannabidiol |
| Translocator protein (by homology) | TSPO | P30536 | CHEMBL5742 | Membrane receptor | 0.101614 | 352 / 0Â Â Â Â Â | Cannabidiol |
| Serotonin 2b (5-HT2b) receptor | HTR2B | P41595 | CHEMBL1833 | Family A G protein-coupled receptor | 0.101614 | 40 / 0Â Â Â Â Â | Cannabidiol |
| Cholesteryl ester transfer protein | CETP | P11597 | CHEMBL3572 | Other ion channel | 0.101614 | 7 / 2Â Â Â Â Â | Cannabidiol |
| Quinone reductase 2 | NQO2 | P16083 | CHEMBL3959 | Enzyme | 0.101614 | 8 / 0Â Â Â Â Â | Cannabidiol |
| Prostanoid EP1 receptor | PTGER1 | P34995 | CHEMBL1811 | Family A G protein-coupled receptor | 0.101614 | 46 / 0Â Â Â Â Â | Cannabidiol |
| Voltage-gated potassium channel subunit Kv1.5 | KCNA5 | P22460 | CHEMBL4306 | Voltage-gated ion channel | 0.101614 | 116 / 0Â Â Â Â Â | Cannabidiol |
| L-lactate dehydrogenase B chain | LDHB | P07195 | CHEMBL4940 | Enzyme | 0.101614 | 2 / 0Â Â Â Â Â | Cannabidiol |
| Thymidylate synthase | TYMS | P04818 | CHEMBL1952 | Transferase | 0.101614 | 23 / 0Â Â Â Â Â | Cannabidiol |
| Glucagon receptor | GCGR | P47871 | CHEMBL1985 | Family B G protein-coupled receptor | 0.101614 | 34 / 0Â Â Â Â Â | Cannabidiol |
| p53-binding protein Mdm-2 | MDM2 | Q00987 | CHEMBL5023 | Other nuclear protein | 0.101614 | 69 / 0Â Â Â Â Â | Cannabidiol |
| Steryl-sulfatase | STS | P08842 | CHEMBL3559 | Enzyme | 0.101614 | 6 / 0Â Â Â Â Â | Cannabidiol |
| L-lactate dehydrogenase A chain | LDHA | P00338 | CHEMBL4835 | Enzyme | 0.101614 | 7 / 0Â Â Â Â Â | Cannabidiol |
| Metabotropic glutamate receptor 2 | GRM2 | Q14416 | CHEMBL5137 | Family C G protein-coupled receptor | 0.101614 | 34 / 0Â Â Â Â Â | Cannabidiol |
| Serine/threonine-protein kinase PAK 1 | PAK1 | Q13153 | CHEMBL4600 | Kinase | 0.101614 | 2 / 0Â Â Â Â Â | Cannabidiol |
| Cholecystokinin B receptor (by homology) | CCKBR | P32239 | CHEMBL298 | Family A G protein-coupled receptor | 0.101614 | 278 / 0Â Â Â Â Â | Cannabidiol |
| Melatonin receptor 1B | MTNR1B | P49286 | CHEMBL1946 | Family A G protein- | 0.101614 | 86 / 0Â Â Â Â Â | Cannabidiol |

| | | | | | | | |
|---|---|---|---|---|---|---|---|
| | | | | coupled receptor | | | |
| Melanocortin receptor 4 | MC4R | P32245 | CHEMBL259 | Family A G protein-coupled receptor | 0.101614 | 21 / 0Â Â Â Â Â | Cannabidiol |
| Serine/threonine-protein kinase Aurora-C | AURKC | Q9UQB9 | CHEMBL3935 | Kinase | 0.101614 | 9 / 0Â Â Â Â Â | Cannabidiol |
| Serine/threonine-protein kinase/endoribonuclease IRE1 | ERN1 | O75460 | CHEMBL1163101 | Enzyme | 0.101614 | 3 / 0Â Â Â Â Â | Cannabidiol |
| Beta secretase 2 | BACE2 | Q9Y5Z0 | CHEMBL2525 | Protease | 0.101614 | 46 / 0Â Â Â Â Â | Cannabidiol |
| Pyruvate kinase isozymes M1/M2 | PKM | P14618 | CHEMBL1075189 | Enzyme | 0.101614 | 7 / 0Â Â Â Â Â | Cannabidiol |
| Aldose reductase (by homology) | AKR1B1 | P15121 | CHEMBL1900 | Enzyme | 0.101614 | 7 / 0Â Â Â Â Â | Cannabidiol |
| Interleukin-8 receptor B | CXCR2 | P25025 | CHEMBL2434 | Family A G protein-coupled receptor | 0.101614 | 47 / 0Â Â Â Â Â | Cannabidiol |
| ATP-binding cassette sub-family G member 2 | ABCG2 | Q9UNQ0 | CHEMBL5393 | Primary active transporter | 0.101614 | 14 / 0Â Â Â Â Â | Cannabidiol |
| Cholecystokinin A receptor | CCKAR | P32238 | CHEMBL1901 | Family A G protein-coupled receptor | 0 | 35 / 0Â Â Â Â Â | Cannabidiol |
| Estradiol 17-beta-dehydrogenase 2 | HSD17B2 | P37059 | CHEMBL2789 | Enzyme | 0 | 20 / 0Â Â Â Â Â | Cannabidiol |
| Neuropeptide Y receptor type 5 | NPY5R | Q15761 | CHEMBL4561 | Family A G protein-coupled receptor | 0 | 139 / 0Â Â Â Â Â | Cannabidiol |
| Sodium channel protein type V alpha subunit | SCN5A | Q14524 | CHEMBL1980 | Voltage-gated ion channel | 0 | 21 / 0Â Â Â Â Â | Cannabidiol |
| Carbonic anhydrase II | CA2 | P00918 | CHEMBL205 | Lyase | 0 | 98 / 0Â Â Â Â Â | Cannabidiol |
| Acyl coenzyme A:cholesterol acyltransferase | CES1 | P23141 | CHEMBL2265 | Enzyme | 0 | 24 / 0Â Â Â Â Â | Cannabidiol |
| 6-phosphofructo-2-kinase/fructose-2,6-bisphosphatase 3 | PFKFB3 | Q16875 | CHEMBL2331053 | Enzyme | 0 | 65 / 0Â Â Â Â Â | Cannabidiol |
| Carboxylesterase 2 | CES2 | O00748 | CHEMBL3180 | Enzyme | 0 | 5 / 0Â Â Â Â Â | Cannabidiol |
| Carbonic anhydrase XII | CA12 | O43570 | CHEMBL3242 | Lyase | 0 | 54 / 0Â Â Â Â Â | Cannabidiol |
| Carbonic anhydrase IX | CA9 | Q16790 | CHEMBL3594 | Lyase | 0 | 72 / 0Â Â Â Â Â | Cannabidiol |
| Sodium channel protein type II alpha subunit | SCN2A | Q99250 | CHEMBL4187 | Voltage-gated ion channel | 0 | 28 / 0Â Â Â Â Â | Cannabidiol |
| Cyclin-dependent kinase 5/CDK5 activator 1 | CDK5R1 CDK5 | Q15078 Q00535 | CHEMBL1907600 | Kinase | 0 | 100 / 0Â Â Â Â Â | Cannabidiol |

| | | | | | | | |
|---|---|---|---|---|---|---|---|
| Serine/threonine-protein kinase PLK1 | PLK1 | P53350 | CHEMBL3024 | Kinase | 0 | 15 / 0Â Â Â Â Â | Cannabidiol |
| Integrin alpha2/beta1 | ITGB1 ITGA2 | P05556 P17301 | CHEMBL3137268 | Unclassified protein | 0 | 1 / 0Â Â Â Â Â | Cannabidiol |
| Serine/threonine-protein kinase Aurora-A | AURKA | O14965 | CHEMBL4722 | Kinase | 0 | 31 / 0Â Â Â Â Â | Cannabidiol |
| Monoamine oxidase B | MAOB | P27338 | CHEMBL2039 | Oxidoreductase | 0 | 158 / 0Â Â Â Â Â | Cannabidiol |
| Bromodomain-containing protein 1 | BRD1 | O95696 | CHEMBL2176774 | Reader | 0 | 7 / 0Â Â Â Â Â | Cannabidiol |
| P2X purinoceptor 3 | P2RX3 | P56373 | CHEMBL2998 | Ligand-gated ion channel | 0 | 42 / 0Â Â Â Â Â | Cannabidiol |
| Transcription intermediary factor 1-alpha | TRIM24 | O15164 | CHEMBL3108638 | Reader | 0 | 10 / 0Â Â Â Â Â | Cannabidiol |
| Peregrin | BRPF1 | P55201 | CHEMBL3132741 | Reader | 0 | 25 / 0Â Â Â Â Â | Cannabidiol |
| Nitric-oxide synthase, brain | NOS1 | P29475 | CHEMBL3568 | Enzyme | 0 | 37 / 0Â Â Â Â Â | Cannabidiol |
| Calcium-activated potassium channel subunit alpha-1 | KCNMA1 | Q12791 | CHEMBL4304 | Voltage-gated ion channel | 0 | 13 / 0Â Â Â Â Â | Cannabidiol |
| Nitric-oxide synthase, endothelial | NOS3 | P29474 | CHEMBL4803 | Enzyme | 0 | 16 / 0Â Â Â Â Â | Cannabidiol |
| Pyroglutamylated RFamide peptide receptor | QRFPR | Q96P65 | CHEMBL5852 | Family A G protein-coupled receptor | 0 | 10 / 0Â Â Â Â Â | Cannabidiol |
| Carnitine O-palmitoyltransferase 1, liver isoform | CPT1A | P50416 | CHEMBL1293194 | Enzyme | 0 | 38 / 0Â Â Â Â Â | Cannabidiol |
| Tyrosine-protein kinase JAK3 | JAK3 | P52333 | CHEMBL2148 | Kinase | 0 | 180 / 0Â Â Â Â Â | Cannabidiol |
| Tyrosine-protein kinase JAK2 | JAK2 | O60674 | CHEMBL2971 | Kinase | 0 | 160 / 0Â Â Â Â Â | Cannabidiol |
| Bombesin receptor subtype-3 | BRS3 | P32247 | CHEMBL4080 | Family A G protein-coupled receptor | 0 | 2 / 0Â Â Â Â Â | Cannabidiol |
| Metabotropic glutamate receptor 5 | GRM5 | P41594 | CHEMBL3227 | Family C G protein-coupled receptor | 0 | 279 / 0Â Â Â Â Â | Cannabidiol |
| Signal transducer and activator of transcription 3 | STAT3 | P40763 | CHEMBL4026 | Transcription factor | 0 | 23 / 0Â Â Â Â Â | Cannabidiol |
| Dopamine D1 receptor | DRD1 | P21728 | CHEMBL2056 | Family A G protein-coupled receptor | 0 | 24 / 0Â Â Â Â Â | Cannabidiol |
| Adenosine A1 receptor | ADORA1 | P30542 | CHEMBL226 | Family A G protein-coupled receptor | 0 | 268 / 0Â Â Â Â Â | Cannabidiol |
| Adenosine A2a receptor | ADORA2A | P29274 | CHEMBL251 | Family A G protein- | 0 | 162 / 0Â Â Â Â Â | Cannabidiol |

| | | | | | | | |
|---|---|---|---|---|---|---|---|
| | | | | coupled receptor | | | |
| Protein kinase C delta | PRKCD | Q05655 | CHEMBL2996 | Kinase | 0 | 24 / 0Â Â Â Â Â | Cannabidiol |
| Tyrosine-protein kinase Lyn | LYN | P07948 | CHEMBL3905 | Kinase | 0 | 5 / 0Â Â Â Â Â | Cannabidiol |
| Protein kinase C theta | PRKCQ | Q04759 | CHEMBL3920 | Kinase | 0 | 39 / 0Â Â Â Â Â | Cannabidiol |
| Peroxisome proliferator-activated receptor alpha | PPARA | Q07869 | CHEMBL239 | Nuclear receptor | 0 | 0 / 13Â Â Â Â Â | Cannabidiol |
| Interleukin-8 receptor A | CXCR1 | P25024 | CHEMBL4029 | Family A G protein-coupled receptor | 0 | 12 / 0Â Â Â Â Â | Cannabidiol |
| Anandamide amidohydrolase | FAAH | O00519 | CHEMBL2243 | Enzyme | 0 | 96 / 0Â Â Â Â Â | Cannabidiol |
| Kinesin-like protein 1 | KIF11 | P52732 | CHEMBL4581 | Other cytosolic protein | 0 | 34 / 0Â Â Â Â Â | Cannabidiol |
| Phosphodiesterase 5A | PDE5A | O76074 | CHEMBL1827 | Phosphodiesterase | 0 | 129 / 0Â Â Â Â Â | Cannabidiol |
| Melatonin receptor 1A | MTNR1A | P48039 | CHEMBL1945 | Family A G protein-coupled receptor | 0 | 115 / 0Â Â Â Â Â | Cannabidiol |
| Protein farnesyltransferase | FNTA FNTB | P49354 P49356 | CHEMBL2094108 | Enzyme | 0 | 169 / 0Â Â Â Â Â | Cannabidiol |
| G-protein coupled bile acid receptor 1 | GPBAR1 | Q8TDU6 | CHEMBL5409 | Family A G protein-coupled receptor | 0 | 24 / 0Â Â Â Â Â | Cannabidiol |
| Isocitrate dehydrogenase [NADP] cytoplasmic | IDH1 | O75874 | CHEMBL2007625 | Enzyme | 0 | 213 / 0Â Â Â Â Â | Cannabidiol |
| Tyrosine-protein kinase SYK | SYK | P43405 | CHEMBL2599 | Kinase | 0 | 37 / 0Â Â Â Â Â | Cannabidiol |
| Tyrosine-protein kinase JAK1 | JAK1 | P23458 | CHEMBL2835 | Kinase | 0 | 72 / 0Â Â Â Â Â | Cannabidiol |
| JAK3/JAK1 | JAK3 JAK1 | P52333 P23458 | CHEMBL3038491 | Kinase | 0 | 15 / 0Â Â Â Â Â | Cannabidiol |
| Hormone sensitive lipase | LIPE | Q05469 | CHEMBL3590 | Enzyme | 0 | 55 / 0Â Â Â Â Â | Cannabidiol |
| | | | | | | | |
| Cannabinoid receptor 1 | CNR1 | P21554 | CHEMBL218 | Family A G protein-coupled receptor | 0.897728 | 564 / 210Â Â Â Â Â | Cannabidivarin |
| Cannabinoid receptor 2 | CNR2 | P34972 | CHEMBL253 | Family A G protein-coupled receptor | 0.897728 | 439 / 174Â Â Â Â Â | Cannabidivarin |
| G-protein coupled receptor 55 | GPR55 | Q9Y2T6 | CHEMBL1075322 | Family A G protein-coupled receptor | 0.641391 | 9 / 2Â Â Â Â Â | Cannabidivarin |
| Arachidonate 5-lipoxygenase | ALOX5 | P09917 | CHEMBL215 | Oxidoreductase | 0.097875 | 42 / 19Â Â Â Â Â | Cannabidivarin |

| | | | | | | | |
|---|---|---|---|---|---|---|---|
| Cystic fibrosis transmembrane conductance regulator | CFTR | P13569 | CHEMBL4051 | Other ion channel | 0.097875 | 9 / 0Â Â Â Â Â | Cannabidivarin |
| Melanocortin receptor 4 | MC4R | P32245 | CHEMBL259 | Family A G protein-coupled receptor | 0.097875 | 16 / 0Â Â Â Â Â | Cannabidivarin |
| Poly [ADP-ribose] polymerase-1 | PARP1 | P09874 | CHEMBL3105 | Enzyme | 0.097875 | 18 / 0Â Â Â Â Â | Cannabidivarin |
| Receptor protein-tyrosine kinase erbB-2 | ERBB2 | P04626 | CHEMBL1824 | Kinase | 0.097875 | 44 / 0Â Â Â Â Â | Cannabidivarin |
| DNA polymerase beta (by homology) | POLB | P06746 | CHEMBL2392 | Enzyme | 0.097875 | 0 / 3Â Â Â Â Â | Cannabidivarin |
| Arachidonate 15-lipoxygenase | ALOX15 | P16050 | CHEMBL2903 | Enzyme | 0.097875 | 19 / 11Â Â Â Â Â | Cannabidivarin |
| Calcium sensing receptor | CASR | P41180 | CHEMBL1878 | Family C G protein-coupled receptor | 0.097875 | 33 / 0Â Â Â Â Â | Cannabidivarin |
| Histone deacetylase 6 | HDAC6 | Q9UBN7 | CHEMBL1865 | Eraser | 0.097875 | 20 / 0Â Â Â Â Â | Cannabidivarin |
| Histone deacetylase 8 | HDAC8 | Q9BY41 | CHEMBL3192 | Eraser | 0.097875 | 12 / 0Â Â Â Â Â | Cannabidivarin |
| NAD-dependent deacetylase sirtuin 2 | SIRT2 | Q8IXJ6 | CHEMBL4462 | Eraser | 0.097875 | 21 / 0Â Â Â Â Â | Cannabidivarin |
| Serotonin 2b (5-HT2b) receptor | HTR2B | P41595 | CHEMBL1833 | Family A G protein-coupled receptor | 0.097875 | 38 / 0Â Â Â Â Â | Cannabidivarin |
| Glucagon receptor | GCGR | P47871 | CHEMBL1985 | Family B G protein-coupled receptor | 0.097875 | 26 / 0Â Â Â Â Â | Cannabidivarin |
| Prenyl protein specific protease | RCE1 | Q9Y256 | CHEMBL3411 | Protease | 0.097875 | 6 / 0Â Â Â Â Â | Cannabidivarin |
| Vascular endothelial growth factor receptor 2 | KDR | P35968 | CHEMBL279 | Kinase | 0.097875 | 214 / 12Â Â Â Â Â | Cannabidivarin |
| N-arachidonyl glycine receptor | GPR18 | Q14330 | CHEMBL2384898 | Family A G protein-coupled receptor | 0.097875 | 2 / 2Â Â Â Â Â | Cannabidivarin |
| Glycine receptor subunit alpha-1 | GLRA1 | P23415 | CHEMBL5845 | Ligand-gated ion channel | 0.097875 | 1 / 1Â Â Â Â Â | Cannabidivarin |
| Phosphodiesterase 4B | PDE4B | Q07343 | CHEMBL275 | Phosphodiesterase | 0.097875 | 21 / 0Â Â Â Â Â | Cannabidivarin |
| Cyclooxygenase-2 | PTGS2 | P35354 | CHEMBL230 | Oxidoreductase | 0.097875 | 120 / 3Â Â Â Â Â | Cannabidivarin |
| Epoxide hydratase | EPHX2 | P34913 | CHEMBL2409 | Protease | 0.097875 | 64 / 0Â Â Â Â Â | Cannabidivarin |
| L-lactate dehydrogenase A chain | LDHA | P00338 | CHEMBL4835 | Enzyme | 0.097875 | 7 / 0Â Â Â Â Â | Cannabidivarin |
| Cathepsin D | CTSD | P07339 | CHEMBL2581 | Protease | 0.097875 | 22 / 0Â Â Â Â Â | Cannabidivarin |

| | | | | | | | |
|---|---|---|---|---|---|---|---|
| Beta-secretase 1 | BACE1 | P56817 | CHEMBL4822 | Protease | 0.097875 | 160 / 0Â Â Â Â Â | Cannabidivarin |
| Cholesteryl ester transfer protein | CETP | P11597 | CHEMBL3572 | Other ion channel | 0.097875 | 5 / 2Â Â Â Â Â | Cannabidivarin |
| Tyrosine-protein kinase JAK2 | JAK2 | O60674 | CHEMBL2971 | Kinase | 0.097875 | 169 / 0Â Â Â Â Â | Cannabidivarin |
| Monoglyceride lipase | MGLL | Q99685 | CHEMBL4191 | Enzyme | 0.097875 | 8 / 0Â Â Â Â Â | Cannabidivarin |
| Quinone reductase 2 | NQO2 | P16083 | CHEMBL3959 | Enzyme | 0.097875 | 8 / 0Â Â Â Â Â | Cannabidivarin |
| Thymidylate synthase | TYMS | P04818 | CHEMBL1952 | Transferase | 0.097875 | 25 / 0Â Â Â Â Â | Cannabidivarin |
| Interleukin-8 receptor B | CXCR2 | P25025 | CHEMBL2434 | Family A G protein-coupled receptor | 0.097875 | 38 / 0Â Â Â Â Â | Cannabidivarin |
| Serine/threonine-protein kinase PAK 1 | PAK1 | Q13153 | CHEMBL4600 | Kinase | 0.097875 | 1 / 0Â Â Â Â Â | Cannabidivarin |
| Cyclin-dependent kinase 5/CDK5 activator 1 | CDK5R1 CDK5 | Q15078 Q00535 | CHEMBL1907600 | Kinase | 0.097875 | 99 / 0Â Â Â Â Â | Cannabidivarin |
| Serine/threonine-protein kinase Aurora-C | AURKC | Q9UQB 9 | CHEMBL3935 | Kinase | 0.097875 | 11 / 0Â Â Â Â Â | Cannabidivarin |
| Serine/threonine-protein kinase Aurora-A | AURKA | O14965 | CHEMBL4722 | Kinase | 0.097875 | 26 / 0Â Â Â Â Â | Cannabidivarin |
| p53-binding protein Mdm-2 | MDM2 | Q00987 | CHEMBL5023 | Other nuclear protein | 0.097875 | 42 / 0Â Â Â Â Â | Cannabidivarin |
| Translocator protein (by homology) | TSPO | P30536 | CHEMBL5742 | Membrane receptor | 0.097875 | 279 / 0Â Â Â Â Â | Cannabidivarin |
| 11-beta-hydroxysteroid dehydrogenase 1 | HSD11B 1 | P28845 | CHEMBL4235 | Enzyme | 0.097875 | 208 / 0Â Â Â Â Â | Cannabidivarin |
| Serine/threonine-protein kinase/endoribonuclease IRE1 | ERN1 | O75460 | CHEMBL1163101 | Enzyme | 0.097875 | 3 / 0Â Â Â Â Â | Cannabidivarin |
| Estradiol 17-beta-dehydrogenase 3 | HSD17B 3 | P37058 | CHEMBL4234 | Enzyme | 0.097875 | 4 / 0Â Â Â Â Â | Cannabidivarin |
| Calcium-activated potassium channel subunit alpha-1 | KCNMA 1 | Q12791 | CHEMBL4304 | Voltage-gated ion channel | 0.097875 | 12 / 0Â Â Â Â Â | Cannabidivarin |
| Anandamide amidohydrolase | FAAH | O00519 | CHEMBL2243 | Enzyme | 0.097875 | 32 / 0Â Â Â Â Â | Cannabidivarin |
| Steryl-sulfatase | STS | P08842 | CHEMBL3559 | Enzyme | 0.097875 | 9 / 0Â Â Â Â Â | Cannabidivarin |
| Estradiol 17-beta-dehydrogenase 2 | HSD17B 2 | P37059 | CHEMBL2789 | Enzyme | 0.097875 | 12 / 0Â Â Â Â Â | Cannabidivarin |
| Cholecystokinin B receptor (by homology) | CCKBR | P32239 | CHEMBL298 | Family A G protein-coupled receptor | 0.097875 | 183 / 0Â Â Â Â Â | Cannabidivarin |
| Protein kinase C delta | PRKCD | Q05655 | CHEMBL2996 | Kinase | 0.097875 | 18 / 0Â Â Â Â Â | Cannabidivarin |
| Tyrosine-protein kinase Lyn | LYN | P07948 | CHEMBL3905 | Kinase | 0.097875 | 4 / 0Â Â Â Â Â | Cannabidivarin |

| | | | | | | | |
|---|---|---|---|---|---|---|---|
| Protein kinase C theta | PRKCQ | Q04759 | CHEMBL3920 | Kinase | 0.097875 | 33 / 0Â Â Â Â Â | Cannabidivarin |
| Macrophage migration inhibitory factor | MIF | P14174 | CHEMBL2085 | Enzyme | 0.097875 | 3 / 0Â Â Â Â Â | Cannabidivarin |
| ADAMTS5 | ADAMTS5 | Q9UNA0 | CHEMBL2285 | Protease | 0.097875 | 20 / 0Â Â Â Â Â | Cannabidivarin |
| Nitric-oxide synthase, brain | NOS1 | P29475 | CHEMBL3568 | Enzyme | 0.097875 | 51 / 0Â Â Â Â Â | Cannabidivarin |
| Nitric-oxide synthase, endothelial | NOS3 | P29474 | CHEMBL4803 | Enzyme | 0.097875 | 20 / 0Â Â Â Â Â | Cannabidivarin |
| Metabotropic glutamate receptor 2 | GRM2 | Q14416 | CHEMBL5137 | Family C G protein-coupled receptor | 0.097875 | 21 / 0Â Â Â Â Â | Cannabidivarin |
| Bromodomain-containing protein 1 | BRD1 | O95696 | CHEMBL2176774 | Reader | 0 | 6 / 0Â Â Â Â Â | Cannabidivarin |
| Transcription intermediary factor 1-alpha | TRIM24 | O15164 | CHEMBL3108638 | Reader | 0 | 9 / 0Â Â Â Â Â | Cannabidivarin |
| Peregrin | BRPF1 | P55201 | CHEMBL3132741 | Reader | 0 | 21 / 0Â Â Â Â Â | Cannabidivarin |
| LDL-associated phospholipase A2 | PLA2G7 | Q13093 | CHEMBL3514 | Enzyme | 0 | 16 / 0Â Â Â Â Â | Cannabidivarin |
| Mitogen-activated protein kinase kinase kinase 8 | MAP3K8 | P41279 | CHEMBL4899 | Kinase | 0 | 37 / 0Â Â Â Â Â | Cannabidivarin |
| Adenosine A1 receptor | ADORA1 | P30542 | CHEMBL226 | Family A G protein-coupled receptor | 0 | 202 / 0Â Â Â Â Â | Cannabidivarin |
| Adenosine A2a receptor | ADORA2A | P29274 | CHEMBL251 | Family A G protein-coupled receptor | 0 | 110 / 0Â Â Â Â Â | Cannabidivarin |
| Prostanoid EP1 receptor | PTGER1 | P34995 | CHEMBL1811 | Family A G protein-coupled receptor | 0 | 22 / 0Â Â Â Â Â | Cannabidivarin |
| Melatonin receptor 1B | MTNR1B | P49286 | CHEMBL1946 | Family A G protein-coupled receptor | 0 | 49 / 0Â Â Â Â Â | Cannabidivarin |
| MAP kinase p38 alpha | MAPK14 | Q16539 | CHEMBL260 | Kinase | 0 | 279 / 0Â Â Â Â Â | Cannabidivarin |
| Monoamine oxidase B | MAOB | P27338 | CHEMBL2039 | Oxidoreductase | 0 | 128 / 0Â Â Â Â Â | Cannabidivarin |
| Egl nine homolog 1 | EGLN1 | Q9GZT9 | CHEMBL5697 | Oxidoreductase | 0 | 9 / 0Â Â Â Â Â | Cannabidivarin |
| Glycogen synthase kinase-3 beta | GSK3B | P49841 | CHEMBL262 | Kinase | 0 | 47 / 0Â Â Â Â Â | Cannabidivarin |
| Sodium channel protein type II alpha subunit | SCN2A | Q99250 | CHEMBL4187 | Voltage-gated ion channel | 0 | 22 / 0Â Â Â Â Â | Cannabidivarin |
| Voltage-gated potassium channel subunit Kv1.5 | KCNA5 | P22460 | CHEMBL4306 | Voltage-gated ion channel | 0 | 93 / 0Â Â Â Â Â | Cannabidivarin |

| | | | | | | | |
|---|---|---|---|---|---|---|---|
| Isocitrate dehydrogenase [NADP] cytoplasmic | IDH1 | O75874 | CHEMBL2007625 | Enzyme | 0 | 191 / 0Â Â Â Â Â | Cannabidivarin |
| Cyclin-dependent kinase 1/cyclin B | CCNB3 CDK1 CCNB1 CCNB2 | Q8WWL7 P06493 P14635 O95067 | CHEMBL2094127 | Other cytosolic protein | 0 | 42 / 0Â Â Â Â Â | Cannabidivarin |
| Cathepsin K | CTSK | P43235 | CHEMBL268 | Protease | 0 | 107 / 2Â Â Â Â Â | Cannabidivarin |
| Peroxisome proliferator-activated receptor alpha | PPARA | Q07869 | CHEMBL239 | Nuclear receptor | 0 | 0 / 13Â Â Â Â Â | Cannabidivarin |
| Dopamine D1 receptor | DRD1 | P21728 | CHEMBL2056 | Family A G protein-coupled receptor | 0 | 19 / 0Â Â Â Â Â | Cannabidivarin |
| Protein farnesyltransferase | FNTA FNTB | P49354 P49356 | CHEMBL2094108 | Enzyme | 0 | 128 / 0Â Â Â Â Â | Cannabidivarin |
| Tyrosine-protein kinase JAK3 | JAK3 | P52333 | CHEMBL2148 | Kinase | 0 | 184 / 0Â Â Â Â Â | Cannabidivarin |
| Bromodomain-containing protein 4 | BRD4 | O60885 | CHEMBL1163125 | Reader | 0 | 38 / 0Â Â Â Â Â | Cannabidivarin |
| Gonadotropin-releasing hormone receptor | GNRHR | P30968 | CHEMBL1855 | Family A G protein-coupled receptor | 0 | 6 / 0Â Â Â Â Â | Cannabidivarin |
| Kinesin-1 heavy chain/ Tyrosine-protein kinase receptor RET | RET | P07949 | CHEMBL2041 | Kinase | 0 | 12 / 0Â Â Â Â Â | Cannabidivarin |
| Adenosine A2b receptor | ADORA2B | P29275 | CHEMBL255 | Family A G protein-coupled receptor | 0 | 27 / 0Â Â Â Â Â | Cannabidivarin |
| Serine/threonine-protein kinase RIPK2 | RIPK2 | O43353 | CHEMBL5014 | Kinase | 0 | 5 / 0Â Â Â Â Â | Cannabidivarin |
| Dual-specificity tyrosine-phosphorylation regulated kinase 1A (by homology) | DYRK1A | Q13627 | CHEMBL2292 | Kinase | 0 | 44 / 0Â Â Â Â Â | Cannabidivarin |
| Beta secretase 2 | BACE2 | Q9Y5Z0 | CHEMBL2525 | Protease | 0 | 38 / 0Â Â Â Â Â | Cannabidivarin |
| Sodium channel protein type V alpha subunit | SCN5A | Q14524 | CHEMBL1980 | Voltage-gated ion channel | 0 | 17 / 0Â Â Â Â Â | Cannabidivarin |
| Serine/threonine-protein kinase 33 | STK33 | Q9BYT3 | CHEMBL6005 | Kinase | 0 | 17 / 0Â Â Â Â Â | Cannabidivarin |
| Corticotropin releasing factor receptor 1 | CRHR1 | P34998 | CHEMBL1800 | Family B G protein-coupled receptor | 0 | 67 / 0Â Â Â Â Â | Cannabidivarin |
| Cholecystokinin A receptor | CCKAR | P32238 | CHEMBL1901 | Family A G protein-coupled receptor | 0 | 16 / 0Â Â Â Â Â | Cannabidivarin |
| Pyruvate kinase isozymes M1/M2 | PKM | P14618 | CHEMBL1075189 | Enzyme | 0 | 6 / 0Â Â Â Â Â | Cannabidivarin |

| | | | | | | | |
|---|---|---|---|---|---|---|---|
| Melatonin receptor 1A | MTNR1A | P48039 | CHEMBL1945 | Family A G protein-coupled receptor | 0 | 69 / 0Â Â Â Â Â | Cannabidivarin |
| Tyrosine-protein kinase JAK1 | JAK1 | P23458 | CHEMBL2835 | Kinase | 0 | 66 / 0Â Â Â Â Â | Cannabidivarin |
| JAK3/JAK1 | JAK3 JAK1 | P52333 P23458 | CHEMBL3038491 | Kinase | 0 | 13 / 0Â Â Â Â Â | Cannabidivarin |
| L-lactate dehydrogenase B chain | LDHB | P07195 | CHEMBL4940 | Enzyme | 0 | 2 / 0Â Â Â Â Â | Cannabidivarin |
| Myosin light chain kinase, smooth muscle | MYLK | Q15746 | CHEMBL2428 | Kinase | 0 | 3 / 0Â Â Â Â Â | Cannabidivarin |
| Cyclin-dependent kinase 2 | CDK2 | P24941 | CHEMBL301 | Kinase | 0 | 45 / 0Â Â Â Â Â | Cannabidivarin |
| Cyclin-dependent kinase 4 | CDK4 | P11802 | CHEMBL331 | Kinase | 0 | 20 / 0Â Â Â Â Â | Cannabidivarin |
| Nischarin | NISCH | Q9Y2I1 | CHEMBL3923 | Other cytosolic protein | 0 | 38 / 0Â Â Â Â Â | Cannabidivarin |
| Glutaminyl-peptide cyclotransferase | QPCT | Q16769 | CHEMBL4508 | Enzyme | 0 | 26 / 0Â Â Â Â Â | Cannabidivarin |
| Matrix metalloproteinase 9 | MMP9 | P14780 | CHEMBL321 | Protease | 0 | 19 / 0Â Â Â Â Â | Cannabidivarin |
| Matrix metalloproteinase 2 | MMP2 | P08253 | CHEMBL333 | Protease | 0 | 23 / 0Â Â Â Â Â | Cannabidivarin |
| GABA receptor alpha-1 subunit (by homology) | GABRA1 | P14867 | CHEMBL1962 | Ligand-gated ion channel | 0 | 8 / 0Â Â Â Â Â | Cannabidivarin |
| | | | | | | | |
| Cannabinoid receptor 1 | CNR1 | P21554 | CHEMBL218 | Family A G protein-coupled receptor | 0.241979 | 432 / 344Â Â Â Â Â | Cannabielsoin |
| Cannabinoid receptor 2 | CNR2 | P34972 | CHEMBL253 | Family A G protein-coupled receptor | 0.241979 | 425 / 329Â Â Â Â Â | Cannabielsoin |
| Vascular endothelial growth factor receptor 2 | KDR | P35968 | CHEMBL279 | Kinase | 0.112748 | 414 / 10Â Â Â Â Â | Cannabielsoin |
| 6-phosphofructo-2-kinase/fructose-2,6-bisphosphatase 3 | PFKFB3 | Q16875 | CHEMBL2331053 | Enzyme | 0.104672 | 138 / 0Â Â Â Â Â | Cannabielsoin |
| Glucagon receptor | GCGR | P47871 | CHEMBL1985 | Family B G protein-coupled receptor | 0.104672 | 13 / 0Â Â Â Â Â | Cannabielsoin |
| Gamma-secretase | PSEN2 PSENEN NCSTN APH1A PSEN1 APH1B | P49810 Q9NZ42 Q92542 Q96BI3 P49768 Q8WW43 | CHEMBL2094135 | Protease | 0.104672 | 208 / 0Â Â Â Â Â | Cannabielsoin |
| Lysosomal Pro-X carboxypeptidase | PRCP | P42785 | CHEMBL2335 | Protease | 0.104672 | 28 / 0Â Â Â Â Â | Cannabielsoin |

| | | | | | | | |
|---|---|---|---|---|---|---|---|
| Mixed lineage kinase 7 | MAP3K20 | Q9NYL2 | CHEMBL3886 | Kinase | 0.104672 | 6 / 0Â Â Â Â Â | Cannabielsoin |
| TGF-beta receptor type II | TGFBR2 | P37173 | CHEMBL4267 | Kinase | 0.104672 | 4 / 0Â Â Â Â Â | Cannabielsoin |
| TGF-beta receptor type I | TGFBR1 | P36897 | CHEMBL4439 | Kinase | 0.104672 | 21 / 0Â Â Â Â Â | Cannabielsoin |
| Serine/threonine-protein kinase Aurora-B | AURKB | Q96GD4 | CHEMBL2185 | Kinase | 0.104672 | 137 / 0Â Â Â Â Â | Cannabielsoin |
| Cyclin-dependent kinase 1 | CDK1 | P06493 | CHEMBL308 | Kinase | 0.104672 | 179 / 0Â Â Â Â Â | Cannabielsoin |
| Serine/threonine-protein kinase Aurora-A | AURKA | O14965 | CHEMBL4722 | Kinase | 0.104672 | 200 / 0Â Â Â Â Â | Cannabielsoin |
| Phosphodiesterase 2A | PDE2A | O00408 | CHEMBL2652 | Phosphodiesterase | 0.104672 | 109 / 0Â Â Â Â Â | Cannabielsoin |
| Cyclin-dependent kinase 2 | CDK2 | P24941 | CHEMBL301 | Kinase | 0.104672 | 175 / 0Â Â Â Â Â | Cannabielsoin |
| Phosphodiesterase 10A | PDE10A | Q9Y233 | CHEMBL4409 | Phosphodiesterase | 0.104672 | 605 / 0Â Â Â Â Â | Cannabielsoin |
| Protein kinase C alpha | PRKCA | P17252 | CHEMBL299 | Kinase | 0.104672 | 107 / 0Â Â Â Â Â | Cannabielsoin |
| Interleukin-6 receptor subunit beta | IL6ST | P40189 | CHEMBL3124734 | Membrane receptor | 0.104672 | 6 / 0Â Â Â Â Â | Cannabielsoin |
| Tyrosine-protein kinase SYK | SYK | P43405 | CHEMBL2599 | Kinase | 0.104672 | 212 / 0Â Â Â Â Â | Cannabielsoin |
| Adenosine A2a receptor | ADORA2A | P29274 | CHEMBL251 | Family A G protein-coupled receptor | 0.104672 | 186 / 0Â Â Â Â Â | Cannabielsoin |
| Period circadian protein homolog 2 | PER2 | O15055 | CHEMBL3751648 | Unclassified protein | 0.104672 | 40 / 0Â Â Â Â Â | Cannabielsoin |
| Toll-like receptor 4 | TLR4 | O00206 | CHEMBL5255 | Toll-like and Il-1 receptors | 0.104672 | 4 / 0Â Â Â Â Â | Cannabielsoin |
| DNA topoisomerase I (by homology) | TOP1 | P11387 | CHEMBL1781 | Isomerase | 0.104672 | 12 / 0Â Â Â Â Â | Cannabielsoin |
| C-C chemokine receptor type 1 | CCR1 | P32246 | CHEMBL2413 | Family A G protein-coupled receptor | 0.104672 | 247 / 0Â Â Â Â Â | Cannabielsoin |
| PI3-kinase p110-alpha subunit | PIK3CA | P42336 | CHEMBL4005 | Enzyme | 0.104672 | 552 / 0Â Â Â Â Â | Cannabielsoin |
| Serine-protein kinase ATR | ATR | Q13535 | CHEMBL5024 | Kinase | 0.104672 | 29 / 0Â Â Â Â Â | Cannabielsoin |
| 11-beta-hydroxysteroid dehydrogenase 1 | HSD11B1 | P28845 | CHEMBL4235 | Enzyme | 0.104672 | 314 / 0Â Â Â Â Â | Cannabielsoin |
| Leukocyte elastase | ELANE | P08246 | CHEMBL248 | Protease | 0.104672 | 36 / 0Â Â Â Â Â | Cannabielsoin |
| Mitogen-activated protein kinase kinase kinase 5 | MAP3K5 | Q99683 | CHEMBL5285 | Kinase | 0.104672 | 16 / 0Â Â Â Â Â | Cannabielsoin |
| Thrombin and coagulation factor X | F10 | P00742 | CHEMBL244 | Protease | 0.104672 | 226 / 0Â Â Â Â Â | Cannabielsoin |
| Tyrosine-protein kinase receptor FLT3 | FLT3 | P36888 | CHEMBL1974 | Kinase | 0.104672 | 106 / 0Â Â Â Â Â | Cannabielsoin |
| Tyrosine-protein kinase YES | YES1 | P07947 | CHEMBL2073 | Kinase | 0.104672 | 16 / 0Â Â Â Â Â | Cannabielsoin |

| | | | | | | | |
|---|---|---|---|---|---|---|---|
| Potassium channel subfamily K member 3 | KCNK3 | O14649 | CHEMBL2321613 | Voltage-gated ion channel | 0.104672 | 25 / 0Â Â Â Â Â | Cannabielsoin |
| Phosphodiesterase 3 | PDE3A | Q14432 | CHEMBL241 | Phosphodiesterase | 0.104672 | 20 / 0Â Â Â Â Â | Cannabielsoin |
| Tyrosine-protein kinase LCK | LCK | P06239 | CHEMBL258 | Kinase | 0.104672 | 75 / 0Â Â Â Â Â | Cannabielsoin |
| Phosphodiesterase 7A | PDE7A | Q13946 | CHEMBL3012 | Phosphodiesterase | 0.104672 | 36 / 0Â Â Â Â Â | Cannabielsoin |
| Tyrosine-protein kinase HCK | HCK | P08631 | CHEMBL3234 | Kinase | 0.104672 | 27 / 0Â Â Â Â Â | Cannabielsoin |
| Tyrosine-protein kinase Lyn | LYN | P07948 | CHEMBL3905 | Kinase | 0.104672 | 19 / 0Â Â Â Â Â | Cannabielsoin |
| Tyrosine-protein kinase FGR | FGR | P09769 | CHEMBL4454 | Kinase | 0.104672 | 9 / 0Â Â Â Â Â | Cannabielsoin |
| Ephrin type-A receptor 3 | EPHA3 | P29320 | CHEMBL4954 | Kinase | 0.104672 | 6 / 0Â Â Â Â Â | Cannabielsoin |
| Discoidin domain-containing receptor 2 | DDR2 | Q16832 | CHEMBL5122 | Kinase | 0.104672 | 7 / 0Â Â Â Â Â | Cannabielsoin |
| Tyrosine-protein kinase BTK | BTK | Q06187 | CHEMBL5251 | Kinase | 0.104672 | 42 / 0Â Â Â Â Â | Cannabielsoin |
| Epithelial discoidin domain-containing receptor 1 | DDR1 | Q08345 | CHEMBL5319 | Kinase | 0.104672 | 10 / 0Â Â Â Â Â | Cannabielsoin |
| N-arachidonyl glycine receptor | GPR18 | Q14330 | CHEMBL2384898 | Family A G protein-coupled receptor | 0.104672 | 0 / 2Â Â Â Â Â | Cannabielsoin |
| Vasopressin V1a receptor | AVPR1A | P37288 | CHEMBL1889 | Family A G protein-coupled receptor | 0.104672 | 49 / 0Â Â Â Â Â | Cannabielsoin |
| Lymphocyte differentiation antigen CD38 | CD38 | P28907 | CHEMBL4660 | Enzyme | 0.104672 | 16 / 0Â Â Â Â Â | Cannabielsoin |
| Tyrosine-protein kinase FYN | FYN | P06241 | CHEMBL1841 | Kinase | 0.104672 | 19 / 0Â Â Â Â Â | Cannabielsoin |
| Cholecystokinin A receptor | CCKAR | P32238 | CHEMBL1901 | Family A G protein-coupled receptor | 0.104672 | 21 / 0Â Â Â Â Â | Cannabielsoin |
| P2X purinoceptor 7 | P2RX7 | Q99572 | CHEMBL4805 | Ligand-gated ion channel | 0.104672 | 37 / 0Â Â Â Â Â | Cannabielsoin |
| Epoxide hydratase | EPHX2 | P34913 | CHEMBL2409 | Protease | 0.104672 | 102 / 0Â Â Â Â Â | Cannabielsoin |
| Protein kinase C delta | PRKCD | Q05655 | CHEMBL2996 | Kinase | 0.104672 | 90 / 0Â Â Â Â Â | Cannabielsoin |
| C-C chemokine receptor type 8 | CCR8 | P51685 | CHEMBL4596 | Family A G protein-coupled receptor | 0.104672 | 5 / 0Â Â Â Â Â | Cannabielsoin |
| DNA-dependent protein kinase | PRKDC | P78527 | CHEMBL3142 | Kinase | 0.104672 | 142 / 0Â Â Â Â Â | Cannabielsoin |
| PI3-kinase p110-beta subunit | PIK3CB | P42338 | CHEMBL3145 | Enzyme | 0.104672 | 265 / 0Â Â Â Â Â | Cannabielsoin |
| Cathepsin D | CTSD | P07339 | CHEMBL2581 | Protease | 0.104672 | 267 / 0Â Â Â Â Â | Cannabielsoin |

| | | | | | | | |
|---|---|---|---|---|---|---|---|
| Delta opioid receptor | OPRD1 | P41143 | CHEMBL236 | Family A G protein-coupled receptor | 0.104672 | 474 / 227Â Â Â Â Â | Cannabielsoin |
| Orexin receptor 1 | HCRTR1 | O43613 | CHEMBL5113 | Family A G protein-coupled receptor | 0.104672 | 40 / 0Â Â Â Â Â | Cannabielsoin |
| Leucine-rich repeat serine/threonine-protein kinase 2 | LRRK2 | Q5S007 | CHEMBL1075104 | Kinase | 0.104672 | 140 / 0Â Â Â Â Â | Cannabielsoin |
| Corticotropin releasing factor receptor 1 | CRHR1 | P34998 | CHEMBL1800 | Family B G protein-coupled receptor | 0.104672 | 82 / 0Â Â Â Â Â | Cannabielsoin |
| Protein kinase C theta | PRKCQ | Q04759 | CHEMBL3920 | Kinase | 0.104672 | 51 / 0Â Â Â Â Â | Cannabielsoin |
| Transient receptor potential cation channel subfamily A member 1 | TRPA1 | O75762 | CHEMBL6007 | Voltage-gated ion channel | 0.104672 | 14 / 0Â Â Â Â Â | Cannabielsoin |
| Tyrosine-protein kinase ABL | ABL1 | P00519 | CHEMBL1862 | Kinase | 0.104672 | 85 / 0Â Â Â Â Â | Cannabielsoin |
| Platelet-derived growth factor receptor | PDGFRA PDGFRB | P16234 P09619 | CHEMBL2095189 | Kinase | 0.104672 | 9 / 0Â Â Â Â Â | Cannabielsoin |
| Tyrosine-protein kinase JAK3 | JAK3 | P52333 | CHEMBL2148 | Kinase | 0.104672 | 199 / 0Â Â Â Â Â | Cannabielsoin |
| Tyrosine-protein kinase JAK2 | JAK2 | O60674 | CHEMBL2971 | Kinase | 0.104672 | 253 / 0Â Â Â Â Â | Cannabielsoin |
| P2X purinoceptor 3 | P2RX3 | P56373 | CHEMBL2998 | Ligand-gated ion channel | 0.104672 | 355 / 0Â Â Â Â Â | Cannabielsoin |
| Phosphodiesterase 1B | PDE1B | Q01064 | CHEMBL4425 | Phosphodiesterase | 0.104672 | 3 / 0Â Â Â Â Â | Cannabielsoin |
| Proto-oncogene tyrosine-protein kinase ROS | ROS1 | P08922 | CHEMBL5568 | Kinase | 0.104672 | 18 / 0Â Â Â Â Â | Cannabielsoin |
| Oxytocin receptor | OXTR | P30559 | CHEMBL2049 | Family A G protein-coupled receptor | 0.104672 | 76 / 0Â Â Â Â Â | Cannabielsoin |
| Cytochrome P450 2C9 | CYP2C9 | P11712 | CHEMBL3397 | Cytochrome P450 | 0.104672 | 7 / 0Â Â Â Â Â | Cannabielsoin |
| Cytochrome P450 3A4 | CYP3A4 | P08684 | CHEMBL340 | Cytochrome P450 | 0.104672 | 5 / 0Â Â Â Â Â | Cannabielsoin |
| Polyadenylate-binding protein 1 | PABPC1 | P11940 | CHEMBL1293286 | Unclassified protein | 0.104672 | 14 / 0Â Â Â Â Â | Cannabielsoin |
| Insulin-like growth factor I receptor | IGF1R | P08069 | CHEMBL1957 | Kinase | 0.104672 | 159 / 0Â Â Â Â Â | Cannabielsoin |
| Insulin receptor | INSR | P06213 | CHEMBL1981 | Kinase | 0.104672 | 29 / 0Â Â Â Â Â | Cannabielsoin |
| Histone deacetylase 3/Nuclear receptor corepressor 2 (HDAC3/NCoR2) | NCOR2 HDAC3 | Q9Y618 O15379 | CHEMBL2111363 | Eraser | 0.104672 | 14 / 0Â Â Â Â Â | Cannabielsoin |
| Tyrosine-protein kinase ITK/TSK | ITK | Q08881 | CHEMBL2959 | Kinase | 0.104672 | 69 / 0Â Â Â Â Â | Cannabielsoin |

| | | | | | | | |
|---|---|---|---|---|---|---|---|
| CDK8/Cyclin C | CCNC CDK8 | P24863 P49336 | CHEMBL3038474 | Kinase | 0.104672 | 11 / 0Â Â Â Â Â | Cannabielsoin |
| Von Hippel-Lindau disease tumor suppressor/Elongin B/Elongin C | VHL | P40337 | CHEMBL3108660 | Unclassified protein | 0.104672 | 6 / 0Â Â Â Â Â | Cannabielsoin |
| Serine/threonine-protein kinase B-raf | BRAF | P15056 | CHEMBL5145 | Kinase | 0.104672 | 130 / 0Â Â Â Â Â | Cannabielsoin |
| Cell division protein kinase 8 | CDK8 | P49336 | CHEMBL5719 | Kinase | 0.104672 | 24 / 0Â Â Â Â Â | Cannabielsoin |
| Isocitrate dehydrogenase [NADP] cytoplasmic | IDH1 | O75874 | CHEMBL2007625 | Enzyme | 0.104672 | 22 / 0Â Â Â Â Â | Cannabielsoin |
| Metabotropic glutamate receptor 5 | GRM5 | P41594 | CHEMBL3227 | Family C G protein-coupled receptor | 0.104672 | 85 / 0Â Â Â Â Â | Cannabielsoin |
| Cystic fibrosis transmembrane conductance regulator | CFTR | P13569 | CHEMBL4051 | Other ion channel | 0.104672 | 22 / 0Â Â Â Â Â | Cannabielsoin |
| Serine/threonine-protein kinase PIM1 | PIM1 | P11309 | CHEMBL2147 | Kinase | 0.104672 | 146 / 0Â Â Â Â Â | Cannabielsoin |
| Liver glycogen phosphorylase | PYGL | P06737 | CHEMBL2568 | Enzyme | 0.104672 | 86 / 0Â Â Â Â Â | Cannabielsoin |
| AMP deaminase 3 | AMPD3 | Q01432 | CHEMBL2912 | Enzyme | 0.104672 | 9 / 0Â Â Â Â Â | Cannabielsoin |
| Heat shock protein HSP 90-beta | HSP90AB1 | P08238 | CHEMBL4303 | Other cytosolic protein | 0.104672 | 55 / 0Â Â Â Â Â | Cannabielsoin |
| Voltage-gated potassium channel subunit Kv1.5 | KCNA5 | P22460 | CHEMBL4306 | Voltage-gated ion channel | 0.104672 | 78 / 0Â Â Â Â Â | Cannabielsoin |
| Kallikrein 5 | KLK5 | Q9Y337 | CHEMBL4447 | Protease | 0.104672 | 3 / 0Â Â Â Â Â | Cannabielsoin |
| Serine/threonine-protein kinase PIM2 | PIM2 | Q9P1W9 | CHEMBL4523 | Kinase | 0.104672 | 108 / 0Â Â Â Â Â | Cannabielsoin |
| Kinesin-like protein 1 | KIF11 | P52732 | CHEMBL4581 | Other cytosolic protein | 0.104672 | 33 / 0Â Â Â Â Â | Cannabielsoin |
| p53-binding protein Mdm-2 | MDM2 | Q00987 | CHEMBL5023 | Other nuclear protein | 0.104672 | 269 / 0Â Â Â Â Â | Cannabielsoin |
| Smoothened homolog | SMO | Q99835 | CHEMBL5971 | Frizzled family G protein-coupled receptor | 0.104672 | 30 / 0Â Â Â Â Â | Cannabielsoin |
| Tyrosine-protein kinase JAK1 | JAK1 | P23458 | CHEMBL2835 | Kinase | 0.104672 | 165 / 0Â Â Â Â Â | Cannabielsoin |
| Sphingosine kinase 2 | SPHK2 | Q9NRA0 | CHEMBL3023 | Enzyme | 0.104672 | 14 / 0Â Â Â Â Â | Cannabielsoin |
| Tyrosine-protein kinase TYK2 | TYK2 | P29597 | CHEMBL3553 | Kinase | 0.104672 | 69 / 0Â Â Â Â Â | Cannabielsoin |
| Interleukin-8 receptor A | CXCR1 | P25024 | CHEMBL4029 | Family A G protein-coupled receptor | 0.104672 | 82 / 0Â Â Â Â Â | Cannabielsoin |

| | | | | | | | |
|---|---|---|---|---|---|---|---|
| Sphingosine kinase 1 | SPHK1 | Q9NYA1 | CHEMBL4394 | Enzyme | 0.104672 | 15 / 0Â Â Â Â Â | Cannabielsoin |
| Acetyl-CoA carboxylase 2 | ACACB | O00763 | CHEMBL4829 | Ligase | 0.104672 | 83 / 0Â Â Â Â Â | Cannabielsoin |
| Cyclin-dependent kinase 2/cyclin E1 | CCNE1 CDK2 | P24864 P24941 | CHEMBL1907605 | Kinase | 0.104672 | 79 / 0Â Â Â Â Â | Cannabielsoin |
| | | | | | | | |
| Cannabinoid receptor 1 | CNR1 | P21554 | CHEMBL218 | Family A G protein-coupled receptor | 0.743196 | 807 / 157Â Â Â Â Â | Cannabigerol |
| Cannabinoid receptor 2 | CNR2 | P34972 | CHEMBL253 | Family A G protein-coupled receptor | 0.651481 | 693 / 136Â Â Â Â Â | Cannabigerol |
| G-protein coupled receptor 55 | GPR55 | Q9Y2T6 | CHEMBL1075322 | Family A G protein-coupled receptor | 0.12661 | 10 / 2Â Â Â Â Â | Cannabigerol |
| Arachidonate 5-lipoxygenase | ALOX5 | P09917 | CHEMBL215 | Oxidoreductase | 0.109946 | 83 / 22Â Â Â Â Â | Cannabigerol |
| DNA polymerase beta (by homology) | POLB | P06746 | CHEMBL2392 | Enzyme | 0.101614 | 0 / 3Â Â Â Â Â | Cannabigerol |
| Arachidonate 15-lipoxygenase | ALOX15 | P16050 | CHEMBL2903 | Enzyme | 0.101614 | 15 / 16Â Â Â Â Â | Cannabigerol |
| Peroxisome proliferator-activated receptor gamma | PPARG | P37231 | CHEMBL235 | Nuclear receptor | 0.101614 | 19 / 17Â Â Â Â Â | Cannabigerol |
| Peroxisome proliferator-activated receptor alpha | PPARA | Q07869 | CHEMBL239 | Nuclear receptor | 0.101614 | 1 / 14Â Â Â Â Â | Cannabigerol |
| Cyclooxygenase-2 | PTGS2 | P35354 | CHEMBL230 | Oxidoreductase | 0.101614 | 137 / 6Â Â Â Â Â | Cannabigerol |
| ADAMTS5 | ADAMTS5 | Q9UNA0 | CHEMBL2285 | Protease | 0.101614 | 48 / 0Â Â Â Â Â | Cannabigerol |
| Poly [ADP-ribose] polymerase-1 | PARP1 | P09874 | CHEMBL3105 | Enzyme | 0.101614 | 55 / 0Â Â Â Â Â | Cannabigerol |
| Cyclin-dependent kinase 4 | CDK4 | P11802 | CHEMBL331 | Kinase | 0.101614 | 30 / 0Â Â Â Â Â | Cannabigerol |
| 11-beta-hydroxysteroid dehydrogenase 1 | HSD11B1 | P28845 | CHEMBL4235 | Enzyme | 0.101614 | 202 / 0Â Â Â Â Â | Cannabigerol |
| Prostanoid EP4 receptor | PTGER4 | P35408 | CHEMBL1836 | Family A G protein-coupled receptor | 0.101614 | 7 / 0Â Â Â Â Â | Cannabigerol |
| Acyl coenzyme A:cholesterol acyltransferase | CES1 | P23141 | CHEMBL2265 | Enzyme | 0.101614 | 23 / 0Â Â Â Â Â | Cannabigerol |
| Carboxylesterase 2 | CES2 | O00748 | CHEMBL3180 | Enzyme | 0.101614 | 3 / 0Â Â Â Â Â | Cannabigerol |
| NAD-dependent deacetylase sirtuin 2 | SIRT2 | Q8IXJ6 | CHEMBL4462 | Eraser | 0.101614 | 37 / 0Â Â Â Â Â | Cannabigerol |
| Adenosine A1 receptor | ADORA1 | P30542 | CHEMBL226 | Family A G protein-coupled receptor | 0.101614 | 196 / 0Â Â Â Â Â | Cannabigerol |
| Beta-secretase 1 | BACE1 | P56817 | CHEMBL4822 | Protease | 0.101614 | 374 / 0Â Â Â Â Â | Cannabigerol |

| | | | | | | | |
|---|---|---|---|---|---|---|---|
| Corticotropin releasing factor receptor 1 | CRHR1 | P34998 | CHEMBL1800 | Family B G protein-coupled receptor | 0.101614 | 111 / 0Â Â Â Â Â | Cannabigerol |
| Phosphodiesterase 10A (by homology) | PDE10A | Q9Y233 | CHEMBL4409 | Phosphodiesterase | 0.101614 | 588 / 0Â Â Â Â Â | Cannabigerol |
| p53-binding protein Mdm-2 | MDM2 | Q00987 | CHEMBL5023 | Other nuclear protein | 0.101614 | 162 / 0Â Â Â Â Â | Cannabigerol |
| Calpain 2 | CAPN2 | P17655 | CHEMBL2382 | Protease | 0.101614 | 42 / 0Â Â Â Â Â | Cannabigerol |
| LDL-associated phospholipase A2 | PLA2G7 | Q13093 | CHEMBL3514 | Enzyme | 0.101614 | 39 / 0Â Â Â Â Â | Cannabigerol |
| Receptor protein-tyrosine kinase erbB-2 | ERBB2 | P04626 | CHEMBL1824 | Kinase | 0.101614 | 65 / 0Â Â Â Â Â | Cannabigerol |
| Epidermal growth factor receptor erbB1 | EGFR | P00533 | CHEMBL203 | Kinase | 0.101614 | 263 / 0Â Â Â Â Â | Cannabigerol |
| Anandamide amidohydrolase | FAAH | O00519 | CHEMBL2243 | Enzyme | 0.101614 | 178 / 0Â Â Â Â Â | Cannabigerol |
| Cystic fibrosis transmembrane conductance regulator | CFTR | P13569 | CHEMBL4051 | Other ion channel | 0.101614 | 18 / 0Â Â Â Â Â | Cannabigerol |
| Neurokinin 1 receptor | TACR1 | P25103 | CHEMBL249 | Family A G protein-coupled receptor | 0.101614 | 121 / 0Â Â Â Â Â | Cannabigerol |
| Histone deacetylase 1 | HDAC1 | Q13547 | CHEMBL325 | Eraser | 0.101614 | 104 / 0Â Â Â Â Â | Cannabigerol |
| G-protein coupled bile acid receptor 1 | GPBAR1 | Q8TDU6 | CHEMBL5409 | Family A G protein-coupled receptor | 0.101614 | 32 / 0Â Â Â Â Â | Cannabigerol |
| Thymidylate synthase | TYMS | P04818 | CHEMBL1952 | Transferase | 0.101614 | 28 / 0Â Â Â Â Â | Cannabigerol |
| Adenosine A2a receptor | ADORA2A | P29274 | CHEMBL251 | Family A G protein-coupled receptor | 0.101614 | 149 / 0Â Â Â Â Â | Cannabigerol |
| Phosphodiesterase 7A | PDE7A | Q13946 | CHEMBL3012 | Phosphodiesterase | 0.101614 | 16 / 0Â Â Â Â Â | Cannabigerol |
| Interleukin-8 receptor A | CXCR1 | P25024 | CHEMBL4029 | Family A G protein-coupled receptor | 0.101614 | 14 / 0Â Â Â Â Â | Cannabigerol |
| Carbonic anhydrase XII | CA12 | O43570 | CHEMBL3242 | Lyase | 0.101614 | 82 / 0Â Â Â Â Â | Cannabigerol |
| Calcium sensing receptor | CASR | P41180 | CHEMBL1878 | Family C G protein-coupled receptor | 0.101614 | 33 / 0Â Â Â Â Â | Cannabigerol |
| Sphingosine 1-phosphate receptor Edg-3 | S1PR3 | Q99500 | CHEMBL3892 | Family A G protein-coupled receptor | 0.101614 | 85 / 0Â Â Â Â Â | Cannabigerol |
| Glutamate NMDA receptor; GRIN1/GRIN2B | GRIN1 GRIN2B | Q05586 Q13224 | CHEMBL1907603 | Ligand-gated ion channel | 0.101614 | 9 / 0Â Â Â Â Â | Cannabigerol |

| | | | | | | | |
|---|---|---|---|---|---|---|---|
| Protein farnesyltransferase | FNTA FNTB | P49354 P49356 | CHEMBL2094108 | Enzyme | 0.101614 | 224 / 0Â Â Â Â Â | Cannabigerol |
| Estradiol 17-beta-dehydrogenase 2 | HSD17B 2 | P37059 | CHEMBL2789 | Enzyme | 0.101614 | 21 / 0Â Â Â Â Â | Cannabigerol |
| Cholesteryl ester transfer protein | CETP | P11597 | CHEMBL3572 | Other ion channel | 0.101614 | 13 / 3Â Â Â Â Â | Cannabigerol |
| Translocator protein (by homology) | TSPO | P30536 | CHEMBL5742 | Membrane receptor | 0.101614 | 241 / 0Â Â Â Â Â | Cannabigerol |
| Melatonin receptor 1A | MTNR1 A | P48039 | CHEMBL1945 | Family A G protein-coupled receptor | 0.101614 | 75 / 0Â Â Â Â Â | Cannabigerol |
| Melatonin receptor 1B | MTNR1 B | P49286 | CHEMBL1946 | Family A G protein-coupled receptor | 0.101614 | 67 / 0Â Â Â Â Â | Cannabigerol |
| Cathepsin K | CTSK | P43235 | CHEMBL268 | Protease | 0.101614 | 261 / 2Â Â Â Â Â | Cannabigerol |
| P-glycoprotein 1 | ABCB1 | P08183 | CHEMBL4302 | Primary active transporter | 0.101614 | 19 / 0Â Â Â Â Â | Cannabigerol |
| NADPH oxidase 4 | NOX4 | Q9NPH 5 | CHEMBL1250375 | Enzyme | 0.101614 | 3 / 0Â Â Â Â Â | Cannabigerol |
| Vasopressin V2 receptor (by homology) | AVPR2 | P30518 | CHEMBL1790 | Family A G protein-coupled receptor | 0.101614 | 62 / 0Â Â Â Â Â | Cannabigerol |
| Oxytocin receptor (by homology) | OXTR | P30559 | CHEMBL2049 | Family A G protein-coupled receptor | 0.101614 | 97 / 0Â Â Â Â Â | Cannabigerol |
| Calpain 1 | CAPN1 | P07384 | CHEMBL3891 | Protease | 0.101614 | 83 / 0Â Â Â Â Â | Cannabigerol |
| Pantothenate kinase 3 | PANK3 | Q9H99 9 | CHEMBL3407328 | Enzyme | 0.101614 | 2 / 0Â Â Â Â Â | Cannabigerol |
| Pyroglutamylated RFamide peptide receptor | QRFPR | Q96P65 | CHEMBL5852 | Family A G protein-coupled receptor | 0.101614 | 7 / 0Â Â Â Â Â | Cannabigerol |
| Monoamine oxidase A | MAOA | P21397 | CHEMBL1951 | Oxidoreductase | 0.101614 | 79 / 0Â Â Â Â Â | Cannabigerol |
| GABA-A receptor; alpha-3/beta-3/gamma-2 | GABRB 3 GABRA 3 GABRG 2 | P28472 P34903 P18507 | CHEMBL2094120 | Ligand-gated ion channel | 0.101614 | 48 / 0Â Â Â Â Â | Cannabigerol |
| GABA-A receptor; alpha-1/beta-3/gamma-2 | GABRB 3 GABRG 2 GABRA 1 | P28472 P18507 P14867 | CHEMBL2094121 | Ligand-gated ion channel | 0.101614 | 40 / 0Â Â Â Â Â | Cannabigerol |
| GABA-A receptor; alpha-5/beta-3/gamma-2 | GABRB 3 GABRG 2 GABRA 5 | P28472 P18507 P31644 | CHEMBL2094122 | Ligand-gated ion channel | 0.101614 | 48 / 0Â Â Â Â Â | Cannabigerol |

| | | | | | | | |
|---|---|---|---|---|---|---|---|
| GABA-A receptor; alpha-2/beta-3/gamma-2 | GABRA2 GABRB3 GABRG2 | P47869 P28472 P18507 | CHEMBL2094130 | Ligand-gated ion channel | 0.101614 | 51 / 0Â Â Â Â Â | Cannabigerol |
| Neurokinin 3 receptor | TACR3 | P29371 | CHEMBL4429 | Family A G protein-coupled receptor | 0.101614 | 112 / 0Â Â Â Â Â | Cannabigerol |
| L-lactate dehydrogenase A chain | LDHA | P00338 | CHEMBL4835 | Enzyme | 0.101614 | 5 / 0Â Â Â Â Â | Cannabigerol |
| Calpain 1 | CAPN1 CAPNS1 | P07384 P04632 | CHEMBL2111357 | Protease | 0.101614 | 26 / 0Â Â Â Â Â | Cannabigerol |
| Cholecystokinin B receptor | CCKBR | P32239 | CHEMBL298 | Family A G protein-coupled receptor | 0.101614 | 400 / 0Â Â Â Â Â | Cannabigerol |
| Serotonin 5a (5-HT5a) receptor | HTR5A | P47898 | CHEMBL3426 | Family A G protein-coupled receptor | 0.101614 | 7 / 0Â Â Â Â Â | Cannabigerol |
| Voltage-gated potassium channel subunit Kv1.5 | KCNA5 | P22460 | CHEMBL4306 | Voltage-gated ion channel | 0.101614 | 105 / 0Â Â Â Â Â | Cannabigerol |
| P2X purinoceptor 7 | P2RX7 | Q99572 | CHEMBL4805 | Ligand-gated ion channel | 0.101614 | 66 / 0Â Â Â Â Â | Cannabigerol |
| Alkaline phosphatase, tissue-nonspecific isozyme | ALPL | P05186 | CHEMBL5979 | Enzyme | 0 | 10 / 0Â Â Â Â Â | Cannabigerol |
| Vascular endothelial growth factor receptor 2 | KDR | P35968 | CHEMBL279 | Kinase | 0 | 348 / 13Â Â Â Â Â | Cannabigerol |
| Sodium channel protein type IX alpha subunit | SCN9A | Q15858 | CHEMBL4296 | Voltage-gated ion channel | 0 | 75 / 0Â Â Â Â Â | Cannabigerol |
| Thrombin and coagulation factor X | F10 | P00742 | CHEMBL244 | Protease | 0 | 231 / 0Â Â Â Â Â | Cannabigerol |
| Serine/threonine-protein kinase PLK1 | PLK1 | P53350 | CHEMBL3024 | Kinase | 0 | 19 / 0Â Â Â Â Â | Cannabigerol |
| Kinesin-1 heavy chain/ Tyrosine-protein kinase receptor RET | RET | P07949 | CHEMBL2041 | Kinase | 0 | 27 / 0Â Â Â Â Â | Cannabigerol |
| Dopamine D4 receptor | DRD4 | P21917 | CHEMBL219 | Family A G protein-coupled receptor | 0 | 72 / 0Â Â Â Â Â | Cannabigerol |
| Beta secretase 2 | BACE2 | Q9Y5Z0 | CHEMBL2525 | Protease | 0 | 97 / 0Â Â Â Â Â | Cannabigerol |
| GABA receptor alpha-1 subunit | GABRA1 | P14867 | CHEMBL1962 | Ligand-gated ion channel | 0 | 24 / 0Â Â Â Â Â | Cannabigerol |
| GABA receptor alpha-2 subunit | GABRA2 | P47869 | CHEMBL4956 | Ligand-gated ion channel | 0 | 32 / 0Â Â Â Â Â | Cannabigerol |

| | | | | | | | |
|---|---|---|---|---|---|---|---|
| Tyrosine-protein kinase JAK3 | JAK3 | P52333 | CHEMBL2148 | Kinase | 0 | 207 / 0Â Â Â Â Â | Cannabigerol |
| Adenosine A2b receptor | ADORA2B | P29275 | CHEMBL255 | Family A G protein-coupled receptor | 0 | 26 / 0Â Â Â Â Â | Cannabigerol |
| Prenyl protein specific protease | RCE1 | Q9Y256 | CHEMBL3411 | Protease | 0 | 6 / 0Â Â Â Â Â | Cannabigerol |
| Cytochrome b-c1 complex subunit 7 | UQCRB | P14927 | CHEMBL1671612 | Transmembrane 1-electron transfer carriers | 0 | 1 / 0Â Â Â Â Â | Cannabigerol |
| Integrin alpha-4/beta-7 | ITGB7 ITGA4 | P26010 P13612 | CHEMBL2095184 | Membrane receptor | 0 | 6 / 0Â Â Â Â Â | Cannabigerol |
| 3-phosphoinositide dependent protein kinase-1 | PDPK1 | O15530 | CHEMBL2534 | Kinase | 0 | 36 / 0Â Â Â Â Â | Cannabigerol |
| Renin | REN | P00797 | CHEMBL286 | Protease | 0 | 15 / 0Â Â Â Â Â | Cannabigerol |
| Leukotriene A4 hydrolase | LTA4H | P09960 | CHEMBL4618 | Protease | 0 | 10 / 0Â Â Â Â Â | Cannabigerol |
| Prostanoid EP1 receptor | PTGER1 | P34995 | CHEMBL1811 | Family A G protein-coupled receptor | 0 | 49 / 0Â Â Â Â Â | Cannabigerol |
| Sodium channel protein type V alpha subunit | SCN5A | Q14524 | CHEMBL1980 | Voltage-gated ion channel | 0 | 21 / 0Â Â Â Â Â | Cannabigerol |
| c-Jun N-terminal kinase 1 | MAPK8 | P45983 | CHEMBL2276 | Kinase | 0 | 123 / 0Â Â Â Â Â | Cannabigerol |
| c-Jun N-terminal kinase 3 | MAPK10 | P53779 | CHEMBL2637 | Kinase | 0 | 89 / 0Â Â Â Â Â | Cannabigerol |
| PI3-kinase p110-alpha subunit | PIK3CA | P42336 | CHEMBL4005 | Enzyme | 0 | 127 / 0Â Â Â Â Â | Cannabigerol |
| c-Jun N-terminal kinase 2 | MAPK9 | P45984 | CHEMBL4179 | Kinase | 0 | 58 / 0Â Â Â Â Â | Cannabigerol |
| Sodium channel protein type II alpha subunit | SCN2A | Q99250 | CHEMBL4187 | Voltage-gated ion channel | 0 | 30 / 0Â Â Â Â Â | Cannabigerol |
| Isocitrate dehydrogenase [NADP] cytoplasmic | IDH1 | O75874 | CHEMBL2007625 | Enzyme | 0 | 112 / 0Â Â Â Â Â | Cannabigerol |
| Alpha-1d adrenergic receptor | ADRA1D | P25100 | CHEMBL223 | Family A G protein-coupled receptor | 0 | 34 / 0Â Â Â Â Â | Cannabigerol |
| Alpha-1a adrenergic receptor | ADRA1A | P35348 | CHEMBL229 | Family A G protein-coupled receptor | 0 | 45 / 0Â Â Â Â Â | Cannabigerol |
| Interleukin-8 receptor B | CXCR2 | P25025 | CHEMBL2434 | Family A G protein-coupled receptor | 0 | 52 / 0Â Â Â Â Â | Cannabigerol |
| Carboxypeptidase B | CPB1 | P15086 | CHEMBL2552 | Protease | 0 | 2 / 0Â Â Â Â Â | Cannabigerol |
| Tyrosine-protein kinase JAK1 | JAK1 | P23458 | CHEMBL2835 | Kinase | 0 | 71 / 0Â Â Â Â Â | Cannabigerol |

| | | | | | | | |
|---|---|---|---|---|---|---|---|
| Tyrosine-protein kinase JAK2 | JAK2 | O60674 | CHEMBL2971 | Kinase | 0 | 160 / 0Â Â Â Â Â | Cannabigerol |
| Cyclin-dependent kinase 2 | CDK2 | P24941 | CHEMBL301 | Kinase | 0 | 84 / 0Â Â Â Â Â | Cannabigerol |
| Cyclin-dependent kinase 1 | CDK1 | P06493 | CHEMBL308 | Kinase | 0 | 65 / 0Â Â Â Â Â | Cannabigerol |
| Heat shock protein HSP 90-beta | HSP90AB1 | P08238 | CHEMBL4303 | Other cytosolic protein | 0 | 64 / 0Â Â Â Â Â | Cannabigerol |
| | | | | | | | |
| Cannabinoid receptor 1 | CNR1 | P21554 | CHEMBL218 | Family A G protein-coupled receptor | 0.226582 | 759 / 73Â Â Â Â Â | Cannabinodiol |
| Cannabinoid receptor 2 | CNR2 | P34972 | CHEMBL253 | Family A G protein-coupled receptor | 0.209877 | 632 / 73Â Â Â Â Â | Cannabinodiol |
| Epidermal growth factor receptor erbB1 | EGFR | P00533 | CHEMBL203 | Kinase | 0.101614 | 230 / 0Â Â Â Â Â | Cannabinodiol |
| Receptor protein-tyrosine kinase erbB-2 | ERBB2 | P04626 | CHEMBL1824 | Kinase | 0.101614 | 44 / 0Â Â Â Â Â | Cannabinodiol |
| Glycine receptor subunit alpha-1 | GLRA1 | P23415 | CHEMBL5845 | Ligand-gated ion channel | 0.101614 | 1 / 0Â Â Â Â Â | Cannabinodiol |
| Cystic fibrosis transmembrane conductance regulator | CFTR | P13569 | CHEMBL4051 | Other ion channel | 0.101614 | 11 / 0Â Â Â Â Â | Cannabinodiol |
| Serotonin 2b (5-HT2b) receptor | HTR2B | P41595 | CHEMBL1833 | Family A G protein-coupled receptor | 0.101614 | 31 / 0Â Â Â Â Â | Cannabinodiol |
| Serotonin 2a (5-HT2a) receptor (by homology) | HTR2A | P28223 | CHEMBL224 | Family A G protein-coupled receptor | 0.101614 | 31 / 0Â Â Â Â Â | Cannabinodiol |
| Serotonin 2c (5-HT2c) receptor | HTR2C | P28335 | CHEMBL225 | Family A G protein-coupled receptor | 0.101614 | 41 / 0Â Â Â Â Â | Cannabinodiol |
| NAD-dependent deacetylase sirtuin 2 | SIRT2 | Q8IXJ6 | CHEMBL4462 | Eraser | 0.101614 | 26 / 0Â Â Â Â Â | Cannabinodiol |
| 5-lipoxygenase activating protein | ALOX5AP | P20292 | CHEMBL4550 | Other cytosolic protein | 0.101614 | 57 / 0Â Â Â Â Â | Cannabinodiol |
| Calcium sensing receptor | CASR | P41180 | CHEMBL1878 | Family C G protein-coupled receptor | 0.101614 | 37 / 0Â Â Â Â Â | Cannabinodiol |
| Prenyl protein specific protease | RCE1 | Q9Y256 | CHEMBL3411 | Protease | 0.101614 | 6 / 0Â Â Â Â Â | Cannabinodiol |
| Melanocortin receptor 4 | MC4R | P32245 | CHEMBL259 | Family A G protein-coupled receptor | 0.101614 | 16 / 0Â Â Â Â Â | Cannabinodiol |
| Glucagon receptor | GCGR | P47871 | CHEMBL1985 | Family B G protein- | 0.101614 | 28 / 0Â Â Â Â Â | Cannabinodiol |

| | | | | | | | |
|---|---|---|---|---|---|---|---|
| | | | | coupled receptor | | | |
| Interleukin-8 receptor B | CXCR2 | P25025 | CHEMBL2434 | Family A G protein-coupled receptor | 0.101614 | 41 / 0Â Â Â Â Â | Cannabinodiol |
| Neurokinin 2 receptor | TACR2 | P21452 | CHEMBL2327 | Family A G protein-coupled receptor | 0.101614 | 8 / 0Â Â Â Â Â | Cannabinodiol |
| Epoxide hydratase | EPHX2 | P34913 | CHEMBL2409 | Protease | 0.101614 | 86 / 0Â Â Â Â Â | Cannabinodiol |
| Cathepsin D | CTSD | P07339 | CHEMBL2581 | Protease | 0.101614 | 18 / 0Â Â Â Â Â | Cannabinodiol |
| Poly [ADP-ribose] polymerase-1 | PARP1 | P09874 | CHEMBL3105 | Enzyme | 0.101614 | 13 / 0Â Â Â Â Â | Cannabinodiol |
| Neurokinin 3 receptor | TACR3 | P29371 | CHEMBL4429 | Family A G protein-coupled receptor | 0.101614 | 102 / 0Â Â Â Â Â | Cannabinodiol |
| Beta-secretase 1 | BACE1 | P56817 | CHEMBL4822 | Protease | 0.101614 | 161 / 0Â Â Â Â Â | Cannabinodiol |
| Translocator protein (by homology) | TSPO | P30536 | CHEMBL5742 | Membrane receptor | 0.101614 | 318 / 0Â Â Â Â Â | Cannabinodiol |
| Phosphodiesterase 4B | PDE4B | Q07343 | CHEMBL275 | Phosphodiesterase | 0.101614 | 25 / 0Â Â Â Â Â | Cannabinodiol |
| Mu opioid receptor | OPRM1 | P35372 | CHEMBL233 | Family A G protein-coupled receptor | 0.101614 | 15 / 0Â Â Â Â Â | Cannabinodiol |
| Estradiol 17-beta-dehydrogenase 3 | HSD17B3 | P37058 | CHEMBL4234 | Enzyme | 0.101614 | 3 / 0Â Â Â Â Â | Cannabinodiol |
| 11-beta-hydroxysteroid dehydrogenase 1 | HSD11B1 | P28845 | CHEMBL4235 | Enzyme | 0.101614 | 222 / 0Â Â Â Â Â | Cannabinodiol |
| ADAMTS5 | ADAMTS5 | Q9UNA0 | CHEMBL2285 | Protease | 0.101614 | 29 / 0Â Â Â Â Â | Cannabinodiol |
| Thymidylate synthase | TYMS | P04818 | CHEMBL1952 | Transferase | 0.101614 | 22 / 0Â Â Â Â Â | Cannabinodiol |
| Metabotropic glutamate receptor 5 | GRM5 | P41594 | CHEMBL3227 | Family C G protein-coupled receptor | 0.101614 | 238 / 0Â Â Â Â Â | Cannabinodiol |
| Monoglyceride lipase | MGLL | Q99685 | CHEMBL4191 | Enzyme | 0.101614 | 13 / 0Â Â Â Â Â | Cannabinodiol |
| Quinone reductase 2 | NQO2 | P16083 | CHEMBL3959 | Enzyme | 0.101614 | 7 / 0Â Â Â Â Â | Cannabinodiol |
| p53-binding protein Mdm-2 | MDM2 | Q00987 | CHEMBL5023 | Other nuclear protein | 0.101614 | 48 / 0Â Â Â Â Â | Cannabinodiol |
| Monoamine oxidase B | MAOB | P27338 | CHEMBL2039 | Oxidoreductase | 0.101614 | 142 / 0Â Â Â Â Â | Cannabinodiol |
| Nitric-oxide synthase, brain | NOS1 | P29475 | CHEMBL3568 | Enzyme | 0.101614 | 32 / 0Â Â Â Â Â | Cannabinodiol |
| Nitric-oxide synthase, endothelial | NOS3 | P29474 | CHEMBL4803 | Enzyme | 0.101614 | 14 / 0Â Â Â Â Â | Cannabinodiol |
| Tyrosine-protein kinase JAK2 | JAK2 | O60674 | CHEMBL2971 | Kinase | 0.101614 | 158 / 0Â Â Â Â Â | Cannabinodiol |

| | | | | | | | |
|---|---|---|---|---|---|---|---|
| Cholecystokinin B receptor (by homology) | CCKBR | P32239 | CHEMBL298 | Family A G protein-coupled receptor | 0.101614 | 221 / 0Â Â Â Â Â | Cannabinodiol |
| Serine/threonine-protein kinase PAK 1 | PAK1 | Q13153 | CHEMBL4600 | Kinase | 0.101614 | 1 / 0Â Â Â Â Â | Cannabinodiol |
| Prostanoid EP1 receptor | PTGER1 | P34995 | CHEMBL1811 | Family A G protein-coupled receptor | 0.101614 | 39 / 0Â Â Â Â Â | Cannabinodiol |
| Pyruvate kinase isozymes M1/M2 | PKM | P14618 | CHEMBL1075189 | Enzyme | 0.101614 | 7 / 0Â Â Â Â Â | Cannabinodiol |
| Cyclin-dependent kinase 5/CDK5 activator 1 | CDK5R1 CDK5 | Q15078 Q00535 | CHEMBL1907600 | Kinase | 0.101614 | 90 / 0Â Â Â Â Â | Cannabinodiol |
| Serine/threonine-protein kinase Aurora-B | AURKB | Q96GD4 | CHEMBL2185 | Kinase | 0.101614 | 25 / 0Â Â Â Â Â | Cannabinodiol |
| Serine/threonine-protein kinase Aurora-C | AURKC | Q9UQB9 | CHEMBL3935 | Kinase | 0.101614 | 11 / 0Â Â Â Â Â | Cannabinodiol |
| Serine/threonine-protein kinase Aurora-A | AURKA | O14965 | CHEMBL4722 | Kinase | 0.101614 | 31 / 0Â Â Â Â Â | Cannabinodiol |
| Corticotropin releasing factor receptor 1 | CRHR1 | P34998 | CHEMBL1800 | Family B G protein-coupled receptor | 0 | 153 / 0Â Â Â Â Â | Cannabinodiol |
| Anandamide amidohydrolase | FAAH | O00519 | CHEMBL2243 | Enzyme | 0 | 57 / 0Â Â Â Â Â | Cannabinodiol |
| MAP kinase p38 alpha | MAPK14 | Q16539 | CHEMBL260 | Kinase | 0 | 344 / 0Â Â Â Â Â | Cannabinodiol |
| Mineralocorticoid receptor | NR3C2 | P08235 | CHEMBL1994 | Nuclear receptor | 0 | 67 / 0Â Â Â Â Â | Cannabinodiol |
| Voltage-gated potassium channel subunit Kv1.5 | KCNA5 | P22460 | CHEMBL4306 | Voltage-gated ion channel | 0 | 99 / 0Â Â Â Â Â | Cannabinodiol |
| ATP-binding cassette sub-family G member 2 | ABCG2 | Q9UNQ0 | CHEMBL5393 | Primary active transporter | 0 | 11 / 0Â Â Â Â Â | Cannabinodiol |
| Serine/threonine-protein kinase/endoribonuclease IRE1 | ERN1 | O75460 | CHEMBL1163101 | Enzyme | 0 | 3 / 0Â Â Â Â Â | Cannabinodiol |
| LDL-associated phospholipase A2 | PLA2G7 | Q13093 | CHEMBL3514 | Enzyme | 0 | 15 / 0Â Â Â Â Â | Cannabinodiol |
| Sodium channel protein type II alpha subunit | SCN2A | Q99250 | CHEMBL4187 | Voltage-gated ion channel | 0 | 24 / 0Â Â Â Â Â | Cannabinodiol |
| Muscarinic acetylcholine receptor M1 (by homology) | CHRM1 | P11229 | CHEMBL216 | Family A G protein-coupled receptor | 0 | 37 / 0Â Â Â Â Â | Cannabinodiol |
| Acetylcholinesterase | ACHE | P22303 | CHEMBL220 | Hydrolase | 0 | 84 / 0Â Â Â Â Â | Cannabinodiol |
| Bombesin receptor subtype-3 | BRS3 | P32247 | CHEMBL4080 | Family A G protein-coupled receptor | 0 | 1 / 0Â Â Â Â Â | Cannabinodiol |

| | | | | | | | |
|---|---|---|---|---|---|---|---|
| Alpha-2a adrenergic receptor | ADRA2A | P08913 | CHEMBL1867 | Family A G protein-coupled receptor | 0 | 63 / 0Â Â Â Â Â | Cannabinodiol |
| Adrenergic receptor alpha-2 | ADRA2C | P18825 | CHEMBL1916 | Family A G protein-coupled receptor | 0 | 59 / 0Â Â Â Â Â | Cannabinodiol |
| Alpha-2b adrenergic receptor | ADRA2B | P18089 | CHEMBL1942 | Family A G protein-coupled receptor | 0 | 57 / 0Â Â Â Â Â | Cannabinodiol |
| Macrophage migration inhibitory factor | MIF | P14174 | CHEMBL2085 | Enzyme | 0 | 5 / 0Â Â Â Â Â | Cannabinodiol |
| Cyclin-dependent kinase 1/cyclin B | CCNB3 CDK1 CCNB1 CCNB2 | Q8WWL7 P06493 P14635 O95067 | CHEMBL2094127 | Other cytosolic protein | 0 | 43 / 0Â Â Â Â Â | Cannabinodiol |
| Bromodomain-containing protein 1 | BRD1 | O95696 | CHEMBL2176774 | Reader | 0 | 6 / 0Â Â Â Â Â | Cannabinodiol |
| Beta secretase 2 | BACE2 | Q9Y5Z0 | CHEMBL2525 | Protease | 0 | 33 / 0Â Â Â Â Â | Cannabinodiol |
| Transcription intermediary factor 1-alpha | TRIM24 | O15164 | CHEMBL3108638 | Reader | 0 | 9 / 0Â Â Â Â Â | Cannabinodiol |
| Peregrin | BRPF1 | P55201 | CHEMBL3132741 | Reader | 0 | 20 / 0Â Â Â Â Â | Cannabinodiol |
| Nischarin | NISCH | Q9Y2I1 | CHEMBL3923 | Other cytosolic protein | 0 | 41 / 0Â Â Â Â Â | Cannabinodiol |
| Metabotropic glutamate receptor 2 | GRM2 | Q14416 | CHEMBL5137 | Family C G protein-coupled receptor | 0 | 38 / 0Â Â Â Â Â | Cannabinodiol |
| Cholecystokinin A receptor | CCKAR | P32238 | CHEMBL1901 | Family A G protein-coupled receptor | 0 | 19 / 0Â Â Â Â Â | Cannabinodiol |
| Tyrosine-protein kinase JAK3 | JAK3 | P52333 | CHEMBL2148 | Kinase | 0 | 169 / 0Â Â Â Â Â | Cannabinodiol |
| Calcium-activated potassium channel subunit alpha-1 | KCNMA1 | Q12791 | CHEMBL4304 | Voltage-gated ion channel | 0 | 13 / 0Â Â Â Â Â | Cannabinodiol |
| Alpha-1d adrenergic receptor | ADRA1D | P25100 | CHEMBL223 | Family A G protein-coupled receptor | 0 | 17 / 0Â Â Â Â Â | Cannabinodiol |
| Alpha-1a adrenergic receptor | ADRA1A | P35348 | CHEMBL229 | Family A G protein-coupled receptor | 0 | 41 / 0Â Â Â Â Â | Cannabinodiol |
| Alpha-1b adrenergic receptor | ADRA1B | P35368 | CHEMBL232 | Family A G protein-coupled receptor | 0 | 19 / 0Â Â Â Â Â | Cannabinodiol |
| Glycogen synthase kinase-3 beta | GSK3B | P49841 | CHEMBL262 | Kinase | 0 | 52 / 0Â Â Â Â Â | Cannabinodiol |

| | | | | | | | |
|---|---|---|---|---|---|---|---|
| Serotonin 6 (5-HT6) receptor | HTR6 | P50406 | CHEMBL3371 | Family A G protein-coupled receptor | 0 | 59 / 0Â Â Â Â Â | Cannabinodiol |
| 6-phosphofructo-2-kinase/fructose-2,6-bisphosphatase 3 | PFKFB3 | Q16875 | CHEMBL2331053 | Enzyme | 0 | 50 / 0Â Â Â Â Â | Cannabinodiol |
| Mitogen-activated protein kinase kinase kinase 8 | MAP3K 8 | P41279 | CHEMBL4899 | Kinase | 0 | 43 / 0Â Â Â Â Â | Cannabinodiol |
| Serine/threonine-protein kinase B-raf | BRAF | P15056 | CHEMBL5145 | Kinase | 0 | 74 / 0Â Â Â Â Â | Cannabinodiol |
| Serine/threonine-protein kinase TNNI3K | TNNI3K | Q59H1 8 | CHEMBL5260 | Kinase | 0 | 35 / 0Â Â Â Â Â | Cannabinodiol |
| GABA-A receptor; alpha-3/beta-3/gamma-2 | GABRB 3 GABRA 3 GABRG 2 | P28472 P34903 P18507 | CHEMBL2094120 | Ligand-gated ion channel | 0 | 62 / 0Â Â Â Â Â | Cannabinodiol |
| GABA-A receptor; alpha-1/beta-3/gamma-2 | GABRB 3 GABRG 2 GABRA 1 | P28472 P18507 P14867 | CHEMBL2094121 | Ligand-gated ion channel | 0 | 53 / 0Â Â Â Â Â | Cannabinodiol |
| GABA-A receptor; alpha-5/beta-3/gamma-2 | GABRB 3 GABRG 2 GABRA 5 | P28472 P18507 P31644 | CHEMBL2094122 | Ligand-gated ion channel | 0 | 58 / 0Â Â Â Â Â | Cannabinodiol |
| GABA-A receptor; alpha-2/beta-3/gamma-2 | GABRA 2 GABRB 3 GABRG 2 | P47869 P28472 P18507 | CHEMBL2094130 | Ligand-gated ion channel | 0 | 59 / 0Â Â Â Â Â | Cannabinodiol |
| Prostaglandin E synthase | PTGES | O14684 | CHEMBL5658 | Enzyme | 0 | 31 / 0Â Â Â Â Â | Cannabinodiol |
| Melatonin receptor 1B | MTNR1 B | P49286 | CHEMBL1946 | Family A G protein-coupled receptor | 0 | 49 / 0Â Â Â Â Â | Cannabinodiol |
| Adenosine A1 receptor (by homology) | ADORA 1 | P30542 | CHEMBL226 | Family A G protein-coupled receptor | 0 | 247 / 0Â Â Â Â Â | Cannabinodiol |
| Protein kinase C delta | PRKCD | Q05655 | CHEMBL2996 | Kinase | 0 | 19 / 0Â Â Â Â Â | Cannabinodiol |
| Tyrosine-protein kinase Lyn | LYN | P07948 | CHEMBL3905 | Kinase | 0 | 6 / 0Â Â Â Â Â | Cannabinodiol |
| Protein kinase C theta | PRKCQ | Q04759 | CHEMBL3920 | Kinase | 0 | 35 / 0Â Â Â Â Â | Cannabinodiol |
| Dual specificity protein phosphatase 3 | DUSP3 | P51452 | CHEMBL2635 | Phosphatase | 0 | 4 / 0Â Â Â Â Â | Cannabinodiol |

| | | | | | | | |
|---|---|---|---|---|---|---|---|
| Monoamine oxidase A | MAOA | P21397 | CHEMBL1951 | Oxidoreductase | 0 | 92 / 0Â Â Â Â Â | Cannabinodiol |
| Adenosine A2a receptor | ADORA2A | P29274 | CHEMBL251 | Family A G protein-coupled receptor | 0 | 138 / 0Â Â Â Â Â | Cannabinodiol |
| Adenosine A3 receptor | ADORA3 | P0DMS8 | CHEMBL256 | Family A G protein-coupled receptor | 0 | 177 / 0Â Â Â Â Â | Cannabinodiol |
| Carbonic anhydrase I | CA1 | P00915 | CHEMBL261 | Lyase | 0 | 87 / 0Â Â Â Â Â | Cannabinodiol |
| Carbonic anhydrase IX | CA9 | Q16790 | CHEMBL3594 | Lyase | 0 | 86 / 0Â Â Â Â Â | Cannabinodiol |
| Androgen Receptor | AR | P10275 | CHEMBL1871 | Nuclear receptor | 0 | 73 / 0Â Â Â Â Â | Cannabinodiol |
| Sodium channel protein type V alpha subunit | SCN5A | Q14524 | CHEMBL1980 | Voltage-gated ion channel | 0 | 19 / 0Â Â Â Â Â | Cannabinodiol |
| Carbonic anhydrase II | CA2 | P00918 | CHEMBL205 | Lyase | 0 | 110 / 0Â Â Â Â Â | Cannabinodiol |
| Carbonic anhydrase XII | CA12 | O43570 | CHEMBL3242 | Lyase | 0 | 67 / 0Â Â Â Â Â | Cannabinodiol |
| | | | | | | | |
| Cannabinoid receptor 1 | CNR1 | P21554 | CHEMBL218 | Family A G protein-coupled receptor | 0.986222 | 822 / 96Â Â Â Â Â | Cannabinol |
| Cannabinoid receptor 2 | CNR2 | P34972 | CHEMBL253 | Family A G protein-coupled receptor | 0.986222 | 736 / 99Â Â Â Â Â | Cannabinol |
| Estrogen receptor beta | ESR2 | Q92731 | CHEMBL242 | Nuclear receptor | 0.101614 | 12 / 35Â Â Â Â Â | Cannabinol |
| Estrogen receptor alpha | ESR1 | P03372 | CHEMBL206 | Nuclear receptor | 0.101614 | 18 / 38Â Â Â Â Â | Cannabinol |
| N-arachidonyl glycine receptor | GPR18 | Q14330 | CHEMBL2384898 | Family A G protein-coupled receptor | 0.101614 | 2 / 0Â Â Â Â Â | Cannabinol |
| Vascular endothelial growth factor receptor 2 | KDR | P35968 | CHEMBL279 | Kinase | 0.101614 | 229 / 0Â Â Â Â Â | Cannabinol |
| G-protein coupled receptor 55 | GPR55 | Q9Y2T6 | CHEMBL1075322 | Family A G protein-coupled receptor | 0.101614 | 10 / 0Â Â Â Â Â | Cannabinol |
| Cystic fibrosis transmembrane conductance regulator | CFTR | P13569 | CHEMBL4051 | Other ion channel | 0.101614 | 12 / 0Â Â Â Â Â | Cannabinol |
| 5-lipoxygenase activating protein | ALOX5AP | P20292 | CHEMBL4550 | Other cytosolic protein | 0.101614 | 56 / 0Â Â Â Â Â | Cannabinol |
| Receptor protein-tyrosine kinase erbB-2 | ERBB2 | P04626 | CHEMBL1824 | Kinase | 0.101614 | 48 / 0Â Â Â Â Â | Cannabinol |
| Epidermal growth factor receptor erbB1 | EGFR | P00533 | CHEMBL203 | Kinase | 0.101614 | 228 / 0Â Â Â Â Â | Cannabinol |

| | | | | | | | |
|---|---|---|---|---|---|---|---|
| Calcium sensing receptor | CASR | P41180 | CHEMBL1878 | Family C G protein-coupled receptor | 0.101614 | 35 / 0Â Â Â Â Â | Cannabinol |
| Corticotropin releasing factor receptor 1 | CRHR1 | P34998 | CHEMBL1800 | Family B G protein-coupled receptor | 0.101614 | 147 / 0Â Â Â Â Â | Cannabinol |
| 11-beta-hydroxysteroid dehydrogenase 1 | HSD11B1 | P28845 | CHEMBL4235 | Enzyme | 0.101614 | 213 / 0Â Â Â Â Â | Cannabinol |
| Phosphodiesterase 4B | PDE4B | Q07343 | CHEMBL275 | Phosphodiesterase | 0.101614 | 36 / 0Â Â Â Â Â | Cannabinol |
| NAD-dependent deacetylase sirtuin 2 | SIRT2 | Q8IXJ6 | CHEMBL4462 | Eraser | 0.101614 | 33 / 0Â Â Â Â Â | Cannabinol |
| ADAMTS5 | ADAMTS5 | Q9UNA0 | CHEMBL2285 | Protease | 0.101614 | 36 / 0Â Â Â Â Â | Cannabinol |
| Cathepsin D | CTSD | P07339 | CHEMBL2581 | Protease | 0.101614 | 41 / 0Â Â Â Â Â | Cannabinol |
| Prenyl protein specific protease | RCE1 | Q9Y256 | CHEMBL3411 | Protease | 0.101614 | 6 / 0Â Â Â Â Â | Cannabinol |
| Epoxide hydratase | EPHX2 | P34913 | CHEMBL2409 | Protease | 0.101614 | 93 / 0Â Â Â Â Â | Cannabinol |
| Adenosine A2a receptor | ADORA2A | P29274 | CHEMBL251 | Family A G protein-coupled receptor | 0.101614 | 184 / 0Â Â Â Â Â | Cannabinol |
| Adenosine A2b receptor | ADORA2B | P29275 | CHEMBL255 | Family A G protein-coupled receptor | 0.101614 | 40 / 0Â Â Â Â Â | Cannabinol |
| Protoporphyrinogen oxidase | PPOX | P50336 | CHEMBL1926488 | Oxidoreductase | 0.101614 | 1 / 0Â Â Â Â Â | Cannabinol |
| Translocator protein (by homology) | TSPO | P30536 | CHEMBL5742 | Membrane receptor | 0.101614 | 344 / 0Â Â Â Â Â | Cannabinol |
| Protein kinase C (PKC) | PRKCZ | Q05513 | CHEMBL3438 | Kinase | 0.101614 | 2 / 0Â Â Â Â Â | Cannabinol |
| Nitric oxide synthase, inducible (by homology) | NOS2 | P35228 | CHEMBL4481 | Enzyme | 0.101614 | 83 / 0Â Â Â Â Â | Cannabinol |
| Muscarinic acetylcholine receptor M1 (by homology) | CHRM1 | P11229 | CHEMBL216 | Family A G protein-coupled receptor | 0.101614 | 35 / 0Â Â Â Â Â | Cannabinol |
| Isocitrate dehydrogenase [NADP] cytoplasmic | IDH1 | O75874 | CHEMBL2007625 | Enzyme | 0.101614 | 170 / 0Â Â Â Â Â | Cannabinol |
| Bile acid receptor FXR | NR1H4 | Q96RI1 | CHEMBL2047 | Nuclear receptor | 0.101614 | 30 / 0Â Â Â Â Â | Cannabinol |
| Thymidylate synthase | TYMS | P04818 | CHEMBL1952 | Transferase | 0.101614 | 25 / 0Â Â Â Â Â | Cannabinol |
| Melanocortin receptor 4 | MC4R | P32245 | CHEMBL259 | Family A G protein-coupled receptor | 0.101614 | 10 / 0Â Â Â Â Â | Cannabinol |
| Acetylcholinesterase | ACHE | P22303 | CHEMBL220 | Hydrolase | 0.101614 | 85 / 0Â Â Â Â Â | Cannabinol |
| Neurokinin 2 receptor | TACR2 | P21452 | CHEMBL2327 | Family A G protein- | 0.101614 | 12 / 0Â Â Â Â Â | Cannabinol |

| | | | | | | | |
|---|---|---|---|---|---|---|---|
| | | | | coupled receptor | | | |
| Beta secretase 2 | BACE2 | Q9Y5Z0 | CHEMBL2525 | Protease | 0.101614 | 64 / 0Â Â Â Â Â | Cannabinol |
| Phosphodiesterase 7A | PDE7A | Q13946 | CHEMBL3012 | Phosphodiesterase | 0.101614 | 15 / 0Â Â Â Â Â | Cannabinol |
| Quinone reductase 2 | NQO2 | P16083 | CHEMBL3959 | Enzyme | 0.101614 | 9 / 0Â Â Â Â Â | Cannabinol |
| Neurokinin 3 receptor | TACR3 | P29371 | CHEMBL4429 | Family A G protein-coupled receptor | 0.101614 | 116 / 0Â Â Â Â Â | Cannabinol |
| Kinesin-like protein 1 | KIF11 | P52732 | CHEMBL4581 | Other cytosolic protein | 0.101614 | 36 / 0Â Â Â Â Â | Cannabinol |
| Cholecystokinin A receptor | CCKAR | P32238 | CHEMBL1901 | Family A G protein-coupled receptor | 0.101614 | 28 / 0Â Â Â Â Â | Cannabinol |
| Interleukin-8 receptor B | CXCR2 | P25025 | CHEMBL2434 | Family A G protein-coupled receptor | 0.101614 | 46 / 0Â Â Â Â Â | Cannabinol |
| LDL-associated phospholipase A2 | PLA2G7 | Q13093 | CHEMBL3514 | Enzyme | 0.101614 | 20 / 0Â Â Â Â Â | Cannabinol |
| G-protein coupled bile acid receptor 1 | GPBAR1 | Q8TDU6 | CHEMBL5409 | Family A G protein-coupled receptor | 0.101614 | 33 / 0Â Â Â Â Â | Cannabinol |
| Dopamine D1 receptor | DRD1 | P21728 | CHEMBL2056 | Family A G protein-coupled receptor | 0.101614 | 22 / 0Â Â Â Â Â | Cannabinol |
| 6-phosphofructo-2-kinase/fructose-2,6-bisphosphatase 3 | PFKFB3 | Q16875 | CHEMBL2331053 | Enzyme | 0.101614 | 52 / 0Â Â Â Â Â | Cannabinol |
| Poly [ADP-ribose] polymerase-1 | PARP1 | P09874 | CHEMBL3105 | Enzyme | 0.101614 | 22 / 0Â Â Â Â Â | Cannabinol |
| Glucocorticoid receptor | NR3C1 | P04150 | CHEMBL2034 | Nuclear receptor | 0.101614 | 126 / 53Â Â Â Â Â | Cannabinol |
| Methyl-CpG-binding protein 2 | MECP2 | P51608 | CHEMBL3638346 | Reader | 0.101614 | 1 / 0Â Â Â Â Â | Cannabinol |
| Methyl-CpG-binding domain protein 2 | MBD2 | Q9UBB5 | CHEMBL3707462 | Reader | 0.101614 | 2 / 0Â Â Â Â Â | Cannabinol |
| Serine/threonine-protein kinase PAK 1 | PAK1 | Q13153 | CHEMBL4600 | Kinase | 0.101614 | 2 / 0Â Â Â Â Â | Cannabinol |
| Histone deacetylase 6 | HDAC6 | Q9UBN7 | CHEMBL1865 | Eraser | 0.101614 | 26 / 0Â Â Â Â Â | Cannabinol |
| Histone deacetylase 2 | HDAC2 | Q92769 | CHEMBL1937 | Eraser | 0.101614 | 10 / 0Â Â Â Â Â | Cannabinol |
| Estradiol 17-beta-dehydrogenase 2 | HSD17B2 | P37059 | CHEMBL2789 | Enzyme | 0.101614 | 20 / 0Â Â Â Â Â | Cannabinol |
| Histone deacetylase 8 | HDAC8 | Q9BY41 | CHEMBL3192 | Eraser | 0.101614 | 11 / 0Â Â Â Â Â | Cannabinol |
| MAP kinase p38 alpha | MAPK14 | Q16539 | CHEMBL260 | Kinase | 0.101614 | 374 / 0Â Â Â Â Â | Cannabinol |
| Prostanoid EP1 receptor | PTGER1 | P34995 | CHEMBL1811 | Family A G protein- | 0 | 43 / 16Â Â Â Â Â | Cannabinol |

| | | | | | | | |
|---|---|---|---|---|---|---|---|
| | | | | coupled receptor | | | |
| Cytochrome b-c1 complex subunit 7 | UQCRB | P14927 | CHEMBL1671612 | Transmembrane 1-electron transfer carriers | 0 | 1 / 0Â Â Â Â Â | Cannabinol |
| Serotonin 2c (5-HT2c) receptor | HTR2C | P28335 | CHEMBL225 | Family A G protein-coupled receptor | 0 | 41 / 0Â Â Â Â Â | Cannabinol |
| Voltage-gated potassium channel subunit Kv1.5 | KCNA5 | P22460 | CHEMBL4306 | Voltage-gated ion channel | 0 | 111 / 0Â Â Â Â Â | Cannabinol |
| Leukotriene A4 hydrolase | LTA4H | P09960 | CHEMBL4618 | Protease | 0 | 9 / 0Â Â Â Â Â | Cannabinol |
| Peregrin | BRPF1 | P55201 | CHEMBL3132741 | Reader | 0 | 27 / 0Â Â Â Â Â | Cannabinol |
| p53-binding protein Mdm-2 | MDM2 | Q00987 | CHEMBL5023 | Other nuclear protein | 0 | 81 / 0Â Â Â Â Â | Cannabinol |
| Bombesin receptor subtype-3 | BRS3 | P32247 | CHEMBL4080 | Family A G protein-coupled receptor | 0 | 1 / 0Â Â Â Â Â | Cannabinol |
| Mu opioid receptor | OPRM1 | P35372 | CHEMBL233 | Family A G protein-coupled receptor | 0 | 18 / 0Â Â Â Â Â | Cannabinol |
| Tyrosine-protein kinase LCK | LCK | P06239 | CHEMBL258 | Kinase | 0 | 62 / 0Â Â Â Â Â | Cannabinol |
| Adenosine A1 receptor (by homology) | ADORA1 | P30542 | CHEMBL226 | Family A G protein-coupled receptor | 0 | 287 / 0Â Â Â Â Â | Cannabinol |
| Estradiol 17-beta-dehydrogenase 1 | HSD17B1 | P14061 | CHEMBL3181 | Enzyme | 0 | 10 / 0Â Â Â Â Â | Cannabinol |
| Sodium channel protein type II alpha subunit | SCN2A | Q99250 | CHEMBL4187 | Voltage-gated ion channel | 0 | 25 / 0Â Â Â Â Â | Cannabinol |
| Vanilloid receptor | TRPV1 | Q8NER1 | CHEMBL4794 | Voltage-gated ion channel | 0 | 116 / 0Â Â Â Â Â | Cannabinol |
| Egl nine homolog 1 | EGLN1 | Q9GZT9 | CHEMBL5697 | Oxidoreductase | 0 | 7 / 0Â Â Â Â Â | Cannabinol |
| Tyrosine-protein kinase JAK3 | JAK3 | P52333 | CHEMBL2148 | Kinase | 0 | 209 / 0Â Â Â Â Â | Cannabinol |
| Phosphodiesterase 2A | PDE2A | O00408 | CHEMBL2652 | Phosphodiesterase | 0 | 56 / 0Â Â Â Â Â | Cannabinol |
| Tyrosine-protein kinase JAK2 | JAK2 | O60674 | CHEMBL2971 | Kinase | 0 | 170 / 0Â Â Â Â Â | Cannabinol |
| Calcium-activated potassium channel subunit alpha-1 | KCNMA1 | Q12791 | CHEMBL4304 | Voltage-gated ion channel | 0 | 13 / 0Â Â Â Â Â | Cannabinol |
| Phosphodiesterase 10A | PDE10A | Q9Y233 | CHEMBL4409 | Phosphodiesterase | 0 | 466 / 0Â Â Â Â Â | Cannabinol |
| Muscarinic acetylcholine receptor M4 | CHRM4 | P08173 | CHEMBL1821 | Family A G protein- | 0 | 11 / 0Â Â Â Â Â | Cannabinol |

| | | | | | | | |
|---|---|---|---|---|---|---|---|
| | | | | coupled receptor | | | |
| Muscarinic acetylcholine receptor M5 | CHRM5 | P08912 | CHEMBL2035 | Family A G protein-coupled receptor | 0 | 9 / 0Â Â Â Â Â | Cannabinol |
| Muscarinic acetylcholine receptor M2 | CHRM2 | P08172 | CHEMBL211 | Family A G protein-coupled receptor | 0 | 9 / 0Â Â Â Â Â | Cannabinol |
| Muscarinic acetylcholine receptor M3 | CHRM3 | P20309 | CHEMBL245 | Family A G protein-coupled receptor | 0 | 61 / 0Â Â Â Â Â | Cannabinol |
| Neuropeptide Y receptor type 5 | NPY5R | Q15761 | CHEMBL4561 | Family A G protein-coupled receptor | 0 | 144 / 0Â Â Â Â Â | Cannabinol |
| ATP-binding cassette sub-family G member 2 | ABCG2 | Q9UNQ0 | CHEMBL5393 | Primary active transporter | 0 | 17 / 0Â Â Â Â Â | Cannabinol |
| Glutamate NMDA receptor; GRIN1/GRIN2B | GRIN1 GRIN2B | Q05586 Q13224 | CHEMBL1907603 | Ligand-gated ion channel | 0 | 2 / 0Â Â Â Â Â | Cannabinol |
| Adenosine A3 receptor | ADORA3 | P0DMS8 | CHEMBL256 | Family A G protein-coupled receptor | 0 | 220 / 0Â Â Â Â Â | Cannabinol |
| Cholecystokinin B receptor | CCKBR | P32239 | CHEMBL298 | Family A G protein-coupled receptor | 0 | 266 / 0Â Â Â Â Â | Cannabinol |
| Hormone sensitive lipase | LIPE | Q05469 | CHEMBL3590 | Enzyme | 0 | 46 / 0Â Â Â Â Â | Cannabinol |
| Cyclin-dependent kinase 5/CDK5 activator 1 | CDK5R1 CDK5 | Q15078 Q00535 | CHEMBL1907600 | Kinase | 0 | 85 / 0Â Â Â Â Â | Cannabinol |
| Cyclin-dependent kinase 1/cyclin B | CCNB3 CDK1 CCNB1 CCNB2 | Q8WWL7 P06493 P14635 O95067 | CHEMBL2094127 | Other cytosolic protein | 0 | 45 / 0Â Â Â Â Â | Cannabinol |
| GABA-A receptor; GABA-A site (alpha1/beta2 interface) | GABRA1 GABRB2 | P14867 P47870 | CHEMBL1907597 | Ligand-gated ion channel | 0 | 5 / 0Â Â Â Â Â | Cannabinol |
| Interleukin-8 receptor A | CXCR1 | P25024 | CHEMBL4029 | Family A G protein-coupled receptor | 0 | 11 / 0Â Â Â Â Â | Cannabinol |
| Leucine-rich repeat serine/threonine-protein kinase 2 | LRRK2 | Q5S007 | CHEMBL1075104 | Kinase | 0 | 26 / 0Â Â Â Â Â | Cannabinol |
| Sodium channel protein type V alpha subunit | SCN5A | Q14524 | CHEMBL1980 | Voltage-gated ion channel | 0 | 19 / 0Â Â Â Â Â | Cannabinol |
| GABA-A receptor; alpha-3/beta-3/gamma-2 | GABRB3 GABRA | P28472 P34903 P18507 | CHEMBL2094120 | Ligand-gated ion channel | 0 | 85 / 0Â Â Â Â Â | Cannabinol |

| | | | | | | | |
|---|---|---|---|---|---|---|---|
| | 3 GABRG 2 | | | | | | |
| GABA-A receptor; alpha-1/beta-3/gamma-2 | GABRB 3 GABRG 2 GABRA 1 | P28472 P18507 P14867 | CHEMBL2094121 | Ligand-gated ion channel | 0 | 73 / 0Â Â Â Â Â | Cannabinol |
| GABA-A receptor; alpha-5/beta-3/gamma-2 | GABRB 3 GABRG 2 GABRA 5 | P28472 P18507 P31644 | CHEMBL2094122 | Ligand-gated ion channel | 0 | 75 / 0Â Â Â Â Â | Cannabinol |
| GABA-A receptor; alpha-2/beta-3/gamma-2 | GABRA 2 GABRB 3 GABRG 2 | P47869 P28472 P18507 | CHEMBL2094130 | Ligand-gated ion channel | 0 | 79 / 0Â Â Â Â Â | Cannabinol |
| Matrix metalloproteinase 9 | MMP9 | P14780 | CHEMBL321 | Protease | 0 | 25 / 0Â Â Â Â Â | Cannabinol |
| Cathepsin (V and K) | CTSV | O60911 | CHEMBL3272 | Protease | 0 | 17 / 0Â Â Â Â Â | Cannabinol |
| Matrix metalloproteinase 2 | MMP2 | P08253 | CHEMBL333 | Protease | 0 | 37 / 0Â Â Â Â Â | Cannabinol |
| FK506-binding protein 1A | FKBP1A | P62942 | CHEMBL1902 | Isomerase | 0 | 70 / 0Â Â Â Â Â | Cannabinol |
| Serine/threonine-protein kinase Aurora-B | AURKB | Q96GD 4 | CHEMBL2185 | Kinase | 0 | 23 / 0Â Â Â Â Â | Cannabinol |
| Myosin light chain kinase, smooth muscle | MYLK | Q15746 | CHEMBL2428 | Kinase | 0 | 9 / 0Â Â Â Â Â | Cannabinol |
| Cannabinoid receptor 1 | CNR1 | P21554 | CHEMBL218 | Family A G protein-coupled receptor | 0.431919 | 484 / 253Â Â Â Â Â | Cannabitroil |
| Cannabinoid receptor 2 | CNR2 | P34972 | CHEMBL253 | Family A G protein-coupled receptor | 0.431919 | 424 / 224Â Â Â Â Â | Cannabitroil |
| Sulfonylurea receptor 2 | ABCC9 | O60706 | CHEMBL1971 | Primary active transporter | 0.09724 | 39 / 0Â Â Â Â Â | Cannabitroil |
| Anandamide amidohydrolase | FAAH | O00519 | CHEMBL2243 | Enzyme | 0.09724 | 71 / 0Â Â Â Â Â | Cannabitroil |
| MAP kinase p38 alpha | MAPK1 4 | Q16539 | CHEMBL260 | Kinase | 0.09724 | 454 / 0Â Â Â Â Â | Cannabitroil |
| Tyrosine-protein kinase ABL | ABL1 | P00519 | CHEMBL1862 | Kinase | 0.09724 | 149 / 0Â Â Â Â Â | Cannabitroil |
| Tyrosine-protein kinase FYN | FYN | P06241 | CHEMBL1841 | Kinase | 0.09724 | 21 / 0Â Â Â Â Â | Cannabitroil |
| Tyrosine-protein kinase YES | YES1 | P07947 | CHEMBL2073 | Kinase | 0.09724 | 19 / 0Â Â Â Â Â | Cannabitroil |
| Serine/threonine-protein kinase Aurora-B | AURKB | Q96GD 4 | CHEMBL2185 | Kinase | 0.09724 | 222 / 0Â Â Â Â Â | Cannabitroil |

| | | | | | | | |
|---|---|---|---|---|---|---|---|
| Serine/threonine-protein kinase Aurora-A | AURKA | O14965 | CHEMBL4722 | Kinase | 0.09724 | 342 / 0Â Â Â Â Â | Cannabitroil |
| Cyclin-dependent kinase 4/cyclin D1 | CCND1 CDK4 | P24385 P11802 | CHEMBL1907601 | Kinase | 0.09724 | 84 / 0Â Â Â Â Â | Cannabitroil |
| Cyclin-dependent kinase 2/cyclin E | CCNE2 CDK2 CCNE1 | O96020 P24941 P24864 | CHEMBL2094126 | Other cytosolic protein | 0.09724 | 109 / 0Â Â Â Â Â | Cannabitroil |
| Phosphodiesterase 4B | PDE4B | Q07343 | CHEMBL275 | Phosphodiesterase | 0.09724 | 107 / 0Â Â Â Â Â | Cannabitroil |
| Cyclin-dependent kinase 2 | CDK2 | P24941 | CHEMBL301 | Kinase | 0.09724 | 237 / 0Â Â Â Â Â | Cannabitroil |
| Metabotropic glutamate receptor 5 (by homology) | GRM5 | P41594 | CHEMBL3227 | Family C G protein-coupled receptor | 0.09724 | 172 / 0Â Â Â Â Â | Cannabitroil |
| PI3-kinase p110-alpha subunit | PIK3CA | P42336 | CHEMBL4005 | Enzyme | 0.09724 | 1296 / 0Â Â Â Â Â | Cannabitroil |
| LDL-associated phospholipase A2 | PLA2G7 | Q13093 | CHEMBL3514 | Enzyme | 0.09724 | 27 / 0Â Â Â Â Â | Cannabitroil |
| Adenosine deaminase | ADA | P00813 | CHEMBL1910 | Hydrolase | 0.09724 | 34 / 0Â Â Â Â Â | Cannabitroil |
| Protein kinase C gamma | PRKCG | P05129 | CHEMBL2938 | Kinase | 0.09724 | 43 / 0Â Â Â Â Â | Cannabitroil |
| Protein kinase C delta | PRKCD | Q05655 | CHEMBL2996 | Kinase | 0.09724 | 121 / 0Â Â Â Â Â | Cannabitroil |
| Protein kinase C alpha | PRKCA | P17252 | CHEMBL299 | Kinase | 0.09724 | 229 / 0Â Â Â Â Â | Cannabitroil |
| Protein kinase C beta | PRKCB | P05771 | CHEMBL3045 | Kinase | 0.09724 | 63 / 0Â Â Â Â Â | Cannabitroil |
| Protein kinase C (PKC) | PRKCZ | Q05513 | CHEMBL3438 | Kinase | 0.09724 | 4 / 0Â Â Â Â Â | Cannabitroil |
| Protein kinase C eta | PRKCH | P24723 | CHEMBL3616 | Kinase | 0.09724 | 31 / 0Â Â Â Â Â | Cannabitroil |
| Voltage-gated calcium channel alpha2/delta subunit 1 | CACNA 2D1 | P54289 | CHEMBL1919 | Calcium channel auxiliary subunit alpha2delta family | 0.09724 | 8 / 0Â Â Â Â Â | Cannabitroil |
| Voltage-gated calcium channel alpha2/delta subunit 2 | CACNA 2D2 | Q9NY4 7 | CHEMBL3896 | Calcium channel auxiliary subunit alpha2delta family | 0.09724 | 5 / 0Â Â Â Â Â | Cannabitroil |
| Glycogen synthase kinase-3 beta | GSK3B | P49841 | CHEMBL262 | Kinase | 0.09724 | 193 / 0Â Â Â Â Â | Cannabitroil |
| Glycogen synthase kinase-3 alpha | GSK3A | P49840 | CHEMBL2850 | Kinase | 0.09724 | 29 / 0Â Â Â Â Â | Cannabitroil |
| Adenosine A2a receptor | ADORA 2A | P29274 | CHEMBL251 | Family A G protein-coupled receptor | 0.09724 | 621 / 0Â Â Â Â Â | Cannabitroil |
| Adenosine A2b receptor | ADORA 2B | P29275 | CHEMBL255 | Family A G protein-coupled receptor | 0.09724 | 122 / 0Â Â Â Â Â | Cannabitroil |

| | | | | | | | |
|---|---|---|---|---|---|---|---|
| Tyrosine-protein kinase SYK | SYK | P43405 | CHEMBL2599 | Kinase | 0.09724 | 499 / 0Â Â Â Â Â | Cannabitroil |
| Tyrosine-protein kinase JAK3 | JAK3 | P52333 | CHEMBL2148 | Kinase | 0.09724 | 300 / 0Â Â Â Â Â | Cannabitroil |
| Phosphodiesterase 10A | PDE10A | Q9Y233 | CHEMBL4409 | Phosphodiesterase | 0.09724 | 1032 / 0Â Â Â Â Â | Cannabitroil |
| Tyrosine-protein kinase receptor FLT3 | FLT3 | P36888 | CHEMBL1974 | Kinase | 0.09724 | 161 / 0Â Â Â Â Â | Cannabitroil |
| Tyrosine-protein kinase LCK | LCK | P06239 | CHEMBL258 | Kinase | 0.09724 | 97 / 0Â Â Â Â Â | Cannabitroil |
| Tyrosine-protein kinase HCK | HCK | P08631 | CHEMBL3234 | Kinase | 0.09724 | 33 / 0Â Â Â Â Â | Cannabitroil |
| Tyrosine-protein kinase Lyn | LYN | P07948 | CHEMBL3905 | Kinase | 0.09724 | 23 / 0Â Â Â Â Â | Cannabitroil |
| Tyrosine-protein kinase FGR | FGR | P09769 | CHEMBL4454 | Kinase | 0.09724 | 11 / 0Â Â Â Â Â | Cannabitroil |
| Ephrin type-A receptor 3 | EPHA3 | P29320 | CHEMBL4954 | Kinase | 0.09724 | 9 / 0Â Â Â Â Â | Cannabitroil |
| Discoidin domain-containing receptor 2 | DDR2 | Q16832 | CHEMBL5122 | Kinase | 0.09724 | 8 / 0Â Â Â Â Â | Cannabitroil |
| Tyrosine-protein kinase BTK | BTK | Q06187 | CHEMBL5251 | Kinase | 0.09724 | 67 / 0Â Â Â Â Â | Cannabitroil |
| Epithelial discoidin domain-containing receptor 1 | DDR1 | Q08345 | CHEMBL5319 | Kinase | 0.09724 | 12 / 0Â Â Â Â Â | Cannabitroil |
| Cyclin-dependent kinase 5/CDK5 activator 1 | CDK5R1 CDK5 | Q15078 Q00535 | CHEMBL1907600 | Kinase | 0.09724 | 133 / 0Â Â Â Â Â | Cannabitroil |
| Casein kinase I gamma 2 | CSNK1G2 | P78368 | CHEMBL2543 | Kinase | 0.09724 | 5 / 0Â Â Â Â Â | Cannabitroil |
| Casein kinase I alpha | CSNK1A1 | P48729 | CHEMBL2793 | Kinase | 0.09724 | 15 / 0Â Â Â Â Â | Cannabitroil |
| Matrix metalloproteinase 13 | MMP13 | P45452 | CHEMBL280 | Protease | 0.09724 | 386 / 0Â Â Â Â Â | Cannabitroil |
| Casein kinase I delta | CSNK1D | P48730 | CHEMBL2828 | Kinase | 0.09724 | 15 / 0Â Â Â Â Â | Cannabitroil |
| Heat shock protein HSP 90-beta | HSP90AB1 | P08238 | CHEMBL4303 | Other cytosolic protein | 0.09724 | 62 / 0Â Â Â Â Â | Cannabitroil |
| Leucine-rich repeat serine/threonine-protein kinase 2 | LRRK2 | Q5S007 | CHEMBL1075104 | Kinase | 0.09724 | 188 / 0Â Â Â Â Â | Cannabitroil |
| G protein-coupled receptor kinase 7 | GRK7 | Q8WTQ7 | CHEMBL1075133 | Kinase | 0.09724 | 5 / 0Â Â Â Â Â | Cannabitroil |
| Serine/threonine-protein kinase 38 | STK38 | Q15208 | CHEMBL1075155 | Kinase | 0.09724 | 3 / 0Â Â Â Â Â | Cannabitroil |
| Homeodomain-interacting protein kinase 4 | HIPK4 | Q8NE63 | CHEMBL1075167 | Kinase | 0.09724 | 8 / 0Â Â Â Â Â | Cannabitroil |
| Serine/threonine-protein kinase TAO2 | TAOK2 | Q9UL54 | CHEMBL1075195 | Kinase | 0.09724 | 3 / 0Â Â Â Â Â | Cannabitroil |
| Serine/threonine-protein kinase/endoribonuclease IRE1 | ERN1 | O75460 | CHEMBL1163101 | Enzyme | 0.09724 | 14 / 0Â Â Â Â Â | Cannabitroil |
| Serine/threonine-protein kinase OSR1 | OXSR1 | O95747 | CHEMBL1163104 | Kinase | 0.09724 | 4 / 0Â Â Â Â Â | Cannabitroil |
| Serine/threonine-protein kinase MAK | MAK | P20794 | CHEMBL1163106 | Kinase | 0.09724 | 2 / 0Â Â Â Â Â | Cannabitroil |

| | | | | | | | |
|---|---|---|---|---|---|---|---|
| STE20/SPS1-related proline-alanine-rich protein kinase | STK39 | Q9UE W8 | CHEMBL1163108 | Kinase | 0.09724 | 3 / 0Â Â Â Â Â | Cannabitroil |
| Cyclin-dependent kinase-like 5 | CDKL5 | O76039 | CHEMBL1163112 | Kinase | 0.09724 | 3 / 0Â Â Â Â Â | Cannabitroil |
| Cyclin-dependent kinase-like 3 | CDKL3 | Q8IVW 4 | CHEMBL1163117 | Kinase | 0.09724 | 2 / 0Â Â Â Â Â | Cannabitroil |
| U4/U6 small nuclear ribonucleoprotein Prp4 | PRPF4 | O43172 | CHEMBL1163119 | Other nuclear protein | 0.09724 | 1 / 0Â Â Â Â Â | Cannabitroil |
| Phosphatidylinositol-4-phosphate 3-kinase C2 domain-containing subunit gamma | PIK3C2 G | O75747 | CHEMBL1163120 | Enzyme | 0.09724 | 8 / 0Â Â Â Â Â | Cannabitroil |
| Mitogen-activated protein kinase kinase kinase 6 | MAP3K 6 | O95382 | CHEMBL1163123 | Kinase | 0.09724 | 2 / 0Â Â Â Â Â | Cannabitroil |
| Mitogen-activated protein kinase kinase kinase 13 | MAP3K 13 | O43283 | CHEMBL1163124 | Kinase | 0.09724 | 3 / 0Â Â Â Â Â | Cannabitroil |
| Serine/threonine-protein kinase ICK | ICK | Q9UPZ 9 | CHEMBL1163126 | Kinase | 0.09724 | 5 / 0Â Â Â Â Â | Cannabitroil |
| Mitogen-activated protein kinase kinase kinase 15 | MAP3K 15 | Q6ZN1 6 | CHEMBL1163127 | Kinase | 0.09724 | 3 / 0Â Â Â Â Â | Cannabitroil |
| Microtubule-associated serine/threonine-protein kinase 1 | MAST1 | Q9Y2H 9 | CHEMBL1163128 | Kinase | 0.09724 | 3 / 0Â Â Â Â Â | Cannabitroil |
| Serine/threonine-protein kinase SBK1 | SBK1 | Q52WX 2 | CHEMBL1163129 | Kinase | 0.09724 | 4 / 0Â Â Â Â Â | Cannabitroil |
| Hormonally up-regulated neu tumor-associated kinase | HUNK | P57058 | CHEMBL1795165 | Enzyme | 0.09724 | 4 / 0Â Â Â Â Â | Cannabitroil |
| Cyclin-dependent kinase 13 | CDK13 | Q14004 | CHEMBL1795192 | Enzyme | 0.09724 | 4 / 0Â Â Â Â Â | Cannabitroil |
| Macrophage colony stimulating factor receptor | CSF1R | P07333 | CHEMBL1844 | Kinase | 0.09724 | 113 / 0Â Â Â Â Â | Cannabitroil |
| Vascular endothelial growth factor receptor 1 | FLT1 | P17948 | CHEMBL1868 | Kinase | 0.09724 | 53 / 0Â Â Â Â Â | Cannabitroil |
| Peripheral plasma membrane protein CASK | CASK | O14936 | CHEMBL1908381 | Enzyme | 0.09724 | 2 / 0Â Â Â Â Â | Cannabitroil |
| Serine/threonine-protein kinase PRP4 homolog | PRPF4B | Q13523 | CHEMBL1908382 | Enzyme | 0.09724 | 4 / 0Â Â Â Â Â | Cannabitroil |
| Dual serine/threonine and tyrosine protein kinase | DSTYK | Q6XUX 3 | CHEMBL1908386 | Enzyme | 0.09724 | 3 / 0Â Â Â Â Â | Cannabitroil |
| Mitogen-activated protein kinase kinase kinase 12 | MAP3K 12 | Q12852 | CHEMBL1908389 | Enzyme | 0.09724 | 95 / 0Â Â Â Â Â | Cannabitroil |
| Platelet-derived growth factor receptor beta | PDGFR B | P09619 | CHEMBL1913 | Kinase | 0.09724 | 56 / 0Â Â Â Â Â | Cannabitroil |

| | | | | | | | |
|---|---|---|---|---|---|---|---|
| Vascular endothelial growth factor receptor 3 | FLT4 | P35916 | CHEMBL1955 | Kinase | 0.09724 | 39 / 0Â Â Â Â Â | Cannabitroil |
| Insulin-like growth factor I receptor | IGF1R | P08069 | CHEMBL1957 | Kinase | 0.09724 | 226 / 0Â Â Â Â Â | Cannabitroil |
| Insulin receptor | INSR | P06213 | CHEMBL1981 | Kinase | 0.09724 | 41 / 0Â Â Â Â Â | Cannabitroil |
| Platelet-derived growth factor receptor alpha | PDGFRA | P16234 | CHEMBL2007 | Kinase | 0.09724 | 17 / 0Â Â Â Â Â | Cannabitroil |
| Epidermal growth factor receptor erbB1 | EGFR | P00533 | CHEMBL203 | Kinase | 0.09724 | 531 / 0Â Â Â Â Â | Cannabitroil |
| Kinesin-1 heavy chain/ Tyrosine-protein kinase receptor RET | RET | P07949 | CHEMBL2041 | Kinase | 0.09724 | 40 / 0Â Â Â Â Â | Cannabitroil |
| Ephrin type-A receptor 2 | EPHA2 | P29317 | CHEMBL2068 | Kinase | 0.09724 | 21 / 0Â Â Â Â Â | Cannabitroil |
| Cyclin-dependent kinase 1/cyclin B | CCNB3 CDK1 CCNB1 CCNB2 | Q8WWL7 P06493 P14635 O95067 | CHEMBL2094127 | Other cytosolic protein | 0.09724 | 47 / 0Â Â Â Â Â | Cannabitroil |
| Dual specificity mitogen-activated protein kinase kinase 3 | MAP2K3 | P46734 | CHEMBL2109 | Kinase | 0.09724 | 3 / 0Â Â Â Â Â | Cannabitroil |
| AMP-activated protein kinase, alpha-2 subunit | PRKAA2 | P54646 | CHEMBL2116 | Kinase | 0.09724 | 3 / 0Â Â Â Â Â | Cannabitroil |
| Serine/threonine-protein kinase PIM1 | PIM1 | P11309 | CHEMBL2147 | Kinase | 0.09724 | 217 / 0Â Â Â Â Â | Cannabitroil |
| Dual specificity mitogen-activated protein kinase kinase 6 | MAP2K6 | P52564 | CHEMBL2171 | Kinase | 0.09724 | 4 / 0Â Â Â Â Â | Cannabitroil |
| MAP kinase-activated protein kinase 2 | MAPKAPK2 | P49137 | CHEMBL2208 | Kinase | 0.09724 | 52 / 0Â Â Â Â Â | Cannabitroil |
| Tyrosine-protein kinase BLK | BLK | P51451 | CHEMBL2250 | Kinase | 0.09724 | 11 / 0Â Â Â Â Â | Cannabitroil |
| c-Jun N-terminal kinase 1 | MAPK8 | P45983 | CHEMBL2276 | Kinase | 0.09724 | 156 / 0Â Â Â Â Â | Cannabitroil |
| Dual-specificity tyrosine-phosphorylation regulated kinase 1A | DYRK1A | Q13627 | CHEMBL2292 | Kinase | 0.09724 | 93 / 0Â Â Â Â Â | Cannabitroil |
| Ribosomal protein S6 kinase alpha 3 | RPS6KA3 | P51812 | CHEMBL2345 | Kinase | 0.09724 | 17 / 0Â Â Â Â Â | Cannabitroil |
| Phosphorylase kinase gamma subunit 2 | PHKG2 | P15735 | CHEMBL2349 | Kinase | 0.09724 | 4 / 0Â Â Â Â Â | Cannabitroil |
| Myosin light chain kinase, smooth muscle | MYLK | Q15746 | CHEMBL2428 | Kinase | 0.09724 | 14 / 0Â Â Â Â Â | Cannabitroil |
| Serine/threonine-protein kinase AKT2 | AKT2 | P31751 | CHEMBL2431 | Kinase | 0.09724 | 11 / 0Â Â Â Â Â | Cannabitroil |
| Death-associated protein kinase 3 | DAPK3 | O43293 | CHEMBL2468 | Kinase | 0.09724 | 8 / 0Â Â Â Â Â | Cannabitroil |

| | | | | | | | |
|---|---|---|---|---|---|---|---|
| CaM kinase I alpha | CAMK1 | Q14012 | CHEMBL2493 | Kinase | 0.09724 | 6 / 0Â Â Â Â Â | Cannabitroil |
| CaM kinase IV | CAMK4 | Q16566 | CHEMBL2494 | Kinase | 0.09724 | 3 / 0Â Â Â Â Â | Cannabitroil |
| Serine/threonine-protein kinase Chk2 | CHEK2 | O96017 | CHEMBL2527 | Kinase | 0.09724 | 16 / 0Â Â Â Â Â | Cannabitroil |
| Cannabinoid receptor 1 | CNR1 | P21554 | CHEMBL218 | Family A G protein-coupled receptor | 0.65802 | 588 / 50Â Â Â Â Â | Cannabivarin |
| Cannabinoid receptor 2 | CNR2 | P34972 | CHEMBL253 | Family A G protein-coupled receptor | 0.592071 | 493 / 55Â Â Â Â Â | Cannabivarin |
| Estrogen receptor beta | ESR2 | Q92731 | CHEMBL242 | Nuclear receptor | 0.1061 | 12 / 20Â Â Â Â Â | Cannabivarin |
| Estrogen receptor alpha | ESR1 | P03372 | CHEMBL206 | Nuclear receptor | 0.1061 | 14 / 22Â Â Â Â Â | Cannabivarin |
| Vascular endothelial growth factor receptor 2 | KDR | P35968 | CHEMBL279 | Kinase | 0.097875 | 203 / 0Â Â Â Â Â | Cannabivarin |
| N-arachidonyl glycine receptor | GPR18 | Q14330 | CHEMBL2384898 | Family A G protein-coupled receptor | 0.097875 | 2 / 0Â Â Â Â Â | Cannabivarin |
| Glycine receptor subunit alpha-1 | GLRA1 | P23415 | CHEMBL5845 | Ligand-gated ion channel | 0.097875 | 1 / 0Â Â Â Â Â | Cannabivarin |
| Glucagon receptor | GCGR | P47871 | CHEMBL1985 | Family B G protein-coupled receptor | 0.097875 | 22 / 0Â Â Â Â Â | Cannabivarin |
| Phosphodiesterase 4B | PDE4B | Q07343 | CHEMBL275 | Phosphodiesterase | 0.097875 | 32 / 0Â Â Â Â Â | Cannabivarin |
| Receptor protein-tyrosine kinase erbB-2 | ERBB2 | P04626 | CHEMBL1824 | Kinase | 0.097875 | 49 / 0Â Â Â Â Â | Cannabivarin |
| Epidermal growth factor receptor erbB1 | EGFR | P00533 | CHEMBL203 | Kinase | 0.097875 | 269 / 0Â Â Â Â Â | Cannabivarin |
| Cystic fibrosis transmembrane conductance regulator | CFTR | P13569 | CHEMBL4051 | Other ion channel | 0.097875 | 2 / 0Â Â Â Â Â | Cannabivarin |
| 11-beta-hydroxysteroid dehydrogenase 1 | HSD11B1 | P28845 | CHEMBL4235 | Enzyme | 0.097875 | 199 / 0Â Â Â Â Â | Cannabivarin |
| Interleukin-8 receptor B | CXCR2 | P25025 | CHEMBL2434 | Family A G protein-coupled receptor | 0.097875 | 39 / 0Â Â Â Â Â | Cannabivarin |
| Serine/threonine-protein kinase PAK 1 | PAK1 | Q13153 | CHEMBL4600 | Kinase | 0.097875 | 1 / 0Â Â Â Â Â | Cannabivarin |
| Histone deacetylase 6 | HDAC6 | Q9UBN7 | CHEMBL1865 | Eraser | 0.097875 | 21 / 0Â Â Â Â Â | Cannabivarin |
| Histone deacetylase 8 | HDAC8 | Q9BY41 | CHEMBL3192 | Eraser | 0.097875 | 10 / 0Â Â Â Â Â | Cannabivarin |
| Estradiol 17-beta-dehydrogenase 3 | HSD17B3 | P37058 | CHEMBL4234 | Enzyme | 0.097875 | 6 / 0Â Â Â Â Â | Cannabivarin |

| | | | | | | | |
|---|---|---|---|---|---|---|---|
| Epoxide hydratase | EPHX2 | P34913 | CHEMBL2409 | Protease | 0.097875 | 64 / 0Â Â Â Â Â | Cannabivarin |
| Melanocortin receptor 4 | MC4R | P32245 | CHEMBL259 | Family A G protein-coupled receptor | 0.097875 | 9 / 0Â Â Â Â Â | Cannabivarin |
| G-protein coupled receptor 55 | GPR55 | Q9Y2T6 | CHEMBL1075322 | Family A G protein-coupled receptor | 0.097875 | 10 / 0Â Â Â Â Â | Cannabivarin |
| Cathepsin D | CTSD | P07339 | CHEMBL2581 | Protease | 0.097875 | 25 / 0Â Â Â Â Â | Cannabivarin |
| Poly [ADP-ribose] polymerase-1 | PARP1 | P09874 | CHEMBL3105 | Enzyme | 0.097875 | 16 / 0Â Â Â Â Â | Cannabivarin |
| Calcium-activated potassium channel subunit alpha-1 | KCNMA1 | Q12791 | CHEMBL4304 | Voltage-gated ion channel | 0.097875 | 11 / 0Â Â Â Â Â | Cannabivarin |
| NAD-dependent deacetylase sirtuin 2 | SIRT2 | Q8IXJ6 | CHEMBL4462 | Eraser | 0.097875 | 19 / 0Â Â Â Â Â | Cannabivarin |
| Prostanoid EP1 receptor | PTGER1 | P34995 | CHEMBL1811 | Family A G protein-coupled receptor | 0.097875 | 22 / 16Â Â Â Â Â | Cannabivarin |
| Cyclin-dependent kinase 5/CDK5 activator 1 | CDK5R1 CDK5 | Q15078 Q00535 | CHEMBL1907600 | Kinase | 0.097875 | 73 / 0Â Â Â Â Â | Cannabivarin |
| Cyclin-dependent kinase 1/cyclin B | CCNB3 CDK1 CCNB1 CCNB2 | Q8WWL7 P06493 P14635 O95067 | CHEMBL2094127 | Other cytosolic protein | 0.097875 | 42 / 0Â Â Â Â Â | Cannabivarin |
| Prenyl protein specific protease | RCE1 | Q9Y256 | CHEMBL3411 | Protease | 0.097875 | 6 / 0Â Â Â Â Â | Cannabivarin |
| Sodium channel protein type II alpha subunit | SCN2A | Q99250 | CHEMBL4187 | Voltage-gated ion channel | 0.097875 | 24 / 0Â Â Â Â Â | Cannabivarin |
| Glucocorticoid receptor | NR3C1 | P04150 | CHEMBL2034 | Nuclear receptor | 0.097875 | 100 / 54Â Â Â Â Â | Cannabivarin |
| Serine/threonine-protein kinase Aurora-B | AURKB | Q96GD4 | CHEMBL2185 | Kinase | 0.097875 | 21 / 0Â Â Â Â Â | Cannabivarin |
| Serine/threonine-protein kinase Aurora-C | AURKC | Q9UQB9 | CHEMBL3935 | Kinase | 0.097875 | 11 / 0Â Â Â Â Â | Cannabivarin |
| Serine/threonine-protein kinase Aurora-A | AURKA | O14965 | CHEMBL4722 | Kinase | 0.097875 | 26 / 0Â Â Â Â Â | Cannabivarin |
| Translocator protein (by homology) | TSPO | P30536 | CHEMBL5742 | Membrane receptor | 0.097875 | 308 / 0Â Â Â Â Â | Cannabivarin |
| ATP-binding cassette sub-family G member 2 | ABCG2 | Q9UNQ0 | CHEMBL5393 | Primary active transporter | 0.097875 | 15 / 0Â Â Â Â Â | Cannabivarin |
| LDL-associated phospholipase A2 | PLA2G7 | Q13093 | CHEMBL3514 | Enzyme | 0.097875 | 14 / 0Â Â Â Â Â | Cannabivarin |
| Protein kinase C (PKC) | PRKCZ | Q05513 | CHEMBL3438 | Kinase | 0.097875 | 2 / 0Â Â Â Â Â | Cannabivarin |
| Nitric oxide synthase, inducible (by homology) | NOS2 | P35228 | CHEMBL4481 | Enzyme | 0.097875 | 78 / 0Â Â Â Â Â | Cannabivarin |

| | | | | | | | |
|---|---|---|---|---|---|---|---|
| Corticotropin releasing factor receptor 1 | CRHR1 | P34998 | CHEMBL1800 | Family B G protein-coupled receptor | 0.097875 | 68 / 0Â Â Â Â Â | Cannabivarin |
| Thymidylate synthase | TYMS | P04818 | CHEMBL1952 | Transferase | 0.097875 | 24 / 0Â Â Â Â Â | Cannabivarin |
| Quinone reductase 2 | NQO2 | P16083 | CHEMBL3959 | Enzyme | 0.097875 | 8 / 0Â Â Â Â Â | Cannabivarin |
| 5-lipoxygenase activating protein | ALOX5AP | P20292 | CHEMBL4550 | Other cytosolic protein | 0.097875 | 37 / 0Â Â Â Â Â | Cannabivarin |
| Gonadotropin-releasing hormone receptor | GNRHR | P30968 | CHEMBL1855 | Family A G protein-coupled receptor | 0.097875 | 7 / 0Â Â Â Â Â | Cannabivarin |
| Estradiol 17-beta-dehydrogenase 2 | HSD17B2 | P37059 | CHEMBL2789 | Enzyme | 0.097875 | 19 / 0Â Â Â Â Â | Cannabivarin |
| Bombesin receptor subtype-3 | BRS3 | P32247 | CHEMBL4080 | Family A G protein-coupled receptor | 0.097875 | 1 / 0Â Â Â Â Â | Cannabivarin |
| Anandamide amidohydrolase | FAAH | O00519 | CHEMBL2243 | Enzyme | 0 | 38 / 0Â Â Â Â Â | Cannabivarin |
| Serotonin 2c (5-HT2c) receptor | HTR2C | P28335 | CHEMBL225 | Family A G protein-coupled receptor | 0 | 38 / 0Â Â Â Â Â | Cannabivarin |
| Protoporphyrinogen oxidase | PPOX | P50336 | CHEMBL1926488 | Oxidoreductase | 0 | 1 / 0Â Â Â Â Â | Cannabivarin |
| Voltage-gated potassium channel subunit Kv1.5 | KCNA5 | P22460 | CHEMBL4306 | Voltage-gated ion channel | 0 | 84 / 0Â Â Â Â Â | Cannabivarin |
| Acetylcholinesterase | ACHE | P22303 | CHEMBL220 | Hydrolase | 0 | 66 / 0Â Â Â Â Â | Cannabivarin |
| Adenosine A1 receptor (by homology) | ADORA1 | P30542 | CHEMBL226 | Family A G protein-coupled receptor | 0 | 232 / 0Â Â Â Â Â | Cannabivarin |
| Beta secretase 2 | BACE2 | Q9Y5Z0 | CHEMBL2525 | Protease | 0 | 43 / 0Â Â Â Â Â | Cannabivarin |
| Tyrosine-protein kinase JAK3 | JAK3 | P52333 | CHEMBL2148 | Kinase | 0 | 196 / 0Â Â Â Â Â | Cannabivarin |
| Tyrosine-protein kinase JAK2 | JAK2 | O60674 | CHEMBL2971 | Kinase | 0 | 172 / 0Â Â Â Â Â | Cannabivarin |
| Tyrosine-protein kinase receptor FLT3 | FLT3 | P36888 | CHEMBL1974 | Kinase | 0 | 35 / 0Â Â Â Â Â | Cannabivarin |
| Histone deacetylase 2 | HDAC2 | Q92769 | CHEMBL1937 | Eraser | 0 | 11 / 0Â Â Â Â Â | Cannabivarin |
| Kinesin-like protein 1 | KIF11 | P52732 | CHEMBL4581 | Other cytosolic protein | 0 | 33 / 0Â Â Â Â Â | Cannabivarin |
| G-protein coupled bile acid receptor 1 | GPBAR1 | Q8TDU6 | CHEMBL5409 | Family A G protein-coupled receptor | 0 | 20 / 0Â Â Â Â Â | Cannabivarin |
| Adenosine A3 receptor | ADORA3 | P0DMS8 | CHEMBL256 | Family A G protein-coupled receptor | 0 | 156 / 0Â Â Â Â Â | Cannabivarin |

| | | | | | | | |
|---|---|---|---|---|---|---|---|
| Isocitrate dehydrogenase [NADP] cytoplasmic | IDH1 | O75874 | CHEMBL2007625 | Enzyme | 0 | 159 / 0Â Â Â Â Â | Cannabivarin |
| Muscarinic acetylcholine receptor M1 (by homology) | CHRM1 | P11229 | CHEMBL216 | Family A G protein-coupled receptor | 0 | 28 / 0Â Â Â Â Â | Cannabivarin |
| Lysosomal Pro-X carboxypeptidase | PRCP | P42785 | CHEMBL2335 | Protease | 0 | 10 / 0Â Â Â Â Â | Cannabivarin |
| Peregrin | BRPF1 | P55201 | CHEMBL3132741 | Reader | 0 | 24 / 0Â Â Â Â Â | Cannabivarin |
| Matrix metalloproteinase 9 | MMP9 | P14780 | CHEMBL321 | Protease | 0 | 14 / 0Â Â Â Â Â | Cannabivarin |
| Cathepsin (V and K) | CTSV | O60911 | CHEMBL3272 | Protease | 0 | 11 / 0Â Â Â Â Â | Cannabivarin |
| Matrix metalloproteinase 2 | MMP2 | P08253 | CHEMBL333 | Protease | 0 | 19 / 0Â Â Â Â Â | Cannabivarin |
| Amine oxidase, copper containing | AOC3 | Q16853 | CHEMBL3437 | Enzyme | 0 | 4 / 0Â Â Â Â Â | Cannabivarin |
| Egl nine homolog 1 | EGLN1 | Q9GZT9 | CHEMBL5697 | Oxidoreductase | 0 | 7 / 0Â Â Â Â Â | Cannabivarin |
| Serine/threonine-protein kinase 33 | STK33 | Q9BYT3 | CHEMBL6005 | Kinase | 0 | 14 / 0Â Â Â Â Â | Cannabivarin |
| Cytochrome b-c1 complex subunit 7 | UQCRB | P14927 | CHEMBL1671612 | Transmembrane 1-electron transfer carriers | 0 | 1 / 0Â Â Â Â Â | Cannabivarin |
| Adenosine A2a receptor | ADORA2A | P29274 | CHEMBL251 | Family A G protein-coupled receptor | 0 | 132 / 0Â Â Â Â Â | Cannabivarin |
| Matrix metalloproteinase 13 | MMP13 | P45452 | CHEMBL280 | Protease | 0 | 7 / 0Â Â Â Â Â | Cannabivarin |
| Estradiol 17-beta-dehydrogenase 1 | HSD17B1 | P14061 | CHEMBL3181 | Enzyme | 0 | 10 / 0Â Â Â Â Â | Cannabivarin |
| Hormone sensitive lipase | LIPE | Q05469 | CHEMBL3590 | Enzyme | 0 | 51 / 0Â Â Â Â Â | Cannabivarin |
| Quinone reductase 1 | NQO1 | P15559 | CHEMBL3623 | Enzyme | 0 | 4 / 0Â Â Â Â Â | Cannabivarin |
| Methyl-CpG-binding protein 2 | MECP2 | P51608 | CHEMBL3638346 | Reader | 0 | 1 / 0Â Â Â Â Â | Cannabivarin |
| Methyl-CpG-binding domain protein 2 | MBD2 | Q9UBB5 | CHEMBL3707462 | Reader | 0 | 2 / 0Â Â Â Â Â | Cannabivarin |
| Vanilloid receptor | TRPV1 | Q8NER1 | CHEMBL4794 | Voltage-gated ion channel | 0 | 72 / 0Â Â Â Â Â | Cannabivarin |
| Steryl-sulfatase | STS | P08842 | CHEMBL3559 | Enzyme | 0 | 7 / 0Â Â Â Â Â | Cannabivarin |
| Dual specificity phosphatase Cdc25B | CDC25B | P30305 | CHEMBL4804 | Phosphatase | 0 | 14 / 0Â Â Â Â Â | Cannabivarin |
| Dual-specificity tyrosine-phosphorylation regulated kinase 1A (by homology) | DYRK1A | Q13627 | CHEMBL2292 | Kinase | 0 | 39 / 0Â Â Â Â Â | Cannabivarin |
| Serine/threonine-protein kinase PIM1 | PIM1 | P11309 | CHEMBL2147 | Kinase | 0 | 75 / 0Â Â Â Â Â | Cannabivarin |

| | | | | | | | |
|---|---|---|---|---|---|---|---|
| Serine/threonine-protein kinase mTOR | MTOR | P42345 | CHEMBL2842 | Kinase | 0 | 28 / 0Â Â Â Â Â | Cannabivarin |
| PI3-kinase p110-alpha subunit | PIK3CA | P42336 | CHEMBL4005 | Enzyme | 0 | 59 / 0Â Â Â Â Â | Cannabivarin |
| Serine/threonine-protein kinase PIM2 | PIM2 | Q9P1W9 | CHEMBL4523 | Kinase | 0 | 63 / 0Â Â Â Â Â | Cannabivarin |
| Bcl2-antagonist of cell death (BAD) | BAD | Q92934 | CHEMBL3817 | Other cytosolic protein | 0 | 10 / 0Â Â Â Â Â | Cannabivarin |
| Serine/threonine-protein kinase PIM3 | PIM3 | Q86V86 | CHEMBL5407 | Kinase | 0 | 10 / 0Â Â Â Â Â | Cannabivarin |
| ADAMTS5 | ADAMTS5 | Q9UNA0 | CHEMBL2285 | Protease | 0 | 16 / 0Â Â Â Â Â | Cannabivarin |
| Glycogen synthase kinase-3 beta | GSK3B | P49841 | CHEMBL262 | Kinase | 0 | 50 / 0Â Â Â Â Â | Cannabivarin |
| Phosphodiesterase 2A | PDE2A | O00408 | CHEMBL2652 | Phosphodiesterase | 0 | 52 / 0Â Â Â Â Â | Cannabivarin |
| Phosphodiesterase 10A | PDE10A | Q9Y233 | CHEMBL4409 | Phosphodiesterase | 0 | 314 / 0Â Â Â Â Â | Cannabivarin |
| TGF-beta receptor type II | TGFBR2 | P37173 | CHEMBL4267 | Kinase | 0 | 5 / 0Â Â Â Â Â | Cannabivarin |
| TGF-beta receptor type I | TGFBR1 | P36897 | CHEMBL4439 | Kinase | 0 | 39 / 0Â Â Â Â Â | Cannabivarin |
| Serine/threonine-protein kinase RIPK2 | RIPK2 | O43353 | CHEMBL5014 | Kinase | 0 | 4 / 0Â Â Â Â Â | Cannabivarin |
| Phosphodiesterase 7A | PDE7A | Q13946 | CHEMBL3012 | Phosphodiesterase | 0 | 15 / 0Â Â Â Â Â | Cannabivarin |
| Cholecystokinin B receptor | CCKBR | P32239 | CHEMBL298 | Family A G protein-coupled receptor | 0 | 180 / 0Â Â Â Â Â | Cannabivarin |
| p53-binding protein Mdm-2 | MDM2 | Q00987 | CHEMBL5023 | Other nuclear protein | 0 | 38 / 0Â Â Â Â Â | Cannabivarin |
| Leucine-rich repeat serine/threonine-protein kinase 2 | LRRK2 | Q5S007 | CHEMBL1075104 | Kinase | 0 | 27 / 0Â Â Â Â Â | Cannabivarin |
| Bile acid receptor FXR | NR1H4 | Q96RI1 | CHEMBL2047 | Nuclear receptor | 0 | 26 / 0Â Â Â Â Â | Cannabivarin |
| | | | | | | | |
| Cannabinoid receptor 1 | CNR1 | P21554 | CHEMBL218 | Family A G protein-coupled receptor | 0.959682 | 818 / 372Â Â Â Â Â | Δ8-Tetrahydrocannabinol |
| Cannabinoid receptor 2 | CNR2 | P34972 | CHEMBL253 | Family A G protein-coupled receptor | 0.959682 | 715 / 360Â Â Â Â Â | Δ8-Tetrahydrocannabinol |
| Vascular endothelial growth factor receptor 2 | KDR | P35968 | CHEMBL279 | Kinase | 0.864805 | 163 / 15Â Â Â Â Â | Δ8-Tetrahydrocannabinol |
| N-arachidonyl glycine receptor | GPR18 | Q14330 | CHEMBL2384898 | Family A G protein-coupled receptor | 0.434822 | 2 / 2Â Â Â Â Â | Δ8-Tetrahydrocannabinol |
| Glycine receptor subunit alpha-1 | GLRA1 | P23415 | CHEMBL5845 | Ligand-gated ion channel | 0.434822 | 1 / 1Â Â Â Â Â | Δ8-Tetrahydrocannabinol |

| | | | | | | | |
|---|---|---|---|---|---|---|---|
| Glucagon receptor | GCGR | P47871 | CHEMBL1985 | Family B G protein-coupled receptor | 0.101614 | 33 / 0Â Â Â Â Â | Δ8-Tetrahydrocannabinol |
| Cystic fibrosis transmembrane conductance regulator | CFTR | P13569 | CHEMBL4051 | Other ion channel | 0.101614 | 3 / 0Â Â Â Â Â | Δ8-Tetrahydrocannabinol |
| 5-lipoxygenase activating protein | ALOX5AP | P20292 | CHEMBL4550 | Other cytosolic protein | 0.101614 | 55 / 0Â Â Â Â Â | Δ8-Tetrahydrocannabinol |
| Cathepsin D | CTSD | P07339 | CHEMBL2581 | Protease | 0.101614 | 15 / 0Â Â Â Â Â | Δ8-Tetrahydrocannabinol |
| Calcium sensing receptor | CASR | P41180 | CHEMBL1878 | Family C G protein-coupled receptor | 0.101614 | 33 / 0Â Â Â Â Â | Δ8-Tetrahydrocannabinol |
| PH domain leucine-rich repeat-containing protein phosphatase 1 | PHLPP1 | O60346 | CHEMBL3414405 | Reader | 0.101614 | 0 / 2Â Â Â Â Â | Δ8-Tetrahydrocannabinol |
| Serine/threonine-protein kinase ILK-1 | ILK | Q13418 | CHEMBL5247 | Kinase | 0.101614 | 0 / 2Â Â Â Â Â | Δ8-Tetrahydrocannabinol |
| Epoxide hydratase | EPHX2 | P34913 | CHEMBL2409 | Protease | 0.101614 | 153 / 0Â Â Â Â Â | Δ8-Tetrahydrocannabinol |
| Protein kinase C (PKC) | PRKCZ | Q05513 | CHEMBL3438 | Kinase | 0.101614 | 2 / 0Â Â Â Â Â | Δ8-Tetrahydrocannabinol |
| Nitric oxide synthase, inducible (by homology) | NOS2 | P35228 | CHEMBL4481 | Enzyme | 0.101614 | 49 / 0Â Â Â Â Â | Δ8-Tetrahydrocannabinol |
| Thromboxane A2 receptor | TBXA2R | P21731 | CHEMBL2069 | Family A G protein-coupled receptor | 0.101614 | 7 / 0Â Â Â Â Â | Δ8-Tetrahydrocannabinol |
| Prenyl protein specific protease | RCE1 | Q9Y256 | CHEMBL3411 | Protease | 0.101614 | 6 / 0Â Â Â Â Â | Δ8-Tetrahydrocannabinol |
| Quinone reductase 2 | NQO2 | P16083 | CHEMBL3959 | Enzyme | 0.101614 | 9 / 0Â Â Â Â Â | Δ8-Tetrahydrocannabinol |
| Corticotropin releasing factor receptor 1 | CRHR1 | P34998 | CHEMBL1800 | Family B G protein-coupled receptor | 0.101614 | 150 / 0Â Â Â Â Â | Δ8-Tetrahydrocannabinol |
| Melanocortin receptor 4 | MC4R | P32245 | CHEMBL259 | Family A G protein-coupled receptor | 0.101614 | 22 / 0Â Â Â Â Â | Δ8-Tetrahydrocannabinol |
| Poly [ADP-ribose] polymerase-1 | PARP1 | P09874 | CHEMBL3105 | Enzyme | 0.101614 | 15 / 0Â Â Â Â Â | Δ8-Tetrahydrocannabinol |
| Neurokinin 2 receptor | TACR2 | P21452 | CHEMBL2327 | Family A G protein-coupled receptor | 0.101614 | 10 / 0Â Â Â Â Â | Δ8-Tetrahydrocannabinol |

| | | | | | | | |
|---|---|---|---|---|---|---|---|
| Estradiol 17-beta-dehydrogenase 3 | HSD17B3 | P37058 | CHEMBL4234 | Enzyme | 0.101614 | 6 / 0Â Â Â Â Â | Δ8-Tetrahydrocannabinol |
| Voltage-gated potassium channel subunit Kv1.5 | KCNA5 | P22460 | CHEMBL4306 | Voltage-gated ion channel | 0.101614 | 106 / 0Â Â Â Â Â | Δ8-Tetrahydrocannabinol |
| Neurokinin 3 receptor | TACR3 | P29371 | CHEMBL4429 | Family A G protein-coupled receptor | 0.101614 | 107 / 0Â Â Â Â Â | Δ8-Tetrahydrocannabinol |
| Serine/threonine-protein kinase AKT | AKT1 | P31749 | CHEMBL4282 | Kinase | 0.101614 | 14 / 18Â Â Â Â Â | Δ8-Tetrahydrocannabinol |
| Translocator protein (by homology) | TSPO | P30536 | CHEMBL5742 | Membrane receptor | 0.101614 | 370 / 0Â Â Â Â Â | Δ8-Tetrahydrocannabinol |
| Prostanoid EP1 receptor | PTGER1 | P34995 | CHEMBL1811 | Family A G protein-coupled receptor | 0.101614 | 36 / 0Â Â Â Â Â | Δ8-Tetrahydrocannabinol |
| G-protein coupled receptor 55 | GPR55 | Q9Y2T6 | CHEMBL1075322 | Family A G protein-coupled receptor | 0.101614 | 14 / 2Â Â Â Â Â | Δ8-Tetrahydrocannabinol |
| Androgen Receptor | AR | P10275 | CHEMBL1871 | Nuclear receptor | 0.101614 | 61 / 30Â Â Â Â Â | Δ8-Tetrahydrocannabinol |
| Histone deacetylase 2 | HDAC2 | Q92769 | CHEMBL1937 | Eraser | 0.101614 | 8 / 0Â Â Â Â Â | Δ8-Tetrahydrocannabinol |
| Protoporphyrinogen oxidase | PPOX | P50336 | CHEMBL1926488 | Oxidoreductase | 0 | 1 / 0Â Â Â Â Â | Δ8-Tetrahydrocannabinol |
| Kinesin-like protein 1 | KIF11 | P52732 | CHEMBL4581 | Other cytosolic protein | 0 | 33 / 0Â Â Â Â Â | Δ8-Tetrahydrocannabinol |
| P2X purinoceptor 3 | P2RX3 | P56373 | CHEMBL2998 | Ligand-gated ion channel | 0 | 38 / 0Â Â Â Â Â | Δ8-Tetrahydrocannabinol |
| Peregrin | BRPF1 | P55201 | CHEMBL3132741 | Reader | 0 | 19 / 0Â Â Â Â Â | Δ8-Tetrahydrocannabinol |
| Sodium channel protein type II alpha subunit | SCN2A | Q99250 | CHEMBL4187 | Voltage-gated ion channel | 0 | 31 / 0Â Â Â Â Â | Δ8-Tetrahydrocannabinol |
| Type-1 angiotensin II receptor (by homology) | AGTR1 | P30556 | CHEMBL227 | Family A G protein-coupled receptor | 0 | 3 / 0Â Â Â Â Â | Δ8-Tetrahydrocannabinol |
| Calcium-activated potassium channel subunit alpha-1 | KCNMA1 | Q12791 | CHEMBL4304 | Voltage-gated ion channel | 0 | 10 / 0Â Â Â Â Â | Δ8-Tetrahydrocannabinol |
| Orexin receptor 2 | HCRTR2 | O43614 | CHEMBL4792 | Family A G protein-coupled receptor | 0 | 240 / 0Â Â Â Â Â | Δ8-Tetrahydrocannabinol |
| Orexin receptor 1 | HCRTR1 | O43613 | CHEMBL5113 | Family A G protein- | 0 | 208 / 0Â Â Â Â Â | Δ8-Tetrahydrocannabinol |

| | | | | | | | |
|---|---|---|---|---|---|---|---|
| | | | | coupled receptor | | | |
| Estradiol 17-beta-dehydrogenase 2 | HSD17B2 | P37059 | CHEMBL2789 | Enzyme | 0 | 19 / 0Â Â Â Â Â | Δ8-Tetrahydrocannabinol |
| Hormone sensitive lipase | LIPE | Q05469 | CHEMBL3590 | Enzyme | 0 | 56 / 0Â Â Â Â Â | Δ8-Tetrahydrocannabinol |
| Beta secretase 2 | BACE2 | Q9Y5Z0 | CHEMBL2525 | Protease | 0 | 25 / 0Â Â Â Â Â | Δ8-Tetrahydrocannabinol |
| LDL-associated phospholipase A2 | PLA2G7 | Q13093 | CHEMBL3514 | Enzyme | 0 | 17 / 0Â Â Â Â Â | Δ8-Tetrahydrocannabinol |
| Cholecystokinin B receptor (by homology) | CCKBR | P32239 | CHEMBL298 | Family A G protein-coupled receptor | 0 | 209 / 0Â Â Â Â Â | Δ8-Tetrahydrocannabinol |
| Phosphodiesterase 10A | PDE10A | Q9Y233 | CHEMBL4409 | Phosphodiesterase | 0 | 301 / 0Â Â Â Â Â | Δ8-Tetrahydrocannabinol |
| Tyrosine-protein kinase ABL | ABL1 | P00519 | CHEMBL1862 | Kinase | 0 | 84 / 0Â Â Â Â Â | Δ8-Tetrahydrocannabinol |
| Thymidylate synthase | TYMS | P04818 | CHEMBL1952 | Transferase | 0 | 13 / 0Â Â Â Â Â | Δ8-Tetrahydrocannabinol |
| Signal transducer and activator of transcription 3 | STAT3 | P40763 | CHEMBL4026 | Transcription factor | 0 | 18 / 0Â Â Â Â Â | Δ8-Tetrahydrocannabinol |
| Anandamide amidohydrolase | FAAH | O00519 | CHEMBL2243 | Enzyme | 0 | 78 / 0Â Â Â Â Â | Δ8-Tetrahydrocannabinol |
| Estrogen receptor beta | ESR2 | Q92731 | CHEMBL242 | Nuclear receptor | 0 | 19 / 41Â Â Â Â Â | Δ8-Tetrahydrocannabinol |
| Serine/threonine-protein kinase PAK 1 | PAK1 | Q13153 | CHEMBL4600 | Kinase | 0 | 1 / 0Â Â Â Â Â | Δ8-Tetrahydrocannabinol |
| ADAMTS5 | ADAMTS5 | Q9UNA0 | CHEMBL2285 | Protease | 0 | 16 / 0Â Â Â Â Â | Δ8-Tetrahydrocannabinol |
| Bombesin receptor subtype-3 | BRS3 | P32247 | CHEMBL4080 | Family A G protein-coupled receptor | 0 | 1 / 0Â Â Â Â Â | Δ8-Tetrahydrocannabinol |
| Integrin alpha2/beta1 | ITGB1 ITGA2 | P05556 P17301 | CHEMBL3137268 | Unclassified protein | 0 | 1 / 0Â Â Â Â Â | Δ8-Tetrahydrocannabinol |
| Voltage-gated potassium channel, KQT; KCNQ2(Kv7.2)/KCNQ3(Kv7.3) | KCNQ3 KCNQ2 | O43525 O43526 | CHEMBL2221348 | Voltage-gated ion channel | 0 | 5 / 0Â Â Â Â Â | Δ8-Tetrahydrocannabinol |
| Cathepsin S | CTSS | P25774 | CHEMBL2954 | Protease | 0 | 104 / 0Â Â Â Â Â | Δ8-Tetrahydrocannabinol |

| | | | | | | | |
|---|---|---|---|---|---|---|---|
| L-lactate dehydrogenase A chain | LDHA | P00338 | CHEMBL4835 | Enzyme | 0 | 7 / 0Â Â Â Â Â | Δ8-Tetrahydrocannabinol |
| Isocitrate dehydrogenase [NADP] cytoplasmic | IDH1 | O75874 | CHEMBL2007625 | Enzyme | 0 | 189 / 0Â Â Â Â Â | Δ8-Tetrahydrocannabinol |
| GABA-A receptor; alpha-3/beta-3/gamma-2 | GABRB3 GABRA3 GABRG2 | P28472 P34903 P18507 | CHEMBL2094120 | Ligand-gated ion channel | 0 | 70 / 0Â Â Â Â Â | Δ8-Tetrahydrocannabinol |
| GABA-A receptor; alpha-1/beta-3/gamma-2 | GABRB3 GABRG2 GABRA1 | P28472 P18507 P14867 | CHEMBL2094121 | Ligand-gated ion channel | 0 | 56 / 0Â Â Â Â Â | Δ8-Tetrahydrocannabinol |
| GABA-A receptor; alpha-5/beta-3/gamma-2 | GABRB3 GABRG2 GABRA5 | P28472 P18507 P31644 | CHEMBL2094122 | Ligand-gated ion channel | 0 | 62 / 0Â Â Â Â Â | Δ8-Tetrahydrocannabinol |
| GABA-A receptor; alpha-2/beta-3/gamma-2 | GABRA2 GABRB3 GABRG2 | P47869 P28472 P18507 | CHEMBL2094130 | Ligand-gated ion channel | 0 | 67 / 0Â Â Â Â Â | Δ8-Tetrahydrocannabinol |
| Monoglyceride lipase | MGLL | Q99685 | CHEMBL4191 | Enzyme | 0 | 15 / 0Â Â Â Â Â | Δ8-Tetrahydrocannabinol |
| Sodium channel protein type V alpha subunit | SCN5A | Q14524 | CHEMBL1980 | Voltage-gated ion channel | 0 | 19 / 0Â Â Â Â Â | Δ8-Tetrahydrocannabinol |
| Egl nine homolog 1 | EGLN1 | Q9GZT9 | CHEMBL5697 | Oxidoreductase | 0 | 7 / 0Â Â Â Â Â | Δ8-Tetrahydrocannabinol |
| Prostanoid IP receptor | PTGIR | P43119 | CHEMBL1995 | Family A G protein-coupled receptor | 0 | 0 / 10Â Â Â Â Â | Δ8-Tetrahydrocannabinol |
| Leucine-rich repeat serine/threonine-protein kinase 2 | LRRK2 | Q5S007 | CHEMBL1075104 | Kinase | 0 | 19 / 0Â Â Â Â Â | Δ8-Tetrahydrocannabinol |
| G-protein coupled bile acid receptor 1 | GPBAR1 | Q8TDU6 | CHEMBL5409 | Family A G protein-coupled receptor | 0 | 23 / 0Â Â Â Â Â | Δ8-Tetrahydrocannabinol |
| Troponin, cardiac muscle | TNNC1 TNNT2 TNNI3 | P63316 P45379 P19429 | CHEMBL2095202 | Unclassified protein | 0 | 3 / 0Â Â Â Â Â | Δ8-Tetrahydrocannabinol |
| Lysosomal Pro-X carboxypeptidase | PRCP | P42785 | CHEMBL2335 | Protease | 0 | 10 / 0Â Â Â Â Â | Δ8-Tetrahydrocannabinol |
| Serine/threonine-protein | ERN1 | O75460 | CHEMBL1163101 | Enzyme | 0 | 2 / 0Â Â Â Â Â | Δ8-Tetrahydrocannabinol |

| | | | | | | | |
|---|---|---|---|---|---|---|---|
| kinase/endoribonuclease IRE1 | | | | | | | |
| Cyclin-dependent kinase 5/CDK5 activator 1 | CDK5R1 CDK5 | Q15078 Q00535 | CHEMBL1907600 | Kinase | 0 | 64 / 0Â Â Â Â Â | Δ8-Tetrahydrocannabinol |
| Tyrosine-protein kinase JAK3 | JAK3 | P52333 | CHEMBL2148 | Kinase | 0 | 143 / 0Â Â Â Â Â | Δ8-Tetrahydrocannabinol |
| c-Jun N-terminal kinase 1 | MAPK8 | P45983 | CHEMBL2276 | Kinase | 0 | 53 / 0Â Â Â Â Â | Δ8-Tetrahydrocannabinol |
| Adenosine A2a receptor | ADORA2A | P29274 | CHEMBL251 | Family A G protein-coupled receptor | 0 | 96 / 0Â Â Â Â Â | Δ8-Tetrahydrocannabinol |
| Tyrosine-protein kinase JAK1 | JAK1 | P23458 | CHEMBL2835 | Kinase | 0 | 59 / 0Â Â Â Â Â | Δ8-Tetrahydrocannabinol |
| Tyrosine-protein kinase JAK2 | JAK2 | O60674 | CHEMBL2971 | Kinase | 0 | 138 / 0Â Â Â Â Â | Δ8-Tetrahydrocannabinol |
| JAK3/JAK1 | JAK3 JAK1 | P52333 P23458 | CHEMBL3038491 | Kinase | 0 | 15 / 0Â Â Â Â Â | Δ8-Tetrahydrocannabinol |
| Serine/threonine-protein kinase Aurora-C | AURKC | Q9UQB9 | CHEMBL3935 | Kinase | 0 | 9 / 0Â Â Â Â Â | Δ8-Tetrahydrocannabinol |
| Serine/threonine-protein kinase Aurora-A | AURKA | O14965 | CHEMBL4722 | Kinase | 0 | 16 / 0Â Â Â Â Â | Δ8-Tetrahydrocannabinol |
| Thrombin and coagulation factor X | F10 | P00742 | CHEMBL244 | Protease | 0 | 79 / 0Â Â Â Â Â | Δ8-Tetrahydrocannabinol |
| Histone deacetylase 4 | HDAC4 | P56524 | CHEMBL3524 | Eraser | 0 | 6 / 0Â Â Â Â Â | Δ8-Tetrahydrocannabinol |
| Histone deacetylase 10 | HDAC10 | Q969S8 | CHEMBL5103 | Eraser | 0 | 5 / 0Â Â Â Â Â | Δ8-Tetrahydrocannabinol |
| Cholecystokinin A receptor (by homology) | CCKAR | P32238 | CHEMBL1901 | Family A G protein-coupled receptor | 0 | 26 / 0Â Â Â Â Â | Δ8-Tetrahydrocannabinol |
| FK506-binding protein 1A | FKBP1A | P62942 | CHEMBL1902 | Isomerase | 0 | 64 / 0Â Â Â Â Â | Δ8-Tetrahydrocannabinol |
| Liver glycogen phosphorylase | PYGL | P06737 | CHEMBL2568 | Enzyme | 0 | 46 / 0Â Â Â Â Â | Δ8-Tetrahydrocannabinol |
| Nitric-oxide synthase, brain | NOS1 | P29475 | CHEMBL3568 | Enzyme | 0 | 34 / 0Â Â Â Â Â | Δ8-Tetrahydrocannabinol |
| Cathepsin (V and K) | CTSV | O60911 | CHEMBL3272 | Protease | 0 | 17 / 0Â Â Â Â Â | Δ8-Tetrahydrocannabinol |
| Sodium channel protein type IX alpha subunit | SCN9A | Q15858 | CHEMBL4296 | Voltage-gated ion channel | 0 | 43 / 0Â Â Â Â Â | Δ8-Tetrahydrocannabinol |

| | | | | | | | |
|---|---|---|---|---|---|---|---|
| Nitric-oxide synthase, endothelial | NOS3 | P29474 | CHEMBL4803 | Enzyme | 0 | 14 / 0Â Â Â Â Â | Δ8-Tetrahydrocannabinol |
| Macrophage migration inhibitory factor | MIF | P14174 | CHEMBL2085 | Enzyme | 0 | 2 / 0Â Â Â Â Â | Δ8-Tetrahydrocannabinol |
| p53-binding protein Mdm-2 | MDM2 | Q00987 | CHEMBL5023 | Other nuclear protein | 0 | 59 / 0Â Â Â Â Â | Δ8-Tetrahydrocannabinol |
| Serine/threonine-protein kinase PIM1 | PIM1 | P11309 | CHEMBL2147 | Kinase | 0 | 71 / 0Â Â Â Â Â | Δ8-Tetrahydrocannabinol |
| Phosphodiesterase 2A | PDE2A | O00408 | CHEMBL2652 | Phosphodiesterase | 0 | 32 / 0Â Â Â Â Â | Δ8-Tetrahydrocannabinol |
| Serine/threonine-protein kinase PIM2 | PIM2 | Q9P1W9 | CHEMBL4523 | Kinase | 0 | 59 / 0Â Â Â Â Â | Δ8-Tetrahydrocannabinol |
| Glutamate NMDA receptor; GRIN1/GRIN2B | GRIN1 GRIN2B | Q05586 Q13224 | CHEMBL1907603 | Ligand-gated ion channel | 0 | 1 / 0Â Â Â Â Â | Δ8-Tetrahydrocannabinol |
| Carboxypeptidase B | CPB1 | P15086 | CHEMBL2552 | Protease | 0 | 3 / 0Â Â Â Â Â | Δ8-Tetrahydrocannabinol |
| Phosphodiesterase 7A | PDE7A | Q13946 | CHEMBL3012 | Phosphodiesterase | 0 | 12 / 0Â Â Â Â Â | Δ8-Tetrahydrocannabinol |
| Tumor necrosis factor receptor R1 | TNFRSF1A | P19438 | CHEMBL3378 | Membrane receptor | 0 | 3 / 0Â Â Â Â Â | Δ8-Tetrahydrocannabinol |
| Cannabinoid receptor 1 | CNR1 | P21554 | CHEMBL218 | Family A G protein-coupled receptor | 0.959682 | 1029 / 367Â Â Â Â Â | Δ9-Tetrahydrocannabinol |
| Cannabinoid receptor 2 | CNR2 | P34972 | CHEMBL253 | Family A G protein-coupled receptor | 0.959682 | 959 / 358Â Â Â Â Â | Δ9-Tetrahydrocannabinol |
| N-arachidonyl glycine receptor | GPR18 | Q14330 | CHEMBL2384898 | Family A G protein-coupled receptor | 0.809839 | 8 / 2Â Â Â Â Â | Δ9-Tetrahydrocannabinol |
| Glycine receptor subunit alpha-1 | GLRA1 | P23415 | CHEMBL5845 | Ligand-gated ion channel | 0.809839 | 1 / 1Â Â Â Â Â | Δ9-Tetrahydrocannabinol |
| Vascular endothelial growth factor receptor 2 | KDR | P35968 | CHEMBL279 | Kinase | 0.63486 | 249 / 13Â Â Â Â Â | Δ9-Tetrahydrocannabinol |
| Glucagon receptor | GCGR | P47871 | CHEMBL1985 | Family B G protein-coupled receptor | 0.101614 | 53 / 0Â Â Â Â Â | Δ9-Tetrahydrocannabinol |
| Cystic fibrosis transmembrane conductance regulator | CFTR | P13569 | CHEMBL4051 | Other ion channel | 0.101614 | 5 / 0Â Â Â Â Â | Δ9-Tetrahydrocannabinol |
| G-protein coupled receptor 55 | GPR55 | Q9Y2T6 | CHEMBL1075322 | Family A G protein- | 0.101614 | 17 / 2Â Â Â Â Â | Δ9-Tetrahydrocannabinol |

| | | | | | | | |
|---|---|---|---|---|---|---|---|
| | | | | coupled receptor | | | |
| 5-lipoxygenase activating protein | ALOX5AP | P20292 | CHEMBL4550 | Other cytosolic protein | 0.101614 | 77 / 0Â Â Â Â Â | Δ9-Tetrahydrocannabinol |
| Cathepsin D | CTSD | P07339 | CHEMBL2581 | Protease | 0.101614 | 34 / 0Â Â Â Â Â | Δ9-Tetrahydrocannabinol |
| Serotonin 2b (5-HT2b) receptor | HTR2B | P41595 | CHEMBL1833 | Family A G protein-coupled receptor | 0.101614 | 43 / 0Â Â Â Â Â | Δ9-Tetrahydrocannabinol |
| Serotonin 2c (5-HT2c) receptor | HTR2C | P28335 | CHEMBL225 | Family A G protein-coupled receptor | 0.101614 | 67 / 0Â Â Â Â Â | Δ9-Tetrahydrocannabinol |
| Epoxide hydratase | EPHX2 | P34913 | CHEMBL2409 | Protease | 0.101614 | 218 / 0Â Â Â Â Â | Δ9-Tetrahydrocannabinol |
| Prenyl protein specific protease | RCE1 | Q9Y256 | CHEMBL3411 | Protease | 0.101614 | 6 / 0Â Â Â Â Â | Δ9-Tetrahydrocannabinol |
| Calcium sensing receptor | CASR | P41180 | CHEMBL1878 | Family C G protein-coupled receptor | 0.101614 | 40 / 0Â Â Â Â Â | Δ9-Tetrahydrocannabinol |
| Monoamine oxidase A | MAOA | P21397 | CHEMBL1951 | Oxidoreductase | 0.101614 | 103 / 0Â Â Â Â Â | Δ9-Tetrahydrocannabinol |
| Protein kinase C (PKC) | PRKCZ | Q05513 | CHEMBL3438 | Kinase | 0.101614 | 2 / 0Â Â Â Â Â | Δ9-Tetrahydrocannabinol |
| Nitric oxide synthase, inducible (by homology) | NOS2 | P35228 | CHEMBL4481 | Enzyme | 0.101614 | 68 / 0Â Â Â Â Â | Δ9-Tetrahydrocannabinol |
| Glycine transporter 1 (by homology) | SLC6A9 | P48067 | CHEMBL2337 | Electrochemical transporter | 0.101614 | 74 / 0Â Â Â Â Â | Δ9-Tetrahydrocannabinol |
| Prostanoid EP1 receptor | PTGER1 | P34995 | CHEMBL1811 | Family A G protein-coupled receptor | 0.101614 | 47 / 0Â Â Â Â Â | Δ9-Tetrahydrocannabinol |
| Thromboxane A2 receptor | TBXA2R | P21731 | CHEMBL2069 | Family A G protein-coupled receptor | 0.101614 | 8 / 0Â Â Â Â Â | Δ9-Tetrahydrocannabinol |
| Autotaxin | ENPP2 | Q13822 | CHEMBL3691 | Enzyme | 0.101614 | 2 / 0Â Â Â Â Â | Δ9-Tetrahydrocannabinol |
| Phosphodiesterase 10A | PDE10A | Q9Y233 | CHEMBL4409 | Phosphodiesterase | 0.101614 | 611 / 0Â Â Â Â Â | Δ9-Tetrahydrocannabinol |
| PH domain leucine-rich repeat-containing protein phosphatase 1 | PHLPP1 | O60346 | CHEMBL3414405 | Reader | 0.101614 | 0 / 2Â Â Â Â Â | Δ9-Tetrahydrocannabinol |
| Serine/threonine-protein kinase ILK-1 | ILK | Q13418 | CHEMBL5247 | Kinase | 0.101614 | 0 / 2Â Â Â Â Â | Δ9-Tetrahydrocannabinol |

| | | | | | | | |
|---|---|---|---|---|---|---|---|
| P2X purinoceptor 3 | P2RX3 | P56373 | CHEMBL2998 | Ligand-gated ion channel | 0.101614 | 46 / 0Â Â Â Â Â | Δ9-Tetrahydrocannabinol |
| Kinesin-like protein 1 | KIF11 | P52732 | CHEMBL4581 | Other cytosolic protein | 0.101614 | 54 / 0Â Â Â Â Â | Δ9-Tetrahydrocannabinol |
| Translocator protein (by homology) | TSPO | P30536 | CHEMBL5742 | Membrane receptor | 0.101614 | 371 / 0Â Â Â Â Â | Δ9-Tetrahydrocannabinol |
| Estradiol 17-beta-dehydrogenase 3 | HSD17B3 | P37058 | CHEMBL4234 | Enzyme | 0.101614 | 6 / 0Â Â Â Â Â | Δ9-Tetrahydrocannabinol |
| Voltage-gated potassium channel subunit Kv1.5 | KCNA5 | P22460 | CHEMBL4306 | Voltage-gated ion channel | 0.101614 | 108 / 0Â Â Â Â Â | Δ9-Tetrahydrocannabinol |
| Melanocortin receptor 4 | MC4R | P32245 | CHEMBL259 | Family A G protein-coupled receptor | 0.101614 | 14 / 0Â Â Â Â Â | Δ9-Tetrahydrocannabinol |
| Methyl-CpG-binding protein 2 | MECP2 | P51608 | CHEMBL3638346 | Reader | 0.101614 | 1 / 0Â Â Â Â Â | Δ9-Tetrahydrocannabinol |
| Methyl-CpG-binding domain protein 2 | MBD2 | Q9UBB5 | CHEMBL3707462 | Reader | 0.101614 | 1 / 0Â Â Â Â Â | Δ9-Tetrahydrocannabinol |
| Quinone reductase 2 | NQO2 | P16083 | CHEMBL3959 | Enzyme | 0.101614 | 12 / 0Â Â Â Â Â | Δ9-Tetrahydrocannabinol |
| Signal transducer and activator of transcription 3 | STAT3 | P40763 | CHEMBL4026 | Transcription factor | 0.101614 | 30 / 0Â Â Â Â Â | Δ9-Tetrahydrocannabinol |
| Histone deacetylase 2 | HDAC2 | Q92769 | CHEMBL1937 | Eraser | 0.101614 | 19 / 0Â Â Â Â Â | Δ9-Tetrahydrocannabinol |
| NADPH oxidase 4 | NOX4 | Q9NPH5 | CHEMBL1250375 | Enzyme | 0.101614 | 3 / 0Â Â Â Â Â | Δ9-Tetrahydrocannabinol |
| Protoporphyrinogen oxidase | PPOX | P50336 | CHEMBL1926488 | Oxidoreductase | 0.101614 | 2 / 0Â Â Â Â Â | Δ9-Tetrahydrocannabinol |
| Corticotropin releasing factor receptor 1 | CRHR1 | P34998 | CHEMBL1800 | Family B G protein-coupled receptor | 0.101614 | 205 / 0Â Â Â Â Â | Δ9-Tetrahydrocannabinol |
| Metabotropic glutamate receptor 5 (by homology) | GRM5 | P41594 | CHEMBL3227 | Family C G protein-coupled receptor | 0.101614 | 487 / 0Â Â Â Â Â | Δ9-Tetrahydrocannabinol |
| Type-1 angiotensin II receptor (by homology) | AGTR1 | P30556 | CHEMBL227 | Family A G protein-coupled receptor | 0.101614 | 8 / 0Â Â Â Â Â | Δ9-Tetrahydrocannabinol |
| Serine/threonine-protein kinase AKT | AKT1 | P31749 | CHEMBL4282 | Kinase | 0 | 24 / 17Â Â Â Â Â | Δ9-Tetrahydrocannabinol |
| Peregrin | BRPF1 | P55201 | CHEMBL3132741 | Reader | 0 | 27 / 0Â Â Â Â Â | Δ9-Tetrahydrocannabinol |

| | | | | | | | |
|---|---|---|---|---|---|---|---|
| Hormone sensitive lipase | LIPE | Q05469 | CHEMBL3590 | Enzyme | 0 | 63 / 0Â Â Â Â Â | Δ9-Tetrahydrocannabinol |
| FK506-binding protein 1A | FKBP1A | P62942 | CHEMBL1902 | Isomerase | 0 | 74 / 0Â Â Â Â Â | Δ9-Tetrahydrocannabinol |
| Neurokinin 2 receptor | TACR2 | P21452 | CHEMBL2327 | Family A G protein-coupled receptor | 0 | 13 / 0Â Â Â Â Â | Δ9-Tetrahydrocannabinol |
| Serotonin 6 (5-HT6) receptor | HTR6 | P50406 | CHEMBL3371 | Family A G protein-coupled receptor | 0 | 101 / 0Â Â Â Â Â | Δ9-Tetrahydrocannabinol |
| Neurokinin 3 receptor | TACR3 | P29371 | CHEMBL4429 | Family A G protein-coupled receptor | 0 | 133 / 0Â Â Â Â Â | Δ9-Tetrahydrocannabinol |
| Anandamide amidohydrolase | FAAH | O00519 | CHEMBL2243 | Enzyme | 0 | 140 / 0Â Â Â Â Â | Δ9-Tetrahydrocannabinol |
| Mitogen-activated protein kinase kinase kinase 5 | MAP3K5 | Q99683 | CHEMBL5285 | Kinase | 0 | 6 / 0Â Â Â Â Â | Δ9-Tetrahydrocannabinol |
| ADAMTS5 | ADAMTS5 | Q9UNA0 | CHEMBL2285 | Protease | 0 | 21 / 0Â Â Â Â Â | Δ9-Tetrahydrocannabinol |
| LDL-associated phospholipase A2 | PLA2G7 | Q13093 | CHEMBL3514 | Enzyme | 0 | 21 / 0Â Â Â Â Â | Δ9-Tetrahydrocannabinol |
| Estradiol 17-beta-dehydrogenase 2 | HSD17B2 | P37059 | CHEMBL2789 | Enzyme | 0 | 29 / 0Â Â Â Â Â | Δ9-Tetrahydrocannabinol |
| Calcium-activated potassium channel subunit alpha-1 | KCNMA1 | Q12791 | CHEMBL4304 | Voltage-gated ion channel | 0 | 10 / 0Â Â Â Â Â | Δ9-Tetrahydrocannabinol |
| Phosphodiesterase 5A | PDE5A | O76074 | CHEMBL1827 | Phosphodiesterase | 0 | 156 / 0Â Â Â Â Â | Δ9-Tetrahydrocannabinol |
| Glutaminyl-peptide cyclotransferase | QPCT | Q16769 | CHEMBL4508 | Enzyme | 0 | 31 / 0Â Â Â Â Â | Δ9-Tetrahydrocannabinol |
| MAP kinase p38 alpha | MAPK14 | Q16539 | CHEMBL260 | Kinase | 0 | 425 / 0Â Â Â Â Â | Δ9-Tetrahydrocannabinol |
| Voltage-gated potassium channel, KQT; KCNQ2(Kv7.2)/KCNQ3(Kv7.3) | KCNQ3 KCNQ2 | O43525 O43526 | CHEMBL2221348 | Voltage-gated ion channel | 0 | 6 / 0Â Â Â Â Â | Δ9-Tetrahydrocannabinol |
| Liver glycogen phosphorylase | PYGL | P06737 | CHEMBL2568 | Enzyme | 0 | 51 / 0Â Â Â Â Â | Δ9-Tetrahydrocannabinol |
| Proteinase-activated receptor 1 | F2R | P25116 | CHEMBL3974 | Family A G protein-coupled receptor | 0 | 54 / 0Â Â Â Â Â | Δ9-Tetrahydrocannabinol |

| | | | | | | | |
|---|---|---|---|---|---|---|---|
| Sodium channel protein type II alpha subunit | SCN2A | Q99250 | CHEMBL4187 | Voltage-gated ion channel | 0 | 37 / 0Â Â Â Â Â | Δ9-Tetrahydrocannabinol |
| Serine/threonine-protein kinase PAK 1 | PAK1 | Q13153 | CHEMBL4600 | Kinase | 0 | 1 / 0Â Â Â Â Â | Δ9-Tetrahydrocannabinol |
| Nuclear receptor ROR-gamma | RORC | P51449 | CHEMBL1741186 | Nuclear receptor | 0 | 79 / 0Â Â Â Â Â | Δ9-Tetrahydrocannabinol |
| C-C chemokine receptor type 1 | CCR1 | P32246 | CHEMBL2413 | Family A G protein-coupled receptor | 0 | 55 / 0Â Â Â Â Â | Δ9-Tetrahydrocannabinol |
| Beta secretase 2 | BACE2 | Q9Y5Z0 | CHEMBL2525 | Protease | 0 | 51 / 0Â Â Â Â Â | Δ9-Tetrahydrocannabinol |
| G-protein coupled bile acid receptor 1 | GPBAR1 | Q8TDU6 | CHEMBL5409 | Family A G protein-coupled receptor | 0 | 33 / 0Â Â Â Â Â | Δ9-Tetrahydrocannabinol |
| Formyl peptide receptor 1 | FPR1 | P21462 | CHEMBL3359 | Family A G protein-coupled receptor | 0 | 3 / 0Â Â Â Â Â | Δ9-Tetrahydrocannabinol |
| Casein kinase II alpha | CSNK2A1 | P68400 | CHEMBL3629 | Kinase | 0 | 5 / 0Â Â Â Â Â | Δ9-Tetrahydrocannabinol |
| Thymidylate synthase | TYMS | P04818 | CHEMBL1952 | Transferase | 0 | 19 / 0Â Â Â Â Â | Δ9-Tetrahydrocannabinol |
| Serine/threonine-protein kinase PIM1 | PIM1 | P11309 | CHEMBL2147 | Kinase | 0 | 77 / 0Â Â Â Â Â | Δ9-Tetrahydrocannabinol |
| Lysosomal Pro-X carboxypeptidase | PRCP | P42785 | CHEMBL2335 | Protease | 0 | 17 / 0Â Â Â Â Â | Δ9-Tetrahydrocannabinol |
| Serine/threonine-protein kinase PIM2 | PIM2 | Q9P1W9 | CHEMBL4523 | Kinase | 0 | 56 / 0Â Â Â Â Â | Δ9-Tetrahydrocannabinol |
| Orexin receptor 2 | HCRTR2 | O43614 | CHEMBL4792 | Family A G protein-coupled receptor | 0 | 469 / 0Â Â Â Â Â | Δ9-Tetrahydrocannabinol |
| L-lactate dehydrogenase B chain | LDHB | P07195 | CHEMBL4940 | Enzyme | 0 | 2 / 0Â Â Â Â Â | Δ9-Tetrahydrocannabinol |
| Orexin receptor 1 | HCRTR1 | O43613 | CHEMBL5113 | Family A G protein-coupled receptor | 0 | 405 / 0Â Â Â Â Â | Δ9-Tetrahydrocannabinol |
| Serine/threonine-protein kinase PIM3 | PIM3 | Q86V86 | CHEMBL5407 | Kinase | 0 | 10 / 0Â Â Â Â Â | Δ9-Tetrahydrocannabinol |
| Pyroglutamylated RFamide peptide receptor | QRFPR | Q96P65 | CHEMBL5852 | Family A G protein-coupled receptor | 0 | 9 / 0Â Â Â Â Â | Δ9-Tetrahydrocannabinol |

| | | | | | | | |
|---|---|---|---|---|---|---|---|
| Microtubule-associated protein tau | MAPT | P10636 | CHEMBL1293224 | Unclassified protein | 0 | 5 / 0Â Â Â Â Â | Δ9-Tetrahydrocannabinol |
| Glutamate NMDA receptor; GRIN1/GRIN2B | GRIN1 GRIN2B | Q05586 Q13224 | CHEMBL1907603 | Ligand-gated ion channel | 0 | 2 / 0Â Â Â Â Â | Δ9-Tetrahydrocannabinol |
| Adenosine A2b receptor | ADORA2B | P29275 | CHEMBL255 | Family A G protein-coupled receptor | 0 | 41 / 0Â Â Â Â Â | Δ9-Tetrahydrocannabinol |
| Sodium channel protein type IX alpha subunit | SCN9A | Q15858 | CHEMBL4296 | Voltage-gated ion channel | 0 | 65 / 0Â Â Â Â Â | Δ9-Tetrahydrocannabinol |
| Leucine-rich repeat serine/threonine-protein kinase 2 | LRRK2 | Q5S007 | CHEMBL1075104 | Kinase | 0 | 31 / 0Â Â Â Â Â | Δ9-Tetrahydrocannabinol |
| Serine/threonine-protein kinase/endoribonuclease IRE1 | ERN1 | O75460 | CHEMBL1163101 | Enzyme | 0 | 2 / 0Â Â Â Â Â | Δ9-Tetrahydrocannabinol |
| Tyrosine-protein kinase ABL | ABL1 | P00519 | CHEMBL1862 | Kinase | 0 | 114 / 0Â Â Â Â Â | Δ9-Tetrahydrocannabinol |
| Poly [ADP-ribose] polymerase-1 | PARP1 | P09874 | CHEMBL3105 | Enzyme | 0 | 36 / 0Â Â Â Â Â | Δ9-Tetrahydrocannabinol |
| Integrin alpha2/beta1 | ITGB1 ITGA2 | P05556 P17301 | CHEMBL3137268 | Unclassified protein | 0 | 1 / 0Â Â Â Â Â | Δ9-Tetrahydrocannabinol |
| P2X purinoceptor 7 | P2RX7 | Q99572 | CHEMBL4805 | Ligand-gated ion channel | 0 | 189 / 0Â Â Â Â Â | Δ9-Tetrahydrocannabinol |
| 6-phosphofructo-2-kinase/fructose-2,6-bisphosphatase 3 | PFKFB3 | Q16875 | CHEMBL2331053 | Enzyme | 0 | 70 / 0Â Â Â Â Â | Δ9-Tetrahydrocannabinol |
| Trace amine-associated receptor 1 (by homology) | TAAR1 | Q96RJ0 | CHEMBL5857 | Family A G protein-coupled receptor | 0 | 21 / 0Â Â Â Â Â | Δ9-Tetrahydrocannabinol |
| Cholesteryl ester transfer protein | CETP | P11597 | CHEMBL3572 | Other ion channel | 0 | 10 / 4Â Â Â Â Â | Δ9-Tetrahydrocannabinol |
| Muscarinic acetylcholine receptor M4 | CHRM4 | P08173 | CHEMBL1821 | Family A G protein-coupled receptor | 0 | 22 / 0Â Â Â Â Â | Δ9-Tetrahydrocannabinol |
| Muscarinic acetylcholine receptor M5 | CHRM5 | P08912 | CHEMBL2035 | Family A G protein-coupled receptor | 0 | 10 / 0Â Â Â Â Â | Δ9-Tetrahydrocannabinol |
| Thrombin and coagulation factor X | F10 | P00742 | CHEMBL244 | Protease | 0 | 133 / 0Â Â Â Â Â | Δ9-Tetrahydrocannabinol |
| Muscarinic acetylcholine receptor M3 | CHRM3 | P20309 | CHEMBL245 | Family A G protein-coupled receptor | 0 | 82 / 0Â Â Â Â Â | Δ9-Tetrahydrocannabinol |

| | | | | | | | |
|---|---|---|---|---|---|---|---|
| Cholecystokinin B receptor | CCKBR | P32239 | CHEMBL298 | Family A G protein-coupled receptor | 0 | 275 / 0Â Â Â Â Â | Δ9-Tetrahydrocannabinol |
| Protein kinase C delta | PRKCD | Q05655 | CHEMBL2996 | Kinase | 0 | 19 / 0Â Â Â Â Â | Δ9-Tetrahydrocannabinol |
| Histone deacetylase 4 | HDAC4 | P56524 | CHEMBL3524 | Eraser | 0 | 9 / 0Â Â Â Â Â | Δ9-Tetrahydrocannabinol |
| Protein kinase C theta | PRKCQ | Q04759 | CHEMBL3920 | Kinase | 0 | 31 / 0Â Â Â Â Â | Δ9-Tetrahydrocannabinol |
| Histone deacetylase 10 | HDAC10 | Q969S8 | CHEMBL5103 | Eraser | 0 | 5 / 0Â Â Â Â Â | Δ9-Tetrahydrocannabinol |
| Gamma-secretase | PSEN2 PSENEN NCSTN APH1A PSEN1 APH1B | P49810 Q9NZ42 Q92542 Q96BI3 P49768 Q8WW43 | CHEMBL2094135 | Protease | 0 | 144 / 0Â Â Â Â Â | Δ9-Tetrahydrocannabinol |
| Cannabinoid receptor 1 | CNR1 | P21554 | CHEMBL218 | Family A G protein-coupled receptor | 0.732225 | 577 / 367Â Â Â Â Â | Tetrahydrocannabivarin |
| N-arachidonyl glycine receptor | GPR18 | Q14330 | CHEMBL2384898 | Family A G protein-coupled receptor | 0.690848 | 2 / 2Â Â Â Â Â | Tetrahydrocannabivarin |
| Cannabinoid receptor 2 | CNR2 | P34972 | CHEMBL253 | Family A G protein-coupled receptor | 0.690848 | 460 / 358Â Â Â Â Â | Tetrahydrocannabivarin |
| Glycine receptor subunit alpha-1 | GLRA1 | P23415 | CHEMBL5845 | Ligand-gated ion channel | 0.690848 | 1 / 1Â Â Â Â Â | Tetrahydrocannabivarin |
| Vascular endothelial growth factor receptor 2 | KDR | P35968 | CHEMBL279 | Kinase | 0.5096 | 171 / 13Â Â Â Â Â | Tetrahydrocannabivarin |
| Glucagon receptor | GCGR | P47871 | CHEMBL1985 | Family B G protein-coupled receptor | 0.097875 | 27 / 0Â Â Â Â Â | Tetrahydrocannabivarin |
| Melanocortin receptor 4 | MC4R | P32245 | CHEMBL259 | Family A G protein-coupled receptor | 0.097875 | 20 / 0Â Â Â Â Â | Tetrahydrocannabivarin |
| Quinone reductase 2 | NQO2 | P16083 | CHEMBL3959 | Enzyme | 0.097875 | 10 / 0Â Â Â Â Â | Tetrahydrocannabivarin |
| Cystic fibrosis transmembrane conductance regulator | CFTR | P13569 | CHEMBL4051 | Other ion channel | 0.097875 | 1 / 0Â Â Â Â Â | Tetrahydrocannabivarin |
| Calcium sensing receptor | CASR | P41180 | CHEMBL1878 | Family C G protein- | 0.097875 | 29 / 0Â Â Â Â Â | Tetrahydrocannabivarin |

| | | | | | | | |
|---|---|---|---|---|---|---|---|
| | | | | coupled receptor | | | |
| Poly [ADP-ribose] polymerase-1 | PARP1 | P09874 | CHEMBL3105 | Enzyme | 0.097875 | 15 / 0Â Â Â Â Â | Tetrahydrocannabivarin |
| Epoxide hydratase | EPHX2 | P34913 | CHEMBL2409 | Protease | 0.097875 | 107 / 0Â Â Â Â Â | Tetrahydrocannabivarin |
| Serotonin 2b (5-HT2b) receptor | HTR2B | P41595 | CHEMBL1833 | Family A G protein-coupled receptor | 0.097875 | 39 / 0Â Â Â Â Â | Tetrahydrocannabivarin |
| Serotonin 2c (5-HT2c) receptor | HTR2C | P28335 | CHEMBL225 | Family A G protein-coupled receptor | 0.097875 | 45 / 0Â Â Â Â Â | Tetrahydrocannabivarin |
| Translocator protein (by homology) | TSPO | P30536 | CHEMBL5742 | Membrane receptor | 0.097875 | 347 / 0Â Â Â Â Â | Tetrahydrocannabivarin |
| Cathepsin D | CTSD | P07339 | CHEMBL2581 | Protease | 0.097875 | 11 / 0Â Â Â Â Â | Tetrahydrocannabivarin |
| L-lactate dehydrogenase A chain | LDHA | P00338 | CHEMBL4835 | Enzyme | 0.097875 | 6 / 0Â Â Â Â Â | Tetrahydrocannabivarin |
| Protein kinase C (PKC) | PRKCZ | Q05513 | CHEMBL3438 | Kinase | 0.097875 | 2 / 0Â Â Â Â Â | Tetrahydrocannabivarin |
| Estradiol 17-beta-dehydrogenase 3 | HSD17B3 | P37058 | CHEMBL4234 | Enzyme | 0.097875 | 9 / 0Â Â Â Â Â | Tetrahydrocannabivarin |
| Nitric oxide synthase, inducible (by homology) | NOS2 | P35228 | CHEMBL4481 | Enzyme | 0.097875 | 72 / 0Â Â Â Â Â | Tetrahydrocannabivarin |
| PH domain leucine-rich repeat-containing protein phosphatase 1 | PHLPP1 | O60346 | CHEMBL3414405 | Reader | 0.097875 | 0 / 2Â Â Â Â Â | Tetrahydrocannabivarin |
| Serine/threonine-protein kinase ILK-1 | ILK | Q13418 | CHEMBL5247 | Kinase | 0.097875 | 0 / 2Â Â Â Â Â | Tetrahydrocannabivarin |
| Anandamide amidohydrolase | FAAH | O00519 | CHEMBL2243 | Enzyme | 0.097875 | 35 / 0Â Â Â Â Â | Tetrahydrocannabivarin |
| LDL-associated phospholipase A2 | PLA2G7 | Q13093 | CHEMBL3514 | Enzyme | 0.097875 | 14 / 0Â Â Â Â Â | Tetrahydrocannabivarin |
| Estradiol 17-beta-dehydrogenase 2 | HSD17B2 | P37059 | CHEMBL2789 | Enzyme | 0.097875 | 15 / 0Â Â Â Â Â | Tetrahydrocannabivarin |
| 5-lipoxygenase activating protein | ALOX5AP | P20292 | CHEMBL4550 | Other cytosolic protein | 0.097875 | 31 / 0Â Â Â Â Â | Tetrahydrocannabivarin |
| GABA-A receptor; alpha-1/beta-2/gamma-2 | GABRA1 GABRB2 GABRG2 | P14867 P47870 P18507 | CHEMBL2095172 | Ligand-gated ion channel | 0.097875 | 14 / 0Â Â Â Â Â | Tetrahydrocannabivarin |
| Sodium channel protein type II alpha subunit | SCN2A | Q99250 | CHEMBL4187 | Voltage-gated ion channel | 0.097875 | 23 / 0Â Â Â Â Â | Tetrahydrocannabivarin |
| Prostanoid EP1 receptor | PTGER1 | P34995 | CHEMBL1811 | Family A G protein-coupled receptor | 0.097875 | 25 / 0Â Â Â Â Â | Tetrahydrocannabivarin |
| Calcium-activated potassium channel subunit alpha-1 | KCNMA1 | Q12791 | CHEMBL4304 | Voltage-gated ion channel | 0.097875 | 11 / 0Â Â Â Â Â | Tetrahydrocannabivarin |

| | | | | | | | |
|---|---|---|---|---|---|---|---|
| Cholecystokinin B receptor | CCKBR | P32239 | CHEMBL298 | Family A G protein-coupled receptor | 0 | 146 / 0Â Â Â Â Â | Tetrahydrocannabivarin |
| Monoglyceride lipase | MGLL | Q99685 | CHEMBL4191 | Enzyme | 0 | 11 / 0Â Â Â Â Â | Tetrahydrocannabivarin |
| Orexin receptor 2 | HCRTR2 | O43614 | CHEMBL4792 | Family A G protein-coupled receptor | 0 | 207 / 0Â Â Â Â Â | Tetrahydrocannabivarin |
| Orexin receptor 1 | HCRTR1 | O43613 | CHEMBL5113 | Family A G protein-coupled receptor | 0 | 170 / 0Â Â Â Â Â | Tetrahydrocannabivarin |
| G-protein coupled receptor 55 | GPR55 | Q9Y2T6 | CHEMBL1075322 | Family A G protein-coupled receptor | 0 | 8 / 2Â Â Â Â Â | Tetrahydrocannabivarin |
| Tyrosine-protein kinase JAK3 | JAK3 | P52333 | CHEMBL2148 | Kinase | 0 | 166 / 0Â Â Â Â Â | Tetrahydrocannabivarin |
| Tyrosine-protein kinase JAK2 | JAK2 | O60674 | CHEMBL2971 | Kinase | 0 | 159 / 0Â Â Â Â Â | Tetrahydrocannabivarin |
| Macrophage migration inhibitory factor | MIF | P14174 | CHEMBL2085 | Enzyme | 0 | 3 / 0Â Â Â Â Â | Tetrahydrocannabivarin |
| Peregrin | BRPF1 | P55201 | CHEMBL3132741 | Reader | 0 | 19 / 0Â Â Â Â Â | Tetrahydrocannabivarin |
| Histone deacetylase 2 | HDAC2 | Q92769 | CHEMBL1937 | Eraser | 0 | 11 / 0Â Â Â Â Â | Tetrahydrocannabivarin |
| Serine/threonine-protein kinase/endoribonuclease IRE1 | ERN1 | O75460 | CHEMBL1163101 | Enzyme | 0 | 2 / 0Â Â Â Â Â | Tetrahydrocannabivarin |
| Myosin light chain kinase, smooth muscle | MYLK | Q15746 | CHEMBL2428 | Kinase | 0 | 6 / 0Â Â Â Â Â | Tetrahydrocannabivarin |
| Endothelin-converting enzyme 2 | ECE2 | O60344 | CHEMBL5890 | Protease | 0 | 1 / 0Â Â Â Â Â | Tetrahydrocannabivarin |
| Thromboxane A2 receptor | TBXA2R | P21731 | CHEMBL2069 | Family A G protein-coupled receptor | 0 | 4 / 0Â Â Â Â Â | Tetrahydrocannabivarin |
| Thymidylate synthase | TYMS | P04818 | CHEMBL1952 | Transferase | 0 | 20 / 0Â Â Â Â Â | Tetrahydrocannabivarin |
| Serine/threonine-protein kinase PAK 1 | PAK1 | Q13153 | CHEMBL4600 | Kinase | 0 | 1 / 0Â Â Â Â Â | Tetrahydrocannabivarin |
| Cholesteryl ester transfer protein | CETP | P11597 | CHEMBL3572 | Other ion channel | 0 | 4 / 4Â Â Â Â Â | Tetrahydrocannabivarin |
| Prenyl protein specific protease | RCE1 | Q9Y256 | CHEMBL3411 | Protease | 0 | 6 / 0Â Â Â Â Â | Tetrahydrocannabivarin |
| Gonadotropin-releasing hormone receptor | GNRHR | P30968 | CHEMBL1855 | Family A G protein-coupled receptor | 0 | 4 / 0Â Â Â Â Â | Tetrahydrocannabivarin |
| Hormone sensitive lipase | LIPE | Q05469 | CHEMBL3590 | Enzyme | 0 | 50 / 0Â Â Â Â Â | Tetrahydrocannabivarin |
| Bombesin receptor subtype-3 | BRS3 | P32247 | CHEMBL4080 | Family A G protein- | 0 | 1 / 0Â Â Â Â Â | Tetrahydrocannabivarin |

| | | | | | | | |
|---|---|---|---|---|---|---|---|
| | | | | coupled receptor | | | |
| Leucine-rich repeat serine/threonine-protein kinase 2 | LRRK2 | Q5S007 | CHEMBL1075104 | Kinase | 0 | 24 / 0Â Â Â Â Â | Tetrahydrocannabivarin |
| Serine/threonine-protein kinase PIM1 | PIM1 | P11309 | CHEMBL2147 | Kinase | 0 | 66 / 0Â Â Â Â Â | Tetrahydrocannabivarin |
| Voltage-gated potassium channel subunit Kv1.5 | KCNA5 | P22460 | CHEMBL4306 | Voltage-gated ion channel | 0 | 99 / 0Â Â Â Â Â | Tetrahydrocannabivarin |
| Serine/threonine-protein kinase PIM2 | PIM2 | Q9P1W9 | CHEMBL4523 | Kinase | 0 | 58 / 0Â Â Â Â Â | Tetrahydrocannabivarin |
| Microtubule-associated protein tau | MAPT | P10636 | CHEMBL1293224 | Unclassified protein | 0 | 2 / 0Â Â Â Â Â | Tetrahydrocannabivarin |
| Cyclin-dependent kinase 5/CDK5 activator 1 | CDK5R1 CDK5 | Q15078 Q00535 | CHEMBL1907600 | Kinase | 0 | 65 / 0Â Â Â Â Â | Tetrahydrocannabivarin |
| Sodium channel protein type V alpha subunit | SCN5A | Q14524 | CHEMBL1980 | Voltage-gated ion channel | 0 | 15 / 0Â Â Â Â Â | Tetrahydrocannabivarin |
| Serotonin 6 (5-HT6) receptor | HTR6 | P50406 | CHEMBL3371 | Family A G protein-coupled receptor | 0 | 71 / 0Â Â Â Â Â | Tetrahydrocannabivarin |
| Serine/threonine-protein kinase Aurora-C | AURKC | Q9UQB9 | CHEMBL3935 | Kinase | 0 | 9 / 0Â Â Â Â Â | Tetrahydrocannabivarin |
| Serine/threonine-protein kinase Aurora-A | AURKA | O14965 | CHEMBL4722 | Kinase | 0 | 19 / 0Â Â Â Â Â | Tetrahydrocannabivarin |
| Protoporphyrinogen oxidase | PPOX | P50336 | CHEMBL1926488 | Oxidoreductase | 0 | 1 / 0Â Â Â Â Â | Tetrahydrocannabivarin |
| Tumor necrosis factor receptor R1 | TNFRSF1A | P19438 | CHEMBL3378 | Membrane receptor | 0 | 2 / 0Â Â Â Â Â | Tetrahydrocannabivarin |
| G-protein coupled bile acid receptor 1 | GPBAR1 | Q8TDU6 | CHEMBL5409 | Family A G protein-coupled receptor | 0 | 24 / 0Â Â Â Â Â | Tetrahydrocannabivarin |
| Serine/threonine-protein kinase AKT | AKT1 | P31749 | CHEMBL4282 | Kinase | 0 | 14 / 17Â Â Â Â Â | Tetrahydrocannabivarin |
| Monoamine oxidase A | MAOA | P21397 | CHEMBL1951 | Oxidoreductase | 0 | 67 / 0Â Â Â Â Â | Tetrahydrocannabivarin |
| Cathepsin (V and K) | CTSV | O60911 | CHEMBL3272 | Protease | 0 | 6 / 0Â Â Â Â Â | Tetrahydrocannabivarin |
| Androgen Receptor | AR | P10275 | CHEMBL1871 | Nuclear receptor | 0 | 56 / 27Â Â Â Â Â | Tetrahydrocannabivarin |
| Dual-specificity tyrosine-phosphorylation regulated kinase 1A (by homology) | DYRK1A | Q13627 | CHEMBL2292 | Kinase | 0 | 38 / 0Â Â Â Â Â | Tetrahydrocannabivarin |
| Serotonin 3a (5-HT3a) receptor | HTR3A | P46098 | CHEMBL1899 | Ligand-gated ion channel | 0 | 11 / 0Â Â Â Â Â | Tetrahydrocannabivarin |
| Lysosomal Pro-X carboxypeptidase | PRCP | P42785 | CHEMBL2335 | Protease | 0 | 8 / 0Â Â Â Â Â | Tetrahydrocannabivarin |
| Kinesin-like protein 1 | KIF11 | P52732 | CHEMBL4581 | Other cytosolic protein | 0 | 26 / 0Â Â Â Â Â | Tetrahydrocannabivarin |

| | | | | | | | |
|---|---|---|---|---|---|---|---|
| Muscarinic acetylcholine receptor M4 | CHRM4 | P08173 | CHEMBL1821 | Family A G protein-coupled receptor | 0 | 7 / 0Â Â Â Â Â | Tetrahydrocannabivarin |
| Beta secretase 2 | BACE2 | Q9Y5Z0 | CHEMBL2525 | Protease | 0 | 22 / 0Â Â Â Â Â | Tetrahydrocannabivarin |
| Serine/threonine-protein kinase mTOR | MTOR | P42345 | CHEMBL2842 | Kinase | 0 | 25 / 0Â Â Â Â Â | Tetrahydrocannabivarin |
| PI3-kinase p110-alpha subunit | PIK3CA | P42336 | CHEMBL4005 | Enzyme | 0 | 54 / 0Â Â Â Â Â | Tetrahydrocannabivarin |
| Pyruvate kinase isozymes M1/M2 | PKM | P14618 | CHEMBL1075189 | Enzyme | 0 | 3 / 0Â Â Â Â Â | Tetrahydrocannabivarin |
| Corticotropin releasing factor receptor 1 | CRHR1 | P34998 | CHEMBL1800 | Family B G protein-coupled receptor | 0 | 68 / 0Â Â Â Â Â | Tetrahydrocannabivarin |
| GABA-A receptor; alpha-1/beta-3/gamma-2 | GABRB3 GABRG2 GABRA1 | P28472 P18507 P14867 | CHEMBL2094121 | Ligand-gated ion channel | 0 | 51 / 0Â Â Â Â Â | Tetrahydrocannabivarin |
| Cyclin-dependent kinase 1/cyclin B | CCNB3 CDK1 CCNB1 CCNB2 | Q8WWL7 P06493 P14635 O95067 | CHEMBL2094127 | Other cytosolic protein | 0 | 35 / 0Â Â Â Â Â | Tetrahydrocannabivarin |
| Norepinephrine transporter | SLC6A2 | P23975 | CHEMBL222 | Electrochemical transporter | 0 | 9 / 0Â Â Â Â Â | Tetrahydrocannabivarin |
| Phosphodiesterase 2A | PDE2A | O00408 | CHEMBL2652 | Phosphodiesterase | 0 | 44 / 0Â Â Â Â Â | Tetrahydrocannabivarin |
| Tyrosine-protein kinase JAK1 | JAK1 | P23458 | CHEMBL2835 | Kinase | 0 | 64 / 0Â Â Â Â Â | Tetrahydrocannabivarin |
| JAK3/JAK1 | JAK3 JAK1 | P52333 P23458 | CHEMBL3038491 | Kinase | 0 | 15 / 0Â Â Â Â Â | Tetrahydrocannabivarin |
| Phosphodiesterase 10A | PDE10A | Q9Y233 | CHEMBL4409 | Phosphodiesterase | 0 | 265 / 0Â Â Â Â Â | Tetrahydrocannabivarin |
| Egl nine homolog 1 | EGLN1 | Q9GZT9 | CHEMBL5697 | Oxidoreductase | 0 | 7 / 0Â Â Â Â Â | Tetrahydrocannabivarin |
| Cholecystokinin A receptor (by homology) | CCKAR | P32238 | CHEMBL1901 | Family A G protein-coupled receptor | 0 | 12 / 0Â Â Â Â Â | Tetrahydrocannabivarin |
| PI3-kinase p110-alpha/p85-alpha | PIK3CA PIK3R1 | P42336 P27986 | CHEMBL2111367 | Enzyme | 0 | 6 / 0Â Â Â Â Â | Tetrahydrocannabivarin |
| Bromodomain-containing protein 1 | BRD1 | O95696 | CHEMBL2176774 | Reader | 0 | 6 / 0Â Â Â Â Â | Tetrahydrocannabivarin |
| Adenosine A2a receptor | ADORA2A | P29274 | CHEMBL251 | Family A G protein-coupled receptor | 0 | 101 / 0Â Â Â Â Â | Tetrahydrocannabivarin |
| Transcription intermediary factor 1-alpha | TRIM24 | O15164 | CHEMBL3108638 | Reader | 0 | 10 / 0Â Â Â Â Â | Tetrahydrocannabivarin |
| Histone deacetylase 9 | HDAC9 | Q9UKV0 | CHEMBL4145 | Eraser | 0 | 5 / 0Â Â Â Â Â | Tetrahydrocannabivarin |

| | | | | | | | |
|---|---|---|---|---|---|---|---|
| GABA-A receptor; alpha-3/beta-3/gamma-2 | GABRB3 GABRA3 GABRG2 | P28472 P34903 P18507 | CHEMBL2094120 | Ligand-gated ion channel | 0 | 58 / 0Â Â Â Â Â | Tetrahydrocannabivarin |
| GABA-A receptor; alpha-5/beta-3/gamma-2 | GABRB3 GABRG2 GABRA5 | P28472 P18507 P31644 | CHEMBL2094122 | Ligand-gated ion channel | 0 | 58 / 0Â Â Â Â Â | Tetrahydrocannabivarin |
| GABA-A receptor; alpha-2/beta-3/gamma-2 | GABRA2 GABRB3 GABRG2 | P47869 P28472 P18507 | CHEMBL2094130 | Ligand-gated ion channel | 0 | 54 / 0Â Â Â Â Â | Tetrahydrocannabivarin |
| Trace amine-associated receptor 1 (by homology) | TAAR1 | Q96RJ0 | CHEMBL5857 | Family A G protein-coupled receptor | 0 | 27 / 0Â Â Â Â Â | Tetrahydrocannabivarin |
| Tyrosine-protein kinase receptor FLT3 | FLT3 | P36888 | CHEMBL1974 | Kinase | 0 | 29 / 0Â Â Â Â Â | Tetrahydrocannabivarin |
| Nitric-oxide synthase, brain | NOS1 | P29475 | CHEMBL3568 | Enzyme | 0 | 52 / 0Â Â Â Â Â | Tetrahydrocannabivarin |
| Bcl2-antagonist of cell death (BAD) | BAD | Q92934 | CHEMBL3817 | Other cytosolic protein | 0 | 6 / 0Â Â Â Â Â | Tetrahydrocannabivarin |
| Nitric-oxide synthase, endothelial | NOS3 | P29474 | CHEMBL4803 | Enzyme | 0 | 19 / 0Â Â Â Â Â | Tetrahydrocannabivarin |

**Table S3. Disgenet-predicted disease targets for rheumatoid arthritis**

| Disease | Disease id | Gene | Geneid | UniProt | Gene Full Name | Protein Class | N_diseases_g | DSI_g | DPI_g | pLI | Score_gda | EL_gda | EI_gda | N_PMIDs | N_SNPs_gda | First_Ref | Last_Ref |
|---|---|---|---|---|---|---|---|---|---|---|---|---|---|---|---|---|---|
| Rheumatoid Arthritis | C0003873 | PTPN22 | 26191 | Q9Y2R2 | protein tyrosine phosphatase non-receptor type 22 | | 400 | 0.438 | 0.846 | 3.49E-21 | 0.7 | | 0.899 | 158 | 7 | 2004 | 2019 |
| Rheumatoid Ar | C0003873 | SLC22A4 | 6583 | Q9H015 | solute carrier family 22 member 4 | Transporter | 90 | 0.603 | 0.769 | 1.83E-08 | 0.7 | | 0.857 | 21 | 3 | 2003 | 2019 |

| | | | | | | | | | | | | | | | | | |
|---|---|---|---|---|---|---|---|---|---|---|---|---|---|---|---|---|---|
| thritis | | | | | | | | | | | | | | | | | |
| Rheumatoid Arthritis | C0003873 | TNF | 7124 | P01375 | tumor necrosis factor | Signaling | 2724 | 0.231 | 0.962 | 0.8033 | 0.7 | | 0.975 | 1014 | 8 | 1989 | 2020 |
| Rheumatoid Arthritis | C0003873 | CRP | 1401 | P02741 | C-reactive protein | | 1483 | 0.299 | 0.962 | 0.003697 | 0.6 | | 0.955 | 199 | 1 | 1989 | 2020 |
| Rheumatoid Arthritis | C0003873 | IL6ST | 3572 | P40189 | interleukin 6 signal transducer | Signaling | 260 | 0.475 | 0.808 | 0.99978 | 0.59 | | 0.909 | 11 | 1 | 2000 | 2020 |
| Rheumatoid Arthritis | C0003873 | CCR6 | 1235 | P51684 | C-C motif chemokine receptor 6 | G-protein coupled receptor | 231 | 0.485 | 0.808 | 0.005787 | 0.5 | | 1 | 27 | 8 | 2001 | 2019 |
| Rheumatoid Arthritis | C0003873 | FCGR2A | 2212 | P12318 | Fc fragment of IgG receptor IIa | Cell adhesion | 227 | 0.48 | 0.769 | 2.01E-06 | 0.5 | | 0.852 | 27 | 3 | 2002 | 2020 |
| Rheumatoid Arthritis | C0003873 | PADI4 | 23569 | Q9UM07 | peptidyl arginine deiminase 4 | | 196 | 0.494 | 0.808 | 4.6E-16 | 0.5 | | 0.912 | 125 | 10 | 2003 | 2020 |
| Rheu | C00 | HLA- | 31 | P04440 | major histoco | Immu | 324 | 0. | 0. | 5.3 | 0.5 | | 0.9 | 32 | 62 | 1988 | 20 |

| | | | | | | | | | | | | | | | | | |
|---|---|---|---|---|---|---|---|---|---|---|---|---|---|---|---|---|---|
| matoid Arthritis | 03873 | DPB1 | 15 | | mpatibility complex, class II, DP beta 1 | neresponse | | 453 | 923 | 3E-10 | | | 38 | | | | 20 |
| Rheumatoid Arthritis | C0003873 | HLA-DRB1 | 3123 | P01911 | major histocompatibility complex, class II, DR beta 1 | Immune response | 1018 | 0.333 | 0.923 | 0.0011 | 0.5 | | 0.936 | 575 | 7 | 1989 | 2020 |
| Rheumatoid Arthritis | C0003873 | IL2RA | 3559 | P01589 | interleukin 2 receptor subunit alpha | Receptor | 540 | 0.389 | 0.885 | 0.0054709 | 0.5 | | 1 | 32 | 3 | 1992 | 2019 |
| Rheumatoid Arthritis | C0003873 | IL6R | 3570 | P08887 | interleukin 6 receptor | Signaling | 261 | 0.471 | 0.846 | 3.17E-05 | 0.5 | | 0.971 | 34 | 5 | 2001 | 2019 |
| Rheumatoid Arthritis | C0003873 | IL10 | 3586 | P22301 | interleukin 10 | | 1679 | 0.281 | 0.923 | 0.005887 | 0.5 | | 0.946 | 129 | 3 | 1994 | 2020 |
| Rheumatoid Arthritis | C0003873 | IRF5 | 3663 | Q13568 | interferon regulatory factor 5 | Transcription factor | 226 | 0.489 | 0.731 | 0.009037 | 0.5 | | 0.906 | 32 | 6 | 2006 | 2019 |
| Rheumatoid Arthritis | C0003873 | CIITA | 4261 | P33076 | class II major histocompatibility complex transactivator | | 149 | 0.536 | 0.808 | 1.36E-07 | 0.5 | | 0.533 | 15 | 3 | 2006 | 2017 |

| | | | | | | | | | | | | | | | | | |
|---|---|---|---|---|---|---|---|---|---|---|---|---|---|---|---|---|---|
| Rheumatoid Arthritis | C0003873 | STAT4 | 6775 | Q14765 | signal transducer and activator of transcription 4 | Nucleic acid binding | 316 | 0.471 | 0.769 | 0.7662 | 0.5 | | 0.889 | 63 | 9 | 2000 | 2019 |
| Rheumatoid Arthritis | C0003873 | TNFAIP3 | 7128 | P21580 | TNF alpha induced protein 3 | Enzyme | 212 | 0.481 | 0.808 | 0.99972 | 0.5 | | 1 | 35 | 8 | 2007 | 2018 |
| Rheumatoid Arthritis | C0003873 | TRAF1 | 7185 | Q13077 | TNF receptor associated factor 1 | Signaling | 119 | 0.554 | 0.731 | 0.003564 | 0.5 | | 0.85 | 40 | 3 | 2007 | 2019 |
| Rheumatoid Arthritis | C0003873 | TRAF6 | 7189 | Q9Y4K3 | TNF receptor associated factor 6 | Signaling | 254 | 0.472 | 0.808 | 0.99739 | 0.5 | | 1 | 13 | 1 | 2008 | 2019 |
| Rheumatoid Arthritis | C0003873 | CD28 | 940 | P10747 | CD28 molecule | | 364 | 0.436 | 0.885 | 0.35779 | 0.5 | | 1 | 36 | 2 | 1996 | 2018 |
| Rheumatoid Arthritis | C0003873 | CD40 | 958 | P25942 | CD40 molecule | | 528 | 0.396 | 0.846 | 0.84603 | 0.5 | | 0.848 | 33 | 5 | 2002 | 2019 |
| Rheumatoid Ar | C0003873 | PTPN2 | 5771 | P17706 | protein tyrosine phosphatase non-receptor type 2 | | 134 | 0.538 | 0.692 | 0.99953 | 0.49 | | 1 | 13 | 4 | 2012 | 2019 |

| | | | | | | | | | | | | | | | | | |
|---|---|---|---|---|---|---|---|---|---|---|---|---|---|---|---|---|---|
| thritis | | | | | | | | | | | | | | | | | |
| Rheumatoid Arthritis | C0003873 | TYK2 | 7297 | P29597 | tyrosine kinase 2 | Kinase | 172 | 0.505 | 0.769 | 4.5E-08 | 0.49 | | 1 | 15 | 3 | 2007 | 2019 |
| Rheumatoid Arthritis | C0003873 | RUNX1 | 861 | Q01196 | RUNX family transcription factor 1 | Transcription factor | 412 | 0.425 | 0.808 | 0.65377 | 0.49 | | 0.786 | 14 | 4 | 2003 | 2019 |
| Rheumatoid Arthritis | C0003873 | PTPRC | 5788 | P08575 | protein tyrosine phosphatase receptor type C | Enzyme | 366 | 0.435 | 0.808 | 1 | 0.48 | | 1 | 10 | 1 | 1991 | 2017 |
| Rheumatoid Arthritis | C0003873 | AGER | 177 | Q15109 | advanced glycosylation end-product specific receptor | Receptor | 450 | 0.42 | 0.885 | 6.35E-16 | 0.47 | | 1 | 10 | 5 | 2002 | 2014 |
| Rheumatoid Arthritis | C0003873 | IL2RB | 3560 | P14784 | interleukin 2 receptor subunit beta | Receptor | 226 | 0.491 | 0.846 | 0.29095 | 0.47 | | 0.909 | 11 | 4 | 1992 | 2019 |
| Rheumatoid Arthritis | C0003873 | NFKBIL1 | 4795 | Q9UBC1 | NFKB inhibitor like 1 | | 41 | 0.653 | 0.615 | 0.025515 | 0.47 | | 1 | 10 | 4 | 2001 | 2011 |
| Rheumatoi | C0003 | AFF3 | 3899 | P51826 | AF4/FMR2 family | Transcripti | 77 | 0.6 | 0.6 | 0.999 | 0.46 | | 1 | 11 | 5 | 2009 | 2016 |

| | | | | | | | | | | | | | | | | | |
|---|---|---|---|---|---|---|---|---|---|---|---|---|---|---|---|---|---|
| d Arthritis | 873 | | | | member 3 | on factor | | 23 | 92 | 96 | | | | | | | |
| Rheumatoid Arthritis | C0003873 | REL | 5966 | Q04864 | REL proto-oncogene, NF-kB subunit | Transcription factor | 90 | 0.579 | 0.692 | 0.99836 | 0.46 | | 0.909 | 11 | 2 | 2009 | 2019 |
| Rheumatoid Arthritis | C0003873 | BLK | 640 | P51451 | BLK proto-oncogene, Src family tyrosine kinase | Kinase | 86 | 0.585 | 0.769 | 8.35E-14 | 0.46 | | 1 | 9 | 3 | 2009 | 2016 |
| Rheumatoid Arthritis | C0003873 | CDK6 | 1021 | Q00534 | cyclin dependent kinase 6 | Kinase | 266 | 0.471 | 0.731 | 0.98315 | 0.44 | | 0.714 | 7 | 2 | 2008 | 2019 |
| Rheumatoid Arthritis | C0003873 | CD244 | 51744 | Q9BZW8 | CD244 molecule | | 42 | 0.653 | 0.5 | 0.057288 | 0.44 | | 0.5 | 4 | 2 | 2008 | 2011 |
| Rheumatoid Arthritis | C0003873 | MMEL1 | 79258 | Q495T6 | membrane metalloendopeptidase like 1 | Enzyme | 74 | 0.606 | 0.692 | 1.24E-22 | 0.44 | | 0.909 | 11 | 5 | 2008 | 2019 |
| Rheumatoid Arthritis | C0003873 | ANKRD55 | 79722 | Q3KP44 | ankyrin repeat domain 55 | | 46 | 0.65 | 0.462 | 6.76E-08 | 0.44 | | 1 | 11 | 3 | 2010 | 2019 |

| | | | | | | | | | | | | | | | | | |
|---|---|---|---|---|---|---|---|---|---|---|---|---|---|---|---|---|---|
| Rheumatoid Arthritis | C0003873 | GATA3 | 2625 | P23771 | GATA binding protein 3 | | 429 | 0.415 | 0.846 | 0.89887 | 0.43 | | 1 | 7 | 3 | 2005 | 2019 |
| Rheumatoid Arthritis | C0003873 | NFKBIE | 4794 | O00221 | NFKB inhibitor epsilon | | 26 | 0.7 | 0.385 | 0.77098 | 0.42 | | 1 | 3 | 2 | 2012 | 2016 |
| Rheumatoid Arthritis | C0003873 | PLD4 | 122618 | Q96BZ4 | phospholipase D family member 4 | | 12 | 0.78 | 0.5 | 7.73E-13 | 0.41 | | 1 | 3 | 2 | 2012 | 2018 |
| Rheumatoid Arthritis | C0003873 | DNASE1L3 | 1776 | Q13609 | deoxyribonuclease 1 like 3 | | 126 | 0.575 | 0.769 | 3E-05 | 0.41 | | 1 | 4 | 1 | 2012 | 2019 |
| Rheumatoid Arthritis | C0003873 | KIF5A | 3798 | Q12840 | kinesin family member 5A | Cellular structure | 112 | 0.593 | 0.731 | 0.99995 | 0.41 | | 1 | 4 | 1 | 2008 | 2014 |
| Rheumatoid Arthritis | C0003873 | ARID5B | 84159 | Q14865 | AT-rich interaction domain 5B | Transcription factor | 56 | 0.638 | 0.5 | 1 | 0.41 | | 1 | 6 | 3 | 2012 | 2019 |
| Rheumatoid Ar | C0003873 | RASGRP1 | 10125 | O95267 | RAS guanyl releasing protein 1 | Enzyme modul | 110 | 0.572 | 0.731 | 0.96554 | 0.4 | | 1 | 4 | 2 | 2012 | 2019 |

| | | | | | | | | | | | | | | | | |
|---|---|---|---|---|---|---|---|---|---|---|---|---|---|---|---|---|
| thritis | | | | | | ator | | | | | | | | | | |
| Rheumatoid Arthritis | C0003873 | CSF2 | 1437 | P04141 | colony stimulating factor 2 | Signaling | 1028 | 0.33 | 0.962 | 0.834668 | 0.4 | | 1 | 39 | 0 | 1989 | 2019 |
| Rheumatoid Arthritis | C0003873 | CTLA4 | 1493 | P16410 | cytotoxic T-lymphocyte associated protein 4 | | 722 | 0.369 | 0.923 | 0.940085 | 0.4 | | 0.931 | 58 | 3 | 1998 | 2019 |
| Rheumatoid Arthritis | C0003873 | ACAN | 176 | P16112 | aggrecan | Extracellular structure | 187 | 0.528 | 0.846 | 1 | 0.4 | | 1 | 12 | 0 | 1998 | 2019 |
| Rheumatoid Arthritis | C0003873 | RCAN1 | 1827 | P53805 | regulator of calcineurin 1 | Signaling | 110 | 0.572 | 0.808 | 0.016675 | 0.4 | | 1 | 2 | 2 | 2012 | 2019 |
| Rheumatoid Arthritis | C0003873 | AHR | 196 | P35869 | aryl hydrocarbon receptor | Transcription factor | 532 | 0.41 | 0.923 | 0.99999 | 0.4 | | 0.929 | 14 | 1 | 2008 | 2020 |
| Rheumatoid Arthritis | C0003873 | SPRED2 | 200734 | Q7Z698 | sprouty related EVH1 domain containing 2 | Receptor | 38 | 0.711 | 0.577 | 2.87E-07 | 0.4 | | 1 | 7 | 6 | 2010 | 2019 |
| Rheumatoi | C0003 | ENO1 | 2023 | P06733 | enolase 1 | Enzyme | 231 | 0.4 | 0.8 | 1.91E | 0.4 | | 0.905 | 21 | 0 | 2008 | 2019 |

| | | | | | | | | | | | | | | | | | |
|---|---|---|---|---|---|---|---|---|---|---|---|---|---|---|---|---|---|
| d Arthritis | 873 | | | | | | | 81 | 85 | -05 | | | | | | | |
| Rheumatoid Arthritis | C0003873 | ANXA3 | 306 | P12429 | annexin A3 | | 66 | 0.61 | 0.615 | 6.52E-12 | 0.4 | | 1 | 1 | 1 | 2012 | 2012 |
| Rheumatoid Arthritis | C0003873 | HLA-DQA2 | 3118 | P01906 | major histocompatibility complex, class II, DQ alpha 2 | Immune response | 58 | 0.617 | 0.769 | 1.01E-05 | 0.4 | | 1 | 4 | 15 | 2007 | 2011 |
| Rheumatoid Arthritis | C0003873 | IFNG | 3458 | P01579 | interferon gamma | | 1519 | 0.288 | 0.962 | 0.47156 | 0.4 | | 0.947 | 76 | 2 | 1990 | 2020 |
| Rheumatoid Arthritis | C0003873 | CCN1 | 3491 | O00622 | cellular communication network factor 1 | Signaling | 246 | 0.473 | 0.731 | 0.71388 | 0.4 | | 0.929 | 14 | 0 | 2006 | 2019 |
| Rheumatoid Arthritis | C0003873 | IL1B | 3553 | P01584 | interleukin 1 beta | | 1801 | 0.276 | 0.962 | 0.13005 | 0.4 | | 0.976 | 247 | 1 | 1989 | 2020 |
| Rheumatoid Arthritis | C0003873 | IL1RN | 3557 | P18510 | interleukin 1 receptor antagonist | | 701 | 0.373 | 0.923 | 0.030914 | 0.4 | | 0.947 | 57 | 1 | 1992 | 2020 |
| Rheu | C00 | IL6 | 35 | P05231 | interleukin 6 | | 2367 | 0. | 0. | 0.3 | 0.4 | | 0.9 | 369 | 3 | 1988 | 20 |

| | | | | | | | | | | | | | | | | | |
|---|---|---|---|---|---|---|---|---|---|---|---|---|---|---|---|---|---|
| matoid Arthritis | 03873 | | 69 | | | | | 248 | 962 | 1536 | | | 76 | | | | 20 |
| Rheumatoid Arthritis | C0003873 | CXCL8 | 3576 | P10145 | C-X-C motif chemokine ligand 8 | Signaling | 1254 | 0.31 | 0.962 | 0.000274 | 0.4 | | 0.968 | 93 | 0 | 1990 | 2020 |
| Rheumatoid Arthritis | C0003873 | IL18 | 3606 | Q14116 | interleukin 18 | | 750 | 0.365 | 0.923 | 0.0030717 | 0.4 | | 0.917 | 48 | 4 | 1999 | 2019 |
| Rheumatoid Arthritis | C0003873 | MIF | 4282 | P14174 | macrophage migration inhibitory factor | | 455 | 0.412 | 0.885 | 0.0122262 | 0.4 | | 0.895 | 19 | 2 | 1999 | 2020 |
| Rheumatoid Arthritis | C0003873 | MMP2 | 4313 | P08253 | matrix metallopeptidase 2 | Enzyme | 1021 | 0.333 | 0.923 | 0.838878 | 0.4 | | 0.923 | 26 | 1 | 1998 | 2019 |
| Rheumatoid Arthritis | C0003873 | MPO | 4353 | P05164 | myeloperoxidase | Enzyme | 653 | 0.377 | 0.923 | 7.11E-15 | 0.4 | | 1 | 13 | 0 | 2012 | 2020 |
| Rheumatoid Arthritis | C0003873 | MTHFR | 4524 | P42898 | methylenetetrahydrofolate reductase | | 985 | 0.337 | 0.885 | 3.21E-10 | 0.4 | | 0.923 | 52 | 6 | 2001 | 2018 |

| | | | | | | | | | | | | | | | | | |
|---|---|---|---|---|---|---|---|---|---|---|---|---|---|---|---|---|---|
| Rheumatoid Arthritis | C0003873 | NCF2 | 4688 | P19878 | neutrophil cytosolic factor 2 | Enzyme | 90 | 0.601 | 0.692 | 4.01E-07 | 0.4 | | 1 | 3 | 1 | 2003 | 2019 |
| Rheumatoid Arthritis | C0003873 | TNFRSF11B | 4982 | O00300 | TNF receptor superfamily member 11b | | 443 | 0.422 | 0.808 | 0.16171 | 0.4 | | 0.929 | 42 | 5 | 2001 | 2019 |
| Rheumatoid Arthritis | C0003873 | FOXP3 | 50943 | Q9BZS1 | forkhead box P3 | | 688 | 0.368 | 0.846 | 0.99424 | 0.4 | | 0.973 | 37 | 1 | 2005 | 2020 |
| Rheumatoid Arthritis | C0003873 | IL23A | 51561 | Q9NPF7 | interleukin 23 subunit alpha | Signaling | 427 | 0.415 | 0.846 | 0.35806 | 0.4 | | 1 | 36 | 0 | 1989 | 2020 |
| Rheumatoid Arthritis | C0003873 | ABCB1 | 5243 | P08183 | ATP binding cassette subfamily B member 1 | Transporter | 933 | 0.344 | 0.885 | 1.26E-05 | 0.4 | | 0.95 | 20 | 1 | 1995 | 2020 |
| Rheumatoid Arthritis | C0003873 | PON1 | 5444 | P27169 | paraoxonase 1 | | 496 | 0.409 | 0.885 | 9.81E-11 | 0.4 | | 0.917 | 12 | 2 | 2003 | 2019 |
| Rheumatoid Ar | C0003873 | PTGS2 | 5743 | P35354 | prostaglandin-endoperoxide synthase 2 | Enzyme | 1234 | 0.314 | 0.962 | 0.99597 | 0.4 | | 0.952 | 42 | 0 | 1994 | 2019 |

| | | | | | | | | | | | | | | | | | |
|---|---|---|---|---|---|---|---|---|---|---|---|---|---|---|---|---|---|
| thritis | | | | | | | | | | | | | | | | | |
| Rheumatoid Arthritis | C0003873 | CCL21 | 6366 | O00585 | C-C motif chemokine ligand 21 | Signaling | 186 | 0.502 | 0.769 | 0.036239 | 0.4 | | 0.857 | 14 | 2 | 2003 | 2019 |
| Rheumatoid Arthritis | C0003873 | SLC11A1 | 6556 | P49279 | solute carrier family 11 member 1 | Transporter | 141 | 0.525 | 0.846 | 1.55E-19 | 0.4 | | 0.938 | 16 | 6 | 1996 | 2019 |
| Rheumatoid Arthritis | C0003873 | STAT1 | 6772 | P42224 | signal transducer and activator of transcription 1 | Nucleic acid binding | 531 | 0.399 | 0.885 | 0.99999 | 0.4 | | 0.867 | 15 | 0 | 2003 | 2019 |
| Rheumatoid Arthritis | C0003873 | TLR2 | 7097 | O60603 | toll like receptor 2 | | 749 | 0.361 | 0.923 | 1.02E-06 | 0.4 | | 0.966 | 29 | 2 | 2003 | 2019 |
| Rheumatoid Arthritis | C0003873 | VEGFA | 7422 | P15692 | vascular endothelial growth factor A | Signaling | 1899 | 0.266 | 0.923 | 2.41E-05 | 0.4 | | 0.987 | 75 | 7 | 1994 | 2020 |
| Rheumatoid Arthritis | C0003873 | CXCR4 | 7852 | P61073 | C-X-C motif chemokine receptor 4 | G-protein coupled receptor | 739 | 0.362 | 0.923 | 0.017804 | 0.4 | | 0.944 | 18 | 0 | 2000 | 2019 |

| | | | | | | | | | | | | | | | | | |
|---|---|---|---|---|---|---|---|---|---|---|---|---|---|---|---|---|---|
| Rheumatoid Arthritis | C0003873 | CAT | 847 | P04040 | catalase | Enzyme | 794 | 0.359 | 0.962 | 1.63E-10 | 0.4 | | 0.917 | 12 | 0 | 2006 | 2019 |
| Rheumatoid Arthritis | C0003873 | ADIPOQ | 9370 | Q15848 | adiponectin, C1Q and collagen domain containing | | 679 | 0.376 | 0.885 | 4.64E-09 | 0.4 | | 0.957 | 23 | 4 | 2003 | 2020 |
| Rheumatoid Arthritis | C0003873 | CCN2 | 1490 | P29279 | cellular communication network factor 2 | Signaling | 518 | 0.399 | 0.846 | 0.000502 | 0.39 | | 1 | 10 | 0 | 2006 | 2020 |
| Rheumatoid Arthritis | C0003873 | TNFSF14 | 8740 | O43557 | TNF superfamily member 14 | Signaling | 143 | 0.529 | 0.769 | 0.051663 | 0.39 | | 1 | 10 | 0 | 2003 | 2017 |
| Rheumatoid Arthritis | C0003873 | DHFR | 1719 | P00374 | dihydrofolate reductase | Enzyme | 191 | 0.513 | 0.808 | 0.0343 | 0.38 | | 0.889 | 9 | 0 | 2003 | 2019 |
| Rheumatoid Arthritis | C0003873 | TNFRSF14 | 8764 | Q92956 | TNF receptor superfamily member 14 | | 100 | 0.568 | 0.731 | 0.80752 | 0.38 | | 0.889 | 9 | 0 | 2003 | 2017 |
| Rheumatoid Ar | C0003873 | TAGAP | 117289 | Q8N103 | T cell activation RhoGTPase | Enzyme modul | 24 | 0.7 | 0.462 | 0.84621 | 0.37 | | 1 | 9 | 2 | 2010 | 2018 |

| | | | | | | | | | | | | | | | | | |
|---|---|---|---|---|---|---|---|---|---|---|---|---|---|---|---|---|---|
| thritis | | | | | activating protein | ator | | | | | | | | | | | |
| Rheumatoid Arthritis | C0003873 | IRAK1 | 3654 | P51617 | interleukin 1 receptor associated kinase 1 | Kinase | 200 | 0.506 | 0.808 | 0.98774 | 0.36 | | 0.857 | 7 | 3 | 2008 | 2018 |
| Rheumatoid Arthritis | C0003873 | COL2A1 | 1280 | P02458 | collagen type II alpha 1 chain | | 496 | 0.444 | 0.846 | 1 | 0.35 | | 0.857 | 7 | 0 | 1993 | 2018 |
| Rheumatoid Arthritis | C0003873 | ALOX5 | 240 | P09917 | arachidonate 5-lipoxygenase | Enzyme | 342 | 0.447 | 0.885 | 1.21E-06 | 0.35 | | 1 | 6 | 0 | 1995 | 2017 |
| Rheumatoid Arthritis | C0003873 | GC | 2638 | P02774 | GC vitamin D binding protein | Transporter | 219 | 0.489 | 0.885 | 2.73E-11 | 0.35 | | 0.8 | 5 | 1 | 1985 | 2019 |
| Rheumatoid Arthritis | C0003873 | HOXD13 | 3239 | P35453 | homeobox D13 | | 259 | 0.483 | 0.808 | 0.008606 | 0.35 | | 1 | 6 | 0 | 1997 | 2019 |
| Rheumatoid Arthritis | C0003873 | NCF1 | 653361 | P14598 | neutrophil cytosolic factor 1 | | 170 | 0.521 | 0.808 | 2.83E-05 | 0.35 | | 1 | 7 | 1 | 2003 | 2018 |
| Rheumatoi | C0003 | TXNDC5 | 815 | Q8NBS9 | thioredoxin domain containing 5 | | 65 | 0.6 | 0.5 | 4.87E | 0.35 | | 1 | 6 | 2 | 2011 | 2018 |

| | | | | | | | | | | | | | | | | | |
|---|---|---|---|---|---|---|---|---|---|---|---|---|---|---|---|---|---|
| d Arthritis | 873 | | 67 | | | | | 15 | 77 | -13 | | | | | | | |
| Rheumatoid Arthritis | C0003873 | GRK2 | 156 | P25098 | G protein-coupled receptor kinase 2 | Kinase | 159 | 0.532 | 0.654 | 0.99998 | 0.34 | | 1 | 4 | 0 | 1999 | 2019 |
| Rheumatoid Arthritis | C0003873 | FPGS | 2356 | Q05932 | folylpolyglutamate synthase | Enzyme | 40 | 0.644 | 0.423 | 1.16E-06 | 0.34 | | 1 | 5 | 3 | 2007 | 2020 |
| Rheumatoid Arthritis | C0003873 | IGFBP3 | 3486 | P17936 | insulin like growth factor binding protein 3 | Enzyme modulator | 350 | 0.439 | 0.808 | 0.91029 | 0.34 | | 0.75 | 4 | 0 | 2007 | 2019 |
| Rheumatoid Arthritis | C0003873 | FASLG | 356 | P48023 | Fas ligand | Signaling | 398 | 0.43 | 0.885 | 0.18345 | 0.34 | | 1 | 5 | 2 | 2008 | 2015 |
| Rheumatoid Arthritis | C0003873 | ATIC | 471 | P31939 | 5-aminoimidazole-4-carboxamide ribonucleotide formyltransferase/IMP cyclohydrolase | Enzyme | 100 | 0.576 | 0.731 | 3.01E-19 | 0.34 | | 1 | 5 | 1 | 2004 | 2016 |
| Rheumatoid | C0003873 | PRKCQ | 5588 | Q04759 | protein kinase C theta | Kinase | 53 | 0.636 | 0.654 | 0.2972 | 0.34 | | 1 | 7 | 0 | 2008 | 2019 |

| | | | | | | | | | | | | | | | | | |
|---|---|---|---|---|---|---|---|---|---|---|---|---|---|---|---|---|---|
| Arthritis | | | | | | | | | | | | | | | | | |
| Rheumatoid Arthritis | C0003873 | PTGS1 | 5742 | P23219 | prostaglandin-endoperoxide synthase 1 | Enzyme | 318 | 0.46 | 0.885 | 6.24E-07 | 0.34 | | 1 | 4 | 0 | 1994 | 2004 |
| Rheumatoid Arthritis | C0003873 | CCL8 | 6355 | P80075 | C-C motif chemokine ligand 8 | Signaling | 115 | 0.546 | 0.692 | 0.041412 | 0.34 | | 1 | 5 | 0 | 2007 | 2019 |
| Rheumatoid Arthritis | C0003873 | SOD2 | 6648 | P04179 | superoxide dismutase 2 | Enzyme | 668 | 0.379 | 0.923 | 0.15499 | 0.34 | | 0.714 | 7 | 1 | 2000 | 2019 |
| Rheumatoid Arthritis | C0003873 | GGH | 8836 | Q92820 | gamma-glutamyl hydrolase | Enzyme | 66 | 0.608 | 0.5 | 0.58132 | 0.34 | | 1 | 5 | 3 | 2004 | 2020 |
| Rheumatoid Arthritis | C0003873 | B3GNT2 | 10678 | Q9NY97 | UDP-GlcNAc:betaGal beta-1,3-N-acetylglucosaminyltransferase 2 | Enzyme | 20 | 0.705 | 0.5 | 0.88722 | 0.33 | | 1 | 3 | 0 | 1988 | 2013 |
| Rheumatoid Arthritis | C0003873 | CP | 1356 | P00450 | ceruloplasmin | Enzyme | 283 | 0.466 | 0.846 | 4.2E-10 | 0.33 | | 1 | 4 | 0 | 2002 | 2018 |
| Rheu | C00 | PLB1 | 15 | Q6P1J6 | phospholipase B1 | | 219 | 0. | 0. | 1.7 | 0.33 | | 1 | 3 | 0 | 2004 | 20 |

| | | | | | | | | | | | | | | | | | |
|---|---|---|---|---|---|---|---|---|---|---|---|---|---|---|---|---|---|
| matoid Arthritis | 03873 | | 1056 | | | | | 488 | 846 | 3E-72 | | | | | | | 14 |
| Rheumatoid Arthritis | C0003873 | MMP12 | 4321 | P39900 | matrix metallopeptidase 12 | Enzyme | 216 | 0.484 | 0.885 | | 0.33 | | 1 | 4 | 1 | 2003 | 2009 |
| Rheumatoid Arthritis | C0003873 | ACKR3 | 57007 | P25106 | atypical chemokine receptor 3 | G-protein coupled receptor | 400 | 0.428 | 0.885 | 0.39134 | 0.33 | | 1 | 4 | 0 | 1999 | 2010 |
| Rheumatoid Arthritis | C0003873 | PRDM1 | 639 | O75626 | PR/SET domain 1 | | 126 | 0.538 | 0.731 | 0.9565 | 0.33 | | 0.833 | 6 | 0 | 2009 | 2019 |
| Rheumatoid Arthritis | C0003873 | CD83 | 9308 | Q01151 | CD83 molecule | | 96 | 0.566 | 0.731 | 0.005214 | 0.33 | | 0.75 | 4 | 0 | 2003 | 2017 |
| Rheumatoid Arthritis | C0003873 | ADORA2A | 135 | P29274 | adenosine A2a receptor | G-protein coupled receptor | 213 | 0.526 | 0.769 | 0.5971 | 0.32 | | 1 | 3 | 1 | 2009 | 2019 |
| Rheumatoi | C0003 | CLEC12A | 1603 | Q5QGZ9 | C-type lectin domain family 12 member A | | 34 | 0.6 | 0.3 | 2.6E- | 0.32 | | 0.667 | 3 | 0 | 2007 | 2016 |

| | | | | | | | | | | | | | | | | | |
|---|---|---|---|---|---|---|---|---|---|---|---|---|---|---|---|---|---|
| d Arthritis | 873 | | 64 | | | | | 86 | 46 | 07 | | | | | | | |
| Rheumatoid Arthritis | C0003873 | IKZF3 | 22806 | Q9UKT9 | IKAROS family zinc finger 3 | Transcription factor | 64 | 0.608 | 0.692 | 0.98416 | 0.32 | | 1 | 3 | 0 | 2012 | 2016 |
| Rheumatoid Arthritis | C0003873 | FKBP5 | 2289 | Q13451 | FKBP prolyl isomerase 5 | Enzyme | 179 | 0.524 | 0.769 | 0.73393 | 0.32 | | 1 | 3 | 0 | 2003 | 2017 |
| Rheumatoid Arthritis | C0003873 | CXCL2 | 2920 | P19875 | C-X-C motif chemokine ligand 2 | Signaling | 186 | 0.513 | 0.885 | 3.61E-07 | 0.32 | | 1 | 3 | 0 | 2004 | 2017 |
| Rheumatoid Arthritis | C0003873 | HSD11B1 | 3290 | P28845 | hydroxysteroid 11-beta dehydrogenase 1 | | 163 | 0.52 | 0.731 | 0.41696 | 0.32 | | 1 | 3 | 0 | 2009 | 2018 |
| Rheumatoid Arthritis | C0003873 | RBPJ | 3516 | Q06330 | recombination signal binding protein for immunoglobulin kappa J region | Nucleic acid binding | 161 | 0.518 | 0.808 | 0.99853 | 0.32 | | 1 | 4 | 0 | 2010 | 2016 |
| Rheumatoid Arthritis | C0003873 | STS | 412 | P08842 | steroid sulfatase | Enzyme | 382 | 0.431 | 0.846 | 0.80886 | 0.32 | | 1 | 3 | 0 | 2005 | 2019 |

| | | | | | | | | | | | | | | | | | |
|---|---|---|---|---|---|---|---|---|---|---|---|---|---|---|---|---|---|
| Rheumatoid Arthritis | C0003873 | MMP10 | 4319 | P09238 | matrix metallopeptidase 10 | Enzyme | 123 | 0.553 | 0.808 | 5.91E-15 | 0.32 | | 1 | 3 | 0 | 1989 | 2009 |
| Rheumatoid Arthritis | C0003873 | BGN | 633 | P21810 | biglycan | | 253 | 0.484 | 0.808 | 0.45229 | 0.32 | | 1 | 3 | 0 | 2004 | 2018 |
| Rheumatoid Arthritis | C0003873 | BMP4 | 652 | P12644 | bone morphogenetic protein 4 | Signaling | 423 | 0.422 | 0.808 | 0.9564 | 0.32 | | 1 | 3 | 0 | 2006 | 2019 |
| Rheumatoid Arthritis | C0003873 | SMS | 6611 | P52788 | spermine synthase | | 263 | 0.491 | 0.846 | 0.97297 | 0.32 | | 1 | 3 | 0 | 1998 | 2017 |
| Rheumatoid Arthritis | C0003873 | ABCC5 | 10057 | O15440 | ATP binding cassette subfamily C member 5 | Transporter | 71 | 0.606 | 0.654 | 0.004152 | 0.31 | | 1 | 1 | 0 | 2013 | 2013 |
| Rheumatoid Arthritis | C0003873 | ABCC4 | 10257 | O15439 | ATP binding cassette subfamily C member 4 | Transporter | 146 | 0.536 | 0.769 | 1.07E-07 | 0.31 | | 1 | 2 | 1 | 2013 | 2017 |
| Rheumatoid Ar | C0003873 | CTSD | 1509 | P07339 | cathepsin D | Enzyme | 242 | 0.478 | 0.846 | 0.0009 | 0.31 | | 1 | 2 | 0 | 1995 | 2003 |

| | | | | | | | | | | | | | | | | | |
|---|---|---|---|---|---|---|---|---|---|---|---|---|---|---|---|---|---|
| thritis | | | | | | | | | | 17 | | | | | | | |
| Rheumatoid Arthritis | C0003873 | DDX6 | 1656 | P26196 | DEAD-box helicase 6 | | 52 | 0.663 | 0.385 | 0.99964 | 0.31 | | 1 | 2 | 0 | 2012 | 2017 |
| Rheumatoid Arthritis | C0003873 | GRK6 | 2870 | P43250 | G protein-coupled receptor kinase 6 | Kinase | 51 | 0.67 | 0.654 | 0.000487 | 0.31 | | 1 | 1 | 0 | 1999 | 1999 |
| Rheumatoid Arthritis | C0003873 | HCLS1 | 3059 | P14317 | hematopoietic cell-specific Lyn substrate 1 | Cellular structure | 132 | 0.539 | 0.846 | 1.32E-06 | 0.31 | | 1 | 2 | 0 | 1992 | 2003 |
| Rheumatoid Arthritis | C0003873 | HOXD10 | 3236 | P28358 | homeobox D10 | | 83 | 0.59 | 0.538 | 0.062918 | 0.31 | | 1 | 2 | 0 | 2007 | 2018 |
| Rheumatoid Arthritis | C0003873 | PXK | 54899 | Q7Z7A4 | PX domain containing serine/threonine kinase like | Kinase | 13 | 0.821 | 0.231 | 0.010847 | 0.31 | | 1 | 2 | 0 | 2010 | 2017 |
| Rheumatoid Arthritis | C0003873 | BCL2A1 | 597 | Q16548 | BCL2 related protein A1 | Signaling | 150 | 0.529 | 0.731 | 0.20785 | 0.31 | | 1 | 2 | 0 | 2009 | 2017 |
| Rheumatoi | C0003 | BMP6 | 654 | P22004 | bone morphogenetic protein 6 | Signaling | 137 | 0.5 | 0.8 | 0.759 | 0.31 | | 1 | 2 | 0 | 2003 | 2010 |

| | | | | | | | | | | | | | | | | | |
|---|---|---|---|---|---|---|---|---|---|---|---|---|---|---|---|---|---|
| d Arthritis | 873 | | | | | | | 31 | 08 | 47 | | | | | | | |
| Rheumatoid Arthritis | C0003873 | NR4A3 | 8013 | Q92570 | nuclear receptor subfamily 4 group A member 3 | Nuclear receptor | 186 | 0.502 | 0.808 | 0.9126 | 0.31 | | 1 | 2 | 0 | 2002 | 2009 |
| Rheumatoid Arthritis | C0003873 | GDF5 | 8200 | P43026 | growth differentiation factor 5 | Signaling | 238 | 0.511 | 0.692 | 0.67002 | 0.31 | | 1 | 2 | 0 | 2008 | 2008 |
| Rheumatoid Arthritis | C0003873 | CAV2 | 858 | P51636 | caveolin 2 | Enzyme modulator | 96 | 0.575 | 0.692 | 0.026973 | 0.31 | | 1 | 1 | 0 | 2007 | 2007 |
| Rheumatoid Arthritis | C0003873 | ABCC3 | 8714 | O15438 | ATP binding cassette subfamily C member 3 | Transporter | 160 | 0.517 | 0.769 | 1.21E-26 | 0.31 | | 1 | 2 | 0 | 2012 | 2013 |
| Rheumatoid Arthritis | C0003873 | GPRC5A | 9052 | Q8NFJ5 | G protein-coupled receptor class C group 5 member A | G-protein coupled receptor | 90 | 0.579 | 0.692 | 6.79E-08 | 0.31 | | 1 | 1 | 0 | 2007 | 2007 |
| Rheumatoid Arthritis | C0003873 | CD2 | 914 | P06729 | CD2 molecule | | 50 | 0.641 | 0.577 | 0.47108 | 0.31 | | 1 | 2 | 0 | 2012 | 2017 |

| | | | | | | | | | | | | | | | | |
|---|---|---|---|---|---|---|---|---|---|---|---|---|---|---|---|---|
| Rheumatoid Arthritis | C0003873 | LHX2 | 9355 | P50458 | LIM homeobox 2 | Nucleic acid binding | 39 | 0.678 | 0.615 | 0.998812 | 0.31 | | 1 | 1 | 0 | 2007 | 2007 |
| Rheumatoid Arthritis | C0003873 | MAB21L2 | 10586 | Q9Y586 | mab-21 like 2 | | 22 | 0.743 | 0.308 | 0.437769 | 0.3 | | 1 | 1 | 0 | 2007 | 2007 |
| Rheumatoid Arthritis | C0003873 | TXNIP | 10628 | Q9H3M7 | thioredoxin interacting protein | | 208 | 0.49 | 0.731 | 0.159188 | 0.3 | | 1 | 1 | 0 | 2009 | 2009 |
| Rheumatoid Arthritis | C0003873 | ABCC2 | 1244 | Q92887 | ATP binding cassette subfamily C member 2 | Transporter | 254 | 0.483 | 0.808 | 5.2E-47 | 0.3 | | 1 | 1 | 0 | 2013 | 2013 |
| Rheumatoid Arthritis | C0003873 | HAPLN1 | 1404 | P10915 | hyaluronan and proteoglycan link protein 1 | Extracellular structure | 36 | 0.663 | 0.577 | 0.602442 | 0.3 | | 1 | 1 | 0 | 2007 | 2007 |
| Rheumatoid Arthritis | C0003873 | GSDME | 1687 | O60443 | gasdermin E | | 44 | 0.656 | 0.423 | 3.28E-09 | 0.3 | | 1 | 1 | 0 | 2007 | 2007 |
| Rheumatoid Ar | C0003873 | F2 | 2147 | P00734 | coagulation factor II, thrombin | Enzyme | 490 | 0.415 | 0.885 | 0.0011 | 0.3 | | 1 | 1 | 0 | 2015 | 2015 |

| | | | | | | | | | | | | | | | | | |
|---|---|---|---|---|---|---|---|---|---|---|---|---|---|---|---|---|---|
| thritis | | | | | | | | | | 12 | | | | | | | |
| Rheumatoid Arthritis | C0003873 | LY96 | 23643 | Q9Y6Y9 | lymphocyte antigen 96 | Receptor | 54 | 0.636 | 0.615 | 7.42E-05 | 0.3 | | 1 | 1 | 0 | 2007 | 2007 |
| Rheumatoid Arthritis | C0003873 | GART | 2618 | P22102 | phosphoribosylglycinamide formyltransferase, phosphoribosylglycinamide synthetase, phosphoribosylaminoimidazole synthetase | Enzyme | 48 | 0.631 | 0.615 | 0.000277 | 0.3 | | 1 | 1 | 0 | 2013 | 2013 |
| Rheumatoid Arthritis | C0003873 | RGMB | 285704 | Q6NW40 | repulsive guidance molecule BMP co-receptor b | | 24 | 0.711 | 0.462 | 0.071955 | 0.3 | | 1 | 1 | 0 | 2007 | 2007 |
| Rheumatoid Arthritis | C0003873 | NR3C1 | 2908 | P04150 | nuclear receptor subfamily 3 group C member 1 | Nuclear receptor | 590 | 0.393 | 0.885 | 0.96958 | 0.3 | | 0.889 | 18 | 3 | 2001 | 2019 |
| Rheumatoid Arthritis | C0003873 | HOXD11 | 3237 | P31277 | homeobox D11 | | 45 | 0.67 | 0.462 | 0.001142 | 0.3 | | 1 | 1 | 0 | 2007 | 2007 |

| | | | | | | | | | | | | | | | | | |
|---|---|---|---|---|---|---|---|---|---|---|---|---|---|---|---|---|---|
| Rheumatoid Arthritis | C0003873 | IRF8 | 3394 | Q02556 | interferon regulatory factor 8 | Transcription factor | 149 | 0.539 | 0.808 | 0.948807 | 0.3 | | 1 | 1 | 0 | 2012 | 2012 |
| Rheumatoid Arthritis | C0003873 | ITGA6 | 3655 | P23229 | integrin subunit alpha 6 | | 137 | 0.539 | 0.769 | 1.9E-07 | 0.3 | | 1 | 1 | 0 | 2007 | 2007 |
| Rheumatoid Arthritis | C0003873 | LCN2 | 3934 | P80188 | lipocalin 2 | Enzyme | 497 | 0.405 | 0.885 | 1.14E-09 | 0.3 | | 1 | 1 | 0 | 1995 | 1995 |
| Rheumatoid Arthritis | C0003873 | MARCKS | 4082 | P29966 | myristoylated alanine rich protein kinase C substrate | | 102 | 0.559 | 0.769 | 0.32648 | 0.3 | | 1 | 1 | 0 | 2009 | 2009 |
| Rheumatoid Arthritis | C0003873 | RAB8A | 4218 | P61006 | RAB8A, member RAS oncogene family | | 69 | 0.61 | 0.577 | 0.97543 | 0.3 | | 1 | 1 | 0 | 2003 | 2003 |
| Rheumatoid Arthritis | C0003873 | PLEK | 5341 | P08567 | pleckstrin | Cellular structure | 83 | 0.588 | 0.731 | 0.001067 | 0.3 | | 1 | 1 | 0 | 2003 | 2003 |
| Rheumatoid Ar | C0003873 | POU3F1 | 5453 | Q03052 | POU class 3 homeobox 1 | | 25 | 0.711 | 0.462 | 0.56469 | 0.3 | | 1 | 1 | 0 | 2012 | 2012 |

| | | | | | | | | | | | | | | | | | |
|---|---|---|---|---|---|---|---|---|---|---|---|---|---|---|---|---|---|
| thritis | | | | | | | | | | | | | | | | | |
| Rheumatoid Arthritis | C0003873 | DDIT4 | 54541 | Q9NX09 | DNA damage inducible transcript 4 | | 92 | 0.576 | 0.769 | 0.000683 | 0.3 | | 1 | 1 | 0 | 2007 | 2007 |
| Rheumatoid Arthritis | C0003873 | GIN1 | 54826 | Q9NXP7 | gypsy retrotransposon integrase 1 | | 4 | 0.89 | 0.269 | 2.42E-05 | 0.3 | | 1 | 1 | 0 | 2012 | 2012 |
| Rheumatoid Arthritis | C0003873 | BAIAP2L1 | 55971 | Q9UHR4 | BAR/IMD domain containing adaptor protein 2 like 1 | Receptor | 15 | 0.805 | 0.308 | 0.000104 | 0.3 | | 1 | 1 | 0 | 2007 | 2007 |
| Rheumatoid Arthritis | C0003873 | PSG5 | 5673 | Q15238 | pregnancy specific beta-1-glycoprotein 5 | | 61 | 0.631 | 0.615 | 8.82E-22 | 0.3 | | 1 | 1 | 0 | 2007 | 2007 |
| Rheumatoid Arthritis | C0003873 | NDUFA4L2 | 56901 | Q9NRX3 | NDUFA4 mitochondrial complex associated like 2 | Enzyme | 19 | 0.736 | 0.346 | 0.086367 | 0.3 | | 1 | 1 | 0 | 2009 | 2009 |
| Rheumatoid Arthritis | C0003873 | PTK2 | 5747 | Q05397 | protein tyrosine kinase 2 | Kinase | 205 | 0.497 | 0.808 | 0.99987 | 0.3 | | 1 | 1 | 0 | 2003 | 2003 |
| Rheumatoi | C0003 | RAP2A | 5911 | P10114 | RAP2A, member of RAS | Enzyme mo | 19 | 0.7 | 0.5 | 0.850 | 0.3 | | 1 | 1 | 0 | 2003 | 2003 |

| | | | | | | | | | | | | | | | | | |
|---|---|---|---|---|---|---|---|---|---|---|---|---|---|---|---|---|---|
| d Arthritis | 873 | | | | oncogene family | dulator | | 51 | 38 | 68 | | | | | | | |
| Rheumatoid Arthritis | C0003873 | BDKRB2 | 624 | P30411 | bradykinin receptor B2 | G-protein coupled receptor | 105 | 0.585 | 0.808 | 0.11863 | 0.3 | | 1 | 1 | 0 | 2003 | 2003 |
| Rheumatoid Arthritis | C0003873 | CXCL6 | 6372 | P80162 | C-X-C motif chemokine ligand 6 | Signaling | 90 | 0.582 | 0.692 | 2.71E-09 | 0.3 | | 1 | 1 | 0 | 2004 | 2004 |
| Rheumatoid Arthritis | C0003873 | TLE3 | 7090 | Q04726 | TLE family member 3, transcriptional corepressor | Transcription factor | 38 | 0.674 | 0.462 | 1 | 0.3 | | 1 | 1 | 0 | 2012 | 2012 |
| Rheumatoid Arthritis | C0003873 | TMPO | 7112 | P42166;P42167 | thymopoietin | Signaling | 58 | 0.631 | 0.577 | 1.55E-07 | 0.3 | | 1 | 1 | 0 | 2003 | 2003 |
| Rheumatoid Arthritis | C0003873 | TNFAIP2 | 7127 | Q03169 | TNF alpha induced protein 2 | Transporter | 25 | 0.711 | 0.423 | 8.25E-05 | 0.3 | | 1 | 1 | 0 | 2009 | 2009 |
| Rheumatoid Arthritis | C0003873 | ZFP36 | 7538 | P26651 | ZFP36 ring finger protein | Nucleic acid binding | 161 | 0.523 | 0.692 | 0.75244 | 0.3 | | 1 | 13 | 0 | 2004 | 2019 |

| | | | | | | | | | | | | | | | | |
|---|---|---|---|---|---|---|---|---|---|---|---|---|---|---|---|---|
| Rheumatoid Arthritis | C0003873 | TFPI2 | 7980 | P48307 | tissue factor pathway inhibitor 2 | Enzyme modulator | 161 | 0.521 | 0.769 | 0.000843 | 0.3 | | 1 | 2 | 0 | 2004 | 2007 |
| Rheumatoid Arthritis | C0003873 | CALD1 | 800 | Q05682 | caldesmon 1 | | 94 | 0.578 | 0.692 | 0.99879 | 0.3 | | 1 | 1 | 0 | 2004 | 2004 |
| Rheumatoid Arthritis | C0003873 | ST6GALNAC5 | 81849 | Q9BVH7 | ST6 N-acetylgalactosaminide alpha-2,6-sialyltransferase 5 | | 23 | 0.729 | 0.346 | 0.16967 | 0.3 | | 1 | 2 | 0 | 2007 | 2007 |
| Rheumatoid Arthritis | C0003873 | MGARP | 84709 | Q8TDB4 | mitochondria localized glutamic acid rich protein | | 1 | 1 | 0.115 | 3.88E-05 | 0.3 | | 1 | 1 | 0 | 2007 | 2007 |
| Rheumatoid Arthritis | C0003873 | B3GNT9 | 84752 | Q6UX72 | UDP-GlcNAc:betaGal beta-1,3-N-acetylglucosaminyltransferase 9 | Enzyme | 1 | 1 | 0.115 | 1.74E-05 | 0.3 | | 1 | 1 | 0 | 2007 | 2007 |
| Rheumatoid Arthritis | C0003873 | RUNX2 | 860 | Q13950 | RUNX family transcription factor 2 | Transcription factor | 405 | 0.437 | 0.846 | 0.94884 | 0.3 | | 1 | 1 | 0 | 2010 | 2010 |
| Rheumatoid | C0003873 | SLC25A12 | 8604 | O75746 | solute carrier family 25 | Transporter | 34 | 0.711 | 0.462 | 0.216 | 0.3 | | 1 | 1 | 0 | 2003 | 2003 |

| | | | | | | | | | | | | | | | | | |
|---|---|---|---|---|---|---|---|---|---|---|---|---|---|---|---|---|---|
| Arthritis | | | | | member 12 | | | | | 04 | | | | | | | |
| Rheumatoid Arthritis | C0003873 | CD3E | 916 | P07766 | CD3e molecule | | 73 | 0.615 | 0.615 | 0.0003345 | 0.3 | | 1 | 1 | 0 | 2003 | 2003 |
| Rheumatoid Arthritis | C0003873 | CD5 | 921 | P06127 | CD5 molecule | | 32 | 0.682 | 0.308 | 2.31E-10 | 0.3 | | 1 | 1 | 0 | 2012 | 2012 |
| Rheumatoid Arthritis | C0003873 | ABCG2 | 9429 | Q9UNQ0 | ATP binding cassette subfamily G member 2 (Junior blood group) | Transporter | 420 | 0.419 | 0.885 | 2.1E-32 | 0.3 | | 1 | 1 | 1 | 2013 | 2013 |
| Rheumatoid Arthritis | C0003873 | MMP14 | 4323 | P50281 | matrix metallopeptidase 14 | Enzyme | 340 | 0.447 | 0.769 | 0.99522 | 0.28 | | 1 | 11 | 0 | 1998 | 2013 |
| Rheumatoid Arthritis | C0003873 | ZAP70 | 7535 | P43403 | zeta chain of T cell receptor associated protein kinase 70 | Kinase | 212 | 0.497 | 0.769 | 0.87696 | 0.24 | | 1 | 6 | 0 | 2003 | 2019 |
| Rheumatoid Arthritis | C0003873 | DNASE2 | 1777 | O00115 | deoxyribonuclease 2, lysosomal | Enzyme | 29 | 0.7 | 0.462 | 0.000452 | 0.23 | | 1 | 3 | 0 | 2009 | 2012 |

| | | | | | | | | | | | | | | | | | |
|---|---|---|---|---|---|---|---|---|---|---|---|---|---|---|---|---|---|
| Rheumatoid Arthritis | C0003873 | HNRNPA2B1 | 3181 | P22626 | heterogeneous nuclear ribonucleoprotein A2/B1 | | 131 | 0.566 | 0.808 | 0.99732 | 0.21 | | 1 | 2 | 0 | 2002 | 2007 |
| Rheumatoid Arthritis | C0003873 | CD226 | 10666 | Q15762 | CD226 molecule | | 124 | 0.548 | 0.769 | 0.03921 | 0.2 | | 0.909 | 11 | 2 | 2009 | 2018 |
| Rheumatoid Arthritis | C0003873 | PADI2 | 11240 | Q9Y2J8 | peptidyl arginine deiminase 2 | | 67 | 0.61 | 0.615 | 4.95E-11 | 0.2 | | 0.857 | 14 | 5 | 2004 | 2019 |
| Rheumatoid Arthritis | C0003873 | FCRL3 | 115352 | Q96P31 | Fc receptor like 3 | Cell adhesion | 62 | 0.612 | 0.769 | 5.97E-21 | 0.2 | | 0.857 | 28 | 4 | 2005 | 2016 |
| Rheumatoid Arthritis | C0003873 | HLA-A | 3105 | P04439 | major histocompatibility complex, class I, A | | 672 | 0.37 | 0.846 | 0.00133 | 0.2 | | 1 | 17 | 2 | 1981 | 2014 |
| Rheumatoid Arthritis | C0003873 | HLA-B | 3106 | P01889 | major histocompatibility complex, class I, B | | 706 | 0.379 | 0.885 | 3.86E-05 | 0.2 | | 1 | 16 | 45 | 1982 | 2018 |
| Rheumatoid Ar | C0003873 | HLA-C | 3107 | P10321 | major histocompatibility complex, class I, C | | 435 | 0.415 | 0.846 | 1.57E- | 0.2 | | 0.944 | 18 | 14 | 1993 | 2019 |

| | | | | | | | | | | | | | | | | | |
|---|---|---|---|---|---|---|---|---|---|---|---|---|---|---|---|---|---|
| thritis | | | | | | | | | | 06 | | | | | | | |
| Rheumatoid Arthritis | C0003873 | HLA-DQA1 | 3117 | P01909 | major histocompatibility complex, class II, DQ alpha 1 | Immune response | 427 | 0.416 | 0.846 | 0.023947 | 0.2 | | 0.962 | 26 | 8 | 1990 | 2016 |
| Rheumatoid Arthritis | C0003873 | HLA-DQB1 | 3119 | P01920 | major histocompatibility complex, class II, DQ beta 1 | Immune response | 504 | 0.407 | 0.885 | 0.008926 | 0.2 | | 1 | 14 | 4 | 1997 | 2017 |
| Rheumatoid Arthritis | C0003873 | ISG20 | 3669 | Q96AZ6 | interferon stimulated exonuclease gene 20 | | 414 | 0.414 | 0.846 | 0.002899 | 0.2 | | 1 | 17 | 1 | 1992 | 2018 |
| Rheumatoid Arthritis | C0003873 | TAP2 | 6891 | Q03519 | transporter 2, ATP binding cassette subfamily B member | Transporter | 155 | 0.517 | 0.808 | 6.89E-11 | 0.2 | | 1 | 14 | 7 | 1994 | 2016 |
| Rheumatoid Arthritis | C0003873 | DPP4 | 1803 | P27487 | dipeptidyl peptidase 4 | Enzyme | 451 | 0.42 | 0.808 | 7.28E-09 | 0.19 | | 0.9 | 10 | 1 | 2014 | 2019 |
| Rheumatoid Arthritis | C0003873 | AIRE | 326 | O43918 | autoimmune regulator | | 175 | 0.516 | 0.808 | 4.05E-12 | 0.19 | | 1 | 9 | 7 | 2011 | 2019 |
| Rheumatoi | C0003 | MICA | 1.01E | Q29983 | MHC class I polypeptide-related sequence A | | 248 | 0.4 | 0.8 | 0.004 | 0.18 | | 1 | 11 | 8 | 2001 | 2019 |

| | | | | | | | | | | | | | | | | | |
|---|---|---|---|---|---|---|---|---|---|---|---|---|---|---|---|---|---|
| d Arthritis | 873 | | +08 | | | | | 73 | 46 | 97 | | | | | | | |
| Rheumatoid Arthritis | C0003873 | HLA-DMB | 3109 | P28068 | major histocompatibility complex, class II, DM beta | Immune response | 41 | 0.638 | 0.615 | 0.002463 | 0.18 | | 0.8 | 10 | 5 | 1997 | 2009 |
| Rheumatoid Arthritis | C0003873 | ATG5 | 9474 | Q9H1Y0 | autophagy related 5 | Transporter | 282 | 0.462 | 0.885 | 0.97758 | 0.17 | | 1 | 11 | 4 | 2004 | 2019 |
| Rheumatoid Arthritis | C0003873 | AIF1 | 199 | P55008 | allograft inflammatory factor 1 | Calcium-binding protein | 166 | 0.521 | 0.808 | 1.88E-06 | 0.16 | | 1 | 8 | 5 | 2007 | 2018 |
| Rheumatoid Arthritis | C0003873 | C5 | 727 | P01031 | complement C5 | Enzyme modulator | 129 | 0.55 | 0.731 | 8.08E-24 | 0.16 | | 1 | 8 | 3 | 2007 | 2013 |
| Rheumatoid Arthritis | C0003873 | MECP2 | 4204 | P51608 | methyl-CpG binding protein 2 | Epigenetic regulator | 664 | 0.414 | 0.846 | 0.89382 | 0.15 | | 1 | 6 | 1 | 2013 | 2018 |
| Rheumatoid Arthritis | C0003873 | NOTCH4 | 4855 | Q99466 | notch receptor 4 | | 150 | 0.534 | 0.731 | 2.05E-23 | 0.15 | | 1 | 8 | 25 | 2001 | 2013 |

| | | | | | | | | | | | | | | | | | |
|---|---|---|---|---|---|---|---|---|---|---|---|---|---|---|---|---|---|
| Rheumatoid Arthritis | C0003873 | TNIP1 | 10318 | Q15025 | TNFAIP3 interacting protein 1 | | 93 | 0.564 | 0.769 | 0.47559 | 0.14 | | 1 | 5 | 1 | 2003 | 2019 |
| Rheumatoid Arthritis | C0003873 | CCR3 | 1232 | P51677 | C-C motif chemokine receptor 3 | G-protein coupled receptor | 149 | 0.527 | 0.808 | 0.00126 | 0.14 | | 1 | 5 | 1 | 1997 | 2017 |
| Rheumatoid Arthritis | C0003873 | HLA-DOA | 3111 | P06340 | major histocompatibility complex, class II, DO alpha | Immune response | 160 | 0.509 | 0.808 | 2.98E-10 | 0.14 | | 1 | 7 | 11 | 1986 | 2016 |
| Rheumatoid Arthritis | C0003873 | HLA-DPA1 | 3113 | P20036 | major histocompatibility complex, class II, DP alpha 1 | Immune response | 139 | 0.552 | 0.846 | 2.5E-06 | 0.14 | | 0.857 | 7 | 8 | 1990 | 2017 |
| Rheumatoid Arthritis | C0003873 | PTPN11 | 5781 | Q06124 | protein tyrosine phosphatase non-receptor type 11 | | 702 | 0.385 | 0.923 | 0.99998 | 0.14 | | 1 | 5 | 1 | 2003 | 2019 |
| Rheumatoid Arthritis | C0003873 | CFB | 629 | P00751 | complement factor B | | 139 | 0.532 | 0.769 | 4.67E-16 | 0.14 | | 0.857 | 7 | 7 | 1985 | 2015 |
| Rheumatoi | C0003 | SFTPD | 6441 | P35247 | surfactant protein D | Surfactant | 194 | 0.5 | 0.8 | 7.49E | 0.14 | | 0.833 | 6 | 2 | 2008 | 2019 |

| | | | | | | | | | | | | | | | | | |
|---|---|---|---|---|---|---|---|---|---|---|---|---|---|---|---|---|---|
| d Arthritis | 873 | | | | | | | | 46 | -05 | | | | | | | |
| Rheumatoid Arthritis | C0003873 | TSBP1 | 10665 | Q5SRN2 | testis expressed basic protein 1 | | 48 | 0.638 | 0.692 | 1.48E-16 | 0.13 | | 0.889 | 9 | 149 | 2007 | 2019 |
| Rheumatoid Arthritis | C0003873 | CCL27 | 10850 | Q9Y4X3 | C-C motif chemokine ligand 27 | Signaling | 185 | 0.502 | 0.846 | 0.0135111 | 0.13 | | 1 | 4 | 1 | 2003 | 2019 |
| Rheumatoid Arthritis | C0003873 | PSORS1C1 | 170679 | Q9UIG5 | psoriasis susceptibility 1 candidate 1 | | 43 | 0.656 | 0.731 | 0.000265 | 0.13 | | 1 | 5 | 11 | 2007 | 2017 |
| Rheumatoid Arthritis | C0003873 | MICB | 4277 | Q29980 | MHC class I polypeptide-related sequence B | | 95 | 0.576 | 0.731 | 4.06E-05 | 0.13 | | 1 | 4 | 1 | 2001 | 2019 |
| Rheumatoid Arthritis | C0003873 | APOM | 55937 | O95445 | apolipoprotein M | | 82 | 0.59 | 0.769 | 0.000956 | 0.13 | | 1 | 5 | 5 | 2007 | 2014 |
| Rheumatoid Arthritis | C0003873 | RAD51B | 5890 | O15315 | RAD51 paralog B | | 126 | 0.556 | 0.769 | 2.08E-15 | 0.13 | | 1 | 7 | 3 | 2013 | 2019 |

| | | | | | | | | | | | | | | | | | |
|---|---|---|---|---|---|---|---|---|---|---|---|---|---|---|---|---|---|
| Rheumatoid Arthritis | C0003873 | BACH2 | 60468 | Q9BYV9 | BTB domain and CNC homolog 2 | | 130 | 0.539 | 0.731 | 0.99803 | 0.13 | | 1 | 5 | 1 | 2012 | 2016 |
| Rheumatoid Arthritis | C0003873 | PRRC2A | 7916 | P48634 | proline rich coiled-coil 2A | Enzyme | 45 | 0.656 | 0.654 | 1 | 0.13 | | 1 | 6 | 15 | 1998 | 2011 |
| Rheumatoid Arthritis | C0003873 | DDX39B | 7919 | Q13838 | DExD-box helicase 39B | | 50 | 0.641 | 0.808 | 0.99725 | 0.13 | | 1 | 6 | 8 | 2001 | 2011 |
| Rheumatoid Arthritis | C0003873 | LST1 | 7940 | O00453 | leukocyte specific transcript 1 | | 17 | 0.792 | 0.308 | 0.00113 | 0.13 | | 1 | 6 | 2 | 2006 | 2016 |
| Rheumatoid Arthritis | C0003873 | CDSN | 1041 | Q15517 | corneodesmosin | | 101 | 0.585 | 0.731 | 0.46391 | 0.12 | | 1 | 3 | 2 | 2002 | 2013 |
| Rheumatoid Arthritis | C0003873 | RPP14 | 11102 | O95059 | ribonuclease P/MRP subunit p14 | | 117 | 0.544 | 0.808 | 0.00192 7 | 0.12 | | 1 | 5 | 1 | 1990 | 2019 |
| Rheumatoid Ar | C0003873 | ICOSLG | 23308 | O75144 | inducible T cell costimulator ligand | | 69 | 0.601 | 0.654 | 0.0003 | 0.12 | | 1 | 4 | 3 | 2003 | 2019 |

| | | | | | | | | | | | | | | | | | |
|---|---|---|---|---|---|---|---|---|---|---|---|---|---|---|---|---|---|
| thritis | | | | | | | | | | 55 | | | | | | | |
| Rheumatoid Arthritis | C0003873 | PHF19 | 26147 | Q5T6S3 | PHD finger protein 19 | Nucleic acid binding | 28 | 0.716 | 0.385 | 0.99946 | 0.12 | | 0.857 | 7 | 6 | 2007 | 2019 |
| Rheumatoid Arthritis | C0003873 | UBASH3A | 53347 | P57075 | ubiquitin associated and SH3 domain containing A | | 43 | 0.638 | 0.538 | 1.05E-10 | 0.12 | | 1 | 6 | 4 | 2014 | 2019 |
| Rheumatoid Arthritis | C0003873 | PRKCH | 5583 | P24723 | protein kinase C eta | Kinase | 55 | 0.653 | 0.577 | 0.42878 | 0.12 | | 1 | 5 | 4 | 2007 | 2019 |
| Rheumatoid Arthritis | C0003873 | GSDMB | 55876 | Q8TAX9 | gasdermin B | | 44 | 0.67 | 0.5 | 3.34E-07 | 0.12 | | 1 | 3 | 1 | 2012 | 2012 |
| Rheumatoid Arthritis | C0003873 | BTNL2 | 56244 | Q9UIR0 | butyrophilin like 2 | Enzyme modulator | 145 | 0.546 | 0.808 | 8.56E-11 | 0.12 | | 1 | 6 | 31 | 2005 | 2013 |
| Rheumatoid Arthritis | C0003873 | COG6 | 57511 | Q9Y2V7 | component of oligomeric golgi complex 6 | | 62 | 0.644 | 0.577 | 6.36E-16 | 0.12 | | 1 | 2 | 1 | 2016 | 2017 |
| Rheumatoi | C0003 | C2 | 717 | P06681 | complement C2 | | 71 | 0.6 | 0.6 | 1.35E | 0.12 | | 1 | 5 | 13 | 1976 | 2011 |

| | | | | | | | | | | | | | | | | | |
|---|---|---|---|---|---|---|---|---|---|---|---|---|---|---|---|---|---|
| d Arthritis | 873 | | | | | | | 17 | 92 | -16 | | | | | | | |
| Rheumatoid Arthritis | C0003873 | UBE2L3 | 7332 | P68036 | ubiquitin conjugating enzyme E2 L3 | Enzyme | 56 | 0.644 | 0.615 | 0.83844 | 0.12 | | 0.667 | 3 | 1 | 2011 | 2017 |
| Rheumatoid Arthritis | C0003873 | NELFE | 7936 | P18615 | negative elongation factor complex member E | | 40 | 0.686 | 0.5 | 0.0068 95 | 0.12 | | 0.667 | 3 | 8 | 1997 | 2011 |
| Rheumatoid Arthritis | C0003873 | KIAA1109 | 84162 | Q2LD37 | KIAA1109 | | 86 | 0.603 | 0.654 | 3.26E-21 | 0.12 | | 1 | 3 | 2 | 2009 | 2010 |
| Rheumatoid Arthritis | C0003873 | CASP10 | 843 | Q92851 | caspase 10 | Enzyme | 166 | 0.528 | 0.692 | 4.64E-12 | 0.12 | | 1 | 3 | 1 | 2003 | 2019 |
| Rheumatoid Arthritis | C0003873 | MACIR | 90355 | Q96GV9 | macrophage immunometabolism regulator | | 32 | 0.691 | 0.462 | 0.42746 | 0.12 | | 1 | 8 | 3 | 2010 | 2019 |
| Rheumatoid Arthritis | C0003873 | SYNGR1 | 9145 | O43759 | synaptogyrin 1 | Transporter | 17 | 0.736 | 0.346 | 0.45121 | 0.12 | | 1 | 5 | 1 | 2014 | 2019 |

| | | | | | | | | | | | | | | | | | |
|---|---|---|---|---|---|---|---|---|---|---|---|---|---|---|---|---|---|
| Rheumatoid Arthritis | C0003873 | CD247 | 919 | P20963 | CD247 molecule | | 140 | 0.539 | 0.731 | 0.0167 34 | 0.12 | | 1 | 3 | 4 | 2012 | 2016 |
| Rheumatoid Arthritis | C0003873 | COL4A1 | 1282 | P02462 | collagen type IV alpha 1 chain | | 277 | 0.484 | 0.731 | 1 | 0.11 | | 1 | 1 | 1 | 2018 | 2018 |
| Rheumatoid Arthritis | C0003873 | COL11A2 | 1302 | P13942 | collagen type XI alpha 2 chain | | 399 | 0.435 | 0.846 | 0.70197 | 0.11 | | 1 | 3 | 3 | 2007 | 2011 |
| Rheumatoid Arthritis | C0003873 | DAXX | 1616 | Q9UER7 | death domain associated protein | | 137 | 0.544 | 0.692 | 0.098633 | 0.11 | | 1 | 2 | 1 | 2007 | 2011 |
| Rheumatoid Arthritis | C0003873 | ETFA | 2108 | P13804 | electron transfer flavoprotein subunit alpha | Enzyme | 195 | 0.508 | 0.731 | 0.002922 | 0.11 | | 1 | 2 | 1 | 2013 | 2018 |
| Rheumatoid Arthritis | C0003873 | RTKN2 | 219790 | Q8IZC4 | rhotekin 2 | | 22 | 0.722 | 0.385 | 5.46E-15 | 0.11 | | 1 | 1 | 1 | 2012 | 2012 |
| Rheumatoid Ar | C0003873 | HSPA1L | 3305 | P34931 | heat shock protein family A (Hsp70) member 1 like | | 65 | 0.603 | 0.654 | 1.06E- | 0.11 | | 1 | 4 | 2 | 2000 | 2011 |

| | | | | | | | | | | | | | | | | | |
|---|---|---|---|---|---|---|---|---|---|---|---|---|---|---|---|---|---|
| thritis | | | | | | | | | | 06 | | | | | | | |
| Rheumatoid Arthritis | C0003873 | ICAM3 | 3385 | P32942 | intercellular adhesion molecule 3 | | 59 | 0.619 | 0.654 | 2.73E-25 | 0.11 | | 1 | 2 | 1 | 2003 | 2017 |
| Rheumatoid Arthritis | C0003873 | SMTNL2 | 342527 | Q2TAL5 | smoothelin like 2 | | 1 | 1 | 0.115 | 0.000238 | 0.11 | | 1 | 1 | 1 | 2017 | 2017 |
| Rheumatoid Arthritis | C0003873 | IL12RB2 | 3595 | Q99665 | interleukin 12 receptor subunit beta 2 | Signaling | 59 | 0.608 | 0.692 | 1.62E-09 | 0.11 | | 1 | 2 | 1 | 2018 | 2019 |
| Rheumatoid Arthritis | C0003873 | FADS1 | 3992 | O60427 | fatty acid desaturase 1 | | 125 | 0.582 | 0.769 | 0.37384 | 0.11 | | 1 | 4 | 1 | 2009 | 2019 |
| Rheumatoid Arthritis | C0003873 | MBP | 4155 | P02686 | myelin basic protein | | 184 | 0.51 | 0.885 | 0.28446 | 0.11 | | 1 | 1 | 1 | 2011 | 2011 |
| Rheumatoid Arthritis | C0003873 | EMCN | 51705 | Q9ULC0 | endomucin | | 25 | 0.716 | 0.423 | 8.64E-05 | 0.11 | | 1 | 2 | 2 | 2011 | 2018 |
| Rheumatoi | C0003 | ATP6V1G2 | 534 | O95670 | ATPase H+ transporting V1 | Transporter | 7 | 0.8 | 0.2 | 0.002 | 0.11 | | 1 | 3 | 3 | 2001 | 2011 |

| | | | | | | | | | | | | | | | | | |
|---|---|---|---|---|---|---|---|---|---|---|---|---|---|---|---|---|---|
| d Arthritis | 873 | | | | subunit G2 | | | 39 | 69 | 86 | | | | | | | |
| Rheumatoid Arthritis | C0003873 | SUPT20H | 55578 | Q8NEM7 | SPT20 homolog, SAGA complex component | Transcription factor | 18 | 0.751 | 0.346 | 2.73E-14 | 0.11 | | 1 | 1 | 1 | 2019 | 2019 |
| Rheumatoid Arthritis | C0003873 | PSMB8 | 5696 | P28062 | proteasome 20S subunit beta 8 | | 165 | 0.533 | 0.808 | 0.021795 | 0.11 | | 1 | 3 | 4 | 1998 | 2009 |
| Rheumatoid Arthritis | C0003873 | PSMB9 | 5698 | P28065 | proteasome 20S subunit beta 9 | | 167 | 0.514 | 0.808 | 0.00275 | 0.11 | | 1 | 2 | 3 | 1998 | 2009 |
| Rheumatoid Arthritis | C0003873 | WDFY4 | 57705 | Q6ZS81 | WDFY family member 4 | | 10 | 0.792 | 0.192 | 0.34009 | 0.11 | | 1 | 5 | 3 | 2009 | 2019 |
| Rheumatoid Arthritis | C0003873 | RARB | 5915 | P10826 | retinoic acid receptor beta | Nuclear receptor | 254 | 0.478 | 0.846 | 0.99983 | 0.11 | | 1 | 1 | 1 | 2019 | 2019 |
| Rheumatoid Arthritis | C0003873 | GPSM3 | 63940 | Q9Y4H4 | G protein signaling modulator 3 | | 17 | 0.751 | 0.423 | 0.28641 | 0.11 | | 1 | 3 | 4 | 2007 | 2016 |

| | | | | | | | | | | | | | | | | | |
|---|---|---|---|---|---|---|---|---|---|---|---|---|---|---|---|---|---|
| Rheumatoid Arthritis | C0003873 | CARD9 | 64170 | Q9H257 | caspase recruitment domain family member 9 | | 103 | 0.566 | 0.654 | 8.15E-13 | 0.11 | | 1 | 2 | 1 | 2015 | 2019 |
| Rheumatoid Arthritis | C0003873 | TNXB | 7148 | P22105 | tenascin XB | | 127 | 0.572 | 0.731 | 0.0185547 | 0.11 | | 1 | 4 | 23 | 2001 | 2011 |
| Rheumatoid Arthritis | C0003873 | TPD52 | 7163 | P55327 | tumor protein D52 | | 76 | 0.597 | 0.692 | 8.49E-05 | 0.11 | | 1 | 3 | 1 | 2001 | 2019 |
| Rheumatoid Arthritis | C0003873 | MPIG6B | 80739 | O95866 | megakaryocyte and platelet inhibitory receptor G6b | | 30 | 0.695 | 0.5 | 1.08E-06 | 0.11 | | 1 | 2 | 1 | 2000 | 2011 |
| Rheumatoid Arthritis | C0003873 | TMEM187 | 8269 | Q14656 | transmembrane protein 187 | | 8 | 0.839 | 0.231 | 0.015717 | 0.11 | | 1 | 2 | 1 | 2012 | 2017 |
| Rheumatoid Arthritis | C0003873 | MYO18B | 84700 | Q8IUG5 | myosin XVIIIB | | 63 | 0.628 | 0.615 | 1.03E-31 | 0.11 | | 1 | 2 | 1 | 2013 | 2019 |
| Rheumatoid Ar | C0003873 | NTN1 | 9423 | O95631 | netrin 1 | Enzyme modul | 209 | 0.509 | 0.846 | 0.99687 | 0.11 | | 1 | 2 | 1 | 2009 | 2009 |

| | | | | | | | | | | | | | | | | | |
|---|---|---|---|---|---|---|---|---|---|---|---|---|---|---|---|---|---|
| thritis | | | | | | ator | | | | | | | | | | | |
| Rheumatoid Arthritis | C0003873 | SH3PXD2A | 9644 | Q5TCZ1 | SH3 and PX domains 2A | | 32 | 0.729 | 0.385 | 0.98897 | 0.11 | | 1 | 2 | 1 | 2017 | 2019 |
| Rheumatoid Arthritis | C0003873 | ELMO1 | 9844 | Q92556 | engulfment and cell motility 1 | Signaling | 72 | 0.608 | 0.615 | 0.89997 | 0.11 | | 1 | 3 | 2 | 2012 | 2016 |
| Rheumatoid Arthritis | C0003873 | NAT2 | 10 | P11245 | N-acetyltransferase 2 | Enzyme | 311 | 0.451 | 0.885 | 3.27E-06 | 0.1 | | 1 | 10 | 3 | 2002 | 2012 |
| Rheumatoid Arthritis | C0003873 | ZDHHC20P2 | 1E+08 | | zinc finger DHHC-type containing 20 pseudogene 2 | | 3 | 0.89 | 0.192 | | 0.1 | | 1 | 1 | 1 | 2011 | 2011 |
| Rheumatoid Arthritis | C0003873 | TMPOP1 | 1E+08 | | thymopoietin pseudogene 1 | | 2 | 1 | 0.115 | | 0.1 | | 1 | 2 | 1 | 2007 | 2009 |
| Rheumatoid Arthritis | C0003873 | WAKMAR2 | 1E+08 | | wound and keratinocyte migration associated lncRNA 2 | | 2 | 0.931 | 0.115 | | 0.1 | | 1 | 1 | 1 | 2017 | 2017 |
| Rheumatoi | C0003 | HNRNPA1P2 | 1E+ | | heterogeneous nuclear ribonucleoprot | | 2 | 1 | 0.1 | | 0.1 | | 1 | 1 | 1 | 2011 | 2011 |

| | | | | | | | | | | | | | | | | |
|---|---|---|---|---|---|---|---|---|---|---|---|---|---|---|---|---|
| d Arthritis | 873 | | 08 | | ein A1 pseudogene 2 | | | 15 | | | | | | | | |
| Rheumatoid Arthritis | C0003873 | NONOP2 | 1E+08 | | non-POU domain containing, octamer-binding pseudogene 2 | 6 | 0.861 | 0.231 | | 0.1 | | 1 | 1 | 1 | 2009 | 2009 |
| Rheumatoid Arthritis | C0003873 | UBDP1 | 1E+08 | | ubiquitin D pseudogene 1 | 2 | 1 | 0.115 | | 0.1 | | 1 | 1 | 1 | 2009 | 2009 |
| Rheumatoid Arthritis | C0003873 | AP4B1-AS1 | 1E+08 | | AP4B1 antisense RNA 1 | 13 | 0.805 | 0.231 | | 0.1 | | 1 | 2 | 12 | 2009 | 2019 |
| Rheumatoid Arthritis | C0003873 | CYP4F23P | 1E+08 | | cytochrome P450 family 4 subfamily F member 23, pseudogene | 1 | 1 | 0.115 | | 0.1 | | 1 | 1 | 1 | 2013 | 2013 |
| Rheumatoid Arthritis | C0003873 | PSMB8-AS1 | 1.01E+08 | | PSMB8 antisense RNA 1 (head to head) | 6 | 0.839 | 0.385 | | 0.1 | | 1 | 1 | 3 | 2009 | 2009 |
| Rheumatoid Arthritis | C0003873 | MUC22 | 1.01E+08 | E2RYF6 | mucin 22 | 19 | 0.729 | 0.5 | 0.014047 | 0.1 | | 1 | 2 | 6 | 2007 | 2009 |
| Rheu | C00 | MSH5- | 1.0 | | MSH5-SAPCD1 | 21 | 0. | 0. | | 0.1 | | 1 | 3 | 10 | 2007 | 20 |

| | | | | | | | | | | | | | | | | |
|---|---|---|---|---|---|---|---|---|---|---|---|---|---|---|---|---|
| matoid Arthritis | 03873 | SAPCD1 | 1E+08 | | readthrough (NMD candidate) | | 76 | 423 | | | | | | | | 11 |
| Rheumatoid Arthritis | C0003873 | ATP6V1G2-DDX39B | 1.01E+08 | | ATP6V1G2-DDX39B readthrough (NMD candidate) | 15 | 0.769 | 0.423 | | 0.1 | | 1 | 3 | 8 | 2007 | 2011 |
| Rheumatoid Arthritis | C0003873 | PPT2-EGFL8 | 1.01E+08 | | PPT2-EGFL8 readthrough (NMD candidate) | 17 | 0.792 | 0.462 | | 0.1 | | 1 | 3 | 6 | 2007 | 2011 |
| Rheumatoid Arthritis | C0003873 | CDH11 | 1009 | P55287 | cadherin 11 | 203 | 0.512 | 0.808 | 0.9999 | 0.1 | | 0.917 | 12 | 0 | 2004 | 2019 |
| Rheumatoid Arthritis | C0003873 | TSPAN5 | 10098 | P62079 | tetraspanin 5 | 9 | 0.78 | 0.308 | 0.90951 | 0.1 | | 1 | 1 | 1 | 2013 | 2013 |
| Rheumatoid Arthritis | C0003873 | TRIM10 | 10107 | Q9UDY6 | tripartite motif containing 10 | 14 | 0.821 | 0.269 | 3.74E-09 | 0.1 | | 1 | 2 | 3 | 2007 | 2009 |
| Rheumatoid Arthritis | C0003873 | LINC01934 | 1.02E+08 | | long intergenic non-protein coding RNA 1934 | 11 | 0.821 | 0.308 | | 0.1 | | 1 | 1 | 1 | 2016 | 2016 |

| | | | | | | | | | | | | | | | | |
|---|---|---|---|---|---|---|---|---|---|---|---|---|---|---|---|---|
| Rheumatoid Arthritis | C0003873 | LNCPRESS1 | 1.02E+08 | | lncRNA p53 regulated and ESC associated 1 | 1 | 1 | 0.115 | | 0.1 | | 1 | 1 | 1 | 2019 | 2019 |
| Rheumatoid Arthritis | C0003873 | LINC00824 | 1.02E+08 | | long intergenic non-protein coding RNA 824 | 24 | 0.695 | 0.346 | | 0.1 | | 1 | 3 | 2 | 2014 | 2019 |
| Rheumatoid Arthritis | C0003873 | TSBP1-AS1 | 1.02E+08 | | TSBP1 and BTNL2 antisense RNA 1 | 63 | 0.638 | 0.692 | | 0.1 | | 1 | 6 | 221 | 2007 | 2014 |
| Rheumatoid Arthritis | C0003873 | C3orf67-AS1 | 1.02E+08 | | C3orf67 antisense RNA 1 | 3 | 0.89 | 0.154 | | 0.1 | | 1 | 1 | 1 | 2019 | 2019 |
| Rheumatoid Arthritis | C0003873 | C2-AS1 | 1.02E+08 | | C2 antisense RNA 1 | 10 | 0.821 | 0.192 | | 0.1 | | 1 | 3 | 5 | 2007 | 2011 |
| Rheumatoid Arthritis | C0003873 | HCG9 | 10255 | | HLA complex group 9 | 22 | 0.7 | 0.5 | | 0.1 | | 1 | 2 | 2 | 2007 | 2009 |
| Rheumatoid Ar | C0003873 | LOC102723407 | 1.03E+ | | immunoglobulin heavy variable 4-38-2-like | 159 | 0.514 | 0.769 | | 0.1 | | 1 | 10 | 0 | 1993 | 2017 |

| | | | | | | | | | | | | | | | | | |
|---|---|---|---|---|---|---|---|---|---|---|---|---|---|---|---|---|---|
| thritis | | | 08 | | | | | | | | | | | | | | |
| Rheumatoid Arthritis | C0003873 | ASB15-AS1 | 1.03E+08 | | ASB15 antisense RNA 1 | | 1 | 1 | 0.115 | | 0.1 | | 1 | 1 | 1 | 2009 | 2009 |
| Rheumatoid Arthritis | C0003873 | LOC102724971 | 1.03E+08 | | putative V-set and immunoglobulin domain-containing-like protein IGHV4OR15-8 | | 139 | 0.527 | 0.769 | | 0.1 | | 1 | 10 | 0 | 1993 | 2017 |
| Rheumatoid Arthritis | C0003873 | STAG1 | 1.0274 | Q8WVM7 | stromal antigen 1 | Nucleic acid binding | 56 | 0.656 | 0.577 | 1 | 0.1 | | 1 | 2 | 1 | 2014 | 2019 |
| Rheumatoid Arthritis | C0003873 | NEBL | 1.0529 | O76041 | nebulette | | 48 | 0.65 | 0.692 | 1.77E-36 | 0.1 | | 1 | 1 | 1 | 2018 | 2018 |
| Rheumatoid Arthritis | C0003873 | LINC02356 | 1.05E+08 | | long intergenic non-protein coding RNA 2356 | | 7 | 0.931 | 0.115 | | 0.1 | | 1 | 2 | 1 | 2014 | 2019 |
| Rheumatoid Arthritis | C0003873 | LINC02357 | 1.05E+08 | | long intergenic non-protein coding RNA 2357 | | 10 | 0.805 | 0.231 | | 0.1 | | 1 | 3 | 4 | 2012 | 2015 |
| Rheumatoi | C0003 | LINC02571 | 1.05E | | long intergenic non-protein | | 28 | 0.7 | 0.5 | | 0.1 | | 1 | 2 | 9 | 2007 | 2011 |

| | | | | | | | | | | | | | | | | | |
|---|---|---|---|---|---|---|---|---|---|---|---|---|---|---|---|---|---|
| d Arthritis | 873 | | +08 | | coding RNA 2571 | | | 43 | | | | | | | | | |
| Rheumatoid Arthritis | C0003873 | ETV7-AS1 | 1.05E+08 | | ETV7 and PTX1 antisense RNA 1 | | 2 | 0.931 | 0.115 | | 0.1 | | 1 | 2 | 1 | 2014 | 2019 |
| Rheumatoid Arthritis | C0003873 | LINC01748 | 1.05E+08 | | long intergenic non-protein coding RNA 1748 | | 3 | 0.89 | 0.154 | | 0.1 | | 1 | 1 | 1 | 2013 | 2013 |
| Rheumatoid Arthritis | C0003873 | AGPAT1 | 10554 | Q99943 | 1-acylglycerol-3-phosphate O-acyltransferase 1 | | 18 | 0.743 | 0.423 | 0.97215 | 0.1 | | 1 | 1 | 10 | 2011 | 2011 |
| Rheumatoid Arthritis | C0003873 | CXCL13 | 10563 | O43927 | C-X-C motif chemokine ligand 13 | Signaling | 246 | 0.472 | 0.731 | 0.82566 | 0.1 | | 1 | 13 | 0 | 2001 | 2020 |
| Rheumatoid Arthritis | C0003873 | AHSA1 | 10598 | O95433 | activator of HSP90 ATPase activity 1 | | 526 | 0.396 | 0.923 | 0.99896 | 0.1 | | 1 | 23 | 1 | 2003 | 2019 |
| Rheumatoid Arthritis | C0003873 | CDC42EP3 | 10602 | Q9UKI2 | CDC42 effector protein 3 | | 24 | 0.722 | 0.423 | 0.18562 | 0.1 | | 1 | 1 | 1 | 2019 | 2019 |
| Rheu | C00 | EHMT | 1.0 | | EHMT2 and SLC44A4 | | 14 | 0. | 0. | | 0.1 | | 1 | 3 | 4 | 2007 | 20 |

| | | | | | | | | | | | | | | | | |
|---|---|---|---|---|---|---|---|---|---|---|---|---|---|---|---|---|
| matoid Arthritis | 03873 | 2-AS1 | 6E+08 | | antisense RNA 1 | | 78 | 462 | | | | | | | | 11 |
| Rheumatoid Arthritis | C0003873 | MDC1-AS1 | 1.06E+08 | | MDC1 antisense RNA 1 | 16 | 0.743 | 0.308 | | 0.1 | | 1 | 1 | 1 | 2009 | 2009 |
| Rheumatoid Arthritis | C0003873 | CELF2 | 10659 | O95319 | CUGBP Elav-like family member 2 | 58 | 0.628 | 0.692 | 0.99965 | 0.1 | | 1 | 1 | 1 | 2019 | 2019 |
| Rheumatoid Arthritis | C0003873 | TNFSF13B | 10673 | Q9Y275 | TNF superfamily member 13b | 282 | 0.46 | 0.731 | 0.98193 | 0.1 | | 1 | 28 | 0 | 2002 | 2019 |
| Rheumatoid Arthritis | C0003873 | MTCO2P12 | 1.07E+08 | | MT-CO2 pseudogene 12 | 703 | 0.368 | 0.962 | | 0.1 | | 1 | 25 | 0 | 1994 | 2019 |
| Rheumatoid Arthritis | C0003873 | PHTF1 | 10745 | Q9UMS5 | putative homeodomain transcription factor 1<br>Transcription factor | 2 | 0.931 | 0.192 | 9.69E-26 | 0.1 | | 1 | 1 | 3 | 2009 | 2009 |
| Rheumatoid Arthritis | C0003873 | LINC02196 | 1.08E+08 | | long intergenic non-protein coding RNA 2196 | 3 | 0.931 | 0.231 | | 0.1 | | 1 | 1 | 2 | 2013 | 2013 |

| | | | | | | | | | | | | | | | | | |
|---|---|---|---|---|---|---|---|---|---|---|---|---|---|---|---|---|---|
| Rheumatoid Arthritis | C0003873 | HCP5 | 10866 | Q6MZN7 | HLA complex P5 | | 79 | 0.595 | 0.808 | | 0.1 | | 1 | 2 | 5 | 2007 | 2009 |
| Rheumatoid Arthritis | C0003873 | PAPOLA | 10914 | P51003 | poly(A) polymerase alpha | Nucleic acid binding | 79 | 0.593 | 0.731 | 0.99998 | 0.1 | | 1 | 1 | 1 | 2009 | 2009 |
| Rheumatoid Arthritis | C0003873 | EHMT2 | 10919 | Q96KQ7 | euchromatic histone lysine methyltransferase 2 | Epigenetic regulator | 86 | 0.59 | 0.808 | 0.000103 | 0.1 | | 1 | 3 | 6 | 2007 | 2011 |
| Rheumatoid Arthritis | C0003873 | OS9 | 10956 | Q13438 | OS9 endoplasmic reticulum lectin | | 5 | 0.931 | 0.154 | 2.86E-09 | 0.1 | | 1 | 3 | 2 | 2014 | 2019 |
| Rheumatoid Arthritis | C0003873 | HTD2 | 1.1E+08 | P86397 | hydroxyacyl-thioester dehydratase type 2 | | 2 | 1 | 0.115 | | 0.1 | | 1 | 3 | 1 | 2014 | 2019 |
| Rheumatoid Arthritis | C0003873 | TRIM31 | 11074 | Q9BZY9 | tripartite motif containing 31 | | 61 | 0.626 | 0.577 | 4.32E-06 | 0.1 | | 1 | 2 | 4 | 2007 | 2009 |
| Rheumatoid Ar | C0003873 | CHI3L1 | 1116 | P36222 | chitinase 3 like 1 | | 420 | 0.419 | 0.885 | 1.11E- | 0.1 | | 0.944 | 18 | 1 | 1993 | 2019 |

| | | | | | | | | | | | | | | | | | |
|---|---|---|---|---|---|---|---|---|---|---|---|---|---|---|---|---|---|
| thritis | | | | | | | | | | 05 | | | | | | | |
| Rheumatoid Arthritis | C0003873 | FAM107A | 11170 | O95990 | family with sequence similarity 107 member A | | 36 | 0.674 | 0.462 | 8.28E-07 | 0.1 | | 1 | 3 | 1 | 2010 | 2012 |
| Rheumatoid Arthritis | C0003873 | NRM | 11270 | Q8IXM6 | nurim | | 16 | 0.76 | 0.231 | 0.021828 | 0.1 | | 1 | 1 | 1 | 2007 | 2007 |
| Rheumatoid Arthritis | C0003873 | IL17F | 112744 | Q96PD4 | interleukin 17F | | 236 | 0.48 | 0.808 | 0.039165 | 0.1 | | 0.867 | 15 | 2 | 2009 | 2020 |
| Rheumatoid Arthritis | C0003873 | SMIM40 | 1.14E+08 | Q5STR5 | small integral membrane protein 40 | | 1 | 1 | 0.115 | | 0.1 | | 1 | 1 | 2 | 2011 | 2011 |
| Rheumatoid Arthritis | C0003873 | NLRP3 | 114548 | Q96P20 | NLR family pyrin domain containing 3 | | 805 | 0.361 | 0.962 | 0.000811 | 0.1 | | 0.87 | 23 | 2 | 2004 | 2020 |
| Rheumatoid Arthritis | C0003873 | CSMD3 | 114788 | Q7Z407 | CUB and Sushi multiple domains 3 | | 31 | 0.695 | 0.5 | 0.057105 | 0.1 | | 1 | 1 | 1 | 2018 | 2018 |
| Rheumatoi | C0003 | PHACTR3 | 1161 | Q96KR7 | phosphatase and actin | Enzyme mo | 6 | 0.8 | 0.2 | 0.996 | 0.1 | | 1 | 3 | 2 | 2007 | 2019 |

| | | | | | | | | | | | | | | | | | |
|---|---|---|---|---|---|---|---|---|---|---|---|---|---|---|---|---|---|
| d Arthritis | 873 | | 54 | | regulator 3 | dulator | | 61 | 31 | 87 | | | | | | | |
| Rheumatoid Arthritis | C0003873 | RPL3P2 | 116935 | | ribosomal protein L3 pseudogene 2 | | 1 | 1 | 0.115 | | 0.1 | | 1 | 1 | 2 | 2011 | 2011 |
| Rheumatoid Arthritis | C0003873 | IP6K3 | 117283 | Q96PC2 | inositol hexakisphosphate kinase 3 | Kinase | 10 | 0.839 | 0.231 | 3.09E-07 | 0.1 | | 1 | 3 | 8 | 2007 | 2011 |
| Rheumatoid Arthritis | C0003873 | CLIC1 | 1192 | O00299 | chloride intracellular channel 1 | Ion channel | 74 | 0.601 | 0.5 | 0.000193 | 0.1 | | 1 | 1 | 2 | 2011 | 2011 |
| Rheumatoid Arthritis | C0003873 | CCR5 | 1234 | P51681 | C-C motif chemokine receptor 5 (gene/pseudogene) | G-protein coupled receptor | 508 | 0.398 | 0.885 | 4.31E-10 | 0.1 | | 0.967 | 30 | 0 | 1998 | 2019 |
| Rheumatoid Arthritis | C0003873 | ZPBP2 | 124626 | Q6X784 | zona pellucida binding protein 2 | | 20 | 0.729 | 0.308 | 1.22E-08 | 0.1 | | 1 | 1 | 1 | 2019 | 2019 |
| Rheumatoid Arthritis | C0003873 | RAVER1 | 125950 | Q8IY67 | ribonucleoprotein, PTB binding 1 | Nucleic acid binding | 9 | 0.805 | 0.231 | 0.91101 | 0.1 | | 1 | 1 | 1 | 2012 | 2012 |

| | | | | | | | | | | | | | | | | | |
|---|---|---|---|---|---|---|---|---|---|---|---|---|---|---|---|---|---|
| Rheumatoid Arthritis | C0003873 | CNTFR | 1271 | P26992 | ciliary neurotrophic factor receptor | Signaling | 19 | 0.722 | 0.385 | 0.95549 | 0.1 | | 1 | 1 | 1 | 2009 | 2009 |
| Rheumatoid Arthritis | C0003873 | RBM45 | 129831 | Q8IUH3 | RNA binding motif protein 45 | | 557 | 0.383 | 0.846 | 2.11E-10 | 0.1 | | 0.89 | 200 | 1 | 1989 | 2019 |
| Rheumatoid Arthritis | C0003873 | FLACC1 | 130540 | Q96Q35 | flagellum associated containing coiled-coil domains 1 | | 18 | 0.729 | 0.269 | 1.31E-22 | 0.1 | | 1 | 2 | 2 | 2014 | 2019 |
| Rheumatoid Arthritis | C0003873 | TRIM40 | 135644 | Q6P9F5 | tripartite motif containing 40 | | 6 | 0.89 | 0.192 | 6.17E-11 | 0.1 | | 1 | 2 | 3 | 2007 | 2009 |
| Rheumatoid Arthritis | C0003873 | MUCL3 | 135656 | Q3MIW9 | mucin like 3 | | 32 | 0.691 | 0.731 | 1.23E-05 | 0.1 | | 1 | 2 | 3 | 2007 | 2009 |
| Rheumatoid Arthritis | C0003873 | SGCZ | 137868 | Q96LD1 | sarcoglycan zeta | Cellular structure | 22 | 0.751 | 0.385 | 6.4E-09 | 0.1 | | 1 | 1 | 1 | 2019 | 2019 |
| Rheumatoid Ar | C0003873 | ATF6B | 1388 | Q99941 | activating transcription factor 6 beta | | 14 | 0.839 | 0.192 | 3.1E-07 | 0.1 | | 1 | 3 | 2 | 2007 | 2011 |

| | | | | | | | | | | | | | | | | | |
|---|---|---|---|---|---|---|---|---|---|---|---|---|---|---|---|---|---|
| thritis | | | | | | | | | | | | | | | | | |
| Rheumatoid Arthritis | C0003873 | CRH | 1392 | P06850 | corticotropin releasing hormone | Signaling | 402 | 0.439 | 0.808 | 0.71614 | 0.1 | | 0.923 | 13 | 0 | 1993 | 2012 |
| Rheumatoid Arthritis | C0003873 | CRK | 1398 | P46108 | CRK proto-oncogene, adaptor protein | | 544 | 0.394 | 0.923 | 0.95936 | 0.1 | | 1 | 23 | 0 | 2003 | 2019 |
| Rheumatoid Arthritis | C0003873 | CRYGD | 1421 | P07320 | crystallin gamma D | | 168 | 0.51 | 0.808 | 0.000303 | 0.1 | | 0.927 | 123 | 0 | 2005 | 2020 |
| Rheumatoid Arthritis | C0003873 | ASB15 | 142685 | Q8WXK1 | ankyrin repeat and SOCS box containing 15 | | 9 | 0.792 | 0.231 | 1.84E-15 | 0.1 | | 1 | 1 | 1 | 2009 | 2009 |
| Rheumatoid Arthritis | C0003873 | MAPK14 | 1432 | Q16539 | mitogen-activated protein kinase 14 | Kinase | 626 | 0.379 | 0.923 | 0.37466 | 0.1 | | 1 | 30 | 0 | 2000 | 2019 |
| Rheumatoid Arthritis | C0003873 | DRAIC | 145837 | | downregulated RNA in cancer, inhibitor of cell invasion and migration | | 21 | 0.736 | 0.423 | | 0.1 | | 1 | 4 | 1 | 2012 | 2019 |
| Rheumatoi | C0003 | CSNK2B | 1460 | P67870 | casein kinase 2 beta | Kinase | 34 | 0.6 | 0.5 | 0.917 | 0.1 | | 1 | 1 | 4 | 2011 | 2011 |

| | | | | | | | | | | | | | | | | | |
|---|---|---|---|---|---|---|---|---|---|---|---|---|---|---|---|---|---|
| d Arthritis | 873 | | | | | | | 86 | 38 | 28 | | | | | | | |
| Rheumatoid Arthritis | C0003873 | IL34 | 146433 | Q6ZMJ4 | interleukin 34 | | 103 | 0.56 | 0.769 | 0.55444 | 0.1 | | 1 | 15 | 0 | 2012 | 2020 |
| Rheumatoid Arthritis | C0003873 | CSTF3 | 1479 | Q12996 | cleavage stimulation factor subunit 3 | Nucleic acid binding | 6 | 0.89 | 0.154 | 0.98279 | 0.1 | | 1 | 1 | 1 | 2013 | 2013 |
| Rheumatoid Arthritis | C0003873 | IL23R | 149233 | Q5VWK5 | interleukin 23 receptor | Signaling | 306 | 0.466 | 0.769 | 0.00056 | 0.1 | | 0.75 | 20 | 8 | 2007 | 2020 |
| Rheumatoid Arthritis | C0003873 | LINC01104 | 150577 | | long intergenic non-protein coding RNA 1104 | | 9 | 0.805 | 0.115 | | 0.1 | | 1 | 2 | 2 | 2014 | 2019 |
| Rheumatoid Arthritis | C0003873 | ZNF595 | 152687 | Q8IYB9 | zinc finger protein 595 | | 1 | 1 | 0.115 | 0.3055 | 0.1 | | 1 | 1 | 1 | 2018 | 2018 |
| Rheumatoid Arthritis | C0003873 | ERICH1 | 157697 | Q86X53 | glutamate rich 1 | | 2 | 0.931 | 0.192 | 1.14E-14 | 0.1 | | 1 | 1 | 1 | 2019 | 2019 |

| | | | | | | | | | | | | | | | | | |
|---|---|---|---|---|---|---|---|---|---|---|---|---|---|---|---|---|---|
| Rheumatoid Arthritis | C0003873 | CYP21A2 | 1589 | P08686 | cytochrome P450 family 21 subfamily A member 2 | | 166 | 0.525 | 0.769 | 0.00026 | 0.1 | | 1 | 3 | 46 | 2007 | 2011 |
| Rheumatoid Arthritis | C0003873 | DGKQ | 1609 | P52824 | diacylglycerol kinase theta | Kinase | 17 | 0.736 | 0.423 | 1.76E-09 | 0.1 | | 1 | 1 | 1 | 2019 | 2019 |
| Rheumatoid Arthritis | C0003873 | MDGA2 | 161357 | Q7Z553 | MAM domain containing glycosylphosphatidylinositol anchor 2 | | 24 | 0.76 | 0.308 | 0.66457 | 0.1 | | 1 | 1 | 1 | 2013 | 2013 |
| Rheumatoid Arthritis | C0003873 | ZNF679 | 168417 | Q8IYX0 | zinc finger protein 679 | | 1 | 1 | 0.115 | 0.17547 | 0.1 | | 1 | 1 | 1 | 2019 | 2019 |
| Rheumatoid Arthritis | C0003873 | CLYBL | 171425 | Q8N0X4 | citrate lyase beta like | | 18 | 0.743 | 0.5 | 1.64E-09 | 0.1 | | 1 | 1 | 1 | 2015 | 2015 |
| Rheumatoid Arthritis | C0003873 | DLG2 | 1740 | Q15700 | discs large MAGUK scaffold protein 2 | Receptor | 44 | 0.67 | 0.577 | 0.70796 | 0.1 | | 1 | 1 | 1 | 2018 | 2018 |
| Rheumatoid Ar | C0003873 | EGFR | 1956 | P00533 | epidermal growth factor receptor | Kinase | 1394 | 0.295 | 0.885 | 0.36837 | 0.1 | | 1 | 10 | 3 | 2005 | 2018 |

| | | | | | | | | | | | | | | | | | |
|---|---|---|---|---|---|---|---|---|---|---|---|---|---|---|---|---|---|
| thritis | | | | | | | | | | | | | | | | | |
| Rheumatoid Arthritis | C0003873 | C3orf67 | 200844 | Q6ZVT6 | chromosome 3 open reading frame 67 | Enzyme | 3 | 0.89 | 0.192 | 9.4E-11 | 0.1 | | 1 | 1 | 1 | 2019 | 2019 |
| Rheumatoid Arthritis | C0003873 | TRIM39-RPP21 | 202658 | | TRIM39-RPP21 readthrough | | 6 | 0.821 | 0.269 | 0.74425 | 0.1 | | 1 | 1 | 1 | 2009 | 2009 |
| Rheumatoid Arthritis | C0003873 | EPHB2 | 2048 | P29323 | EPH receptor B2 | Kinase | 649 | 0.374 | 0.846 | 0.99997 | 0.1 | | 0.923 | 13 | 0 | 2007 | 2020 |
| Rheumatoid Arthritis | C0003873 | AKT1 | 207 | P31749 | AKT serine/threonine kinase 1 | Kinase | 1250 | 0.311 | 0.962 | 0.9759 | 0.1 | | 0.938 | 16 | 0 | 2007 | 2019 |
| Rheumatoid Arthritis | C0003873 | ESR1 | 2099 | P03372 | estrogen receptor 1 | Nuclear receptor | 1101 | 0.324 | 0.962 | 0.9992 | 0.1 | | 0.977 | 44 | 0 | 2000 | 2020 |
| Rheumatoid Arthritis | C0003873 | ALB | 213 | P02768 | albumin | Transporter | 1198 | 0.317 | 0.962 | 0.64329 | 0.1 | | 0.95 | 20 | 0 | 1996 | 2020 |
| Rheumatoi | C0003 | FCGR3A | 2214 | P08637 | Fc fragment of IgG | Cell ad | 352 | 0.4 | 0.9 | 3.7E- | 0.1 | | 0.909 | 33 | 3 | 1990 | 2019 |

| | | | | | | | | | | | | | | | | | |
|---|---|---|---|---|---|---|---|---|---|---|---|---|---|---|---|---|---|
| d Arthritis | 873 | | | | receptor IIIa | hesion | | 32 | 62 | 06 | | | | | | | |
| Rheumatoid Arthritis | C0003873 | FCGR3B | 2215 | O75015 | Fc fragment of IgG receptor IIIb | Cell adhesion | 291 | 0.452 | 0.962 | 3.03E-07 | 0.1 | | 0.923 | 26 | 2 | 1990 | 2019 |
| Rheumatoid Arthritis | C0003873 | JAZF1 | 221895 | Q86VZ6 | JAZF zinc finger 1 | | 102 | 0.582 | 0.654 | 0.895582 | 0.1 | | 1 | 1 | 1 | 2014 | 2014 |
| Rheumatoid Arthritis | C0003873 | NKAPL | 222698 | Q5M9Q1 | NFKB activating protein like | | 4 | 0.89 | 0.231 | 9.22E-05 | 0.1 | | 1 | 1 | 1 | 2009 | 2009 |
| Rheumatoid Arthritis | C0003873 | FGF2 | 2247 | P09038 | fibroblast growth factor 2 | Signaling | 635 | 0.383 | 0.923 | 0.0184 | 0.1 | | 1 | 11 | 0 | 1992 | 2018 |
| Rheumatoid Arthritis | C0003873 | GPC5 | 2262 | P78333 | glypican 5 | | 65 | 0.626 | 0.654 | 3.99E-10 | 0.1 | | 1 | 1 | 1 | 2019 | 2019 |
| Rheumatoid Arthritis | C0003873 | DKK1 | 22943 | O94907 | dickkopf WNT signaling pathway inhibitor 1 | | 372 | 0.439 | 0.885 | 0.17423 | 0.1 | | 1 | 16 | 0 | 2008 | 2019 |

| | | | | | | | | | | | | | | | | | |
|---|---|---|---|---|---|---|---|---|---|---|---|---|---|---|---|---|---|
| Rheumatoid Arthritis | C0003873 | ABCF1 | 23 | Q8NE71 | ATP binding cassette subfamily F member 1 | Enzyme | 31 | 0.674 | 0.538 | 1.28E-09 | 0.1 | | 1 | 2 | 1 | 2007 | 2009 |
| Rheumatoid Arthritis | C0003873 | PLCL2 | 23228 | Q9UPR0 | phospholipase C like 2 | Enzyme | 30 | 0.743 | 0.308 | 0.99999 | 0.1 | | 1 | 2 | 1 | 2014 | 2019 |
| Rheumatoid Arthritis | C0003873 | PDS5A | 23244 | Q29RF7 | PDS5 cohesin associated factor A | Nucleic acid binding | 16 | 0.792 | 0.346 | 1 | 0.1 | | 1 | 1 | 2 | 2009 | 2009 |
| Rheumatoid Arthritis | C0003873 | FN1 | 2335 | P02751 | fibronectin 1 | Signaling | 724 | 0.365 | 0.962 | 0.001405 | 0.1 | | 1 | 18 | 0 | 1992 | 2019 |
| Rheumatoid Arthritis | C0003873 | SIRT1 | 23411 | Q96EB6 | sirtuin 1 | Epigenetic regulator | 675 | 0.378 | 0.885 | 0.087326 | 0.1 | | 0.938 | 16 | 0 | 2011 | 2020 |
| Rheumatoid Arthritis | C0003873 | TNPO3 | 23534 | Q9Y5L0 | transportin 3 | Transporter | 66 | 0.663 | 0.577 | 1.75E-06 | 0.1 | | 1 | 2 | 4 | 2017 | 2019 |
| Rheumatoid Ar | C0003873 | SDF2L1 | 23753 | Q9HCN8 | stromal cell derived factor 2 like 1 | | 12 | 0.805 | 0.385 | 0.189557 | 0.1 | | 1 | 1 | 1 | 2019 | 2019 |

| | | | | | | | | | | | | | | | | | |
|---|---|---|---|---|---|---|---|---|---|---|---|---|---|---|---|---|---|
| thritis | | | | | | | | | | | | | | | | | |
| Rheumatoid Arthritis | C0003873 | IL27 | 246778 | Q8NEV9 | interleukin 27 | | 286 | 0.457 | 0.808 | 0.78232 | 0.1 | | 1 | 10 | 0 | 2010 | 2019 |
| Rheumatoid Arthritis | C0003873 | HCG27 | 253018 | | HLA complex group 27 | | 14 | 0.805 | 0.346 | 0.023063 | 0.1 | | 1 | 3 | 4 | 2007 | 2011 |
| Rheumatoid Arthritis | C0003873 | WDR27 | 253769 | A2RRH5 | WD repeat domain 27 | | 4 | 0.931 | 0.192 | 3.8E-17 | 0.1 | | 1 | 1 | 1 | 2016 | 2016 |
| Rheumatoid Arthritis | C0003873 | GABBR1 | 2550 | Q9UBS5 | gamma-aminobutyric acid type B receptor subunit 1 | G-protein coupled receptor | 82 | 0.587 | 0.769 | 0.99991 | 0.1 | | 1 | 1 | 6 | 2009 | 2009 |
| Rheumatoid Arthritis | C0003873 | ASF1A | 25842 | Q9Y294 | anti-silencing function 1A histone chaperone | Nucleic acid binding | 36 | 0.67 | 0.615 | 0.80542 | 0.1 | | 1 | 10 | 0 | 2002 | 2020 |
| Rheumatoid Arthritis | C0003873 | RNF19A | 25897 | Q9NV58 | ring finger protein 19A, RBR E3 ubiquitin protein ligase | Enzyme | 523 | 0.397 | 0.923 | 0.025305 | 0.1 | | 1 | 23 | 0 | 2003 | 2019 |

| | | | | | | | | | | | | | | | | | |
|---|---|---|---|---|---|---|---|---|---|---|---|---|---|---|---|---|---|
| Rheumatoid Arthritis | C0003873 | RCHY1 | 25898 | Q96PM5 | ring finger and CHY zinc finger domain containing 1 | Enzyme | 26 | 0.716 | 0.462 | 0.0662203 | 0.1 | | 1 | 1 | 1 | 2013 | 2013 |
| Rheumatoid Arthritis | C0003873 | LY6G6F | 259215 | Q5SQ64 | lymphocyte antigen 6 family member G6F | | 5 | 0.861 | 0.308 | 9.56E-07 | 0.1 | | 1 | 2 | 3 | 2007 | 2009 |
| Rheumatoid Arthritis | C0003873 | MAGI3 | 260425 | Q5TCQ9 | membrane associated guanylate kinase, WW and PDZ domain containing 3 | | 17 | 0.751 | 0.346 | 0.02978 | 0.1 | | 1 | 1 | 1 | 2009 | 2009 |
| Rheumatoid Arthritis | C0003873 | POLDIP2 | 26073 | Q9Y2S7 | DNA polymerase delta interacting protein 2 | | 530 | 0.396 | 0.923 | 0.41407 | 0.1 | | 1 | 23 | 0 | 2003 | 2019 |
| Rheumatoid Arthritis | C0003873 | FBXW8 | 26259 | Q8N3Y1 | F-box and WD repeat domain containing 8 | | 18 | 0.743 | 0.385 | 5.24E-16 | 0.1 | | 1 | 1 | 1 | 2019 | 2019 |
| Rheumatoid Arthritis | C0003873 | GCH1 | 2643 | P30793 | GTP cyclohydrolase 1 | Enzyme | 254 | 0.511 | 0.846 | 0.90292 | 0.1 | | | 0 | 0 | | |
| Rheumatoid | C0003873 | GEM | 2669 | P55040 | GTP binding protein overexpressed in skeletal muscle | | 251 | 0.467 | 0.769 | 1.18E- | 0.1 | | 0.9 | 10 | 0 | 2006 | 2019 |

| | | | | | | | | | | | | | | | | |
|---|---|---|---|---|---|---|---|---|---|---|---|---|---|---|---|---|
| Arthritis | | | | | | | | | 08 | | | | | | | |
| Rheumatoid Arthritis | C0003873 | HLA-S | 267015 | | major histocompatibility complex, class I, S (pseudogene) | 2 | 0.931 | 0.192 | | 0.1 | | 1 | 3 | 1 | 2007 | 2011 |
| Rheumatoid Arthritis | C0003873 | GFRA1 | 2674 | P56159 | GDNF family receptor alpha 1 | 73 | 0.619 | 0.615 | 0.1175 | 0.1 | | 1 | 1 | 1 | 2016 | 2016 |
| Rheumatoid Arthritis | C0003873 | DKK3 | 27122 | Q9UBP4 | dickkopf WNT signaling pathway inhibitor 3 | 186 | 0.5 | 0.731 | 4.1E-06 | 0.1 | | 1 | 1 | 1 | 2013 | 2013 |
| Rheumatoid Arthritis | C0003873 | IL37 | 27178 | Q9NZH6 | interleukin 37 | 280 | 0.459 | 0.846 | 0.000123 | 0.1 | | 1 | 12 | 1 | 2007 | 2019 |
| Rheumatoid Arthritis | C0003873 | IL17B | 27190 | Q9UHF5 | interleukin 17B | 116 | 0.553 | 0.769 | 0.033631 | 0.1 | | 0.923 | 13 | 0 | 2006 | 2019 |
| Rheumatoid Arthritis | C0003873 | HPGDS | 27306 | O60760 | hematopoietic prostaglandin D synthase | 570 | 0.388 | 0.923 | 1.81E-06 | 0.1 | | 1 | 10 | 0 | 1999 | 2019 |
| Rheuma | C0003 | GNL1 | 27 | P36915 | G protein nucleolar 1 (putative) | 11 | 0.8 | 0.4 | 0.99 | 0.1 | | 1 | 2 | 1 | 2007 | 20 |

| | | | | | | | | | | | | | | | | | |
|---|---|---|---|---|---|---|---|---|---|---|---|---|---|---|---|---|---|
| toid Arthritis | 873 | | 94 | | | | | 05 | 23 | 928 | | | | | | | 09 |
| Rheumatoid Arthritis | C0003873 | GPI | 2821 | P06744 | glucose-6-phosphate isomerase | Enzyme | 218 | 0.491 | 0.769 | 2.84E-06 | 0.1 | | 1 | 16 | 0 | 2002 | 2020 |
| Rheumatoid Arthritis | C0003873 | LINC00294 | 283267 | | long intergenic non-protein coding RNA 294 | | 1 | 1 | 0.115 | | 0.1 | | 1 | 1 | 1 | 2013 | 2013 |
| Rheumatoid Arthritis | C0003873 | CXCR3 | 2833 | P49682 | C-X-C motif chemokine receptor 3 | G-protein coupled receptor | 367 | 0.436 | 0.808 | 0.181 | 0.1 | | 1 | 11 | 0 | 2001 | 2018 |
| Rheumatoid Arthritis | C0003873 | HECTD4 | 283450 | Q9Y4D8 | HECT domain E3 ubiquitin protein ligase 4 | | 55 | 0.7 | 0.462 | 1 | 0.1 | | 1 | 1 | 1 | 2016 | 2016 |
| Rheumatoid Arthritis | C0003873 | TMEM235 | 283999 | A6NFC5 | transmembrane protein 235 | | 1 | 1 | 0.115 | 0.000458 | 0.1 | | 1 | 1 | 1 | 2019 | 2019 |
| Rheumatoid Ar | C0003873 | HCG22 | 285834 | E2RYF7 | HLA complex group 22 (gene/pseudogene) | | 20 | 0.736 | 0.423 | | 0.1 | | 1 | 2 | 2 | 2007 | 2009 |

| | | | | | | | | | | | | | | | | | |
|---|---|---|---|---|---|---|---|---|---|---|---|---|---|---|---|---|---|
| thritis | | | | | | | | | | | | | | | | | |
| Rheumatoid Arthritis | C0003873 | GSTM1 | 2944 | P09488 | glutathione S-transferase mu 1 | | 627 | 0.38 | 0.923 | 0.0020064 | 0.1 | | 0.8 | 15 | 0 | 1999 | 2019 |
| Rheumatoid Arthritis | C0003873 | GSTT1 | 2952 | P30711 | glutathione S-transferase theta 1 | | 541 | 0.393 | 0.923 | 0.000148 | 0.1 | | 0.9 | 10 | 0 | 1999 | 2019 |
| Rheumatoid Arthritis | C0003873 | GUCY1B2 | 2974 | O75343 | guanylate cyclase 1 soluble subunit beta 2 (pseudogene) | | 2 | 1 | 0.115 | | 0.1 | | 1 | 1 | 1 | 2015 | 2015 |
| Rheumatoid Arthritis | C0003873 | HIF1A | 3091 | Q16665 | hypoxia inducible factor 1 subunit alpha | Transcription factor | 1044 | 0.327 | 0.923 | 0.9777 | 0.1 | | 1 | 19 | 0 | 2003 | 2020 |
| Rheumatoid Arthritis | C0003873 | HLA-DOB | 3112 | P13765 | major histocompatibility complex, class II, DO beta | Immune response | 24 | 0.705 | 0.5 | 1.92E-06 | 0.1 | | 1 | 4 | 8 | 2007 | 2016 |
| Rheumatoid Arthritis | C0003873 | HLA-DPB2 | 3116 | | major histocompatibility complex, class II, DP beta 2 (pseudogene) | | 15 | 0.743 | 0.5 | | 0.1 | | 1 | 1 | 1 | 2009 | 2009 |
| Rheumatoi | C0003 | HLA-DQB2 | 3120 | P05538 | major histocompatibility | Immune res | 91 | 0.5 | 0.7 | 0.042 | 0.1 | | 1 | 3 | 3 | 2007 | 2011 |

| | | | | | | | | | | | | | | | | | |
|---|---|---|---|---|---|---|---|---|---|---|---|---|---|---|---|---|---|
| d Arthritis | 873 | | | | complex, class II, DQ beta 2 | ponse | | 73 | 31 | 679 | | | | | | | |
| Rheumatoid Arthritis | C0003873 | HLA-DQB3 | 3121 | | major histocompatibility complex, class II, DQ beta 3 | | 2 | 0.931 | 0.192 | | 0.1 | | 1 | 1 | 11 | 2011 | 2011 |
| Rheumatoid Arthritis | C0003873 | HLA-DRA | 3122 | P01903 | major histocompatibility complex, class II, DR alpha | Immune response | 92 | 0.581 | 0.769 | 0.51949 | 0.1 | | 1 | 3 | 36 | 2007 | 2011 |
| Rheumatoid Arthritis | C0003873 | HLA-DRB4 | 3126 | P13762 | major histocompatibility complex, class II, DR beta 4 | Immune response | 87 | 0.565 | 0.846 | | 0.1 | | 0.8 | 10 | 0 | 1989 | 2019 |
| Rheumatoid Arthritis | C0003873 | HLA-DRB9 | 3132 | | major histocompatibility complex, class II, DR beta 9 (pseudogene) | | 33 | 0.682 | 0.654 | | 0.1 | | 1 | 6 | 21 | 2007 | 2019 |
| Rheumatoid Arthritis | C0003873 | HLA-G | 3135 | P17693 | major histocompatibility complex, class I, G | | 279 | 0.461 | 0.769 | 1.37E-11 | 0.1 | | 0.9 | 10 | 2 | 2006 | 2017 |
| Rheumatoid Arthritis | C0003873 | HMGB1 | 3146 | P09429 | high mobility group box 1 | Nucleic acid binding | 724 | 0.368 | 0.923 | 0.82035 | 0.1 | | 1 | 20 | 3 | 2003 | 2020 |

| | | | | | | | | | | | | | | | | |
|---|---|---|---|---|---|---|---|---|---|---|---|---|---|---|---|---|
| Rheumatoid Arthritis | C0003873 | HSPA4 | 3308 | P34932 | heat shock protein family A (Hsp70) member 4 | 550 | 0.394 | 0.923 | 0.99954 | 0.1 | | 0.929 | 14 | 0 | 1996 | 2019 |
| Rheumatoid Arthritis | C0003873 | HSPD1 | 3329 | P10809 | heat shock protein family D (Hsp60) member 1 | 398 | 0.432 | 0.808 | 0.99257 | 0.1 | | 1 | 12 | 1 | 1993 | 2020 |
| Rheumatoid Arthritis | C0003873 | ICAM1 | 3383 | P05362 | intercellular adhesion molecule 1 | 737 | 0.364 | 0.962 | 0.035295 | 0.1 | | 0.963 | 27 | 3 | 1996 | 2020 |
| Rheumatoid Arthritis | C0003873 | IFNA1 | 3439 | P01562 | interferon alpha 1 | 662 | 0.371 | 0.923 | | 0.1 | | 0.857 | 14 | 0 | 2003 | 2019 |
| Rheumatoid Arthritis | C0003873 | IFNA13 | 3447 | P01562 | interferon alpha 13 | 646 | 0.374 | 0.923 | | 0.1 | | 0.857 | 14 | 0 | 2003 | 2019 |
| Rheumatoid Arthritis | C0003873 | IGF1 | 3479 | P05019 | insulin like growth factor 1 | 1206 | 0.318 | 0.885 | 0.2716 | 0.1 | | 0.941 | 17 | 0 | 1995 | 2019 |
| Rheumatoid Ar | C0003873 | FAS | 355 | P25445 | Fas cell surface death receptor | 754 | 0.372 | 0.923 | 0.8117 | 0.1 | | 0.7 | 10 | 2 | 1999 | 2015 |

| | | | | | | | | | | | | | | | | |
|---|---|---|---|---|---|---|---|---|---|---|---|---|---|---|---|---|
| thritis | | | | | | | | | | | | | | | | |
| Rheumatoid Arthritis | C0003873 | IL1A | 3552 | P01583 | interleukin 1 alpha | 1002 | 0.333 | 0.962 | 0.00016 | 0.1 | | 1 | 104 | 1 | 1988 | 2020 |
| Rheumatoid Arthritis | C0003873 | IL2 | 3558 | P60568 | interleukin 2 | 950 | 0.336 | 0.885 | 0.47968 | 0.1 | | 0.978 | 46 | 1 | 1986 | 2019 |
| Rheumatoid Arthritis | C0003873 | IL4 | 3565 | P05112 | interleukin 4 | 996 | 0.332 | 0.962 | 0.004737 | 0.1 | | 0.942 | 52 | 1 | 1992 | 2019 |
| Rheumatoid Arthritis | C0003873 | IL7 | 3574 | P13232 | interleukin 7 | 275 | 0.464 | 0.808 | 0.92574 | 0.1 | | 1 | 11 | 0 | 2005 | 2019 |
| Rheumatoid Arthritis | C0003873 | IL9 | 3578 | P15248 | interleukin 9 | 291 | 0.461 | 0.846 | 8.11E-07 | 0.1 | | 1 | 12 | 0 | 2000 | 2019 |
| Rheumatoid Arthritis | C0003873 | IL13 | 3596 | P35225 | interleukin 13 | 587 | 0.386 | 0.846 | 0.014426 | 0.1 | | 1 | 13 | 1 | 1996 | 2019 |
| Rheumatoi | C0003 | IL15 | 3600 | P40933 | interleukin 15 | 426 | 0.4 | 0.8 | 0.708 | 0.1 | | 1 | 27 | 0 | 1999 | 2019 |

| | | | | | | | | | | | | | | | | | |
|---|---|---|---|---|---|---|---|---|---|---|---|---|---|---|---|---|---|
| d Arthritis | 873 | | | | | | | 22 | 46 | 59 | | | | | | | |
| Rheumatoid Arthritis | C0003873 | IL17A | 3605 | Q16552 | interleukin 17A | | 1074 | 0.324 | 0.923 | 0.043049 | 0.1 | | 0.959 | 194 | 7 | 1999 | 2020 |
| Rheumatoid Arthritis | C0003873 | ILF3 | 3609 | Q12906 | interleukin enhancer binding factor 3 | Nucleic acid binding | 68 | 0.621 | 0.654 | 1 | 0.1 | | 1 | 1 | 1 | 2019 | 2019 |
| Rheumatoid Arthritis | C0003873 | CXCL10 | 3627 | P02778 | C-X-C motif chemokine ligand 10 | Signaling | 631 | 0.378 | 0.885 | 0.36865 | 0.1 | | 0.9 | 20 | 1 | 2001 | 2019 |
| Rheumatoid Arthritis | C0003873 | AR | 367 | P10275 | androgen receptor | Nuclear receptor | 854 | 0.351 | 0.846 | 0.98837 | 0.1 | | 0.9 | 10 | 0 | 1999 | 2016 |
| Rheumatoid Arthritis | C0003873 | ITPR3 | 3710 | Q14573 | inositol 1,4,5-trisphosphate receptor type 3 | Ion channel | 112 | 0.555 | 0.808 | 2.55E-22 | 0.1 | | 1 | 3 | 8 | 2007 | 2011 |
| Rheumatoid Arthritis | C0003873 | JAK1 | 3716 | P23458 | Janus kinase 1 | Kinase | 239 | 0.474 | 0.769 | 0.99998 | 0.1 | | 1 | 30 | 0 | 2013 | 2020 |

| | | | | | | | | | | | | | | | | |
|---|---|---|---|---|---|---|---|---|---|---|---|---|---|---|---|---|
| Rheumatoid Arthritis | C0003873 | JAK2 | 3717 | O60674 | Janus kinase 2 | Kinase | 644 | 0.385 | 0.885 | 0.65372 | 0.1 | | 1 | 19 | 0 | 2011 | 2020 |
| Rheumatoid Arthritis | C0003873 | JAK3 | 3718 | P52333 | Janus kinase 3 | Kinase | 194 | 0.505 | 0.731 | 5.93E-06 | 0.1 | | 0.923 | 13 | 0 | 2011 | 2020 |
| Rheumatoid Arthritis | C0003873 | GALNT18 | 374378 | Q6P9A2 | polypeptide N-acetylgalactosaminyltransferase 18 | | 3 | 0.931 | 0.192 | 0.000303 | 0.1 | | 1 | 1 | 1 | 2013 | 2013 |
| Rheumatoid Arthritis | C0003873 | KDR | 3791 | P35968 | kinase insert domain receptor | Kinase | 623 | 0.378 | 0.885 | 0.99982 | 0.1 | | 1 | 10 | 3 | 2002 | 2019 |
| Rheumatoid Arthritis | C0003873 | KIR3DL1 | 3811 | P43629 | killer cell immunoglobulin like receptor, three Ig domains and long cytoplasmic tail 1 | Receptor | 231 | 0.475 | 0.769 | 8.36E-14 | 0.1 | | 0.909 | 11 | 0 | 2006 | 2019 |
| Rheumatoid Arthritis | C0003873 | ZKSCAN4 | 387032 | Q969J2 | zinc finger with KRAB and SCAN domains 4 | | 8 | 0.839 | 0.269 | 0.000142 | 0.1 | | 1 | 1 | 2 | 2009 | 2009 |

| | | | | | | | | | | | | | | | | |
|---|---|---|---|---|---|---|---|---|---|---|---|---|---|---|---|---|
| Rheumatoid Arthritis | C0003873 | TMEM179 | 388021 | Q6ZVK1 | transmembrane protein 179 | | 1 | 1 | 0.115 | 0.67923 | 0.1 | | 1 | 1 | 1 | 2019 | 2019 |
| Rheumatoid Arthritis | C0003873 | SFTA2 | 389376 | Q6UW10 | surfactant associated 2 | | 25 | 0.695 | 0.577 | 0.036441 | 0.1 | | 1 | 2 | 3 | 2007 | 2009 |
| Rheumatoid Arthritis | C0003873 | COL11A2P1 | 394214 | | collagen type XI alpha 2 pseudogene 1 | | 7 | 0.805 | 0.462 | | 0.1 | | 1 | 3 | 5 | 2007 | 2011 |
| Rheumatoid Arthritis | C0003873 | LEP | 3952 | P41159 | leptin | | 931 | 0.349 | 0.846 | 0.46491 | 0.1 | | 0.929 | 14 | 1 | 2007 | 2020 |
| Rheumatoid Arthritis | C0003873 | LGALS3 | 3958 | P17931 | galectin 3 | Signaling | 557 | 0.392 | 0.846 | 2.32E-05 | 0.1 | | 0.917 | 12 | 2 | 2000 | 2019 |
| Rheumatoid Arthritis | C0003873 | LINC02649 | 399715 | | long intergenic non-protein coding RNA 2649 | | 10 | 0.792 | 0.308 | | 0.1 | | 1 | 5 | 2 | 2012 | 2019 |
| Rheumatoid Ar | C0003873 | LINC02656 | 399716 | | long intergenic non-protein coding RNA 2656 | | 4 | 0.931 | 0.154 | | 0.1 | | 1 | 1 | 2 | 2012 | 2012 |

| | | | | | | | | | | | | | | | | |
|---|---|---|---|---|---|---|---|---|---|---|---|---|---|---|---|---|
| thritis | | | | | | | | | | | | | | | | |
| Rheumatoid Arthritis | C0003873 | GATA3-AS1 | 399717 | | GATA3 antisense RNA 1 | 2 | 0.931 | 0.192 | | 0.1 | | 1 | 1 | 1 | 2012 | 2012 |
| Rheumatoid Arthritis | C0003873 | LMNA | 4000 | P02545 | lamin A/C | 824 | 0.384 | 0.885 | 0.9994 | 0.1 | | 1 | 1 | 1 | 2019 | 2019 |
| Rheumatoid Arthritis | C0003873 | LINC01185 | 400957 | | long intergenic non-protein coding RNA 1185 | 13 | 0.743 | 0.346 | | 0.1 | | 1 | 2 | 2 | 2009 | 2016 |
| Rheumatoid Arthritis | C0003873 | LINC00243 | 401247 | | long intergenic non-protein coding RNA 243 | 24 | 0.729 | 0.423 | | 0.1 | | 1 | 2 | 6 | 2007 | 2009 |
| Rheumatoid Arthritis | C0003873 | MCCD1 | 401250 | P59942 | mitochondrial coiled-coil domain 1 | 6 | 0.89 | 0.231 | 0.001932 | 0.1 | | 1 | 1 | 3 | 2011 | 2011 |
| Rheumatoid Arthritis | C0003873 | TRIM26BP | 404024 | | tripartite motif containing 26B, pseudogene | 4 | 0.861 | 0.269 | | 0.1 | | 1 | 1 | 1 | 2009 | 2009 |
| Rheumatoi | C0003 | MTCO3P1 | 4040 | | MT-CO3 pseudogene 1 | 5 | 0.8 | 0.2 | | 0.1 | | 1 | 1 | 2 | 2011 | 2011 |

| | | | | | | | | | | | | | | | | | |
|---|---|---|---|---|---|---|---|---|---|---|---|---|---|---|---|---|---|
| d Arthritis | 873 | | 26 | | | | | 61 | 31 | | | | | | | | |
| Rheumatoid Arthritis | C0003873 | LTA | 4049 | P01374 | lymphotoxin alpha | Signaling | 353 | 0.434 | 0.885 | 0.196885 | 0.1 | | 0.867 | 15 | 7 | 1994 | 2019 |
| Rheumatoid Arthritis | C0003873 | MIR146A | 406938 | | microRNA 146a | | 505 | 0.398 | 0.885 | | 0.1 | | 0.889 | 36 | 1 | 2008 | 2019 |
| Rheumatoid Arthritis | C0003873 | MIR155 | 406947 | | microRNA 155 | | 584 | 0.384 | 0.885 | | 0.1 | | 1 | 29 | 1 | 2008 | 2019 |
| Rheumatoid Arthritis | C0003873 | MIR223 | 407008 | | microRNA 223 | | 348 | 0.433 | 0.808 | | 0.1 | | 1 | 15 | 0 | 2010 | 2019 |
| Rheumatoid Arthritis | C0003873 | DDR1-DT | 414771 | | DDR1 divergent transcript | | 2 | 0.931 | 0.115 | | 0.1 | | 1 | 2 | 1 | 2007 | 2009 |
| Rheumatoid Arthritis | C0003873 | HCG18 | 414777 | | HLA complex group 18 | | 27 | 0.722 | 0.538 | | 0.1 | | 1 | 2 | 4 | 2007 | 2009 |

| | | | | | | | | | | | | | | | | |
|---|---|---|---|---|---|---|---|---|---|---|---|---|---|---|---|---|
| Rheumatoid Arthritis | C0003873 | HCG17 | 414778 | | HLA complex group 17 | | 17 | 0.769 | 0.346 | | 0.1 | | 1 | 2 | 6 | 2007 | 2009 |
| Rheumatoid Arthritis | C0003873 | MBL2 | 4153 | P11226 | mannose binding lectin 2 | Receptor | 563 | 0.39 | 0.846 | 0.002684 | 0.1 | | 0.875 | 32 | 5 | 1998 | 2019 |
| Rheumatoid Arthritis | C0003873 | MEFV | 4210 | O15553 | MEFV innate immuity regulator, pyrin | | 410 | 0.44 | 0.885 | 1.02E-14 | 0.1 | | 0.929 | 14 | 2 | 2004 | 2019 |
| Rheumatoid Arthritis | C0003873 | MAP3K4 | 4216 | Q9Y6R4 | mitogen-activated protein kinase kinase kinase 4 | Kinase | 21 | 0.729 | 0.462 | 1 | 0.1 | | 1 | 1 | 2 | 2013 | 2013 |
| Rheumatoid Arthritis | C0003873 | MMP1 | 4312 | P03956 | matrix metallopeptidase 1 | Enzyme | 589 | 0.385 | 0.885 | 7.72E-18 | 0.1 | | 0.982 | 56 | 0 | 1992 | 2019 |
| Rheumatoid Arthritis | C0003873 | MMP3 | 4314 | P08254 | matrix metallopeptidase 3 | Enzyme | 473 | 0.408 | 0.885 | 5.74E-15 | 0.1 | | 0.974 | 78 | 0 | 1996 | 2019 |
| Rheumatoid Ar | C0003873 | MMP9 | 4318 | P14780 | matrix metallopeptidase 9 | Enzyme | 1337 | 0.305 | 0.923 | 1.89E- | 0.1 | | 1 | 32 | 1 | 1999 | 2019 |

| | | | | | | | | | | | | | | | | | |
|---|---|---|---|---|---|---|---|---|---|---|---|---|---|---|---|---|---|
| thritis | | | | | | | | | | 17 | | | | | | | |
| Rheumatoid Arthritis | C0003873 | MMP13 | 4322 | P45452 | matrix metallopeptidase 13 | Enzyme | 328 | 0.452 | 0.923 | 7.88E-18 | 0.1 | | 1 | 34 | 0 | 1996 | 2020 |
| Rheumatoid Arthritis | C0003873 | BCL2L15 | 440603 | Q5TBC7 | BCL2 like 15 | | 4 | 0.89 | 0.192 | 0.048246 | 0.1 | | 1 | 1 | 2 | 2009 | 2009 |
| Rheumatoid Arthritis | C0003873 | MYL8P | 442204 | | myosin light chain 8, pseudogene | | 1 | 1 | 0.115 | | 0.1 | | 1 | 1 | 1 | 2011 | 2011 |
| Rheumatoid Arthritis | C0003873 | MSH5 | 4439 | O43196 | mutS homolog 5 | Nucleic acid binding | 51 | 0.641 | 0.692 | 3.2E-11 | 0.1 | | 1 | 3 | 10 | 2007 | 2011 |
| Rheumatoid Arthritis | C0003873 | MSI1 | 4440 | O43347 | musashi RNA binding protein 1 | | 116 | 0.55 | 0.615 | 0.99357 | 0.1 | | 1 | 1 | 1 | 2019 | 2019 |
| Rheumatoid Arthritis | C0003873 | COX2 | 4513 | P00403 | cytochrome c oxidase subunit II | Enzyme | 875 | 0.352 | 0.962 | | 0.1 | | 1 | 27 | 0 | 1994 | 2019 |
| Rheumatoi | C0003 | MTF1 | 4520 | Q14872 | metal regulatory transcrip | Transcripti | 21 | 0.7 | 0.5 | 0.968 | 0.1 | | 1 | 2 | 1 | 2014 | 2019 |

| | | | | | | | | | | | | | | | | | |
|---|---|---|---|---|---|---|---|---|---|---|---|---|---|---|---|---|---|
| d Arthritis | 873 | | | | tion factor 1 | on factor | | 51 | | 97 | | | | | | | |
| Rheumatoid Arthritis | C0003873 | MTX1 | 4580 | Q13505 | metaxin 1 | | 124 | 0.546 | 0.731 | 0.013271 | 0.1 | | 0.969 | 32 | 0 | 2002 | 2020 |
| Rheumatoid Arthritis | C0003873 | LRRC18 | 474354 | Q8N456 | leucine rich repeat containing 18 | | 2 | 0.931 | 0.115 | 3.51E-05 | 0.1 | | 1 | 1 | 2 | 2009 | 2009 |
| Rheumatoid Arthritis | C0003873 | NFKB1 | 4790 | P19838 | nuclear factor kappa B subunit 1 | Transcription factor | 551 | 0.396 | 0.923 | 1 | 0.1 | | 0.929 | 14 | 2 | 2002 | 2019 |
| Rheumatoid Arthritis | C0003873 | NM | 4827 | | neutrophil migration | | 250 | 0.473 | 0.808 | | 0.1 | | 0.917 | 12 | 0 | 2000 | 2020 |
| Rheumatoid Arthritis | C0003873 | NOS3 | 4846 | P29474 | nitric oxide synthase 3 | | 706 | 0.378 | 0.885 | 6.72E-07 | 0.1 | | 1 | 10 | 1 | 2004 | 2020 |
| Rheumatoid Arthritis | C0003873 | ACR | 49 | P10323 | acrosin | Enzyme | 230 | 0.493 | 0.846 | 0.0122215 | 0.1 | | 1 | 51 | 1 | 1993 | 2020 |
| Rheu | C00 | NUP88 | 49 | Q99567 | nucleoporin 88 | Trans | 58 | 0. | 0. | 1.9 | 0.1 | | 1 | 2 | 1 | 2014 | 20 |

| | | | | | | | | | | | | | | | | | |
|---|---|---|---|---|---|---|---|---|---|---|---|---|---|---|---|---|---|
| matoid Arthritis | 03873 | | 27 | | | porter | | 659 | 577 | 6E-08 | | | | | | | 19 |
| Rheumatoid Arthritis | C0003873 | IL20 | 50604 | Q9NYY1 | interleukin 20 | | 111 | 0.556 | 0.769 | 1.34E-06 | 0.1 | | 0.929 | 14 | 0 | 2006 | 2019 |
| Rheumatoid Arthritis | C0003873 | IL22 | 50616 | Q9GZX6 | interleukin 22 | | 551 | 0.393 | 0.885 | 0.000188 | 0.1 | | 0.933 | 45 | 0 | 2005 | 2020 |
| Rheumatoid Arthritis | C0003873 | SNHG32 | 50854 | | small nucleolar RNA host gene 32 | | 12 | 0.805 | 0.308 | 0.023621 | 0.1 | | 1 | 1 | 1 | 2011 | 2011 |
| Rheumatoid Arthritis | C0003873 | PBX2 | 5089 | P40425 | PBX homeobox 2 | Transcription factor | 29 | 0.686 | 0.462 | 0.99532 | 0.1 | | 1 | 2 | 5 | 2007 | 2011 |
| Rheumatoid Arthritis | C0003873 | CPA4 | 51200 | Q9UI42 | carboxypeptidase A4 | Enzyme | 49 | 0.633 | 0.5 | 9.39E-18 | 0.1 | | 1 | 1 | 1 | 2019 | 2019 |
| Rheumatoid Arthritis | C0003873 | PDCD1 | 5133 | Q15116 | programmed cell death 1 | | 497 | 0.402 | 0.846 | 0.41706 | 0.1 | | 0.9 | 20 | 4 | 2004 | 2019 |

| | | | | | | | | | | | | | | | | | |
|---|---|---|---|---|---|---|---|---|---|---|---|---|---|---|---|---|---|
| Rheumatoid Arthritis | C0003873 | PDE2A | 5138 | O00408 | phosphodiesterase 2A | | 47 | 0.666 | 0.577 | 0.631 | 0.1 | | 1 | 1 | 1 | 2012 | 2012 |
| Rheumatoid Arthritis | C0003873 | WNT16 | 51384 | Q9UBV4 | Wnt family member 16 | Signaling | 29 | 0.686 | 0.654 | 1.65E-06 | 0.1 | | 1 | 1 | 1 | 2019 | 2019 |
| Rheumatoid Arthritis | C0003873 | RAB14 | 51552 | P61106 | RAB14, member RAS oncogene family | | 35 | 0.674 | 0.615 | 0.99047 | 0.1 | | 1 | 1 | 1 | 2009 | 2009 |
| Rheumatoid Arthritis | C0003873 | CYRIB | 51571 | Q9NUQ9 | CYFIP related Rac1 interactor B | | 25 | 0.76 | 0.538 | 0.97612 | 0.1 | | 1 | 1 | 1 | 2019 | 2019 |
| Rheumatoid Arthritis | C0003873 | SERPINA1 | 5265 | P01009 | serpin family A member 1 | Enzyme modulator | 482 | 0.41 | 0.923 | 6.85E-08 | 0.1 | | 0.933 | 15 | 0 | 1976 | 2017 |
| Rheumatoid Arthritis | C0003873 | PIK3CA | 5290 | P42336 | phosphatidylinositol-4,5-bisphosphate 3-kinase catalytic subunit alpha | Kinase | 1511 | 0.292 | 0.923 | 1 | 0.1 | | 1 | 30 | 0 | 2005 | 2020 |
| Rheumatoid | C0003873 | PIK3CB | 5291 | P42338 | phosphatidylinositol-4,5-bisphosphate 3- | Kinase | 1083 | 0.322 | 0.885 | 0.999 | 0.1 | | 1 | 29 | 0 | 2005 | 2020 |

| | | | | | | | | | | | | | | | | | |
|---|---|---|---|---|---|---|---|---|---|---|---|---|---|---|---|---|---|
| Arthritis | | | | | kinase catalytic subunit beta | | | | | 64 | | | | | | | |
| Rheumatoid Arthritis | C0003873 | PIK3CD | 5293 | O00329 | phosphatidylinositol-4,5-bisphosphate 3-kinase catalytic subunit delta | Kinase | 1119 | 0.319 | 0.885 | 0.99999 | 0.1 | | 1 | 33 | 0 | 2005 | 2020 |
| Rheumatoid Arthritis | C0003873 | PIK3CG | 5294 | P48736 | phosphatidylinositol-4,5-bisphosphate 3-kinase catalytic subunit gamma | Kinase | 1101 | 0.32 | 0.885 | 1.41E-06 | 0.1 | | 1 | 31 | 0 | 2005 | 2020 |
| Rheumatoid Arthritis | C0003873 | PLCL1 | 5334 | Q15111 | phospholipase C like 1 (inactive) | Enzyme | 69 | 0.608 | 0.654 | 0.012059 | 0.1 | | 1 | 1 | 1 | 2019 | 2019 |
| Rheumatoid Arthritis | C0003873 | IL17D | 53342 | Q8TAD2 | interleukin 17D | | 259 | 0.464 | 0.808 | 0.19796 | 0.1 | | 1 | 10 | 0 | 2010 | 2019 |
| Rheumatoid Arthritis | C0003873 | PLG | 5340 | P00747 | plasminogen | Enzyme | 586 | 0.389 | 0.923 | 0.010345 | 0.1 | | 1 | 13 | 0 | 1996 | 2019 |
| Rheumatoid Arthritis | C0003873 | GDAP1 | 54332 | Q8TB36 | ganglioside induced differentiation associated protein 1 | | 96 | 0.612 | 0.538 | 3.24E-08 | 0.1 | | 1 | 1 | 1 | 2013 | 2013 |

| | | | | | | | | | | | | | | | | | |
|---|---|---|---|---|---|---|---|---|---|---|---|---|---|---|---|---|---|
| Rheumatoid Arthritis | C0003873 | KRT20 | 54474 | P35900 | keratin 20 | | 559 | 0.388 | 0.846 | 9.08E-11 | 0.1 | | 0.882 | 17 | 1 | 2001 | 2019 |
| Rheumatoid Arthritis | C0003873 | CCHCR1 | 54535 | Q8TD31 | coiled-coil alpha-helical rod protein 1 | | 193 | 0.505 | 0.808 | 1.01E-16 | 0.1 | | 1 | 2 | 2 | 2007 | 2009 |
| Rheumatoid Arthritis | C0003873 | NECAB2 | 54550 | Q7Z6G3 | N-terminal EF-hand calcium binding protein 2 | | 1 | 1 | 0.115 | 2.24E-13 | 0.1 | | 1 | 1 | 1 | 2019 | 2019 |
| Rheumatoid Arthritis | C0003873 | POU5F1 | 5460 | Q01860 | POU class 5 homeobox 1 | | 328 | 0.441 | 0.846 | 0.95371 | 0.1 | | 1 | 1 | 1 | 2007 | 2007 |
| Rheumatoid Arthritis | C0003873 | RSBN1 | 54665 | Q5VWQ0 | round spermatid basic protein 1 | | 3 | 0.89 | 0.115 | 0.099107 | 0.1 | | 1 | 1 | 2 | 2009 | 2009 |
| Rheumatoid Arthritis | C0003873 | RBFOX1 | 54715 | Q9NWB1 | RNA binding fox-1 homolog 1 | Nucleic acid binding | 103 | 0.584 | 0.692 | 0.95263 | 0.1 | | 1 | 1 | 1 | 2019 | 2019 |
| Rheumatoid Ar | C0003873 | PPIAP9 | 5491 | | peptidylprolyl isomerase A pseudogene 9 | | 1 | 1 | 0.115 | | 0.1 | | 1 | 1 | 1 | 2011 | 2011 |

| | | | | | | | | | | | | | | | | | |
|---|---|---|---|---|---|---|---|---|---|---|---|---|---|---|---|---|---|
| thritis | | | | | | | | | | | | | | | | | |
| Rheumatoid Arthritis | C0003873 | VPS37C | 55048 | A5D8V6 | VPS37C subunit of ESCRT-I | | 5 | 0.861 | 0.115 | 0.0382336 | 0.1 | | 1 | 1 | 1 | 2012 | 2012 |
| Rheumatoid Arthritis | C0003873 | ENOX1 | 55068 | Q8TC92 | ecto-NOX disulfide-thiol exchanger 1 | Enzyme | 15 | 0.76 | 0.538 | 2.33E-06 | 0.1 | | 1 | 1 | 6 | 2013 | 2013 |
| Rheumatoid Arthritis | C0003873 | KIF26B | 55083 | Q2KJY2 | kinesin family member 26B | Cellular structure | 38 | 0.67 | 0.5 | 0.99999 | 0.1 | | 1 | 1 | 1 | 2019 | 2019 |
| Rheumatoid Arthritis | C0003873 | PPP1R10 | 5514 | Q96QC0 | protein phosphatase 1 regulatory subunit 10 | | 22 | 0.751 | 0.423 | 1 | 0.1 | | 1 | 1 | 1 | 2009 | 2009 |
| Rheumatoid Arthritis | C0003873 | ACOXL | 55289 | Q9NUZ1 | acyl-CoA oxidase like | Enzyme | 46 | 0.674 | 0.577 | 5.25E-15 | 0.1 | | 1 | 1 | 1 | 2014 | 2014 |
| Rheumatoid Arthritis | C0003873 | TCP11L1 | 55346 | Q9NUJ3 | t-complex 11 like 1 | Receptor | 1 | 1 | 0.115 | 0.00437 | 0.1 | | 1 | 1 | 1 | 2013 | 2013 |
| Rheumatoi | C0003 | CDK5RAP2 | 557 | Q96SN8 | CDK5 regulatory subunit | | 42 | 0.6 | 0.5 | 4.62E | 0.1 | | 1 | 1 | 1 | 2014 | 2014 |

| | | | | | | | | | | | | | | | | | |
|---|---|---|---|---|---|---|---|---|---|---|---|---|---|---|---|---|---|
| d Arthritis | 873 | | 55 | | associated protein 2 | | | 82 | 77 | -23 | | | | | | | |
| Rheumatoid Arthritis | C0003873 | ZNF302 | 55900 | Q9NR11 | zinc finger protein 302 | | 1 | 1 | 0.115 | 0.000194 | 0.1 | | 1 | 1 | 1 | 2011 | 2011 |
| Rheumatoid Arthritis | C0003873 | MAPK1 | 5594 | P28482 | mitogen-activated protein kinase 1 | Kinase | 1059 | 0.33 | 0.923 | 0.99698 | 0.1 | | 0.976 | 42 | 0 | 2000 | 2020 |
| Rheumatoid Arthritis | C0003873 | MAPK8 | 5599 | P45983 | mitogen-activated protein kinase 8 | Kinase | 520 | 0.397 | 0.885 | 0.99823 | 0.1 | | 1 | 14 | 0 | 2004 | 2018 |
| Rheumatoid Arthritis | C0003873 | PRL | 5617 | P01236 | prolactin | Signaling | 506 | 0.406 | 0.885 | 2.9E-07 | 0.1 | | 1 | 13 | 1 | 1996 | 2019 |
| Rheumatoid Arthritis | C0003873 | PRTN3 | 5657 | P24158 | proteinase 3 | Enzyme | 215 | 0.503 | 0.808 | 3.8E-05 | 0.1 | | 0.947 | 38 | 0 | 1999 | 2020 |
| Rheumatoid Arthritis | C0003873 | TRIM39 | 56658 | Q9HCM9 | tripartite motif containing 39 | | 9 | 0.792 | 0.462 | 0.99474 | 0.1 | | 1 | 1 | 1 | 2009 | 2009 |
| Rheu | C00 | RETN | 56 | Q9HD89 | resistin | | 302 | 0. | 0. | 0.0 | 0.1 | | 0.8 | 12 | 3 | 2005 | 20 |

| | | | | | | | | | | | | | | | | | |
|---|---|---|---|---|---|---|---|---|---|---|---|---|---|---|---|---|---|
| matoid Arthritis | 03873 | | 729 | | | | | 454 | 808 | 36545 | | | 33 | | | | 19 |
| Rheumatoid Arthritis | C0003873 | PSMA4 | 5685 | P25789 | proteasome 20S subunit alpha 4 | | 30 | 0.716 | 0.385 | 0.59959 | 0.1 | | 1 | 1 | 1 | 2013 | 2013 |
| Rheumatoid Arthritis | C0003873 | PRDM10 | 56980 | Q9NQV6 | PR/SET domain 10 | Transcription factor | 19 | 0.76 | 0.385 | 0.15304 | 0.1 | | 1 | 1 | 1 | 2019 | 2019 |
| Rheumatoid Arthritis | C0003873 | VARS2 | 57176 | Q5ST30 | valyl-tRNA synthetase 2, mitochondrial | | 37 | 0.666 | 0.577 | 5.83E-17 | 0.1 | | 1 | 2 | 4 | 2007 | 2009 |
| Rheumatoid Arthritis | C0003873 | MIR499A | 574501 | | microRNA 499a | | 156 | 0.519 | 0.846 | | 0.1 | | 0.9 | 10 | 1 | 2011 | 2019 |
| Rheumatoid Arthritis | C0003873 | PRR12 | 57479 | Q9ULL5 | proline rich 12 | | 35 | 0.716 | 0.385 | 1 | 0.1 | | 1 | 1 | 1 | 2019 | 2019 |
| Rheumatoid Arthritis | C0003873 | DENND1A | 57706 | Q8TEH3 | DENN domain containing 1A | | 21 | 0.736 | 0.538 | 0.99976 | 0.1 | | 1 | 1 | 1 | 2019 | 2019 |

| | | | | | | | | | | | | | | | | | |
|---|---|---|---|---|---|---|---|---|---|---|---|---|---|---|---|---|---|
| Rheumatoid Arthritis | C0003873 | ANO8 | 57719 | Q9HCE9 | anoctamin 8 | | 3 | 0.89 | 0.192 | 0.95634 | 0.1 | | 1 | 1 | 1 | 2014 | 2014 |
| Rheumatoid Arthritis | C0003873 | NCOA5 | 57727 | Q9HCD5 | nuclear receptor coactivator 5 | Receptor | 53 | 0.631 | 0.692 | 0.99926 | 0.1 | | 1 | 11 | 0 | 2002 | 2020 |
| Rheumatoid Arthritis | C0003873 | C6orf47 | 57827 | O95873 | chromosome 6 open reading frame 47 | | 21 | 0.722 | 0.423 | 0.066412 | 0.1 | | 1 | 3 | 2 | 2007 | 2011 |
| Rheumatoid Arthritis | C0003873 | LY6G5B | 58496 | Q8NDX9 | lymphocyte antigen 6 family member G5B | | 3 | 0.931 | 0.192 | 8.18E-06 | 0.1 | | 1 | 1 | 2 | 2011 | 2011 |
| Rheumatoid Arthritis | C0003873 | LY6G6D | 58530 | O95868 | lymphocyte antigen 6 family member G6D | | 11 | 0.805 | 0.385 | 0.032554 | 0.1 | | 1 | 3 | 2 | 2007 | 2011 |
| Rheumatoid Arthritis | C0003873 | IL21 | 59067 | Q9HBE4 | interleukin 21 | | 321 | 0.441 | 0.769 | 0.6254 | 0.1 | | 0.912 | 34 | 2 | 2004 | 2020 |
| Rheumatoid Ar | C0003873 | RARA | 5914 | P10276 | retinoic acid receptor alpha | Nuclear receptor | 274 | 0.47 | 0.846 | 0.96305 | 0.1 | | 1 | 10 | 0 | 1994 | 2019 |

| | | | | | | | | | | | | | | | | | |
|---|---|---|---|---|---|---|---|---|---|---|---|---|---|---|---|---|---|
| thritis | | | | | | | | | | | | | | | | | |
| Rheumatoid Arthritis | C0003873 | BCL2 | 596 | P10415 | BCL2 apoptosis regulator | Signaling | 1456 | 0.291 | 0.885 | 0.55903 | 0.1 | | 1 | 22 | 1 | 1994 | 2019 |
| Rheumatoid Arthritis | C0003873 | RELA | 5970 | Q04206 | RELA proto-oncogene, NF-kB subunit | Transcription factor | 483 | 0.406 | 0.885 | 0.99948 | 0.1 | | 1 | 13 | 0 | 2002 | 2019 |
| Rheumatoid Arthritis | C0003873 | ACTB | 60 | P60709 | actin beta | Cellular structure | 1110 | 0.325 | 0.923 | 0.98564 | 0.1 | | 1 | 11 | 0 | 1991 | 2019 |
| Rheumatoid Arthritis | C0003873 | RIT2 | 6014 | Q99578 | Ras like without CAAX 2 | Enzyme modulator | 46 | 0.647 | 0.538 | 0.000632 | 0.1 | | 1 | 1 | 1 | 2013 | 2013 |
| Rheumatoid Arthritis | C0003873 | RNF5 | 6048 | Q99942 | ring finger protein 5 | Enzyme | 28 | 0.716 | 0.538 | 3.7E-05 | 0.1 | | 1 | 3 | 7 | 2007 | 2011 |
| Rheumatoid Arthritis | C0003873 | RPL37A | 6168 | P61513 | ribosomal protein L37a | | 8 | 0.839 | 0.346 | 0.005126 | 0.1 | | 1 | 1 | 1 | 2016 | 2016 |
| Rheumatoi | C0003 | RPS18 | 6222 | P62269 | ribosomal protein S18 | Nucleic aci | 7 | 0.8 | 0.1 | 0.97 | 0.1 | | 1 | 1 | 1 | 2009 | 2009 |

| | | | | | | | | | | | | | | | | | |
|---|---|---|---|---|---|---|---|---|---|---|---|---|---|---|---|---|---|
| d Arthritis | 873 | | | | | d binding | | 61 | 92 | 59 | | | | | | | |
| Rheumatoid Arthritis | C0003873 | RPS19 | 6223 | P39019 | ribosomal protein S19 | Nucleic acid binding | 256 | 0.48 | 0.846 | 0.92111 | 0.1 | | 1 | 11 | 0 | 2006 | 2020 |
| Rheumatoid Arthritis | C0003873 | S100A8 | 6279 | P05109 | S100 calcium binding protein A8 | Calcium-binding protein | 369 | 0.43 | 0.962 | 0.13761 | 0.1 | | 0.938 | 16 | 0 | 2005 | 2019 |
| Rheumatoid Arthritis | C0003873 | SAA1 | 6288 | P0DJI8 | serum amyloid A1 | Transporter | 188 | 0.513 | 0.808 | 0.01493 | 0.1 | | 0.941 | 17 | 0 | 1986 | 2019 |
| Rheumatoid Arthritis | C0003873 | VPS52 | 6293 | Q8N1B4 | VPS52 subunit of GARP complex | Transporter | 11 | 0.78 | 0.231 | 0.000389 | 0.1 | | 1 | 2 | 2 | 2007 | 2009 |
| Rheumatoid Arthritis | C0003873 | CCL2 | 6347 | P13500 | C-C motif chemokine ligand 2 | Signaling | 1157 | 0.321 | 0.962 | 0.60786 | 0.1 | | 0.942 | 52 | 0 | 1992 | 2019 |
| Rheumatoid Arthritis | C0003873 | CCL5 | 6352 | P13501 | C-C motif chemokine ligand 5 | Signaling | 514 | 0.403 | 0.885 | 0.001752 | 0.1 | | 0.962 | 26 | 0 | 1993 | 2019 |

| | | | | | | | | | | | | | | | | | |
|---|---|---|---|---|---|---|---|---|---|---|---|---|---|---|---|---|---|
| Rheumatoid Arthritis | C0003873 | CCL20 | 6364 | P78556 | C-C motif chemokine ligand 20 | Signaling | 242 | 0.474 | 0.846 | 0.000242 | 0.1 | | 1 | 15 | 0 | 2001 | 2020 |
| Rheumatoid Arthritis | C0003873 | CXCL12 | 6387 | P48061 | C-X-C motif chemokine ligand 12 | | 626 | 0.379 | 0.846 | 0.11851 | 0.1 | | 0.913 | 23 | 0 | 2000 | 2019 |
| Rheumatoid Arthritis | C0003873 | PRDM16 | 63976 | Q9HAZ2 | PR/SET domain 16 | | 200 | 0.522 | 0.808 | 0.99999 | 0.1 | | 1 | 1 | 1 | 2019 | 2019 |
| Rheumatoid Arthritis | C0003873 | SELE | 6401 | P16581 | selectin E | | 327 | 0.447 | 0.808 | 7.91E-10 | 0.1 | | 0.917 | 12 | 0 | 1991 | 2020 |
| Rheumatoid Arthritis | C0003873 | CLSTN2 | 64084 | Q9H4D0 | calsyntenin 2 | Calcium-binding protein | 25 | 0.716 | 0.423 | 3.52E-06 | 0.1 | | 1 | 1 | 1 | 2009 | 2009 |
| Rheumatoid Arthritis | C0003873 | NOD2 | 64127 | Q9HC29 | nucleotide binding oligomerization domain containing 2 | Enzyme | 434 | 0.423 | 0.923 | 1.98E-30 | 0.1 | | 0.818 | 11 | 1 | 2003 | 2019 |
| Rheumatoi | C0003 | LINC00452 | 6433 | | long intergenic non-protein | | 2 | 1 | 0.1 | | 0.1 | | 1 | 1 | 3 | 2013 | 2013 |

| | | | | | | | | | | | | | | | | | |
|---|---|---|---|---|---|---|---|---|---|---|---|---|---|---|---|---|---|
| d Arthritis | 873 | | 65 | | coding RNA 452 | | | | 15 | | | | | | | | |
| Rheumatoid Arthritis | C0003873 | HLA-DPA2 | 646702 | | major histocompatibility complex, class II, DP alpha 2 (pseudogene) | | 10 | 0.78 | 0.538 | | 0.1 | | 1 | 3 | 7 | 2007 | 2011 |
| Rheumatoid Arthritis | C0003873 | GORASP1 | 64689 | Q9BQQ3 | golgi reassembly stacking protein 1 | | 369 | 0.428 | 0.846 | 0.019543 | 0.1 | | 1 | 12 | 0 | 2002 | 2019 |
| Rheumatoid Arthritis | C0003873 | SKIV2L | 6499 | Q15477 | Ski2 like RNA helicase | | 96 | 0.615 | 0.654 | 1.29E-22 | 0.1 | | 1 | 3 | 6 | 2007 | 2011 |
| Rheumatoid Arthritis | C0003873 | WNK1 | 65125 | Q9H4A3 | WNK lysine deficient protein kinase 1 | Kinase | 460 | 0.416 | 0.846 | 1 | 0.1 | | 1 | 12 | 0 | 2002 | 2019 |
| Rheumatoid Arthritis | C0003873 | PCDH15 | 65217 | Q96QU1 | protocadherin related 15 | | 94 | 0.601 | 0.577 | 8.03E-25 | 0.1 | | 1 | 1 | 1 | 2013 | 2013 |
| Rheumatoid Arthritis | C0003873 | CASTOR1 | 652968 | Q8WTX7 | cytosolic arginine sensor for mTORC1 subunit 1 | | 10 | 0.821 | 0.231 | 0.027533 | 0.1 | | 1 | 1 | 1 | 2014 | 2014 |
| Rheu | C00 | SLC6 | 65 | P48066 | solute carrier | Trans | 21 | 0. | 0. | 4.8 | 0.1 | | 1 | 1 | 1 | 2013 | 20 |

| | | | | | | | | | | | | | | | | | |
|---|---|---|---|---|---|---|---|---|---|---|---|---|---|---|---|---|---|
| matoid Arthritis | 03873 | A11 | 38 | | family 6 member 11 | porter | | 716 | 462 | 3E-05 | | | | | | | 13 |
| Rheumatoid Arthritis | C0003873 | SLC19A1 | 6573 | P41440 | solute carrier family 19 member 1 | Transporter | 155 | 0.523 | 0.692 | 6.47E-06 | 0.1 | | 1 | 16 | 7 | 2004 | 2019 |
| Rheumatoid Arthritis | C0003873 | SOAT1 | 6646 | P35610 | sterol O-acyltransferase 1 | Enzyme | 389 | 0.424 | 0.846 | 7.36E-10 | 0.1 | | 1 | 10 | 0 | 2011 | 2019 |
| Rheumatoid Arthritis | C0003873 | SPP1 | 6696 | P10451 | secreted phosphoprotein 1 | | 824 | 0.353 | 0.885 | 2.15E-06 | 0.1 | | 0.923 | 26 | 2 | 2000 | 2019 |
| Rheumatoid Arthritis | C0003873 | STAT3 | 6774 | P40763 | signal transducer and activator of transcription 3 | Nucleic acid binding | 1193 | 0.32 | 0.923 | 1 | 0.1 | | 0.978 | 45 | 0 | 2001 | 2020 |
| Rheumatoid Arthritis | C0003873 | SNORA38 | 677820 | | small nucleolar RNA, H/ACA box 38 | | 6 | 0.89 | 0.154 | | 0.1 | | 1 | 1 | 2 | 2011 | 2011 |
| Rheumatoid Arthritis | C0003873 | BSG | 682 | P35613 | basigin (Ok blood group) | | 287 | 0.458 | 0.769 | 2.59E-05 | 0.1 | | 1 | 13 | 0 | 2000 | 2019 |

| | | | | | | | | | | | | | | | | | |
|---|---|---|---|---|---|---|---|---|---|---|---|---|---|---|---|---|---|
| Rheumatoid Arthritis | C0003873 | SUOX | 6821 | P51687 | sulfite oxidase | | 67 | 0.638 | 0.615 | 9.4E-07 | 0.1 | | 1 | 2 | 1 | 2014 | 2019 |
| Rheumatoid Arthritis | C0003873 | SYT1 | 6857 | P21579 | synaptotagmin 1 | Transporter | 460 | 0.412 | 0.846 | 0.60038 | 0.1 | | 1 | 12 | 0 | 2002 | 2019 |
| Rheumatoid Arthritis | C0003873 | TAPBP | 6892 | O15533 | TAP binding protein | Receptor | 86 | 0.576 | 0.769 | 6.6E-09 | 0.1 | | 1 | 1 | 6 | 2011 | 2011 |
| Rheumatoid Arthritis | C0003873 | BTF3P11 | 690 | | basic transcription factor 3 pseudogene 11 | | 154 | 0.519 | 0.731 | | 0.1 | | 0.941 | 17 | 0 | 2004 | 2019 |
| Rheumatoid Arthritis | C0003873 | TCF19 | 6941 | Q9Y242 | transcription factor 19 | | 33 | 0.674 | 0.692 | 0.000938 | 0.1 | | 1 | 1 | 1 | 2009 | 2009 |
| Rheumatoid Arthritis | C0003873 | BTK | 695 | Q06187 | Bruton tyrosine kinase | Kinase | 290 | 0.464 | 0.808 | 0.999996 | 0.1 | | 1 | 23 | 0 | 2009 | 2019 |
| Rheumatoid Ar | C0003873 | TRBV20OR9-2 | 6962 | | T cell receptor beta variable 20/OR9-2 (non-functional) | | 456 | 0.403 | 0.923 | | 0.1 | | 0.88 | 25 | 0 | 1989 | 2019 |

| | | | | | | | | | | | | | | | | | |
|---|---|---|---|---|---|---|---|---|---|---|---|---|---|---|---|---|---|
| thritis | | | | | | | | | | | | | | | | | |
| Rheumatoid Arthritis | C0003873 | TEC | 7006 | P42680 | tec protein tyrosine kinase | Kinase | 1 | 1 | 0.115 | | 0.1 | | 1 | 1 | 1 | 2014 | 2014 |
| Rheumatoid Arthritis | C0003873 | TGFB1 | 7040 | P01137 | transforming growth factor beta 1 | Signaling | 1558 | 0.287 | 0.962 | 0.03685 | 0.1 | | 0.946 | 37 | 3 | 1990 | 2019 |
| Rheumatoid Arthritis | C0003873 | TIMP1 | 7076 | P01033 | TIMP metallopeptidase inhibitor 1 | Enzyme modulator | 603 | 0.38 | 0.885 | 0.4993 | 0.1 | | 1 | 13 | 0 | 1994 | 2019 |
| Rheumatoid Arthritis | C0003873 | TLR3 | 7098 | O15455 | toll like receptor 3 | Receptor | 393 | 0.427 | 0.885 | 1.63E-07 | 0.1 | | 1 | 10 | 0 | 2005 | 2018 |
| Rheumatoid Arthritis | C0003873 | TLR4 | 7099 | O00206 | toll like receptor 4 | | 1174 | 0.321 | 0.962 | 4.61E-09 | 0.1 | | 0.918 | 61 | 5 | 2003 | 2020 |
| Rheumatoid Arthritis | C0003873 | TNFRSF1A | 7132 | P19438 | TNF receptor superfamily member 1A | | 487 | 0.409 | 0.923 | 0.9949 | 0.1 | | 0.885 | 26 | 7 | 1992 | 2020 |
| Rheumatoi | C0003 | TNFRSF1B | 7133 | P20333 | TNF receptor superfamily member 1B | | 417 | 0.4 | 0.8 | 0.502 | 0.1 | | 0.812 | 32 | 1 | 1992 | 2020 |

| | | | | | | | | | | | | | | | | | |
|---|---|---|---|---|---|---|---|---|---|---|---|---|---|---|---|---|---|
| d Arthritis | 873 | | | | | | | 25 | 85 | 01 | | | | | | | |
| Rheumatoid Arthritis | C0003873 | TP53 | 7157 | P04637 | tumor protein p53 | Transcription factor | 2494 | 0.236 | 0.962 | 0.53235 | 0.1 | | 0.891 | 46 | 5 | 1996 | 2018 |
| Rheumatoid Arthritis | C0003873 | TRPS1 | 7227 | Q9UHF7 | transcriptional repressor GATA binding 1 | | 248 | 0.537 | 0.769 | 1 | 0.1 | | 1 | 1 | 1 | 2013 | 2013 |
| Rheumatoid Arthritis | C0003873 | DCDC2C | 728597 | A8MYV0 | doublecortin domain containing 2C | | 2 | 0.931 | 0.192 | 0.000717 | 0.1 | | 1 | 1 | 1 | 2013 | 2013 |
| Rheumatoid Arthritis | C0003873 | TYMS | 7298 | P04818 | thymidylate synthetase | Enzyme | 406 | 0.425 | 0.846 | 0.72247 | 0.1 | | 1 | 10 | 1 | 2003 | 2018 |
| Rheumatoid Arthritis | C0003873 | VARS1 | 7407 | P26640 | valyl-tRNA synthetase 1 | | 36 | 0.711 | 0.5 | 1.8E-08 | 0.1 | | 1 | 1 | 1 | 2011 | 2011 |
| Rheumatoid Arthritis | C0003873 | VCAM1 | 7412 | P19320 | vascular cell adhesion molecule 1 | | 406 | 0.422 | 0.808 | 0.79791 | 0.1 | | 1 | 27 | 1 | 1995 | 2020 |

| | | | | | | | | | | | | | | | | | |
|---|---|---|---|---|---|---|---|---|---|---|---|---|---|---|---|---|---|
| Rheumatoid Arthritis | C0003873 | VDR | 7421 | P11473 | vitamin D receptor | Nuclear receptor | 852 | 0.352 | 0.885 | 1.68E-05 | 0.1 | | 0.96 | 25 | 6 | 1998 | 2019 |
| Rheumatoid Arthritis | C0003873 | VIM | 7431 | P08670 | vimentin | | 644 | 0.372 | 0.846 | 0.71255 | 0.1 | | 0.973 | 37 | 0 | 2004 | 2020 |
| Rheumatoid Arthritis | C0003873 | VIP | 7432 | P01282 | vasoactive intestinal peptide | Signaling | 376 | 0.437 | 0.846 | 0.00042 | 0.1 | | 1 | 16 | 0 | 1998 | 2019 |
| Rheumatoid Arthritis | C0003873 | TRIM26 | 7726 | Q12899 | tripartite motif containing 26 | | 31 | 0.705 | 0.577 | 0.17836 | 0.1 | | 1 | 2 | 1 | 2007 | 2009 |
| Rheumatoid Arthritis | C0003873 | ZNF175 | 7728 | Q9Y473 | zinc finger protein 175 | | 7 | 0.821 | 0.231 | 1.27E-07 | 0.1 | | 1 | 1 | 1 | 2013 | 2013 |
| Rheumatoid Arthritis | C0003873 | ZSCAN26 | 7741 | Q16670 | zinc finger and SCAN domain containing 26 | | 2 | 0.931 | 0.115 | | 0.1 | | 1 | 1 | 1 | 2009 | 2009 |
| Rheumatoid Ar | C0003873 | DDR1 | 780 | Q08345 | discoidin domain receptor tyrosine kinase 1 | Kinase | 195 | 0.497 | 0.808 | 4.63E- | 0.1 | | 1 | 2 | 1 | 2007 | 2009 |

| | | | | | | | | | | | | | | | | | |
|---|---|---|---|---|---|---|---|---|---|---|---|---|---|---|---|---|---|
| thritis | | | | | | | | | | 06 | | | | | | | |
| Rheumatoid Arthritis | C0003873 | DDA1 | 79016 | Q9BW61 | DET1 and DDB1 associated 1 | | 17 | 0.736 | 0.308 | 0.93718 | 0.1 | | 1 | 1 | 1 | 2014 | 2014 |
| Rheumatoid Arthritis | C0003873 | FTO | 79068 | Q9C0B1 | FTO alpha-ketoglutarate dependent dioxygenase | | 286 | 0.486 | 0.885 | 8.36E-08 | 0.1 | | 1 | 1 | 1 | 2018 | 2018 |
| Rheumatoid Arthritis | C0003873 | BAG6 | 7917 | P46379 | BAG cochaperone 6 | Enzyme modulator | 40 | 0.674 | 0.615 | 1 | 0.1 | | 1 | 3 | 12 | 2007 | 2011 |
| Rheumatoid Arthritis | C0003873 | GPANK1 | 7918 | O95872 | G-patch domain and ankyrin repeats 1 | | 12 | 0.769 | 0.385 | 3.24E-07 | 0.1 | | 1 | 3 | 3 | 2007 | 2011 |
| Rheumatoid Arthritis | C0003873 | HSD17B8 | 7923 | Q92506 | hydroxysteroid 17-beta dehydrogenase 8 | Enzyme | 9 | 0.805 | 0.346 | 3.69E-05 | 0.1 | | 1 | 2 | 2 | 2007 | 2009 |
| Rheumatoid Arthritis | C0003873 | OR2H2 | 7932 | O95918 | olfactory receptor family 2 subfamily H member 2 | G-protein coupled receptor | 1 | 1 | 0.115 | 1.91E-05 | 0.1 | | 1 | 1 | 2 | 2009 | 2009 |
| Rheu | C00 | RNAS | 79 | Q5TBB1 | ribonuclease H2 subunit B | | 115 | 0. | 0. | 3.2 | 0.1 | | 1 | 1 | 1 | 2015 | 20 |

| | | | | | | | | | | | | | | | | | |
|---|---|---|---|---|---|---|---|---|---|---|---|---|---|---|---|---|---|
| matoid Arthritis | 03873 | EH2B | 621 | | | | | 595 | 615 | 6E-09 | | | | | | | 15 |
| Rheumatoid Arthritis | C0003873 | AIMP2 | 7965 | Q13155 | aminoacyl tRNA synthetase complex interacting multifunctional protein 2 | | 555 | 0.393 | 0.923 | 0.008024 | 0.1 | | 1 | 23 | 0 | 2003 | 2019 |
| Rheumatoid Arthritis | C0003873 | AGBL2 | 79841 | Q5U5Z8 | ATP/GTP binding protein like 2 | Enzyme | 55 | 0.644 | 0.654 | 3.96E-16 | 0.1 | | 0.885 | 26 | 1 | 2007 | 2020 |
| Rheumatoid Arthritis | C0003873 | RPP21 | 79897 | Q9H633 | ribonuclease P/MRP subunit p21 | Enzyme | 5 | 0.839 | 0.269 | 4.9E-05 | 0.1 | | 1 | 1 | 1 | 2009 | 2009 |
| Rheumatoid Arthritis | C0003873 | ATAT1 | 79969 | Q5SQI0 | alpha tubulin acetyltransferase 1 | | 31 | 0.7 | 0.5 | 1.32E-07 | 0.1 | | 1 | 2 | 1 | 2007 | 2009 |
| Rheumatoid Arthritis | C0003873 | SPSB1 | 80176 | Q96BD6 | splA/ryanodine receptor domain and SOCS box containing 1 | | 26 | 0.722 | 0.577 | 0.51982 | 0.1 | | 1 | 1 | 1 | 2013 | 2013 |
| Rheumatoid Arthritis | C0003873 | ALPK1 | 80216 | Q96QP1 | alpha kinase 1 | Kinase | 54 | 0.644 | 0.615 | 1.02E-19 | 0.1 | | 1 | 1 | 1 | 2019 | 2019 |

| | | | | | | | | | | | | | | | | | |
|---|---|---|---|---|---|---|---|---|---|---|---|---|---|---|---|---|---|
| Rheumatoid Arthritis | C0003873 | CUL5 | 8065 | Q93034 | cullin 5 | Enzyme | 32 | 0.691 | 0.577 | 1 | 0.1 | | 1 | 1 | 1 | 2019 | 2019 |
| Rheumatoid Arthritis | C0003873 | SLC44A4 | 80736 | Q53GD3 | solute carrier family 44 member 4 | Transporter | 39 | 0.678 | 0.615 | 1E-14 | 0.1 | | 1 | 3 | 9 | 2007 | 2011 |
| Rheumatoid Arthritis | C0003873 | VWA7 | 80737 | Q9Y334 | von Willebrand factor A domain containing 7 | | 6 | 0.839 | 0.308 | 2.31E-11 | 0.1 | | 1 | 1 | 3 | 2011 | 2011 |
| Rheumatoid Arthritis | C0003873 | LY6G6C | 80740 | O95867 | lymphocyte antigen 6 family member G6C | | 7 | 0.821 | 0.385 | 0.000493 | 0.1 | | 1 | 1 | 1 | 2011 | 2011 |
| Rheumatoid Arthritis | C0003873 | LY6G5C | 80741 | Q5SRR4 | lymphocyte antigen 6 family member G5C | | 3 | 0.931 | 0.192 | 0.40546 | 0.1 | | 1 | 2 | 1 | 2007 | 2009 |
| Rheumatoid Arthritis | C0003873 | ZNRD1ASP | 80862 | | zinc ribbon domain containing 1 antisense, pseudogene | | 34 | 0.691 | 0.538 | | 0.1 | | 1 | 2 | 2 | 2007 | 2009 |
| Rheumatoid Ar | C0003873 | PRRT1 | 80863 | Q99946 | proline rich transmembrane protein 1 | | 6 | 0.821 | 0.269 | 0.29845 | 0.1 | | 1 | 1 | 1 | 2011 | 2011 |

| | | | | | | | | | | | | | | | | | |
|---|---|---|---|---|---|---|---|---|---|---|---|---|---|---|---|---|---|
| thritis | | | | | | | | | | | | | | | | | |
| Rheumatoid Arthritis | C0003873 | EGFL8 | 80864 | Q99944 | EGF like domain multiple 8 | Calcium-binding protein | 12 | 0.792 | 0.269 | 1.13E-07 | 0.1 | | 1 | 3 | 2 | 2007 | 2011 |
| Rheumatoid Arthritis | C0003873 | CDR3 | 8163 | | Cerebellar degeneration-related autoantigen-3 | | 233 | 0.472 | 0.808 | | 0.1 | | 0.875 | 16 | 0 | 1992 | 2019 |
| Rheumatoid Arthritis | C0003873 | OR5V1 | 81696 | Q9UGF6 | olfactory receptor family 5 subfamily V member 1 | G-protein coupled receptor | 23 | 0.769 | 0.385 | 0.013153 | 0.1 | | 1 | 1 | 2 | 2009 | 2009 |
| Rheumatoid Arthritis | C0003873 | OR12D3 | 81797 | Q9UGF7 | olfactory receptor family 12 subfamily D member 3 | G-protein coupled receptor | 27 | 0.743 | 0.385 | 0.007853 | 0.1 | | 1 | 1 | 1 | 2009 | 2009 |
| Rheumatoid Arthritis | C0003873 | CASP3 | 836 | P42574 | caspase 3 | Enzyme | 819 | 0.351 | 0.923 | 0.10017 | 0.1 | | 1 | 10 | 0 | 2006 | 2018 |
| Rheumatoid Ar | C0003873 | UQCC2 | 84300 | Q9BRT2 | ubiquinol-cytochrome c reductase complex assembly factor 2 | | 30 | 0.705 | 0.462 | 0.01866 | 0.1 | | 1 | 3 | 10 | 2007 | 2011 |

| | | | | | | | | | | | | | | | | | |
|---|---|---|---|---|---|---|---|---|---|---|---|---|---|---|---|---|---|
| thritis | | | | | | | | | | | | | | | | | |
| Rheumatoid Arthritis | C0003873 | DHX16 | 8449 | O60231 | DEAH-box helicase 16 | Enzyme | 24 | 0.716 | 0.5 | 1.6E-12 | 0.1 | | 1 | 2 | 3 | 2007 | 2009 |
| Rheumatoid Arthritis | C0003873 | PGBD1 | 84547 | Q96JS3 | piggyBac transposable element derived 1 | | 7 | 0.821 | 0.269 | 4.73E-10 | 0.1 | | 1 | 1 | 1 | 2009 | 2009 |
| Rheumatoid Arthritis | C0003873 | HAVCR2 | 84868 | Q8TDQ0 | hepatitis A virus cellular receptor 2 | | 299 | 0.45 | 0.808 | 0.03866 | 0.1 | | 0.857 | 14 | 2 | 2004 | 2019 |
| Rheumatoid Arthritis | C0003873 | AIFM2 | 84883 | Q9BRQ8 | apoptosis inducing factor mitochondria associated 2 | Enzyme | 9 | 0.839 | 0.192 | 1.11E-05 | 0.1 | | 1 | 1 | 1 | 2019 | 2019 |
| Rheumatoid Arthritis | C0003873 | CEP89 | 84902 | Q96ST8 | centrosomal protein 89 | | 9 | 0.931 | 0.192 | 8.35E-26 | 0.1 | | 1 | 1 | 1 | 2011 | 2011 |
| Rheumatoid Arthritis | C0003873 | PPIL4 | 85313 | Q8WUA2 | peptidylprolyl isomerase like 4 | | 6 | 0.861 | 0.154 | 1.69E-09 | 0.1 | | 1 | 2 | 1 | 2014 | 2019 |
| Rheumatoi | C0003 | TNFSF11 | 8600 | O14788 | TNF superfamily | Signaling | 327 | 0.4 | 0.8 | 0.004 | 0.1 | | 1 | 58 | 3 | 1999 | 2020 |

| | | | | | | | | | | | | | | | | | |
|---|---|---|---|---|---|---|---|---|---|---|---|---|---|---|---|---|---|
| d Arthritis | 873 | | | | member 11 | | | 53 | 46 | 916 | | | | | | | |
| Rheumatoid Arthritis | C0003873 | TNFRSF11A | 8792 | Q9Y6Q6 | TNF receptor superfamily member 11a | | 270 | 0.48 | 0.846 | 0.008767 | 0.1 | | 0.867 | 15 | 2 | 2001 | 2019 |
| Rheumatoid Arthritis | C0003873 | SYNGAP1 | 8831 | Q96PV0 | synaptic Ras GTPase activating protein 1 | Enzyme modulator | 145 | 0.584 | 0.577 | 1 | 0.1 | | 1 | 1 | 2 | 2009 | 2009 |
| Rheumatoid Arthritis | C0003873 | STK19 | 8859 | P49842 | serine/threonine kinase 19 | | 28 | 0.691 | 0.5 | 6.74E-09 | 0.1 | | 1 | 3 | 5 | 2007 | 2011 |
| Rheumatoid Arthritis | C0003873 | NAV2 | 89797 | Q8IVL1 | neuron navigator 2 | | 27 | 0.722 | 0.385 | 0.99995 | 0.1 | | 1 | 1 | 1 | 2018 | 2018 |
| Rheumatoid Arthritis | C0003873 | TRIM15 | 89870 | Q9C019 | tripartite motif containing 15 | | 17 | 0.743 | 0.308 | 7.76E-09 | 0.1 | | 1 | 2 | 1 | 2007 | 2009 |
| Rheumatoid Arthritis | C0003873 | CCNG2 | 901 | Q16589 | cyclin G2 | Enzyme modulator | 59 | 0.615 | 0.577 | 0.009686 | 0.1 | | 1 | 1 | 1 | 2013 | 2013 |

| | | | | | | | | | | | | | | | | | |
|---|---|---|---|---|---|---|---|---|---|---|---|---|---|---|---|---|---|
| Rheumatoid Arthritis | C0003873 | SOCS3 | 9021 | O14543 | suppressor of cytokine signaling 3 | Enzyme modulator | 375 | 0.434 | 0.808 | 0.74613 | 0.1 | | 0.9 | 10 | 0 | 2001 | 2019 |
| Rheumatoid Arthritis | C0003873 | IL33 | 90865 | O95760 | interleukin 33 | | 487 | 0.409 | 0.885 | 8.29E-10 | 0.1 | | 1 | 23 | 2 | 2007 | 2019 |
| Rheumatoid Arthritis | C0003873 | RABEP1 | 9135 | Q15276 | rabaptin, RAB GTPase binding effector protein 1 | | 15 | 0.792 | 0.423 | 0.99851 | 0.1 | | 1 | 2 | 1 | 2014 | 2019 |
| Rheumatoid Arthritis | C0003873 | IL32 | 9235 | P24001 | interleukin 32 | | 200 | 0.495 | 0.731 | 3.75E-09 | 0.1 | | 1 | 19 | 1 | 2005 | 2018 |
| Rheumatoid Arthritis | C0003873 | CD14 | 929 | P08571 | CD14 molecule | | 551 | 0.392 | 0.885 | 4.88E-05 | 0.1 | | 0.943 | 35 | 0 | 1992 | 2019 |
| Rheumatoid Arthritis | C0003873 | MS4A1 | 931 | P11836 | membrane spanning 4-domains A1 | Receptor | 451 | 0.411 | 0.846 | 5.08E-05 | 0.1 | | 0.882 | 17 | 0 | 2001 | 2019 |
| Rheumatoid Ar | C0003873 | PPT2 | 9374 | Q9UMR5 | palmitoyl-protein thioesterase 2 | Enzyme | 27 | 0.751 | 0.5 | 0.00045 | 0.1 | | 1 | 3 | 6 | 2007 | 2011 |

| | | | | | | | | | | | | | | | | |
|---|---|---|---|---|---|---|---|---|---|---|---|---|---|---|---|---|
| thritis | | | | | | | | | | 42 | | | | | | |
| Rheumatoid Arthritis | C0003873 | GRAP2 | 9402 | O75791 | GRB2 related adaptor protein 2 | | 538 | 0.394 | 0.923 | 0.000153 | 0.1 | | 1 | 23 | 1 | 2003 | 2019 |
| Rheumatoid Arthritis | C0003873 | FADS2 | 9415 | O95864 | fatty acid desaturase 2 | | 140 | 0.575 | 0.692 | 0.99815 | 0.1 | | 1 | 3 | 1 | 2009 | 2019 |
| Rheumatoid Arthritis | C0003873 | GABBR2 | 9568 | O75899 | gamma-aminobutyric acid type B receptor subunit 2 | G-protein coupled receptor | 81 | 0.65 | 0.538 | 0.99999 | 0.1 | | 1 | 1 | 1 | 2018 | 2018 |
| Rheumatoid Arthritis | C0003873 | CLOCK | 9575 | O15516 | clock circadian regulator | | 309 | 0.463 | 0.885 | 0.99957 | 0.1 | | 1 | 10 | 0 | 2013 | 2020 |
| Rheumatoid Arthritis | C0003873 | CD40LG | 959 | P29965 | CD40 ligand | Signaling | 453 | 0.414 | 0.846 | 0.72079 | 0.1 | | 0.917 | 24 | 0 | 1995 | 2019 |
| Rheumatoid Arthritis | C0003873 | CD44 | 960 | P16070 | CD44 molecule (Indian blood group) | | 711 | 0.363 | 0.962 | 2.45E-07 | 0.1 | | 1 | 12 | 0 | 1997 | 2018 |

| | | | | | | | | | | | | | | | | | |
|---|---|---|---|---|---|---|---|---|---|---|---|---|---|---|---|---|---|
| Rheumatoid Arthritis | C0003873 | MDC1 | 9656 | Q14676 | mediator of DNA damage checkpoint 1 | | 56 | 0.628 | 0.692 | 2.27E-08 | 0.1 | | 1 | 1 | 1 | 2009 | 2009 |
| Rheumatoid Arthritis | C0003873 | CD68 | 968 | P34810 | CD68 molecule | Transporter | 452 | 0.408 | 0.808 | 1.41E-06 | 0.1 | | 1 | 14 | 0 | 1989 | 2015 |
| Rheumatoid Arthritis | C0003873 | N4BP1 | 9683 | O75113 | NEDD4 binding protein 1 | Nucleic acid binding | 12 | 0.78 | 0.308 | 0.620056 | 0.1 | | 1 | 1 | 1 | 2013 | 2013 |
| Rheumatoid Arthritis | C0003873 | PRORP | 9692 | O15091 | protein only RNase P catalytic subunit | | 12 | 0.769 | 0.269 | 1.33E-13 | 0.1 | | 1 | 1 | 1 | 2018 | 2018 |
| Rheumatoid Arthritis | C0003873 | RIPOR2 | 9750 | Q9Y4F9 | RHO family interacting cell polarization regulator 2 | | 20 | 0.792 | 0.269 | 0.92284 | 0.1 | | 1 | 1 | 1 | 2009 | 2009 |
| Rheumatoid Arthritis | C0003873 | CTIF | 9811 | O43310 | cap binding complex dependent translation initiation factor | Nucleic acid binding | 10 | 0.821 | 0.231 | 0.99994 | 0.1 | | 1 | 1 | 1 | 2009 | 2009 |
| Rheumatoid | C0003873 | SMG7 | 9887 | Q92540 | SMG7 nonsense mediated mRNA decay factor | | 10 | 0.805 | 0.346 | 0.999 | 0.1 | | 1 | 2 | 1 | 2016 | 2019 |

| | | | | | | | | | | | | | | | | | |
|---|---|---|---|---|---|---|---|---|---|---|---|---|---|---|---|---|---|
| Arthritis | | | | | | | | | | 98 | | | | | | | |
| Rheumatoid Arthritis | C0003873 | TNFSF15 | 9966 | O95150 | TNF superfamily member 15 | Signaling | 143 | 0.531 | 0.692 | 0.002816 | 0.1 | | 1 | 12 | 3 | 2007 | 2019 |
| Rheumatoid Arthritis | C0003873 | EBI3 | 10148 | Q14213 | Epstein-Barr virus induced 3 | Signaling | 233 | 0.474 | 0.808 | 0.10797 | 0.09 | | 1 | 9 | 0 | 2004 | 2019 |
| Rheumatoid Arthritis | C0003873 | CDKN2A | 1029 | P42771;Q8N726 | cyclin dependent kinase inhibitor 2A | | 1314 | 0.3 | 0.885 | 0.39474 | 0.09 | | 1 | 9 | 1 | 1989 | 2018 |
| Rheumatoid Arthritis | C0003873 | CNTRL | 11064 | Q7Z7A1 | centriolin | | 29 | 0.711 | 0.423 | 1.89E-54 | 0.09 | | 0.889 | 9 | 0 | 2011 | 2019 |
| Rheumatoid Arthritis | C0003873 | CDC42EP1 | 11135 | Q00587 | CDC42 effector protein 1 | | 17 | 0.751 | 0.346 | 2.54E-05 | 0.09 | | 0.889 | 9 | 0 | 2011 | 2019 |
| Rheumatoid Arthritis | C0003873 | COMP | 1311 | P49747 | cartilage oligomeric matrix protein | | 230 | 0.503 | 0.808 | 1.35E-09 | 0.09 | | 1 | 9 | 0 | 1997 | 2017 |
| Rheuma | C0003 | PARP1 | 142 | P09874 | poly(ADP-ribose) polymerase 1 | | 565 | 0.3 | 0.9 | 0.00 | 0.09 | | 0.7 | 9 | 3 | 2003 | 20 |

| | | | | | | | | | | | | | | | | | |
|---|---|---|---|---|---|---|---|---|---|---|---|---|---|---|---|---|---|
| toid Arthritis | 873 | | | | | | | 89 | 23 | 0334 | | | 78 | | | | 15 |
| Rheumatoid Arthritis | C0003873 | CX3CR1 | 1524 | P49238 | C-X3-C motif chemokine receptor 1 | G-protein coupled receptor | 310 | 0.457 | 0.885 | 0.061371 | 0.09 | | 0.889 | 9 | 0 | 2001 | 2018 |
| Rheumatoid Arthritis | C0003873 | ACE | 1636 | P12821 | angiotensin I converting enzyme | Enzyme | 1082 | 0.328 | 0.923 | 1.03E-37 | 0.09 | | 1 | 9 | 0 | 2000 | 2020 |
| Rheumatoid Arthritis | C0003873 | FCGR2B | 2213 | P31994 | Fc fragment of IgG receptor IIb | Cell adhesion | 155 | 0.522 | 0.846 | 0.456337 | 0.09 | | 0.778 | 9 | 2 | 2002 | 2017 |
| Rheumatoid Arthritis | C0003873 | FOXO3 | 2309 | O43524 | forkhead box O3 | | 381 | 0.431 | 0.808 | 0.98806 | 0.09 | | 0.889 | 9 | 1 | 2008 | 2019 |
| Rheumatoid Arthritis | C0003873 | FOS | 2353 | P01100 | Fos proto-oncogene, AP-1 transcription factor subunit | Transcription factor | 528 | 0.411 | 0.885 | 0.26176 | 0.09 | | 1 | 9 | 0 | 1992 | 2017 |
| Rheumatoid Ar | C0003873 | HAVCR1 | 26762 | Q96D42 | hepatitis A virus cellular receptor 1 | | 217 | 0.489 | 0.885 | 8.51E-07 | 0.09 | | 1 | 9 | 1 | 2004 | 2019 |

| | | | | | | | | | | | | | | | | | |
|---|---|---|---|---|---|---|---|---|---|---|---|---|---|---|---|---|---|
| thritis | | | | | | | | | | | | | | | | | |
| Rheumatoid Arthritis | C0003873 | HLA-DMA | 3108 | P28067 | major histocompatibility complex, class II, DM alpha | Immune response | 63 | 0.608 | 0.654 | 0.002381 | 0.09 | | 0.556 | 9 | 0 | 1997 | 2013 |
| Rheumatoid Arthritis | C0003873 | IL3 | 3562 | P08700 | interleukin 3 | | 211 | 0.489 | 0.731 | 2.59E-09 | 0.09 | | 0.889 | 9 | 2 | 1988 | 2018 |
| Rheumatoid Arthritis | C0003873 | JUN | 3725 | P05412 | Jun proto-oncogene, AP-1 transcription factor subunit | Transcription factor | 344 | 0.442 | 0.885 | 0.059962 | 0.09 | | 1 | 9 | 0 | 1996 | 2019 |
| Rheumatoid Arthritis | C0003873 | CX3CL1 | 6376 | P78423 | C-X3-C motif chemokine ligand 1 | Signaling | 243 | 0.478 | 0.808 | 0.608337 | 0.09 | | 0.889 | 9 | 0 | 1999 | 2018 |
| Rheumatoid Arthritis | C0003873 | THBS1 | 7057 | P07996 | thrombospondin 1 | | 480 | 0.407 | 0.885 | 0.99985 | 0.09 | | 1 | 9 | 0 | 2006 | 2018 |
| Rheumatoid Arthritis | C0003873 | CCR2 | 729230 | P41597 | C-C motif chemokine receptor 2 | | 436 | 0.418 | 0.846 | 0.023412 | 0.09 | | 0.889 | 9 | 0 | 2003 | 2019 |
| Rheumatoi | C0003 | LRPPRC | 101 | P42704 | leucine rich pentatricopeptide repeat containing | | 220 | 0.4 | 0.7 | 2.3E- | 0.08 | | 0.875 | 8 | 0 | 2000 | 2020 |

| | | | | | | | | | | | | | | | | | |
|---|---|---|---|---|---|---|---|---|---|---|---|---|---|---|---|---|---|
| d Arthritis | 873 | | 28 | | | | | 93 | 69 | 10 | | | | | | | |
| Rheumatoid Arthritis | C0003873 | FSTL1 | 11167 | Q12841 | follistatin like 1 | Enzyme modulator | 128 | 0.541 | 0.808 | 0.89403 | 0.08 | | 0.875 | 8 | 0 | 1998 | 2018 |
| Rheumatoid Arthritis | C0003873 | LINC02605 | 1.13E+08 | | long intergenic non-protein coding RNA 2605 | | 203 | 0.49 | 0.769 | | 0.08 | | 1 | 8 | 0 | 2005 | 2018 |
| Rheumatoid Arthritis | C0003873 | CSF1 | 1435 | P09603 | colony stimulating factor 1 | | 259 | 0.473 | 0.808 | 0.99634 | 0.08 | | 1 | 8 | 0 | 1988 | 2019 |
| Rheumatoid Arthritis | C0003873 | CARD8 | 22900 | Q9Y2G2 | caspase recruitment domain family member 8 | | 78 | 0.59 | 0.731 | 1.77E-06 | 0.08 | | 0.75 | 8 | 1 | 2007 | 2018 |
| Rheumatoid Arthritis | C0003873 | IL4R | 3566 | P24394 | interleukin 4 receptor | Receptor | 242 | 0.474 | 0.846 | 0.00023 | 0.08 | | 1 | 8 | 4 | 2006 | 2014 |
| Rheumatoid Arthritis | C0003873 | IL7R | 3575 | P16871 | interleukin 7 receptor | Receptor | 231 | 0.487 | 0.769 | 3.76E-07 | 0.08 | | 1 | 8 | 2 | 2009 | 2019 |

| | | | | | | | | | | | | | | | | | |
|---|---|---|---|---|---|---|---|---|---|---|---|---|---|---|---|---|---|
| Rheumatoid Arthritis | C0003873 | MMP8 | 4317 | P22894 | matrix metallopeptidase 8 | Enzyme | 213 | 0.488 | 0.808 | 1.07E-24 | 0.08 | | 1 | 8 | 0 | 1999 | 2019 |
| Rheumatoid Arthritis | C0003873 | WG | 474168 | | Wegener granulomatosis | | 61 | 0.595 | 0.808 | | 0.08 | | 1 | 8 | 0 | 2009 | 2019 |
| Rheumatoid Arthritis | C0003873 | PRKN | 5071 | O60260 | parkin RBR E3 ubiquitin protein ligase | Enzyme | 409 | 0.431 | 0.846 | 6.93E-07 | 0.08 | | 1 | 8 | 0 | 2005 | 2019 |
| Rheumatoid Arthritis | C0003873 | SOST | 50964 | Q9BQB4 | sclerostin | | 270 | 0.474 | 0.808 | 0.86731 | 0.08 | | 0.875 | 8 | 0 | 2017 | 2020 |
| Rheumatoid Arthritis | C0003873 | TREM1 | 54210 | Q9NP99 | triggering receptor expressed on myeloid cells 1 | | 172 | 0.515 | 0.808 | 0.006278 | 0.08 | | 0.875 | 8 | 0 | 2009 | 2019 |
| Rheumatoid Arthritis | C0003873 | MAPK3 | 5595 | P27361 | mitogen-activated protein kinase 3 | Kinase | 647 | 0.379 | 0.885 | 0.036884 | 0.08 | | 1 | 8 | 0 | 2006 | 2019 |
| Rheumatoid Ar | C0003873 | MYDGF | 56005 | Q969H8 | myeloid derived growth factor | | 295 | 0.451 | 0.846 | 3.49E- | 0.08 | | 1 | 8 | 0 | 2007 | 2019 |

| | | | | | | | | | | | | | | | | | |
|---|---|---|---|---|---|---|---|---|---|---|---|---|---|---|---|---|---|
| thritis | | | | | | | | | | 06 | | | | | | | |
| Rheumatoid Arthritis | C0003873 | PTX3 | 5806 | P26022 | pentraxin 3 | | 296 | 0.457 | 0.808 | 6.98E-05 | 0.08 | | 0.875 | 8 | 0 | 2000 | 2020 |
| Rheumatoid Arthritis | C0003873 | RFC1 | 5981 | P35251 | replication factor C subunit 1 | | 144 | 0.541 | 0.808 | 0.97305 | 0.08 | | 1 | 8 | 0 | 2007 | 2019 |
| Rheumatoid Arthritis | C0003873 | CXCR5 | 643 | P32302 | C-X-C motif chemokine receptor 5 | G-protein coupled receptor | 146 | 0.524 | 0.769 | 0.72861 | 0.08 | | 1 | 8 | 0 | 2005 | 2020 |
| Rheumatoid Arthritis | C0003873 | ADAM17 | 6868 | P78536 | ADAM metallopeptidase domain 17 | | 316 | 0.453 | 0.808 | 0.99528 | 0.08 | | 1 | 8 | 0 | 2001 | 2019 |
| Rheumatoid Arthritis | C0003873 | TPO | 7173 | P07202 | thyroid peroxidase | Enzyme | 306 | 0.455 | 0.846 | 1.82E-21 | 0.08 | | 0.875 | 8 | 0 | 2017 | 2020 |
| Rheumatoid Arthritis | C0003873 | C3 | 718 | P01024 | complement C3 | Enzyme modulator | 343 | 0.445 | 0.885 | 0.90421 | 0.08 | | 1 | 8 | 0 | 1986 | 2018 |
| Rheu | C00 | ARHG | 79 | Q12774 | Rho guanine nucleotide | | 140 | 0. | 0. | 7.5 | 0.08 | | 1 | 8 | 0 | 2004 | 20 |

| | | | | | | | | | | | | | | | | | |
|---|---|---|---|---|---|---|---|---|---|---|---|---|---|---|---|---|---|
| matoid Arthritis | 03873 | EF5 | 84 | | exchange factor 5 | | | 526 | 808 | 9E-14 | | | | | | | 19 |
| Rheumatoid Arthritis | C0003873 | SYVN1 | 84447 | Q86TM6 | synoviolin 1 | | 79 | 0.599 | 0.731 | 0.9997 | 0.08 | | 1 | 8 | 0 | 2003 | 2019 |
| Rheumatoid Arthritis | C0003873 | TNFRSF6B | 8771 | O95407 | TNF receptor superfamily member 6b | | 145 | 0.528 | 0.731 | 3.87E-06 | 0.08 | | 1 | 8 | 0 | 2007 | 2018 |
| Rheumatoid Arthritis | C0003873 | IL18R1 | 8809 | Q13478 | interleukin 18 receptor 1 | Receptor | 234 | 0.473 | 0.808 | 8.63E-06 | 0.08 | | 0.875 | 8 | 0 | 2003 | 2019 |
| Rheumatoid Arthritis | C0003873 | TIMELESS | 8914 | Q9UNS1 | timeless circadian regulator | | 140 | 0.533 | 0.808 | 9.85E-35 | 0.08 | | 1 | 8 | 0 | 2004 | 2019 |
| Rheumatoid Arthritis | C0003873 | PTGES | 9536 | O14684 | prostaglandin E synthase | | 150 | 0.54 | 0.769 | 0.71317 | 0.08 | | 1 | 8 | 0 | 2003 | 2017 |
| Rheumatoid Arthritis | C0003873 | SEMA3A | 10371 | Q14563 | semaphorin 3A | Signaling | 202 | 0.5 | 0.808 | 0.9779 | 0.07 | | 1 | 7 | 0 | 2013 | 2020 |

| | | | | | | | | | | | | | | | | | |
|---|---|---|---|---|---|---|---|---|---|---|---|---|---|---|---|---|---|
| Rheumatoid Arthritis | C0003873 | ANP32B | 10541 | Q92688 | acidic nuclear phosphoprotein 32 family member B | Enzyme modulator | 120 | 0.541 | 0.731 | 0.95827 | 0.07 | | 1 | 7 | 0 | 2005 | 2019 |
| Rheumatoid Arthritis | C0003873 | CDAN1 | 146059 | Q8IWY9 | codanin 1 | | 77 | 0.61 | 0.731 | 1.11E-12 | 0.07 | | 1 | 7 | 0 | 2017 | 2020 |
| Rheumatoid Arthritis | C0003873 | CTSK | 1513 | P43235 | cathepsin K | Enzyme | 221 | 0.497 | 0.808 | 3.1E-05 | 0.07 | | 1 | 7 | 0 | 1997 | 2020 |
| Rheumatoid Arthritis | C0003873 | CBLL2 | 158506 | Q8N7E2 | Cbl proto-oncogene like 2 | Enzyme | 235 | 0.476 | 0.808 | | 0.07 | | 1 | 7 | 0 | 2005 | 2019 |
| Rheumatoid Arthritis | C0003873 | ETS1 | 2113 | P14921 | ETS proto-oncogene 1, transcription factor | Transcription factor | 327 | 0.444 | 0.731 | 0.7816 | 0.07 | | 1 | 7 | 2 | 2001 | 2017 |
| Rheumatoid Arthritis | C0003873 | F2RL1 | 2150 | P55085 | F2R like trypsin receptor 1 | G-protein coupled receptor | 256 | 0.48 | 0.769 | 0.000148 | 0.07 | | 1 | 7 | 0 | 2007 | 2018 |
| Rheumatoi | C0003 | IFNL1 | 2826 | Q8IU54 | interferon lambda 1 | | 73 | 0.5 | 0.6 | 0.000 | 0.07 | | 0.714 | 7 | 0 | 2013 | 2020 |

| | | | | | | | | | | | | | | | | | |
|---|---|---|---|---|---|---|---|---|---|---|---|---|---|---|---|---|---|
| d Arthritis | 873 | | 18 | | | | | 99 | 54 | 285 | | | | | | | |
| Rheumatoid Arthritis | C0003873 | ANGPT2 | 285 | O15123 | angiopoietin 2 | Signaling | 457 | 0.41 | 0.846 | 0.83208 | 0.07 | | 1 | 7 | 5 | 2002 | 2019 |
| Rheumatoid Arthritis | C0003873 | CD274 | 29126 | Q9NZQ7 | CD274 molecule | Receptor | 1011 | 0.324 | 0.923 | 0.01916 | 0.07 | | 0.857 | 7 | 0 | 2007 | 2018 |
| Rheumatoid Arthritis | C0003873 | ICOS | 29851 | Q9Y6W8 | inducible T cell costimulator | | 193 | 0.502 | 0.731 | 0.031284 | 0.07 | | 1 | 7 | 0 | 2003 | 2018 |
| Rheumatoid Arthritis | C0003873 | CFH | 3075 | P08603 | complement factor H | | 393 | 0.441 | 0.885 | 0.86061 | 0.07 | | 0.857 | 7 | 2 | 1990 | 2012 |
| Rheumatoid Arthritis | C0003873 | IL12B | 3593 | P29460 | interleukin 12B | Signaling | 249 | 0.48 | 0.885 | 0.000114 | 0.07 | | 0.714 | 7 | 1 | 2005 | 2017 |
| Rheumatoid Arthritis | C0003873 | KIR2DS1 | 3806 | Q14954 | killer cell immunoglobulin like receptor, two Ig domains and short | Receptor | 123 | 0.537 | 0.769 | | 0.07 | | 1 | 7 | 0 | 2006 | 2019 |

| | | | | | | | | | | | | | | | | | |
|---|---|---|---|---|---|---|---|---|---|---|---|---|---|---|---|---|---|
| | | | | | cytoplasmic tail 1 | | | | | | | | | | | | |
| Rheumatoid Arthritis | C0003873 | LPA | 4018 | P08519 | lipoprotein(a) | Enzyme | 340 | 0.444 | 0.769 | 6.59E-89 | 0.07 | | 1 | 7 | 0 | 1995 | 2019 |
| Rheumatoid Arthritis | C0003873 | MIR21 | 4069991 | | microRNA 21 | | 726 | 0.363 | 0.846 | | 0.07 | | 1 | 7 | 0 | 2014 | 2019 |
| Rheumatoid Arthritis | C0003873 | NFE2L2 | 4780 | Q16236 | nuclear factor, erythroid 2 like 2 | Enzyme | 823 | 0.357 | 0.885 | 0.003572 | 0.07 | | 1 | 7 | 0 | 2016 | 2019 |
| Rheumatoid Arthritis | C0003873 | NOS2 | 4843 | P35228 | nitric oxide synthase 2 | | 783 | 0.364 | 0.923 | 4.03E-15 | 0.07 | | 0.714 | 7 | 1 | 2002 | 2019 |
| Rheumatoid Arthritis | C0003873 | OSM | 5008 | P13725 | oncostatin M | Signaling | 188 | 0.505 | 0.769 | 0.58916 | 0.07 | | 1 | 7 | 0 | 2000 | 2015 |
| Rheumatoid Arthritis | C0003873 | P2RX7 | 5027 | Q99572 | purinergic receptor P2X 7 | Ion channel | 337 | 0.45 | 0.808 | 3.33E-11 | 0.07 | | 0.857 | 7 | 2 | 2008 | 2019 |
| Rheuma | C0003 | SERPI | 50 | P05121 | serpin family E | Enzyme | 770 | 0.3 | 0.8 | 0.04 | 0.07 | | 1 | 7 | 0 | 2005 | 20 |

| | | | | | | | | | | | | | | | | | |
|---|---|---|---|---|---|---|---|---|---|---|---|---|---|---|---|---|---|
| toid Arthritis | 873 | NE1 | 54 | | member 1 | modulator | | 59 | 85 | 3609 | | | | | | | 18 |
| Rheumatoid Arthritis | C0003873 | MBL3P | 50639 | | mannose-binding lectin family member 3, pseudogene | | 152 | 0.518 | 0.769 | | 0.07 | | 0.857 | 7 | 0 | 2000 | 2011 |
| Rheumatoid Arthritis | C0003873 | PML | 5371 | P29590 | promyelocytic leukemia | | 274 | 0.477 | 0.846 | 0.21291 | 0.07 | | 1 | 7 | 0 | 1998 | 2018 |
| Rheumatoid Arthritis | C0003873 | PPARG | 5468 | P37231 | peroxisome proliferator activated receptor gamma | Nuclear receptor | 877 | 0.358 | 0.885 | 0.029194 | 0.07 | | 1 | 7 | 2 | 2002 | 2019 |
| Rheumatoid Arthritis | C0003873 | S100A9 | 6280 | P06702 | S100 calcium binding protein A9 | Calcium-binding protein | 363 | 0.433 | 0.885 | 0.4777 | 0.07 | | 0.857 | 7 | 0 | 1990 | 2019 |
| Rheumatoid Arthritis | C0003873 | SAA2 | 6289 | P0DJI9 | serum amyloid A2 | Transporter | 103 | 0.568 | 0.731 | 2.7E-06 | 0.07 | | 1 | 7 | 0 | 1998 | 2004 |
| Rheumatoid Ar | C0003873 | CCL3 | 6348 | P10147 | C-C motif chemokine ligand 3 | Signaling | 282 | 0.46 | 0.885 | 0.34981 | 0.07 | | 1 | 7 | 0 | 1999 | 2019 |

| | | | | | | | | | | | | | | | | | |
|---|---|---|---|---|---|---|---|---|---|---|---|---|---|---|---|---|---|
| thritis | | | | | | | | | | | | | | | | | |
| Rheumatoid Arthritis | C0003873 | TRIM21 | 6737 | P19474 | tripartite motif containing 21 | | 229 | 0.485 | 0.808 | 3.35E-09 | 0.07 | | 1 | 7 | 0 | 1985 | 2018 |
| Rheumatoid Arthritis | C0003873 | TAC1 | 6863 | P20366 | tachykinin precursor 1 | | 478 | 0.424 | 0.923 | 0.74608 | 0.07 | | 1 | 7 | 0 | 1998 | 2018 |
| Rheumatoid Arthritis | C0003873 | TLR5 | 7100 | O60602 | toll like receptor 5 | | 163 | 0.518 | 0.885 | 2.48E-13 | 0.07 | | 1 | 7 | 1 | 2010 | 2019 |
| Rheumatoid Arthritis | C0003873 | C4B | 721 | P0C0L4;P0C0L5 | complement C4B (Chido blood group) | Enzyme modulator | 66 | 0.603 | 0.808 | 0.834664 | 0.07 | | 1 | 7 | 0 | 1989 | 2012 |
| Rheumatoid Arthritis | C0003873 | MUL1 | 79594 | Q969V5 | mitochondrial E3 ubiquitin protein ligase 1 | | 241 | 0.473 | 0.808 | 1.91E-06 | 0.07 | | 1 | 7 | 0 | 2005 | 2019 |
| Rheumatoid Arthritis | C0003873 | CALR | 811 | P27797 | calreticulin | Calcium-binding protein | 487 | 0.413 | 0.923 | 0.89133 | 0.07 | | 1 | 7 | 0 | 1985 | 2019 |

| | | | | | | | | | | | | | | | | | |
|---|---|---|---|---|---|---|---|---|---|---|---|---|---|---|---|---|---|
| Rheumatoid Arthritis | C0003873 | CASP1 | 834 | P29466 | caspase 1 | Enzyme | 444 | 0.413 | 0.885 | 3.52E-05 | 0.07 | | 1 | 7 | 0 | 2010 | 2019 |
| Rheumatoid Arthritis | C0003873 | TNFSF13 | 8741 | O75888 | TNF superfamily member 13 | | 189 | 0.499 | 0.769 | 0.81724 | 0.07 | | 1 | 7 | 0 | 2005 | 2019 |
| Rheumatoid Arthritis | C0003873 | CD19 | 930 | P15391 | CD19 molecule | | 365 | 0.43 | 0.885 | 0.90496 | 0.07 | | 1 | 7 | 0 | 1996 | 2016 |
| Rheumatoid Arthritis | C0003873 | CD34 | 947 | P28906 | CD34 molecule | | 674 | 0.368 | 0.808 | 4.99E-06 | 0.07 | | 0.857 | 7 | 0 | 2004 | 2019 |
| Rheumatoid Arthritis | C0003873 | CD69 | 969 | Q07108 | CD69 molecule | | 206 | 0.488 | 0.923 | 0.0296691 | 0.07 | | 1 | 7 | 0 | 2001 | 2019 |
| Rheumatoid Arthritis | C0003873 | C4B_2 | 1E+08 | P0C0L4;P0C0L5 | complement component 4B (Chido blood group), copy 2 | | 31 | 0.67 | 0.731 | | 0.06 | | 1 | 6 | 0 | 1989 | 1992 |
| Rheumatoid Ar | C0003873 | KLRC4-KLRK1 | 1.01E+ | P26718 | KLRC4-KLRK1 readthrough | | 246 | 0.469 | 0.769 | | 0.06 | | 1 | 6 | 0 | 2007 | 2018 |

| | | | | | | | | | | | | | | | | | |
|---|---|---|---|---|---|---|---|---|---|---|---|---|---|---|---|---|---|
| thritis | | | 08 | | | | | | | | | | | | | | |
| Rheumatoid Arthritis | C0003873 | ADM | 133 | P35318 | adrenomedullin | Signaling | 405 | 0.436 | 0.808 | 0.040831 | 0.06 | | 1 | 6 | 0 | 2004 | 2014 |
| Rheumatoid Arthritis | C0003873 | HT | 140805 | | Hashimoto thyroiditis | | 170 | 0.505 | 0.731 | | 0.06 | | 0.833 | 6 | 0 | 2004 | 2019 |
| Rheumatoid Arthritis | C0003873 | DMD | 1756 | P11532 | dystrophin | | 484 | 0.423 | 0.923 | 1 | 0.06 | | 1 | 6 | 0 | 2002 | 2019 |
| Rheumatoid Arthritis | C0003873 | ESR2 | 2100 | Q92731 | estrogen receptor 2 | Nuclear receptor | 528 | 0.4 | 0.923 | 4.45E-08 | 0.06 | | 1 | 6 | 1 | 2009 | 2018 |
| Rheumatoid Arthritis | C0003873 | KLRK1 | 22914 | P26718 | killer cell lectin like receptor K1 | | 255 | 0.467 | 0.769 | 6.98E-14 | 0.06 | | 1 | 6 | 0 | 2007 | 2018 |
| Rheumatoid Arthritis | C0003873 | TBC1D9 | 23158 | Q6ZT07 | TBC1 domain family member 9 | Enzyme | 491 | 0.399 | 0.846 | 2.8E-05 | 0.06 | | 1 | 6 | 0 | 1995 | 2015 |
| Rheumatoi | C0003 | FLT1 | 2321 | P17948 | fms related receptor | Kinase | 424 | 0.4 | 0.8 | 0.999 | 0.06 | | 1 | 6 | 2 | 2006 | 2019 |

| | | | | | | | | | | | | | | | | | |
|---|---|---|---|---|---|---|---|---|---|---|---|---|---|---|---|---|---|
| d Arthritis | 873 | | | | tyrosine kinase 1 | | | 19 | 46 | 83 | | | | | | | |
| Rheumatoid Arthritis | C0003873 | FOLR2 | 2350 | P14207 | folate receptor beta | | 105 | 0.559 | 0.654 | 5.48E-06 | 0.06 | | 1 | 6 | 0 | 1999 | 2019 |
| Rheumatoid Arthritis | C0003873 | IL17RA | 23765 | Q96F46 | interleukin 17 receptor A | | 159 | 0.529 | 0.808 | 0.000356 | 0.06 | | 1 | 6 | 0 | 2002 | 2019 |
| Rheumatoid Arthritis | C0003873 | GABPA | 2551 | Q06546 | GA binding protein transcription factor subunit alpha | Transcription factor | 632 | 0.379 | 0.885 | 0.99812 | 0.06 | | 1 | 6 | 0 | 2016 | 2019 |
| Rheumatoid Arthritis | C0003873 | GCG | 2641 | P01275 | glucagon | | 441 | 0.431 | 0.885 | 0.034814 | 0.06 | | 0.833 | 6 | 0 | 2013 | 2019 |
| Rheumatoid Arthritis | C0003873 | GLP1R | 2740 | P43220 | glucagon like peptide 1 receptor | G-protein coupled receptor | 288 | 0.471 | 0.846 | 0.29347 | 0.06 | | 1 | 6 | 0 | 2017 | 2019 |
| Rheumatoid Arthritis | C0003873 | ANGPT1 | 284 | Q15389 | angiopoietin 1 | Signaling | 340 | 0.446 | 0.808 | 0.95208 | 0.06 | | 1 | 6 | 0 | 2002 | 2012 |

| | | | | | | | | | | | | | | | | | |
|---|---|---|---|---|---|---|---|---|---|---|---|---|---|---|---|---|---|
| Rheumatoid Arthritis | C0003873 | IGHV3-69-1 | 28402 | | immunoglobulin heavy variable 3-69-1 (pseudogene) | | 102 | 0.561 | 0.654 | | 0.06 | | 1 | 6 | 0 | 1995 | 2007 |
| Rheumatoid Arthritis | C0003873 | GZMB | 3002 | P10144 | granzyme B | Enzyme | 290 | 0.453 | 0.808 | 3.67E-14 | 0.06 | | 1 | 6 | 1 | 2003 | 2018 |
| Rheumatoid Arthritis | C0003873 | HP | 3240 | P00738 | haptoglobin | Enzyme | 464 | 0.412 | 0.885 | 1.74E-05 | 0.06 | | 1 | 6 | 0 | 1985 | 2017 |
| Rheumatoid Arthritis | C0003873 | AGBL3 | 340351 | Q8NEM8 | ATP/GTP binding protein like 3 | | 5 | 0.861 | 0.115 | 4.18E-16 | 0.06 | | 1 | 6 | 0 | 2017 | 2019 |
| Rheumatoid Arthritis | C0003873 | IL16 | 3603 | Q14005 | interleukin 16 | Signaling | 193 | 0.501 | 0.846 | 1.24E-10 | 0.06 | | 1 | 6 | 0 | 2000 | 2010 |
| Rheumatoid Arthritis | C0003873 | IRF4 | 3662 | Q15306 | interferon regulatory factor 4 | Transcription factor | 203 | 0.491 | 0.769 | 0.85677 | 0.06 | | 1 | 6 | 0 | 2014 | 2018 |
| Rheumatoid Ar | C0003873 | ITGAV | 3685 | P06756 | integrin subunit alpha V | | 141 | 0.526 | 0.769 | 0.0002 | 0.06 | | 1 | 6 | 4 | 2007 | 2017 |

| | | | | | | | | | | | | | | | | | |
|---|---|---|---|---|---|---|---|---|---|---|---|---|---|---|---|---|---|
| thritis | | | | | | | | | | 28 | | | | | | | |
| Rheumatoid Arthritis | C0003873 | KIR3DL2 | 3812 | P43630 | killer cell immunoglobulin like receptor, three Ig domains and long cytoplasmic tail 2 | Receptor | 118 | 0.543 | 0.769 | 6.08E-12 | 0.06 | | 0.833 | 6 | 0 | 2006 | 2019 |
| Rheumatoid Arthritis | C0003873 | LAIR1 | 3903 | Q6GTX8 | leukocyte associated immunoglobulin like receptor 1 | Receptor | 89 | 0.569 | 0.692 | 3.26E-07 | 0.06 | | 1 | 6 | 0 | 1995 | 2018 |
| Rheumatoid Arthritis | C0003873 | LGALS9 | 3965 | O00182 | galectin 9 | Signaling | 197 | 0.496 | 0.769 | 6.55E-06 | 0.06 | | 1 | 6 | 2 | 2007 | 2019 |
| Rheumatoid Arthritis | C0003873 | LIF | 3976 | P15018 | LIF interleukin 6 family cytokine | Signaling | 236 | 0.483 | 0.769 | 0.5242 | 0.06 | | 1 | 6 | 0 | 1992 | 2019 |
| Rheumatoid Arthritis | C0003873 | MDK | 4192 | P21741 | midkine | Signaling | 209 | 0.494 | 0.808 | 0.23005 | 0.06 | | 1 | 6 | 0 | 2004 | 2020 |
| Rheumatoid Ar | C0003873 | MYD88 | 4615 | Q99836 | MYD88 innate immune signal transduction adaptor | Enzyme modulator | 480 | 0.414 | 0.923 | 0.12443 | 0.06 | | 1 | 6 | 0 | 2006 | 2019 |

| | | | | | | | | | | | | | | | | | |
|---|---|---|---|---|---|---|---|---|---|---|---|---|---|---|---|---|---|
| thritis | | | | | | | | | | | | | | | | | |
| Rheumatoid Arthritis | C0003873 | NOTCH1 | 4851 | P46531 | notch receptor 1 | | 693 | 0.369 | 0.885 | 1 | 0.06 | | 1 | 6 | 0 | 2001 | 2018 |
| Rheumatoid Arthritis | C0003873 | IL21R | 50615 | Q9HBE5 | interleukin 21 receptor | Receptor | 110 | 0.55 | 0.731 | 0.99835 | 0.06 | | 1 | 6 | 0 | 2004 | 2019 |
| Rheumatoid Arthritis | C0003873 | CLEC4A | 50856 | Q9UMR7 | C-type lectin domain family 4 member A | Receptor | 14 | 0.792 | 0.269 | 4.19E-05 | 0.06 | | 0.833 | 6 | 4 | 2007 | 2015 |
| Rheumatoid Arthritis | C0003873 | TLR7 | 51284 | Q9NYK1 | toll like receptor 7 | | 276 | 0.464 | 0.885 | 0.97912 | 0.06 | | 1 | 6 | 2 | 2010 | 2019 |
| Rheumatoid Arthritis | C0003873 | GDE1 | 51573 | Q9NZC3 | glycerophosphodiester phosphodiesterase 1 | | 228 | 0.481 | 0.808 | 6.12E-06 | 0.06 | | 1 | 6 | 0 | 2014 | 2019 |
| Rheumatoid Arthritis | C0003873 | TLR9 | 54106 | Q9NR96 | toll like receptor 9 | | 457 | 0.411 | 0.885 | 9.81E-06 | 0.06 | | 1 | 6 | 1 | 2009 | 2020 |
| Rheumatoi | C0003 | BANK1 | 550 | Q8NDB2 | B cell scaffold protein with ankyrin repeats 1 | | 51 | 0.6 | 0.5 | 4.79E | 0.06 | | 0.5 | 6 | 3 | 2009 | 2018 |

| | | | | | | | | | | | | | | | | | |
|---|---|---|---|---|---|---|---|---|---|---|---|---|---|---|---|---|---|
| d Arthritis | 873 | | 24 | | | | | 53 | 38 | -12 | | | | | | | |
| Rheumatoid Arthritis | C0003873 | MAP2K7 | 5609 | O14733 | mitogen-activated protein kinase kinase 7 | Kinase | 408 | 0.419 | 0.808 | 0.99552 | 0.06 | | 1 | 6 | 0 | 2003 | 2018 |
| Rheumatoid Arthritis | C0003873 | HAMP | 57817 | P81172 | hepcidin antimicrobial peptide | | 377 | 0.433 | 0.846 | 0.00818 | 0.06 | | 1 | 6 | 0 | 2008 | 2019 |
| Rheumatoid Arthritis | C0003873 | BDNF | 627 | P23560 | brain derived neurotrophic factor | Signaling | 992 | 0.345 | 0.923 | 0.65626 | 0.06 | | 1 | 6 | 0 | 2009 | 2018 |
| Rheumatoid Arthritis | C0003873 | CCL7 | 6354 | P80098 | C-C motif chemokine ligand 7 | Signaling | 124 | 0.548 | 0.846 | 0.00893 | 0.06 | | 1 | 6 | 0 | 2005 | 2019 |
| Rheumatoid Arthritis | C0003873 | BRAF | 673 | P15056 | B-Raf proto-oncogene, serine/threonine kinase | Kinase | 1228 | 0.319 | 0.846 | 0.99995 | 0.06 | | 1 | 6 | 0 | 2010 | 2019 |
| Rheumatoid Arthritis | C0003873 | TEK | 7010 | Q02763 | TEK receptor tyrosine kinase | Kinase | 300 | 0.462 | 0.692 | 1 | 0.06 | | 1 | 6 | 0 | 2002 | 2020 |

| | | | | | | | | | | | | | | | | | |
|---|---|---|---|---|---|---|---|---|---|---|---|---|---|---|---|---|---|
| Rheumatoid Arthritis | C0003873 | C4A | 720 | P0C0L4;P0C0L5 | complement C4A (Rodgers blood group) | Enzyme modulator | 145 | 0.563 | 0.769 | 1.54E-11 | 0.06 | | 1 | 6 | 0 | 1989 | 1992 |
| Rheumatoid Arthritis | C0003873 | SUMO1 | 7341 | P63165 | small ubiquitin like modifier 1 | | 133 | 0.533 | 0.731 | 0.86128 | 0.06 | | 1 | 6 | 0 | 2000 | 2019 |
| Rheumatoid Arthritis | C0003873 | BEST1 | 7439 | O76090 | bestrophin 1 | Ion channel | 292 | 0.465 | 0.885 | 1.82E-12 | 0.06 | | 1 | 6 | 0 | 2002 | 2019 |
| Rheumatoid Arthritis | C0003873 | WNT5A | 7474 | P41221 | Wnt family member 5A | Signaling | 375 | 0.443 | 0.808 | 0.98732 | 0.06 | | 1 | 6 | 0 | 2000 | 2019 |
| Rheumatoid Arthritis | C0003873 | XRCC1 | 7515 | P18887 | X-ray repair cross complementing 1 | | 410 | 0.421 | 0.923 | 6.34E-09 | 0.06 | | 0.667 | 6 | 3 | 2006 | 2018 |
| Rheumatoid Arthritis | C0003873 | ZBTB16 | 7704 | Q05516 | zinc finger and BTB domain containing 16 | Transcription factor | 154 | 0.545 | 0.808 | 0.99907 | 0.06 | | 1 | 6 | 0 | 1997 | 2019 |
| Rheumatoid Ar | C0003873 | CAST | 831 | P20810 | calpastatin | | 141 | 0.541 | 0.846 | 4.12E- | 0.06 | | 1 | 6 | 0 | 1995 | 2011 |

| | | | | | | | | | | | | | | | | | |
|---|---|---|---|---|---|---|---|---|---|---|---|---|---|---|---|---|---|
| thritis | | | | | | | | | | 12 | | | | | | | |
| Rheumatoid Arthritis | C0003873 | CASP8 | 841 | Q14790 | caspase 8 | Enzyme | 480 | 0.404 | 0.923 | 3.72E-06 | 0.06 | | 1 | 6 | 0 | 2000 | 2017 |
| Rheumatoid Arthritis | C0003873 | TNFRSF25 | 8718 | Q93038 | TNF receptor superfamily member 25 | | 96 | 0.573 | 0.769 | 0.0086488 | 0.06 | | 1 | 6 | 0 | 2004 | 2018 |
| Rheumatoid Arthritis | C0003873 | ADAM15 | 8751 | Q13444 | ADAM metallopeptidase domain 15 | Enzyme | 59 | 0.626 | 0.577 | 2.44E-16 | 0.06 | | 1 | 6 | 0 | 2001 | 2019 |
| Rheumatoid Arthritis | C0003873 | NR1I2 | 8856 | O75469 | nuclear receptor subfamily 1 group I member 2 | Nuclear receptor | 462 | 0.418 | 0.846 | 7.27E-09 | 0.06 | | 1 | 6 | 0 | 2004 | 2018 |
| Rheumatoid Arthritis | C0003873 | CD86 | 942 | P42081 | CD86 molecule | | 219 | 0.483 | 0.769 | 0.948779 | 0.06 | | 0.833 | 6 | 2 | 2000 | 2019 |
| Rheumatoid Arthritis | C0003873 | ADA | 100 | P00813 | adenosine deaminase | Enzyme | 379 | 0.44 | 0.885 | 2.88E-12 | 0.05 | | 1 | 5 | 0 | 2003 | 2018 |
| Rheumatoi | C0003 | KIR2DS2 | 1E+ | P43631 | killer cell immunoglobulin like receptor, two | | 69 | 0.5 | 0.6 | | 0.05 | | 1 | 5 | 0 | 2001 | 2016 |

| | | | | | | | | | | | | | | | | | |
|---|---|---|---|---|---|---|---|---|---|---|---|---|---|---|---|---|---|
| d Arthritis | 873 | | 08 | | Ig domains and short cytoplasmic tail 2 | | | 99 | 15 | | | | | | | | |
| Rheumatoid Arthritis | C0003873 | CDKN1A | 1026 | P38936 | cyclin dependent kinase inhibitor 1A | | 490 | 0.403 | 0.769 | 0.001858 | 0.05 | | 1 | 5 | 0 | 2000 | 2018 |
| Rheumatoid Arthritis | C0003873 | NFAT5 | 10725 | O94916 | nuclear factor of activated T cells 5 | Transcription factor | 88 | 0.601 | 0.692 | 1 | 0.05 | | 1 | 5 | 0 | 2011 | 2019 |
| Rheumatoid Arthritis | C0003873 | LILRB1 | 10859 | Q8NHL6 | leukocyte immunoglobulin like receptor B1 | Receptor | 168 | 0.51 | 0.769 | 5.65E-14 | 0.05 | | 1 | 5 | 0 | 2005 | 2019 |
| Rheumatoid Arthritis | C0003873 | BTG3 | 10950 | Q14201 | BTG anti-proliferation factor 3 | | 141 | 0.528 | 0.808 | 0.91582 | 0.05 | | 1 | 5 | 0 | 1983 | 2019 |
| Rheumatoid Arthritis | C0003873 | CISH | 1154 | Q9NSE2 | cytokine inducible SH2 containing protein | Enzyme modulator | 195 | 0.5 | 0.808 | 0.000539 | 0.05 | | 1 | 5 | 0 | 2007 | 2018 |
| Rheumatoid Arthritis | C0003873 | TNFRSF13C | 115650 | Q96RJ3 | TNF receptor superfamily member 13C | | 127 | 0.54 | 0.731 | 0.26993 | 0.05 | | 1 | 5 | 1 | 2007 | 2017 |
| Rheu | C00 | CCR7 | 12 | P32248 | C-C motif | G-pro | 320 | 0. | 0. | 0.0 | 0.05 | | 1 | 5 | 0 | 2017 | 20 |

| | | | | | | | | | | | | | | | | | |
|---|---|---|---|---|---|---|---|---|---|---|---|---|---|---|---|---|---|
| matoid Arthritis | 03873 | | 36 | | chemokine receptor 7 | tein coupled receptor | | 443 | 769 | 57623 | | | | | | | 19 |
| Rheumatoid Arthritis | C0003873 | CNR2 | 1269 | P34972 | cannabinoid receptor 2 | G-protein coupled receptor | 197 | 0.509 | 0.846 | 6.54E-06 | 0.05 | | 1 | 5 | 3 | 2009 | 2019 |
| Rheumatoid Arthritis | C0003873 | SLCO6A1 | 133482 | Q86UG4 | solute carrier organic anion transporter family member 6A1 | Transporter | 449 | 0.412 | 0.885 | 2.88E-11 | 0.05 | | 0.8 | 5 | 0 | 1999 | 2011 |
| Rheumatoid Arthritis | C0003873 | CREB1 | 1385 | P16220 | cAMP responsive element binding protein 1 | | 294 | 0.463 | 0.885 | 0.99673 | 0.05 | | 1 | 5 | 0 | 2002 | 2017 |
| Rheumatoid Arthritis | C0003873 | CTSL | 1514 | P07711 | cathepsin L | Enzyme | 225 | 0.488 | 0.846 | 0.008045 | 0.05 | | 1 | 5 | 0 | 1990 | 2004 |
| Rheumatoid Arthritis | C0003873 | ADRB2 | 154 | P07550 | adrenoceptor beta 2 | G-protein coupled receptor | 387 | 0.442 | 0.923 | 0.52545 | 0.05 | | 1 | 5 | 0 | 2004 | 2018 |

| | | | | | | | | | | | | | | | | | |
|---|---|---|---|---|---|---|---|---|---|---|---|---|---|---|---|---|---|
| Rheumatoid Arthritis | C0003873 | CYP1A2 | 1544 | P05177 | cytochrome P450 family 1 subfamily A member 2 | Enzyme | 218 | 0.494 | 0.923 | 2.48E-10 | 0.05 | | 0.8 | 5 | 0 | 2008 | 2018 |
| Rheumatoid Arthritis | C0003873 | OLIG3 | 167826 | Q7RTU3 | oligodendrocyte transcription factor 3 | Enzyme | 4 | 0.861 | 0.192 | 0.83391 | 0.05 | | 1 | 5 | 1 | 2007 | 2018 |
| Rheumatoid Arthritis | C0003873 | DHODH | 1723 | Q02127 | dihydroorotate dehydrogenase (quinone) | Enzyme | 100 | 0.576 | 0.769 | 0.002988 | 0.05 | | 1 | 5 | 0 | 2009 | 2019 |
| Rheumatoid Arthritis | C0003873 | DNMT1 | 1786 | P26358 | DNA methyltransferase 1 | Epigenetic regulator | 496 | 0.406 | 0.885 | 1 | 0.05 | | 1 | 5 | 0 | 2013 | 2018 |
| Rheumatoid Arthritis | C0003873 | EGR1 | 1958 | P18146 | early growth response 1 | Nucleic acid binding | 378 | 0.433 | 0.923 | 0.3509 | 0.05 | | 1 | 5 | 0 | 1992 | 2017 |
| Rheumatoid Arthritis | C0003873 | FCGR1A | 2209 | P12314 | Fc fragment of IgG receptor Ia | Cell adhesion | 141 | 0.532 | 0.731 | 0.000131 | 0.05 | | 1 | 5 | 0 | 1997 | 2019 |
| Rheumatoid Ar | C0003873 | NLRP1 | 22861 | Q9C000 | NLR family pyrin domain containing 1 | | 200 | 0.502 | 0.769 | 4.97E- | 0.05 | | 0.8 | 5 | 1 | 2008 | 2017 |

| | | | | | | | | | | | | | | | | |
|---|---|---|---|---|---|---|---|---|---|---|---|---|---|---|---|---|
| thritis | | | | | | | | | | 19 | | | | | | |
| Rheumatoid Arthritis | C0003873 | FOSB | 2354 | P53539 | FosB proto-oncogene, AP-1 transcription factor subunit | Transcription factor | 278 | 0.463 | 0.846 | 0.97553 | 0.05 | | 1 | 5 | 0 | 1996 | 2017 |
| Rheumatoid Arthritis | C0003873 | MTOR | 2475 | P42345 | mechanistic target of rapamycin kinase | Kinase | 960 | 0.343 | 0.885 | 1 | 0.05 | | 1 | 5 | 0 | 2017 | 2019 |
| Rheumatoid Arthritis | C0003873 | ACAD8 | 27034 | Q9UKU7 | acyl-CoA dehydrogenase family member 8 | Enzyme | 203 | 0.506 | 0.885 | 5.64E-14 | 0.05 | | 0.8 | 5 | 0 | 2017 | 2019 |
| Rheumatoid Arthritis | C0003873 | B3GAT1 | 27087 | Q9P2W7 | beta-1,3-glucuronyltransferase 1 | Enzyme | 199 | 0.494 | 0.769 | 0.135221 | 0.05 | | 1 | 5 | 0 | 1990 | 1998 |
| Rheumatoid Arthritis | C0003873 | GSTM2 | 2946 | P28161 | glutathione S-transferase mu 2 | | 195 | 0.488 | 0.808 | 3.39E-07 | 0.05 | | 1 | 5 | 0 | 2002 | 2018 |
| Rheumatoid Arthritis | C0003873 | GSTP1 | 2950 | P09211 | glutathione S-transferase pi 1 | | 610 | 0.383 | 0.923 | 0.014155 | 0.05 | | 0.8 | 5 | 0 | 1999 | 2012 |
| Rheumatoi | C0003 | TNC | 3371 | P24821 | tenascin C | | 369 | 0.4 | 0.8 | 1.26E | 0.05 | | 1 | 5 | 0 | 2005 | 2018 |

| | | | | | | | | | | | | | | | | | |
|---|---|---|---|---|---|---|---|---|---|---|---|---|---|---|---|---|---|
| d Arthritis | 873 | | | | | | | 33 | 46 | -07 | | | | | | | |
| Rheumatoid Arthritis | C0003873 | APOE | 348 | P02649 | apolipoprotein E | | 1049 | 0.338 | 0.962 | 0.0018669 | 0.05 | | 1 | 5 | 0 | 1996 | 2013 |
| Rheumatoid Arthritis | C0003873 | IGF1R | 3480 | P08069 | insulin like growth factor 1 receptor | Kinase | 556 | 0.399 | 0.885 | 0.96799 | 0.05 | | 1 | 5 | 0 | 2014 | 2019 |
| Rheumatoid Arthritis | C0003873 | LINC01193 | 348120 | | long intergenic non-protein coding RNA 1193 | | 91 | 0.565 | 0.692 | | 0.05 | | 1 | 5 | 0 | 2004 | 2013 |
| Rheumatoid Arthritis | C0003873 | IGHG3 | 3502 | P01860 | immunoglobulin heavy constant gamma 3 (G3m marker) | | 161 | 0.513 | 0.769 | | 0.05 | | 1 | 5 | 0 | 1997 | 2020 |
| Rheumatoid Arthritis | C0003873 | IL12A | 3592 | P29459 | interleukin 12A | | 261 | 0.491 | 0.808 | 0.04746 | 0.05 | | 1 | 5 | 1 | 2012 | 2019 |
| Rheumatoid Arthritis | C0003873 | ITGAM | 3684 | P11215 | integrin subunit alpha M | | 343 | 0.443 | 0.808 | 3.31E-11 | 0.05 | | 0.8 | 5 | 1 | 2009 | 2019 |

| | | | | | | | | | | | | | | | | | |
|---|---|---|---|---|---|---|---|---|---|---|---|---|---|---|---|---|---|
| Rheumatoid Arthritis | C0003873 | ITGB1 | 3688 | P05556 | integrin subunit beta 1 | Receptor | 210 | 0.496 | 0.808 | 0.98208 | 0.05 | | 1 | 5 | 0 | 1993 | 2019 |
| Rheumatoid Arthritis | C0003873 | JUNB | 3726 | P17275 | JunB proto-oncogene, AP-1 transcription factor subunit | Transcription factor | 264 | 0.467 | 0.769 | 0.86299 | 0.05 | | 1 | 5 | 0 | 1996 | 2017 |
| Rheumatoid Arthritis | C0003873 | JUND | 3727 | P17535 | JunD proto-oncogene, AP-1 transcription factor subunit | Transcription factor | 242 | 0.475 | 0.808 | 0.70054 | 0.05 | | 1 | 5 | 0 | 1996 | 2017 |
| Rheumatoid Arthritis | C0003873 | GSTK1 | 373156 | Q9Y2Q3 | glutathione S-transferase kappa 1 | | 445 | 0.412 | 0.885 | 1.94E-06 | 0.05 | | 0.8 | 5 | 0 | 1999 | 2011 |
| Rheumatoid Arthritis | C0003873 | KLRB1 | 3820 | Q12918 | killer cell lectin like receptor B1 | | 71 | 0.601 | 0.615 | 2.41E-07 | 0.05 | | 1 | 5 | 0 | 2010 | 2019 |
| Rheumatoid Arthritis | C0003873 | RPSA | 3921 | P08865 | ribosomal protein SA | Nucleic acid binding | 221 | 0.485 | 0.769 | 0.93037 | 0.05 | | 1 | 5 | 0 | 2000 | 2019 |
| Rheumatoid Ar | C0003873 | LGALS1 | 3956 | P09382 | galectin 1 | Signaling | 362 | 0.435 | 0.846 | 0.0044 | 0.05 | | 1 | 5 | 0 | 2003 | 2019 |

| | | | | | | | | | | | | | | | | | |
|---|---|---|---|---|---|---|---|---|---|---|---|---|---|---|---|---|---|
| thritis | | | | | | | | | | 88 | | | | | | | |
| Rheumatoid Arthritis | C0003873 | MIR126 | 406913 | | microRNA 126 | | 305 | 0.447 | 0.846 | | 0.05 | | 1 | 5 | 0 | 2015 | 2018 |
| Rheumatoid Arthritis | C0003873 | MIR22 | 407004 | | microRNA 22 | | 195 | 0.496 | 0.808 | | 0.05 | | 1 | 5 | 0 | 2014 | 2020 |
| Rheumatoid Arthritis | C0003873 | MIR221 | 407006 | | microRNA 221 | | 304 | 0.449 | 0.846 | | 0.05 | | 1 | 5 | 0 | 2012 | 2020 |
| Rheumatoid Arthritis | C0003873 | MIR34A | 407040 | | microRNA 34a | | 428 | 0.421 | 0.846 | | 0.05 | | 1 | 5 | 0 | 2012 | 2019 |
| Rheumatoid Arthritis | C0003873 | MCL1 | 4170 | Q07820 | MCL1 apoptosis regulator, BCL2 family member | Signaling | 375 | 0.43 | 0.808 | 0.96349 | 0.05 | | 1 | 5 | 0 | 2005 | 2019 |
| Rheumatoid Arthritis | C0003873 | MDM2 | 4193 | Q00987 | MDM2 proto-oncogene | Nucleic acid binding | 702 | 0.362 | 0.846 | 0.99981 | 0.05 | | 1 | 5 | 1 | 2005 | 2016 |
| Rheumatoi | C0003 | CXCL9 | 4283 | Q07325 | C-X-C motif chemoki | Signaling | 269 | 0.4 | 0.8 | 0.002 | 0.05 | | 1 | 5 | 1 | 2003 | 2015 |

| | | | | | | | | | | | | | | | | | |
|---|---|---|---|---|---|---|---|---|---|---|---|---|---|---|---|---|---|
| d Arthritis | 873 | | | | ne ligand 9 | | | 62 | 08 | 93 | | | | | | | |
| Rheumatoid Arthritis | C0003873 | NFATC1 | 4772 | O95644 | nuclear factor of activated T cells 1 | Transcription factor | 161 | 0.517 | 0.731 | 0.156552 | 0.05 | | 1 | 5 | 0 | 2007 | 2019 |
| Rheumatoid Arthritis | C0003873 | NR4A2 | 4929 | P43354 | nuclear receptor subfamily 4 group A member 2 | Nuclear receptor | 166 | 0.529 | 0.846 | 0.99959 | 0.05 | | 1 | 5 | 0 | 2001 | 2013 |
| Rheumatoid Arthritis | C0003873 | HSPA14 | 51182 | Q0VDF9 | heat shock protein family A (Hsp70) member 14 | | 178 | 0.502 | 0.769 | 0.67359 | 0.05 | | 1 | 5 | 0 | 2007 | 2019 |
| Rheumatoid Arthritis | C0003873 | PLA2G1B | 5319 | P04054 | phospholipase A2 group IB | Enzyme | 268 | 0.468 | 0.846 | 1.01E-10 | 0.05 | | 1 | 5 | 0 | 1991 | 2011 |
| Rheumatoid Arthritis | C0003873 | PLAU | 5328 | P00749 | plasminogen activator, urokinase | Enzyme | 439 | 0.425 | 0.923 | 2.31E-06 | 0.05 | | 1 | 5 | 0 | 2000 | 2018 |
| Rheumatoid Arthritis | C0003873 | PTEN | 5728 | P60484 | phosphatase and tensin homolog | Enzyme | 1349 | 0.305 | 0.923 | 0.256551 | 0.05 | | 0.6 | 5 | 0 | 1986 | 2017 |
| Rheu | C00 | PTH | 57 | P01270 | parathyroid hormone | | 528 | 0. | 0. | 0.0 | 0.05 | | 1 | 5 | 0 | 2001 | 20 |

| | | | | | | | | | | | | | | | | | |
|---|---|---|---|---|---|---|---|---|---|---|---|---|---|---|---|---|---|
| matoid Arthritis | 003873 | | 41 | | | | | 397 | 846 | 12093 | | | | | | | 19 |
| Rheumatoid Arthritis | C0003873 | CXCL16 | 58191 | Q9H2A7 | C-X-C motif chemokine ligand 16 | Signaling | 178 | 0.502 | 0.769 | 3.45E-05 | 0.05 | | 1 | 5 | 0 | 2005 | 2016 |
| Rheumatoid Arthritis | C0003873 | RAC1 | 5879 | P63000 | Rac family small GTPase 1 | Enzyme modulator | 415 | 0.429 | 0.962 | 0.76228 | 0.05 | | 1 | 5 | 0 | 2007 | 2019 |
| Rheumatoid Arthritis | C0003873 | RARRES2 | 5919 | Q99969 | retinoic acid receptor responder 2 | | 180 | 0.519 | 0.731 | 2.72E-05 | 0.05 | | 1 | 5 | 0 | 2012 | 2019 |
| Rheumatoid Arthritis | C0003873 | TNFRSF17 | 608 | Q02223 | TNF receptor superfamily member 17 | Receptor | 81 | 0.588 | 0.692 | 1.37E-06 | 0.05 | | 1 | 5 | 0 | 2001 | 2019 |
| Rheumatoid Arthritis | C0003873 | ROS1 | 6098 | P08922 | ROS proto-oncogene 1, receptor tyrosine kinase | Kinase | 356 | 0.439 | 0.885 | 1.62E-72 | 0.05 | | 0.8 | 5 | 0 | 2017 | 2019 |
| Rheumatoid Arthritis | C0003873 | S100A4 | 6275 | P26447 | S100 calcium binding protein A4 | Calcium-binding pro | 308 | 0.448 | 0.769 | 0.006184 | 0.05 | | 1 | 5 | 0 | 2006 | 2019 |

| | | | | | | | | | | | | | | | | | |
|---|---|---|---|---|---|---|---|---|---|---|---|---|---|---|---|---|---|
| | | | | | | tein | | | | | | | | | | | |
| Rheumatoid Arthritis | C0003873 | S100A12 | 6283 | P80511 | S100 calcium binding protein A12 | Calcium-binding protein | 221 | 0.488 | 0.846 | 0.482334 | 0.05 | | 1 | 5 | 0 | 2005 | 2019 |
| Rheumatoid Arthritis | C0003873 | SP1 | 6667 | P08047 | Sp1 transcription factor | Nucleic acid binding | 209 | 0.493 | 0.769 | 0.99997 | 0.05 | | 1 | 5 | 0 | 2017 | 2019 |
| Rheumatoid Arthritis | C0003873 | MAP3K7 | 6885 | O43318 | mitogen-activated protein kinase kinase kinase 7 | Kinase | 273 | 0.477 | 0.808 | 0.99971 | 0.05 | | 1 | 5 | 0 | 2004 | 2020 |
| Rheumatoid Arthritis | C0003873 | TRB | 6957 | P0DSE2 | T cell receptor beta locus | | 49 | 0.636 | 0.538 | | 0.05 | | 0.8 | 5 | 0 | 1994 | 2003 |
| Rheumatoid Arthritis | C0003873 | TSPO | 706 | P30536 | translocator protein | | 341 | 0.444 | 0.885 | 0.000274 | 0.05 | | 1 | 5 | 0 | 2017 | 2020 |
| Rheumatoid Arthritis | C0003873 | TM7SF2 | 7108 | O76062 | transmembrane 7 superfamily member 2 | Enzyme | 217 | 0.486 | 0.808 | 1.37E-07 | 0.05 | | 1 | 5 | 0 | 2002 | 2012 |

| | | | | | | | | | | | | | | | | | |
|---|---|---|---|---|---|---|---|---|---|---|---|---|---|---|---|---|---|
| Rheumatoid Arthritis | C0003873 | VPS51 | 738 | Q9UID3 | VPS51 subunit of GARP complex | | 292 | 0.452 | 0.846 | 1.05E-08 | 0.05 | | 1 | 5 | 0 | 2002 | 2016 |
| Rheumatoid Arthritis | C0003873 | VEGFC | 7424 | P49767 | vascular endothelial growth factor C | Signaling | 291 | 0.459 | 0.769 | 0.495665 | 0.05 | | 1 | 5 | 0 | 2002 | 2019 |
| Rheumatoid Arthritis | C0003873 | DHX40 | 79665 | Q8IX18 | DEAH-box helicase 40 | Enzyme | 64 | 0.608 | 0.769 | 0.7556 | 0.05 | | 1 | 5 | 0 | 2007 | 2019 |
| Rheumatoid Arthritis | C0003873 | TSLP | 85480 | Q969D9 | thymic stromal lymphopoietin | | 220 | 0.486 | 0.808 | 0.004036 | 0.05 | | 1 | 5 | 0 | 2014 | 2018 |
| Rheumatoid Arthritis | C0003873 | SOCS1 | 8651 | O15524 | suppressor of cytokine signaling 1 | Enzyme modulator | 315 | 0.445 | 0.808 | 0.58544 | 0.05 | | 1 | 5 | 0 | 2004 | 2012 |
| Rheumatoid Arthritis | C0003873 | TNFSF10 | 8743 | P50591 | TNF superfamily member 10 | Signaling | 445 | 0.413 | 0.885 | 0.134995 | 0.05 | | 1 | 5 | 0 | 2009 | 2017 |
| Rheumatoid Ar | C0003873 | TNFRSF10A | 8797 | O00220 | TNF receptor superfamily member 10a | | 155 | 0.521 | 0.731 | 5.07E- | 0.05 | | 0.8 | 5 | 1 | 1989 | 2012 |

| | | | | | | | | | | | | | | | | | |
|---|---|---|---|---|---|---|---|---|---|---|---|---|---|---|---|---|---|
| thritis | | | | | | | | | | 15 | | | | | | | |
| Rheumatoid Arthritis | C0003873 | CFLAR | 8837 | O15519 | CASP8 and FADD like apoptosis regulator | Enzyme | 215 | 0.485 | 0.846 | 0.99967 | 0.05 | | 1 | 5 | 0 | 2000 | 2017 |
| Rheumatoid Arthritis | C0003873 | SQSTM1 | 8878 | Q13501 | sequestosome 1 | | 470 | 0.428 | 0.885 | 0.00086 | 0.05 | | 1 | 5 | 0 | 2005 | 2019 |
| Rheumatoid Arthritis | C0003873 | CD80 | 941 | P33681 | CD80 molecule | | 233 | 0.475 | 0.769 | 0.010898 | 0.05 | | 1 | 5 | 0 | 2000 | 2018 |
| Rheumatoid Arthritis | C0003873 | ADAMTS4 | 9507 | O75173 | ADAM metallopeptidase with thrombospondin type 1 motif 4 | Enzyme | 84 | 0.582 | 0.692 | 1.95E-06 | 0.05 | | 1 | 5 | 0 | 2002 | 2015 |
| Rheumatoid Arthritis | C0003873 | GDF15 | 9518 | Q99988 | growth differentiation factor 15 | Signaling | 389 | 0.429 | 0.808 | 3.01E-07 | 0.05 | | 1 | 5 | 1 | 2007 | 2019 |
| Rheumatoid Arthritis | C0003873 | CD38 | 952 | P28907 | CD38 molecule | Enzyme | 473 | 0.407 | 0.885 | 2.31E-06 | 0.05 | | 1 | 5 | 0 | 2003 | 2019 |
| Rheumatoi | C0003 | HOTAIR | 1E+ | | HOX transcript antisense RNA | | 234 | 0.4 | 0.7 | | 0.04 | | 0.75 | 4 | 0 | 2017 | 2019 |

| | | | | | | | | | | | | | | | | | |
|---|---|---|---|---|---|---|---|---|---|---|---|---|---|---|---|---|---|
| d Arthritis | 873 | | 08 | | | | | 75 | 69 | | | | | | | | |
| Rheumatoid Arthritis | C0003873 | ZGLP1 | 1E+08 | P0C6A0 | zinc finger GATA like protein 1 | | 186 | 0.512 | 0.808 | 0.3868 | 0.04 | | 1 | 4 | 0 | 2017 | 2018 |
| Rheumatoid Arthritis | C0003873 | IL18BP | 10068 | O95998 | interleukin 18 binding protein | | 75 | 0.603 | 0.731 | 1.48E-08 | 0.04 | | 1 | 4 | 0 | 2003 | 2016 |
| Rheumatoid Arthritis | C0003873 | NAMPT | 10135 | P43490 | nicotinamide phosphoribosyltransferase | Signaling | 199 | 0.502 | 0.808 | 0.97914 | 0.04 | | 1 | 4 | 2 | 2011 | 2017 |
| Rheumatoid Arthritis | C0003873 | RABEPK | 10244 | Q7Z6M1 | Rab9 effector protein with kelch motifs | | 112 | 0.553 | 0.692 | 2.84E-11 | 0.04 | | 1 | 4 | 0 | 2000 | 2018 |
| Rheumatoid Arthritis | C0003873 | LANCL1 | 10314 | O43813 | LanC like 1 | | 113 | 0.552 | 0.692 | 1.08E-11 | 0.04 | | 1 | 4 | 0 | 2000 | 2018 |
| Rheumatoid Arthritis | C0003873 | CDKN2D | 1032 | P55273 | cyclin dependent kinase inhibitor 2D | | 110 | 0.547 | 0.769 | 0.49473 | 0.04 | | 1 | 4 | 0 | 1989 | 2009 |
| Rheu | C00 | NXF1 | 10 | Q9UBU9 | nuclear RNA | Nucle | 141 | 0. | 0. | 0.9 | 0.04 | | 1 | 4 | 0 | 1994 | 20 |

| | | | | | | | | | | | | | | | | | |
|---|---|---|---|---|---|---|---|---|---|---|---|---|---|---|---|---|---|
| matoid Arthritis | 03873 | | 482 | | export factor 1 | ic acid binding | | 531 | 885 | 763 | | | | | | | 00 |
| Rheumatoid Arthritis | C0003873 | LOC105379528 | 1.05E+08 | | uncharacterized LOC105379528 | | 55 | 0.617 | 0.615 | | 0.04 | | 1 | 4 | 0 | 1995 | 2007 |
| Rheumatoid Arthritis | C0003873 | KHDRBS1 | 10657 | Q07666 | KH RNA binding domain containing, signal transduction associated 1 | Nucleic acid binding | 289 | 0.457 | 0.846 | 0.99423 | 0.04 | | 1 | 4 | 0 | 2018 | 2019 |
| Rheumatoid Arthritis | C0003873 | CXCR6 | 10663 | O00574 | C-X-C motif chemokine receptor 6 | G-protein coupled receptor | 325 | 0.448 | 0.885 | 5.72E-05 | 0.04 | | 1 | 4 | 0 | 1999 | 2016 |
| Rheumatoid Arthritis | C0003873 | MALT1 | 10892 | Q9UDY8 | MALT1 paracaspase | Enzyme | 148 | 0.532 | 0.769 | 0.98651 | 0.04 | | 1 | 4 | 0 | 2014 | 2019 |
| Rheumatoid Arthritis | C0003873 | EBNA1BP2 | 10969 | Q99848 | EBNA1 binding protein 2 | | 116 | 0.55 | 0.692 | 1.37E-09 | 0.04 | | 1 | 4 | 0 | 2000 | 2018 |
| Rheuma | C0003 | H3P13 | 1.15 | | H3 histone pseudogene 13 | | 78 | 0.5 | 0.7 | | 0.04 | | 1 | 4 | 0 | 1989 | 20 |

| | | | | | | | | | | | | | | | | | |
|---|---|---|---|---|---|---|---|---|---|---|---|---|---|---|---|---|---|
| toid Arthritis | 873 | | E+08 | | | | | 84 | 69 | | | | | | | | 09 |
| Rheumatoid Arthritis | C0003873 | H3P28 | 1.15E+08 | | H3 histone pseudogene 28 | | 111 | 0.553 | 0.692 | | 0.04 | | 1 | 4 | 0 | 2000 | 2018 |
| Rheumatoid Arthritis | C0003873 | LRG1 | 116844 | P02750 | leucine rich alpha-2-glycoprotein 1 | | 109 | 0.564 | 0.731 | 0.0951143 | 0.04 | | 1 | 4 | 0 | 2012 | 2018 |
| Rheumatoid Arthritis | C0003873 | CLU | 1191 | P10909 | clusterin | | 412 | 0.426 | 0.885 | 7.81E-05 | 0.04 | | 1 | 4 | 0 | 2006 | 2015 |
| Rheumatoid Arthritis | C0003873 | SERPINA3 | 12 | P01011 | serpin family A member 3 | Enzyme modulator | 229 | 0.486 | 0.846 | 8.83E-14 | 0.04 | | 1 | 4 | 0 | 1990 | 2019 |
| Rheumatoid Arthritis | C0003873 | CCR4 | 1233 | P51679 | C-C motif chemokine receptor 4 | G-protein coupled receptor | 168 | 0.51 | 0.731 | 0.06863 | 0.04 | | 1 | 4 | 0 | 2004 | 2019 |
| Rheumatoid Ar | C0003873 | CR2 | 1380 | P20023 | complement C3d receptor 2 | | 123 | 0.556 | 0.654 | 4.74E-19 | 0.04 | | 1 | 4 | 0 | 2010 | 2013 |

| | | | | | | | | | | | | | | | | | |
|---|---|---|---|---|---|---|---|---|---|---|---|---|---|---|---|---|---|
| thritis | | | | | | | | | | | | | | | | | |
| Rheumatoid Arthritis | C0003873 | CRABP2 | 1382 | P29373 | cellular retinoic acid binding protein 2 | | 79 | 0.593 | 0.615 | 0.00151 | 0.04 | | 1 | 4 | 0 | 1996 | 2018 |
| Rheumatoid Arthritis | C0003873 | CSF1R | 1436 | P07333 | colony stimulating factor 1 receptor | Kinase | 356 | 0.448 | 0.808 | 0.13278 | 0.04 | | 1 | 4 | 0 | 2016 | 2019 |
| Rheumatoid Arthritis | C0003873 | CTNNB1 | 1499 | P35222 | catenin beta 1 | | 1368 | 0.303 | 0.885 | 0.99999 | 0.04 | | 1 | 4 | 0 | 2011 | 2019 |
| Rheumatoid Arthritis | C0003873 | CYBB | 1536 | P04839 | cytochrome b-245 beta chain | Ion channel | 343 | 0.452 | 0.885 | 0.99904 | 0.04 | | 1 | 4 | 0 | 2006 | 2019 |
| Rheumatoid Arthritis | C0003873 | DECR1 | 1666 | Q16698 | 2,4-dienoyl-CoA reductase 1 | Enzyme | 399 | 0.426 | 0.885 | 4.47E-14 | 0.04 | | 1 | 4 | 0 | 2006 | 2019 |
| Rheumatoid Arthritis | C0003873 | ATN1 | 1822 | P54259 | atrophin 1 | | 499 | 0.409 | 0.885 | 0.99992 | 0.04 | | 1 | 4 | 1 | 2002 | 2016 |
| Rheumatoi | C0003 | S1PR1 | 1901 | P21453 | sphingosine-1-phosphate | G-protein | 126 | 0.5 | 0.7 | 0.732 | 0.04 | | 1 | 4 | 0 | 2008 | 2019 |

| | | | | | | | | | | | | | | | | | |
|---|---|---|---|---|---|---|---|---|---|---|---|---|---|---|---|---|---|
| d Arthritis | 873 | | | | receptor 1 | coupled receptor | | 46 | 69 | 69 | | | | | | | |
| Rheumatoid Arthritis | C0003873 | LPAR1 | 1902 | Q92633 | lysophosphatidic acid receptor 1 | G-protein coupled receptor | 145 | 0.534 | 0.808 | 0.186033 | 0.04 | | 1 | 4 | 0 | 2008 | 2019 |
| Rheumatoid Arthritis | C0003873 | EDNRA | 1909 | P25101 | endothelin receptor type A | G-protein coupled receptor | 427 | 0.426 | 0.885 | 0.991158 | 0.04 | | 0.75 | 4 | 0 | 1999 | 2014 |
| Rheumatoid Arthritis | C0003873 | EGF | 1950 | P01133 | epidermal growth factor | | 774 | 0.357 | 0.923 | 8.53E-17 | 0.04 | | 1 | 4 | 2 | 2002 | 2019 |
| Rheumatoid Arthritis | C0003873 | F3 | 2152 | P13726 | coagulation factor III, tissue factor | Receptor | 456 | 0.415 | 0.846 | 0.018872 | 0.04 | | 1 | 4 | 0 | 2003 | 2018 |
| Rheumatoid Arthritis | C0003873 | F9 | 2158 | P00740 | coagulation factor IX | Enzyme | 276 | 0.465 | 0.885 | 0.99776 | 0.04 | | 1 | 4 | 1 | 1989 | 2009 |
| Rheumatoi | C0003 | MMRN1 | 229 | Q13201 | multimerin 1 | | 272 | 0.4 | 0.8 | 2.07E | 0.04 | | 1 | 4 | 0 | 1993 | 2019 |

| | | | | | | | | | | | | | | | | | |
|---|---|---|---|---|---|---|---|---|---|---|---|---|---|---|---|---|---|
| d Arthritis | 873 | | 15 | | | | | 65 | 46 | -25 | | | | | | | |
| Rheumatoid Arthritis | C0003873 | FOXC1 | 2296 | Q12948 | forkhead box C1 | Transcription factor | 242 | 0.483 | 0.846 | 0.95337 | 0.04 | | 1 | 4 | 0 | 1988 | 2018 |
| Rheumatoid Arthritis | C0003873 | FLNB | 2317 | O75369 | filamin B | | 374 | 0.458 | 0.923 | 6.05E-10 | 0.04 | | 1 | 4 | 0 | 1994 | 2000 |
| Rheumatoid Arthritis | C0003873 | SEC14L2 | 23541 | O76054 | SEC14 like lipid binding 2 | | 198 | 0.494 | 0.885 | 2.92E-08 | 0.04 | | 1 | 4 | 0 | 1994 | 2000 |
| Rheumatoid Arthritis | C0003873 | FPR2 | 2358 | P25090 | formyl peptide receptor 2 | G-protein coupled receptor | 124 | 0.547 | 0.769 | 0.40137 | 0.04 | | 1 | 4 | 0 | 2004 | 2018 |
| Rheumatoid Arthritis | C0003873 | BBC3 | 27113 | Q96PG8;Q9BXH1 | BCL2 binding component 3 | | 157 | 0.517 | 0.769 | 0.003976 | 0.04 | | 0.75 | 4 | 0 | 2006 | 2017 |
| Rheumatoid Arthritis | C0003873 | GLB1 | 2720 | P16278 | galactosidase beta 1 | Enzyme | 385 | 0.448 | 0.808 | 7.92E-10 | 0.04 | | 1 | 4 | 0 | 1996 | 2019 |

| | | | | | | | | | | | | | | | | | |
|---|---|---|---|---|---|---|---|---|---|---|---|---|---|---|---|---|---|
| Rheumatoid Arthritis | C0003873 | IGHV3OR16-7 | 28309 | | immunoglobulin heavy variable 3/OR16-7 (pseudogene) | | 98 | 0.566 | 0.654 | | 0.04 | | 1 | 4 | 0 | 1995 | 2007 |
| Rheumatoid Arthritis | C0003873 | IGHV3-52 | 28421 | | immunoglobulin heavy variable 3-52 (pseudogene) | | 37 | 0.674 | 0.423 | | 0.04 | | 1 | 4 | 0 | 1993 | 2010 |
| Rheumatoid Arthritis | C0003873 | TRBV16 | 28571 | A0A087WV62 | T cell receptor beta variable 16 (gene/pseudogene) | | 29 | 0.695 | 0.5 | | 0.04 | | 0.75 | 4 | 0 | 1994 | 1997 |
| Rheumatoid Arthritis | C0003873 | TRBV7-9 | 28589 | P04435 | T cell receptor beta variable 7-9 | | 29 | 0.695 | 0.5 | | 0.04 | | 0.75 | 4 | 0 | 1994 | 1997 |
| Rheumatoid Arthritis | C0003873 | TRBC1 | 28639 | P01850 | T cell receptor beta constant 1 | | 29 | 0.695 | 0.5 | | 0.04 | | 0.75 | 4 | 0 | 1994 | 1997 |
| Rheumatoid Arthritis | C0003873 | GPT | 2875 | P24298 | glutamic--pyruvic transaminase | Enzyme | 511 | 0.403 | 0.923 | 3.59E-19 | 0.04 | | 1 | 4 | 0 | 2001 | 2019 |
| Rheumatoid Ar | C0003873 | GRN | 2896 | P28799 | granulin precursor | | 412 | 0.435 | 0.846 | 0.0696 | 0.04 | | 0.75 | 4 | 0 | 2015 | 2020 |

| | | | | | | | | | | | | | | | | | |
|---|---|---|---|---|---|---|---|---|---|---|---|---|---|---|---|---|---|
| thritis | | | | | | | | | | 66 | | | | | | | |
| Rheumatoid Arthritis | C0003873 | RMC1 | 29919 | Q96DM3 | regulator of MON1-CCZ1 | | 110 | 0.555 | 0.731 | 0.98823 | 0.04 | | 1 | 4 | 0 | 2007 | 2019 |
| Rheumatoid Arthritis | C0003873 | IL19 | 29949 | Q9UHD0 | interleukin 19 | | 103 | 0.558 | 0.769 | 4.33E-05 | 0.04 | | 1 | 4 | 5 | 2010 | 2018 |
| Rheumatoid Arthritis | C0003873 | ANXA1 | 301 | P04083 | annexin A1 | | 336 | 0.442 | 0.885 | 1.2E-07 | 0.04 | | 1 | 4 | 0 | 2007 | 2019 |
| Rheumatoid Arthritis | C0003873 | ACACA | 31 | Q13085 | acetyl-CoA carboxylase alpha | | 241 | 0.48 | 0.846 | 1 | 0.04 | | 0.75 | 4 | 0 | 2008 | 2019 |
| Rheumatoid Arthritis | C0003873 | HLA-DRB3 | 3125 | P79483 | major histocompatibility complex, class II, DR beta 3 | Immune response | 85 | 0.57 | 0.885 | | 0.04 | | 0.5 | 4 | 0 | 1992 | 2019 |
| Rheumatoid Arthritis | C0003873 | HOXD9 | 3235 | P28356 | homeobox D9 | | 34 | 0.67 | 0.462 | 0.017004 | 0.04 | | 1 | 4 | 0 | 1996 | 2002 |
| Rheumatoi | C0003 | APC | 324 | P25054 | APC regulator of WNT signaling pathway | | 703 | 0.3 | 0.9 | 1 | 0.04 | | 1 | 4 | 0 | 1999 | 2011 |

| | | | | | | | | | | | | | | | | | |
|---|---|---|---|---|---|---|---|---|---|---|---|---|---|---|---|---|---|
| d Arthritis | 873 | | | | | | | 73 | 62 | | | | | | | | |
| Rheumatoid Arthritis | C0003873 | HTR2A | 3356 | P28223 | 5-hydroxytryptamine receptor 2A | G-protein coupled receptor | 289 | 0.473 | 0.846 | 0.51124 | 0.04 | | 1 | 4 | 3 | 2006 | 2013 |
| Rheumatoid Arthritis | C0003873 | ARMH1 | 339541 | Q6PIY5 | armadillo like helical domain containing 1 | | 111 | 0.553 | 0.692 | 1.65E-05 | 0.04 | | 1 | 4 | 0 | 2000 | 2018 |
| Rheumatoid Arthritis | C0003873 | IFNB1 | 3456 | P01574 | interferon beta 1 | | 426 | 0.421 | 0.846 | | 0.04 | | 1 | 4 | 0 | 2012 | 2018 |
| Rheumatoid Arthritis | C0003873 | IGFBP5 | 3488 | P24593 | insulin like growth factor binding protein 5 | Enzyme modulator | 126 | 0.544 | 0.692 | 0.96283 | 0.04 | | 1 | 4 | 0 | 2002 | 2018 |
| Rheumatoid Arthritis | C0003873 | LCE3B | 353143 | Q5TA77 | late cornified envelope 3B | | 14 | 0.751 | 0.231 | 0.003432 | 0.04 | | 0.75 | 4 | 0 | 2010 | 2012 |
| Rheumatoid Arthritis | C0003873 | LCE3C | 353144 | Q5T5A8 | late cornified envelope 3C | | 15 | 0.743 | 0.269 | 0.47523 | 0.04 | | 0.75 | 4 | 0 | 2010 | 2012 |

| | | | | | | | | | | | | | | | | | |
|---|---|---|---|---|---|---|---|---|---|---|---|---|---|---|---|---|---|
| Rheumatoid Arthritis | C0003873 | IKBKB | 3551 | O14920 | inhibitor of nuclear factor kappa B kinase subunit beta | Kinase | 191 | 0.506 | 0.846 | 0.99192 | 0.04 | | 1 | 4 | 0 | 2001 | 2020 |
| Rheumatoid Arthritis | C0003873 | IL5 | 3567 | P05113 | interleukin 5 | | 359 | 0.437 | 0.846 | 0.0056553 | 0.04 | | 0.75 | 4 | 0 | 1996 | 2019 |
| Rheumatoid Arthritis | C0003873 | CXCR2 | 3579 | P25025 | C-X-C motif chemokine receptor 2 | G-protein coupled receptor | 335 | 0.445 | 0.923 | 0.0137336 | 0.04 | | 1 | 4 | 0 | 1997 | 2015 |
| Rheumatoid Arthritis | C0003873 | IL10RA | 3587 | Q13651 | interleukin 10 receptor subunit alpha | Receptor | 90 | 0.579 | 0.692 | 0.017808 | 0.04 | | 1 | 4 | 3 | 1999 | 2017 |
| Rheumatoid Arthritis | C0003873 | ITGA1 | 3672 | P56199 | integrin subunit alpha 1 | | 58 | 0.644 | 0.654 | 2.61E-16 | 0.04 | | 1 | 4 | 0 | 1988 | 2019 |
| Rheumatoid Arthritis | C0003873 | KIR2DL2 | 3803 | P43627 | killer cell immunoglobulin like receptor, two Ig domains and long cytoplasmic tail 2 | Receptor | 65 | 0.612 | 0.654 | | 0.04 | | 1 | 4 | 0 | 2012 | 2019 |

| | | | | | | | | | | | | | | | | |
|---|---|---|---|---|---|---|---|---|---|---|---|---|---|---|---|---|
| Rheumatoid Arthritis | C0003873 | KLRC1 | 3821 | P26715 | killer cell lectin like receptor C1 | 67 | 0.604 | 0.731 | 4.14E-05 | 0.04 | | 1 | 4 | 1 | 2003 | 2017 |
| Rheumatoid Arthritis | C0003873 | SUMO4 | 387082 | Q6EEV6 | small ubiquitin like modifier 4 | 39 | 0.653 | 0.577 | | 0.04 | | 0.75 | 4 | 1 | 2005 | 2006 |
| Rheumatoid Arthritis | C0003873 | LOC390714 | 390714 | | immunoglobulin heavy chain variable region | 55 | 0.617 | 0.615 | | 0.04 | | 1 | 4 | 0 | 1995 | 2007 |
| Rheumatoid Arthritis | C0003873 | LBP | 3929 | P18428 | lipopolysaccharide binding protein | 156 | 0.525 | 0.808 | 4.19E-15 | 0.04 | | 1 | 4 | 0 | 2018 | 2019 |
| Rheumatoid Arthritis | C0003873 | LOX | 4015 | P28300 | lysyl oxidase | 328 | 0.447 | 0.846 | 0.98769 | 0.04 | | 1 | 4 | 0 | 1995 | 2018 |
| Rheumatoid Arthritis | C0003873 | MIR10A | 406902 | | microRNA 10a | 158 | 0.513 | 0.846 | | 0.04 | | 1 | 4 | 0 | 2016 | 2018 |
| Rheumatoid Ar | C0003873 | MIR145 | 406937 | | microRNA 145 | 366 | 0.431 | 0.846 | | 0.04 | | 1 | 4 | 0 | 2017 | 2019 |

| | | | | | | | | | | | | | | | | | |
|---|---|---|---|---|---|---|---|---|---|---|---|---|---|---|---|---|---|
| thritis | | | | | | | | | | | | | | | | | |
| Rheumatoid Arthritis | C0003873 | CD46 | 4179 | P15529 | CD46 molecule | | 258 | 0.477 | 0.808 | 4.7E-07 | 0.04 | | 1 | 4 | 0 | 2017 | 2019 |
| Rheumatoid Arthritis | C0003873 | MIP | 4284 | P30301 | major intrinsic protein of lens fiber | Ion channel | 181 | 0.507 | 0.846 | 0.0309556 | 0.04 | | 1 | 4 | 0 | 1999 | 2003 |
| Rheumatoid Arthritis | C0003873 | MMP19 | 4327 | Q99542 | matrix metallopeptidase 19 | Enzyme | 64 | 0.623 | 0.615 | 2E-20 | 0.04 | | 1 | 4 | 0 | 1997 | 2004 |
| Rheumatoid Arthritis | C0003873 | PAEP | 5047 | P09466 | progestagen associated endometrial protein | Enzyme | 397 | 0.43 | 0.846 | 3.11E-08 | 0.04 | | 1 | 4 | 0 | 2003 | 2017 |
| Rheumatoid Arthritis | C0003873 | ANGPTL4 | 51129 | Q9BY76 | angiopoietin like 4 | Signaling | 198 | 0.498 | 0.731 | 2.31E-11 | 0.04 | | 1 | 4 | 0 | 2014 | 2017 |
| Rheumatoid Arthritis | C0003873 | PECAM1 | 5175 | P16284 | platelet and endothelial cell adhesion molecule 1 | Cell adhesion | 396 | 0.426 | 0.846 | | 0.04 | | 0.75 | 4 | 3 | 2004 | 2014 |
| Rheumatoi | C0003 | PGF | 5228 | P49763 | placental growth factor | Signaling | 288 | 0.46 | 0.8 | 0.12 | 0.04 | | 1 | 4 | 0 | 2009 | 2015 |

| | | | | | | | | | | | | | | | | | |
|---|---|---|---|---|---|---|---|---|---|---|---|---|---|---|---|---|---|
| d Arthritis | 873 | | | | | | | | 46 | 88 | | | | | | | |
| Rheumatoid Arthritis | C0003873 | PRKCA | 5578 | P17252 | protein kinase C alpha | Kinase | 368 | 0.44 | 0.846 | 0.3677 | 0.04 | | 1 | 4 | 0 | 2006 | 2020 |
| Rheumatoid Arthritis | C0003873 | IL26 | 55801 | Q9NPH9 | interleukin 26 | | 51 | 0.631 | 0.538 | 0.000535 | 0.04 | | 1 | 4 | 0 | 2003 | 2020 |
| Rheumatoid Arthritis | C0003873 | PSMD7 | 5713 | P51665 | proteasome 26S subunit, non-ATPase 7 | Enzyme | 114 | 0.551 | 0.692 | 0.26367 | 0.04 | | 1 | 4 | 0 | 2000 | 2018 |
| Rheumatoid Arthritis | C0003873 | MIR451A | 574411 | | microRNA 451a | | 177 | 0.505 | 0.769 | | 0.04 | | 1 | 4 | 0 | 2014 | 2018 |
| Rheumatoid Arthritis | C0003873 | CCND1 | 595 | P24385 | cyclin D1 | Enzyme modulator | 859 | 0.344 | 0.923 | 0.89057 | 0.04 | | 1 | 4 | 0 | 2001 | 2017 |
| Rheumatoid Arthritis | C0003873 | REG1A | 5967 | P05451 | regenerating family member 1 alpha | | 264 | 0.467 | 0.846 | 1.35E-05 | 0.04 | | 1 | 4 | 0 | 1989 | 2009 |

| | | | | | | | | | | | | | | | | | |
|---|---|---|---|---|---|---|---|---|---|---|---|---|---|---|---|---|---|
| Rheumatoid Arthritis | C0003873 | REN | 5972 | P00797 | renin | Enzyme | 721 | 0.37 | 0.923 | 2.43E-07 | 0.04 | | 1 | 4 | 0 | 2017 | 2020 |
| Rheumatoid Arthritis | C0003873 | S100A1 | 6271 | P23297 | S100 calcium binding protein A1 | Calcium-binding protein | 396 | 0.421 | 0.846 | 0.006004 | 0.04 | | 1 | 4 | 0 | 2002 | 2019 |
| Rheumatoid Arthritis | C0003873 | S100B | 6285 | P04271 | S100 calcium binding protein B | Calcium-binding protein | 599 | 0.383 | 0.923 | 0.04387 | 0.04 | | 1 | 4 | 0 | 2002 | 2019 |
| Rheumatoid Arthritis | C0003873 | CCL3L1 | 6349 | P16619 | C-C motif chemokine ligand 3 like 1 | | 40 | 0.65 | 0.654 | 0.12057 | 0.04 | | 0.75 | 4 | 0 | 2008 | 2016 |
| Rheumatoid Arthritis | C0003873 | CXCL5 | 6374 | P42830 | C-X-C motif chemokine ligand 5 | Signaling | 160 | 0.519 | 0.769 | 5.04E-06 | 0.04 | | 1 | 4 | 0 | 1997 | 2019 |
| Rheumatoid Arthritis | C0003873 | SELL | 6402 | P14151 | selectin L | | 171 | 0.505 | 0.846 | 4.96E-07 | 0.04 | | 1 | 4 | 0 | 2006 | 2015 |

| | | | | | | | | | | | | | | | | |
|---|---|---|---|---|---|---|---|---|---|---|---|---|---|---|---|---|
| Rheumatoid Arthritis | C0003873 | SELP | 6403 | P16109 | selectin P | | 293 | 0.46 | 0.769 | 5.04E-32 | 0.04 | | 0.75 | 4 | 1 | 2007 | 2018 |
| Rheumatoid Arthritis | C0003873 | MAP2K4 | 6416 | P45985 | mitogen-activated protein kinase kinase 4 | Kinase | 100 | 0.559 | 0.692 | 0.99709 | 0.04 | | 1 | 4 | 0 | 2003 | 2015 |
| Rheumatoid Arthritis | C0003873 | SFRP1 | 6422 | Q8N474 | secreted frizzled related protein 1 | | 227 | 0.479 | 0.846 | 0.96457 | 0.04 | | 0.75 | 4 | 0 | 1998 | 2011 |
| Rheumatoid Arthritis | C0003873 | SHBG | 6462 | P04278 | sex hormone binding globulin | | 368 | 0.438 | 0.808 | 1.25E-06 | 0.04 | | 1 | 4 | 0 | 1992 | 2019 |
| Rheumatoid Arthritis | C0003873 | SLC22A5 | 6584 | O76082 | solute carrier family 22 member 5 | Transporter | 103 | 0.608 | 0.731 | 5.27E-16 | 0.04 | | 1 | 4 | 0 | 2005 | 2013 |
| Rheumatoid Arthritis | C0003873 | FSCN1 | 6624 | Q16658 | fascin actin-bundling protein 1 | Cellular structure | 175 | 0.504 | 0.731 | 0.86852 | 0.04 | | 0.75 | 4 | 0 | 1992 | 2003 |
| Rheumatoid Ar | C0003873 | SOX5 | 6660 | P35711 | SRY-box transcription factor 5 | | 150 | 0.545 | 0.769 | 0.99995 | 0.04 | | 1 | 4 | 0 | 2016 | 2018 |

| | | | | | | | | | | | | | | | | | |
|---|---|---|---|---|---|---|---|---|---|---|---|---|---|---|---|---|---|
| thritis | | | | | | | | | | | | | | | | | |
| Rheumatoid Arthritis | C0003873 | TAP1 | 6890 | Q03518 | transporter 1, ATP binding cassette subfamily B member | Transporter | 181 | 0.5 | 0.808 | 5.34E-06 | 0.04 | | 1 | 4 | 2 | 1997 | 2004 |
| Rheumatoid Arthritis | C0003873 | TBX5 | 6910 | Q99593 | T-box transcription factor 5 | Transcription factor | 170 | 0.528 | 0.769 | 0.99954 | 0.04 | | 1 | 4 | 0 | 2014 | 2018 |
| Rheumatoid Arthritis | C0003873 | TPMT | 7172 | P51580 | thiopurine S-methyltransferase | | 83 | 0.593 | 0.692 | 7.11E-06 | 0.04 | | 1 | 4 | 0 | 2003 | 2018 |
| Rheumatoid Arthritis | C0003873 | C5AR1 | 728 | P21730 | complement C5a receptor 1 | G-protein coupled receptor | 179 | 0.513 | 0.808 | 0.40249 | 0.04 | | 1 | 4 | 0 | 2002 | 2018 |
| Rheumatoid Arthritis | C0003873 | TXN | 7295 | P10599 | thioredoxin | | 338 | 0.447 | 0.885 | 0.20119 | 0.04 | | 1 | 4 | 0 | 2006 | 2018 |
| Rheumatoid Arthritis | C0003873 | VWF | 7450 | P04275 | von Willebrand factor | Enzyme modulator | 498 | 0.408 | 0.885 | 3.22E-25 | 0.04 | | 1 | 4 | 0 | 1984 | 2019 |

| | | | | | | | | | | | | | | | | | |
|---|---|---|---|---|---|---|---|---|---|---|---|---|---|---|---|---|---|
| Rheumatoid Arthritis | C0003873 | STEAP4 | 79689 | Q687X5 | STEAP4 metalloreductase | | 34 | 0.686 | 0.462 | 1.2E-07 | 0.04 | | 1 | 4 | 0 | 2009 | 2018 |
| Rheumatoid Arthritis | C0003873 | EHMT1 | 79813 | Q9H9B1 | euchromatic histone lysine methyltransferase 1 | Epigenetic regulator | 371 | 0.46 | 0.808 | 1 | 0.04 | | 1 | 4 | 0 | 2017 | 2018 |
| Rheumatoid Arthritis | C0003873 | CD276 | 80381 | Q5ZPR3 | CD276 molecule | Enzyme modulator | 169 | 0.505 | 0.731 | 2.47E-09 | 0.04 | | 1 | 4 | 0 | 2008 | 2019 |
| Rheumatoid Arthritis | C0003873 | IL1F10 | 84639 | Q8WWZ1 | interleukin 1 family member 10 | | 57 | 0.636 | 0.462 | 2.02E-06 | 0.04 | | 1 | 4 | 0 | 2010 | 2019 |
| Rheumatoid Arthritis | C0003873 | USO1 | 8615 | O60763 | USO1 vesicle transport factor | Transporter | 153 | 0.522 | 0.885 | 0.33417 | 0.04 | | 1 | 4 | 0 | 1994 | 2000 |
| Rheumatoid Arthritis | C0003873 | TP63 | 8626 | Q9H3D4 | tumor protein p63 | Transcription factor | 816 | 0.362 | 0.769 | 0.99728 | 0.04 | | 1 | 4 | 0 | 2000 | 2018 |
| Rheumatoid Ar | C0003873 | CD1C | 911 | P29017 | CD1c molecule | | 49 | 0.633 | 0.692 | 1.6E-08 | 0.04 | | 1 | 4 | 0 | 2017 | 2019 |

| | | | | | | | | | | | | | | | | | |
|---|---|---|---|---|---|---|---|---|---|---|---|---|---|---|---|---|---|
| thritis | | | | | | | | | | | | | | | | | |
| Rheumatoid Arthritis | C0003873 | ARHGEF2 | 9181 | Q92974 | Rho/Rac guanine nucleotide exchange factor 2 | | 205 | 0.502 | 0.769 | 1 | 0.04 | | 1 | 4 | 0 | 2000 | 2018 |
| Rheumatoid Arthritis | C0003873 | TIMD4 | 91937 | Q96H15 | T cell immunoglobulin and mucin domain containing 4 | | 49 | 0.644 | 0.654 | 2.64E-10 | 0.04 | | 0.75 | 4 | 1 | 2012 | 2015 |
| Rheumatoid Arthritis | C0003873 | MSC | 9242 | O60682 | musculin | | 241 | 0.475 | 0.846 | 0.024651 | 0.04 | | 1 | 4 | 0 | 2018 | 2019 |
| Rheumatoid Arthritis | C0003873 | CD27 | 939 | P26842 | CD27 molecule | | 154 | 0.517 | 0.692 | 0.010011 | 0.04 | | 0.75 | 4 | 0 | 1996 | 2018 |
| Rheumatoid Arthritis | C0003873 | ADAM10 | 102 | O14672 | ADAM metallopeptidase domain 10 | | 224 | 0.489 | 0.846 | 1 | 0.03 | | 1 | 3 | 0 | 2015 | 2018 |
| Rheumatoid Arthritis | C0003873 | DDX39A | 10212 | O00148 | DExD-box helicase 39A | | 37 | 0.674 | 0.692 | 0.60151 | 0.03 | | 1 | 3 | 0 | 2001 | 2008 |
| Rheumatoi | C0003 | SPRY2 | 102 | O43597 | sprouty RTK signaling | Signaling | 111 | 0.5 | 0.6 | 0.971 | 0.03 | | 1 | 3 | 0 | 2015 | 2018 |

| | | | | | | | | | | | | | | | | | |
|---|---|---|---|---|---|---|---|---|---|---|---|---|---|---|---|---|---|
| d Arthritis | 873 | | 53 | | antagonist 2 | | | 54 | 54 | 26 | | | | | | | |
| Rheumatoid Arthritis | C0003873 | SIK1B | 1.03E+08 | A0A0B4J2F2;P57059 | salt inducible kinase 1B (putative) | | 48 | 0.653 | 0.385 | | 0.03 | | 1 | 3 | 0 | 2017 | 2019 |
| Rheumatoid Arthritis | C0003873 | KLF2 | 10365 | Q9Y5W3 | Kruppel like factor 2 | Nucleic acid binding | 132 | 0.542 | 0.692 | 0.56044 | 0.03 | | 1 | 3 | 0 | 2015 | 2019 |
| Rheumatoid Arthritis | C0003873 | NOD1 | 10392 | Q9Y239 | nucleotide binding oligomerization domain containing 1 | Enzyme | 136 | 0.541 | 0.769 | 2.46E-18 | 0.03 | | 1 | 3 | 0 | 2008 | 2013 |
| Rheumatoid Arthritis | C0003873 | PDPN | 10630 | Q86YL7 | podoplanin | | 256 | 0.47 | 0.769 | 0.00032 | 0.03 | | 1 | 3 | 0 | 2014 | 2019 |
| Rheumatoid Arthritis | C0003873 | TRAF3IP2 | 10758 | O43734 | TRAF3 interacting protein 2 | | 103 | 0.585 | 0.692 | 1.62E-09 | 0.03 | | 1 | 3 | 0 | 2013 | 2017 |
| Rheumatoid Arthritis | C0003873 | HPSE | 10855 | Q9Y251 | heparanase | Enzyme | 393 | 0.425 | 0.808 | 2.27E-14 | 0.03 | | 1 | 3 | 0 | 2008 | 2017 |

| | | | | | | | | | | | | | | | | | |
|---|---|---|---|---|---|---|---|---|---|---|---|---|---|---|---|---|---|
| Rheumatoid Arthritis | C0003873 | LILRA3 | 11026 | Q8N6C8 | leukocyte immunoglobulin like receptor A3 | Receptor | 19 | 0.722 | 0.5 | 1.45E-06 | 0.03 | | 1 | 3 | 0 | 2010 | 2015 |
| Rheumatoid Arthritis | C0003873 | ADRM1 | 11047 | Q16186 | adhesion regulating molecule 1 | | 51 | 0.633 | 0.538 | 0.99704 | 0.03 | | 1 | 3 | 0 | 1989 | 2007 |
| Rheumatoid Arthritis | C0003873 | ADAMTS5 | 11096 | Q9UNA0 | ADAM metallopeptidase with thrombospondin type 1 motif 5 | Enzyme | 85 | 0.597 | 0.654 | 8.03E-07 | 0.03 | | 1 | 3 | 0 | 2002 | 2012 |
| Rheumatoid Arthritis | C0003873 | CD160 | 11126 | O95971 | CD160 molecule | | 87 | 0.59 | 0.615 | 6.84E-05 | 0.03 | | 1 | 3 | 1 | 1998 | 2012 |
| Rheumatoid Arthritis | C0003873 | SLC7A9 | 11136 | P82251 | solute carrier family 7 member 9 | Transporter | 48 | 0.666 | 0.692 | 9.06E-10 | 0.03 | | 1 | 3 | 0 | 2001 | 2008 |
| Rheumatoid Arthritis | C0003873 | POLG2 | 11232 | Q9UHN1 | DNA polymerase gamma 2, accessory subunit | Enzyme | 158 | 0.558 | 0.769 | 1.53E-10 | 0.03 | | 1 | 3 | 0 | 1992 | 2002 |
| Rheumatoid Ar | C0003873 | PRRT2 | 112476 | Q7Z6L0 | proline rich transmembrane protein 2 | | 353 | 0.448 | 0.808 | 0.57919 | 0.03 | | 1 | 3 | 0 | 2006 | 2012 |

| | | | | | | | | | | | | | | | | | |
|---|---|---|---|---|---|---|---|---|---|---|---|---|---|---|---|---|---|
| thritis | | | | | | | | | | | | | | | | | |
| Rheumatoid Arthritis | C0003873 | ACOT7 | 11332 | O00154 | acyl-CoA thioesterase 7 | Enzyme | 146 | 0.531 | 0.769 | 0.759926 | 0.03 | | 1 | 3 | 0 | 2017 | 2019 |
| Rheumatoid Arthritis | C0003873 | CTHRC1 | 115908 | Q96CG8 | collagen triple helix repeat containing 1 | Enzyme | 105 | 0.556 | 0.692 | 2.67E-05 | 0.03 | | 1 | 3 | 0 | 2012 | 2019 |
| Rheumatoid Arthritis | C0003873 | H4-16 | 121504 | P62805 | H4 histone 16 | Nucleic acid binding | 122 | 0.54 | 0.692 | 0.0222279 | 0.03 | | 1 | 3 | 0 | 2018 | 2019 |
| Rheumatoid Arthritis | C0003873 | CCR1 | 1230 | P32246 | C-C motif chemokine receptor 1 | G-protein coupled receptor | 192 | 0.531 | 0.769 | 0.38775 | 0.03 | | 1 | 3 | 0 | 2005 | 2018 |
| Rheumatoid Arthritis | C0003873 | OSCAR | 126014 | Q8IYS5 | osteoclast associated Ig-like receptor | | 17 | 0.743 | 0.462 | 0.000336 | 0.03 | | 1 | 3 | 0 | 2011 | 2017 |
| Rheumatoid Arthritis | C0003873 | SIK1 | 150094 | A0A0B4J2F2;P57059 | salt inducible kinase 1 | Kinase | 157 | 0.544 | 0.731 | 0.93716 | 0.03 | | 1 | 3 | 0 | 2017 | 2019 |
| Rheu | C00 | CD55 | 16 | P08174 | CD55 molecule | | 187 | 0. | 0. | 5.6 | 0.03 | | 1 | 3 | 0 | 2010 | 20 |

| | | | | | | | | | | | | | | | | | |
|---|---|---|---|---|---|---|---|---|---|---|---|---|---|---|---|---|---|
| matoid Arthritis | 03873 | | 04 | | (Cromer blood group) | | | 51 | 808 | 8E-12 | | | | | | | 19 |
| Rheumatoid Arthritis | C0003873 | DBP | 1628 | Q10586 | D-box binding PAR bZIP transcription factor | Nucleic acid binding | 105 | 0.556 | 0.731 | 0.233376 | 0.03 | | 1 | 3 | 0 | 2014 | 2018 |
| Rheumatoid Arthritis | C0003873 | GADD45A | 1647 | P24522 | growth arrest and DNA damage inducible alpha | | 151 | 0.526 | 0.769 | 0.0440075 | 0.03 | | 1 | 3 | 1 | 2008 | 2019 |
| Rheumatoid Arthritis | C0003873 | DNMT3B | 1789 | Q9UBC3 | DNA methyltransferase 3 beta | | 315 | 0.453 | 0.846 | 0.199964 | 0.03 | | 1 | 3 | 0 | 2010 | 2018 |
| Rheumatoid Arthritis | C0003873 | DPEP1 | 1800 | P16444 | dipeptidase 1 | | 100 | 0.57 | 0.654 | 3.47E-10 | 0.03 | | 1 | 3 | 0 | 2017 | 2019 |
| Rheumatoid Arthritis | C0003873 | E2F2 | 1870 | Q14209 | E2F transcription factor 2 | Nucleic acid binding | 75 | 0.599 | 0.692 | 0.25505 | 0.03 | | 1 | 3 | 0 | 2014 | 2018 |
| Rheumatoid Arthritis | C0003873 | TYMP | 1890 | P19971 | thymidine phosphorylase | Enzyme | 246 | 0.495 | 0.769 | 0.000117 | 0.03 | | 1 | 3 | 0 | 2014 | 2019 |

| | | | | | | | | | | | | | | | | | |
|---|---|---|---|---|---|---|---|---|---|---|---|---|---|---|---|---|---|
| Rheumatoid Arthritis | C0003873 | ELAVL2 | 1993 | Q12926 | ELAV like RNA binding protein 2 | | 199 | 0.486 | 0.808 | 0.99514 | 0.03 | | 1 | 3 | 0 | 2019 | 2019 |
| Rheumatoid Arthritis | C0003873 | PRSS55 | 203074 | Q6UWB4 | serine protease 55 | Enzyme | 163 | 0.513 | 0.769 | 3.17E-11 | 0.03 | | 1 | 3 | 0 | 2006 | 2017 |
| Rheumatoid Arthritis | C0003873 | EPHB1 | 2047 | P54762 | EPH receptor B1 | Kinase | 169 | 0.518 | 0.846 | 0.99985 | 0.03 | | 1 | 3 | 0 | 2008 | 2020 |
| Rheumatoid Arthritis | C0003873 | ERBB2 | 2064 | P04626 | erb-b2 receptor tyrosine kinase 2 | Kinase | 995 | 0.328 | 0.923 | 0.005989 | 0.03 | | 1 | 3 | 0 | 2005 | 2017 |
| Rheumatoid Arthritis | C0003873 | ERG | 2078 | P11308 | ETS transcription factor ERG | Transcription factor | 298 | 0.453 | 0.808 | 0.96413 | 0.03 | | 1 | 3 | 0 | 1992 | 2002 |
| Rheumatoid Arthritis | C0003873 | EZH2 | 2146 | Q15910 | enhancer of zeste 2 polycomb repressive complex 2 subunit | Epigenetic regulator | 653 | 0.376 | 0.885 | 1 | 0.03 | | 1 | 3 | 0 | 2011 | 2019 |
| Rheumatoi | C0003 | FCGR1B | 2210 | Q92637 | Fc fragment of IgG | Cell ad | 57 | 0.6 | 0.6 | 1.34E | 0.03 | | 1 | 3 | 0 | 1997 | 2019 |

| | | | | | | | | | | | | | | | | | |
|---|---|---|---|---|---|---|---|---|---|---|---|---|---|---|---|---|---|
| d Arthritis | 873 | | | | receptor Ib | hesion | | 33 | 92 | -10 | | | | | | | |
| Rheumatoid Arthritis | C0003873 | FCN1 | 2219 | O00602 | ficolin 1 | Signaling | 43 | 0.647 | 0.577 | 3.97E-17 | 0.03 | | 0.667 | 3 | 1 | 2007 | 2019 |
| Rheumatoid Arthritis | C0003873 | FHL1 | 2273 | Q13642 | four and a half LIM domains 1 | | 195 | 0.532 | 0.731 | 0.96979 | 0.03 | | 1 | 3 | 0 | 2000 | 2017 |
| Rheumatoid Arthritis | C0003873 | FLG | 2312 | P20930 | filaggrin | Cellular structure | 173 | 0.525 | 0.846 | 0.000288 | 0.03 | | 1 | 3 | 0 | 1999 | 2019 |
| Rheumatoid Arthritis | C0003873 | ACSBG1 | 2205 | Q96GR2 | acyl-CoA synthetase bubblegum family member 1 | Enzyme | 73 | 0.603 | 0.654 | 3.96E-05 | 0.03 | | 1 | 3 | 0 | 2007 | 2017 |
| Rheumatoid Arthritis | C0003873 | FLT3LG | 2323 | P49771 | fms related receptor tyrosine kinase 3 ligand | | 100 | 0.564 | 0.577 | 0.9404 | 0.03 | | 1 | 3 | 0 | 2008 | 2013 |
| Rheumatoid Arthritis | C0003873 | CLEC16A | 2274 | Q2KHT3 | C-type lectin domain containing 16A | | 67 | 0.612 | 0.654 | 0.27595 | 0.03 | | 0.667 | 3 | 4 | 2010 | 2010 |

| | | | | | | | | | | | | | | | | | |
|---|---|---|---|---|---|---|---|---|---|---|---|---|---|---|---|---|---|
| Rheumatoid Arthritis | C0003873 | DICER1 | 23405 | Q9UPY3 | dicer 1, ribonuclease III | Enzyme | 302 | 0.446 | 0.846 | 1 | 0.03 | | 1 | 3 | 0 | 2013 | 2018 |
| Rheumatoid Arthritis | C0003873 | SMUG1 | 23583 | Q53HV7 | single-strand-selective monofunctional uracil-DNA glycosylase 1 | | 1034 | 0.322 | 0.923 | 0.000452 | 0.03 | | 1 | 3 | 0 | 2017 | 2019 |
| Rheumatoid Arthritis | C0003873 | NUP62 | 23636 | P37198 | nucleoporin 62 | Transporter | 273 | 0.47 | 0.885 | 0.95006 | 0.03 | | 1 | 3 | 0 | 2018 | 2019 |
| Rheumatoid Arthritis | C0003873 | SH3BP4 | 23677 | Q9P0V3 | SH3 domain binding protein 4 | | 74 | 0.593 | 0.692 | 0.002534 | 0.03 | | 1 | 3 | 0 | 2017 | 2019 |
| Rheumatoid Arthritis | C0003873 | ALOX15 | 246 | P16050 | arachidonate 15-lipoxygenase | Enzyme | 205 | 0.502 | 0.846 | 1.14E-23 | 0.03 | | 1 | 3 | 0 | 1999 | 2013 |
| Rheumatoid Arthritis | C0003873 | FRZB | 2487 | Q92765 | frizzled related protein | | 89 | 0.573 | 0.654 | 0.001084 | 0.03 | | 0.667 | 3 | 0 | 1998 | 2017 |
| Rheumatoid Ar | C0003873 | AOC1 | 26 | P19801 | amine oxidase copper containing 1 | | 61 | 0.628 | 0.769 | 6.72E- | 0.03 | | 1 | 3 | 0 | 2018 | 2019 |

| | | | | | | | | | | | | | | | | | |
|---|---|---|---|---|---|---|---|---|---|---|---|---|---|---|---|---|---|
| thritis | | | | | | | | | | 08 | | | | | | | |
| Rheumatoid Arthritis | C0003873 | COPD | 260431 | | Pulmonary disease, chronic obstructive, severe early-onset | | 349 | 0.456 | 0.885 | | 0.03 | | 1 | 3 | 0 | 2017 | 2019 |
| Rheumatoid Arthritis | C0003873 | MSTN | 2660 | O14793 | myostatin | Signaling | 159 | 0.528 | 0.731 | 0.5052 | 0.03 | | 1 | 3 | 0 | 2017 | 2019 |
| Rheumatoid Arthritis | C0003873 | GDNF | 2668 | P39905 | glial cell derived neurotrophic factor | Signaling | 409 | 0.434 | 0.885 | 0.2035 | 0.03 | | 1 | 3 | 0 | 2000 | 2018 |
| Rheumatoid Arthritis | C0003873 | SGSM3 | 27352 | Q96HU1 | small G protein signaling modulator 3 | Enzyme | 259 | 0.469 | 0.808 | 3.41E-15 | 0.03 | | 1 | 3 | 0 | 2004 | 2018 |
| Rheumatoid Arthritis | C0003873 | GLS | 2744 | O94925 | glutaminase | Enzyme | 217 | 0.489 | 0.808 | 0.96154 | 0.03 | | 1 | 3 | 0 | 2007 | 2017 |
| Rheumatoid Arthritis | C0003873 | PYCARD | 29108 | Q9ULZ3 | PYD and CARD domain containing | Enzyme | 294 | 0.457 | 0.808 | 0.000198 | 0.03 | | 1 | 3 | 0 | 2005 | 2019 |
| Rheumatoi | C0003 | CXCL1 | 2919 | P09341 | C-X-C motif chemoki | Signaling | 315 | 0.4 | 0.9 | 0.118 | 0.03 | | 1 | 3 | 0 | 1999 | 2017 |

| | | | | | | | | | | | | | | | | | |
|---|---|---|---|---|---|---|---|---|---|---|---|---|---|---|---|---|---|
| d Arthritis | 873 | | | | ne ligand 1 | | | 49 | 23 | 45 | | | | | | | |
| Rheumatoid Arthritis | C0003873 | GTF2H1 | 2965 | P32780 | general transcription factor IIH subunit 1 | Transcription factor | 258 | 0.469 | 0.846 | 0.989982 | 0.03 | | 1 | 3 | 0 | 2018 | 2019 |
| Rheumatoid Arthritis | C0003873 | GTF2I | 2969 | P78347 | general transcription factor IIi | | 249 | 0.5 | 0.808 | 0.999592 | 0.03 | | 1 | 3 | 1 | 2016 | 2019 |
| Rheumatoid Arthritis | C0003873 | GUSB | 2990 | P08236 | glucuronidase beta | | 183 | 0.53 | 0.846 | 4.04E-08 | 0.03 | | 1 | 3 | 0 | 2003 | 2014 |
| Rheumatoid Arthritis | C0003873 | GZMA | 3001 | P12544 | granzyme A | Enzyme | 65 | 0.597 | 0.731 | 2.05E-06 | 0.03 | | 1 | 3 | 0 | 1992 | 2017 |
| Rheumatoid Arthritis | C0003873 | HFE | 3077 | Q30201 | homeostatic iron regulator | | 415 | 0.436 | 0.846 | 2.56E-08 | 0.03 | | 1 | 3 | 2 | 2000 | 2006 |
| Rheumatoid Arthritis | C0003873 | HLA-DRB5 | 3127 | Q30154 | major histocompatibility complex, class II, DR beta 5 | Immune response | 62 | 0.608 | 0.731 | 3.64E-07 | 0.03 | | 1 | 3 | 0 | 1990 | 2019 |

| | | | | | | | | | | | | | | | | | |
|---|---|---|---|---|---|---|---|---|---|---|---|---|---|---|---|---|---|
| Rheumatoid Arthritis | C0003873 | HMOX1 | 3162 | P09601 | heme oxygenase 1 | | 666 | 0.381 | 0.923 | 0.009682 | 0.03 | | 1 | 3 | 0 | 2007 | 2010 |
| Rheumatoid Arthritis | C0003873 | HOXC6 | 3223 | P09630 | homeobox C6 | Transcription factor | 80 | 0.595 | 0.692 | 0.51165 | 0.03 | | 1 | 3 | 1 | 2019 | 2019 |
| Rheumatoid Arthritis | C0003873 | HSP90AA1 | 3320 | P07900 | heat shock protein 90 alpha family class A member 1 | Chaperone | 455 | 0.411 | 0.923 | 0.86025 | 0.03 | | 1 | 3 | 0 | 2018 | 2019 |
| Rheumatoid Arthritis | C0003873 | APOB | 338 | P04114 | apolipoprotein B | | 339 | 0.453 | 0.808 | 1.92E-16 | 0.03 | | 1 | 3 | 0 | 2012 | 2019 |
| Rheumatoid Arthritis | C0003873 | IDDM8 | 3407 | | insulin dependent diabetes mellitus 8 | | 7 | 0.821 | 0.192 | | 0.03 | | 0.667 | 3 | 0 | 2000 | 2010 |
| Rheumatoid Arthritis | C0003873 | ACTBL2 | 345651 | Q562R1 | actin beta like 2 | | 134 | 0.539 | 0.769 | 5.82E-16 | 0.03 | | 1 | 3 | 0 | 2017 | 2019 |
| Rheumatoid Ar | C0003873 | H3P44 | 347376 | | H3 histone pseudogene 44 | | 40 | 0.636 | 0.462 | | 0.03 | | 1 | 3 | 0 | 1992 | 2002 |

| | | | | | | | | | | | | | | | | | |
|---|---|---|---|---|---|---|---|---|---|---|---|---|---|---|---|---|---|
| thritis | | | | | | | | | | | | | | | | | |
| Rheumatoid Arthritis | C0003873 | IGF2 | 3481 | P01344 | insulin like growth factor 2 | | 604 | 0.39 | 0.885 | 0.044127 | 0.03 | | 1 | 3 | 0 | 1995 | 2010 |
| Rheumatoid Arthritis | C0003873 | IL1R1 | 3554 | P14778 | interleukin 1 receptor type 1 | Receptor | 184 | 0.509 | 0.769 | 0.151126 | 0.03 | | 1 | 3 | 0 | 1997 | 2015 |
| Rheumatoid Arthritis | C0003873 | CXCR1 | 3577 | P25024 | C-X-C motif chemokine receptor 1 | G-protein coupled receptor | 167 | 0.508 | 0.769 | 3.03E-07 | 0.03 | | 1 | 3 | 0 | 2003 | 2019 |
| Rheumatoid Arthritis | C0003873 | AQP1 | 358 | P29972 | aquaporin 1 (Colton blood group) | Ion channel | 268 | 0.467 | 0.885 | 2.57E-07 | 0.03 | | 1 | 3 | 0 | 2013 | 2018 |
| Rheumatoid Arthritis | C0003873 | TNFRSF9 | 3604 | Q07011 | TNF receptor superfamily member 9 | | 177 | 0.502 | 0.769 | 0.32153 | 0.03 | | 1 | 3 | 0 | 1998 | 2008 |
| Rheumatoid Arthritis | C0003873 | IRF1 | 3659 | P10914 | interferon regulatory factor 1 | Transcription factor | 257 | 0.468 | 0.769 | 0.9909 | 0.03 | | 1 | 3 | 0 | 2012 | 2016 |

| | | | | | | | | | | | | | | | | | |
|---|---|---|---|---|---|---|---|---|---|---|---|---|---|---|---|---|---|
| Rheumatoid Arthritis | C0003873 | IRS1 | 3667 | P35568 | insulin receptor substrate 1 | | 233 | 0.488 | 0.769 | 0.023645 | 0.03 | | 1 | 3 | 1 | 2015 | 2018 |
| Rheumatoid Arthritis | C0003873 | ITGA4 | 3676 | P13612 | integrin subunit alpha 4 | | 123 | 0.558 | 0.769 | 0.001001 | 0.03 | | 1 | 3 | 0 | 2000 | 2017 |
| Rheumatoid Arthritis | C0003873 | ABCC6 | 368 | O95255 | ATP binding cassette subfamily C member 6 | Transporter | 181 | 0.54 | 0.654 | 5.62E-35 | 0.03 | | 1 | 3 | 0 | 1988 | 2017 |
| Rheumatoid Arthritis | C0003873 | ITGAL | 3683 | P20701 | integrin subunit alpha L | | 169 | 0.511 | 0.885 | 0.004321 | 0.03 | | 1 | 3 | 0 | 1988 | 2016 |
| Rheumatoid Arthritis | C0003873 | ITGB2 | 3689 | P05107 | integrin subunit beta 2 | Receptor | 340 | 0.444 | 0.923 | 5.08E-15 | 0.03 | | 1 | 3 | 0 | 1988 | 2009 |
| Rheumatoid Arthritis | C0003873 | KIR2DL1 | 3802 | P43626 | killer cell immunoglobulin like receptor, two Ig domains and long cytoplasmic tail 1 | Receptor | 49 | 0.631 | 0.615 | 3.57E-15 | 0.03 | | 1 | 3 | 0 | 2007 | 2015 |

| | | | | | | | | | | | | | | | | | |
|---|---|---|---|---|---|---|---|---|---|---|---|---|---|---|---|---|---|
| Rheumatoid Arthritis | C0003873 | KLRC2 | 3822 | P26717 | killer cell lectin like receptor C2 | | 50 | 0.636 | 0.731 | 4.87E-10 | 0.03 | | 1 | 3 | 0 | 2003 | 2008 |
| Rheumatoid Arthritis | C0003873 | KLRD1 | 3824 | Q13241 | killer cell lectin like receptor D1 | | 53 | 0.636 | 0.692 | 0.000875 | 0.03 | | 1 | 3 | 1 | 2003 | 2016 |
| Rheumatoid Arthritis | C0003873 | NPSR1 | 387129 | Q6W5P4 | neuropeptide S receptor 1 | | 69 | 0.608 | 0.615 | 2.05E-09 | 0.03 | | 1 | 3 | 3 | 2010 | 2019 |
| Rheumatoid Arthritis | C0003873 | LRP5 | 4041 | O75197 | LDL receptor related protein 5 | | 283 | 0.485 | 0.846 | 0.50992 | 0.03 | | 1 | 3 | 3 | 2009 | 2018 |
| Rheumatoid Arthritis | C0003873 | LTB | 4050 | Q06643 | lymphotoxin beta | Signaling | 110 | 0.555 | 0.769 | 9.61E-11 | 0.03 | | 1 | 3 | 0 | 1999 | 2015 |
| Rheumatoid Arthritis | C0003873 | ARNTL | 406 | O00327 | aryl hydrocarbon receptor nuclear translocator like | Transcription factor | 200 | 0.51 | 0.885 | 0.99444 | 0.03 | | 1 | 3 | 0 | 2013 | 2015 |
| Rheumatoid Ar | C0003873 | MIR140 | 406932 | | microRNA 140 | | 144 | 0.522 | 0.769 | | 0.03 | | 1 | 3 | 0 | 2016 | 2018 |

| | | | | | | | | | | | | | | | | | |
|---|---|---|---|---|---|---|---|---|---|---|---|---|---|---|---|---|---|
| thritis | | | | | | | | | | | | | | | | | |
| Rheumatoid Arthritis | C0003873 | MIR143 | 406935 | | microRNA 143 | | 267 | 0.462 | 0.885 | | 0.03 | | 1 | 3 | 0 | 2017 | 2018 |
| Rheumatoid Arthritis | C0003873 | MIR20A | 406982 | | microRNA 20a | | 180 | 0.498 | 0.769 | | 0.03 | | 1 | 3 | 0 | 2016 | 2018 |
| Rheumatoid Arthritis | C0003873 | MIR23B | 407011 | | microRNA 23b | | 137 | 0.523 | 0.808 | | 0.03 | | 1 | 3 | 0 | 2012 | 2019 |
| Rheumatoid Arthritis | C0003873 | MIR27A | 407018 | | microRNA 27a | | 248 | 0.466 | 0.846 | | 0.03 | | 1 | 3 | 0 | 2016 | 2019 |
| Rheumatoid Arthritis | C0003873 | MIR17HG | 407975 | Q75NE6 | miR-17-92a-1 cluster host gene | | 224 | 0.483 | 0.808 | | 0.03 | | 1 | 3 | 0 | 2013 | 2018 |
| Rheumatoid Arthritis | C0003873 | SMAD3 | 4088 | P84022 | SMAD family member 3 | Transcription factor | 470 | 0.415 | 0.923 | 0.7976 | 0.03 | 0.333 | 3 | 3 | | 2013 | 2019 |
| Rheumatoi | C0003 | MAP3K5 | 4217 | Q99683 | mitogen-activated protein | Kinase | 170 | 0.5 | 0.8 | 0.519 | 0.03 | | 1 | 3 | 0 | 2005 | 2018 |

| | | | | | | | | | | | | | | | | | |
|---|---|---|---|---|---|---|---|---|---|---|---|---|---|---|---|---|---|
| d Arthritis | 873 | | | | kinase kinase kinase 5 | | | 13 | 08 | 64 | | | | | | | |
| Rheumatoid Arthritis | C0003873 | FOXO4 | 4303 | P98177 | forkhead box O4 | | 84 | 0.588 | 0.769 | 0.81332 | 0.03 | | 1 | 3 | 0 | 2007 | 2019 |
| Rheumatoid Arthritis | C0003873 | MPG | 4350 | P29372 | N-methylpurine DNA glycosylase | Enzyme | 74 | 0.597 | 0.731 | 1.95E-12 | 0.03 | | 1 | 3 | 4 | 2010 | 2019 |
| Rheumatoid Arthritis | C0003873 | MPP1 | 4354 | Q00013 | membrane palmitoylated protein 1 | Enzyme | 54 | 0.617 | 0.5 | 0.98777 | 0.03 | | 1 | 3 | 0 | 1992 | 2002 |
| Rheumatoid Arthritis | C0003873 | POTEKP | 440915 | Q9BYX7 | POTE ankyrin domain family member K, pseudogene | | 131 | 0.542 | 0.769 | | 0.03 | | 1 | 3 | 0 | 2017 | 2019 |
| Rheumatoid Arthritis | C0003873 | MIR323A | 442897 | | microRNA 323a | | 46 | 0.641 | 0.577 | | 0.03 | | 1 | 3 | 0 | 2012 | 2014 |
| Rheumatoid Arthritis | C0003873 | MIR338 | 442906 | | microRNA 338 | | 128 | 0.535 | 0.808 | | 0.03 | | 1 | 3 | 0 | 2017 | 2018 |

| | | | | | | | | | | | | | | | | | |
|---|---|---|---|---|---|---|---|---|---|---|---|---|---|---|---|---|---|
| Rheumatoid Arthritis | C0003873 | MSRA | 4482 | Q9UJ68 | methionine sulfoxide reductase A | Enzyme | 89 | 0.608 | 0.615 | 1.53E-15 | 0.03 | | 1 | 3 | 1 | 2010 | 2013 |
| Rheumatoid Arthritis | C0003873 | MTR | 4548 | Q99707 | 5-methyltetrahydrofolate-homocysteine methyltransferase | | 245 | 0.482 | 0.808 | 2.43E-12 | 0.03 | | 1 | 3 | 1 | 2007 | 2018 |
| Rheumatoid Arthritis | C0003873 | MTRR | 4552 | Q9UBK8 | 5-methyltetrahydrofolate-homocysteine methyltransferase reductase | | 207 | 0.502 | 0.808 | 2.18E-14 | 0.03 | | 0.667 | 3 | 1 | 2007 | 2018 |
| Rheumatoid Arthritis | C0003873 | MUC1 | 4582 | P15941 | mucin 1, cell surface associated | | 594 | 0.384 | 0.885 | 0.018774 | 0.03 | | 1 | 3 | 0 | 2008 | 2018 |
| Rheumatoid Arthritis | C0003873 | MYC | 4609 | P01106 | MYC proto-oncogene, bHLH transcription factor | Transcription factor | 821 | 0.344 | 0.923 | 0.998801 | 0.03 | | 1 | 3 | 0 | 1990 | 2004 |
| Rheumatoid Arthritis | C0003873 | GADD45B | 4616 | O75293 | growth arrest and DNA damage inducible beta | | 76 | 0.585 | 0.769 | 0.210058 | 0.03 | | 1 | 3 | 1 | 2009 | 2019 |
| Rheumatoid Ar | C0003873 | NCAM1 | 4684 | P13591 | neural cell adhesion molecule 1 | | 445 | 0.415 | 0.885 | 0.99999 | 0.03 | | 1 | 3 | 0 | 1996 | 2014 |

| | | | | | | | | | | | | | | | | | |
|---|---|---|---|---|---|---|---|---|---|---|---|---|---|---|---|---|---|
| thritis | | | | | | | | | | | | | | | | | |
| Rheumatoid Arthritis | C0003873 | NFKBIA | 4792 | P25963 | NFKB inhibitor alpha | | 226 | 0.487 | 0.885 | 0.99452 | 0.03 | | 1 | 3 | 0 | 2007 | 2019 |
| Rheumatoid Arthritis | C0003873 | DDR2 | 4921 | Q16832 | discoidin domain receptor tyrosine kinase 2 | Kinase | 147 | 0.541 | 0.769 | 0.5503 | 0.03 | | 1 | 3 | 0 | 2006 | 2017 |
| Rheumatoid Arthritis | C0003873 | OGG1 | 4968 | O15527 | 8-oxoguanine DNA glycosylase | | 313 | 0.453 | 0.808 | 3.93E-12 | 0.03 | | 0.333 | 3 | 4 | 2012 | 2016 |
| Rheumatoid Arthritis | C0003873 | OLR1 | 4973 | P78380 | oxidized low density lipoprotein receptor 1 | | 133 | 0.544 | 0.769 | 0.007199 | 0.03 | | 1 | 3 | 0 | 2004 | 2019 |
| Rheumatoid Arthritis | C0003873 | P4HB | 5034 | P07237 | prolyl 4-hydroxylase subunit beta | | 222 | 0.488 | 0.808 | 0.99662 | 0.03 | | 1 | 3 | 0 | 1992 | 2002 |
| Rheumatoid Arthritis | C0003873 | PCSK6 | 5046 | P29122 | proprotein convertase subtilisin/kexin type 6 | Enzyme | 65 | 0.626 | 0.538 | 5.31E-13 | 0.03 | | 1 | 3 | 1 | 2015 | 2017 |
| Rheumatoi | C0003 | PAK3 | 5063 | O75914 | p21 (RAC1) activate | Kinase | 134 | 0.5 | 0.7 | 0.987 | 0.03 | | 1 | 3 | 0 | 1988 | 2017 |

| | | | | | | | | | | | | | | | | | |
|---|---|---|---|---|---|---|---|---|---|---|---|---|---|---|---|---|---|
| d Arthritis | 873 | | | | d kinase 3 | | | 69 | 31 | 16 | | | | | | | |
| Rheumatoid Arthritis | C0003873 | DCTN4 | 51164 | Q9UJW0 | dynactin subunit 4 | | 253 | 0.472 | 0.846 | 0.73221 | 0.03 | | 1 | 3 | 0 | 2018 | 2019 |
| Rheumatoid Arthritis | C0003873 | GP6 | 51206 | Q9HCN6 | glycoprotein VI platelet | Receptor | 89 | 0.597 | 0.615 | 1.64E-11 | 0.03 | | 0.667 | 3 | 0 | 2013 | 2019 |
| Rheumatoid Arthritis | C0003873 | TLR8 | 51311 | Q9NR97 | toll like receptor 8 | | 122 | 0.543 | 0.769 | 0.32401 | 0.03 | | 1 | 3 | 0 | 2008 | 2019 |
| Rheumatoid Arthritis | C0003873 | PDE3A | 5139 | Q14432 | phosphodiesterase 3A | | 58 | 0.67 | 0.577 | 0.000193 | 0.03 | | 0.667 | 3 | 0 | 2013 | 2016 |
| Rheumatoid Arthritis | C0003873 | NCKIPSD | 51517 | Q9NZQ3 | NCK interacting protein with SH3 domain | | 62 | 0.612 | 0.577 | 0.000223 | 0.03 | | 1 | 3 | 0 | 2000 | 2006 |
| Rheumatoid Arthritis | C0003873 | SIRT6 | 51548 | Q8N6T7 | sirtuin 6 | Epigenetic regulator | 195 | 0.496 | 0.846 | 0.039119 | 0.03 | | 1 | 3 | 0 | 2013 | 2018 |

| | | | | | | | | | | | | | | | | | |
|---|---|---|---|---|---|---|---|---|---|---|---|---|---|---|---|---|---|
| Rheumatoid Arthritis | C0003873 | PADI3 | 51702 | Q9ULW8 | peptidyl arginine deiminase 3 | | 21 | 0.78 | 0.192 | 2.36E-10 | 0.03 | | 0.667 | 3 | 0 | 2007 | 2018 |
| Rheumatoid Arthritis | C0003873 | SERPINF1 | 5176 | P36955 | serpin family F member 1 | Enzyme modulator | 294 | 0.46 | 0.846 | 7.8E-08 | 0.03 | | 1 | 3 | 0 | 2016 | 2018 |
| Rheumatoid Arthritis | C0003873 | PF4 | 5196 | P02776 | platelet factor 4 | Signaling | 158 | 0.522 | 0.846 | 0.0430662 | 0.03 | | 1 | 3 | 0 | 2007 | 2019 |
| Rheumatoid Arthritis | C0003873 | PFKFB3 | 5209 | Q16875 | 6-phosphofructo-2-kinase/fructose-2,6-biphosphatase 3 | Kinase | 104 | 0.561 | 0.808 | 0.24579 | 0.03 | | 1 | 3 | 0 | 2013 | 2017 |
| Rheumatoid Arthritis | C0003873 | PIK3R2 | 5296 | O00459 | phosphoinositide-3-kinase regulatory subunit 2 | Enzyme modulator | 120 | 0.551 | 0.808 | 0.016273 | 0.03 | | 1 | 3 | 0 | 2016 | 2019 |
| Rheumatoid Arthritis | C0003873 | SLCO1C1 | 53919 | Q9NYB5 | solute carrier organic anion transporter family member 1C1 | Transporter | 20 | 0.751 | 0.423 | 3.99E-12 | 0.03 | | 0.667 | 3 | 1 | 2013 | 2016 |
| Rheumatoid Ar | C0003873 | ACP5 | 54 | P13686 | acid phosphatase 5, tartrate resistant | | 130 | 0.561 | 0.731 | 0.0786 | 0.03 | | 1 | 3 | 0 | 2002 | 2017 |

| | | | | | | | | | | | | | | | | | |
|---|---|---|---|---|---|---|---|---|---|---|---|---|---|---|---|---|---|
| thritis | | | | | | | | | | 51 | | | | | | | |
| Rheumatoid Arthritis | C0003873 | PPIA | 5478 | P62937 | peptidylprolyl isomerase A | | 180 | 0.51 | 0.769 | 0.557734 | 0.03 | | 1 | 3 | 0 | 2008 | 2011 |
| Rheumatoid Arthritis | C0003873 | ASPN | 54829 | Q9BXN1 | asporin | | 51 | 0.636 | 0.5 | 1.04E-07 | 0.03 | | 0.667 | 3 | 0 | 2007 | 2018 |
| Rheumatoid Arthritis | C0003873 | SLC52A1 | 55065 | Q9NWF4 | solute carrier family 52 member 1 | Transporter | 160 | 0.526 | 0.769 | 3.52E-06 | 0.03 | | 1 | 3 | 0 | 2007 | 2012 |
| Rheumatoid Arthritis | C0003873 | ADI1 | 55256 | Q9BV57 | acireductone dioxygenase 1 | Enzyme | 36 | 0.674 | 0.654 | 3.15E-05 | 0.03 | | 1 | 3 | 0 | 2013 | 2017 |
| Rheumatoid Arthritis | C0003873 | MEG3 | 55384 | | maternally expressed 3 | | 239 | 0.471 | 0.846 | | 0.03 | | 1 | 3 | 0 | 2019 | 2019 |
| Rheumatoid Arthritis | C0003873 | H4C15 | 554313 | P62805 | H4 clustered histone 15 | | 121 | 0.541 | 0.692 | | 0.03 | | 1 | 3 | 0 | 2018 | 2019 |
| Rheumatoi | C0003 | PRKCB | 5579 | P05771 | protein kinase C beta | Kinase | 317 | 0.4 | 0.8 | 1 | 0.03 | | 1 | 3 | 0 | 2006 | 2012 |

| | | | | | | | | | | | | | | | | | |
|---|---|---|---|---|---|---|---|---|---|---|---|---|---|---|---|---|---|
| d Arthritis | 873 | | | | | | | 53 | 85 | | | | | | | | |
| Rheumatoid Arthritis | C0003873 | PROC | 5624 | P04070 | protein C, inactivator of coagulation factors Va and VIIIa | Enzyme | 200 | 0.504 | 0.808 | 1.91E-05 | 0.03 | | 1 | 3 | 0 | 2004 | 2019 |
| Rheumatoid Arthritis | C0003873 | HTRA1 | 5654 | Q92743 | HtrA serine peptidase 1 | Enzyme | 164 | 0.532 | 0.692 | 0.000492 | 0.03 | | 1 | 3 | 0 | 2013 | 2018 |
| Rheumatoid Arthritis | C0003873 | PSMD12 | 5718 | O00232 | proteasome 26S subunit, non-ATPase 12 | Enzyme modulator | 105 | 0.578 | 0.808 | 0.99984 | 0.03 | | 1 | 3 | 0 | 1992 | 2002 |
| Rheumatoid Arthritis | C0003873 | PTCH1 | 5727 | Q13635 | patched 1 | | 604 | 0.398 | 0.885 | 1 | 0.03 | | 1 | 3 | 0 | 2014 | 2018 |
| Rheumatoid Arthritis | C0003873 | PTHLH | 5744 | P12272 | parathyroid hormone like hormone | | 321 | 0.453 | 0.846 | 0.81146 | 0.03 | | 1 | 3 | 0 | 1998 | 2015 |
| Rheumatoid Arthritis | C0003873 | SEMA6A | 5756 | Q9H2E6 | semaphorin 6A | Signaling | 211 | 0.486 | 0.808 | 0.99976 | 0.03 | | 1 | 3 | 0 | 2017 | 2019 |

| | | | | | | | | | | | | | | | | |
|---|---|---|---|---|---|---|---|---|---|---|---|---|---|---|---|---|
| Rheumatoid Arthritis | C0003873 | SIGIRR | 59307 | Q6IA17 | single Ig and TIR domain containing | Receptor | 36 | 0.67 | 0.615 | 5.93E-06 | 0.03 | | 1 | 3 | 0 | 2010 | 2020 |
| Rheumatoid Arthritis | C0003873 | HRH4 | 59340 | Q9H3N8 | histamine receptor H4 | G-protein coupled receptor | 48 | 0.659 | 0.462 | 5.22E-08 | 0.03 | | 1 | 3 | 0 | 2005 | 2017 |
| Rheumatoid Arthritis | C0003873 | RHO | 6010 | P08100 | rhodopsin | G-protein coupled receptor | 178 | 0.525 | 0.769 | 0.000131 | 0.03 | | 1 | 3 | 0 | 1999 | 2019 |
| Rheumatoid Arthritis | C0003873 | BCR | 613 | P11274 | BCR activator of RhoGEF and GTPase | Enzyme modulator | 392 | 0.431 | 0.885 | 0.98761 | 0.03 | | 1 | 3 | 0 | 2017 | 2020 |
| Rheumatoid Arthritis | C0003873 | RPS6KA3 | 6197 | P51812 | ribosomal protein S6 kinase A3 | Kinase | 315 | 0.491 | 0.846 | 0.99999 | 0.03 | | 1 | 3 | 0 | 2015 | 2019 |
| Rheumatoid Arthritis | C0003873 | BGLAP | 632 | P02818 | bone gamma-carboxyglutamate protein | | 274 | 0.462 | 0.846 | 4.62E-08 | 0.03 | | 1 | 3 | 0 | 2007 | 2019 |

| | | | | | | | | | | | | | | | | | |
|---|---|---|---|---|---|---|---|---|---|---|---|---|---|---|---|---|---|
| Rheumatoid Arthritis | C0003873 | CCL13 | 6357 | Q99616 | C-C motif chemokine ligand 13 | Signaling | 50 | 0.628 | 0.692 | 0.0409987 | 0.03 | | 1 | 3 | 0 | 2006 | 2010 |
| Rheumatoid Arthritis | C0003873 | CCL18 | 6362 | P55774 | C-C motif chemokine ligand 18 | Signaling | 200 | 0.489 | 0.846 | 0.0091774 | 0.03 | | 1 | 3 | 0 | 2007 | 2015 |
| Rheumatoid Arthritis | C0003873 | CXCL11 | 6373 | O14625 | C-X-C motif chemokine ligand 11 | Signaling | 164 | 0.519 | 0.808 | 0.31408 | 0.03 | | 1 | 3 | 0 | 2005 | 2018 |
| Rheumatoid Arthritis | C0003873 | SDC1 | 6382 | P18827 | syndecan 1 | Extracellular structure | 323 | 0.445 | 0.769 | 0.008412 | 0.03 | | 1 | 3 | 0 | 2001 | 2019 |
| Rheumatoid Arthritis | C0003873 | IFIH1 | 64135 | Q9BYX4 | interferon induced with helicase C domain 1 | | 319 | 0.471 | 0.808 | 6.98E-44 | 0.03 | | 0.333 | 3 | 1 | 2007 | 2013 |
| Rheumatoid Arthritis | C0003873 | POTEM | 641455 | A6NI47 | POTE ankyrin domain family member M | | 133 | 0.541 | 0.769 | 0.000316 | 0.03 | | 1 | 3 | 0 | 2017 | 2019 |
| Rheumatoid Ar | C0003873 | ST6GAL1 | 6480 | P15907 | ST6 beta-galactoside alpha-2,6-sialyltransferase 1 | | 101 | 0.569 | 0.731 | 0.2341 | 0.03 | | 1 | 3 | 0 | 2018 | 2019 |

| | | | | | | | | | | | | | | | | | |
|---|---|---|---|---|---|---|---|---|---|---|---|---|---|---|---|---|---|
| thritis | | | | | | | | | | | | | | | | | |
| Rheumatoid Arthritis | C0003873 | BMP2 | 650 | P12643 | bone morphogenetic protein 2 | Signaling | 428 | 0.432 | 0.846 | 0.98799 | 0.03 | | 1 | 3 | 0 | 2003 | 2019 |
| Rheumatoid Arthritis | C0003873 | RAPH1 | 65059 | Q70E73 | Ras association (RalGDS/AF-6) and pleckstrin homology domains 1 | Receptor | 80 | 0.593 | 0.654 | 0.99985 | 0.03 | | 1 | 3 | 0 | 2007 | 2017 |
| Rheumatoid Arthritis | C0003873 | SLC22A1 | 6580 | O15245 | solute carrier family 22 member 1 | Transporter | 119 | 0.556 | 0.731 | 1.46E-22 | 0.03 | | 1 | 3 | 0 | 1998 | 2017 |
| Rheumatoid Arthritis | C0003873 | SLPI | 6590 | P03973 | secretory leukocyte peptidase inhibitor | Enzyme modulator | 310 | 0.449 | 0.846 | 0.000897 | 0.03 | | 1 | 3 | 0 | 2003 | 2019 |
| Rheumatoid Arthritis | C0003873 | SST | 6750 | P61278 | somatostatin | Signaling | 535 | 0.399 | 0.885 | 0.34128 | 0.03 | | 1 | 3 | 0 | 1997 | 2018 |
| Rheumatoid Arthritis | C0003873 | STAT5B | 6777 | P51692 | signal transducer and activator of transcription 5B | Nucleic acid binding | 357 | 0.442 | 0.808 | 0.99988 | 0.03 | | 1 | 3 | 0 | 2000 | 2019 |

| | | | | | | | | | | | | | | | | | |
|---|---|---|---|---|---|---|---|---|---|---|---|---|---|---|---|---|---|
| Rheumatoid Arthritis | C0003873 | SULT2A1 | 6822 | Q06520 | sulfotransferase family 2A member 1 | | 126 | 0.545 | 0.769 | 1.25E-15 | 0.03 | | 1 | 3 | 0 | 1999 | 2018 |
| Rheumatoid Arthritis | C0003873 | ABCC8 | 6833 | Q09428 | ATP binding cassette subfamily C member 8 | Transporter | 317 | 0.474 | 0.846 | 7.96E-24 | 0.03 | | 1 | 3 | 0 | 2005 | 2019 |
| Rheumatoid Arthritis | C0003873 | TACR1 | 6869 | P25103 | tachykinin receptor 1 | G-protein coupled receptor | 217 | 0.502 | 0.885 | 0.000384 | 0.03 | | 1 | 3 | 0 | 1998 | 2019 |
| Rheumatoid Arthritis | C0003873 | TRA | 6955 | P0DSE1 | T cell receptor alpha locus | | 51 | 0.628 | 0.577 | | 0.03 | | 0.667 | 3 | 0 | 1997 | 2019 |
| Rheumatoid Arthritis | C0003873 | TFF3 | 7033 | Q07654 | trefoil factor 3 | Signaling | 163 | 0.518 | 0.885 | 0.01591 | 0.03 | | 1 | 3 | 0 | 2017 | 2019 |
| Rheumatoid Arthritis | C0003873 | TIE1 | 7075 | P35590 | tyrosine kinase with immunoglobulin like and EGF like domains 1 | Kinase | 66 | 0.61 | 0.654 | 1.24E-19 | 0.03 | | 1 | 3 | 0 | 2002 | 2011 |
| Rheuma | C0003 | TIMP2 | 70 | P16035 | TIMP metallopeptidase | Enzyme | 404 | 0.4 | 0.8 | 0.78 | 0.03 | | 1 | 3 | 0 | 1998 | 20 |

| | | | | | | | | | | | | | | | | | |
|---|---|---|---|---|---|---|---|---|---|---|---|---|---|---|---|---|---|
| toid Arthritis | 873 | | 77 | | inhibitor 2 | modulator | | 25 | 46 | 893 | | | | | | | 09 |
| Rheumatoid Arthritis | C0003873 | TIMP3 | 7078 | P35625 | TIMP metallopeptidase inhibitor 3 | Enzyme modulator | 325 | 0.445 | 0.923 | 0.630337 | 0.03 | | 1 | 3 | 0 | 2003 | 2017 |
| Rheumatoid Arthritis | C0003873 | ACTG1 | 71 | P63261 | actin gamma 1 | Cellular structure | 291 | 0.477 | 0.846 | 0.004784 | 0.03 | | 1 | 3 | 0 | 2017 | 2019 |
| Rheumatoid Arthritis | C0003873 | TPI1 | 7167 | P60174 | triosephosphate isomerase 1 | Enzyme | 184 | 0.517 | 0.846 | 0.030193 | 0.03 | | 1 | 3 | 0 | 2004 | 2019 |
| Rheumatoid Arthritis | C0003873 | CRISP2 | 7180 | P16562 | cysteine rich secretory protein 2 | Immune response | 168 | 0.512 | 0.769 | 1.44E-12 | 0.03 | | 1 | 3 | 0 | 2006 | 2017 |
| Rheumatoid Arthritis | C0003873 | ACTG2 | 72 | P63267 | actin gamma 2, smooth muscle | Cellular structure | 205 | 0.505 | 0.808 | 0.071036 | 0.03 | | 1 | 3 | 0 | 2017 | 2019 |
| Rheumatoid Arthritis | C0003873 | MUC5B | 727897 | Q9HC84 | mucin 5B, oligomeric mucus/gel-forming | | 141 | 0.532 | 0.808 | 0.99999 | 0.03 | | 1 | 3 | 0 | 2018 | 2019 |

| | | | | | | | | | | | | | | | | | |
|---|---|---|---|---|---|---|---|---|---|---|---|---|---|---|---|---|---|
| Rheumatoid Arthritis | C0003873 | TNFSF4 | 7292 | P23510 | TNF superfamily member 4 | Signaling | 129 | 0.54 | 0.808 | 0.0493378 | 0.03 | | 1 | 3 | 4 | 2015 | 2019 |
| Rheumatoid Arthritis | C0003873 | TXNRD1 | 7296 | Q16881 | thioredoxin reductase 1 | Enzyme | 136 | 0.529 | 0.692 | 1.61E-06 | 0.03 | | 0.667 | 3 | 0 | 2008 | 2019 |
| Rheumatoid Arthritis | C0003873 | VIL1 | 7429 | P09327 | villin 1 | Cellular structure | 32 | 0.691 | 0.423 | 2.64E-14 | 0.03 | | 1 | 3 | 0 | 1996 | 1997 |
| Rheumatoid Arthritis | C0003873 | VTN | 7448 | P04004 | vitronectin | | 178 | 0.509 | 0.769 | 1.5E-10 | 0.03 | | 1 | 3 | 0 | 1999 | 2014 |
| Rheumatoid Arthritis | C0003873 | YY1 | 7528 | P25490 | YY1 transcription factor | Transcription factor | 245 | 0.481 | 0.808 | 0.9938 | 0.03 | | 1 | 3 | 0 | 1990 | 2019 |
| Rheumatoid Arthritis | C0003873 | VTCN1 | 79679 | Q7Z7D3 | V-set domain containing T cell activation inhibitor 1 | Enzyme modulator | 139 | 0.529 | 0.692 | 1.37E-12 | 0.03 | | 1 | 3 | 0 | 2012 | 2017 |
| Rheumatoid Ar | C0003873 | SCD5 | 79966 | Q86SK9 | stearoyl-CoA desaturase 5 | | 40 | 0.682 | 0.654 | 0.00104 | 0.03 | | 1 | 3 | 0 | 2002 | 2014 |

| | | | | | | | | | | | | | | | | | |
|---|---|---|---|---|---|---|---|---|---|---|---|---|---|---|---|---|---|
| thritis | | | | | | | | | | | | | | | | | |
| Rheumatoid Arthritis | C0003873 | PDCD1LG2 | 80380 | Q9BQ51 | programmed cell death 1 ligand 2 | | 191 | 0.496 | 0.769 | 1.2E-05 | 0.03 | | 0.667 | 3 | 0 | 2007 | 2018 |
| Rheumatoid Arthritis | C0003873 | SLC7A5 | 8140 | Q01650 | solute carrier family 7 member 5 | Transporter | 173 | 0.513 | 0.808 | 0.235505 | 0.03 | | 1 | 3 | 0 | 2018 | 2018 |
| Rheumatoid Arthritis | C0003873 | ADAMTS12 | 81792 | P58397 | ADAM metallopeptidase with thrombospondin type 1 motif 12 | Enzyme | 26 | 0.7 | 0.462 | 2.48E-17 | 0.03 | | 1 | 3 | 3 | 2012 | 2018 |
| Rheumatoid Arthritis | C0003873 | TLR10 | 81793 | Q9BXR5 | toll like receptor 10 | | 71 | 0.592 | 0.654 | 3.48E-09 | 0.03 | | 1 | 3 | 1 | 2016 | 2019 |
| Rheumatoid Arthritis | C0003873 | CAMP | 820 | P49913 | cathelicidin antimicrobial peptide | | 262 | 0.466 | 0.846 | 0.00439 | 0.03 | | 1 | 3 | 0 | 2019 | 2019 |
| Rheumatoid Arthritis | C0003873 | H4C9 | 8294 | P62805 | H4 clustered histone 9 | | 121 | 0.541 | 0.692 | 0.015809 | 0.03 | | 1 | 3 | 0 | 2018 | 2019 |
| Rheumatoi | C0003 | H4C1 | 8359 | P62805 | H4 clustered histone 1 | | 121 | 0.5 | 0.6 | 0.000 | 0.03 | | 1 | 3 | 0 | 2018 | 2019 |

| | | | | | | | | | | | | | | | | |
|---|---|---|---|---|---|---|---|---|---|---|---|---|---|---|---|---|
| d Arthritis | 873 | | | | | | 41 | 92 | 206 | | | | | | | |
| Rheumatoid Arthritis | C0003873 | H4C4 | 8360 | P62805 | H4 clustered histone 4 | 125 | 0.537 | 0.692 | 0.001985 | 0.03 | | 1 | 3 | 0 | 2018 | 2019 |
| Rheumatoid Arthritis | C0003873 | H4C6 | 8361 | P62805 | H4 clustered histone 6 | 121 | 0.541 | 0.692 | 0.13072 | 0.03 | | 1 | 3 | 0 | 2018 | 2019 |
| Rheumatoid Arthritis | C0003873 | H4C12 | 8362 | P62805 | H4 clustered histone 12 | 121 | 0.541 | 0.692 | 0.001574 | 0.03 | | 1 | 3 | 0 | 2018 | 2019 |
| Rheumatoid Arthritis | C0003873 | H4C11 | 8363 | P62805 | H4 clustered histone 11 | 121 | 0.541 | 0.692 | 0.38683 | 0.03 | | 1 | 3 | 0 | 2018 | 2019 |
| Rheumatoid Arthritis | C0003873 | H4C3 | 8364 | P62805 | H4 clustered histone 3 | 132 | 0.534 | 0.731 | 0.00036 | 0.03 | | 1 | 3 | 0 | 2018 | 2019 |
| Rheumatoid Arthritis | C0003873 | H4C8 | 8365 | P62805 | H4 clustered histone 8 | 122 | 0.541 | 0.692 | 7.15E-06 | 0.03 | | 1 | 3 | 0 | 2018 | 2019 |
| Rheu | C00 | H4C2 | 83 | P62805 | H4 clustered histone 2 | 128 | 0. | 0. | 0.0 | 0.03 | | 1 | 3 | 0 | 2018 | 20 |

| | | | | | | | | | | | | | | | | | |
|---|---|---|---|---|---|---|---|---|---|---|---|---|---|---|---|---|---|
| matoid Arthritis | 03873 | | 66 | | | | | 537 | 731 | 02915 | | | | | | | 19 |
| Rheumatoid Arthritis | C0003873 | H4C5 | 8367 | P62805 | H4 clustered histone 5 | | 121 | 0.541 | 0.692 | 0.013453 | 0.03 | | 1 | 3 | 0 | 2018 | 2019 |
| Rheumatoid Arthritis | C0003873 | H4C13 | 8368 | P62805 | H4 clustered histone 13 | | 121 | 0.541 | 0.692 | 0.00035 | 0.03 | | 1 | 3 | 0 | 2018 | 2019 |
| Rheumatoid Arthritis | C0003873 | H4C14 | 8370 | P62805 | H4 clustered histone 14 | | 121 | 0.541 | 0.692 | | 0.03 | | 1 | 3 | 0 | 2018 | 2019 |
| Rheumatoid Arthritis | C0003873 | PRAM1 | 84106 | Q96QH2 | PML-RARA regulated adaptor molecule 1 | | 27 | 0.7 | 0.462 | 0.0003 | 0.03 | | 1 | 3 | 0 | 1995 | 2005 |
| Rheumatoid Arthritis | C0003873 | SPZ1 | 84654 | Q9BXG8 | spermatogenic leucine zipper 1 | | 173 | 0.507 | 0.769 | | 0.03 | | 1 | 3 | 0 | 2006 | 2017 |
| Rheumatoid Arthritis | C0003873 | FCRLA | 84824 | Q7L513 | Fc receptor like A | Cell adhesion | 15 | 0.805 | 0.346 | 2.44E-09 | 0.03 | | 1 | 3 | 0 | 2003 | 2017 |

| | | | | | | | | | | | | | | | | | |
|---|---|---|---|---|---|---|---|---|---|---|---|---|---|---|---|---|---|
| Rheumatoid Arthritis | C0003873 | ORAI1 | 84876 | Q96D31 | ORAI calcium release-activated calcium modulator 1 | Ion channel | 195 | 0.52 | 0.808 | 1.91E-05 | 0.03 | | 1 | 3 | 1 | 2014 | 2019 |
| Rheumatoid Arthritis | C0003873 | PIK3R3 | 8503 | Q92569 | phosphoinositide-3-kinase regulatory subunit 3 | Enzyme modulator | 101 | 0.556 | 0.692 | 3.36E-10 | 0.03 | | 1 | 3 | 0 | 1992 | 2002 |
| Rheumatoid Arthritis | C0003873 | ABCC11 | 85320 | Q96J66 | ATP binding cassette subfamily C member 11 | Transporter | 91 | 0.565 | 0.769 | 3.99E-46 | 0.03 | | 1 | 3 | 0 | 2005 | 2019 |
| Rheumatoid Arthritis | C0003873 | BECN1 | 8678 | Q14457 | beclin 1 | Enzyme modulator | 373 | 0.432 | 0.846 | 0.93717 | 0.03 | | 1 | 3 | 0 | 2013 | 2019 |
| Rheumatoid Arthritis | C0003873 | FADD | 8772 | Q13158 | Fas associated via death domain | | 137 | 0.537 | 0.846 | 0.52595 | 0.03 | | 1 | 3 | 0 | 2000 | 2006 |
| Rheumatoid Arthritis | C0003873 | CD84 | 8832 | Q9UIB8 | CD84 molecule | | 22 | 0.76 | 0.308 | 6.91E-09 | 0.03 | | 1 | 3 | 0 | 2013 | 2019 |
| Rheumatoid Ar | C0003873 | HDAC3 | 8841 | O15379 | histone deacetylase 3 | Epigenetic reg | 179 | 0.511 | 0.731 | 0.56737 | 0.03 | | 1 | 3 | 0 | 2012 | 2018 |

| | | | | | | | | | | | | | | | | | |
|---|---|---|---|---|---|---|---|---|---|---|---|---|---|---|---|---|---|
| thritis | | | | | | ulator | | | | | | | | | | | |
| Rheumatoid Arthritis | C0003873 | CCK | 885 | P06307 | cholecystokinin | | 326 | 0.453 | 0.846 | 3.3E-05 | 0.03 | | 1 | 3 | 0 | 2015 | 2019 |
| Rheumatoid Arthritis | C0003873 | APLN | 8862 | Q9ULZ1 | apelin | | 226 | 0.497 | 0.769 | 0.458897 | 0.03 | | 1 | 3 | 0 | 2017 | 2019 |
| Rheumatoid Arthritis | C0003873 | PER2 | 8864 | O15055 | period circadian regulator 2 | Transcription factor | 238 | 0.488 | 0.846 | 0.71148 | 0.03 | | 1 | 3 | 3 | 2013 | 2017 |
| Rheumatoid Arthritis | C0003873 | CCRL2 | 9034 | O00421 | C-C motif chemokine receptor like 2 | G-protein coupled receptor | 59 | 0.621 | 0.654 | 0.368861 | 0.03 | | 1 | 3 | 0 | 2004 | 2010 |
| Rheumatoid Arthritis | C0003873 | FCGR2C | 9103 | P31995 | Fc fragment of IgG receptor IIc (gene/pseudogene) | Cell adhesion | 71 | 0.61 | 0.654 | | 0.03 | | 1 | 3 | 0 | 2004 | 2017 |
| Rheumatoid Arthritis | C0003873 | LPAR2 | 9170 | Q9HBW0 | lysophosphatidic acid receptor 2 | G-protein coupled receptor | 285 | 0.463 | 0.846 | 0.0366658 | 0.03 | | 1 | 3 | 0 | 1999 | 2012 |

| | | | | | | | | | | | | | | | | | |
|---|---|---|---|---|---|---|---|---|---|---|---|---|---|---|---|---|---|
| Rheumatoid Arthritis | C0003873 | XPR1 | 9213 | Q9UBH6 | xenotropic and polytropic retrovirus receptor 1 | Transporter | 217 | 0.488 | 0.731 | 0.99999 | 0.03 | | 1 | 3 | 0 | 2012 | 2018 |
| Rheumatoid Arthritis | C0003873 | CD6 | 923 | P30203 | CD6 molecule | Enzyme | 216 | 0.489 | 0.808 | 9.27E-05 | 0.03 | | 1 | 3 | 0 | 2009 | 2016 |
| Rheumatoid Arthritis | C0003873 | ASIC3 | 9311 | Q9UHC3 | acid sensing ion channel subunit 3 | Ion channel | 51 | 0.663 | 0.538 | 3.41E-07 | 0.03 | | 1 | 3 | 0 | 2014 | 2018 |
| Rheumatoid Arthritis | C0003873 | CD163 | 9332 | Q86VB7 | CD163 molecule | Enzyme | 369 | 0.427 | 0.846 | 0.000834 | 0.03 | | 1 | 3 | 0 | 2012 | 2019 |
| Rheumatoid Arthritis | C0003873 | KL | 9365 | Q9UEF7 | klotho | Enzyme | 332 | 0.448 | 0.769 | 0.000224 | 0.03 | | 1 | 3 | 0 | 2007 | 2020 |
| Rheumatoid Arthritis | C0003873 | FHL5 | 9457 | Q5TD97 | four and a half LIM domains 5 | Transcription factor | 153 | 0.53 | 0.769 | 4.85E-10 | 0.03 | | 1 | 3 | 0 | 2017 | 2019 |
| Rheumatoid Ar | C0003873 | ENTPD1 | 953 | P49961 | ectonucleoside triphosphate diphosp | Enzyme | 206 | 0.499 | 0.769 | 0.0080 | 0.03 | | 1 | 3 | 0 | 2018 | 2019 |

| | | | | | | | | | | | | | | | | | |
|---|---|---|---|---|---|---|---|---|---|---|---|---|---|---|---|---|---|
| thritis | | | | | hohydrolase 1 | | | | | 72 | | | | | | | |
| Rheumatoid Arthritis | C0003873 | BMS1 | 9790 | Q14692 | BMS1 ribosome biogenesis factor | | 192 | 0.499 | 0.846 | 0.000175 | 0.03 | | 0.667 | 3 | 0 | 2008 | 2019 |
| Rheumatoid Arthritis | C0003873 | CDC42 | 998 | P60953 | cell division cycle 42 | Enzyme modulator | 327 | 0.458 | 0.846 | 0.78728 | 0.03 | | 1 | 3 | 0 | 1989 | 2016 |
| Rheumatoid Arthritis | C0003873 | SCO2 | 9997 | O43819 | synthesis of cytochrome C oxidase 2 | Enzyme | 294 | 0.479 | 0.846 | 7.55E-07 | 0.03 | | 1 | 3 | 0 | 2014 | 2019 |
| Rheumatoid Arthritis | C0003873 | MIR760 | 1E+08 | | microRNA 760 | | 37 | 0.674 | 0.385 | | 0.02 | | 1 | 2 | 0 | 2017 | 2019 |
| Rheumatoid Arthritis | C0003873 | SH2B3 | 10019 | Q9UQQ2 | SH2B adaptor protein 3 | | 212 | 0.527 | 0.692 | 0.000103 | 0.02 | | 1 | 2 | 0 | 2009 | 2017 |
| Rheumatoid Arthritis | C0003873 | LINC01672 | 1.01E+08 | | long intergenic non-protein coding RNA 1672 | | 268 | 0.462 | 0.846 | | 0.02 | | 1 | 2 | 0 | 2017 | 2018 |
| Rheumatoi | C0003 | RPL17-C1 | 1.01E | | RPL17-C18orf32 readthrough | | 93 | 0.5 | 0.6 | 0.575 | 0.02 | | 1 | 2 | 0 | 2004 | 2017 |

| | | | | | | | | | | | | | | | | | |
|---|---|---|---|---|---|---|---|---|---|---|---|---|---|---|---|---|---|
| d Arthritis | 873 | 8orf32 | +08 | | | | | 72 | 15 | 02 | | | | | | | |
| Rheumatoid Arthritis | C0003873 | LINC-ROR | 1.01E+08 | | long intergenic non-protein coding RNA, regulator of reprogramming | | 108 | 0.548 | 0.769 | | 0.02 | | 0.5 | 2 | 0 | 2001 | 2012 |
| Rheumatoid Arthritis | C0003873 | ADAM8 | 101 | P78325 | ADAM metallopeptidase domain 8 | Enzyme | 81 | 0.59 | 0.731 | 2.2E-14 | 0.02 | | 1 | 2 | 0 | 2009 | 2014 |
| Rheumatoid Arthritis | C0003873 | CDK2 | 1017 | P24941 | cyclin dependent kinase 2 | Kinase | 270 | 0.456 | 0.692 | 0.61054 | 0.02 | | 1 | 2 | 0 | 1997 | 2011 |
| Rheumatoid Arthritis | C0003873 | CALCRL | 10203 | Q16602 | calcitonin receptor like receptor | G-protein coupled receptor | 47 | 0.653 | 0.692 | 0.9853 | 0.02 | | 0.5 | 2 | 0 | 2006 | 2009 |
| Rheumatoid Arthritis | C0003873 | PRG4 | 10216 | Q92954 | proteoglycan 4 | | 73 | 0.601 | 0.615 | 1.7E-08 | 0.02 | | 1 | 2 | 0 | 2015 | 2017 |
| Rheumatoid Arthritis | C0003873 | SPRY1 | 10252 | O43609 | sprouty RTK signaling antagonist 1 | Signaling | 63 | 0.603 | 0.577 | 0.03004 | 0.02 | | 1 | 2 | 0 | 2017 | 2019 |

| | | | | | | | | | | | | | | | | | |
|---|---|---|---|---|---|---|---|---|---|---|---|---|---|---|---|---|---|
| Rheumatoid Arthritis | C0003873 | RAMP2 | 10266 | O60895 | receptor activity modifying protein 2 | Receptor | 60 | 0.626 | 0.654 | 0.051127 | 0.02 | | 0.5 | 2 | 0 | 2006 | 2009 |
| Rheumatoid Arthritis | C0003873 | CCL26 | 10344 | Q9Y258 | C-C motif chemokine ligand 26 | Signaling | 60 | 0.617 | 0.654 | 0.010114 | 0.02 | | 0.5 | 2 | 0 | 2005 | 2009 |
| Rheumatoid Arthritis | C0003873 | WARS2 | 10352 | Q9UGM6 | tryptophanyl tRNA synthetase 2, mitochondrial | | 110 | 0.617 | 0.577 | 0.050885 | 0.02 | | 1 | 2 | 0 | 2015 | 2019 |
| Rheumatoid Arthritis | C0003873 | LINC02210-CRHR1 | 1.05E+08 | P34998 | LINC02210-CRHR1 readthrough | | 123 | 0.57 | 0.692 | | 0.02 | | 1 | 2 | 0 | 2001 | 2011 |
| Rheumatoid Arthritis | C0003873 | SEMA4D | 10507 | Q92854 | semaphorin 4D | Signaling | 109 | 0.564 | 0.769 | 0.97472 | 0.02 | | 1 | 2 | 0 | 2015 | 2018 |
| Rheumatoid Arthritis | C0003873 | CEBPD | 1052 | P49716 | CCAAT enhancer binding protein delta | Transcription factor | 97 | 0.57 | 0.769 | 0.61219 | 0.02 | | 1 | 2 | 0 | 2012 | 2017 |
| Rheumatoid Ar | C0003873 | KAT5 | 10524 | Q92993 | lysine acetyltransferase 5 | | 138 | 0.534 | 0.769 | 0.0882 | 0.02 | | 1 | 2 | 0 | 2010 | 2019 |

| | | | | | | | | | | | | | | | | | |
|---|---|---|---|---|---|---|---|---|---|---|---|---|---|---|---|---|---|
| thritis | | | | | | | | | | 34 | | | | | | | |
| Rheumatoid Arthritis | C0003873 | IVNS1ABP | 10625 | Q9Y6Y0 | influenza virus NS1A binding protein | | 67 | 0.608 | 0.769 | 0.99442 | 0.02 | | 1 | 2 | 0 | 2003 | 2004 |
| Rheumatoid Arthritis | C0003873 | EBP | 10682 | Q15125 | EBP cholestenol delta-isomerase | Enzyme | 243 | 0.494 | 0.846 | 0.93645 | 0.02 | | 1 | 2 | 0 | 2000 | 2009 |
| Rheumatoid Arthritis | C0003873 | CTSC | 1075 | P53634 | cathepsin C | Enzyme | 139 | 0.541 | 0.731 | 1.15E-07 | 0.02 | | 1 | 2 | 0 | 2017 | 2017 |
| Rheumatoid Arthritis | C0003873 | CFTR | 1080 | P13569 | CF transmembrane conductance regulator | Ion channel | 476 | 0.424 | 0.885 | 2.17E-58 | 0.02 | | 1 | 2 | 0 | 1999 | 2011 |
| Rheumatoid Arthritis | C0003873 | CCR9 | 10803 | P51686 | C-C motif chemokine receptor 9 | G-protein coupled receptor | 90 | 0.573 | 0.692 | 0.004199 | 0.02 | | 1 | 2 | 0 | 2010 | 2014 |
| Rheumatoid Arthritis | C0003873 | CHAT | 1103 | P28329 | choline O-acetyltransferase | Enzyme | 230 | 0.52 | 0.808 | 5.63E-11 | 0.02 | | 1 | 2 | 0 | 2008 | 2012 |

| | | | | | | | | | | | | | | | | | |
|---|---|---|---|---|---|---|---|---|---|---|---|---|---|---|---|---|---|
| Rheumatoid Arthritis | C0003873 | PSIP1 | 11168 | O75475 | PC4 and SFRS1 interacting protein 1 | Signaling | 210 | 0.49 | 0.846 | 0.99668 | 0.02 | | 1 | 2 | 0 | 1992 | 2019 |
| Rheumatoid Arthritis | C0003873 | IRAK3 | 11213 | Q9Y616 | interleukin 1 receptor associated kinase 3 | Kinase | 59 | 0.615 | 0.615 | 5.31E-21 | 0.02 | | 1 | 2 | 0 | 2017 | 2018 |
| Rheumatoid Arthritis | C0003873 | KLF12 | 11278 | Q9Y4X4 | Kruppel like factor 12 | Nucleic acid binding | 58 | 0.647 | 0.654 | 0.98067 | 0.02 | | 0.5 | 2 | 2 | 2008 | 2011 |
| Rheumatoid Arthritis | C0003873 | SAAL1 | 113174 | Q96ER3 | serum amyloid A like 1 | | 4 | 0.89 | 0.115 | 4.32E-09 | 0.02 | | 1 | 2 | 0 | 2011 | 2018 |
| Rheumatoid Arthritis | C0003873 | TIRAP | 114609 | P58753 | TIR domain containing adaptor protein | | 74 | 0.604 | 0.769 | 0.000761 | 0.02 | | 1 | 2 | 1 | 2008 | 2011 |
| Rheumatoid Arthritis | C0003873 | MIR155HG | 114614 | | MIR155 host gene | | 56 | 0.617 | 0.615 | | 0.02 | | 1 | 2 | 1 | 2008 | 2017 |
| Rheumatoid Ar | C0003873 | CHUK | 1147 | O15111 | component of inhibitor of nuclear factor | Kinase | 125 | 0.556 | 0.731 | 0.99403 | 0.02 | | 1 | 2 | 0 | 2003 | 2017 |

| | | | | | | | | | | | | | | | | | |
|---|---|---|---|---|---|---|---|---|---|---|---|---|---|---|---|---|---|
| thritis | | | | | kappa B kinase complex | | | | | | | | | | | | |
| Rheumatoid Arthritis | C0003873 | C1QTNF3 | 114899 | Q9BXJ4 | C1q and TNF related 3 | | 26 | 0.695 | 0.577 | 1.81E-07 | 0.02 | | 1 | 2 | 0 | 2014 | 2019 |
| Rheumatoid Arthritis | C0003873 | C1QTNF6 | 114904 | Q9BXI9 | C1q and TNF related 6 | | 33 | 0.682 | 0.577 | 9.01E-06 | 0.02 | | 1 | 2 | 0 | 2010 | 2015 |
| Rheumatoid Arthritis | C0003873 | TADA1 | 117143 | Q96BN2 | transcriptional adaptor 1 | | 23 | 0.705 | 0.654 | 0.090708 | 0.02 | | 1 | 2 | 0 | 2003 | 2010 |
| Rheumatoid Arthritis | C0003873 | LTB4R | 1241 | Q15722 | leukotriene B4 receptor | G-protein coupled receptor | 107 | 0.576 | 0.692 | 0.11994 | 0.02 | | 1 | 2 | 0 | 2003 | 2017 |
| Rheumatoid Arthritis | C0003873 | CNR1 | 1268 | P21554 | cannabinoid receptor 1 | G-protein coupled receptor | 393 | 0.446 | 0.808 | 0.50652 | 0.02 | | 1 | 2 | 0 | 2009 | 2019 |
| Rheumatoid Ar | C0003873 | PPARGC1B | 133522 | Q86YN6 | PPARG coactivator 1 beta | Transcription factor | 102 | 0.569 | 0.731 | 0.50321 | 0.02 | | 1 | 2 | 0 | 2014 | 2019 |

| | | | | | | | | | | | | | | | | | |
|---|---|---|---|---|---|---|---|---|---|---|---|---|---|---|---|---|---|
| thritis | | | | | | | | | | | | | | | | | |
| Rheumatoid Arthritis | C0003873 | COX8A | 1351 | P10176 | cytochrome c oxidase subunit 8A | Enzyme | 526 | 0.4 | 0.846 | 0.116066 | 0.02 | | 1 | 2 | 0 | 2000 | 2018 |
| Rheumatoid Arthritis | C0003873 | CPB1 | 1360 | P15086 | carboxypeptidase B1 | Enzyme | 57 | 0.626 | 0.654 | 2.61E-22 | 0.02 | | 1 | 2 | 0 | 2011 | 2014 |
| Rheumatoid Arthritis | C0003873 | CR1 | 1378 | P17927 | complement C3b/C4b receptor 1 (Knops blood group) | | 129 | 0.533 | 0.808 | 1.65E-35 | 0.02 | | 1 | 2 | 0 | 2013 | 2014 |
| Rheumatoid Arthritis | C0003873 | ATF2 | 1386 | P15336 | activating transcription factor 2 | Transcription factor | 122 | 0.551 | 0.808 | 0.99131 | 0.02 | | 1 | 2 | 0 | 2007 | 2019 |
| Rheumatoid Arthritis | C0003873 | CREBBP | 1387 | Q92793 | CREB binding protein | | 438 | 0.428 | 0.808 | 1 | 0.02 | | 1 | 2 | 0 | 2003 | 2018 |
| Rheumatoid Arthritis | C0003873 | CRHR1 | 1394 | P34998 | corticotropin releasing hormone receptor 1 | | 183 | 0.517 | 0.769 | 3.79E-05 | 0.02 | | 1 | 2 | 0 | 2001 | 2011 |
| Rheumatoi | C0003 | CSF3 | 1440 | P09919 | colony stimulating factor 3 | | 687 | 0.3 | 0.8 | 0.380 | 0.02 | | 1 | 2 | 0 | 2017 | 2017 |

| | | | | | | | | | | | | | | | | | |
|---|---|---|---|---|---|---|---|---|---|---|---|---|---|---|---|---|---|
| d Arthritis | 873 | | | | | | | 77 | 85 | 46 | | | | | | | |
| Rheumatoid Arthritis | C0003873 | CSK | 1445 | P41240 | C-terminal Src kinase | Kinase | 91 | 0.606 | 0.692 | 0.99977 | 0.02 | | 1 | 2 | 2 | 1999 | 2017 |
| Rheumatoid Arthritis | C0003873 | CST3 | 1471 | P01034 | cystatin C | | 370 | 0.436 | 0.808 | 0.0039001 | 0.02 | | 1 | 2 | 0 | 2007 | 2018 |
| Rheumatoid Arthritis | C0003873 | ADRA1A | 148 | P35348 | adrenoceptor alpha 1A | G-protein coupled receptor | 294 | 0.462 | 0.846 | 3.37E-07 | 0.02 | | 1 | 2 | 0 | 1999 | 2004 |
| Rheumatoid Arthritis | C0003873 | CTSB | 1508 | P07858 | cathepsin B | Enzyme | 304 | 0.457 | 0.846 | 5.88E-11 | 0.02 | | 1 | 2 | 0 | 1991 | 2014 |
| Rheumatoid Arthritis | C0003873 | ADRA2B | 151 | P18089 | adrenoceptor alpha 2B | G-protein coupled receptor | 296 | 0.465 | 0.846 | 4.45E-06 | 0.02 | | 1 | 2 | 0 | 1999 | 2004 |
| Rheumatoid Ar | C0003873 | BTLA | 151888 | Q7Z6A9 | B and T lymphocyte associated | | 74 | 0.593 | 0.577 | 0.0209 | 0.02 | | 1 | 2 | 0 | 2006 | 2017 |

| | | | | | | | | | | | | | | | | |
|---|---|---|---|---|---|---|---|---|---|---|---|---|---|---|---|---|
| thritis | | | | | | | | | | 87 | | | | | | |
| Rheumatoid Arthritis | C0003873 | CYP2D6 | 1565 | P10635 | cytochrome P450 family 2 subfamily D member 6 | | 432 | 0.437 | 0.885 | 1.08E-25 | 0.02 | | 0.5 | 2 | 0 | 1994 | 1996 |
| Rheumatoid Arthritis | C0003873 | CYP3A5 | 1577 | P20815 | cytochrome P450 family 3 subfamily A member 5 | Enzyme | 179 | 0.511 | 0.846 | 6.29E-17 | 0.02 | | 1 | 2 | 0 | 2015 | 2017 |
| Rheumatoid Arthritis | C0003873 | CYP17A1 | 1586 | P05093 | cytochrome P450 family 17 subfamily A member 1 | | 326 | 0.462 | 0.769 | 0.000263 | 0.02 | | 1 | 2 | 0 | 2000 | 2005 |
| Rheumatoid Arthritis | C0003873 | CYP19A1 | 1588 | P11511 | cytochrome P450 family 19 subfamily A member 1 | Enzyme | 519 | 0.41 | 0.885 | 1.7E-05 | 0.02 | | 1 | 2 | 0 | 1999 | 2009 |
| Rheumatoid Arthritis | C0003873 | CYP26A1 | 1592 | O43174 | cytochrome P450 family 26 subfamily A member 1 | Enzyme | 59 | 0.619 | 0.577 | 8.43E-11 | 0.02 | | 1 | 2 | 0 | 2002 | 2019 |
| Rheumatoid Arthritis | C0003873 | DNAJB1P1 | 171221 | | DnaJ heat shock protein family (Hsp40) member B1 pseudogene 1 | | 51 | 0.65 | 0.654 | | 0.02 | | 1 | 2 | 0 | 2019 | 2019 |
| Rheumatoi | C0003 | NLRP6 | 1713 | P59044 | NLR family pyrin domain containing 6 | | 58 | 0.6 | 0.7 | 0.000 | 0.02 | | 1 | 2 | 0 | 2017 | 2017 |

| | | | | | | | | | | | | | | | | | |
|---|---|---|---|---|---|---|---|---|---|---|---|---|---|---|---|---|---|
| d Arthritis | 873 | | 89 | | | | | 28 | 69 | 274 | | | | | | | |
| Rheumatoid Arthritis | C0003873 | DHCR7 | 1717 | Q9UBM7 | 7-dehydrocholesterol reductase | Enzyme | 266 | 0.5 | 0.846 | 3.95E-13 | 0.02 | | 1 | 2 | 0 | 2017 | 2019 |
| Rheumatoid Arthritis | C0003873 | AFP | 174 | P02771 | alpha fetoprotein | Transporter | 392 | 0.429 | 0.885 | 1.14E-11 | 0.02 | | 1 | 2 | 0 | 2017 | 2019 |
| Rheumatoid Arthritis | C0003873 | DNASE1 | 1773 | P24855 | deoxyribonuclease 1 | | 180 | 0.505 | 0.846 | 6.98E-21 | 0.02 | | 1 | 2 | 0 | 2009 | 2014 |
| Rheumatoid Arthritis | C0003873 | DNTT | 1791 | P04053 | DNA nucleotidylexotransferase | Enzyme | 70 | 0.606 | 0.692 | 2.95E-10 | 0.02 | | 0.5 | 2 | 0 | 1999 | 2002 |
| Rheumatoid Arthritis | C0003873 | DR1 | 1810 | Q01658 | down-regulator of transcription 1 | | 10 | 0.839 | 0.231 | 0.92117 | 0.02 | | 1 | 2 | 0 | 1998 | 2004 |
| Rheumatoid Arthritis | C0003873 | DRD2 | 1813 | P14416 | dopamine receptor D2 | G-protein coupled receptor | 437 | 0.436 | 0.846 | 0.74709 | 0.02 | | 1 | 2 | 0 | 2011 | 2015 |

| | | | | | | | | | | | | | | | | | |
|---|---|---|---|---|---|---|---|---|---|---|---|---|---|---|---|---|---|
| Rheumatoid Arthritis | C0003873 | JAG1 | 182 | P78504 | jagged canonical Notch ligand 1 | | 420 | 0.429 | 0.808 | 1 | 0.02 | | 1 | 2 | 0 | 2005 | 2009 |
| Rheumatoid Arthritis | C0003873 | TSC22D3 | 1831 | Q99576 | TSC22 domain family member 3 | | 64 | 0.619 | 0.654 | 0.4339 | 0.02 | | 1 | 2 | 0 | 2010 | 2014 |
| Rheumatoid Arthritis | C0003873 | DUSP1 | 1843 | P28562 | dual specificity phosphatase 1 | | 214 | 0.491 | 0.808 | 0.010151 | 0.02 | | 1 | 2 | 0 | 2010 | 2019 |
| Rheumatoid Arthritis | C0003873 | EDN1 | 1906 | P05305 | endothelin 1 | Signaling | 679 | 0.38 | 0.846 | 0.45504 | 0.02 | | 1 | 2 | 0 | 1999 | 2008 |
| Rheumatoid Arthritis | C0003873 | EFNB1 | 1947 | P98172 | ephrin B1 | Signaling | 134 | 0.561 | 0.769 | 0.93251 | 0.02 | | 1 | 2 | 0 | 2008 | 2015 |
| Rheumatoid Arthritis | C0003873 | EIF4EBP1 | 1978 | Q13541 | eukaryotic translation initiation factor 4E binding protein 1 | Nucleic acid binding | 201 | 0.494 | 0.885 | 0.20762 | 0.02 | | 1 | 2 | 0 | 2018 | 2019 |
| Rheumatoid | C0003873 | ELAVL1 | 1994 | Q15717 | ELAV like RNA binding protein 1 | | 103 | 0.56 | 0.731 | 0.988 | 0.02 | | 1 | 2 | 0 | 2006 | 2007 |

| | | | | | | | | | | | | | | | | | |
|---|---|---|---|---|---|---|---|---|---|---|---|---|---|---|---|---|---|
| Arthritis | | | | | | | | | | 49 | | | | | | | |
| Rheumatoid Arthritis | C0003873 | ELF3 | 1999 | P78545 | E74 like ETS transcription factor 3 | Transcription factor | 112 | 0.551 | 0.808 | 0.98323 | 0.02 | | 1 | 2 | 0 | 2003 | 2004 |
| Rheumatoid Arthritis | C0003873 | ELK3 | 2004 | P41970 | ETS transcription factor ELK3 | Transcription factor | 220 | 0.49 | 0.846 | 0.083508 | 0.02 | | 1 | 2 | 0 | 2019 | 2020 |
| Rheumatoid Arthritis | C0003873 | EPAS1 | 2034 | Q99814 | endothelial PAS domain protein 1 | Transcription factor | 293 | 0.454 | 0.731 | 0.5961 | 0.02 | | 1 | 2 | 0 | 2003 | 2017 |
| Rheumatoid Arthritis | C0003873 | FBL | 2091 | P22087 | fibrillarin | Enzyme | 94 | 0.572 | 0.654 | 0.99319 | 0.02 | | 1 | 2 | 0 | 2019 | 2020 |
| Rheumatoid Arthritis | C0003873 | ETS2 | 2114 | P15036 | ETS proto-oncogene 2, transcription factor | Transcription factor | 115 | 0.548 | 0.808 | 0.99888 | 0.02 | | 1 | 2 | 0 | 1996 | 2001 |
| Rheumatoid Arthritis | C0003873 | F2R | 2149 | P25116 | coagulation factor II thrombin receptor | G-protein coupled receptor | 347 | 0.444 | 0.846 | 0.008468 | 0.02 | | 1 | 2 | 0 | 1995 | 2012 |

| | | | | | | | | | | | | | | | | | |
|---|---|---|---|---|---|---|---|---|---|---|---|---|---|---|---|---|---|
| Rheumatoid Arthritis | C0003873 | ALDH1A1 | 216 | P00352 | aldehyde dehydrogenase 1 family member A1 | Enzyme | 270 | 0.46 | 0.846 | 0.95448 | 0.02 | | 1 | 2 | 0 | 2015 | 2019 |
| Rheumatoid Arthritis | C0003873 | PTK2B | 2185 | Q14289 | protein tyrosine kinase 2 beta | Kinase | 319 | 0.448 | 0.808 | 0.93536 | 0.02 | | 1 | 2 | 0 | 2007 | 2007 |
| Rheumatoid Arthritis | C0003873 | FCER2 | 2208 | P06734 | Fc fragment of IgE receptor II | Receptor | 104 | 0.56 | 0.654 | 7.61E-12 | 0.02 | | 0.5 | 2 | 0 | 1994 | 2011 |
| Rheumatoid Arthritis | C0003873 | FCN2 | 2220 | Q15485 | ficolin 2 | Signaling | 159 | 0.517 | 0.846 | 3.56E-11 | 0.02 | | 0.5 | 2 | 1 | 2015 | 2016 |
| Rheumatoid Arthritis | C0003873 | FGB | 2244 | P02675 | fibrinogen beta chain | Signaling | 95 | 0.584 | 0.692 | 0.56616 | 0.02 | | 1 | 2 | 1 | 2012 | 2017 |
| Rheumatoid Arthritis | C0003873 | FGF1 | 2246 | P05230 | fibroblast growth factor 1 | Signaling | 184 | 0.507 | 0.846 | 0.44406 | 0.02 | | 1 | 2 | 0 | 1990 | 2001 |
| Rheumatoid Ar | C0003873 | FGF13 | 2258 | Q92913 | fibroblast growth factor 13 | Signaling | 167 | 0.514 | 0.808 | 0.89489 | 0.02 | | 1 | 2 | 0 | 2002 | 2003 |

| | | | | | | | | | | | | | | | | | |
|---|---|---|---|---|---|---|---|---|---|---|---|---|---|---|---|---|---|
| thritis | | | | | | | | | | | | | | | | | |
| Rheumatoid Arthritis | C0003873 | CASC3 | 22794 | O15234 | CASC3 exon junction complex subunit | | 10 | 0.792 | 0.231 | 0.61338 | 0.02 | | 1 | 2 | 0 | 2006 | 2008 |
| Rheumatoid Arthritis | C0003873 | COG2 | 22796 | Q14746 | component of oligomeric golgi complex 2 | | 30 | 0.729 | 0.423 | 9.37E-06 | 0.02 | | 1 | 2 | 0 | 2017 | 2018 |
| Rheumatoid Arthritis | C0003873 | FOXO1 | 2308 | Q12778 | forkhead box O1 | | 380 | 0.433 | 0.846 | 0.99725 | 0.02 | | 1 | 2 | 0 | 2008 | 2015 |
| Rheumatoid Arthritis | C0003873 | KDM6B | 23135 | O15054 | lysine demethylase 6B | Epigenetic regulator | 173 | 0.514 | 0.808 | 1 | 0.02 | | 1 | 2 | 0 | 2018 | 2019 |
| Rheumatoid Arthritis | C0003873 | FLT4 | 2324 | P35916 | fms related receptor tyrosine kinase 4 | Kinase | 306 | 0.457 | 0.808 | 1 | 0.02 | | 1 | 2 | 0 | 2002 | 2018 |
| Rheumatoid Arthritis | C0003873 | BRD4 | 23476 | O60885 | bromodomain containing 4 | Epigenetic regulator | 335 | 0.446 | 0.885 | 1 | 0.02 | | 1 | 2 | 0 | 2015 | 2016 |
| Rheumatoi | C0003 | ALPP | 250 | P05187 | alkaline phosphatase, | Enzyme | 209 | 0.4 | 0.8 | 1.81E | 0.02 | | 1 | 2 | 0 | 2003 | 2019 |

| | | | | | | | | | | | | | | | | | |
|---|---|---|---|---|---|---|---|---|---|---|---|---|---|---|---|---|---|
| d Arthritis | 873 | | | | placental | | | 87 | 08 | -13 | | | | | | | |
| Rheumatoid Arthritis | C0003873 | TMEM158 | 25907 | Q8WZ71 | transmembrane protein 158 (gene/pseudogene) | | 22 | 0.736 | 0.385 | 0.14862 | 0.02 | | 1 | 2 | 0 | 1994 | 1996 |
| Rheumatoid Arthritis | C0003873 | ASPM | 259266 | Q8IZT6 | abnormal spindle microtubule assembly | | 159 | 0.526 | 0.769 | 3.35E-49 | 0.02 | | 1 | 2 | 0 | 2004 | 2018 |
| Rheumatoid Arthritis | C0003873 | GAPDH | 2597 | P04406 | glyceraldehyde-3-phosphate dehydrogenase | Enzyme | 305 | 0.447 | 0.808 | 0.11388 | 0.02 | | 1 | 2 | 0 | 2007 | 2019 |
| Rheumatoid Arthritis | C0003873 | ATRNL1 | 26033 | Q5VV63 | attractin like 1 | Enzyme modulator | 117 | 0.546 | 0.769 | 0.99915 | 0.02 | | 1 | 2 | 0 | 2003 | 2019 |
| Rheumatoid Arthritis | C0003873 | GAS6 | 2621 | Q14393 | growth arrest specific 6 | Extracellular structure | 190 | 0.5 | 0.769 | 0.034477 | 0.02 | | 1 | 2 | 0 | 1999 | 2017 |
| Rheumatoid Arthritis | C0003873 | HSPB8 | 26353 | Q9UJY1 | heat shock protein family B (small) member 8 | | 122 | 0.57 | 0.577 | 0.043128 | 0.02 | | 1 | 2 | 0 | 2006 | 2008 |

| | | | | | | | | | | | | | | | | | |
|---|---|---|---|---|---|---|---|---|---|---|---|---|---|---|---|---|---|
| Rheumatoid Arthritis | C0003873 | GH1 | 2688 | P01241 | growth hormone 1 | Signaling | 686 | 0.373 | 0.923 | 0.030057 | 0.02 | | 1 | 2 | 0 | 2006 | 2010 |
| Rheumatoid Arthritis | C0003873 | CBLIF | 2694 | P27352 | cobalamin binding intrinsic factor | | 185 | 0.507 | 0.808 | 0.000249 | 0.02 | | 1 | 2 | 0 | 2017 | 2019 |
| Rheumatoid Arthritis | C0003873 | LAMP3 | 27074 | Q9UQV4 | lysosomal associated membrane protein 3 | Transporter | 104 | 0.56 | 0.692 | 0.000267 | 0.02 | | 0.5 | 2 | 0 | 2003 | 2008 |
| Rheumatoid Arthritis | C0003873 | HPLH1 | 27259 | | hemophagocytic lymphohistiocytosis 1 | | 22 | 0.695 | 0.538 | | 0.02 | | 1 | 2 | 0 | 2000 | 2003 |
| Rheumatoid Arthritis | C0003873 | PDLIM3 | 27295 | Q53GG5 | PDZ and LIM domain 3 | Cellular structure | 118 | 0.547 | 0.769 | 3.03E-07 | 0.02 | | 1 | 2 | 0 | 2003 | 2019 |
| Rheumatoid Arthritis | C0003873 | ABO | 28 | P16442 | ABO, alpha 1-3-N-acetylgalactosaminyltransferase and alpha 1-3-galactosyltransferase | Enzyme | 443 | 0.427 | 0.885 | | 0.02 | | 1 | 2 | 0 | 1982 | 1987 |

| | | | | | | | | | | | | | | | | | |
|---|---|---|---|---|---|---|---|---|---|---|---|---|---|---|---|---|---|
| Rheumatoid Arthritis | C0003873 | CCR10 | 2826 | P46092 | C-C motif chemokine receptor 10 | G-protein coupled receptor | 57 | 0.626 | 0.654 | 0.000505 | 0.02 | | 1 | 2 | 0 | 2015 | 2018 |
| Rheumatoid Arthritis | C0003873 | GPR15 | 2838 | P49685 | G protein-coupled receptor 15 | G-protein coupled receptor | 18 | 0.743 | 0.308 | 1.11E-06 | 0.02 | | 1 | 2 | 0 | 2014 | 2018 |
| Rheumatoid Arthritis | C0003873 | VSTM1 | 284415 | Q6UX27 | V-set and transmembrane domain containing 1 | Receptor | 8 | 0.821 | 0.192 | 6.37E-08 | 0.02 | | 1 | 2 | 0 | 2014 | 2016 |
| Rheumatoid Arthritis | C0003873 | DLL1 | 28514 | O00548 | delta like canonical Notch ligand 1 | | 212 | 0.492 | 0.808 | 0.99999 | 0.02 | | 1 | 2 | 0 | 1990 | 2005 |
| Rheumatoid Arthritis | C0003873 | TRAV29DV5 | 28653 | P04437 | T cell receptor alpha variable 29/delta variable 5 (gene/pseudogene) | | 14 | 0.751 | 0.346 | | 0.02 | | 0.5 | 2 | 0 | 1997 | 1997 |
| Rheumatoid Arthritis | C0003873 | GPR42 | 2866 | O15529 | G protein-coupled receptor 42 (gene/pseudogene) | G-protein coupled receptor | 255 | 0.475 | 0.846 | 0.050905 | 0.02 | | 1 | 2 | 0 | 1999 | 2004 |

| | | | | | | | | | | | | | | | | | |
|---|---|---|---|---|---|---|---|---|---|---|---|---|---|---|---|---|---|
| Rheumatoid Arthritis | C0003873 | TRAJ60 | 28695 | | T cell receptor alpha joining 60 (pseudogene) | | 14 | 0.751 | 0.346 | | 0.02 | | 0.5 | 2 | 0 | 1997 | 1997 |
| Rheumatoid Arthritis | C0003873 | TRAC | 28755 | P01848 | T cell receptor alpha constant | Receptor | 28 | 0.691 | 0.615 | | 0.02 | | 0.5 | 2 | 0 | 1997 | 1997 |
| Rheumatoid Arthritis | C0003873 | GPX1 | 2876 | P07203 | glutathione peroxidase 1 | Enzyme | 290 | 0.458 | 0.923 | 3.85E-05 | 0.02 | | 1 | 2 | 0 | 2019 | 2019 |
| Rheumatoid Arthritis | C0003873 | ANPEP | 290 | P15144 | alanyl aminopeptidase, membrane | Enzyme | 280 | 0.462 | 0.769 | 3.75E-09 | 0.02 | | 1 | 2 | 0 | 2002 | 2019 |
| Rheumatoid Arthritis | C0003873 | SETD2 | 29072 | Q9BYW2 | SET domain containing 2, histone lysine methyltransferase | Epigenetic regulator | 447 | 0.424 | 0.846 | 1 | 0.02 | | 1 | 2 | 0 | 2009 | 2018 |
| Rheumatoid Arthritis | C0003873 | GRP | 2922 | P07492 | gastrin releasing peptide | | 172 | 0.524 | 0.769 | 0.002535 | 0.02 | | 1 | 2 | 0 | 2008 | 2017 |
| Rheumatoid | C0003873 | GSK3B | 2932 | P49841 | glycogen synthase kinase 3 beta | Kinase | 393 | 0.43 | 0.846 | 0.955 | 0.02 | | 1 | 2 | 0 | 2011 | 2018 |

| | | | | | | | | | | | | | | | | | |
|---|---|---|---|---|---|---|---|---|---|---|---|---|---|---|---|---|---|
| Arthritis | | | | | | | | | | 71 | | | | | | | |
| Rheumatoid Arthritis | C0003873 | MSH6 | 2956 | P52701 | mutS homolog 6 | Nucleic acid binding | 296 | 0.462 | 0.731 | 3.67E-05 | 0.02 | | 1 | 2 | 0 | 2003 | 2003 |
| Rheumatoid Arthritis | C0003873 | SENP1 | 29843 | Q9P0U3 | SUMO specific peptidase 1 | Enzyme | 79 | 0.593 | 0.654 | 0.993358 | 0.02 | | 1 | 2 | 0 | 2007 | 2010 |
| Rheumatoid Arthritis | C0003873 | A1CF | 29974 | Q9NQ94 | APOBEC1 complementation factor | | 71 | 0.604 | 0.692 | 5.92E-10 | 0.02 | | 1 | 2 | 0 | 2004 | 2018 |
| Rheumatoid Arthritis | C0003873 | TBX21 | 30009 | Q9UL17 | T-box transcription factor 21 | Transcription factor | 124 | 0.541 | 0.808 | 0.99636 | 0.02 | | 1 | 2 | 1 | 2009 | 2019 |
| Rheumatoid Arthritis | C0003873 | SLC40A1 | 30061 | Q9NP59 | solute carrier family 40 member 1 | Transporter | 101 | 0.57 | 0.731 | 0.98835 | 0.02 | | 1 | 2 | 0 | 2018 | 2019 |
| Rheumatoid Arthritis | C0003873 | HDAC1 | 3065 | Q13547 | histone deacetylase 1 | Epigenetic regulator | 277 | 0.46 | 0.846 | 0.61427 | 0.02 | | 1 | 2 | 0 | 2010 | 2015 |
| Rheuma | C0003 | HDGF | 30 | P51858 | heparin binding | Signaling | 89 | 0.5 | 0.6 | 0.27 | 0.02 | | 1 | 2 | 0 | 1990 | 20 |

| | | | | | | | | | | | | | | | | | |
|---|---|---|---|---|---|---|---|---|---|---|---|---|---|---|---|---|---|
| toid Arthritis | 873 | | 68 | | growth factor | | | 75 | 54 | 272 | | | | | | | 04 |
| Rheumatoid Arthritis | C0003873 | ADGRE2 | 30817 | Q9UHX3 | adhesion G protein-coupled receptor E2 | G-protein coupled receptor | 86 | 0.581 | 0.615 | 2.76E-28 | 0.02 | | 1 | 2 | 0 | 2000 | 2010 |
| Rheumatoid Arthritis | C0003873 | HGF | 3082 | P14210 | hepatocyte growth factor | Enzyme | 671 | 0.374 | 0.885 | 0.999947 | 0.02 | | 1 | 2 | 0 | 1997 | 2001 |
| Rheumatoid Arthritis | C0003873 | ANXA6 | 309 | P08133 | annexin A6 | | 156 | 0.514 | 0.846 | 1.08E-08 | 0.02 | | 1 | 2 | 0 | 1998 | 2017 |
| Rheumatoid Arthritis | C0003873 | HIP1 | 3092 | O00291 | huntingtin interacting protein 1 | Cellular structure | 56 | 0.653 | 0.577 | 0.76173 | 0.02 | | 1 | 2 | 0 | 2018 | 2018 |
| Rheumatoid Arthritis | C0003873 | NR4A1 | 3164 | P22736 | nuclear receptor subfamily 4 group A member 1 | Nuclear receptor | 216 | 0.49 | 0.731 | 0.37074 | 0.02 | | 1 | 2 | 0 | 2002 | 2019 |
| Rheumatoid Ar | C0003873 | HRAS | 3265 | P01112 | HRas proto-oncogene, GTPase | Enzyme modulator | 698 | 0.378 | 0.885 | 0.079774 | 0.02 | | 0.5 | 2 | 0 | 1995 | 2000 |

| | | | | | | | | | | | | | | | | | |
|---|---|---|---|---|---|---|---|---|---|---|---|---|---|---|---|---|---|
| thritis | | | | | | | | | | | | | | | | | |
| Rheumatoid Arthritis | C0003873 | HSD11B2 | 3291 | P80365 | hydroxysteroid 11-beta dehydrogenase 2 | Enzyme | 163 | 0.532 | 0.808 | 0.11777 | 0.02 | | 1 | 2 | 0 | 2006 | 2015 |
| Rheumatoid Arthritis | C0003873 | HSPA8 | 3312 | P11142 | heat shock protein family A (Hsp70) member 8 | | 124 | 0.553 | 0.808 | 0.99863 | 0.02 | | 0.5 | 2 | 0 | 2002 | 2018 |
| Rheumatoid Arthritis | C0003873 | BIRC5 | 3332 | O15392 | baculoviral IAP repeat containing 5 | Enzyme modulator | 199 | 0.495 | 0.731 | 0.05854 | 0.02 | | 1 | 2 | 0 | 2007 | 2016 |
| Rheumatoid Arthritis | C0003873 | DNAJB1 | 3337 | P25685 | DnaJ heat shock protein family (Hsp40) member B1 | | 82 | 0.597 | 0.692 | 0.039437 | 0.02 | | 1 | 2 | 0 | 2019 | 2019 |
| Rheumatoid Arthritis | C0003873 | ICA1 | 3382 | Q05084 | islet cell autoantigen 1 | | 34 | 0.686 | 0.5 | 0.000991 | 0.02 | | 1 | 2 | 0 | 1995 | 1999 |
| Rheumatoid Arthritis | C0003873 | ID1 | 3397 | P41134 | inhibitor of DNA binding 1, HLH protein | Transcription factor | 125 | 0.544 | 0.692 | 0.00031 | 0.02 | | 1 | 2 | 0 | 2001 | 2019 |
| Rheumatoi | C0003 | IFNGR1 | 3459 | P15260 | interferon gamma | Receptor | 153 | 0.5 | 0.6 | 0.015 | 0.02 | | 0.5 | 2 | 0 | 2006 | 2015 |

| | | | | | | | | | | | | | | | | | |
|---|---|---|---|---|---|---|---|---|---|---|---|---|---|---|---|---|---|
| d Arthritis | 873 | | | | receptor 1 | | | 29 | 92 | 503 | | | | | | | |
| Rheumatoid Arthritis | C0003873 | IGFBP1 | 3484 | P08833 | insulin like growth factor binding protein 1 | Enzyme modulator | 191 | 0.502 | 0.808 | 0.009947 | 0.02 | | 1 | 2 | 0 | 2017 | 2018 |
| Rheumatoid Arthritis | C0003873 | IGHA1 | 3493 | P01876 | immunoglobulin heavy constant alpha 1 | | 64 | 0.617 | 0.692 | | 0.02 | | 1 | 2 | 0 | 1991 | 2017 |
| Rheumatoid Arthritis | C0003873 | APP | 351 | P05067 | amyloid beta precursor protein | Enzyme modulator | 485 | 0.422 | 0.846 | 0.046544 | 0.02 | | 1 | 2 | 0 | 2011 | 2016 |
| Rheumatoid Arthritis | C0003873 | APRT | 353 | P07741 | adenine phosphoribosyltransferase | | 384 | 0.423 | 0.846 | 5.45E-13 | 0.02 | | 1 | 2 | 0 | 2018 | 2019 |
| Rheumatoid Arthritis | C0003873 | KLK3 | 354 | P07288 | kallikrein related peptidase 3 | Enzyme | 358 | 0.443 | 0.885 | 4.43E-06 | 0.02 | | 1 | 2 | 0 | 1994 | 2016 |
| Rheumatoid Arthritis | C0003873 | IL1RAP | 3556 | Q9NPH3 | interleukin 1 receptor accessory protein | Receptor | 49 | 0.647 | 0.654 | 0.25846 | 0.02 | | 1 | 2 | 0 | 2019 | 2020 |

| | | | | | | | | | | | | | | | | | |
|---|---|---|---|---|---|---|---|---|---|---|---|---|---|---|---|---|---|
| Rheumatoid Arthritis | C0003873 | IL5RA | 3568 | Q01344 | interleukin 5 receptor subunit alpha | Signaling | 48 | 0.647 | 0.654 | 2.68E-08 | 0.02 | | 0.5 | 2 | 0 | 1995 | 1998 |
| Rheumatoid Arthritis | C0003873 | IL9R | 3581 | Q01113 | interleukin 9 receptor | Receptor | 44 | 0.659 | 0.654 | 2.68E-22 | 0.02 | | 1 | 2 | 1 | 2009 | 2018 |
| Rheumatoid Arthritis | C0003873 | IL10RB | 3588 | Q08334 | interleukin 10 receptor subunit beta | Receptor | 50 | 0.636 | 0.577 | 2.51E-06 | 0.02 | | 1 | 2 | 0 | 2006 | 2013 |
| Rheumatoid Arthritis | C0003873 | IL11 | 3589 | P20809 | interleukin 11 | | 244 | 0.481 | 0.808 | 0.000318 | 0.02 | | 1 | 2 | 0 | 2000 | 2018 |
| Rheumatoid Arthritis | C0003873 | IDO1 | 3620 | P14902 | indoleamine 2,3-dioxygenase 1 | | 295 | 0.454 | 0.846 | 3.3E-06 | 0.02 | | 1 | 2 | 0 | 2011 | 2017 |
| Rheumatoid Arthritis | C0003873 | INHBA | 3624 | P08476 | inhibin subunit beta A | Signaling | 83 | 0.601 | 0.615 | 0.97979 | 0.02 | | 0.5 | 2 | 0 | 1998 | 2006 |
| Rheumatoid Ar | C0003873 | INSR | 3643 | P06213 | insulin receptor | Kinase | 452 | 0.432 | 0.846 | 3.61E- | 0.02 | | 1 | 2 | 0 | 2016 | 2017 |

| | | | | | | | | | | | | | | | | | |
|---|---|---|---|---|---|---|---|---|---|---|---|---|---|---|---|---|---|
| thritis | | | | | | | | | | 05 | | | | | | | |
| Rheumatoid Arthritis | C0003873 | INSRR | 3645 | P14616 | insulin receptor related receptor | Kinase | 186 | 0.508 | 0.808 | 5.4E-24 | 0.02 | | 1 | 2 | 0 | 2017 | 2018 |
| Rheumatoid Arthritis | C0003873 | AQP9 | 366 | O43315 | aquaporin 9 | Ion channel | 100 | 0.565 | 0.808 | 0.240227 | 0.02 | | 1 | 2 | 0 | 2016 | 2017 |
| Rheumatoid Arthritis | C0003873 | ITGA2 | 3673 | P17301 | integrin subunit alpha 2 | | 166 | 0.522 | 0.731 | 6.28E-11 | 0.02 | | 0.5 | 2 | 0 | 2009 | 2018 |
| Rheumatoid Arthritis | C0003873 | ITGAE | 3682 | P38570 | integrin subunit alpha E | | 95 | 0.579 | 0.692 | 3.92E-20 | 0.02 | | 1 | 2 | 0 | 2017 | 2019 |
| Rheumatoid Arthritis | C0003873 | ITGAX | 3687 | P20702 | integrin subunit alpha X | | 170 | 0.504 | 0.769 | 1.37E-15 | 0.02 | | 1 | 2 | 0 | 2010 | 2012 |
| Rheumatoid Arthritis | C0003873 | AREG | 374 | P15514 | amphiregulin | Signaling | 221 | 0.487 | 0.769 | 0.354407 | 0.02 | | 1 | 2 | 0 | 2006 | 2010 |
| Rheumatoi | C0003 | KCNMA1 | 3778 | Q12791 | potassium calcium-activate | Ion channel | 205 | 0.5 | 0.8 | 0.997 | 0.02 | | 1 | 2 | 0 | 2017 | 2018 |

| | | | | | | | | | | | | | | | | | |
|---|---|---|---|---|---|---|---|---|---|---|---|---|---|---|---|---|---|
| d Arthritis | 873 | | | | d channel subfamily M alpha 1 | | | 18 | 46 | 31 | | | | | | | |
| Rheumatoid Arthritis | C0003873 | MALAT1 | 378938 | | metastasis associated lung adenocarcinoma transcript 1 | | 336 | 0.435 | 0.846 | | 0.02 | | 1 | 2 | 0 | 2019 | 2019 |
| Rheumatoid Arthritis | C0003873 | KIR2DL3 | 3804 | P43628 | killer cell immunoglobulin like receptor, two Ig domains and long cytoplasmic tail 3 | Receptor | 57 | 0.612 | 0.654 | 2.68E-09 | 0.02 | | 1 | 2 | 0 | 2012 | 2015 |
| Rheumatoid Arthritis | C0003873 | KIR2DS5 | 3810 | Q14953 | killer cell immunoglobulin like receptor, two Ig domains and short cytoplasmic tail 5 | Receptor | 27 | 0.678 | 0.5 | | 0.02 | | 1 | 2 | 0 | 2010 | 2015 |
| Rheumatoid Arthritis | C0003873 | KLK1 | 3816 | P06870 | kallikrein 1 | Enzyme | 132 | 0.533 | 0.769 | 0.000153 | 0.02 | | 1 | 2 | 0 | 2007 | 2019 |
| Rheumatoid Arthritis | C0003873 | KNG1 | 3827 | P01042 | kininogen 1 | Enzyme modulator | 279 | 0.481 | 0.885 | 3.21E-10 | 0.02 | | 1 | 2 | 0 | 2017 | 2018 |

| | | | | | | | | | | | | | | | | | |
|---|---|---|---|---|---|---|---|---|---|---|---|---|---|---|---|---|---|
| Rheumatoid Arthritis | C0003873 | ARG1 | 383 | P05089 | arginase 1 | Enzyme | 273 | 0.476 | 0.846 | 0.0003359 | 0.02 | | 1 | 2 | 0 | 2013 | 2020 |
| Rheumatoid Arthritis | C0003873 | KPNA1 | 3836 | P52294 | karyopherin subunit alpha 1 | Transporter | 22 | 0.736 | 0.577 | 0.99818 | 0.02 | | 1 | 2 | 0 | 2008 | 2008 |
| Rheumatoid Arthritis | C0003873 | CCL4L1 | 388372 | P13236;Q8NHW4 | C-C motif chemokine ligand 4 like 1 | | 135 | 0.534 | 0.885 | 0.33576 | 0.02 | | 1 | 2 | 0 | 1994 | 1995 |
| Rheumatoid Arthritis | C0003873 | LDLR | 3949 | P01130 | low density lipoprotein receptor | | 336 | 0.449 | 0.885 | 9.84E-24 | 0.02 | | 1 | 2 | 0 | 2013 | 2017 |
| Rheumatoid Arthritis | C0003873 | LGALS2 | 3957 | P05162 | galectin 2 | Signaling | 27 | 0.7 | 0.385 | 0.020219 | 0.02 | | 0.5 | 2 | 0 | 2008 | 2009 |
| Rheumatoid Arthritis | C0003873 | LGALS3BP | 3959 | Q08380 | galectin 3 binding protein | | 127 | 0.546 | 0.769 | 2.68E-07 | 0.02 | | 1 | 2 | 0 | 2000 | 2003 |
| Rheumatoid Ar | C0003873 | LGALS8 | 3964 | O00214 | galectin 8 | Signaling | 51 | 0.628 | 0.615 | 3.88E- | 0.02 | | 1 | 2 | 1 | 2009 | 2012 |

| | | | | | | | | | | | | | | | | |
|---|---|---|---|---|---|---|---|---|---|---|---|---|---|---|---|---|
| thritis | | | | | | | | | 06 | | | | | | | |
| Rheumatoid Arthritis | C0003873 | LIG4 | 3981 | P49917 | DNA ligase 4 | 293 | 0.478 | 0.846 | 4.16E-06 | 0.02 | | 1 | 2 | 0 | 2003 | 2009 |
| Rheumatoid Arthritis | C0003873 | LRPAP1 | 4043 | P30533 | LDL receptor related protein associated protein 1 | 95 | 0.579 | 0.731 | 4.5E-07 | 0.02 | | 1 | 2 | 0 | 2003 | 2003 |
| Rheumatoid Arthritis | C0003873 | SH2D1A | 4068 | O60880 | SH2 domain containing 1A | 160 | 0.525 | 0.731 | 0.38935 | 0.02 | | 1 | 2 | 0 | 2001 | 2016 |
| Rheumatoid Arthritis | C0003873 | MIR132 | 406921 | | microRNA 132 | 222 | 0.485 | 0.846 | | 0.02 | | 1 | 2 | 0 | 2008 | 2019 |
| Rheumatoid Arthritis | C0003873 | MIR152 | 406943 | | microRNA 152 | 136 | 0.521 | 0.769 | | 0.02 | | 1 | 2 | 0 | 2014 | 2018 |
| Rheumatoid Arthritis | C0003873 | MIR17 | 406952 | | microRNA 17 | 270 | 0.459 | 0.846 | | 0.02 | | 1 | 2 | 0 | 2018 | 2018 |
| Rheumatoi | C0003 | MIR192 | 4069 | | microRNA 192 | 173 | 0.5 | 0.8 | | 0.02 | | 1 | 2 | 0 | 2017 | 2020 |

| | | | | | | | | | | | | | | | | |
|---|---|---|---|---|---|---|---|---|---|---|---|---|---|---|---|---|
| d Arthritis | 873 | | 67 | | | | 03 | 46 | | | | | | | | |
| Rheumatoid Arthritis | C0003873 | MIR210 | 406992 | | microRNA 210 | 277 | 0.46 | 0.846 | | 0.02 | | 1 | 2 | 0 | 2017 | 2018 |
| Rheumatoid Arthritis | C0003873 | MIR214 | 406996 | | microRNA 214 | 221 | 0.48 | 0.808 | | 0.02 | | 1 | 2 | 0 | 2017 | 2019 |
| Rheumatoid Arthritis | C0003873 | MIR222 | 407007 | | microRNA 222 | 210 | 0.486 | 0.808 | | 0.02 | | 1 | 2 | 0 | 2019 | 2019 |
| Rheumatoid Arthritis | C0003873 | MIR29A | 407021 | | microRNA 29a | 278 | 0.459 | 0.808 | | 0.02 | | 1 | 2 | 0 | 2014 | 2017 |
| Rheumatoid Arthritis | C0003873 | MIR30A | 407029 | | microRNA 30a | 218 | 0.478 | 0.808 | | 0.02 | | 1 | 2 | 0 | 2013 | 2014 |
| Rheumatoid Arthritis | C0003873 | MIR98 | 407054 | | microRNA 98 | 104 | 0.565 | 0.731 | | 0.02 | | 1 | 2 | 0 | 2016 | 2019 |

| | | | | | | | | | | | | | | | | | |
|---|---|---|---|---|---|---|---|---|---|---|---|---|---|---|---|---|---|
| Rheumatoid Arthritis | C0003873 | SMAD7 | 4092 | O15105 | SMAD family member 7 | Transcription factor | 269 | 0.47 | 0.769 | 0.54854 | 0.02 | | 1 | 2 | 0 | 2016 | 2018 |
| Rheumatoid Arthritis | C0003873 | MCAM | 4162 | P43121 | melanoma cell adhesion molecule | Cell adhesion | 169 | 0.514 | 0.731 | 0.00016 | 0.02 | | 1 | 2 | 0 | 1999 | 2015 |
| Rheumatoid Arthritis | C0003873 | MET | 4233 | P08581 | MET proto-oncogene, receptor tyrosine kinase | Kinase | 594 | 0.38 | 0.846 | 0.96998 | 0.02 | | 1 | 2 | 0 | 2001 | 2019 |
| Rheumatoid Arthritis | C0003873 | MFAP1 | 4236 | P55081 | microfibril associated protein 1 | Extracellular structure | 357 | 0.431 | 0.846 | 0.79602 | 0.02 | | 1 | 2 | 0 | 2018 | 2019 |
| Rheumatoid Arthritis | C0003873 | MFAP4 | 4239 | P55083 | microfibril associated protein 4 | Signaling | 31 | 0.691 | 0.462 | 0.11416 | 0.02 | | 1 | 2 | 0 | 2019 | 2020 |
| Rheumatoid Arthritis | C0003873 | MKI67 | 4288 | P46013 | marker of proliferation Ki-67 | | 351 | 0.431 | 0.808 | 1.26E-28 | 0.02 | | 1 | 2 | 0 | 2001 | 2002 |
| Rheumatoid Ar | C0003873 | ASIP | 434 | P42127 | agouti signaling protein | Signaling | 89 | 0.584 | 0.692 | 0.01662 | 0.02 | | 1 | 2 | 0 | 2004 | 2018 |

| | | | | | | | | | | | | | | | | | |
|---|---|---|---|---|---|---|---|---|---|---|---|---|---|---|---|---|---|
| thritis | | | | | | | | | | 98 | | | | | | | |
| Rheumatoid Arthritis | C0003873 | MOG | 4340 | Q16653 | myelin oligodendrocyte glycoprotein | Enzyme modulator | 136 | 0.546 | 0.731 | 0.137478 | 0.02 | | 1 | 2 | 0 | 2011 | 2017 |
| Rheumatoid Arthritis | C0003873 | MRC1 | 4360 | P22897 | mannose receptor C-type 1 | | 431 | 0.413 | 0.846 | 0.94335 | 0.02 | | 1 | 2 | 0 | 2003 | 2017 |
| Rheumatoid Arthritis | C0003873 | MRE11 | 4361 | P49959 | MRE11 homolog, double strand break repair nuclease | | 171 | 0.527 | 0.808 | 1.26E-12 | 0.02 | | 1 | 2 | 0 | 2009 | 2019 |
| Rheumatoid Arthritis | C0003873 | ZFAS1 | 441951 | | ZNFX1 antisense RNA 1 | | 82 | 0.582 | 0.615 | | 0.02 | | 0.5 | 2 | 0 | 2018 | 2019 |
| Rheumatoid Arthritis | C0003873 | MIR346 | 442911 | | microRNA 346 | | 52 | 0.631 | 0.615 | | 0.02 | | 1 | 2 | 0 | 2009 | 2011 |
| Rheumatoid Arthritis | C0003873 | ASPA | 443 | P45381 | aspartoacylase | | 123 | 0.564 | 0.731 | 0.000411 | 0.02 | | 1 | 2 | 0 | 2004 | 2018 |
| Rheumatoi | C0003 | MST1 | 4485 | P26927 | macrophage stimulating 1 | Enzyme | 281 | 0.4 | 0.8 | 4.78E | 0.02 | | 1 | 2 | 0 | 2015 | 2017 |

| | | | | | | | | | | | | | | | | | |
|---|---|---|---|---|---|---|---|---|---|---|---|---|---|---|---|---|---|
| d Arthritis | 873 | | | | | | | 65 | 85 | -19 | | | | | | | |
| Rheumatoid Arthritis | C0003873 | COX1 | 4512 | P00395 | cytochrome c oxidase subunit I | Enzyme | 421 | 0.441 | 0.885 | | 0.02 | | 1 | 2 | 0 | 1994 | 2002 |
| Rheumatoid Arthritis | C0003873 | MUTYH | 4595 | Q9UIF7 | mutY DNA glycosylase | Enzyme | 156 | 0.521 | 0.769 | 1.26E-18 | 0.02 | | 1 | 2 | 2 | 2015 | 2017 |
| Rheumatoid Arthritis | C0003873 | MYO9B | 4650 | Q13459 | myosin IXB | | 65 | 0.644 | 0.615 | 1 | 0.02 | | 1 | 2 | 2 | 2007 | 2008 |
| Rheumatoid Arthritis | C0003873 | ATHS | 470 | | atherosclerosis susceptibility (lipoprotein associated) | | 112 | 0.551 | 0.769 | | 0.02 | | 1 | 2 | 0 | 2003 | 2019 |
| Rheumatoid Arthritis | C0003873 | ATM | 472 | Q13315 | ATM serine/threonine kinase | Kinase | 684 | 0.374 | 0.885 | 5.59E-47 | 0.02 | | 1 | 2 | 0 | 2018 | 2019 |
| Rheumatoid Arthritis | C0003873 | NGF | 4803 | P01138 | nerve growth factor | Signaling | 616 | 0.391 | 0.885 | 0.82026 | 0.02 | | 1 | 2 | 0 | 1993 | 2009 |

| | | | | | | | | | | | | | | | | | |
|---|---|---|---|---|---|---|---|---|---|---|---|---|---|---|---|---|---|
| Rheumatoid Arthritis | C0003873 | NHS | 4810 | Q6T4R5 | NHS actin remodeling regulator | | 295 | 0.466 | 0.846 | 0.99999 | 0.02 | | 1 | 2 | 1 | 2017 | 2019 |
| Rheumatoid Arthritis | C0003873 | NME1 | 4830 | P15531 | NME/NM23 nucleoside diphosphate kinase 1 | Kinase | 329 | 0.443 | 0.808 | 0.003176 | 0.02 | | 1 | 2 | 0 | 1996 | 2017 |
| Rheumatoid Arthritis | C0003873 | NOS1 | 4842 | P29475 | nitric oxide synthase 1 | | 521 | 0.408 | 0.885 | 1 | 0.02 | | 1 | 2 | 0 | 2009 | 2010 |
| Rheumatoid Arthritis | C0003873 | NOTCH3 | 4854 | Q9UM47 | notch receptor 3 | | 418 | 0.435 | 0.808 | 0.40757 | 0.02 | | 1 | 2 | 0 | 2001 | 2016 |
| Rheumatoid Arthritis | C0003873 | NPPA | 4878 | P01160 | natriuretic peptide A | | 217 | 0.505 | 0.769 | 0.000545 | 0.02 | | 1 | 2 | 0 | 1993 | 2018 |
| Rheumatoid Arthritis | C0003873 | NT5E | 4907 | P21589 | 5'-nucleotidase ecto | Enzyme | 376 | 0.436 | 0.885 | 2.29E-11 | 0.02 | | 1 | 2 | 0 | 1999 | 2019 |
| Rheumatoid Ar | C0003873 | ODC1 | 4953 | P11926 | ornithine decarboxylase 1 | Enzyme | 184 | 0.51 | 0.731 | 0.016966 | 0.02 | | 1 | 2 | 0 | 2000 | 2018 |

| | | | | | | | | | | | | | | | | | |
|---|---|---|---|---|---|---|---|---|---|---|---|---|---|---|---|---|---|
| thritis | | | | | | | | | | | | | | | | | |
| Rheumatoid Arthritis | C0003873 | OXA1L | 5018 | Q15070 | OXA1L mitochondrial inner membrane protein | Transporter | 75 | 0.59 | 0.731 | 1.87E-10 | 0.02 | | 1 | 2 | 0 | 2019 | 2019 |
| Rheumatoid Arthritis | C0003873 | G0S2 | 50486 | P27469 | G0/G1 switch 2 | | 47 | 0.656 | 0.423 | 0.37788 | 0.02 | | 1 | 2 | 0 | 2008 | 2014 |
| Rheumatoid Arthritis | C0003873 | NOX4 | 50507 | Q9NPH5 | NADPH oxidase 4 | Enzyme | 260 | 0.471 | 0.885 | 5.76E-20 | 0.02 | | 1 | 2 | 0 | 2006 | 2019 |
| Rheumatoid Arthritis | C0003873 | PAK1 | 5058 | Q13153 | p21 (RAC1) activated kinase 1 | Kinase | 221 | 0.494 | 0.808 | 0.001331 | 0.02 | | 1 | 2 | 0 | 2012 | 2019 |
| Rheumatoid Arthritis | C0003873 | PAM | 5066 | P19021 | peptidylglycine alpha-amidating monooxygenase | Enzyme | 155 | 0.542 | 0.692 | 6.93E-06 | 0.02 | | 1 | 2 | 0 | 2002 | 2019 |
| Rheumatoid Arthritis | C0003873 | PAX5 | 5079 | Q02548 | paired box 5 | | 199 | 0.487 | 0.654 | 0.99798 | 0.02 | | 1 | 2 | 0 | 2013 | 2014 |
| Rheumatoi | C0003 | ASCC1 | 510 | Q8N9N2 | activating signal cointegrator 1 | | 209 | 0.4 | 0.8 | 2.61E | 0.02 | | 1 | 2 | 1 | 2002 | 2015 |

| | | | | | | | | | | | | | | | | | |
|---|---|---|---|---|---|---|---|---|---|---|---|---|---|---|---|---|---|
| d Arthritis | 873 | | 08 | | complex subunit 1 | | | 95 | 46 | -14 | | | | | | | |
| Rheumatoid Arthritis | C0003873 | ADIPOR1 | 51094 | Q96A54 | adiponectin receptor 1 | Receptor | 148 | 0.529 | 0.808 | 0.6584 | 0.02 | | 1 | 2 | 1 | 2009 | 2011 |
| Rheumatoid Arthritis | C0003873 | PCNA | 5111 | P12004 | proliferating cell nuclear antigen | Nucleic acid binding | 581 | 0.382 | 0.846 | 0.97606 | 0.02 | | 1 | 2 | 0 | 1997 | 2001 |
| Rheumatoid Arthritis | C0003873 | PLEKHO1 | 51177 | Q53GL0 | pleckstrin homology domain containing O1 | | 44 | 0.638 | 0.615 | 0.48341 | 0.02 | | 1 | 2 | 0 | 2018 | 2019 |
| Rheumatoid Arthritis | C0003873 | PDC | 5132 | P20941 | phosducin | | 95 | 0.581 | 0.731 | 0.000101 | 0.02 | | 1 | 2 | 0 | 2012 | 2020 |
| Rheumatoid Arthritis | C0003873 | TNFRSF12A | 51330 | Q9NP84 | TNF receptor superfamily member 12A | | 173 | 0.509 | 0.692 | 0.017995 | 0.02 | | 1 | 2 | 0 | 2007 | 2012 |
| Rheumatoid Arthritis | C0003873 | TRPV2 | 51393 | Q9Y5S1 | transient receptor potential cation channel subfamily V member 2 | Ion channel | 90 | 0.581 | 0.615 | 7.92E-13 | 0.02 | | 1 | 2 | 0 | 2015 | 2019 |

| | | | | | | | | | | | | | | | | | |
|---|---|---|---|---|---|---|---|---|---|---|---|---|---|---|---|---|---|
| Rheumatoid Arthritis | C0003873 | PDE4A | 5141 | P27815 | phosphodiesterase 4A | | 168 | 0.523 | 0.846 | 0.98167 | 0.02 | | 1 | 2 | 0 | 2011 | 2019 |
| Rheumatoid Arthritis | C0003873 | PDE7A | 5150 | Q13946 | phosphodiesterase 7A | | 40 | 0.659 | 0.577 | 0.004565 | 0.02 | | 1 | 2 | 0 | 2006 | 2018 |
| Rheumatoid Arthritis | C0003873 | PDGFRA | 5156 | P16234 | platelet derived growth factor receptor alpha | Kinase | 452 | 0.415 | 0.808 | 1 | 0.02 | | 1 | 2 | 0 | 2007 | 2018 |
| Rheumatoid Arthritis | C0003873 | ENPP2 | 5168 | Q13822 | ectonucleotide pyrophosphatase/phosphodiesterase 2 | Enzyme | 95 | 0.582 | 0.769 | 0.002791 | 0.02 | | 1 | 2 | 0 | 2001 | 2015 |
| Rheumatoid Arthritis | C0003873 | ADA2 | 51816 | Q9NZK5 | adenosine deaminase 2 | Enzyme | 165 | 0.545 | 0.846 | 8.14E-08 | 0.02 | | 1 | 2 | 0 | 2003 | 2010 |
| Rheumatoid Arthritis | C0003873 | PGR | 5241 | P06401 | progesterone receptor | Nuclear receptor | 392 | 0.426 | 0.885 | 0.051042 | 0.02 | | 1 | 2 | 0 | 2004 | 2009 |
| Rheumatoid Ar | C0003873 | PITX2 | 5308 | Q99697 | paired like homeodomain 2 | | 248 | 0.489 | 0.808 | 0.97618 | 0.02 | | 1 | 2 | 0 | 2011 | 2020 |

| | | | | | | | | | | | | | | | | | |
|---|---|---|---|---|---|---|---|---|---|---|---|---|---|---|---|---|---|
| thritis | | | | | | | | | | | | | | | | | |
| Rheumatoid Arthritis | C0003873 | PLA2G4A | 5321 | P47712 | phospholipase A2 group IVA | Enzyme | 202 | 0.496 | 0.846 | 5.98E-05 | 0.02 | | 1 | 2 | 0 | 2004 | 2010 |
| Rheumatoid Arthritis | C0003873 | TREM2 | 54209 | Q9NZC2 | triggering receptor expressed on myeloid cells 2 | | 239 | 0.519 | 0.769 | 3.29E-09 | 0.02 | | 1 | 2 | 0 | 2012 | 2018 |
| Rheumatoid Arthritis | C0003873 | POU2AF1 | 5450 | Q16633 | POU class 2 homeobox associating factor 1 | | 70 | 0.608 | 0.615 | 0.56553 | 0.02 | | 1 | 2 | 0 | 2017 | 2019 |
| Rheumatoid Arthritis | C0003873 | ARL15 | 54622 | Q9NXU5 | ADP ribosylation factor like GTPase 15 | | 34 | 0.769 | 0.346 | 0.000936 | 0.02 | | 1 | 2 | 1 | 2013 | 2018 |
| Rheumatoid Arthritis | C0003873 | AHI1 | 54806 | Q8N157 | Abelson helper integration site 1 | | 221 | 0.513 | 0.846 | 1.08E-25 | 0.02 | | 1 | 2 | 0 | 2000 | 2004 |
| Rheumatoid Arthritis | C0003873 | ATG16L1 | 55054 | Q676U5 | autophagy related 16 like 1 | | 120 | 0.543 | 0.769 | 0.96452 | 0.02 | | 1 | 2 | 0 | 2012 | 2019 |
| Rheumatoi | C0003 | NAT10 | 552 | Q9H0A0 | N-acetyltransferase 10 | | 133 | 0.5 | 0.7 | 4.12E | 0.02 | | 1 | 2 | 0 | 2003 | 2019 |

| | | | | | | | | | | | | | | | | | |
|---|---|---|---|---|---|---|---|---|---|---|---|---|---|---|---|---|---|
| d Arthritis | 873 | | 26 | | | | | 36 | 69 | -22 | | | | | | | |
| Rheumatoid Arthritis | C0003873 | PRELP | 5549 | P51888 | proline and arginine rich end leucine rich repeat protein | | 26 | 0.695 | 0.5 | 4.71E-05 | 0.02 | | 1 | 2 | 0 | 2012 | 2018 |
| Rheumatoid Arthritis | C0003873 | CHD7 | 55636 | Q9P2D1 | chromodomain helicase DNA binding protein 7 | | 419 | 0.441 | 0.846 | 1 | 0.02 | | 1 | 2 | 0 | 2014 | 2018 |
| Rheumatoid Arthritis | C0003873 | WDR11 | 55717 | Q9BZH6 | WD repeat domain 11 | | 179 | 0.524 | 0.808 | 1.37E-08 | 0.02 | | 1 | 2 | 0 | 2001 | 2007 |
| Rheumatoid Arthritis | C0003873 | MAP2K1 | 5604 | Q02750 | mitogen-activated protein kinase kinase 1 | Kinase | 389 | 0.439 | 0.846 | 0.89669 | 0.02 | | 1 | 2 | 0 | 2010 | 2019 |
| Rheumatoid Arthritis | C0003873 | CCL28 | 56477 | Q9NRJ3 | C-C motif chemokine ligand 28 | | 85 | 0.587 | 0.769 | 0.051599 | 0.02 | | 1 | 2 | 0 | 2015 | 2018 |
| Rheumatoid Arthritis | C0003873 | SLC12A9 | 56996 | Q9BXP2 | solute carrier family 12 member 9 | Transporter | 280 | 0.454 | 0.769 | 1.18E-15 | 0.02 | | 1 | 2 | 0 | 2000 | 2003 |

| | | | | | | | | | | | | | | | | | |
|---|---|---|---|---|---|---|---|---|---|---|---|---|---|---|---|---|---|
| Rheumatoid Arthritis | C0003873 | CEMIP | 57214 | Q8WUJ3 | cell migration inducing hyaluronidase 1 | | 64 | 0.617 | 0.615 | 4.4E-17 | 0.02 | | 1 | 2 | 0 | 2013 | 2015 |
| Rheumatoid Arthritis | C0003873 | AICDA | 57379 | Q9GZX7 | activation induced cytidine deaminase | Enzyme | 265 | 0.463 | 0.885 | 9.21E-06 | 0.02 | | 1 | 2 | 0 | 2009 | 2010 |
| Rheumatoid Arthritis | C0003873 | MIR410 | 574434 | | microRNA 410 | | 87 | 0.575 | 0.692 | | 0.02 | | 1 | 2 | 0 | 2019 | 2019 |
| Rheumatoid Arthritis | C0003873 | KIDINS220 | 57498 | Q9ULH0 | kinase D interacting substrate 220 | | 145 | 0.534 | 0.769 | 0.073562 | 0.02 | | 1 | 2 | 1 | 2009 | 2018 |
| Rheumatoid Arthritis | C0003873 | MIB1 | 57534 | Q86YT6 | mindbomb E3 ubiquitin protein ligase 1 | Enzyme | 329 | 0.438 | 0.769 | 3.36E-88 | 0.02 | | 1 | 2 | 0 | 2001 | 2002 |
| Rheumatoid Arthritis | C0003873 | PTN | 5764 | P21246 | pleiotrophin | Signaling | 164 | 0.515 | 0.808 | 0.007507 | 0.02 | | 1 | 2 | 0 | 2003 | 2017 |
| Rheumatoid Ar | C0003873 | CIP2A | 57650 | Q8TCG1 | cellular inhibitor of PP2A | | 141 | 0.524 | 0.808 | 1.36E- | 0.02 | | 1 | 2 | 0 | 2012 | 2013 |

| | | | | | | | | | | | | | | | | | |
|---|---|---|---|---|---|---|---|---|---|---|---|---|---|---|---|---|---|
| thritis | | | | | | | | | | 13 | | | | | | | |
| Rheumatoid Arthritis | C0003873 | PHRF1 | 57661 | Q9P1Y6 | PHD and ring finger domains 1 | | 17 | 0.743 | 0.346 | 0.0056521 | 0.02 | | 1 | 2 | 0 | 2011 | 2014 |
| Rheumatoid Arthritis | C0003873 | BAK1 | 578 | Q16611 | BCL2 antagonist/killer 1 | Signaling | 115 | 0.555 | 0.769 | 7.4E-06 | 0.02 | | 1 | 2 | 0 | 2007 | 2018 |
| Rheumatoid Arthritis | C0003873 | RAC2 | 5880 | P15153 | Rac family small GTPase 2 | Enzyme modulator | 119 | 0.556 | 0.731 | 0.966627 | 0.02 | | 1 | 2 | 0 | 2007 | 2020 |
| Rheumatoid Arthritis | C0003873 | MOK | 5891 | Q9UQ07 | MOK protein kinase | Kinase | 251 | 0.476 | 0.769 | 8.84E-22 | 0.02 | | 1 | 2 | 0 | 2009 | 2014 |
| Rheumatoid Arthritis | C0003873 | RAF1 | 5894 | P04049 | Raf-1 proto-oncogene, serine/threonine kinase | Kinase | 470 | 0.418 | 0.885 | 0.852995 | 0.02 | | 1 | 2 | 0 | 2004 | 2013 |
| Rheumatoid Arthritis | C0003873 | BCHE | 590 | P06276 | butyrylcholinesterase | | 392 | 0.447 | 0.923 | 1.06E-13 | 0.02 | | 1 | 2 | 0 | 2015 | 2019 |
| Rheumatoi | C0003 | PLAAT4 | 5920 | Q9UL19 | phospholipase A and acyltransferase 4 | | 190 | 0.5 | 0.8 | 1.22E | 0.02 | | 1 | 2 | 0 | 2008 | 2009 |

| | | | | | | | | | | | | | | | | | |
|---|---|---|---|---|---|---|---|---|---|---|---|---|---|---|---|---|---|
| d Arthritis | 873 | | | | | | | | 08 | -07 | | | | | | | |
| Rheumatoid Arthritis | C0003873 | RB1 | 5925 | P06400 | RB transcriptional corepressor 1 | Nucleic acid binding | 339 | 0.444 | 0.885 | 1 | 0.02 | | 1 | 2 | 0 | 2004 | 2018 |
| Rheumatoid Arthritis | C0003873 | UBL5 | 59286 | Q9BZL1 | ubiquitin like 5 | | 29 | 0.686 | 0.5 | 0.016321 | 0.02 | | 1 | 2 | 0 | 2018 | 2018 |
| Rheumatoid Arthritis | C0003873 | RBP2 | 5948 | P50120 | retinol binding protein 2 | | 60 | 0.615 | 0.615 | 0.22089 | 0.02 | | 1 | 2 | 0 | 1996 | 2005 |
| Rheumatoid Arthritis | C0003873 | RDX | 5962 | P35241 | radixin | Cellular structure | 80 | 0.593 | 0.615 | 0.85245 | 0.02 | | 1 | 2 | 0 | 2014 | 2020 |
| Rheumatoid Arthritis | C0003873 | RNASE2 | 6036 | P10153 | ribonuclease A family member 2 | | 61 | 0.621 | 0.692 | | 0.02 | | 1 | 2 | 1 | 2015 | 2019 |
| Rheumatoid Arthritis | C0003873 | BCL6 | 604 | P41182 | BCL6 transcription repressor | | 309 | 0.444 | 0.808 | 0.96038 | 0.02 | | 0.5 | 2 | 0 | 2018 | 2019 |
| Rheu | C00 | RNF2 | 60 | Q99496 | ring finger protein 2 | | 63 | 0. | 0. | 0.9 | 0.02 | | 1 | 2 | 0 | 2009 | 20 |

| | | | | | | | | | | | | | | | | | |
|---|---|---|---|---|---|---|---|---|---|---|---|---|---|---|---|---|---|
| matoid Arthritis | 03873 | | 45 | | | | | 608 | 615 | 9743 | | | | | | | 18 |
| Rheumatoid Arthritis | C0003873 | IGAN1 | 60498 | | IgA nephropathy | | 140 | 0.524 | 0.808 | | 0.02 | | 1 | 2 | 0 | 1994 | 2019 |
| Rheumatoid Arthritis | C0003873 | RORC | 6097 | P51449 | RAR related orphan receptor C | Nuclear receptor | 88 | 0.576 | 0.769 | 0.99985 | 0.02 | | 1 | 2 | 0 | 2016 | 2019 |
| Rheumatoid Arthritis | C0003873 | RPL17 | 6139 | P18621 | ribosomal protein L17 | | 104 | 0.559 | 0.731 | 0.92412 | 0.02 | | 1 | 2 | 0 | 2004 | 2017 |
| Rheumatoid Arthritis | C0003873 | SAA3P | 6290 | | serum amyloid A3, pseudogene | | 23 | 0.722 | 0.5 | | 0.02 | | 1 | 2 | 0 | 2001 | 2011 |
| Rheumatoid Arthritis | C0003873 | SAT1 | 6303 | P21673 | spermidine/spermine N1-acetyltransferase 1 | | 135 | 0.539 | 0.808 | 0.8706 | 0.02 | | 1 | 2 | 0 | 2000 | 2014 |
| Rheumatoid Arthritis | C0003873 | CCL4 | 6351 | P13236;Q8NHW4 | C-C motif chemokine ligand 4 | Signaling | 276 | 0.466 | 0.923 | 0.24134 | 0.02 | | 1 | 2 | 1 | 1994 | 1995 |

| | | | | | | | | | | | | | | | | |
|---|---|---|---|---|---|---|---|---|---|---|---|---|---|---|---|---|
| Rheumatoid Arthritis | C0003873 | CCL17 | 6361 | Q92583 | C-C motif chemokine ligand 17 | Signaling | 162 | 0.518 | 0.808 | 0.000139 | 0.02 | | 1 | 2 | 0 | 2001 | 2017 |
| Rheumatoid Arthritis | C0003873 | CCL19 | 6363 | Q99731 | C-C motif chemokine ligand 19 | Signaling | 126 | 0.542 | 0.692 | 0.31691 | 0.02 | | 1 | 2 | 0 | 2017 | 2017 |
| Rheumatoid Arthritis | C0003873 | CCL22 | 6367 | O00626 | C-C motif chemokine ligand 22 | Signaling | 182 | 0.5 | 0.846 | 0.008401 | 0.02 | | 1 | 2 | 0 | 2010 | 2017 |
| Rheumatoid Arthritis | C0003873 | CCL25 | 6370 | O15444 | C-C motif chemokine ligand 25 | Signaling | 61 | 0.61 | 0.692 | 0.015241 | 0.02 | | 1 | 2 | 0 | 2010 | 2014 |
| Rheumatoid Arthritis | C0003873 | SDC2 | 6383 | P34741 | syndecan 2 | Extracellular structure | 120 | 0.536 | 0.808 | 0.4049 | 0.02 | | 1 | 2 | 0 | 2005 | 2012 |
| Rheumatoid Arthritis | C0003873 | TINAGL1 | 64129 | Q9GZM7 | tubulointerstitial nephritis antigen like 1 | Enzyme | 38 | 0.659 | 0.538 | 0.000329 | 0.02 | | 1 | 2 | 0 | 2012 | 2013 |
| Rheumatoid Ar | C0003873 | SEMA4A | 64218 | Q9H3S1 | semaphorin 4A | Signaling | 141 | 0.569 | 0.692 | 1.84E- | 0.02 | | 1 | 2 | 0 | 2015 | 2015 |

| | | | | | | | | | | | | | | | | | |
|---|---|---|---|---|---|---|---|---|---|---|---|---|---|---|---|---|---|
| thritis | | | | | | | | | | 05 | | | | | | | |
| Rheumatoid Arthritis | C0003873 | CLEC7A | 64581 | Q9BXN2 | C-type lectin domain containing 7A | | 126 | 0.553 | 0.769 | 5.51E-12 | 0.02 | | 1 | 2 | 1 | 2010 | 2013 |
| Rheumatoid Arthritis | C0003873 | AGXT2 | 64902 | Q9BYV1 | alanine--glyoxylate aminotransferase 2 | | 25 | 0.729 | 0.385 | 3.71E-28 | 0.02 | | 1 | 2 | 0 | 2017 | 2019 |
| Rheumatoid Arthritis | C0003873 | SLC2A3 | 6515 | P11169 | solute carrier family 2 member 3 | Transporter | 144 | 0.534 | 0.769 | 0.010333 | 0.02 | | 1 | 2 | 0 | 2014 | 2019 |
| Rheumatoid Arthritis | C0003873 | SLC6A2 | 6530 | P23975 | solute carrier family 6 member 2 | Transporter | 238 | 0.49 | 0.846 | 0.081524 | 0.02 | | 1 | 2 | 0 | 2019 | 2020 |
| Rheumatoid Arthritis | C0003873 | SUMO3 | 6612 | P55854 | small ubiquitin like modifier 3 | | 30 | 0.686 | 0.538 | 0.32841 | 0.02 | | 1 | 2 | 0 | 2013 | 2019 |
| Rheumatoid Arthritis | C0003873 | SUMO2 | 6613 | P61956 | small ubiquitin like modifier 2 | | 42 | 0.666 | 0.615 | 0.86943 | 0.02 | | 1 | 2 | 0 | 2013 | 2019 |
| Rheumatoi | C0003 | SNCA | 6622 | P37840 | synuclein alpha | Transporter | 449 | 0.4 | 0.8 | 0.88 | 0.02 | | 1 | 2 | 0 | 2004 | 2017 |

| | | | | | | | | | | | | | | | | | |
|---|---|---|---|---|---|---|---|---|---|---|---|---|---|---|---|---|---|
| d Arthritis | 873 | | | | | | | 27 | 85 | 29 | | | | | | | |
| Rheumatoid Arthritis | C0003873 | SOD1 | 6647 | P00441 | superoxide dismutase 1 | Enzyme | 689 | 0.379 | 0.962 | 0.1773 | 0.02 | | 1 | 2 | 0 | 2008 | 2008 |
| Rheumatoid Arthritis | C0003873 | SOD3 | 6649 | P08294 | superoxide dismutase 3 | Enzyme | 138 | 0.531 | 0.808 | 0.000723 | 0.02 | | 1 | 2 | 0 | 2001 | 2009 |
| Rheumatoid Arthritis | C0003873 | SPARC | 6678 | P09486 | secreted protein acidic and cysteine rich | Signaling | 344 | 0.445 | 0.846 | 0.89148 | 0.02 | | 1 | 2 | 0 | 2015 | 2019 |
| Rheumatoid Arthritis | C0003873 | SSRP1 | 6749 | Q08945 | structure specific recognition protein 1 | | 86 | 0.575 | 0.731 | 0.99585 | 0.02 | | 1 | 2 | 0 | 2003 | 2017 |
| Rheumatoid Arthritis | C0003873 | SSTR4 | 6754 | P31391 | somatostatin receptor 4 | G-protein coupled receptor | 273 | 0.469 | 0.846 | 3.7E-13 | 0.02 | | 1 | 2 | 0 | 1999 | 2004 |
| Rheumatoid Arthritis | C0003873 | STAT5A | 6776 | P42229 | signal transducer and activator of transcription 5A | Nucleic acid binding | 318 | 0.445 | 0.808 | 0.99994 | 0.02 | | 1 | 2 | 0 | 2012 | 2019 |

| | | | | | | | | | | | | | | | | | |
|---|---|---|---|---|---|---|---|---|---|---|---|---|---|---|---|---|---|
| Rheumatoid Arthritis | C0003873 | STAT6 | 6778 | P42226 | signal transducer and activator of transcription 6 | Nucleic acid binding | 281 | 0.475 | 0.808 | 0.98849 | 0.02 | | 1 | 2 | 0 | 2000 | 2010 |
| Rheumatoid Arthritis | C0003873 | BRS3 | 680 | P32247 | bombesin receptor subtype 3 | G-protein coupled receptor | 261 | 0.474 | 0.846 | 0.94849 | 0.02 | | 1 | 2 | 0 | 1999 | 2004 |
| Rheumatoid Arthritis | C0003873 | CNTN2 | 6900 | Q02246 | contactin 2 | Receptor | 99 | 0.581 | 0.692 | 0.00211 | 0.02 | | 1 | 2 | 0 | 1995 | 2003 |
| Rheumatoid Arthritis | C0003873 | TBCA | 6902 | O75347 | tubulin folding cofactor A | Chaperone | 43 | 0.682 | 0.538 | 0.16004 | 0.02 | | 1 | 2 | 0 | 2018 | 2019 |
| Rheumatoid Arthritis | C0003873 | GCFC2 | 6936 | P16383 | GC-rich sequence DNA-binding factor 2 | Nucleic acid binding | 21 | 0.722 | 0.462 | 2.56E-24 | 0.02 | | 1 | 2 | 0 | 2019 | 2019 |
| Rheumatoid Arthritis | C0003873 | TG | 7038 | P01266 | thyroglobulin | Enzyme modulator | 240 | 0.48 | 0.808 | 2.88E-59 | 0.02 | | 1 | 2 | 1 | 2018 | 2018 |
| Rheumatoi | C0003 | TGFA | 7039 | P01135 | transforming growth | Signaling | 376 | 0.4 | 0.8 | 0.719 | 0.02 | | 1 | 2 | 0 | 1993 | 2005 |

| | | | | | | | | | | | | | | | | | |
|---|---|---|---|---|---|---|---|---|---|---|---|---|---|---|---|---|---|
| d Arthritis | 873 | | | | factor alpha | | | 32 | 85 | 81 | | | | | | | |
| Rheumatoid Arthritis | C0003873 | TGM2 | 7052 | P21980 | transglutaminase 2 | Enzyme | 315 | 0.445 | 0.808 | 6.81E-18 | 0.02 | | 1 | 2 | 0 | 2007 | 2013 |
| Rheumatoid Arthritis | C0003873 | THBD | 7056 | P07204 | thrombomodulin | | 303 | 0.457 | 0.769 | 0.0537769 | 0.02 | | 1 | 2 | 0 | 2012 | 2018 |
| Rheumatoid Arthritis | C0003873 | THOP1 | 7064 | P52888 | thimet oligopeptidase 1 | Enzyme | 91 | 0.573 | 0.846 | 0.000121 | 0.02 | | 1 | 2 | 0 | 2017 | 2017 |
| Rheumatoid Arthritis | C0003873 | THY1 | 7070 | P04216 | Thy-1 cell surface antigen | | 197 | 0.496 | 0.808 | 0.049895 | 0.02 | | 1 | 2 | 0 | 2017 | 2019 |
| Rheumatoid Arthritis | C0003873 | TIA1 | 7072 | P31483 | TIA1 cytotoxic granule associated RNA binding protein | | 96 | 0.595 | 0.577 | 0.27493 | 0.02 | | 1 | 2 | 0 | 2006 | 2007 |
| Rheumatoid Arthritis | C0003873 | TLR1 | 7096 | Q15399 | toll like receptor 1 | | 198 | 0.496 | 0.808 | 5.87E-23 | 0.02 | | 1 | 2 | 1 | 2011 | 2017 |
| Rheu | C00 | TTR | 72 | P02766 | transthyretin | Trans | 461 | 0. | 0. | 0.5 | 0.02 | | 1 | 2 | 0 | 2015 | 20 |

| | | | | | | | | | | | | | | | | | |
|---|---|---|---|---|---|---|---|---|---|---|---|---|---|---|---|---|---|
| matoid Arthritis | 03873 | | 76 | | | porter | | 423 | 885 | 1566 | | | | | | | 19 |
| Rheumatoid Arthritis | C0003873 | POTEF | 728378 | A5A3E0 | POTE ankyrin domain family member F | | 215 | 0.481 | 0.885 | 5.45E-06 | 0.02 | | 1 | 2 | 0 | 2018 | 2019 |
| Rheumatoid Arthritis | C0003873 | TNFRSF4 | 7293 | P43489 | TNF receptor superfamily member 4 | | 123 | 0.547 | 0.692 | 0.000151 | 0.02 | | 1 | 2 | 0 | 2017 | 2019 |
| Rheumatoid Arthritis | C0003873 | TYROBP | 7305 | O43914 | transmembrane immune signaling adaptor TYROBP | | 135 | 0.573 | 0.769 | 0.20238 | 0.02 | | 0.5 | 2 | 0 | 2014 | 2015 |
| Rheumatoid Arthritis | C0003873 | UQCRFS1 | 7386 | P47985 | ubiquinol-cytochrome c reductase, Rieske iron-sulfur polypeptide 1 | | 109 | 0.558 | 0.654 | 0.18923 | 0.02 | | 1 | 2 | 0 | 1994 | 1996 |
| Rheumatoid Arthritis | C0003873 | VPREB1 | 7441 | P12018 | V-set pre-B cell surrogate light chain 1 | Immune response | 28 | 0.695 | 0.5 | 0.096491 | 0.02 | | 1 | 2 | 0 | 2011 | 2014 |
| Rheumatoid Arthritis | C0003873 | WARS1 | 7453 | P23381 | tryptophanyl-tRNA synthetase 1 | Enzyme | 60 | 0.65 | 0.654 | 0.30288 | 0.02 | | 1 | 2 | 0 | 2015 | 2019 |

| | | | | | | | | | | | | | | | | | |
|---|---|---|---|---|---|---|---|---|---|---|---|---|---|---|---|---|---|
| Rheumatoid Arthritis | C0003873 | WNT3 | 7473 | P56703 | Wnt family member 3 | Signaling | 140 | 0.536 | 0.808 | 0.87515 | 0.02 | | 0.5 | 2 | 0 | 2009 | 2018 |
| Rheumatoid Arthritis | C0003873 | WNT10B | 7480 | O00744 | Wnt family member 10B | Signaling | 90 | 0.576 | 0.654 | 0.000362 | 0.02 | | 1 | 2 | 0 | 2006 | 2014 |
| Rheumatoid Arthritis | C0003873 | YWHAZ | 7534 | P63104 | tyrosine 3-monooxygenase/tryptophan 5-monooxygenase activation protein zeta | Chaperone | 247 | 0.475 | 0.846 | 0.939965 | 0.02 | | 1 | 2 | 0 | 2004 | 2011 |
| Rheumatoid Arthritis | C0003873 | CA1 | 759 | P00915 | carbonic anhydrase 1 | | 66 | 0.623 | 0.769 | 4.75E-11 | 0.02 | | 1 | 2 | 1 | 2001 | 2018 |
| Rheumatoid Arthritis | C0003873 | IL1R2 | 7850 | P27930 | interleukin 1 receptor type 2 | Receptor | 76 | 0.593 | 0.846 | 6.54E-15 | 0.02 | | 1 | 2 | 0 | 2012 | 2018 |
| Rheumatoid Arthritis | C0003873 | IRX1 | 79192 | P78414 | iroquois homeobox 1 | Transcription factor | 28 | 0.695 | 0.538 | 0.000477 | 0.02 | | 1 | 2 | 0 | 2013 | 2016 |
| Rheuma | C0003 | ADIPOR2 | 796 | Q86V24 | adiponectin | Rece | 103 | 0.5 | 0.7 | 0.36 | 0.02 | | 1 | 2 | 1 | 2009 | 20 |

| | | | | | | | | | | | | | | | | | |
|---|---|---|---|---|---|---|---|---|---|---|---|---|---|---|---|---|---|
| toid Arthritis | 873 | | 02 | | receptor 2 | ptor | | 64 | 31 | 218 | | | | | | | 11 |
| Rheumatoid Arthritis | C0003873 | RHBDF2 | 79651 | Q6PJF5 | rhomboid 5 homolog 2 | Enzyme | 90 | 0.595 | 0.538 | 0.0011192 | 0.02 | | 1 | 2 | 0 | 2018 | 2019 |
| Rheumatoid Arthritis | C0003873 | ASRGL1 | 80150 | Q7L266 | asparaginase and isoaspartyl peptidase 1 | Enzyme | 205 | 0.494 | 0.808 | 2.76E-05 | 0.02 | | 1 | 2 | 0 | 2003 | 2019 |
| Rheumatoid Arthritis | C0003873 | TAM | 8205 | | Myeloproliferative syndrome, transient (transient abnormal | | 141 | 0.528 | 0.731 | | 0.02 | | 1 | 2 | 0 | 2017 | 2018 |
| Rheumatoid Arthritis | C0003873 | CAPG | 822 | P40121 | capping actin protein, gelsolin like | Cellular structure | 111 | 0.548 | 0.731 | 9.36E-14 | 0.02 | | 1 | 2 | 0 | 2018 | 2019 |
| Rheumatoid Arthritis | C0003873 | FCRL4 | 83417 | Q96PJ5 | Fc receptor like 4 | Cell adhesion | 35 | 0.67 | 0.5 | 7.91E-10 | 0.02 | | 1 | 2 | 0 | 2015 | 2017 |
| Rheumatoid Arthritis | C0003873 | JAM3 | 83700 | Q9BX67 | junctional adhesion molecule 3 | | 66 | 0.619 | 0.654 | 0.0006616 | 0.02 | | 1 | 2 | 0 | 2007 | 2008 |

| | | | | | | | | | | | | | | | | | |
|---|---|---|---|---|---|---|---|---|---|---|---|---|---|---|---|---|---|
| Rheumatoid Arthritis | C0003873 | LOH19CR1 | 8378 | | loss of heterozygosity, 19, chromosomal region 1 | | 96 | 0.569 | 0.808 | | 0.02 | | 1 | 2 | 0 | 2017 | 2019 |
| Rheumatoid Arthritis | C0003873 | CASP5 | 838 | P51878 | caspase 5 | Enzyme | 47 | 0.653 | 0.5 | 1.75E-16 | 0.02 | | 0.5 | 2 | 2 | 2017 | 2018 |
| Rheumatoid Arthritis | C0003873 | ROPN1L | 83853 | Q96C74 | rhophilin associated tail protein 1 like | | 62 | 0.617 | 0.692 | 2.75E-06 | 0.02 | | 1 | 2 | 0 | 2004 | 2018 |
| Rheumatoid Arthritis | C0003873 | CASP7 | 840 | P55210 | caspase 7 | Enzyme | 83 | 0.581 | 0.654 | 5.36E-05 | 0.02 | | 0.5 | 2 | 1 | 2007 | 2008 |
| Rheumatoid Arthritis | C0003873 | NLRC5 | 84166 | Q86WI3 | NLR family CARD domain containing 5 | Enzyme | 47 | 0.653 | 0.5 | 9.42E-08 | 0.02 | | 1 | 2 | 0 | 2017 | 2019 |
| Rheumatoid Arthritis | C0003873 | MINDY4 | 84182 | Q4G0A6 | MINDY lysine 48 deubiquitinase 4 | | 122 | 0.538 | 0.731 | 3.22E-19 | 0.02 | | 1 | 2 | 0 | 2013 | 2018 |
| Rheumatoid Ar | C0003873 | CUL1 | 8454 | Q13616 | cullin 1 | Enzyme | 58 | 0.617 | 0.615 | 1 | 0.02 | | 1 | 2 | 0 | 2005 | 2011 |

| | | | | | | | | | | | | | | | | | |
|---|---|---|---|---|---|---|---|---|---|---|---|---|---|---|---|---|---|
| thritis | | | | | | | | | | | | | | | | | |
| Rheumatoid Arthritis | C0003873 | IL17RC | 84818 | Q8NAC3 | interleukin 17 receptor C | | 65 | 0.633 | 0.615 | 2.3E-14 | 0.02 | | 0.5 | 2 | 0 | 2013 | 2018 |
| Rheumatoid Arthritis | C0003873 | SEMA7A | 8482 | O75326 | semaphorin 7A (John Milton Hagen blood group) | Signaling | 53 | 0.636 | 0.615 | 0.17976 | 0.02 | | 1 | 2 | 0 | 2017 | 2020 |
| Rheumatoid Arthritis | C0003873 | UBASH3B | 84959 | Q8TF42 | ubiquitin associated and SH3 domain containing B | | 104 | 0.561 | 0.769 | 4.71E-05 | 0.02 | | 1 | 2 | 0 | 1998 | 2017 |
| Rheumatoid Arthritis | C0003873 | CAV1 | 857 | Q03135 | caveolin 1 | Enzyme modulator | 633 | 0.388 | 0.885 | 0.008274 | 0.02 | | 1 | 2 | 0 | 2017 | 2018 |
| Rheumatoid Arthritis | C0003873 | SERPINH1 | 871 | P50454 | serpin family H member 1 | Enzyme modulator | 148 | 0.545 | 0.769 | 0.027553 | 0.02 | | 1 | 2 | 0 | 1998 | 2005 |
| Rheumatoid Arthritis | C0003873 | TNFSF12 | 8742 | O43508 | TNF superfamily member 12 | | 123 | 0.556 | 0.769 | 0.76997 | 0.02 | | 1 | 2 | 0 | 2007 | 2012 |
| Rheumatoi | C0003 | TNFRSF10B | 8795 | O14763 | TNF receptor superfamily member 10b | | 179 | 0.5 | 0.7 | 0.000 | 0.02 | | 1 | 2 | 0 | 2003 | 2005 |

| | | | | | | | | | | | | | | | | | |
|---|---|---|---|---|---|---|---|---|---|---|---|---|---|---|---|---|---|
| d Arthritis | 873 | | | | | | | 05 | 69 | 199 | | | | | | | |
| Rheumatoid Arthritis | C0003873 | IL18RAP | 8807 | O95256 | interleukin 18 receptor accessory protein | Receptor | 41 | 0.674 | 0.577 | 0.00218 | 0.02 | | 1 | 2 | 0 | 2003 | 2017 |
| Rheumatoid Arthritis | C0003873 | NRP1 | 8829 | O14786 | neuropilin 1 | | 246 | 0.476 | 0.808 | 0.98584 | 0.02 | | 1 | 2 | 0 | 2006 | 2012 |
| Rheumatoid Arthritis | C0003873 | CCN6 | 8838 | O95389 | cellular communication network factor 6 | Signaling | 126 | 0.568 | 0.731 | 0.00619 | 0.02 | | 1 | 2 | 0 | 2004 | 2015 |
| Rheumatoid Arthritis | C0003873 | SPHK1 | 8877 | Q9NYA1 | sphingosine kinase 1 | Kinase | 236 | 0.48 | 0.808 | 3.03E-07 | 0.02 | | 1 | 2 | 0 | 2014 | 2019 |
| Rheumatoid Arthritis | C0003873 | MBD4 | 8930 | O95243 | methyl-CpG binding domain 4, DNA glycosylase | Enzyme | 62 | 0.61 | 0.615 | 1.97E-14 | 0.02 | | 0.5 | 2 | 2 | 2012 | 2012 |
| Rheumatoid Arthritis | C0003873 | MBD2 | 8932 | Q9UBB5 | methyl-CpG binding domain protein 2 | Epigenetic regulator | 256 | 0.466 | 0.846 | 0.34957 | 0.02 | | 1 | 2 | 0 | 2018 | 2019 |

| | | | | | | | | | | | | | | | | | |
|---|---|---|---|---|---|---|---|---|---|---|---|---|---|---|---|---|---|
| Rheumatoid Arthritis | C0003873 | PGLYRP1 | 8993 | O75594 | peptidoglycan recognition protein 1 | | 20 | 0.78 | 0.346 | 1.79E-05 | 0.02 | | 1 | 2 | 1 | 2015 | 2019 |
| Rheumatoid Arthritis | C0003873 | PSTPIP1 | 9051 | O43586 | proline-serine-threonine phosphatase interacting protein 1 | Cellular structure | 54 | 0.65 | 0.577 | 2.46E-06 | 0.02 | | 1 | 2 | 0 | 2002 | 2016 |
| Rheumatoid Arthritis | C0003873 | CD1D | 912 | P15813 | CD1d molecule | | 140 | 0.532 | 0.769 | 1.88E-06 | 0.02 | | 1 | 2 | 0 | 2003 | 2009 |
| Rheumatoid Arthritis | C0003873 | PDCD5 | 9141 | O14737 | programmed cell death 5 | Enzyme modulator | 67 | 0.61 | 0.538 | 0.003642 | 0.02 | | 1 | 2 | 0 | 2007 | 2013 |
| Rheumatoid Arthritis | C0003873 | INTS4 | 92105 | Q96HW7 | integrator complex subunit 4 | | 23 | 0.705 | 0.538 | 1.13E-11 | 0.02 | | 0.5 | 2 | 0 | 2009 | 2018 |
| Rheumatoid Arthritis | C0003873 | CD8A | 925 | P01732 | CD8a molecule | | 87 | 0.587 | 0.769 | 1.68E-05 | 0.02 | | 0.5 | 2 | 0 | 1992 | 1992 |
| Rheumatoid | C0003873 | DNER | 92737 | Q8NFT8 | delta/notch like EGF repeat containing | | 203 | 0.493 | 0.769 | 0.1859 | 0.02 | | 1 | 2 | 0 | 2016 | 2019 |

| | | | | | | | | | | | | | | | | | |
|---|---|---|---|---|---|---|---|---|---|---|---|---|---|---|---|---|---|
| Arthritis | | | | | | | | | | | | | | | | | |
| Rheumatoid Arthritis | C0003873 | KLF4 | 9314 | O43474 | Kruppel like factor 4 | Nucleic acid binding | 307 | 0.449 | 0.769 | 0.99714 | 0.02 | | 1 | 2 | 0 | 2016 | 2018 |
| Rheumatoid Arthritis | C0003873 | CD22 | 933 | P20273 | CD22 molecule | | 106 | 0.556 | 0.615 | 0.80044 | 0.02 | | 1 | 2 | 0 | 1999 | 2019 |
| Rheumatoid Arthritis | C0003873 | TNFRSF8 | 943 | P28908 | TNF receptor superfamily member 8 | | 301 | 0.444 | 0.808 | 0.86407 | 0.02 | | 1 | 2 | 0 | 2000 | 2003 |
| Rheumatoid Arthritis | C0003873 | ADAMTS3 | 9508 | O15072 | ADAM metallopeptidase with thrombospondin type 1 motif 3 | Enzyme | 116 | 0.581 | 0.808 | 1.03E-10 | 0.02 | | 1 | 2 | 0 | 2012 | 2015 |
| Rheumatoid Arthritis | C0003873 | TBPL1 | 9519 | P62380 | TATA-box binding protein like 1 | Transcription factor | 198 | 0.506 | 0.846 | 0.37902 | 0.02 | | 0.5 | 2 | 0 | 2000 | 2019 |
| Rheumatoid Arthritis | C0003873 | CCL4L2 | 9560 | P13236;Q8NHW4 | C-C motif chemokine ligand 4 like 2 | | 133 | 0.535 | 0.885 | 0.088586 | 0.02 | | 1 | 2 | 0 | 1994 | 1995 |
| Rheuma | C0003 | CD48 | 962 | P09326 | CD48 molecule | | 74 | 0.5 | 0.6 | 1.73 | 0.02 | | 1 | 2 | 0 | 1990 | 19 |

| | | | | | | | | | | | | | | | | | |
|---|---|---|---|---|---|---|---|---|---|---|---|---|---|---|---|---|---|
| toid Arthritis | 873 | | | | | | | 92 | 92 | E-05 | | | | | | | 90 |
| Rheumatoid Arthritis | C0003873 | IKBKE | 9641 | Q14164 | inhibitor of nuclear factor kappa B kinase subunit epsilon | Kinase | 79 | 0.597 | 0.731 | 0.0090005 | 0.02 | | 1 | 2 | 0 | 2005 | 2017 |
| Rheumatoid Arthritis | C0003873 | SDC3 | 9672 | O75056 | syndecan 3 | Extracellular structure | 30 | 0.711 | 0.654 | 0.41455 | 0.02 | | 1 | 2 | 0 | 2005 | 2019 |
| Rheumatoid Arthritis | C0003873 | FAM53B | 9679 | Q14153 | family with sequence similarity 53 member B | | 28 | 0.716 | 0.423 | 0.85523 | 0.02 | | 1 | 2 | 0 | 2010 | 2017 |
| Rheumatoid Arthritis | C0003873 | CD70 | 970 | P32970 | CD70 molecule | | 127 | 0.542 | 0.692 | 0.010394 | 0.02 | | 1 | 2 | 0 | 2007 | 2016 |
| Rheumatoid Arthritis | C0003873 | HDAC9 | 9734 | Q9UKV0 | histone deacetylase 9 | Epigenetic regulator | 340 | 0.439 | 0.846 | 0.99973 | 0.02 | | 1 | 2 | 0 | 2012 | 2015 |
| Rheumatoid Arthritis | C0003873 | ADGRE5 | 976 | P48960 | adhesion G protein-coupled receptor E5 | G-protein coupled rec | 74 | 0.601 | 0.538 | 3.08E-06 | 0.02 | | 1 | 2 | 0 | 2000 | 2010 |

| | | | | | | | | | | | | | | | | | |
|---|---|---|---|---|---|---|---|---|---|---|---|---|---|---|---|---|---|
| | | | | | | eptor | | | | | | | | | | | |
| Rheumatoid Arthritis | C0003873 | PIEZO1 | 9780 | Q92508 | piezo type mechanosensitive ion channel component 1 | Ion channel | 130 | 0.552 | 0.769 | 3.94E-21 | 0.02 | | 1 | 2 | 0 | 2001 | 2002 |
| Rheumatoid Arthritis | C0003873 | KEAP1 | 9817 | Q14145 | kelch like ECH associated protein 1 | | 251 | 0.475 | 0.808 | 0.000388 | 0.02 | | 1 | 2 | 0 | 2018 | 2019 |
| Rheumatoid Arthritis | C0003873 | SPATA2 | 9825 | Q9UM82 | spermatogenesis associated 2 | | 107 | 0.556 | 0.615 | 0.97889 | 0.02 | | 1 | 2 | 0 | 2004 | 2017 |
| Rheumatoid Arthritis | C0003873 | CDKN2B-AS1 | 1E+08 | | CDKN2B antisense RNA 1 | | 213 | 0.491 | 0.769 | | 0.01 | | 1 | 1 | 3 | 2019 | 2019 |
| Rheumatoid Arthritis | C0003873 | TEC | 1E+08 | | transient erythroblastopenia of childhood | | 40 | 0.659 | 0.577 | | 0.01 | | 1 | 1 | 0 | 2017 | 2017 |
| Rheumatoid Arthritis | C0003873 | MIR708 | 1E+08 | | microRNA 708 | | 86 | 0.57 | 0.692 | | 0.01 | | 1 | 1 | 0 | 2018 | 2018 |
| Rheumatoi | C0003 | MIR887 | 1E+ | | microRNA 887 | | 6 | 0.8 | 0.2 | | 0.01 | | 1 | 1 | 0 | 2017 | 2017 |

| | | | | | | | | | | | | | | | | | |
|---|---|---|---|---|---|---|---|---|---|---|---|---|---|---|---|---|---|
| d Arthritis | 873 | | 08 | | | | | 39 | 31 | | | | | | | | |
| Rheumatoid Arthritis | C0003873 | C20orf181 | 1E+08 | | chromosome 20 open reading frame 181 | | 246 | 0.479 | 0.885 | | 0.01 | | 1 | 1 | 0 | 2019 | 2019 |
| Rheumatoid Arthritis | C0003873 | HDAC6 | 10013 | Q9UBN7 | histone deacetylase 6 | Epigenetic regulator | 288 | 0.465 | 0.846 | 1 | 0.01 | | 0 | 1 | 0 | 2016 | 2016 |
| Rheumatoid Arthritis | C0003873 | HDAC5 | 10014 | Q9UQL6 | histone deacetylase 5 | Epigenetic regulator | 114 | 0.552 | 0.769 | 1 | 0.01 | | 1 | 1 | 0 | 2016 | 2016 |
| Rheumatoid Arthritis | C0003873 | BCL2L11 | 10018 | O43521 | BCL2 like 11 | | 173 | 0.51 | 0.731 | 0.88857 | 0.01 | | 1 | 1 | 0 | 2015 | 2015 |
| Rheumatoid Arthritis | C0003873 | TRAP | 1E+08 | | triiodothyronine receptor auxiliary protein | | 80 | 0.579 | 0.731 | | 0.01 | | 1 | 1 | 0 | 2014 | 2014 |
| Rheumatoid Arthritis | C0003873 | MIR1246 | 1E+08 | | microRNA 1246 | | 76 | 0.592 | 0.808 | | 0.01 | | 1 | 1 | 0 | 2015 | 2015 |

| | | | | | | | | | | | | | | | | | |
|---|---|---|---|---|---|---|---|---|---|---|---|---|---|---|---|---|---|
| Rheumatoid Arthritis | C0003873 | MIR1908 | 1E+08 | | microRNA 1908 | | 37 | 0.67 | 0.577 | | 0.01 | | 1 | 1 | 0 | 2018 | 2018 |
| Rheumatoid Arthritis | C0003873 | TMED7-TICAM2 | 1E+08 | Q86XR7;Q9Y3B3 | TMED7-TICAM2 readthrough | | 329 | 0.438 | 0.769 | 0.002594 | 0.01 | | 1 | 1 | 0 | 2005 | 2005 |
| Rheumatoid Arthritis | C0003873 | MIR762 | 1E+08 | | microRNA 762 | | 13 | 0.769 | 0.423 | | 0.01 | | 1 | 1 | 0 | 2018 | 2018 |
| Rheumatoid Arthritis | C0003873 | AAA1 | 1E+08 | | aortic aneurysm, familial abdominal 1 | | 29 | 0.695 | 0.615 | | 0.01 | | 1 | 1 | 0 | 2016 | 2016 |
| Rheumatoid Arthritis | C0003873 | PARP2 | 10038 | Q9UGN5 | poly(ADP-ribose) polymerase 2 | | 40 | 0.663 | 0.538 | 2.33E-13 | 0.01 | | 1 | 1 | 0 | 2019 | 2019 |
| Rheumatoid Arthritis | C0003873 | DNAJB6 | 10049 | O75190 | DnaJ heat shock protein family (Hsp40) member B6 | | 65 | 0.659 | 0.462 | 0.85436 | 0.01 | | 1 | 1 | 0 | 1999 | 1999 |
| Rheumatoid Ar | C0003873 | SLC17A4 | 10050 | Q9Y2C5 | solute carrier family 17 member 4 | Transporter | 15 | 0.78 | 0.385 | 1.57E- | 0.01 | | 1 | 1 | 0 | 2017 | 2017 |

| | | | | | | | | | | | | | | | | |
|---|---|---|---|---|---|---|---|---|---|---|---|---|---|---|---|---|
| thritis | | | | | | | | | | 09 | | | | | | |
| Rheumatoid Arthritis | C0003873 | GAPLINC | 1.01E+08 | | gastric adenocarcinoma associated, positive CD44 regulator, long intergenic non-coding RNA | 20 | 0.736 | 0.269 | | 0.01 | | 1 | 1 | 0 | 2018 | 2018 |
| Rheumatoid Arthritis | C0003873 | LOC100505909 | 1.01E+08 | | histidine-rich glycoprotein | 17 | 0.722 | 0.385 | | 0.01 | | 1 | 1 | 0 | 2018 | 2018 |
| Rheumatoid Arthritis | C0003873 | LOC100506023 | 1.01E+08 | | uncharacterized LOC100506023 | 1 | 1 | 0.115 | | 0.01 | | 1 | 1 | 3 | 2016 | 2016 |
| Rheumatoid Arthritis | C0003873 | CNNM3-DT | 1.01E+08 | | CNNM3 divergent transcript | 1 | 1 | 0.115 | | 0.01 | | 1 | 1 | 0 | 2016 | 2016 |
| Rheumatoid Arthritis | C0003873 | OCLN | 1.01E+08 | Q16625 | occludin | 195 | 0.507 | 0.846 | 8.56E-07 | 0.01 | | 1 | 1 | 0 | 2018 | 2018 |
| Rheumatoid Arthritis | C0003873 | CASP12 | 1.01E+08 | Q6UXS9 | caspase 12 (gene/pseudogene) | 44 | 0.638 | 0.808 | 0.000127 | 0.01 | | 1 | 1 | 0 | 2014 | 2014 |
| Rheumatoi | C0003 | H3P43 | 1.01E | | H3 histone pseudogene 43 | 1 | 1 | 0.1 | | 0.01 | | 1 | 1 | 0 | 2017 | 2017 |

| | | | | | | | | | | | | | | | | | |
|---|---|---|---|---|---|---|---|---|---|---|---|---|---|---|---|---|---|
| d Arthritis | 873 | | +08 | | | | | | 15 | | | | | | | | |
| Rheumatoid Arthritis | C0003873 | P2RX5-TAX1BP3 | 1.01E+08 | | P2RX5-TAX1BP3 readthrough (NMD candidate) | | 91 | 0.588 | 0.769 | | 0.01 | | 1 | 1 | 0 | 2010 | 2010 |
| Rheumatoid Arthritis | C0003873 | DNM1L | 10059 | O00429 | dynamin 1 like | Enzyme modulator | 273 | 0.475 | 0.808 | 0.00568 | 0.01 | | 1 | 1 | 0 | 2020 | 2020 |
| Rheumatoid Arthritis | C0003873 | MIR4701 | 1.01E+08 | | microRNA 4701 | | 2 | 0.931 | 0.192 | | 0.01 | | 1 | 1 | 0 | 2019 | 2019 |
| Rheumatoid Arthritis | C0003873 | MIR4764 | 1.01E+08 | | microRNA 4764 | | 1 | 1 | 0.115 | | 0.01 | | 1 | 1 | 0 | 2015 | 2015 |
| Rheumatoid Arthritis | C0003873 | PSC | 1.01E+08 | | Cholangitis, primary sclerosing | | 67 | 0.603 | 0.731 | | 0.01 | | 1 | 1 | 0 | 1997 | 1997 |
| Rheumatoid Arthritis | C0003873 | PTPRU | 10076 | Q92729 | protein tyrosine phosphatase receptor type U | Enzyme | 88 | 0.581 | 0.654 | 0.39251 | 0.01 | | 1 | 1 | 0 | 2017 | 2017 |

| | | | | | | | | | | | | | | | | | |
|---|---|---|---|---|---|---|---|---|---|---|---|---|---|---|---|---|---|
| Rheumatoid Arthritis | C0003873 | MIR5571 | 1.01E+08 | | microRNA 5571 | | 3 | 0.89 | 0.154 | | 0.01 | | 1 | 1 | 0 | 2019 | 2019 |
| Rheumatoid Arthritis | C0003873 | MIR5100 | 1.01E+08 | | microRNA 5100 | | 16 | 0.769 | 0.269 | | 0.01 | | 1 | 1 | 0 | 2018 | 2018 |
| Rheumatoid Arthritis | C0003873 | G3BP1 | 10146 | Q13283 | G3BP stress granule assembly factor 1 | Nucleic acid binding | 78 | 0.597 | 0.654 | 0.033122 | 0.01 | | 1 | 1 | 0 | 2003 | 2003 |
| Rheumatoid Arthritis | C0003873 | CHST4 | 10164 | Q8NCG5 | carbohydrate sulfotransferase 4 | | 12 | 0.792 | 0.269 | 2E-09 | 0.01 | | 1 | 1 | 0 | 2005 | 2005 |
| Rheumatoid Arthritis | C0003873 | CDK4 | 1019 | P11802 | cyclin dependent kinase 4 | Kinase | 433 | 0.412 | 0.808 | 0.062909 | 0.01 | | 1 | 1 | 0 | 2006 | 2006 |
| Rheumatoid Arthritis | C0003873 | CDK7 | 1022 | P50613 | cyclin dependent kinase 7 | Kinase | 89 | 0.566 | 0.731 | 4.47E-07 | 0.01 | | 1 | 1 | 0 | 2018 | 2018 |
| Rheumatoid Ar | C0003873 | GDF11 | 10220 | O95390 | growth differentiation factor 11 | Signaling | 86 | 0.584 | 0.692 | 0.97645 | 0.01 | | 1 | 1 | 0 | 2019 | 2019 |

| | | | | | | | | | | | | | | | | | |
|---|---|---|---|---|---|---|---|---|---|---|---|---|---|---|---|---|---|
| thritis | | | | | | | | | | | | | | | | | |
| Rheumatoid Arthritis | C0003873 | RASGRP2 | 10235 | Q7LDG7 | RAS guanyl releasing protein 2 | Enzyme modulator | 18 | 0.769 | 0.231 | 0.2436 | 0.01 | | 1 | 1 | 0 | 2018 | 2018 |
| Rheumatoid Arthritis | C0003873 | SLC17A2 | 10246 | O00624 | solute carrier family 17 member 2 | Transporter | 13 | 0.805 | 0.269 | 1.42E-06 | 0.01 | | 1 | 1 | 0 | 2018 | 2018 |
| Rheumatoid Arthritis | C0003873 | MIR6089 | 1.02E+08 | | microRNA 6089 | | 2 | 0.931 | 0.192 | | 0.01 | | 1 | 1 | 0 | 2019 | 2019 |
| Rheumatoid Arthritis | C0003873 | MIR6716 | 1.02E+08 | | microRNA 6716 | | 6 | 0.861 | 0.192 | | 0.01 | | 0 | 1 | 0 | 2017 | 2017 |
| Rheumatoid Arthritis | C0003873 | MIR6785 | 1.02E+08 | | microRNA 6785 | | 2 | 0.931 | 0.154 | | 0.01 | | 1 | 1 | 0 | 2019 | 2019 |
| Rheumatoid Arthritis | C0003873 | CDK2AP2 | 10263 | O75956 | cyclin dependent kinase 2 associated protein 2 | Enzyme modulator | 117 | 0.544 | 0.808 | 0.78801 | 0.01 | | 1 | 1 | 0 | 1990 | 1990 |
| Rheumatoi | C0003 | IRX5 | 102 | P78411 | iroquois homeobox 5 | Transcripti | 63 | 0.6 | 0.8 | 0.97 | 0.01 | | 1 | 1 | 0 | 2019 | 2019 |

| | | | | | | | | | | | | | | | | | |
|---|---|---|---|---|---|---|---|---|---|---|---|---|---|---|---|---|---|
| d Arthritis | 873 | | 65 | | | on factor | | 23 | 46 | 47 | | | | | | | |
| Rheumatoid Arthritis | C0003873 | THRIL | 1.03E+08 | | TNF and HNRNPL related immunoregulatory long non-coding RNA | | 16 | 0.743 | 0.5 | | 0.01 | | 1 | 1 | 0 | 2019 | 2019 |
| Rheumatoid Arthritis | C0003873 | CDKN1B | 1027 | P46527 | cyclin dependent kinase inhibitor 1B | | 454 | 0.409 | 0.769 | 0.623776 | 0.01 | | 1 | 1 | 0 | 2015 | 2015 |
| Rheumatoid Arthritis | C0003873 | FSTL3 | 10272 | O95633 | follistatin like 3 | Enzyme modulator | 33 | 0.695 | 0.423 | 0.686658 | 0.01 | | 0 | 1 | 0 | 2005 | 2005 |
| Rheumatoid Arthritis | C0003873 | LOC102724334 | 1.03E+08 | P57053 | histone H2B type F-S-like | | 22 | 0.711 | 0.615 | | 0.01 | | 1 | 1 | 0 | 2019 | 2019 |
| Rheumatoid Arthritis | C0003873 | LOC102725035 | 1.03E+08 | O75022 | leukocyte immunoglobulin-like receptor subfamily B member 3 | | 6 | 0.839 | 0.231 | | 0.01 | | 1 | 1 | 0 | 2016 | 2016 |
| Rheumatoid Arthritis | C0003873 | LILRB2 | 10288 | | leukocyte immunoglobulin like receptor B2 | | 56 | 0.623 | 0.615 | 1.28E-14 | 0.01 | | 1 | 1 | 0 | 2002 | 2002 |

| | | | | | | | | | | | | | | | | | |
|---|---|---|---|---|---|---|---|---|---|---|---|---|---|---|---|---|---|
| Rheumatoid Arthritis | C0003873 | DNAJA2 | 10294 | O60884 | DnaJ heat shock protein family (Hsp40) member A2 | | 10 | 0.839 | 0.308 | 0.99796 | 0.01 | | 1 | 1 | 0 | 1999 | 1999 |
| Rheumatoid Arthritis | C0003873 | ADAR | 103 | P55265 | adenosine deaminase RNA specific | Enzyme | 256 | 0.501 | 0.808 | 0.026282 | 0.01 | | 1 | 1 | 0 | 2020 | 2020 |
| Rheumatoid Arthritis | C0003873 | CDKN2C | 1031 | P42773 | cyclin dependent kinase inhibitor 2C | | 149 | 0.527 | 0.654 | 0.39526 | 0.01 | | 1 | 1 | 0 | 2006 | 2006 |
| Rheumatoid Arthritis | C0003873 | CRISP3 | 10321 | P54108 | cysteine rich secretory protein 3 | Immune response | 31 | 0.716 | 0.577 | 2.57E-06 | 0.01 | | 1 | 1 | 0 | 2019 | 2019 |
| Rheumatoid Arthritis | C0003873 | CDKN3 | 1033 | Q16667 | cyclin dependent kinase inhibitor 3 | | 150 | 0.514 | 0.769 | 0.001946 | 0.01 | | 1 | 1 | 0 | 2006 | 2006 |
| Rheumatoid Arthritis | C0003873 | CDR1-AS | 1.04E+08 | | CDR1 antisense RNA | | 51 | 0.623 | 0.654 | | 0.01 | | 1 | 1 | 0 | 2019 | 2019 |
| Rheumatoid Ar | C0003873 | CPQ | 10404 | Q9Y646 | carboxypeptidase Q | Enzyme | 87 | 0.579 | 0.769 | 1.32E- | 0.01 | | 1 | 1 | 0 | 2002 | 2002 |

| | | | | | | | | | | | | | | | | | |
|---|---|---|---|---|---|---|---|---|---|---|---|---|---|---|---|---|---|
| thritis | | | | | | | | | | 14 | | | | | | | |
| Rheumatoid Arthritis | C0003873 | SPAG11B | 10407 | Q08648;Q6PDA7 | sperm associated antigen 11B | | 44 | 0.65 | 0.731 | 0.0277751 | 0.01 | | 1 | 1 | 0 | 2009 | 2009 |
| Rheumatoid Arthritis | C0003873 | YAP1 | 10413 | P46937 | Yes associated protein 1 | Enzyme modulator | 374 | 0.432 | 0.808 | 0.99918 | 0.01 | | 1 | 1 | 0 | 2019 | 2019 |
| Rheumatoid Arthritis | C0003873 | PRMT5 | 10419 | O14744 | protein arginine methyltransferase 5 | Epigenetic regulator | 131 | 0.539 | 0.769 | 0.99999 | 0.01 | | 1 | 1 | 0 | 2017 | 2017 |
| Rheumatoid Arthritis | C0003873 | CFDP1 | 10428 | Q9UEE9 | craniofacial development protein 1 | | 71 | 0.619 | 0.615 | 0.003654 | 0.01 | | 1 | 1 | 0 | 2014 | 2014 |
| Rheumatoid Arthritis | C0003873 | TMEM147 | 10430 | Q9BVK8 | transmembrane protein 147 | Receptor | 4 | 0.861 | 0.192 | 0.00014 | 0.01 | | 1 | 1 | 0 | 2019 | 2019 |
| Rheumatoid Arthritis | C0003873 | MERTK | 10461 | Q12866 | MER proto-oncogene, tyrosine kinase | Kinase | 154 | 0.531 | 0.692 | 2.66E-11 | 0.01 | | 1 | 1 | 0 | 2018 | 2018 |
| Rheumatoi | C0003 | CLEC10A | 104 | Q8IUN9 | C-type lectin domain | Receptor | 64 | 0.6 | 0.6 | 7.76E | 0.01 | | 1 | 1 | 0 | 2006 | 2006 |

| | | | | | | | | | | | | | | | | | |
|---|---|---|---|---|---|---|---|---|---|---|---|---|---|---|---|---|---|
| d Arthritis | 873 | | 62 | | containing 10A | | | 06 | 92 | -06 | | | | | | | |
| Rheumatoid Arthritis | C0003873 | FST | 10468 | P19883 | follistatin | Enzyme modulator | 182 | 0.513 | 0.731 | 0.98014 | 0.01 | | 1 | 1 | 0 | 2019 | 2019 |
| Rheumatoid Arthritis | C0003873 | CREB3 | 10488 | O43889 | cAMP responsive element binding protein 3 | | 39 | 0.663 | 0.654 | 1.85E-05 | 0.01 | | 1 | 1 | 0 | 2006 | 2006 |
| Rheumatoid Arthritis | C0003873 | SEMA6B | 10501 | Q9H3T3 | semaphorin 6B | Signaling | 13 | 0.78 | 0.231 | 0.06227 | 0.01 | | 1 | 1 | 0 | 2018 | 2018 |
| Rheumatoid Arthritis | C0003873 | CEBPB | 1051 | P17676 | CCAAT enhancer binding protein beta | Transcription factor | 182 | 0.508 | 0.731 | 0.35809 | 0.01 | | 1 | 1 | 0 | 2015 | 2015 |
| Rheumatoid Arthritis | C0003873 | SEMA3C | 10512 | Q99985 | semaphorin 3C | Signaling | 71 | 0.619 | 0.731 | 0.00874 | 0.01 | | 1 | 1 | 0 | 2004 | 2004 |
| Rheumatoid Arthritis | C0003873 | MYBBP1A | 10514 | Q9BQG0 | MYB binding protein 1a | Enzyme | 31 | 0.695 | 0.615 | 5.23E-42 | 0.01 | | 1 | 1 | 0 | 2003 | 2003 |

| | | | | | | | | | | | | | | | | | |
|---|---|---|---|---|---|---|---|---|---|---|---|---|---|---|---|---|---|
| Rheumatoid Arthritis | C0003873 | CIB1 | 10519 | Q99828 | calcium and integrin binding 1 | | 275 | 0.458 | 0.769 | 0.00326 | 0.01 | | 1 | 1 | 0 | 2015 | 2015 |
| Rheumatoid Arthritis | C0003873 | ZNRD2 | 10534 | O60232 | zinc ribbon domain containing 2 | | 311 | 0.443 | 0.769 | 1.84E-07 | 0.01 | | 1 | 1 | 0 | 2005 | 2005 |
| Rheumatoid Arthritis | C0003873 | UPK3B | 1.05E+08 | Q9BT76 | uroplakin 3B | | 82 | 0.584 | 0.808 | 0.00234 | 0.01 | | 1 | 1 | 0 | 2015 | 2015 |
| Rheumatoid Arthritis | C0003873 | CERNA3 | 1.05E+08 | | competing endogenous lncRNA 3 for miR-645 | | 140 | 0.531 | 0.808 | | 0.01 | | 1 | 1 | 0 | 2011 | 2011 |
| Rheumatoid Arthritis | C0003873 | BATF | 10538 | Q16520 | basic leucine zipper ATF-like transcription factor | Transcription factor | 31 | 0.674 | 0.5 | 0.85462 | 0.01 | | 1 | 1 | 0 | 2014 | 2014 |
| Rheumatoid Arthritis | C0003873 | CECR | 1055 | | cat eye syndrome chromosome region | | 81 | 0.599 | 0.731 | | 0.01 | | 1 | 1 | 0 | 2019 | 2019 |
| Rheumatoid Ar | C0003873 | ARPC1A | 10552 | Q92747 | actin related protein 2/3 complex | Cellular structure | 15 | 0.78 | 0.308 | 0.73997 | 0.01 | | 1 | 1 | 1 | 2018 | 2018 |

| | | | | | | | | | | | | | | | | | |
|---|---|---|---|---|---|---|---|---|---|---|---|---|---|---|---|---|---|
| thritis | | | | | subunit 1A | | | | | | | | | | | | |
| Rheumatoid Arthritis | C0003873 | IFI44 | 10561 | Q8TCB0 | interferon induced protein 44 | | 38 | 0.678 | 0.538 | 1.94E-15 | 0.01 | | 1 | 1 | 0 | 2013 | 2013 |
| Rheumatoid Arthritis | C0003873 | NPC2 | 10577 | P61916 | NPC intracellular cholesterol transporter 2 | | 81 | 0.612 | 0.769 | 0.001776 | 0.01 | | 1 | 1 | 0 | 2011 | 2011 |
| Rheumatoid Arthritis | C0003873 | LOC106007493 | 1.06E+08 | | regulatory region in intron 4 of PAX6 | | 1 | 1 | 0.115 | | 0.01 | | 1 | 1 | 0 | 1999 | 1999 |
| Rheumatoid Arthritis | C0003873 | TRIM3 | 10612 | O75382 | tripartite motif containing 3 | Enzyme | 31 | 0.682 | 0.423 | 0.9893 | 0.01 | | 1 | 1 | 0 | 2019 | 2019 |
| Rheumatoid Arthritis | C0003873 | CENPE | 1062 | Q02224 | centromere protein E | Cellular structure | 78 | 0.636 | 0.692 | 0.24146 | 0.01 | | 0 | 1 | 0 | 1999 | 1999 |
| Rheumatoid Arthritis | C0003873 | POSTN | 10631 | Q15063 | periostin | Signaling | 378 | 0.428 | 0.885 | 1.07E-09 | 0.01 | | 1 | 1 | 0 | 2019 | 2019 |
| Rheumatoi | C0003 | DCTN6 | 106 | O00399 | dynactin subunit 6 | Cellular str | 315 | 0.4 | 0.7 | 0.055 | 0.01 | | 1 | 1 | 0 | 2005 | 2005 |

| | | | | | | | | | | | | | | | | | |
|---|---|---|---|---|---|---|---|---|---|---|---|---|---|---|---|---|---|
| d Arthritis | 873 | | 71 | | | ucture | | 42 | 69 | 741 | | | | | | | |
| Rheumatoid Arthritis | C0003873 | YME1L1 | 10730 | Q96TA2 | YME1 like 1 ATPase | Enzyme | 60 | 0.633 | 0.654 | 0.0289888 | 0.01 | | 1 | 1 | 0 | 2014 | 2014 |
| Rheumatoid Arthritis | C0003873 | MAP3K2 | 10746 | Q9Y2U5 | mitogen-activated protein kinase kinase kinase 2 | Kinase | 37 | 0.686 | 0.231 | 0.999999 | 0.01 | | 1 | 1 | 0 | 2004 | 2004 |
| Rheumatoid Arthritis | C0003873 | MASP2 | 10747 | O00187 | mannan binding lectin serine peptidase 2 | Enzyme | 86 | 0.582 | 0.692 | 3.04E-14 | 0.01 | | 1 | 1 | 0 | 2014 | 2014 |
| Rheumatoid Arthritis | C0003873 | LOC107987462 | 1.08E+08 | O75022 | leukocyte immunoglobulin-like receptor subfamily B member 3 | | 6 | 0.839 | 0.231 | | 0.01 | | 1 | 1 | 0 | 2016 | 2016 |
| Rheumatoid Arthritis | C0003873 | CYSLTR1 | 10800 | Q9Y271 | cysteinyl leukotriene receptor 1 | G-protein coupled receptor | 81 | 0.593 | 0.769 | 0.01203 | 0.01 | | 1 | 1 | 0 | 2017 | 2017 |
| Rheumatoid Arthritis | C0003873 | NEU3 | 10825 | Q9UQ49 | neuraminidase 3 | Enzyme | 39 | 0.666 | 0.654 | 2.44E-08 | 0.01 | | 1 | 1 | 0 | 2019 | 2019 |

| | | | | | | | | | | | | | | | | | |
|---|---|---|---|---|---|---|---|---|---|---|---|---|---|---|---|---|---|
| Rheumatoid Arthritis | C0003873 | CEACAM8 | 1088 | P31997 | CEA cell adhesion molecule 8 | | 29 | 0.682 | 0.538 | 3.3E-07 | 0.01 | | 1 | 1 | 0 | 2019 | 2019 |
| Rheumatoid Arthritis | C0003873 | LYVE1 | 10894 | Q9Y5Y7 | lymphatic vessel endothelial hyaluronan receptor 1 | | 80 | 0.587 | 0.731 | 4.72E-07 | 0.01 | | 1 | 1 | 0 | 2017 | 2017 |
| Rheumatoid Arthritis | C0003873 | JTB | 10899 | O76095 | jumping translocation breakpoint | | 64 | 0.615 | 0.769 | 0.42432 | 0.01 | | 1 | 1 | 0 | 2018 | 2018 |
| Rheumatoid Arthritis | C0003873 | SUB1 | 10923 | P53999 | SUB1 regulator of transcription | | 260 | 0.465 | 0.846 | 0.89245 | 0.01 | | 1 | 1 | 0 | 1990 | 1990 |
| Rheumatoid Arthritis | C0003873 | TMED2 | 10959 | Q15363 | transmembrane p24 trafficking protein 2 | Transporter | 73 | 0.61 | 0.692 | 0.89231 | 0.01 | | 1 | 1 | 0 | 1989 | 1989 |
| Rheumatoid Arthritis | C0003873 | IFI44L | 10964 | Q53G44 | interferon induced protein 44 like | | 23 | 0.729 | 0.462 | 1.35E-12 | 0.01 | | 1 | 1 | 0 | 2016 | 2016 |
| Rheumatoid Ar | C0003873 | RAB40B | 10966 | Q12829 | RAB40B, member RAS oncogene family | | 99 | 0.563 | 0.731 | 8.66E- | 0.01 | | 1 | 1 | 0 | 2017 | 2017 |

| | | | | | | | | | | | | | | | | | |
|---|---|---|---|---|---|---|---|---|---|---|---|---|---|---|---|---|---|
| thritis | | | | | | | | | | 06 | | | | | | | |
| Rheumatoid Arthritis | C0003873 | ERVK-32 | 1.1E+08 | | endogenous retrovirus group K member 32, envelope | | 158 | 0.522 | 0.692 | | 0.01 | | 1 | 1 | 0 | 2017 | 2017 |
| Rheumatoid Arthritis | C0003873 | IL24 | 11009 | Q13007 | interleukin 24 | | 202 | 0.498 | 0.769 | 5.88E-07 | 0.01 | | 1 | 1 | 0 | 2008 | 2008 |
| Rheumatoid Arthritis | C0003873 | LILRA1 | 11024 | O75019 | leukocyte immunoglobulin like receptor A1 | Receptor | 8 | 0.805 | 0.308 | 7.39E-20 | 0.01 | | 1 | 1 | 0 | 2008 | 2008 |
| Rheumatoid Arthritis | C0003873 | LILRB3 | 11025 | O75022 | leukocyte immunoglobulin like receptor B3 | | 10 | 0.805 | 0.269 | 3.11E-06 | 0.01 | | 1 | 1 | 0 | 2016 | 2016 |
| Rheumatoid Arthritis | C0003873 | LILRA2 | 11027 | Q8N149 | leukocyte immunoglobulin like receptor A2 | Receptor | 6 | 0.861 | 0.231 | 6.91E-20 | 0.01 | | 1 | 1 | 0 | 2002 | 2002 |
| Rheumatoid Arthritis | C0003873 | PIM2 | 11040 | Q9P1W9 | Pim-2 proto-oncogene, serine/threonine kinase | Kinase | 68 | 0.606 | 0.538 | 0.94801 | 0.01 | | 1 | 1 | 0 | 2015 | 2015 |
| Rheumatoi | C0003 | C5-OT1 | 1.1E | | C5 3' UTR overlapping transcript 1 | | 1 | 1 | 0.1 | | 0.01 | | 1 | 1 | 3 | 2016 | 2016 |

| | | | | | | | | | | | | | | | | | |
|---|---|---|---|---|---|---|---|---|---|---|---|---|---|---|---|---|---|
| d Arthritis | 873 | | +08 | | | | | | 15 | | | | | | | | |
| Rheumatoid Arthritis | C0003873 | TPPP | 11076 | O94811 | tubulin polymerization promoting protein | Cellular structure | 110 | 0.57 | 0.692 | 0.20334 | 0.01 | | 1 | 1 | 0 | 1989 | 1989 |
| Rheumatoid Arthritis | C0003873 | DNAJB4 | 11080 | Q9UDY4 | DnaJ heat shock protein family (Hsp40) member B4 | | 37 | 0.705 | 0.346 | 0.016227 | 0.01 | | 1 | 1 | 0 | 2014 | 2014 |
| Rheumatoid Arthritis | C0003873 | RCAN3 | 11123 | Q9UKA8 | RCAN family member 3 | Signaling | 9 | 0.821 | 0.154 | 0.00157 | 0.01 | | 1 | 1 | 0 | 2017 | 2017 |
| Rheumatoid Arthritis | C0003873 | CHGA | 1113 | P10645 | chromogranin A | | 218 | 0.49 | 0.808 | 0.030369 | 0.01 | | 1 | 1 | 0 | 2008 | 2008 |
| Rheumatoid Arthritis | C0003873 | IL1RAPL1 | 11141 | Q9NZN1 | interleukin 1 receptor accessory protein like 1 | Receptor | 103 | 0.617 | 0.692 | 0.99886 | 0.01 | | 1 | 1 | 0 | 2020 | 2020 |
| Rheumatoid Arthritis | C0003873 | KAT7 | 11143 | O95251 | lysine acetyltransferase 7 | Enzyme | 35 | 0.682 | 0.538 | 0.99999 | 0.01 | | 1 | 1 | 0 | 2017 | 2017 |

| | | | | | | | | | | | | | | | | | | |
|---|---|---|---|---|---|---|---|---|---|---|---|---|---|---|---|---|---|---|
| Rheumatoid Arthritis | C0003873 | ADAMTS7 | 11173 | Q9UKP4 | ADAM metallopeptidase with thrombospondin type 1 motif 7 | Enzyme | 40 | 0.666 | 0.577 | 2.25E-14 | 0.01 | | 1 | 1 | 0 | 2006 | 2006 |
| Rheumatoid Arthritis | C0003873 | TREH | 11181 | O43280 | trehalase | | 42 | 0.67 | 0.654 | 8.96E-15 | 0.01 | | 0 | 1 | 0 | 2017 | 2017 |
| Rheumatoid Arthritis | C0003873 | WIF1 | 11197 | Q9Y5W5 | WNT inhibitory factor 1 | Calcium-binding protein | 171 | 0.507 | 0.731 | 1.32E-09 | 0.01 | | 1 | 1 | 0 | 2013 | 2013 |
| Rheumatoid Arthritis | C0003873 | CHEK2 | 11200 | O96017 | checkpoint kinase 2 | Kinase | 297 | 0.46 | 0.808 | 1.21E-24 | 0.01 | | 1 | 1 | 0 | 2005 | 2005 |
| Rheumatoid Arthritis | C0003873 | CHN2 | 1124 | P52757 | chimerin 2 | | 25 | 0.711 | 0.615 | 0.002301 | 0.01 | | 1 | 1 | 0 | 2018 | 2018 |
| Rheumatoid Arthritis | C0003873 | CHP1 | 11261 | Q99653 | calcineurin like EF-hand protein 1 | | 141 | 0.546 | 0.769 | 0.62667 | 0.01 | | 1 | 1 | 0 | 1989 | 1989 |
| Rheumatoi | C0003 | TREX1 | 112 | Q9NSU2 | three prime repair exonuclease 1 | | 241 | 0.5 | 0.8 | 0.575 | 0.01 | | 1 | 1 | 0 | 2010 | 2010 |

| | | | | | | | | | | | | | | | | | |
|---|---|---|---|---|---|---|---|---|---|---|---|---|---|---|---|---|---|
| d Arthritis | 873 | | 77 | | | | | 17 | 08 | 28 | | | | | | | |
| Rheumatoid Arthritis | C0003873 | B4GALT7 | 11285 | Q9UBV7 | beta-1,4-galactosyltransferase 7 | | 79 | 0.628 | 0.654 | 0.00113 | 0.01 | | 1 | 1 | 0 | 2010 | 2010 |
| Rheumatoid Arthritis | C0003873 | CHRM3 | 1131 | P20309 | cholinergic receptor muscarinic 3 | G-protein coupled receptor | 284 | 0.464 | 0.885 | 0.99253 | 0.01 | | 1 | 1 | 0 | 2020 | 2020 |
| Rheumatoid Arthritis | C0003873 | CDCA5 | 113130 | Q96FF9 | cell division cycle associated 5 | | 105 | 0.563 | 0.808 | 0.01158 | 0.01 | | 1 | 1 | 0 | 2015 | 2015 |
| Rheumatoid Arthritis | C0003873 | SLC46A1 | 113235 | Q96NT5 | solute carrier family 46 member 1 | Transporter | 97 | 0.606 | 0.731 | 0.021858 | 0.01 | | 1 | 1 | 0 | 2018 | 2018 |
| Rheumatoid Arthritis | C0003873 | VSIG4 | 11326 | Q9Y279 | V-set and immunoglobulin domain containing 4 | | 51 | 0.628 | 0.615 | 1.5E-09 | 0.01 | | 1 | 1 | 0 | 2002 | 2002 |
| Rheumatoid Arthritis | C0003873 | GLCCI1 | 113263 | Q86VQ1 | glucocorticoid induced 1 | | 27 | 0.716 | 0.385 | 0.05903 | 0.01 | | 1 | 1 | 1 | 2015 | 2015 |

| | | | | | | | | | | | | | | | | | |
|---|---|---|---|---|---|---|---|---|---|---|---|---|---|---|---|---|---|
| Rheumatoid Arthritis | C0003873 | CHRNA7 | 1139 | P36544;Q494W8 | cholinergic receptor nicotinic alpha 7 subunit | Ion channel | 172 | 0.532 | 0.654 | 0.000415 | 0.01 | | 1 | 1 | 0 | 2019 | 2019 |
| Rheumatoid Arthritis | C0003873 | DBA2 | 114086 | | Diamond-Blackfan anemia 2 | | 74 | 0.599 | 0.731 | | 0.01 | | 1 | 1 | 0 | 1999 | 1999 |
| Rheumatoid Arthritis | C0003873 | UCN3 | 114131 | Q969E3 | urocortin 3 | | 82 | 0.593 | 0.731 | 0.043878 | 0.01 | | 1 | 1 | 0 | 2011 | 2011 |
| Rheumatoid Arthritis | C0003873 | CIRBP | 1153 | Q14011 | cold inducible RNA binding protein | Nucleic acid binding | 72 | 0.592 | 0.731 | 0.33636 | 0.01 | | 1 | 1 | 0 | 2018 | 2018 |
| Rheumatoid Arthritis | C0003873 | H3P8 | 1.15E+08 | | H3 histone pseudogene 8 | | 116 | 0.544 | 0.808 | | 0.01 | | 1 | 1 | 0 | 1990 | 1990 |
| Rheumatoid Arthritis | C0003873 | H3P23 | 1.15E+08 | | H3 histone pseudogene 23 | | 310 | 0.443 | 0.769 | | 0.01 | | 1 | 1 | 0 | 2005 | 2005 |
| Rheumatoid Ar | C0003873 | H3P19 | 1.15E+ | | H3 histone pseudogene 19 | | 62 | 0.631 | 0.577 | | 0.01 | | 1 | 1 | 0 | 1989 | 1989 |

| | | | | | | | | | | | | | | | | | |
|---|---|---|---|---|---|---|---|---|---|---|---|---|---|---|---|---|---|
| thritis | | | 08 | | | | | | | | | | | | | | |
| Rheumatoid Arthritis | C0003873 | H3P40 | 1.15E+08 | | H3 histone pseudogene 40 | | 175 | 0.506 | 0.846 | | 0.01 | | 1 | 1 | 0 | 2002 | 2002 |
| Rheumatoid Arthritis | C0003873 | ADCYAP1R1 | 117 | P41586 | ADCYAP receptor type I | G-protein coupled receptor | 118 | 0.561 | 0.769 | 7.23E-10 | 0.01 | | 1 | 1 | 0 | 2018 | 2018 |
| Rheumatoid Arthritis | C0003873 | PRAP1 | 118471 | Q96NZ9 | proline rich acidic protein 1 | | 59 | 0.621 | 0.615 | 0.032463 | 0.01 | | 1 | 1 | 0 | 2005 | 2005 |
| Rheumatoid Arthritis | C0003873 | TPP1 | 1200 | O14773 | tripeptidyl peptidase 1 | Enzyme | 151 | 0.551 | 0.808 | 6.26E-07 | 0.01 | | 1 | 1 | 0 | 2012 | 2012 |
| Rheumatoid Arthritis | C0003873 | CYP2R1 | 120227 | Q6VVX0 | cytochrome P450 family 2 subfamily R member 1 | | 139 | 0.563 | 0.731 | 3.79E-10 | 0.01 | | 1 | 1 | 0 | 2014 | 2014 |
| Rheumatoid Arthritis | C0003873 | CLTA | 1211 | P09496 | clathrin light chain A | Transporter | 55 | 0.628 | 0.654 | 0.96319 | 0.01 | | 1 | 1 | 0 | 2005 | 2005 |
| Rheu | C00 | TPH2 | 12 | Q8IWU9 | tryptophan hydroxylase 2 | | 137 | 0. | 0. | 2.5 | 0.01 | | 1 | 1 | 0 | 2012 | 20 |

| | | | | | | | | | | | | | | | | | |
|---|---|---|---|---|---|---|---|---|---|---|---|---|---|---|---|---|---|
| matoid Arthritis | 03873 | | 1278 | | | | | 546 | 654 | 4E-07 | | | | | | | 12 |
| Rheumatoid Arthritis | C0003873 | CMKLR1 | 1240 | Q99788 | chemerin chemokine-like receptor 1 | G-protein coupled receptor | 75 | 0.612 | 0.692 | 0.010767 | 0.01 | | 1 | 1 | 0 | 2012 | 2012 |
| Rheumatoid Arthritis | C0003873 | GGT6 | 124975 | Q6P531 | gamma-glutamyltransferase 6 | | 2 | 0.931 | 0.115 | 2.98E-08 | 0.01 | | 1 | 1 | 0 | 2017 | 2017 |
| Rheumatoid Arthritis | C0003873 | PLK3 | 1263 | Q9H4B4 | polo like kinase 3 | Kinase | 52 | 0.644 | 0.538 | 1.85E-06 | 0.01 | | 1 | 1 | 0 | 2017 | 2017 |
| Rheumatoid Arthritis | C0003873 | CNN2 | 1265 | Q99439 | calponin 2 | Cellular structure | 36 | 0.666 | 0.538 | 0.024399 | 0.01 | | 1 | 1 | 0 | 2016 | 2016 |
| Rheumatoid Arthritis | C0003873 | COL1A1 | 1277 | P02452 | collagen type I alpha 1 chain | | 487 | 0.43 | 0.808 | 1 | 0.01 | | 1 | 1 | 0 | 2015 | 2015 |
| Rheumatoid Ar | C0003873 | OSCP1 | 127700 | Q8WVF1 | organic solute carrier partner 1 | | 61 | 0.61 | 0.692 | 7.03E- | 0.01 | | 1 | 1 | 0 | 2002 | 2002 |

| | | | | | | | | | | | | | | | | | |
|---|---|---|---|---|---|---|---|---|---|---|---|---|---|---|---|---|---|
| thritis | | | | | | | | | | 10 | | | | | | | |
| Rheumatoid Arthritis | C0003873 | COL1A2 | 1278 | P08123 | collagen type I alpha 2 chain | | 271 | 0.486 | 0.846 | 1 | 0.01 | | 1 | 1 | 0 | 2017 | 2017 |
| Rheumatoid Arthritis | C0003873 | COL4A5 | 1287 | P29400 | collagen type IV alpha 5 chain | | 124 | 0.553 | 0.654 | 1 | 0.01 | | 1 | 1 | 0 | 2019 | 2019 |
| Rheumatoid Arthritis | C0003873 | COL9A2 | 1298 | Q14055 | collagen type IX alpha 2 chain | | 116 | 0.572 | 0.769 | 1.05E-08 | 0.01 | | 0 | 1 | 0 | 2006 | 2006 |
| Rheumatoid Arthritis | C0003873 | COL12A1 | 1303 | Q99715 | collagen type XII alpha 1 chain | Receptor | 93 | 0.615 | 0.692 | 0.97361 | 0.01 | | 1 | 1 | 0 | 2015 | 2015 |
| Rheumatoid Arthritis | C0003873 | KLF6 | 1316 | Q99612 | Kruppel like factor 6 | Nucleic acid binding | 204 | 0.501 | 0.846 | 0.97946 | 0.01 | | 1 | 1 | 0 | 2018 | 2018 |
| Rheumatoid Arthritis | C0003873 | ADAD1 | 132612 | Q96M93 | adenosine deaminase domain containing 1 | Enzyme | 8 | 0.821 | 0.269 | 0.038952 | 0.01 | | 1 | 1 | 1 | 2010 | 2010 |
| Rheumatoi | C0003 | ZFP42 | 1326 | Q96MM3 | ZFP42 zinc finger protein | Transcripti | 30 | 0.6 | 0.3 | | 0.01 | | 1 | 1 | 0 | 2010 | 2010 |

| | | | | | | | | | | | | | | | | | |
|---|---|---|---|---|---|---|---|---|---|---|---|---|---|---|---|---|---|
| d Arthritis | 873 | | 25 | | | on factor | | 78 | 46 | | | | | | | | |
| Rheumatoid Arthritis | C0003873 | CD109 | 135228 | Q6YHK3 | CD109 molecule | | 81 | 0.595 | 0.654 | 2.27E-44 | 0.01 | | 1 | 1 | 0 | 2019 | 2019 |
| Rheumatoid Arthritis | C0003873 | CPA1 | 1357 | P15085 | carboxypeptidase A1 | Enzyme | 61 | 0.65 | 0.577 | 2.66E-11 | 0.01 | | 1 | 1 | 0 | 2019 | 2019 |
| Rheumatoid Arthritis | C0003873 | CPB2 | 1361 | Q96IY4 | carboxypeptidase B2 | Enzyme | 148 | 0.536 | 0.846 | 2.71E-17 | 0.01 | | 1 | 1 | 0 | 2019 | 2019 |
| Rheumatoid Arthritis | C0003873 | CLDN7 | 1366 | O95471 | claudin 7 | Cell-cell junction | 192 | 0.494 | 0.808 | 0.002883 | 0.01 | | 1 | 1 | 0 | 2018 | 2018 |
| Rheumatoid Arthritis | C0003873 | CPOX | 1371 | P36551 | coproporphyrinogen oxidase | Enzyme | 246 | 0.488 | 0.846 | 0.171152 | 0.01 | | 1 | 1 | 0 | 2000 | 2000 |
| Rheumatoid Arthritis | C0003873 | CRABP1 | 1381 | P29762 | cellular retinoic acid binding protein 1 | | 53 | 0.631 | 0.615 | 0.0058788 | 0.01 | | 1 | 1 | 0 | 2015 | 2015 |

| | | | | | | | | | | | | | | | | | |
|---|---|---|---|---|---|---|---|---|---|---|---|---|---|---|---|---|---|
| Rheumatoid Arthritis | C0003873 | CRHR2 | 1395 | Q13324 | corticotropin releasing hormone receptor 2 | G-protein coupled receptor | 89 | 0.584 | 0.731 | 8.55E-17 | 0.01 | | 1 | 1 | 0 | 2011 | 2011 |
| Rheumatoid Arthritis | C0003873 | CRKL | 1399 | P46109 | CRK like proto-oncogene, adaptor protein | | 140 | 0.553 | 0.769 | 0.44604 | 0.01 | | 1 | 1 | 0 | 2013 | 2013 |
| Rheumatoid Arthritis | C0003873 | ADORA3 | 140 | P0DMS8 | adenosine A3 receptor | G-protein coupled receptor | 36 | 0.682 | 0.5 | 0.000144 | 0.01 | | 1 | 1 | 0 | 2019 | 2019 |
| Rheumatoid Arthritis | C0003873 | MUC17 | 140453 | Q685J3 | mucin 17, cell surface associated | | 53 | 0.638 | 0.577 | 7.9E-63 | 0.01 | | 1 | 1 | 0 | 2008 | 2008 |
| Rheumatoid Arthritis | C0003873 | NRSN1 | 140767 | Q8IZ57 | neurensin 1 | | 76 | 0.604 | 0.731 | 0.000308 | 0.01 | | 1 | 1 | 0 | 1989 | 1989 |
| Rheumatoid Arthritis | C0003873 | SIRPA | 140885 | P78324 | signal regulatory protein alpha | Signaling | 82 | 0.592 | 0.769 | 0.67197 | 0.01 | | 1 | 1 | 0 | 2008 | 2008 |

| | | | | | | | | | | | | | | | | | |
|---|---|---|---|---|---|---|---|---|---|---|---|---|---|---|---|---|---|
| Rheumatoid Arthritis | C0003873 | CSE1L | 1434 | P55060 | chromosome segregation 1 like | Enzyme modulator | 163 | 0.521 | 0.808 | 1 | 0.01 | | 1 | 1 | 0 | 2001 | 2001 |
| Rheumatoid Arthritis | C0003873 | PIWIL4 | 143689 | Q7Z3Z4 | piwi like RNA-mediated gene silencing 4 | | 83 | 0.579 | 0.692 | 1.83E-06 | 0.01 | | 1 | 1 | 0 | 2016 | 2016 |
| Rheumatoid Arthritis | C0003873 | CSF2RA | 1438 | P15509 | colony stimulating factor 2 receptor subunit alpha | Signaling | 49 | 0.67 | 0.615 | 2.4E-10 | 0.01 | | 1 | 1 | 0 | 1994 | 1994 |
| Rheumatoid Arthritis | C0003873 | CSF3R | 1441 | Q99062 | colony stimulating factor 3 receptor | Signaling | 120 | 0.55 | 0.692 | 1.52E-10 | 0.01 | | 1 | 1 | 0 | 2016 | 2016 |
| Rheumatoid Arthritis | C0003873 | OR2AG1 | 144125 | Q9H205 | olfactory receptor family 2 subfamily AG member 1 (gene/pseudogene) | G-protein coupled receptor | 100 | 0.582 | 0.808 | 3.12E-11 | 0.01 | | 1 | 1 | 0 | 2008 | 2008 |
| Rheumatoid Arthritis | C0003873 | CSNK1E | 1454 | P49674 | casein kinase 1 epsilon | | 61 | 0.619 | 0.615 | 0.99926 | 0.01 | | 1 | 1 | 0 | 2019 | 2019 |
| Rheuma | C0003 | PWAR1 | 145 | | Prader Willi/Angelm | | 246 | 0. | 0.7 | | 0.01 | | 1 | 1 | 0 | 2012 | 20 |

| | | | | | | | | | | | | | | | | | |
|---|---|---|---|---|---|---|---|---|---|---|---|---|---|---|---|---|---|
| toid Arthritis | 873 | | 624 | | an region RNA 1 | | | 49 | 69 | | | | | | | | 12 |
| Rheumatoid Arthritis | C0003873 | B3GNTL1 | 146712 | Q67FW5 | UDP-GlcNAc:betaGal beta-1,3-N-acetylglucosaminyltransferase like 1 | Enzyme | 12 | 0.76 | 0.346 | 4.07E-28 | 0.01 | | 1 | 1 | 0 | 1988 | 1988 |
| Rheumatoid Arthritis | C0003873 | PTPRVP | 148713 | | protein tyrosine phosphatase receptor type V, pseudogene | | 27 | 0.705 | 0.577 | | 0.01 | | 1 | 1 | 0 | 2016 | 2016 |
| Rheumatoid Arthritis | C0003873 | CTH | 1491 | P32929 | cystathionine gamma-lyase | Enzyme | 93 | 0.578 | 0.615 | 7.48E-08 | 0.01 | | 1 | 1 | 0 | 2019 | 2019 |
| Rheumatoid Arthritis | C0003873 | CTRB1 | 1504 | P17538 | chymotrypsinogen B1 | Enzyme | 18 | 0.76 | 0.385 | 2.42E-06 | 0.01 | | 0 | 1 | 0 | 2013 | 2013 |
| Rheumatoid Arthritis | C0003873 | COMMD1 | 150684 | Q8N668 | copper metabolism domain containing 1 | | 55 | 0.633 | 0.692 | 0.2201 | 0.01 | | 1 | 1 | 0 | 2017 | 2017 |
| Rheumatoid Ar | C0003873 | GPBAR1 | 151306 | Q8TDU6 | G protein-coupled bile acid receptor 1 | G-protein coupled | 122 | 0.548 | 0.731 | 1.36E-09 | 0.01 | | 1 | 1 | 0 | 2019 | 2019 |

| | | | | | | | | | | | | | | | | | |
|---|---|---|---|---|---|---|---|---|---|---|---|---|---|---|---|---|---|
| thritis | | | | | | receptor | | | | | | | | | | | |
| Rheumatoid Arthritis | C0003873 | RMDN2 | 151393 | Q96LZ7 | regulator of microtubule dynamics 2 | | 137 | 0.531 | 0.769 | 4.41E-24 | 0.01 | | 1 | 1 | 0 | 2019 | 2019 |
| Rheumatoid Arthritis | C0003873 | CTSS | 1520 | P25774 | cathepsin S | Enzyme | 131 | 0.539 | 0.731 | 0.341178 | 0.01 | | 1 | 1 | 0 | 2002 | 2002 |
| Rheumatoid Arthritis | C0003873 | CUX1 | 1523 | P39880;Q13948 | cut like homeobox 1 | Transcription factor | 283 | 0.465 | 0.846 | 1 | 0.01 | | 1 | 1 | 0 | 1992 | 1992 |
| Rheumatoid Arthritis | C0003873 | CYB5A | 1528 | P00167 | cytochrome b5 type A | Enzyme | 95 | 0.601 | 0.654 | 0.001582 | 0.01 | | 1 | 1 | 1 | 2015 | 2015 |
| Rheumatoid Arthritis | C0003873 | CREBRF | 153222 | Q8IUR6 | CREB3 regulatory factor | | 50 | 0.636 | 0.5 | 0.99997 | 0.01 | | 1 | 1 | 0 | 2011 | 2011 |
| Rheumatoid Arthritis | C0003873 | CYBA | 1535 | P13498 | cytochrome b-245 alpha chain | | 177 | 0.52 | 0.769 | 8.87E-06 | 0.01 | | 1 | 1 | 0 | 2018 | 2018 |
| Rheuma | C0003 | CYLD | 15 | Q9NQC7 | CYLD lysine 63 | Enzyme | 211 | 0.4 | 0.8 | 0.99 | 0.01 | | 1 | 1 | 0 | 2005 | 20 |

| | | | | | | | | | | | | | | | | | |
|---|---|---|---|---|---|---|---|---|---|---|---|---|---|---|---|---|---|
| toid Arthritis | 873 | | 40 | | deubiquitinase | | | 98 | 46 | 993 | | | | | | | 05 |
| Rheumatoid Arthritis | C0003873 | CYP1A1 | 1543 | P04798 | cytochrome P450 family 1 subfamily A member 1 | Enzyme | 379 | 0.436 | 0.846 | 1.06E-17 | 0.01 | | 0 | 1 | 1 | 2003 | 2003 |
| Rheumatoid Arthritis | C0003873 | AMOT | 154796 | Q4VCS5 | angiomotin | | 52 | 0.641 | 0.538 | 0.99666 | 0.01 | | 1 | 1 | 0 | 2009 | 2009 |
| Rheumatoid Arthritis | C0003873 | ADRB3 | 155 | P13945 | adrenoceptor beta 3 | G-protein coupled receptor | 142 | 0.533 | 0.731 | 2.46E-07 | 0.01 | | 1 | 1 | 0 | 2003 | 2003 |
| Rheumatoid Arthritis | C0003873 | CYP2B6 | 1555 | P20813 | cytochrome P450 family 2 subfamily B member 6 | | 388 | 0.428 | 0.846 | 1.93E-10 | 0.01 | | 1 | 1 | 1 | 1994 | 1994 |
| Rheumatoid Arthritis | C0003873 | CYP2C19 | 1557 | P33261 | cytochrome P450 family 2 subfamily C member 19 | | 274 | 0.467 | 0.846 | 6.49E-20 | 0.01 | | 1 | 1 | 0 | 2012 | 2012 |
| Rheumatoid Ar | C0003873 | CYP2E1 | 1571 | P05181 | cytochrome P450 family 2 subfamily E member 1 | | 306 | 0.459 | 0.885 | 8.07E-09 | 0.01 | | 1 | 1 | 0 | 2018 | 2018 |

| | | | | | | | | | | | | | | | | | |
|---|---|---|---|---|---|---|---|---|---|---|---|---|---|---|---|---|---|
| thritis | | | | | | | | | | | | | | | | | |
| Rheumatoid Arthritis | C0003873 | CYP3A4 | 1576 | P08684 | cytochrome P450 family 3 subfamily A member 4 | Enzyme | 291 | 0.462 | 0.885 | 5.8E-11 | 0.01 | | 1 | 1 | 0 | 2017 | 2017 |
| Rheumatoid Arthritis | C0003873 | DCC | 1630 | P43146 | DCC netrin 1 receptor | | 279 | 0.471 | 0.808 | 0.99448 | 0.01 | | 0 | 1 | 1 | 2003 | 2003 |
| Rheumatoid Arthritis | C0003873 | DCK | 1633 | P27707 | deoxycytidine kinase | Kinase | 102 | 0.561 | 0.692 | 1.55E-05 | 0.01 | | 1 | 1 | 0 | 2013 | 2013 |
| Rheumatoid Arthritis | C0003873 | IFNLR1 | 163702 | Q8IU57 | interferon lambda receptor 1 | Receptor | 40 | 0.647 | 0.615 | 0.062532 | 0.01 | | 1 | 1 | 0 | 2013 | 2013 |
| Rheumatoid Arthritis | C0003873 | KANK4 | 163782 | Q5T7N3 | KN motif and ankyrin repeat domains 4 | | 7 | 0.839 | 0.269 | 2.07E-09 | 0.01 | | 1 | 1 | 0 | 2014 | 2014 |
| Rheumatoid Arthritis | C0003873 | DEFA1 | 1667 | P59665 | defensin alpha 1 | | 57 | 0.636 | 0.731 | | 0.01 | | 1 | 1 | 0 | 2003 | 2003 |
| Rheumatoi | C0003 | DEFA3 | 1668 | P59666 | defensin alpha 3 | | 23 | 0.7 | 0.5 | 0.090 | 0.01 | | 1 | 1 | 0 | 2003 | 2003 |

| | | | | | | | | | | | | | | | | | |
|---|---|---|---|---|---|---|---|---|---|---|---|---|---|---|---|---|---|
| d Arthritis | 873 | | | | | | | 16 | 38 | 546 | | | | | | | |
| Rheumatoid Arthritis | C0003873 | IDO2 | 169355 | Q6ZQW0 | indoleamine 2,3-dioxygenase 2 | | 31 | 0.695 | 0.577 | 3.14E-09 | 0.01 | | 1 | 1 | 0 | 2017 | 2017 |
| Rheumatoid Arthritis | C0003873 | AAVS1 | 17 | | adeno-associated virus integration site 1 | | 78 | 0.595 | 0.731 | | 0.01 | | 1 | 1 | 0 | 2006 | 2006 |
| Rheumatoid Arthritis | C0003873 | NQO1 | 1728 | P15559 | NAD(P)H quinone dehydrogenase 1 | | 368 | 0.434 | 0.885 | 1.19E-09 | 0.01 | | 1 | 1 | 0 | 2018 | 2018 |
| Rheumatoid Arthritis | C0003873 | SARDH | 1757 | Q9UL12 | sarcosine dehydrogenase | Enzyme | 166 | 0.523 | 0.846 | 5.27E-16 | 0.01 | | 1 | 1 | 0 | 2004 | 2004 |
| Rheumatoid Arthritis | C0003873 | DNAH8 | 1769 | Q96JB1 | dynein axonemal heavy chain 8 | Enzyme | 120 | 0.556 | 0.731 | 1.15E-60 | 0.01 | | 1 | 1 | 0 | 2016 | 2016 |
| Rheumatoid Arthritis | C0003873 | DOCK2 | 1794 | Q92608 | dedicator of cytokinesis 2 | | 40 | 0.674 | 0.462 | 0.99969 | 0.01 | | 1 | 1 | 0 | 2019 | 2019 |

| | | | | | | | | | | | | | | | | |
|---|---|---|---|---|---|---|---|---|---|---|---|---|---|---|---|---|
| Rheumatoid Arthritis | C0003873 | AGT | 183 | P01019 | angiotensinogen | Enzyme modulator | 765 | 0.367 | 0.923 | 4.97E-08 | 0.01 | | 1 | 1 | 0 | 2018 | 2018 |
| Rheumatoid Arthritis | C0003873 | DSPP | 1834 | Q9NZW4 | dentin sialophosphoprotein | | 114 | 0.556 | 0.769 | 1.12E-07 | 0.01 | | 1 | 1 | 0 | 2018 | 2018 |
| Rheumatoid Arthritis | C0003873 | DUSP2 | 1844 | Q05923 | dual specificity phosphatase 2 | | 118 | 0.558 | 0.769 | 4.47E-05 | 0.01 | | 1 | 1 | 0 | 2018 | 2018 |
| Rheumatoid Arthritis | C0003873 | AGTR1 | 185 | P30556 | angiotensin II receptor type 1 | G-protein coupled receptor | 440 | 0.423 | 0.846 | 0.000532 | 0.01 | | 1 | 1 | 0 | 2018 | 2018 |
| Rheumatoid Arthritis | C0003873 | DUSP8 | 1850 | Q13202 | dual specificity phosphatase 8 | | 12 | 0.769 | 0.385 | 0.69975 | 0.01 | | 1 | 1 | 0 | 2001 | 2001 |
| Rheumatoid Arthritis | C0003873 | DVL2 | 1856 | O14641 | dishevelled segment polarity protein 2 | Signaling | 58 | 0.621 | 0.577 | 1.36E-05 | 0.01 | | 1 | 1 | 0 | 2017 | 2017 |
| Rheumatoi | C0003 | DYRK1A | 1859 | Q13627 | dual specificity tyrosine | Kinase | 212 | 0.5 | 0.7 | 0.999 | 0.01 | | 0 | 1 | 0 | 2018 | 2018 |

| | | | | | | | | | | | | | | | | | |
|---|---|---|---|---|---|---|---|---|---|---|---|---|---|---|---|---|---|
| d Arthritis | 873 | | | | phosphorylation regulated kinase 1A | | | 33 | 69 | 67 | | | | | | | |
| Rheumatoid Arthritis | C0003873 | AGTR2 | 186 | P50052 | angiotensin II receptor type 2 | G-protein coupled receptor | 229 | 0.496 | 0.808 | 0.008235 | 0.01 | | 1 | 1 | 0 | 2017 | 2017 |
| Rheumatoid Arthritis | C0003873 | S1PR3 | 1903 | Q99500 | sphingosine-1-phosphate receptor 3 | G-protein coupled receptor | 47 | 0.638 | 0.654 | 0.046945 | 0.01 | | 1 | 1 | 0 | 2008 | 2008 |
| Rheumatoid Arthritis | C0003873 | EDNRB | 1910 | P24530 | endothelin receptor type B | G-protein coupled receptor | 300 | 0.47 | 0.846 | 0.009242 | 0.01 | | 1 | 1 | 0 | 1999 | 1999 |
| Rheumatoid Arthritis | C0003873 | EFNB2 | 1948 | P52799 | ephrin B2 | Signaling | 109 | 0.561 | 0.692 | 0.98845 | 0.01 | | 1 | 1 | 0 | 2015 | 2015 |
| Rheumatoid Arthritis | C0003873 | EGR2 | 1959 | P11161 | early growth response 2 | Nucleic acid binding | 133 | 0.547 | 0.769 | 0.50292 | 0.01 | | 1 | 1 | 0 | 2010 | 2010 |
| Rheuma | C0003 | AHSG | 197 | P02765 | alpha 2-HS | Enzyme | 204 | 0.5 | 0.8 | 3.21 | 0.01 | | 1 | 1 | 0 | 2017 | 20 |

| | | | | | | | | | | | | | | | | | |
|---|---|---|---|---|---|---|---|---|---|---|---|---|---|---|---|---|---|
| toid Arthritis | 873 | | | | glycoprotein | modulator | | 06 | 46 | E-10 | | | | | | | 17 |
| Rheumatoid Arthritis | C0003873 | NLRC3 | 197358 | Q7RTR2 | NLR family CARD domain containing 3 | Enzyme | 21 | 0.769 | 0.269 | 2.23E-25 | 0.01 | | 1 | 1 | 0 | 2017 | 2017 |
| Rheumatoid Arthritis | C0003873 | EIF4E | 1977 | P06730 | eukaryotic translation initiation factor 4E | Nucleic acid binding | 317 | 0.448 | 0.846 | 0.94133 | 0.01 | | 1 | 1 | 0 | 2018 | 2018 |
| Rheumatoid Arthritis | C0003873 | EIF4G1 | 1981 | Q04637 | eukaryotic translation initiation factor 4 gamma 1 | Nucleic acid binding | 139 | 0.553 | 0.808 | 1 | 0.01 | | 1 | 1 | 0 | 2006 | 2006 |
| Rheumatoid Arthritis | C0003873 | ELANE | 1991 | P08246 | elastase, neutrophil expressed | Enzyme | 346 | 0.447 | 0.846 | 0.001227 | 0.01 | | 1 | 1 | 1 | 2014 | 2014 |
| Rheumatoid Arthritis | C0003873 | A2M | 2 | P01023 | alpha-2-macroglobulin | Enzyme modulator | 147 | 0.529 | 0.769 | 4.52E-11 | 0.01 | | 1 | 1 | 0 | 1999 | 1999 |
| Rheumatoid Arthritis | C0003873 | ELN | 2006 | P15502 | elastin | | 545 | 0.415 | 0.885 | 2.14E-14 | 0.01 | | 1 | 1 | 0 | 2019 | 2019 |

| | | | | | | | | | | | | | | | | |
|---|---|---|---|---|---|---|---|---|---|---|---|---|---|---|---|---|
| Rheumatoid Arthritis | C0003873 | EMD | 2010 | P50402 | emerin | | 163 | 0.542 | 0.731 | 0.931778 | 0.01 | | 1 | 1 | 0 | 2017 | 2017 |
| Rheumatoid Arthritis | C0003873 | MARK2 | 2011 | Q7KZI7 | microtubule affinity regulating kinase 2 | Kinase | 115 | 0.555 | 0.692 | 0.99999 | 0.01 | | 1 | 1 | 0 | 2012 | 2012 |
| Rheumatoid Arthritis | C0003873 | TIGIT | 201633 | Q495A1 | T cell immunoreceptor with Ig and ITIM domains | | 56 | 0.623 | 0.538 | 0.017915 | 0.01 | | 0 | 1 | 0 | 2019 | 2019 |
| Rheumatoid Arthritis | C0003873 | ENG | 2022 | P17813 | endoglin | | 371 | 0.446 | 0.846 | 0.99858 | 0.01 | | 1 | 1 | 0 | 2003 | 2003 |
| Rheumatoid Arthritis | C0003873 | C9orf72 | 203228 | Q96LT7 | C9orf72-SMCR8 complex subunit | | 258 | 0.496 | 0.769 | 1.06E-06 | 0.01 | | 1 | 1 | 0 | 2019 | 2019 |
| Rheumatoid Arthritis | C0003873 | EPHA1 | 2041 | P21709 | EPH receptor A1 | Kinase | 119 | 0.544 | 0.731 | 1.46E-23 | 0.01 | | 1 | 1 | 0 | 2008 | 2008 |
| Rheumatoid Ar | C0003873 | EPHA3 | 2042 | P29320 | EPH receptor A3 | Kinase | 193 | 0.497 | 0.692 | 1.2E-05 | 0.01 | | 1 | 1 | 0 | 2004 | 2004 |

| | | | | | | | | | | | | | | | | | |
|---|---|---|---|---|---|---|---|---|---|---|---|---|---|---|---|---|---|
| thritis | | | | | | | | | | | | | | | | | |
| Rheumatoid Arthritis | C0003873 | CLN8 | 2055 | Q9UBY8 | CLN8 transmembrane ER and ERGIC protein | | 50 | 0.682 | 0.423 | 0.003559 | 0.01 | | 1 | 1 | 0 | 2007 | 2007 |
| Rheumatoid Arthritis | C0003873 | EPO | 2056 | P01588 | erythropoietin | | 646 | 0.381 | 0.885 | 0.01125 | 0.01 | | 1 | 1 | 0 | 2019 | 2019 |
| Rheumatoid Arthritis | C0003873 | LVRN | 206338 | Q6Q4G3 | laeverin | Enzyme | 7 | 0.89 | 0.192 | 5.3E-17 | 0.01 | | 1 | 1 | 0 | 2006 | 2006 |
| Rheumatoid Arthritis | C0003873 | ERBB3 | 2065 | P21860 | erb-b2 receptor tyrosine kinase 3 | Kinase | 318 | 0.448 | 0.808 | 1.7E-15 | 0.01 | | 1 | 1 | 0 | 2016 | 2016 |
| Rheumatoid Arthritis | C0003873 | ERCC2 | 2068 | P18074 | ERCC excision repair 2, TFIIH core complex helicase subunit | Enzyme | 499 | 0.42 | 0.846 | 7.07E-20 | 0.01 | | 1 | 1 | 0 | 2015 | 2015 |
| Rheumatoid Arthritis | C0003873 | EREG | 2069 | O14944 | epiregulin | Signaling | 121 | 0.554 | 0.692 | 1.17E-05 | 0.01 | | 1 | 1 | 0 | 2014 | 2014 |
| Rheumatoi | C0003 | AKT2 | 208 | P31751 | AKT serine/threonine kinase 2 | Kinase | 264 | 0.4 | 0.7 | 0.644 | 0.01 | | 1 | 1 | 0 | 2018 | 2018 |

| | | | | | | | | | | | | | | | | | |
|---|---|---|---|---|---|---|---|---|---|---|---|---|---|---|---|---|---|
| d Arthritis | 873 | | | | | | | 74 | 69 | 32 | | | | | | | |
| Rheumatoid Arthritis | C0003873 | ERV3-1 | 2086 | Q14264 | endogenous retrovirus group 3 member 1, envelope | | 27 | 0.7 | 0.423 | | 0.01 | | 0 | 1 | 0 | 1995 | 1995 |
| Rheumatoid Arthritis | C0003873 | ESRRB | 2103 | O95718 | estrogen related receptor beta | Nuclear receptor | 102 | 0.565 | 0.846 | 0.12609 | 0.01 | | 1 | 1 | 0 | 2017 | 2017 |
| Rheumatoid Arthritis | C0003873 | MECOM | 2122 | Q03112 | MDS1 and EVI1 complex locus | | 191 | 0.513 | 0.731 | 1 | 0.01 | | 1 | 1 | 0 | 2017 | 2017 |
| Rheumatoid Arthritis | C0003873 | EXT1 | 2131 | Q16394 | exostosin glycosyltransferase 1 | Enzyme | 205 | 0.522 | 0.769 | 0.99732 | 0.01 | | 1 | 1 | 0 | 2005 | 2005 |
| Rheumatoid Arthritis | C0003873 | ALCAM | 214 | Q13740 | activated leukocyte cell adhesion molecule | Cell adhesion | 181 | 0.502 | 0.808 | 0.73267 | 0.01 | | 1 | 1 | 0 | 2016 | 2016 |
| Rheumatoid Arthritis | C0003873 | F10 | 2159 | P00742 | coagulation factor X | Enzyme | 228 | 0.493 | 0.769 | 2.35E-06 | 0.01 | | 1 | 1 | 0 | 2017 | 2017 |

| | | | | | | | | | | | | | | | | | |
|---|---|---|---|---|---|---|---|---|---|---|---|---|---|---|---|---|---|
| Rheumatoid Arthritis | C0003873 | ALDH2 | 217 | P05091 | aldehyde dehydrogenase 2 family member | Enzyme | 337 | 0.457 | 0.885 | 3.4E-10 | 0.01 | | 1 | 1 | 0 | 1994 | 1994 |
| Rheumatoid Arthritis | C0003873 | FABP5 | 2171 | Q01469 | fatty acid binding protein 5 | | 85 | 0.585 | 0.731 | 0.047008 | 0.01 | | 1 | 1 | 0 | 2018 | 2018 |
| Rheumatoid Arthritis | C0003873 | FAP | 2191 | Q12884 | fibroblast activation protein alpha | Enzyme | 216 | 0.496 | 0.808 | 1.9E-28 | 0.01 | | 1 | 1 | 0 | 2017 | 2017 |
| Rheumatoid Arthritis | C0003873 | FAT1 | 2195 | Q14517 | FAT atypical cadherin 1 | | 178 | 0.508 | 0.808 | 1.2E-12 | 0.01 | | 1 | 1 | 0 | 2015 | 2015 |
| Rheumatoid Arthritis | C0003873 | UNC5B | 219699 | Q8IZJ1 | unc-5 netrin receptor B | Receptor | 38 | 0.682 | 0.462 | 2.69E-07 | 0.01 | | 1 | 1 | 0 | 2009 | 2009 |
| Rheumatoid Arthritis | C0003873 | ALDH1A3 | 220 | P47895 | aldehyde dehydrogenase 1 family member A3 | Enzyme | 83 | 0.578 | 0.654 | 0.14128 | 0.01 | | 1 | 1 | 0 | 2015 | 2015 |
| Rheumatoid Ar | C0003873 | DLEU7 | 220107 | Q6UYE1 | deleted in lymphocytic leukemia 7 | | 34 | 0.705 | 0.346 | 0.18452 | 0.01 | | 1 | 1 | 0 | 1990 | 1990 |

| | | | | | | | | | | | | | | | | | |
|---|---|---|---|---|---|---|---|---|---|---|---|---|---|---|---|---|---|
| thritis | | | | | | | | | | | | | | | | | |
| Rheumatoid Arthritis | C0003873 | EFEMP1 | 2202 | Q12805 | EGF containing fibulin extracellular matrix protein 1 | Extracellular structure | 153 | 0.54 | 0.769 | 0.99992 | 0.01 | | 1 | 1 | 0 | 2018 | 2018 |
| Rheumatoid Arthritis | C0003873 | FCGRT | 2217 | P55899 | Fc fragment of IgG receptor and transporter | | 102 | 0.563 | 0.654 | 0.041493 | 0.01 | | 1 | 1 | 0 | 2015 | 2015 |
| Rheumatoid Arthritis | C0003873 | FGD1 | 2245 | P98174 | FYVE, RhoGEF and PH domain containing 1 | Enzyme modulator | 131 | 0.573 | 0.731 | 0.9997 | 0.01 | | 1 | 1 | 0 | 2017 | 2017 |
| Rheumatoid Arthritis | C0003873 | FGF4 | 2249 | P08620 | fibroblast growth factor 4 | Signaling | 86 | 0.588 | 0.577 | 0.1406 | 0.01 | | 1 | 1 | 0 | 1998 | 1998 |
| Rheumatoid Arthritis | C0003873 | FGF7 | 2252 | P21781 | fibroblast growth factor 7 | Signaling | 167 | 0.519 | 0.846 | 0.77089 | 0.01 | | 1 | 1 | 0 | 1992 | 1992 |
| Rheumatoid Arthritis | C0003873 | FGF9 | 2254 | P31371 | fibroblast growth factor 9 | Signaling | 153 | 0.528 | 0.846 | 0.94704 | 0.01 | | 1 | 1 | 0 | 2018 | 2018 |
| Rheumatoi | C0003 | FGFR1 | 2260 | P11362 | fibroblast growth factor | Kinase | 816 | 0.3 | 0.8 | 0.999 | 0.01 | | 1 | 1 | 0 | 2003 | 2003 |

| | | | | | | | | | | | | | | | | | |
|---|---|---|---|---|---|---|---|---|---|---|---|---|---|---|---|---|---|
| d Arthritis | 873 | | | | receptor 1 | | | 62 | 85 | 84 | | | | | | | |
| Rheumatoid Arthritis | C0003873 | FGFR3 | 2261 | P22607 | fibroblast growth factor receptor 3 | Kinase | 654 | 0.391 | 0.846 | 1.64E-05 | 0.01 | | 1 | 1 | 0 | 2018 | 2018 |
| Rheumatoid Arthritis | C0003873 | FHL2 | 2274 | Q14192 | four and a half LIM domains 2 | Transcription factor | 121 | 0.556 | 0.731 | 2.87E-05 | 0.01 | | 1 | 1 | 0 | 2015 | 2015 |
| Rheumatoid Arthritis | C0003873 | VEGFD | 2277 | O43915 | vascular endothelial growth factor D | Signaling | 132 | 0.538 | 0.769 | 2.5E-05 | 0.01 | | 1 | 1 | 0 | 2002 | 2002 |
| Rheumatoid Arthritis | C0003873 | IKZF2 | 22807 | Q9UKS7 | IKAROS family zinc finger 2 | Transcription factor | 39 | 0.656 | 0.5 | 0.98731 | 0.01 | | 1 | 1 | 0 | 2018 | 2018 |
| Rheumatoid Arthritis | C0003873 | NR1I4 | 22817 | | nuclear receptor subfamily 1 group I member 4 | | 3 | 0.89 | 0.385 | | 0.01 | | 1 | 1 | 0 | 2001 | 2001 |
| Rheumatoid Arthritis | C0003873 | PUF60 | 22827 | Q9UHX1 | poly(U) binding splicing factor 60 | | 188 | 0.536 | 0.769 | 0.99677 | 0.01 | | 1 | 1 | 0 | 2018 | 2018 |
| Rheu | C00 | FOXJ3 | 22 | Q9UPW0 | forkhead box J3 | | 5 | 0. | 0. | 0.9 | 0.01 | | 1 | 1 | 2 | 2013 | 20 |

| | | | | | | | | | | | | | | | | | |
|---|---|---|---|---|---|---|---|---|---|---|---|---|---|---|---|---|---|
| matoid Arthritis | 03873 | | 887 | | | | | 89 | 192 | 9598 | | | | | | | 13 |
| Rheumatoid Arthritis | C0003873 | SACM1L | 22908 | Q9NTJ5 | SAC1 like phosphatidylinositide phosphatase | | 31 | 0.682 | 0.5 | 0.69536 | 0.01 | | 1 | 1 | 0 | 1989 | 1989 |
| Rheumatoid Arthritis | C0003873 | CD93 | 22918 | Q9NPY3 | CD93 molecule | Calcium-binding protein | 36 | 0.663 | 0.5 | 1.98E-10 | 0.01 | | 1 | 1 | 0 | 1993 | 1993 |
| Rheumatoid Arthritis | C0003873 | SNW1 | 22938 | Q13573 | SNW domain containing 1 | Transcription factor | 29 | 0.682 | 0.423 | 0.99961 | 0.01 | | 1 | 1 | 0 | 2019 | 2019 |
| Rheumatoid Arthritis | C0003873 | P2RX2 | 22953 | Q9UBL9 | purinergic receptor P2X 2 | Ion channel | 99 | 0.585 | 0.769 | 9.65E-13 | 0.01 | | 1 | 1 | 0 | 2010 | 2010 |
| Rheumatoid Arthritis | C0003873 | FOXJ1 | 2302 | Q92949 | forkhead box J1 | | 61 | 0.619 | 0.577 | 0.96901 | 0.01 | | 1 | 1 | 0 | 2007 | 2007 |
| Rheumatoid Ar | C0003873 | MAST3 | 23031 | O60307 | microtubule associated serine/th | Kinase | 10 | 0.931 | 0.154 | 0.99991 | 0.01 | | 1 | 1 | 0 | 2019 | 2019 |

| | | | | | | | | | | | | | | | | | |
|---|---|---|---|---|---|---|---|---|---|---|---|---|---|---|---|---|---|
| thritis | | | | | reonine kinase 3 | | | | | | | | | | | | |
| Rheumatoid Arthritis | C0003873 | ZNF292 | 23036 | O60281 | zinc finger protein 292 | Enzyme | 53 | 0.695 | 0.423 | 1 | 0.01 | | 1 | 1 | 0 | 2015 | 2015 |
| Rheumatoid Arthritis | C0003873 | MON2 | 23041 | Q7Z3U7 | MON2 homolog, regulator of endosome-to-Golgi trafficking | | 15 | 0.751 | 0.308 | 0.59388 | 0.01 | | 1 | 1 | 0 | 2014 | 2014 |
| Rheumatoid Arthritis | C0003873 | SMG1 | 23049 | Q96Q15 | SMG1 nonsense mediated mRNA decay associated PI3K related kinase | Kinase | 90 | 0.575 | 0.808 | 1 | 0.01 | | 1 | 1 | 0 | 2001 | 2001 |
| Rheumatoid Arthritis | C0003873 | ZNF423 | 23090 | Q2M1K9 | zinc finger protein 423 | | 97 | 0.592 | 0.808 | 0.99996 | 0.01 | | 1 | 1 | 0 | 2010 | 2010 |
| Rheumatoid Arthritis | C0003873 | TAB2 | 23118 | Q9NYJ8 | TGF-beta activated kinase 1 (MAP3K7) binding protein 2 | | 72 | 0.617 | 0.654 | 0.99998 | 0.01 | | 1 | 1 | 1 | 2006 | 2006 |
| Rheumatoid Arthritis | C0003873 | CIC | 23152 | Q96RK0 | capicua transcriptional repressor | | 141 | 0.523 | 0.692 | 0.99999 | 0.01 | | 1 | 1 | 0 | 2010 | 2010 |
| Rheu | C00 | FLNA | 23 | P21333 | filamin A | | 571 | 0. | 0. | 1 | 0.01 | | 1 | 1 | 0 | 2017 | 20 |

| | | | | | | | | | | | | | | | | | |
|---|---|---|---|---|---|---|---|---|---|---|---|---|---|---|---|---|---|
| matoid Arthritis | 03873 | | 16 | | | | | 419 | 846 | | | | | | | | 17 |
| Rheumatoid Arthritis | C0003873 | PHLDB1 | 23187 | Q86UU1 | pleckstrin homology like domain family B member 1 | | 20 | 0.722 | 0.423 | 0.10667 | 0.01 | | 0 | 1 | 0 | 2017 | 2017 |
| Rheumatoid Arthritis | C0003873 | FLT3 | 2322 | P36888 | fms related receptor tyrosine kinase 3 | Kinase | 270 | 0.462 | 0.654 | 0.60777 | 0.01 | | 1 | 1 | 0 | 2019 | 2019 |
| Rheumatoid Arthritis | C0003873 | ARC | 23237 | Q7LC44 | activity regulated cytoskeleton associated protein | | 101 | 0.573 | 0.769 | 0.59433 | 0.01 | | 1 | 1 | 0 | 1997 | 1997 |
| Rheumatoid Arthritis | C0003873 | SATB2 | 23314 | Q9UPW6 | SATB homeobox 2 | Transcription factor | 233 | 0.503 | 0.769 | 0.99999 | 0.01 | | 1 | 1 | 0 | 2013 | 2013 |
| Rheumatoid Arthritis | C0003873 | SYNM | 23336 | O15061 | synemin | | 93 | 0.575 | 0.731 | 3.03E-24 | 0.01 | | 1 | 1 | 0 | 2019 | 2019 |
| Rheumatoid Arthritis | C0003873 | COTL1 | 23406 | Q14019 | coactosin like F-actin binding protein 1 | Cellular structure | 62 | 0.615 | 0.538 | 0.039077 | 0.01 | | 1 | 1 | 0 | 2009 | 2009 |

| | | | | | | | | | | | | | | | | | |
|---|---|---|---|---|---|---|---|---|---|---|---|---|---|---|---|---|---|
| Rheumatoid Arthritis | C0003873 | HARS2 | 23438 | P49590 | histidyl-tRNA synthetase 2, mitochondrial | Enzyme | 25 | 0.705 | 0.462 | 2.79E-05 | 0.01 | | 1 | 1 | 0 | 2006 | 2006 |
| Rheumatoid Arthritis | C0003873 | FOLR1 | 2348 | P15328 | folate receptor alpha | | 174 | 0.522 | 0.885 | 0.126442 | 0.01 | | 1 | 1 | 0 | 2017 | 2017 |
| Rheumatoid Arthritis | C0003873 | CABIN1 | 23523 | Q9Y6J0 | calcineurin binding protein 1 | Enzyme modulator | 136 | 0.536 | 0.846 | 3.28E-17 | 0.01 | | 1 | 1 | 0 | 2010 | 2010 |
| Rheumatoid Arthritis | C0003873 | SNAPIN | 23557 | O95295 | SNAP associated protein | | 4 | 0.931 | 0.192 | 0.001533 | 0.01 | | 1 | 1 | 0 | 2012 | 2012 |
| Rheumatoid Arthritis | C0003873 | DDAH2 | 23564 | O95865 | dimethylarginine dimethylaminohydrolase 2 | Enzyme | 36 | 0.67 | 0.538 | 0.013773 | 0.01 | | 0 | 1 | 0 | 2014 | 2014 |
| Rheumatoid Arthritis | C0003873 | LPAR3 | 23566 | Q9UBY5 | lysophosphatidic acid receptor 3 | G-protein coupled receptor | 194 | 0.509 | 0.923 | 0.67935 | 0.01 | | 1 | 1 | 0 | 2008 | 2008 |
| Rheumatoi | C0003 | FPR1 | 2357 | P21462 | formyl peptide receptor 1 | G-protein | 93 | 0.5 | 0.7 | | 0.01 | | 1 | 1 | 0 | 2008 | 2008 |

| | | | | | | | | | | | | | | | | | |
|---|---|---|---|---|---|---|---|---|---|---|---|---|---|---|---|---|---|
| d Arthritis | 873 | | | | | coupled receptor | | 76 | 69 | | | | | | | | |
| Rheumatoid Arthritis | C0003873 | DDAH1 | 23576 | O94760 | dimethylarginine dimethylaminohydrolase 1 | Enzyme | 73 | 0.604 | 0.615 | 0.002797 | 0.01 | | 0 | 1 | 0 | 2014 | 2014 |
| Rheumatoid Arthritis | C0003873 | FAM215A | 23591 | Q9Y5M1 | family with sequence similarity 215 member A | | 43 | 0.65 | 0.5 | | 0.01 | | 1 | 1 | 0 | 2013 | 2013 |
| Rheumatoid Arthritis | C0003873 | AMACR | 23600 | Q9UHK6 | alpha-methylacyl-CoA racemase | | 179 | 0.51 | 0.769 | 0.027363 | 0.01 | | 1 | 1 | 0 | 1998 | 1998 |
| Rheumatoid Arthritis | C0003873 | CLEC5A | 23601 | Q9NY25 | C-type lectin domain containing 5A | | 37 | 0.663 | 0.346 | 0.12568 | 0.01 | | 1 | 1 | 0 | 2014 | 2014 |
| Rheumatoid Arthritis | C0003873 | CD2AP | 23607 | Q9Y5K6 | CD2 associated protein | | 113 | 0.558 | 0.731 | 0.22989 | 0.01 | | 1 | 1 | 0 | 2018 | 2018 |
| Rheumatoid Arthritis | C0003873 | HSPBP1 | 23640 | Q9NZL4 | HSPA (Hsp70) binding protein 1 | | 14 | 0.861 | 0.269 | 0.057208 | 0.01 | | 1 | 1 | 0 | 2011 | 2011 |

| | | | | | | | | | | | | | | | | | |
|---|---|---|---|---|---|---|---|---|---|---|---|---|---|---|---|---|---|
| Rheumatoid Arthritis | C0003873 | PPP1R15A | 23645 | O75807 | protein phosphatase 1 regulatory subunit 15A | | 65 | 0.608 | 0.692 | 2.21E-12 | 0.01 | | 1 | 1 | 0 | 2016 | 2016 |
| Rheumatoid Arthritis | C0003873 | PLD3 | 23646 | Q8IV08 | phospholipase D family member 3 | | 29 | 0.7 | 0.462 | 1.75E-05 | 0.01 | | 1 | 1 | 0 | 2018 | 2018 |
| Rheumatoid Arthritis | C0003873 | BRD1 | 23774 | O95696 | bromodomain containing 1 | | 50 | 0.644 | 0.654 | 0.99949 | 0.01 | | 1 | 1 | 0 | 2018 | 2018 |
| Rheumatoid Arthritis | C0003873 | ALOX12 | 239 | P18054 | arachidonate 12-lipoxygenase, 12S type | Enzyme | 119 | 0.558 | 0.692 | 1.44E-15 | 0.01 | | 1 | 1 | 0 | 1997 | 1997 |
| Rheumatoid Arthritis | C0003873 | ABCA4 | 24 | P78363 | ATP binding cassette subfamily A member 4 | Transporter | 227 | 0.493 | 0.769 | 5.35E-48 | 0.01 | | 1 | 1 | 0 | 2014 | 2014 |
| Rheumatoid Arthritis | C0003873 | ALOX5AP | 241 | P20292 | arachidonate 5-lipoxygenase activating protein | Enzyme | 76 | 0.612 | 0.731 | 0.070729 | 0.01 | | 1 | 1 | 0 | 1995 | 1995 |
| Rheumatoid Ar | C0003873 | SPESP1 | 246777 | Q6UW49 | sperm equatorial segment protein 1 | | 25 | 0.711 | 0.538 | 0.39465 | 0.01 | | 1 | 1 | 0 | 2016 | 2016 |

| | | | | | | | | | | | | | | | | | |
|---|---|---|---|---|---|---|---|---|---|---|---|---|---|---|---|---|---|
| thritis | | | | | | | | | | | | | | | | | |
| Rheumatoid Arthritis | C0003873 | FTL | 2512 | P02792 | ferritin light chain | Storage | 135 | 0.559 | 0.692 | 1.66E-09 | 0.01 | | 1 | 1 | 0 | 1997 | 1997 |
| Rheumatoid Arthritis | C0003873 | FUT4 | 2526 | P22083 | fucosyltransferase 4 | Enzyme | 168 | 0.505 | 0.731 | 7.73E-06 | 0.01 | | 1 | 1 | 0 | 2016 | 2016 |
| Rheumatoid Arthritis | C0003873 | ZBTB38 | 253461 | Q8NAP3 | zinc finger and BTB domain containing 38 | Transcription factor | 60 | 0.663 | 0.615 | 0.99949 | 0.01 | | 1 | 1 | 0 | 2018 | 2018 |
| Rheumatoid Arthritis | C0003873 | EBF3 | 253738 | Q9H4W6 | EBF transcription factor 3 | | 124 | 0.595 | 0.731 | 0.99993 | 0.01 | | 1 | 1 | 0 | 2013 | 2013 |
| Rheumatoid Arthritis | C0003873 | TAC4 | 255061 | Q86UU9 | tachykinin precursor 4 | | 24 | 0.711 | 0.462 | 3.61E-05 | 0.01 | | 1 | 1 | 0 | 2019 | 2019 |
| Rheumatoid Arthritis | C0003873 | PCSK9 | 255738 | Q8NBP7 | proprotein convertase subtilisin/kexin type 9 | Enzyme | 257 | 0.482 | 0.808 | 2.71E-17 | 0.01 | | 1 | 1 | 0 | 2019 | 2019 |
| Rheumatoi | C0003 | CIZ1 | 257 | Q9ULV3 | CDKN1A interacting zinc finger protein 1 | | 80 | 0.6 | 0.5 | 0.063 | 0.01 | | 1 | 1 | 0 | 2016 | 2016 |

| | | | | | | | | | | | | | | | | | |
|---|---|---|---|---|---|---|---|---|---|---|---|---|---|---|---|---|---|
| d Arthritis | 873 | | 92 | | | | | 17 | | 172 | | | | | | | |
| Rheumatoid Arthritis | C0003873 | QPCT | 25797 | Q16769 | glutaminyl-peptide cyclotransferase | Enzyme | 94 | 0.568 | 0.731 | 1.2E-15 | 0.01 | | 1 | 1 | 0 | 2019 | 2019 |
| Rheumatoid Arthritis | C0003873 | PRDX5 | 25824 | P30044 | peroxiredoxin 5 | | 153 | 0.522 | 0.808 | 0.000179 | 0.01 | | 1 | 1 | 0 | 2007 | 2007 |
| Rheumatoid Arthritis | C0003873 | PART1 | 25859 | | prostate androgen-regulated transcript 1 | | 250 | 0.483 | 0.885 | | 0.01 | | 1 | 1 | 0 | 2017 | 2017 |
| Rheumatoid Arthritis | C0003873 | POT1 | 25913 | Q9NUX5 | protection of telomeres 1 | Nucleic acid binding | 107 | 0.56 | 0.731 | 0.85343 | 0.01 | | 1 | 1 | 0 | 2012 | 2012 |
| Rheumatoid Arthritis | C0003873 | NCR3 | 259197 | O14931 | natural cytotoxicity triggering receptor 3 | | 53 | 0.638 | 0.769 | 0.000368 | 0.01 | | 1 | 1 | 1 | 2016 | 2016 |
| Rheumatoid Arthritis | C0003873 | IL4I1 | 259307 | Q96RQ9 | interleukin 4 induced 1 | | 36 | 0.686 | 0.5 | 0.00396 | 0.01 | | 1 | 1 | 0 | 2007 | 2007 |
| Rheu | C00 | IBTK | 25 | Q9P2D0 | inhibitor of Bruton | | 33 | 0. | 0. | 0.0 | 0.01 | | 1 | 1 | 0 | 2019 | 20 |

| | | | | | | | | | | | | | | | | | |
|---|---|---|---|---|---|---|---|---|---|---|---|---|---|---|---|---|---|
| matoid Arthritis | 03873 | | 998 | | tyrosine kinase | | | 678 | 538 | 022214 | | | | | | | 19 |
| Rheumatoid Arthritis | C0003873 | LRIG1 | 26018 | Q96JA1 | leucine rich repeats and immunoglobulin like domains 1 | Receptor | 83 | 0.601 | 0.538 | 0.0350031 | 0.01 | | 1 | 1 | 0 | 2017 | 2017 |
| Rheumatoid Arthritis | C0003873 | PYDC1 | 260434 | Q8WXC3 | pyrin domain containing 1 | | 13 | 0.805 | 0.192 | | 0.01 | | 1 | 1 | 0 | 2015 | 2015 |
| Rheumatoid Arthritis | C0003873 | LRIT1 | 26103 | Q9P2V4 | leucine rich repeat, Ig-like and transmembrane domains 1 | | 53 | 0.641 | 0.615 | 4.59E-15 | 0.01 | | 1 | 1 | 0 | 2002 | 2002 |
| Rheumatoid Arthritis | C0003873 | KIFBP | 26128 | Q96EK5 | kinesin family binding protein | | 83 | 0.615 | 0.731 | 0.0756682 | 0.01 | | 1 | 1 | 0 | 2019 | 2019 |
| Rheumatoid Arthritis | C0003873 | GARS1 | 2617 | P41250 | glycyl-tRNA synthetase 1 | Enzyme | 73 | 0.623 | 0.5 | 0.3065 | 0.01 | | 1 | 1 | 0 | 2018 | 2018 |
| Rheumatoid Ar | C0003873 | AMD1 | 262 | P17707 | adenosylmethionine decarboxylase 1 | Enzyme | 84 | 0.593 | 0.577 | 0.98227 | 0.01 | | 1 | 1 | 0 | 2019 | 2019 |

| | | | | | | | | | | | | | | | | | |
|---|---|---|---|---|---|---|---|---|---|---|---|---|---|---|---|---|---|
| thritis | | | | | | | | | | | | | | | | | |
| Rheumatoid Arthritis | C0003873 | GATA1 | 2623 | P15976 | GATA binding protein 1 | | 264 | 0.483 | 0.808 | 0.94798 | 0.01 | | 1 | 1 | 0 | 2018 | 2018 |
| Rheumatoid Arthritis | C0003873 | FBXO2 | 26232 | Q9UK22 | F-box protein 2 | | 11 | 0.805 | 0.346 | 0.000215 | 0.01 | | 1 | 1 | 0 | 2013 | 2013 |
| Rheumatoid Arthritis | C0003873 | GATA2 | 2624 | P23769 | GATA binding protein 2 | | 229 | 0.496 | 0.654 | 0.97927 | 0.01 | | 1 | 1 | 0 | 2019 | 2019 |
| Rheumatoid Arthritis | C0003873 | CLEC4E | 26253 | Q9ULY5 | C-type lectin domain family 4 member E | Receptor | 35 | 0.695 | 0.5 | 1.14E-05 | 0.01 | | 0 | 1 | 1 | 2012 | 2012 |
| Rheumatoid Arthritis | C0003873 | GATA4 | 2626 | P43694 | GATA binding protein 4 | | 336 | 0.458 | 0.846 | 0.49308 | 0.01 | | 1 | 1 | 0 | 2018 | 2018 |
| Rheumatoid Arthritis | C0003873 | FGF21 | 26291 | Q9NSA1 | fibroblast growth factor 21 | Signaling | 236 | 0.485 | 0.769 | 0.029439 | 0.01 | | 1 | 1 | 0 | 2017 | 2017 |
| Rheumatoi | C0003 | AMD1P2 | 263 | | adenosylmethionine decarboxylase | | 71 | 0.6 | 0.5 | | 0.01 | | 1 | 1 | 0 | 2019 | 2019 |

| | | | | | | | | | | | | | | | | | |
|---|---|---|---|---|---|---|---|---|---|---|---|---|---|---|---|---|---|
| d Arthritis | 873 | | | | 1 pseudogene 2 | | | 08 | 38 | | | | | | | | |
| Rheumatoid Arthritis | C0003873 | GCHFR | 2644 | P30047 | GTP cyclohydrolase I feedback regulator | | 89 | 0.579 | 0.808 | 0.0482251 | 0.01 | | 1 | 1 | 0 | 2015 | 2015 |
| Rheumatoid Arthritis | C0003873 | GCK | 2645 | P35557 | glucokinase | Kinase | 210 | 0.523 | 0.808 | 0.634664 | 0.01 | | 1 | 1 | 0 | 2013 | 2013 |
| Rheumatoid Arthritis | C0003873 | KAT2A | 2648 | Q92830 | lysine acetyltransferase 2A | | 66 | 0.61 | 0.731 | 0.57331 | 0.01 | | 1 | 1 | 0 | 2017 | 2017 |
| Rheumatoid Arthritis | C0003873 | SLC17A5 | 26503 | Q9NRA2 | solute carrier family 17 member 5 | Transporter | 252 | 0.491 | 0.808 | 6.85E-10 | 0.01 | | 1 | 1 | 0 | 2019 | 2019 |
| Rheumatoid Arthritis | C0003873 | GCY | 2656 | | growth control, Y chromosome influenced | | 44 | 0.631 | 0.615 | | 0.01 | | 1 | 1 | 0 | 2017 | 2017 |
| Rheumatoid Arthritis | C0003873 | GDF2 | 2658 | Q9UK05 | growth differentiation factor 2 | Signaling | 152 | 0.529 | 0.731 | 0.002466 | 0.01 | | 1 | 1 | 0 | 2019 | 2019 |

| | | | | | | | | | | | | | | | | | |
|---|---|---|---|---|---|---|---|---|---|---|---|---|---|---|---|---|---|
| Rheumatoid Arthritis | C0003873 | GREM1 | 26585 | O60565 | gremlin 1, DAN family BMP antagonist | | 179 | 0.508 | 0.808 | 0.47436 | 0.01 | | 1 | 1 | 0 | 2016 | 2016 |
| Rheumatoid Arthritis | C0003873 | AMH | 268 | P03971 | anti-Mullerian hormone | | 242 | 0.476 | 0.846 | 3.4E-12 | 0.01 | | 1 | 1 | 0 | 2019 | 2019 |
| Rheumatoid Arthritis | C0003873 | B4GALT1 | 2683 | P15291 | beta-1,4-galactosyltransferase 1 | Enzyme | 45 | 0.674 | 0.538 | 0.037435 | 0.01 | | 1 | 1 | 0 | 2017 | 2017 |
| Rheumatoid Arthritis | C0003873 | RNU1-1 | 26871 | | RNA, U1 small nuclear 1 | | 66 | 0.608 | 0.731 | | 0.01 | | 0 | 1 | 0 | 2006 | 2006 |
| Rheumatoid Arthritis | C0003873 | GHSR | 2693 | Q92847 | growth hormone secretagogue receptor | G-protein coupled receptor | 171 | 0.528 | 0.769 | 0.006583 | 0.01 | | 0 | 1 | 0 | 2010 | 2010 |
| Rheumatoid Arthritis | C0003873 | SMR3A | 26952 | Q99954 | submaxillary gland androgen regulated protein 3A | | 19 | 0.751 | 0.538 | 0.39147 | 0.01 | | 1 | 1 | 0 | 2017 | 2017 |
| Rheumatoi | C0003 | GJA1 | 2697 | P17302 | gap junction protein alpha 1 | Cell-cell | 662 | 0.3 | 0.8 | 0.155 | 0.01 | | 1 | 1 | 0 | 2020 | 2020 |

| | | | | | | | | | | | | | | | | | |
|---|---|---|---|---|---|---|---|---|---|---|---|---|---|---|---|---|---|
| d Arthritis | 873 | | | | | junction | | 93 | 85 | 23 | | | | | | | |
| Rheumatoid Arthritis | C0003873 | AMPD1 | 270 | P23109 | adenosine monophosphate deaminase 1 | Enzyme | 106 | 0.573 | 0.692 | 1.91E-21 | 0.01 | | 1 | 1 | 1 | 2006 | 2006 |
| Rheumatoid Arthritis | C0003873 | SIGLEC7 | 27036 | Q9Y286 | sialic acid binding Ig like lectin 7 | Receptor | 137 | 0.535 | 0.846 | 2.39E-07 | 0.01 | | 1 | 1 | 0 | 1992 | 1992 |
| Rheumatoid Arthritis | C0003873 | LAT | 27040 | O43561 | linker for activation of T cells | | 124 | 0.548 | 0.731 | 0.009238 | 0.01 | | 1 | 1 | 0 | 2018 | 2018 |
| Rheumatoid Arthritis | C0003873 | PELP1 | 27043 | Q8IZL8 | proline, glutamate and leucine rich protein 1 | | 64 | 0.612 | 0.577 | 0.99998 | 0.01 | | 1 | 1 | 0 | 2003 | 2003 |
| Rheumatoid Arthritis | C0003873 | DKK2 | 27123 | Q9UBU2 | dickkopf WNT signaling pathway inhibitor 2 | | 74 | 0.599 | 0.692 | 0.75318 | 0.01 | | 1 | 1 | 0 | 2014 | 2014 |
| Rheumatoid Arthritis | C0003873 | IL36B | 27177 | Q9NZH7 | interleukin 36 beta | | 5 | 0.861 | 0.154 | 0.003298 | 0.01 | | 1 | 1 | 0 | 2006 | 2006 |

| | | | | | | | | | | | | | | | | | |
|---|---|---|---|---|---|---|---|---|---|---|---|---|---|---|---|---|---|
| Rheumatoid Arthritis | C0003873 | SIGLEC9 | 27180 | Q9Y336 | sialic acid binding Ig like lectin 9 | Receptor | 36 | 0.666 | 0.423 | 2.53E-05 | 0.01 | | 1 | 1 | 0 | 2017 | 2017 |
| Rheumatoid Arthritis | C0003873 | GCLC | 27729 | P48506 | glutamate-cysteine ligase catalytic subunit | Enzyme | 198 | 0.509 | 0.885 | 0.42718 | 0.01 | | 1 | 1 | 0 | 1999 | 1999 |
| Rheumatoid Arthritis | C0003873 | BMP10 | 27302 | O95393 | bone morphogenetic protein 10 | Signaling | 32 | 0.674 | 0.5 | 0.90838 | 0.01 | | 1 | 1 | 0 | 2019 | 2019 |
| Rheumatoid Arthritis | C0003873 | RABGEF1 | 27342 | Q9UJ41 | RAB guanine nucleotide exchange factor 1 | Enzyme modulator | 122 | 0.55 | 0.731 | 0.000631 | 0.01 | | 1 | 1 | 0 | 2006 | 2006 |
| Rheumatoid Arthritis | C0003873 | GLI1 | 2735 | P08151 | GLI family zinc finger 1 | | 369 | 0.431 | 0.846 | 7.66E-14 | 0.01 | | 1 | 1 | 0 | 2014 | 2014 |
| Rheumatoid Arthritis | C0003873 | APOBEC3C | 27350 | Q9NRW3 | apolipoprotein B mRNA editing enzyme catalytic subunit 3C | Enzyme | 27 | 0.722 | 0.538 | 0.026691 | 0.01 | | 1 | 1 | 0 | 2017 | 2017 |
| Rheumatoid Ar | C0003873 | GNA12 | 2768 | Q03113 | G protein subunit alpha 12 | Enzyme modul | 130 | 0.547 | 0.769 | 0.96994 | 0.01 | | 1 | 1 | 0 | 2014 | 2014 |

| | | | | | | | | | | | | | | | | | |
|---|---|---|---|---|---|---|---|---|---|---|---|---|---|---|---|---|---|
| thritis | | | | | | ator | | | | | | | | | | | |
| Rheumatoid Arthritis | C0003873 | GNAQ | 2776 | P50148 | G protein subunit alpha q | Enzyme modulator | 219 | 0.516 | 0.808 | 0.98069 | 0.01 | | 0 | 1 | 0 | 2013 | 2013 |
| Rheumatoid Arthritis | C0003873 | GNB3 | 2784 | P16520 | G protein subunit beta 3 | Enzyme | 192 | 0.518 | 0.808 | 3.33E-11 | 0.01 | | 1 | 1 | 1 | 2009 | 2009 |
| Rheumatoid Arthritis | C0003873 | GNS | 2799 | P15586 | glucosamine (N-acetyl)-6-sulfatase | Enzyme | 51 | 0.705 | 0.538 | 0.000564 | 0.01 | | 1 | 1 | 0 | 2017 | 2017 |
| Rheumatoid Arthritis | C0003873 | GPC1 | 2817 | P35052 | glypican 1 | | 96 | 0.587 | 0.731 | 0.015547 | 0.01 | | 1 | 1 | 0 | 2005 | 2005 |
| Rheumatoid Arthritis | C0003873 | GPLD1 | 2822 | P80108 | glycosylphosphatidylinositol specific phospholipase D1 | | 44 | 0.656 | 0.577 | 1.59E-15 | 0.01 | | 1 | 1 | 0 | 2011 | 2011 |
| Rheumatoid Arthritis | C0003873 | IFNL2 | 282616 | Q8IZJ0 | interferon lambda 2 | | 38 | 0.666 | 0.615 | 4.99E-08 | 0.01 | | 1 | 1 | 0 | 2017 | 2017 |
| Rheumatoi | C0003 | GPR3 | 2827 | P46089 | G protein-coupled | G-protein | 18 | 0.7 | 0.5 | 0.000 | 0.01 | | 1 | 1 | 0 | 2019 | 2019 |

| | | | | | | | | | | | | | | | | | |
|---|---|---|---|---|---|---|---|---|---|---|---|---|---|---|---|---|---|
| d Arthritis | 873 | | | | receptor 3 | coupled receptor | | 43 | | 263 | | | | | | | |
| Rheumatoid Arthritis | C0003873 | ANG | 283 | P03950 | angiogenin | | 185 | 0.511 | 0.769 | 0.28738 | 0.01 | | 1 | 1 | 0 | 2003 | 2003 |
| Rheumatoid Arthritis | C0003873 | H19 | 283120 | | H19 imprinted maternally expressed transcript | | 242 | 0.494 | 0.923 | | 0.01 | | 1 | 1 | 2 | 2003 | 2003 |
| Rheumatoid Arthritis | C0003873 | NEAT1 | 283131 | | nuclear paraspeckle assembly transcript 1 | | 179 | 0.502 | 0.769 | | 0.01 | | 1 | 1 | 0 | 2019 | 2019 |
| Rheumatoid Arthritis | C0003873 | UTS2R | 2837 | Q9UKP6 | urotensin 2 receptor | G-protein coupled receptor | 98 | 0.566 | 0.692 | 2.32E-05 | 0.01 | | 1 | 1 | 0 | 2018 | 2018 |
| Rheumatoid Arthritis | C0003873 | IGHV5-78 | 28387 | | immunoglobulin heavy variable 5-78 (pseudogene) | | 5 | 0.839 | 0.231 | | 0.01 | | 1 | 1 | 0 | 2001 | 2001 |
| Rheumatoid Ar | C0003873 | IGHV1-69 | 28461 | P01742 | immunoglobulin heavy variable 1-69 | | 19 | 0.729 | 0.462 | | 0.01 | | 1 | 1 | 0 | 2002 | 2002 |

| | | | | | | | | | | | | | | | | | |
|---|---|---|---|---|---|---|---|---|---|---|---|---|---|---|---|---|---|
| thritis | | | | | | | | | | | | | | | | | |
| Rheumatoid Arthritis | C0003873 | MIF-AS1 | 284889 | | MIF antisense RNA 1 | | 38 | 0.663 | 0.577 | | 0.01 | | 1 | 1 | 2 | 1999 | 1999 |
| Rheumatoid Arthritis | C0003873 | GPER1 | 2852 | Q99527 | G protein-coupled estrogen receptor 1 | G-protein coupled receptor | 180 | 0.505 | 0.731 | 0.57434 | 0.01 | | 1 | 1 | 0 | 2018 | 2018 |
| Rheumatoid Arthritis | C0003873 | CYP4V2 | 285440 | Q6ZWL3 | cytochrome P450 family 4 subfamily V member 2 | Enzyme | 42 | 0.67 | 0.577 | 2.2E-10 | 0.01 | | 1 | 1 | 0 | 2019 | 2019 |
| Rheumatoid Arthritis | C0003873 | TRBV28 | 28559 | A0A5B6 | T cell receptor beta variable 28 | | 4 | 0.89 | 0.192 | | 0.01 | | 1 | 1 | 0 | 2003 | 2003 |
| Rheumatoid Arthritis | C0003873 | TRBV27 | 28560 | A0A0K0K1C4 | T cell receptor beta variable 27 | | 5 | 0.861 | 0.231 | | 0.01 | | 1 | 1 | 0 | 1998 | 1998 |
| Rheumatoid Arthritis | C0003873 | TRBV20-1 | 28567 | A0A075B6N2 | T cell receptor beta variable 20-1 | | 7 | 0.821 | 0.346 | | 0.01 | | 1 | 1 | 0 | 1993 | 1993 |

| | | | | | | | | | | | | | | | | | |
|---|---|---|---|---|---|---|---|---|---|---|---|---|---|---|---|---|---|
| Rheumatoid Arthritis | C0003873 | TRBV14 | 28573 | A0A5B0 | T cell receptor beta variable 14 | | 4 | 0.89 | 0.154 | | 0.01 | | 1 | 1 | 0 | 1998 | 1998 |
| Rheumatoid Arthritis | C0003873 | TRBV6-7 | 28600 | | T cell receptor beta variable 6-7 (non-functional) | | 1 | 1 | 0.115 | | 0.01 | | 1 | 1 | 0 | 1996 | 1996 |
| Rheumatoid Arthritis | C0003873 | TRBV3-1 | 28619 | A0A576 | T cell receptor beta variable 3-1 | | 4 | 0.89 | 0.192 | | 0.01 | | 1 | 1 | 0 | 2003 | 2003 |
| Rheumatoid Arthritis | C0003873 | TRBV2 | 28620 | A0A1B0GX68 | T cell receptor beta variable 2 | | 4 | 0.861 | 0.269 | | 0.01 | | 1 | 1 | 0 | 1993 | 1993 |
| Rheumatoid Arthritis | C0003873 | GPR39 | 2863 | O43194 | G protein-coupled receptor 39 | G-protein coupled receptor | 45 | 0.65 | 0.615 | 2.81E-09 | 0.01 | | 1 | 1 | 0 | 2019 | 2019 |
| Rheumatoid Arthritis | C0003873 | TRBC2 | 28638 | A0A5B9 | T cell receptor beta constant 2 | | 4 | 0.931 | 0.192 | | 0.01 | | 1 | 1 | 0 | 1994 | 1994 |
| Rheumatoi | C0003 | TRAV5 | 286 | A0A0B4J249 | T cell receptor alpha variable 5 | | 1 | 1 | 0.1 | | 0.01 | | 1 | 1 | 0 | 1998 | 1998 |

| | | | | | | | | | | | | | | | | | |
|---|---|---|---|---|---|---|---|---|---|---|---|---|---|---|---|---|---|
| d Arthritis | 873 | | 88 | | | | | | 15 | | | | | | | | |
| Rheumatoid Arthritis | C0003873 | GRK5 | 2869 | P34947 | G protein-coupled receptor kinase 5 | Kinase | 86 | 0.588 | 0.769 | 0.67567 | 0.01 | | 1 | 1 | 0 | 1999 | 1999 |
| Rheumatoid Arthritis | C0003873 | GRB2 | 2885 | P62993 | growth factor receptor bound protein 2 | | 122 | 0.546 | 0.769 | 0.93934 | 0.01 | | 1 | 1 | 0 | 2003 | 2003 |
| Rheumatoid Arthritis | C0003873 | IGKV3D-15 | 28875 | A0A087WSY6 | immunoglobulin kappa variable 3D-15 (gene/pseudogene) | Immune response | 4 | 0.861 | 0.192 | | 0.01 | | 1 | 1 | 0 | 1998 | 1998 |
| Rheumatoid Arthritis | C0003873 | GRIA1 | 2890 | P42261 | glutamate ionotropic receptor AMPA type subunit 1 | Ion channel | 126 | 0.57 | 0.577 | 0.99869 | 0.01 | | 1 | 1 | 0 | 2019 | 2019 |
| Rheumatoid Arthritis | C0003873 | IGKV1D-8 | 28904 | A0A087WSZ0 | immunoglobulin kappa variable 1D-8 | Immune response | 4 | 0.861 | 0.192 | | 0.01 | | 1 | 1 | 0 | 1998 | 1998 |
| Rheumatoid Arthritis | C0003873 | IGKV3-20 | 28912 | P01619 | immunoglobulin kappa variable 3-20 | Immune response | 19 | 0.736 | 0.308 | | 0.01 | | 1 | 1 | 0 | 1998 | 1998 |

| | | | | | | | | | | | | | | | | | |
|---|---|---|---|---|---|---|---|---|---|---|---|---|---|---|---|---|---|
| Rheumatoid Arthritis | C0003873 | IGKV2-29 | 28920 | A2NJV5 | immunoglobulin kappa variable 2-29 (gene/pseudogene) | Immune response | 12 | 0.769 | 0.231 | | 0.01 | | 1 | 1 | 0 | 1998 | 1998 |
| Rheumatoid Arthritis | C0003873 | REM1 | 28954 | O75628 | RRAD and GEM like GTPase 1 | | 177 | 0.531 | 0.885 | 5.34E-08 | 0.01 | | 1 | 1 | 0 | 2018 | 2018 |
| Rheumatoid Arthritis | C0003873 | LAMTOR2 | 28956 | Q9Y2Q5 | late endosomal/lysosomal adaptor, MAPK and MTOR activator 2 | | 129 | 0.538 | 0.808 | 0.19816 | 0.01 | | 1 | 1 | 0 | 1990 | 1990 |
| Rheumatoid Arthritis | C0003873 | RGCC | 28984 | Q9H4X1 | regulator of cell cycle | | 81 | 0.595 | 0.769 | 0.004815 | 0.01 | | 1 | 1 | 0 | 2018 | 2018 |
| Rheumatoid Arthritis | C0003873 | HIPK2 | 28996 | Q9H2X6 | homeodomain interacting protein kinase 2 | Kinase | 102 | 0.566 | 0.731 | 1 | 0.01 | | 0 | 1 | 0 | 2019 | 2019 |
| Rheumatoid Arthritis | C0003873 | GRIK4 | 2900 | Q16099 | glutamate ionotropic receptor kainate type subunit 4 | Ion channel | 41 | 0.678 | 0.423 | 0.11126 | 0.01 | | 1 | 1 | 0 | 2014 | 2014 |
| Rheumatoid | C0003873 | STXBP6 | 29091 | Q8NFX7 | syntaxin binding protein 6 | Transporter | 8 | 0.805 | 0.269 | 7.87E- | 0.01 | | 1 | 1 | 0 | 2012 | 2012 |

| | | | | | | | | | | | | | | | | | |
|---|---|---|---|---|---|---|---|---|---|---|---|---|---|---|---|---|---|
| Arthritis | | | | | | | | | | 05 | | | | | | | |
| Rheumatoid Arthritis | C0003873 | UHRF1 | 29128 | Q96T88 | ubiquitin like with PHD and ring finger domains 1 | | 125 | 0.54 | 0.692 | | 0.01 | | 1 | 1 | 0 | 2016 | 2016 |
| Rheumatoid Arthritis | C0003873 | CXCL3 | 2921 | P19876 | C-X-C motif chemokine ligand 3 | Signaling | 47 | 0.65 | 0.692 | 0.000302 | 0.01 | | 1 | 1 | 0 | 2017 | 2017 |
| Rheumatoid Arthritis | C0003873 | PDIA3 | 2923 | P30101 | protein disulfide isomerase family A member 3 | | 100 | 0.566 | 0.769 | 0.99608 | 0.01 | | 1 | 1 | 0 | 2016 | 2016 |
| Rheumatoid Arthritis | C0003873 | GRPR | 2925 | P30550 | gastrin releasing peptide receptor | G-protein coupled receptor | 85 | 0.595 | 0.538 | 0.22444 | 0.01 | | 1 | 1 | 0 | 2018 | 2018 |
| Rheumatoid Arthritis | C0003873 | GSN | 2934 | P06396 | gelsolin | Cellular structure | 262 | 0.475 | 0.885 | 3.89E-11 | 0.01 | | 1 | 1 | 0 | 2008 | 2008 |
| Rheumatoid Arthritis | C0003873 | GTS | 2973 | | Gilles de la Tourette syndrome | | 32 | 0.682 | 0.615 | | 0.01 | | 1 | 1 | 0 | 2018 | 2018 |

| | | | | | | | | | | | | | | | | | |
|---|---|---|---|---|---|---|---|---|---|---|---|---|---|---|---|---|---|
| Rheumatoid Arthritis | C0003873 | GYS1 | 2997 | P13807 | glycogen synthase 1 | | 26 | 0.722 | 0.385 | 0.000291 | 0.01 | | 1 | 1 | 0 | 2018 | 2018 |
| Rheumatoid Arthritis | C0003873 | EFEMP2 | 30008 | O95967 | EGF containing fibulin extracellular matrix protein 2 | Calcium-binding protein | 126 | 0.559 | 0.692 | 0.008194 | 0.01 | | 1 | 1 | 0 | 2006 | 2006 |
| Rheumatoid Arthritis | C0003873 | GZMM | 3004 | P51124 | granzyme M | Enzyme | 36 | 0.686 | 0.577 | 0.011275 | 0.01 | | 1 | 1 | 0 | 2020 | 2020 |
| Rheumatoid Arthritis | C0003873 | ANXA2 | 302 | P07355 | annexin A2 | | 294 | 0.453 | 0.808 | 9.77E-05 | 0.01 | | 1 | 1 | 0 | 2019 | 2019 |
| Rheumatoid Arthritis | C0003873 | HAGH | 3029 | Q16775 | hydroxyacylglutathione hydrolase | | 20 | 0.729 | 0.462 | 0.000177 | 0.01 | | 1 | 1 | 0 | 1990 | 1990 |
| Rheumatoid Arthritis | C0003873 | HARS1 | 3035 | P12081 | histidyl-tRNA synthetase 1 | Enzyme | 127 | 0.569 | 0.808 | 2.38E-06 | 0.01 | | 1 | 1 | 0 | 2006 | 2006 |
| Rheumatoi | C0003 | HAS1 | 3036 | Q92839 | hyaluronan synthase 1 | | 45 | 0.67 | 0.5 | 1.31E | 0.01 | | 1 | 1 | 0 | 2004 | 2004 |

| | | | | | | | | | | | | | | | | | |
|---|---|---|---|---|---|---|---|---|---|---|---|---|---|---|---|---|---|
| d Arthritis | 873 | | | | | | | | 77 | -12 | | | | | | | |
| Rheumatoid Arthritis | C0003873 | HAS3 | 3038 | O00219 | hyaluronan synthase 3 | | 51 | 0.644 | 0.692 | 0.88316 | 0.01 | | 1 | 1 | 0 | 2004 | 2004 |
| Rheumatoid Arthritis | C0003873 | HCRTR1 | 3061 | O43613 | hypocretin receptor 1 | G-protein coupled receptor | 71 | 0.623 | 0.654 | 0.000174 | 0.01 | | 1 | 1 | 0 | 2018 | 2018 |
| Rheumatoid Arthritis | C0003873 | HDC | 3067 | P19113 | histidine decarboxylase | | 92 | 0.584 | 0.692 | 0.004109 | 0.01 | | 1 | 1 | 0 | 2004 | 2004 |
| Rheumatoid Arthritis | C0003873 | ANXA4 | 307 | P09525 | annexin A4 | | 74 | 0.599 | 0.692 | 1.19E-08 | 0.01 | | 1 | 1 | 0 | 2017 | 2017 |
| Rheumatoid Arthritis | C0003873 | HEXA | 3073 | P06865 | hexosaminidase subunit alpha | Enzyme | 79 | 0.633 | 0.615 | 1.34E-11 | 0.01 | | 1 | 1 | 0 | 2002 | 2002 |
| Rheumatoid Arthritis | C0003873 | HEXB | 3074 | P07686 | hexosaminidase subunit beta | Enzyme | 62 | 0.644 | 0.654 | 3.49E-10 | 0.01 | | 1 | 1 | 0 | 2002 | 2002 |

| | | | | | | | | | | | | | | | | | |
|---|---|---|---|---|---|---|---|---|---|---|---|---|---|---|---|---|---|
| Rheumatoid Arthritis | C0003873 | ANXA5 | 308 | P08758 | annexin A5 | | 283 | 0.458 | 0.769 | 3.8E-15 | 0.01 | | 1 | 1 | 0 | 2013 | 2013 |
| Rheumatoid Arthritis | C0003873 | KCNIP3 | 30818 | Q9Y2W7 | potassium voltage-gated channel interacting protein 3 | Ion channel | 60 | 0.626 | 0.654 | 0.037604 | 0.01 | | 1 | 1 | 0 | 2016 | 2016 |
| Rheumatoid Arthritis | C0003873 | CD209 | 30835 | Q9NNX6 | CD209 molecule | Receptor | 57 | 0.626 | 0.769 | 2.74E-13 | 0.01 | | 1 | 1 | 0 | 2009 | 2009 |
| Rheumatoid Arthritis | C0003873 | TAX1BP3 | 30851 | O14907 | Tax1 binding protein 3 | | 27 | 0.716 | 0.615 | 0.005243 | 0.01 | | 1 | 1 | 0 | 2019 | 2019 |
| Rheumatoid Arthritis | C0003873 | HK1 | 3098 | P19367 | hexokinase 1 | Kinase | 161 | 0.542 | 0.731 | 0.91459 | 0.01 | | 1 | 1 | 0 | 2019 | 2019 |
| Rheumatoid Arthritis | C0003873 | HK2 | 3099 | P52789 | hexokinase 2 | Kinase | 152 | 0.515 | 0.769 | 0.004589 | 0.01 | | 1 | 1 | 0 | 2018 | 2018 |
| Rheumatoid Ar | C0003873 | HLA-DRB2 | 3124 | | major histocompatibility complex, class II, DR beta 2 (pseudogene) | | 8 | 0.861 | 0.269 | | 0.01 | | 1 | 1 | 0 | 2018 | 2018 |

| | | | | | | | | | | | | | | | | |
|---|---|---|---|---|---|---|---|---|---|---|---|---|---|---|---|---|
| thritis | | | | | | | | | | | | | | | | |
| Rheumatoid Arthritis | C0003873 | HLA-DRB6 | 3128 | | major histocompatibility complex, class II, DR beta 6 (pseudogene) | 9 | 0.821 | 0.308 | | 0.01 | | 1 | 1 | 0 | 2012 | 2012 |
| Rheumatoid Arthritis | C0003873 | HLA-E | 3133 | P13747 | major histocompatibility complex, class I, E | 118 | 0.545 | 0.769 | 0.036773 | 0.01 | | 1 | 1 | 0 | 2015 | 2015 |
| Rheumatoid Arthritis | C0003873 | HMGCR | 3156 | P04035 | 3-hydroxy-3-methylglutaryl-CoA reductase | 154 | 0.529 | 0.692 | 0.998661 | 0.01 | | 1 | 1 | 0 | 2006 | 2006 |
| Rheumatoid Arthritis | C0003873 | HMMR | 3161 | O75330 | hyaluronan mediated motility receptor | 117 | 0.544 | 0.615 | 5.52E-21 | 0.01 | | 1 | 1 | 0 | 2018 | 2018 |
| Rheumatoid Arthritis | C0003873 | APAF1 | 317 | O14727 | apoptotic peptidase activating factor 1 | 145 | 0.525 | 0.769 | 9.62E-06 | 0.01 | | 1 | 1 | 0 | 2017 | 2017 |
| Rheumatoid Arthritis | C0003873 | HNRNPC | 3183 | P07910 | heterogeneous nuclear ribonucleoprotein C | 61 | 0.606 | 0.538 | 0.97772 | 0.01 | | 1 | 1 | 0 | 1996 | 1996 |
| Rheumatoi | C0003 | HOXB2 | 3212 | P14652 | homeobox B2 | 52 | 0.6 | 0.5 | 0.000 | 0.01 | | 1 | 1 | 0 | 2005 | 2005 |

| | | | | | | | | | | | | | | | | | |
|---|---|---|---|---|---|---|---|---|---|---|---|---|---|---|---|---|---|
| d Arthritis | 873 | | | | | | | 12 | 77 | 146 | | | | | | | |
| Rheumatoid Arthritis | C0003873 | HOXD4 | 3233 | P09016 | homeobox D4 | | 15 | 0.76 | 0.346 | 0.005448 | 0.01 | | 1 | 1 | 0 | 1996 | 1996 |
| Rheumatoid Arthritis | C0003873 | APCS | 325 | P02743 | amyloid P component, serum | | 141 | 0.531 | 0.808 | 0.031714 | 0.01 | | 1 | 1 | 0 | 2011 | 2011 |
| Rheumatoid Arthritis | C0003873 | HPRT1 | 3251 | P00492 | hypoxanthine phosphoribosyltransferase 1 | Enzyme | 210 | 0.5 | 0.808 | 0.93728 | 0.01 | | 1 | 1 | 0 | 1998 | 1998 |
| Rheumatoid Arthritis | C0003873 | HRG | 3273 | P04196 | histidine rich glycoprotein | Enzyme modulator | 72 | 0.603 | 0.577 | 5.89E-14 | 0.01 | | 1 | 1 | 0 | 2018 | 2018 |
| Rheumatoid Arthritis | C0003873 | PRMT1 | 3276 | Q99873 | protein arginine methyltransferase 1 | Epigenetic regulator | 110 | 0.552 | 0.808 | 0.99907 | 0.01 | | 1 | 1 | 0 | 2018 | 2018 |
| Rheumatoid Arthritis | C0003873 | HES1 | 3280 | Q14469 | hes family bHLH transcription factor 1 | Transcription factor | 243 | 0.468 | 0.885 | 0.7072 | 0.01 | | 1 | 1 | 0 | 2017 | 2017 |

| | | | | | | | | | | | | | | | | | |
|---|---|---|---|---|---|---|---|---|---|---|---|---|---|---|---|---|---|
| Rheumatoid Arthritis | C0003873 | BIRC2 | 329 | Q13490 | baculoviral IAP repeat containing 2 | Enzyme modulator | 125 | 0.535 | 0.731 | 0.42708 | 0.01 | | 1 | 1 | 0 | 2019 | 2019 |
| Rheumatoid Arthritis | C0003873 | BIRC3 | 330 | Q13489 | baculoviral IAP repeat containing 3 | Enzyme modulator | 209 | 0.491 | 0.769 | 0.065597 | 0.01 | | 1 | 1 | 0 | 2019 | 2019 |
| Rheumatoid Arthritis | C0003873 | HSPA1A | 3303 | P0DMV8; P0DMV9 | heat shock protein family A (Hsp70) member 1A | | 229 | 0.486 | 0.769 | 0.030228 | 0.01 | | 1 | 1 | 0 | 1996 | 1996 |
| Rheumatoid Arthritis | C0003873 | HSPA1B | 3304 | P0DMV8; P0DMV9 | heat shock protein family A (Hsp70) member 1B | | 251 | 0.474 | 0.808 | 0.005346 | 0.01 | | 1 | 1 | 0 | 1996 | 1996 |
| Rheumatoid Arthritis | C0003873 | HSPA5 | 3309 | P11021 | heat shock protein family A (Hsp70) member 5 | | 350 | 0.434 | 0.885 | 0.77341 | 0.01 | | 1 | 1 | 0 | 2014 | 2014 |
| Rheumatoid Arthritis | C0003873 | XIAP | 331 | P98170 | X-linked inhibitor of apoptosis | Enzyme modulator | 321 | 0.447 | 0.808 | 0.91837 | 0.01 | | 1 | 1 | 0 | 2000 | 2000 |
| Rheumatoid Ar | C0003873 | HSPB1 | 3315 | P04792 | heat shock protein family B (small) member 1 | | 395 | 0.436 | 0.808 | 2.78E- | 0.01 | | 1 | 1 | 0 | 2011 | 2011 |

| | | | | | | | | | | | | | | | | | |
|---|---|---|---|---|---|---|---|---|---|---|---|---|---|---|---|---|---|
| thritis | | | | | | | | | | 07 | | | | | | | |
| Rheumatoid Arthritis | C0003873 | HSPB2 | 3316 | Q16082 | heat shock protein family B (small) member 2 | | 290 | 0.457 | 0.769 | 0.00033 | 0.01 | | 1 | 1 | 0 | 2011 | 2011 |
| Rheumatoid Arthritis | C0003873 | NDST1 | 3340 | P52848 | N-deacetylase and N-sulfotransferase 1 | | 93 | 0.608 | 0.692 | 0.99995 | 0.01 | | 1 | 1 | 0 | 2019 | 2019 |
| Rheumatoid Arthritis | C0003873 | APOA1 | 335 | P02647 | apolipoprotein A1 | | 416 | 0.429 | 0.885 | 0.000549 | 0.01 | | 0 | 1 | 0 | 2015 | 2015 |
| Rheumatoid Arthritis | C0003873 | APOA2 | 336 | P02652 | apolipoprotein A2 | Transporter | 77 | 0.582 | 0.769 | 0.0448 | 0.01 | | 1 | 1 | 0 | 2003 | 2003 |
| Rheumatoid Arthritis | C0003873 | IAPP | 3375 | P10997 | islet amyloid polypeptide | Signaling | 183 | 0.507 | 0.808 | 0.003181 | 0.01 | | 1 | 1 | 0 | 2018 | 2018 |
| Rheumatoid Arthritis | C0003873 | RSPO2 | 340419 | Q6UXX9 | R-spondin 2 | | 113 | 0.558 | 0.577 | 0.024608 | 0.01 | | 1 | 1 | 0 | 2012 | 2012 |
| Rheumatoi | C0003 | IDDM7 | 3406 | | insulin dependent diabetes mellitus 7 | | 5 | 0.8 | 0.1 | | 0.01 | | 0 | 1 | 0 | 2000 | 2000 |

| | | | | | | | | | | | | | | | | | |
|---|---|---|---|---|---|---|---|---|---|---|---|---|---|---|---|---|---|
| d Arthritis | 873 | | | | | | | 61 | 92 | | | | | | | | |
| Rheumatoid Arthritis | C0003873 | IDDM13 | 3412 | | insulin dependent diabetes mellitus 13 | | 3 | 0.89 | 0.192 | | 0.01 | | 1 | 1 | 0 | 2002 | 2002 |
| Rheumatoid Arthritis | C0003873 | IFI16 | 3428 | Q16666 | interferon gamma inducible protein 16 | Transcription factor | 79 | 0.603 | 0.769 | 1.78E-11 | 0.01 | | 1 | 1 | 0 | 2017 | 2017 |
| Rheumatoid Arthritis | C0003873 | IFI27 | 3429 | P40305 | interferon alpha inducible protein 27 | | 332 | 0.437 | 0.769 | 0.0106698 | 0.01 | | 1 | 1 | 0 | 2005 | 2005 |
| Rheumatoid Arthritis | C0003873 | IFI35 | 3430 | P80217 | interferon induced protein 35 | Transcription factor | 17 | 0.76 | 0.462 | 4.28E-07 | 0.01 | | 1 | 1 | 0 | 2013 | 2013 |
| Rheumatoid Arthritis | C0003873 | CYCSP51 | 343045 | | CYCS pseudogene 51 | | 15 | 0.751 | 0.423 | | 0.01 | | 1 | 1 | 0 | 2018 | 2018 |
| Rheumatoid Arthritis | C0003873 | IFIT2 | 3433 | P09913 | interferon induced protein with tetratricopeptide repeats 2 | | 42 | 0.656 | 0.5 | 0.25099 | 0.01 | | 1 | 1 | 0 | 2017 | 2017 |

| | | | | | | | | | | | | | | | | | |
|---|---|---|---|---|---|---|---|---|---|---|---|---|---|---|---|---|---|
| Rheumatoid Arthritis | C0003873 | IFIT1 | 3434 | P09914 | interferon induced protein with tetratricopeptide repeats 1 | | 39 | 0.666 | 0.5 | 0.00278 | 0.01 | | 1 | 1 | 0 | 2017 | 2017 |
| Rheumatoid Arthritis | C0003873 | IFNGR2 | 3460 | P38484 | interferon gamma receptor 2 | Receptor | 71 | 0.597 | 0.577 | 0.95281 | 0.01 | | 1 | 1 | 0 | 2015 | 2015 |
| Rheumatoid Arthritis | C0003873 | ZNF391 | 346157 | Q9UJN7 | zinc finger protein 391 | | 1 | 1 | 0.115 | 6.45E-08 | 0.01 | | 1 | 1 | 0 | 2011 | 2011 |
| Rheumatoid Arthritis | C0003873 | IGFBP4 | 3487 | P22692 | insulin like growth factor binding protein 4 | Enzyme modulator | 94 | 0.572 | 0.731 | 0.0230057 | 0.01 | | 1 | 1 | 0 | 2005 | 2005 |
| Rheumatoid Arthritis | C0003873 | IGFBP6 | 3489 | P24592 | insulin like growth factor binding protein 6 | Enzyme modulator | 71 | 0.601 | 0.692 | 0.02915 | 0.01 | | 1 | 1 | 0 | 2017 | 2017 |
| Rheumatoid Arthritis | C0003873 | IGFBP7 | 3490 | Q16270 | insulin like growth factor binding protein 7 | | 241 | 0.476 | 0.769 | 0.001287 | 0.01 | | 1 | 1 | 0 | 2011 | 2011 |
| Rheumatoid Ar | C0003873 | IGH | 3492 | | immunoglobulin heavy locus | | 238 | 0.473 | 0.654 | | 0.01 | | 1 | 1 | 0 | 2017 | 2017 |

| | | | | | | | | | | | | | | | | | |
|---|---|---|---|---|---|---|---|---|---|---|---|---|---|---|---|---|---|
| thritis | | | | | | | | | | | | | | | | | |
| Rheumatoid Arthritis | C0003873 | NMNAT3 | 349565 | Q96T66 | nicotinamide nucleotide adenylyltransferase 3 | | 7 | 0.821 | 0.346 | 6.24E-08 | 0.01 | | 1 | 1 | 0 | 2019 | 2019 |
| Rheumatoid Arthritis | C0003873 | IGKC | 3514 | P01834 | immunoglobulin kappa constant | | 26 | 0.7 | 0.423 | | 0.01 | | 1 | 1 | 0 | 1989 | 1989 |
| Rheumatoid Arthritis | C0003873 | HLA-P | 352963 | | major histocompatibility complex, class I, P (pseudogene) | | 2 | 0.931 | 0.192 | | 0.01 | | 1 | 1 | 0 | 2009 | 2009 |
| Rheumatoid Arthritis | C0003873 | PADI6 | 353238 | Q6TGC4 | peptidyl arginine deiminase 6 | | 14 | 0.76 | 0.269 | | 0.01 | | 1 | 1 | 0 | 2007 | 2007 |
| Rheumatoid Arthritis | C0003873 | TICAM2 | 353376 | Q86XR7;Q9Y3B3 | toll like receptor adaptor molecule 2 | | 332 | 0.438 | 0.769 | 0.002594 | 0.01 | | 1 | 1 | 0 | 2005 | 2005 |
| Rheumatoid Arthritis | C0003873 | IL2RG | 3561 | P31785 | interleukin 2 receptor subunit gamma | Signaling | 164 | 0.535 | 0.769 | 0.99152 | 0.01 | | 1 | 1 | 0 | 2014 | 2014 |
| Rheumatoi | C0003 | IL11RA | 3590 | Q14626 | interleukin 11 receptor | Signaling | 46 | 0.6 | 0.6 | 6.29E | 0.01 | | 1 | 1 | 0 | 2018 | 2018 |

| | | | | | | | | | | | | | | | | | |
|---|---|---|---|---|---|---|---|---|---|---|---|---|---|---|---|---|---|
| d Arthritis | 873 | | | | subunit alpha | | | 63 | 54 | -21 | | | | | | | |
| Rheumatoid Arthritis | C0003873 | IL12RB1 | 3594 | P42701 | interleukin 12 receptor subunit beta 1 | Signaling | 118 | 0.555 | 0.731 | 9.36E-15 | 0.01 | | 0 | 1 | 0 | 2005 | 2005 |
| Rheumatoid Arthritis | C0003873 | IL15RA | 3601 | Q13261 | interleukin 15 receptor subunit alpha | | 72 | 0.603 | 0.769 | 2.33E-06 | 0.01 | | 1 | 1 | 0 | 2019 | 2019 |
| Rheumatoid Arthritis | C0003873 | CYCSP52 | 360155 | | CYCS pseudogene 52 | | 1 | 1 | 0.115 | | 0.01 | | 1 | 1 | 0 | 2018 | 2018 |
| Rheumatoid Arthritis | C0003873 | INS | 3630 | P01308 | insulin | | 405 | 0.445 | 0.923 | 0.30091 | 0.01 | | 1 | 1 | 0 | 2006 | 2006 |
| Rheumatoid Arthritis | C0003873 | INPP5D | 3635 | Q92835 | inositol polyphosphate-5-phosphatase D | | 109 | 0.558 | 0.731 | 0.97198 | 0.01 | | 1 | 1 | 0 | 2011 | 2011 |
| Rheumatoid Arthritis | C0003873 | IRAK2 | 3656 | O43187 | interleukin 1 receptor associated kinase 2 | Kinase | 22 | 0.722 | 0.385 | 2.61E-19 | 0.01 | | 1 | 1 | 2 | 2018 | 2018 |
| Rheu | C00 | IRF3 | 36 | Q14653 | interferon | Trans | 174 | 0. | 0. | 0.0 | 0.01 | | 1 | 1 | 0 | 2007 | 20 |

| | | | | | | | | | | | | | | | | | |
|---|---|---|---|---|---|---|---|---|---|---|---|---|---|---|---|---|---|
| matoid Arthritis | 03873 | | 61 | | regulatory factor 3 | cription factor | | 517 | 808 | 00116 | | | | | | | 07 |
| Rheumatoid Arthritis | C0003873 | ISL1 | 3670 | P61371 | ISL LIM homeobox 1 | Transcription factor | 94 | 0.579 | 0.654 | 0.868857 | 0.01 | | 1 | 1 | 0 | 2015 | 2015 |
| Rheumatoid Arthritis | C0003873 | ITGA2B | 3674 | P08514 | integrin subunit alpha 2b | | 215 | 0.493 | 0.692 | 1.52E-16 | 0.01 | | 1 | 1 | 0 | 1990 | 1990 |
| Rheumatoid Arthritis | C0003873 | ITGA5 | 3678 | P08648 | integrin subunit alpha 5 | | 145 | 0.524 | 0.769 | 0.424455 | 0.01 | | 1 | 1 | 0 | 2017 | 2017 |
| Rheumatoid Arthritis | C0003873 | ITGA9 | 3680 | Q13797 | integrin subunit alpha 9 | | 62 | 0.621 | 0.692 | 0.008158 | 0.01 | | 1 | 1 | 0 | 2017 | 2017 |
| Rheumatoid Arthritis | C0003873 | ITIH1 | 3697 | P19827 | inter-alpha-trypsin inhibitor heavy chain 1 | Enzyme modulator | 38 | 0.691 | 0.462 | 2.08E-31 | 0.01 | | 1 | 1 | 0 | 2017 | 2017 |
| Rheumatoid Arthritis | C0003873 | ITIH4 | 3700 | Q14624 | inter-alpha-trypsin inhibitor heavy chain 4 | Enzyme modulator | 129 | 0.559 | 0.769 | 6.44E-13 | 0.01 | | 1 | 1 | 0 | 2018 | 2018 |

| | | | | | | | | | | | | | | | | | |
|---|---|---|---|---|---|---|---|---|---|---|---|---|---|---|---|---|---|
| Rheumatoid Arthritis | C0003873 | ITPR2 | 3709 | Q14571 | inositol 1,4,5-trisphosphate receptor type 2 | Ion channel | 55 | 0.631 | 0.654 | 5.71E-29 | 0.01 | | 1 | 1 | 0 | 2007 | 2007 |
| Rheumatoid Arthritis | C0003873 | CD82 | 3732 | P27701 | CD82 molecule | | 172 | 0.507 | 0.731 | 1.27E-08 | 0.01 | | 1 | 1 | 0 | 2007 | 2007 |
| Rheumatoid Arthritis | C0003873 | KCNA4 | 3739 | P22459 | potassium voltage-gated channel subfamily A member 4 | Ion channel | 28 | 0.716 | 0.423 | 0.983327 | 0.01 | | 1 | 1 | 0 | 2019 | 2019 |
| Rheumatoid Arthritis | C0003873 | MIA3 | 375056 | Q5JRA6 | MIA SH3 domain ER export factor 3 | | 27 | 0.716 | 0.423 | 0.036675 | 0.01 | | 1 | 1 | 1 | 2012 | 2012 |
| Rheumatoid Arthritis | C0003873 | ENHO | 375704 | Q6UWT2 | energy homeostasis associated | | 71 | 0.599 | 0.769 | 0.105260 | 0.01 | | 1 | 1 | 0 | 2018 | 2018 |
| Rheumatoid Arthritis | C0003873 | KCNQ1 | 3784 | P51787 | potassium voltage-gated channel subfamily Q member 1 | Ion channel | 281 | 0.485 | 0.769 | 4.55E-08 | 0.01 | | 1 | 1 | 1 | 2018 | 2018 |
| Rheumatoi | C0003 | PICSAR | 3788 | | P38 inhibited cutaneous squamous cell carcinoma | | 11 | 0.8 | 0.3 | | 0.01 | | 1 | 1 | 0 | 2019 | 2019 |

| | | | | | | | | | | | | | | | | | |
|---|---|---|---|---|---|---|---|---|---|---|---|---|---|---|---|---|---|
| d Arthritis | 873 | | 25 | | associated lincRNA | | | 21 | 08 | | | | | | | | |
| Rheumatoid Arthritis | C0003873 | KIR2DS3 | 3808 | Q14952 | killer cell immunoglobulin like receptor, two Ig domains and short cytoplasmic tail 3 | Receptor | 20 | 0.716 | 0.423 | | 0.01 | | 1 | 1 | 0 | 2014 | 2014 |
| Rheumatoid Arthritis | C0003873 | KIR2DS4 | 3809 | P43632 | killer cell immunoglobulin like receptor, two Ig domains and short cytoplasmic tail 4 | Receptor | 30 | 0.674 | 0.5 | | 0.01 | | 1 | 1 | 0 | 2006 | 2006 |
| Rheumatoid Arthritis | C0003873 | KIR3DS1 | 3813 | Q14943 | killer cell immunoglobulin like receptor, three Ig domains and short cytoplasmic tail 1 | Receptor | 57 | 0.617 | 0.692 | | 0.01 | | 1 | 1 | 0 | 2014 | 2014 |
| Rheumatoid Arthritis | C0003873 | KIT | 3815 | P10721 | KIT proto-oncogene, receptor tyrosine kinase | Kinase | 715 | 0.366 | 0.808 | 0.98087 | 0.01 | | 1 | 1 | 0 | 2020 | 2020 |
| Rheuma | C0003 | KPNA2 | 38 | P52292 | karyopherin | Trans | 79 | 0.5 | 0.6 | 0.54 | 0.01 | | 1 | 1 | 0 | 2015 | 20 |

| | | | | | | | | | | | | | | | | | |
|---|---|---|---|---|---|---|---|---|---|---|---|---|---|---|---|---|---|
| toid Arthritis | 873 | | 38 | | subunit alpha 2 | porter | | 88 | 54 | 133 | | | | | | | 15 |
| Rheumatoid Arthritis | C0003873 | ARG2 | 384 | P78540 | arginase 2 | Enzyme | 103 | 0.569 | 0.731 | 0.000182 | 0.01 | | 1 | 1 | 0 | 2002 | 2002 |
| Rheumatoid Arthritis | C0003873 | KRTAP5-9 | 3846 | P26371 | keratin associated protein 5-9 | | 2 | 0.931 | 0.115 | 0.014981 | 0.01 | | 1 | 1 | 0 | 2011 | 2011 |
| Rheumatoid Arthritis | C0003873 | KRT5 | 3852 | P13647 | keratin 5 | | 203 | 0.512 | 0.731 | 0.64172 | 0.01 | | 1 | 1 | 0 | 2017 | 2017 |
| Rheumatoid Arthritis | C0003873 | KRT7 | 3855 | P08729 | keratin 7 | | 287 | 0.463 | 0.808 | 3.09E-12 | 0.01 | | 1 | 1 | 0 | 2004 | 2004 |
| Rheumatoid Arthritis | C0003873 | KRT8 | 3856 | P05787 | keratin 8 | | 166 | 0.517 | 0.654 | 2.36E-08 | 0.01 | | 1 | 1 | 0 | 2015 | 2015 |
| Rheumatoid Arthritis | C0003873 | KRT13 | 3860 | P13646 | keratin 13 | | 53 | 0.631 | 0.692 | 0.000641 | 0.01 | | 1 | 1 | 0 | 2017 | 2017 |

| | | | | | | | | | | | | | | | | | |
|---|---|---|---|---|---|---|---|---|---|---|---|---|---|---|---|---|---|
| Rheumatoid Arthritis | C0003873 | KRT16 | 3868 | P08779 | keratin 16 | | 97 | 0.565 | 0.769 | 1.5E-14 | 0.01 | | 1 | 1 | 0 | 2001 | 2001 |
| Rheumatoid Arthritis | C0003873 | THEMIS | 387357 | Q8N1K5 | thymocyte selection associated | | 32 | 0.682 | 0.654 | 1.96E-06 | 0.01 | | 1 | 1 | 0 | 2017 | 2017 |
| Rheumatoid Arthritis | C0003873 | RTL1 | 388015 | A6NKG5 | retrotransposon Gag like 1 | | 119 | 0.554 | 0.808 | 0.005614 | 0.01 | | 1 | 1 | 0 | 2018 | 2018 |
| Rheumatoid Arthritis | C0003873 | LAG3 | 3902 | P18627 | lymphocyte activating 3 | Receptor | 154 | 0.517 | 0.692 | 0.27623 | 0.01 | | 1 | 1 | 0 | 2017 | 2017 |
| Rheumatoid Arthritis | C0003873 | LAIR2 | 3904 | Q6ISS4 | leukocyte associated immunoglobulin like receptor 2 | Receptor | 12 | 0.78 | 0.269 | 2.6E-07 | 0.01 | | 1 | 1 | 0 | 2008 | 2008 |
| Rheumatoid Arthritis | C0003873 | LAMA3 | 3909 | Q16787 | laminin subunit alpha 3 | Enzyme modulator | 110 | 0.576 | 0.731 | 8.83E-46 | 0.01 | | 1 | 1 | 0 | 2017 | 2017 |
| Rheumatoid | C0003873 | RPS12P4 | 3913 | | ribosomal protein S12 pseudogene 4 | | 1 | 1 | 0.115 | | 0.01 | | 1 | 1 | 0 | 2011 | 2011 |

| | | | | | | | | | | | | | | | | | |
|---|---|---|---|---|---|---|---|---|---|---|---|---|---|---|---|---|---|
| Arthritis | | | 70 | | | | | | | | | | | | | | |
| Rheumatoid Arthritis | C0003873 | LBR | 3930 | Q14739 | lamin B receptor | Enzyme | 307 | 0.473 | 0.808 | 0.18143 | 0.01 | | 1 | 1 | 0 | 2019 | 2019 |
| Rheumatoid Arthritis | C0003873 | LCAT | 3931 | P04180 | lecithin-cholesterol acyltransferase | Enzyme | 94 | 0.588 | 0.654 | 0.08102 | 0.01 | | 1 | 1 | 0 | 2003 | 2003 |
| Rheumatoid Arthritis | C0003873 | LCK | 3932 | P06239 | LCK proto-oncogene, Src family tyrosine kinase | Kinase | 103 | 0.564 | 0.769 | 0.99926 | 0.01 | | 1 | 1 | 0 | 2017 | 2017 |
| Rheumatoid Arthritis | C0003873 | LECT2 | 3950 | O14960 | leukocyte cell derived chemotaxin 2 | Signaling | 47 | 0.653 | 0.5 | 0.001481 | 0.01 | | 1 | 1 | 1 | 2005 | 2005 |
| Rheumatoid Arthritis | C0003873 | LIFR | 3977 | P42702 | LIF receptor subunit alpha | Signaling | 198 | 0.523 | 0.808 | 1.49E-13 | 0.01 | | 1 | 1 | 0 | 2018 | 2018 |
| Rheumatoid Arthritis | C0003873 | LNPEP | 4012 | Q9UIQ6 | leucyl and cystinyl aminopeptidase | Enzyme | 174 | 0.514 | 0.731 | 0.99957 | 0.01 | | 1 | 1 | 0 | 1992 | 1992 |
| Rheuma | C0003 | SAPCD1 | 401 | Q5SSQ6 | suppressor APC domain containing 1 | | 2 | 0.9 | 0.1 | 1.81 | 0.01 | | 1 | 1 | 0 | 2011 | 20 |

| | | | | | | | | | | | | | | | | |
|---|---|---|---|---|---|---|---|---|---|---|---|---|---|---|---|---|
| toid Arthritis | 873 | | 251 | | | | 31 | 54 | E-07 | | | | | | | 11 |
| Rheumatoid Arthritis | C0003873 | CRIP3 | 401262 | Q6Q6R5 | cysteine rich protein 3 | 17 | 0.805 | 0.308 | 0.000648 | 0.01 | | 1 | 1 | 0 | 2017 | 2017 |
| Rheumatoid Arthritis | C0003873 | LOXL2 | 4017 | Q9Y4K0 | lysyl oxidase like 2 | 149 | 0.527 | 0.731 | 3.38E-07 | 0.01 | | 1 | 1 | 0 | 2018 | 2018 |
| Rheumatoid Arthritis | C0003873 | LERFS | 403323 | | lncRNA negative regulator of fibroblast-like synoviocyte migration, SYNCRIP interacting | 1 | 1 | 0.115 | | 0.01 | | 1 | 1 | 0 | 2018 | 2018 |
| Rheumatoid Arthritis | C0003873 | LRP1 | 4035 | Q07954 | LDL receptor related protein 1 | 252 | 0.482 | 0.769 | 1 | 0.01 | | 1 | 1 | 0 | 2008 | 2008 |
| Rheumatoid Arthritis | C0003873 | LRP2 | 4036 | P98164 | LDL receptor related protein 2 | 254 | 0.491 | 0.846 | 0.99999 | 0.01 | | 1 | 1 | 0 | 2003 | 2003 |
| Rheumatoid Arthritis | C0003873 | LRP6 | 4040 | O75581 | LDL receptor related protein 6 | 134 | 0.536 | 0.808 | 0.69664 | 0.01 | | 1 | 1 | 0 | 2014 | 2014 |

| | | | | | | | | | | | | | | | | |
|---|---|---|---|---|---|---|---|---|---|---|---|---|---|---|---|---|
| Rheumatoid Arthritis | C0003873 | LSP1 | 4046 | P33241 | lymphocyte specific protein 1 | 41 | 0.7 | 0.462 | 1.47E-06 | 0.01 | | 1 | 1 | 0 | 2015 | 2015 |
| Rheumatoid Arthritis | C0003873 | LTBR | 4055 | P36941 | lymphotoxin beta receptor | 78 | 0.592 | 0.692 | 0.3058 | 0.01 | | 1 | 1 | 0 | 2008 | 2008 |
| Rheumatoid Arthritis | C0003873 | MIRLET7B | 406884 | | microRNA let-7b | 186 | 0.493 | 0.769 | | 0.01 | | 1 | 1 | 0 | 2016 | 2016 |
| Rheumatoid Arthritis | C0003873 | MIR106A | 406899 | | microRNA 106a | 140 | 0.524 | 0.846 | | 0.01 | | 1 | 1 | 0 | 2018 | 2018 |
| Rheumatoid Arthritis | C0003873 | LYZ | 4069 | P61626 | lysozyme | 178 | 0.526 | 0.846 | 3.41E-06 | 0.01 | | 1 | 1 | 0 | 1989 | 1989 |
| Rheumatoid Arthritis | C0003873 | MIR125A | 406910 | | microRNA 125a | 225 | 0.48 | 0.769 | | 0.01 | | 1 | 1 | 0 | 2019 | 2019 |
| Rheumatoid Ar | C0003873 | MIR137 | 406928 | | microRNA 137 | 171 | 0.513 | 0.846 | | 0.01 | | 1 | 1 | 0 | 2018 | 2018 |

| | | | | | | | | | | | | | | | | |
|---|---|---|---|---|---|---|---|---|---|---|---|---|---|---|---|---|
| thritis | | | | | | | | | | | | | | | | |
| Rheumatoid Arthritis | C0003873 | MIR141 | 406933 | | microRNA 141 | 179 | 0.497 | 0.808 | | 0.01 | | 1 | 1 | 0 | 2019 | 2019 |
| Rheumatoid Arthritis | C0003873 | MIR142 | 406934 | | microRNA 142 | 220 | 0.48 | 0.846 | | 0.01 | | 1 | 1 | 0 | 2019 | 2019 |
| Rheumatoid Arthritis | C0003873 | MIR147A | 406939 | | microRNA 147a | 21 | 0.716 | 0.423 | | 0.01 | | 1 | 1 | 0 | 2019 | 2019 |
| Rheumatoid Arthritis | C0003873 | MIR150 | 406942 | | microRNA 150 | 282 | 0.455 | 0.808 | | 0.01 | | 1 | 1 | 0 | 2018 | 2018 |
| Rheumatoid Arthritis | C0003873 | MIR16-1 | 406950 | | microRNA 16-1 | 57 | 0.623 | 0.5 | | 0.01 | | 1 | 1 | 0 | 2008 | 2008 |
| Rheumatoid Arthritis | C0003873 | MIR18A | 406953 | | microRNA 18a | 154 | 0.52 | 0.846 | | 0.01 | | 1 | 1 | 0 | 2013 | 2013 |
| Rheumatoi | C0003 | MIR182 | 4069 | | microRNA 182 | 211 | 0.4 | 0.8 | | 0.01 | | 1 | 1 | 0 | 2018 | 2018 |

| | | | | | | | | | | | | | | | | |
|---|---|---|---|---|---|---|---|---|---|---|---|---|---|---|---|---|
| d Arthritis | 873 | | 58 | | | | 86 | 46 | | | | | | | | |
| Rheumatoid Arthritis | C0003873 | MIR183 | 406959 | | microRNA 183 | 190 | 0.494 | 0.885 | | 0.01 | | 1 | 1 | 0 | 2017 | 2017 |
| Rheumatoid Arthritis | C0003873 | MIR188 | 406964 | | microRNA 188 | 61 | 0.612 | 0.808 | | 0.01 | | 1 | 1 | 0 | 2015 | 2015 |
| Rheumatoid Arthritis | C0003873 | MIR193A | 406968 | | microRNA 193a | 116 | 0.541 | 0.731 | | 0.01 | | 1 | 1 | 0 | 2019 | 2019 |
| Rheumatoid Arthritis | C0003873 | MIR196A2 | 406973 | | microRNA 196a-2 | 70 | 0.599 | 0.577 | | 0.01 | | 1 | 1 | 1 | 2016 | 2016 |
| Rheumatoid Arthritis | C0003873 | MIR199A1 | 406976 | | microRNA 199a-1 | 87 | 0.578 | 0.577 | | 0.01 | | 1 | 1 | 0 | 2018 | 2018 |
| Rheumatoid Arthritis | C0003873 | MIR199A2 | 406977 | | microRNA 199a-2 | 87 | 0.578 | 0.577 | | 0.01 | | 1 | 1 | 0 | 2018 | 2018 |

| | | | | | | | | | | | | | | | | |
|---|---|---|---|---|---|---|---|---|---|---|---|---|---|---|---|---|
| Rheumatoid Arthritis | C0003873 | MIR19A | 406979 | | microRNA 19a | 151 | 0.512 | 0.808 | | 0.01 | | 1 | 1 | 0 | 2019 | 2019 |
| Rheumatoid Arthritis | C0003873 | MIR19B1 | 406980 | | microRNA 19b-1 | 144 | 0.529 | 0.769 | | 0.01 | | 1 | 1 | 0 | 2012 | 2012 |
| Rheumatoid Arthritis | C0003873 | MIR200C | 406985 | | microRNA 200c | 202 | 0.486 | 0.846 | | 0.01 | | 1 | 1 | 0 | 2017 | 2017 |
| Rheumatoid Arthritis | C0003873 | MIR203A | 406986 | | microRNA 203a | 237 | 0.471 | 0.808 | | 0.01 | | 1 | 1 | 0 | 2011 | 2011 |
| Rheumatoid Arthritis | C0003873 | MIR204 | 406987 | | microRNA 204 | 203 | 0.487 | 0.808 | | 0.01 | | 1 | 1 | 0 | 2018 | 2018 |
| Rheumatoid Arthritis | C0003873 | MIR212 | 406994 | | microRNA 212 | 126 | 0.535 | 0.808 | | 0.01 | | 1 | 1 | 0 | 2018 | 2018 |
| Rheumatoid Ar | C0003873 | MIR219A2 | 407003 | | microRNA 219a-2 | 8 | 0.839 | 0.269 | | 0.01 | | 1 | 1 | 0 | 2015 | 2015 |

| | | | | | | | | | | | | | | | | |
|---|---|---|---|---|---|---|---|---|---|---|---|---|---|---|---|---|
| thritis | | | | | | | | | | | | | | | | |
| Rheumatoid Arthritis | C0003873 | MIR23A | 407010 | | microRNA 23a | 174 | 0.499 | 0.808 | | 0.01 | | 1 | 1 | 0 | 2017 | 2017 |
| Rheumatoid Arthritis | C0003873 | MIR26B | 407017 | | microRNA 26b | 128 | 0.529 | 0.808 | | 0.01 | | 1 | 1 | 0 | 2015 | 2015 |
| Rheumatoid Arthritis | C0003873 | MIR27B | 407019 | | microRNA 27b | 141 | 0.529 | 0.769 | | 0.01 | | 1 | 1 | 0 | 2019 | 2019 |
| Rheumatoid Arthritis | C0003873 | MIR29B1 | 407024 | | microRNA 29b-1 | 212 | 0.485 | 0.846 | | 0.01 | | 1 | 1 | 0 | 2019 | 2019 |
| Rheumatoid Arthritis | C0003873 | MIR29B2 | 407025 | | microRNA 29b-2 | 214 | 0.484 | 0.885 | | 0.01 | | 1 | 1 | 0 | 2019 | 2019 |
| Rheumatoid Arthritis | C0003873 | MIR301A | 407027 | | microRNA 301a | 102 | 0.558 | 0.808 | | 0.01 | | 1 | 1 | 0 | 2016 | 2016 |
| Rheumatoi | C0003 | MIR320A | 4070 | | microRNA 320a | 145 | 0.5 | 0.8 | | 0.01 | | 1 | 1 | 0 | 2019 | 2019 |

| | | | | | | | | | | | | | | | | |
|---|---|---|---|---|---|---|---|---|---|---|---|---|---|---|---|---|
| d Arthritis | 873 | | 37 | | | | 26 | 08 | | | | | | | | |
| Rheumatoid Arthritis | C0003873 | MIR33A | 407039 | | microRNA 33a | 100 | 0.56 | 0.654 | | 0.01 | | 1 | 1 | 0 | 2018 | 2018 |
| Rheumatoid Arthritis | C0003873 | MIR34B | 407041 | | microRNA 34b | 165 | 0.509 | 0.808 | | 0.01 | | 1 | 1 | 0 | 2014 | 2014 |
| Rheumatoid Arthritis | C0003873 | MIR34C | 407042 | | microRNA 34c | 131 | 0.541 | 0.885 | | 0.01 | | 1 | 1 | 0 | 2018 | 2018 |
| Rheumatoid Arthritis | C0003873 | MIR7-1 | 407043 | | microRNA 7-1 | 52 | 0.628 | 0.692 | | 0.01 | | 1 | 1 | 0 | 2017 | 2017 |
| Rheumatoid Arthritis | C0003873 | MIR7-2 | 407044 | | microRNA 7-2 | 54 | 0.623 | 0.692 | | 0.01 | | 1 | 1 | 0 | 2017 | 2017 |
| Rheumatoid Arthritis | C0003873 | MIR7-3 | 407045 | | microRNA 7-3 | 53 | 0.626 | 0.692 | | 0.01 | | 1 | 1 | 0 | 2017 | 2017 |

| | | | | | | | | | | | | | | | | |
|---|---|---|---|---|---|---|---|---|---|---|---|---|---|---|---|---|
| Rheumatoid Arthritis | C0003873 | MIR99B | 407056 | | microRNA 99b | | 52 | 0.633 | 0.615 | | 0.01 | | 1 | 1 | 0 | 2020 | 2020 |
| Rheumatoid Arthritis | C0003873 | CAPRIN1 | 4076 | Q14444 | cell cycle associated protein 1 | Transporter | 29 | 0.691 | 0.5 | 0.97247 | 0.01 | | 1 | 1 | 0 | 2020 | 2020 |
| Rheumatoid Arthritis | C0003873 | TNFSF12-TNFSF13 | 407977 | O43508 | TNFSF12-TNFSF13 readthrough | | 91 | 0.579 | 0.615 | 0.76997 | 0.01 | | 1 | 1 | 0 | 2007 | 2007 |
| Rheumatoid Arthritis | C0003873 | ARRB1 | 408 | P49407 | arrestin beta 1 | Enzyme modulator | 92 | 0.588 | 0.846 | 0.99394 | 0.01 | | 1 | 1 | 0 | 2011 | 2011 |
| Rheumatoid Arthritis | C0003873 | MAA | 4080 | | microphthalmia or anophthalmia and associated anomalies | | 37 | 0.666 | 0.577 | | 0.01 | | 1 | 1 | 0 | 2019 | 2019 |
| Rheumatoid Arthritis | C0003873 | SMAD1 | 4086 | Q15797 | SMAD family member 1 | Transcription factor | 138 | 0.533 | 0.731 | 0.98803 | 0.01 | | 1 | 1 | 0 | 2018 | 2018 |
| Rheumatoid Ar | C0003873 | ARRB2 | 409 | P32121 | arrestin beta 2 | Enzyme modul | 119 | 0.561 | 0.769 | 0.50658 | 0.01 | | 1 | 1 | 0 | 2019 | 2019 |

| | | | | | | | | | | | | | | | | | |
|---|---|---|---|---|---|---|---|---|---|---|---|---|---|---|---|---|---|
| thritis | | | | | | ator | | | | | | | | | | | |
| Rheumatoid Arthritis | C0003873 | ASIC1 | 41 | P78348 | acid sensing ion channel subunit 1 | Ion channel | 52 | 0.653 | 0.615 | 0.930017 | 0.01 | | 1 | 1 | 0 | 2014 | 2014 |
| Rheumatoid Arthritis | C0003873 | MAL | 4118 | P21145 | mal, T cell differentiation protein | Transporter | 102 | 0.57 | 0.577 | 0.831197 | 0.01 | | 1 | 1 | 0 | 2008 | 2008 |
| Rheumatoid Arthritis | C0003873 | MAPT | 4137 | P10636 | microtubule associated protein tau | | 469 | 0.446 | 0.923 | 0.006026 | 0.01 | | 1 | 1 | 0 | 2017 | 2017 |
| Rheumatoid Arthritis | C0003873 | MARK1 | 4139 | Q9P0L2 | microtubule affinity regulating kinase 1 | Kinase | 40 | 0.663 | 0.577 | 0.60045 | 0.01 | | 1 | 1 | 0 | 2019 | 2019 |
| Rheumatoid Arthritis | C0003873 | MBNL1 | 4154 | Q9NR56 | muscleblind like splicing regulator 1 | Nucleic acid binding | 52 | 0.65 | 0.615 | 0.70579 | 0.01 | | 1 | 1 | 0 | 2018 | 2018 |
| Rheumatoid Arthritis | C0003873 | ADAM11 | 4185 | O75078 | ADAM metallopeptidase domain 11 | Enzyme | 42 | 0.67 | 0.538 | 1.04E-05 | 0.01 | | 1 | 1 | 0 | 2010 | 2010 |
| Rheumatoi | C0003 | MDM4 | 4194 | O15151 | MDM4 regulator of p53 | Nucleic aci | 265 | 0.4 | 0.8 | 0.999 | 0.01 | | 1 | 1 | 0 | 2010 | 2010 |

| | | | | | | | | | | | | | | | | | |
|---|---|---|---|---|---|---|---|---|---|---|---|---|---|---|---|---|---|
| d Arthritis | 873 | | | | | d binding | | 61 | 08 | 92 | | | | | | | |
| Rheumatoid Arthritis | C0003873 | MAP3K3 | 4215 | Q99759 | mitogen-activated protein kinase kinase kinase 3 | Kinase | 57 | 0.626 | 0.692 | 0.98535 | 0.01 | | 1 | 1 | 0 | 2004 | 2004 |
| Rheumatoid Arthritis | C0003873 | MGAT5 | 4249 | Q09328 | alpha-1,6-mannosylglycoprotein 6-beta-N-acetylglucosaminyltransferase | Enzyme | 59 | 0.638 | 0.5 | 0.99998 | 0.01 | | 1 | 1 | 0 | 2010 | 2010 |
| Rheumatoid Arthritis | C0003873 | KITLG | 4254 | P21583 | KIT ligand | | 249 | 0.473 | 0.885 | 0.85024 | 0.01 | | 1 | 1 | 0 | 1988 | 1988 |
| Rheumatoid Arthritis | C0003873 | ASAH1 | 427 | Q13510 | N-acylsphingosine amidohydrolase 1 | Enzyme | 176 | 0.526 | 0.808 | 1.26E-12 | 0.01 | | 1 | 1 | 0 | 2016 | 2016 |
| Rheumatoid Arthritis | C0003873 | MITF | 4286 | O75030 | melanocyte inducing transcription factor | | 244 | 0.499 | 0.808 | 0.98119 | 0.01 | | 1 | 1 | 0 | 2007 | 2007 |
| Rheumatoid Arthritis | C0003873 | MAP3K11 | 4296 | Q16584 | mitogen-activated protein kinase kinase kinase 11 | Kinase | 74 | 0.597 | 0.615 | 0.010875 | 0.01 | | 1 | 1 | 0 | 2004 | 2004 |

| | | | | | | | | | | | | | | | | | |
|---|---|---|---|---|---|---|---|---|---|---|---|---|---|---|---|---|---|
| Rheumatoid Arthritis | C0003873 | KMT2A | 4297 | Q03164 | lysine methyltransferase 2A | | 535 | 0.41 | 0.885 | 1 | 0.01 | | 1 | 1 | 0 | 2012 | 2012 |
| Rheumatoid Arthritis | C0003873 | AFF1 | 4299 | P51825 | AF4/FMR2 family member 1 | Transcription factor | 49 | 0.659 | 0.423 | 0.71038 | 0.01 | | 1 | 1 | 1 | 2018 | 2018 |
| Rheumatoid Arthritis | C0003873 | MMP7 | 4316 | P09237 | matrix metallopeptidase 7 | Enzyme | 320 | 0.446 | 0.885 | 1.04E-09 | 0.01 | | 1 | 1 | 0 | 2007 | 2007 |
| Rheumatoid Arthritis | C0003873 | MMP15 | 4324 | P51511 | matrix metallopeptidase 15 | Enzyme | 54 | 0.644 | 0.5 | 0.0733354 | 0.01 | | 1 | 1 | 0 | 1999 | 1999 |
| Rheumatoid Arthritis | C0003873 | MMP16 | 4325 | P51512 | matrix metallopeptidase 16 | Enzyme | 58 | 0.626 | 0.577 | 0.917197 | 0.01 | | 0 | 1 | 0 | 2000 | 2000 |
| Rheumatoid Arthritis | C0003873 | MMP17 | 4326 | Q9ULZ9 | matrix metallopeptidase 17 | Enzyme | 30 | 0.705 | 0.308 | 2.2E-08 | 0.01 | | 1 | 1 | 0 | 2000 | 2000 |
| Rheumatoid Ar | C0003873 | MNAT1 | 4331 | P51948 | MNAT1 component of CDK activating kinase | Enzyme modul | 131 | 0.539 | 0.846 | 1.31E- | 0.01 | | 1 | 1 | 0 | 2015 | 2015 |

| | | | | | | | | | | | | | | | | | |
|---|---|---|---|---|---|---|---|---|---|---|---|---|---|---|---|---|---|
| thritis | | | | | | ator | | | | 12 | | | | | | | |
| Rheumatoid Arthritis | C0003873 | MOBP | 4336 | Q13875 | myelin associated oligodendrocyte basic protein | Cellular structure | 20 | 0.7 | 0.423 | 0.54623 | 0.01 | | 1 | 1 | 0 | 2014 | 2014 |
| Rheumatoid Arthritis | C0003873 | MOS | 4342 | P00540 | MOS proto-oncogene, serine/threonine kinase | Kinase | 78 | 0.617 | 0.5 | 0.58928 | 0.01 | | 1 | 1 | 0 | 2019 | 2019 |
| Rheumatoid Arthritis | C0003873 | MS | 4397 | | multiple sclerosis | | 95 | 0.599 | 0.692 | | 0.01 | | 1 | 1 | 0 | 2010 | 2010 |
| Rheumatoid Arthritis | C0003873 | CCDC88C | 440193 | Q9P219 | coiled-coil domain containing 88C | | 54 | 0.647 | 0.346 | 3.3E-08 | 0.01 | | 1 | 1 | 0 | 2017 | 2017 |
| Rheumatoid Arthritis | C0003873 | CALHM6 | 441168 | Q5R3K3 | calcium homeostasis modulator family member 6 | | 7 | 0.89 | 0.192 | 0.009461 | 0.01 | | 1 | 1 | 0 | 2016 | 2016 |
| Rheumatoid Arthritis | C0003873 | MIR135B | 442891 | | microRNA 135b | | 111 | 0.554 | 0.731 | | 0.01 | | 1 | 1 | 0 | 2019 | 2019 |
| Rheumatoi | C0003 | MIR148B | 4428 | | microRNA 148b | | 78 | 0.5 | 0.6 | | 0.01 | | 1 | 1 | 0 | 2018 | 2018 |

| | | | | | | | | | | | | | | | | | |
|---|---|---|---|---|---|---|---|---|---|---|---|---|---|---|---|---|---|
| d Arthritis | 873 | | 92 | | | | | 92 | 92 | | | | | | | | |
| Rheumatoid Arthritis | C0003873 | MSD | 4434 | | microcephaly with spastic diplegia (Paine syndrome) | | 35 | 0.674 | 0.654 | | 0.01 | | 1 | 1 | 0 | 2018 | 2018 |
| Rheumatoid Arthritis | C0003873 | MSH2 | 4436 | P43246 | mutS homolog 2 | Nucleic acid binding | 490 | 0.406 | 0.808 | 0.89539 | 0.01 | | 1 | 1 | 0 | 2003 | 2003 |
| Rheumatoid Arthritis | C0003873 | MSH3 | 4437 | P20585 | mutS homolog 3 | Nucleic acid binding | 245 | 0.474 | 0.769 | 7.44E-31 | 0.01 | | 1 | 1 | 0 | 2003 | 2003 |
| Rheumatoid Arthritis | C0003873 | TARP | 445347 | | TCR gamma alternate reading frame protein | | 44 | 0.638 | 0.5 | | 0.01 | | 0 | 1 | 0 | 1995 | 1995 |
| Rheumatoid Arthritis | C0003873 | MSMB | 4477 | P08118 | microseminoprotein beta | Signaling | 195 | 0.498 | 0.846 | 0.016199 | 0.01 | | 1 | 1 | 0 | 2017 | 2017 |
| Rheumatoid Arthritis | C0003873 | PLF | 450095 | | Pulmonary function | | 124 | 0.544 | 0.731 | | 0.01 | | 0 | 1 | 0 | 2019 | 2019 |

| | | | | | | | | | | | | | | | | | |
|---|---|---|---|---|---|---|---|---|---|---|---|---|---|---|---|---|---|
| Rheumatoid Arthritis | C0003873 | ND1 | 4535 | P03886 | NADH dehydrogenase, subunit 1 (complex I) | Enzyme | 260 | 0.522 | 0.769 | | 0.01 | | 1 | 1 | 0 | 2005 | 2005 |
| Rheumatoid Arthritis | C0003873 | MTNR1B | 4544 | P49286 | melatonin receptor 1B | G-protein coupled receptor | 93 | 0.588 | 0.731 | 0.000648 | 0.01 | | 0 | 1 | 0 | 2005 | 2005 |
| Rheumatoid Arthritis | C0003873 | MUC2 | 4583 | Q02817 | mucin 2, oligomeric mucus/gel-forming | Enzyme modulator | 225 | 0.486 | 0.808 | 9.43E-10 | 0.01 | | 1 | 1 | 0 | 2008 | 2008 |
| Rheumatoid Arthritis | C0003873 | MUC3A | 4584 | Q02505 | mucin 3A, cell surface associated | | 52 | 0.636 | 0.5 | 1.48E-10 | 0.01 | | 1 | 1 | 0 | 2008 | 2008 |
| Rheumatoid Arthritis | C0003873 | MUC4 | 4585 | Q99102 | mucin 4, cell surface associated | | 183 | 0.506 | 0.769 | 2.79E-54 | 0.01 | | 1 | 1 | 0 | 2005 | 2005 |
| Rheumatoid Arthritis | C0003873 | MUC5AC | 4586 | P98088 | mucin 5AC, oligomeric mucus/gel-forming | Enzyme modulator | 224 | 0.483 | 0.808 | 9.99E-09 | 0.01 | | 1 | 1 | 0 | 2008 | 2008 |
| Rheumatoi | C0003 | MX1 | 4599 | P20591 | MX dynamin like | Enzyme mo | 71 | 0.5 | 0.7 | 3.42E | 0.01 | | 1 | 1 | 0 | 2017 | 2017 |

| | | | | | | | | | | | | | | | | | |
|---|---|---|---|---|---|---|---|---|---|---|---|---|---|---|---|---|---|
| d Arthritis | 873 | | | | GTPase 1 | dulator | | 99 | 31 | -18 | | | | | | | |
| Rheumatoid Arthritis | C0003873 | MYCN | 4613 | P04198 | MYCN proto-oncogene, bHLH transcription factor | Transcription factor | 314 | 0.454 | 0.808 | 0.88897 | 0.01 | | 1 | 1 | 0 | 2005 | 2005 |
| Rheumatoid Arthritis | C0003873 | SERPINC1 | 462 | P01008 | serpin family C member 1 | Enzyme modulator | 184 | 0.507 | 0.769 | 0.99892 | 0.01 | | 1 | 1 | 0 | 1996 | 1996 |
| Rheumatoid Arthritis | C0003873 | ATF3 | 467 | P18847 | activating transcription factor 3 | Transcription factor | 228 | 0.494 | 0.885 | 0.089296 | 0.01 | | 1 | 1 | 0 | 2019 | 2019 |
| Rheumatoid Arthritis | C0003873 | NBN | 4683 | O60934 | nibrin | Nucleic acid binding | 291 | 0.467 | 0.808 | 2.18E-16 | 0.01 | | 1 | 1 | 0 | 2017 | 2017 |
| Rheumatoid Arthritis | C0003873 | NCF4 | 4689 | Q15080 | neutrophil cytosolic factor 4 | | 58 | 0.644 | 0.615 | 9.02E-08 | 0.01 | | 1 | 1 | 0 | 2007 | 2007 |
| Rheumatoid Arthritis | C0003873 | NDUFA7 | 4701 | O95182 | NADH:ubiquinone oxidoreductase subunit A7 | | 2 | 0.931 | 0.115 | 6.6E-05 | 0.01 | | 1 | 1 | 0 | 2015 | 2015 |

| | | | | | | | | | | | | | | | | | |
|---|---|---|---|---|---|---|---|---|---|---|---|---|---|---|---|---|---|
| Rheumatoid Arthritis | C0003873 | NDUFS3 | 4722 | O75489 | NADH:ubiquinone oxidoreductase core subunit S3 | | 120 | 0.581 | 0.769 | 0.000583 | 0.01 | | 1 | 1 | 0 | 2007 | 2007 |
| Rheumatoid Arthritis | C0003873 | NEDD9 | 4739 | Q14511 | neural precursor cell expressed, developmentally down-regulated 9 | | 113 | 0.548 | 0.769 | 0.12634 | 0.01 | | 1 | 1 | 0 | 2003 | 2003 |
| Rheumatoid Arthritis | C0003873 | ATOH1 | 474 | Q92858 | atonal bHLH transcription factor 1 | Enzyme | 53 | 0.633 | 0.5 | 0.017833 | 0.01 | | 1 | 1 | 0 | 1997 | 1997 |
| Rheumatoid Arthritis | C0003873 | NELL1 | 4745 | Q92832 | neural EGFL like 1 | Calcium-binding protein | 122 | 0.539 | 0.731 | 8.67E-12 | 0.01 | | 1 | 1 | 0 | 2012 | 2012 |
| Rheumatoid Arthritis | C0003873 | NEU1 | 4758 | Q99519 | neuraminidase 1 | Enzyme | 207 | 0.513 | 0.769 | 7.34E-05 | 0.01 | | 1 | 1 | 0 | 2019 | 2019 |
| Rheumatoid Arthritis | C0003873 | ATP1A1 | 476 | P05023 | ATPase Na+/K+ transporting subunit alpha 1 | Transporter | 89 | 0.601 | 0.808 | 1 | 0.01 | | 1 | 1 | 0 | 2005 | 2005 |
| Rheumatoi | C0003 | NFATC2 | 4773 | Q13469 | nuclear factor of activate | Transcripti | 154 | 0.5 | 0.7 | 0.999 | 0.01 | | 1 | 1 | 0 | 2015 | 2015 |

| | | | | | | | | | | | | | | | | | |
|---|---|---|---|---|---|---|---|---|---|---|---|---|---|---|---|---|---|
| d Arthritis | 873 | | | | d T cells 2 | on factor | | 25 | 31 | 95 | | | | | | | |
| Rheumatoid Arthritis | C0003873 | NFIA | 4774 | Q12857 | nuclear factor I A | Nucleic acid binding | 95 | 0.599 | 0.808 | 0.99959 | 0.01 | | 1 | 1 | 0 | 2016 | 2016 |
| Rheumatoid Arthritis | C0003873 | NFIL3 | 4783 | Q16649 | nuclear factor, interleukin 3 regulated | Transcription factor | 54 | 0.633 | 0.615 | 0.81979 | 0.01 | | 1 | 1 | 0 | 2013 | 2013 |
| Rheumatoid Arthritis | C0003873 | NFKBIB | 4793 | Q15653 | NFKB inhibitor beta | | 16 | 0.743 | 0.462 | 0.42245 | 0.01 | | 1 | 1 | 0 | 2017 | 2017 |
| Rheumatoid Arthritis | C0003873 | NFYA | 4800 | P23511 | nuclear transcription factor Y subunit alpha | Nucleic acid binding | 43 | 0.65 | 0.654 | 0.14619 | 0.01 | | 1 | 1 | 0 | 2013 | 2013 |
| Rheumatoid Arthritis | C0003873 | NNAT | 4826 | Q16517 | neuronatin | | 50 | 0.633 | 0.538 | 0.40136 | 0.01 | | 1 | 1 | 0 | 2017 | 2017 |
| Rheumatoid Arthritis | C0003873 | NMT1 | 4836 | P30419 | N-myristoyltransferase 1 | Enzyme | 22 | 0.78 | 0.423 | 0.9955 | 0.01 | | 1 | 1 | 0 | 2019 | 2019 |
| Rheu | C00 | NONO | 48 | Q15233 | non-POU | Nucle | 142 | 0. | 0. | 0.9 | 0.01 | | 1 | 1 | 0 | 2017 | 20 |

| | | | | | | | | | | | | | | | | | |
|---|---|---|---|---|---|---|---|---|---|---|---|---|---|---|---|---|---|
| matoid Arthritis | 038733 | | 41 | | domain containing octamer binding | ic acid binding | | 0.554 | 0.731 | 935 | | | | | | | 17 |
| Rheumatoid Arthritis | C0003873 | NPAS2 | 4862 | Q99743 | neuronal PAS domain protein 2 | | 82 | 0.595 | 0.654 | 1 | 0.01 | | 1 | 1 | 0 | 2015 | 2015 |
| Rheumatoid Arthritis | C0003873 | NPPB | 4879 | P16860 | natriuretic peptide B | | 193 | 0.513 | 0.808 | 9.88E-06 | 0.01 | | 1 | 1 | 0 | 2008 | 2008 |
| Rheumatoid Arthritis | C0003873 | NTRK1 | 4914 | P04629 | neurotrophic receptor tyrosine kinase 1 | Kinase | 443 | 0.422 | 0.808 | 1.96E-06 | 0.01 | | 1 | 1 | 0 | 2017 | 2017 |
| Rheumatoid Arthritis | C0003873 | NUCB2 | 4925 | P80303 | nucleobindin 2 | Calcium-binding protein | 140 | 0.54 | 0.731 | 2E-10 | 0.01 | | 1 | 1 | 0 | 2018 | 2018 |
| Rheumatoid Arthritis | C0003873 | OAP | 4937 | | osteoarthrosis, precocious | | 9 | 0.792 | 0.192 | | 0.01 | | 1 | 1 | 0 | 2016 | 2016 |
| Rheumatoid Ar | C0003873 | MIR382 | 494331 | | microRNA 382 | | 71 | 0.603 | 0.692 | | 0.01 | | 1 | 1 | 0 | 2017 | 2017 |

| | | | | | | | | | | | | | | | | | |
|---|---|---|---|---|---|---|---|---|---|---|---|---|---|---|---|---|---|
| thritis | | | | | | | | | | | | | | | | | |
| Rheumatoid Arthritis | C0003873 | OAZ1 | 4946 | P54368 | ornithine decarboxylase antizyme 1 | Enzyme modulator | 22 | 0.722 | 0.423 | 0.17847 | 0.01 | | 1 | 1 | 0 | 2010 | 2010 |
| Rheumatoid Arthritis | C0003873 | ODF1 | 4956 | Q14990 | outer dense fiber of sperm tails 1 | | 14 | 0.769 | 0.385 | 0.9721 | 0.01 | | 1 | 1 | 0 | 2000 | 2000 |
| Rheumatoid Arthritis | C0003873 | OPRK1 | 4986 | P41145 | opioid receptor kappa 1 | G-protein coupled receptor | 156 | 0.555 | 0.615 | 4.06E-05 | 0.01 | | 1 | 1 | 0 | 2000 | 2000 |
| Rheumatoid Arthritis | C0003873 | P2RX1 | 5023 | P51575 | purinergic receptor P2X 1 | Ion channel | 94 | 0.584 | 0.808 | 6.76E-11 | 0.01 | | 1 | 1 | 0 | 2010 | 2010 |
| Rheumatoid Arthritis | C0003873 | P2RX3 | 5024 | P56373 | purinergic receptor P2X 3 | Ion channel | 125 | 0.565 | 0.769 | 1.03E-06 | 0.01 | | 1 | 1 | 0 | 2010 | 2010 |
| Rheumatoid Arthritis | C0003873 | P2RX4 | 5025 | Q99571 | purinergic receptor P2X 4 | Ion channel | 138 | 0.547 | 0.808 | 4.98E-12 | 0.01 | | 1 | 1 | 0 | 2010 | 2010 |

| | | | | | | | | | | | | | | | | |
|---|---|---|---|---|---|---|---|---|---|---|---|---|---|---|---|---|
| Rheumatoid Arthritis | C0003873 | P2RX5 | 5026 | Q93086 | purinergic receptor P2X 5 | Ion channel | 132 | 0.548 | 0.769 | 2.85E-13 | 0.01 | | 1 | 1 | 0 | 2010 | 2010 |
| Rheumatoid Arthritis | C0003873 | P2RY1 | 5028 | P47900 | purinergic receptor P2Y1 | G-protein coupled receptor | 126 | 0.551 | 0.769 | 0.515222 | 0.01 | | 1 | 1 | 0 | 2010 | 2010 |
| Rheumatoid Arthritis | C0003873 | P2RY2 | 5029 | P41231 | purinergic receptor P2Y2 | G-protein coupled receptor | 136 | 0.543 | 0.808 | 0.0842445 | 0.01 | | 1 | 1 | 0 | 2010 | 2010 |
| Rheumatoid Arthritis | C0003873 | P2RY11 | 5032 | Q96G91 | purinergic receptor P2Y11 | G-protein coupled receptor | 27 | 0.705 | 0.538 | 0.0077706 | 0.01 | | 1 | 1 | 0 | 2019 | 2019 |
| Rheumatoid Arthritis | C0003873 | PEBP1 | 5037 | P30086 | phosphatidylethanolamine binding protein 1 | Enzyme modulator | 181 | 0.5 | 0.808 | 0.09167 | 0.01 | | 1 | 1 | 0 | 2012 | 2012 |
| Rheumatoid Arthritis | C0003873 | FURIN | 5045 | P09958 | furin, paired basic amino acid cleaving enzyme | Enzyme | 80 | 0.612 | 0.769 | 0.99994 | 0.01 | | 1 | 1 | 0 | 2019 | 2019 |

| | | | | | | | | | | | | | | | | | |
|---|---|---|---|---|---|---|---|---|---|---|---|---|---|---|---|---|---|
| Rheumatoid Arthritis | C0003873 | SERPINB2 | 5055 | P05120 | serpin family B member 2 | Enzyme modulator | 183 | 0.502 | 0.808 | 6.38E-12 | 0.01 | | 1 | 1 | 0 | 2017 | 2017 |
| Rheumatoid Arthritis | C0003873 | DEF6 | 50619 | Q9H4E7 | DEF6 guanine nucleotide exchange factor | Nucleic acid binding | 22 | 0.751 | 0.346 | 0.997768 | 0.01 | | 1 | 1 | 0 | 2017 | 2017 |
| Rheumatoid Arthritis | C0003873 | PARD6A | 50855 | Q9NPB6 | par-6 family cell polarity regulator alpha | Cell-cell junction | 6 | 0.861 | 0.231 | 0.006472 | 0.01 | | 1 | 1 | 0 | 2008 | 2008 |
| Rheumatoid Arthritis | C0003873 | PCBP1 | 5093 | Q15365 | poly(rC) binding protein 1 | Enzyme | 49 | 0.65 | 0.538 | 0.909945 | 0.01 | | 1 | 1 | 0 | 2017 | 2017 |
| Rheumatoid Arthritis | C0003873 | IBD5 | 50941 | | inflammatory bowel disease 5 | | 21 | 0.705 | 0.538 | | 0.01 | | 1 | 1 | 0 | 2004 | 2004 |
| Rheumatoid Arthritis | C0003873 | EXOSC1 | 51013 | Q9Y3B2 | exosome component 1 | Enzyme | 3 | 0.931 | 0.154 | 7.35E-09 | 0.01 | | 1 | 1 | 0 | 2016 | 2016 |
| Rheumatoid Ar | C0003873 | TMED7 | 51014 | Q86XR7;Q9Y3B3 | transmembrane p24 trafficking protein 7 | Transporter | 336 | 0.436 | 0.808 | 0.479666 | 0.01 | | 1 | 1 | 0 | 2005 | 2005 |

| | | | | | | | | | | | | | | | | |
|---|---|---|---|---|---|---|---|---|---|---|---|---|---|---|---|---|
| thritis | | | | | | | | | | | | | | | | |
| Rheumatoid Arthritis | C0003873 | NDUFA13 | 51079 | Q9P0J0 | NADH: ubiquinone oxidoreductase subunit A13 | Enzyme | 131 | 0.556 | 0.692 | 3.8E-06 | 0.01 | | 1 | 1 | 0 | 2018 | 2018 |
| Rheumatoid Arthritis | C0003873 | RMDN1 | 51115 | Q96DB5 | regulator of microtubule dynamics 1 | | 123 | 0.546 | 0.769 | 5.9E-10 | 0.01 | | 1 | 1 | 0 | 2019 | 2019 |
| Rheumatoid Arthritis | C0003873 | IRAK4 | 51135 | Q9NWZ3 | interleukin 1 receptor associated kinase 4 | Kinase | 90 | 0.578 | 0.769 | 7.8E-09 | 0.01 | | 1 | 1 | 0 | 2019 | 2019 |
| Rheumatoid Arthritis | C0003873 | DBR1 | 51163 | Q9UK59 | debranching RNA lariats 1 | Enzyme | 15 | 0.769 | 0.385 | 1.18E-08 | 0.01 | | 1 | 1 | 0 | 2016 | 2016 |
| Rheumatoid Arthritis | C0003873 | ZDHHC2 | 51201 | Q9UIJ5 | zinc finger DHHC-type palmitoyltransferase 2 | | 24 | 0.729 | 0.462 | 0.001001 | 0.01 | | 1 | 1 | 0 | 2006 | 2006 |
| Rheumatoid Arthritis | C0003873 | PCSK1 | 5122 | P29120 | proprotein convertase subtilisin/kexin type 1 | Enzyme | 140 | 0.548 | 0.615 | 2.75E-09 | 0.01 | | 1 | 1 | 0 | 2001 | 2001 |
| Rheumatoi | C0003 | CLEC1B | 512 | Q9P126 | C-type lectin domain family 1 member B | | 58 | 0.65 | 0.6 | 4.94E | 0.01 | | 1 | 1 | 0 | 2014 | 2014 |

| | | | | | | | | | | | | | | | | | |
|---|---|---|---|---|---|---|---|---|---|---|---|---|---|---|---|---|---|
| d Arthritis | 873 | | 66 | | | | | | 15 | -08 | | | | | | | |
| Rheumatoid Arthritis | C0003873 | RASL12 | 51285 | Q9NYN1 | RAS like family 12 | Enzyme modulator | 14 | 0.751 | 0.385 | 0.0008871 | 0.01 | | 1 | 1 | 0 | 2018 | 2018 |
| Rheumatoid Arthritis | C0003873 | PCYT1A | 51330 | P49585 | phosphate cytidylyltransferase 1, choline, alpha | | 256 | 0.485 | 0.885 | 2.02E-05 | 0.01 | | 1 | 1 | 0 | 2011 | 2011 |
| Rheumatoid Arthritis | C0003873 | ZBTB7A | 51341 | O95365 | zinc finger and BTB domain containing 7A | Transcription factor | 103 | 0.564 | 0.769 | 0.95915 | 0.01 | | 1 | 1 | 0 | 2011 | 2011 |
| Rheumatoid Arthritis | C0003873 | HOOK1 | 51361 | Q9UJC3 | hook microtubule tethering protein 1 | Enzyme modulator | 24 | 0.716 | 0.462 | 3.87E-08 | 0.01 | | 1 | 1 | 0 | 2019 | 2019 |
| Rheumatoid Arthritis | C0003873 | PDE4D | 5144 | Q08499 | phosphodiesterase 4D | | 210 | 0.519 | 0.846 | 0.9998 | 0.01 | | 1 | 1 | 0 | 2016 | 2016 |
| Rheumatoid Arthritis | C0003873 | ISYNA1 | 51477 | Q9NPH2 | inositol-3-phosphate synthase 1 | | 168 | 0.51 | 0.769 | 2.49E-05 | 0.01 | | 1 | 1 | 0 | 2004 | 2004 |

| | | | | | | | | | | | | | | | | | |
|---|---|---|---|---|---|---|---|---|---|---|---|---|---|---|---|---|---|
| Rheumatoid Arthritis | C0003873 | NELFCD | 51497 | Q8IXH7 | negative elongation factor complex member C/D | | 24 | 0.705 | 0.538 | 0.17607 | 0.01 | | 1 | 1 | 0 | 2010 | 2010 |
| Rheumatoid Arthritis | C0003873 | ZC3HC1 | 51530 | Q86WB0 | zinc finger C3HC-type containing 1 | | 23 | 0.76 | 0.308 | 0.000256 | 0.01 | | 1 | 1 | 1 | 2013 | 2013 |
| Rheumatoid Arthritis | C0003873 | SCLY | 51540 | Q96I15 | selenocysteine lyase | Enzyme | 161 | 0.53 | 0.731 | 2.33E-09 | 0.01 | | 1 | 1 | 0 | 2004 | 2004 |
| Rheumatoid Arthritis | C0003873 | ATP6V1H | 51606 | Q9UI12 | ATPase H+ transporting V1 subunit H | Transporter | 13 | 0.78 | 0.346 | 0.000145 | 0.01 | | 1 | 1 | 0 | 2017 | 2017 |
| Rheumatoid Arthritis | C0003873 | PDK1 | 5163 | Q15118 | pyruvate dehydrogenase kinase 1 | Kinase | 164 | 0.51 | 0.808 | 1.1E-05 | 0.01 | | 1 | 1 | 0 | 2019 | 2019 |
| Rheumatoid Arthritis | C0003873 | SF3B6 | 51639 | Q9Y3B4 | splicing factor 3b subunit 6 | | 116 | 0.544 | 0.808 | 0.58902 | 0.01 | | 1 | 1 | 0 | 1990 | 1990 |
| Rheumatoid Ar | C0003873 | ENPP1 | 5167 | P22413 | ectonucleotide pyrophosphatase/phosph | Enzyme | 247 | 0.498 | 0.808 | 4.99E- | 0.01 | | 1 | 1 | 0 | 2001 | 2001 |

| | | | | | | | | | | | | | | | | | |
|---|---|---|---|---|---|---|---|---|---|---|---|---|---|---|---|---|---|
| thritis | | | | | odiesterase 1 | | | | | 08 | | | | | | | |
| Rheumatoid Arthritis | C0003873 | PDPK1 | 5170 | O15530 | 3-phosphoinositide dependent protein kinase 1 | Kinase | 77 | 0.588 | 0.808 | 0.98013 | 0.01 | | 1 | 1 | 0 | 2019 | 2019 |
| Rheumatoid Arthritis | C0003873 | GHRL | 51738 | Q9UBU3 | ghrelin and obestatin prepropeptide | | 183 | 0.517 | 0.769 | 1.39E-11 | 0.01 | | 1 | 1 | 1 | 2008 | 2008 |
| Rheumatoid Arthritis | C0003873 | ACP1 | 52 | P24666 | acid phosphatase 1 | | 107 | 0.566 | 0.731 | 1.75E-08 | 0.01 | | 1 | 1 | 0 | 2011 | 2011 |
| Rheumatoid Arthritis | C0003873 | PGK1 | 5230 | P00558 | phosphoglycerate kinase 1 | Kinase | 144 | 0.542 | 0.769 | 0.76537 | 0.01 | | 1 | 1 | 0 | 2016 | 2016 |
| Rheumatoid Arthritis | C0003873 | PGM1 | 5236 | P36871 | phosphoglucomutase 1 | | 72 | 0.628 | 0.577 | 5.06E-15 | 0.01 | | 1 | 1 | 0 | 1985 | 1985 |
| Rheumatoid Arthritis | C0003873 | PHEX | 5251 | P78562 | phosphate regulating endopeptidase homolog X-linked | Enzyme | 142 | 0.548 | 0.808 | 0.99996 | 0.01 | | 1 | 1 | 0 | 2018 | 2018 |
| Rheumatoi | C0003 | PI3 | 5266 | P19957 | peptidase inhibitor 3 | Enzymemo | 92 | 0.57 | 0.6 | 0.169 | 0.01 | | 1 | 1 | 0 | 2017 | 2017 |

| | | | | | | | | | | | | | | | | | |
|---|---|---|---|---|---|---|---|---|---|---|---|---|---|---|---|---|---|
| d Arthritis | 873 | | | | | dulator | | | 92 | 65 | | | | | | | |
| Rheumatoid Arthritis | C0003873 | PIGR | 5284 | P01833 | polymeric immunoglobulin receptor | | 71 | 0.615 | 0.808 | 0.81331 | 0.01 | | 1 | 1 | 0 | 1998 | 1998 |
| Rheumatoid Arthritis | C0003873 | PIM1 | 5292 | P11309 | Pim-1 proto-oncogene, serine/threonine kinase | Kinase | 224 | 0.482 | 0.769 | 0.84846 | 0.01 | | 1 | 1 | 0 | 2019 | 2019 |
| Rheumatoid Arthritis | C0003873 | PIN1 | 5300 | Q13526 | peptidylprolyl cis/trans isomerase, NIMA-interacting 1 | | 212 | 0.488 | 0.769 | 0.74031 | 0.01 | | 1 | 1 | 0 | 2009 | 2009 |
| Rheumatoid Arthritis | C0003873 | PKD1 | 5310 | P98161 | polycystin 1, transient receptor potential channel interacting | | 217 | 0.493 | 0.769 | 1 | 0.01 | | 1 | 1 | 0 | 2001 | 2001 |
| Rheumatoid Arthritis | C0003873 | PLA2G2A | 5320 | P14555 | phospholipase A2 group IIA | Enzyme | 215 | 0.488 | 0.808 | 0.097278 | 0.01 | | 1 | 1 | 0 | 2011 | 2011 |
| Rheumatoid Arthritis | C0003873 | PLAT | 5327 | P00750 | plasminogen activator, tissue type | Enzyme | 392 | 0.445 | 0.885 | 5.66E-05 | 0.01 | | 1 | 1 | 0 | 2006 | 2006 |

| | | | | | | | | | | | | | | | | | |
|---|---|---|---|---|---|---|---|---|---|---|---|---|---|---|---|---|---|
| Rheumatoid Arthritis | C0003873 | PLAUR | 5329 | Q03405 | plasminogen activator, urokinase receptor | | 147 | 0.536 | 0.731 | 0.005338 | 0.01 | | 1 | 1 | 0 | 2018 | 2018 |
| Rheumatoid Arthritis | C0003873 | SPA17 | 53340 | Q15506 | sperm autoantigenic protein 17 | | 48 | 0.647 | 0.577 | 0.006616 | 0.01 | | 1 | 1 | 0 | 2002 | 2002 |
| Rheumatoid Arthritis | C0003873 | PLCG2 | 5336 | P16885 | phospholipase C gamma 2 | Enzyme | 82 | 0.588 | 0.808 | 0.99562 | 0.01 | | 1 | 1 | 0 | 2017 | 2017 |
| Rheumatoid Arthritis | C0003873 | PLD1 | 5337 | Q13393 | phospholipase D1 | | 104 | 0.576 | 0.731 | 4.78E-21 | 0.01 | | 1 | 1 | 0 | 2011 | 2011 |
| Rheumatoid Arthritis | C0003873 | SERPINF2 | 5345 | P08697 | serpin family F member 2 | Enzyme modulator | 104 | 0.575 | 0.808 | 0.013624 | 0.01 | | 1 | 1 | 0 | 1996 | 1996 |
| Rheumatoid Arthritis | C0003873 | PLK1 | 5347 | P53350 | polo like kinase 1 | Kinase | 253 | 0.467 | 0.808 | 0.97851 | 0.01 | | 1 | 1 | 0 | 2007 | 2007 |
| Rheumatoid Ar | C0003873 | PLTP | 5360 | P55058 | phospholipid transfer protein | | 62 | 0.633 | 0.654 | 9E-09 | 0.01 | | 1 | 1 | 0 | 2018 | 2018 |

| | | | | | | | | | | | | | | | | | |
|---|---|---|---|---|---|---|---|---|---|---|---|---|---|---|---|---|---|
| thritis | | | | | | | | | | | | | | | | | |
| Rheumatoid Arthritis | C0003873 | IL20RA | 53832 | Q9UHF4 | interleukin 20 receptor subunit alpha | Receptor | 25 | 0.716 | 0.346 | 0.027129 | 0.01 | | 1 | 1 | 0 | 2019 | 2019 |
| Rheumatoid Arthritis | C0003873 | IL20RB | 53833 | Q6UXL0 | interleukin 20 receptor subunit beta | Receptor | 12 | 0.769 | 0.346 | 3.65E-08 | 0.01 | | 0 | 1 | 0 | 2016 | 2016 |
| Rheumatoid Arthritis | C0003873 | H2BS1 | 54145 | P57053 | H2B.S histone 1 | | 22 | 0.711 | 0.615 | | 0.01 | | 1 | 1 | 0 | 2019 | 2019 |
| Rheumatoid Arthritis | C0003873 | CYCS | 54205 | P99999 | cytochrome c, somatic | | 67 | 0.599 | 0.769 | 0.63647 | 0.01 | | 1 | 1 | 0 | 2012 | 2012 |
| Rheumatoid Arthritis | C0003873 | POLD1 | 5424 | P28340 | DNA polymerase delta 1, catalytic subunit | | 189 | 0.507 | 0.769 | 2.42E-06 | 0.01 | | 1 | 1 | 0 | 1999 | 1999 |
| Rheumatoid Arthritis | C0003873 | TERF2IP | 54386 | Q9NYB0 | TERF2 interacting protein | | 126 | 0.548 | 0.731 | 2.67E-05 | 0.01 | | 1 | 1 | 0 | 2006 | 2006 |
| Rheumatoi | C0003 | SIAE | 544 | Q9HAT2 | sialic acid acetylesterase | Enzyme | 30 | 0.7 | 0.5 | 7.79E | 0.01 | | 1 | 1 | 0 | 2015 | 2015 |

| | | | | | | | | | | | | | | | | | |
|---|---|---|---|---|---|---|---|---|---|---|---|---|---|---|---|---|---|
| d Arthritis | 873 | | 14 | | | | | 11 | 77 | -12 | | | | | | | |
| Rheumatoid Arthritis | C0003873 | POMC | 5443 | P01189 | proopiomelanocortin | | 873 | 0.356 | 0.846 | 0.0001117 | 0.01 | | 1 | 1 | 0 | 2001 | 2001 |
| Rheumatoid Arthritis | C0003873 | PON2 | 5445 | Q15165 | paraoxonase 2 | | 117 | 0.566 | 0.808 | 5.44E-05 | 0.01 | | 1 | 1 | 0 | 2018 | 2018 |
| Rheumatoid Arthritis | C0003873 | SMOX | 54498 | Q9NWM0 | spermine oxidase | | 91 | 0.603 | 0.769 | 0.39964 | 0.01 | | 1 | 1 | 0 | 2017 | 2017 |
| Rheumatoid Arthritis | C0003873 | ATR | 545 | Q13535 | ATR serine/threonine kinase | Kinase | 321 | 0.456 | 0.923 | 3.52E-09 | 0.01 | | 1 | 1 | 0 | 2006 | 2006 |
| Rheumatoid Arthritis | C0003873 | POU2F1 | 5451 | P14859 | POU class 2 homeobox 1 | | 115 | 0.558 | 0.769 | 0.90229 | 0.01 | | 1 | 1 | 0 | 1998 | 1998 |
| Rheumatoid Arthritis | C0003873 | POU2F2 | 5452 | P09086 | POU class 2 homeobox 2 | | 69 | 0.599 | 0.692 | 0.99884 | 0.01 | | 1 | 1 | 0 | 2019 | 2019 |
| Rheu | C00 | UGT1 | 54 | Q9HAW8 | UDP glucuronosyltr | | 88 | 0. | 0. | 4.5 | 0.01 | | 1 | 1 | 0 | 2012 | 20 |

| | | | | | | | | | | | | | | | | | |
|---|---|---|---|---|---|---|---|---|---|---|---|---|---|---|---|---|---|
| matoid Arthritis | 03873 | A10 | 575 | | ansferase family 1 member A10 | | | 588 | 615 | 9E-19 | | | | | | | 12 |
| Rheumatoid Arthritis | C0003873 | UGT1A8 | 54576 | Q9HAW9 | UDP glucuronosyltransferase family 1 member A8 | | 90 | 0.588 | 0.654 | 1.31E-09 | 0.01 | | 1 | 1 | 0 | 2012 | 2012 |
| Rheumatoid Arthritis | C0003873 | UGT1A7 | 54577 | Q9HAW7 | UDP glucuronosyltransferase family 1 member A7 | | 125 | 0.55 | 0.692 | 8.66E-14 | 0.01 | | 1 | 1 | 0 | 2012 | 2012 |
| Rheumatoid Arthritis | C0003873 | UGT1A6 | 54578 | P19224 | UDP glucuronosyltransferase family 1 member A6 | | 115 | 0.56 | 0.654 | 3.05E-07 | 0.01 | | 1 | 1 | 0 | 2012 | 2012 |
| Rheumatoid Arthritis | C0003873 | EGLN1 | 54583 | Q9GZT9 | egl-9 family hypoxia inducible factor 1 | | 130 | 0.54 | 0.731 | 0.97403 | 0.01 | | 1 | 1 | 0 | 2012 | 2012 |
| Rheumatoid Arthritis | C0003873 | PPARA | 5465 | Q07869 | peroxisome proliferator activated receptor alpha | Nuclear receptor | 408 | 0.432 | 0.885 | 0.030182 | 0.01 | | 1 | 1 | 0 | 2019 | 2019 |
| Rheumatoid Arthritis | C0003873 | UGT1A4 | 54657 | P22310 | UDP glucuronosyltransferase family 1 member A4 | | 84 | 0.595 | 0.654 | 5.98E-07 | 0.01 | | 1 | 1 | 0 | 2012 | 2012 |

| | | | | | | | | | | | | | | | | | |
|---|---|---|---|---|---|---|---|---|---|---|---|---|---|---|---|---|---|
| Rheumatoid Arthritis | C0003873 | UGT1A1 | 54658 | P22309 | UDP glucuronosyltransferase family 1 member A1 | | 302 | 0.464 | 0.769 | 3.26E-06 | 0.01 | | 1 | 1 | 0 | 2012 | 2012 |
| Rheumatoid Arthritis | C0003873 | PPBP | 5473 | P02775 | pro-platelet basic protein | Signaling | 111 | 0.544 | 0.808 | 0.0577739 | 0.01 | | 1 | 1 | 0 | 2016 | 2016 |
| Rheumatoid Arthritis | C0003873 | IL17RD | 54756 | Q8NFM7 | interleukin 17 receptor D | | 82 | 0.617 | 0.654 | 1.05E-06 | 0.01 | | 1 | 1 | 0 | 2017 | 2017 |
| Rheumatoid Arthritis | C0003873 | SAMD9 | 54809 | Q5K651 | sterile alpha motif domain containing 9 | | 90 | 0.606 | 0.808 | 1.05E-33 | 0.01 | | 1 | 1 | 0 | 2019 | 2019 |
| Rheumatoid Arthritis | C0003873 | QPCTL | 54814 | Q9NXS2 | glutaminyl-peptide cyclotransferase like | Enzyme | 18 | 0.805 | 0.346 | 1.57E-08 | 0.01 | | 1 | 1 | 0 | 2019 | 2019 |
| Rheumatoid Arthritis | C0003873 | ZCCHC2 | 54877 | Q9C0B9 | zinc finger CCHC-type containing 2 | | 9 | 0.805 | 0.308 | 0.99376 | 0.01 | | 1 | 1 | 0 | 2009 | 2009 |
| Rheumatoid Ar | C0003873 | UHRF1BP1 | 54887 | Q6BDS2 | UHRF1 binding protein 1 | | 26 | 0.769 | 0.269 | 4.47E- | 0.01 | | 1 | 1 | 0 | 2016 | 2016 |

| | | | | | | | | | | | | | | | | | |
|---|---|---|---|---|---|---|---|---|---|---|---|---|---|---|---|---|---|
| thritis | | | | | | | | | | 13 | | | | | | | |
| Rheumatoid Arthritis | C0003873 | CDKAL1 | 54901 | Q5VV42 | CDK5 regulatory subunit associated protein 1 like 1 | | 86 | 0.617 | 0.615 | 0.0160021 | 0.01 | | 1 | 1 | 1 | 2017 | 2017 |
| Rheumatoid Arthritis | C0003873 | PPM1A | 5494 | P35813 | protein phosphatase, Mg2+/Mn2+ dependent 1A | Enzyme | 33 | 0.691 | 0.5 | 0.99834 | 0.01 | | 1 | 1 | 0 | 2020 | 2020 |
| Rheumatoid Arthritis | C0003873 | BANP | 54971 | Q8N9N5 | BTG3 associated nuclear protein | | 27 | 0.743 | 0.269 | 0.99388 | 0.01 | | 1 | 1 | 0 | 2015 | 2015 |
| Rheumatoid Arthritis | C0003873 | MARCHF1 | 55016 | Q8TCQ1 | membrane associated ring-CH-type finger 1 | | 126 | 0.54 | 0.808 | 0.008678 | 0.01 | | 1 | 1 | 0 | 2017 | 2017 |
| Rheumatoid Arthritis | C0003873 | PPP1R1A | 5502 | Q13522 | protein phosphatase 1 regulatory inhibitor subunit 1A | Enzyme modulator | 106 | 0.559 | 0.692 | 0.021894 | 0.01 | | 1 | 1 | 0 | 2016 | 2016 |
| Rheumatoid Arthritis | C0003873 | TTC38 | 55020 | Q5R3I4 | tetratricopeptide repeat domain 38 | | 1 | 1 | 0.115 | 3.26E-13 | 0.01 | | 1 | 1 | 0 | 2017 | 2017 |
| Rheumatoi | C0003 | AKIRIN2 | 551 | Q53H80 | akirin 2 | | 28 | 0.7 | 0.3 | 0.923 | 0.01 | | 1 | 1 | 0 | 2011 | 2011 |

| | | | | | | | | | | | | | | | | |
|---|---|---|---|---|---|---|---|---|---|---|---|---|---|---|---|---|
| d Arthritis | 873 | | 22 | | | | | 16 | 46 | 55 | | | | | | |
| Rheumatoid Arthritis | C0003873 | PIWIL2 | 55124 | Q8TC59 | piwi like RNA-mediated gene silencing 2 | 65 | 0.612 | 0.577 | 2.71E-08 | 0.01 | | 1 | 1 | 0 | 2016 | 2016 |
| Rheumatoid Arthritis | C0003873 | RMDN3 | 55177 | Q96TC7 | regulator of microtubule dynamics 3 | 134 | 0.534 | 0.769 | 5.17E-05 | 0.01 | | 1 | 1 | 0 | 2019 | 2019 |
| Rheumatoid Arthritis | C0003873 | NADSYN1 | 55191 | Q6IA69 | NAD synthetase 1 | 14 | 0.78 | 0.231 | 1.25E-16 | 0.01 | | 1 | 1 | 0 | 2014 | 2014 |
| Rheumatoid Arthritis | C0003873 | THNSL2 | 55258 | Q86YJ6 | threonine synthase like 2 | 4 | 0.861 | 0.154 | 1.48E-06 | 0.01 | | 1 | 1 | 0 | 2009 | 2009 |
| Rheumatoid Arthritis | C0003873 | KIR2DL5B | 553128 | Q8NHK3 | killer cell immunoglobulin like receptor, two Ig domains and long cytoplasmic tail 5B | 27 | 0.682 | 0.423 | | 0.01 | | 1 | 1 | 0 | 2015 | 2015 |
| Rheumatoid Arthritis | C0003873 | IMPACT | 55364 | Q9P2X3 | impact RWD domain protein | 85 | 0.593 | 0.808 | 4.83E-08 | 0.01 | | 1 | 1 | 0 | 2019 | 2019 |

| | | | | | | | | | | | | | | | | | |
|---|---|---|---|---|---|---|---|---|---|---|---|---|---|---|---|---|---|
| Rheumatoid Arthritis | C0003873 | MIR448 | 554212 | | microRNA 448 | | 63 | 0.61 | 0.615 | | 0.01 | | 1 | 1 | 0 | 2018 | 2018 |
| Rheumatoid Arthritis | C0003873 | PRCP | 5547 | P42785 | prolylcarboxypeptidase | Enzyme | 68 | 0.633 | 0.692 | 8.76E-12 | 0.01 | | 1 | 1 | 0 | 2009 | 2009 |
| Rheumatoid Arthritis | C0003873 | PRF1 | 5551 | P14222 | perforin 1 | | 163 | 0.54 | 0.692 | 5.49E-06 | 0.01 | | 1 | 1 | 0 | 2013 | 2013 |
| Rheumatoid Arthritis | C0003873 | SYBU | 55638 | Q9NX95 | syntabulin | Transporter | 120 | 0.546 | 0.769 | 9.77E-05 | 0.01 | | 1 | 1 | 0 | 2019 | 2019 |
| Rheumatoid Arthritis | C0003873 | NLRP2 | 55655 | Q9NX02 | NLR family pyrin domain containing 2 | | 155 | 0.515 | 0.731 | 1.42E-38 | 0.01 | | 1 | 1 | 2 | 2015 | 2015 |
| Rheumatoid Arthritis | C0003873 | ZNF334 | 55713 | Q9HCZ1 | zinc finger protein 334 | | 1 | 1 | 0.115 | 1.61E-09 | 0.01 | | 1 | 1 | 0 | 2011 | 2011 |
| Rheumatoid Ar | C0003873 | ATF7IP | 55729 | Q6VMQ6 | activating transcription factor 7 interacting protein | | 145 | 0.543 | 0.769 | 0.99993 | 0.01 | | 1 | 1 | 0 | 2015 | 2015 |

| | | | | | | | | | | | | | | | | | |
|---|---|---|---|---|---|---|---|---|---|---|---|---|---|---|---|---|---|
| thritis | | | | | | | | | | | | | | | | | |
| Rheumatoid Arthritis | C0003873 | DHX32 | 55760 | Q7L7V1 | DEAH-box helicase 32 (putative) | Enzyme | 20 | 0.76 | 0.269 | 1.65E-09 | 0.01 | | 1 | 1 | 0 | 2015 | 2015 |
| Rheumatoid Arthritis | C0003873 | PAG1 | 55824 | Q9NWQ8 | phosphoprotein membrane anchor with glycosphingolipid microdomains 1 | | 174 | 0.505 | 0.808 | 0.017764 | 0.01 | | 1 | 1 | 0 | 2018 | 2018 |
| Rheumatoid Arthritis | C0003873 | SELENOS | 55829 | Q9BQE4 | selenoprotein S | | 55 | 0.628 | 0.615 | 1.43E-09 | 0.01 | | 1 | 1 | 0 | 2009 | 2009 |
| Rheumatoid Arthritis | C0003873 | CENPJ | 55835 | Q9HC77 | centromere protein J | Enzyme | 150 | 0.546 | 0.846 | 2.06E-27 | 0.01 | | 1 | 1 | 0 | 2008 | 2008 |
| Rheumatoid Arthritis | C0003873 | PKN1 | 5585 | Q16512 | protein kinase N1 | Kinase | 48 | 0.647 | 0.692 | 0.20442 | 0.01 | | 1 | 1 | 0 | 2012 | 2012 |
| Rheumatoid Arthritis | C0003873 | PRKCZ | 5590 | Q05513 | protein kinase C zeta | Kinase | 88 | 0.604 | 0.654 | 0.541 | 0.01 | | 1 | 1 | 0 | 2008 | 2008 |
| Rheumatoi | C0003 | MAPK9 | 5601 | P45984 | mitogen-activate | Kinase | 115 | 0.5 | 0.7 | 0.069 | 0.01 | | 1 | 1 | 0 | 2007 | 2007 |

| | | | | | | | | | | | | | | | | | |
|---|---|---|---|---|---|---|---|---|---|---|---|---|---|---|---|---|---|
| d Arthritis | 873 | | | | d protein kinase 9 | | | 61 | 31 | 738 | | | | | | | |
| Rheumatoid Arthritis | C0003873 | MAPK10 | 5602 | P53779 | mitogen-activated protein kinase 10 | Kinase | 75 | 0.606 | 0.692 | 0.27553 | 0.01 | | 1 | 1 | 0 | 2019 | 2019 |
| Rheumatoid Arthritis | C0003873 | MAPK13 | 5603 | O15264 | mitogen-activated protein kinase 13 | Kinase | 26 | 0.705 | 0.577 | 6.72E-11 | 0.01 | | 1 | 1 | 0 | 2004 | 2004 |
| Rheumatoid Arthritis | C0003873 | EIF2AK2 | 5610 | P19525 | eukaryotic translation initiation factor 2 alpha kinase 2 | Kinase | 142 | 0.538 | 0.769 | 0.017643 | 0.01 | | 1 | 1 | 0 | 2015 | 2015 |
| Rheumatoid Arthritis | C0003873 | ANKH | 56172 | Q9HCJ1 | ANKH inorganic pyrophosphate transport regulator | Transporter | 89 | 0.606 | 0.692 | 0.27856 | 0.01 | | 1 | 1 | 0 | 2007 | 2007 |
| Rheumatoid Arthritis | C0003873 | PROP1 | 5626 | O75360 | PROP paired-like homeobox 1 | | 117 | 0.576 | 0.615 | 0.087746 | 0.01 | | 1 | 1 | 0 | 2018 | 2018 |
| Rheumatoid Arthritis | C0003873 | AZGP1 | 563 | P25311 | alpha-2-glycoprotein 1, zinc-binding | | 101 | 0.566 | 0.769 | 4.15E-06 | 0.01 | | 1 | 1 | 0 | 2017 | 2017 |

| | | | | | | | | | | | | | | | | | |
|---|---|---|---|---|---|---|---|---|---|---|---|---|---|---|---|---|---|
| Rheumatoid Arthritis | C0003873 | METTL3 | 56339 | Q86U44 | methyltransferase like 3 | | 79 | 0.585 | 0.538 | 1.8E-07 | 0.01 | | 1 | 1 | 0 | 2019 | 2019 |
| Rheumatoid Arthritis | C0003873 | PPAN | 56342 | Q9NQ55 | peter pan homolog | | 18 | 0.729 | 0.577 | 1.72E-12 | 0.01 | | 1 | 1 | 0 | 2019 | 2019 |
| Rheumatoid Arthritis | C0003873 | LTB4R2 | 56413 | Q9NPC1 | leukotriene B4 receptor 2 | G-protein coupled receptor | 49 | 0.631 | 0.615 | 1.38E-05 | 0.01 | | 1 | 1 | 0 | 2003 | 2003 |
| Rheumatoid Arthritis | C0003873 | PRSS2 | 5645 | P07478 | serine protease 2 | Enzyme | 66 | 0.628 | 0.769 | | 0.01 | | 1 | 1 | 0 | 2005 | 2005 |
| Rheumatoid Arthritis | C0003873 | MASP1 | 5648 | P48740 | mannan binding lectin serine peptidase 1 | Enzyme | 123 | 0.565 | 0.885 | 1.23E-11 | 0.01 | | 1 | 1 | 0 | 2016 | 2016 |
| Rheumatoid Arthritis | C0003873 | CYP26B1 | 56603 | Q9NR63 | cytochrome P450 family 26 subfamily B member 1 | Enzyme | 36 | 0.666 | 0.5 | 0.97987 | 0.01 | | 1 | 1 | 0 | 2018 | 2018 |
| Rheumatoi | C0003 | PSEN1 | 5663 | P49768 | presenilin 1 | Enzyme | 369 | 0.4 | 0.8 | 0.973 | 0.01 | | 1 | 1 | 1 | 1998 | 1998 |

| | | | | | | | | | | | | | | | | | |
|---|---|---|---|---|---|---|---|---|---|---|---|---|---|---|---|---|---|
| d Arthritis | 873 | | | | | | | 69 | 46 | 22 | | | | | | | |
| Rheumatoid Arthritis | C0003873 | SAR1A | 56681 | Q9NR31 | secretion associated Ras related GTPase 1A | | 25 | 0.743 | 0.538 | 0.37178 | 0.01 | | 1 | 1 | 0 | 2007 | 2007 |
| Rheumatoid Arthritis | C0003873 | SPHK2 | 56848 | Q9NRA0 | sphingosine kinase 2 | Kinase | 140 | 0.546 | 0.731 | 1.28E-07 | 0.01 | | 1 | 1 | 0 | 2009 | 2009 |
| Rheumatoid Arthritis | C0003873 | PSMB5 | 5693 | P28074 | proteasome 20S subunit beta 5 | | 27 | 0.711 | 0.385 | 0.95893 | 0.01 | | 1 | 1 | 0 | 2012 | 2012 |
| Rheumatoid Arthritis | C0003873 | ARNTL2 | 56938 | Q8WYA1 | aryl hydrocarbon receptor nuclear translocator like 2 | Transcription factor | 41 | 0.644 | 0.654 | 6.04E-10 | 0.01 | | 1 | 1 | 0 | 2015 | 2015 |
| Rheumatoid Arthritis | C0003873 | PSMB6 | 5694 | P28072 | proteasome 20S subunit beta 6 | | 100 | 0.561 | 0.731 | 0.015779 | 0.01 | | 1 | 1 | 0 | 1990 | 1990 |
| Rheumatoid Arthritis | C0003873 | DUSP22 | 56940 | Q9NRW4 | dual specificity phosphatase 22 | | 40 | 0.659 | 0.615 | 0.009342 | 0.01 | | 1 | 1 | 0 | 2018 | 2018 |

| | | | | | | | | | | | | | | | | | |
|---|---|---|---|---|---|---|---|---|---|---|---|---|---|---|---|---|---|
| Rheumatoid Arthritis | C0003873 | PRDM8 | 56978 | Q9NQV8 | PR/SET domain 8 | | 39 | 0.678 | 0.462 | 0.77626 | 0.01 | | 1 | 1 | 0 | 2019 | 2019 |
| Rheumatoid Arthritis | C0003873 | ADAMTS9 | 56999 | Q9P2N4 | ADAM metallopeptidase with thrombospondin type 1 motif 9 | Enzyme | 53 | 0.633 | 0.692 | 1E-06 | 0.01 | | 1 | 1 | 0 | 2018 | 2018 |
| Rheumatoid Arthritis | C0003873 | PCBP4 | 57060 | P57723 | poly(rC) binding protein 4 | Enzyme | 147 | 0.523 | 0.808 | 0.0023771 | 0.01 | | 1 | 1 | 0 | 2018 | 2018 |
| Rheumatoid Arthritis | C0003873 | PCNP | 57092 | Q8WW12 | PEST proteolytic signal containing nuclear protein | | 11 | 0.839 | 0.192 | 0.78553 | 0.01 | | 1 | 1 | 0 | 2017 | 2017 |
| Rheumatoid Arthritis | C0003873 | PNPLA2 | 57104 | Q96AD5 | patatin like phospholipase domain containing 2 | Enzyme | 199 | 0.511 | 0.846 | 0.004107 | 0.01 | | 1 | 1 | 0 | 2017 | 2017 |
| Rheumatoid Arthritis | C0003873 | CD248 | 57124 | Q9HCU0 | CD248 molecule | | 76 | 0.615 | 0.577 | 0.059017 | 0.01 | | 1 | 1 | 0 | 2010 | 2010 |
| Rheumatoid Ar | C0003873 | PSMD9 | 5715 | O00233 | proteasome 26S subunit, non-ATPase 9 | Enzyme modul | 344 | 0.435 | 0.846 | 4.86E- | 0.01 | | 1 | 1 | 0 | 2005 | 2005 |

| | | | | | | | | | | | | | | | | | |
|---|---|---|---|---|---|---|---|---|---|---|---|---|---|---|---|---|---|
| thritis | | | | | | ator | | | | 06 | | | | | | | |
| Rheumatoid Arthritis | C0003873 | SLURP1 | 57152 | P55000 | secreted LY6/PLAUR domain containing 1 | | 105 | 0.56 | 0.654 | 0.25934 | 0.01 | | 1 | 1 | 0 | 2019 | 2019 |
| Rheumatoid Arthritis | C0003873 | PTBP1 | 5725 | P26599 | polypyrimidine tract binding protein 1 | | 172 | 0.517 | 0.769 | 0.99961 | 0.01 | | 1 | 1 | 0 | 1996 | 1996 |
| Rheumatoid Arthritis | C0003873 | KIR2DL5A | 57292 | Q8N109 | killer cell immunoglobulin like receptor, two Ig domains and long cytoplasmic tail 5A | Receptor | 21 | 0.711 | 0.423 | | 0.01 | | 1 | 1 | 0 | 2015 | 2015 |
| Rheumatoid Arthritis | C0003873 | PTGER2 | 5732 | P43116 | prostaglandin E receptor 2 | G-protein coupled receptor | 79 | 0.597 | 0.731 | 0.00749 | 0.01 | | 1 | 1 | 0 | 2009 | 2009 |
| Rheumatoid Arthritis | C0003873 | PTGER4 | 5734 | P35408 | prostaglandin E receptor 4 | G-protein coupled receptor | 120 | 0.558 | 0.731 | 0.9755 | 0.01 | | 1 | 1 | 0 | 2015 | 2015 |
| Rheumatoi | C0003 | MIR363 | 5740 | | microRNA 363 | | 83 | 0.5 | 0.6 | | 0.01 | | 1 | 1 | 0 | 2017 | 2017 |

| | | | | | | | | | | | | | | | | | |
|---|---|---|---|---|---|---|---|---|---|---|---|---|---|---|---|---|---|
| d Arthritis | 873 | | 31 | | | | | 78 | 92 | | | | | | | | |
| Rheumatoid Arthritis | C0003873 | ABHD6 | 57406 | Q9BV23 | abhydrolase domain containing 6 | Enzyme | 24 | 0.751 | 0.385 | 2.23E-05 | 0.01 | | 1 | 1 | 0 | 2017 | 2017 |
| Rheumatoid Arthritis | C0003873 | MIR432 | 574451 | | microRNA 432 | | 44 | 0.644 | 0.577 | | 0.01 | | 1 | 1 | 0 | 2018 | 2018 |
| Rheumatoid Arthritis | C0003873 | MIR181D | 574457 | | microRNA 181d | | 44 | 0.641 | 0.654 | | 0.01 | | 1 | 1 | 0 | 2015 | 2015 |
| Rheumatoid Arthritis | C0003873 | MIR498 | 574460 | | microRNA 498 | | 49 | 0.633 | 0.5 | | 0.01 | | 1 | 1 | 0 | 2019 | 2019 |
| Rheumatoid Arthritis | C0003873 | MIR522 | 574495 | | microRNA 522 | | 21 | 0.722 | 0.346 | | 0.01 | | 1 | 1 | 0 | 2018 | 2018 |
| Rheumatoid Arthritis | C0003873 | MIR506 | 574511 | | microRNA 506 | | 76 | 0.585 | 0.615 | | 0.01 | | 1 | 1 | 0 | 2019 | 2019 |

| | | | | | | | | | | | | | | | | | |
|---|---|---|---|---|---|---|---|---|---|---|---|---|---|---|---|---|---|
| Rheumatoid Arthritis | C0003873 | REXO1 | 57455 | Q8N1G1 | RNA exonuclease 1 homolog | Enzyme | 30 | 0.695 | 0.346 | 0.9864 | 0.01 | | 1 | 1 | 0 | 2010 | 2010 |
| Rheumatoid Arthritis | C0003873 | SRGAP1 | 57522 | Q7Z6B7 | SLIT-ROBO Rho GTPase activating protein 1 | Enzyme modulator | 29 | 0.686 | 0.5 | 0.99845 | 0.01 | | 1 | 1 | 0 | 2018 | 2018 |
| Rheumatoid Arthritis | C0003873 | PTMA | 5757 | P06454 | prothymosin alpha | | 71 | 0.604 | 0.731 | 0.0344451 | 0.01 | | 1 | 1 | 0 | 2007 | 2007 |
| Rheumatoid Arthritis | C0003873 | PTMAP4 | 5761 | | prothymosin alpha pseudogene 4 | | 49 | 0.638 | 0.731 | | 0.01 | | 1 | 1 | 0 | 2007 | 2007 |
| Rheumatoid Arthritis | C0003873 | EPB41L5 | 57669 | Q9HCM4 | erythrocyte membrane protein band 4.1 like 5 | | 16 | 0.78 | 0.346 | 0.020795 | 0.01 | | 1 | 1 | 0 | 2018 | 2018 |
| Rheumatoid Arthritis | C0003873 | MAGEE1 | 57692 | Q9HCI5 | MAGE family member E1 | Cell adhesion | 19 | 0.736 | 0.423 | 0.97046 | 0.01 | | 1 | 1 | 0 | 2006 | 2006 |
| Rheumatoid Ar | C0003873 | GRHL3 | 57822 | Q8TE85 | grainyhead like transcription factor 3 | Transcription | 106 | 0.572 | 0.692 | 0.99292 | 0.01 | | 1 | 1 | 0 | 1997 | 1997 |

| | | | | | | | | | | | | | | | | | |
|---|---|---|---|---|---|---|---|---|---|---|---|---|---|---|---|---|---|
| thritis | | | | | | factor | | | | | | | | | | | |
| Rheumatoid Arthritis | C0003873 | PTPN14 | 5784 | Q15678 | protein tyrosine phosphatase non-receptor type 14 | | 48 | 0.638 | 0.615 | 0.22055 | 0.01 | | 1 | 1 | 0 | 2019 | 2019 |
| Rheumatoid Arthritis | C0003873 | MUC3B | 57876 | Q9H195 | mucin 3B, cell surface associated | | 41 | 0.666 | 0.462 | | 0.01 | | 1 | 1 | 0 | 2008 | 2008 |
| Rheumatoid Arthritis | C0003873 | PTPRJ | 5795 | Q12913 | protein tyrosine phosphatase receptor type J | Enzyme | 79 | 0.606 | 0.615 | 1.29E-11 | 0.01 | | 1 | 1 | 0 | 2013 | 2013 |
| Rheumatoid Arthritis | C0003873 | PTPRK | 5796 | Q15262 | protein tyrosine phosphatase receptor type K | Enzyme | 34 | 0.686 | 0.385 | 0.90291 | 0.01 | | 1 | 1 | 0 | 2016 | 2016 |
| Rheumatoid Arthritis | C0003873 | BAX | 581 | Q07812 | BCL2 associated X, apoptosis regulator | Signaling | 420 | 0.417 | 0.885 | 0.31746 | 0.01 | | 0 | 1 | 0 | 1999 | 1999 |
| Rheumatoid Arthritis | C0003873 | RBPJP4 | 58163 | | RBPJ pseudogene 4 | | 12 | 0.805 | 0.423 | | 0.01 | | 1 | 1 | 0 | 2006 | 2006 |
| Rheumatoi | C0003 | JAM2 | 584 | P57087 | junctional adhesion molecule 2 | | 17 | 0.7 | 0.4 | 0.006 | 0.01 | | 0 | 1 | 1 | 2014 | 2014 |

| | | | | | | | | | | | | | | | | | |
|---|---|---|---|---|---|---|---|---|---|---|---|---|---|---|---|---|---|
| d Arthritis | 873 | | 94 | | | | | 36 | 62 | 155 | | | | | | | |
| Rheumatoid Arthritis | C0003873 | RAG1 | 5896 | P15918 | recombination activating 1 | Enzyme | 209 | 0.509 | 0.769 | 2.77E-05 | 0.01 | | 1 | 1 | 0 | 2009 | 2009 |
| Rheumatoid Arthritis | C0003873 | RAG2 | 5897 | P55895 | recombination activating 2 | | 207 | 0.507 | 0.769 | 0.020287 | 0.01 | | 1 | 1 | 0 | 2009 | 2009 |
| Rheumatoid Arthritis | C0003873 | IL22RA1 | 58985 | Q8N6P7 | interleukin 22 receptor subunit alpha 1 | Receptor | 28 | 0.682 | 0.538 | 0.005686 | 0.01 | | 1 | 1 | 0 | 2018 | 2018 |
| Rheumatoid Arthritis | C0003873 | ACTA2 | 59 | P62736 | actin alpha 2, smooth muscle | Cellular structure | 200 | 0.508 | 0.846 | 0.930017 | 0.01 | | 1 | 1 | 2 | 2019 | 2019 |
| Rheumatoid Arthritis | C0003873 | RAP1A | 5906 | P62834 | RAP1A, member of RAS oncogene family | Enzyme modulator | 215 | 0.502 | 0.808 | 0.622227 | 0.01 | | 1 | 1 | 0 | 2006 | 2006 |
| Rheumatoid Arthritis | C0003873 | RARG | 5916 | P13631 | retinoic acid receptor gamma | Nuclear receptor | 62 | 0.608 | 0.731 | 0.99478 | 0.01 | | 1 | 1 | 0 | 2015 | 2015 |

| | | | | | | | | | | | | | | | | |
|---|---|---|---|---|---|---|---|---|---|---|---|---|---|---|---|---|
| Rheumatoid Arthritis | C0003873 | HIVEP3 | 59269 | Q5T1R4 | HIVEP zinc finger 3 | | 31 | 0.722 | 0.462 | 0.90584 | 0.01 | | 1 | 1 | 0 | 2016 | 2016 |
| Rheumatoid Arthritis | C0003873 | TRPV4 | 59341 | Q9HBA0 | transient receptor potential cation channel subfamily V member 4 | Ion channel | 422 | 0.457 | 0.808 | 2.24E-16 | 0.01 | | 1 | 1 | 0 | 2017 | 2017 |
| Rheumatoid Arthritis | C0003873 | PBOV1 | 59351 | Q9GZY1 | prostate and breast cancer overexpressed 1 | | 21 | 0.743 | 0.308 | | 0.01 | | 1 | 1 | 0 | 2018 | 2018 |
| Rheumatoid Arthritis | C0003873 | RBP4 | 5950 | P02753 | retinol binding protein 4 | Transporter | 217 | 0.493 | 0.808 | 0.51951 | 0.01 | | 1 | 1 | 0 | 2019 | 2019 |
| Rheumatoid Arthritis | C0003873 | OPN1LW | 5956 | P04000 | opsin 1, long wave sensitive | G-protein coupled receptor | 197 | 0.497 | 0.808 | 0.97535 | 0.01 | | 1 | 1 | 0 | 2018 | 2018 |
| Rheumatoid Arthritis | C0003873 | RET | 5979 | P07949 | ret proto-oncogene | Kinase | 607 | 0.392 | 0.885 | 1 | 0.01 | | 1 | 1 | 0 | 1995 | 1995 |
| Rheuma | C0003 | BCL2L1 | 598 | Q07817 | BCL2 like 1 | Signaling | 212 | 0.4 | 0.8 | 0.93 | 0.01 | | 1 | 1 | 0 | 2006 | 20 |

| | | | | | | | | | | | | | | | | | |
|---|---|---|---|---|---|---|---|---|---|---|---|---|---|---|---|---|---|
| toid Arthritis | 873 | | | | | | | 93 | 85 | 667 | | | | | | | 06 |
| Rheumatoid Arthritis | C0003873 | REV3L | 5980 | O60673 | REV3 like, DNA directed polymerase zeta catalytic subunit | | 95 | 0.588 | 0.654 | 1 | 0.01 | | 1 | 1 | 0 | 2014 | 2014 |
| Rheumatoid Arthritis | C0003873 | RGS1 | 5996 | Q08116 | regulator of G protein signaling 1 | Enzyme modulator | 47 | 0.653 | 0.731 | 0.78178 | 0.01 | | 1 | 1 | 0 | 2019 | 2019 |
| Rheumatoid Arthritis | C0003873 | RGS12 | 6002 | O14924 | regulator of G protein signaling 12 | | 22 | 0.769 | 0.462 | 4.09E-09 | 0.01 | | 1 | 1 | 0 | 2015 | 2015 |
| Rheumatoid Arthritis | C0003873 | RHCE | 6006 | P18577 | Rh blood group CcEe antigens | | 44 | 0.666 | 0.5 | 0.001163 | 0.01 | | 1 | 1 | 0 | 2018 | 2018 |
| Rheumatoid Arthritis | C0003873 | GRK1 | 6011 | Q15835 | G protein-coupled receptor kinase 1 | Kinase | 26 | 0.695 | 0.423 | 3.07E-05 | 0.01 | | 1 | 1 | 0 | 1999 | 1999 |
| Rheumatoid Arthritis | C0003873 | BCL3 | 602 | P20749 | BCL3 transcription coactivator | | 136 | 0.541 | 0.731 | 0.98871 | 0.01 | | 1 | 1 | 0 | 2015 | 2015 |

| | | | | | | | | | | | | | | | | | |
|---|---|---|---|---|---|---|---|---|---|---|---|---|---|---|---|---|---|
| Rheumatoid Arthritis | C0003873 | RMRP | 6023 | | RNA component of mitochondrial RNA processing endoribonuclease | | 239 | 0.519 | 0.731 | | 0.01 | | 1 | 1 | 0 | 2019 | 2019 |
| Rheumatoid Arthritis | C0003873 | RNASE1 | 6035 | P07998 | ribonuclease A family member 1, pancreatic | | 68 | 0.604 | 0.692 | 0.025608 | 0.01 | | 1 | 1 | 0 | 2016 | 2016 |
| Rheumatoid Arthritis | C0003873 | RNASE3 | 6037 | P12724 | ribonuclease A family member 3 | | 207 | 0.49 | 0.808 | | 0.01 | | 1 | 1 | 0 | 2004 | 2004 |
| Rheumatoid Arthritis | C0003873 | EXOC4 | 60412 | Q96A65 | exocyst complex component 4 | Transporter | 20 | 0.743 | 0.5 | 3.41E-13 | 0.01 | | 1 | 1 | 0 | 2005 | 2005 |
| Rheumatoid Arthritis | C0003873 | BRD2 | 6046 | P25440 | bromodomain containing 2 | Epigenetic regulator | 303 | 0.45 | 0.808 | 0.99957 | 0.01 | | 1 | 1 | 0 | 2016 | 2016 |
| Rheumatoid Arthritis | C0003873 | SLC5A7 | 60482 | Q9GZV3 | solute carrier family 5 member 7 | Transporter | 127 | 0.597 | 0.654 | 0.015272 | 0.01 | | 1 | 1 | 0 | 2015 | 2015 |
| Rheumatoid Ar | C0003873 | PPCDC | 60490 | Q96CD2 | phosphopantothenoylcysteine decarboxylase | | 8 | 0.861 | 0.192 | 0.0004 | 0.01 | | 1 | 1 | 0 | 2018 | 2018 |

| | | | | | | | | | | | | | | | | | |
|---|---|---|---|---|---|---|---|---|---|---|---|---|---|---|---|---|---|
| thritis | | | | | | | | | | 79 | | | | | | | |
| Rheumatoid Arthritis | C0003873 | GAS5 | 60674 | | growth arrest specific 5 | | 184 | 0.497 | 0.808 | | 0.01 | | 1 | 1 | 0 | 2019 | 2019 |
| Rheumatoid Arthritis | C0003873 | ROBO1 | 6091 | Q9Y6N7 | roundabout guidance receptor 1 | | 144 | 0.535 | 0.731 | 7.27E-16 | 0.01 | | 1 | 1 | 0 | 2018 | 2018 |
| Rheumatoid Arthritis | C0003873 | RORA | 6095 | P35398 | RAR related orphan receptor A | Nuclear receptor | 158 | 0.533 | 0.808 | 0.400096 | 0.01 | | 1 | 1 | 0 | 2019 | 2019 |
| Rheumatoid Arthritis | C0003873 | BCS1L | 617 | Q9Y276 | BCS1 homolog, ubiquinol-cytochrome c reductase complex chaperone | | 172 | 0.534 | 0.808 | 4.7E-13 | 0.01 | | 1 | 1 | 0 | 2017 | 2017 |
| Rheumatoid Arthritis | C0003873 | RPS4X | 6191 | P62701 | ribosomal protein S4 X-linked | Nucleic acid binding | 28 | 0.711 | 0.5 | 0.932222 | 0.01 | | 1 | 1 | 0 | 2013 | 2013 |
| Rheumatoid Arthritis | C0003873 | MIR486-1 | 619554 | | microRNA 486-1 | | 142 | 0.521 | 0.808 | | 0.01 | | 1 | 1 | 0 | 2017 | 2017 |
| Rheumatoi | C0003 | RRAS | 6237 | P10301 | RAS related | Enzyme mo | 149 | 0.5 | 0.8 | 0.004 | 0.01 | | 1 | 1 | 0 | 2020 | 2020 |

| | | | | | | | | | | | | | | | | | |
|---|---|---|---|---|---|---|---|---|---|---|---|---|---|---|---|---|---|
| d Arthritis | 873 | | | | | dulator | | 43 | 08 | 663 | | | | | | | |
| Rheumatoid Arthritis | C0003873 | RRM2 | 6241 | P31350 | ribonucleotide reductase regulatory subunit M2 | Enzyme | 101 | 0.555 | 0.769 | 0.2964 | 0.01 | | 1 | 1 | 0 | 2018 | 2018 |
| Rheumatoid Arthritis | C0003873 | RXRA | 6256 | P19793 | retinoid X receptor alpha | Nuclear receptor | 132 | 0.543 | 0.769 | 0.99852 | 0.01 | | 1 | 1 | 0 | 2018 | 2018 |
| Rheumatoid Arthritis | C0003873 | RYR1 | 6261 | P21817 | ryanodine receptor 1 | Ion channel | 320 | 0.489 | 0.808 | 1.59E-29 | 0.01 | | 0 | 1 | 0 | 1989 | 1989 |
| Rheumatoid Arthritis | C0003873 | S100A10 | 6281 | P60903 | S100 calcium binding protein A10 | Calcium-binding protein | 150 | 0.528 | 0.769 | 0.16843 | 0.01 | | 1 | 1 | 0 | 2018 | 2018 |
| Rheumatoid Arthritis | C0003873 | S100A11 | 6282 | P31949 | S100 calcium binding protein A11 | Calcium-binding protein | 103 | 0.556 | 0.731 | 0.008466 | 0.01 | | 1 | 1 | 0 | 2017 | 2017 |
| Rheumatoid Ar | C0003873 | SAA4 | 6291 | P35542 | serum amyloid A4, constitutive | Transporter | 13 | 0.78 | 0.269 | 0.0123 | 0.01 | | 1 | 1 | 0 | 2017 | 2017 |

| | | | | | | | | | | | | | | | | | |
|---|---|---|---|---|---|---|---|---|---|---|---|---|---|---|---|---|---|
| thritis | | | | | | | | | | 46 | | | | | | | |
| Rheumatoid Arthritis | C0003873 | SCD | 6319 | O00767 | stearoyl-CoA desaturase | | 225 | 0.49 | 0.808 | 0.96968 | 0.01 | | 1 | 1 | 0 | 2019 | 2019 |
| Rheumatoid Arthritis | C0003873 | SRL | 6345 | Q86TD4 | sarcalumenin | Enzyme modulator | 77 | 0.597 | 0.731 | 8E-08 | 0.01 | | 1 | 1 | 0 | 2004 | 2004 |
| Rheumatoid Arthritis | C0003873 | CCL1 | 6346 | P22362 | C-C motif chemokine ligand 1 | Signaling | 79 | 0.587 | 0.692 | 0.007967 | 0.01 | | 1 | 1 | 0 | 2017 | 2017 |
| Rheumatoid Arthritis | C0003873 | CCL11 | 6356 | P51671 | C-C motif chemokine ligand 11 | Signaling | 250 | 0.473 | 0.885 | 0.010211 | 0.01 | | 1 | 1 | 0 | 2017 | 2017 |
| Rheumatoid Arthritis | C0003873 | CCL14 | 6358 | Q16627 | C-C motif chemokine ligand 14 | Signaling | 29 | 0.695 | 0.423 | 0.000109 | 0.01 | | 1 | 1 | 0 | 2017 | 2017 |
| Rheumatoid Arthritis | C0003873 | CCL23 | 6368 | P55773 | C-C motif chemokine ligand 23 | Signaling | 18 | 0.76 | 0.346 | 0.000597 | 0.01 | | 1 | 1 | 0 | 2008 | 2008 |
| Rheumatoi | C0003 | CCL24 | 6369 | O00175 | C-C motif chemokine | Signaling | 67 | 0.6 | 0.7 | 0.007 | 0.01 | | 1 | 1 | 0 | 2005 | 2005 |

| | | | | | | | | | | | | | | | | | |
|---|---|---|---|---|---|---|---|---|---|---|---|---|---|---|---|---|---|
| d Arthritis | 873 | | | | ligand 24 | | | 08 | 31 | 089 | | | | | | | |
| Rheumatoid Arthritis | C0003873 | XCL1 | 6375 | P47992 | X-C motif chemokine ligand 1 | Signaling | 67 | 0.601 | 0.692 | 0.001298 | 0.01 | | 1 | 1 | 0 | 2003 | 2003 |
| Rheumatoid Arthritis | C0003873 | SDC4 | 6385 | P31431 | syndecan 4 | Extracellular structure | 71 | 0.603 | 0.615 | 0.004148 | 0.01 | | 1 | 1 | 0 | 2020 | 2020 |
| Rheumatoid Arthritis | C0003873 | MRPL17 | 63875 | Q9NRX2 | mitochondrial ribosomal protein L17 | Nucleic acid binding | 4 | 0.861 | 0.192 | 0.028198 | 0.01 | | 1 | 1 | 0 | 2008 | 2008 |
| Rheumatoid Arthritis | C0003873 | ERVK-6 | 64006 | | endogenous retrovirus group K member 6, envelope | | 162 | 0.521 | 0.808 | | 0.01 | | 1 | 1 | 0 | 2017 | 2017 |
| Rheumatoid Arthritis | C0003873 | ERAP2 | 64167 | Q6P179 | endoplasmic reticulum aminopeptidase 2 | Enzyme | 58 | 0.615 | 0.577 | 1E-28 | 0.01 | | 1 | 1 | 0 | 2016 | 2016 |
| Rheumatoid Arthritis | C0003873 | SETMAR | 6419 | Q53H47 | SET domain and mariner transposase fusion gene | Epigenetic regulator | 61 | 0.617 | 0.692 | 0.001002 | 0.01 | | 1 | 1 | 0 | 2018 | 2018 |

| | | | | | | | | | | | | | | | | | |
|---|---|---|---|---|---|---|---|---|---|---|---|---|---|---|---|---|---|
| Rheumatoid Arthritis | C0003873 | STRA6 | 64220 | Q9BX79 | signaling receptor and transporter of retinol STRA6 | | 100 | 0.597 | 0.731 | 1.51E-10 | 0.01 | | 1 | 1 | 0 | 2013 | 2013 |
| Rheumatoid Arthritis | C0003873 | ROBO3 | 64221 | Q96MS0 | roundabout guidance receptor 3 | Receptor | 154 | 0.52 | 0.808 | 2E-15 | 0.01 | | 1 | 1 | 0 | 2010 | 2010 |
| Rheumatoid Arthritis | C0003873 | SFRP2 | 6423 | Q96HF1 | secreted frizzled related protein 2 | | 134 | 0.544 | 0.731 | 0.001669 | 0.01 | | 1 | 1 | 0 | 2003 | 2003 |
| Rheumatoid Arthritis | C0003873 | SFRP4 | 6424 | Q6FHJ7 | secreted frizzled related protein 4 | | 155 | 0.524 | 0.654 | 9.26E-05 | 0.01 | | 1 | 1 | 0 | 2014 | 2014 |
| Rheumatoid Arthritis | C0003873 | SFRP5 | 6425 | Q5T4F7 | secreted frizzled related protein 5 | | 106 | 0.559 | 0.692 | 5.18E-06 | 0.01 | | 1 | 1 | 0 | 2014 | 2014 |
| Rheumatoid Arthritis | C0003873 | MMP25 | 64386 | Q9NPA2 | matrix metallopeptidase 25 | Enzyme | 37 | 0.678 | 0.5 | 8.73E-14 | 0.01 | | 1 | 1 | 0 | 1999 | 1999 |
| Rheumatoid Ar | C0003873 | SGCA | 6442 | Q16586 | sarcoglycan alpha | Calcium-bindin | 111 | 0.592 | 0.654 | 0.0027 | 0.01 | | 0 | 1 | 0 | 2019 | 2019 |

| | | | | | | | | | | | | | | | | | |
|---|---|---|---|---|---|---|---|---|---|---|---|---|---|---|---|---|---|
| thritis | | | | | | g protein | | | | 12 | | | | | | | |
| Rheumatoid Arthritis | C0003873 | DCLRE1C | 64421 | Q96SD1 | DNA cross-link repair 1C | | 150 | 0.544 | 0.808 | 2.49E-07 | 0.01 | | 1 | 1 | 0 | 2011 | 2011 |
| Rheumatoid Arthritis | C0003873 | CIAO3 | 64428 | Q9H6Q4 | cytosolic iron-sulfur assembly component 3 | Enzyme | 30 | 0.7 | 0.462 | 1.89E-09 | 0.01 | | 1 | 1 | 0 | 2019 | 2019 |
| Rheumatoid Arthritis | C0003873 | SGCG | 6445 | Q13326 | sarcoglycan gamma | Cellular structure | 93 | 0.621 | 0.615 | 4.82E-05 | 0.01 | | 1 | 1 | 0 | 2017 | 2017 |
| Rheumatoid Arthritis | C0003873 | SH3BP2 | 6452 | P78314 | SH3 domain binding protein 2 | | 65 | 0.623 | 0.654 | 1.16E-11 | 0.01 | | 1 | 1 | 0 | 2015 | 2015 |
| Rheumatoid Arthritis | C0003873 | ITSN1 | 6453 | Q15811 | intersectin 1 | Enzyme modulator | 51 | 0.656 | 0.577 | 1 | 0.01 | | 1 | 1 | 0 | 2019 | 2019 |
| Rheumatoid Arthritis | C0003873 | SHH | 6469 | Q15465 | sonic hedgehog signaling molecule | | 303 | 0.461 | 0.846 | 0.98258 | 0.01 | | 1 | 1 | 0 | 2019 | 2019 |

| | | | | | | | | | | | | | | | | | |
|---|---|---|---|---|---|---|---|---|---|---|---|---|---|---|---|---|---|
| Rheumatoid Arthritis | C0003873 | ENGASE | 64772 | Q8NFI3 | endo-beta-N-acetylglucosaminidase | Enzyme | 4 | 0.861 | 0.231 | 4.41E-25 | 0.01 | | 1 | 1 | 0 | 2003 | 2003 |
| Rheumatoid Arthritis | C0003873 | P2RY12 | 64805 | Q9H244 | purinergic receptor P2Y12 | G-protein coupled receptor | 153 | 0.533 | 0.731 | 0.001686 | 0.01 | | 1 | 1 | 0 | 2019 | 2019 |
| Rheumatoid Arthritis | C0003873 | IL25 | 64806 | Q9H293 | interleukin 25 | | 139 | 0.529 | 0.846 | 0.000923 | 0.01 | | 1 | 1 | 0 | 2017 | 2017 |
| Rheumatoid Arthritis | C0003873 | CDCP1 | 64866 | Q9H5V8 | CUB domain containing protein 1 | | 81 | 0.604 | 0.654 | 0.0001 | 0.01 | | 1 | 1 | 0 | 2017 | 2017 |
| Rheumatoid Arthritis | C0003873 | LINC00273 | 649159 | | long intergenic non-protein coding RNA 273 | | 82 | 0.585 | 0.846 | | 0.01 | | 1 | 1 | 0 | 2017 | 2017 |
| Rheumatoid Arthritis | C0003873 | SLAMF1 | 6504 | Q13291 | signaling lymphocytic activation molecule family member 1 | | 70 | 0.612 | 0.615 | 0.007037 | 0.01 | | 1 | 1 | 0 | 1999 | 1999 |
| Rheumatoi | C0003 | SLC2A1 | 6513 | P11166 | solute carrier family 2 | Transporter | 687 | 0.3 | 0.8 | 0.99 | 0.01 | | 1 | 1 | 0 | 2019 | 2019 |

| | | | | | | | | | | | | | | | | | |
|---|---|---|---|---|---|---|---|---|---|---|---|---|---|---|---|---|---|
| d Arthritis | 873 | | | | member 1 | | | 88 | 08 | 41 | | | | | | | |
| Rheumatoid Arthritis | C0003873 | SLC3A2 | 6520 | P08195 | solute carrier family 3 member 2 | Transporter | 108 | 0.565 | 0.731 | 4.22E-08 | 0.01 | | 1 | 1 | 0 | 2018 | 2018 |
| Rheumatoid Arthritis | C0003873 | SLC5A5 | 6528 | Q92911 | solute carrier family 5 member 5 | Transporter | 195 | 0.499 | 0.769 | 0.0029449 | 0.01 | | 1 | 1 | 0 | 2005 | 2005 |
| Rheumatoid Arthritis | C0003873 | UCA1 | 652995 | | urothelial cancer associated 1 | | 152 | 0.512 | 0.808 | | 0.01 | | 1 | 1 | 0 | 2018 | 2018 |
| Rheumatoid Arthritis | C0003873 | BMP5 | 653 | P22003 | bone morphogenetic protein 5 | Signaling | 43 | 0.653 | 0.577 | 0.0009901 | 0.01 | | 1 | 1 | 0 | 2006 | 2006 |
| Rheumatoid Arthritis | C0003873 | SPAG11A | 653423 | Q08648;Q6PDA7 | sperm associated antigen 11A | | 44 | 0.65 | 0.731 | 6.96E-05 | 0.01 | | 1 | 1 | 0 | 2009 | 2009 |
| Rheumatoid Arthritis | C0003873 | SLC7A4 | 6545 | O43246 | solute carrier family 7 member 4 | Transporter | 31 | 0.666 | 0.423 | 2.83E-07 | 0.01 | | 1 | 1 | 0 | 1997 | 1997 |

| | | | | | | | | | | | | | | | | | |
|---|---|---|---|---|---|---|---|---|---|---|---|---|---|---|---|---|---|
| Rheumatoid Arthritis | C0003873 | SLC8A3 | 6547 | P57103 | solute carrier family 8 member A3 | Transporter | 51 | 0.666 | 0.346 | 0.00734 | 0.01 | | 1 | 1 | 0 | 2016 | 2016 |
| Rheumatoid Arthritis | C0003873 | SLC22A2 | 6582 | O15244 | solute carrier family 22 member 2 | Transporter | 98 | 0.572 | 0.769 | 2.02E-12 | 0.01 | | 1 | 1 | 0 | 2019 | 2019 |
| Rheumatoid Arthritis | C0003873 | SLIT3 | 6586 | O75094 | slit guidance ligand 3 | | 62 | 0.623 | 0.615 | 0.75233 | 0.01 | | 1 | 1 | 0 | 2010 | 2010 |
| Rheumatoid Arthritis | C0003873 | SNAI2 | 6591 | O43623 | snail family transcriptional repressor 2 | | 189 | 0.505 | 0.692 | 0.79531 | 0.01 | | 1 | 1 | 0 | 2010 | 2010 |
| Rheumatoid Arthritis | C0003873 | BMX | 660 | P51813 | BMX non-receptor tyrosine kinase | Kinase | 38 | 0.663 | 0.385 | 0.00673 | 0.01 | | 1 | 1 | 0 | 2008 | 2008 |
| Rheumatoid Arthritis | C0003873 | CHID1 | 66005 | Q9BWS9 | chitinase domain containing 1 | | 5 | 0.861 | 0.192 | 1.38E-05 | 0.01 | | 1 | 1 | 0 | 2014 | 2014 |
| Rheumatoid Ar | C0003873 | SMO | 6608 | Q99835 | smoothened, frizzled class receptor | G-protein coupl | 215 | 0.489 | 0.731 | 4.28E- | 0.01 | | 1 | 1 | 0 | 2017 | 2017 |

| | | | | | | | | | | | | | | | | | |
|---|---|---|---|---|---|---|---|---|---|---|---|---|---|---|---|---|---|
| thritis | | | | | | ed receptor | | | | 07 | | | | | | | |
| Rheumatoid Arthritis | C0003873 | SMPD1 | 6609 | P17405 | sphingomyelin phosphodiesterase 1 | Enzyme | 247 | 0.499 | 0.885 | 3.89E-10 | 0.01 | | 1 | 1 | 0 | 2017 | 2017 |
| Rheumatoid Arthritis | C0003873 | SIGLEC1 | 6614 | Q9BZZ2 | sialic acid binding Ig like lectin 1 | | 75 | 0.597 | 0.654 | 5.33E-46 | 0.01 | | 1 | 1 | 0 | 2013 | 2013 |
| Rheumatoid Arthritis | C0003873 | SNAI1 | 6615 | O95863 | snail family transcriptional repressor 1 | | 243 | 0.474 | 0.808 | 0.21754 | 0.01 | | 1 | 1 | 0 | 2019 | 2019 |
| Rheumatoid Arthritis | C0003873 | MIR539 | 664612 | | microRNA 539 | | 62 | 0.61 | 0.654 | | 0.01 | | 1 | 1 | 0 | 2018 | 2018 |
| Rheumatoid Arthritis | C0003873 | SOX4 | 6659 | Q06945 | SRY-box transcription factor 4 | Transcription factor | 246 | 0.489 | 0.808 | 0.92656 | 0.01 | | 1 | 1 | 0 | 2018 | 2018 |
| Rheumatoid Arthritis | C0003873 | DST | 667 | Q03001 | dystonin | Cellular structure | 147 | 0.552 | 0.846 | 1 | 0.01 | | 1 | 1 | 0 | 2009 | 2009 |
| Rheu | C00 | SPR | 66 | P35270 | sepiapterin reductase | | 90 | 0. | 0. | 0.0 | 0.01 | | 1 | 1 | 0 | 2020 | 20 |

| | | | | | | | | | | | | | | | | | |
|---|---|---|---|---|---|---|---|---|---|---|---|---|---|---|---|---|---|
| matoid Arthritis | 03873 | | 97 | | | | | 631 | 654 | 434 | | | | | | | 20 |
| Rheumatoid Arthritis | C0003873 | BPI | 671 | P17213 | bactericidal permeability increasing protein | | 83 | 0.61 | 0.769 | 1.65E-14 | 0.01 | | 1 | 1 | 0 | 2003 | 2003 |
| Rheumatoid Arthritis | C0003873 | SRC | 6714 | P12931 | SRC proto-oncogene, non-receptor tyrosine kinase | Kinase | 265 | 0.467 | 0.885 | 0.99691 | 0.01 | | 1 | 1 | 0 | 2016 | 2016 |
| Rheumatoid Arthritis | C0003873 | AKR1D1 | 6718 | P51857 | aldo-keto reductase family 1 member D1 | Enzyme | 40 | 0.711 | 0.385 | 1.72E-07 | 0.01 | | 1 | 1 | 0 | 2017 | 2017 |
| Rheumatoid Arthritis | C0003873 | SRF | 6722 | P11831 | serum response factor | Transcription factor | 147 | 0.534 | 0.808 | 0.97545 | 0.01 | | 1 | 1 | 0 | 1999 | 1999 |
| Rheumatoid Arthritis | C0003873 | SRP54 | 6729 | P61011 | signal recognition particle 54 | Nucleic acid binding | 96 | 0.612 | 0.769 | 0.99895 | 0.01 | | 1 | 1 | 0 | 2018 | 2018 |
| Rheumatoid Arthritis | C0003873 | SRY | 6736 | Q05066 | sex determining region Y | Transcription factor | 315 | 0.456 | 0.808 | | 0.01 | | 1 | 1 | 0 | 2008 | 2008 |

| | | | | | | | | | | | | | | | | | |
|---|---|---|---|---|---|---|---|---|---|---|---|---|---|---|---|---|---|
| Rheumatoid Arthritis | C0003873 | RO60 | 6738 | P10155 | Ro60, Y RNA binding protein | Nucleic acid binding | 61 | 0.61 | 0.692 | 0.58278 | 0.01 | | 1 | 1 | 0 | 2018 | 2018 |
| Rheumatoid Arthritis | C0003873 | SSB | 6741 | P05455 | small RNA binding exonuclease protection factor La | Nucleic acid binding | 60 | 0.612 | 0.692 | 0.089615 | 0.01 | | 1 | 1 | 0 | 2013 | 2013 |
| Rheumatoid Arthritis | C0003873 | ST2 | 6761 | | suppression of tumorigenicity 2 | | 96 | 0.576 | 0.692 | | 0.01 | | 1 | 1 | 0 | 2018 | 2018 |
| Rheumatoid Arthritis | C0003873 | STATH | 6779 | P02808 | statherin | | 57 | 0.608 | 0.769 | 7.98E-05 | 0.01 | | 1 | 1 | 0 | 2002 | 2002 |
| Rheumatoid Arthritis | C0003873 | STIM1 | 6786 | Q13586 | stromal interaction molecule 1 | | 247 | 0.501 | 0.808 | 0.77534 | 0.01 | | 1 | 1 | 0 | 2019 | 2019 |
| Rheumatoid Arthritis | C0003873 | AURKA | 6790 | O14965 | aurora kinase A | Kinase | 245 | 0.475 | 0.731 | 0.8676 | 0.01 | | 1 | 1 | 0 | 2006 | 2006 |
| Rheumatoid Ar | C0003873 | TACR2 | 6865 | P21452 | tachykinin receptor 2 | G-protein coupl | 33 | 0.7 | 0.5 | 8.883E- | 0.01 | | 1 | 1 | 0 | 2005 | 2005 |

| | | | | | | | | | | | | | | | | | |
|---|---|---|---|---|---|---|---|---|---|---|---|---|---|---|---|---|---|
| thritis | | | | | | ed receptor | | | | 05 | | | | | | | |
| Rheumatoid Arthritis | C0003873 | TACR3 | 6870 | P29371 | tachykinin receptor 3 | G-protein coupled receptor | 147 | 0.553 | 0.769 | 1.81E-17 | 0.01 | | 1 | 1 | 0 | 2005 | 2005 |
| Rheumatoid Arthritis | C0003873 | TAGLN | 6876 | Q01995 | transgelin | Cellular structure | 131 | 0.54 | 0.692 | 0.08527 | 0.01 | | 1 | 1 | 0 | 2019 | 2019 |
| Rheumatoid Arthritis | C0003873 | TAL1 | 6886 | P17542 | TAL bHLH transcription factor 1, erythroid differentiation factor | Transcription factor | 202 | 0.505 | 0.731 | 0.56559 | 0.01 | | 1 | 1 | 0 | 2004 | 2004 |
| Rheumatoid Arthritis | C0003873 | TAT | 6898 | P17735 | tyrosine aminotransferase | | 230 | 0.492 | 0.846 | 0.0069 | 0.01 | | 1 | 1 | 0 | 2006 | 2006 |
| Rheumatoid Arthritis | C0003873 | TBX1 | 6899 | O43435 | T-box transcription factor 1 | Transcription factor | 417 | 0.433 | 0.808 | 0.83661 | 0.01 | | 1 | 1 | 0 | 2018 | 2018 |
| Rheumatoid Ar | C0003873 | TCEA1 | 6917 | P23193 | transcription elongation factor A1 | Nucleic acid bin | 34 | 0.695 | 0.615 | 0.89541 | 0.01 | | 1 | 1 | 0 | 2017 | 2017 |

| | | | | | | | | | | | | | | | | | |
|---|---|---|---|---|---|---|---|---|---|---|---|---|---|---|---|---|---|
| thritis | | | | | | ding | | | | | | | | | | | |
| Rheumatoid Arthritis | C0003873 | PPAN-P2RY11 | 6923112 | Q9NQ55 | PPAN-P2RY11 readthrough | | 17 | 0.736 | 0.577 | 1.72E-12 | 0.01 | | 1 | 1 | 0 | 2019 | 2019 |
| Rheumatoid Arthritis | C0003873 | TBX3 | 6926 | O15119 | T-box transcription factor 3 | Transcription factor | 179 | 0.525 | 0.769 | 0.989907 | 0.01 | | 1 | 1 | 0 | 2019 | 2019 |
| Rheumatoid Arthritis | C0003873 | TCF3 | 6929 | P15923 | transcription factor 3 | Transcription factor | 244 | 0.476 | 0.846 | 0.025824 | 0.01 | | 1 | 1 | 0 | 1992 | 1992 |
| Rheumatoid Arthritis | C0003873 | MIR421 | 693122 | | microRNA 421 | | 73 | 0.592 | 0.692 | | 0.01 | | 1 | 1 | 0 | 2019 | 2019 |
| Rheumatoid Arthritis | C0003873 | MIR551B | 693136 | | microRNA 551b | | 31 | 0.682 | 0.538 | | 0.01 | | 1 | 1 | 0 | 2018 | 2018 |
| Rheumatoid Arthritis | C0003873 | MIR573 | 693158 | | microRNA 573 | | 17 | 0.743 | 0.385 | | 0.01 | | 1 | 1 | 0 | 2016 | 2016 |
| Rheumatoi | C0003 | MIR590 | 6931 | | microRNA 590 | | 99 | 0.5 | 0.6 | | 0.01 | | 1 | 1 | 0 | 2019 | 2019 |

| | | | | | | | | | | | | | | | | |
|---|---|---|---|---|---|---|---|---|---|---|---|---|---|---|---|---|
| d Arthritis | 873 | | 75 | | | | 53 | 92 | | | | | | | | |
| Rheumatoid Arthritis | C0003873 | MIR613 | 693198 | | microRNA 613 | 60 | 0.608 | 0.615 | | 0.01 | | 1 | 1 | 0 | 2019 | 2019 |
| Rheumatoid Arthritis | C0003873 | MIR633 | 693218 | | microRNA 633 | 12 | 0.769 | 0.346 | | 0.01 | | 1 | 1 | 0 | 2018 | 2018 |
| Rheumatoid Arthritis | C0003873 | MIR642A | 693227 | | microRNA 642a | 12 | 0.769 | 0.308 | | 0.01 | | 1 | 1 | 0 | 2019 | 2019 |
| Rheumatoid Arthritis | C0003873 | TCF7L2 | 6934 | Q9NQB0 | transcription factor 7 like 2 | 257 | 0.485 | 0.846 | 0.99537 | 0.01 | | 1 | 1 | 1 | 2012 | 2012 |
| Rheumatoid Arthritis | C0003873 | TCN2 | 6948 | P20062 | transcobalamin 2 | 104 | 0.578 | 0.615 | 1.05E-06 | 0.01 | | 1 | 1 | 0 | 2015 | 2015 |
| Rheumatoid Arthritis | C0003873 | TRAV6 | 6956 | A0A075B6T7 | T cell receptor alpha variable 6 | 1 | 1 | 0.115 | | 0.01 | | 1 | 1 | 0 | 1998 | 1998 |

| | | | | | | | | | | | | | | | | | |
|---|---|---|---|---|---|---|---|---|---|---|---|---|---|---|---|---|---|
| Rheumatoid Arthritis | C0003873 | BTN1A1 | 696 | Q13410 | butyrophilin subfamily 1 member A1 | Enzyme modulator | 9 | 0.839 | 0.346 | 1.95E-08 | 0.01 | | 1 | 1 | 0 | 2011 | 2011 |
| Rheumatoid Arthritis | C0003873 | TRD | 6964 | | T cell receptor delta locus | | 45 | 0.65 | 0.692 | | 0.01 | | 1 | 1 | 0 | 1994 | 1994 |
| Rheumatoid Arthritis | C0003873 | TRG | 6965 | | T cell receptor gamma locus | | 54 | 0.631 | 0.538 | | 0.01 | | 0 | 1 | 0 | 1995 | 1995 |
| Rheumatoid Arthritis | C0003873 | TRGC1 | 6966 | P0CF51 | T cell receptor gamma constant 1 | | 35 | 0.663 | 0.385 | | 0.01 | | 0 | 1 | 0 | 1995 | 1995 |
| Rheumatoid Arthritis | C0003873 | PPP1R11 | 6992 | O60927 | protein phosphatase 1 regulatory inhibitor subunit 11 | Enzyme modulator | 66 | 0.604 | 0.731 | 0.35781 | 0.01 | | 1 | 1 | 1 | 2017 | 2017 |
| Rheumatoid Arthritis | C0003873 | TDGF1 | 6997 | P13385 | teratocarcinoma-derived growth factor 1 | Calcium-binding protein | 95 | 0.584 | 0.731 | 9.16E-11 | 0.01 | | 1 | 1 | 0 | 1996 | 1996 |
| Rheumatoi | C0003 | TDGF1P3 | 6998 | P51864 | teratocarcinoma-derived growth | Calcium- | 52 | 0.6 | 0.5 | | 0.01 | | 1 | 1 | 0 | 1996 | 1996 |

| | | | | | | | | | | | | | | | | | |
|---|---|---|---|---|---|---|---|---|---|---|---|---|---|---|---|---|---|
| d Arthritis | 873 | | | | factor 1 pseudogene 3 | binding protein | | 31 | | | | | | | | | |
| Rheumatoid Arthritis | C0003873 | PRDX2 | 7001 | P32119 | peroxiredoxin 2 | Enzyme | 243 | 0.474 | 0.885 | 0.0865133 | 0.01 | | 1 | 1 | 0 | 2012 | 2012 |
| Rheumatoid Arthritis | C0003873 | TEF | 7008 | Q10587 | TEF transcription factor, PAR bZIP family member | Nucleic acid binding | 34 | 0.686 | 0.538 | 0.632291 | 0.01 | | 1 | 1 | 0 | 2013 | 2013 |
| Rheumatoid Arthritis | C0003873 | TEP1 | 7011 | Q99973 | telomerase associated protein 1 | | 54 | 0.621 | 0.654 | 4.38E-49 | 0.01 | | 0 | 1 | 0 | 2000 | 2000 |
| Rheumatoid Arthritis | C0003873 | TERF2 | 7014 | Q15554 | telomeric repeat binding factor 2 | | 125 | 0.541 | 0.731 | 0.992337 | 0.01 | | 0 | 1 | 0 | 2000 | 2000 |
| Rheumatoid Arthritis | C0003873 | TERT | 7015 | O14746 | telomerase reverse transcriptase | Enzyme | 703 | 0.374 | 0.846 | 0.990446 | 0.01 | | 1 | 1 | 1 | 2007 | 2007 |
| Rheumatoid Arthritis | C0003873 | TF | 7018 | P02787 | transferrin | Enzyme | 168 | 0.527 | 0.846 | 4.1E-08 | 0.01 | | 1 | 1 | 0 | 2002 | 2002 |

| | | | | | | | | | | | | | | | | | |
|---|---|---|---|---|---|---|---|---|---|---|---|---|---|---|---|---|---|
| Rheumatoid Arthritis | C0003873 | NR2F1 | 7025 | P10589 | nuclear receptor subfamily 2 group F member 1 | Nuclear receptor | 53 | 0.656 | 0.654 | 0.99482 | 0.01 | | 1 | 1 | 0 | 2000 | 2000 |
| Rheumatoid Arthritis | C0003873 | TFDP1 | 7027 | Q14186 | transcription factor Dp-1 | Nucleic acid binding | 56 | 0.623 | 0.692 | 0.97174 | 0.01 | | 1 | 1 | 0 | 2019 | 2019 |
| Rheumatoid Arthritis | C0003873 | TFF1 | 7031 | P04155 | trefoil factor 1 | Signaling | 147 | 0.529 | 0.769 | 0.000889 | 0.01 | | 1 | 1 | 0 | 2019 | 2019 |
| Rheumatoid Arthritis | C0003873 | TFF2 | 7032 | Q03403 | trefoil factor 2 | Signaling | 72 | 0.61 | 0.538 | 2.22E-07 | 0.01 | | 1 | 1 | 0 | 2019 | 2019 |
| Rheumatoid Arthritis | C0003873 | TGFB2 | 7042 | P61812 | transforming growth factor beta 2 | Signaling | 389 | 0.433 | 0.885 | 0.99905 | 0.01 | | 1 | 1 | 0 | 2007 | 2007 |
| Rheumatoid Arthritis | C0003873 | TGFB3 | 7043 | P10600 | transforming growth factor beta 3 | Signaling | 230 | 0.498 | 0.808 | 0.98727 | 0.01 | | 1 | 1 | 0 | 2010 | 2010 |
| Rheumatoid Ar | C0003873 | TGFBI | 7045 | Q15582 | transforming growth factor beta induced | Signaling | 225 | 0.484 | 0.769 | 5.97E- | 0.01 | | 1 | 1 | 0 | 2006 | 2006 |

| | | | | | | | | | | | | | | | | | |
|---|---|---|---|---|---|---|---|---|---|---|---|---|---|---|---|---|---|
| thritis | | | | | | | | | | 08 | | | | | | | |
| Rheumatoid Arthritis | C0003873 | TGFBR1 | 7046 | P36897 | transforming growth factor beta receptor 1 | Kinase | 347 | 0.451 | 0.885 | 0.853798 | 0.01 | | 1 | 1 | 0 | 2007 | 2007 |
| Rheumatoid Arthritis | C0003873 | TGIF1 | 7050 | Q15583 | TGFB induced factor homeobox 1 | Enzyme | 100 | 0.588 | 0.769 | 0.010454 | 0.01 | | 1 | 1 | 0 | 2005 | 2005 |
| Rheumatoid Arthritis | C0003873 | THAS | 7055 | | thoracoabdominal syndrome | | 120 | 0.56 | 0.808 | | 0.01 | | 1 | 1 | 0 | 2018 | 2018 |
| Rheumatoid Arthritis | C0003873 | NKX2-1 | 7080 | P43699 | NK2 homeobox 1 | Transcription factor | 319 | 0.457 | 0.808 | 0.35874 | 0.01 | | 1 | 1 | 0 | 2018 | 2018 |
| Rheumatoid Arthritis | C0003873 | TNNC1 | 7134 | P63316 | troponin C1, slow skeletal and cardiac type | Calcium-binding protein | 54 | 0.647 | 0.462 | 0.67833 | 0.01 | | 1 | 1 | 0 | 2018 | 2018 |
| Rheumatoid Arthritis | C0003873 | TNNI3 | 7137 | P19429 | troponin I3, cardiac type | Cellular structure | 159 | 0.52 | 0.769 | 0.096284 | 0.01 | | 1 | 1 | 0 | 2018 | 2018 |

| | | | | | | | | | | | | | | | | | |
|---|---|---|---|---|---|---|---|---|---|---|---|---|---|---|---|---|---|
| Rheumatoid Arthritis | C0003873 | TP73 | 7161 | O15350 | tumor protein p73 | Transcription factor | 300 | 0.449 | 0.808 | 0.99696 | 0.01 | | 1 | 1 | 0 | 2017 | 2017 |
| Rheumatoid Arthritis | C0003873 | TPSAB1 | 7177 | P20231;Q15661 | tryptase alpha/beta 1 | Enzyme | 23 | 0.722 | 0.5 | 1.27E-13 | 0.01 | | 1 | 1 | 0 | 2007 | 2007 |
| Rheumatoid Arthritis | C0003873 | TPT1 | 7178 | P13693 | tumor protein, translationally-controlled 1 | Cellular structure | 143 | 0.528 | 0.731 | 0.80548 | 0.01 | | 1 | 1 | 0 | 2008 | 2008 |
| Rheumatoid Arthritis | C0003873 | NR2C2 | 7182 | P49116 | nuclear receptor subfamily 2 group C member 2 | Nuclear receptor | 131 | 0.534 | 0.808 | 0.99892 | 0.01 | | 1 | 1 | 0 | 2020 | 2020 |
| Rheumatoid Arthritis | C0003873 | HSP90B1 | 7184 | P14625 | heat shock protein 90 beta family member 1 | Chaperone | 155 | 0.522 | 0.808 | 0.99972 | 0.01 | | 1 | 1 | 0 | 2009 | 2009 |
| Rheumatoid Arthritis | C0003873 | TRAF3 | 7187 | Q13114 | TNF receptor associated factor 3 | Signaling | 129 | 0.541 | 0.769 | 0.99869 | 0.01 | | 1 | 1 | 0 | 2018 | 2018 |
| Rheumatoid Ar | C0003873 | TRAF5 | 7188 | O00463 | TNF receptor associated factor 5 | Signaling | 36 | 0.659 | 0.654 | 1.67E- | 0.01 | | 1 | 1 | 0 | 2007 | 2007 |

| | | | | | | | | | | | | | | | | |
|---|---|---|---|---|---|---|---|---|---|---|---|---|---|---|---|---|
| thritis | | | | | | | | | | 12 | | | | | | |
| Rheumatoid Arthritis | C0003873 | C3AR1 | 719 | Q16581 | complement C3a receptor 1 | G-protein coupled receptor | 73 | 0.601 | 0.731 | 3.31E-09 | 0.01 | | 1 | 1 | 0 | 2017 | 2017 |
| Rheumatoid Arthritis | C0003873 | HSP90B2P | 7190 | Q58FF3 | heat shock protein 90 beta family member 2, pseudogene | Chaperone | 110 | 0.558 | 0.846 | | 0.01 | | 1 | 1 | 0 | 2011 | 2011 |
| Rheumatoid Arthritis | C0003873 | CCT3 | 7203 | P49368 | chaperonin containing TCP1 subunit 3 | Chaperone | 43 | 0.65 | 0.577 | 0.99998 | 0.01 | | 1 | 1 | 0 | 2015 | 2015 |
| Rheumatoid Arthritis | C0003873 | TRPC1 | 7220 | P48995 | transient receptor potential cation channel subfamily C member 1 | Ion channel | 97 | 0.566 | 0.769 | 0.012904 | 0.01 | | 1 | 1 | 0 | 2008 | 2008 |
| Rheumatoid Arthritis | C0003873 | TRPC5 | 7224 | Q9UL62 | transient receptor potential cation channel subfamily C member 5 | Ion channel | 58 | 0.653 | 0.615 | 0.99973 | 0.01 | | 1 | 1 | 0 | 2008 | 2008 |
| Rheumatoid Ar | C0003873 | TRPM2 | 7226 | O94759 | transient receptor potential cation channel subfamily M | Ion channel | 133 | 0.546 | 0.731 | 8.96E-44 | 0.01 | | 1 | 1 | 0 | 2019 | 2019 |

| | | | | | | | | | | | | | | | | | |
|---|---|---|---|---|---|---|---|---|---|---|---|---|---|---|---|---|---|
| thritis | | | | | member 2 | | | | | | | | | | | | |
| Rheumatoid Arthritis | C0003873 | MIR650 | 7237778 | | microRNA 650 | | 35 | 0.67 | 0.577 | | 0.01 | | 1 | 1 | 0 | 2017 | 2017 |
| Rheumatoid Arthritis | C0003873 | MIR663A | 7240333 | | microRNA 663a | | 81 | 0.576 | 0.769 | | 0.01 | | 1 | 1 | 0 | 2015 | 2015 |
| Rheumatoid Arthritis | C0003873 | TTN | 7273 | Q8WZ42 | titin | Kinase | 366 | 0.47 | 0.885 | 2.56E-96 | 0.01 | | 1 | 1 | 0 | 2014 | 2014 |
| Rheumatoid Arthritis | C0003873 | SFTPA2 | 729238 | Q8IWL1 | surfactant protein A2 | | 111 | 0.563 | 0.731 | 0.009251 | 0.01 | | 1 | 1 | 0 | 2006 | 2006 |
| Rheumatoid Arthritis | C0003873 | TYRO3 | 7301 | Q06418 | TYRO3 protein tyrosine kinase | Kinase | 109 | 0.559 | 0.769 | 8.33E-10 | 0.01 | | 1 | 1 | 0 | 2018 | 2018 |
| Rheumatoid Arthritis | C0003873 | TYRP1 | 7306 | P17643 | tyrosinase related protein 1 | Enzyme | 200 | 0.507 | 0.846 | 6.56E-25 | 0.01 | | 1 | 1 | 0 | 2019 | 2019 |
| Rheumatoi | C0003 | UBE2N | 7334 | P61088 | ubiquitin conjugating enzyme E2 N | | 80 | 0.5 | 0.6 | 0.881 | 0.01 | | 1 | 1 | 0 | 2018 | 2018 |

| | | | | | | | | | | | | | | | | | |
|---|---|---|---|---|---|---|---|---|---|---|---|---|---|---|---|---|---|
| d Arthritis | 873 | | | | | | | 93 | 54 | 56 | | | | | | | |
| Rheumatoid Arthritis | C0003873 | UBTF | 7343 | P17480 | upstream binding transcription factor | | 44 | 0.705 | 0.462 | 1 | 0.01 | | 1 | 1 | 0 | 1993 | 1993 |
| Rheumatoid Arthritis | C0003873 | UCN | 7349 | P55089 | urocortin | Signaling | 134 | 0.539 | 0.769 | 0.44958 | 0.01 | | 1 | 1 | 0 | 2001 | 2001 |
| Rheumatoid Arthritis | C0003873 | UCP2 | 7351 | P55851 | uncoupling protein 2 | Transporter | 235 | 0.493 | 0.808 | 1.55E-10 | 0.01 | | 1 | 1 | 0 | 2009 | 2009 |
| Rheumatoid Arthritis | C0003873 | UGCG | 7357 | Q16739 | UDP-glucose ceramide glucosyltransferase | Enzyme | 111 | 0.56 | 0.808 | 0.94147 | 0.01 | | 1 | 1 | 0 | 1999 | 1999 |
| Rheumatoid Arthritis | C0003873 | UNG | 7374 | P13051 | uracil DNA glycosylase | | 66 | 0.617 | 0.654 | 0.001725 | 0.01 | | 1 | 1 | 0 | 2012 | 2012 |
| Rheumatoid Arthritis | C0003873 | UTRN | 7402 | P46939 | utrophin | | 152 | 0.525 | 0.692 | 1E-19 | 0.01 | | 1 | 1 | 0 | 2020 | 2020 |

| | | | | | | | | | | | | | | | | | |
|---|---|---|---|---|---|---|---|---|---|---|---|---|---|---|---|---|---|
| Rheumatoid Arthritis | C0003873 | KDM6A | 7403 | O15550 | lysine demethylase 6A | Epigenetic regulator | 238 | 0.498 | 0.769 | 1 | 0.01 | | 1 | 1 | 0 | 2019 | 2019 |
| Rheumatoid Arthritis | C0003873 | VAV1 | 7409 | P15498 | vav guanine nucleotide exchange factor 1 | | 103 | 0.556 | 0.769 | 0.99993 | 0.01 | | 1 | 1 | 4 | 2017 | 2017 |
| Rheumatoid Arthritis | C0003873 | VDAC1 | 7416 | P21796 | voltage dependent anion channel 1 | | 116 | 0.556 | 0.654 | 0.97266 | 0.01 | | 1 | 1 | 0 | 2018 | 2018 |
| Rheumatoid Arthritis | C0003873 | VIPR1 | 7433 | P32241 | vasoactive intestinal peptide receptor 1 | G-protein coupled receptor | 102 | 0.573 | 0.654 | 7.44E-27 | 0.01 | | 1 | 1 | 0 | 2008 | 2008 |
| Rheumatoid Arthritis | C0003873 | TRPV1 | 7442 | Q8NER1 | transient receptor potential cation channel subfamily V member 1 | Ion channel | 404 | 0.446 | 0.885 | 1.01E-16 | 0.01 | | 1 | 1 | 0 | 2007 | 2007 |
| Rheumatoid Arthritis | C0003873 | WNT1 | 7471 | P04628 | Wnt family member 1 | Signaling | 216 | 0.495 | 0.769 | 0.3263 | 0.01 | | 1 | 1 | 0 | 2000 | 2000 |
| Rheuma | C0003 | XBP1 | 74 | P17861 | X-box binding protein 1 | | 234 | 0.4 | 0.8 | 0.03 | 0.01 | | 1 | 1 | 0 | 2014 | 20 |

| | | | | | | | | | | | | | | | | |
|---|---|---|---|---|---|---|---|---|---|---|---|---|---|---|---|---|
| toid Arthritis | 873 | | 94 | | | | 77 | 46 | 2014 | | | | | | | 14 |
| Rheumatoid Arthritis | C0003873 | XBP1P1 | 7495 | | X-box binding protein 1 pseudogene 1 | 42 | 0.653 | 0.692 | | 0.01 | | 1 | 1 | 0 | 2014 | 2014 |
| Rheumatoid Arthritis | C0003873 | XRCC4 | 7518 | Q13426 | X-ray repair cross complementing 4 | 192 | 0.528 | 0.769 | 8.28E-07 | 0.01 | | 1 | 1 | 0 | 2015 | 2015 |
| Rheumatoid Arthritis | C0003873 | CA2 | 760 | P00918 | carbonic anhydrase 2 | 210 | 0.506 | 0.808 | 1.69E-06 | 0.01 | | 1 | 1 | 0 | 2001 | 2001 |
| Rheumatoid Arthritis | C0003873 | CA3 | 761 | P07451 | carbonic anhydrase 3 | 37 | 0.682 | 0.5 | 0.001725 | 0.01 | | 1 | 1 | 0 | 2015 | 2015 |
| Rheumatoid Arthritis | C0003873 | MIR671 | 768213 | | microRNA 671 | 47 | 0.636 | 0.577 | | 0.01 | | 1 | 1 | 0 | 2019 | 2019 |
| Rheumatoid Arthritis | C0003873 | MIR766 | 768218 | | microRNA 766 | 46 | 0.633 | 0.577 | | 0.01 | | 1 | 1 | 0 | 2019 | 2019 |

| | | | | | | | | | | | | | | | | | |
|---|---|---|---|---|---|---|---|---|---|---|---|---|---|---|---|---|---|
| Rheumatoid Arthritis | C0003873 | CACNA1S | 779 | Q13698 | calcium voltage-gated channel subunit alpha1 S | Ion channel | 118 | 0.595 | 0.692 | 6.26E-12 | 0.01 | | 1 | 1 | 0 | 2009 | 2009 |
| Rheumatoid Arthritis | C0003873 | PSS | 780904 | | Potocki-Shaffer syndrome | | 66 | 0.608 | 0.654 | | 0.01 | | 1 | 1 | 0 | 1991 | 1991 |
| Rheumatoid Arthritis | C0003873 | SCG2 | 7857 | P13521 | secretogranin II | | 57 | 0.628 | 0.615 | 2.12E-05 | 0.01 | | 1 | 1 | 0 | 2000 | 2000 |
| Rheumatoid Arthritis | C0003873 | MANF | 7873 | P55145 | mesencephalic astrocyte derived neurotrophic factor | | 62 | 0.621 | 0.654 | 0.001391 | 0.01 | | 1 | 1 | 0 | 2018 | 2018 |
| Rheumatoid Arthritis | C0003873 | GGCT | 79017 | O75223 | gamma-glutamylcyclotransferase | | 108 | 0.552 | 0.808 | 0.000143 | 0.01 | | 1 | 1 | 0 | 2007 | 2007 |
| Rheumatoid Arthritis | C0003873 | AHNAK | 79026 | Q09666 | AHNAK nucleoprotein | | 60 | 0.623 | 0.615 | 0.89827 | 0.01 | | 1 | 1 | 0 | 2002 | 2002 |
| Rheumatoid Ar | C0003873 | ASPSCR1 | 79058 | Q9BZE9 | ASPSCR1 tether for SLC2A4, UBX domain containing | | 54 | 0.653 | 0.538 | 4.42E- | 0.01 | | 1 | 1 | 0 | 2019 | 2019 |

| | | | | | | | | | | | | | | | | | |
|---|---|---|---|---|---|---|---|---|---|---|---|---|---|---|---|---|---|
| thritis | | | | | | | | | | 12 | | | | | | | |
| Rheumatoid Arthritis | C0003873 | TSL | 7909 53 | | testis-expressed, seven-twelve, leukemia | | 18 | 0.743 | 0.346 | | 0.01 | | 1 | 1 | 0 | 2017 | 2017 |
| Rheumatoid Arthritis | C0003873 | DEK | 7913 | P35659 | DEK proto-oncogene | | 122 | 0.542 | 0.769 | 0.13065 | 0.01 | | 1 | 1 | 0 | 2004 | 2004 |
| Rheumatoid Arthritis | C0003873 | OPLL | 7933 | | ossification of posterior longitudinal ligament of spine | | 6 | 0.861 | 0.154 | | 0.01 | | 1 | 1 | 0 | 2017 | 2017 |
| Rheumatoid Arthritis | C0003873 | BHLHE41 | 79365 | Q9C0J9 | basic helix-loop-helix family member e41 | Transcription factor | 63 | 0.623 | 0.577 | 0.99212 | 0.01 | | 1 | 1 | 0 | 2015 | 2015 |
| Rheumatoid Arthritis | C0003873 | TFEB | 7942 | P19484 | transcription factor EB | | 150 | 0.527 | 0.731 | 0.89931 | 0.01 | | 1 | 1 | 0 | 2020 | 2020 |
| Rheumatoid Arthritis | C0003873 | NTT | 7956 | | Noncoding transcript in T cells | | 16 | 0.743 | 0.462 | | 0.01 | | 1 | 1 | 0 | 2018 | 2018 |
| Rheumatoi | C0003 | NKAP | 795 | Q8N5F7 | NFKB activating protein | | 15 | 0.76 | 0.2 | 0.997 | 0.01 | | 1 | 1 | 0 | 2013 | 2013 |

| | | | | | | | | | | | | | | | | | |
|---|---|---|---|---|---|---|---|---|---|---|---|---|---|---|---|---|---|
| d Arthritis | 873 | | 76 | | | | | | 69 | 35 | | | | | | | |
| Rheumatoid Arthritis | C0003873 | SLC52A2 | 79581 | Q9HAB3 | solute carrier family 52 member 2 | Transporter | 261 | 0.483 | 0.808 | 0.002478 | 0.01 | | 1 | 1 | 0 | 2012 | 2012 |
| Rheumatoid Arthritis | C0003873 | SPAG16 | 79582 | Q8N0X2 | sperm associated antigen 16 | Cellular structure | 17 | 0.76 | 0.385 | 1.4E-34 | 0.01 | | 1 | 1 | 1 | 2014 | 2014 |
| Rheumatoid Arthritis | C0003873 | TNFAIP8L2 | 79626 | Q6P589 | TNF alpha induced protein 8 like 2 | | 90 | 0.575 | 0.615 | 0.038364 | 0.01 | | 1 | 1 | 0 | 2017 | 2017 |
| Rheumatoid Arthritis | C0003873 | NLRX1 | 79671 | Q86UT6 | NLR family member X1 | Enzyme | 51 | 0.641 | 0.654 | 7.73E-31 | 0.01 | | 0 | 1 | 0 | 2017 | 2017 |
| Rheumatoid Arthritis | C0003873 | LRRC31 | 79782 | Q6UY01 | leucine rich repeat containing 31 | | 7 | 0.839 | 0.269 | 2E-06 | 0.01 | | 1 | 1 | 0 | 2017 | 2017 |
| Rheumatoid Arthritis | C0003873 | SHCBP1 | 79801 | Q8NEM2 | SHC binding and spindle associated 1 | | 72 | 0.606 | 0.654 | 9.38E-08 | 0.01 | | 1 | 1 | 0 | 2002 | 2002 |

| | | | | | | | | | | | | | | | | |
|---|---|---|---|---|---|---|---|---|---|---|---|---|---|---|---|---|
| Rheumatoid Arthritis | C0003873 | CAMKMT | 79823 | Q7Z624 | calmodulin-lysine N-methyltransferase | | 185 | 0.51 | 0.808 | 1.38E-15 | 0.01 | | 1 | 1 | 0 | 2020 | 2020 |
| Rheumatoid Arthritis | C0003873 | CCDC134 | 79879 | Q9H6E4 | coiled-coil domain containing 134 | | 19 | 0.769 | 0.308 | 6E-07 | 0.01 | | 1 | 1 | 0 | 2017 | 2017 |
| Rheumatoid Arthritis | C0003873 | TNIP3 | 79931 | Q96KP6 | TNFAIP3 interacting protein 3 | | 12 | 0.76 | 0.308 | 0.01544 | 0.01 | | 1 | 1 | 0 | 2012 | 2012 |
| Rheumatoid Arthritis | C0003873 | NAA25 | 80018 | Q14CX7 | N-alpha-acetyltransferase 25, NatB auxiliary subunit | Enzyme | 81 | 0.603 | 0.692 | 1 | 0.01 | | 1 | 1 | 0 | 2009 | 2009 |
| Rheumatoid Arthritis | C0003873 | TRPM3 | 80036 | Q9HCF6 | transient receptor potential cation channel subfamily M member 3 | Ion channel | 51 | 0.663 | 0.769 | 5.69E-07 | 0.01 | | 1 | 1 | 0 | 2010 | 2010 |
| Rheumatoid Arthritis | C0003873 | CALM1 | 801 | P0DP23;P0DP24;P0DP25 | calmodulin 1 | Calcium-binding protein | 253 | 0.472 | 0.808 | 0.94473 | 0.01 | | 1 | 1 | 0 | 2020 | 2020 |
| Rheuma | C0003 | ZC3H | 801 | Q5D1E8 | zinc finger CCCH- | Nucleic | 88 | 0.5 | 0.6 | 0.14 | 0.01 | | 1 | 1 | 0 | 2012 | 20 |

| | | | | | | | | | | | | | | | | | |
|---|---|---|---|---|---|---|---|---|---|---|---|---|---|---|---|---|---|
| toid Arthritis | 873 | 12A | 49 | | type containing 12A | acid binding | | 78 | 54 | 015 | | | | | | | 12 |
| Rheumatoid Arthritis | C0003873 | BRD3 | 8019 | Q15059 | bromodomain containing 3 | Epigenetic regulator | 42 | 0.7 | 0.5 | 0.97468 | 0.01 | | 1 | 1 | 0 | 2016 | 2016 |
| Rheumatoid Arthritis | C0003873 | WDR26 | 80232 | Q9H7D7 | WD repeat domain 26 | | 90 | 0.606 | 0.769 | 1 | 0.01 | | 1 | 1 | 0 | 2017 | 2017 |
| Rheumatoid Arthritis | C0003873 | EFHC2 | 80258 | Q5JST6 | EF-hand domain containing 2 | | 8 | 0.792 | 0.538 | 0.9991 | 0.01 | | 1 | 1 | 0 | 2018 | 2018 |
| Rheumatoid Arthritis | C0003873 | FER1L4 | 80307 | A9Z1Z3 | fer-1 like family member 4 (pseudogene) | Transporter | 32 | 0.711 | 0.346 | | 0.01 | | 1 | 1 | 0 | 2019 | 2019 |
| Rheumatoid Arthritis | C0003873 | PDGFD | 80310 | Q9GZP0 | platelet derived growth factor D | Signaling | 94 | 0.579 | 0.538 | 0.001715 | 0.01 | | 1 | 1 | 0 | 2014 | 2014 |
| Rheumatoid Arthritis | C0003873 | TET1 | 80312 | Q8NFU7 | tet methylcytosine dioxygenase 1 | | 156 | 0.523 | 0.808 | 1 | 0.01 | | 1 | 1 | 0 | 2003 | 2003 |

| | | | | | | | | | | | | | | | | | |
|---|---|---|---|---|---|---|---|---|---|---|---|---|---|---|---|---|---|
| Rheumatoid Arthritis | C0003873 | PPP1R2C | 80316 | O14990 | PPP1R2C family member C | Enzyme modulator | 231 | 0.478 | 0.846 | | 0.01 | | 1 | 1 | 0 | 2019 | 2019 |
| Rheumatoid Arthritis | C0003873 | CEP70 | 80321 | Q8NHQ1 | centrosomal protein 70 | | 20 | 0.743 | 0.462 | 4.56E-15 | 0.01 | | 1 | 1 | 0 | 2018 | 2018 |
| Rheumatoid Arthritis | C0003873 | CSRP3 | 8048 | P50461 | cysteine and glycine rich protein 3 | Cellular structure | 108 | 0.572 | 0.731 | 0.000159 | 0.01 | | 1 | 1 | 0 | 2017 | 2017 |
| Rheumatoid Arthritis | C0003873 | CALM2 | 805 | P0DP23;P0DP24;P0DP25 | calmodulin 2 | Calcium-binding protein | 252 | 0.472 | 0.808 | 0.92086 | 0.01 | | 1 | 1 | 0 | 2020 | 2020 |
| Rheumatoid Arthritis | C0003873 | FOSL1 | 8061 | P15407 | FOS like 1, AP-1 transcription factor subunit | Transcription factor | 150 | 0.519 | 0.846 | 0.15849 | 0.01 | | 1 | 1 | 0 | 2019 | 2019 |
| Rheumatoid Arthritis | C0003873 | FGF23 | 8074 | Q9GZV9 | fibroblast growth factor 23 | Signaling | 305 | 0.46 | 0.731 | 0.031014 | 0.01 | | 1 | 1 | 0 | 2020 | 2020 |
| Rheumatoi | C0003 | COL18A1 | 807 | P39060 | collagen type XVIII alpha 1 chain | | 323 | 0.4 | 0.8 | 1.74E | 0.01 | | 1 | 1 | 1 | 2018 | 2018 |

| | | | | | | | | | | | | | | | | | |
|---|---|---|---|---|---|---|---|---|---|---|---|---|---|---|---|---|---|
| d Arthritis | 873 | | 81 | | | | | 49 | 08 | -10 | | | | | | | |
| Rheumatoid Arthritis | C0003873 | CALM3 | 808 | P0DP23;P0DP24;P0DP25 | calmodulin 3 | Calcium-binding protein | 215 | 0.485 | 0.846 | 0.92749 | 0.01 | | 1 | 1 | 0 | 2020 | 2020 |
| Rheumatoid Arthritis | C0003873 | APOL6 | 80830 | Q9BWW8 | apolipoprotein L6 | Transporter | 6 | 0.839 | 0.269 | 0.096029 | 0.01 | | 1 | 1 | 0 | 2017 | 2017 |
| Rheumatoid Arthritis | C0003873 | AAAS | 8086 | Q9NRG9 | aladin WD repeat nucleoporin | | 92 | 0.601 | 0.654 | 2.44E-13 | 0.01 | | 1 | 1 | 0 | 2016 | 2016 |
| Rheumatoid Arthritis | C0003873 | HMGA2 | 8091 | P52926 | high mobility group AT-hook 2 | Nucleic acid binding | 382 | 0.429 | 0.808 | 0.87015 | 0.01 | | 1 | 1 | 0 | 2017 | 2017 |
| Rheumatoid Arthritis | C0003873 | SLC38A1 | 81539 | Q9H2H9 | solute carrier family 38 member 1 | Transporter | 64 | 0.615 | 0.769 | 0.93067 | 0.01 | | 1 | 1 | 0 | 2012 | 2012 |
| Rheumatoid Arthritis | C0003873 | GDPD5 | 81544 | Q8WTR4 | glycerophosphodiester phosphodiesterase domain containing 5 | | 18 | 0.76 | 0.308 | 0.00025 | 0.01 | | 1 | 1 | 0 | 2007 | 2007 |

| | | | | | | | | | | | | | | | | |
|---|---|---|---|---|---|---|---|---|---|---|---|---|---|---|---|---|
| Rheumatoid Arthritis | C0003873 | LBH | 81606 | Q53QV2 | LBH regulator of WNT signaling pathway | | 27 | 0.686 | 0.5 | 0.01436 | 0.01 | | 1 | 1 | 0 | 2016 | 2016 |
| Rheumatoid Arthritis | C0003873 | FIP1L1 | 81608 | Q6UN15 | factor interacting with PAPOLA and CPSF1 | | 111 | 0.575 | 0.577 | 0.7889 | 0.01 | | 1 | 1 | 0 | 2018 | 2018 |
| Rheumatoid Arthritis | C0003873 | MAP1LC3B | 81631 | Q9GZQ8 | microtubule associated protein 1 light chain 3 beta | Cellular structure | 167 | 0.506 | 0.846 | 0.001907 | 0.01 | | 1 | 1 | 0 | 2019 | 2019 |
| Rheumatoid Arthritis | C0003873 | SLC14A2 | 8170 | Q15849 | solute carrier family 14 member 2 | Transporter | 71 | 0.606 | 0.692 | 1.06E-17 | 0.01 | | 1 | 1 | 0 | 2018 | 2018 |
| Rheumatoid Arthritis | C0003873 | MIA | 8190 | Q16674 | MIA SH3 domain containing | | 130 | 0.537 | 0.769 | 1.73E-05 | 0.01 | | 1 | 1 | 0 | 2007 | 2007 |
| Rheumatoid Arthritis | C0003873 | NCOA3 | 8202 | Q9Y6Q9 | nuclear receptor coactivator 3 | Kinase | 142 | 0.522 | 0.654 | 0.54065 | 0.01 | | 1 | 1 | 0 | 2003 | 2003 |
| Rheumatoid Ar | C0003873 | COLQ | 8292 | Q9Y215 | collagen like tail subunit of asymmetric acetylcholinesterase | | 92 | 0.628 | 0.654 | 7.94E- | 0.01 | | 1 | 1 | 0 | 2019 | 2019 |

| | | | | | | | | | | | | | | | | | |
|---|---|---|---|---|---|---|---|---|---|---|---|---|---|---|---|---|---|
| thritis | | | | | | | | | | 14 | | | | | | | |
| Rheumatoid Arthritis | C0003873 | EOMES | 8320 | O95936 | eomesodermin | Transcription factor | 60 | 0.619 | 0.692 | 0.98443 | 0.01 | | 1 | 1 | 1 | 2018 | 2018 |
| Rheumatoid Arthritis | C0003873 | ARHGAP24 | 83478 | Q8N264 | Rho GTPase activating protein 24 | | 273 | 0.456 | 0.808 | 3.95E-10 | 0.01 | | 1 | 1 | 0 | 2017 | 2017 |
| Rheumatoid Arthritis | C0003873 | BCL2L12 | 83596 | Q9HB09 | BCL2 like 12 | | 67 | 0.606 | 0.654 | 3.83E-09 | 0.01 | | 1 | 1 | 0 | 2019 | 2019 |
| Rheumatoid Arthritis | C0003873 | FAM167A | 83648 | Q96KS9 | family with sequence similarity 167 member A | | 31 | 0.678 | 0.692 | 1.02E-11 | 0.01 | | 1 | 1 | 0 | 2011 | 2011 |
| Rheumatoid Arthritis | C0003873 | PARP9 | 83666 | Q8IXQ6 | poly(ADP-ribose) polymerase family member 9 | | 116 | 0.554 | 0.808 | 5E-09 | 0.01 | | 1 | 1 | 0 | 2019 | 2019 |
| Rheumatoid Arthritis | C0003873 | SESN2 | 83667 | P58004 | sestrin 2 | Enzyme | 169 | 0.508 | 0.808 | 4.19E-07 | 0.01 | | 1 | 1 | 0 | 2018 | 2018 |
| Rheumatoi | C0003 | ITCH | 837 | Q96J02 | itchy E3 ubiquitin protein ligase | Enzyme | 123 | 0.5 | 0.7 | 0.999 | 0.01 | | 1 | 1 | 0 | 2008 | 2008 |

| | | | | | | | | | | | | | | | | | |
|---|---|---|---|---|---|---|---|---|---|---|---|---|---|---|---|---|---|
| d Arthritis | 873 | | 37 | | | | | 56 | 69 | 97 | | | | | | | |
| Rheumatoid Arthritis | C0003873 | PLA2G10 | 8399 | O15496 | phospholipase A2 group X | Enzyme | 86 | 0.581 | 0.692 | 0.069507 | 0.01 | | 1 | 1 | 0 | 2011 | 2011 |
| Rheumatoid Arthritis | C0003873 | TMPRSS13 | 8400 | Q9BYE2 | transmembrane serine protease 13 | Enzyme | 116 | 0.545 | 0.846 | 1.48E-08 | 0.01 | | 1 | 1 | 0 | 2017 | 2017 |
| Rheumatoid Arthritis | C0003873 | ZNF644 | 8414 6 | Q9H582 | zinc finger protein 644 | Transcription factor | 6 | 0.821 | 0.192 | 0.99914 | 0.01 | | 1 | 1 | 0 | 2015 | 2015 |
| Rheumatoid Arthritis | C0003873 | RECK | 8434 | O95980 | reversion inducing cysteine rich protein with kazal motifs | Enzyme modulator | 123 | 0.547 | 0.731 | 1.93E-10 | 0.01 | | 1 | 1 | 0 | 2005 | 2005 |
| Rheumatoid Arthritis | C0003873 | CAVIN2 | 8436 | O95810 | caveolae associated protein 2 | Transcription factor | 61 | 0.617 | 0.615 | 7.24E-09 | 0.01 | | 1 | 1 | 0 | 2012 | 2012 |
| Rheumatoid Arthritis | C0003873 | DOT1L | 84444 | Q8TEK3 | DOT1 like histone lysine methyltransferase | Epigenetic regulator | 79 | 0.599 | 0.692 | 1 | 0.01 | | 1 | 1 | 0 | 2018 | 2018 |

| | | | | | | | | | | | | | | | | | |
|---|---|---|---|---|---|---|---|---|---|---|---|---|---|---|---|---|---|
| Rheumatoid Arthritis | C0003873 | MAP1LC3A | 84557 | Q9H492 | microtubule associated protein 1 light chain 3 alpha | Cellular structure | 67 | 0.61 | 0.654 | 0.027922 | 0.01 | | 1 | 1 | 0 | 2018 | 2018 |
| Rheumatoid Arthritis | C0003873 | CASR | 846 | P41180 | calcium sensing receptor | G-protein coupled receptor | 517 | 0.41 | 0.846 | 0.046698 | 0.01 | | 1 | 1 | 0 | 2017 | 2017 |
| Rheumatoid Arthritis | C0003873 | ZNF469 | 84627 | Q96JG9 | zinc finger protein 469 | | 101 | 0.584 | 0.769 | 0.71866 | 0.01 | | 1 | 1 | 0 | 2017 | 2017 |
| Rheumatoid Arthritis | C0003873 | GPR65 | 8477 | Q8IYL9 | G protein-coupled receptor 65 | G-protein coupled receptor | 47 | 0.653 | 0.5 | 0.1114 | 0.01 | | 1 | 1 | 0 | 2017 | 2017 |
| Rheumatoid Arthritis | C0003873 | CILP | 8483 | O75339 | cartilage intermediate layer protein | | 19 | 0.736 | 0.423 | 5.37E-26 | 0.01 | | 1 | 1 | 0 | 2004 | 2004 |
| Rheumatoid Arthritis | C0003873 | MAP4K3 | 8491 | Q8IVH8 | mitogen-activated protein kinase kinase kinase kinase 3 | Kinase | 29 | 0.711 | 0.423 | 0.10987 | 0.01 | | 1 | 1 | 0 | 2013 | 2013 |

| | | | | | | | | | | | | | | | | | |
|---|---|---|---|---|---|---|---|---|---|---|---|---|---|---|---|---|---|
| Rheumatoid Arthritis | C0003873 | PPM1D | 8493 | O15297 | protein phosphatase, Mg2+/ Mn2+ dependent 1D | Enzyme | 177 | 0.513 | 0.808 | 2.23E-11 | 0.01 | | 1 | 1 | 0 | 2019 | 2019 |
| Rheumatoid Arthritis | C0003873 | TMEM60 | 85025 | Q9H2L4 | transmembrane protein 60 | | 10 | 0.821 | 0.115 | 0.75498 | 0.01 | | 1 | 1 | 0 | 2018 | 2018 |
| Rheumatoid Arthritis | C0003873 | DNAJC14 | 85406 | Q6Y2X3 | DnaJ heat shock protein family (Hsp40) member C14 | | 11 | 0.805 | 0.423 | 0.99999 | 0.01 | | 1 | 1 | 0 | 1999 | 1999 |
| Rheumatoid Arthritis | C0003873 | PIR | 8544 | O00625 | pirin | Transcription factor | 26 | 0.716 | 0.385 | 5.34E-10 | 0.01 | | 1 | 1 | 0 | 2012 | 2012 |
| Rheumatoid Arthritis | C0003873 | DCLK3 | 85443 | Q9C098 | doublecortin like kinase 3 | Kinase | 47 | 0.647 | 0.615 | 0.13686 | 0.01 | | 0 | 1 | 0 | 2016 | 2016 |
| Rheumatoid Arthritis | C0003873 | KHSRP | 8570 | Q92945 | KH-type splicing regulatory protein | Enzyme | 148 | 0.526 | 0.846 | 1 | 0.01 | | 1 | 1 | 0 | 1992 | 1992 |
| Rheumatoid Ar | C0003873 | STC2 | 8614 | O76061 | stanniocalcin 2 | Signaling | 88 | 0.573 | 0.692 | 0.26588 | 0.01 | | 1 | 1 | 0 | 2008 | 2008 |

| | | | | | | | | | | | | | | | | | |
|---|---|---|---|---|---|---|---|---|---|---|---|---|---|---|---|---|---|
| thritis | | | | | | | | | | | | | | | | | |
| Rheumatoid Arthritis | C0003873 | PSMG1 | 8624 | O95456 | proteasome assembly chaperone 1 | | 97 | 0.579 | 0.731 | 0.0003342 | 0.01 | | 1 | 1 | 0 | 2018 | 2018 |
| Rheumatoid Arthritis | C0003873 | HSD17B6 | 8630 | O14756 | hydroxysteroid 17-beta dehydrogenase 6 | Enzyme | 82 | 0.592 | 0.692 | 0.34807 | 0.01 | | 0 | 1 | 0 | 2019 | 2019 |
| Rheumatoid Arthritis | C0003873 | UNC5C | 8633 | O95185 | unc-5 netrin receptor C | Receptor | 47 | 0.65 | 0.462 | 0.000881 | 0.01 | | 1 | 1 | 0 | 2009 | 2009 |
| Rheumatoid Arthritis | C0003873 | NCOA1 | 8648 | Q15788 | nuclear receptor coactivator 1 | Kinase | 108 | 0.563 | 0.731 | 0.99981 | 0.01 | | 1 | 1 | 0 | 2003 | 2003 |
| Rheumatoid Arthritis | C0003873 | NUMB | 8650 | P49757 | NUMB endocytic adaptor protein | Signaling | 55 | 0.619 | 0.731 | 0.003996 | 0.01 | | 1 | 1 | 0 | 2010 | 2010 |
| Rheumatoid Arthritis | C0003873 | MARCO | 8685 | Q9UEW3 | macrophage receptor with collagenous structure | | 42 | 0.659 | 0.5 | 7.77E-21 | 0.01 | | 1 | 1 | 0 | 2001 | 2001 |
| Rheumatoi | C0003 | HYAL2 | 8692 | Q12891 | hyaluronidase 2 | Enzyme | 54 | 0.6 | 0.5 | 0.186 | 0.01 | | 1 | 1 | 0 | 2004 | 2004 |

| | | | | | | | | | | | | | | | | | |
|---|---|---|---|---|---|---|---|---|---|---|---|---|---|---|---|---|---|
| d Arthritis | 873 | | | | | | | 44 | 77 | 82 | | | | | | | |
| Rheumatoid Arthritis | C0003873 | ACTN1 | 87 | P12814 | actinin alpha 1 | | 34 | 0.695 | 0.577 | 0.99997 | 0.01 | | 1 | 1 | 0 | 2014 | 2014 |
| Rheumatoid Arthritis | C0003873 | URI1 | 8725 | O94763 | URI1 prefoldin like chaperone | Transcription factor | 53 | 0.633 | 0.654 | 1.11E-05 | 0.01 | | 1 | 1 | 0 | 2014 | 2014 |
| Rheumatoid Arthritis | C0003873 | RIPK1 | 8737 | Q13546 | receptor interacting serine/threonine kinase 1 | Kinase | 250 | 0.479 | 0.769 | 0.007944 | 0.01 | | 1 | 1 | 0 | 2018 | 2018 |
| Rheumatoid Arthritis | C0003873 | TNFSF9 | 8744 | P41273 | TNF superfamily member 9 | | 90 | 0.579 | 0.692 | 0.31823 | 0.01 | | 1 | 1 | 0 | 2004 | 2004 |
| Rheumatoid Arthritis | C0003873 | TNFRSF18 | 8784 | Q9Y5U5 | TNF receptor superfamily member 18 | | 53 | 0.631 | 0.538 | 1.45E-06 | 0.01 | | 1 | 1 | 0 | 2008 | 2008 |
| Rheumatoid Arthritis | C0003873 | CREG1 | 8804 | O75629 | cellular repressor of E1A stimulated genes 1 | | 37 | 0.663 | 0.423 | 0.001223 | 0.01 | | 1 | 1 | 0 | 1987 | 1987 |

| | | | | | | | | | | | | | | | | |
|---|---|---|---|---|---|---|---|---|---|---|---|---|---|---|---|---|
| Rheumatoid Arthritis | C0003873 | HESX1 | 8820 | Q9UBX0 | HESX homeobox 1 | | 143 | 0.561 | 0.654 | 0.00797 | 0.01 | | 1 | 1 | 0 | 1993 | 1993 |
| Rheumatoid Arthritis | C0003873 | SOCS2 | 8835 | O14508 | suppressor of cytokine signaling 2 | Enzyme modulator | 104 | 0.56 | 0.731 | 0.62999 | 0.01 | | 1 | 1 | 0 | 2008 | 2008 |
| Rheumatoid Arthritis | C0003873 | CCN5 | 8839 | O76076 | cellular communication network factor 5 | Signaling | 61 | 0.621 | 0.462 | 1.97E-05 | 0.01 | | 1 | 1 | 0 | 2015 | 2015 |
| Rheumatoid Arthritis | C0003873 | CCN4 | 8840 | O95388 | cellular communication network factor 4 | Signaling | 114 | 0.554 | 0.692 | 6.39E-07 | 0.01 | | 1 | 1 | 5 | 2019 | 2019 |
| Rheumatoid Arthritis | C0003873 | PROM1 | 8842 | O43490 | prominin 1 | Transporter | 477 | 0.41 | 0.846 | 1.95E-22 | 0.01 | | 1 | 1 | 0 | 2019 | 2019 |
| Rheumatoid Arthritis | C0003873 | CDK5R1 | 8851 | Q15078 | cyclin dependent kinase 5 regulatory subunit 1 | Enzyme modulator | 153 | 0.529 | 0.808 | 0.9149 | 0.01 | | 1 | 1 | 0 | 2015 | 2015 |
| Rheumatoid Ar | C0003873 | ALDH1A2 | 8854 | O94788 | aldehyde dehydrogenase 1 family | Enzyme | 109 | 0.578 | 0.692 | 0.36331 | 0.01 | | 1 | 1 | 0 | 2015 | 2015 |

| | | | | | | | | | | | | | | | | | |
|---|---|---|---|---|---|---|---|---|---|---|---|---|---|---|---|---|---|
| thritis | | | | | member A2 | | | | | | | | | | | | |
| Rheumatoid Arthritis | C0003873 | PER3 | 8863 | P56645 | period circadian regulator 3 | Transcription factor | 122 | 0.559 | 0.731 | 1.37E-17 | 0.01 | | 1 | 1 | 0 | 2015 | 2015 |
| Rheumatoid Arthritis | C0003873 | IER3 | 8870 | P46695 | immediate early response 3 | | 78 | 0.593 | 0.769 | 0.631122 | 0.01 | | 1 | 1 | 0 | 2016 | 2016 |
| Rheumatoid Arthritis | C0003873 | ARHGEF7 | 8874 | Q14155 | Rho guanine nucleotide exchange factor 7 | | 215 | 0.486 | 0.846 | 0.99995 | 0.01 | | 1 | 1 | 1 | 2002 | 2002 |
| Rheumatoid Arthritis | C0003873 | KRIT1 | 889 | O00522 | KRIT1 ankyrin repeat containing | | 201 | 0.5 | 0.808 | 0.000693 | 0.01 | | 1 | 1 | 0 | 2020 | 2020 |
| Rheumatoid Arthritis | C0003873 | MTMR3 | 8897 | Q13615 | myotubularin related protein 3 | Enzyme | 41 | 0.682 | 0.462 | 0.99937 | 0.01 | | 1 | 1 | 1 | 2019 | 2019 |
| Rheumatoid Arthritis | C0003873 | CCNB1 | 891 | P14635 | cyclin B1 | Enzyme modulator | 155 | 0.512 | 0.731 | 0.15993 | 0.01 | | 1 | 1 | 0 | 2012 | 2012 |
| Rheumatoi | C0003 | KYNU | 8942 | Q16719 | kynureninase | Enzyme | 88 | 0.6 | 0.6 | 4.18E | 0.01 | | 1 | 1 | 0 | 2018 | 2018 |

| | | | | | | | | | | | | | | | | | |
|---|---|---|---|---|---|---|---|---|---|---|---|---|---|---|---|---|---|
| d Arthritis | 873 | | | | | | | 17 | 92 | -18 | | | | | | | |
| Rheumatoid Arthritis | C0003873 | LMLN | 89782 | Q96KR4 | leishmanolysin like peptidase | Enzyme | 138 | 0.528 | 0.846 | 1.87E-14 | 0.01 | | 1 | 1 | 0 | 2017 | 2017 |
| Rheumatoid Arthritis | C0003873 | RPS6KA4 | 89986 | O75676 | ribosomal protein S6 kinase A4 | Kinase | 17 | 0.743 | 0.269 | 0.99715 | 0.01 | | 1 | 1 | 0 | 2014 | 2014 |
| Rheumatoid Arthritis | C0003873 | HSPB3 | 89988 | Q12988 | heat shock protein family B (small) member 3 | | 327 | 0.448 | 0.769 | 6.09E-05 | 0.01 | | 1 | 1 | 0 | 2011 | 2011 |
| Rheumatoid Arthritis | C0003873 | UCN2 | 90226 | Q96RP3 | urocortin 2 | | 56 | 0.633 | 0.577 | 0.037947 | 0.01 | | 1 | 1 | 0 | 2011 | 2011 |
| Rheumatoid Arthritis | C0003873 | PKD2L1 | 90333 | Q9P0L9 | polycystin 2 like 1, transient receptor potential cation channel | Ion channel | 124 | 0.565 | 0.731 | 1.57E-27 | 0.01 | | 1 | 1 | 0 | 2018 | 2018 |
| Rheumatoid Arthritis | C0003873 | GADD45GIP1 | 90480 | Q8TAE8 | GADD45G interacting protein 1 | | 22 | 0.716 | 0.423 | 0.000103 | 0.01 | | 1 | 1 | 0 | 2019 | 2019 |

| | | | | | | | | | | | | | | | | | |
|---|---|---|---|---|---|---|---|---|---|---|---|---|---|---|---|---|---|
| Rheumatoid Arthritis | C0003873 | CCT | 907 | | cataract, congenital, total | | 75 | 0.588 | 0.769 | | 0.01 | | 1 | 1 | 0 | 2015 | 2015 |
| Rheumatoid Arthritis | C0003873 | CLDN1 | 9076 | O95832 | claudin 1 | Cell-cell junction | 234 | 0.481 | 0.808 | 0.0101 | 0.01 | | 1 | 1 | 0 | 2018 | 2018 |
| Rheumatoid Arthritis | C0003873 | MTA1 | 9112 | Q13330 | metastasis associated 1 | Nucleic acid binding | 118 | 0.547 | 0.692 | 1 | 0.01 | | 1 | 1 | 0 | 2016 | 2016 |
| Rheumatoid Arthritis | C0003873 | SLC16A4 | 9122 | O15374 | solute carrier family 16 member 4 | Transporter | 114 | 0.55 | 0.731 | 9.67E-08 | 0.01 | | 1 | 1 | 0 | 2015 | 2015 |
| Rheumatoid Arthritis | C0003873 | SLC16A3 | 9123 | O15427 | solute carrier family 16 member 3 | Transporter | 122 | 0.542 | 0.731 | 0.000294 | 0.01 | | 1 | 1 | 0 | 2015 | 2015 |
| Rheumatoid Arthritis | C0003873 | P2RX6 | 9127 | O15547 | purinergic receptor P2X 6 | Ion channel | 85 | 0.599 | 0.769 | 4.47E-09 | 0.01 | | 1 | 1 | 0 | 2010 | 2010 |
| Rheumatoid Ar | C0003873 | SLC28A2 | 9153 | O43868 | solute carrier family 28 member 2 | Transporter | 12 | 0.78 | 0.385 | 1.13E- | 0.01 | | 0 | 1 | 0 | 2018 | 2018 |

| | | | | | | | | | | | | | | | | | |
|---|---|---|---|---|---|---|---|---|---|---|---|---|---|---|---|---|---|
| thritis | | | | | | | | | | 27 | | | | | | | |
| Rheumatoid Arthritis | C0003873 | EBAG9 | 9166 | O00559 | estrogen receptor binding site associated antigen 9 | | 69 | 0.615 | 0.462 | 0.077101 | 0.01 | | 1 | 1 | 0 | 2008 | 2008 |
| Rheumatoid Arthritis | C0003873 | MFSD4B | 91749 | Q5TF39 | major facilitator superfamily domain containing 4B | Transporter | 13 | 0.805 | 0.269 | 8.3E-06 | 0.01 | | 1 | 1 | 0 | 2014 | 2014 |
| Rheumatoid Arthritis | C0003873 | OSMR | 9180 | Q99650 | oncostatin M receptor | Signaling | 77 | 0.599 | 0.577 | 9.21E-18 | 0.01 | | 1 | 1 | 0 | 2014 | 2014 |
| Rheumatoid Arthritis | C0003873 | DEDD | 9191 | O75618 | death effector domain containing | | 24 | 0.711 | 0.346 | 0.95912 | 0.01 | | 1 | 1 | 0 | 2010 | 2010 |
| Rheumatoid Arthritis | C0003873 | CD4 | 920 | P01730 | CD4 molecule | | 73 | 0.604 | 0.654 | 0.000789 | 0.01 | | 1 | 1 | 0 | 2008 | 2008 |
| Rheumatoid Arthritis | C0003873 | MTDH | 92140 | Q86UE4 | metadherin | | 200 | 0.491 | 0.769 | 0.32924 | 0.01 | | 1 | 1 | 0 | 2017 | 2017 |
| Rheumatoi | C0003 | LRAT | 9227 | O95237 | lecithin retinol acyltransferase | | 84 | 0.6 | 0.6 | 0.028 | 0.01 | | 1 | 1 | 0 | 2009 | 2009 |

| | | | | | | | | | | | | | | | | |
|---|---|---|---|---|---|---|---|---|---|---|---|---|---|---|---|---|
| d Arthritis | 873 | | | | | | | 08 | 54 | 354 | | | | | | |
| Rheumatoid Arthritis | C0003873 | NAF1 | 92345 | Q96HR8 | nuclear assembly factor 1 ribonucleoprotein | | 27 | 0.7 | 0.5 | 0.97793 | 0.01 | | 1 | 1 | 0 | 2003 | 2003 |
| Rheumatoid Arthritis | C0003873 | RPS6KA5 | 9252 | O75582 | ribosomal protein S6 kinase A5 | Kinase | 28 | 0.722 | 0.577 | 0.99909 | 0.01 | | 1 | 1 | 0 | 2014 | 2014 |
| Rheumatoid Arthritis | C0003873 | AIMP1 | 9255 | Q12904 | aminoacyl tRNA synthetase complex interacting multifunctional protein 1 | Enzyme | 125 | 0.566 | 0.769 | 3.04E-07 | 0.01 | | 1 | 1 | 0 | 2002 | 2002 |
| Rheumatoid Arthritis | C0003873 | PDLIM7 | 9260 | Q9NR12 | PDZ and LIM domain 7 | Cellular structure | 241 | 0.474 | 0.808 | 0.70838 | 0.01 | | 1 | 1 | 0 | 2013 | 2013 |
| Rheumatoid Arthritis | C0003873 | PIWIL1 | 9271 | Q96J94 | piwi like RNA-mediated gene silencing 1 | | 99 | 0.57 | 0.731 | 7.28E-14 | 0.01 | | 1 | 1 | 0 | 2016 | 2016 |
| Rheumatoid Arthritis | C0003873 | CD9 | 928 | P21926 | CD9 molecule | | 156 | 0.528 | 0.769 | 0.004722 | 0.01 | | 1 | 1 | 0 | 1989 | 1989 |

| | | | | | | | | | | | | | | | | | |
|---|---|---|---|---|---|---|---|---|---|---|---|---|---|---|---|---|---|
| Rheumatoid Arthritis | C0003873 | ADGRG1 | 9289 | Q9Y653 | adhesion G protein-coupled receptor G1 | G-protein coupled receptor | 106 | 0.59 | 0.654 | 5.35E-10 | 0.01 | | 1 | 1 | 0 | 2018 | 2018 |
| Rheumatoid Arthritis | C0003873 | S1PR2 | 9294 | O95136 | sphingosine-1-phosphate receptor 2 | G-protein coupled receptor | 93 | 0.585 | 0.731 | 0.018286 | 0.01 | | 1 | 1 | 0 | 2008 | 2008 |
| Rheumatoid Arthritis | C0003873 | SRSF11 | 9295 | Q05519 | serine and arginine rich splicing factor 11 | Nucleic acid binding | 11 | 0.821 | 0.269 | 0.998006 | 0.01 | | 1 | 1 | 0 | 2017 | 2017 |
| Rheumatoid Arthritis | C0003873 | MMP20 | 9313 | O60882 | matrix metallopeptidase 20 | Enzyme | 40 | 0.663 | 0.538 | 6.08E-14 | 0.01 | | 1 | 1 | 0 | 1999 | 1999 |
| Rheumatoid Arthritis | C0003873 | PPIG | 9360 | Q13427 | peptidylprolyl isomerase G | | 203 | 0.497 | 0.846 | 0.962292 | 0.01 | | 1 | 1 | 0 | 2017 | 2017 |
| Rheumatoid Arthritis | C0003873 | LONP1 | 9361 | P36776 | lon peptidase 1, mitochondrial | Enzyme | 197 | 0.517 | 0.808 | 0.9993 | 0.01 | | 1 | 1 | 0 | 2019 | 2019 |

| | | | | | | | | | | | | | | | | | |
|---|---|---|---|---|---|---|---|---|---|---|---|---|---|---|---|---|---|
| Rheumatoid Arthritis | C0003873 | ZFYVE9 | 9372 | O95405 | zinc finger FYVE-type containing 9 | | 59 | 0.647 | 0.615 | 0.90244 | 0.01 | | 1 | 1 | 0 | 2007 | 2007 |
| Rheumatoid Arthritis | C0003873 | COX5A | 9377 | P20674 | cytochrome c oxidase subunit 5A | Enzyme | 171 | 0.515 | 0.846 | 0.49407 | 0.01 | | 1 | 1 | 0 | 2000 | 2000 |
| Rheumatoid Arthritis | C0003873 | CIAO1 | 9391 | O76071 | cytosolic iron-sulfur assembly component 1 | | 3 | 0.931 | 0.154 | 0.001245 | 0.01 | | 1 | 1 | 0 | 1998 | 1998 |
| Rheumatoid Arthritis | C0003873 | CLEC6A | 93978 | Q6EIG7 | C-type lectin domain containing 6A | Receptor | 35 | 0.666 | 0.423 | 1.13E-06 | 0.01 | | 1 | 1 | 2 | 2013 | 2013 |
| Rheumatoid Arthritis | C0003873 | ORMDL3 | 94103 | Q8N138 | ORMDL sphingolipid biosynthesis regulator 3 | | 35 | 0.666 | 0.423 | 0.78329 | 0.01 | | 1 | 1 | 0 | 2012 | 2012 |
| Rheumatoid Arthritis | C0003873 | CYP7B1 | 9420 | O75881 | cytochrome P450 family 7 subfamily B member 1 | Enzyme | 141 | 0.563 | 0.692 | 3.3E-06 | 0.01 | | 1 | 1 | 0 | 2005 | 2005 |
| Rheumatoid Ar | C0003873 | AIM2 | 9447 | O14862 | absent in melanoma 2 | | 167 | 0.521 | 0.769 | 2.19E- | 0.01 | | 1 | 1 | 0 | 2019 | 2019 |

| | | | | | | | | | | | | | | | | | |
|---|---|---|---|---|---|---|---|---|---|---|---|---|---|---|---|---|---|
| thritis | | | | | | | | | | 09 | | | | | | | |
| Rheumatoid Arthritis | C0003873 | IL27RA | 9466 | Q6UWB1 | interleukin 27 receptor subunit alpha | Signaling | 40 | 0.659 | 0.731 | 3.9E-12 | 0.01 | | 1 | 1 | 0 | 2010 | 2010 |
| Rheumatoid Arthritis | C0003873 | CHST3 | 9469 | Q7LGC8 | carbohydrate sulfotransferase 3 | | 214 | 0.517 | 0.846 | 0.007243 | 0.01 | | 0 | 1 | 0 | 2013 | 2013 |
| Rheumatoid Arthritis | C0003873 | CD36 | 948 | P16671 | CD36 molecule | Receptor | 351 | 0.446 | 0.846 | 1.09E-60 | 0.01 | | 1 | 1 | 0 | 2013 | 2013 |
| Rheumatoid Arthritis | C0003873 | ATP6V1G1 | 9550 | O75348 | ATPase H+ transporting V1 subunit G1 | Transporter | 2 | 0.931 | 0.154 | 0.10407 | 0.01 | | 1 | 1 | 0 | 2001 | 2001 |
| Rheumatoid Arthritis | C0003873 | GTF2IRD1 | 9569 | Q9UHL9 | GTF2I repeat domain containing 1 | | 222 | 0.522 | 0.769 | 0.90039 | 0.01 | | 1 | 1 | 0 | 2019 | 2019 |
| Rheumatoid Arthritis | C0003873 | PRDX6 | 9588 | P30041 | peroxiredoxin 6 | Enzyme | 93 | 0.572 | 0.731 | 4.82E-05 | 0.01 | | 1 | 1 | 0 | 2015 | 2015 |
| Rheumatoi | C0003 | AKAP12 | 9590 | Q02952 | A-kinase anchoring protein 12 | | 99 | 0.5 | 0.6 | 0.035 | 0.01 | | 1 | 1 | 0 | 2017 | 2017 |

| | | | | | | | | | | | | | | | | | |
|---|---|---|---|---|---|---|---|---|---|---|---|---|---|---|---|---|---|
| d Arthritis | 873 | | | | | | | 66 | 15 | 666 | | | | | | | |
| Rheumatoid Arthritis | C0003873 | CD47 | 961 | Q08722 | CD47 molecule | | 253 | 0.468 | 0.769 | 0.91924 | 0.01 | | 1 | 1 | 0 | 2017 | 2017 |
| Rheumatoid Arthritis | C0003873 | NCOR1 | 9611 | O75376 | nuclear receptor corepressor 1 | Transcription factor | 94 | 0.597 | 0.731 | 1 | 0.01 | | 1 | 1 | 0 | 2018 | 2018 |
| Rheumatoid Arthritis | C0003873 | ISG15 | 9636 | P05161 | ISG15 ubiquitin like modifier | Nucleic acid binding | 148 | 0.531 | 0.808 | 0.40527 | 0.01 | | 1 | 1 | 0 | 2016 | 2016 |
| Rheumatoid Arthritis | C0003873 | CD58 | 965 | P19256 | CD58 molecule | | 80 | 0.584 | 0.769 | 0.25092 | 0.01 | | 1 | 1 | 0 | 1996 | 1996 |
| Rheumatoid Arthritis | C0003873 | CD59 | 966 | P13987 | CD59 molecule (CD59 blood group) | | 262 | 0.469 | 0.808 | 0.60568 | 0.01 | | 1 | 1 | 0 | 2006 | 2006 |
| Rheumatoid Arthritis | C0003873 | BMS1P20 | 96610 | | BMS1 pseudogene 20 | | 28 | 0.682 | 0.577 | | 0.01 | | 1 | 1 | 0 | 1998 | 1998 |
| Rheu | C00 | LPIN2 | 96 | Q92539 | lipin 2 | | 81 | 0. | 0. | 5.0 | 0.01 | | 1 | 1 | 0 | 2017 | 20 |

| | | | | | | | | | | | | | | | | | |
|---|---|---|---|---|---|---|---|---|---|---|---|---|---|---|---|---|---|
| matoid Arthritis | 03873 | | 63 | | | | | 628 | 615 | 3E-05 | | | | | | | 17 |
| Rheumatoid Arthritis | C0003873 | MARF1 | 9665 | Q9Y4F3 | meiosis regulator and mRNA stability factor 1 | Nucleic acid binding | 4 | 0.931 | 0.154 | 1 | 0.01 | | 1 | 1 | 0 | 2018 | 2018 |
| Rheumatoid Arthritis | C0003873 | CD63 | 967 | P08962 | CD63 molecule | | 128 | 0.542 | 0.731 | 0.017439 | 0.01 | | 1 | 1 | 0 | 2007 | 2007 |
| Rheumatoid Arthritis | C0003873 | RIMS2 | 9699 | Q9UQ26 | regulating synaptic membrane exocytosis 2 | Enzyme modulator | 16 | 0.769 | 0.423 | 1 | 0.01 | | 0 | 1 | 0 | 1997 | 1997 |
| Rheumatoid Arthritis | C0003873 | CD74 | 972 | P04233 | CD74 molecule | Enzyme modulator | 170 | 0.512 | 0.808 | 0.24889 | 0.01 | | 1 | 1 | 0 | 2020 | 2020 |
| Rheumatoid Arthritis | C0003873 | CD79A | 973 | P11912 | CD79a molecule | Receptor | 134 | 0.536 | 0.731 | 0.69226 | 0.01 | | 1 | 1 | 0 | 2017 | 2017 |
| Rheumatoid Arthritis | C0003873 | HDAC4 | 9759 | P56524 | histone deacetylase 4 | Epigenetic regulator | 256 | 0.484 | 0.885 | 1 | 0.01 | | 1 | 1 | 0 | 2019 | 2019 |

| | | | | | | | | | | | | | | | | | |
|---|---|---|---|---|---|---|---|---|---|---|---|---|---|---|---|---|---|
| Rheumatoid Arthritis | C0003873 | PCLAF | 9768 | Q15004 | PCNA clamp associated factor | | 125 | 0.538 | 0.769 | 0.16276 | 0.01 | | 1 | 1 | 0 | 2017 | 2017 |
| Rheumatoid Arthritis | C0003873 | CDA | 978 | P32320 | cytidine deaminase | Enzyme | 103 | 0.558 | 0.731 | 0.008104 | 0.01 | | 1 | 1 | 0 | 2018 | 2018 |
| Rheumatoid Arthritis | C0003873 | GIT2 | 9815 | Q14161 | GIT ArfGAP 2 | | 26 | 0.716 | 0.385 | 0.99793 | 0.01 | | 1 | 1 | 0 | 2018 | 2018 |
| Rheumatoid Arthritis | C0003873 | CDK1 | 983 | P06493 | cyclin dependent kinase 1 | Kinase | 214 | 0.482 | 0.808 | 0.99206 | 0.01 | | 1 | 1 | 0 | 1999 | 1999 |
| Rheumatoid Arthritis | C0003873 | TESPA1 | 9840 | A2RU30 | thymocyte expressed, positive selection associated 1 | | 5 | 0.839 | 0.154 | 4.69E-06 | 0.01 | | 1 | 1 | 1 | 2015 | 2015 |
| Rheumatoid Arthritis | C0003873 | GAB2 | 9846 | Q9UQC2 | GRB2 associated binding protein 2 | Receptor | 110 | 0.563 | 0.692 | 0.98325 | 0.01 | | 1 | 1 | 0 | 2005 | 2005 |
| Rheumatoid Ar | C0003873 | NR1I3 | 9970 | Q14994 | nuclear receptor subfamily 1 group I | Nuclear receptor | 318 | 0.448 | 0.846 | 3.53E- | 0.01 | | 1 | 1 | 0 | 2001 | 2001 |

| | | | | | | | | | | | | | | | | |
|---|---|---|---|---|---|---|---|---|---|---|---|---|---|---|---|---|
| thritis | | | | | member 3 | | | | 08 | | | | | | | |
| Rheumatoid Arthritis | C0003873 | HNRNPDL | 9987 | O14979 | heterogeneous nuclear ribonucleoprotein D like | 82 | 0.597 | 0.654 | 0.077954 | 0.01 | | 1 | 1 | 0 | 1996 | 1996 |

**Table S4. GeneCards predicted disease targets for rheumatoid arthritis**

| Gene Symbol | Description | Category | Gifts | GC Id | Relevance score | GeneCards Link |
|---|---|---|---|---|---|---|
| PTPN22 | Protein Tyrosine Phosphatase Non-Receptor Type 22 | Protein Coding | 47 | GC01M113813 | 101.3037 | https://www.genecards.org/cgi-bin/carddisp.pl?gene=PTPN22 |
| IL6 | Interleukin 6 | Protein Coding | 47 | GC07P022725 | 92.88411 | https://www.genecards.org/cgi-bin/carddisp.pl?gene=IL6 |
| HLA-DRB1 | Major Histocompatibility Complex, Class II, DR Beta 1 | Protein Coding | 46 | GC06M066007 | 92.80508 | https://www.genecards.org/cgi-bin/carddisp.pl?gene=HLA-DRB1 |
| IL10 | Interleukin 10 | Protein Coding | 45 | GC01M206767 | 91.45555 | https://www.genecards.org/cgi-bin/carddisp.pl?gene=IL10 |
| TNF | Tumor Necrosis Factor | Protein Coding | 49 | GC06P083695 | 72.99629 | https://www.genecards.org/cgi-bin/carddisp.pl?gene=TNF |
| CIITA | Class II Major Histocompatibility Complex Transactivator | Protein Coding | 43 | GC16P012093 | 72.05125 | https://www.genecards.org/cgi-bin/carddisp.pl?gene=CIITA |
| STAT4 | Signal Transducer And Activator Of Transcription 4 | Protein Coding | 45 | GC02M191029 | 71.54918 | https://www.genecards.org/cgi-bin/carddisp.pl?gene=STAT4 |
| MIF | Macrophage Migration Inhibitory Factor | Protein Coding | 46 | GC22P023894 | 68.33475 | https://www.genecards.org/cgi-bin/carddisp.pl?gene=MIF |
| SLC22A4 | Solute Carrier Family 22 Member 4 | Protein Coding | 44 | GC05P132294 | 67.25108 | https://www.genecards.org/cgi-bin/carddisp.pl?gene=SLC22A4 |

| | | | | | | |
|---|---|---|---|---|---|---|
| IRF5 | Interferon Regulatory Factor 5 | Protein Coding | 45 | GC07P 128937 | 65.47 908 | https://www.genecards .org/cgi-bin/carddisp.pl?gene=I RF5 |
| IL2RA | Interleukin 2 Receptor Subunit Alpha | Protein Coding | 48 | GC10M 006010 | 63.51 823 | https://www.genecards .org/cgi-bin/carddisp.pl?gene=I L2RA |
| MIR146A | MicroRNA 146a | RNA Gene | 22 | GC05P 160485 | 61.58 196 | https://www.genecards .org/cgi-bin/carddisp.pl?gene= MIR146A |
| NFKBIL1 | NFKB Inhibitor Like 1 | Protein Coding | 37 | GC06P 083692 | 59.85 94 | https://www.genecards .org/cgi-bin/carddisp.pl?gene= NFKBIL1 |
| CD244 | CD244 Molecule | Protein Coding | 42 | GC01M 160830 | 59.49 183 | https://www.genecards .org/cgi-bin/carddisp.pl?gene= CD244 |
| PSTPIP1 | Proline-Serine-Threonine Phosphatase Interacting Protein 1 | Protein Coding | 43 | GC15P 076993 | 57.40 873 | https://www.genecards .org/cgi-bin/carddisp.pl?gene= PSTPIP1 |
| HLA-B | Major Histocompatibility Complex, Class I, B | Protein Coding | 45 | GC06M 065915 | 56.75 78 | https://www.genecards .org/cgi-bin/carddisp.pl?gene= HLA-B |
| IL2RB | Interleukin 2 Receptor Subunit Beta | Protein Coding | 46 | GC22M 037125 | 54.85 216 | https://www.genecards .org/cgi-bin/carddisp.pl?gene=I L2RB |
| MIR155 | MicroRNA 155 | RNA Gene | 21 | GC21P 025573 | 50.48 026 | https://www.genecards .org/cgi-bin/carddisp.pl?gene= MIR155 |
| TLR1 | Toll Like Receptor 1 | Protein Coding | 45 | GC04M 038793 | 50.32 65 | https://www.genecards .org/cgi-bin/carddisp.pl?gene= TLR1 |
| LACC1 | Laccase Domain Containing 1 | Protein Coding | 34 | GC13P 043879 | 49.96 17 | https://www.genecards .org/cgi-bin/carddisp.pl?gene= LACC1 |
| LTA | Lymphotoxin Alpha | Protein Coding | 40 | GC06P 083693 | 48.15 864 | https://www.genecards .org/cgi-bin/carddisp.pl?gene= LTA |
| MIR132 | MicroRNA 132 | RNA Gene | 22 | GC17M 002049 | 45.78 649 | https://www.genecards .org/cgi-bin/carddisp.pl?gene= MIR132 |
| CHRNG | Cholinergic Receptor Nicotinic Gamma Subunit | Protein Coding | 40 | GC02P 232539 | 45.76 495 | https://www.genecards .org/cgi- |

| | | | | | | |
|---|---|---|---|---|---|---|
| | | | | | | bin/carddisp.pl?gene=CHRNG |
| MIR150 | MicroRNA 150 | RNA Gene | 22 | GC19M049500 | 44.93126 | https://www.genecards.org/cgi-bin/carddisp.pl?gene=MIR150 |
| PADI4 | Peptidyl Arginine Deiminase 4 | Protein Coding | 42 | GC01P017308 | 43.63272 | https://www.genecards.org/cgi-bin/carddisp.pl?gene=PADI4 |
| IL1B | Interleukin 1 Beta | Protein Coding | 45 | GC02M112829 | 42.90001 | https://www.genecards.org/cgi-bin/carddisp.pl?gene=IL1B |
| CTLA4 | Cytotoxic T-Lymphocyte Associated Protein 4 | Protein Coding | 44 | GC02P203854 | 41.95121 | https://www.genecards.org/cgi-bin/carddisp.pl?gene=CTLA4 |
| CRP | C-Reactive Protein | Protein Coding | 44 | GC01M159727 | 41.41481 | https://www.genecards.org/cgi-bin/carddisp.pl?gene=CRP |
| COPA | COPI Coat Complex Subunit Alpha | Protein Coding | 41 | GC01M160288 | 40.80445 | https://www.genecards.org/cgi-bin/carddisp.pl?gene=COPA |
| PTPN2 | Protein Tyrosine Phosphatase Non-Receptor Type 2 | Protein Coding | 45 | GC18M024779 | 39.99292 | https://www.genecards.org/cgi-bin/carddisp.pl?gene=PTPN2 |
| CD247 | CD247 Molecule | Protein Coding | 47 | GC01M167399 | 39.31931 | https://www.genecards.org/cgi-bin/carddisp.pl?gene=CD247 |
| NOD2 | Nucleotide Binding Oligomerization Domain Containing 2 | Protein Coding | 46 | GC16P050693 | 38.51827 | https://www.genecards.org/cgi-bin/carddisp.pl?gene=NOD2 |
| TNFRSF1A | TNF Receptor Superfamily Member 1A | Protein Coding | 46 | GC12M006328 | 37.86541 | https://www.genecards.org/cgi-bin/carddisp.pl?gene=TNFRSF1A |
| IL17A | Interleukin 17A | Protein Coding | 40 | GC06P052186 | 37.71717 | https://www.genecards.org/cgi-bin/carddisp.pl?gene=IL17A |
| ANKRD55 | Ankyrin Repeat Domain 55 | Protein Coding | 32 | GC05M056099 | 37.61171 | https://www.genecards.org/cgi-bin/carddisp.pl?gene=ANKRD55 |
| NLRP1 | NLR Family Pyrin Domain Containing 1 | Protein Coding | 42 | GC17M005499 | 36.6207 | https://www.genecards.org/cgi-bin/carddisp.pl?gene=NLRP1 |

| | | | | | | |
|---|---|---|---|---|---|---|
| MMP3 | Matrix Metallopeptidase 3 | Protein Coding | 48 | GC11M 102835 | 35.73 695 | https://www.genecards .org/cgi- bin/carddisp.pl?gene= MMP3 |
| SYK | Spleen Associated Tyrosine Kinase | Protein Coding | 48 | GC09P 092021 | 35.20 958 | https://www.genecards .org/cgi- bin/carddisp.pl?gene= SYK |
| IFNG | Interferon Gamma | Protein Coding | 46 | GC12M 068154 | 34.14 323 | https://www.genecards .org/cgi- bin/carddisp.pl?gene=I FNG |
| IL1RN | Interleukin 1 Receptor Antagonist | Protein Coding | 46 | GC02P 122460 | 33.94 598 | https://www.genecards .org/cgi- bin/carddisp.pl?gene=I L1RN |
| CXCL8 | C-X-C Motif Chemokine Ligand 8 | Protein Coding | 38 | GC04P 073740 | 32.69 721 | https://www.genecards .org/cgi- bin/carddisp.pl?gene= CXCL8 |
| IL2 | Interleukin 2 | Protein Coding | 43 | GC04M 122451 | 32.21 674 | https://www.genecards .org/cgi- bin/carddisp.pl?gene=I L2 |
| TNFRSF1 B | TNF Receptor Superfamily Member 1B | Protein Coding | 45 | GC01P 012235 | 32.18 861 | https://www.genecards .org/cgi- bin/carddisp.pl?gene= TNFRSF1B |
| IL18 | Interleukin 18 | Protein Coding | 41 | GC11M 112143 | 31.71 845 | https://www.genecards .org/cgi- bin/carddisp.pl?gene=I L18 |
| TLR4 | Toll Like Receptor 4 | Protein Coding | 49 | GC09P 117704 | 31.29 188 | https://www.genecards .org/cgi- bin/carddisp.pl?gene= TLR4 |
| CCL2 | C-C Motif Chemokine Ligand 2 | Protein Coding | 46 | GC17P 034255 | 31.17 136 | https://www.genecards .org/cgi- bin/carddisp.pl?gene= CCL2 |
| FAS | Fas Cell Surface Death Receptor | Protein Coding | 48 | GC10P 092502 | 30.14 841 | https://www.genecards .org/cgi- bin/carddisp.pl?gene= FAS |
| TNFSF11 | TNF Superfamily Member 11 | Protein Coding | 45 | GC13P 042562 | 29.90 066 | https://www.genecards .org/cgi- bin/carddisp.pl?gene= TNFSF11 |
| MMP1 | Matrix Metallopeptidase 1 | Protein Coding | 49 | GC11M 102810 | 29.77 418 | https://www.genecards .org/cgi- bin/carddisp.pl?gene= MMP1 |
| IL4 | Interleukin 4 | Protein Coding | 44 | GC05P 132673 | 29.63 962 | https://www.genecards .org/cgi- |

| | | | | | | |
|---|---|---|---|---|---|---|
| | | | | | | bin/carddisp.pl?gene=IL4 |
| MEFV | MEFV Innate Immuity Regulator, Pyrin | Protein Coding | 42 | GC16M007269 | 29.47571 | https://www.genecards.org/cgi-bin/carddisp.pl?gene=MEFV |
| COMP | Cartilage Oligomeric Matrix Protein | Protein Coding | 45 | GC19M018783 | 28.75249 | https://www.genecards.org/cgi-bin/carddisp.pl?gene=COMP |
| FCRL3 | Fc Receptor Like 3 | Protein Coding | 37 | GC01M157674 | 28.64016 | https://www.genecards.org/cgi-bin/carddisp.pl?gene=FCRL3 |
| IL1R1 | Interleukin 1 Receptor Type 1 | Protein Coding | 44 | GC02P102136 | 28.62865 | https://www.genecards.org/cgi-bin/carddisp.pl?gene=IL1R1 |
| ACP5 | Acid Phosphatase 5, Tartrate Resistant | Protein Coding | 45 | GC19M011574 | 28.59747 | https://www.genecards.org/cgi-bin/carddisp.pl?gene=ACP5 |
| CCR6 | C-C Motif Chemokine Receptor 6 | Protein Coding | 42 | GC06P167111 | 27.92742 | https://www.genecards.org/cgi-bin/carddisp.pl?gene=CCR6 |
| PTGS2 | Prostaglandin-Endoperoxide Synthase 2 | Protein Coding | 47 | GC01M186640 | 27.54896 | https://www.genecards.org/cgi-bin/carddisp.pl?gene=PTGS2 |
| IL23R | Interleukin 23 Receptor | Protein Coding | 43 | GC01P067138 | 27.39661 | https://www.genecards.org/cgi-bin/carddisp.pl?gene=IL23R |
| TRAF1 | TNF Receptor Associated Factor 1 | Protein Coding | 41 | GC09M120902 | 27.28319 | https://www.genecards.org/cgi-bin/carddisp.pl?gene=TRAF1 |
| HLA-DQB1 | Major Histocompatibility Complex, Class II, DQ Beta 1 | Protein Coding | 42 | GC06M066016 | 27.15154 | https://www.genecards.org/cgi-bin/carddisp.pl?gene=HLA-DQB1 |
| IL1A | Interleukin 1 Alpha | Protein Coding | 42 | GC02M112773 | 27.0717 | https://www.genecards.org/cgi-bin/carddisp.pl?gene=IL1A |
| TNFRSF11B | TNF Receptor Superfamily Member 11b | Protein Coding | 46 | GC08M118923 | 26.88093 | https://www.genecards.org/cgi-bin/carddisp.pl?gene=TNFRSF11B |
| SAA1 | Serum Amyloid A1 | Protein Coding | 40 | GC11P018510 | 26.81518 | https://www.genecards.org/cgi-bin/carddisp.pl?gene=SAA1 |

| | | | | | | |
|---|---|---|---|---|---|---|
| COL2A1 | Collagen Type II Alpha 1 Chain | Protein Coding | 46 | GC12M 047972 | 26.33 231 | https://www.genecards .org/cgi-bin/carddisp.pl?gene= COL2A1 |
| CD40 | CD40 Molecule | Protein Coding | 46 | GC20P 046118 | 26.27 834 | https://www.genecards .org/cgi-bin/carddisp.pl?gene= CD40 |
| MMP13 | Matrix Metallopeptidase 13 | Protein Coding | 49 | GC11M 102942 | 25.94 187 | https://www.genecards .org/cgi-bin/carddisp.pl?gene= MMP13 |
| CCR5 | C-C Motif Chemokine Receptor 5 | Protein Coding | 44 | GC03P 046776 | 25.52 2 | https://www.genecards .org/cgi-bin/carddisp.pl?gene= CCR5 |
| MMP9 | Matrix Metallopeptidase 9 | Protein Coding | 52 | GC20P 046008 | 24.86 951 | https://www.genecards .org/cgi-bin/carddisp.pl?gene= MMP9 |
| MTHFR | Methylenetetrahydrofolate Reductase | Protein Coding | 46 | GC01M 011785 | 24.83 603 | https://www.genecards .org/cgi-bin/carddisp.pl?gene= MTHFR |
| MACIR | Macrophage Immunometabolism Regulator | Protein Coding | 24 | GC05P 103259 | 24.80 777 | https://www.genecards .org/cgi-bin/carddisp.pl?gene= MACIR |
| FCGR2A | Fc Gamma Receptor IIa | Protein Coding | 45 | GC01P 161505 | 24.80 118 | https://www.genecards .org/cgi-bin/carddisp.pl?gene= FCGR2A |
| SLC11A1 | Solute Carrier Family 11 Member 1 | Protein Coding | 45 | GC02P 218382 | 24.69 929 | https://www.genecards .org/cgi-bin/carddisp.pl?gene= SLC11A1 |
| IL6R | Interleukin 6 Receptor | Protein Coding | 46 | GC01P 154405 | 24.43 249 | https://www.genecards .org/cgi-bin/carddisp.pl?gene=I L6R |
| S100A12 | S100 Calcium Binding Protein A12 | Protein Coding | 38 | GC01M 153373 | 24.30 909 | https://www.genecards .org/cgi-bin/carddisp.pl?gene= S100A12 |
| MMP2 | Matrix Metallopeptidase 2 | Protein Coding | 50 | GC16P 055390 | 24.25 435 | https://www.genecards .org/cgi-bin/carddisp.pl?gene= MMP2 |
| TLR2 | Toll Like Receptor 2 | Protein Coding | 49 | GC04P 153684 | 24.10 735 | https://www.genecards .org/cgi-bin/carddisp.pl?gene= TLR2 |
| IL1RAPL 2 | Interleukin 1 Receptor Accessory Protein Like 2 | Protein Coding | 38 | GC0XP 104566 | 24.05 863 | https://www.genecards .org/cgi- |

| | | | | | | |
|---|---|---|---|---|---|---|
| | | | | | | bin/carddisp.pl?gene=IL1RAPL2 |
| IL15 | Interleukin 15 | Protein Coding | 38 | GC04P141636 | 24.05667 | https://www.genecards.org/cgi-bin/carddisp.pl?gene=IL15 |
| VEGFA | Vascular Endothelial Growth Factor A | Protein Coding | 46 | GC06P043770 | 23.87633 | https://www.genecards.org/cgi-bin/carddisp.pl?gene=VEGFA |
| TGFB1 | Transforming Growth Factor Beta 1 | Protein Coding | 49 | GC19M041301 | 23.78368 | https://www.genecards.org/cgi-bin/carddisp.pl?gene=TGFB1 |
| VCAM1 | Vascular Cell Adhesion Molecule 1 | Protein Coding | 44 | GC01P100719 | 23.69912 | https://www.genecards.org/cgi-bin/carddisp.pl?gene=VCAM1 |
| BLK | BLK Proto-Oncogene, Src Family Tyrosine Kinase | Protein Coding | 48 | GC08P011486 | 23.68699 | https://www.genecards.org/cgi-bin/carddisp.pl?gene=BLK |
| CCL5 | C-C Motif Chemokine Ligand 5 | Protein Coding | 41 | GC17M035871 | 23.44533 | https://www.genecards.org/cgi-bin/carddisp.pl?gene=CCL5 |
| NLRP3 | NLR Family Pyrin Domain Containing 3 | Protein Coding | 45 | GC01P247415 | 23.39787 | https://www.genecards.org/cgi-bin/carddisp.pl?gene=NLRP3 |
| S100A9 | S100 Calcium Binding Protein A9 | Protein Coding | 41 | GC01P153357 | 23.16271 | https://www.genecards.org/cgi-bin/carddisp.pl?gene=S100A9 |
| IL6ST | Interleukin 6 Cytokine Family Signal Transducer | Protein Coding | 46 | GC05M055935 | 23.02585 | https://www.genecards.org/cgi-bin/carddisp.pl?gene=IL6ST |
| FOXP3 | Forkhead Box P3 | Protein Coding | 45 | GC0XM049250 | 22.8975 | https://www.genecards.org/cgi-bin/carddisp.pl?gene=FOXP3 |
| FASLG | Fas Ligand | Protein Coding | 45 | GC01P172628 | 22.8768 | https://www.genecards.org/cgi-bin/carddisp.pl?gene=FASLG |
| TTR | Transthyretin | Protein Coding | 46 | GC18P031557 | 22.84327 | https://www.genecards.org/cgi-bin/carddisp.pl?gene=TTR |
| SPP1 | Secreted Phosphoprotein 1 | Protein Coding | 44 | GC04P087975 | 22.7118 | https://www.genecards.org/cgi-bin/carddisp.pl?gene=SPP1 |

| | | | | | | |
|---|---|---|---|---|---|---|
| FCGR3B | Fc Gamma Receptor IIIb | Protein Coding | 42 | GC01M 161623 | 22.61 53 | https://www.genecards .org/cgi-bin/carddisp.pl?gene= FCGR3B |
| PDCD1 | Programmed Cell Death 1 | Protein Coding | 45 | GC02M 241849 | 22.54 808 | https://www.genecards .org/cgi-bin/carddisp.pl?gene= PDCD1 |
| RELN | Reelin | Protein Coding | 42 | GC07M 103471 | 22.50 375 | https://www.genecards .org/cgi-bin/carddisp.pl?gene= RELN |
| SERPINH 1 | Serpin Family H Member 1 | Protein Coding | 44 | GC11P 075562 | 22.43 176 | https://www.genecards .org/cgi-bin/carddisp.pl?gene= SERPINH1 |
| TNIP1 | TNFAIP3 Interacting Protein 1 | Protein Coding | 39 | GC05M 151029 | 22.39 55 | https://www.genecards .org/cgi-bin/carddisp.pl?gene= TNIP1 |
| ICAM1 | Intercellular Adhesion Molecule 1 | Protein Coding | 48 | GC19P 010466 | 22.32 33 | https://www.genecards .org/cgi-bin/carddisp.pl?gene=I CAM1 |
| TIMP1 | TIMP Metallopeptidase Inhibitor 1 | Protein Coding | 42 | GC0XP 047596 | 22.24 322 | https://www.genecards .org/cgi-bin/carddisp.pl?gene= TIMP1 |
| FCGR3A | Fc Gamma Receptor IIIa | Protein Coding | 44 | GC01M 161541 | 22.21 11 | https://www.genecards .org/cgi-bin/carddisp.pl?gene= FCGR3A |
| CSF2 | Colony Stimulating Factor 2 | Protein Coding | 41 | GC05P 132073 | 22.16 519 | https://www.genecards .org/cgi-bin/carddisp.pl?gene= CSF2 |
| HLA-DQA1 | Major Histocompatibility Complex, Class II, DQ Alpha 1 | Protein Coding | 42 | GC06P 083727 | 22.13 786 | https://www.genecards .org/cgi-bin/carddisp.pl?gene= HLA-DQA1 |
| IL13 | Interleukin 13 | Protein Coding | 41 | GC05P 132656 | 21.81 797 | https://www.genecards .org/cgi-bin/carddisp.pl?gene=I L13 |
| STAT3 | Signal Transducer And Activator Of Transcription 3 | Protein Coding | 50 | GC17M 042313 | 21.56 902 | https://www.genecards .org/cgi-bin/carddisp.pl?gene= STAT3 |
| BGLAP | Bone Gamma-Carboxyglutamate Protein | Protein Coding | 38 | GC01P 156242 | 21.53 297 | https://www.genecards .org/cgi-bin/carddisp.pl?gene= BGLAP |
| TNFAIP3 | TNF Alpha Induced Protein 3 | Protein Coding | 46 | GC06P 137866 | 21.48 916 | https://www.genecards .org/cgi- |

| | | | | | | |
|---|---|---|---|---|---|---|
| | | | | | | bin/carddisp.pl?gene=TNFAIP3 |
| FCGR2B | Fc Gamma Receptor IIb | Protein Coding | 45 | GC01P161724 | 21.43319 | https://www.genecards.org/cgi-bin/carddisp.pl?gene=FCGR2B |
| IL21 | Interleukin 21 | Protein Coding | 42 | GC04M122612 | 21.09099 | https://www.genecards.org/cgi-bin/carddisp.pl?gene=IL21 |
| CD40LG | CD40 Ligand | Protein Coding | 46 | GC0XP136649 | 21.07109 | https://www.genecards.org/cgi-bin/carddisp.pl?gene=CD40LG |
| SELE | Selectin E | Protein Coding | 41 | GC01M169722 | 21.0079 | https://www.genecards.org/cgi-bin/carddisp.pl?gene=SELE |
| CSF1 | Colony Stimulating Factor 1 | Protein Coding | 42 | GC01P109911 | 20.96239 | https://www.genecards.org/cgi-bin/carddisp.pl?gene=CSF1 |
| CCR1 | C-C Motif Chemokine Receptor 1 | Protein Coding | 44 | GC03M046218 | 20.89093 | https://www.genecards.org/cgi-bin/carddisp.pl?gene=CCR1 |
| CD79A | CD79a Molecule | Protein Coding | 45 | GC19P041877 | 20.05658 | https://www.genecards.org/cgi-bin/carddisp.pl?gene=CD79A |
| MBL2 | Mannose Binding Lectin 2 | Protein Coding | 45 | GC10M052760 | 20.04278 | https://www.genecards.org/cgi-bin/carddisp.pl?gene=MBL2 |
| TNFRSF11A | TNF Receptor Superfamily Member 11a | Protein Coding | 44 | GC18P062325 | 20.01063 | https://www.genecards.org/cgi-bin/carddisp.pl?gene=TNFRSF11A |
| ITGAM | Integrin Subunit Alpha M | Protein Coding | 46 | GC16P041126 | 19.97729 | https://www.genecards.org/cgi-bin/carddisp.pl?gene=ITGAM |
| FLG | Filaggrin | Protein Coding | 40 | GC01M152274 | 19.95661 | https://www.genecards.org/cgi-bin/carddisp.pl?gene=FLG |
| HMGB1 | High Mobility Group Box 1 | Protein Coding | 44 | GC13M030456 | 19.69697 | https://www.genecards.org/cgi-bin/carddisp.pl?gene=HMGB1 |
| CXCL10 | C-X-C Motif Chemokine Ligand 10 | Protein Coding | 42 | GC04M076021 | 19.56744 | https://www.genecards.org/cgi-bin/carddisp.pl?gene=CXCL10 |

| | | | | | | |
|---|---|---|---|---|---|---|
| REL | REL Proto-Oncogene, NF-KB Subunit | Protein Coding | 45 | GC02P 060881 | 19.27 32 | https://www.genecards .org/cgi-bin/carddisp.pl?gene= REL |
| ADAM17 | ADAM Metallopeptidase Domain 17 | Protein Coding | 49 | GC02M 009488 | 19.21 072 | https://www.genecards .org/cgi-bin/carddisp.pl?gene= ADAM17 |
| ALB | Albumin | Protein Coding | 48 | GC04P 073397 | 19.17 338 | https://www.genecards .org/cgi-bin/carddisp.pl?gene= ALB |
| HLA-DPB1 | Major Histocompatibility Complex, Class II, DP Beta 1 | Protein Coding | 44 | GC06P 083734 | 19.14 252 | https://www.genecards .org/cgi-bin/carddisp.pl?gene= HLA-DPB1 |
| IL23A | Interleukin 23 Subunit Alpha | Protein Coding | 36 | GC12P 057293 | 19.07 604 | https://www.genecards .org/cgi-bin/carddisp.pl?gene=I L23A |
| IL17RA | Interleukin 17 Receptor A | Protein Coding | 43 | GC22P 018684 | 18.97 851 | https://www.genecards .org/cgi-bin/carddisp.pl?gene=I L17RA |
| GPI | Glucose-6-Phosphate Isomerase | Protein Coding | 46 | GC19P 034359 | 18.90 178 | https://www.genecards .org/cgi-bin/carddisp.pl?gene= GPI |
| TNFSF13 B | TNF Superfamily Member 13b | Protein Coding | 43 | GC13P 108251 | 18.76 754 | https://www.genecards .org/cgi-bin/carddisp.pl?gene= TNFSF13B |
| FSTL1 | Follistatin Like 1 | Protein Coding | 41 | GC03M 120392 | 18.68 401 | https://www.genecards .org/cgi-bin/carddisp.pl?gene= FSTL1 |
| PRTN3 | Proteinase 3 | Protein Coding | 44 | GC19P 000840 | 18.60 434 | https://www.genecards .org/cgi-bin/carddisp.pl?gene= PRTN3 |
| IL10RA | Interleukin 10 Receptor Subunit Alpha | Protein Coding | 44 | GC11P 117987 | 18.60 401 | https://www.genecards .org/cgi-bin/carddisp.pl?gene=I L10RA |
| MMP12 | Matrix Metallopeptidase 12 | Protein Coding | 45 | GC11M 102862 | 18.59 571 | https://www.genecards .org/cgi-bin/carddisp.pl?gene= MMP12 |
| HLA-C | Major Histocompatibility Complex, Class I, C | Protein Coding | 44 | GC06M 065914 | 18.48 296 | https://www.genecards .org/cgi-bin/carddisp.pl?gene= HLA-C |
| TNFAIP6 | TNF Alpha Induced Protein 6 | Protein Coding | 41 | GC02P 151357 | 18.47 494 | https://www.genecards .org/cgi- |

| | | | | | | |
|---|---|---|---|---|---|---|
| | | | | | | bin/carddisp.pl?gene=TNFAIP6 |
| PTGS1 | Prostaglandin-Endoperoxide Synthase 1 | Protein Coding | 45 | GC09P122370 | 18.27404 | https://www.genecards.org/cgi-bin/carddisp.pl?gene=PTGS1 |
| BTNL2 | Butyrophilin Like 2 | Protein Coding | 38 | GC06M032393 | 18.27045 | https://www.genecards.org/cgi-bin/carddisp.pl?gene=BTNL2 |
| HSPD1 | Heat Shock Protein Family D (Hsp60) Member 1 | Protein Coding | 46 | GC02M197486 | 18.14313 | https://www.genecards.org/cgi-bin/carddisp.pl?gene=HSPD1 |
| MICA | MHC Class I Polypeptide-Related Sequence A | Protein Coding | 38 | GC06P031399 | 18.13344 | https://www.genecards.org/cgi-bin/carddisp.pl?gene=MICA |
| CXCR3 | C-X-C Motif Chemokine Receptor 3 | Protein Coding | 42 | GC0XM071615 | 18.09852 | https://www.genecards.org/cgi-bin/carddisp.pl?gene=CXCR3 |
| MPO | Myeloperoxidase | Protein Coding | 49 | GC17M058269 | 18.08973 | https://www.genecards.org/cgi-bin/carddisp.pl?gene=MPO |
| CCL3 | C-C Motif Chemokine Ligand 3 | Protein Coding | 37 | GC17M036088 | 18.05998 | https://www.genecards.org/cgi-bin/carddisp.pl?gene=CCL3 |
| ITGAV | Integrin Subunit Alpha V | Protein Coding | 45 | GC02P186589 | 17.84391 | https://www.genecards.org/cgi-bin/carddisp.pl?gene=ITGAV |
| ADIPOQ | Adiponectin, C1Q And Collagen Domain Containing | Protein Coding | 44 | GC03P186842 | 17.7664 | https://www.genecards.org/cgi-bin/carddisp.pl?gene=ADIPOQ |
| MAFB | MAF BZIP Transcription Factor B | Protein Coding | 42 | GC20M040685 | 17.73362 | https://www.genecards.org/cgi-bin/carddisp.pl?gene=MAFB |
| MMP8 | Matrix Metallopeptidase 8 | Protein Coding | 46 | GC11M102617 | 17.72693 | https://www.genecards.org/cgi-bin/carddisp.pl?gene=MMP8 |
| CPT2 | Carnitine Palmitoyltransferase 2 | Protein Coding | 48 | GC01P053196 | 17.68966 | https://www.genecards.org/cgi-bin/carddisp.pl?gene=CPT2 |
| SLC19A1 | Solute Carrier Family 19 Member 1 | Protein Coding | 45 | GC21M045493 | 17.67178 | https://www.genecards.org/cgi-bin/carddisp.pl?gene=SLC19A1 |

| | | | | | | |
|---|---|---|---|---|---|---|
| TAGAP | T Cell Activation RhoGTPase Activating Protein | Protein Coding | 38 | GC06M 159034 | 17.66 101 | https://www.genecards.org/cgi-bin/carddisp.pl?gene=TAGAP |
| CCR2 | C-C Motif Chemokine Receptor 2 | Protein Coding | 44 | GC03P 046775 | 17.63 825 | https://www.genecards.org/cgi-bin/carddisp.pl?gene=CCR2 |
| OLAH | Oleoyl-ACP Hydrolase | Protein Coding | 33 | GC10P 015032 | 17.63 729 | https://www.genecards.org/cgi-bin/carddisp.pl?gene=OLAH |
| SIAE | Sialic Acid Acetylesterase | Protein Coding | 37 | GC11M 124633 | 17.62 323 | https://www.genecards.org/cgi-bin/carddisp.pl?gene=SIAE |
| IFNB1 | Interferon Beta 1 | Protein Coding | 41 | GC09M 021077 | 17.61 863 | https://www.genecards.org/cgi-bin/carddisp.pl?gene=IFNB1 |
| RELB | RELB Proto-Oncogene, NF-KB Subunit | Protein Coding | 43 | GC19P 066884 | 17.61 034 | https://www.genecards.org/cgi-bin/carddisp.pl?gene=RELB |
| NFKBIA | NFKB Inhibitor Alpha | Protein Coding | 48 | GC14M 035401 | 17.58 748 | https://www.genecards.org/cgi-bin/carddisp.pl?gene=NFKBIA |
| CAT | Catalase | Protein Coding | 49 | GC11P 034460 | 17.58 331 | https://www.genecards.org/cgi-bin/carddisp.pl?gene=CAT |
| MAPK14 | Mitogen-Activated Protein Kinase 14 | Protein Coding | 49 | GC06P 083841 | 17.47 903 | https://www.genecards.org/cgi-bin/carddisp.pl?gene=MAPK14 |
| CD28 | CD28 Molecule | Protein Coding | 45 | GC02P 203706 | 17.39 115 | https://www.genecards.org/cgi-bin/carddisp.pl?gene=CD28 |
| MAPK1 | Mitogen-Activated Protein Kinase 1 | Protein Coding | 49 | GC22M 021759 | 17.20 773 | https://www.genecards.org/cgi-bin/carddisp.pl?gene=MAPK1 |
| NOS2 | Nitric Oxide Synthase 2 | Protein Coding | 47 | GC17M 027756 | 17.16 114 | https://www.genecards.org/cgi-bin/carddisp.pl?gene=NOS2 |
| IL37 | Interleukin 37 | Protein Coding | 36 | GC02P 122457 | 17.11 699 | https://www.genecards.org/cgi-bin/carddisp.pl?gene=IL37 |
| CD44 | CD44 Molecule (Indian Blood Group) | Protein Coding | 45 | GC11P 035139 | 17.08 488 | https://www.genecards.org/cgi- |

| | | | | | | |
|---|---|---|---|---|---|---|
| | | | | | | bin/carddisp.pl?gene=CD44 |
| CHI3L1 | Chitinase 3 Like 1 | Protein Coding | 44 | GC01M203148 | 17.0572 | https://www.genecards.org/cgi-bin/carddisp.pl?gene=CHI3L1 |
| HSPA4 | Heat Shock Protein Family A (Hsp70) Member 4 | Protein Coding | 40 | GC05P133051 | 16.94462 | https://www.genecards.org/cgi-bin/carddisp.pl?gene=HSPA4 |
| STEAP4 | STEAP4 Metalloreductase | Protein Coding | 40 | GC07M088392 | 16.91617 | https://www.genecards.org/cgi-bin/carddisp.pl?gene=STEAP4 |
| CD36 | CD36 Molecule | Protein Coding | 46 | GC07P080369 | 16.88031 | https://www.genecards.org/cgi-bin/carddisp.pl?gene=CD36 |
| ATIC | 5-Aminoimidazole-4-Carboxamide Ribonucleotide Formyltransferase/IMP Cyclohydrolase | Protein Coding | 44 | GC02P215311 | 16.79704 | https://www.genecards.org/cgi-bin/carddisp.pl?gene=ATIC |
| HP | Haptoglobin | Protein Coding | 44 | GC16P072086 | 16.76994 | https://www.genecards.org/cgi-bin/carddisp.pl?gene=HP |
| S100A8 | S100 Calcium Binding Protein A8 | Protein Coding | 41 | GC01M153391 | 16.75831 | https://www.genecards.org/cgi-bin/carddisp.pl?gene=S100A8 |
| CXCL12 | C-X-C Motif Chemokine Ligand 12 | Protein Coding | 42 | GC10M044370 | 16.75396 | https://www.genecards.org/cgi-bin/carddisp.pl?gene=CXCL12 |
| HLA-DMA | Major Histocompatibility Complex, Class II, DM Alpha | Protein Coding | 39 | GC06M066028 | 16.75094 | https://www.genecards.org/cgi-bin/carddisp.pl?gene=HLA-DMA |
| TNFRSF13C | TNF Receptor Superfamily Member 13C | Protein Coding | 42 | GC22M057604 | 16.65233 | https://www.genecards.org/cgi-bin/carddisp.pl?gene=TNFRSF13C |
| MICB | MHC Class I Polypeptide-Related Sequence B | Protein Coding | 40 | GC06P083690 | 16.64386 | https://www.genecards.org/cgi-bin/carddisp.pl?gene=MICB |
| IL7 | Interleukin 7 | Protein Coding | 40 | GC08M078689 | 16.61375 | https://www.genecards.org/cgi-bin/carddisp.pl?gene=IL7 |
| PIK3CG | Phosphatidylinositol-4,5-Bisphosphate 3-Kinase Catalytic Subunit Gamma | Protein Coding | 46 | GC07P106865 | 16.56232 | https://www.genecards.org/cgi- |

| | | | | | | |
|---|---|---|---|---|---|---|
| | | | | | | bin/carddisp.pl?gene=PIK3CG |
| HSPA5 | Heat Shock Protein Family A (Hsp70) Member 5 | Protein Coding | 46 | GC09M125234 | 16.54642 | https://www.genecards.org/cgi-bin/carddisp.pl?gene=HSPA5 |
| CARD8 | Caspase Recruitment Domain Family Member 8 | Protein Coding | 39 | GC19M066028 | 16.53558 | https://www.genecards.org/cgi-bin/carddisp.pl?gene=CARD8 |
| TFRC | Transferrin Receptor | Protein Coding | 47 | GC03M196027 | 16.45774 | https://www.genecards.org/cgi-bin/carddisp.pl?gene=TFRC |
| TLR9 | Toll Like Receptor 9 | Protein Coding | 44 | GC03M052222 | 16.39536 | https://www.genecards.org/cgi-bin/carddisp.pl?gene=TLR9 |
| JUN | Jun Proto-Oncogene, AP-1 Transcription Factor Subunit | Protein Coding | 46 | GC01M058780 | 16.35247 | https://www.genecards.org/cgi-bin/carddisp.pl?gene=JUN |
| ACAN | Aggrecan | Protein Coding | 46 | GC15P118102 | 16.27408 | https://www.genecards.org/cgi-bin/carddisp.pl?gene=ACAN |
| OSM | Oncostatin M | Protein Coding | 41 | GC22M030262 | 16.26777 | https://www.genecards.org/cgi-bin/carddisp.pl?gene=OSM |
| ADAMTS4 | ADAM Metallopeptidase With Thrombospondin Type 1 Motif 4 | Protein Coding | 42 | GC01M161184 | 16.23673 | https://www.genecards.org/cgi-bin/carddisp.pl?gene=ADAMTS4 |
| IL22 | Interleukin 22 | Protein Coding | 40 | GC12M068248 | 16.14384 | https://www.genecards.org/cgi-bin/carddisp.pl?gene=IL22 |
| PHF19 | PHD Finger Protein 19 | Protein Coding | 38 | GC09M120855 | 16.09995 | https://www.genecards.org/cgi-bin/carddisp.pl?gene=PHF19 |
| TRAF6 | TNF Receptor Associated Factor 6 | Protein Coding | 44 | GC11M036467 | 15.98972 | https://www.genecards.org/cgi-bin/carddisp.pl?gene=TRAF6 |
| RIPK1 | Receptor Interacting Serine/Threonine Kinase 1 | Protein Coding | 46 | GC06P003275 | 15.95637 | https://www.genecards.org/cgi-bin/carddisp.pl?gene=RIPK1 |
| AP4B1-AS1 | AP4B1 Antisense RNA 1 | RNA Gene | 14 | GC01P113813 | 15.88724 | https://www.genecards.org/cgi-bin/carddisp.pl?gene=AP4B1-AS1 |

| | | | | | | |
|---|---|---|---|---|---|---|
| ITGB2 | Integrin Subunit Beta 2 | Protein Coding | 49 | GC21M 044885 | 15.84 304 | https://www.genecards.org/cgi-bin/carddisp.pl?gene=ITGB2 |
| RETN | Resistin | Protein Coding | 41 | GC19P 007669 | 15.84 085 | https://www.genecards.org/cgi-bin/carddisp.pl?gene=RETN |
| C4A | Complement C4A (Rodgers Blood Group) | Protein Coding | 43 | GC06P 083718 | 15.77 849 | https://www.genecards.org/cgi-bin/carddisp.pl?gene=C4A |
| BMP6 | Bone Morphogenetic Protein 6 | Protein Coding | 42 | GC06P 007726 | 15.76 888 | https://www.genecards.org/cgi-bin/carddisp.pl?gene=BMP6 |
| MAPK8 | Mitogen-Activated Protein Kinase 8 | Protein Coding | 47 | GC10P 048306 | 15.74 175 | https://www.genecards.org/cgi-bin/carddisp.pl?gene=MAPK8 |
| CTSK | Cathepsin K | Protein Coding | 49 | GC01M 152112 | 15.65 356 | https://www.genecards.org/cgi-bin/carddisp.pl?gene=CTSK |
| DHODH | Dihydroorotate Dehydrogenase (Quinone) | Protein Coding | 45 | GC16P 072008 | 15.58 341 | https://www.genecards.org/cgi-bin/carddisp.pl?gene=DHODH |
| HLA-DMB | Major Histocompatibility Complex, Class II, DM Beta | Protein Coding | 40 | GC06M 032934 | 15.56 456 | https://www.genecards.org/cgi-bin/carddisp.pl?gene=HLA-DMB |
| APOH | Apolipoprotein H | Protein Coding | 43 | GC17M 066212 | 15.56 3 | https://www.genecards.org/cgi-bin/carddisp.pl?gene=APOH |
| CSF1R | Colony Stimulating Factor 1 Receptor | Protein Coding | 49 | GC05M 150053 | 15.54 609 | https://www.genecards.org/cgi-bin/carddisp.pl?gene=CSF1R |
| FOLR2 | Folate Receptor Beta | Protein Coding | 41 | GC11P 072216 | 15.53 863 | https://www.genecards.org/cgi-bin/carddisp.pl?gene=FOLR2 |
| CXCL1 | C-X-C Motif Chemokine Ligand 1 | Protein Coding | 40 | GC04P 073869 | 15.52 249 | https://www.genecards.org/cgi-bin/carddisp.pl?gene=CXCL1 |
| PADI2 | Peptidyl Arginine Deiminase 2 | Protein Coding | 41 | GC01M 017066 | 15.46 547 | https://www.genecards.org/cgi-bin/carddisp.pl?gene=PADI2 |
| CLEC16 A | C-Type Lectin Domain Containing 16A | Protein Coding | 36 | GC16P 010944 | 15.43 327 | https://www.genecards.org/cgi- |

| | | | | | | |
|---|---|---|---|---|---|---|
| | | | | | | bin/carddisp.pl?gene=CLEC16A |
| CXCR4 | C-X-C Motif Chemokine Receptor 4 | Protein Coding | 49 | GC02M136114 | 15.41358 | https://www.genecards.org/cgi-bin/carddisp.pl?gene=CXCR4 |
| CCL20 | C-C Motif Chemokine Ligand 20 | Protein Coding | 41 | GC02P227842 | 15.30789 | https://www.genecards.org/cgi-bin/carddisp.pl?gene=CCL20 |
| CR1 | Complement C3b/C4b Receptor 1 (Knops Blood Group) | Protein Coding | 45 | GC01P207496 | 15.30057 | https://www.genecards.org/cgi-bin/carddisp.pl?gene=CR1 |
| NR4A2 | Nuclear Receptor Subfamily 4 Group A Member 2 | Protein Coding | 45 | GC02M156324 | 15.28979 | https://www.genecards.org/cgi-bin/carddisp.pl?gene=NR4A2 |
| NAMPT | Nicotinamide Phosphoribosyltransferase | Protein Coding | 46 | GC07M106248 | 15.26194 | https://www.genecards.org/cgi-bin/carddisp.pl?gene=NAMPT |
| BTLA | B And T Lymphocyte Associated | Protein Coding | 38 | GC03M112463 | 15.24469 | https://www.genecards.org/cgi-bin/carddisp.pl?gene=BTLA |
| ANXA5 | Annexin A5 | Protein Coding | 44 | GC04M121667 | 15.18699 | https://www.genecards.org/cgi-bin/carddisp.pl?gene=ANXA5 |
| PLA2G2A | Phospholipase A2 Group IIA | Protein Coding | 45 | GC01M019975 | 15.15402 | https://www.genecards.org/cgi-bin/carddisp.pl?gene=PLA2G2A |
| NR3C1 | Nuclear Receptor Subfamily 3 Group C Member 1 | Protein Coding | 48 | GC05M143277 | 15.1537 | https://www.genecards.org/cgi-bin/carddisp.pl?gene=NR3C1 |
| CCRL2 | C-C Motif Chemokine Receptor Like 2 | Protein Coding | 38 | GC03P046407 | 15.15227 | https://www.genecards.org/cgi-bin/carddisp.pl?gene=CCRL2 |
| ELANE | Elastase, Neutrophil Expressed | Protein Coding | 47 | GC19P002810 | 15.10468 | https://www.genecards.org/cgi-bin/carddisp.pl?gene=ELANE |
| HFE | Homeostatic Iron Regulator | Protein Coding | 44 | GC06P026087 | 15.09504 | https://www.genecards.org/cgi-bin/carddisp.pl?gene=HFE |
| DDX39B | DExD-Box Helicase 39B | Protein Coding | 38 | GC06M031530 | 15.08054 | https://www.genecards.org/cgi-bin/carddisp.pl?gene=DDX39B |

| | | | | | | |
|---|---|---|---|---|---|---|
| CRH | Corticotropin Releasing Hormone | Protein Coding | 42 | GC08M066176 | 15.02802 | https://www.genecards.org/cgi-bin/carddisp.pl?gene=CRH |
| CD69 | CD69 Molecule | Protein Coding | 40 | GC12M021100 | 15.0032 | https://www.genecards.org/cgi-bin/carddisp.pl?gene=CD69 |
| IL11 | Interleukin 11 | Protein Coding | 39 | GC19M055364 | 14.99198 | https://www.genecards.org/cgi-bin/carddisp.pl?gene=IL11 |
| PPP1CC | Protein Phosphatase 1 Catalytic Subunit Gamma | Protein Coding | 43 | GC12M110709 | 14.94761 | https://www.genecards.org/cgi-bin/carddisp.pl?gene=PPP1CC |
| TIMP2 | TIMP Metallopeptidase Inhibitor 2 | Protein Coding | 41 | GC17M078852 | 14.94626 | https://www.genecards.org/cgi-bin/carddisp.pl?gene=TIMP2 |
| IL16 | Interleukin 16 | Protein Coding | 40 | GC15P081159 | 14.94118 | https://www.genecards.org/cgi-bin/carddisp.pl?gene=IL16 |
| CD4 | CD4 Molecule | Protein Coding | 48 | GC12P006786 | 14.94051 | https://www.genecards.org/cgi-bin/carddisp.pl?gene=CD4 |
| CILP | Cartilage Intermediate Layer Protein | Protein Coding | 41 | GC15M065194 | 14.93618 | https://www.genecards.org/cgi-bin/carddisp.pl?gene=CILP |
| SAA4 | Serum Amyloid A4, Constitutive | Protein Coding | 38 | GC11M018234 | 14.90598 | https://www.genecards.org/cgi-bin/carddisp.pl?gene=SAA4 |
| CCR7 | C-C Motif Chemokine Receptor 7 | Protein Coding | 42 | GC17M042422 | 14.88598 | https://www.genecards.org/cgi-bin/carddisp.pl?gene=CCR7 |
| BST1 | Bone Marrow Stromal Cell Antigen 1 | Protein Coding | 41 | GC04P018192 | 14.84058 | https://www.genecards.org/cgi-bin/carddisp.pl?gene=BST1 |
| CX3CR1 | C-X3-C Motif Chemokine Receptor 1 | Protein Coding | 42 | GC03M039279 | 14.82107 | https://www.genecards.org/cgi-bin/carddisp.pl?gene=CX3CR1 |
| CCN6 | Cellular Communication Network Factor 6 | Protein Coding | 33 | GC06P112053 | 14.79699 | https://www.genecards.org/cgi-bin/carddisp.pl?gene=CCN6 |
| ERAP1 | Endoplasmic Reticulum Aminopeptidase 1 | Protein Coding | 44 | GC05M096760 | 14.75821 | https://www.genecards.org/cgi- |

| | | | | | | |
|---|---|---|---|---|---|---|
| | | | | | | bin/carddisp.pl?gene=ERAP1 |
| CX3CL1 | C-X3-C Motif Chemokine Ligand 1 | Protein Coding | 41 | GC16P057372 | 14.75633 | https://www.genecards.org/cgi-bin/carddisp.pl?gene=CX3CL1 |
| ANXA1 | Annexin A1 | Protein Coding | 46 | GC09P073151 | 14.74983 | https://www.genecards.org/cgi-bin/carddisp.pl?gene=ANXA1 |
| TNFRSF6B | TNF Receptor Superfamily Member 6b | Protein Coding | 41 | GC20P063696 | 14.70909 | https://www.genecards.org/cgi-bin/carddisp.pl?gene=TNFRSF6B |
| MAP3K8 | Mitogen-Activated Protein Kinase Kinase Kinase 8 | Protein Coding | 44 | GC10P030510 | 14.62116 | https://www.genecards.org/cgi-bin/carddisp.pl?gene=MAP3K8 |
| LRRK2 | Leucine Rich Repeat Kinase 2 | Protein Coding | 47 | GC12P040196 | 14.61646 | https://www.genecards.org/cgi-bin/carddisp.pl?gene=LRRK2 |
| CCL4 | C-C Motif Chemokine Ligand 4 | Protein Coding | 37 | GC17P036103 | 14.42948 | https://www.genecards.org/cgi-bin/carddisp.pl?gene=CCL4 |
| ALOX5 | Arachidonate 5-Lipoxygenase | Protein Coding | 46 | GC10P045374 | 14.36469 | https://www.genecards.org/cgi-bin/carddisp.pl?gene=ALOX5 |
| TNFRSF10A | TNF Receptor Superfamily Member 10a | Protein Coding | 43 | GC08M023190 | 14.36466 | https://www.genecards.org/cgi-bin/carddisp.pl?gene=TNFRSF10A |
| ANKH | ANKH Inorganic Pyrophosphate Transport Regulator | Protein Coding | 41 | GC05M014720 | 14.35605 | https://www.genecards.org/cgi-bin/carddisp.pl?gene=ANKH |
| CXCR5 | C-X-C Motif Chemokine Receptor 5 | Protein Coding | 38 | GC11P118924 | 14.33774 | https://www.genecards.org/cgi-bin/carddisp.pl?gene=CXCR5 |
| NFKB1 | Nuclear Factor Kappa B Subunit 1 | Protein Coding | 49 | GC04P102501 | 14.24818 | https://www.genecards.org/cgi-bin/carddisp.pl?gene=NFKB1 |
| CXCL9 | C-X-C Motif Chemokine Ligand 9 | Protein Coding | 37 | GC04M076001 | 14.21463 | https://www.genecards.org/cgi-bin/carddisp.pl?gene=CXCL9 |
| SH2D1A | SH2 Domain Containing 1A | Protein Coding | 44 | GC0XP124227 | 14.19325 | https://www.genecards.org/cgi-bin/carddisp.pl?gene=SH2D1A |

| | | | | | | |
|---|---|---|---|---|---|---|
| PRRC2A | Proline Rich Coiled-Coil 2A | Protein Coding | 33 | GC06P 083697 | 14.17 116 | https://www.genecards .org/cgi-bin/carddisp.pl?gene= PRRC2A |
| CD86 | CD86 Molecule | Protein Coding | 42 | GC03P 122055 | 14.17 063 | https://www.genecards .org/cgi-bin/carddisp.pl?gene= CD86 |
| DEK | DEK Proto-Oncogene | Protein Coding | 40 | GC06M 018224 | 14.14 616 | https://www.genecards .org/cgi-bin/carddisp.pl?gene= DEK |
| IL32 | Interleukin 32 | Protein Coding | 37 | GC16P 011732 | 14.11 727 | https://www.genecards .org/cgi-bin/carddisp.pl?gene=I L32 |
| SELL | Selectin L | Protein Coding | 42 | GC01M 169690 | 14.06 612 | https://www.genecards .org/cgi-bin/carddisp.pl?gene= SELL |
| IL18R1 | Interleukin 18 Receptor 1 | Protein Coding | 41 | GC02P 102311 | 14.04 528 | https://www.genecards .org/cgi-bin/carddisp.pl?gene=I L18R1 |
| ENO1 | Enolase 1 | Protein Coding | 45 | GC01M 008861 | 14.01 664 | https://www.genecards .org/cgi-bin/carddisp.pl?gene= ENO1 |
| LIF | LIF Interleukin 6 Family Cytokine | Protein Coding | 40 | GC22M 030240 | 13.95 521 | https://www.genecards .org/cgi-bin/carddisp.pl?gene= LIF |
| CTSB | Cathepsin B | Protein Coding | 50 | GC08M 011842 | 13.92 418 | https://www.genecards .org/cgi-bin/carddisp.pl?gene= CTSB |
| CXCL5 | C-X-C Motif Chemokine Ligand 5 | Protein Coding | 39 | GC04M 073995 | 13.89 449 | https://www.genecards .org/cgi-bin/carddisp.pl?gene= CXCL5 |
| CAST | Calpastatin | Protein Coding | 44 | GC05P 096525 | 13.80 69 | https://www.genecards .org/cgi-bin/carddisp.pl?gene= CAST |
| IFNGR1 | Interferon Gamma Receptor 1 | Protein Coding | 48 | GC06M 137197 | 13.67 502 | https://www.genecards .org/cgi-bin/carddisp.pl?gene=I FNGR1 |
| HTRA1 | HtrA Serine Peptidase 1 | Protein Coding | 44 | GC10P 122461 | 13.67 453 | https://www.genecards .org/cgi-bin/carddisp.pl?gene= HTRA1 |
| SYVN1 | Synoviolin 1 | Protein Coding | 40 | GC11M 089758 | 13.67 208 | https://www.genecards .org/cgi- |

| | | | | | | |
|---|---|---|---|---|---|---|
| | | | | | | bin/carddisp.pl?gene=SYVN1 |
| GNAQ | G Protein Subunit Alpha Q | Protein Coding | 48 | GC09M077716 | 13.6626 | https://www.genecards.org/cgi-bin/carddisp.pl?gene=GNAQ |
| CTSG | Cathepsin G | Protein Coding | 44 | GC14M024573 | 13.63105 | https://www.genecards.org/cgi-bin/carddisp.pl?gene=CTSG |
| MMP14 | Matrix Metallopeptidase 14 | Protein Coding | 49 | GC14P032700 | 13.60407 | https://www.genecards.org/cgi-bin/carddisp.pl?gene=MMP14 |
| HAS1 | Hyaluronan Synthase 1 | Protein Coding | 38 | GC19M066232 | 13.36506 | https://www.genecards.org/cgi-bin/carddisp.pl?gene=HAS1 |
| CR2 | Complement C3d Receptor 2 | Protein Coding | 45 | GC01P207454 | 13.35006 | https://www.genecards.org/cgi-bin/carddisp.pl?gene=CR2 |
| TNFRSF9 | TNF Receptor Superfamily Member 9 | Protein Coding | 41 | GC01M007915 | 13.33525 | https://www.genecards.org/cgi-bin/carddisp.pl?gene=TNFRSF9 |
| SAA2 | Serum Amyloid A2 | Protein Coding | 34 | GC11M018238 | 13.30334 | https://www.genecards.org/cgi-bin/carddisp.pl?gene=SAA2 |
| ABCB1 | ATP Binding Cassette Subfamily B Member 1 | Protein Coding | 49 | GC07M087504 | 13.1671 | https://www.genecards.org/cgi-bin/carddisp.pl?gene=ABCB1 |
| IKBKB | Inhibitor Of Nuclear Factor Kappa B Kinase Subunit Beta | Protein Coding | 50 | GC08P042271 | 13.14229 | https://www.genecards.org/cgi-bin/carddisp.pl?gene=IKBKB |
| SRF | Serum Response Factor | Protein Coding | 41 | GC06P043171 | 13.09601 | https://www.genecards.org/cgi-bin/carddisp.pl?gene=SRF |
| NPSR1 | Neuropeptide S Receptor 1 | Protein Coding | 40 | GC07P034664 | 13.078 | https://www.genecards.org/cgi-bin/carddisp.pl?gene=NPSR1 |
| ITGAL | Integrin Subunit Alpha L | Protein Coding | 45 | GC16P030472 | 13.05874 | https://www.genecards.org/cgi-bin/carddisp.pl?gene=ITGAL |
| LTBR | Lymphotoxin Beta Receptor | Protein Coding | 40 | GC12P006375 | 13.0238 | https://www.genecards.org/cgi-bin/carddisp.pl?gene=LTBR |

| | | | | | | |
|---|---|---|---|---|---|---|
| IL18BP | Interleukin 18 Binding Protein | Protein Coding | 40 | GC11P 071998 | 13.02 169 | https://www.genecards .org/cgi-bin/carddisp.pl?gene=I L18BP |
| JAM3 | Junctional Adhesion Molecule 3 | Protein Coding | 42 | GC11P 134068 | 12.99 21 | https://www.genecards .org/cgi-bin/carddisp.pl?gene=J AM3 |
| PRSS2 | Serine Protease 2 | Protein Coding | 39 | GC07P 148358 | 12.98 954 | https://www.genecards .org/cgi-bin/carddisp.pl?gene= PRSS2 |
| HGD | Homogentisate 1,2-Dioxygenase | Protein Coding | 42 | GC03M 120628 | 12.93 446 | https://www.genecards .org/cgi-bin/carddisp.pl?gene= HGD |
| TNFSF14 | TNF Superfamily Member 14 | Protein Coding | 40 | GC19M 006663 | 12.90 687 | https://www.genecards .org/cgi-bin/carddisp.pl?gene= TNFSF14 |
| CDH11 | Cadherin 11 | Protein Coding | 46 | GC16M 064943 | 12.90 221 | https://www.genecards .org/cgi-bin/carddisp.pl?gene= CDH11 |
| LECT2 | Leukocyte Cell Derived Chemotaxin 2 | Protein Coding | 35 | GC05M 135922 | 12.90 004 | https://www.genecards .org/cgi-bin/carddisp.pl?gene= LECT2 |
| IL12B | Interleukin 12B | Protein Coding | 42 | GC05M 159314 | 12.84 875 | https://www.genecards .org/cgi-bin/carddisp.pl?gene=I L12B |
| H19 | H19 Imprinted Maternally Expressed Transcript | RNA Gene | 28 | GC11M 001995 | 12.76 628 | https://www.genecards .org/cgi-bin/carddisp.pl?gene= H19 |
| PRKCD | Protein Kinase C Delta | Protein Coding | 50 | GC03P 053156 | 12.68 714 | https://www.genecards .org/cgi-bin/carddisp.pl?gene= PRKCD |
| HOTAIR | HOX Transcript Antisense RNA | RNA Gene | 25 | GC12M 053962 | 12.66 906 | https://www.genecards .org/cgi-bin/carddisp.pl?gene= HOTAIR |
| IRAK1 | Interleukin 1 Receptor Associated Kinase 1 | Protein Coding | 47 | GC0X M1540 10 | 12.56 267 | https://www.genecards .org/cgi-bin/carddisp.pl?gene=I RAK1 |
| SIGLEC1 | Sialic Acid Binding Ig Like Lectin 1 | Protein Coding | 40 | GC20M 003686 | 12.41 731 | https://www.genecards .org/cgi-bin/carddisp.pl?gene= SIGLEC1 |
| ZNF334 | Zinc Finger Protein 334 | Protein Coding | 34 | GC20M 046462 | 12.35 258 | https://www.genecards .org/cgi- |

| | | | | | | |
|---|---|---|---|---|---|---|
| | | | | | | bin/carddisp.pl?gene=ZNF334 |
| IL36RN | Interleukin 36 Receptor Antagonist | Protein Coding | 39 | GC02P122459 | 12.33298 | https://www.genecards.org/cgi-bin/carddisp.pl?gene=IL36RN |
| PLB1 | Phospholipase B1 | Protein Coding | 36 | GC02P028460 | 12.27903 | https://www.genecards.org/cgi-bin/carddisp.pl?gene=PLB1 |
| PLA2G10 | Phospholipase A2 Group X | Protein Coding | 42 | GC16M014672 | 12.27668 | https://www.genecards.org/cgi-bin/carddisp.pl?gene=PLA2G10 |
| VDR | Vitamin D Receptor | Protein Coding | 49 | GC12M047841 | 12.25633 | https://www.genecards.org/cgi-bin/carddisp.pl?gene=VDR |
| TAP2 | Transporter 2, ATP Binding Cassette Subfamily B Member | Protein Coding | 43 | GC06M032821 | 12.21779 | https://www.genecards.org/cgi-bin/carddisp.pl?gene=TAP2 |
| TNFRSF4 | TNF Receptor Superfamily Member 4 | Protein Coding | 40 | GC01M001211 | 12.18949 | https://www.genecards.org/cgi-bin/carddisp.pl?gene=TNFRSF4 |
| IL26 | Interleukin 26 | Protein Coding | 34 | GC12M068201 | 12.08641 | https://www.genecards.org/cgi-bin/carddisp.pl?gene=IL26 |
| IL22RA1 | Interleukin 22 Receptor Subunit Alpha 1 | Protein Coding | 38 | GC01M024119 | 11.95796 | https://www.genecards.org/cgi-bin/carddisp.pl?gene=IL22RA1 |
| HLA-A | Major Histocompatibility Complex, Class I, A | Protein Coding | 45 | GC06P083654 | 11.8462 | https://www.genecards.org/cgi-bin/carddisp.pl?gene=HLA-A |
| IL1RAP | Interleukin 1 Receptor Accessory Protein | Protein Coding | 43 | GC03P190514 | 11.84372 | https://www.genecards.org/cgi-bin/carddisp.pl?gene=IL1RAP |
| HAPLN1 | Hyaluronan And Proteoglycan Link Protein 1 | Protein Coding | 41 | GC05M083637 | 11.79408 | https://www.genecards.org/cgi-bin/carddisp.pl?gene=HAPLN1 |
| IL17B | Interleukin 17B | Protein Coding | 38 | GC05M149371 | 11.7879 | https://www.genecards.org/cgi-bin/carddisp.pl?gene=IL17B |
| CSF3 | Colony Stimulating Factor 3 | Protein Coding | 38 | GC17P040015 | 11.78711 | https://www.genecards.org/cgi-bin/carddisp.pl?gene=CSF3 |

| | | | | | | |
|---|---|---|---|---|---|---|
| SUPT20H | SPT20 Homolog, SAGA Complex Component | Protein Coding | 35 | GC13M 037009 | 11.74 313 | https://www.genecards .org/cgi-bin/carddisp.pl?gene= SUPT20H |
| MUC1 | Mucin 1, Cell Surface Associated | Protein Coding | 47 | GC01M 155185 | 11.64 341 | https://www.genecards .org/cgi-bin/carddisp.pl?gene= MUC1 |
| GCH1 | GTP Cyclohydrolase 1 | Protein Coding | 46 | GC14M 054842 | 11.55 303 | https://www.genecards .org/cgi-bin/carddisp.pl?gene= GCH1 |
| C5 | Complement C5 | Protein Coding | 45 | GC09M 120933 | 11.51 311 | https://www.genecards .org/cgi-bin/carddisp.pl?gene= C5 |
| MALAT1 | Metastasis Associated Lung Adenocarcinoma Transcript 1 | RNA Gene | 24 | GC11P 069984 | 11.41 199 | https://www.genecards .org/cgi-bin/carddisp.pl?gene= MALAT1 |
| C4B | Complement C4B (Chido Blood Group) | Protein Coding | 43 | GC06P 032014 | 11.37 74 | https://www.genecards .org/cgi-bin/carddisp.pl?gene= C4B |
| IL12A | Interleukin 12A | Protein Coding | 42 | GC03P 159988 | 11.37 363 | https://www.genecards .org/cgi-bin/carddisp.pl?gene=I L12A |
| CCL18 | C-C Motif Chemokine Ligand 18 | Protein Coding | 34 | GC17P 036064 | 11.30 127 | https://www.genecards .org/cgi-bin/carddisp.pl?gene= CCL18 |
| CASP10 | Caspase 10 | Protein Coding | 46 | GC02P 201182 | 11.29 622 | https://www.genecards .org/cgi-bin/carddisp.pl?gene= CASP10 |
| CCR3 | C-C Motif Chemokine Receptor 3 | Protein Coding | 44 | GC03P 046771 | 11.23 719 | https://www.genecards .org/cgi-bin/carddisp.pl?gene= CCR3 |
| ELMO2 | Engulfment And Cell Motility 2 | Protein Coding | 41 | GC20M 046366 | 11.21 355 | https://www.genecards .org/cgi-bin/carddisp.pl?gene= ELMO2 |
| CALCA | Calcitonin Related Polypeptide Alpha | Protein Coding | 42 | GC11M 014945 | 11.21 201 | https://www.genecards .org/cgi-bin/carddisp.pl?gene= CALCA |
| RASGRP 1 | RAS Guanyl Releasing Protein 1 | Protein Coding | 46 | GC15M 038488 | 11.18 871 | https://www.genecards .org/cgi-bin/carddisp.pl?gene= RASGRP1 |
| SFTPD | Surfactant Protein D | Protein Coding | 42 | GC10M 079937 | 11.13 456 | https://www.genecards .org/cgi- |

| | | | | | | |
|---|---|---|---|---|---|---|
| | | | | | | bin/carddisp.pl?gene=SFTPD |
| PSMB9 | Proteasome 20S Subunit Beta 9 | Protein Coding | 45 | GC06P083730 | 11.08946 | https://www.genecards.org/cgi-bin/carddisp.pl?gene=PSMB9 |
| MVK | Mevalonate Kinase | Protein Coding | 46 | GC12P109573 | 11.01072 | https://www.genecards.org/cgi-bin/carddisp.pl?gene=MVK |
| WAS | WASP Actin Nucleation Promoting Factor | Protein Coding | 46 | GC0XP048676 | 11.00922 | https://www.genecards.org/cgi-bin/carddisp.pl?gene=WAS |
| IL17F | Interleukin 17F | Protein Coding | 41 | GC06M066315 | 10.98434 | https://www.genecards.org/cgi-bin/carddisp.pl?gene=IL17F |
| ZFAS1 | ZNFX1 Antisense RNA 1 | RNA Gene | 20 | GC20P049276 | 10.97871 | https://www.genecards.org/cgi-bin/carddisp.pl?gene=ZFAS1 |
| SNHG29 | Small Nucleolar RNA Host Gene 29 | RNA Gene | 18 | GC17P017038 | 10.97871 | https://www.genecards.org/cgi-bin/carddisp.pl?gene=SNHG29 |
| GSTM1 | Glutathione S-Transferase Mu 1 | Protein Coding | 40 | GC01P109687 | 10.92024 | https://www.genecards.org/cgi-bin/carddisp.pl?gene=GSTM1 |
| CCL21 | C-C Motif Chemokine Ligand 21 | Protein Coding | 40 | GC09M034709 | 10.91666 | https://www.genecards.org/cgi-bin/carddisp.pl?gene=CCL21 |
| GZMB | Granzyme B | Protein Coding | 45 | GC14M024630 | 10.86115 | https://www.genecards.org/cgi-bin/carddisp.pl?gene=GZMB |
| INS | Insulin | Protein Coding | 45 | GC11M002159 | 10.84706 | https://www.genecards.org/cgi-bin/carddisp.pl?gene=INS |
| MMP7 | Matrix Metallopeptidase 7 | Protein Coding | 46 | GC11M102425 | 10.82843 | https://www.genecards.org/cgi-bin/carddisp.pl?gene=MMP7 |
| UCA1 | Urothelial Cancer Associated 1 | RNA Gene | 22 | GC19P015828 | 10.79663 | https://www.genecards.org/cgi-bin/carddisp.pl?gene=UCA1 |
| IGKC | Immunoglobulin Kappa Constant | Protein Coding | 34 | GC02M090885 | 10.79204 | https://www.genecards.org/cgi-bin/carddisp.pl?gene=IGKC |

| | | | | | | |
|---|---|---|---|---|---|---|
| IL33 | Interleukin 33 | Protein Coding | 37 | GC09P 006572 | 10.77 691 | https://www.genecards .org/cgi-bin/carddisp.pl?gene=I L33 |
| RAG2 | Recombination Activating 2 | Protein Coding | 41 | GC11M 036575 | 10.72 379 | https://www.genecards .org/cgi-bin/carddisp.pl?gene= RAG2 |
| PIK3CD | Phosphatidylinositol-4,5-Bisphosphate 3-Kinase Catalytic Subunit Delta | Protein Coding | 51 | GC01P 009629 | 10.71 171 | https://www.genecards .org/cgi-bin/carddisp.pl?gene= PIK3CD |
| HMGB2 | High Mobility Group Box 2 | Protein Coding | 40 | GC04M 173331 | 10.70 827 | https://www.genecards .org/cgi-bin/carddisp.pl?gene= HMGB2 |
| TLR3 | Toll Like Receptor 3 | Protein Coding | 49 | GC04P 186059 | 10.48 099 | https://www.genecards .org/cgi-bin/carddisp.pl?gene= TLR3 |
| STAT1 | Signal Transducer And Activator Of Transcription 1 | Protein Coding | 50 | GC02M 190908 | 10.46 094 | https://www.genecards .org/cgi-bin/carddisp.pl?gene= STAT1 |
| ICOS | Inducible T Cell Costimulator | Protein Coding | 42 | GC02P 203937 | 10.37 501 | https://www.genecards .org/cgi-bin/carddisp.pl?gene=I COS |
| B2M | Beta-2-Microglobulin | Protein Coding | 46 | GC15P 044711 | 10.33 602 | https://www.genecards .org/cgi-bin/carddisp.pl?gene= B2M |
| TPMT | Thiopurine S-Methyltransferase | Protein Coding | 45 | GC06M 018128 | 10.27 546 | https://www.genecards .org/cgi-bin/carddisp.pl?gene= TPMT |
| MYD88 | MYD88 Innate Immune Signal Transduction Adaptor | Protein Coding | 48 | GC03P 038139 | 10.18 017 | https://www.genecards .org/cgi-bin/carddisp.pl?gene= MYD88 |
| BTK | Bruton Tyrosine Kinase | Protein Coding | 50 | GC0X M1013 49 | 10.14 62 | https://www.genecards .org/cgi-bin/carddisp.pl?gene= BTK |
| TP53 | Tumor Protein P53 | Protein Coding | 50 | GC17M 007661 | 10.10 886 | https://www.genecards .org/cgi-bin/carddisp.pl?gene= TP53 |
| HPRT1 | Hypoxanthine Phosphoribosyltransferase 1 | Protein Coding | 45 | GC0XP 134460 | 10.08 449 | https://www.genecards .org/cgi-bin/carddisp.pl?gene= HPRT1 |
| LTF | Lactotransferrin | Protein Coding | 44 | GC03M 046435 | 10.04 714 | https://www.genecards .org/cgi- |

| | | | | | | |
|---|---|---|---|---|---|---|
| | | | | | | bin/carddisp.pl?gene=LTF |
| PRL | Prolactin | Protein Coding | 41 | GC06M022287 | 9.99847 | https://www.genecards.org/cgi-bin/carddisp.pl?gene=PRL |
| ACTA2 | Actin Alpha 2, Smooth Muscle | Protein Coding | 45 | GC10M088935 | 9.997743 | https://www.genecards.org/cgi-bin/carddisp.pl?gene=ACTA2 |
| ANGPT2 | Angiopoietin 2 | Protein Coding | 45 | GC08M006499 | 9.826532 | https://www.genecards.org/cgi-bin/carddisp.pl?gene=ANGPT2 |
| VWF | Von Willebrand Factor | Protein Coding | 47 | GC12M005917 | 9.81198 | https://www.genecards.org/cgi-bin/carddisp.pl?gene=VWF |
| PTGES | Prostaglandin E Synthase | Protein Coding | 40 | GC09M129738 | 9.793758 | https://www.genecards.org/cgi-bin/carddisp.pl?gene=PTGES |
| ADA | Adenosine Deaminase | Protein Coding | 49 | GC20M044620 | 9.783412 | https://www.genecards.org/cgi-bin/carddisp.pl?gene=ADA |
| MMP19 | Matrix Metallopeptidase 19 | Protein Coding | 47 | GC12M055835 | 9.769567 | https://www.genecards.org/cgi-bin/carddisp.pl?gene=MMP19 |
| PTPRC | Protein Tyrosine Phosphatase Receptor Type C | Protein Coding | 49 | GC01P198607 | 9.747452 | https://www.genecards.org/cgi-bin/carddisp.pl?gene=PTPRC |
| SNHG28 | Small Nucleolar RNA Host Gene 28 | RNA Gene | 17 | GC01M159834 | 9.636161 | https://www.genecards.org/cgi-bin/carddisp.pl?gene=SNHG28 |
| IL3 | Interleukin 3 | Protein Coding | 41 | GC05P132060 | 9.605054 | https://www.genecards.org/cgi-bin/carddisp.pl?gene=IL3 |
| ACE | Angiotensin I Converting Enzyme | Protein Coding | 49 | GC17P063477 | 9.555857 | https://www.genecards.org/cgi-bin/carddisp.pl?gene=ACE |
| GPT | Glutamic--Pyruvic Transaminase | Protein Coding | 40 | GC08P144502 | 9.512686 | https://www.genecards.org/cgi-bin/carddisp.pl?gene=GPT |
| FBN1 | Fibrillin 1 | Protein Coding | 45 | GC15M048408 | 9.487437 | https://www.genecards.org/cgi-bin/carddisp.pl?gene=FBN1 |

| | | | | | | |
|---|---|---|---|---|---|---|
| ETS1 | ETS Proto-Oncogene 1, Transcription Factor | Protein Coding | 45 | GC11M 128458 | 9.442 19 | https://www.genecards .org/cgi-bin/carddisp.pl?gene= ETS1 |
| TNFSF4 | TNF Superfamily Member 4 | Protein Coding | 39 | GC01M 173183 | 9.435 301 | https://www.genecards .org/cgi-bin/carddisp.pl?gene= TNFSF4 |
| TREX1 | Three Prime Repair Exonuclease 1 | Protein Coding | 40 | GC03P 048977 | 9.427 769 | https://www.genecards .org/cgi-bin/carddisp.pl?gene= TREX1 |
| CFI | Complement Factor I | Protein Coding | 45 | GC04M 109732 | 9.424 962 | https://www.genecards .org/cgi-bin/carddisp.pl?gene= CFI |
| CXCL13 | C-X-C Motif Chemokine Ligand 13 | Protein Coding | 40 | GC04P 077511 | 9.318 037 | https://www.genecards .org/cgi-bin/carddisp.pl?gene= CXCL13 |
| CD80 | CD80 Molecule | Protein Coding | 40 | GC03M 119524 | 9.295 761 | https://www.genecards .org/cgi-bin/carddisp.pl?gene= CD80 |
| HAMP | Hepcidin Antimicrobial Peptide | Protein Coding | 42 | GC19P 066535 | 9.292 628 | https://www.genecards .org/cgi-bin/carddisp.pl?gene= HAMP |
| TAP1 | Transporter 1, ATP Binding Cassette Subfamily B Member | Protein Coding | 45 | GC06M 066023 | 9.286 879 | https://www.genecards .org/cgi-bin/carddisp.pl?gene= TAP1 |
| DNASE1 | Deoxyribonuclease 1 | Protein Coding | 42 | GC16P 003611 | 9.285 975 | https://www.genecards .org/cgi-bin/carddisp.pl?gene= DNASE1 |
| MYC | MYC Proto-Oncogene, BHLH Transcription Factor | Protein Coding | 49 | GC08P 127735 | 9.263 966 | https://www.genecards .org/cgi-bin/carddisp.pl?gene= MYC |
| ICOSLG | Inducible T Cell Costimulator Ligand | Protein Coding | 39 | GC21M 044222 | 9.258 209 | https://www.genecards .org/cgi-bin/carddisp.pl?gene=I COSLG |
| NLRP12 | NLR Family Pyrin Domain Containing 12 | Protein Coding | 42 | GC19M 053793 | 9.176 813 | https://www.genecards .org/cgi-bin/carddisp.pl?gene= NLRP12 |
| BIRC5 | Baculoviral IAP Repeat Containing 5 | Protein Coding | 45 | GC17P 078214 | 9.157 294 | https://www.genecards .org/cgi-bin/carddisp.pl?gene= BIRC5 |
| DNASE1 L3 | Deoxyribonuclease 1 Like 3 | Protein Coding | 42 | GC03M 058192 | 9.154 231 | https://www.genecards .org/cgi- |

| | | | | | | |
|---|---|---|---|---|---|---|
| | | | | | | bin/carddisp.pl?gene=DNASE1L3 |
| TNFSF12 | TNF Superfamily Member 12 | Protein Coding | 39 | GC17P 011166 | 9.109 595 | https://www.genecards.org/cgi-bin/carddisp.pl?gene=TNFSF12 |
| FBXL19-AS1 | FBXL19 Antisense RNA 1 | RNA Gene | 19 | GC16M 037214 | 9.105 168 | https://www.genecards.org/cgi-bin/carddisp.pl?gene=FBXL19-AS1 |
| CASP1 | Caspase 1 | Protein Coding | 48 | GC11M 105025 | 9.031 401 | https://www.genecards.org/cgi-bin/carddisp.pl?gene=CASP1 |
| MMP10 | Matrix Metallopeptidase 10 | Protein Coding | 44 | GC11M 102770 | 8.983 663 | https://www.genecards.org/cgi-bin/carddisp.pl?gene=MMP10 |
| TYMS | Thymidylate Synthetase | Protein Coding | 46 | GC18P 000657 | 8.978 648 | https://www.genecards.org/cgi-bin/carddisp.pl?gene=TYMS |
| TLR5 | Toll Like Receptor 5 | Protein Coding | 45 | GC01M 223109 | 8.953 194 | https://www.genecards.org/cgi-bin/carddisp.pl?gene=TLR5 |
| RELA | RELA Proto-Oncogene, NF-KB Subunit | Protein Coding | 49 | GC11M 065653 | 8.933 392 | https://www.genecards.org/cgi-bin/carddisp.pl?gene=RELA |
| PXK | PX Domain Containing Serine/Threonine Kinase Like | Protein Coding | 37 | GC03P 058333 | 8.857 019 | https://www.genecards.org/cgi-bin/carddisp.pl?gene=PXK |
| PON1 | Paraoxonase 1 | Protein Coding | 46 | GC07M 095297 | 8.855 175 | https://www.genecards.org/cgi-bin/carddisp.pl?gene=PON1 |
| SLC26A2 | Solute Carrier Family 26 Member 2 | Protein Coding | 44 | GC05P 149944 | 8.787 96 | https://www.genecards.org/cgi-bin/carddisp.pl?gene=SLC26A2 |
| IFIH1 | Interferon Induced With Helicase C Domain 1 | Protein Coding | 45 | GC02M 162267 | 8.767 211 | https://www.genecards.org/cgi-bin/carddisp.pl?gene=IFIH1 |
| CCN2 | Cellular Communication Network Factor 2 | Protein Coding | 37 | GC06M 131948 | 8.727 752 | https://www.genecards.org/cgi-bin/carddisp.pl?gene=CCN2 |
| BMP2 | Bone Morphogenetic Protein 2 | Protein Coding | 44 | GC20P 006767 | 8.629 303 | https://www.genecards.org/cgi-bin/carddisp.pl?gene=BMP2 |

| | | | | | | |
|---|---|---|---|---|---|---|
| AIRE | Autoimmune Regulator | Protein Coding | 44 | GC21P 044285 | 8.624 952 | https://www.genecards .org/cgi-bin/carddisp.pl?gene= AIRE |
| GHRL | Ghrelin And Obestatin Prepropeptide | Protein Coding | 42 | GC03M 010285 | 8.599 505 | https://www.genecards .org/cgi-bin/carddisp.pl?gene= GHRL |
| PRF1 | Perforin 1 | Protein Coding | 44 | GC10M 070597 | 8.597 99 | https://www.genecards .org/cgi-bin/carddisp.pl?gene= PRF1 |
| TRAF3IP 2 | TRAF3 Interacting Protein 2 | Protein Coding | 44 | GC06M 111555 | 8.525 12 | https://www.genecards .org/cgi-bin/carddisp.pl?gene= TRAF3IP2 |
| TF | Transferrin | Protein Coding | 48 | GC03P 134296 | 8.517 799 | https://www.genecards .org/cgi-bin/carddisp.pl?gene= TF |
| TNFRSF1 3B | TNF Receptor Superfamily Member 13B | Protein Coding | 44 | GC17M 016929 | 8.511 412 | https://www.genecards .org/cgi-bin/carddisp.pl?gene= TNFRSF13B |
| RAG1 | Recombination Activating 1 | Protein Coding | 45 | GC11P 036538 | 8.504 069 | https://www.genecards .org/cgi-bin/carddisp.pl?gene= RAG1 |
| PPARG | Peroxisome Proliferator Activated Receptor Gamma | Protein Coding | 50 | GC03P 012287 | 8.483 553 | https://www.genecards .org/cgi-bin/carddisp.pl?gene= PPARG |
| KIT | KIT Proto-Oncogene, Receptor Tyrosine Kinase | Protein Coding | 50 | GC04P 054657 | 8.483 397 | https://www.genecards .org/cgi-bin/carddisp.pl?gene= KIT |
| MIF-AS1 | MIF Antisense RNA 1 | RNA Gene | 15 | GC22M 023893 | 8.450 796 | https://www.genecards .org/cgi-bin/carddisp.pl?gene= MIF-AS1 |
| CD58 | CD58 Molecule | Protein Coding | 39 | GC01M 116514 | 8.422 945 | https://www.genecards .org/cgi-bin/carddisp.pl?gene= CD58 |
| ERAP2 | Endoplasmic Reticulum Aminopeptidase 2 | Protein Coding | 39 | GC05P 096875 | 8.396 812 | https://www.genecards .org/cgi-bin/carddisp.pl?gene= ERAP2 |
| COL11A 1 | Collagen Type XI Alpha 1 Chain | Protein Coding | 42 | GC01M 102876 | 8.360 456 | https://www.genecards .org/cgi-bin/carddisp.pl?gene= COL11A1 |
| PTH | Parathyroid Hormone | Protein Coding | 44 | GC11M 013492 | 8.356 497 | https://www.genecards .org/cgi- |

| | | | | | | |
|---|---|---|---|---|---|---|
| | | | | | | bin/carddisp.pl?gene=PTH |
| BANK1 | B Cell Scaffold Protein With Ankyrin Repeats 1 | Protein Coding | 37 | GC04P101411 | 8.329576 | https://www.genecards.org/cgi-bin/carddisp.pl?gene=BANK1 |
| HGF | Hepatocyte Growth Factor | Protein Coding | 49 | GC07M081699 | 8.308176 | https://www.genecards.org/cgi-bin/carddisp.pl?gene=HGF |
| BCL2 | BCL2 Apoptosis Regulator | Protein Coding | 48 | GC18M063123 | 8.289707 | https://www.genecards.org/cgi-bin/carddisp.pl?gene=BCL2 |
| TFR2 | Transferrin Receptor 2 | Protein Coding | 44 | GC07M100620 | 8.28105 | https://www.genecards.org/cgi-bin/carddisp.pl?gene=TFR2 |
| ENG | Endoglin | Protein Coding | 46 | GC09M127815 | 8.271666 | https://www.genecards.org/cgi-bin/carddisp.pl?gene=ENG |
| IRF4 | Interferon Regulatory Factor 4 | Protein Coding | 42 | GC06P000391 | 8.261472 | https://www.genecards.org/cgi-bin/carddisp.pl?gene=IRF4 |
| UBE2L3 | Ubiquitin Conjugating Enzyme E2 L3 | Protein Coding | 43 | GC22P021549 | 8.220354 | https://www.genecards.org/cgi-bin/carddisp.pl?gene=UBE2L3 |
| P4HA2 | Prolyl 4-Hydroxylase Subunit Alpha 2 | Protein Coding | 45 | GC05M132191 | 8.194843 | https://www.genecards.org/cgi-bin/carddisp.pl?gene=P4HA2 |
| TREM1 | Triggering Receptor Expressed On Myeloid Cells 1 | Protein Coding | 40 | GC06M041267 | 8.190091 | https://www.genecards.org/cgi-bin/carddisp.pl?gene=TREM1 |
| LRBA | LPS Responsive Beige-Like Anchor Protein | Protein Coding | 40 | GC04M150264 | 8.170541 | https://www.genecards.org/cgi-bin/carddisp.pl?gene=LRBA |
| LBR | Lamin B Receptor | Protein Coding | 45 | GC01M225401 | 8.169616 | https://www.genecards.org/cgi-bin/carddisp.pl?gene=LBR |
| LEP | Leptin | Protein Coding | 45 | GC07P128241 | 8.160183 | https://www.genecards.org/cgi-bin/carddisp.pl?gene=LEP |
| LCE3C | Late Cornified Envelope 3C | Protein Coding | 27 | GC01P152600 | 8.146751 | https://www.genecards.org/cgi-bin/carddisp.pl?gene=LCE3C |

| | | | | | | |
|---|---|---|---|---|---|---|
| FGFR1 | Fibroblast Growth Factor Receptor 1 | Protein Coding | 52 | GC08M 038400 | 8.140 184 | https://www.genecards .org/cgi-bin/carddisp.pl?gene= FGFR1 |
| CYP2C9 | Cytochrome P450 Family 2 Subfamily C Member 9 | Protein Coding | 46 | GC10P 094938 | 8.136 342 | https://www.genecards .org/cgi-bin/carddisp.pl?gene= CYP2C9 |
| LCE3B | Late Cornified Envelope 3B | Protein Coding | 25 | GC01P 152613 | 8.135 294 | https://www.genecards .org/cgi-bin/carddisp.pl?gene= LCE3B |
| MECP2 | Methyl-CpG Binding Protein 2 | Protein Coding | 44 | GC0X M1540 21 | 8.128 887 | https://www.genecards .org/cgi-bin/carddisp.pl?gene= MECP2 |
| PLA2G4 A | Phospholipase A2 Group IVA | Protein Coding | 47 | GC01P 186798 | 8.127 202 | https://www.genecards .org/cgi-bin/carddisp.pl?gene= PLA2G4A |
| IL6-AS1 | IL6 Antisense RNA 1 | RNA Gene | 12 | GC07M 022728 | 8.120 523 | https://www.genecards .org/cgi-bin/carddisp.pl?gene=I L6-AS1 |
| COL1A1 | Collagen Type I Alpha 1 Chain | Protein Coding | 48 | GC17M 050183 | 8.073 373 | https://www.genecards .org/cgi-bin/carddisp.pl?gene= COL1A1 |
| ESR1 | Estrogen Receptor 1 | Protein Coding | 50 | GC06P 151656 | 8.039 339 | https://www.genecards .org/cgi-bin/carddisp.pl?gene= ESR1 |
| GC | GC Vitamin D Binding Protein | Protein Coding | 44 | GC04M 071741 | 8.032 885 | https://www.genecards .org/cgi-bin/carddisp.pl?gene= GC |
| CD14 | CD14 Molecule | Protein Coding | 44 | GC05M 140631 | 8.009 14 | https://www.genecards .org/cgi-bin/carddisp.pl?gene= CD14 |
| IL4R | Interleukin 4 Receptor | Protein Coding | 46 | GC16P 027992 | 7.993 469 | https://www.genecards .org/cgi-bin/carddisp.pl?gene=I L4R |
| JAZF1 | JAZF Zinc Finger 1 | Protein Coding | 37 | GC07M 027830 | 7.976 355 | https://www.genecards .org/cgi-bin/carddisp.pl?gene=J AZF1 |
| PRG4 | Proteoglycan 4 | Protein Coding | 41 | GC01P 186296 | 7.910 367 | https://www.genecards .org/cgi-bin/carddisp.pl?gene= PRG4 |
| SMAD3 | SMAD Family Member 3 | Protein Coding | 48 | GC15P 067063 | 7.910 223 | https://www.genecards .org/cgi- |

| | | | | | | |
|---|---|---|---|---|---|---|
| | | | | | | bin/carddisp.pl?gene=SMAD3 |
| ADA2 | Adenosine Deaminase 2 | Protein Coding | 33 | GC22M017197 | 7.857287 | https://www.genecards.org/cgi-bin/carddisp.pl?gene=ADA2 |
| SERPINE1 | Serpin Family E Member 1 | Protein Coding | 48 | GC07P101127 | 7.806879 | https://www.genecards.org/cgi-bin/carddisp.pl?gene=SERPINE1 |
| DKK1 | Dickkopf WNT Signaling Pathway Inhibitor 1 | Protein Coding | 43 | GC10P052314 | 7.773871 | https://www.genecards.org/cgi-bin/carddisp.pl?gene=DKK1 |
| NFKB2 | Nuclear Factor Kappa B Subunit 2 | Protein Coding | 49 | GC10P102394 | 7.757963 | https://www.genecards.org/cgi-bin/carddisp.pl?gene=NFKB2 |
| HLA-DRA | Major Histocompatibility Complex, Class II, DR Alpha | Protein Coding | 45 | GC06P032439 | 7.756925 | https://www.genecards.org/cgi-bin/carddisp.pl?gene=HLA-DRA |
| HJV | Hemojuvelin BMP Co-Receptor | Protein Coding | 35 | GC01M146846 | 7.719653 | https://www.genecards.org/cgi-bin/carddisp.pl?gene=HJV |
| DPP4 | Dipeptidyl Peptidase 4 | Protein Coding | 48 | GC02M161992 | 7.705974 | https://www.genecards.org/cgi-bin/carddisp.pl?gene=DPP4 |
| IL5 | Interleukin 5 | Protein Coding | 43 | GC05M132541 | 7.693769 | https://www.genecards.org/cgi-bin/carddisp.pl?gene=IL5 |
| PRKCQ | Protein Kinase C Theta | Protein Coding | 47 | GC10M006526 | 7.68408 | https://www.genecards.org/cgi-bin/carddisp.pl?gene=PRKCQ |
| NAT2 | N-Acetyltransferase 2 | Protein Coding | 41 | GC08P018391 | 7.676551 | https://www.genecards.org/cgi-bin/carddisp.pl?gene=NAT2 |
| FGF2 | Fibroblast Growth Factor 2 | Protein Coding | 44 | GC04P122826 | 7.660335 | https://www.genecards.org/cgi-bin/carddisp.pl?gene=FGF2 |
| DCLRE1C | DNA Cross-Link Repair 1C | Protein Coding | 42 | GC10M014897 | 7.638009 | https://www.genecards.org/cgi-bin/carddisp.pl?gene=DCLRE1C |
| NOS3 | Nitric Oxide Synthase 3 | Protein Coding | 49 | GC07P150990 | 7.567809 | https://www.genecards.org/cgi-bin/carddisp.pl?gene=NOS3 |

| | | | | | | |
|---|---|---|---|---|---|---|
| MS4A1 | Membrane Spanning 4-Domains A1 | Protein Coding | 45 | GC11P060512 | 7.549659 | https://www.genecards.org/cgi-bin/carddisp.pl?gene=MS4A1 |
| HLA-G | Major Histocompatibility Complex, Class I, G | Protein Coding | 42 | GC06P083645 | 7.544229 | https://www.genecards.org/cgi-bin/carddisp.pl?gene=HLA-G |
| CCN1 | Cellular Communication Network Factor 1 | Protein Coding | 29 | GC01P085581 | 7.528159 | https://www.genecards.org/cgi-bin/carddisp.pl?gene=CCN1 |
| CD8A | CD8a Molecule | Protein Coding | 44 | GC02M086784 | 7.513724 | https://www.genecards.org/cgi-bin/carddisp.pl?gene=CD8A |
| TH | Tyrosine Hydroxylase | Protein Coding | 49 | GC11M002163 | 7.499124 | https://www.genecards.org/cgi-bin/carddisp.pl?gene=TH |
| COL11A2 | Collagen Type XI Alpha 2 Chain | Protein Coding | 42 | GC06M033162 | 7.498151 | https://www.genecards.org/cgi-bin/carddisp.pl?gene=COL11A2 |
| HIF1A | Hypoxia Inducible Factor 1 Subunit Alpha | Protein Coding | 45 | GC14P061695 | 7.455225 | https://www.genecards.org/cgi-bin/carddisp.pl?gene=HIF1A |
| IGF1 | Insulin Like Growth Factor 1 | Protein Coding | 46 | GC12M102395 | 7.454007 | https://www.genecards.org/cgi-bin/carddisp.pl?gene=IGF1 |
| POMC | Proopiomelanocortin | Protein Coding | 46 | GC02M025160 | 7.414512 | https://www.genecards.org/cgi-bin/carddisp.pl?gene=POMC |
| ADRB2 | Adrenoceptor Beta 2 | Protein Coding | 47 | GC05P148825 | 7.395474 | https://www.genecards.org/cgi-bin/carddisp.pl?gene=ADRB2 |
| MMP15 | Matrix Metallopeptidase 15 | Protein Coding | 44 | GC16P058025 | 7.291743 | https://www.genecards.org/cgi-bin/carddisp.pl?gene=MMP15 |
| FCER2 | Fc Epsilon Receptor II | Protein Coding | 42 | GC19M007689 | 7.287531 | https://www.genecards.org/cgi-bin/carddisp.pl?gene=FCER2 |
| CDK6 | Cyclin Dependent Kinase 6 | Protein Coding | 50 | GC07M092604 | 7.238047 | https://www.genecards.org/cgi-bin/carddisp.pl?gene=CDK6 |
| XDH | Xanthine Dehydrogenase | Protein Coding | 45 | GC02M031334 | 7.18777 | https://www.genecards.org/cgi- |

| | | | | | | |
|---|---|---|---|---|---|---|
| | | | | | | bin/carddisp.pl?gene=XDH |
| IGHG2 | Immunoglobulin Heavy Constant Gamma 2 (G2m Marker) | Protein Coding | 30 | GC14M112709 | 7.169412 | https://www.genecards.org/cgi-bin/carddisp.pl?gene=IGHG2 |
| IL10RB | Interleukin 10 Receptor Subunit Beta | Protein Coding | 42 | GC21P033266 | 7.163627 | https://www.genecards.org/cgi-bin/carddisp.pl?gene=IL10RB |
| TYK2 | Tyrosine Kinase 2 | Protein Coding | 50 | GC19M010350 | 7.14624 | https://www.genecards.org/cgi-bin/carddisp.pl?gene=TYK2 |
| IL1RL1 | Interleukin 1 Receptor Like 1 | Protein Coding | 40 | GC02P102294 | 7.121621 | https://www.genecards.org/cgi-bin/carddisp.pl?gene=IL1RL1 |
| FN1 | Fibronectin 1 | Protein Coding | 48 | GC02M215360 | 7.120795 | https://www.genecards.org/cgi-bin/carddisp.pl?gene=FN1 |
| JAK2 | Janus Kinase 2 | Protein Coding | 50 | GC09P004985 | 7.1167 | https://www.genecards.org/cgi-bin/carddisp.pl?gene=JAK2 |
| CFLAR | CASP8 And FADD Like Apoptosis Regulator | Protein Coding | 43 | GC02P201117 | 7.103952 | https://www.genecards.org/cgi-bin/carddisp.pl?gene=CFLAR |
| CARD14 | Caspase Recruitment Domain Family Member 14 | Protein Coding | 41 | GC17P080170 | 7.082204 | https://www.genecards.org/cgi-bin/carddisp.pl?gene=CARD14 |
| VIM | Vimentin | Protein Coding | 48 | GC10P017227 | 7.064986 | https://www.genecards.org/cgi-bin/carddisp.pl?gene=VIM |
| OPRM1 | Opioid Receptor Mu 1 | Protein Coding | 46 | GC06P154075 | 7.063599 | https://www.genecards.org/cgi-bin/carddisp.pl?gene=OPRM1 |
| AFF3 | AF4/FMR2 Family Member 3 | Protein Coding | 37 | GC02M099545 | 7.046799 | https://www.genecards.org/cgi-bin/carddisp.pl?gene=AFF3 |
| IGHM | Immunoglobulin Heavy Constant Mu | Protein Coding | 36 | GC14M112722 | 7.043907 | https://www.genecards.org/cgi-bin/carddisp.pl?gene=IGHM |
| BSG | Basigin (Ok Blood Group) | Protein Coding | 42 | GC19P000571 | 7.027616 | https://www.genecards.org/cgi-bin/carddisp.pl?gene=BSG |

| | | | | | | |
|---|---|---|---|---|---|---|
| PPP1CA | Protein Phosphatase 1 Catalytic Subunit Alpha | Protein Coding | 45 | GC11M 089964 | 6.985 15 | https://www.genecards .org/cgi-bin/carddisp.pl?gene= PPP1CA |
| CD274 | CD274 Molecule | Protein Coding | 42 | GC09P 005450 | 6.985 113 | https://www.genecards .org/cgi-bin/carddisp.pl?gene= CD274 |
| SOD1 | Superoxide Dismutase 1 | Protein Coding | 50 | GC21P 031659 | 6.984 756 | https://www.genecards .org/cgi-bin/carddisp.pl?gene= SOD1 |
| TGFBR2 | Transforming Growth Factor Beta Receptor 2 | Protein Coding | 49 | GC03P 030623 | 6.981 699 | https://www.genecards .org/cgi-bin/carddisp.pl?gene= TGFBR2 |
| CP | Ceruloplasmin | Protein Coding | 48 | GC03M 149162 | 6.978 659 | https://www.genecards .org/cgi-bin/carddisp.pl?gene= CP |
| TNFSF13 | TNF Superfamily Member 13 | Protein Coding | 43 | GC17P 007558 | 6.969 742 | https://www.genecards .org/cgi-bin/carddisp.pl?gene= TNFSF13 |
| ADAMT S5 | ADAM Metallopeptidase With Thrombospondin Type 1 Motif 5 | Protein Coding | 43 | GC21M 026918 | 6.961 925 | https://www.genecards .org/cgi-bin/carddisp.pl?gene= ADAMTS5 |
| P2RX7 | Purinergic Receptor P2X 7 | Protein Coding | 45 | GC12P 126220 | 6.924 053 | https://www.genecards .org/cgi-bin/carddisp.pl?gene= P2RX7 |
| KIF5A | Kinesin Family Member 5A | Protein Coding | 44 | GC12P 057549 | 6.896 127 | https://www.genecards .org/cgi-bin/carddisp.pl?gene= KIF5A |
| IRF1 | Interferon Regulatory Factor 1 | Protein Coding | 45 | GC05M 132440 | 6.884 698 | https://www.genecards .org/cgi-bin/carddisp.pl?gene=I RF1 |
| CALR | Calreticulin | Protein Coding | 49 | GC19P 012938 | 6.875 739 | https://www.genecards .org/cgi-bin/carddisp.pl?gene= CALR |
| TNFRSF1 4 | TNF Receptor Superfamily Member 14 | Protein Coding | 41 | GC01P 003935 | 6.871 451 | https://www.genecards .org/cgi-bin/carddisp.pl?gene= TNFRSF14 |
| IL19 | Interleukin 19 | Protein Coding | 38 | GC01P 206770 | 6.866 909 | https://www.genecards .org/cgi-bin/carddisp.pl?gene=I L19 |
| BPI | Bactericidal Permeability Increasing Protein | Protein Coding | 41 | GC20P 038304 | 6.862 829 | https://www.genecards .org/cgi- |

| | | | | | | |
|---|---|---|---|---|---|---|
| | | | | | | bin/carddisp.pl?gene=BPI |
| AGER | Advanced Glycosylation End-Product Specific Receptor | Protein Coding | 44 | GC06M032180 | 6.844262 | https://www.genecards.org/cgi-bin/carddisp.pl?gene=AGER |
| HSPA8 | Heat Shock Protein Family A (Hsp70) Member 8 | Protein Coding | 46 | GC11M123057 | 6.773395 | https://www.genecards.org/cgi-bin/carddisp.pl?gene=HSPA8 |
| TRPV1 | Transient Receptor Potential Cation Channel Subfamily V Member 1 | Protein Coding | 45 | GC17M003565 | 6.770291 | https://www.genecards.org/cgi-bin/carddisp.pl?gene=TRPV1 |
| RUNX1 | RUNX Family Transcription Factor 1 | Protein Coding | 46 | GC21M034787 | 6.767153 | https://www.genecards.org/cgi-bin/carddisp.pl?gene=RUNX1 |
| CD226 | CD226 Molecule | Protein Coding | 41 | GC18M069831 | 6.742834 | https://www.genecards.org/cgi-bin/carddisp.pl?gene=CD226 |
| BCL2L1 | BCL2 Like 1 | Protein Coding | 45 | GC20M031664 | 6.736197 | https://www.genecards.org/cgi-bin/carddisp.pl?gene=BCL2L1 |
| IFNA1 | Interferon Alpha 1 | Protein Coding | 38 | GC09P021593 | 6.710809 | https://www.genecards.org/cgi-bin/carddisp.pl?gene=IFNA1 |
| CD19 | CD19 Molecule | Protein Coding | 48 | GC16P040925 | 6.688719 | https://www.genecards.org/cgi-bin/carddisp.pl?gene=CD19 |
| CCL11 | C-C Motif Chemokine Ligand 11 | Protein Coding | 41 | GC17P034285 | 6.646458 | https://www.genecards.org/cgi-bin/carddisp.pl?gene=CCL11 |
| CD5 | CD5 Molecule | Protein Coding | 40 | GC11P061114 | 6.610099 | https://www.genecards.org/cgi-bin/carddisp.pl?gene=CD5 |
| GDF5 | Growth Differentiation Factor 5 | Protein Coding | 45 | GC20M035433 | 6.588206 | https://www.genecards.org/cgi-bin/carddisp.pl?gene=GDF5 |
| REN | Renin | Protein Coding | 47 | GC01M204154 | 6.565386 | https://www.genecards.org/cgi-bin/carddisp.pl?gene=REN |
| TTC7A | Tetratricopeptide Repeat Domain 7A | Protein Coding | 37 | GC02P046906 | 6.552021 | https://www.genecards.org/cgi-bin/carddisp.pl?gene=TTC7A |

| | | | | | | |
|---|---|---|---|---|---|---|
| CXCR2 | C-X-C Motif Chemokine Receptor 2 | Protein Coding | 48 | GC02P 218125 | 6.547 76 | https://www.genecards.org/cgi-bin/carddisp.pl?gene=CXCR2 |
| RAB27A | RAB27A, Member RAS Oncogene Family | Protein Coding | 47 | GC15M 055202 | 6.525 616 | https://www.genecards.org/cgi-bin/carddisp.pl?gene=RAB27A |
| PPP1CB | Protein Phosphatase 1 Catalytic Subunit Beta | Protein Coding | 45 | GC02P 028752 | 6.525 34 | https://www.genecards.org/cgi-bin/carddisp.pl?gene=PPP1CB |
| ABCG2 | ATP Binding Cassette Subfamily G Member 2 (Junior Blood Group) | Protein Coding | 48 | GC04M 088090 | 6.523 985 | https://www.genecards.org/cgi-bin/carddisp.pl?gene=ABCG2 |
| HRH2 | Histamine Receptor H2 | Protein Coding | 42 | GC05P 175659 | 6.494 742 | https://www.genecards.org/cgi-bin/carddisp.pl?gene=HRH2 |
| MTRR | 5-Methyltetrahydrofolate-Homocysteine Methyltransferase Reductase | Protein Coding | 42 | GC05P 007851 | 6.485 223 | https://www.genecards.org/cgi-bin/carddisp.pl?gene=MTRR |
| ATM | ATM Serine/Threonine Kinase | Protein Coding | 50 | GC11P 108222 | 6.477 387 | https://www.genecards.org/cgi-bin/carddisp.pl?gene=ATM |
| ATRIP | ATR Interacting Protein | Protein Coding | 41 | GC03P 048964 | 6.474 064 | https://www.genecards.org/cgi-bin/carddisp.pl?gene=ATRIP |
| ATRIP-TREX1 | ATRIP-TREX1 Readthrough | RNA Gene | 6 | GC03P 048966 | 6.474 064 | https://www.genecards.org/cgi-bin/carddisp.pl?gene=ATRIP-TREX1 |
| SLC17A5 | Solute Carrier Family 17 Member 5 | Protein Coding | 42 | GC06M 073593 | 6.462 096 | https://www.genecards.org/cgi-bin/carddisp.pl?gene=SLC17A5 |
| SUMO4 | Small Ubiquitin Like Modifier 4 | Protein Coding | 38 | GC06P 149401 | 6.445 432 | https://www.genecards.org/cgi-bin/carddisp.pl?gene=SUMO4 |
| CXCL11 | C-X-C Motif Chemokine Ligand 11 | Protein Coding | 39 | GC04M 076033 | 6.439 353 | https://www.genecards.org/cgi-bin/carddisp.pl?gene=CXCL11 |
| SQSTM1 | Sequestosome 1 | Protein Coding | 46 | GC05P 179806 | 6.422 469 | https://www.genecards.org/cgi-bin/carddisp.pl?gene=SQSTM1 |
| C3 | Complement C3 | Protein Coding | 46 | GC19M 006677 | 6.418 372 | https://www.genecards.org/cgi- |

| | | | | | | |
|---|---|---|---|---|---|---|
| | | | | | | bin/carddisp.pl?gene=C3 |
| HLA-DRB4 | Major Histocompatibility Complex, Class II, DR Beta 4 | Protein Coding | 29 | GC06Mo03851 | 6.408561 | https://www.genecards.org/cgi-bin/carddisp.pl?gene=HLA-DRB4 |
| WIPF1 | WAS/WASL Interacting Protein Family Member 1 | Protein Coding | 42 | GC02M174559 | 6.408106 | https://www.genecards.org/cgi-bin/carddisp.pl?gene=WIPF1 |
| ENPP1 | Ectonucleotide Pyrophosphatase/Phosphodiesterase 1 | Protein Coding | 47 | GC06P131808 | 6.387311 | https://www.genecards.org/cgi-bin/carddisp.pl?gene=ENPP1 |
| HMOX1 | Heme Oxygenase 1 | Protein Coding | 49 | GC22P035380 | 6.37917 | https://www.genecards.org/cgi-bin/carddisp.pl?gene=HMOX1 |
| MTR | 5-Methyltetrahydrofolate-Homocysteine Methyltransferase | Protein Coding | 46 | GC01P236795 | 6.375112 | https://www.genecards.org/cgi-bin/carddisp.pl?gene=MTR |
| OLIG3 | Oligodendrocyte Transcription Factor 3 | Protein Coding | 37 | GC06M137492 | 6.364253 | https://www.genecards.org/cgi-bin/carddisp.pl?gene=OLIG3 |
| PTX3 | Pentraxin 3 | Protein Coding | 41 | GC03P157436 | 6.361873 | https://www.genecards.org/cgi-bin/carddisp.pl?gene=PTX3 |
| GSTP1 | Glutathione S-Transferase Pi 1 | Protein Coding | 48 | GC11P067583 | 6.341878 | https://www.genecards.org/cgi-bin/carddisp.pl?gene=GSTP1 |
| CD27 | CD27 Molecule | Protein Coding | 44 | GC12P021037 | 6.311881 | https://www.genecards.org/cgi-bin/carddisp.pl?gene=CD27 |
| FCAR | Fc Alpha Receptor | Protein Coding | 41 | GC19P067387 | 6.284546 | https://www.genecards.org/cgi-bin/carddisp.pl?gene=FCAR |
| HAVCR2 | Hepatitis A Virus Cellular Receptor 2 | Protein Coding | 44 | GC05M157063 | 6.249082 | https://www.genecards.org/cgi-bin/carddisp.pl?gene=HAVCR2 |
| ICAM3 | Intercellular Adhesion Molecule 3 | Protein Coding | 41 | GC19M010405 | 6.227779 | https://www.genecards.org/cgi-bin/carddisp.pl?gene=ICAM3 |
| LOC100287329 | Uncharacterized LOC100287329 | RNA Gene | 12 | GC06M065958 | 6.225444 | https://www.genecards.org/cgi-bin/carddisp.pl?gene=LOC100287329 |

| | | | | | | |
|---|---|---|---|---|---|---|
| MTHFD1 | Methylenetetrahydrofolate Dehydrogenase, Cyclohydrolase And Formyltetrahydrofolate Synthetase 1 | Protein Coding | 45 | GC14P064388 | 6.218381 | https://www.genecards.org/cgi-bin/carddisp.pl?gene=MTHFD1 |
| TNFSF15 | TNF Superfamily Member 15 | Protein Coding | 42 | GC09M114784 | 6.201623 | https://www.genecards.org/cgi-bin/carddisp.pl?gene=TNFSF15 |
| MMEL1 | Membrane Metalloendopeptidase Like 1 | Protein Coding | 36 | GC01M002590 | 6.187993 | https://www.genecards.org/cgi-bin/carddisp.pl?gene=MMEL1 |
| APOE | Apolipoprotein E | Protein Coding | 48 | GC19P066879 | 6.18077 | https://www.genecards.org/cgi-bin/carddisp.pl?gene=APOE |
| HLA-DQA2 | Major Histocompatibility Complex, Class II, DQ Alpha 2 | Protein Coding | 36 | GC06P032741 | 6.169909 | https://www.genecards.org/cgi-bin/carddisp.pl?gene=HLA-DQA2 |
| SELP | Selectin P | Protein Coding | 44 | GC01M169558 | 6.164362 | https://www.genecards.org/cgi-bin/carddisp.pl?gene=SELP |
| PPARA | Peroxisome Proliferator Activated Receptor Alpha | Protein Coding | 44 | GC22P046150 | 6.159652 | https://www.genecards.org/cgi-bin/carddisp.pl?gene=PPARA |
| JAK3 | Janus Kinase 3 | Protein Coding | 49 | GC19M017824 | 6.12565 | https://www.genecards.org/cgi-bin/carddisp.pl?gene=JAK3 |
| EPO | Erythropoietin | Protein Coding | 40 | GC07P100720 | 6.119741 | https://www.genecards.org/cgi-bin/carddisp.pl?gene=EPO |
| CXCL16 | C-X-C Motif Chemokine Ligand 16 | Protein Coding | 38 | GC17M004733 | 6.098456 | https://www.genecards.org/cgi-bin/carddisp.pl?gene=CXCL16 |
| CCR4 | C-C Motif Chemokine Receptor 4 | Protein Coding | 43 | GC03P032951 | 6.09411 | https://www.genecards.org/cgi-bin/carddisp.pl?gene=CCR4 |
| TAPBP | TAP Binding Protein | Protein Coding | 44 | GC06M033299 | 6.079946 | https://www.genecards.org/cgi-bin/carddisp.pl?gene=TAPBP |
| CASP3 | Caspase 3 | Protein Coding | 47 | GC04M184627 | 6.066989 | https://www.genecards.org/cgi-bin/carddisp.pl?gene=CASP3 |

| | | | | | | |
|---|---|---|---|---|---|---|
| PNP | Purine Nucleoside Phosphorylase | Protein Coding | 45 | GC14P 020468 | 6.061 817 | https://www.genecards.org/cgi-bin/carddisp.pl?gene=PNP |
| CASP8 | Caspase 8 | Protein Coding | 49 | GC02P 201233 | 6.056 796 | https://www.genecards.org/cgi-bin/carddisp.pl?gene=CASP8 |
| TRPV4 | Transient Receptor Potential Cation Channel Subfamily V Member 4 | Protein Coding | 48 | GC12M 109783 | 6.025 859 | https://www.genecards.org/cgi-bin/carddisp.pl?gene=TRPV4 |
| CD83 | CD83 Molecule | Protein Coding | 38 | GC06P 014117 | 6.022 503 | https://www.genecards.org/cgi-bin/carddisp.pl?gene=CD83 |
| CCL19 | C-C Motif Chemokine Ligand 19 | Protein Coding | 39 | GC09M 034698 | 6.020 863 | https://www.genecards.org/cgi-bin/carddisp.pl?gene=CCL19 |
| TGFBR1 | Transforming Growth Factor Beta Receptor 1 | Protein Coding | 50 | GC09P 099104 | 5.994 832 | https://www.genecards.org/cgi-bin/carddisp.pl?gene=TGFBR1 |
| CALCR | Calcitonin Receptor | Protein Coding | 46 | GC07M 093424 | 5.985 188 | https://www.genecards.org/cgi-bin/carddisp.pl?gene=CALCR |
| FOS | Fos Proto-Oncogene, AP-1 Transcription Factor Subunit | Protein Coding | 49 | GC14P 075278 | 5.979 385 | https://www.genecards.org/cgi-bin/carddisp.pl?gene=FOS |
| COL9A3 | Collagen Type IX Alpha 3 Chain | Protein Coding | 41 | GC20P 062816 | 5.978 811 | https://www.genecards.org/cgi-bin/carddisp.pl?gene=COL9A3 |
| ITGA2 | Integrin Subunit Alpha 2 | Protein Coding | 45 | GC05P 052989 | 5.968 368 | https://www.genecards.org/cgi-bin/carddisp.pl?gene=ITGA2 |
| TLR7 | Toll Like Receptor 7 | Protein Coding | 46 | GC0XP 012867 | 5.958 578 | https://www.genecards.org/cgi-bin/carddisp.pl?gene=TLR7 |
| TRIM21 | Tripartite Motif Containing 21 | Protein Coding | 41 | GC11M 004384 | 5.957 069 | https://www.genecards.org/cgi-bin/carddisp.pl?gene=TRIM21 |
| B3GAT1 | Beta-1,3-Glucuronyltransferase 1 | Protein Coding | 42 | GC11M 134378 | 5.942 695 | https://www.genecards.org/cgi-bin/carddisp.pl?gene=B3GAT1 |
| GATA3 | GATA Binding Protein 3 | Protein Coding | 47 | GC10P 008045 | 5.924 076 | https://www.genecards.org/cgi- |

| | | | | | | |
|---|---|---|---|---|---|---|
| | | | | | | bin/carddisp.pl?gene=GATA3 |
| MMP17 | Matrix Metallopeptidase 17 | Protein Coding | 42 | GC12P 131828 | 5.905 069 | https://www.genecards.org/cgi-bin/carddisp.pl?gene=MMP17 |
| ITGA4 | Integrin Subunit Alpha 4 | Protein Coding | 46 | GC02P 181456 | 5.899 445 | https://www.genecards.org/cgi-bin/carddisp.pl?gene=ITGA4 |
| SERPINA 3 | Serpin Family A Member 3 | Protein Coding | 42 | GC14P 094612 | 5.893 335 | https://www.genecards.org/cgi-bin/carddisp.pl?gene=SERPINA3 |
| XIAP | X-Linked Inhibitor Of Apoptosis | Protein Coding | 46 | GC0XP 123859 | 5.881 82 | https://www.genecards.org/cgi-bin/carddisp.pl?gene=XIAP |
| IRAK4 | Interleukin 1 Receptor Associated Kinase 4 | Protein Coding | 45 | GC12P 043758 | 5.876 334 | https://www.genecards.org/cgi-bin/carddisp.pl?gene=IRAK4 |
| VIP | Vasoactive Intestinal Peptide | Protein Coding | 43 | GC06P 152750 | 5.874 033 | https://www.genecards.org/cgi-bin/carddisp.pl?gene=VIP |
| ASPN | Asporin | Protein Coding | 41 | GC09M 094207 | 5.865 178 | https://www.genecards.org/cgi-bin/carddisp.pl?gene=ASPN |
| AKT1 | AKT Serine/Threonine Kinase 1 | Protein Coding | 50 | GC14M 104769 | 5.835 953 | https://www.genecards.org/cgi-bin/carddisp.pl?gene=AKT1 |
| EGF | Epidermal Growth Factor | Protein Coding | 49 | GC04P 109912 | 5.833 037 | https://www.genecards.org/cgi-bin/carddisp.pl?gene=EGF |
| BMP4 | Bone Morphogenetic Protein 4 | Protein Coding | 46 | GC14M 053949 | 5.830 775 | https://www.genecards.org/cgi-bin/carddisp.pl?gene=BMP4 |
| SERPINA 1 | Serpin Family A Member 1 | Protein Coding | 47 | GC14M 094376 | 5.826 702 | https://www.genecards.org/cgi-bin/carddisp.pl?gene=SERPINA1 |
| ANGPT1 | Angiopoietin 1 | Protein Coding | 44 | GC08M 107246 | 5.818 006 | https://www.genecards.org/cgi-bin/carddisp.pl?gene=ANGPT1 |
| TNFSF10 | TNF Superfamily Member 10 | Protein Coding | 42 | GC03M 172505 | 5.817 306 | https://www.genecards.org/cgi-bin/carddisp.pl?gene=TNFSF10 |

| | | | | | | |
|---|---|---|---|---|---|---|
| CD163 | CD163 Molecule | Protein Coding | 42 | GC12M 007819 | 5.807 335 | https://www.genecards .org/cgi- bin/carddisp.pl?gene= CD163 |
| ITGB1 | Integrin Subunit Beta 1 | Protein Coding | 48 | GC10M 033116 | 5.803 552 | https://www.genecards .org/cgi- bin/carddisp.pl?gene=I TGB1 |
| SOST | Sclerostin | Protein Coding | 42 | GC17M 043753 | 5.798 334 | https://www.genecards .org/cgi- bin/carddisp.pl?gene= SOST |
| SRC | SRC Proto-Oncogene, Non-Receptor Tyrosine Kinase | Protein Coding | 49 | GC20P 037344 | 5.788 343 | https://www.genecards .org/cgi- bin/carddisp.pl?gene= SRC |
| MUC5B | Mucin 5B, Oligomeric Mucus/Gel-Forming | Protein Coding | 41 | GC11P 001831 | 5.763 791 | https://www.genecards .org/cgi- bin/carddisp.pl?gene= MUC5B |
| SOCS3 | Suppressor Of Cytokine Signaling 3 | Protein Coding | 41 | GC17M 078356 | 5.740 062 | https://www.genecards .org/cgi- bin/carddisp.pl?gene= SOCS3 |
| TRB | T Cell Receptor Beta Locus | Protein Coding | 19 | GC07P 148690 | 5.738 567 | https://www.genecards .org/cgi- bin/carddisp.pl?gene= TRB |
| PRKCH | Protein Kinase C Eta | Protein Coding | 48 | GC14P 061187 | 5.737 882 | https://www.genecards .org/cgi- bin/carddisp.pl?gene= PRKCH |
| FPGS | Folylpolyglutamate Synthase | Protein Coding | 41 | GC09P 127794 | 5.735 916 | https://www.genecards .org/cgi- bin/carddisp.pl?gene= FPGS |
| PTEN | Phosphatase And Tensin Homolog | Protein Coding | 50 | GC10P 092487 | 5.722 382 | https://www.genecards .org/cgi- bin/carddisp.pl?gene= PTEN |
| MTOR | Mechanistic Target Of Rapamycin Kinase | Protein Coding | 51 | GC01M 011106 | 5.715 071 | https://www.genecards .org/cgi- bin/carddisp.pl?gene= MTOR |
| PIK3R1 | Phosphoinositide-3-Kinase Regulatory Subunit 1 | Protein Coding | 49 | GC05P 068215 | 5.710 825 | https://www.genecards .org/cgi- bin/carddisp.pl?gene= PIK3R1 |
| SH2B3 | SH2B Adaptor Protein 3 | Protein Coding | 45 | GC12P 111405 | 5.707 057 | https://www.genecards .org/cgi- bin/carddisp.pl?gene= SH2B3 |
| F3 | Coagulation Factor III, Tissue Factor | Protein Coding | 44 | GC01M 094590 | 5.683 108 | https://www.genecards .org/cgi- |

| | | | | | | |
|---|---|---|---|---|---|---|
| | | | | | | bin/carddisp.pl?gene=F3 |
| GGH | Gamma-Glutamyl Hydrolase | Protein Coding | 42 | GC08M063014 | 5.675788 | https://www.genecards.org/cgi-bin/carddisp.pl?gene=GGH |
| IL7R | Interleukin 7 Receptor | Protein Coding | 44 | GC05P035852 | 5.666647 | https://www.genecards.org/cgi-bin/carddisp.pl?gene=IL7R |
| NPY | Neuropeptide Y | Protein Coding | 44 | GC07P024290 | 5.647635 | https://www.genecards.org/cgi-bin/carddisp.pl?gene=NPY |
| IMPDH2 | Inosine Monophosphate Dehydrogenase 2 | Protein Coding | 46 | GC03M051162 | 5.643008 | https://www.genecards.org/cgi-bin/carddisp.pl?gene=IMPDH2 |
| SOCS1 | Suppressor Of Cytokine Signaling 1 | Protein Coding | 42 | GC16M012261 | 5.640675 | https://www.genecards.org/cgi-bin/carddisp.pl?gene=SOCS1 |
| SLC22A5 | Solute Carrier Family 22 Member 5 | Protein Coding | 46 | GC05P132369 | 5.637074 | https://www.genecards.org/cgi-bin/carddisp.pl?gene=SLC22A5 |
| GSTT1 | Glutathione S-Transferase Theta 1 | Protein Coding | 33 | GC22Mi00270 | 5.630356 | https://www.genecards.org/cgi-bin/carddisp.pl?gene=GSTT1 |
| MATN1 | Matrilin 1 | Protein Coding | 40 | GC01M030711 | 5.624109 | https://www.genecards.org/cgi-bin/carddisp.pl?gene=MATN1 |
| KITLG | KIT Ligand | Protein Coding | 43 | GC12M088492 | 5.614833 | https://www.genecards.org/cgi-bin/carddisp.pl?gene=KITLG |
| THBD | Thrombomodulin | Protein Coding | 44 | GC20M023026 | 5.603651 | https://www.genecards.org/cgi-bin/carddisp.pl?gene=THBD |
| CNR2 | Cannabinoid Receptor 2 | Protein Coding | 44 | GC01M023870 | 5.572924 | https://www.genecards.org/cgi-bin/carddisp.pl?gene=CNR2 |
| DEFB4A | Defensin Beta 4A | Protein Coding | 34 | GC08P007895 | 5.548926 | https://www.genecards.org/cgi-bin/carddisp.pl?gene=DEFB4A |
| AIF1 | Allograft Inflammatory Factor 1 | Protein Coding | 37 | GC06P083694 | 5.534014 | https://www.genecards.org/cgi-bin/carddisp.pl?gene=AIF1 |

| | | | | | | |
|---|---|---|---|---|---|---|
| SOD2 | Superoxide Dismutase 2 | Protein Coding | 48 | GC06M 159669 | 5.532 454 | https://www.genecards .org/cgi-bin/carddisp.pl?gene= SOD2 |
| NFKBIE | NFKB Inhibitor Epsilon | Protein Coding | 38 | GC06M 044258 | 5.517 525 | https://www.genecards .org/cgi-bin/carddisp.pl?gene= NFKBIE |
| PRDM1 | PR/SET Domain 1 | Protein Coding | 43 | GC06P 105993 | 5.491 177 | https://www.genecards .org/cgi-bin/carddisp.pl?gene= PRDM1 |
| CYP2C19 | Cytochrome P450 Family 2 Subfamily C Member 19 | Protein Coding | 46 | GC10P 094762 | 5.480 085 | https://www.genecards .org/cgi-bin/carddisp.pl?gene= CYP2C19 |
| TNFRSF2 5 | TNF Receptor Superfamily Member 25 | Protein Coding | 40 | GC01M 006460 | 5.475 738 | https://www.genecards .org/cgi-bin/carddisp.pl?gene= TNFRSF25 |
| F2RL1 | F2R Like Trypsin Receptor 1 | Protein Coding | 44 | GC05P 076818 | 5.467 809 | https://www.genecards .org/cgi-bin/carddisp.pl?gene= F2RL1 |
| PLAUR | Plasminogen Activator, Urokinase Receptor | Protein Coding | 42 | GC19M 043646 | 5.436 357 | https://www.genecards .org/cgi-bin/carddisp.pl?gene= PLAUR |
| EDN1 | Endothelin 1 | Protein Coding | 46 | GC06P 012256 | 5.424 318 | https://www.genecards .org/cgi-bin/carddisp.pl?gene= EDN1 |
| PTPN11 | Protein Tyrosine Phosphatase Non-Receptor Type 11 | Protein Coding | 50 | GC12P 112418 | 5.418 618 | https://www.genecards .org/cgi-bin/carddisp.pl?gene= PTPN11 |
| ZAP70 | Zeta Chain Of T Cell Receptor Associated Protein Kinase 70 | Protein Coding | 49 | GC02P 097753 | 5.417 872 | https://www.genecards .org/cgi-bin/carddisp.pl?gene= ZAP70 |
| RNASEH 2B | Ribonuclease H2 Subunit B | Protein Coding | 36 | GC13P 050909 | 5.408 074 | https://www.genecards .org/cgi-bin/carddisp.pl?gene= RNASEH2B |
| TEK | TEK Receptor Tyrosine Kinase | Protein Coding | 49 | GC09P 027109 | 5.394 337 | https://www.genecards .org/cgi-bin/carddisp.pl?gene= TEK |
| H2AC18 | H2A Clustered Histone 18 | Protein Coding | 26 | GC01M 152015 | 5.390 277 | https://www.genecards .org/cgi-bin/carddisp.pl?gene= H2AC18 |
| ZFP36 | ZFP36 Ring Finger Protein | Protein Coding | 37 | GC19P 039406 | 5.388 78 | https://www.genecards .org/cgi- |

| | | | | | | |
|---|---|---|---|---|---|---|
| | | | | | | bin/carddisp.pl?gene=ZFP36 |
| RBPJ | Recombination Signal Binding Protein For Immunoglobulin Kappa J Region | Protein Coding | 45 | GC04P026105 | 5.386807 | https://www.genecards.org/cgi-bin/carddisp.pl?gene=RBPJ |
| HAVCR1 | Hepatitis A Virus Cellular Receptor 1 | Protein Coding | 41 | GC05M157028 | 5.377156 | https://www.genecards.org/cgi-bin/carddisp.pl?gene=HAVCR1 |
| GZMA | Granzyme A | Protein Coding | 42 | GC05P055102 | 5.359779 | https://www.genecards.org/cgi-bin/carddisp.pl?gene=GZMA |
| TAC1 | Tachykinin Precursor 1 | Protein Coding | 41 | GC07P097731 | 5.356418 | https://www.genecards.org/cgi-bin/carddisp.pl?gene=TAC1 |
| RNPC3 | RNA Binding Region (RNP1, RRM) Containing 3 | Protein Coding | 35 | GC01P103525 | 5.353688 | https://www.genecards.org/cgi-bin/carddisp.pl?gene=RNPC3 |
| ITPA | Inosine Triphosphatase | Protein Coding | 44 | GC20P004250 | 5.335377 | https://www.genecards.org/cgi-bin/carddisp.pl?gene=ITPA |
| ATXN2 | Ataxin 2 | Protein Coding | 41 | GC12M111443 | 5.328629 | https://www.genecards.org/cgi-bin/carddisp.pl?gene=ATXN2 |
| HTR2A | 5-Hydroxytryptamine Receptor 2A | Protein Coding | 46 | GC13M046831 | 5.319273 | https://www.genecards.org/cgi-bin/carddisp.pl?gene=HTR2A |
| FCGR1A | Fc Gamma Receptor Ia | Protein Coding | 40 | GC01P150197 | 5.309157 | https://www.genecards.org/cgi-bin/carddisp.pl?gene=FCGR1A |
| CD55 | CD55 Molecule (Cromer Blood Group) | Protein Coding | 45 | GC01P207321 | 5.290905 | https://www.genecards.org/cgi-bin/carddisp.pl?gene=CD55 |
| PLAT | Plasminogen Activator, Tissue Type | Protein Coding | 48 | GC08M042174 | 5.276639 | https://www.genecards.org/cgi-bin/carddisp.pl?gene=PLAT |
| KLRK1 | Killer Cell Lectin Like Receptor K1 | Protein Coding | 40 | GC12M021107 | 5.271939 | https://www.genecards.org/cgi-bin/carddisp.pl?gene=KLRK1 |
| PLAU | Plasminogen Activator, Urokinase | Protein Coding | 50 | GC10P073909 | 5.263295 | https://www.genecards.org/cgi-bin/carddisp.pl?gene=PLAU |

| | | | | | | |
|---|---|---|---|---|---|---|
| IL9 | Interleukin 9 | Protein Coding | 41 | GC05M 135891 | 5.260 829 | https://www.genecards .org/cgi-bin/carddisp.pl?gene=IL9 |
| TNFRSF17 | TNF Receptor Superfamily Member 17 | Protein Coding | 42 | GC16P 011965 | 5.259 591 | https://www.genecards .org/cgi-bin/carddisp.pl?gene= TNFRSF17 |
| GAS5 | Growth Arrest Specific 5 | RNA Gene | 24 | GC01M 173947 | 5.258 753 | https://www.genecards .org/cgi-bin/carddisp.pl?gene= GAS5 |
| LRP5 | LDL Receptor Related Protein 5 | Protein Coding | 47 | GC11P 068298 | 5.258 183 | https://www.genecards .org/cgi-bin/carddisp.pl?gene= LRP5 |
| IBSP | Integrin Binding Sialoprotein | Protein Coding | 37 | GC04P 087799 | 5.229 711 | https://www.genecards .org/cgi-bin/carddisp.pl?gene=IBSP |
| IL20 | Interleukin 20 | Protein Coding | 37 | GC01P 206866 | 5.205 414 | https://www.genecards .org/cgi-bin/carddisp.pl?gene=IL20 |
| LEPQTL1 | Leptin, Serum Levels Of | Genetic Locus | 3 | GC02U 903086 | 5.204 472 | https://www.genecards .org/cgi-bin/carddisp.pl?gene= LEPQTL1 |
| PTGER4 | Prostaglandin E Receptor 4 | Protein Coding | 45 | GC05P 040679 | 5.203 375 | https://www.genecards .org/cgi-bin/carddisp.pl?gene= PTGER4 |
| INPP5E | Inositol Polyphosphate-5-Phosphatase E | Protein Coding | 40 | GC09M 136428 | 5.197 738 | https://www.genecards .org/cgi-bin/carddisp.pl?gene=INPP5E |
| MIR140 | MicroRNA 140 | RNA Gene | 24 | GC16P 069934 | 5.193 281 | https://www.genecards .org/cgi-bin/carddisp.pl?gene= MIR140 |
| CDKN1A | Cyclin Dependent Kinase Inhibitor 1A | Protein Coding | 46 | GC06P 083850 | 5.184 971 | https://www.genecards .org/cgi-bin/carddisp.pl?gene= CDKN1A |
| AR | Androgen Receptor | Protein Coding | 50 | GC0XP 067544 | 5.170 87 | https://www.genecards .org/cgi-bin/carddisp.pl?gene= AR |
| NGF | Nerve Growth Factor | Protein Coding | 47 | GC01M 115285 | 5.167 191 | https://www.genecards .org/cgi-bin/carddisp.pl?gene= NGF |
| PSMB8 | Proteasome 20S Subunit Beta 8 | Protein Coding | 48 | GC06M 032840 | 5.164 179 | https://www.genecards .org/cgi- |

| | | | | | | |
|---|---|---|---|---|---|---|
| | | | | | | bin/carddisp.pl?gene=PSMB8 |
| ASAH1 | N-Acylsphingosine Amidohydrolase 1 | Protein Coding | 45 | GC08M018055 | 5.158409 | https://www.genecards.org/cgi-bin/carddisp.pl?gene=ASAH1 |
| EGFR | Epidermal Growth Factor Receptor | Protein Coding | 51 | GC07P055019 | 5.122825 | https://www.genecards.org/cgi-bin/carddisp.pl?gene=EGFR |
| NPPB | Natriuretic Peptide B | Protein Coding | 42 | GC01M011858 | 5.110035 | https://www.genecards.org/cgi-bin/carddisp.pl?gene=NPPB |
| TACR1 | Tachykinin Receptor 1 | Protein Coding | 44 | GC02M075112 | 5.092575 | https://www.genecards.org/cgi-bin/carddisp.pl?gene=TACR1 |
| IGFBP1 | Insulin Like Growth Factor Binding Protein 1 | Protein Coding | 41 | GC07P047770 | 5.092317 | https://www.genecards.org/cgi-bin/carddisp.pl?gene=IGFBP1 |
| NOTCH4 | Notch Receptor 4 | Protein Coding | 44 | GC06M065988 | 5.078926 | https://www.genecards.org/cgi-bin/carddisp.pl?gene=NOTCH4 |
| COL9A2 | Collagen Type IX Alpha 2 Chain | Protein Coding | 42 | GC01M040300 | 5.077526 | https://www.genecards.org/cgi-bin/carddisp.pl?gene=COL9A2 |
| ESR2 | Estrogen Receptor 2 | Protein Coding | 47 | GC14M064084 | 5.073864 | https://www.genecards.org/cgi-bin/carddisp.pl?gene=ESR2 |
| SPRED2 | Sprouty Related EVH1 Domain Containing 2 | Protein Coding | 41 | GC02M065307 | 5.062191 | https://www.genecards.org/cgi-bin/carddisp.pl?gene=SPRED2 |
| LPIN2 | Lipin 2 | Protein Coding | 41 | GC18M002916 | 5.054539 | https://www.genecards.org/cgi-bin/carddisp.pl?gene=LPIN2 |
| CCL13 | C-C Motif Chemokine Ligand 13 | Protein Coding | 36 | GC17P034356 | 5.029807 | https://www.genecards.org/cgi-bin/carddisp.pl?gene=CCL13 |
| UBASH3A | Ubiquitin Associated And SH3 Domain Containing A | Protein Coding | 38 | GC21P042403 | 5.027813 | https://www.genecards.org/cgi-bin/carddisp.pl?gene=UBASH3A |
| STING1 | Stimulator Of Interferon Response CGAMP Interactor 1 | Protein Coding | 32 | GC05M139476 | 5.005392 | https://www.genecards.org/cgi-bin/carddisp.pl?gene=STING1 |

| | | | | | | |
|---|---|---|---|---|---|---|
| APOM | Apolipoprotein M | Protein Coding | 40 | GC06P 083702 | 5.000 204 | https://www.genecards.org/cgi-bin/carddisp.pl?gene=APOM |
| CST3 | Cystatin C | Protein Coding | 42 | GC20M 023667 | 4.991 611 | https://www.genecards.org/cgi-bin/carddisp.pl?gene=CST3 |
| DHFR | Dihydrofolate Reductase | Protein Coding | 47 | GC05M 080626 | 4.982 999 | https://www.genecards.org/cgi-bin/carddisp.pl?gene=DHFR |
| CD2 | CD2 Molecule | Protein Coding | 42 | GC01P 116754 | 4.977 384 | https://www.genecards.org/cgi-bin/carddisp.pl?gene=CD2 |
| ELN | Elastin | Protein Coding | 43 | GC07P 074027 | 4.954 332 | https://www.genecards.org/cgi-bin/carddisp.pl?gene=ELN |
| MIR223 | MicroRNA 223 | RNA Gene | 22 | GC0XP 066018 | 4.915 719 | https://www.genecards.org/cgi-bin/carddisp.pl?gene=MIR223 |
| STAT6 | Signal Transducer And Activator Of Transcription 6 | Protein Coding | 48 | GC12M 057095 | 4.905 521 | https://www.genecards.org/cgi-bin/carddisp.pl?gene=STAT6 |
| TNXB | Tenascin XB | Protein Coding | 43 | GC06M 065977 | 4.873 197 | https://www.genecards.org/cgi-bin/carddisp.pl?gene=TNXB |
| IRF8 | Interferon Regulatory Factor 8 | Protein Coding | 44 | GC16P 085898 | 4.869 159 | https://www.genecards.org/cgi-bin/carddisp.pl?gene=IRF8 |
| PARP1 | Poly(ADP-Ribose) Polymerase 1 | Protein Coding | 47 | GC01M 226360 | 4.865 338 | https://www.genecards.org/cgi-bin/carddisp.pl?gene=PARP1 |
| NOD1 | Nucleotide Binding Oligomerization Domain Containing 1 | Protein Coding | 42 | GC07M 030424 | 4.851 906 | https://www.genecards.org/cgi-bin/carddisp.pl?gene=NOD1 |
| MIR21 | MicroRNA 21 | RNA Gene | 24 | GC17P 059841 | 4.831 304 | https://www.genecards.org/cgi-bin/carddisp.pl?gene=MIR21 |
| AMPD1 | Adenosine Monophosphate Deaminase 1 | Protein Coding | 45 | GC01M 114673 | 4.829 415 | https://www.genecards.org/cgi-bin/carddisp.pl?gene=AMPD1 |
| F2 | Coagulation Factor II, Thrombin | Protein Coding | 48 | GC11P 046720 | 4.828 295 | https://www.genecards.org/cgi- |

| | | | | | | |
|---|---|---|---|---|---|---|
| | | | | | | bin/carddisp.pl?gene=F2 |
| IL21R | Interleukin 21 Receptor | Protein Coding | 41 | GC16P 027995 | 4.825 69 | https://www.genecards.org/cgi-bin/carddisp.pl?gene=IL21R |
| DDX41 | DEAD-Box Helicase 41 | Protein Coding | 43 | GC05M 177511 | 4.806 009 | https://www.genecards.org/cgi-bin/carddisp.pl?gene=DDX41 |
| STAT5B | Signal Transducer And Activator Of Transcription 5B | Protein Coding | 47 | GC17M 042199 | 4.797 704 | https://www.genecards.org/cgi-bin/carddisp.pl?gene=STAT5B |
| IL27 | Interleukin 27 | Protein Coding | 36 | GC16M 036923 | 4.790 442 | https://www.genecards.org/cgi-bin/carddisp.pl?gene=IL27 |
| TBX21 | T-Box Transcription Factor 21 | Protein Coding | 44 | GC17P 047733 | 4.768 191 | https://www.genecards.org/cgi-bin/carddisp.pl?gene=TBX21 |
| FGFR2 | Fibroblast Growth Factor Receptor 2 | Protein Coding | 52 | GC10M 121478 | 4.751 418 | https://www.genecards.org/cgi-bin/carddisp.pl?gene=FGFR2 |
| MDM2 | MDM2 Proto-Oncogene | Protein Coding | 51 | GC12P 068808 | 4.748 178 | https://www.genecards.org/cgi-bin/carddisp.pl?gene=MDM2 |
| APOA1 | Apolipoprotein A1 | Protein Coding | 46 | GC11M 116835 | 4.745 225 | https://www.genecards.org/cgi-bin/carddisp.pl?gene=APOA1 |
| PDPN | Podoplanin | Protein Coding | 40 | GC01P 013583 | 4.719 225 | https://www.genecards.org/cgi-bin/carddisp.pl?gene=PDPN |
| HLA-DOA | Major Histocompatibility Complex, Class II, DO Alpha | Protein Coding | 39 | GC06M 033004 | 4.718 634 | https://www.genecards.org/cgi-bin/carddisp.pl?gene=HLA-DOA |
| MIR125A | MicroRNA 125a | RNA Gene | 22 | GC19P 067235 | 4.701 859 | https://www.genecards.org/cgi-bin/carddisp.pl?gene=MIR125A |
| S100A4 | S100 Calcium Binding Protein A4 | Protein Coding | 43 | GC01M 153543 | 4.697 592 | https://www.genecards.org/cgi-bin/carddisp.pl?gene=S100A4 |
| BRD2 | Bromodomain Containing 2 | Protein Coding | 43 | GC06P 083733 | 4.694 026 | https://www.genecards.org/cgi-bin/carddisp.pl?gene=BRD2 |

| | | | | | | |
|---|---|---|---|---|---|---|
| FLT1 | Fms Related Receptor Tyrosine Kinase 1 | Protein Coding | 49 | GC13M 028300 | 4.670 346 | https://www.genecards.org/cgi-bin/carddisp.pl?gene=FLT1 |
| ADAR | Adenosine Deaminase RNA Specific | Protein Coding | 43 | GC01M 154582 | 4.662 095 | https://www.genecards.org/cgi-bin/carddisp.pl?gene=ADAR |
| CNDP2 | Carnosine Dipeptidase 2 | Protein Coding | 41 | GC18P 074495 | 4.652 85 | https://www.genecards.org/cgi-bin/carddisp.pl?gene=CNDP2 |
| CAV1 | Caveolin 1 | Protein Coding | 46 | GC07P 116524 | 4.651 944 | https://www.genecards.org/cgi-bin/carddisp.pl?gene=CAV1 |
| CYP1A2 | Cytochrome P450 Family 1 Subfamily A Member 2 | Protein Coding | 45 | GC15P 074748 | 4.650 348 | https://www.genecards.org/cgi-bin/carddisp.pl?gene=CYP1A2 |
| NTRK1 | Neurotrophic Receptor Tyrosine Kinase 1 | Protein Coding | 46 | GC01P 156815 | 4.650 138 | https://www.genecards.org/cgi-bin/carddisp.pl?gene=NTRK1 |
| LGALS3 | Galectin 3 | Protein Coding | 43 | GC14P 055124 | 4.642 893 | https://www.genecards.org/cgi-bin/carddisp.pl?gene=LGALS3 |
| PIP4K2C | Phosphatidylinositol-5-Phosphate 4-Kinase Type 2 Gamma | Protein Coding | 37 | GC12P 057591 | 4.636 696 | https://www.genecards.org/cgi-bin/carddisp.pl?gene=PIP4K2C |
| COX5A | Cytochrome C Oxidase Subunit 5A | Protein Coding | 42 | GC15M 074919 | 4.629 854 | https://www.genecards.org/cgi-bin/carddisp.pl?gene=COX5A |
| IGFBP3 | Insulin Like Growth Factor Binding Protein 3 | Protein Coding | 44 | GC07M 045912 | 4.626 127 | https://www.genecards.org/cgi-bin/carddisp.pl?gene=IGFBP3 |
| IL1RL2 | Interleukin 1 Receptor Like 2 | Protein Coding | 41 | GC02P 102186 | 4.623 078 | https://www.genecards.org/cgi-bin/carddisp.pl?gene=IL1RL2 |
| LGALS9 | Galectin 9 | Protein Coding | 38 | GC17P 027629 | 4.622 476 | https://www.genecards.org/cgi-bin/carddisp.pl?gene=LGALS9 |
| H3C14 | H3 Clustered Histone 14 | Protein Coding | 27 | GC01M 152021 | 4.616 464 | https://www.genecards.org/cgi-bin/carddisp.pl?gene=H3C14 |
| RAD51B | RAD51 Paralog B | Protein Coding | 36 | GC14P 067819 | 4.597 606 | https://www.genecards.org/cgi- |

| | | | | | | |
|---|---|---|---|---|---|---|
| | | | | | | bin/carddisp.pl?gene=RAD51B |
| JAK1 | Janus Kinase 1 | Protein Coding | 51 | GC01M064833 | 4.586927 | https://www.genecards.org/cgi-bin/carddisp.pl?gene=JAK1 |
| MIR499A | MicroRNA 499a | RNA Gene | 22 | GC20P034990 | 4.585382 | https://www.genecards.org/cgi-bin/carddisp.pl?gene=MIR499A |
| BLTP1 | Bridge-Like Lipid Transfer Protein Family Member 1 | Protein Coding | 26 | GC04P122154 | 4.580682 | https://www.genecards.org/cgi-bin/carddisp.pl?gene=BLTP1 |
| CD81 | CD81 Molecule | Protein Coding | 44 | GC11P002532 | 4.570866 | https://www.genecards.org/cgi-bin/carddisp.pl?gene=CD81 |
| ADORA3 | Adenosine A3 Receptor | Protein Coding | 44 | GC01M111499 | 4.561628 | https://www.genecards.org/cgi-bin/carddisp.pl?gene=ADORA3 |
| CA2 | Carbonic Anhydrase 2 | Protein Coding | 49 | GC08P085463 | 4.554908 | https://www.genecards.org/cgi-bin/carddisp.pl?gene=CA2 |
| KIR2DS2 | Killer Cell Immunoglobulin Like Receptor, Two Ig Domains And Short Cytoplasmic Tail 2 | Protein Coding | 25 | GC19MR00122 | 4.553277 | https://www.genecards.org/cgi-bin/carddisp.pl?gene=KIR2DS2 |
| RO60 | Ro60, Y RNA Binding Protein | Protein Coding | 29 | GC01P193059 | 4.552504 | https://www.genecards.org/cgi-bin/carddisp.pl?gene=RO60 |
| LTB | Lymphotoxin Beta | Protein Coding | 38 | GC06M065959 | 4.551324 | https://www.genecards.org/cgi-bin/carddisp.pl?gene=LTB |
| COL1A2 | Collagen Type I Alpha 2 Chain | Protein Coding | 46 | GC07P094394 | 4.538369 | https://www.genecards.org/cgi-bin/carddisp.pl?gene=COL1A2 |
| COL10A1 | Collagen Type X Alpha 1 Chain | Protein Coding | 41 | GC06M116118 | 4.536696 | https://www.genecards.org/cgi-bin/carddisp.pl?gene=COL10A1 |
| DCN | Decorin | Protein Coding | 45 | GC12M091140 | 4.536048 | https://www.genecards.org/cgi-bin/carddisp.pl?gene=DCN |
| PECAM1 | Platelet And Endothelial Cell Adhesion Molecule 1 | Protein Coding | 40 | GC17M064319 | 4.526588 | https://www.genecards.org/cgi- |

| | | | | | | |
|---|---|---|---|---|---|---|
| | | | | | | bin/carddisp.pl?gene=PECAM1 |
| HLA-DQB2 | Major Histocompatibility Complex, Class II, DQ Beta 2 | Protein Coding | 38 | GC06M032756 | 4.519013 | https://www.genecards.org/cgi-bin/carddisp.pl?gene=HLA-DQB2 |
| ACP1 | Acid Phosphatase 1 | Protein Coding | 42 | GC02P000344 | 4.517788 | https://www.genecards.org/cgi-bin/carddisp.pl?gene=ACP1 |
| CYP19A1 | Cytochrome P450 Family 19 Subfamily A Member 1 | Protein Coding | 47 | GC15M051208 | 4.512114 | https://www.genecards.org/cgi-bin/carddisp.pl?gene=CYP19A1 |
| CFHR2 | Complement Factor H Related 2 | Protein Coding | 38 | GC01P196943 | 4.511574 | https://www.genecards.org/cgi-bin/carddisp.pl?gene=CFHR2 |
| IL12RB1 | Interleukin 12 Receptor Subunit Beta 1 | Protein Coding | 42 | GC19M018058 | 4.509627 | https://www.genecards.org/cgi-bin/carddisp.pl?gene=IL12RB1 |
| FTH1 | Ferritin Heavy Chain 1 | Protein Coding | 48 | GC11M061959 | 4.501761 | https://www.genecards.org/cgi-bin/carddisp.pl?gene=FTH1 |
| IKBKE | Inhibitor Of Nuclear Factor Kappa B Kinase Subunit Epsilon | Protein Coding | 44 | GC01P206470 | 4.484868 | https://www.genecards.org/cgi-bin/carddisp.pl?gene=IKBKE |
| MIR143 | MicroRNA 143 | RNA Gene | 25 | GC05P149410 | 4.473273 | https://www.genecards.org/cgi-bin/carddisp.pl?gene=MIR143 |
| LBP | Lipopolysaccharide Binding Protein | Protein Coding | 42 | GC20P038346 | 4.469586 | https://www.genecards.org/cgi-bin/carddisp.pl?gene=LBP |
| DDR2 | Discoidin Domain Receptor Tyrosine Kinase 2 | Protein Coding | 49 | GC01P162631 | 4.465885 | https://www.genecards.org/cgi-bin/carddisp.pl?gene=DDR2 |
| CARD10 | Caspase Recruitment Domain Family Member 10 | Protein Coding | 39 | GC22M057585 | 4.451181 | https://www.genecards.org/cgi-bin/carddisp.pl?gene=CARD10 |
| CCL17 | C-C Motif Chemokine Ligand 17 | Protein Coding | 38 | GC16P057414 | 4.444296 | https://www.genecards.org/cgi-bin/carddisp.pl?gene=CCL17 |
| SPTAN1 | Spectrin Alpha, Non-Erythrocytic 1 | Protein Coding | 46 | GC09P128552 | 4.438413 | https://www.genecards.org/cgi-bin/carddisp.pl?gene=SPTAN1 |

| | | | | | | |
|---|---|---|---|---|---|---|
| BDNF | Brain Derived Neurotrophic Factor | Protein Coding | 46 | GC11M 027654 | 4.437 177 | https://www.genecards.org/cgi-bin/carddisp.pl?gene=BDNF |
| NFATC1 | Nuclear Factor Of Activated T Cells 1 | Protein Coding | 45 | GC18P 079395 | 4.426 399 | https://www.genecards.org/cgi-bin/carddisp.pl?gene=NFATC1 |
| IL34 | Interleukin 34 | Protein Coding | 37 | GC16P 071585 | 4.412 497 | https://www.genecards.org/cgi-bin/carddisp.pl?gene=IL34 |
| EEF1A1 | Eukaryotic Translation Elongation Factor 1 Alpha 1 | Protein Coding | 42 | GC06M 073515 | 4.397 603 | https://www.genecards.org/cgi-bin/carddisp.pl?gene=EEF1A1 |
| APRT | Adenine Phosphoribosyltransferase | Protein Coding | 45 | GC16M 088810 | 4.397 479 | https://www.genecards.org/cgi-bin/carddisp.pl?gene=APRT |
| SUMO1 | Small Ubiquitin Like Modifier 1 | Protein Coding | 44 | GC02M 202206 | 4.394 073 | https://www.genecards.org/cgi-bin/carddisp.pl?gene=SUMO1 |
| TSBP1 | Testis Expressed Basic Protein 1 | Protein Coding | 24 | GC06M 032288 | 4.390 658 | https://www.genecards.org/cgi-bin/carddisp.pl?gene=TSBP1 |
| HLA-DRB5 | Major Histocompatibility Complex, Class II, DR Beta 5 | Protein Coding | 39 | GC06M 066002 | 4.381 423 | https://www.genecards.org/cgi-bin/carddisp.pl?gene=HLA-DRB5 |
| FGF1 | Fibroblast Growth Factor 1 | Protein Coding | 45 | GC05M 142555 | 4.375 101 | https://www.genecards.org/cgi-bin/carddisp.pl?gene=FGF1 |
| IL17D | Interleukin 17D | Protein Coding | 36 | GC13P 020702 | 4.367 647 | https://www.genecards.org/cgi-bin/carddisp.pl?gene=IL17D |
| CENPB | Centromere Protein B | Protein Coding | 36 | GC20M 003783 | 4.356 783 | https://www.genecards.org/cgi-bin/carddisp.pl?gene=CENPB |
| ATG5 | Autophagy Related 5 | Protein Coding | 42 | GC06M 106045 | 4.350 111 | https://www.genecards.org/cgi-bin/carddisp.pl?gene=ATG5 |
| CFH | Complement Factor H | Protein Coding | 45 | GC01P 196621 | 4.343 126 | https://www.genecards.org/cgi-bin/carddisp.pl?gene=CFH |
| RORC | RAR Related Orphan Receptor C | Protein Coding | 43 | GC01M 151806 | 4.328 93 | https://www.genecards.org/cgi- |

| | | | | | | |
|---|---|---|---|---|---|---|
| | | | | | | bin/carddisp.pl?gene=RORC |
| CD209 | CD209 Molecule | Protein Coding | 42 | GC19M007739 | 4.326128 | https://www.genecards.org/cgi-bin/carddisp.pl?gene=CD209 |
| FCN2 | Ficolin 2 | Protein Coding | 42 | GC09P134864 | 4.325276 | https://www.genecards.org/cgi-bin/carddisp.pl?gene=FCN2 |
| CD68 | CD68 Molecule | Protein Coding | 38 | GC17P007579 | 4.32193 | https://www.genecards.org/cgi-bin/carddisp.pl?gene=CD68 |
| SLC2A9 | Solute Carrier Family 2 Member 9 | Protein Coding | 44 | GC04M009772 | 4.316371 | https://www.genecards.org/cgi-bin/carddisp.pl?gene=SLC2A9 |
| COG6 | Component Of Oligomeric Golgi Complex 6 | Protein Coding | 38 | GC13P039655 | 4.313921 | https://www.genecards.org/cgi-bin/carddisp.pl?gene=COG6 |
| TLR8 | Toll Like Receptor 8 | Protein Coding | 46 | GC0XP012924 | 4.307923 | https://www.genecards.org/cgi-bin/carddisp.pl?gene=TLR8 |
| SIRT1 | Sirtuin 1 | Protein Coding | 46 | GC10P067884 | 4.305996 | https://www.genecards.org/cgi-bin/carddisp.pl?gene=SIRT1 |
| GSN | Gelsolin | Protein Coding | 46 | GC09P121201 | 4.298401 | https://www.genecards.org/cgi-bin/carddisp.pl?gene=GSN |
| GGT1 | Gamma-Glutamyltransferase 1 | Protein Coding | 48 | GC22P024583 | 4.295577 | https://www.genecards.org/cgi-bin/carddisp.pl?gene=GGT1 |
| IDO1 | Indoleamine 2,3-Dioxygenase 1 | Protein Coding | 43 | GC08P039891 | 4.293201 | https://www.genecards.org/cgi-bin/carddisp.pl?gene=IDO1 |
| ADAM10 | ADAM Metallopeptidase Domain 10 | Protein Coding | 50 | GC15M058588 | 4.287847 | https://www.genecards.org/cgi-bin/carddisp.pl?gene=ADAM10 |
| IFNA2 | Interferon Alpha 2 | Protein Coding | 41 | GC09M021384 | 4.281237 | https://www.genecards.org/cgi-bin/carddisp.pl?gene=IFNA2 |
| KDR | Kinase Insert Domain Receptor | Protein Coding | 51 | GC04M055078 | 4.277594 | https://www.genecards.org/cgi-bin/carddisp.pl?gene=KDR |

| | | | | | | |
|---|---|---|---|---|---|---|
| IGHE | Immunoglobulin Heavy Constant Epsilon | Protein Coding | 30 | GC14M 112707 | 4.276 135 | https://www.genecards.org/cgi-bin/carddisp.pl?gene=IGHE |
| DNTT | DNA Nucleotidylexotransferase | Protein Coding | 40 | GC10P 096304 | 4.272 124 | https://www.genecards.org/cgi-bin/carddisp.pl?gene=DNTT |
| CD70 | CD70 Molecule | Protein Coding | 41 | GC19M 006647 | 4.270 958 | https://www.genecards.org/cgi-bin/carddisp.pl?gene=CD70 |
| CCL22 | C-C Motif Chemokine Ligand 22 | Protein Coding | 36 | GC16P 057359 | 4.266 844 | https://www.genecards.org/cgi-bin/carddisp.pl?gene=CCL22 |
| PRDX5 | Peroxiredoxin 5 | Protein Coding | 43 | GC11P 064370 | 4.266 634 | https://www.genecards.org/cgi-bin/carddisp.pl?gene=PRDX5 |
| TSLP | Thymic Stromal Lymphopoietin | Protein Coding | 37 | GC05P 111070 | 4.266 327 | https://www.genecards.org/cgi-bin/carddisp.pl?gene=TSLP |
| NAGLU | N-Acetyl-Alpha-Glucosaminidase | Protein Coding | 41 | GC17P 055669 | 4.265 631 | https://www.genecards.org/cgi-bin/carddisp.pl?gene=NAGLU |
| ADAD1 | Adenosine Deaminase Domain Containing 1 | Protein Coding | 36 | GC04P 122378 | 4.263 219 | https://www.genecards.org/cgi-bin/carddisp.pl?gene=ADAD1 |
| SERPINC 1 | Serpin Family C Member 1 | Protein Coding | 47 | GC01M 174559 | 4.260 211 | https://www.genecards.org/cgi-bin/carddisp.pl?gene=SERPINC1 |
| EPCAM | Epithelial Cell Adhesion Molecule | Protein Coding | 46 | GC02P 047345 | 4.258 68 | https://www.genecards.org/cgi-bin/carddisp.pl?gene=EPCAM |
| CSN1S1 | Casein Alpha S1 | Protein Coding | 36 | GC04P 069932 | 4.255 772 | https://www.genecards.org/cgi-bin/carddisp.pl?gene=CSN1S1 |
| IL15RA | Interleukin 15 Receptor Subunit Alpha | Protein Coding | 39 | GC10M 005943 | 4.250 093 | https://www.genecards.org/cgi-bin/carddisp.pl?gene=IL15RA |
| IL24 | Interleukin 24 | Protein Coding | 39 | GC01P 206897 | 4.243 191 | https://www.genecards.org/cgi-bin/carddisp.pl?gene=IL24 |
| CCR8 | C-C Motif Chemokine Receptor 8 | Protein Coding | 41 | GC03P 039886 | 4.226 926 | https://www.genecards.org/cgi- |

| | | | | | | |
|---|---|---|---|---|---|---|
| | | | | | | bin/carddisp.pl?gene=CCR8 |
| UBA1 | Ubiquitin Like Modifier Activating Enzyme 1 | Protein Coding | 45 | GC0XP047190 | 4.226644 | https://www.genecards.org/cgi-bin/carddisp.pl?gene=UBA1 |
| ALOX15 | Arachidonate 15-Lipoxygenase | Protein Coding | 44 | GC17M004630 | 4.225246 | https://www.genecards.org/cgi-bin/carddisp.pl?gene=ALOX15 |
| CFB | Complement Factor B | Protein Coding | 45 | GC06P031945 | 4.203203 | https://www.genecards.org/cgi-bin/carddisp.pl?gene=CFB |
| LSM2 | LSM2 Homolog, U6 Small Nuclear RNA And MRNA Degradation Associated | Protein Coding | 38 | GC06M065974 | 4.192565 | https://www.genecards.org/cgi-bin/carddisp.pl?gene=LSM2 |
| TYR | Tyrosinase | Protein Coding | 46 | GC11P089177 | 4.188845 | https://www.genecards.org/cgi-bin/carddisp.pl?gene=TYR |
| CD1D | CD1d Molecule | Protein Coding | 42 | GC01P158178 | 4.188049 | https://www.genecards.org/cgi-bin/carddisp.pl?gene=CD1D |
| PVT1 | Pvt1 Oncogene | RNA Gene | 26 | GC08P127869 | 4.187071 | https://www.genecards.org/cgi-bin/carddisp.pl?gene=PVT1 |
| RARRES2 | Retinoic Acid Receptor Responder 2 | Protein Coding | 38 | GC07M150333 | 4.179746 | https://www.genecards.org/cgi-bin/carddisp.pl?gene=RARRES2 |
| ATOD1 | Dermatitis, Atopic | Genetic Locus | 2 | GC03U902187 | 4.165978 | https://www.genecards.org/cgi-bin/carddisp.pl?gene=ATOD1 |
| ATOD3 | Dermatitis, Atopic, 3 | Genetic Locus | 2 | GC20U990080 | 4.165978 | https://www.genecards.org/cgi-bin/carddisp.pl?gene=ATOD3 |
| ATOD5 | Dermatitis, Atopic, 5 | Genetic Locus | 2 | GC13U900216 | 4.165978 | https://www.genecards.org/cgi-bin/carddisp.pl?gene=ATOD5 |
| ATOD6 | Dermatitis, Atopic, 6 | Genetic Locus | 2 | GC05U990174 | 4.165978 | https://www.genecards.org/cgi-bin/carddisp.pl?gene=ATOD6 |
| DPEP1 | Dipeptidase 1 | Protein Coding | 41 | GC16P089613 | 4.158097 | https://www.genecards.org/cgi-bin/carddisp.pl?gene=DPEP1 |

| | | | | | | |
|---|---|---|---|---|---|---|
| GBA | Glucosylceramidase Beta | Protein Coding | 48 | GC01M 155234 | 4.152 47 | https://www.genecards .org/cgi- bin/carddisp.pl?gene= GBA |
| HRH4 | Histamine Receptor H4 | Protein Coding | 44 | GC18P 024460 | 4.148 339 | https://www.genecards .org/cgi- bin/carddisp.pl?gene= HRH4 |
| NEAT1 | Nuclear Paraspeckle Assembly Transcript 1 | RNA Gene | 24 | GC11P 069943 | 4.134 098 | https://www.genecards .org/cgi- bin/carddisp.pl?gene= NEAT1 |
| THBS1 | Thrombospondin 1 | Protein Coding | 44 | GC15P 039581 | 4.133 282 | https://www.genecards .org/cgi- bin/carddisp.pl?gene= THBS1 |
| PDGFB | Platelet Derived Growth Factor Subunit B | Protein Coding | 47 | GC22M 058085 | 4.116 092 | https://www.genecards .org/cgi- bin/carddisp.pl?gene= PDGFB |
| CCL26 | C-C Motif Chemokine Ligand 26 | Protein Coding | 36 | GC07M 075769 | 4.114 658 | https://www.genecards .org/cgi- bin/carddisp.pl?gene= CCL26 |
| MEG3 | Maternally Expressed 3 | RNA Gene | 29 | GC14P 109962 | 4.104 683 | https://www.genecards .org/cgi- bin/carddisp.pl?gene= MEG3 |
| ADAMT S12 | ADAM Metallopeptidase With Thrombospondin Type 1 Motif 12 | Protein Coding | 40 | GC05M 033524 | 4.103 42 | https://www.genecards .org/cgi- bin/carddisp.pl?gene= ADAMTS12 |
| TNFRSF8 | TNF Receptor Superfamily Member 8 | Protein Coding | 41 | GC01P 012063 | 4.098 128 | https://www.genecards .org/cgi- bin/carddisp.pl?gene= TNFRSF8 |
| CHUK | Component Of Inhibitor Of Nuclear Factor Kappa B Kinase Complex | Protein Coding | 49 | GC10M 100188 | 4.098 116 | https://www.genecards .org/cgi- bin/carddisp.pl?gene= CHUK |
| LGALS2 | Galectin 2 | Protein Coding | 40 | GC22M 037570 | 4.093 354 | https://www.genecards .org/cgi- bin/carddisp.pl?gene= LGALS2 |
| ABCC1 | ATP Binding Cassette Subfamily C Member 1 | Protein Coding | 47 | GC16P 015949 | 4.084 396 | https://www.genecards .org/cgi- bin/carddisp.pl?gene= ABCC1 |
| ADGRE5 | Adhesion G Protein-Coupled Receptor E5 | Protein Coding | 38 | GC19P 014392 | 4.077 285 | https://www.genecards .org/cgi- bin/carddisp.pl?gene= ADGRE5 |

| | | | | | | |
|---|---|---|---|---|---|---|
| YDJC | YdjC Chitooligosaccharide Deacetylase Homolog | Protein Coding | 32 | GC22M 021628 | 4.067 303 | https://www.genecards .org/cgi-bin/carddisp.pl?gene= YDJC |
| TPO | Thyroid Peroxidase | Protein Coding | 47 | GC02P 001374 | 4.065 034 | https://www.genecards .org/cgi-bin/carddisp.pl?gene= TPO |
| CXCR1 | C-X-C Motif Chemokine Receptor 1 | Protein Coding | 41 | GC02M 218162 | 4.063 628 | https://www.genecards .org/cgi-bin/carddisp.pl?gene= CXCR1 |
| COL5A2 | Collagen Type V Alpha 2 Chain | Protein Coding | 42 | GC02M 189031 | 4.055 742 | https://www.genecards .org/cgi-bin/carddisp.pl?gene= COL5A2 |
| NT5E | 5'-Nucleotidase Ecto | Protein Coding | 49 | GC06P 085449 | 4.054 715 | https://www.genecards .org/cgi-bin/carddisp.pl?gene= NT5E |
| GTF2I | General Transcription Factor IIi | Protein Coding | 40 | GC07P 075448 | 4.054 162 | https://www.genecards .org/cgi-bin/carddisp.pl?gene= GTF2I |
| LBH | LBH Regulator Of WNT Signaling Pathway | Protein Coding | 34 | GC02P 030231 | 4.052 624 | https://www.genecards .org/cgi-bin/carddisp.pl?gene= LBH |
| POU3F1 | POU Class 3 Homeobox 1 | Protein Coding | 37 | GC01M 038095 | 4.051 453 | https://www.genecards .org/cgi-bin/carddisp.pl?gene= POU3F1 |
| ANPEP | Alanyl Aminopeptidase, Membrane | Protein Coding | 48 | GC15M 089784 | 4.039 893 | https://www.genecards .org/cgi-bin/carddisp.pl?gene= ANPEP |
| LCK | LCK Proto-Oncogene, Src Family Tyrosine Kinase | Protein Coding | 50 | GC01P 032251 | 4.039 072 | https://www.genecards .org/cgi-bin/carddisp.pl?gene= LCK |
| B3GNT2 | UDP-GlcNAc:BetaGal Beta-1,3-N-Acetylglucosaminyltransfe rase 2 | Protein Coding | 38 | GC02P 062196 | 4.034 41 | https://www.genecards .org/cgi-bin/carddisp.pl?gene= B3GNT2 |
| IGF1R | Insulin Like Growth Factor 1 Receptor | Protein Coding | 52 | GC15P 098648 | 4.034 027 | https://www.genecards .org/cgi-bin/carddisp.pl?gene=I GF1R |
| ADRB3 | Adrenoceptor Beta 3 | Protein Coding | 43 | GC08M 037962 | 4.005 707 | https://www.genecards .org/cgi-bin/carddisp.pl?gene= ADRB3 |
| WDFY4 | WDFY Family Member 4 | Protein Coding | 34 | GC10P 048684 | 4.004 28 | https://www.genecards .org/cgi- |

| | | | | | | |
|---|---|---|---|---|---|---|
| | | | | | | bin/carddisp.pl?gene=WDFY4 |
| PTPN12 | Protein Tyrosine Phosphatase Non-Receptor Type 12 | Protein Coding | 46 | GC07P 077537 | 4.002 909 | https://www.genecards.org/cgi-bin/carddisp.pl?gene=PTPN12 |
| RHOA | Ras Homolog Family Member A | Protein Coding | 46 | GC03M 049359 | 3.999 915 | https://www.genecards.org/cgi-bin/carddisp.pl?gene=RHOA |
| PGF | Placental Growth Factor | Protein Coding | 41 | GC14M 074941 | 3.988 466 | https://www.genecards.org/cgi-bin/carddisp.pl?gene=PGF |
| COL9A1 | Collagen Type IX Alpha 1 Chain | Protein Coding | 41 | GC06M 070215 | 3.982 416 | https://www.genecards.org/cgi-bin/carddisp.pl?gene=COL9A1 |
| CD38 | CD38 Molecule | Protein Coding | 44 | GC04P 018197 | 3.981 544 | https://www.genecards.org/cgi-bin/carddisp.pl?gene=CD38 |
| PDGFRB | Platelet Derived Growth Factor Receptor Beta | Protein Coding | 52 | GC05M 150113 | 3.979 126 | https://www.genecards.org/cgi-bin/carddisp.pl?gene=PDGFRB |
| IFI16 | Interferon Gamma Inducible Protein 16 | Protein Coding | 41 | GC01P 158969 | 3.950 812 | https://www.genecards.org/cgi-bin/carddisp.pl?gene=IFI16 |
| MBP | Myelin Basic Protein | Protein Coding | 44 | GC18M 076978 | 3.950 043 | https://www.genecards.org/cgi-bin/carddisp.pl?gene=MBP |
| MET | MET Proto-Oncogene, Receptor Tyrosine Kinase | Protein Coding | 51 | GC07P 116672 | 3.946 303 | https://www.genecards.org/cgi-bin/carddisp.pl?gene=MET |
| TIMP3 | TIMP Metallopeptidase Inhibitor 3 | Protein Coding | 43 | GC22P 036985 | 3.945 199 | https://www.genecards.org/cgi-bin/carddisp.pl?gene=TIMP3 |
| GJB6 | Gap Junction Protein Beta 6 | Protein Coding | 42 | GC13M 020221 | 3.939 862 | https://www.genecards.org/cgi-bin/carddisp.pl?gene=GJB6 |
| CREM | CAMP Responsive Element Modulator | Protein Coding | 40 | GC10P 035126 | 3.939 312 | https://www.genecards.org/cgi-bin/carddisp.pl?gene=CREM |
| FADD | Fas Associated Via Death Domain | Protein Coding | 44 | GC11P 070203 | 3.923 491 | https://www.genecards.org/cgi-bin/carddisp.pl?gene=FADD |

| | | | | | | |
|---|---|---|---|---|---|---|
| SAAL1 | Serum Amyloid A Like 1 | Protein Coding | 32 | GC11M 018069 | 3.923 332 | https://www.genecards .org/cgi-bin/carddisp.pl?gene= SAAL1 |
| SMAD2 | SMAD Family Member 2 | Protein Coding | 48 | GC18M 047809 | 3.923 233 | https://www.genecards .org/cgi-bin/carddisp.pl?gene= SMAD2 |
| C5AR1 | Complement C5a Receptor 1 | Protein Coding | 42 | GC19P 047290 | 3.919 319 | https://www.genecards .org/cgi-bin/carddisp.pl?gene= C5AR1 |
| SPARC | Secreted Protein Acidic And Cysteine Rich | Protein Coding | 48 | GC05M 151661 | 3.917 453 | https://www.genecards .org/cgi-bin/carddisp.pl?gene= SPARC |
| RPA3 | Replication Protein A3 | Protein Coding | 40 | GC07M 008189 | 3.912 56 | https://www.genecards .org/cgi-bin/carddisp.pl?gene= RPA3 |
| TDRKH | Tudor And KH Domain Containing | Protein Coding | 38 | GC01M 152176 | 3.912 56 | https://www.genecards .org/cgi-bin/carddisp.pl?gene= TDRKH |
| CSK | C-Terminal Src Kinase | Protein Coding | 45 | GC15P 074782 | 3.907 964 | https://www.genecards .org/cgi-bin/carddisp.pl?gene= CSK |
| PGR | Progesterone Receptor | Protein Coding | 47 | GC11M 101030 | 3.907 366 | https://www.genecards .org/cgi-bin/carddisp.pl?gene= PGR |
| COMT | Catechol-O-Methyltransferase | Protein Coding | 49 | GC22P 019941 | 3.903 66 | https://www.genecards .org/cgi-bin/carddisp.pl?gene= COMT |
| HNRNPA 2B1 | Heterogeneous Nuclear Ribonucleoprotein A2/B1 | Protein Coding | 44 | GC07M 026174 | 3.903 66 | https://www.genecards .org/cgi-bin/carddisp.pl?gene= HNRNPA2B1 |
| XBP1 | X-Box Binding Protein 1 | Protein Coding | 44 | GC22M 028794 | 3.902 87 | https://www.genecards .org/cgi-bin/carddisp.pl?gene= XBP1 |
| TCF3 | Transcription Factor 3 | Protein Coding | 43 | GC19M 001609 | 3.902 445 | https://www.genecards .org/cgi-bin/carddisp.pl?gene= TCF3 |
| F13A1 | Coagulation Factor XIII A Chain | Protein Coding | 45 | GC06M 006144 | 3.898 983 | https://www.genecards .org/cgi-bin/carddisp.pl?gene= F13A1 |
| TGFB2 | Transforming Growth Factor Beta 2 | Protein Coding | 49 | GC01P 218345 | 3.896 17 | https://www.genecards .org/cgi- |

| | | | | | | |
|---|---|---|---|---|---|---|
| | | | | | | bin/carddisp.pl?gene=TGFB2 |
| NFE2L2 | NFE2 Like BZIP Transcription Factor 2 | Protein Coding | 48 | GC02M177227 | 3.889893 | https://www.genecards.org/cgi-bin/carddisp.pl?gene=NFE2L2 |
| LINC01104 | Long Intergenic Non-Protein Coding RNA 1104 | RNA Gene | 14 | GC02P100208 | 3.885221 | https://www.genecards.org/cgi-bin/carddisp.pl?gene=LINC01104 |
| TEC | Tec Protein Tyrosine Kinase | Protein Coding | 44 | GC04M048150 | 3.884967 | https://www.genecards.org/cgi-bin/carddisp.pl?gene=TEC |
| FAM167A | Family With Sequence Similarity 167 Member A | Protein Coding | 34 | GC08M011421 | 3.88454 | https://www.genecards.org/cgi-bin/carddisp.pl?gene=FAM167A |
| KIR2DL3 | Killer Cell Immunoglobulin Like Receptor, Two Ig Domains And Long Cytoplasmic Tail 3 | Protein Coding | 36 | GC19P068014 | 3.877167 | https://www.genecards.org/cgi-bin/carddisp.pl?gene=KIR2DL3 |
| DUSP1 | Dual Specificity Phosphatase 1 | Protein Coding | 45 | GC05M172768 | 3.876289 | https://www.genecards.org/cgi-bin/carddisp.pl?gene=DUSP1 |
| LIN54 | Lin-54 DREAM MuvB Core Complex Component | Protein Coding | 36 | GC04M082909 | 3.87335 | https://www.genecards.org/cgi-bin/carddisp.pl?gene=LIN54 |
| ZNF354A | Zinc Finger Protein 354A | Protein Coding | 36 | GC05M178711 | 3.87335 | https://www.genecards.org/cgi-bin/carddisp.pl?gene=ZNF354A |
| USP50 | Ubiquitin Specific Peptidase 50 | Protein Coding | 32 | GC15M050493 | 3.87335 | https://www.genecards.org/cgi-bin/carddisp.pl?gene=USP50 |
| FRG2C | FSHD Region Gene 2 Family Member C | Protein Coding | 24 | GC03P075664 | 3.87335 | https://www.genecards.org/cgi-bin/carddisp.pl?gene=FRG2C |
| IRF2BP2 | Interferon Regulatory Factor 2 Binding Protein 2 | Protein Coding | 36 | GC01M234604 | 3.86865 | https://www.genecards.org/cgi-bin/carddisp.pl?gene=IRF2BP2 |
| AIS2 | Autoimmune Susceptibility 2 | Genetic Locus | 2 | GC07U901482 | 3.86865 | https://www.genecards.org/cgi-bin/carddisp.pl?gene=AIS2 |
| AIS3 | Autoimmune Susceptibility 3 (Vitiligo Specific) | Genetic Locus | 2 | GC08U900492 | 3.86865 | https://www.genecards.org/cgi- |

| | | | | | | |
|---|---|---|---|---|---|---|
| | | | | | | bin/carddisp.pl?gene=AIS3 |
| AIS4 | Autoimmune Disease, Susceptibility To, 4 | Genetic Locus | 2 | GC04U900482 | 3.86865 | https://www.genecards.org/cgi-bin/carddisp.pl?gene=AIS4 |
| ATOD7 | Dermatitis, Atopic, Susceptibility To, 7 | Genetic Locus | 1 | GC11U901436 | 3.86865 | https://www.genecards.org/cgi-bin/carddisp.pl?gene=ATOD7 |
| ATOD8 | Dermatitis, Atopic, 8 | Genetic Locus | 1 | GC04U902095 | 3.86865 | https://www.genecards.org/cgi-bin/carddisp.pl?gene=ATOD8 |
| ATOD9 | Dermatitis, Atopic, 9 | Genetic Locus | 1 | GC03U902459 | 3.86865 | https://www.genecards.org/cgi-bin/carddisp.pl?gene=ATOD9 |
| TYRO3 | TYRO3 Protein Tyrosine Kinase | Protein Coding | 46 | GC15P041557 | 3.866479 | https://www.genecards.org/cgi-bin/carddisp.pl?gene=TYRO3 |
| ALPL | Alkaline Phosphatase, Biomineralization Associated | Protein Coding | 49 | GC01P021508 | 3.861889 | https://www.genecards.org/cgi-bin/carddisp.pl?gene=ALPL |
| IGF2 | Insulin Like Growth Factor 2 | Protein Coding | 46 | GC11M003294 | 3.855555 | https://www.genecards.org/cgi-bin/carddisp.pl?gene=IGF2 |
| ALPP | Alkaline Phosphatase, Placental | Protein Coding | 44 | GC02P232378 | 3.853659 | https://www.genecards.org/cgi-bin/carddisp.pl?gene=ALPP |
| IRF3 | Interferon Regulatory Factor 3 | Protein Coding | 44 | GC19M049659 | 3.852698 | https://www.genecards.org/cgi-bin/carddisp.pl?gene=IRF3 |
| CLU | Clusterin | Protein Coding | 45 | GC08M027596 | 3.852247 | https://www.genecards.org/cgi-bin/carddisp.pl?gene=CLU |
| TBX1 | T-Box Transcription Factor 1 | Protein Coding | 41 | GC22P035812 | 3.841673 | https://www.genecards.org/cgi-bin/carddisp.pl?gene=TBX1 |
| GNAS | GNAS Complex Locus | Protein Coding | 49 | GC20P058839 | 3.83968 | https://www.genecards.org/cgi-bin/carddisp.pl?gene=GNAS |
| LOC102723407 | Immunoglobulin Heavy Variable 4-38-2-Like | Protein Coding | 6 | GC14P8B0041 | 3.839319 | https://www.genecards.org/cgi-bin/carddisp.pl?gene=LOC102723407 |

| | | | | | | |
|---|---|---|---|---|---|---|
| ELMO1 | Engulfment And Cell Motility 1 | Protein Coding | 39 | GC07M 036860 | 3.834 863 | https://www.genecards .org/cgi-bin/carddisp.pl?gene= ELMO1 |
| TRPS1 | Transcriptional Repressor GATA Binding 1 | Protein Coding | 45 | GC08M 115408 | 3.828 751 | https://www.genecards .org/cgi-bin/carddisp.pl?gene= TRPS1 |
| SLC22A1 2 | Solute Carrier Family 22 Member 12 | Protein Coding | 43 | GC11P 064609 | 3.827 631 | https://www.genecards .org/cgi-bin/carddisp.pl?gene= SLC22A12 |
| F5 | Coagulation Factor V | Protein Coding | 45 | GC01M 169511 | 3.820 146 | https://www.genecards .org/cgi-bin/carddisp.pl?gene= F5 |
| IFNGR2 | Interferon Gamma Receptor 2 | Protein Coding | 42 | GC21P 033402 | 3.816 897 | https://www.genecards .org/cgi-bin/carddisp.pl?gene=I FNGR2 |
| AICDA | Activation Induced Cytidine Deaminase | Protein Coding | 42 | GC12M 008602 | 3.806 782 | https://www.genecards .org/cgi-bin/carddisp.pl?gene= AICDA |
| TMSB4X | Thymosin Beta 4 X-Linked | Protein Coding | 39 | GC0XP 012975 | 3.805 577 | https://www.genecards .org/cgi-bin/carddisp.pl?gene= TMSB4X |
| FRZB | Frizzled Related Protein | Protein Coding | 40 | GC02M 182833 | 3.797 774 | https://www.genecards .org/cgi-bin/carddisp.pl?gene= FRZB |
| CDK2 | Cyclin Dependent Kinase 2 | Protein Coding | 49 | GC12P 055966 | 3.792 599 | https://www.genecards .org/cgi-bin/carddisp.pl?gene= CDK2 |
| MAPK10 | Mitogen-Activated Protein Kinase 10 | Protein Coding | 48 | GC04M 085990 | 3.789 578 | https://www.genecards .org/cgi-bin/carddisp.pl?gene= MAPK10 |
| EP300 | E1A Binding Protein P300 | Protein Coding | 49 | GC22P 041091 | 3.788 47 | https://www.genecards .org/cgi-bin/carddisp.pl?gene= EP300 |
| BACH2 | BTB Domain And CNC Homolog 2 | Protein Coding | 42 | GC06M 089926 | 3.787 713 | https://www.genecards .org/cgi-bin/carddisp.pl?gene= BACH2 |
| LGALS3 BP | Galectin 3 Binding Protein | Protein Coding | 41 | GC17M 078971 | 3.787 081 | https://www.genecards .org/cgi-bin/carddisp.pl?gene= LGALS3BP |
| MIR17 | MicroRNA 17 | RNA Gene | 20 | GC13P 091350 | 3.775 56 | https://www.genecards .org/cgi- |

| | | | | | | |
|---|---|---|---|---|---|---|
| | | | | | | bin/carddisp.pl?gene=MIR17 |
| BMP7 | Bone Morphogenetic Protein 7 | Protein Coding | 44 | GC20M057168 | 3.773507 | https://www.genecards.org/cgi-bin/carddisp.pl?gene=BMP7 |
| ARL15 | ADP Ribosylation Factor Like GTPase 15 | Protein Coding | 34 | GC05M053883 | 3.772785 | https://www.genecards.org/cgi-bin/carddisp.pl?gene=ARL15 |
| GAST | Gastrin | Protein Coding | 38 | GC17P041712 | 3.769227 | https://www.genecards.org/cgi-bin/carddisp.pl?gene=GAST |
| CD276 | CD276 Molecule | Protein Coding | 40 | GC15P073683 | 3.767734 | https://www.genecards.org/cgi-bin/carddisp.pl?gene=CD276 |
| ADORA2A | Adenosine A2a Receptor | Protein Coding | 44 | GC22P024417 | 3.764112 | https://www.genecards.org/cgi-bin/carddisp.pl?gene=ADORA2A |
| MAP3K14 | Mitogen-Activated Protein Kinase Kinase Kinase 14 | Protein Coding | 42 | GC17M045263 | 3.763119 | https://www.genecards.org/cgi-bin/carddisp.pl?gene=MAP3K14 |
| PSORS1C1 | Psoriasis Susceptibility 1 Candidate 1 | Protein Coding | 30 | GC06P031114 | 3.762619 | https://www.genecards.org/cgi-bin/carddisp.pl?gene=PSORS1C1 |
| CTSL | Cathepsin L | Protein Coding | 45 | GC09P087725 | 3.761764 | https://www.genecards.org/cgi-bin/carddisp.pl?gene=CTSL |
| PTHLH | Parathyroid Hormone Like Hormone | Protein Coding | 45 | GC12M027959 | 3.761654 | https://www.genecards.org/cgi-bin/carddisp.pl?gene=PTHLH |
| CXCL2 | C-X-C Motif Chemokine Ligand 2 | Protein Coding | 38 | GC04M074097 | 3.75755 | https://www.genecards.org/cgi-bin/carddisp.pl?gene=CXCL2 |
| ARID5B | AT-Rich Interaction Domain 5B | Protein Coding | 40 | GC10P061901 | 3.745826 | https://www.genecards.org/cgi-bin/carddisp.pl?gene=ARID5B |
| TNPO3 | Transportin 3 | Protein Coding | 40 | GC07M128954 | 3.745826 | https://www.genecards.org/cgi-bin/carddisp.pl?gene=TNPO3 |
| SSB | Small RNA Binding Exonuclease Protection Factor La | Protein Coding | 41 | GC02P169791 | 3.73923 | https://www.genecards.org/cgi-bin/carddisp.pl?gene=SSB |

| | | | | | | |
|---|---|---|---|---|---|---|
| MCL1 | MCL1 Apoptosis Regulator, BCL2 Family Member | Protein Coding | 45 | GC01M 152066 | 3.725 513 | https://www.genecards .org/cgi-bin/carddisp.pl?gene= MCL1 |
| VAV1 | Vav Guanine Nucleotide Exchange Factor 1 | Protein Coding | 44 | GC19P 006772 | 3.723 102 | https://www.genecards .org/cgi-bin/carddisp.pl?gene= VAV1 |
| PLD4 | Phospholipase D Family Member 4 | Protein Coding | 38 | GC14P 104924 | 3.722 507 | https://www.genecards .org/cgi-bin/carddisp.pl?gene= PLD4 |
| HDAC1 | Histone Deacetylase 1 | Protein Coding | 47 | GC01P 032292 | 3.696 424 | https://www.genecards .org/cgi-bin/carddisp.pl?gene= HDAC1 |
| KLRD1 | Killer Cell Lectin Like Receptor D1 | Protein Coding | 41 | GC12P 010226 | 3.693 325 | https://www.genecards .org/cgi-bin/carddisp.pl?gene= KLRD1 |
| FCN3 | Ficolin 3 | Protein Coding | 44 | GC01M 027843 | 3.692 076 | https://www.genecards .org/cgi-bin/carddisp.pl?gene= FCN3 |
| MIR221 | MicroRNA 221 | RNA Gene | 21 | GC0X M0457 46 | 3.690 581 | https://www.genecards .org/cgi-bin/carddisp.pl?gene= MIR221 |
| GSR | Glutathione-Disulfide Reductase | Protein Coding | 48 | GC08M 030678 | 3.690 055 | https://www.genecards .org/cgi-bin/carddisp.pl?gene= GSR |
| KLF12 | Kruppel Like Factor 12 | Protein Coding | 37 | GC13M 073686 | 3.678 336 | https://www.genecards .org/cgi-bin/carddisp.pl?gene= KLF12 |
| AIM2 | Absent In Melanoma 2 | Protein Coding | 40 | GC01M 159062 | 3.673 544 | https://www.genecards .org/cgi-bin/carddisp.pl?gene= AIM2 |
| LINC026 56 | Long Intergenic Non-Protein Coding RNA 2656 | RNA Gene | 10 | GC10P 006350 | 3.673 22 | https://www.genecards .org/cgi-bin/carddisp.pl?gene= LINC02656 |
| ADM | Adrenomedullin | Protein Coding | 43 | GC11P 010304 | 3.673 102 | https://www.genecards .org/cgi-bin/carddisp.pl?gene= ADM |
| HSPG2 | Heparan Sulfate Proteoglycan 2 | Protein Coding | 46 | GC01M 021822 | 3.671 193 | https://www.genecards .org/cgi-bin/carddisp.pl?gene= HSPG2 |
| DNMT1 | DNA Methyltransferase 1 | Protein Coding | 49 | GC19M 010133 | 3.669 86 | https://www.genecards .org/cgi- |

| | | | | | | |
|---|---|---|---|---|---|---|
| | | | | | | bin/carddisp.pl?gene=DNMT1 |
| PPBP | Pro-Platelet Basic Protein | Protein Coding | 41 | GC04M073986 | 3.669165 | https://www.genecards.org/cgi-bin/carddisp.pl?gene=PPBP |
| EGR1 | Early Growth Response 1 | Protein Coding | 42 | GC05P138465 | 3.668759 | https://www.genecards.org/cgi-bin/carddisp.pl?gene=EGR1 |
| SAMHD1 | SAM And HD Domain Containing Deoxynucleoside Triphosphate Triphosphohydrolase 1 | Protein Coding | 41 | GC20M036890 | 3.6658 | https://www.genecards.org/cgi-bin/carddisp.pl?gene=SAMHD1 |
| LPA | Lipoprotein(A) | Protein Coding | 40 | GC06M160531 | 3.664589 | https://www.genecards.org/cgi-bin/carddisp.pl?gene=LPA |
| THPO | Thrombopoietin | Protein Coding | 42 | GC03M184371 | 3.663482 | https://www.genecards.org/cgi-bin/carddisp.pl?gene=THPO |
| TGIF1 | TGFB Induced Factor Homeobox 1 | Protein Coding | 44 | GC18P003411 | 3.663274 | https://www.genecards.org/cgi-bin/carddisp.pl?gene=TGIF1 |
| LGALS1 | Galectin 1 | Protein Coding | 41 | GC22P037675 | 3.658767 | https://www.genecards.org/cgi-bin/carddisp.pl?gene=LGALS1 |
| MAPKAPK2 | MAPK Activated Protein Kinase 2 | Protein Coding | 46 | GC01P206684 | 3.656003 | https://www.genecards.org/cgi-bin/carddisp.pl?gene=MAPKAPK2 |
| CREB1 | CAMP Responsive Element Binding Protein 1 | Protein Coding | 47 | GC02P207529 | 3.655792 | https://www.genecards.org/cgi-bin/carddisp.pl?gene=CREB1 |
| ERBB3 | Erb-B2 Receptor Tyrosine Kinase 3 | Protein Coding | 51 | GC12P057264 | 3.653791 | https://www.genecards.org/cgi-bin/carddisp.pl?gene=ERBB3 |
| MIR22 | MicroRNA 22 | RNA Gene | 21 | GC17M001713 | 3.651958 | https://www.genecards.org/cgi-bin/carddisp.pl?gene=MIR22 |
| CDK4 | Cyclin Dependent Kinase 4 | Protein Coding | 50 | GC12M057743 | 3.645252 | https://www.genecards.org/cgi-bin/carddisp.pl?gene=CDK4 |
| FGFR3 | Fibroblast Growth Factor Receptor 3 | Protein Coding | 52 | GC04P001795 | 3.64524 | https://www.genecards.org/cgi- |

| | | | | | | |
|---|---|---|---|---|---|---|
| | | | | | | bin/carddisp.pl?gene=FGFR3 |
| PHTF1 | Putative Homeodomain Transcription Factor 1 | Protein Coding | 34 | GC01M113696 | 3.634562 | https://www.genecards.org/cgi-bin/carddisp.pl?gene=PHTF1 |
| MAP2K4 | Mitogen-Activated Protein Kinase Kinase 4 | Protein Coding | 45 | GC17P012020 | 3.628612 | https://www.genecards.org/cgi-bin/carddisp.pl?gene=MAP2K4 |
| AHSG | Alpha 2-HS Glycoprotein | Protein Coding | 42 | GC03P186629 | 3.618032 | https://www.genecards.org/cgi-bin/carddisp.pl?gene=AHSG |
| BAX | BCL2 Associated X, Apoptosis Regulator | Protein Coding | 47 | GC19P048954 | 3.615888 | https://www.genecards.org/cgi-bin/carddisp.pl?gene=BAX |
| UNC13D | Unc-13 Homolog D | Protein Coding | 43 | GC17M075827 | 3.613799 | https://www.genecards.org/cgi-bin/carddisp.pl?gene=UNC13D |
| TIE1 | Tyrosine Kinase With Immunoglobulin Like And EGF Like Domains 1 | Protein Coding | 42 | GC01P043300 | 3.609891 | https://www.genecards.org/cgi-bin/carddisp.pl?gene=TIE1 |
| RIPK2 | Receptor Interacting Serine/Threonine Kinase 2 | Protein Coding | 44 | GC08P089804 | 3.609241 | https://www.genecards.org/cgi-bin/carddisp.pl?gene=RIPK2 |
| IREB2 | Iron Responsive Element Binding Protein 2 | Protein Coding | 43 | GC15P078437 | 3.606062 | https://www.genecards.org/cgi-bin/carddisp.pl?gene=IREB2 |
| CHRM3 | Cholinergic Receptor Muscarinic 3 | Protein Coding | 47 | GC01P239386 | 3.602444 | https://www.genecards.org/cgi-bin/carddisp.pl?gene=CHRM3 |
| ETS2 | ETS Proto-Oncogene 2, Transcription Factor | Protein Coding | 40 | GC21P038805 | 3.601353 | https://www.genecards.org/cgi-bin/carddisp.pl?gene=ETS2 |
| KIR2DL2 | Killer Cell Immunoglobulin Like Receptor, Two Ig Domains And Long Cytoplasmic Tail 2 | Protein Coding | 25 | GC19Mr00108 | 3.60015 | https://www.genecards.org/cgi-bin/carddisp.pl?gene=KIR2DL2 |
| SDC3 | Syndecan 3 | Protein Coding | 41 | GC01M030869 | 3.591435 | https://www.genecards.org/cgi-bin/carddisp.pl?gene=SDC3 |
| SLCO1B1 | Solute Carrier Organic Anion Transporter Family Member 1B1 | Protein Coding | 46 | GC12P021653 | 3.590797 | https://www.genecards.org/cgi- |

| | | | | | | |
|---|---|---|---|---|---|---|
| | | | | | | bin/carddisp.pl?gene=SLCO1B1 |
| CHST11 | Carbohydrate Sulfotransferase 11 | Protein Coding | 41 | GC12P104455 | 3.585589 | https://www.genecards.org/cgi-bin/carddisp.pl?gene=CHST11 |
| AHR | Aryl Hydrocarbon Receptor | Protein Coding | 47 | GC07P016916 | 3.584531 | https://www.genecards.org/cgi-bin/carddisp.pl?gene=AHR |
| IL18RAP | Interleukin 18 Receptor Accessory Protein | Protein Coding | 37 | GC02P102418 | 3.584322 | https://www.genecards.org/cgi-bin/carddisp.pl?gene=IL18RAP |
| CAMP | Cathelicidin Antimicrobial Peptide | Protein Coding | 39 | GC03P048954 | 3.583385 | https://www.genecards.org/cgi-bin/carddisp.pl?gene=CAMP |
| ANGPTL4 | Angiopoietin Like 4 | Protein Coding | 43 | GC19P008363 | 3.579341 | https://www.genecards.org/cgi-bin/carddisp.pl?gene=ANGPTL4 |
| SLAMF7 | SLAM Family Member 7 | Protein Coding | 41 | GC01P160740 | 3.577115 | https://www.genecards.org/cgi-bin/carddisp.pl?gene=SLAMF7 |
| MIR34A | MicroRNA 34a | RNA Gene | 23 | GC01M009151 | 3.575555 | https://www.genecards.org/cgi-bin/carddisp.pl?gene=MIR34A |
| BMP5 | Bone Morphogenetic Protein 5 | Protein Coding | 41 | GC06M055753 | 3.572934 | https://www.genecards.org/cgi-bin/carddisp.pl?gene=BMP5 |
| MMP11 | Matrix Metallopeptidase 11 | Protein Coding | 44 | GC22P023768 | 3.569426 | https://www.genecards.org/cgi-bin/carddisp.pl?gene=MMP11 |
| VEGFC | Vascular Endothelial Growth Factor C | Protein Coding | 45 | GC04M176683 | 3.562657 | https://www.genecards.org/cgi-bin/carddisp.pl?gene=VEGFC |
| PPIA | Peptidylprolyl Isomerase A | Protein Coding | 45 | GC07P044807 | 3.559993 | https://www.genecards.org/cgi-bin/carddisp.pl?gene=PPIA |
| TRAF2 | TNF Receptor Associated Factor 2 | Protein Coding | 42 | GC09P136881 | 3.556872 | https://www.genecards.org/cgi-bin/carddisp.pl?gene=TRAF2 |
| LILRB1 | Leukocyte Immunoglobulin Like Receptor B1 | Protein Coding | 42 | GC19P067376 | 3.553421 | https://www.genecards.org/cgi-bin/carddisp.pl?gene=LILRB1 |

| | | | | | | |
|---|---|---|---|---|---|---|
| RSBN1 | Round Spermatid Basic Protein 1 | Protein Coding | 32 | GC01M 113761 | 3.551 713 | https://www.genecards.org/cgi-bin/carddisp.pl?gene=RSBN1 |
| FCGRT | Fc Gamma Receptor And Transporter | Protein Coding | 42 | GC19P 049506 | 3.550 697 | https://www.genecards.org/cgi-bin/carddisp.pl?gene=FCGRT |
| MAP3K7 | Mitogen-Activated Protein Kinase Kinase Kinase 7 | Protein Coding | 48 | GC06M 090513 | 3.544 87 | https://www.genecards.org/cgi-bin/carddisp.pl?gene=MAP3K7 |
| GP1BB | Glycoprotein Ib Platelet Subunit Beta | Protein Coding | 42 | GC22P 035810 | 3.544 345 | https://www.genecards.org/cgi-bin/carddisp.pl?gene=GP1BB |
| KRT5 | Keratin 5 | Protein Coding | 45 | GC12M 052514 | 3.542 5 | https://www.genecards.org/cgi-bin/carddisp.pl?gene=KRT5 |
| ADAM15 | ADAM Metallopeptidase Domain 15 | Protein Coding | 42 | GC01P 155050 | 3.540 148 | https://www.genecards.org/cgi-bin/carddisp.pl?gene=ADAM15 |
| MAP3K1 | Mitogen-Activated Protein Kinase Kinase Kinase 1 | Protein Coding | 47 | GC05P 056815 | 3.538 762 | https://www.genecards.org/cgi-bin/carddisp.pl?gene=MAP3K1 |
| MIR126 | MicroRNA 126 | RNA Gene | 22 | GC09P 136670 | 3.526 884 | https://www.genecards.org/cgi-bin/carddisp.pl?gene=MIR126 |
| GUSB | Glucuronidase Beta | Protein Coding | 46 | GC07M 065960 | 3.524 533 | https://www.genecards.org/cgi-bin/carddisp.pl?gene=GUSB |
| CLEC3B | C-Type Lectin Domain Family 3 Member B | Protein Coding | 39 | GC03P 046742 | 3.521 83 | https://www.genecards.org/cgi-bin/carddisp.pl?gene=CLEC3B |
| TERT | Telomerase Reverse Transcriptase | Protein Coding | 49 | GC05M 001253 | 3.517 506 | https://www.genecards.org/cgi-bin/carddisp.pl?gene=TERT |
| ANXA2 | Annexin A2 | Protein Coding | 46 | GC15M 060347 | 3.517 336 | https://www.genecards.org/cgi-bin/carddisp.pl?gene=ANXA2 |
| CCL7 | C-C Motif Chemokine Ligand 7 | Protein Coding | 41 | GC17P 034270 | 3.515 968 | https://www.genecards.org/cgi-bin/carddisp.pl?gene=CCL7 |
| IFNL1 | Interferon Lambda 1 | Protein Coding | 33 | GC19P 039296 | 3.509 798 | https://www.genecards.org/cgi- |

| | | | | | | |
|---|---|---|---|---|---|---|
| | | | | | | bin/carddisp.pl?gene=IFNL1 |
| SALL3 | Spalt Like Transcription Factor 3 | Protein Coding | 35 | GC18P078980 | 3.507736 | https://www.genecards.org/cgi-bin/carddisp.pl?gene=SALL3 |
| CXCR6 | C-X-C Motif Chemokine Receptor 6 | Protein Coding | 38 | GC03P046765 | 3.505186 | https://www.genecards.org/cgi-bin/carddisp.pl?gene=CXCR6 |
| MIR30A | MicroRNA 30a | RNA Gene | 20 | GC06M071403 | 3.503458 | https://www.genecards.org/cgi-bin/carddisp.pl?gene=MIR30A |
| TNFRSF12A | TNF Receptor Superfamily Member 12A | Protein Coding | 41 | GC16P003018 | 3.499381 | https://www.genecards.org/cgi-bin/carddisp.pl?gene=TNFRSF12A |
| AKT2 | AKT Serine/Threonine Kinase 2 | Protein Coding | 51 | GC19M040230 | 3.491042 | https://www.genecards.org/cgi-bin/carddisp.pl?gene=AKT2 |
| PFKM | Phosphofructokinase, Muscle | Protein Coding | 48 | GC12P048105 | 3.490663 | https://www.genecards.org/cgi-bin/carddisp.pl?gene=PFKM |
| LERFS | LncRNA Negative Regulator Of Fibroblast-Like Synoviocyte Migration, SYNCRIP Interacting | RNA Gene | 9 | GC09M062859 | 3.490208 | https://www.genecards.org/cgi-bin/carddisp.pl?gene=LERFS |
| BCL6 | BCL6 Transcription Repressor | Protein Coding | 43 | GC03M187721 | 3.487766 | https://www.genecards.org/cgi-bin/carddisp.pl?gene=BCL6 |
| APOB | Apolipoprotein B | Protein Coding | 45 | GC02M020956 | 3.4875 | https://www.genecards.org/cgi-bin/carddisp.pl?gene=APOB |
| KIR2DL1 | Killer Cell Immunoglobulin Like Receptor, Two Ig Domains And Long Cytoplasmic Tail 1 | Protein Coding | 36 | GC19P067381 | 3.486579 | https://www.genecards.org/cgi-bin/carddisp.pl?gene=KIR2DL1 |
| CYBC1 | Cytochrome B-245 Chaperone 1 | Protein Coding | 25 | GC17M082443 | 3.481138 | https://www.genecards.org/cgi-bin/carddisp.pl?gene=CYBC1 |
| HNRNPC | Heterogeneous Nuclear Ribonucleoprotein C | Protein Coding | 40 | GC14M022503 | 3.476026 | https://www.genecards.org/cgi-bin/carddisp.pl?gene=HNRNPC |
| KRT18 | Keratin 18 | Protein Coding | 46 | GC12P052948 | 3.469151 | https://www.genecards.org/cgi- |

| | | | | | | |
|---|---|---|---|---|---|---|
| | | | | | | bin/carddisp.pl?gene=KRT18 |
| HLA-DPA1 | Major Histocompatibility Complex, Class II, DP Alpha 1 | Protein Coding | 40 | GC06M033064 | 3.46679 | https://www.genecards.org/cgi-bin/carddisp.pl?gene=HLA-DPA1 |
| GOLGB1 | Golgin B1 | Protein Coding | 36 | GC03M121663 | 3.465222 | https://www.genecards.org/cgi-bin/carddisp.pl?gene=GOLGB1 |
| ITGAX | Integrin Subunit Alpha X | Protein Coding | 44 | GC16P041137 | 3.465128 | https://www.genecards.org/cgi-bin/carddisp.pl?gene=ITGAX |
| ICAM2 | Intercellular Adhesion Molecule 2 | Protein Coding | 42 | GC17M064002 | 3.453012 | https://www.genecards.org/cgi-bin/carddisp.pl?gene=ICAM2 |
| LGALS8 | Galectin 8 | Protein Coding | 40 | GC01P236518 | 3.446825 | https://www.genecards.org/cgi-bin/carddisp.pl?gene=LGALS8 |
| IFNG-AS1 | IFNG Antisense RNA 1 | RNA Gene | 18 | GC12P067989 | 3.44533 | https://www.genecards.org/cgi-bin/carddisp.pl?gene=IFNG-AS1 |
| MSN | Moesin | Protein Coding | 45 | GC0XP065588 | 3.428742 | https://www.genecards.org/cgi-bin/carddisp.pl?gene=MSN |
| GHSR | Growth Hormone Secretagogue Receptor | Protein Coding | 45 | GC03M172443 | 3.424213 | https://www.genecards.org/cgi-bin/carddisp.pl?gene=GHSR |
| MAP2K6 | Mitogen-Activated Protein Kinase Kinase 6 | Protein Coding | 44 | GC17P069414 | 3.418328 | https://www.genecards.org/cgi-bin/carddisp.pl?gene=MAP2K6 |
| RAC1 | Rac Family Small GTPase 1 | Protein Coding | 47 | GC07P006377 | 3.405939 | https://www.genecards.org/cgi-bin/carddisp.pl?gene=RAC1 |
| SHMT1 | Serine Hydroxymethyltransferase 1 | Protein Coding | 44 | GC17M025713 | 3.405916 | https://www.genecards.org/cgi-bin/carddisp.pl?gene=SHMT1 |
| XRCC1 | X-Ray Repair Cross Complementing 1 | Protein Coding | 43 | GC19M043543 | 3.405916 | https://www.genecards.org/cgi-bin/carddisp.pl?gene=XRCC1 |
| MSH5 | MutS Homolog 5 | Protein Coding | 40 | GC06P083709 | 3.405401 | https://www.genecards.org/cgi-bin/carddisp.pl?gene=MSH5 |

| | | | | | | |
|---|---|---|---|---|---|---|
| YWHAH | Tyrosine 3-Monooxygenase/Tryptophan 5-Monooxygenase Activation Protein Eta | Protein Coding | 45 | GC22P031944 | 3.397234 | https://www.genecards.org/cgi-bin/carddisp.pl?gene=YWHAH |
| ENO2 | Enolase 2 | Protein Coding | 45 | GC12P006913 | 3.397164 | https://www.genecards.org/cgi-bin/carddisp.pl?gene=ENO2 |
| ITLN1 | Intelectin 1 | Protein Coding | 38 | GC01M160876 | 3.395515 | https://www.genecards.org/cgi-bin/carddisp.pl?gene=ITLN1 |
| TLR6 | Toll Like Receptor 6 | Protein Coding | 42 | GC04M038828 | 3.38742 | https://www.genecards.org/cgi-bin/carddisp.pl?gene=TLR6 |
| LAG3 | Lymphocyte Activating 3 | Protein Coding | 38 | GC12P021074 | 3.384944 | https://www.genecards.org/cgi-bin/carddisp.pl?gene=LAG3 |
| HDAC4 | Histone Deacetylase 4 | Protein Coding | 49 | GC02M239048 | 3.382961 | https://www.genecards.org/cgi-bin/carddisp.pl?gene=HDAC4 |
| CARD9 | Caspase Recruitment Domain Family Member 9 | Protein Coding | 42 | GC09M136364 | 3.378241 | https://www.genecards.org/cgi-bin/carddisp.pl?gene=CARD9 |
| CEBPB | CCAAT Enhancer Binding Protein Beta | Protein Coding | 42 | GC20P050190 | 3.374365 | https://www.genecards.org/cgi-bin/carddisp.pl?gene=CEBPB |
| GPX3 | Glutathione Peroxidase 3 | Protein Coding | 41 | GC05P150997 | 3.369219 | https://www.genecards.org/cgi-bin/carddisp.pl?gene=GPX3 |
| CYP1A1 | Cytochrome P450 Family 1 Subfamily A Member 1 | Protein Coding | 46 | GC15M074719 | 3.366565 | https://www.genecards.org/cgi-bin/carddisp.pl?gene=CYP1A1 |
| FPR2 | Formyl Peptide Receptor 2 | Protein Coding | 44 | GC19P051752 | 3.365879 | https://www.genecards.org/cgi-bin/carddisp.pl?gene=FPR2 |
| TAC3 | Tachykinin Precursor 3 | Protein Coding | 43 | GC12M057009 | 3.365708 | https://www.genecards.org/cgi-bin/carddisp.pl?gene=TAC3 |
| PLCL2 | Phospholipase C Like 2 | Protein Coding | 37 | GC03P017121 | 3.365654 | https://www.genecards.org/cgi-bin/carddisp.pl?gene=PLCL2 |
| A2M | Alpha-2-Macroglobulin | Protein Coding | 43 | GC12M009067 | 3.359872 | https://www.genecards.org/cgi- |

| | | | | | | |
|---|---|---|---|---|---|---|
| | | | | | | bin/carddisp.pl?gene=A2M |
| VTN | Vitronectin | Protein Coding | 42 | GC17M035135 | 3.35919 | https://www.genecards.org/cgi-bin/carddisp.pl?gene=VTN |
| SLAMF6 | SLAM Family Member 6 | Protein Coding | 39 | GC01M160454 | 3.356922 | https://www.genecards.org/cgi-bin/carddisp.pl?gene=SLAMF6 |
| MIR16-1 | MicroRNA 16-1 | RNA Gene | 21 | GC13M050048 | 3.355897 | https://www.genecards.org/cgi-bin/carddisp.pl?gene=MIR16-1 |
| IRAK3 | Interleukin 1 Receptor Associated Kinase 3 | Protein Coding | 45 | GC12P066278 | 3.355262 | https://www.genecards.org/cgi-bin/carddisp.pl?gene=IRAK3 |
| IKZF3 | IKAROS Family Zinc Finger 3 | Protein Coding | 41 | GC17M042354 | 3.352949 | https://www.genecards.org/cgi-bin/carddisp.pl?gene=IKZF3 |
| RNASE3 | Ribonuclease A Family Member 3 | Protein Coding | 40 | GC14P020891 | 3.351332 | https://www.genecards.org/cgi-bin/carddisp.pl?gene=RNASE3 |
| MTNR1B | Melatonin Receptor 1B | Protein Coding | 44 | GC11P092969 | 3.349228 | https://www.genecards.org/cgi-bin/carddisp.pl?gene=MTNR1B |
| CYP17A1 | Cytochrome P450 Family 17 Subfamily A Member 1 | Protein Coding | 47 | GC10M102830 | 3.347181 | https://www.genecards.org/cgi-bin/carddisp.pl?gene=CYP17A1 |
| ANXA3 | Annexin A3 | Protein Coding | 41 | GC04P078551 | 3.345737 | https://www.genecards.org/cgi-bin/carddisp.pl?gene=ANXA3 |
| FAM107A | Family With Sequence Similarity 107 Member A | Protein Coding | 36 | GC03M058741 | 3.345737 | https://www.genecards.org/cgi-bin/carddisp.pl?gene=FAM107A |
| CYP2D6 | Cytochrome P450 Family 2 Subfamily D Member 6 | Protein Coding | 46 | GC22M042126 | 3.341916 | https://www.genecards.org/cgi-bin/carddisp.pl?gene=CYP2D6 |
| CCL8 | C-C Motif Chemokine Ligand 8 | Protein Coding | 37 | GC17P034319 | 3.340772 | https://www.genecards.org/cgi-bin/carddisp.pl?gene=CCL8 |
| ITGA5 | Integrin Subunit Alpha 5 | Protein Coding | 47 | GC12M054899 | 3.33706 | https://www.genecards.org/cgi-bin/carddisp.pl?gene=ITGA5 |

| | | | | | | |
|---|---|---|---|---|---|---|
| RPL7 | Ribosomal Protein L7 | Protein Coding | 41 | GC08M 073290 | 3.335 141 | https://www.genecards.org/cgi-bin/carddisp.pl?gene=RPL7 |
| SUOX | Sulfite Oxidase | Protein Coding | 44 | GC12P 055997 | 3.330 769 | https://www.genecards.org/cgi-bin/carddisp.pl?gene=SUOX |
| TXNDC5 | Thioredoxin Domain Containing 5 | Protein Coding | 37 | GC06M 007893 | 3.329 13 | https://www.genecards.org/cgi-bin/carddisp.pl?gene=TXNDC5 |
| CYP3A4 | Cytochrome P450 Family 3 Subfamily A Member 4 | Protein Coding | 49 | GC07M 099759 | 3.326 074 | https://www.genecards.org/cgi-bin/carddisp.pl?gene=CYP3A4 |
| MB | Myoglobin | Protein Coding | 42 | GC22M 035606 | 3.325 997 | https://www.genecards.org/cgi-bin/carddisp.pl?gene=MB |
| PROCR | Protein C Receptor | Protein Coding | 41 | GC20P 035171 | 3.325 412 | https://www.genecards.org/cgi-bin/carddisp.pl?gene=PROCR |
| TSPO | Translocator Protein | Protein Coding | 43 | GC22P 043151 | 3.324 191 | https://www.genecards.org/cgi-bin/carddisp.pl?gene=TSPO |
| MIR145 | MicroRNA 145 | RNA Gene | 22 | GC05P 149430 | 3.322 383 | https://www.genecards.org/cgi-bin/carddisp.pl?gene=MIR145 |
| COL5A1 | Collagen Type V Alpha 1 Chain | Protein Coding | 45 | GC09P 134641 | 3.315 518 | https://www.genecards.org/cgi-bin/carddisp.pl?gene=COL5A1 |
| LCN2 | Lipocalin 2 | Protein Coding | 43 | GC09P 128149 | 3.315 227 | https://www.genecards.org/cgi-bin/carddisp.pl?gene=LCN2 |
| GLA | Galactosidase Alpha | Protein Coding | 47 | GC0X M1013 93 | 3.312 398 | https://www.genecards.org/cgi-bin/carddisp.pl?gene=GLA |
| TNFRSF1 8 | TNF Receptor Superfamily Member 18 | Protein Coding | 40 | GC01M 001203 | 3.311 267 | https://www.genecards.org/cgi-bin/carddisp.pl?gene=TNFRSF18 |
| WNT1 | Wnt Family Member 1 | Protein Coding | 44 | GC12P 049348 | 3.305 258 | https://www.genecards.org/cgi-bin/carddisp.pl?gene=WNT1 |
| PADI6 | Peptidyl Arginine Deiminase 6 | Protein Coding | 37 | GC01P 017851 | 3.304 638 | https://www.genecards.org/cgi- |

| | | | | | | |
|---|---|---|---|---|---|---|
| | | | | | | bin/carddisp.pl?gene=PADI6 |
| SULT1A3 | Sulfotransferase Family 1A Member 3 | Protein Coding | 37 | GC16P030199 | 3.301711 | https://www.genecards.org/cgi-bin/carddisp.pl?gene=SULT1A3 |
| PDE2A | Phosphodiesterase 2A | Protein Coding | 46 | GC11M072576 | 3.296451 | https://www.genecards.org/cgi-bin/carddisp.pl?gene=PDE2A |
| PRKCB | Protein Kinase C Beta | Protein Coding | 45 | GC16P024288 | 3.296451 | https://www.genecards.org/cgi-bin/carddisp.pl?gene=PRKCB |
| ZNF774 | Zinc Finger Protein 774 | Protein Coding | 28 | GC15P090352 | 3.296451 | https://www.genecards.org/cgi-bin/carddisp.pl?gene=ZNF774 |
| MPIG6B | Megakaryocyte And Platelet Inhibitory Receptor G6b | Protein Coding | 30 | GC06P085586 | 3.293161 | https://www.genecards.org/cgi-bin/carddisp.pl?gene=MPIG6B |
| ELAVL1 | ELAV Like RNA Binding Protein 1 | Protein Coding | 40 | GC19M007958 | 3.292305 | https://www.genecards.org/cgi-bin/carddisp.pl?gene=ELAVL1 |
| FADS1 | Fatty Acid Desaturase 1 | Protein Coding | 43 | GC11M061799 | 3.286213 | https://www.genecards.org/cgi-bin/carddisp.pl?gene=FADS1 |
| LEPR | Leptin Receptor | Protein Coding | 48 | GC01P065421 | 3.283623 | https://www.genecards.org/cgi-bin/carddisp.pl?gene=LEPR |
| CALCRL | Calcitonin Receptor Like Receptor | Protein Coding | 44 | GC02M187341 | 3.278847 | https://www.genecards.org/cgi-bin/carddisp.pl?gene=CALCRL |
| TXN | Thioredoxin | Protein Coding | 44 | GC09M110243 | 3.277952 | https://www.genecards.org/cgi-bin/carddisp.pl?gene=TXN |
| CD59 | CD59 Molecule (CD59 Blood Group) | Protein Coding | 44 | GC11M033704 | 3.277409 | https://www.genecards.org/cgi-bin/carddisp.pl?gene=CD59 |
| HAS2 | Hyaluronan Synthase 2 | Protein Coding | 40 | GC08M121594 | 3.272159 | https://www.genecards.org/cgi-bin/carddisp.pl?gene=HAS2 |
| ADAMTS7 | ADAM Metallopeptidase With Thrombospondin Type 1 Motif 7 | Protein Coding | 40 | GC15M078759 | 3.27092 | https://www.genecards.org/cgi-bin/carddisp.pl?gene=ADAMTS7 |

| | | | | | | |
|---|---|---|---|---|---|---|
| FCN1 | Ficolin 1 | Protein Coding | 40 | GC09M 135133 | 3.267 613 | https://www.genecards .org/cgi-bin/carddisp.pl?gene= FCN1 |
| SHBG | Sex Hormone Binding Globulin | Protein Coding | 40 | GC17P 007613 | 3.265 517 | https://www.genecards .org/cgi-bin/carddisp.pl?gene= SHBG |
| IL1F10 | Interleukin 1 Family Member 10 | Protein Coding | 37 | GC02P 113067 | 3.264 74 | https://www.genecards .org/cgi-bin/carddisp.pl?gene=I L1F10 |
| TGFA | Transforming Growth Factor Alpha | Protein Coding | 44 | GC02M 070447 | 3.262 326 | https://www.genecards .org/cgi-bin/carddisp.pl?gene= TGFA |
| TNFRSF1 0B | TNF Receptor Superfamily Member 10b | Protein Coding | 48 | GC08M 023020 | 3.261 706 | https://www.genecards .org/cgi-bin/carddisp.pl?gene= TNFRSF10B |
| VTCN1 | V-Set Domain Containing T Cell Activation Inhibitor 1 | Protein Coding | 39 | GC01M 117143 | 3.256 84 | https://www.genecards .org/cgi-bin/carddisp.pl?gene= VTCN1 |
| IAPP | Islet Amyloid Polypeptide | Protein Coding | 41 | GC12P 021354 | 3.253 519 | https://www.genecards .org/cgi-bin/carddisp.pl?gene=I APP |
| GPSM3 | G Protein Signaling Modulator 3 | Protein Coding | 33 | GC06M 065987 | 3.252 458 | https://www.genecards .org/cgi-bin/carddisp.pl?gene= GPSM3 |
| GRN | Granulin Precursor | Protein Coding | 46 | GC17P 044345 | 3.248 304 | https://www.genecards .org/cgi-bin/carddisp.pl?gene= GRN |
| CASP7 | Caspase 7 | Protein Coding | 47 | GC10P 113679 | 3.247 452 | https://www.genecards .org/cgi-bin/carddisp.pl?gene= CASP7 |
| MIR483 | MicroRNA 483 | RNA Gene | 20 | GC11M 003297 | 3.240 749 | https://www.genecards .org/cgi-bin/carddisp.pl?gene= MIR483 |
| CYP21A2 | Cytochrome P450 Family 21 Subfamily A Member 2 | Protein Coding | 44 | GC06P 083719 | 3.234 226 | https://www.genecards .org/cgi-bin/carddisp.pl?gene= CYP21A2 |
| CYP21A1 P | Cytochrome P450 Family 21 Subfamily A Member 1, Pseudogene | Pseudo gene | 16 | GC06P 032005 | 3.233 522 | https://www.genecards .org/cgi-bin/carddisp.pl?gene= CYP21A1P |
| KLRC1 | Killer Cell Lectin Like Receptor C1 | Protein Coding | 41 | GC12M 021112 | 3.232 145 | https://www.genecards .org/cgi- |

| | | | | | | |
|---|---|---|---|---|---|---|
| | | | | | | bin/carddisp.pl?gene=KLRC1 |
| NCF2 | Neutrophil Cytosolic Factor 2 | Protein Coding | 45 | GC01M183555 | 3.229803 | https://www.genecards.org/cgi-bin/carddisp.pl?gene=NCF2 |
| ARHGEF3 | Rho Guanine Nucleotide Exchange Factor 3 | Protein Coding | 40 | GC03M056736 | 3.229774 | https://www.genecards.org/cgi-bin/carddisp.pl?gene=ARHGEF3 |
| RFC1 | Replication Factor C Subunit 1 | Protein Coding | 46 | GC04M039291 | 3.227977 | https://www.genecards.org/cgi-bin/carddisp.pl?gene=RFC1 |
| LPL | Lipoprotein Lipase | Protein Coding | 48 | GC08P019901 | 3.224157 | https://www.genecards.org/cgi-bin/carddisp.pl?gene=LPL |
| MIR27A | MicroRNA 27a | RNA Gene | 23 | GC19M014484 | 3.223328 | https://www.genecards.org/cgi-bin/carddisp.pl?gene=MIR27A |
| IL20RB | Interleukin 20 Receptor Subunit Beta | Protein Coding | 36 | GC03P136946 | 3.222666 | https://www.genecards.org/cgi-bin/carddisp.pl?gene=IL20RB |
| FOXO1 | Forkhead Box O1 | Protein Coding | 47 | GC13M040555 | 3.217533 | https://www.genecards.org/cgi-bin/carddisp.pl?gene=FOXO1 |
| CD22 | CD22 Molecule | Protein Coding | 44 | GC19P035319 | 3.214668 | https://www.genecards.org/cgi-bin/carddisp.pl?gene=CD22 |
| PF4 | Platelet Factor 4 | Protein Coding | 39 | GC04M073980 | 3.210268 | https://www.genecards.org/cgi-bin/carddisp.pl?gene=PF4 |
| AXL | AXL Receptor Tyrosine Kinase | Protein Coding | 50 | GC19P041219 | 3.205276 | https://www.genecards.org/cgi-bin/carddisp.pl?gene=AXL |
| FOXO3 | Forkhead Box O3 | Protein Coding | 44 | GC06P108559 | 3.204709 | https://www.genecards.org/cgi-bin/carddisp.pl?gene=FOXO3 |
| DNMT3A | DNA Methyltransferase 3 Alpha | Protein Coding | 49 | GC02M025228 | 3.195729 | https://www.genecards.org/cgi-bin/carddisp.pl?gene=DNMT3A |
| DUSP22 | Dual Specificity Phosphatase 22 | Protein Coding | 38 | GC06P000302 | 3.19063 | https://www.genecards.org/cgi-bin/carddisp.pl?gene=DUSP22 |

| | | | | | | |
|---|---|---|---|---|---|---|
| ALOX5AP | Arachidonate 5-Lipoxygenase Activating Protein | Protein Coding | 42 | GC13P030713 | 3.185986 | https://www.genecards.org/cgi-bin/carddisp.pl?gene=ALOX5AP |
| CYCS | Cytochrome C, Somatic | Protein Coding | 46 | GC07M025118 | 3.182876 | https://www.genecards.org/cgi-bin/carddisp.pl?gene=CYCS |
| FLNB | Filamin B | Protein Coding | 46 | GC03P058008 | 3.182026 | https://www.genecards.org/cgi-bin/carddisp.pl?gene=FLNB |
| C1QA | Complement C1q A Chain | Protein Coding | 45 | GC01P022636 | 3.180901 | https://www.genecards.org/cgi-bin/carddisp.pl?gene=C1QA |
| IKBKG | Inhibitor Of Nuclear Factor Kappa B Kinase Regulatory Subunit Gamma | Protein Coding | 46 | GC0XP154541 | 3.180798 | https://www.genecards.org/cgi-bin/carddisp.pl?gene=IKBKG |
| HLA-E | Major Histocompatibility Complex, Class I, E | Protein Coding | 41 | GC06P083670 | 3.179679 | https://www.genecards.org/cgi-bin/carddisp.pl?gene=HLA-E |
| HTR3A | 5-Hydroxytryptamine Receptor 3A | Protein Coding | 45 | GC11P113974 | 3.176747 | https://www.genecards.org/cgi-bin/carddisp.pl?gene=HTR3A |
| MIR124-1 | MicroRNA 124-1 | RNA Gene | 21 | GC08M009903 | 3.164538 | https://www.genecards.org/cgi-bin/carddisp.pl?gene=MIR124-1 |
| TAB2 | TGF-Beta Activated Kinase 1 (MAP3K7) Binding Protein 2 | Protein Coding | 46 | GC06P149218 | 3.163508 | https://www.genecards.org/cgi-bin/carddisp.pl?gene=TAB2 |
| WNT5A | Wnt Family Member 5A | Protein Coding | 48 | GC03M055465 | 3.161349 | https://www.genecards.org/cgi-bin/carddisp.pl?gene=WNT5A |
| FAP | Fibroblast Activation Protein Alpha | Protein Coding | 44 | GC02M162170 | 3.161313 | https://www.genecards.org/cgi-bin/carddisp.pl?gene=FAP |
| CDKN3 | Cyclin Dependent Kinase Inhibitor 3 | Protein Coding | 42 | GC14P054398 | 3.154123 | https://www.genecards.org/cgi-bin/carddisp.pl?gene=CDKN3 |
| BRAF | B-Raf Proto-Oncogene, Serine/Threonine Kinase | Protein Coding | 50 | GC07M140737 | 3.152922 | https://www.genecards.org/cgi-bin/carddisp.pl?gene=BRAF |
| MC4R | Melanocortin 4 Receptor | Protein Coding | 44 | GC18M060371 | 3.149513 | https://www.genecards.org/cgi- |

| | | | | | | |
|---|---|---|---|---|---|---|
| | | | | | | bin/carddisp.pl?gene=MC4R |
| VIPR1 | Vasoactive Intestinal Peptide Receptor 1 | Protein Coding | 44 | GC03P042490 | 3.148824 | https://www.genecards.org/cgi-bin/carddisp.pl?gene=VIPR1 |
| PHRF1 | PHD And Ring Finger Domains 1 | Protein Coding | 34 | GC11P001742 | 3.148697 | https://www.genecards.org/cgi-bin/carddisp.pl?gene=PHRF1 |
| PDE4A | Phosphodiesterase 4A | Protein Coding | 44 | GC19P010416 | 3.147764 | https://www.genecards.org/cgi-bin/carddisp.pl?gene=PDE4A |
| CFTR | CF Transmembrane Conductance Regulator | Protein Coding | 49 | GC07P117287 | 3.143525 | https://www.genecards.org/cgi-bin/carddisp.pl?gene=CFTR |
| GAS6 | Growth Arrest Specific 6 | Protein Coding | 44 | GC13M113820 | 3.141688 | https://www.genecards.org/cgi-bin/carddisp.pl?gene=GAS6 |
| ENPP2 | Ectonucleotide Pyrophosphatase/Phosphodiesterase 2 | Protein Coding | 43 | GC08M119556 | 3.138681 | https://www.genecards.org/cgi-bin/carddisp.pl?gene=ENPP2 |
| SPHK1 | Sphingosine Kinase 1 | Protein Coding | 45 | GC17P076376 | 3.133767 | https://www.genecards.org/cgi-bin/carddisp.pl?gene=SPHK1 |
| PLG | Plasminogen | Protein Coding | 48 | GC06P160702 | 3.132704 | https://www.genecards.org/cgi-bin/carddisp.pl?gene=PLG |
| RPS12P4 | Ribosomal Protein S12 Pseudogene 4 | Pseudogene | 9 | GC02M041850 | 3.132163 | https://www.genecards.org/cgi-bin/carddisp.pl?gene=RPS12P4 |
| RNASEH2A | Ribonuclease H2 Subunit A | Protein Coding | 42 | GC19P014276 | 3.127436 | https://www.genecards.org/cgi-bin/carddisp.pl?gene=RNASEH2A |
| ABCC2 | ATP Binding Cassette Subfamily C Member 2 | Protein Coding | 46 | GC10P099782 | 3.125788 | https://www.genecards.org/cgi-bin/carddisp.pl?gene=ABCC2 |
| CCL3L1 | C-C Motif Chemokine Ligand 3 Like 1 | Protein Coding | 28 | GC17Mj00250 | 3.120484 | https://www.genecards.org/cgi-bin/carddisp.pl?gene=CCL3L1 |
| SEMA3C | Semaphorin 3C | Protein Coding | 43 | GC07M080742 | 3.116683 | https://www.genecards.org/cgi-bin/carddisp.pl?gene=SEMA3C |

| | | | | | | |
|---|---|---|---|---|---|---|
| SOD3 | Superoxide Dismutase 3 | Protein Coding | 41 | GC04P 024798 | 3.111 549 | https://www.genecards .org/cgi-bin/carddisp.pl?gene= SOD3 |
| TCN1 | Transcobalamin 1 | Protein Coding | 41 | GC11M 089510 | 3.111 306 | https://www.genecards .org/cgi-bin/carddisp.pl?gene= TCN1 |
| EZH2 | Enhancer Of Zeste 2 Polycomb Repressive Complex 2 Subunit | Protein Coding | 52 | GC07M 148807 | 3.109 519 | https://www.genecards .org/cgi-bin/carddisp.pl?gene= EZH2 |
| CCL15 | C-C Motif Chemokine Ligand 15 | Protein Coding | 34 | GC17M 035996 | 3.106 022 | https://www.genecards .org/cgi-bin/carddisp.pl?gene= CCL15 |
| OSCAR | Osteoclast Associated Ig-Like Receptor | Protein Coding | 37 | GC19M 054094 | 3.105 77 | https://www.genecards .org/cgi-bin/carddisp.pl?gene= OSCAR |
| ATG16L1 | Autophagy Related 16 Like 1 | Protein Coding | 41 | GC02P 233253 | 3.103 968 | https://www.genecards .org/cgi-bin/carddisp.pl?gene= ATG16L1 |
| HLA-DRB9 | Major Histocompatibility Complex, Class II, DR Beta 9 (Pseudogene) | Pseudo gene | 13 | GC06M 066001 | 3.103 507 | https://www.genecards .org/cgi-bin/carddisp.pl?gene= HLA-DRB9 |
| RNASEH 2C | Ribonuclease H2 Subunit C | Protein Coding | 38 | GC11M 065714 | 3.099 708 | https://www.genecards .org/cgi-bin/carddisp.pl?gene= RNASEH2C |
| CNR1 | Cannabinoid Receptor 1 | Protein Coding | 45 | GC06M 088139 | 3.099 246 | https://www.genecards .org/cgi-bin/carddisp.pl?gene= CNR1 |
| CHRNA7 | Cholinergic Receptor Nicotinic Alpha 7 Subunit | Protein Coding | 44 | GC15P 031923 | 3.095 434 | https://www.genecards .org/cgi-bin/carddisp.pl?gene= CHRNA7 |
| MMP16 | Matrix Metallopeptidase 16 | Protein Coding | 43 | GC08M 088032 | 3.094 251 | https://www.genecards .org/cgi-bin/carddisp.pl?gene= MMP16 |
| APLN | Apelin | Protein Coding | 36 | GC0X M1296 45 | 3.088 194 | https://www.genecards .org/cgi-bin/carddisp.pl?gene= APLN |
| TIRAP | TIR Domain Containing Adaptor Protein | Protein Coding | 41 | GC11P 126284 | 3.084 987 | https://www.genecards .org/cgi-bin/carddisp.pl?gene= TIRAP |
| NRP1 | Neuropilin 1 | Protein Coding | 45 | GC10M 033177 | 3.081 483 | https://www.genecards .org/cgi- |

| | | | | | | |
|---|---|---|---|---|---|---|
| | | | | | | bin/carddisp.pl?gene=NRP1 |
| EFEMP1 | EGF Containing Fibulin Extracellular Matrix Protein 1 | Protein Coding | 44 | GC02M055865 | 3.081483 | https://www.genecards.org/cgi-bin/carddisp.pl?gene=EFEMP1 |
| QPCT | Glutaminyl-Peptide Cyclotransferase | Protein Coding | 43 | GC02P037344 | 3.078385 | https://www.genecards.org/cgi-bin/carddisp.pl?gene=QPCT |
| CD200R1 | CD200 Receptor 1 | Protein Coding | 38 | GC03M112921 | 3.073316 | https://www.genecards.org/cgi-bin/carddisp.pl?gene=CD200R1 |
| MAP2K3 | Mitogen-Activated Protein Kinase Kinase 3 | Protein Coding | 46 | GC17P054789 | 3.073071 | https://www.genecards.org/cgi-bin/carddisp.pl?gene=MAP2K3 |
| SLC17A1 | Solute Carrier Family 17 Member 1 | Protein Coding | 39 | GC06M025723 | 3.072752 | https://www.genecards.org/cgi-bin/carddisp.pl?gene=SLC17A1 |
| SERPINF1 | Serpin Family F Member 1 | Protein Coding | 44 | GC17P001761 | 3.071793 | https://www.genecards.org/cgi-bin/carddisp.pl?gene=SERPINF1 |
| PFKFB3 | 6-Phosphofructo-2-Kinase/Fructose-2,6-Biphosphatase 3 | Protein Coding | 43 | GC10P006144 | 3.069526 | https://www.genecards.org/cgi-bin/carddisp.pl?gene=PFKFB3 |
| BMS1P20 | BMS1 Pseudogene 20 | Pseudo gene | 16 | GC22P022298 | 3.069526 | https://www.genecards.org/cgi-bin/carddisp.pl?gene=BMS1P20 |
| YY1 | YY1 Transcription Factor | Protein Coding | 46 | GC14P100238 | 3.066354 | https://www.genecards.org/cgi-bin/carddisp.pl?gene=YY1 |
| KLRB1 | Killer Cell Lectin Like Receptor B1 | Protein Coding | 39 | GC12M021098 | 3.063033 | https://www.genecards.org/cgi-bin/carddisp.pl?gene=KLRB1 |
| EBI3 | Epstein-Barr Virus Induced 3 | Protein Coding | 37 | GC19P004233 | 3.062869 | https://www.genecards.org/cgi-bin/carddisp.pl?gene=EBI3 |
| TIA1 | TIA1 Cytotoxic Granule Associated RNA Binding Protein | Protein Coding | 42 | GC02M070209 | 3.062519 | https://www.genecards.org/cgi-bin/carddisp.pl?gene=TIA1 |
| HDAC9 | Histone Deacetylase 9 | Protein Coding | 46 | GC07P018086 | 3.061987 | https://www.genecards.org/cgi-bin/carddisp.pl?gene=HDAC9 |

| | | | | | | |
|---|---|---|---|---|---|---|
| IL17RC | Interleukin 17 Receptor C | Protein Coding | 38 | GC03P 009917 | 3.061 243 | https://www.genecards .org/cgi-bin/carddisp.pl?gene=I L17RC |
| BAG6 | BAG Cochaperone 6 | Protein Coding | 36 | GC06M 031639 | 3.060 708 | https://www.genecards .org/cgi-bin/carddisp.pl?gene= BAG6 |
| STAT5A | Signal Transducer And Activator Of Transcription 5A | Protein Coding | 45 | GC17P 042287 | 3.055 726 | https://www.genecards .org/cgi-bin/carddisp.pl?gene= STAT5A |
| HSP90A B1 | Heat Shock Protein 90 Alpha Family Class B Member 1 | Protein Coding | 46 | GC06P 044246 | 3.052 162 | https://www.genecards .org/cgi-bin/carddisp.pl?gene= HSP90AB1 |
| DNASE2 | Deoxyribonuclease 2, Lysosomal | Protein Coding | 39 | GC19M 012875 | 3.051 737 | https://www.genecards .org/cgi-bin/carddisp.pl?gene= DNASE2 |
| IL36A | Interleukin 36 Alpha | Protein Coding | 36 | GC02P 113005 | 3.050 442 | https://www.genecards .org/cgi-bin/carddisp.pl?gene=I L36A |
| TNC | Tenascin C | Protein Coding | 47 | GC09M 115019 | 3.050 395 | https://www.genecards .org/cgi-bin/carddisp.pl?gene= TNC |
| TNFSF18 | TNF Superfamily Member 18 | Protein Coding | 37 | GC01M 173009 | 3.046 634 | https://www.genecards .org/cgi-bin/carddisp.pl?gene= TNFSF18 |
| MAP2K7 | Mitogen-Activated Protein Kinase Kinase 7 | Protein Coding | 44 | GC19P 007903 | 3.045 817 | https://www.genecards .org/cgi-bin/carddisp.pl?gene= MAP2K7 |
| PYCARD | PYD And CARD Domain Containing | Protein Coding | 40 | GC16M 031201 | 3.041 759 | https://www.genecards .org/cgi-bin/carddisp.pl?gene= PYCARD |
| HOXD13 | Homeobox D13 | Protein Coding | 41 | GC02P 176092 | 3.039 764 | https://www.genecards .org/cgi-bin/carddisp.pl?gene= HOXD13 |
| MIR222 | MicroRNA 222 | RNA Gene | 21 | GC0X M0457 47 | 3.037 139 | https://www.genecards .org/cgi-bin/carddisp.pl?gene= MIR222 |
| RTKN2 | Rhotekin 2 | Protein Coding | 35 | GC10M 062183 | 3.036 466 | https://www.genecards .org/cgi-bin/carddisp.pl?gene= RTKN2 |
| MAPK3 | Mitogen-Activated Protein Kinase 3 | Protein Coding | 46 | GC16M 037120 | 3.034 573 | https://www.genecards .org/cgi- |

| | | | | | | |
|---|---|---|---|---|---|---|
| | | | | | | bin/carddisp.pl?gene=MAPK3 |
| SLAMF1 | Signaling Lymphocytic Activation Molecule Family Member 1 | Protein Coding | 40 | GC01M160608 | 3.02949 | https://www.genecards.org/cgi-bin/carddisp.pl?gene=SLAMF1 |
| ADAMTSL1 | ADAMTS Like 1 | Protein Coding | 41 | GC09P017906 | 3.028688 | https://www.genecards.org/cgi-bin/carddisp.pl?gene=ADAMTSL1 |
| USH2A | Usherin | Protein Coding | 38 | GC01M215622 | 3.026695 | https://www.genecards.org/cgi-bin/carddisp.pl?gene=USH2A |
| ADSL | Adenylosuccinate Lyase | Protein Coding | 46 | GC22P040346 | 3.019983 | https://www.genecards.org/cgi-bin/carddisp.pl?gene=ADSL |
| PSENEN | Presenilin Enhancer, Gamma-Secretase Subunit | Protein Coding | 42 | GC19P066549 | 3.014358 | https://www.genecards.org/cgi-bin/carddisp.pl?gene=PSENEN |
| SCGB1A1 | Secretoglobin Family 1A Member 1 | Protein Coding | 39 | GC11P062405 | 3.013911 | https://www.genecards.org/cgi-bin/carddisp.pl?gene=SCGB1A1 |
| FCGR2C | Fc Gamma Receptor IIc (Gene/Pseudogene) | Protein Coding | 35 | GC01P161718 | 3.01309 | https://www.genecards.org/cgi-bin/carddisp.pl?gene=FCGR2C |
| BAD | BCL2 Associated Agonist Of Cell Death | Protein Coding | 43 | GC11M089708 | 3.011511 | https://www.genecards.org/cgi-bin/carddisp.pl?gene=BAD |
| SYNGR1 | Synaptogyrin 1 | Protein Coding | 40 | GC22P039350 | 3.009294 | https://www.genecards.org/cgi-bin/carddisp.pl?gene=SYNGR1 |
| ALDH2 | Aldehyde Dehydrogenase 2 Family Member | Protein Coding | 49 | GC12P111766 | 3.007002 | https://www.genecards.org/cgi-bin/carddisp.pl?gene=ALDH2 |
| SLPI | Secretory Leukocyte Peptidase Inhibitor | Protein Coding | 39 | GC20M045252 | 3.006694 | https://www.genecards.org/cgi-bin/carddisp.pl?gene=SLPI |
| IL31 | Interleukin 31 | Protein Coding | 34 | GC12M122295 | 3.000791 | https://www.genecards.org/cgi-bin/carddisp.pl?gene=IL31 |
| RBP4 | Retinol Binding Protein 4 | Protein Coding | 44 | GC10M093591 | 2.99993 | https://www.genecards.org/cgi-bin/carddisp.pl?gene=RBP4 |

| | | | | | | |
|---|---|---|---|---|---|---|
| ICA1 | Islet Cell Autoantigen 1 | Protein Coding | 40 | GC07M 008206 | 2.993 47 | https://www.genecards .org/cgi-bin/carddisp.pl?gene=I CA1 |
| PLOD2 | Procollagen-Lysine,2-Oxoglutarate 5-Dioxygenase 2 | Protein Coding | 44 | GC03M 146069 | 2.993 037 | https://www.genecards .org/cgi-bin/carddisp.pl?gene= PLOD2 |
| SEMA4D | Semaphorin 4D | Protein Coding | 45 | GC09M 089360 | 2.992 128 | https://www.genecards .org/cgi-bin/carddisp.pl?gene= SEMA4D |
| APP | Amyloid Beta Precursor Protein | Protein Coding | 49 | GC21M 025880 | 2.991 846 | https://www.genecards .org/cgi-bin/carddisp.pl?gene= APP |
| CCL1 | C-C Motif Chemokine Ligand 1 | Protein Coding | 36 | GC17M 035584 | 2.991 845 | https://www.genecards .org/cgi-bin/carddisp.pl?gene= CCL1 |
| FNDC1 | Fibronectin Type III Domain Containing 1 | Protein Coding | 34 | GC06P 160538 | 2.985 362 | https://www.genecards .org/cgi-bin/carddisp.pl?gene= FNDC1 |
| MIR99B | MicroRNA 99b | RNA Gene | 20 | GC19P 051692 | 2.980 7 | https://www.genecards .org/cgi-bin/carddisp.pl?gene= MIR99B |
| BCL2L11 | BCL2 Like 11 | Protein Coding | 42 | GC02P 111119 | 2.978 083 | https://www.genecards .org/cgi-bin/carddisp.pl?gene= BCL2L11 |
| BST2 | Bone Marrow Stromal Cell Antigen 2 | Protein Coding | 38 | GC19M 017403 | 2.977 444 | https://www.genecards .org/cgi-bin/carddisp.pl?gene= BST2 |
| IL12RB2 | Interleukin 12 Receptor Subunit Beta 2 | Protein Coding | 42 | GC01P 067307 | 2.975 707 | https://www.genecards .org/cgi-bin/carddisp.pl?gene=I L12RB2 |
| ATF2 | Activating Transcription Factor 2 | Protein Coding | 44 | GC02M 175072 | 2.975 466 | https://www.genecards .org/cgi-bin/carddisp.pl?gene= ATF2 |
| NCF4 | Neutrophil Cytosolic Factor 4 | Protein Coding | 46 | GC22P 036860 | 2.973 231 | https://www.genecards .org/cgi-bin/carddisp.pl?gene= NCF4 |
| CISH | Cytokine Inducible SH2 Containing Protein | Protein Coding | 44 | GC03M 051268 | 2.971 012 | https://www.genecards .org/cgi-bin/carddisp.pl?gene= CISH |
| FOXJ1 | Forkhead Box J1 | Protein Coding | 37 | GC17M 076136 | 2.969 518 | https://www.genecards .org/cgi- |

| | | | | | | |
|---|---|---|---|---|---|---|
| | | | | | | bin/carddisp.pl?gene=FOXJ1 |
| MAPK9 | Mitogen-Activated Protein Kinase 9 | Protein Coding | 46 | GC05M180254 | 2.965781 | https://www.genecards.org/cgi-bin/carddisp.pl?gene=MAPK9 |
| MMP20 | Matrix Metallopeptidase 20 | Protein Coding | 44 | GC11M102576 | 2.964606 | https://www.genecards.org/cgi-bin/carddisp.pl?gene=MMP20 |
| PERP | P53 Apoptosis Effector Related To PMP22 | Protein Coding | 40 | GC06M138088 | 2.95774 | https://www.genecards.org/cgi-bin/carddisp.pl?gene=PERP |
| DDX58 | DExD/H-Box Helicase 58 | Protein Coding | 45 | GC09M032455 | 2.956203 | https://www.genecards.org/cgi-bin/carddisp.pl?gene=DDX58 |
| C1S | Complement C1s | Protein Coding | 47 | GC12P021098 | 2.955665 | https://www.genecards.org/cgi-bin/carddisp.pl?gene=C1S |
| SDC1 | Syndecan 1 | Protein Coding | 41 | GC02M020200 | 2.952341 | https://www.genecards.org/cgi-bin/carddisp.pl?gene=SDC1 |
| RNGTT | RNA Guanylyltransferase And 5'-Phosphatase | Protein Coding | 41 | GC06M088609 | 2.947067 | https://www.genecards.org/cgi-bin/carddisp.pl?gene=RNGTT |
| GHR | Growth Hormone Receptor | Protein Coding | 44 | GC05P042429 | 2.943426 | https://www.genecards.org/cgi-bin/carddisp.pl?gene=GHR |
| UGDH | UDP-Glucose 6-Dehydrogenase | Protein Coding | 44 | GC04M039502 | 2.939529 | https://www.genecards.org/cgi-bin/carddisp.pl?gene=UGDH |
| CYP27B1 | Cytochrome P450 Family 27 Subfamily B Member 1 | Protein Coding | 46 | GC12M057757 | 2.937761 | https://www.genecards.org/cgi-bin/carddisp.pl?gene=CYP27B1 |
| PFKL | Phosphofructokinase, Liver Type | Protein Coding | 44 | GC21P044300 | 2.932446 | https://www.genecards.org/cgi-bin/carddisp.pl?gene=PFKL |
| CD101 | CD101 Molecule | Protein Coding | 38 | GC01P117001 | 2.932446 | https://www.genecards.org/cgi-bin/carddisp.pl?gene=CD101 |
| TAX1BP1 | Tax1 Binding Protein 1 | Protein Coding | 38 | GC07P027739 | 2.932446 | https://www.genecards.org/cgi-bin/carddisp.pl?gene=TAX1BP1 |

| | | | | | | |
|---|---|---|---|---|---|---|
| FCRL4 | Fc Receptor Like 4 | Protein Coding | 37 | GC01M 157543 | 2.932 446 | https://www.genecards .org/cgi-bin/carddisp.pl?gene= FCRL4 |
| PHACTR 3 | Phosphatase And Actin Regulator 3 | Protein Coding | 36 | GC20P 059577 | 2.932 446 | https://www.genecards .org/cgi-bin/carddisp.pl?gene= PHACTR3 |
| TRHDE | Thyrotropin Releasing Hormone Degrading Enzyme | Protein Coding | 36 | GC12P 072087 | 2.932 446 | https://www.genecards .org/cgi-bin/carddisp.pl?gene= TRHDE |
| C5-OT1 | C5 3' UTR Overlapping Transcript 1 | RNA Gene | 8 | GC09M 120944 | 2.932 446 | https://www.genecards .org/cgi-bin/carddisp.pl?gene= C5-OT1 |
| ELP1 | Elongator Acetyltransferase Complex Subunit 1 | Protein Coding | 34 | GC09M 108868 | 2.930 565 | https://www.genecards .org/cgi-bin/carddisp.pl?gene= ELP1 |
| PSORS1 C3 | Psoriasis Susceptibility 1 Candidate 3 | RNA Gene | 21 | GC06M 065907 | 2.929 77 | https://www.genecards .org/cgi-bin/carddisp.pl?gene= PSORS1C3 |
| ALPK1 | Alpha Kinase 1 | Protein Coding | 40 | GC04P 112285 | 2.929 518 | https://www.genecards .org/cgi-bin/carddisp.pl?gene= ALPK1 |
| ARAP1 | ArfGAP With RhoGAP Domain, Ankyrin Repeat And PH Domain 1 | Protein Coding | 40 | GC11M 090324 | 2.922 596 | https://www.genecards .org/cgi-bin/carddisp.pl?gene= ARAP1 |
| PCNA | Proliferating Cell Nuclear Antigen | Protein Coding | 48 | GC20M 005114 | 2.920 493 | https://www.genecards .org/cgi-bin/carddisp.pl?gene= PCNA |
| KEAP1 | Kelch Like ECH Associated Protein 1 | Protein Coding | 46 | GC19M 010486 | 2.919 772 | https://www.genecards .org/cgi-bin/carddisp.pl?gene= KEAP1 |
| FLT4 | Fms Related Receptor Tyrosine Kinase 4 | Protein Coding | 49 | GC05M 180607 | 2.912 264 | https://www.genecards .org/cgi-bin/carddisp.pl?gene= FLT4 |
| TACR3 | Tachykinin Receptor 3 | Protein Coding | 48 | GC04M 103586 | 2.910 977 | https://www.genecards .org/cgi-bin/carddisp.pl?gene= TACR3 |
| BAK1 | BCL2 Antagonist/Killer 1 | Protein Coding | 42 | GC06M 033572 | 2.909 388 | https://www.genecards .org/cgi-bin/carddisp.pl?gene= BAK1 |
| CD1A | CD1a Molecule | Protein Coding | 40 | GC01P 158255 | 2.903 334 | https://www.genecards .org/cgi- |

| | | | | | | |
|---|---|---|---|---|---|---|
| | | | | | | bin/carddisp.pl?gene=CD1A |
| TGFB3 | Transforming Growth Factor Beta 3 | Protein Coding | 45 | GC14M075958 | 2.90309 | https://www.genecards.org/cgi-bin/carddisp.pl?gene=TGFB3 |
| TAB1 | TGF-Beta Activated Kinase 1 (MAP3K7) Binding Protein 1 | Protein Coding | 40 | GC22P039429 | 2.902945 | https://www.genecards.org/cgi-bin/carddisp.pl?gene=TAB1 |
| DDR1 | Discoidin Domain Receptor Tyrosine Kinase 1 | Protein Coding | 45 | GC06P083678 | 2.898831 | https://www.genecards.org/cgi-bin/carddisp.pl?gene=DDR1 |
| MAP3K2 | Mitogen-Activated Protein Kinase Kinase Kinase 2 | Protein Coding | 44 | GC02M127298 | 2.898114 | https://www.genecards.org/cgi-bin/carddisp.pl?gene=MAP3K2 |
| CASP9 | Caspase 9 | Protein Coding | 45 | GC01M015491 | 2.897199 | https://www.genecards.org/cgi-bin/carddisp.pl?gene=CASP9 |
| ZPBP2 | Zona Pellucida Binding Protein 2 | Protein Coding | 34 | GC17P055526 | 2.894372 | https://www.genecards.org/cgi-bin/carddisp.pl?gene=ZPBP2 |
| PTK2B | Protein Tyrosine Kinase 2 Beta | Protein Coding | 47 | GC08P027311 | 2.893253 | https://www.genecards.org/cgi-bin/carddisp.pl?gene=PTK2B |
| TIMELESS | Timeless Circadian Regulator | Protein Coding | 38 | GC12M056416 | 2.887687 | https://www.genecards.org/cgi-bin/carddisp.pl?gene=TIMELESS |
| BCR | BCR Activator Of RhoGEF And GTPase | Protein Coding | 50 | GC22P023179 | 2.884006 | https://www.genecards.org/cgi-bin/carddisp.pl?gene=BCR |
| MLKL | Mixed Lineage Kinase Domain Like Pseudokinase | Protein Coding | 40 | GC16M074672 | 2.880919 | https://www.genecards.org/cgi-bin/carddisp.pl?gene=MLKL |
| IRX1 | Iroquois Homeobox 1 | Protein Coding | 38 | GC05P003596 | 2.878638 | https://www.genecards.org/cgi-bin/carddisp.pl?gene=IRX1 |
| TRD | T Cell Receptor Delta Locus | Protein Coding | 13 | GC14P032626 | 2.8778 | https://www.genecards.org/cgi-bin/carddisp.pl?gene=TRD |
| NOTCH1 | Notch Receptor 1 | Protein Coding | 49 | GC09M137524 | 2.872792 | https://www.genecards.org/cgi-bin/carddisp.pl?gene=NOTCH1 |

| | | | | | | |
|---|---|---|---|---|---|---|
| GATA4 | GATA Binding Protein 4 | Protein Coding | 46 | GC08P 011676 | 2.871 077 | https://www.genecards .org/cgi-bin/carddisp.pl?gene= GATA4 |
| TXNRD1 | Thioredoxin Reductase 1 | Protein Coding | 45 | GC12P 104215 | 2.870 043 | https://www.genecards .org/cgi-bin/carddisp.pl?gene= TXNRD1 |
| SNRNP7 0 | Small Nuclear Ribonucleoprotein U1 Subunit 70 | Protein Coding | 37 | GC19P 049085 | 2.866 608 | https://www.genecards .org/cgi-bin/carddisp.pl?gene= SNRNP70 |
| SLC22A7 | Solute Carrier Family 22 Member 7 | Protein Coding | 41 | GC06P 083920 | 2.865 655 | https://www.genecards .org/cgi-bin/carddisp.pl?gene= SLC22A7 |
| CLEC7A | C-Type Lectin Domain Containing 7A | Protein Coding | 44 | GC12M 021105 | 2.862 968 | https://www.genecards .org/cgi-bin/carddisp.pl?gene= CLEC7A |
| MAPK13 | Mitogen-Activated Protein Kinase 13 | Protein Coding | 46 | GC06P 083842 | 2.860 585 | https://www.genecards .org/cgi-bin/carddisp.pl?gene= MAPK13 |
| CCL27 | C-C Motif Chemokine Ligand 27 | Protein Coding | 34 | GC09M 034662 | 2.859 911 | https://www.genecards .org/cgi-bin/carddisp.pl?gene= CCL27 |
| CASR | Calcium Sensing Receptor | Protein Coding | 49 | GC03P 122183 | 2.859 241 | https://www.genecards .org/cgi-bin/carddisp.pl?gene= CASR |
| SST | Somatostatin | Protein Coding | 40 | GC03M 187668 | 2.854 44 | https://www.genecards .org/cgi-bin/carddisp.pl?gene= SST |
| EZR | Ezrin | Protein Coding | 43 | GC06M 158765 | 2.850 79 | https://www.genecards .org/cgi-bin/carddisp.pl?gene= EZR |
| CD34 | CD34 Molecule | Protein Coding | 42 | GC01M 207880 | 2.845 316 | https://www.genecards .org/cgi-bin/carddisp.pl?gene= CD34 |
| NFKBIB | NFKB Inhibitor Beta | Protein Coding | 40 | GC19P 038899 | 2.841 966 | https://www.genecards .org/cgi-bin/carddisp.pl?gene= NFKBIB |
| KLRC2 | Killer Cell Lectin Like Receptor C2 | Protein Coding | 36 | GC12M 021111 | 2.841 174 | https://www.genecards .org/cgi-bin/carddisp.pl?gene= KLRC2 |
| TYMP | Thymidine Phosphorylase | Protein Coding | 45 | GC22M 050525 | 2.838 929 | https://www.genecards .org/cgi- |

| | | | | | | |
|---|---|---|---|---|---|---|
| | | | | | | bin/carddisp.pl?gene=TYMP |
| NGFR | Nerve Growth Factor Receptor | Protein Coding | 44 | GC17P049495 | 2.835482 | https://www.genecards.org/cgi-bin/carddisp.pl?gene=NGFR |
| LAIR1 | Leukocyte Associated Immunoglobulin Like Receptor 1 | Protein Coding | 41 | GC19M054351 | 2.834127 | https://www.genecards.org/cgi-bin/carddisp.pl?gene=LAIR1 |
| HNF4A | Hepatocyte Nuclear Factor 4 Alpha | Protein Coding | 48 | GC20P044355 | 2.831234 | https://www.genecards.org/cgi-bin/carddisp.pl?gene=HNF4A |
| SEMA3A | Semaphorin 3A | Protein Coding | 44 | GC07M083955 | 2.830889 | https://www.genecards.org/cgi-bin/carddisp.pl?gene=SEMA3A |
| CCK | Cholecystokinin | Protein Coding | 41 | GC03M042274 | 2.829787 | https://www.genecards.org/cgi-bin/carddisp.pl?gene=CCK |
| CTSC | Cathepsin C | Protein Coding | 46 | GC11M090619 | 2.82807 | https://www.genecards.org/cgi-bin/carddisp.pl?gene=CTSC |
| IL1R2 | Interleukin 1 Receptor Type 2 | Protein Coding | 44 | GC02P101991 | 2.823656 | https://www.genecards.org/cgi-bin/carddisp.pl?gene=IL1R2 |
| INPP5B | Inositol Polyphosphate-5-Phosphatase B | Protein Coding | 41 | GC01M037860 | 2.822069 | https://www.genecards.org/cgi-bin/carddisp.pl?gene=INPP5B |
| FMOD | Fibromodulin | Protein Coding | 41 | GC01M203340 | 2.820914 | https://www.genecards.org/cgi-bin/carddisp.pl?gene=FMOD |
| LPAR1 | Lysophosphatidic Acid Receptor 1 | Protein Coding | 44 | GC09M110873 | 2.819573 | https://www.genecards.org/cgi-bin/carddisp.pl?gene=LPAR1 |
| AGTR1 | Angiotensin II Receptor Type 1 | Protein Coding | 48 | GC03P148697 | 2.818228 | https://www.genecards.org/cgi-bin/carddisp.pl?gene=AGTR1 |
| CLEC12A | C-Type Lectin Domain Family 12 Member A | Protein Coding | 38 | GC12P009951 | 2.817895 | https://www.genecards.org/cgi-bin/carddisp.pl?gene=CLEC12A |
| MIR204 | MicroRNA 204 | RNA Gene | 24 | GC09M070809 | 2.816488 | https://www.genecards.org/cgi-bin/carddisp.pl?gene=MIR204 |

| | | | | | | |
|---|---|---|---|---|---|---|
| MIR146B | MicroRNA 146b | RNA Gene | 21 | GC10P 102436 | 2.813 499 | https://www.genecards .org/cgi-bin/carddisp.pl?gene= MIR146B |
| GYPC | Glycophorin C (Gerbich Blood Group) | Protein Coding | 42 | GC02P 126655 | 2.809 01 | https://www.genecards .org/cgi-bin/carddisp.pl?gene= GYPC |
| KIR3DL1 | Killer Cell Immunoglobulin Like Receptor, Three Ig Domains And Long Cytoplasmic Tail 1 | Protein Coding | 39 | GC19P 067384 | 2.806 866 | https://www.genecards .org/cgi-bin/carddisp.pl?gene= KIR3DL1 |
| TCF7L2 | Transcription Factor 7 Like 2 | Protein Coding | 45 | GC10P 112950 | 2.806 362 | https://www.genecards .org/cgi-bin/carddisp.pl?gene= TCF7L2 |
| PDGFRA | Platelet Derived Growth Factor Receptor Alpha | Protein Coding | 51 | GC04P 054229 | 2.805 772 | https://www.genecards .org/cgi-bin/carddisp.pl?gene= PDGFRA |
| AFP | Alpha Fetoprotein | Protein Coding | 45 | GC04P 073431 | 2.804 905 | https://www.genecards .org/cgi-bin/carddisp.pl?gene= AFP |
| RUNX2 | RUNX Family Transcription Factor 2 | Protein Coding | 45 | GC06P 083940 | 2.801 021 | https://www.genecards .org/cgi-bin/carddisp.pl?gene= RUNX2 |
| CEACA M8 | CEA Cell Adhesion Molecule 8 | Protein Coding | 38 | GC19M 042580 | 2.800 923 | https://www.genecards .org/cgi-bin/carddisp.pl?gene= CEACAM8 |
| AOX1 | Aldehyde Oxidase 1 | Protein Coding | 44 | GC02P 200585 | 2.792 653 | https://www.genecards .org/cgi-bin/carddisp.pl?gene= AOX1 |
| TRA | T Cell Receptor Alpha Locus | Protein Coding | 19 | GC14P 021621 | 2.791 31 | https://www.genecards .org/cgi-bin/carddisp.pl?gene= TRA |
| KL | Klotho | Protein Coding | 45 | GC13P 033016 | 2.787 392 | https://www.genecards .org/cgi-bin/carddisp.pl?gene= KL |
| C1R | Complement C1r | Protein Coding | 46 | GC12M 007750 | 2.784 245 | https://www.genecards .org/cgi-bin/carddisp.pl?gene= C1R |
| MST1 | Macrophage Stimulating 1 | Protein Coding | 45 | GC03M 049683 | 2.776 713 | https://www.genecards .org/cgi-bin/carddisp.pl?gene= MST1 |

| | | | | | | |
|---|---|---|---|---|---|---|
| OS9 | OS9 Endoplasmic Reticulum Lectin | Protein Coding | 40 | GC12P 057693 | 2.776 713 | https://www.genecards .org/cgi-bin/carddisp.pl?gene= OS9 |
| UCN | Urocortin | Protein Coding | 36 | GC02M 027308 | 2.776 207 | https://www.genecards .org/cgi-bin/carddisp.pl?gene= UCN |
| TLR10 | Toll Like Receptor 10 | Protein Coding | 38 | GC04M 038773 | 2.775 997 | https://www.genecards .org/cgi-bin/carddisp.pl?gene= TLR10 |
| MITF | Melanocyte Inducing Transcription Factor | Protein Coding | 46 | GC03P 069788 | 2.774 539 | https://www.genecards .org/cgi-bin/carddisp.pl?gene= MITF |
| PABIR2 | PABIR Family Member 2 | Protein Coding | 24 | GC0X M1348 26 | 2.774 215 | https://www.genecards .org/cgi-bin/carddisp.pl?gene= PABIR2 |
| IRF7 | Interferon Regulatory Factor 7 | Protein Coding | 46 | GC11M 000612 | 2.773 942 | https://www.genecards .org/cgi-bin/carddisp.pl?gene=I RF7 |
| LALBA | Lactalbumin Alpha | Protein Coding | 38 | GC12M 048567 | 2.772 724 | https://www.genecards .org/cgi-bin/carddisp.pl?gene= LALBA |
| CYP2E1 | Cytochrome P450 Family 2 Subfamily E Member 1 | Protein Coding | 44 | GC10P 133520 | 2.772 125 | https://www.genecards .org/cgi-bin/carddisp.pl?gene= CYP2E1 |
| LTB4R | Leukotriene B4 Receptor | Protein Coding | 43 | GC14P 024311 | 2.771 675 | https://www.genecards .org/cgi-bin/carddisp.pl?gene= LTB4R |
| CHIT1 | Chitinase 1 | Protein Coding | 43 | GC01M 203213 | 2.769 509 | https://www.genecards .org/cgi-bin/carddisp.pl?gene= CHIT1 |
| PROC | Protein C, Inactivator Of Coagulation Factors Va And VIIIa | Protein Coding | 48 | GC02P 127418 | 2.768 957 | https://www.genecards .org/cgi-bin/carddisp.pl?gene= PROC |
| PDCD5 | Programmed Cell Death 5 | Protein Coding | 38 | GC19P 032581 | 2.767 897 | https://www.genecards .org/cgi-bin/carddisp.pl?gene= PDCD5 |
| WDR26 | WD Repeat Domain 26 | Protein Coding | 40 | GC01M 224385 | 2.766 876 | https://www.genecards .org/cgi-bin/carddisp.pl?gene= WDR26 |

| | | | | | | |
|---|---|---|---|---|---|---|
| CPEB4 | Cytoplasmic Polyadenylation Element Binding Protein 4 | Protein Coding | 36 | GC05P 173888 | 2.766 876 | https://www.genecards .org/cgi-bin/carddisp.pl?gene= CPEB4 |
| MOB3B | MOB Kinase Activator 3B | Protein Coding | 34 | GC09M 028330 | 2.766 743 | https://www.genecards .org/cgi-bin/carddisp.pl?gene= MOB3B |
| GDF15 | Growth Differentiation Factor 15 | Protein Coding | 40 | GC19P 066244 | 2.766 581 | https://www.genecards .org/cgi-bin/carddisp.pl?gene= GDF15 |
| SPRY2 | Sprouty RTK Signaling Antagonist 2 | Protein Coding | 44 | GC13M 080335 | 2.758 616 | https://www.genecards .org/cgi-bin/carddisp.pl?gene= SPRY2 |
| PLA2G5 | Phospholipase A2 Group V | Protein Coding | 41 | GC01P 020028 | 2.757 529 | https://www.genecards .org/cgi-bin/carddisp.pl?gene= PLA2G5 |
| CMKLR1 | Chemerin Chemokine-Like Receptor 1 | Protein Coding | 40 | GC12M 108288 | 2.747 658 | https://www.genecards .org/cgi-bin/carddisp.pl?gene= CMKLR1 |
| MIR212 | MicroRNA 212 | RNA Gene | 20 | GC17M 002050 | 2.747 593 | https://www.genecards .org/cgi-bin/carddisp.pl?gene= MIR212 |
| E2F2 | E2F Transcription Factor 2 | Protein Coding | 40 | GC01M 023656 | 2.746 572 | https://www.genecards .org/cgi-bin/carddisp.pl?gene= E2F2 |
| PRKDC | Protein Kinase, DNA-Activated, Catalytic Subunit | Protein Coding | 48 | GC08M 047773 | 2.745 072 | https://www.genecards .org/cgi-bin/carddisp.pl?gene= PRKDC |
| BATF | Basic Leucine Zipper ATF-Like Transcription Factor | Protein Coding | 40 | GC14P 075523 | 2.737 839 | https://www.genecards .org/cgi-bin/carddisp.pl?gene= BATF |
| GGT6 | Gamma-Glutamyltransferase 6 | Protein Coding | 34 | GC17M 004559 | 2.735 911 | https://www.genecards .org/cgi-bin/carddisp.pl?gene= GGT6 |
| SMTNL2 | Smoothelin Like 2 | Protein Coding | 32 | GC17P 004583 | 2.735 911 | https://www.genecards .org/cgi-bin/carddisp.pl?gene= SMTNL2 |
| SIRT4 | Sirtuin 4 | Protein Coding | 39 | GC12P 120291 | 2.729 069 | https://www.genecards .org/cgi-bin/carddisp.pl?gene= SIRT4 |

| | | | | | | |
|---|---|---|---|---|---|---|
| IGES | Immunoglobulin E Concentration, Serum | Genetic Locus | 4 | GC05U990033 | 2.727148 | https://www.genecards.org/cgi-bin/carddisp.pl?gene=IGES |
| CYB5A | Cytochrome B5 Type A | Protein Coding | 42 | GC18M074250 | 2.726281 | https://www.genecards.org/cgi-bin/carddisp.pl?gene=CYB5A |
| OPTN | Optineurin | Protein Coding | 43 | GC10P013099 | 2.717163 | https://www.genecards.org/cgi-bin/carddisp.pl?gene=OPTN |
| SERPINF2 | Serpin Family F Member 2 | Protein Coding | 44 | GC17P001742 | 2.708069 | https://www.genecards.org/cgi-bin/carddisp.pl?gene=SERPINF2 |
| ITGA1 | Integrin Subunit Alpha 1 | Protein Coding | 42 | GC05P052788 | 2.707134 | https://www.genecards.org/cgi-bin/carddisp.pl?gene=ITGA1 |
| HARS1 | Histidyl-TRNA Synthetase 1 | Protein Coding | 34 | GC05M140673 | 2.701486 | https://www.genecards.org/cgi-bin/carddisp.pl?gene=HARS1 |
| GIN1 | Gypsy Retrotransposon Integrase 1 | Protein Coding | 32 | GC05M103086 | 2.701116 | https://www.genecards.org/cgi-bin/carddisp.pl?gene=GIN1 |
| KIR2DS1 | Killer Cell Immunoglobulin Like Receptor, Two Ig Domains And Short Cytoplasmic Tail 1 | Protein Coding | 24 | GC19Mr00063 | 2.699276 | https://www.genecards.org/cgi-bin/carddisp.pl?gene=KIR2DS1 |
| XIST | X Inactive Specific Transcript | RNA Gene | 25 | GC0XM073820 | 2.692909 | https://www.genecards.org/cgi-bin/carddisp.pl?gene=XIST |
| RPLP1 | Ribosomal Protein Lateral Stalk Subunit P1 | Protein Coding | 38 | GC15P117548 | 2.692557 | https://www.genecards.org/cgi-bin/carddisp.pl?gene=RPLP1 |
| VPS37C | VPS37C Subunit Of ESCRT-I | Protein Coding | 34 | GC11M061130 | 2.692076 | https://www.genecards.org/cgi-bin/carddisp.pl?gene=VPS37C |
| SMIM21 | Small Integral Membrane Protein 21 | Protein Coding | 26 | GC18M075409 | 2.692076 | https://www.genecards.org/cgi-bin/carddisp.pl?gene=SMIM21 |
| EPHB1 | EPH Receptor B1 | Protein Coding | 45 | GC03P134795 | 2.691434 | https://www.genecards.org/cgi-bin/carddisp.pl?gene=EPHB1 |

| | | | | | | |
|---|---|---|---|---|---|---|
| EOMES | Eomesodermin | Protein Coding | 42 | GC03M 027715 | 2.686 572 | https://www.genecards .org/cgi-bin/carddisp.pl?gene= EOMES |
| OLR1 | Oxidized Low Density Lipoprotein Receptor 1 | Protein Coding | 41 | GC12M 021106 | 2.685 989 | https://www.genecards .org/cgi-bin/carddisp.pl?gene= OLR1 |
| RAC2 | Rac Family Small GTPase 2 | Protein Coding | 49 | GC22M 057489 | 2.681 681 | https://www.genecards .org/cgi-bin/carddisp.pl?gene= RAC2 |
| RAMP2 | Receptor Activity Modifying Protein 2 | Protein Coding | 41 | GC17P 042758 | 2.677 371 | https://www.genecards .org/cgi-bin/carddisp.pl?gene= RAMP2 |
| HSD11B2 | Hydroxysteroid 11-Beta Dehydrogenase 2 | Protein Coding | 45 | GC16P 067433 | 2.675 774 | https://www.genecards .org/cgi-bin/carddisp.pl?gene= HSD11B2 |
| CHD7 | Chromodomain Helicase DNA Binding Protein 7 | Protein Coding | 44 | GC08P 060678 | 2.674 856 | https://www.genecards .org/cgi-bin/carddisp.pl?gene= CHD7 |
| HBEGF | Heparin Binding EGF Like Growth Factor | Protein Coding | 41 | GC05M 140332 | 2.668 864 | https://www.genecards .org/cgi-bin/carddisp.pl?gene= HBEGF |
| ENTPD1 | Ectonucleoside Triphosphate Diphosphohydrolase 1 | Protein Coding | 46 | GC10P 095711 | 2.668 351 | https://www.genecards .org/cgi-bin/carddisp.pl?gene= ENTPD1 |
| BMX | BMX Non-Receptor Tyrosine Kinase | Protein Coding | 44 | GC0XP 015392 | 2.667 382 | https://www.genecards .org/cgi-bin/carddisp.pl?gene= BMX |
| MIR451A | MicroRNA 451a | RNA Gene | 19 | GC17M 028861 | 2.665 875 | https://www.genecards .org/cgi-bin/carddisp.pl?gene= MIR451A |
| MALT1 | MALT1 Paracaspase | Protein Coding | 45 | GC18P 058671 | 2.664 839 | https://www.genecards .org/cgi-bin/carddisp.pl?gene= MALT1 |
| C1QC | Complement C1q C Chain | Protein Coding | 44 | GC01P 022643 | 2.663 178 | https://www.genecards .org/cgi-bin/carddisp.pl?gene= C1QC |
| C1QB | Complement C1q B Chain | Protein Coding | 43 | GC01P 022652 | 2.663 178 | https://www.genecards .org/cgi-bin/carddisp.pl?gene= C1QB |

| | | | | | | |
|---|---|---|---|---|---|---|
| HSPA1A | Heat Shock Protein Family A (Hsp70) Member 1A | Protein Coding | 44 | GC06P083713 | 2.660803 | https://www.genecards.org/cgi-bin/carddisp.pl?gene=HSPA1A |
| FLI1 | Fli-1 Proto-Oncogene, ETS Transcription Factor | Protein Coding | 46 | GC11P128686 | 2.660119 | https://www.genecards.org/cgi-bin/carddisp.pl?gene=FLI1 |
| JDP2 | Jun Dimerization Protein 2 | Protein Coding | 39 | GC14P075427 | 2.660119 | https://www.genecards.org/cgi-bin/carddisp.pl?gene=JDP2 |
| CCL23 | C-C Motif Chemokine Ligand 23 | Protein Coding | 33 | GC17M036013 | 2.658659 | https://www.genecards.org/cgi-bin/carddisp.pl?gene=CCL23 |
| FABP4 | Fatty Acid Binding Protein 4 | Protein Coding | 43 | GC08M081478 | 2.658094 | https://www.genecards.org/cgi-bin/carddisp.pl?gene=FABP4 |
| CLEC4A | C-Type Lectin Domain Family 4 Member A | Protein Coding | 37 | GC12P021118 | 2.65647 | https://www.genecards.org/cgi-bin/carddisp.pl?gene=CLEC4A |
| FADS2 | Fatty Acid Desaturase 2 | Protein Coding | 43 | GC11P061792 | 2.655647 | https://www.genecards.org/cgi-bin/carddisp.pl?gene=FADS2 |
| CD46 | CD46 Molecule | Protein Coding | 45 | GC01P207752 | 2.653578 | https://www.genecards.org/cgi-bin/carddisp.pl?gene=CD46 |
| HPX | Hemopexin | Protein Coding | 40 | GC11M006578 | 2.652345 | https://www.genecards.org/cgi-bin/carddisp.pl?gene=HPX |
| CDKAL1 | CDK5 Regulatory Subunit Associated Protein 1 Like 1 | Protein Coding | 38 | GC06P020534 | 2.650796 | https://www.genecards.org/cgi-bin/carddisp.pl?gene=CDKAL1 |
| PDLIM4 | PDZ And LIM Domain 4 | Protein Coding | 39 | GC05P132257 | 2.64983 | https://www.genecards.org/cgi-bin/carddisp.pl?gene=PDLIM4 |
| MAP3K5 | Mitogen-Activated Protein Kinase Kinase Kinase 5 | Protein Coding | 45 | GC06M136557 | 2.6483 | https://www.genecards.org/cgi-bin/carddisp.pl?gene=MAP3K5 |
| FAAH | Fatty Acid Amide Hydrolase | Protein Coding | 46 | GC01P046394 | 2.647988 | https://www.genecards.org/cgi-bin/carddisp.pl?gene=FAAH |

| | | | | | | |
|---|---|---|---|---|---|---|
| TPR | Translocated Promoter Region, Nuclear Basket Protein | Protein Coding | 42 | GC01M 186319 | 2.644 225 | https://www.genecards .org/cgi-bin/carddisp.pl?gene= TPR |
| WARS1 | Tryptophanyl-TRNA Synthetase 1 | Protein Coding | 36 | GC14M 101280 | 2.642 249 | https://www.genecards .org/cgi-bin/carddisp.pl?gene= WARS1 |
| CDSN | Corneodesmosin | Protein Coding | 41 | GC06M 031115 | 2.635 215 | https://www.genecards .org/cgi-bin/carddisp.pl?gene= CDSN |
| VPREB1 | V-Set Pre-B Cell Surrogate Light Chain 1 | Protein Coding | 38 | GC22P 022244 | 2.633 273 | https://www.genecards .org/cgi-bin/carddisp.pl?gene= VPREB1 |
| COL3A1 | Collagen Type III Alpha 1 Chain | Protein Coding | 45 | GC02P 188974 | 2.632 158 | https://www.genecards .org/cgi-bin/carddisp.pl?gene= COL3A1 |
| CDKN2A | Cyclin Dependent Kinase Inhibitor 2A | Protein Coding | 48 | GC09M 021967 | 2.632 003 | https://www.genecards .org/cgi-bin/carddisp.pl?gene= CDKN2A |
| MTNR1A | Melatonin Receptor 1A | Protein Coding | 41 | GC04M 186533 | 2.628 5 | https://www.genecards .org/cgi-bin/carddisp.pl?gene= MTNR1A |
| RNF182 | Ring Finger Protein 182 | Protein Coding | 36 | GC06P 013924 | 2.625 268 | https://www.genecards .org/cgi-bin/carddisp.pl?gene= RNF182 |
| GABAR APL3 | GABA Type A Receptor Associated Protein Like 3 Pseudogene | Pseudo gene | 20 | GC15M 090346 | 2.625 268 | https://www.genecards .org/cgi-bin/carddisp.pl?gene= GABARAPL3 |
| PDE3A | Phosphodiesterase 3A | Protein Coding | 48 | GC12P 021516 | 2.625 078 | https://www.genecards .org/cgi-bin/carddisp.pl?gene= PDE3A |
| SLCO1C 1 | Solute Carrier Organic Anion Transporter Family Member 1C1 | Protein Coding | 38 | GC12P 020695 | 2.625 078 | https://www.genecards .org/cgi-bin/carddisp.pl?gene= SLCO1C1 |
| FTL | Ferritin Light Chain | Protein Coding | 45 | GC19P 048965 | 2.623 077 | https://www.genecards .org/cgi-bin/carddisp.pl?gene= FTL |
| HLA-DRB3 | Major Histocompatibility Complex, Class II, DR Beta 3 | Protein Coding | 29 | GC06M n03715 | 2.621 905 | https://www.genecards .org/cgi-bin/carddisp.pl?gene= HLA-DRB3 |
| CGAS | Cyclic GMP-AMP Synthase | Protein Coding | 29 | GC06M 073414 | 2.619 544 | https://www.genecards .org/cgi- |

| | | | | | | |
|---|---|---|---|---|---|---|
| | | | | | | bin/carddisp.pl?gene=CGAS |
| MAP2K1 | Mitogen-Activated Protein Kinase Kinase 1 | Protein Coding | 51 | GC15P066386 | 2.619184 | https://www.genecards.org/cgi-bin/carddisp.pl?gene=MAP2K1 |
| NFAT5 | Nuclear Factor Of Activated T Cells 5 | Protein Coding | 41 | GC16P069565 | 2.618396 | https://www.genecards.org/cgi-bin/carddisp.pl?gene=NFAT5 |
| FKBP5 | FKBP Prolyl Isomerase 5 | Protein Coding | 45 | GC06M066105 | 2.616987 | https://www.genecards.org/cgi-bin/carddisp.pl?gene=FKBP5 |
| LST1 | Leukocyte Specific Transcript 1 | Protein Coding | 36 | GC06P083696 | 2.615083 | https://www.genecards.org/cgi-bin/carddisp.pl?gene=LST1 |
| IL13RA2 | Interleukin 13 Receptor Subunit Alpha 2 | Protein Coding | 38 | GC0XM115003 | 2.609865 | https://www.genecards.org/cgi-bin/carddisp.pl?gene=IL13RA2 |
| IL36G | Interleukin 36 Gamma | Protein Coding | 36 | GC02P112973 | 2.609079 | https://www.genecards.org/cgi-bin/carddisp.pl?gene=IL36G |
| SFTPB | Surfactant Protein B | Protein Coding | 44 | GC02M085657 | 2.607846 | https://www.genecards.org/cgi-bin/carddisp.pl?gene=SFTPB |
| HDAC2 | Histone Deacetylase 2 | Protein Coding | 49 | GC06M113933 | 2.606236 | https://www.genecards.org/cgi-bin/carddisp.pl?gene=HDAC2 |
| CFP | Complement Factor Properdin | Protein Coding | 41 | GC0XM047624 | 2.603795 | https://www.genecards.org/cgi-bin/carddisp.pl?gene=CFP |
| SFRP1 | Secreted Frizzled Related Protein 1 | Protein Coding | 42 | GC08M041262 | 2.602892 | https://www.genecards.org/cgi-bin/carddisp.pl?gene=SFRP1 |
| MYB | MYB Proto-Oncogene, Transcription Factor | Protein Coding | 48 | GC06P135180 | 2.601309 | https://www.genecards.org/cgi-bin/carddisp.pl?gene=MYB |
| TCN2 | Transcobalamin 2 | Protein Coding | 42 | GC22P036867 | 2.600578 | https://www.genecards.org/cgi-bin/carddisp.pl?gene=TCN2 |
| ITGB7 | Integrin Subunit Beta 7 | Protein Coding | 44 | GC12M053191 | 2.598345 | https://www.genecards.org/cgi-bin/carddisp.pl?gene=ITGB7 |

| | | | | | | |
|---|---|---|---|---|---|---|
| S100A1 | S100 Calcium Binding Protein A1 | Protein Coding | 40 | GC01P 153627 | 2.598 16 | https://www.genecards .org/cgi-bin/carddisp.pl?gene= S100A1 |
| TPSAB1 | Tryptase Alpha/Beta 1 | Protein Coding | 43 | GC16P 001240 | 2.598 006 | https://www.genecards .org/cgi-bin/carddisp.pl?gene= TPSAB1 |
| MIR141 | MicroRNA 141 | RNA Gene | 22 | GC12P 021095 | 2.593 911 | https://www.genecards .org/cgi-bin/carddisp.pl?gene= MIR141 |
| ACTR2 | Actin Related Protein 2 | Protein Coding | 42 | GC02P 065227 | 2.593 283 | https://www.genecards .org/cgi-bin/carddisp.pl?gene= ACTR2 |
| KCNIP4 | Potassium Voltage-Gated Channel Interacting Protein 4 | Protein Coding | 38 | GC04M 020728 | 2.582 652 | https://www.genecards .org/cgi-bin/carddisp.pl?gene= KCNIP4 |
| LRP1 | LDL Receptor Related Protein 1 | Protein Coding | 46 | GC12P 057128 | 2.573 7 | https://www.genecards .org/cgi-bin/carddisp.pl?gene= LRP1 |
| NRM | Nurim | Protein Coding | 33 | GC06M 065880 | 2.569 693 | https://www.genecards .org/cgi-bin/carddisp.pl?gene= NRM |
| CASTOR 1 | Cytosolic Arginine Sensor For MTORC1 Subunit 1 | Protein Coding | 23 | GC22M 057166 | 2.567 595 | https://www.genecards .org/cgi-bin/carddisp.pl?gene= CASTOR1 |
| SIRT6 | Sirtuin 6 | Protein Coding | 43 | GC19M 004174 | 2.565 393 | https://www.genecards .org/cgi-bin/carddisp.pl?gene= SIRT6 |
| CNTF | Ciliary Neurotrophic Factor | Protein Coding | 39 | GC11P 058622 | 2.564 64 | https://www.genecards .org/cgi-bin/carddisp.pl?gene= CNTF |
| MIR210 | MicroRNA 210 | RNA Gene | 21 | GC11M 003193 | 2.556 592 | https://www.genecards .org/cgi-bin/carddisp.pl?gene= MIR210 |
| TANK | TRAF Family Member Associated NFKB Activator | Protein Coding | 41 | GC02P 161136 | 2.555 561 | https://www.genecards .org/cgi-bin/carddisp.pl?gene= TANK |
| MIR129-1 | MicroRNA 129-1 | RNA Gene | 18 | GC07P 128207 | 2.552 329 | https://www.genecards .org/cgi-bin/carddisp.pl?gene= MIR129-1 |
| SMO | Smoothened, Frizzled Class Receptor | Protein Coding | 47 | GC07P 131934 | 2.549 778 | https://www.genecards .org/cgi- |

| | | | | | | |
|---|---|---|---|---|---|---|
| | | | | | | bin/carddisp.pl?gene=SMO |
| CTHRC1 | Collagen Triple Helix Repeat Containing 1 | Protein Coding | 40 | GC08P103371 | 2.548314 | https://www.genecards.org/cgi-bin/carddisp.pl?gene=CTHRC1 |
| ABCA1 | ATP Binding Cassette Subfamily A Member 1 | Protein Coding | 46 | GC09M104781 | 2.548271 | https://www.genecards.org/cgi-bin/carddisp.pl?gene=ABCA1 |
| PTPRT | Protein Tyrosine Phosphatase Receptor Type T | Protein Coding | 43 | GC20M042072 | 2.544767 | https://www.genecards.org/cgi-bin/carddisp.pl?gene=PTPRT |
| MERTK | MER Proto-Oncogene, Tyrosine Kinase | Protein Coding | 49 | GC02P111898 | 2.538073 | https://www.genecards.org/cgi-bin/carddisp.pl?gene=MERTK |
| TACR2 | Tachykinin Receptor 2 | Protein Coding | 41 | GC10M069403 | 2.537549 | https://www.genecards.org/cgi-bin/carddisp.pl?gene=TACR2 |
| LSP1 | Lymphocyte Specific Protein 1 | Protein Coding | 40 | GC11P001878 | 2.530858 | https://www.genecards.org/cgi-bin/carddisp.pl?gene=LSP1 |
| ZNRD2 | Zinc Ribbon Domain Containing 2 | Protein Coding | 28 | GC11P069991 | 2.530775 | https://www.genecards.org/cgi-bin/carddisp.pl?gene=ZNRD2 |
| LINC00426 | Long Intergenic Non-Protein Coding RNA 426 | RNA Gene | 16 | GC13M030377 | 2.530775 | https://www.genecards.org/cgi-bin/carddisp.pl?gene=LINC00426 |
| CXCR2P1 | C-X-C Motif Chemokine Receptor 2 Pseudogene 1 | Pseudogene | 14 | GC02M218059 | 2.530775 | https://www.genecards.org/cgi-bin/carddisp.pl?gene=CXCR2P1 |
| LINC02384 | Long Intergenic Non-Protein Coding RNA 2384 | RNA Gene | 13 | GC12M068334 | 2.530775 | https://www.genecards.org/cgi-bin/carddisp.pl?gene=LINC02384 |
| CD160 | CD160 Molecule | Protein Coding | 38 | GC01P145719 | 2.527282 | https://www.genecards.org/cgi-bin/carddisp.pl?gene=CD160 |
| TYROBP | Transmembrane Immune Signaling Adaptor TYROBP | Protein Coding | 42 | GC19M035904 | 2.523565 | https://www.genecards.org/cgi-bin/carddisp.pl?gene=TYROBP |
| VSIG4 | V-Set And Immunoglobulin Domain Containing 4 | Protein Coding | 36 | GC0XM066021 | 2.520731 | https://www.genecards.org/cgi-bin/carddisp.pl?gene=VSIG4 |

| | | | | | | |
|---|---|---|---|---|---|---|
| ADIPOR 1 | Adiponectin Receptor 1 | Protein Coding | 43 | GC01M 202940 | 2.518 602 | https://www.genecards .org/cgi-bin/carddisp.pl?gene= ADIPOR1 |
| MIR214 | MicroRNA 214 | RNA Gene | 22 | GC01M 172234 | 2.517 413 | https://www.genecards .org/cgi-bin/carddisp.pl?gene= MIR214 |
| RDX | Radixin | Protein Coding | 46 | GC11M 109864 | 2.515 399 | https://www.genecards .org/cgi-bin/carddisp.pl?gene= RDX |
| TNFRSF1 0C | TNF Receptor Superfamily Member 10c | Protein Coding | 37 | GC08P 023102 | 2.511 104 | https://www.genecards .org/cgi-bin/carddisp.pl?gene= TNFRSF10C |
| GMCL2 | Germ Cell-Less 2, Spermatogenesis Associated | Protein Coding | 19 | GC05M 178189 | 2.509 099 | https://www.genecards .org/cgi-bin/carddisp.pl?gene= GMCL2 |
| MIR346 | MicroRNA 346 | RNA Gene | 21 | GC10M 086264 | 2.506 927 | https://www.genecards .org/cgi-bin/carddisp.pl?gene= MIR346 |
| LILRA3 | Leukocyte Immunoglobulin Like Receptor A3 | Protein Coding | 31 | GC19M p00270 | 2.505 285 | https://www.genecards .org/cgi-bin/carddisp.pl?gene= LILRA3 |
| S1PR1 | Sphingosine-1-Phosphate Receptor 1 | Protein Coding | 42 | GC01P 101236 | 2.504 356 | https://www.genecards .org/cgi-bin/carddisp.pl?gene= S1PR1 |
| PGK1 | Phosphoglycerate Kinase 1 | Protein Coding | 47 | GC0XP 077994 | 2.502 916 | https://www.genecards .org/cgi-bin/carddisp.pl?gene= PGK1 |
| GLI3 | GLI Family Zinc Finger 3 | Protein Coding | 46 | GC07M 041960 | 2.501 774 | https://www.genecards .org/cgi-bin/carddisp.pl?gene= GLI3 |
| GAPDH | Glyceraldehyde-3-Phosphate Dehydrogenase | Protein Coding | 47 | GC12P 021056 | 2.501 425 | https://www.genecards .org/cgi-bin/carddisp.pl?gene= GAPDH |
| FOSL1 | FOS Like 1, AP-1 Transcription Factor Subunit | Protein Coding | 43 | GC11M 089830 | 2.501 411 | https://www.genecards .org/cgi-bin/carddisp.pl?gene= FOSL1 |
| SIL1 | SIL1 Nucleotide Exchange Factor | Protein Coding | 40 | GC05M 138957 | 2.498 796 | https://www.genecards .org/cgi-bin/carddisp.pl?gene= SIL1 |
| POU2F1 | POU Class 2 Homeobox 1 | Protein Coding | 42 | GC01P 167190 | 2.497 87 | https://www.genecards .org/cgi- |

| | | | | | | |
|---|---|---|---|---|---|---|
| | | | | | | bin/carddisp.pl?gene=POU2F1 |
| KRT18P39 | Keratin 18 Pseudogene 39 | Pseudo gene | 9 | GC02M203765 | 2.49787 | https://www.genecards.org/cgi-bin/carddisp.pl?gene=KRT18P39 |
| PSMG2 | Proteasome Assembly Chaperone 2 | Protein Coding | 37 | GC18P017449 | 2.496347 | https://www.genecards.org/cgi-bin/carddisp.pl?gene=PSMG2 |
| TNFSF8 | TNF Superfamily Member 8 | Protein Coding | 36 | GC09M114893 | 2.495427 | https://www.genecards.org/cgi-bin/carddisp.pl?gene=TNFSF8 |
| ITGB3 | Integrin Subunit Beta 3 | Protein Coding | 49 | GC17P055923 | 2.493982 | https://www.genecards.org/cgi-bin/carddisp.pl?gene=ITGB3 |
| MIR518A1 | MicroRNA 518a-1 | RNA Gene | 18 | GC19P067345 | 2.491659 | https://www.genecards.org/cgi-bin/carddisp.pl?gene=MIR518A1 |
| RASGRP4 | RAS Guanyl Releasing Protein 4 | Protein Coding | 36 | GC19M038409 | 2.490752 | https://www.genecards.org/cgi-bin/carddisp.pl?gene=RASGRP4 |
| VEGFD | Vascular Endothelial Growth Factor D | Protein Coding | 32 | GC0XM015345 | 2.487922 | https://www.genecards.org/cgi-bin/carddisp.pl?gene=VEGFD |
| PTGER2 | Prostaglandin E Receptor 2 | Protein Coding | 46 | GC14P052314 | 2.478814 | https://www.genecards.org/cgi-bin/carddisp.pl?gene=PTGER2 |
| KCNJ11 | Potassium Inwardly Rectifying Channel Subfamily J Member 11 | Protein Coding | 46 | GC11M017592 | 2.473814 | https://www.genecards.org/cgi-bin/carddisp.pl?gene=KCNJ11 |
| CYLD | CYLD Lysine 63 Deubiquitinase | Protein Coding | 47 | GC16P050742 | 2.469159 | https://www.genecards.org/cgi-bin/carddisp.pl?gene=CYLD |
| TMPO | Thymopoietin | Protein Coding | 44 | GC12P098515 | 2.466669 | https://www.genecards.org/cgi-bin/carddisp.pl?gene=TMPO |
| HSPA1L | Heat Shock Protein Family A (Hsp70) Member 1 Like | Protein Coding | 44 | GC06M031809 | 2.465886 | https://www.genecards.org/cgi-bin/carddisp.pl?gene=HSPA1L |
| WEE1 | WEE1 G2 Checkpoint Kinase | Protein Coding | 45 | GC11P009573 | 2.461549 | https://www.genecards.org/cgi-bin/carddisp.pl?gene=WEE1 |

| | | | | | | |
|---|---|---|---|---|---|---|
| GPX1 | Glutathione Peroxidase 1 | Protein Coding | 45 | GC03M 051185 | 2.459 566 | https://www.genecards.org/cgi-bin/carddisp.pl?gene=GPX1 |
| PTPN3 | Protein Tyrosine Phosphatase Non-Receptor Type 3 | Protein Coding | 44 | GC09M 109375 | 2.457 529 | https://www.genecards.org/cgi-bin/carddisp.pl?gene=PTPN3 |
| TRG | T Cell Receptor Gamma Locus | Protein Coding | 13 | GC07M 038240 | 2.456 943 | https://www.genecards.org/cgi-bin/carddisp.pl?gene=TRG |
| RAP1A | RAP1A, Member Of RAS Oncogene Family | Protein Coding | 43 | GC01P 111542 | 2.454 115 | https://www.genecards.org/cgi-bin/carddisp.pl?gene=RAP1A |
| NRP2 | Neuropilin 2 | Protein Coding | 44 | GC02P 205681 | 2.450 089 | https://www.genecards.org/cgi-bin/carddisp.pl?gene=NRP2 |
| SEMA3F | Semaphorin 3F | Protein Coding | 41 | GC03P 050360 | 2.450 089 | https://www.genecards.org/cgi-bin/carddisp.pl?gene=SEMA3F |
| SPI1 | Spi-1 Proto-Oncogene | Protein Coding | 42 | GC11M 089364 | 2.447 559 | https://www.genecards.org/cgi-bin/carddisp.pl?gene=SPI1 |
| ATP1A1 | ATPase Na+/K+ Transporting Subunit Alpha 1 | Protein Coding | 48 | GC01P 116372 | 2.441 431 | https://www.genecards.org/cgi-bin/carddisp.pl?gene=ATP1A1 |
| C1QBP | Complement C1q Binding Protein | Protein Coding | 43 | GC17M 005432 | 2.441 431 | https://www.genecards.org/cgi-bin/carddisp.pl?gene=C1QBP |
| MED1 | Mediator Complex Subunit 1 | Protein Coding | 40 | GC17M 039404 | 2.441 431 | https://www.genecards.org/cgi-bin/carddisp.pl?gene=MED1 |
| AQP5 | Aquaporin 5 | Protein Coding | 45 | GC12P 049961 | 2.440 441 | https://www.genecards.org/cgi-bin/carddisp.pl?gene=AQP5 |
| SSNA1 | SS Nuclear Autoantigen 1 | Protein Coding | 35 | GC09P 137188 | 2.440 441 | https://www.genecards.org/cgi-bin/carddisp.pl?gene=SSNA1 |
| FGF23 | Fibroblast Growth Factor 23 | Protein Coding | 44 | GC12M 004368 | 2.439 505 | https://www.genecards.org/cgi-bin/carddisp.pl?gene=FGF23 |

| | | | | | | |
|---|---|---|---|---|---|---|
| HLA-S | Major Histocompatibility Complex, Class I, S (Pseudogene) | Pseudo gene | 9 | GC06M 031381 | 2.431 741 | https://www.genecards .org/cgi-bin/carddisp.pl?gene= HLA-S |
| LOC1113 65141 | NOS2 5' Regulatory Region | Biologi cal Region | 3 | GC17P 027800 | 2.429 671 | https://www.genecards .org/cgi-bin/carddisp.pl?gene= LOC111365141 |
| XCL1 | X-C Motif Chemokine Ligand 1 | Protein Coding | 35 | GC01P 168576 | 2.429 318 | https://www.genecards .org/cgi-bin/carddisp.pl?gene= XCL1 |
| AQP1 | Aquaporin 1 (Colton Blood Group) | Protein Coding | 45 | GC07P 030911 | 2.423 884 | https://www.genecards .org/cgi-bin/carddisp.pl?gene= AQP1 |
| HSP90B1 | Heat Shock Protein 90 Beta Family Member 1 | Protein Coding | 45 | GC12P 103930 | 2.418 81 | https://www.genecards .org/cgi-bin/carddisp.pl?gene= HSP90B1 |
| CYBA | Cytochrome B-245 Alpha Chain | Protein Coding | 46 | GC16M 088643 | 2.417 008 | https://www.genecards .org/cgi-bin/carddisp.pl?gene= CYBA |
| PPAT | Phosphoribosyl Pyrophosphate Amidotransferase | Protein Coding | 43 | GC04M 056393 | 2.416 131 | https://www.genecards .org/cgi-bin/carddisp.pl?gene= PPAT |
| LTB4R2 | Leukotriene B4 Receptor 2 | Protein Coding | 41 | GC14P 032722 | 2.416 099 | https://www.genecards .org/cgi-bin/carddisp.pl?gene= LTB4R2 |
| LY6G6E | Lymphocyte Antigen 6 Family Member G6E | Pseudo gene | 24 | GC06M 065966 | 2.414 783 | https://www.genecards .org/cgi-bin/carddisp.pl?gene= LY6G6E |
| C1orf141 | Chromosome 1 Open Reading Frame 141 | Protein Coding | 28 | GC01M 067092 | 2.414 706 | https://www.genecards .org/cgi-bin/carddisp.pl?gene= C1orf141 |
| PLAA | Phospholipase A2 Activating Protein | Protein Coding | 43 | GC09M 026903 | 2.412 54 | https://www.genecards .org/cgi-bin/carddisp.pl?gene= PLAA |
| BBC3 | BCL2 Binding Component 3 | Protein Coding | 38 | GC19M 047220 | 2.411 876 | https://www.genecards .org/cgi-bin/carddisp.pl?gene= BBC3 |
| SOX9 | SRY-Box Transcription Factor 9 | Protein Coding | 44 | GC17P 072121 | 2.411 608 | https://www.genecards .org/cgi-bin/carddisp.pl?gene= SOX9 |
| OGG1 | 8-Oxoguanine DNA Glycosylase | Protein Coding | 45 | GC03P 012414 | 2.408 346 | https://www.genecards .org/cgi- |

| | | | | | | |
|---|---|---|---|---|---|---|
| | | | | | | bin/carddisp.pl?gene=OGG1 |
| CUL1 | Cullin 1 | Protein Coding | 42 | GC07P148697 | 2.408346 | https://www.genecards.org/cgi-bin/carddisp.pl?gene=CUL1 |
| PRRT1 | Proline Rich Transmembrane Protein 1 | Protein Coding | 32 | GC06M065984 | 2.408346 | https://www.genecards.org/cgi-bin/carddisp.pl?gene=PRRT1 |
| CTNNB1 | Catenin Beta 1 | Protein Coding | 50 | GC03P041236 | 2.405236 | https://www.genecards.org/cgi-bin/carddisp.pl?gene=CTNNB1 |
| TGM2 | Transglutaminase 2 | Protein Coding | 46 | GC20M038127 | 2.405123 | https://www.genecards.org/cgi-bin/carddisp.pl?gene=TGM2 |
| MIR18A | MicroRNA 18a | RNA Gene | 18 | GC13P091560 | 2.404482 | https://www.genecards.org/cgi-bin/carddisp.pl?gene=MIR18A |
| GRP | Gastrin Releasing Peptide | Protein Coding | 39 | GC18P059220 | 2.402391 | https://www.genecards.org/cgi-bin/carddisp.pl?gene=GRP |
| SIRT3 | Sirtuin 3 | Protein Coding | 45 | GC11M000215 | 2.399146 | https://www.genecards.org/cgi-bin/carddisp.pl?gene=SIRT3 |
| SPN | Sialophorin | Protein Coding | 38 | GC16P029662 | 2.392903 | https://www.genecards.org/cgi-bin/carddisp.pl?gene=SPN |
| GSDMB | Gasdermin B | Protein Coding | 36 | GC17M039904 | 2.392119 | https://www.genecards.org/cgi-bin/carddisp.pl?gene=GSDMB |
| C6orf47 | Chromosome 6 Open Reading Frame 47 | Protein Coding | 29 | GC06M065961 | 2.392119 | https://www.genecards.org/cgi-bin/carddisp.pl?gene=C6orf47 |
| HTRA2 | HtrA Serine Peptidase 2 | Protein Coding | 44 | GC02P074529 | 2.389671 | https://www.genecards.org/cgi-bin/carddisp.pl?gene=HTRA2 |
| CHGA | Chromogranin A | Protein Coding | 43 | GC14P092944 | 2.38497 | https://www.genecards.org/cgi-bin/carddisp.pl?gene=CHGA |
| ABCC11 | ATP Binding Cassette Subfamily C Member 11 | Protein Coding | 42 | GC16M048166 | 2.38497 | https://www.genecards.org/cgi-bin/carddisp.pl?gene=ABCC11 |

| | | | | | | |
|---|---|---|---|---|---|---|
| MSRA | Methionine Sulfoxide Reductase A | Protein Coding | 41 | GC08P 010054 | 2.384 97 | https://www.genecards .org/cgi-bin/carddisp.pl?gene= MSRA |
| MPG | N-Methylpurine DNA Glycosylase | Protein Coding | 39 | GC16P 011593 | 2.384 97 | https://www.genecards .org/cgi-bin/carddisp.pl?gene= MPG |
| TIMD4 | T Cell Immunoglobulin And Mucin Domain Containing 4 | Protein Coding | 36 | GC05M 156919 | 2.384 97 | https://www.genecards .org/cgi-bin/carddisp.pl?gene= TIMD4 |
| GSK3B | Glycogen Synthase Kinase 3 Beta | Protein Coding | 47 | GC03M 119821 | 2.380 347 | https://www.genecards .org/cgi-bin/carddisp.pl?gene= GSK3B |
| DCAF5 | DDB1 And CUL4 Associated Factor 5 | Protein Coding | 33 | GC14M 069050 | 2.380 035 | https://www.genecards .org/cgi-bin/carddisp.pl?gene= DCAF5 |
| SLC46A1 | Solute Carrier Family 46 Member 1 | Protein Coding | 41 | GC17M 035136 | 2.379 748 | https://www.genecards .org/cgi-bin/carddisp.pl?gene= SLC46A1 |
| MIR338 | MicroRNA 338 | RNA Gene | 18 | GC17M 081126 | 2.370 824 | https://www.genecards .org/cgi-bin/carddisp.pl?gene= MIR338 |
| IL9R | Interleukin 9 Receptor | Protein Coding | 35 | GC0XP 155997 | 2.370 452 | https://www.genecards .org/cgi-bin/carddisp.pl?gene=I L9R |
| VCP | Valosin Containing Protein | Protein Coding | 48 | GC09M 035056 | 2.368 829 | https://www.genecards .org/cgi-bin/carddisp.pl?gene= VCP |
| CTSD | Cathepsin D | Protein Coding | 50 | GC11M 001752 | 2.368 061 | https://www.genecards .org/cgi-bin/carddisp.pl?gene= CTSD |
| GRM5 | Glutamate Metabotropic Receptor 5 | Protein Coding | 46 | GC11M 088504 | 2.367 877 | https://www.genecards .org/cgi-bin/carddisp.pl?gene= GRM5 |
| DDX6 | DEAD-Box Helicase 6 | Protein Coding | 44 | GC11M 118748 | 2.367 877 | https://www.genecards .org/cgi-bin/carddisp.pl?gene= DDX6 |
| PTPRM | Protein Tyrosine Phosphatase Receptor Type M | Protein Coding | 43 | GC18P 007557 | 2.367 877 | https://www.genecards .org/cgi-bin/carddisp.pl?gene= PTPRM |
| CDK5RA P2 | CDK5 Regulatory Subunit Associated Protein 2 | Protein Coding | 41 | GC09M 120388 | 2.367 877 | https://www.genecards .org/cgi- |

| | | | | | | |
|---|---|---|---|---|---|---|
| | | | | | | bin/carddisp.pl?gene=CDK5RAP2 |
| CEP57 | Centrosomal Protein 57 | Protein Coding | 41 | GC11P 095789 | 2.367 877 | https://www.genecards.org/cgi-bin/carddisp.pl?gene=CEP57 |
| STAG1 | Stromal Antigen 1 | Protein Coding | 41 | GC03M 136336 | 2.367 877 | https://www.genecards.org/cgi-bin/carddisp.pl?gene=STAG1 |
| MTF1 | Metal Regulatory Transcription Factor 1 | Protein Coding | 40 | GC01M 037810 | 2.367 877 | https://www.genecards.org/cgi-bin/carddisp.pl?gene=MTF1 |
| PTTG1 | PTTG1 Regulator Of Sister Chromatid Separation, Securin | Protein Coding | 40 | GC05P 160422 | 2.367 877 | https://www.genecards.org/cgi-bin/carddisp.pl?gene=PTTG1 |
| TPD52 | Tumor Protein D52 | Protein Coding | 40 | GC08M 080034 | 2.367 877 | https://www.genecards.org/cgi-bin/carddisp.pl?gene=TPD52 |
| TREH | Trehalase | Protein Coding | 40 | GC11M 118657 | 2.367 877 | https://www.genecards.org/cgi-bin/carddisp.pl?gene=TREH |
| GRHL2 | Grainyhead Like Transcription Factor 2 | Protein Coding | 39 | GC08P 101492 | 2.367 877 | https://www.genecards.org/cgi-bin/carddisp.pl?gene=GRHL2 |
| ABHD6 | Abhydrolase Domain Containing 6, Acylglycerol Lipase | Protein Coding | 38 | GC03P 058238 | 2.367 877 | https://www.genecards.org/cgi-bin/carddisp.pl?gene=ABHD6 |
| RABEP1 | Rabaptin, RAB GTPase Binding Effector Protein 1 | Protein Coding | 38 | GC17P 005282 | 2.367 877 | https://www.genecards.org/cgi-bin/carddisp.pl?gene=RABEP1 |
| CLYBL | Citramalyl-CoA Lyase | Protein Coding | 37 | GC13P 099606 | 2.367 877 | https://www.genecards.org/cgi-bin/carddisp.pl?gene=CLYBL |
| FADS3 | Fatty Acid Desaturase 3 | Protein Coding | 36 | GC11M 061873 | 2.367 877 | https://www.genecards.org/cgi-bin/carddisp.pl?gene=FADS3 |
| PPIL4 | Peptidylprolyl Isomerase Like 4 | Protein Coding | 36 | GC06M 149504 | 2.367 877 | https://www.genecards.org/cgi-bin/carddisp.pl?gene=PPIL4 |
| PPM1L | Protein Phosphatase, Mg2+/Mn2+ Dependent 1L | Protein Coding | 36 | GC03P 160755 | 2.367 877 | https://www.genecards.org/cgi-bin/carddisp.pl?gene=PPM1L |

| | | | | | | |
|---|---|---|---|---|---|---|
| YPEL5 | Yippee Like 5 | Protein Coding | 36 | GC02P 030108 | 2.367 877 | https://www.genecards .org/cgi-bin/carddisp.pl?gene= YPEL5 |
| CLNK | Cytokine Dependent Hematopoietic Cell Linker | Protein Coding | 35 | GC04M 010491 | 2.367 877 | https://www.genecards .org/cgi-bin/carddisp.pl?gene= CLNK |
| FAM124 A | Family With Sequence Similarity 124 Member A | Protein Coding | 34 | GC13P 051222 | 2.367 877 | https://www.genecards .org/cgi-bin/carddisp.pl?gene= FAM124A |
| RPP14 | Ribonuclease P/MRP Subunit P14 | Protein Coding | 34 | GC03P 058295 | 2.367 877 | https://www.genecards .org/cgi-bin/carddisp.pl?gene= RPP14 |
| TXNDC1 1 | Thioredoxin Domain Containing 11 | Protein Coding | 34 | GC16M 011679 | 2.367 877 | https://www.genecards .org/cgi-bin/carddisp.pl?gene= TXNDC11 |
| DDA1 | DET1 And DDB1 Associated 1 | Protein Coding | 33 | GC19P 066218 | 2.367 877 | https://www.genecards .org/cgi-bin/carddisp.pl?gene= DDA1 |
| ACOXL | Acyl-CoA Oxidase Like | Protein Coding | 32 | GC02P 110732 | 2.367 877 | https://www.genecards .org/cgi-bin/carddisp.pl?gene= ACOXL |
| AHNAK2 | AHNAK Nucleoprotein 2 | Protein Coding | 32 | GC14M 104937 | 2.367 877 | https://www.genecards .org/cgi-bin/carddisp.pl?gene= AHNAK2 |
| ANO8 | Anoctamin 8 | Protein Coding | 32 | GC19M 017324 | 2.367 877 | https://www.genecards .org/cgi-bin/carddisp.pl?gene= ANO8 |
| GUCY1B 2 | Guanylate Cyclase 1 Soluble Subunit Beta 2 (Pseudogene) | Pseudo gene | 31 | GC13M 050994 | 2.367 877 | https://www.genecards .org/cgi-bin/carddisp.pl?gene= GUCY1B2 |
| ARL14 | ADP Ribosylation Factor Like GTPase 14 | Protein Coding | 30 | GC03P 160677 | 2.367 877 | https://www.genecards .org/cgi-bin/carddisp.pl?gene= ARL14 |
| ZNF438 | Zinc Finger Protein 438 | Protein Coding | 30 | GC10M 030820 | 2.367 877 | https://www.genecards .org/cgi-bin/carddisp.pl?gene= ZNF438 |
| TMEM18 7 | Transmembrane Protein 187 | Protein Coding | 28 | GC0XP 153972 | 2.367 877 | https://www.genecards .org/cgi-bin/carddisp.pl?gene= TMEM187 |

| | | | | | | |
|---|---|---|---|---|---|---|
| FLACC1 | Flagellum Associated Containing Coiled-Coil Domains 1 | Protein Coding | 26 | GC02M 201423 | 2.367 877 | https://www.genecards .org/cgi-bin/carddisp.pl?gene= FLACC1 |
| DRAIC | Downregulated RNA In Cancer, Inhibitor Of Cell Invasion And Migration | RNA Gene | 15 | GC15P 117547 | 2.367 877 | https://www.genecards .org/cgi-bin/carddisp.pl?gene= DRAIC |
| LOC1005 06023 | Uncharacterized LOC100506023 | RNA Gene | 11 | GC01M 173235 | 2.367 877 | https://www.genecards .org/cgi-bin/carddisp.pl?gene= LOC100506023 |
| LOC1005 06403 | Uncharacterized LOC100506403 | RNA Gene | 10 | GC21M 035372 | 2.367 877 | https://www.genecards .org/cgi-bin/carddisp.pl?gene= LOC100506403 |
| RPL17P2 2 | Ribosomal Protein L17 Pseudogene 22 | Pseudo gene | 8 | GC05P 056136 | 2.367 877 | https://www.genecards .org/cgi-bin/carddisp.pl?gene= RPL17P22 |
| FNIP1 | Folliculin Interacting Protein 1 | Protein Coding | 37 | GC05M 131641 | 2.362 982 | https://www.genecards .org/cgi-bin/carddisp.pl?gene= FNIP1 |
| PREP | Prolyl Endopeptidase | Protein Coding | 41 | GC06M 105277 | 2.361 022 | https://www.genecards .org/cgi-bin/carddisp.pl?gene= PREP |
| SHH | Sonic Hedgehog Signaling Molecule | Protein Coding | 48 | GC07M 155799 | 2.360 765 | https://www.genecards .org/cgi-bin/carddisp.pl?gene= SHH |
| FOLR1 | Folate Receptor Alpha | Protein Coding | 46 | GC11P 072190 | 2.360 765 | https://www.genecards .org/cgi-bin/carddisp.pl?gene= FOLR1 |
| PIN1 | Peptidylprolyl Cis/Trans Isomerase, NIMA-Interacting 1 | Protein Coding | 45 | GC19P 009835 | 2.360 765 | https://www.genecards .org/cgi-bin/carddisp.pl?gene= PIN1 |
| RB1 | RB Transcriptional Corepressor 1 | Protein Coding | 45 | GC13P 048303 | 2.360 765 | https://www.genecards .org/cgi-bin/carddisp.pl?gene= RB1 |
| NKIRAS 1 | NFKB Inhibitor Interacting Ras Like 1 | Protein Coding | 35 | GC03M 024218 | 2.360 765 | https://www.genecards .org/cgi-bin/carddisp.pl?gene= NKIRAS1 |
| MIR410 | MicroRNA 410 | RNA Gene | 18 | GC14P 109990 | 2.360 765 | https://www.genecards .org/cgi-bin/carddisp.pl?gene= MIR410 |
| PTGER3 | Prostaglandin E Receptor 3 | Protein Coding | 45 | GC01M 070852 | 2.358 193 | https://www.genecards .org/cgi- |

| | | | | | | |
|---|---|---|---|---|---|---|
| | | | | | | bin/carddisp.pl?gene=PTGER3 |
| DEFA1 | Defensin Alpha 1 | Protein Coding | 40 | GC08M006977 | 2.354609 | https://www.genecards.org/cgi-bin/carddisp.pl?gene=DEFA1 |
| PRELP | Proline And Arginine Rich End Leucine Rich Repeat Protein | Protein Coding | 41 | GC01P203475 | 2.349858 | https://www.genecards.org/cgi-bin/carddisp.pl?gene=PRELP |
| GAL | Galanin And GMAP Prepropeptide | Protein Coding | 43 | GC11P070219 | 2.349816 | https://www.genecards.org/cgi-bin/carddisp.pl?gene=GAL |
| IGFBP5 | Insulin Like Growth Factor Binding Protein 5 | Protein Coding | 41 | GC02M216672 | 2.349499 | https://www.genecards.org/cgi-bin/carddisp.pl?gene=IGFBP5 |
| FNDC5 | Fibronectin Type III Domain Containing 5 | Protein Coding | 33 | GC01M033185 | 2.348042 | https://www.genecards.org/cgi-bin/carddisp.pl?gene=FNDC5 |
| MIR199A1 | MicroRNA 199a-1 | RNA Gene | 20 | GC19M010817 | 2.348042 | https://www.genecards.org/cgi-bin/carddisp.pl?gene=MIR199A1 |
| IFI44L | Interferon Induced Protein 44 Like | Protein Coding | 33 | GC01P078619 | 2.345003 | https://www.genecards.org/cgi-bin/carddisp.pl?gene=IFI44L |
| ABHD16A | Abhydrolase Domain Containing 16A, Phospholipase | Protein Coding | 34 | GC06M065964 | 2.341229 | https://www.genecards.org/cgi-bin/carddisp.pl?gene=ABHD16A |
| CYBB | Cytochrome B-245 Beta Chain | Protein Coding | 46 | GC0XP037780 | 2.334452 | https://www.genecards.org/cgi-bin/carddisp.pl?gene=CYBB |
| EXOSC10 | Exosome Component 10 | Protein Coding | 37 | GC01M011067 | 2.327669 | https://www.genecards.org/cgi-bin/carddisp.pl?gene=EXOSC10 |
| G3BP1 | G3BP Stress Granule Assembly Factor 1 | Protein Coding | 39 | GC05P151771 | 2.326586 | https://www.genecards.org/cgi-bin/carddisp.pl?gene=G3BP1 |
| MYDGF | Myeloid Derived Growth Factor | Protein Coding | 32 | GC19M004641 | 2.326419 | https://www.genecards.org/cgi-bin/carddisp.pl?gene=MYDGF |
| PLA2G7 | Phospholipase A2 Group VII | Protein Coding | 48 | GC06M046704 | 2.323493 | https://www.genecards.org/cgi-bin/carddisp.pl?gene=PLA2G7 |

| | | | | | | |
|---|---|---|---|---|---|---|
| MDK | Midkine | Protein Coding | 42 | GC11P 046380 | 2.320 974 | https://www.genecards .org/cgi-bin/carddisp.pl?gene= MDK |
| DNAH8 | Dynein Axonemal Heavy Chain 8 | Protein Coding | 37 | GC06P 083874 | 2.320 009 | https://www.genecards .org/cgi-bin/carddisp.pl?gene= DNAH8 |
| DHX9 | DExH-Box Helicase 9 | Protein Coding | 40 | GC01P 182839 | 2.319 866 | https://www.genecards .org/cgi-bin/carddisp.pl?gene= DHX9 |
| AREG | Amphiregulin | Protein Coding | 41 | GC04P 074445 | 2.319 048 | https://www.genecards .org/cgi-bin/carddisp.pl?gene= AREG |
| PTH1R | Parathyroid Hormone 1 Receptor | Protein Coding | 47 | GC03P 046877 | 2.318 319 | https://www.genecards .org/cgi-bin/carddisp.pl?gene= PTH1R |
| EPHA2 | EPH Receptor A2 | Protein Coding | 50 | GC01M 016124 | 2.317 036 | https://www.genecards .org/cgi-bin/carddisp.pl?gene= EPHA2 |
| DSPP | Dentin Sialophosphoprotein | Protein Coding | 38 | GC04P 087608 | 2.315 514 | https://www.genecards .org/cgi-bin/carddisp.pl?gene= DSPP |
| FBXL19 | F-Box And Leucine Rich Repeat Protein 19 | Protein Coding | 34 | GC16P 041107 | 2.315 316 | https://www.genecards .org/cgi-bin/carddisp.pl?gene= FBXL19 |
| CYP11B2 | Cytochrome P450 Family 11 Subfamily B Member 2 | Protein Coding | 46 | GC08M 142910 | 2.315 202 | https://www.genecards .org/cgi-bin/carddisp.pl?gene= CYP11B2 |
| IFN1@ | Interferon, Type 1, Cluster | Gene Cluster | 4 | GC09U 990039 | 2.312 373 | https://www.genecards .org/cgi-bin/carddisp.pl?gene=I FN1%40 |
| XK | X-Linked Kx Blood Group | Protein Coding | 40 | GC0XP 037685 | 2.310 287 | https://www.genecards .org/cgi-bin/carddisp.pl?gene= XK |
| RPS6KA 4 | Ribosomal Protein S6 Kinase A4 | Protein Coding | 45 | GC11P 064372 | 2.304 78 | https://www.genecards .org/cgi-bin/carddisp.pl?gene= RPS6KA4 |
| RPS6KA 5 | Ribosomal Protein S6 Kinase A5 | Protein Coding | 44 | GC14M 090847 | 2.304 78 | https://www.genecards .org/cgi-bin/carddisp.pl?gene= RPS6KA5 |

| | | | | | | |
|---|---|---|---|---|---|---|
| PIBF1 | Progesterone Immunomodulatory Binding Factor 1 | Protein Coding | 40 | GC13P 072782 | 2.304 326 | https://www.genecards .org/cgi-bin/carddisp.pl?gene= PIBF1 |
| BRD4 | Bromodomain Containing 4 | Protein Coding | 46 | GC19M 015236 | 2.299 45 | https://www.genecards .org/cgi-bin/carddisp.pl?gene= BRD4 |
| PMEL | Premelanosome Protein | Protein Coding | 39 | GC12M 055954 | 2.299 239 | https://www.genecards .org/cgi-bin/carddisp.pl?gene= PMEL |
| RAF1 | Raf-1 Proto-Oncogene, Serine/Threonine Kinase | Protein Coding | 52 | GC03M 012583 | 2.297 447 | https://www.genecards .org/cgi-bin/carddisp.pl?gene= RAF1 |
| DHCR7 | 7-Dehydrocholesterol Reductase | Protein Coding | 45 | GC11M 071428 | 2.296 626 | https://www.genecards .org/cgi-bin/carddisp.pl?gene= DHCR7 |
| NCAM1 | Neural Cell Adhesion Molecule 1 | Protein Coding | 45 | GC11P 112961 | 2.296 626 | https://www.genecards .org/cgi-bin/carddisp.pl?gene= NCAM1 |
| CDH13 | Cadherin 13 | Protein Coding | 43 | GC16P 082626 | 2.296 626 | https://www.genecards .org/cgi-bin/carddisp.pl?gene= CDH13 |
| MIR125B 1 | MicroRNA 125b-1 | RNA Gene | 21 | GC11M 122100 | 2.296 583 | https://www.genecards .org/cgi-bin/carddisp.pl?gene= MIR125B1 |
| PLA2G3 | Phospholipase A2 Group III | Protein Coding | 37 | GC22M 057204 | 2.296 221 | https://www.genecards .org/cgi-bin/carddisp.pl?gene= PLA2G3 |
| P2RX4 | Purinergic Receptor P2X 4 | Protein Coding | 44 | GC12P 126222 | 2.293 557 | https://www.genecards .org/cgi-bin/carddisp.pl?gene= P2RX4 |
| NTF3 | Neurotrophin 3 | Protein Coding | 41 | GC12P 021021 | 2.293 143 | https://www.genecards .org/cgi-bin/carddisp.pl?gene= NTF3 |
| RAD23A | RAD23 Homolog A, Nucleotide Excision Repair Protein | Protein Coding | 41 | GC19P 014288 | 2.291 109 | https://www.genecards .org/cgi-bin/carddisp.pl?gene= RAD23A |
| IHH | Indian Hedgehog Signaling Molecule | Protein Coding | 45 | GC02M 219054 | 2.290 858 | https://www.genecards .org/cgi-bin/carddisp.pl?gene=I HH |
| SP1 | Sp1 Transcription Factor | Protein Coding | 44 | GC12P 053380 | 2.289 666 | https://www.genecards .org/cgi- |

| | | | | | | |
|---|---|---|---|---|---|---|
| | | | | | | bin/carddisp.pl?gene=SP1 |
| MIR671 | MicroRNA 671 | RNA Gene | 18 | GC07P151238 | 2.284522 | https://www.genecards.org/cgi-bin/carddisp.pl?gene=MIR671 |
| GABPA | GA Binding Protein Transcription Factor Subunit Alpha | Protein Coding | 40 | GC21P025734 | 2.281812 | https://www.genecards.org/cgi-bin/carddisp.pl?gene=GABPA |
| AARS1 | Alanyl-TRNA Synthetase 1 | Protein Coding | 36 | GC16M070726 | 2.280768 | https://www.genecards.org/cgi-bin/carddisp.pl?gene=AARS1 |
| RBM5 | RNA Binding Motif Protein 5 | Protein Coding | 38 | GC03P050340 | 2.280638 | https://www.genecards.org/cgi-bin/carddisp.pl?gene=RBM5 |
| ERBB2 | Erb-B2 Receptor Tyrosine Kinase 2 | Protein Coding | 52 | GC17P039687 | 2.276579 | https://www.genecards.org/cgi-bin/carddisp.pl?gene=ERBB2 |
| CCR9 | C-C Motif Chemokine Receptor 9 | Protein Coding | 39 | GC03P046762 | 2.276031 | https://www.genecards.org/cgi-bin/carddisp.pl?gene=CCR9 |
| NR1H2 | Nuclear Receptor Subfamily 1 Group H Member 2 | Protein Coding | 45 | GC19P050329 | 2.275399 | https://www.genecards.org/cgi-bin/carddisp.pl?gene=NR1H2 |
| FTO | FTO Alpha-Ketoglutarate Dependent Dioxygenase | Protein Coding | 44 | GC16P053832 | 2.27202 | https://www.genecards.org/cgi-bin/carddisp.pl?gene=FTO |
| HSP90AA1 | Heat Shock Protein 90 Alpha Family Class A Member 1 | Protein Coding | 46 | GC14M102080 | 2.271402 | https://www.genecards.org/cgi-bin/carddisp.pl?gene=HSP90AA1 |
| FCRLA | Fc Receptor Like A | Protein Coding | 36 | GC01P161712 | 2.270361 | https://www.genecards.org/cgi-bin/carddisp.pl?gene=FCRLA |
| HLA-DOB | Major Histocompatibility Complex, Class II, DO Beta | Protein Coding | 39 | GC06M066021 | 2.267646 | https://www.genecards.org/cgi-bin/carddisp.pl?gene=HLA-DOB |
| FLT3 | Fms Related Receptor Tyrosine Kinase 3 | Protein Coding | 50 | GC13M028003 | 2.267602 | https://www.genecards.org/cgi-bin/carddisp.pl?gene=FLT3 |
| ORM1 | Orosomucoid 1 | Protein Coding | 40 | GC09P114323 | 2.265629 | https://www.genecards.org/cgi-bin/carddisp.pl?gene=ORM1 |

| | | | | | | |
|---|---|---|---|---|---|---|
| CD24 | CD24 Molecule | Protein Coding | 34 | GC06M 106969 | 2.262 617 | https://www.genecards.org/cgi-bin/carddisp.pl?gene=CD24 |
| MIR23A | MicroRNA 23a | RNA Gene | 21 | GC19M 014485 | 2.262 336 | https://www.genecards.org/cgi-bin/carddisp.pl?gene=MIR23A |
| HSD11B1 | Hydroxysteroid 11-Beta Dehydrogenase 1 | Protein Coding | 49 | GC01P 209686 | 2.259 933 | https://www.genecards.org/cgi-bin/carddisp.pl?gene=HSD11B1 |
| LOX | Lysyl Oxidase | Protein Coding | 45 | GC05M 122063 | 2.259 019 | https://www.genecards.org/cgi-bin/carddisp.pl?gene=LOX |
| XRCC4 | X-Ray Repair Cross Complementing 4 | Protein Coding | 42 | GC05P 083077 | 2.258 352 | https://www.genecards.org/cgi-bin/carddisp.pl?gene=XRCC4 |
| C9orf72 | C9orf72-SMCR8 Complex Subunit | Protein Coding | 40 | GC09M 028335 | 2.254 973 | https://www.genecards.org/cgi-bin/carddisp.pl?gene=C9orf72 |
| CCL16 | C-C Motif Chemokine Ligand 16 | Protein Coding | 36 | GC17M 035976 | 2.254 636 | https://www.genecards.org/cgi-bin/carddisp.pl?gene=CCL16 |
| CLEC4E | C-Type Lectin Domain Family 4 Member E | Protein Coding | 36 | GC12M 008535 | 2.252 613 | https://www.genecards.org/cgi-bin/carddisp.pl?gene=CLEC4E |
| HSPB8 | Heat Shock Protein Family B (Small) Member 8 | Protein Coding | 43 | GC12P 119173 | 2.251 756 | https://www.genecards.org/cgi-bin/carddisp.pl?gene=HSPB8 |
| STATH | Statherin | Protein Coding | 32 | GC04P 069995 | 2.251 756 | https://www.genecards.org/cgi-bin/carddisp.pl?gene=STATH |
| SDHB | Succinate Dehydrogenase Complex Iron Sulfur Subunit B | Protein Coding | 46 | GC01M 018063 | 2.249 802 | https://www.genecards.org/cgi-bin/carddisp.pl?gene=SDHB |
| FURIN | Furin, Paired Basic Amino Acid Cleaving Enzyme | Protein Coding | 44 | GC15P 090868 | 2.248 796 | https://www.genecards.org/cgi-bin/carddisp.pl?gene=FURIN |
| XRCC6 | X-Ray Repair Cross Complementing 6 | Protein Coding | 43 | GC22P 041622 | 2.248 682 | https://www.genecards.org/cgi-bin/carddisp.pl?gene=XRCC6 |
| CD200 | CD200 Molecule | Protein Coding | 39 | GC03P 112332 | 2.247 104 | https://www.genecards.org/cgi- |

| | | | | | | |
|---|---|---|---|---|---|---|
| | | | | | | bin/carddisp.pl?gene=CD200 |
| CRYGD | Crystallin Gamma D | Protein Coding | 40 | GC02M208121 | 2.243869 | https://www.genecards.org/cgi-bin/carddisp.pl?gene=CRYGD |
| SMAD7 | SMAD Family Member 7 | Protein Coding | 42 | GC18M048919 | 2.243227 | https://www.genecards.org/cgi-bin/carddisp.pl?gene=SMAD7 |
| KCNQ1 | Potassium Voltage-Gated Channel Subfamily Q Member 1 | Protein Coding | 48 | GC11P002444 | 2.241961 | https://www.genecards.org/cgi-bin/carddisp.pl?gene=KCNQ1 |
| MSMB | Microseminoprotein Beta | Protein Coding | 40 | GC10M046033 | 2.241961 | https://www.genecards.org/cgi-bin/carddisp.pl?gene=MSMB |
| F2R | Coagulation Factor II Thrombin Receptor | Protein Coding | 45 | GC05P076716 | 2.241956 | https://www.genecards.org/cgi-bin/carddisp.pl?gene=F2R |
| GCG | Glucagon | Protein Coding | 40 | GC02M162142 | 2.241231 | https://www.genecards.org/cgi-bin/carddisp.pl?gene=GCG |
| ID1 | Inhibitor Of DNA Binding 1, HLH Protein | Protein Coding | 40 | GC20P031605 | 2.23832 | https://www.genecards.org/cgi-bin/carddisp.pl?gene=ID1 |
| XCR1 | X-C Motif Chemokine Receptor 1 | Protein Coding | 36 | GC03M046016 | 2.236719 | https://www.genecards.org/cgi-bin/carddisp.pl?gene=XCR1 |
| EFNB1 | Ephrin B1 | Protein Coding | 44 | GC0XP068828 | 2.236017 | https://www.genecards.org/cgi-bin/carddisp.pl?gene=EFNB1 |
| EPHB4 | EPH Receptor B4 | Protein Coding | 50 | GC07M101954 | 2.235579 | https://www.genecards.org/cgi-bin/carddisp.pl?gene=EPHB4 |
| SPHK2 | Sphingosine Kinase 2 | Protein Coding | 42 | GC19P048619 | 2.233071 | https://www.genecards.org/cgi-bin/carddisp.pl?gene=SPHK2 |
| RPS19 | Ribosomal Protein S19 | Protein Coding | 47 | GC19P066764 | 2.23246 | https://www.genecards.org/cgi-bin/carddisp.pl?gene=RPS19 |
| TMPRSS6 | Transmembrane Serine Protease 6 | Protein Coding | 44 | GC22M057362 | 2.231264 | https://www.genecards.org/cgi-bin/carddisp.pl?gene=TMPRSS6 |

| | | | | | | |
|---|---|---|---|---|---|---|
| PTN | Pleiotrophin | Protein Coding | 41 | GC07M 137227 | 2.230 502 | https://www.genecards .org/cgi-bin/carddisp.pl?gene= PTN |
| IL17C | Interleukin 17C | Protein Coding | 36 | GC16P 088638 | 2.229 349 | https://www.genecards .org/cgi-bin/carddisp.pl?gene=I L17C |
| AMH | Anti-Mullerian Hormone | Protein Coding | 43 | GC19P 002938 | 2.229 279 | https://www.genecards .org/cgi-bin/carddisp.pl?gene= AMH |
| PNOC | Prepronociceptin | Protein Coding | 37 | GC08P 028316 | 2.228 83 | https://www.genecards .org/cgi-bin/carddisp.pl?gene= PNOC |
| HEXB | Hexosaminidase Subunit Beta | Protein Coding | 47 | GC05P 074640 | 2.227 483 | https://www.genecards .org/cgi-bin/carddisp.pl?gene= HEXB |
| NOTCH2 | Notch Receptor 2 | Protein Coding | 48 | GC01M 119911 | 2.224 988 | https://www.genecards .org/cgi-bin/carddisp.pl?gene= NOTCH2 |
| PIK3R2 | Phosphoinositide-3-Kinase Regulatory Subunit 2 | Protein Coding | 48 | GC19P 018153 | 2.224 885 | https://www.genecards .org/cgi-bin/carddisp.pl?gene= PIK3R2 |
| GNB3 | G Protein Subunit Beta 3 | Protein Coding | 45 | GC12P 006839 | 2.224 885 | https://www.genecards .org/cgi-bin/carddisp.pl?gene= GNB3 |
| SOCS2 | Suppressor Of Cytokine Signaling 2 | Protein Coding | 42 | GC12P 093569 | 2.224 885 | https://www.genecards .org/cgi-bin/carddisp.pl?gene= SOCS2 |
| APOA4 | Apolipoprotein A4 | Protein Coding | 40 | GC11M 116820 | 2.224 885 | https://www.genecards .org/cgi-bin/carddisp.pl?gene= APOA4 |
| FGF21 | Fibroblast Growth Factor 21 | Protein Coding | 37 | GC19P 067053 | 2.224 885 | https://www.genecards .org/cgi-bin/carddisp.pl?gene= FGF21 |
| MST1R | Macrophage Stimulating 1 Receptor | Protein Coding | 47 | GC03M 051216 | 2.224 122 | https://www.genecards .org/cgi-bin/carddisp.pl?gene= MST1R |
| EPHA1 | EPH Receptor A1 | Protein Coding | 45 | GC07M 143390 | 2.224 122 | https://www.genecards .org/cgi-bin/carddisp.pl?gene= EPHA1 |
| THY1 | Thy-1 Cell Surface Antigen | Protein Coding | 41 | GC11M 119417 | 2.220 407 | https://www.genecards .org/cgi- |

| | | | | | | |
|---|---|---|---|---|---|---|
| | | | | | | bin/carddisp.pl?gene=THY1 |
| HSPA1B | Heat Shock Protein Family A (Hsp70) Member 1B | Protein Coding | 39 | GC06P083712 | 2.2197 | https://www.genecards.org/cgi-bin/carddisp.pl?gene=HSPA1B |
| NR2F2 | Nuclear Receptor Subfamily 2 Group F Member 2 | Protein Coding | 48 | GC15P096325 | 2.218796 | https://www.genecards.org/cgi-bin/carddisp.pl?gene=NR2F2 |
| SMPD1 | Sphingomyelin Phosphodiesterase 1 | Protein Coding | 47 | GC11P006390 | 2.215846 | https://www.genecards.org/cgi-bin/carddisp.pl?gene=SMPD1 |
| CBL | Cbl Proto-Oncogene | Protein Coding | 49 | GC11P119206 | 2.212377 | https://www.genecards.org/cgi-bin/carddisp.pl?gene=CBL |
| GPBAR1 | G Protein-Coupled Bile Acid Receptor 1 | Protein Coding | 39 | GC02P218259 | 2.208249 | https://www.genecards.org/cgi-bin/carddisp.pl?gene=GPBAR1 |
| CCND1 | Cyclin D1 | Protein Coding | 49 | GC11P069641 | 2.206554 | https://www.genecards.org/cgi-bin/carddisp.pl?gene=CCND1 |
| FECH | Ferrochelatase | Protein Coding | 45 | GC18M057544 | 2.202175 | https://www.genecards.org/cgi-bin/carddisp.pl?gene=FECH |
| CRYAA | Crystallin Alpha A | Protein Coding | 44 | GC21P043169 | 2.202175 | https://www.genecards.org/cgi-bin/carddisp.pl?gene=CRYAA |
| PON2 | Paraoxonase 2 | Protein Coding | 43 | GC07M095404 | 2.202175 | https://www.genecards.org/cgi-bin/carddisp.pl?gene=PON2 |
| CPVL | Carboxypeptidase Vitellogenic Like | Protein Coding | 40 | GC07M028995 | 2.202175 | https://www.genecards.org/cgi-bin/carddisp.pl?gene=CPVL |
| SERPINB2 | Serpin Family B Member 2 | Protein Coding | 42 | GC18P063871 | 2.199308 | https://www.genecards.org/cgi-bin/carddisp.pl?gene=SERPINB2 |
| NQO1 | NAD(P)H Quinone Dehydrogenase 1 | Protein Coding | 46 | GC16M069706 | 2.196864 | https://www.genecards.org/cgi-bin/carddisp.pl?gene=NQO1 |
| CA3 | Carbonic Anhydrase 3 | Protein Coding | 41 | GC08P085373 | 2.194562 | https://www.genecards.org/cgi-bin/carddisp.pl?gene=CA3 |

| | | | | | | |
|---|---|---|---|---|---|---|
| LCAT | Lecithin-Cholesterol Acyltransferase | Protein Coding | 46 | GC16M 067939 | 2.191 862 | https://www.genecards .org/cgi-bin/carddisp.pl?gene= LCAT |
| TNNI3 | Troponin I3, Cardiac Type | Protein Coding | 47 | GC19M 055151 | 2.191 213 | https://www.genecards .org/cgi-bin/carddisp.pl?gene= TNNI3 |
| CDH6 | Cadherin 6 | Protein Coding | 41 | GC05P 031193 | 2.190 388 | https://www.genecards .org/cgi-bin/carddisp.pl?gene= CDH6 |
| SENP1 | SUMO Specific Peptidase 1 | Protein Coding | 42 | GC12M 048042 | 2.190 304 | https://www.genecards .org/cgi-bin/carddisp.pl?gene= SENP1 |
| MLN | Motilin | Protein Coding | 34 | GC06M 033794 | 2.186 746 | https://www.genecards .org/cgi-bin/carddisp.pl?gene= MLN |
| PLA2G6 | Phospholipase A2 Group VI | Protein Coding | 46 | GC22M 058815 | 2.186 3 | https://www.genecards .org/cgi-bin/carddisp.pl?gene= PLA2G6 |
| MFAP4 | Microfibril Associated Protein 4 | Protein Coding | 39 | GC17M 019383 | 2.183 26 | https://www.genecards .org/cgi-bin/carddisp.pl?gene= MFAP4 |
| TBX5 | T-Box Transcription Factor 5 | Protein Coding | 45 | GC12M 114353 | 2.182 817 | https://www.genecards .org/cgi-bin/carddisp.pl?gene= TBX5 |
| BSN | Bassoon Presynaptic Cytomatrix Protein | Protein Coding | 36 | GC03P 049554 | 2.174 019 | https://www.genecards .org/cgi-bin/carddisp.pl?gene= BSN |
| PAX5 | Paired Box 5 | Protein Coding | 45 | GC09M 036834 | 2.172 556 | https://www.genecards .org/cgi-bin/carddisp.pl?gene= PAX5 |
| KNG1 | Kininogen 1 | Protein Coding | 45 | GC03P 186717 | 2.170 991 | https://www.genecards .org/cgi-bin/carddisp.pl?gene= KNG1 |
| KIR2DS4 | Killer Cell Immunoglobulin Like Receptor, Two Ig Domains And Short Cytoplasmic Tail 4 | Protein Coding | 32 | GC19P 067385 | 2.170 483 | https://www.genecards .org/cgi-bin/carddisp.pl?gene= KIR2DS4 |
| POU2AF 1 | POU Class 2 Homeobox Associating Factor 1 | Protein Coding | 37 | GC11M 111352 | 2.169 985 | https://www.genecards .org/cgi-bin/carddisp.pl?gene= POU2AF1 |

| | | | | | | |
|---|---|---|---|---|---|---|
| CYP7B1 | Cytochrome P450 Family 7 Subfamily B Member 1 | Protein Coding | 45 | GC08M 064587 | 2.169 137 | https://www.genecards .org/cgi-bin/carddisp.pl?gene= CYP7B1 |
| TIMMDC 1 | Translocase Of Inner Mitochondrial Membrane Domain Containing 1 | Protein Coding | 36 | GC03P 119498 | 2.166 089 | https://www.genecards .org/cgi-bin/carddisp.pl?gene= TIMMDC1 |
| IRS1 | Insulin Receptor Substrate 1 | Protein Coding | 45 | GC02M 226731 | 2.165 745 | https://www.genecards .org/cgi-bin/carddisp.pl?gene=I RS1 |
| ITGA9 | Integrin Subunit Alpha 9 | Protein Coding | 42 | GC03P 037468 | 2.165 147 | https://www.genecards .org/cgi-bin/carddisp.pl?gene=I TGA9 |
| CSNK2B | Casein Kinase 2 Beta | Protein Coding | 46 | GC06P 083698 | 2.163 656 | https://www.genecards .org/cgi-bin/carddisp.pl?gene= CSNK2B |
| LY6G5C | Lymphocyte Antigen 6 Family Member G5C | Protein Coding | 26 | GC06M 031676 | 2.163 656 | https://www.genecards .org/cgi-bin/carddisp.pl?gene= LY6G5C |
| AOC3 | Amine Oxidase Copper Containing 3 | Protein Coding | 44 | GC17P 042851 | 2.160 178 | https://www.genecards .org/cgi-bin/carddisp.pl?gene= AOC3 |
| HSPE1 | Heat Shock Protein Family E (Hsp10) Member 1 | Protein Coding | 41 | GC02P 197501 | 2.159 625 | https://www.genecards .org/cgi-bin/carddisp.pl?gene= HSPE1 |
| SCARB1 | Scavenger Receptor Class B Member 1 | Protein Coding | 45 | GC12M 124776 | 2.158 652 | https://www.genecards .org/cgi-bin/carddisp.pl?gene= SCARB1 |
| SLC35D1 | Solute Carrier Family 35 Member D1 | Protein Coding | 40 | GC01M 066999 | 2.158 569 | https://www.genecards .org/cgi-bin/carddisp.pl?gene= SLC35D1 |
| LIFR | LIF Receptor Subunit Alpha | Protein Coding | 45 | GC05M 038475 | 2.157 37 | https://www.genecards .org/cgi-bin/carddisp.pl?gene= LIFR |
| MC3R | Melanocortin 3 Receptor | Protein Coding | 41 | GC20P 056248 | 2.156 837 | https://www.genecards .org/cgi-bin/carddisp.pl?gene= MC3R |
| TTN | Titin | Protein Coding | 45 | GC02M 178525 | 2.156 324 | https://www.genecards .org/cgi-bin/carddisp.pl?gene= TTN |

| | | | | | | |
|---|---|---|---|---|---|---|
| MIR98 | MicroRNA 98 | RNA Gene | 19 | GC0XM053636 | 2.155567 | https://www.genecards.org/cgi-bin/carddisp.pl?gene=MIR98 |
| IL36B | Interleukin 36 Beta | Protein Coding | 33 | GC02M113022 | 2.155266 | https://www.genecards.org/cgi-bin/carddisp.pl?gene=IL36B |
| SERPINA6 | Serpin Family A Member 6 | Protein Coding | 44 | GC14M101180 | 2.1537 | https://www.genecards.org/cgi-bin/carddisp.pl?gene=SERPINA6 |
| FLNA | Filamin A | Protein Coding | 46 | GC0XM154348 | 2.15173 | https://www.genecards.org/cgi-bin/carddisp.pl?gene=FLNA |
| MIR9-1 | MicroRNA 9-1 | RNA Gene | 21 | GC01M156420 | 2.150877 | https://www.genecards.org/cgi-bin/carddisp.pl?gene=MIR9-1 |
| IL2RG | Interleukin 2 Receptor Subunit Gamma | Protein Coding | 45 | GC0XM071108 | 2.150013 | https://www.genecards.org/cgi-bin/carddisp.pl?gene=IL2RG |
| RNASE2 | Ribonuclease A Family Member 2 | Protein Coding | 39 | GC14P032471 | 2.146551 | https://www.genecards.org/cgi-bin/carddisp.pl?gene=RNASE2 |
| PLD1 | Phospholipase D1 | Protein Coding | 47 | GC03M171600 | 2.145625 | https://www.genecards.org/cgi-bin/carddisp.pl?gene=PLD1 |
| SNRPD3 | Small Nuclear Ribonucleoprotein D3 Polypeptide | Protein Coding | 35 | GC22P024555 | 2.145243 | https://www.genecards.org/cgi-bin/carddisp.pl?gene=SNRPD3 |
| GP6 | Glycoprotein VI Platelet | Protein Coding | 44 | GC19M055013 | 2.143559 | https://www.genecards.org/cgi-bin/carddisp.pl?gene=GP6 |
| GAB2 | GRB2 Associated Binding Protein 2 | Protein Coding | 42 | GC11M078215 | 2.143083 | https://www.genecards.org/cgi-bin/carddisp.pl?gene=GAB2 |
| ITGB5 | Integrin Subunit Beta 5 | Protein Coding | 45 | GC03M124761 | 2.142881 | https://www.genecards.org/cgi-bin/carddisp.pl?gene=ITGB5 |
| PLA2G1B | Phospholipase A2 Group IB | Protein Coding | 43 | GC12M120322 | 2.142442 | https://www.genecards.org/cgi-bin/carddisp.pl?gene=PLA2G1B |
| IRGM | Immunity Related GTPase M | Protein Coding | 35 | GC05P150846 | 2.142442 | https://www.genecards.org/cgi- |

| | | | | | | |
|---|---|---|---|---|---|---|
| | | | | | | bin/carddisp.pl?gene=IRGM |
| SFTPA1 | Surfactant Protein A1 | Protein Coding | 42 | GC10P093438 | 2.142258 | https://www.genecards.org/cgi-bin/carddisp.pl?gene=SFTPA1 |
| SFTPC | Surfactant Protein C | Protein Coding | 41 | GC08P022156 | 2.142258 | https://www.genecards.org/cgi-bin/carddisp.pl?gene=SFTPC |
| CTF1 | Cardiotrophin 1 | Protein Coding | 37 | GC16P041104 | 2.137983 | https://www.genecards.org/cgi-bin/carddisp.pl?gene=CTF1 |
| TNFRSF10D | TNF Receptor Superfamily Member 10d | Protein Coding | 40 | GC08M023135 | 2.133998 | https://www.genecards.org/cgi-bin/carddisp.pl?gene=TNFRSF10D |
| ASIC1 | Acid Sensing Ion Channel Subunit 1 | Protein Coding | 42 | GC12P050057 | 2.133133 | https://www.genecards.org/cgi-bin/carddisp.pl?gene=ASIC1 |
| CAPN2 | Calpain 2 | Protein Coding | 45 | GC01P223701 | 2.13271 | https://www.genecards.org/cgi-bin/carddisp.pl?gene=CAPN2 |
| HOXD9 | Homeobox D9 | Protein Coding | 40 | GC02P176122 | 2.128438 | https://www.genecards.org/cgi-bin/carddisp.pl?gene=HOXD9 |
| MKNK1 | MAPK Interacting Serine/Threonine Kinase 1 | Protein Coding | 45 | GC01M046557 | 2.127274 | https://www.genecards.org/cgi-bin/carddisp.pl?gene=MKNK1 |
| TP63 | Tumor Protein P63 | Protein Coding | 46 | GC03P189598 | 2.124065 | https://www.genecards.org/cgi-bin/carddisp.pl?gene=TP63 |
| PSMA6 | Proteasome 20S Subunit Alpha 6 | Protein Coding | 44 | GC14P035278 | 2.119048 | https://www.genecards.org/cgi-bin/carddisp.pl?gene=PSMA6 |
| EPRS1 | Glutamyl-Prolyl-TRNA Synthetase 1 | Protein Coding | 35 | GC01M219969 | 2.118319 | https://www.genecards.org/cgi-bin/carddisp.pl?gene=EPRS1 |
| PRKN | Parkin RBR E3 Ubiquitin Protein Ligase | Protein Coding | 37 | GC06M161348 | 2.1134 | https://www.genecards.org/cgi-bin/carddisp.pl?gene=PRKN |
| PRKAA1 | Protein Kinase AMP-Activated Catalytic Subunit Alpha 1 | Protein Coding | 45 | GC05M040759 | 2.113122 | https://www.genecards.org/cgi-bin/carddisp.pl?gene=PRKAA1 |

| | | | | | | |
|---|---|---|---|---|---|---|
| REL-DT | REL Divergent Transcript | RNA Gene | 13 | GC02M 060822 | 2.112 596 | https://www.genecards .org/cgi-bin/carddisp.pl?gene= REL-DT |
| RPL21P3 3 | Ribosomal Protein L21 Pseudogene 33 | Pseudo gene | 8 | GC02P 060852 | 2.112 596 | https://www.genecards .org/cgi-bin/carddisp.pl?gene= RPL21P33 |
| HEXD | Hexosaminidase D | Protein Coding | 25 | GC17P 082419 | 2.111 394 | https://www.genecards .org/cgi-bin/carddisp.pl?gene= HEXD |
| PLEK | Pleckstrin | Protein Coding | 39 | GC02P 068365 | 2.109 304 | https://www.genecards .org/cgi-bin/carddisp.pl?gene= PLEK |
| PSME3 | Proteasome Activator Subunit 3 | Protein Coding | 40 | GC17P 042824 | 2.107 673 | https://www.genecards .org/cgi-bin/carddisp.pl?gene= PSME3 |
| HNRNPD L | Heterogeneous Nuclear Ribonucleoprotein D Like | Protein Coding | 40 | GC04M 082422 | 2.105 206 | https://www.genecards .org/cgi-bin/carddisp.pl?gene= HNRNPDL |
| SEMA4A | Semaphorin 4A | Protein Coding | 43 | GC01P 156147 | 2.105 062 | https://www.genecards .org/cgi-bin/carddisp.pl?gene= SEMA4A |
| PROK2 | Prokineticin 2 | Protein Coding | 42 | GC03M 071771 | 2.104 84 | https://www.genecards .org/cgi-bin/carddisp.pl?gene= PROK2 |
| F8 | Coagulation Factor VIII | Protein Coding | 45 | GC0X M1548 35 | 2.101 871 | https://www.genecards .org/cgi-bin/carddisp.pl?gene= F8 |
| SETD2 | SET Domain Containing 2, Histone Lysine Methyltransferase | Protein Coding | 45 | GC03M 047033 | 2.099 041 | https://www.genecards .org/cgi-bin/carddisp.pl?gene= SETD2 |
| AMBP | Alpha-1-Microglobulin/Bikunin Precursor | Protein Coding | 42 | GC09M 114060 | 2.097 699 | https://www.genecards .org/cgi-bin/carddisp.pl?gene= AMBP |
| MIR15A | MicroRNA 15a | RNA Gene | 17 | GC13M 050049 | 2.094 632 | https://www.genecards .org/cgi-bin/carddisp.pl?gene= MIR15A |
| KIR2DS3 | Killer Cell Immunoglobulin Like Receptor, Two Ig Domains And Short Cytoplasmic Tail 3 | Protein Coding | 24 | GC19M E00037 | 2.093 691 | https://www.genecards .org/cgi-bin/carddisp.pl?gene= KIR2DS3 |

| | | | | | | |
|---|---|---|---|---|---|---|
| KIR3DS1 | Killer Cell Immunoglobulin Like Receptor, Three Ig Domains And Short Cytoplasmic Tail 1 | Protein Coding | 24 | GC19M R00058 | 2.093 691 | https://www.genecards .org/cgi-bin/carddisp.pl?gene= KIR3DS1 |
| SEC61A1 | SEC61 Translocon Subunit Alpha 1 | Protein Coding | 41 | GC03P 128051 | 2.091 11 | https://www.genecards .org/cgi-bin/carddisp.pl?gene= SEC61A1 |
| HLA-DRB6 | Major Histocompatibility Complex, Class II, DR Beta 6 (Pseudogene) | Pseudo gene | 16 | GC06M 066005 | 2.090 073 | https://www.genecards .org/cgi-bin/carddisp.pl?gene= HLA-DRB6 |
| HLA-DQB1-AS1 | HLA-DQB1 Antisense RNA 1 | RNA Gene | 13 | GC06P 032659 | 2.090 073 | https://www.genecards .org/cgi-bin/carddisp.pl?gene= HLA-DQB1-AS1 |
| FBL | Fibrillarin | Protein Coding | 41 | GC19M 039834 | 2.081 807 | https://www.genecards .org/cgi-bin/carddisp.pl?gene= FBL |
| ILF3 | Interleukin Enhancer Binding Factor 3 | Protein Coding | 38 | GC19P 010655 | 2.080 433 | https://www.genecards .org/cgi-bin/carddisp.pl?gene=I LF3 |
| HSPB1 | Heat Shock Protein Family B (Small) Member 1 | Protein Coding | 49 | GC07P 076302 | 2.078 754 | https://www.genecards .org/cgi-bin/carddisp.pl?gene= HSPB1 |
| ACKR1 | Atypical Chemokine Receptor 1 (Duffy Blood Group) | Protein Coding | 36 | GC01P 159203 | 2.074 645 | https://www.genecards .org/cgi-bin/carddisp.pl?gene= ACKR1 |
| IFNAR1 | Interferon Alpha And Beta Receptor Subunit 1 | Protein Coding | 44 | GC21P 033324 | 2.073 772 | https://www.genecards .org/cgi-bin/carddisp.pl?gene=I FNAR1 |
| CD9 | CD9 Molecule | Protein Coding | 41 | GC12P 021026 | 2.073 496 | https://www.genecards .org/cgi-bin/carddisp.pl?gene= CD9 |
| ANGPTL 2 | Angiopoietin Like 2 | Protein Coding | 37 | GC09M 127087 | 2.072 181 | https://www.genecards .org/cgi-bin/carddisp.pl?gene= ANGPTL2 |
| ABCG5 | ATP Binding Cassette Subfamily G Member 5 | Protein Coding | 44 | GC02M 043806 | 2.070 686 | https://www.genecards .org/cgi-bin/carddisp.pl?gene= ABCG5 |
| DSG1 | Desmoglein 1 | Protein Coding | 42 | GC18P 031318 | 2.069 865 | https://www.genecards .org/cgi-bin/carddisp.pl?gene= DSG1 |

| | | | | | | |
|---|---|---|---|---|---|---|
| KRAS | KRAS Proto-Oncogene, GTPase | Protein Coding | 49 | GC12M 025204 | 2.069 338 | https://www.genecards .org/cgi-bin/carddisp.pl?gene= KRAS |
| MRPL28 | Mitochondrial Ribosomal Protein L28 | Protein Coding | 36 | GC16M 000366 | 2.068 802 | https://www.genecards .org/cgi-bin/carddisp.pl?gene= MRPL28 |
| CYP3A5 | Cytochrome P450 Family 3 Subfamily A Member 5 | Protein Coding | 45 | GC07M 099648 | 2.068 704 | https://www.genecards .org/cgi-bin/carddisp.pl?gene= CYP3A5 |
| LAT | Linker For Activation Of T Cells | Protein Coding | 44 | GC16P 040930 | 2.068 704 | https://www.genecards .org/cgi-bin/carddisp.pl?gene= LAT |
| PTPN13 | Protein Tyrosine Phosphatase Non-Receptor Type 13 | Protein Coding | 42 | GC04P 086594 | 2.068 704 | https://www.genecards .org/cgi-bin/carddisp.pl?gene= PTPN13 |
| ACD | ACD Shelterin Complex Subunit And Telomerase Recruitment Factor | Protein Coding | 41 | GC16M 067658 | 2.068 704 | https://www.genecards .org/cgi-bin/carddisp.pl?gene= ACD |
| SERPINA 7 | Serpin Family A Member 7 | Protein Coding | 40 | GC0X M1060 32 | 2.068 704 | https://www.genecards .org/cgi-bin/carddisp.pl?gene= SERPINA7 |
| VIPR2 | Vasoactive Intestinal Peptide Receptor 2 | Protein Coding | 42 | GC07M 159028 | 2.067 124 | https://www.genecards .org/cgi-bin/carddisp.pl?gene= VIPR2 |
| CCN4 | Cellular Communication Network Factor 4 | Protein Coding | 30 | GC08P 133192 | 2.066 181 | https://www.genecards .org/cgi-bin/carddisp.pl?gene= CCN4 |
| ADAMT S13 | ADAM Metallopeptidase With Thrombospondin Type 1 Motif 13 | Protein Coding | 47 | GC09P 133414 | 2.062 452 | https://www.genecards .org/cgi-bin/carddisp.pl?gene= ADAMTS13 |
| DSG3 | Desmoglein 3 | Protein Coding | 40 | GC18P 031447 | 2.055 178 | https://www.genecards .org/cgi-bin/carddisp.pl?gene= DSG3 |
| CD6 | CD6 Molecule | Protein Coding | 41 | GC11P 060971 | 2.053 894 | https://www.genecards .org/cgi-bin/carddisp.pl?gene= CD6 |
| PADI3 | Peptidyl Arginine Deiminase 3 | Protein Coding | 40 | GC01P 017249 | 2.053 202 | https://www.genecards .org/cgi-bin/carddisp.pl?gene= PADI3 |
| MIR155H G | MIR155 Host Gene | RNA Gene | 22 | GC21P 025589 | 2.052 902 | https://www.genecards .org/cgi- |

| | | | | | | |
|---|---|---|---|---|---|---|
| | | | | | | bin/carddisp.pl?gene=MIR155HG |
| DNMT3B | DNA Methyltransferase 3 Beta | Protein Coding | 49 | GC20P 032762 | 2.052 171 | https://www.genecards.org/cgi-bin/carddisp.pl?gene=DNMT3B |
| TFF2 | Trefoil Factor 2 | Protein Coding | 40 | GC21M 042346 | 2.048 805 | https://www.genecards.org/cgi-bin/carddisp.pl?gene=TFF2 |
| PPARGC 1B | PPARG Coactivator 1 Beta | Protein Coding | 34 | GC05P 149730 | 2.048 602 | https://www.genecards.org/cgi-bin/carddisp.pl?gene=PPARGC1B |
| F2RL2 | Coagulation Factor II Thrombin Receptor Like 2 | Protein Coding | 40 | GC05M 076615 | 2.047 254 | https://www.genecards.org/cgi-bin/carddisp.pl?gene=F2RL2 |
| SLC25A2 8 | Solute Carrier Family 25 Member 28 | Protein Coding | 37 | GC10M 099610 | 2.045 891 | https://www.genecards.org/cgi-bin/carddisp.pl?gene=SLC25A28 |
| CXCL6 | C-X-C Motif Chemokine Ligand 6 | Protein Coding | 38 | GC04P 073837 | 2.044 777 | https://www.genecards.org/cgi-bin/carddisp.pl?gene=CXCL6 |
| PGLYRP 1 | Peptidoglycan Recognition Protein 1 | Protein Coding | 40 | GC19M 065951 | 2.043 633 | https://www.genecards.org/cgi-bin/carddisp.pl?gene=PGLYRP1 |
| RUNX3 | RUNX Family Transcription Factor 3 | Protein Coding | 42 | GC01M 024899 | 2.042 32 | https://www.genecards.org/cgi-bin/carddisp.pl?gene=RUNX3 |
| ITCH | Itchy E3 Ubiquitin Protein Ligase | Protein Coding | 44 | GC20P 034363 | 2.042 234 | https://www.genecards.org/cgi-bin/carddisp.pl?gene=ITCH |
| SEMA7A | Semaphorin 7A (John Milton Hagen Blood Group) | Protein Coding | 42 | GC15M 074409 | 2.038 297 | https://www.genecards.org/cgi-bin/carddisp.pl?gene=SEMA7A |
| SIGIRR | Single Ig And TIR Domain Containing | Protein Coding | 39 | GC11M 003183 | 2.038 297 | https://www.genecards.org/cgi-bin/carddisp.pl?gene=SIGIRR |
| BIRC3 | Baculoviral IAP Repeat Containing 3 | Protein Coding | 45 | GC11P 102317 | 2.033 995 | https://www.genecards.org/cgi-bin/carddisp.pl?gene=BIRC3 |
| ING4 | Inhibitor Of Growth Family Member 4 | Protein Coding | 36 | GC12M 006650 | 2.033 898 | https://www.genecards.org/cgi-bin/carddisp.pl?gene=ING4 |

| | | | | | | |
|---|---|---|---|---|---|---|
| LDLR | Low Density Lipoprotein Receptor | Protein Coding | 49 | GC19P 011091 | 2.031 577 | https://www.genecards .org/cgi-bin/carddisp.pl?gene= LDLR |
| EPAS1 | Endothelial PAS Domain Protein 1 | Protein Coding | 48 | GC02P 046293 | 2.031 577 | https://www.genecards .org/cgi-bin/carddisp.pl?gene= EPAS1 |
| CD3G | CD3 Gamma Subunit Of T-Cell Receptor Complex | Protein Coding | 45 | GC11P 118344 | 2.031 577 | https://www.genecards .org/cgi-bin/carddisp.pl?gene= CD3G |
| AGFG1 | ArfGAP With FG Repeats 1 | Protein Coding | 39 | GC02P 227473 | 2.031 577 | https://www.genecards .org/cgi-bin/carddisp.pl?gene= AGFG1 |
| MIR650 | MicroRNA 650 | RNA Gene | 13 | GC22P 022822 | 2.031 577 | https://www.genecards .org/cgi-bin/carddisp.pl?gene= MIR650 |
| IGLV@ | Immunoglobulin Lambda Variable Cluster | Gene Cluster | 8 | GC22U 990033 | 2.031 577 | https://www.genecards .org/cgi-bin/carddisp.pl?gene=I GLV%40 |
| FGFR4 | Fibroblast Growth Factor Receptor 4 | Protein Coding | 49 | GC05P 177086 | 2.031 178 | https://www.genecards .org/cgi-bin/carddisp.pl?gene= FGFR4 |
| LZTR1 | Leucine Zipper Like Transcription Regulator 1 | Protein Coding | 42 | GC22P 035893 | 2.030 736 | https://www.genecards .org/cgi-bin/carddisp.pl?gene= LZTR1 |
| TOP3B | DNA Topoisomerase III Beta | Protein Coding | 40 | GC22M 021957 | 2.030 736 | https://www.genecards .org/cgi-bin/carddisp.pl?gene= TOP3B |
| THAP7 | THAP Domain Containing 7 | Protein Coding | 32 | GC22M 020999 | 2.030 736 | https://www.genecards .org/cgi-bin/carddisp.pl?gene= THAP7 |
| CAPZB | Capping Actin Protein Of Muscle Z-Line Subunit Beta | Protein Coding | 40 | GC01M 019339 | 2.029 269 | https://www.genecards .org/cgi-bin/carddisp.pl?gene= CAPZB |
| PTGER1 | Prostaglandin E Receptor 1 | Protein Coding | 40 | GC19M 014515 | 2.028 983 | https://www.genecards .org/cgi-bin/carddisp.pl?gene= PTGER1 |
| MAP3K3 | Mitogen-Activated Protein Kinase Kinase Kinase 3 | Protein Coding | 45 | GC17P 063622 | 2.027 966 | https://www.genecards .org/cgi-bin/carddisp.pl?gene= MAP3K3 |
| SH2D2A | SH2 Domain Containing 2A | Protein Coding | 38 | GC01M 156917 | 2.026 004 | https://www.genecards .org/cgi- |

| | | | | | | |
|---|---|---|---|---|---|---|
| | | | | | | bin/carddisp.pl?gene=SH2D2A |
| MAPKAP1 | MAPK Associated Protein 1 | Protein Coding | 41 | GC09M125437 | 2.017874 | https://www.genecards.org/cgi-bin/carddisp.pl?gene=MAPKAP1 |
| NEDD9 | Neural Precursor Cell Expressed, Developmentally Down-Regulated 9 | Protein Coding | 41 | GC06M011183 | 2.017874 | https://www.genecards.org/cgi-bin/carddisp.pl?gene=NEDD9 |
| CAMLG | Calcium Modulating Ligand | Protein Coding | 40 | GC05P134738 | 2.017698 | https://www.genecards.org/cgi-bin/carddisp.pl?gene=CAMLG |
| CNMD | Chondromodulin | Protein Coding | 29 | GC13M052704 | 2.014921 | https://www.genecards.org/cgi-bin/carddisp.pl?gene=CNMD |
| SOX5 | SRY-Box Transcription Factor 5 | Protein Coding | 44 | GC12M023529 | 2.014562 | https://www.genecards.org/cgi-bin/carddisp.pl?gene=SOX5 |
| TPI1 | Triosephosphate Isomerase 1 | Protein Coding | 45 | GC12P006867 | 2.010789 | https://www.genecards.org/cgi-bin/carddisp.pl?gene=TPI1 |
| BRCA2 | BRCA2 DNA Repair Associated | Protein Coding | 46 | GC13P032315 | 2.010146 | https://www.genecards.org/cgi-bin/carddisp.pl?gene=BRCA2 |
| IGFBP2 | Insulin Like Growth Factor Binding Protein 2 | Protein Coding | 42 | GC02P216632 | 2.007969 | https://www.genecards.org/cgi-bin/carddisp.pl?gene=IGFBP2 |
| MYOM2 | Myomesin 2 | Protein Coding | 37 | GC08P002045 | 2.007447 | https://www.genecards.org/cgi-bin/carddisp.pl?gene=MYOM2 |
| MCAM | Melanoma Cell Adhesion Molecule | Protein Coding | 41 | GC11M119308 | 2.006366 | https://www.genecards.org/cgi-bin/carddisp.pl?gene=MCAM |
| RBP3 | Retinol Binding Protein 3 | Protein Coding | 41 | GC10P047348 | 2.001217 | https://www.genecards.org/cgi-bin/carddisp.pl?gene=RBP3 |
| SNRPD1 | Small Nuclear Ribonucleoprotein D1 Polypeptide | Protein Coding | 37 | GC18P021612 | 1.999572 | https://www.genecards.org/cgi-bin/carddisp.pl?gene=SNRPD1 |
| ACTN1 | Actinin Alpha 1 | Protein Coding | 47 | GC14M068874 | 1.999129 | https://www.genecards.org/cgi-bin/carddisp.pl?gene=ACTN1 |

| | | | | | | |
|---|---|---|---|---|---|---|
| NKX2-3 | NK2 Homeobox 3 | Protein Coding | 38 | GC10P 099532 | 1.996 514 | https://www.genecards .org/cgi-bin/carddisp.pl?gene= NKX2-3 |
| HRAS | HRas Proto-Oncogene, GTPase | Protein Coding | 50 | GC11M 003189 | 1.995 996 | https://www.genecards .org/cgi-bin/carddisp.pl?gene= HRAS |
| MIR29A | MicroRNA 29a | RNA Gene | 22 | GC07M 130876 | 1.995 667 | https://www.genecards .org/cgi-bin/carddisp.pl?gene= MIR29A |
| GRB2 | Growth Factor Receptor Bound Protein 2 | Protein Coding | 45 | GC17M 075318 | 1.993 8 | https://www.genecards .org/cgi-bin/carddisp.pl?gene= GRB2 |
| THBS2 | Thrombospondin 2 | Protein Coding | 44 | GC06M 169215 | 1.993 781 | https://www.genecards .org/cgi-bin/carddisp.pl?gene= THBS2 |
| MIR590 | MicroRNA 590 | RNA Gene | 20 | GC07P 074191 | 1.993 555 | https://www.genecards .org/cgi-bin/carddisp.pl?gene= MIR590 |
| CHD4 | Chromodomain Helicase DNA Binding Protein 4 | Protein Coding | 43 | GC12M 006570 | 1.993 327 | https://www.genecards .org/cgi-bin/carddisp.pl?gene= CHD4 |
| MIR106A | MicroRNA 106a | RNA Gene | 20 | GC0X M1343 13 | 1.991 832 | https://www.genecards .org/cgi-bin/carddisp.pl?gene= MIR106A |
| MIR192 | MicroRNA 192 | RNA Gene | 22 | GC11M 064891 | 1.989 437 | https://www.genecards .org/cgi-bin/carddisp.pl?gene= MIR192 |
| PIK3CA | Phosphatidylinositol-4,5-Bisphosphate 3-Kinase Catalytic Subunit Alpha | Protein Coding | 50 | GC03P 179148 | 1.987 665 | https://www.genecards .org/cgi-bin/carddisp.pl?gene= PIK3CA |
| HLA-DQB3 | Major Histocompatibility Complex, Class II, DQ Beta 3 | Protein Coding | 12 | GC06M 066018 | 1.986 088 | https://www.genecards .org/cgi-bin/carddisp.pl?gene= HLA-DQB3 |
| MTCO3P 1 | MT-CO3 Pseudogene 1 | Pseudo gene | 8 | GC06M 032706 | 1.986 088 | https://www.genecards .org/cgi-bin/carddisp.pl?gene= MTCO3P1 |
| NRXN1 | Neurexin 1 | Protein Coding | 47 | GC02M 049918 | 1.984 516 | https://www.genecards .org/cgi-bin/carddisp.pl?gene= NRXN1 |
| VCL | Vinculin | Protein Coding | 45 | GC10P 073995 | 1.984 516 | https://www.genecards .org/cgi- |

| | | | | | | |
|---|---|---|---|---|---|---|
| | | | | | | bin/carddisp.pl?gene=VCL |
| HEXA | Hexosaminidase Subunit Alpha | Protein Coding | 45 | GC15M072340 | 1.984349 | https://www.genecards.org/cgi-bin/carddisp.pl?gene=HEXA |
| PCSK9 | Proprotein Convertase Subtilisin/Kexin Type 9 | Protein Coding | 49 | GC01P055039 | 1.983996 | https://www.genecards.org/cgi-bin/carddisp.pl?gene=PCSK9 |
| ETFA | Electron Transfer Flavoprotein Subunit Alpha | Protein Coding | 44 | GC15M090338 | 1.983996 | https://www.genecards.org/cgi-bin/carddisp.pl?gene=ETFA |
| HPSE | Heparanase | Protein Coding | 43 | GC04M083292 | 1.983996 | https://www.genecards.org/cgi-bin/carddisp.pl?gene=HPSE |
| COPS5 | COP9 Signalosome Subunit 5 | Protein Coding | 41 | GC08M067043 | 1.983996 | https://www.genecards.org/cgi-bin/carddisp.pl?gene=COPS5 |
| QKI | QKI, KH Domain Containing RNA Binding | Protein Coding | 41 | GC06P163414 | 1.983996 | https://www.genecards.org/cgi-bin/carddisp.pl?gene=QKI |
| MDGA2 | MAM Domain Containing Glycosylphosphatidylinositol Anchor 2 | Protein Coding | 37 | GC14M046839 | 1.983996 | https://www.genecards.org/cgi-bin/carddisp.pl?gene=MDGA2 |
| ERV3-1 | Endogenous Retrovirus Group 3 Member 1, Envelope | Protein Coding | 31 | GC07M065766 | 1.983996 | https://www.genecards.org/cgi-bin/carddisp.pl?gene=ERV3-1 |
| MIR449A | MicroRNA 449a | RNA Gene | 21 | GC05M055171 | 1.983996 | https://www.genecards.org/cgi-bin/carddisp.pl?gene=MIR449A |
| MUTYH | MutY DNA Glycosylase | Protein Coding | 44 | GC01M045329 | 1.980473 | https://www.genecards.org/cgi-bin/carddisp.pl?gene=MUTYH |
| PIAS3 | Protein Inhibitor Of Activated STAT 3 | Protein Coding | 39 | GC01M145848 | 1.980473 | https://www.genecards.org/cgi-bin/carddisp.pl?gene=PIAS3 |
| SLC2A3 | Solute Carrier Family 2 Member 3 | Protein Coding | 46 | GC12M007919 | 1.979011 | https://www.genecards.org/cgi-bin/carddisp.pl?gene=SLC2A3 |
| TNFSF9 | TNF Superfamily Member 9 | Protein Coding | 35 | GC19P006531 | 1.97866 | https://www.genecards.org/cgi-bin/carddisp.pl?gene=TNFSF9 |

| | | | | | | |
|---|---|---|---|---|---|---|
| MGP | Matrix Gla Protein | Protein Coding | 41 | GC12M 021179 | 1.978 456 | https://www.genecards.org/cgi-bin/carddisp.pl?gene=MGP |
| ZMIZ1 | Zinc Finger MIZ-Type Containing 1 | Protein Coding | 41 | GC10P 079068 | 1.975 965 | https://www.genecards.org/cgi-bin/carddisp.pl?gene=ZMIZ1 |
| CD72 | CD72 Molecule | Protein Coding | 38 | GC09M 035610 | 1.972 569 | https://www.genecards.org/cgi-bin/carddisp.pl?gene=CD72 |
| CLEC5A | C-Type Lectin Domain Containing 5A | Protein Coding | 34 | GC07M 141927 | 1.970 131 | https://www.genecards.org/cgi-bin/carddisp.pl?gene=CLEC5A |
| SLC29A1 | Solute Carrier Family 29 Member 1 (Augustine Blood Group) | Protein Coding | 46 | GC06P 044219 | 1.968 774 | https://www.genecards.org/cgi-bin/carddisp.pl?gene=SLC29A1 |
| MIR497 | MicroRNA 497 | RNA Gene | 18 | GC17M 007022 | 1.963 809 | https://www.genecards.org/cgi-bin/carddisp.pl?gene=MIR497 |
| ARNTL | Aryl Hydrocarbon Receptor Nuclear Translocator Like | Protein Coding | 40 | GC11P 013276 | 1.963 676 | https://www.genecards.org/cgi-bin/carddisp.pl?gene=ARNTL |
| ARG1 | Arginase 1 | Protein Coding | 47 | GC06P 131473 | 1.963 675 | https://www.genecards.org/cgi-bin/carddisp.pl?gene=ARG1 |
| SIGLEC9 | Sialic Acid Binding Ig Like Lectin 9 | Protein Coding | 36 | GC19P 067192 | 1.962 502 | https://www.genecards.org/cgi-bin/carddisp.pl?gene=SIGLEC9 |
| POLR3B | RNA Polymerase III Subunit B | Protein Coding | 42 | GC12P 106357 | 1.962 202 | https://www.genecards.org/cgi-bin/carddisp.pl?gene=POLR3B |
| RPL36A-HNRNPH2 | RPL36A-HNRNPH2 Readthrough | Protein Coding | 17 | GC0XP 101390 | 1.960 071 | https://www.genecards.org/cgi-bin/carddisp.pl?gene=RPL36A-HNRNPH2 |
| IL11RA | Interleukin 11 Receptor Subunit Alpha | Protein Coding | 44 | GC09P 047112 | 1.958 033 | https://www.genecards.org/cgi-bin/carddisp.pl?gene=IL11RA |
| PPIL2 | Peptidylprolyl Isomerase Like 2 | Protein Coding | 39 | GC22P 038720 | 1.957 664 | https://www.genecards.org/cgi-bin/carddisp.pl?gene=PPIL2 |
| MIR31 | MicroRNA 31 | RNA Gene | 20 | GC09M 021761 | 1.957 645 | https://www.genecards.org/cgi- |

| | | | | | | |
|---|---|---|---|---|---|---|
| | | | | | | bin/carddisp.pl?gene=MIR31 |
| GIMAP5 | GTPase, IMAP Family Member 5 | Protein Coding | 38 | GC07P 150722 | 1.956 36 | https://www.genecards.org/cgi-bin/carddisp.pl?gene=GIMAP5 |
| EFNA5 | Ephrin A5 | Protein Coding | 42 | GC05M 107376 | 1.956 268 | https://www.genecards.org/cgi-bin/carddisp.pl?gene=EFNA5 |
| SOCS6 | Suppressor Of Cytokine Signaling 6 | Protein Coding | 40 | GC18P 070288 | 1.956 268 | https://www.genecards.org/cgi-bin/carddisp.pl?gene=SOCS6 |
| LINC006 38 | Long Intergenic Non-Protein Coding RNA 638 | RNA Gene | 14 | GC14P 109735 | 1.956 268 | https://www.genecards.org/cgi-bin/carddisp.pl?gene=LINC00638 |
| ALDOA | Aldolase, Fructose-Bisphosphate A | Protein Coding | 46 | GC16P 030064 | 1.955 285 | https://www.genecards.org/cgi-bin/carddisp.pl?gene=ALDOA |
| CASP4 | Caspase 4 | Protein Coding | 45 | GC11M 104942 | 1.955 285 | https://www.genecards.org/cgi-bin/carddisp.pl?gene=CASP4 |
| NR1H3 | Nuclear Receptor Subfamily 1 Group H Member 3 | Protein Coding | 45 | GC11P 047248 | 1.955 285 | https://www.genecards.org/cgi-bin/carddisp.pl?gene=NR1H3 |
| MDM4 | MDM4 Regulator Of P53 | Protein Coding | 43 | GC01P 204516 | 1.955 285 | https://www.genecards.org/cgi-bin/carddisp.pl?gene=MDM4 |
| DAXX | Death Domain Associated Protein | Protein Coding | 41 | GC06M 033318 | 1.955 285 | https://www.genecards.org/cgi-bin/carddisp.pl?gene=DAXX |
| C1QTNF 6 | C1q And TNF Related 6 | Protein Coding | 37 | GC22M 037180 | 1.950 598 | https://www.genecards.org/cgi-bin/carddisp.pl?gene=C1QTNF6 |
| JUNB | JunB Proto-Oncogene, AP-1 Transcription Factor Subunit | Protein Coding | 40 | GC19P 012791 | 1.947 224 | https://www.genecards.org/cgi-bin/carddisp.pl?gene=JUNB |
| BID | BH3 Interacting Domain Death Agonist | Protein Coding | 42 | GC22M 017734 | 1.946 494 | https://www.genecards.org/cgi-bin/carddisp.pl?gene=BID |
| CD52 | CD52 Molecule | Protein Coding | 37 | GC01P 026317 | 1.944 578 | https://www.genecards.org/cgi-bin/carddisp.pl?gene=CD52 |

| | | | | | | |
|---|---|---|---|---|---|---|
| NTRK2 | Neurotrophic Receptor Tyrosine Kinase 2 | Protein Coding | 51 | GC09P 084668 | 1.942 75 | https://www.genecards .org/cgi-bin/carddisp.pl?gene= NTRK2 |
| METAP2 | Methionyl Aminopeptidase 2 | Protein Coding | 44 | GC12P 095473 | 1.942 644 | https://www.genecards .org/cgi-bin/carddisp.pl?gene= METAP2 |
| HRH1 | Histamine Receptor H1 | Protein Coding | 44 | GC03P 012473 | 1.937 509 | https://www.genecards .org/cgi-bin/carddisp.pl?gene= HRH1 |
| VASH1 | Vasohibin 1 | Protein Coding | 36 | GC14P 076761 | 1.929 318 | https://www.genecards .org/cgi-bin/carddisp.pl?gene= VASH1 |
| PAH | Phenylalanine Hydroxylase | Protein Coding | 46 | GC12M 102836 | 1.928 018 | https://www.genecards .org/cgi-bin/carddisp.pl?gene= PAH |
| SNRPN | Small Nuclear Ribonucleoprotein Polypeptide N | Protein Coding | 42 | GC15P 024823 | 1.928 018 | https://www.genecards .org/cgi-bin/carddisp.pl?gene= SNRPN |
| PLK1 | Polo Like Kinase 1 | Protein Coding | 48 | GC16P 024283 | 1.927 557 | https://www.genecards .org/cgi-bin/carddisp.pl?gene= PLK1 |
| HDAC3 | Histone Deacetylase 3 | Protein Coding | 47 | GC05M 141583 | 1.927 557 | https://www.genecards .org/cgi-bin/carddisp.pl?gene= HDAC3 |
| EIF2S3 | Eukaryotic Translation Initiation Factor 2 Subunit Gamma | Protein Coding | 44 | GC0XP 024054 | 1.927 557 | https://www.genecards .org/cgi-bin/carddisp.pl?gene= EIF2S3 |
| NTN1 | Netrin 1 | Protein Coding | 44 | GC17P 011249 | 1.927 557 | https://www.genecards .org/cgi-bin/carddisp.pl?gene= NTN1 |
| HIPK2 | Homeodomain Interacting Protein Kinase 2 | Protein Coding | 43 | GC07M 139561 | 1.927 557 | https://www.genecards .org/cgi-bin/carddisp.pl?gene= HIPK2 |
| PSMA4 | Proteasome 20S Subunit Alpha 4 | Protein Coding | 42 | GC15P 078540 | 1.927 557 | https://www.genecards .org/cgi-bin/carddisp.pl?gene= PSMA4 |
| SNAI2 | Snail Family Transcriptional Repressor 2 | Protein Coding | 42 | GC08M 048917 | 1.927 557 | https://www.genecards .org/cgi-bin/carddisp.pl?gene= SNAI2 |
| MYO3A | Myosin IIIA | Protein Coding | 41 | GC10P 025934 | 1.927 557 | https://www.genecards .org/cgi- |

| | | | | | | |
|---|---|---|---|---|---|---|
| | | | | | | bin/carddisp.pl?gene=MYO3A |
| S100A11 | S100 Calcium Binding Protein A11 | Protein Coding | 41 | GC01M152032 | 1.927557 | https://www.genecards.org/cgi-bin/carddisp.pl?gene=S100A11 |
| TNFAIP1 | TNF Alpha Induced Protein 1 | Protein Coding | 41 | GC17P028335 | 1.927557 | https://www.genecards.org/cgi-bin/carddisp.pl?gene=TNFAIP1 |
| AGXT2 | Alanine--Glyoxylate Aminotransferase 2 | Protein Coding | 40 | GC05M034998 | 1.927557 | https://www.genecards.org/cgi-bin/carddisp.pl?gene=AGXT2 |
| CD109 | CD109 Molecule | Protein Coding | 40 | GC06P084244 | 1.927557 | https://www.genecards.org/cgi-bin/carddisp.pl?gene=CD109 |
| EXOC5 | Exocyst Complex Component 5 | Protein Coding | 40 | GC14M057200 | 1.927557 | https://www.genecards.org/cgi-bin/carddisp.pl?gene=EXOC5 |
| IL13RA1 | Interleukin 13 Receptor Subunit Alpha 1 | Protein Coding | 40 | GC0XP118727 | 1.927557 | https://www.genecards.org/cgi-bin/carddisp.pl?gene=IL13RA1 |
| SLC6A11 | Solute Carrier Family 6 Member 11 | Protein Coding | 40 | GC03P012471 | 1.927557 | https://www.genecards.org/cgi-bin/carddisp.pl?gene=SLC6A11 |
| UNC5B | Unc-5 Netrin Receptor B | Protein Coding | 39 | GC10P071212 | 1.927557 | https://www.genecards.org/cgi-bin/carddisp.pl?gene=UNC5B |
| CD99 | CD99 Molecule (Xg Blood Group) | Protein Coding | 38 | GC0XP002691 | 1.927557 | https://www.genecards.org/cgi-bin/carddisp.pl?gene=CD99 |
| EXOC4 | Exocyst Complex Component 4 | Protein Coding | 38 | GC07P133253 | 1.927557 | https://www.genecards.org/cgi-bin/carddisp.pl?gene=EXOC4 |
| GSTM4 | Glutathione S-Transferase Mu 4 | Protein Coding | 38 | GC01P109657 | 1.927557 | https://www.genecards.org/cgi-bin/carddisp.pl?gene=GSTM4 |
| LMO4 | LIM Domain Only 4 | Protein Coding | 38 | GC01P087329 | 1.927557 | https://www.genecards.org/cgi-bin/carddisp.pl?gene=LMO4 |
| NHLH2 | Nescient Helix-Loop-Helix 2 | Protein Coding | 38 | GC01M115836 | 1.927557 | https://www.genecards.org/cgi-bin/carddisp.pl?gene=NHLH2 |

| | | | | | | |
|---|---|---|---|---|---|---|
| NKIRAS 2 | NFKB Inhibitor Interacting Ras Like 2 | Protein Coding | 38 | GC17P 042011 | 1.927 557 | https://www.genecards .org/cgi-bin/carddisp.pl?gene= NKIRAS2 |
| PPP1R9A | Protein Phosphatase 1 Regulatory Subunit 9A | Protein Coding | 38 | GC07P 094907 | 1.927 557 | https://www.genecards .org/cgi-bin/carddisp.pl?gene= PPP1R9A |
| RCHY1 | Ring Finger And CHY Zinc Finger Domain Containing 1 | Protein Coding | 38 | GC04M 075479 | 1.927 557 | https://www.genecards .org/cgi-bin/carddisp.pl?gene= RCHY1 |
| RIT2 | Ras Like Without CAAX 2 | Protein Coding | 38 | GC18M 042743 | 1.927 557 | https://www.genecards .org/cgi-bin/carddisp.pl?gene= RIT2 |
| CEP72 | Centrosomal Protein 72 | Protein Coding | 37 | GC05P 000612 | 1.927 557 | https://www.genecards .org/cgi-bin/carddisp.pl?gene= CEP72 |
| GBP6 | Guanylate Binding Protein Family Member 6 | Protein Coding | 37 | GC01P 089363 | 1.927 557 | https://www.genecards .org/cgi-bin/carddisp.pl?gene= GBP6 |
| IFNK | Interferon Kappa | Protein Coding | 37 | GC09P 027514 | 1.927 557 | https://www.genecards .org/cgi-bin/carddisp.pl?gene=I FNK |
| SPSB1 | SplA/Ryanodine Receptor Domain And SOCS Box Containing 1 | Protein Coding | 37 | GC01P 009292 | 1.927 557 | https://www.genecards .org/cgi-bin/carddisp.pl?gene= SPSB1 |
| TNIP2 | TNFAIP3 Interacting Protein 2 | Protein Coding | 37 | GC04M 002741 | 1.927 557 | https://www.genecards .org/cgi-bin/carddisp.pl?gene= TNIP2 |
| ZNF175 | Zinc Finger Protein 175 | Protein Coding | 37 | GC19P 051571 | 1.927 557 | https://www.genecards .org/cgi-bin/carddisp.pl?gene= ZNF175 |
| CST5 | Cystatin D | Protein Coding | 36 | GC20M 023875 | 1.927 557 | https://www.genecards .org/cgi-bin/carddisp.pl?gene= CST5 |
| ENOX1 | Ecto-NOX Disulfide-Thiol Exchanger 1 | Protein Coding | 36 | GC13M 043213 | 1.927 557 | https://www.genecards .org/cgi-bin/carddisp.pl?gene= ENOX1 |
| N4BP1 | NEDD4 Binding Protein 1 | Protein Coding | 36 | GC16M 048538 | 1.927 557 | https://www.genecards .org/cgi-bin/carddisp.pl?gene= N4BP1 |
| SPA17 | Sperm Autoantigenic Protein 17 | Protein Coding | 36 | GC11P 124673 | 1.927 557 | https://www.genecards .org/cgi- |

| | | | | | | |
|---|---|---|---|---|---|---|
| | | | | | | bin/carddisp.pl?gene=SPA17 |
| WFDC1 | WAP Four-Disulfide Core Domain 1 | Protein Coding | 36 | GC16P084328 | 1.927557 | https://www.genecards.org/cgi-bin/carddisp.pl?gene=WFDC1 |
| CCL24 | C-C Motif Chemokine Ligand 24 | Protein Coding | 35 | GC07M076814 | 1.927557 | https://www.genecards.org/cgi-bin/carddisp.pl?gene=CCL24 |
| LVRN | Laeverin | Protein Coding | 35 | GC05P115962 | 1.927557 | https://www.genecards.org/cgi-bin/carddisp.pl?gene=LVRN |
| AHRR | Aryl Hydrocarbon Receptor Repressor | Protein Coding | 34 | GC05P000321 | 1.927557 | https://www.genecards.org/cgi-bin/carddisp.pl?gene=AHRR |
| ZNF311 | Zinc Finger Protein 311 | Protein Coding | 30 | GC06M065802 | 1.927557 | https://www.genecards.org/cgi-bin/carddisp.pl?gene=ZNF311 |
| CASC2 | Cancer Susceptibility 2 | RNA Gene | 25 | GC10P118046 | 1.927557 | https://www.genecards.org/cgi-bin/carddisp.pl?gene=CASC2 |
| MIR135B | MicroRNA 135b | RNA Gene | 21 | GC01M205448 | 1.927557 | https://www.genecards.org/cgi-bin/carddisp.pl?gene=MIR135B |
| MIR375 | MicroRNA 375 | RNA Gene | 20 | GC02M219001 | 1.927557 | https://www.genecards.org/cgi-bin/carddisp.pl?gene=MIR375 |
| PRKG1-AS1 | PRKG1 Antisense RNA 1 | RNA Gene | 15 | GC10M052194 | 1.927557 | https://www.genecards.org/cgi-bin/carddisp.pl?gene=PRKG1-AS1 |
| SVIL-AS1 | SVIL Antisense RNA 1 | RNA Gene | 14 | GC10P029410 | 1.927557 | https://www.genecards.org/cgi-bin/carddisp.pl?gene=SVIL-AS1 |
| LINC02099 | Long Intergenic Non-Protein Coding RNA 2099 | RNA Gene | 13 | GC08P029787 | 1.927557 | https://www.genecards.org/cgi-bin/carddisp.pl?gene=LINC02099 |
| PGM5P2 | Phosphoglucomutase 5 Pseudogene 2 | Pseudogene | 11 | GC09M041006 | 1.927557 | https://www.genecards.org/cgi-bin/carddisp.pl?gene=PGM5P2 |
| VPS33B-DT | VPS33B Divergent Transcript | RNA Gene | 11 | GC15P118202 | 1.927557 | https://www.genecards.org/cgi-bin/carddisp.pl?gene=VPS33B-DT |

| | | | | | | |
|---|---|---|---|---|---|---|
| PRODH | Proline Dehydrogenase 1 | Protein Coding | 45 | GC22M018912 | 1.926246 | https://www.genecards.org/cgi-bin/carddisp.pl?gene=PRODH |
| MASP2 | MBL Associated Serine Protease 2 | Protein Coding | 44 | GC01M011026 | 1.925959 | https://www.genecards.org/cgi-bin/carddisp.pl?gene=MASP2 |
| CCN3 | Cellular Communication Network Factor 3 | Protein Coding | 32 | GC08P119416 | 1.925529 | https://www.genecards.org/cgi-bin/carddisp.pl?gene=CCN3 |
| MKKS | MKKS Centrosomal Shuttling Protein | Protein Coding | 38 | GC20M010424 | 1.919375 | https://www.genecards.org/cgi-bin/carddisp.pl?gene=MKKS |
| LY96 | Lymphocyte Antigen 96 | Protein Coding | 41 | GC08P073991 | 1.918517 | https://www.genecards.org/cgi-bin/carddisp.pl?gene=LY96 |
| MIR23B | MicroRNA 23b | RNA Gene | 21 | GC09P095085 | 1.917947 | https://www.genecards.org/cgi-bin/carddisp.pl?gene=MIR23B |
| FCGBP | Fc Gamma Binding Protein | Protein Coding | 35 | GC19M039863 | 1.916951 | https://www.genecards.org/cgi-bin/carddisp.pl?gene=FCGBP |
| NCF1 | Neutrophil Cytosolic Factor 1 | Protein Coding | 45 | GC07P075454 | 1.916344 | https://www.genecards.org/cgi-bin/carddisp.pl?gene=NCF1 |
| RECK | Reversion Inducing Cysteine Rich Protein With Kazal Motifs | Protein Coding | 40 | GC09P036036 | 1.913467 | https://www.genecards.org/cgi-bin/carddisp.pl?gene=RECK |
| BIRC2 | Baculoviral IAP Repeat Containing 2 | Protein Coding | 44 | GC11P102347 | 1.911999 | https://www.genecards.org/cgi-bin/carddisp.pl?gene=BIRC2 |
| XRCC5 | X-Ray Repair Cross Complementing 5 | Protein Coding | 42 | GC02P216107 | 1.910465 | https://www.genecards.org/cgi-bin/carddisp.pl?gene=XRCC5 |
| FCRL6 | Fc Receptor Like 6 | Protein Coding | 34 | GC01P159800 | 1.90864 | https://www.genecards.org/cgi-bin/carddisp.pl?gene=FCRL6 |
| AMPH | Amphiphysin | Protein Coding | 42 | GC07M039077 | 1.905197 | https://www.genecards.org/cgi-bin/carddisp.pl?gene=AMPH |
| PPL | Periplakin | Protein Coding | 40 | GC16M007350 | 1.905197 | https://www.genecards.org/cgi- |

| | | | | | | |
|---|---|---|---|---|---|---|
| | | | | | | bin/carddisp.pl?gene=PPL |
| EVPL | Envoplakin | Protein Coding | 36 | GC17M076004 | 1.905197 | https://www.genecards.org/cgi-bin/carddisp.pl?gene=EVPL |
| UGT1A1 | UDP Glucuronosyltransferase Family 1 Member A1 | Protein Coding | 48 | GC02P233760 | 1.894173 | https://www.genecards.org/cgi-bin/carddisp.pl?gene=UGT1A1 |
| PRLR | Prolactin Receptor | Protein Coding | 46 | GC05M035048 | 1.890139 | https://www.genecards.org/cgi-bin/carddisp.pl?gene=PRLR |
| TRPV2 | Transient Receptor Potential Cation Channel Subfamily V Member 2 | Protein Coding | 40 | GC17P016415 | 1.88929 | https://www.genecards.org/cgi-bin/carddisp.pl?gene=TRPV2 |
| PTK2 | Protein Tyrosine Kinase 2 | Protein Coding | 45 | GC08M140657 | 1.888314 | https://www.genecards.org/cgi-bin/carddisp.pl?gene=PTK2 |
| P2RY11 | Purinergic Receptor P2Y11 | Protein Coding | 42 | GC19P010454 | 1.887588 | https://www.genecards.org/cgi-bin/carddisp.pl?gene=P2RY11 |
| HK2 | Hexokinase 2 | Protein Coding | 45 | GC02P074833 | 1.885739 | https://www.genecards.org/cgi-bin/carddisp.pl?gene=HK2 |
| PTPN6 | Protein Tyrosine Phosphatase Non-Receptor Type 6 | Protein Coding | 47 | GC12P021091 | 1.885293 | https://www.genecards.org/cgi-bin/carddisp.pl?gene=PTPN6 |
| VCAN | Versican | Protein Coding | 46 | GC05P083471 | 1.88336 | https://www.genecards.org/cgi-bin/carddisp.pl?gene=VCAN |
| NKX2-1 | NK2 Homeobox 1 | Protein Coding | 44 | GC14M036516 | 1.882484 | https://www.genecards.org/cgi-bin/carddisp.pl?gene=NKX2-1 |
| MSTN | Myostatin | Protein Coding | 45 | GC02M190055 | 1.881957 | https://www.genecards.org/cgi-bin/carddisp.pl?gene=MSTN |
| PCDH15 | Protocadherin Related 15 | Protein Coding | 40 | GC10M053802 | 1.877976 | https://www.genecards.org/cgi-bin/carddisp.pl?gene=PCDH15 |
| MIR326 | MicroRNA 326 | RNA Gene | 22 | GC11M075335 | 1.877976 | https://www.genecards.org/cgi-bin/carddisp.pl?gene=MIR326 |

| | | | | | | |
|---|---|---|---|---|---|---|
| MIR181A1 | MicroRNA 181a-1 | RNA Gene | 20 | GC01M198860 | 1.877976 | https://www.genecards.org/cgi-bin/carddisp.pl?gene=MIR181A1 |
| IGHV1-69 | Immunoglobulin Heavy Variable 1-69 | Protein Coding | 21 | GC14M112841 | 1.872855 | https://www.genecards.org/cgi-bin/carddisp.pl?gene=IGHV1-69 |
| CEMIP | Cell Migration Inducing Hyaluronidase 1 | Protein Coding | 34 | GC15P080779 | 1.870194 | https://www.genecards.org/cgi-bin/carddisp.pl?gene=CEMIP |
| MMP26 | Matrix Metallopeptidase 26 | Protein Coding | 34 | GC11P004706 | 1.870128 | https://www.genecards.org/cgi-bin/carddisp.pl?gene=MMP26 |
| RIPK3 | Receptor Interacting Serine/Threonine Kinase 3 | Protein Coding | 40 | GC14M024336 | 1.869082 | https://www.genecards.org/cgi-bin/carddisp.pl?gene=RIPK3 |
| TNNT2 | Troponin T2, Cardiac Type | Protein Coding | 46 | GC01M201359 | 1.867014 | https://www.genecards.org/cgi-bin/carddisp.pl?gene=TNNT2 |
| TPH1 | Tryptophan Hydroxylase 1 | Protein Coding | 42 | GC11M018040 | 1.866312 | https://www.genecards.org/cgi-bin/carddisp.pl?gene=TPH1 |
| PRG2 | Proteoglycan 2, Pro Eosinophil Major Basic Protein | Protein Coding | 39 | GC11M057386 | 1.865162 | https://www.genecards.org/cgi-bin/carddisp.pl?gene=PRG2 |
| KCNN4 | Potassium Calcium-Activated Channel Subfamily N Member 4 | Protein Coding | 47 | GC19M065881 | 1.864761 | https://www.genecards.org/cgi-bin/carddisp.pl?gene=KCNN4 |
| BMP3 | Bone Morphogenetic Protein 3 | Protein Coding | 41 | GC04P081030 | 1.864489 | https://www.genecards.org/cgi-bin/carddisp.pl?gene=BMP3 |
| LILRA1 | Leukocyte Immunoglobulin Like Receptor A1 | Protein Coding | 36 | GC19P054593 | 1.862653 | https://www.genecards.org/cgi-bin/carddisp.pl?gene=LILRA1 |
| DRD4 | Dopamine Receptor D4 | Protein Coding | 45 | GC11P001768 | 1.860697 | https://www.genecards.org/cgi-bin/carddisp.pl?gene=DRD4 |
| VDAC1 | Voltage Dependent Anion Channel 1 | Protein Coding | 45 | GC05M133975 | 1.860697 | https://www.genecards.org/cgi-bin/carddisp.pl?gene=VDAC1 |
| DSCAM | DS Cell Adhesion Molecule | Protein Coding | 41 | GC21M040010 | 1.860697 | https://www.genecards.org/cgi- |

| | | | | | | |
|---|---|---|---|---|---|---|
| | | | | | | bin/carddisp.pl?gene=DSCAM |
| EIF2AK3 | Eukaryotic Translation Initiation Factor 2 Alpha Kinase 3 | Protein Coding | 46 | GC02M088556 | 1.857245 | https://www.genecards.org/cgi-bin/carddisp.pl?gene=EIF2AK3 |
| ANXA4 | Annexin A4 | Protein Coding | 42 | GC02P069644 | 1.854004 | https://www.genecards.org/cgi-bin/carddisp.pl?gene=ANXA4 |
| SFRP5 | Secreted Frizzled Related Protein 5 | Protein Coding | 38 | GC10M097766 | 1.854004 | https://www.genecards.org/cgi-bin/carddisp.pl?gene=SFRP5 |
| CIP2A | Cellular Inhibitor Of PP2A | Protein Coding | 28 | GC03M108573 | 1.854004 | https://www.genecards.org/cgi-bin/carddisp.pl?gene=CIP2A |
| CYSLTR2 | Cysteinyl Leukotriene Receptor 2 | Protein Coding | 45 | GC13P048653 | 1.850214 | https://www.genecards.org/cgi-bin/carddisp.pl?gene=CYSLTR2 |
| REV3L | REV3 Like, DNA Directed Polymerase Zeta Catalytic Subunit | Protein Coding | 44 | GC06M111299 | 1.850214 | https://www.genecards.org/cgi-bin/carddisp.pl?gene=REV3L |
| DDAH2 | Dimethylarginine Dimethylaminohydrolase 2 | Protein Coding | 42 | GC06M031727 | 1.850214 | https://www.genecards.org/cgi-bin/carddisp.pl?gene=DDAH2 |
| VARS2 | Valyl-TRNA Synthetase 2, Mitochondrial | Protein Coding | 42 | GC06P083680 | 1.850214 | https://www.genecards.org/cgi-bin/carddisp.pl?gene=VARS2 |
| PPT2 | Palmitoyl-Protein Thioesterase 2 | Protein Coding | 38 | GC06P032153 | 1.850214 | https://www.genecards.org/cgi-bin/carddisp.pl?gene=PPT2 |
| IER3 | Immediate Early Response 3 | Protein Coding | 37 | GC06M030743 | 1.850214 | https://www.genecards.org/cgi-bin/carddisp.pl?gene=IER3 |
| LY6G6C | Lymphocyte Antigen 6 Family Member G6C | Protein Coding | 34 | GC06M065967 | 1.850214 | https://www.genecards.org/cgi-bin/carddisp.pl?gene=LY6G6C |
| GPX4 | Glutathione Peroxidase 4 | Protein Coding | 45 | GC19P001103 | 1.847338 | https://www.genecards.org/cgi-bin/carddisp.pl?gene=GPX4 |
| SKIV2L | Ski2 Like RNA Helicase | Protein Coding | 42 | GC06P083717 | 1.842764 | https://www.genecards.org/cgi-bin/carddisp.pl?gene=SKIV2L |

| | | | | | | |
|---|---|---|---|---|---|---|
| DICER1 | Dicer 1, Ribonuclease III | Protein Coding | 46 | GC14M 095086 | 1.842 058 | https://www.genecards.org/cgi-bin/carddisp.pl?gene=DICER1 |
| TG | Thyroglobulin | Protein Coding | 43 | GC08P 132866 | 1.841 997 | https://www.genecards.org/cgi-bin/carddisp.pl?gene=TG |
| FZD4 | Frizzled Class Receptor 4 | Protein Coding | 48 | GC11M 086945 | 1.839 318 | https://www.genecards.org/cgi-bin/carddisp.pl?gene=FZD4 |
| DEFB103 B | Defensin Beta 103B | Protein Coding | 28 | GC08M 007430 | 1.839 135 | https://www.genecards.org/cgi-bin/carddisp.pl?gene=DEFB103B |
| RPS6KB1 | Ribosomal Protein S6 Kinase B1 | Protein Coding | 46 | GC17P 059893 | 1.837 89 | https://www.genecards.org/cgi-bin/carddisp.pl?gene=RPS6KB1 |
| GFAP | Glial Fibrillary Acidic Protein | Protein Coding | 45 | GC17M 044917 | 1.837 89 | https://www.genecards.org/cgi-bin/carddisp.pl?gene=GFAP |
| MIR138-1 | MicroRNA 138-1 | RNA Gene | 20 | GC03P 044115 | 1.837 89 | https://www.genecards.org/cgi-bin/carddisp.pl?gene=MIR138-1 |
| FLT3LG | Fms Related Receptor Tyrosine Kinase 3 Ligand | Protein Coding | 38 | GC19P 067098 | 1.831 33 | https://www.genecards.org/cgi-bin/carddisp.pl?gene=FLT3LG |
| PDCD1L G2 | Programmed Cell Death 1 Ligand 2 | Protein Coding | 40 | GC09P 005510 | 1.830 708 | https://www.genecards.org/cgi-bin/carddisp.pl?gene=PDCD1LG2 |
| WASF5P | WASP Family Member 5, Pseudogene | Pseudo gene | 10 | GC06M 031287 | 1.830 359 | https://www.genecards.org/cgi-bin/carddisp.pl?gene=WASF5P |
| MIR10A | MicroRNA 10a | RNA Gene | 22 | GC17M 048579 | 1.829 958 | https://www.genecards.org/cgi-bin/carddisp.pl?gene=MIR10A |
| LRP2 | LDL Receptor Related Protein 2 | Protein Coding | 47 | GC02M 169127 | 1.827 882 | https://www.genecards.org/cgi-bin/carddisp.pl?gene=LRP2 |
| KCNMA 1 | Potassium Calcium-Activated Channel Subfamily M Alpha 1 | Protein Coding | 48 | GC10M 076869 | 1.826 22 | https://www.genecards.org/cgi-bin/carddisp.pl?gene=KCNMA1 |
| CMA1 | Chymase 1 | Protein Coding | 42 | GC14M 024506 | 1.822 592 | https://www.genecards.org/cgi- |

| | | | | | | |
|---|---|---|---|---|---|---|
| | | | | | | bin/carddisp.pl?gene=CMA1 |
| RAD50 | RAD50 Double Strand Break Repair Protein | Protein Coding | 48 | GC05P132556 | 1.821556 | https://www.genecards.org/cgi-bin/carddisp.pl?gene=RAD50 |
| AFF4 | AF4/FMR2 Family Member 4 | Protein Coding | 41 | GC05M132875 | 1.821556 | https://www.genecards.org/cgi-bin/carddisp.pl?gene=AFF4 |
| ZFP36L1 | ZFP36 Ring Finger Protein Like 1 | Protein Coding | 41 | GC14M068787 | 1.821556 | https://www.genecards.org/cgi-bin/carddisp.pl?gene=ZFP36L1 |
| SRSF5 | Serine And Arginine Rich Splicing Factor 5 | Protein Coding | 38 | GC14P069727 | 1.821556 | https://www.genecards.org/cgi-bin/carddisp.pl?gene=SRSF5 |
| ZFYVE26 | Zinc Finger FYVE-Type Containing 26 | Protein Coding | 38 | GC14M067727 | 1.821556 | https://www.genecards.org/cgi-bin/carddisp.pl?gene=ZFYVE26 |
| ZCCHC10 | Zinc Finger CCHC-Type Containing 10 | Protein Coding | 32 | GC05M132996 | 1.821556 | https://www.genecards.org/cgi-bin/carddisp.pl?gene=ZCCHC10 |
| SUSD6 | Sushi Domain Containing 6 | Protein Coding | 31 | GC14P069611 | 1.821556 | https://www.genecards.org/cgi-bin/carddisp.pl?gene=SUSD6 |
| IRF1-AS1 | IRF1 Antisense RNA 1 | RNA Gene | 20 | GC05P132420 | 1.821556 | https://www.genecards.org/cgi-bin/carddisp.pl?gene=IRF1-AS1 |
| MEIKIN | Meiotic Kinetochore Factor | Protein Coding | 20 | GC05M131806 | 1.821556 | https://www.genecards.org/cgi-bin/carddisp.pl?gene=MEIKIN |
| MIR3936HG | MIR3936 Host Gene | RNA Gene | 13 | GC05M132312 | 1.821556 | https://www.genecards.org/cgi-bin/carddisp.pl?gene=MIR3936HG |
| LINC01882 | Long Intergenic Non-Protein Coding RNA 1882 | RNA Gene | 11 | GC18M024974 | 1.821556 | https://www.genecards.org/cgi-bin/carddisp.pl?gene=LINC01882 |
| DDX18P1 | DEAD-Box Helicase 18 Pseudogene 1 | Pseudogene | 9 | GC14P069083 | 1.821556 | https://www.genecards.org/cgi-bin/carddisp.pl?gene=DDX18P1 |
| LINC02863 | Long Intergenic Non-Protein Coding RNA 2863 | RNA Gene | 9 | GC05M132422 | 1.821556 | https://www.genecards.org/cgi-bin/carddisp.pl?gene=LINC02863 |

| | | | | | | |
|---|---|---|---|---|---|---|
| RNU6-921P | RNA, U6 Small Nuclear 921, Pseudogene | Pseudo gene | 9 | GC14M 068735 | 1.821 556 | https://www.genecards.org/cgi-bin/carddisp.pl?gene=RNU6-921P |
| ENSG00000202533 | Y RNA | RNA Gene | 8 | GC05P 132468 | 1.821 556 | https://www.genecards.org/cgi-bin/carddisp.pl?gene=ENSG00000202533 |
| HMGN1P3 | High Mobility Group Nucleosome Binding Domain 1 Pseudogene 3 | Pseudo gene | 8 | GC14M 068886 | 1.821 556 | https://www.genecards.org/cgi-bin/carddisp.pl?gene=HMGN1P3 |
| ENSG00000283782 | Novel Protein | Protein Coding | 7 | GC05P 132417 | 1.821 556 | https://www.genecards.org/cgi-bin/carddisp.pl?gene=ENSG00000283782 |
| ENSG00000289583 | Novel Transcript | RNA Gene | 6 | GC14M 068812 | 1.821 556 | https://www.genecards.org/cgi-bin/carddisp.pl?gene=ENSG00000289583 |
| ENSG00000224431 | Cytoplasmic Linker Associated Protein 2 (CLASP2) Pseudogene | Pseudo gene | 5 | GC05P 132199 | 1.821 556 | https://www.genecards.org/cgi-bin/carddisp.pl?gene=ENSG00000224431 |
| ENSG00000289626 | Novel Transcript, Sense Intronic To ZFP36L1 | RNA Gene | 5 | GC14M 068804 | 1.821 556 | https://www.genecards.org/cgi-bin/carddisp.pl?gene=ENSG00000289626 |
| HSALNG0102112 | | RNA Gene | 5 | GC14P 068766 | 1.821 556 | https://www.genecards.org/cgi-bin/carddisp.pl?gene=HSALNG0102112 |
| HSALNG0102114 | | RNA Gene | 5 | GC14P 068787 | 1.821 556 | https://www.genecards.org/cgi-bin/carddisp.pl?gene=HSALNG0102114 |
| HSALNG0102115 | | RNA Gene | 5 | GC14P 068789 | 1.821 556 | https://www.genecards.org/cgi-bin/carddisp.pl?gene=HSALNG0102115 |
| HSALNG0102116 | | RNA Gene | 5 | GC14P 068790 | 1.821 556 | https://www.genecards.org/cgi-bin/carddisp.pl?gene=HSALNG0102116 |
| lnc-IRF1-1 | | RNA Gene | 5 | GC05M 132473 | 1.821 556 | https://www.genecards.org/cgi-bin/carddisp.pl?gene=lnc-IRF1-1 |
| lnc-RAD51B-6 | | RNA Gene | 5 | GC14P 068795 | 1.821 556 | https://www.genecards.org/cgi-bin/carddisp.pl?gene=lnc-RAD51B-6 |
| HSALNG0044846 | | RNA Gene | 4 | GC05P 132467 | 1.821 556 | https://www.genecards.org/cgi- |

| | | | | | | |
|---|---|---|---|---|---|---|
| | | | | | | bin/carddisp.pl?gene=HSALNG0044846 |
| LOC107984709 | Uncharacterized LOC107984709 | RNA Gene | 4 | GC14M068793 | 1.821556 | https://www.genecards.org/cgi-bin/carddisp.pl?gene=LOC107984709 |
| MN298214 | | RNA Gene | 4 | GC05P132451 | 1.821556 | https://www.genecards.org/cgi-bin/carddisp.pl?gene=MN298214 |
| NPHS1 | NPHS1 Adhesion Molecule, Nephrin | Protein Coding | 44 | GC19M035825 | 1.821475 | https://www.genecards.org/cgi-bin/carddisp.pl?gene=NPHS1 |
| ANXA11 | Annexin A11 | Protein Coding | 43 | GC10M080150 | 1.820759 | https://www.genecards.org/cgi-bin/carddisp.pl?gene=ANXA11 |
| LAMP3 | Lysosomal Associated Membrane Protein 3 | Protein Coding | 38 | GC03M183122 | 1.819405 | https://www.genecards.org/cgi-bin/carddisp.pl?gene=LAMP3 |
| TICAM1 | TIR Domain Containing Adaptor Molecule 1 | Protein Coding | 42 | GC19M004815 | 1.818682 | https://www.genecards.org/cgi-bin/carddisp.pl?gene=TICAM1 |
| PLA2G2D | Phospholipase A2 Group IID | Protein Coding | 41 | GC01M020111 | 1.818682 | https://www.genecards.org/cgi-bin/carddisp.pl?gene=PLA2G2D |
| HABP2 | Hyaluronan Binding Protein 2 | Protein Coding | 45 | GC10P113550 | 1.818093 | https://www.genecards.org/cgi-bin/carddisp.pl?gene=HABP2 |
| IVNS1ABP | Influenza Virus NS1A Binding Protein | Protein Coding | 40 | GC01M185306 | 1.817918 | https://www.genecards.org/cgi-bin/carddisp.pl?gene=IVNS1ABP |
| GAD2 | Glutamate Decarboxylase 2 | Protein Coding | 42 | GC10P026216 | 1.817205 | https://www.genecards.org/cgi-bin/carddisp.pl?gene=GAD2 |
| ADAMTS9 | ADAM Metallopeptidase With Thrombospondin Type 1 Motif 9 | Protein Coding | 38 | GC03M064501 | 1.815725 | https://www.genecards.org/cgi-bin/carddisp.pl?gene=ADAMTS9 |
| CDK1 | Cyclin Dependent Kinase 1 | Protein Coding | 44 | GC10P060772 | 1.815159 | https://www.genecards.org/cgi-bin/carddisp.pl?gene=CDK1 |
| ARL1 | ADP Ribosylation Factor Like GTPase 1 | Protein Coding | 40 | GC12M101393 | 1.803441 | https://www.genecards.org/cgi-bin/carddisp.pl?gene=ARL1 |

| | | | | | | |
|---|---|---|---|---|---|---|
| PLCG1 | Phospholipase C Gamma 1 | Protein Coding | 46 | GC20P 041136 | 1.799 927 | https://www.genecards.org/cgi-bin/carddisp.pl?gene=PLCG1 |
| C4BPA | Complement Component 4 Binding Protein Alpha | Protein Coding | 41 | GC01P 207105 | 1.798 78 | https://www.genecards.org/cgi-bin/carddisp.pl?gene=C4BPA |
| POLR1C | RNA Polymerase I And III Subunit C | Protein Coding | 42 | GC06P 083925 | 1.794 698 | https://www.genecards.org/cgi-bin/carddisp.pl?gene=POLR1C |
| EIF4H | Eukaryotic Translation Initiation Factor 4H | Protein Coding | 40 | GC07P 074174 | 1.794 698 | https://www.genecards.org/cgi-bin/carddisp.pl?gene=EIF4H |
| FSTL4 | Follistatin Like 4 | Protein Coding | 35 | GC05M 133196 | 1.794 698 | https://www.genecards.org/cgi-bin/carddisp.pl?gene=FSTL4 |
| ANAPC4 | Anaphase Promoting Complex Subunit 4 | Protein Coding | 34 | GC04P 025379 | 1.794 698 | https://www.genecards.org/cgi-bin/carddisp.pl?gene=ANAPC4 |
| CREBBP | CREB Binding Protein | Protein Coding | 50 | GC16M 007298 | 1.792 465 | https://www.genecards.org/cgi-bin/carddisp.pl?gene=CREBBP |
| MIR193A | MicroRNA 193a | RNA Gene | 20 | GC17P 031559 | 1.790 309 | https://www.genecards.org/cgi-bin/carddisp.pl?gene=MIR193A |
| DEFA3 | Defensin Alpha 3 | Protein Coding | 36 | GC08M 007015 | 1.784 046 | https://www.genecards.org/cgi-bin/carddisp.pl?gene=DEFA3 |
| MC2R | Melanocortin 2 Receptor | Protein Coding | 46 | GC18M 024785 | 1.783 474 | https://www.genecards.org/cgi-bin/carddisp.pl?gene=MC2R |
| APOC3 | Apolipoprotein C3 | Protein Coding | 44 | GC11P 116829 | 1.783 474 | https://www.genecards.org/cgi-bin/carddisp.pl?gene=APOC3 |
| SMS | Spermine Synthase | Protein Coding | 42 | GC0XP 021958 | 1.783 474 | https://www.genecards.org/cgi-bin/carddisp.pl?gene=SMS |
| MIR29B1 | MicroRNA 29b-1 | RNA Gene | 21 | GC07M 130877 | 1.783 474 | https://www.genecards.org/cgi-bin/carddisp.pl?gene=MIR29B1 |
| MIR532 | MicroRNA 532 | RNA Gene | 17 | GC0XP 050769 | 1.783 474 | https://www.genecards.org/cgi- |

| | | | | | | |
|---|---|---|---|---|---|---|
| | | | | | | bin/carddisp.pl?gene=MIR532 |
| MANF | Mesencephalic Astrocyte Derived Neurotrophic Factor | Protein Coding | 37 | GC03P051385 | 1.778156 | https://www.genecards.org/cgi-bin/carddisp.pl?gene=MANF |
| MIR26B | MicroRNA 26b | RNA Gene | 22 | GC02P218402 | 1.778156 | https://www.genecards.org/cgi-bin/carddisp.pl?gene=MIR26B |
| AGT | Angiotensinogen | Protein Coding | 47 | GC01M230702 | 1.774172 | https://www.genecards.org/cgi-bin/carddisp.pl?gene=AGT |
| CCL14 | C-C Motif Chemokine Ligand 14 | Protein Coding | 34 | GC17M041940 | 1.773492 | https://www.genecards.org/cgi-bin/carddisp.pl?gene=CCL14 |
| TFF3 | Trefoil Factor 3 | Protein Coding | 40 | GC21M042311 | 1.772983 | https://www.genecards.org/cgi-bin/carddisp.pl?gene=TFF3 |
| CLEC2D | C-Type Lectin Domain Family 2 Member D | Protein Coding | 37 | GC12P021193 | 1.771824 | https://www.genecards.org/cgi-bin/carddisp.pl?gene=CLEC2D |
| SELENOS | Selenoprotein S | Protein Coding | 30 | GC15M117315 | 1.771824 | https://www.genecards.org/cgi-bin/carddisp.pl?gene=SELENOS |
| CPOX | Coproporphyrinogen Oxidase | Protein Coding | 42 | GC03M098576 | 1.770074 | https://www.genecards.org/cgi-bin/carddisp.pl?gene=CPOX |
| FUT1 | Fucosyltransferase 1 (H Blood Group) | Protein Coding | 39 | GC19M048748 | 1.769892 | https://www.genecards.org/cgi-bin/carddisp.pl?gene=FUT1 |
| BDP1P | B Double Prime 1 Pseudogene | Pseudo gene | 8 | GC18M077447 | 1.76978 | https://www.genecards.org/cgi-bin/carddisp.pl?gene=BDP1P |
| RPS26 | Ribosomal Protein S26 | Protein Coding | 41 | GC12P056043 | 1.764588 | https://www.genecards.org/cgi-bin/carddisp.pl?gene=RPS26 |
| TSC22D3 | TSC22 Domain Family Member 3 | Protein Coding | 39 | GC0XM107713 | 1.761598 | https://www.genecards.org/cgi-bin/carddisp.pl?gene=TSC22D3 |
| KRT8 | Keratin 8 | Protein Coding | 45 | GC12M052897 | 1.760741 | https://www.genecards.org/cgi-bin/carddisp.pl?gene=KRT8 |

| | | | | | | |
|---|---|---|---|---|---|---|
| AFF2 | AF4/FMR2 Family Member 2 | Protein Coding | 41 | GC0XP 148500 | 1.760 741 | https://www.genecards.org/cgi-bin/carddisp.pl?gene=AFF2 |
| ASRGL1 | Asparaginase And Isoaspartyl Peptidase 1 | Protein Coding | 40 | GC11P 062337 | 1.760 741 | https://www.genecards.org/cgi-bin/carddisp.pl?gene=ASRGL1 |
| BCL2L2 | BCL2 Like 2 | Protein Coding | 43 | GC14P 032837 | 1.759 391 | https://www.genecards.org/cgi-bin/carddisp.pl?gene=BCL2L2 |
| MUSK | Muscle Associated Receptor Tyrosine Kinase | Protein Coding | 46 | GC09P 110668 | 1.759 109 | https://www.genecards.org/cgi-bin/carddisp.pl?gene=MUSK |
| B4GALT 1 | Beta-1,4-Galactosyltransferase 1 | Protein Coding | 44 | GC09M 033100 | 1.758 877 | https://www.genecards.org/cgi-bin/carddisp.pl?gene=B4GALT1 |
| NCR3 | Natural Cytotoxicity Triggering Receptor 3 | Protein Coding | 38 | GC06M 031588 | 1.758 201 | https://www.genecards.org/cgi-bin/carddisp.pl?gene=NCR3 |
| CDH5 | Cadherin 5 | Protein Coding | 45 | GC16P 066366 | 1.757 975 | https://www.genecards.org/cgi-bin/carddisp.pl?gene=CDH5 |
| ID2 | Inhibitor Of DNA Binding 2 | Protein Coding | 42 | GC02P 008678 | 1.757 975 | https://www.genecards.org/cgi-bin/carddisp.pl?gene=ID2 |
| SPINK5 | Serine Peptidase Inhibitor Kazal Type 5 | Protein Coding | 41 | GC05P 148025 | 1.757 052 | https://www.genecards.org/cgi-bin/carddisp.pl?gene=SPINK5 |
| RPLP0 | Ribosomal Protein Lateral Stalk Subunit P0 | Protein Coding | 40 | GC12M 120196 | 1.753 61 | https://www.genecards.org/cgi-bin/carddisp.pl?gene=RPLP0 |
| LPXN | Leupaxin | Protein Coding | 40 | GC11M 089460 | 1.751 273 | https://www.genecards.org/cgi-bin/carddisp.pl?gene=LPXN |
| NTAN1 | N-Terminal Asparagine Amidase | Protein Coding | 37 | GC16M 015037 | 1.751 273 | https://www.genecards.org/cgi-bin/carddisp.pl?gene=NTAN1 |
| MIR629 | MicroRNA 629 | RNA Gene | 19 | GC15M 070079 | 1.749 573 | https://www.genecards.org/cgi-bin/carddisp.pl?gene=MIR629 |
| MIR600 | MicroRNA 600 | RNA Gene | 15 | GC09M 123111 | 1.749 573 | https://www.genecards.org/cgi- |

| | | | | | | |
|---|---|---|---|---|---|---|
| | | | | | | bin/carddisp.pl?gene=MIR600 |
| CRHR1 | Corticotropin Releasing Hormone Receptor 1 | Protein Coding | 42 | GC17P045784 | 1.747556 | https://www.genecards.org/cgi-bin/carddisp.pl?gene=CRHR1 |
| NLRC4 | NLR Family CARD Domain Containing 4 | Protein Coding | 42 | GC02M032224 | 1.743058 | https://www.genecards.org/cgi-bin/carddisp.pl?gene=NLRC4 |
| TXK | TXK Tyrosine Kinase | Protein Coding | 44 | GC04M048066 | 1.742146 | https://www.genecards.org/cgi-bin/carddisp.pl?gene=TXK |
| RHOD | Ras Homolog Family Member D | Protein Coding | 37 | GC11P070125 | 1.741763 | https://www.genecards.org/cgi-bin/carddisp.pl?gene=RHOD |
| RPS2P14 | Ribosomal Protein S2 Pseudogene 14 | Pseudo gene | 7 | GC01M113698 | 1.740258 | https://www.genecards.org/cgi-bin/carddisp.pl?gene=RPS2P14 |
| LOC102724971 | Putative V-Set And Immunoglobulin Domain-Containing-Like Protein IGHV4OR15-8 | Protein Coding | 5 | GC15M021742 | 1.73744 | https://www.genecards.org/cgi-bin/carddisp.pl?gene=LOC102724971 |
| ROBO3 | Roundabout Guidance Receptor 3 | Protein Coding | 43 | GC11P124865 | 1.73387 | https://www.genecards.org/cgi-bin/carddisp.pl?gene=ROBO3 |
| MIR424 | MicroRNA 424 | RNA Gene | 18 | GC0XM134693 | 1.73387 | https://www.genecards.org/cgi-bin/carddisp.pl?gene=MIR424 |
| MIR188 | MicroRNA 188 | RNA Gene | 17 | GC0XP050003 | 1.73387 | https://www.genecards.org/cgi-bin/carddisp.pl?gene=MIR188 |
| PITRM1 | Pitrilysin Metallopeptidase 1 | Protein Coding | 40 | GC10M003138 | 1.732475 | https://www.genecards.org/cgi-bin/carddisp.pl?gene=PITRM1 |
| ASIC3 | Acid Sensing Ion Channel Subunit 3 | Protein Coding | 38 | GC07P151048 | 1.730679 | https://www.genecards.org/cgi-bin/carddisp.pl?gene=ASIC3 |
| CHST4 | Carbohydrate Sulfotransferase 4 | Protein Coding | 36 | GC16P072011 | 1.730679 | https://www.genecards.org/cgi-bin/carddisp.pl?gene=CHST4 |
| CTSH | Cathepsin H | Protein Coding | 46 | GC15M090448 | 1.729593 | https://www.genecards.org/cgi-bin/carddisp.pl?gene=CTSH |

| | | | | | | |
|---|---|---|---|---|---|---|
| TRAF3IP2-AS1 | TRAF3IP2 Antisense RNA 1 | RNA Gene | 19 | GC06P111483 | 1.729137 | https://www.genecards.org/cgi-bin/carddisp.pl?gene=TRAF3IP2-AS1 |
| CD74 | CD74 Molecule | Protein Coding | 42 | GC05M150378 | 1.729081 | https://www.genecards.org/cgi-bin/carddisp.pl?gene=CD74 |
| MOCOS | Molybdenum Cofactor Sulfurase | Protein Coding | 40 | GC18P036187 | 1.726709 | https://www.genecards.org/cgi-bin/carddisp.pl?gene=MOCOS |
| MFGE8 | Milk Fat Globule EGF And Factor V/VIII Domain Containing | Protein Coding | 42 | GC15M088898 | 1.725653 | https://www.genecards.org/cgi-bin/carddisp.pl?gene=MFGE8 |
| GNS | Glucosamine (N-Acetyl)-6-Sulfatase | Protein Coding | 43 | GC12M064713 | 1.724605 | https://www.genecards.org/cgi-bin/carddisp.pl?gene=GNS |
| SIRT5 | Sirtuin 5 | Protein Coding | 44 | GC06P013574 | 1.72418 | https://www.genecards.org/cgi-bin/carddisp.pl?gene=SIRT5 |
| SLC39A8 | Solute Carrier Family 39 Member 8 | Protein Coding | 42 | GC04M102252 | 1.719299 | https://www.genecards.org/cgi-bin/carddisp.pl?gene=SLC39A8 |
| NFATC2 | Nuclear Factor Of Activated T Cells 2 | Protein Coding | 42 | GC20M051386 | 1.718933 | https://www.genecards.org/cgi-bin/carddisp.pl?gene=NFATC2 |
| DNAJA2 | DnaJ Heat Shock Protein Family (Hsp40) Member A2 | Protein Coding | 39 | GC16M046955 | 1.718933 | https://www.genecards.org/cgi-bin/carddisp.pl?gene=DNAJA2 |
| SLC2A1 | Solute Carrier Family 2 Member 1 | Protein Coding | 50 | GC01M042925 | 1.718165 | https://www.genecards.org/cgi-bin/carddisp.pl?gene=SLC2A1 |
| SLC7A5 | Solute Carrier Family 7 Member 5 | Protein Coding | 43 | GC16M087830 | 1.715385 | https://www.genecards.org/cgi-bin/carddisp.pl?gene=SLC7A5 |
| MAF | MAF BZIP Transcription Factor | Protein Coding | 44 | GC16M079204 | 1.714878 | https://www.genecards.org/cgi-bin/carddisp.pl?gene=MAF |
| DLL1 | Delta Like Canonical Notch Ligand 1 | Protein Coding | 44 | GC06M170282 | 1.713073 | https://www.genecards.org/cgi-bin/carddisp.pl?gene=DLL1 |
| SSRP1 | Structure Specific Recognition Protein 1 | Protein Coding | 39 | GC11M089441 | 1.712 | https://www.genecards.org/cgi- |

| | | | | | | |
|---|---|---|---|---|---|---|
| | | | | | | bin/carddisp.pl?gene=SSRP1 |
| BMPR2 | Bone Morphogenetic Protein Receptor Type 2 | Protein Coding | 48 | GC02P202376 | 1.711393 | https://www.genecards.org/cgi-bin/carddisp.pl?gene=BMPR2 |
| HSF1 | Heat Shock Transcription Factor 1 | Protein Coding | 43 | GC08P144291 | 1.707303 | https://www.genecards.org/cgi-bin/carddisp.pl?gene=HSF1 |
| CD96 | CD96 Molecule | Protein Coding | 42 | GC03P111292 | 1.707303 | https://www.genecards.org/cgi-bin/carddisp.pl?gene=CD96 |
| MIR26A1 | MicroRNA 26a-1 | RNA Gene | 21 | GC03P037969 | 1.707303 | https://www.genecards.org/cgi-bin/carddisp.pl?gene=MIR26A1 |
| ST2 | Suppression Of Tumorigenicity 2 | Genetic Locus | 7 | GC11U990127 | 1.707303 | https://www.genecards.org/cgi-bin/carddisp.pl?gene=ST2 |
| GLS | Glutaminase | Protein Coding | 47 | GC02P190880 | 1.706537 | https://www.genecards.org/cgi-bin/carddisp.pl?gene=GLS |
| MPPE1 | Metallophosphoesterase 1 | Protein Coding | 37 | GC18M024771 | 1.706537 | https://www.genecards.org/cgi-bin/carddisp.pl?gene=MPPE1 |
| RBM17 | RNA Binding Motif Protein 17 | Protein Coding | 37 | GC10P006089 | 1.706537 | https://www.genecards.org/cgi-bin/carddisp.pl?gene=RBM17 |
| CEP192 | Centrosomal Protein 192 | Protein Coding | 36 | GC18P017459 | 1.706537 | https://www.genecards.org/cgi-bin/carddisp.pl?gene=CEP192 |
| CEP76 | Centrosomal Protein 76 | Protein Coding | 35 | GC18M024777 | 1.706537 | https://www.genecards.org/cgi-bin/carddisp.pl?gene=CEP76 |
| RAVER1 | Ribonucleoprotein, PTB Binding 1 | Protein Coding | 33 | GC19M010316 | 1.706537 | https://www.genecards.org/cgi-bin/carddisp.pl?gene=RAVER1 |
| CCDC116 | Coiled-Coil Domain Containing 116 | Protein Coding | 32 | GC22P037047 | 1.706537 | https://www.genecards.org/cgi-bin/carddisp.pl?gene=CCDC116 |
| PRR5L | Proline Rich 5 Like | Protein Coding | 30 | GC11P036297 | 1.706537 | https://www.genecards.org/cgi-bin/carddisp.pl?gene=PRR5L |

| | | | | | | |
|---|---|---|---|---|---|---|
| RIMBP3C | RIMS Binding Protein 3C | Protein Coding | 26 | GC22M023542 | 1.706537 | https://www.genecards.org/cgi-bin/carddisp.pl?gene=RIMBP3C |
| PI4KAP2 | Phosphatidylinositol 4-Kinase Alpha Pseudogene 2 | Pseudogene | 22 | GC22M021472 | 1.706537 | https://www.genecards.org/cgi-bin/carddisp.pl?gene=PI4KAP2 |
| THAP7-AS1 | THAP7 Antisense RNA 1 | RNA Gene | 15 | GC22P035904 | 1.706537 | https://www.genecards.org/cgi-bin/carddisp.pl?gene=THAP7-AS1 |
| IGLV5-52 | Immunoglobulin Lambda Variable 5-52 | Protein Coding | 13 | GC22P022318 | 1.706537 | https://www.genecards.org/cgi-bin/carddisp.pl?gene=IGLV5-52 |
| ABHD17AP4 | ABHD17A Pseudogene 4 | Pseudogene | 10 | GC22M020667 | 1.706537 | https://www.genecards.org/cgi-bin/carddisp.pl?gene=ABHD17AP4 |
| STAT4-AS1 | STAT4 Antisense RNA 1 | RNA Gene | 10 | GC02P191023 | 1.706537 | https://www.genecards.org/cgi-bin/carddisp.pl?gene=STAT4-AS1 |
| TSBP1-AS1 | TSBP1 And BTNL2 Antisense RNA 1 | RNA Gene | 10 | GC06P087405 | 1.706537 | https://www.genecards.org/cgi-bin/carddisp.pl?gene=TSBP1-AS1 |
| ENSG00000234630 | Novel Transcript | RNA Gene | 9 | GC22P035947 | 1.706537 | https://www.genecards.org/cgi-bin/carddisp.pl?gene=ENSG00000234630 |
| ENSG00000267199 | Novel Transcript, Antisense SPIRE1 | RNA Gene | 9 | GC18P017448 | 1.706537 | https://www.genecards.org/cgi-bin/carddisp.pl?gene=ENSG00000267199 |
| ENSG00000267480 | Novel Transcript, Antisense IMPA2 | RNA Gene | 9 | GC18M024891 | 1.706537 | https://www.genecards.org/cgi-bin/carddisp.pl?gene=ENSG00000267480 |
| HSALNG0120134 | | RNA Gene | 5 | GC18P017594 | 1.706537 | https://www.genecards.org/cgi-bin/carddisp.pl?gene=HSALNG0120134 |
| HSALNG0120135 | | RNA Gene | 5 | GC18M024927 | 1.706537 | https://www.genecards.org/cgi-bin/carddisp.pl?gene=HSALNG0120135 |
| lnc-SEH1L-5 | | RNA Gene | 5 | GC18P017450 | 1.706537 | https://www.genecards.org/cgi-bin/carddisp.pl?gene=lnc-SEH1L-5 |
| piR-53431-298 | | RNA Gene | 5 | GC18P017731 | 1.706537 | https://www.genecards.org/cgi- |

| | | | | | | |
|---|---|---|---|---|---|---|
| | | | | | | bin/carddisp.pl?gene=piR-53431-298 |
| lnc-ICAM3-1 | | RNA Gene | 4 | GC19M010402 | 1.706537 | https://www.genecards.org/cgi-bin/carddisp.pl?gene=lnc-ICAM3-1 |
| piR-61101-324 | | RNA Gene | 4 | GC19P010348 | 1.706537 | https://www.genecards.org/cgi-bin/carddisp.pl?gene=piR-61101-324 |
| lnc-ICAM3-2 | | RNA Gene | 3 | GC19M010354 | 1.706537 | https://www.genecards.org/cgi-bin/carddisp.pl?gene=lnc-ICAM3-2 |
| EREG | Epiregulin | Protein Coding | 41 | GC04P074366 | 1.701794 | https://www.genecards.org/cgi-bin/carddisp.pl?gene=EREG |
| LAP3 | Leucine Aminopeptidase 3 | Protein Coding | 41 | GC04P018237 | 1.698339 | https://www.genecards.org/cgi-bin/carddisp.pl?gene=LAP3 |
| PER2 | Period Circadian Regulator 2 | Protein Coding | 42 | GC02M238244 | 1.697734 | https://www.genecards.org/cgi-bin/carddisp.pl?gene=PER2 |
| MIRLET7A1 | MicroRNA Let-7a-1 | RNA Gene | 21 | GC09P094175 | 1.697734 | https://www.genecards.org/cgi-bin/carddisp.pl?gene=MIRLET7A1 |
| HLA-DRB2 | Major Histocompatibility Complex, Class II, DR Beta 2 (Pseudogene) | Pseudo gene | 10 | GC06Mn03753 | 1.697423 | https://www.genecards.org/cgi-bin/carddisp.pl?gene=HLA-DRB2 |
| SERPINA4 | Serpin Family A Member 4 | Protein Coding | 41 | GC14P094561 | 1.697273 | https://www.genecards.org/cgi-bin/carddisp.pl?gene=SERPINA4 |
| DNM1L | Dynamin 1 Like | Protein Coding | 46 | GC12P032679 | 1.696452 | https://www.genecards.org/cgi-bin/carddisp.pl?gene=DNM1L |
| FOXM1 | Forkhead Box M1 | Protein Coding | 42 | GC12M002857 | 1.696452 | https://www.genecards.org/cgi-bin/carddisp.pl?gene=FOXM1 |
| RASGRF1 | Ras Protein Specific Guanine Nucleotide Releasing Factor 1 | Protein Coding | 40 | GC15M078959 | 1.696452 | https://www.genecards.org/cgi-bin/carddisp.pl?gene=RASGRF1 |
| SNRPA | Small Nuclear Ribonucleoprotein Polypeptide A | Protein Coding | 39 | GC19P040750 | 1.696393 | https://www.genecards.org/cgi-bin/carddisp.pl?gene=SNRPA |

| | | | | | | |
|---|---|---|---|---|---|---|
| SLC22A1 | Solute Carrier Family 22 Member 1 | Protein Coding | 42 | GC06P 160121 | 1.696 227 | https://www.genecards.org/cgi-bin/carddisp.pl?gene=SLC22A1 |
| LINC02381 | Long Intergenic Non-Protein Coding RNA 2381 | RNA Gene | 10 | GC12P 055052 | 1.696 227 | https://www.genecards.org/cgi-bin/carddisp.pl?gene=LINC02381 |
| IL17RB | Interleukin 17 Receptor B | Protein Coding | 40 | GC03P 053855 | 1.694 728 | https://www.genecards.org/cgi-bin/carddisp.pl?gene=IL17RB |
| PLCG2 | Phospholipase C Gamma 2 | Protein Coding | 50 | GC16P 081773 | 1.689 247 | https://www.genecards.org/cgi-bin/carddisp.pl?gene=PLCG2 |
| CTSS | Cathepsin S | Protein Coding | 44 | GC01M 150730 | 1.687 668 | https://www.genecards.org/cgi-bin/carddisp.pl?gene=CTSS |
| IRF9 | Interferon Regulatory Factor 9 | Protein Coding | 42 | GC14P 024161 | 1.687 188 | https://www.genecards.org/cgi-bin/carddisp.pl?gene=IRF9 |
| GP2 | Glycoprotein 2 | Protein Coding | 40 | GC16M 020309 | 1.687 188 | https://www.genecards.org/cgi-bin/carddisp.pl?gene=GP2 |
| ITGAE | Integrin Subunit Alpha E | Protein Coding | 39 | GC17M 004158 | 1.687 188 | https://www.genecards.org/cgi-bin/carddisp.pl?gene=ITGAE |
| CTBS | Chitobiase | Protein Coding | 38 | GC01M 084549 | 1.687 188 | https://www.genecards.org/cgi-bin/carddisp.pl?gene=CTBS |
| IFI27 | Interferon Alpha Inducible Protein 27 | Protein Coding | 38 | GC14P 094104 | 1.687 188 | https://www.genecards.org/cgi-bin/carddisp.pl?gene=IFI27 |
| MIR495 | MicroRNA 495 | RNA Gene | 17 | GC14P 109998 | 1.687 188 | https://www.genecards.org/cgi-bin/carddisp.pl?gene=MIR495 |
| THRIL | TNF And HNRNPL Related Immunoregulatory Long Non-Coding RNA | RNA Gene | 12 | GC12M 125025 | 1.687 188 | https://www.genecards.org/cgi-bin/carddisp.pl?gene=THRIL |
| FGF7 | Fibroblast Growth Factor 7 | Protein Coding | 40 | GC15P 049423 | 1.685 435 | https://www.genecards.org/cgi-bin/carddisp.pl?gene=FGF7 |
| ITIH2 | Inter-Alpha-Trypsin Inhibitor Heavy Chain 2 | Protein Coding | 40 | GC10P 007703 | 1.684 724 | https://www.genecards.org/cgi- |

| | | | | | | |
|---|---|---|---|---|---|---|
| | | | | | | bin/carddisp.pl?gene=ITIH2 |
| LTA4H | Leukotriene A4 Hydrolase | Protein Coding | 44 | GC12M096000 | 1.684635 | https://www.genecards.org/cgi-bin/carddisp.pl?gene=LTA4H |
| STK16 | Serine/Threonine Kinase 16 | Protein Coding | 39 | GC02P219248 | 1.681913 | https://www.genecards.org/cgi-bin/carddisp.pl?gene=STK16 |
| WBP1 | WW Domain Binding Protein 1 | Protein Coding | 33 | GC02P074458 | 1.678863 | https://www.genecards.org/cgi-bin/carddisp.pl?gene=WBP1 |
| FGB | Fibrinogen Beta Chain | Protein Coding | 46 | GC04P154595 | 1.67643 | https://www.genecards.org/cgi-bin/carddisp.pl?gene=FGB |
| KLF5 | Kruppel Like Factor 5 | Protein Coding | 44 | GC13P073054 | 1.67643 | https://www.genecards.org/cgi-bin/carddisp.pl?gene=KLF5 |
| MMP28 | Matrix Metallopeptidase 28 | Protein Coding | 36 | GC17M035756 | 1.67643 | https://www.genecards.org/cgi-bin/carddisp.pl?gene=MMP28 |
| CNNM3-DT | CNNM3 Divergent Transcript | RNA Gene | 9 | GC02M098269 | 1.67643 | https://www.genecards.org/cgi-bin/carddisp.pl?gene=CNNM3-DT |
| ARNT | Aryl Hydrocarbon Receptor Nuclear Translocator | Protein Coding | 42 | GC01M150809 | 1.674134 | https://www.genecards.org/cgi-bin/carddisp.pl?gene=ARNT |
| FGF5 | Fibroblast Growth Factor 5 | Protein Coding | 42 | GC04P080266 | 1.674134 | https://www.genecards.org/cgi-bin/carddisp.pl?gene=FGF5 |
| NR2C2 | Nuclear Receptor Subfamily 2 Group C Member 2 | Protein Coding | 42 | GC03P014947 | 1.674134 | https://www.genecards.org/cgi-bin/carddisp.pl?gene=NR2C2 |
| GPANK1 | G-Patch Domain And Ankyrin Repeats 1 | Protein Coding | 32 | GC06M065962 | 1.672641 | https://www.genecards.org/cgi-bin/carddisp.pl?gene=GPANK1 |
| AHNAK | AHNAK Nucleoprotein | Protein Coding | 37 | GC11M089610 | 1.664649 | https://www.genecards.org/cgi-bin/carddisp.pl?gene=AHNAK |
| SERPINA12 | Serpin Family A Member 12 | Protein Coding | 38 | GC14M094487 | 1.661815 | https://www.genecards.org/cgi-bin/carddisp.pl?gene=SERPINA12 |

| | | | | | | |
|---|---|---|---|---|---|---|
| BCHE | Butyrylcholinesterase | Protein Coding | 49 | GC03M 165772 | 1.660 317 | https://www.genecards .org/cgi-bin/carddisp.pl?gene= BCHE |
| CETP | Cholesteryl Ester Transfer Protein | Protein Coding | 46 | GC16P 056961 | 1.660 317 | https://www.genecards .org/cgi-bin/carddisp.pl?gene= CETP |
| CPT1A | Carnitine Palmitoyltransferase 1A | Protein Coding | 46 | GC11M 068754 | 1.660 317 | https://www.genecards .org/cgi-bin/carddisp.pl?gene= CPT1A |
| PLTP | Phospholipid Transfer Protein | Protein Coding | 43 | GC20M 045898 | 1.660 317 | https://www.genecards .org/cgi-bin/carddisp.pl?gene= PLTP |
| ADIPOR 2 | Adiponectin Receptor 2 | Protein Coding | 41 | GC12P 001670 | 1.660 317 | https://www.genecards .org/cgi-bin/carddisp.pl?gene= ADIPOR2 |
| REG1A | Regenerating Family Member 1 Alpha | Protein Coding | 40 | GC02P 079120 | 1.660 317 | https://www.genecards .org/cgi-bin/carddisp.pl?gene= REG1A |
| ATP6V0 E2 | ATPase H+ Transporting V0 Subunit E2 | Protein Coding | 36 | GC07P 149873 | 1.660 317 | https://www.genecards .org/cgi-bin/carddisp.pl?gene= ATP6V0E2 |
| MIR25 | MicroRNA 25 | RNA Gene | 21 | GC07M 100093 | 1.660 317 | https://www.genecards .org/cgi-bin/carddisp.pl?gene= MIR25 |
| MIR27B | MicroRNA 27b | RNA Gene | 21 | GC09P 095097 | 1.660 317 | https://www.genecards .org/cgi-bin/carddisp.pl?gene= MIR27B |
| MIR378A | MicroRNA 378a | RNA Gene | 21 | GC05P 149732 | 1.660 317 | https://www.genecards .org/cgi-bin/carddisp.pl?gene= MIR378A |
| MIR574 | MicroRNA 574 | RNA Gene | 20 | GC04P 038948 | 1.660 317 | https://www.genecards .org/cgi-bin/carddisp.pl?gene= MIR574 |
| MIR224 | MicroRNA 224 | RNA Gene | 18 | GC0X M1519 58 | 1.660 317 | https://www.genecards .org/cgi-bin/carddisp.pl?gene= MIR224 |
| MIR340 | MicroRNA 340 | RNA Gene | 20 | GC05M 180015 | 1.655 23 | https://www.genecards .org/cgi-bin/carddisp.pl?gene= MIR340 |
| MX1 | MX Dynamin Like GTPase 1 | Protein Coding | 40 | GC21P 041420 | 1.654 687 | https://www.genecards .org/cgi- |

| | | | | | | |
|---|---|---|---|---|---|---|
| | | | | | | bin/carddisp.pl?gene=MX1 |
| AGRN | Agrin | Protein Coding | 44 | GC01P001020 | 1.654424 | https://www.genecards.org/cgi-bin/carddisp.pl?gene=AGRN |
| TNXA | Tenascin XA (Pseudogene) | Pseudo gene | 23 | GC06M065980 | 1.654226 | https://www.genecards.org/cgi-bin/carddisp.pl?gene=TNXA |
| RPS6KA3 | Ribosomal Protein S6 Kinase A3 | Protein Coding | 50 | GC0XM020149 | 1.653955 | https://www.genecards.org/cgi-bin/carddisp.pl?gene=RPS6KA3 |
| SLIT3 | Slit Guidance Ligand 3 | Protein Coding | 41 | GC05M168661 | 1.650758 | https://www.genecards.org/cgi-bin/carddisp.pl?gene=SLIT3 |
| IDDM8 | Insulin Dependent Diabetes Mellitus 8 | Genetic Locus | 4 | GC06U990066 | 1.650758 | https://www.genecards.org/cgi-bin/carddisp.pl?gene=IDDM8 |
| ADGRG1 | Adhesion G Protein-Coupled Receptor G1 | Protein Coding | 38 | GC16P057610 | 1.648388 | https://www.genecards.org/cgi-bin/carddisp.pl?gene=ADGRG1 |
| OR6C4 | Olfactory Receptor Family 6 Subfamily C Member 4 | Protein Coding | 32 | GC12P055553 | 1.648388 | https://www.genecards.org/cgi-bin/carddisp.pl?gene=OR6C4 |
| DEFB1 | Defensin Beta 1 | Protein Coding | 38 | GC08M006870 | 1.646858 | https://www.genecards.org/cgi-bin/carddisp.pl?gene=DEFB1 |
| C1D | C1D Nuclear Receptor Corepressor | Protein Coding | 38 | GC02M068041 | 1.645422 | https://www.genecards.org/cgi-bin/carddisp.pl?gene=C1D |
| PLP1 | Proteolipid Protein 1 | Protein Coding | 41 | GC0XP103773 | 1.644365 | https://www.genecards.org/cgi-bin/carddisp.pl?gene=PLP1 |
| RPTOR | Regulatory Associated Protein Of MTOR Complex 1 | Protein Coding | 42 | GC17P080544 | 1.64344 | https://www.genecards.org/cgi-bin/carddisp.pl?gene=RPTOR |
| GBP5 | Guanylate Binding Protein 5 | Protein Coding | 36 | GC01M089259 | 1.642158 | https://www.genecards.org/cgi-bin/carddisp.pl?gene=GBP5 |
| SH3PXD2A | SH3 And PX Domains 2A | Protein Coding | 38 | GC10M103594 | 1.641088 | https://www.genecards.org/cgi-bin/carddisp.pl?gene=SH3PXD2A |

| | | | | | | |
|---|---|---|---|---|---|---|
| DPP7 | Dipeptidyl Peptidase 7 | Protein Coding | 39 | GC09M 137569 | 1.638 876 | https://www.genecards.org/cgi-bin/carddisp.pl?gene=DPP7 |
| THNSL2 | Threonine Synthase Like 2 | Protein Coding | 33 | GC02P 088170 | 1.638 876 | https://www.genecards.org/cgi-bin/carddisp.pl?gene=THNSL2 |
| TKT | Transketolase | Protein Coding | 45 | GC03M 053224 | 1.637 501 | https://www.genecards.org/cgi-bin/carddisp.pl?gene=TKT |
| TOP1 | DNA Topoisomerase I | Protein Coding | 47 | GC20P 041028 | 1.630 69 | https://www.genecards.org/cgi-bin/carddisp.pl?gene=TOP1 |
| LOC1067 80803 | Tenascin XB Recombination Region | Biologi cal Region | 3 | GC06P 085230 | 1.630 337 | https://www.genecards.org/cgi-bin/carddisp.pl?gene=LOC106780803 |
| LOC1067 80804 | Tenascin XA (Pseudogene) Recombination Region | Biologi cal Region | 2 | GC06P 085231 | 1.630 337 | https://www.genecards.org/cgi-bin/carddisp.pl?gene=LOC106780804 |
| IL20RA | Interleukin 20 Receptor Subunit Alpha | Protein Coding | 36 | GC06M 136999 | 1.629 896 | https://www.genecards.org/cgi-bin/carddisp.pl?gene=IL20RA |
| SLC37A1 | Solute Carrier Family 37 Member 1 | Protein Coding | 37 | GC21P 042571 | 1.626 416 | https://www.genecards.org/cgi-bin/carddisp.pl?gene=SLC37A1 |
| SDC4 | Syndecan 4 | Protein Coding | 42 | GC20M 045325 | 1.625 002 | https://www.genecards.org/cgi-bin/carddisp.pl?gene=SDC4 |
| SELENO N | Selenoprotein N | Protein Coding | 32 | GC01P 025800 | 1.623 947 | https://www.genecards.org/cgi-bin/carddisp.pl?gene=SELENON |
| CD47 | CD47 Molecule | Protein Coding | 41 | GC03M 108043 | 1.623 184 | https://www.genecards.org/cgi-bin/carddisp.pl?gene=CD47 |
| SCUBE2 | Signal Peptide, CUB Domain And EGF Like Domain Containing 2 | Protein Coding | 40 | GC11M 009019 | 1.623 03 | https://www.genecards.org/cgi-bin/carddisp.pl?gene=SCUBE2 |
| EGLN1 | Egl-9 Family Hypoxia Inducible Factor 1 | Protein Coding | 48 | GC01M 231363 | 1.622 046 | https://www.genecards.org/cgi-bin/carddisp.pl?gene=EGLN1 |
| CPQ | Carboxypeptidase Q | Protein Coding | 36 | GC08P 096645 | 1.622 046 | https://www.genecards.org/cgi- |

| | | | | | | |
|---|---|---|---|---|---|---|
| | | | | | | bin/carddisp.pl?gene=CPQ |
| TRIM3 | Tripartite Motif Containing 3 | Protein Coding | 35 | GC11M006459 | 1.622046 | https://www.genecards.org/cgi-bin/carddisp.pl?gene=TRIM3 |
| NAA25 | N-Alpha-Acetyltransferase 25, NatB Auxiliary Subunit | Protein Coding | 34 | GC12M112026 | 1.620996 | https://www.genecards.org/cgi-bin/carddisp.pl?gene=NAA25 |
| TAB3 | TGF-Beta Activated Kinase 1 (MAP3K7) Binding Protein 3 | Protein Coding | 39 | GC0XM030901 | 1.620379 | https://www.genecards.org/cgi-bin/carddisp.pl?gene=TAB3 |
| LORICRIN | Loricrin Cornified Envelope Precursor Protein | Protein Coding | 27 | GC01P153262 | 1.61466 | https://www.genecards.org/cgi-bin/carddisp.pl?gene=LORICRIN |
| PGLYRP2 | Peptidoglycan Recognition Protein 2 | Protein Coding | 38 | GC19M015468 | 1.608178 | https://www.genecards.org/cgi-bin/carddisp.pl?gene=PGLYRP2 |
| SCG2 | Secretogranin II | Protein Coding | 39 | GC02M223596 | 1.607782 | https://www.genecards.org/cgi-bin/carddisp.pl?gene=SCG2 |
| STK4 | Serine/Threonine Kinase 4 | Protein Coding | 48 | GC20P044966 | 1.606022 | https://www.genecards.org/cgi-bin/carddisp.pl?gene=STK4 |
| MIR19A | MicroRNA 19a | RNA Gene | 20 | GC13P091554 | 1.604466 | https://www.genecards.org/cgi-bin/carddisp.pl?gene=MIR19A |
| RNPEPL1 | Arginyl Aminopeptidase Like 1 | Protein Coding | 35 | GC02P240565 | 1.604185 | https://www.genecards.org/cgi-bin/carddisp.pl?gene=RNPEPL1 |
| ID3 | Inhibitor Of DNA Binding 3, HLH Protein | Protein Coding | 40 | GC01M023557 | 1.60176 | https://www.genecards.org/cgi-bin/carddisp.pl?gene=ID3 |
| DPYSL5 | Dihydropyrimidinase Like 5 | Protein Coding | 39 | GC02P026847 | 1.601079 | https://www.genecards.org/cgi-bin/carddisp.pl?gene=DPYSL5 |
| ERCC6 | ERCC Excision Repair 6, Chromatin Remodeling Factor | Protein Coding | 45 | GC10M049454 | 1.600263 | https://www.genecards.org/cgi-bin/carddisp.pl?gene=ERCC6 |
| ITGA6 | Integrin Subunit Alpha 6 | Protein Coding | 48 | GC02P172427 | 1.594318 | https://www.genecards.org/cgi-bin/carddisp.pl?gene=ITGA6 |

| | | | | | | |
|---|---|---|---|---|---|---|
| COL4A1 | Collagen Type IV Alpha 1 Chain | Protein Coding | 46 | GC13M 110148 | 1.594 318 | https://www.genecards.org/cgi-bin/carddisp.pl?gene=COL4A1 |
| DRD1 | Dopamine Receptor D1 | Protein Coding | 43 | GC05M 175440 | 1.594 318 | https://www.genecards.org/cgi-bin/carddisp.pl?gene=DRD1 |
| DRD3 | Dopamine Receptor D3 | Protein Coding | 43 | GC03M 114128 | 1.594 318 | https://www.genecards.org/cgi-bin/carddisp.pl?gene=DRD3 |
| PSMB1 | Proteasome 20S Subunit Beta 1 | Protein Coding | 43 | GC06M 170535 | 1.594 318 | https://www.genecards.org/cgi-bin/carddisp.pl?gene=PSMB1 |
| FSCN1 | Fascin Actin-Bundling Protein 1 | Protein Coding | 42 | GC07P 005592 | 1.594 318 | https://www.genecards.org/cgi-bin/carddisp.pl?gene=FSCN1 |
| ORAI1 | ORAI Calcium Release-Activated Calcium Modulator 1 | Protein Coding | 42 | GC12P 126225 | 1.594 318 | https://www.genecards.org/cgi-bin/carddisp.pl?gene=ORAI1 |
| MRC1 | Mannose Receptor C-Type 1 | Protein Coding | 40 | GC10P 017809 | 1.594 318 | https://www.genecards.org/cgi-bin/carddisp.pl?gene=MRC1 |
| MRE11 | MRE11 Homolog, Double Strand Break Repair Nuclease | Protein Coding | 39 | GC11M 096553 | 1.594 318 | https://www.genecards.org/cgi-bin/carddisp.pl?gene=MRE11 |
| LSM4 | LSM4 Homolog, U6 Small Nuclear RNA And MRNA Degradation Associated | Protein Coding | 38 | GC19M 018306 | 1.594 318 | https://www.genecards.org/cgi-bin/carddisp.pl?gene=LSM4 |
| NLRC5 | NLR Family CARD Domain Containing 5 | Protein Coding | 38 | GC16P 057035 | 1.594 318 | https://www.genecards.org/cgi-bin/carddisp.pl?gene=NLRC5 |
| AFF1 | AF4/FMR2 Family Member 1 | Protein Coding | 37 | GC04P 086934 | 1.594 318 | https://www.genecards.org/cgi-bin/carddisp.pl?gene=AFF1 |
| CIRBP | Cold Inducible RNA Binding Protein | Protein Coding | 37 | GC19P 001259 | 1.594 318 | https://www.genecards.org/cgi-bin/carddisp.pl?gene=CIRBP |
| IKZF2 | IKAROS Family Zinc Finger 2 | Protein Coding | 37 | GC02M 213001 | 1.594 318 | https://www.genecards.org/cgi-bin/carddisp.pl?gene=IKZF2 |

| | | | | | | |
|---|---|---|---|---|---|---|
| MIR766 | MicroRNA 766 | RNA Gene | 18 | GC0X M1196 46 | 1.594 318 | https://www.genecards .org/cgi-bin/carddisp.pl?gene= MIR766 |
| MIR498 | MicroRNA 498 | RNA Gene | 17 | GC19P 067334 | 1.594 318 | https://www.genecards .org/cgi-bin/carddisp.pl?gene= MIR498 |
| MIR6089 | MicroRNA 6089 | RNA Gene | 8 | GC0XP 002632 | 1.594 318 | https://www.genecards .org/cgi-bin/carddisp.pl?gene= MIR6089 |
| DDC | Dopa Decarboxylase | Protein Coding | 49 | GC07M 050458 | 1.594 307 | https://www.genecards .org/cgi-bin/carddisp.pl?gene= DDC |
| MARCO | Macrophage Receptor With Collagenous Structure | Protein Coding | 40 | GC02P 118942 | 1.589 498 | https://www.genecards .org/cgi-bin/carddisp.pl?gene= MARCO |
| SNX9 | Sorting Nexin 9 | Protein Coding | 40 | GC06P 157685 | 1.588 394 | https://www.genecards .org/cgi-bin/carddisp.pl?gene= SNX9 |
| DSP | Desmoplakin | Protein Coding | 48 | GC06P 007541 | 1.587 361 | https://www.genecards .org/cgi-bin/carddisp.pl?gene= DSP |
| KLKB1 | Kallikrein B1 | Protein Coding | 46 | GC04P 186208 | 1.585 96 | https://www.genecards .org/cgi-bin/carddisp.pl?gene= KLKB1 |
| BLVRB | Biliverdin Reductase B | Protein Coding | 38 | GC19M 040447 | 1.585 96 | https://www.genecards .org/cgi-bin/carddisp.pl?gene= BLVRB |
| ZNF148 | Zinc Finger Protein 148 | Protein Coding | 40 | GC03M 125225 | 1.584 283 | https://www.genecards .org/cgi-bin/carddisp.pl?gene= ZNF148 |
| GADD45 B | Growth Arrest And DNA Damage Inducible Beta | Protein Coding | 36 | GC19P 002476 | 1.584 283 | https://www.genecards .org/cgi-bin/carddisp.pl?gene= GADD45B |
| MIR147B | MicroRNA 147b | RNA Gene | 19 | GC15P 045433 | 1.584 283 | https://www.genecards .org/cgi-bin/carddisp.pl?gene= MIR147B |
| ACVRL1 | Activin A Receptor Like Type 1 | Protein Coding | 48 | GC12P 051906 | 1.583 642 | https://www.genecards .org/cgi-bin/carddisp.pl?gene= ACVRL1 |
| ELF3 | E74 Like ETS Transcription Factor 3 | Protein Coding | 39 | GC01P 202007 | 1.582 903 | https://www.genecards .org/cgi- |

| | | | | | | |
|---|---|---|---|---|---|---|
| | | | | | | bin/carddisp.pl?gene=ELF3 |
| PIM2 | Pim-2 Proto-Oncogene, Serine/Threonine Kinase | Protein Coding | 42 | GC0XM048913 | 1.582481 | https://www.genecards.org/cgi-bin/carddisp.pl?gene=PIM2 |
| SNTB1 | Syntrophin Beta 1 | Protein Coding | 37 | GC08M120535 | 1.582481 | https://www.genecards.org/cgi-bin/carddisp.pl?gene=SNTB1 |
| P2RX3 | Purinergic Receptor P2X 3 | Protein Coding | 41 | GC11P057356 | 1.581138 | https://www.genecards.org/cgi-bin/carddisp.pl?gene=P2RX3 |
| MUC7 | Mucin 7, Secreted | Protein Coding | 36 | GC04P070430 | 1.579775 | https://www.genecards.org/cgi-bin/carddisp.pl?gene=MUC7 |
| HSD17B8 | Hydroxysteroid 17-Beta Dehydrogenase 8 | Protein Coding | 39 | GC06P083755 | 1.575986 | https://www.genecards.org/cgi-bin/carddisp.pl?gene=HSD17B8 |
| ATF6B | Activating Transcription Factor 6 Beta | Protein Coding | 37 | GC06M032115 | 1.575986 | https://www.genecards.org/cgi-bin/carddisp.pl?gene=ATF6B |
| HCG23 | HLA Complex Group 23 | RNA Gene | 16 | GC06P083724 | 1.575986 | https://www.genecards.org/cgi-bin/carddisp.pl?gene=HCG23 |
| ENSG00000250264 | Novel Protein, TAP2-HLA-DOB Readthrough | Protein Coding | 9 | GC06M066022 | 1.575986 | https://www.genecards.org/cgi-bin/carddisp.pl?gene=ENSG00000250264 |
| HSALNG0049451 | | RNA Gene | 4 | GC06P084618 | 1.575986 | https://www.genecards.org/cgi-bin/carddisp.pl?gene=HSALNG0049451 |
| lnc-PSMB9-14 | | RNA Gene | 4 | GC06P085131 | 1.575986 | https://www.genecards.org/cgi-bin/carddisp.pl?gene=lnc-PSMB9-14 |
| CABIN1 | Calcineurin Binding Protein 1 | Protein Coding | 41 | GC22P024011 | 1.574012 | https://www.genecards.org/cgi-bin/carddisp.pl?gene=CABIN1 |
| LAMC2 | Laminin Subunit Gamma 2 | Protein Coding | 45 | GC01P183186 | 1.572692 | https://www.genecards.org/cgi-bin/carddisp.pl?gene=LAMC2 |
| COL4A2 | Collagen Type IV Alpha 2 Chain | Protein Coding | 44 | GC13P110305 | 1.570701 | https://www.genecards.org/cgi-bin/carddisp.pl?gene=COL4A2 |

| | | | | | | |
|---|---|---|---|---|---|---|
| AGTR2 | Angiotensin II Receptor Type 2 | Protein Coding | 42 | GC0XP 116170 | 1.570 701 | https://www.genecards.org/cgi-bin/carddisp.pl?gene=AGTR2 |
| CFD | Complement Factor D | Protein Coding | 43 | GC19P 000859 | 1.569 983 | https://www.genecards.org/cgi-bin/carddisp.pl?gene=CFD |
| ACKR3 | Atypical Chemokine Receptor 3 | Protein Coding | 40 | GC02P 236537 | 1.567 161 | https://www.genecards.org/cgi-bin/carddisp.pl?gene=ACKR3 |
| PDIA3 | Protein Disulfide Isomerase Family A Member 3 | Protein Coding | 42 | GC15P 043746 | 1.565 365 | https://www.genecards.org/cgi-bin/carddisp.pl?gene=PDIA3 |
| RASGRP 2 | RAS Guanyl Releasing Protein 2 | Protein Coding | 42 | GC11M 064726 | 1.565 365 | https://www.genecards.org/cgi-bin/carddisp.pl?gene=RASGRP2 |
| GOT1 | Glutamic-Oxaloacetic Transaminase 1 | Protein Coding | 44 | GC10M 099396 | 1.563 368 | https://www.genecards.org/cgi-bin/carddisp.pl?gene=GOT1 |
| LINC014 75 | Long Intergenic Non-Protein Coding RNA 1475 | RNA Gene | 12 | GC10M 099526 | 1.563 368 | https://www.genecards.org/cgi-bin/carddisp.pl?gene=LINC01475 |
| EEF1B2P 6 | Eukaryotic Translation Elongation Factor 1 Beta 2 Pseudogene 6 | Pseudo gene | 7 | GC07M 131661 | 1.563 368 | https://www.genecards.org/cgi-bin/carddisp.pl?gene=EEF1B2P6 |
| MT2A | Metallothionein 2A | Protein Coding | 41 | GC16P 057038 | 1.562 4 | https://www.genecards.org/cgi-bin/carddisp.pl?gene=MT2A |
| CHI3L2 | Chitinase 3 Like 2 | Protein Coding | 37 | GC01P 111201 | 1.561 887 | https://www.genecards.org/cgi-bin/carddisp.pl?gene=CHI3L2 |
| PRDX2 | Peroxiredoxin 2 | Protein Coding | 44 | GC19M 012796 | 1.561 156 | https://www.genecards.org/cgi-bin/carddisp.pl?gene=PRDX2 |
| MT-CYB | Mitochondrially Encoded Cytochrome B | Protein Coding | 32 | GCMT P01474 9 | 1.560 21 | https://www.genecards.org/cgi-bin/carddisp.pl?gene=MT-CYB |
| MIR20A | MicroRNA 20a | RNA Gene | 20 | GC13P 091555 | 1.558 542 | https://www.genecards.org/cgi-bin/carddisp.pl?gene=MIR20A |
| CCL25 | C-C Motif Chemokine Ligand 25 | Protein Coding | 36 | GC19P 008052 | 1.558 183 | https://www.genecards.org/cgi- |

| | | | | | | |
|---|---|---|---|---|---|---|
| | | | | | | bin/carddisp.pl?gene=CCL25 |
| CENPA | Centromere Protein A | Protein Coding | 38 | GC02P 026765 | 1.556 901 | https://www.genecards.org/cgi-bin/carddisp.pl?gene=CENPA |
| EGR2 | Early Growth Response 2 | Protein Coding | 42 | GC10M 062811 | 1.553 344 | https://www.genecards.org/cgi-bin/carddisp.pl?gene=EGR2 |
| SUCNR1 | Succinate Receptor 1 | Protein Coding | 38 | GC03P 151873 | 1.548 542 | https://www.genecards.org/cgi-bin/carddisp.pl?gene=SUCNR1 |
| INAVA | Innate Immunity Activator | Protein Coding | 27 | GC01P 200892 | 1.546 315 | https://www.genecards.org/cgi-bin/carddisp.pl?gene=INAVA |
| IKZF1 | IKAROS Family Zinc Finger 1 | Protein Coding | 46 | GC07P 050303 | 1.543 654 | https://www.genecards.org/cgi-bin/carddisp.pl?gene=IKZF1 |
| HDAC6 | Histone Deacetylase 6 | Protein Coding | 50 | GC0XP 048801 | 1.541 989 | https://www.genecards.org/cgi-bin/carddisp.pl?gene=HDAC6 |
| IL25 | Interleukin 25 | Protein Coding | 36 | GC14P 032705 | 1.541 989 | https://www.genecards.org/cgi-bin/carddisp.pl?gene=IL25 |
| DERL3 | Derlin 3 | Protein Coding | 34 | GC22M 023834 | 1.541 989 | https://www.genecards.org/cgi-bin/carddisp.pl?gene=DERL3 |
| MIR106B | MicroRNA 106b | RNA Gene | 22 | GC07M 101892 | 1.541 989 | https://www.genecards.org/cgi-bin/carddisp.pl?gene=MIR106B |
| NPPC | Natriuretic Peptide C | Protein Coding | 41 | GC02M 231921 | 1.540 562 | https://www.genecards.org/cgi-bin/carddisp.pl?gene=NPPC |
| LILRA2 | Leukocyte Immunoglobulin Like Receptor A2 | Protein Coding | 40 | GC19P 054572 | 1.540 562 | https://www.genecards.org/cgi-bin/carddisp.pl?gene=LILRA2 |
| OIP5 | Opa Interacting Protein 5 | Protein Coding | 34 | GC15M 041309 | 1.540 562 | https://www.genecards.org/cgi-bin/carddisp.pl?gene=OIP5 |
| MIR138-2 | MicroRNA 138-2 | RNA Gene | 21 | GC16P 057067 | 1.540 562 | https://www.genecards.org/cgi-bin/carddisp.pl?gene=MIR138-2 |

| | | | | | | |
|---|---|---|---|---|---|---|
| MIR448 | MicroRNA 448 | RNA Gene | 13 | GC0XP 114823 | 1.540 562 | https://www.genecards .org/cgi- bin/carddisp.pl?gene= MIR448 |
| KISS1R | KISS1 Receptor | Protein Coding | 44 | GC19P 000917 | 1.533 888 | https://www.genecards .org/cgi- bin/carddisp.pl?gene= KISS1R |
| CRAT | Carnitine O- Acetyltransferase | Protein Coding | 44 | GC09M 129094 | 1.531 042 | https://www.genecards .org/cgi- bin/carddisp.pl?gene= CRAT |
| MFNG | MFNG O-Fucosylpeptide 3-Beta-N- Acetylglucosaminyltransfe rase | Protein Coding | 39 | GC22M 037469 | 1.530 903 | https://www.genecards .org/cgi- bin/carddisp.pl?gene= MFNG |
| CHID1 | Chitinase Domain Containing 1 | Protein Coding | 36 | GC11M 000867 | 1.527 948 | https://www.genecards .org/cgi- bin/carddisp.pl?gene= CHID1 |
| GSDME | Gasdermin E | Protein Coding | 31 | GC07M 024699 | 1.527 948 | https://www.genecards .org/cgi- bin/carddisp.pl?gene= GSDME |
| MC1R | Melanocortin 1 Receptor | Protein Coding | 45 | GC16P 089912 | 1.524 795 | https://www.genecards .org/cgi- bin/carddisp.pl?gene= MC1R |
| TARS1 | Threonyl-TRNA Synthetase 1 | Protein Coding | 36 | GC05P 033441 | 1.522 445 | https://www.genecards .org/cgi- bin/carddisp.pl?gene= TARS1 |
| PSMB7 | Proteasome 20S Subunit Beta 7 | Protein Coding | 44 | GC09M 124353 | 1.520 765 | https://www.genecards .org/cgi- bin/carddisp.pl?gene= PSMB7 |
| HMMR | Hyaluronan Mediated Motility Receptor | Protein Coding | 41 | GC05P 163480 | 1.520 765 | https://www.genecards .org/cgi- bin/carddisp.pl?gene= HMMR |
| GNLY | Granulysin | Protein Coding | 37 | GC02P 085685 | 1.520 765 | https://www.genecards .org/cgi- bin/carddisp.pl?gene= GNLY |
| ALCAM | Activated Leukocyte Cell Adhesion Molecule | Protein Coding | 42 | GC03P 105366 | 1.517 186 | https://www.genecards .org/cgi- bin/carddisp.pl?gene= ALCAM |
| A1BG | Alpha-1-B Glycoprotein | Protein Coding | 37 | GC19M 058345 | 1.517 186 | https://www.genecards .org/cgi- bin/carddisp.pl?gene= A1BG |
| DDIT3 | DNA Damage Inducible Transcript 3 | Protein Coding | 44 | GC12M 057516 | 1.516 357 | https://www.genecards .org/cgi- |

| | | | | | | |
|---|---|---|---|---|---|---|
| | | | | | | bin/carddisp.pl?gene=DDIT3 |
| HYAL2 | Hyaluronidase 2 | Protein Coding | 43 | GC03M050317 | 1.516357 | https://www.genecards.org/cgi-bin/carddisp.pl?gene=HYAL2 |
| CHAD | Chondroadherin | Protein Coding | 37 | GC17M050464 | 1.516357 | https://www.genecards.org/cgi-bin/carddisp.pl?gene=CHAD |
| IGH | Immunoglobulin Heavy Locus | Protein Coding | 18 | GC14M112706 | 1.509174 | https://www.genecards.org/cgi-bin/carddisp.pl?gene=IGH |
| ELMOD2 | ELMO Domain Containing 2 | Protein Coding | 35 | GC04P140524 | 1.508702 | https://www.genecards.org/cgi-bin/carddisp.pl?gene=ELMOD2 |
| SPPL2C | Signal Peptide Peptidase Like 2C | Protein Coding | 32 | GC17P045844 | 1.508702 | https://www.genecards.org/cgi-bin/carddisp.pl?gene=SPPL2C |
| SAG | S-Antigen Visual Arrestin | Protein Coding | 44 | GC02P233393 | 1.504816 | https://www.genecards.org/cgi-bin/carddisp.pl?gene=SAG |
| CTH | Cystathionine Gamma-Lyase | Protein Coding | 48 | GC01P070411 | 1.504572 | https://www.genecards.org/cgi-bin/carddisp.pl?gene=CTH |
| SLAMF8 | SLAM Family Member 8 | Protein Coding | 34 | GC01P159826 | 1.504572 | https://www.genecards.org/cgi-bin/carddisp.pl?gene=SLAMF8 |
| SLC6A3 | Solute Carrier Family 6 Member 3 | Protein Coding | 48 | GC05M001392 | 1.497591 | https://www.genecards.org/cgi-bin/carddisp.pl?gene=SLC6A3 |
| MAP4K3 | Mitogen-Activated Protein Kinase Kinase Kinase Kinase 3 | Protein Coding | 40 | GC02M039249 | 1.496403 | https://www.genecards.org/cgi-bin/carddisp.pl?gene=MAP4K3 |
| TRIM38 | Tripartite Motif Containing 38 | Protein Coding | 38 | GC06P025962 | 1.492981 | https://www.genecards.org/cgi-bin/carddisp.pl?gene=TRIM38 |
| MIR425 | MicroRNA 425 | RNA Gene | 20 | GC03M051161 | 1.492981 | https://www.genecards.org/cgi-bin/carddisp.pl?gene=MIR425 |
| MIR361 | MicroRNA 361 | RNA Gene | 17 | GC0XM085903 | 1.492981 | https://www.genecards.org/cgi-bin/carddisp.pl?gene=MIR361 |

| | | | | | | |
|---|---|---|---|---|---|---|
| DLG5 | Discs Large MAGUK Scaffold Protein 5 | Protein Coding | 38 | GC10M 077790 | 1.492 894 | https://www.genecards.org/cgi-bin/carddisp.pl?gene=DLG5 |
| LNPEP | Leucyl And Cystinyl Aminopeptidase | Protein Coding | 45 | GC05P 096935 | 1.492 114 | https://www.genecards.org/cgi-bin/carddisp.pl?gene=LNPEP |
| ATP8B2 | ATPase Phospholipid Transporting 8B2 | Protein Coding | 38 | GC01P 154325 | 1.492 114 | https://www.genecards.org/cgi-bin/carddisp.pl?gene=ATP8B2 |
| CLIC4 | Chloride Intracellular Channel 4 | Protein Coding | 38 | GC01P 024745 | 1.492 114 | https://www.genecards.org/cgi-bin/carddisp.pl?gene=CLIC4 |
| LINC023 41 | Long Intergenic Non-Protein Coding RNA 2341 | RNA Gene | 10 | GC13P 042339 | 1.492 114 | https://www.genecards.org/cgi-bin/carddisp.pl?gene=LINC02341 |
| NOS1 | Nitric Oxide Synthase 1 | Protein Coding | 48 | GC12M 117208 | 1.491 924 | https://www.genecards.org/cgi-bin/carddisp.pl?gene=NOS1 |
| ODC1 | Ornithine Decarboxylase 1 | Protein Coding | 46 | GC02M 010432 | 1.489 458 | https://www.genecards.org/cgi-bin/carddisp.pl?gene=ODC1 |
| ADAMT SL2 | ADAMTS Like 2 | Protein Coding | 41 | GC09P 134257 | 1.482 744 | https://www.genecards.org/cgi-bin/carddisp.pl?gene=ADAMTSL2 |
| RPL3P2 | Ribosomal Protein L3 Pseudogene 2 | Pseudo gene | 10 | GC06P 031280 | 1.481 575 | https://www.genecards.org/cgi-bin/carddisp.pl?gene=RPL3P2 |
| MAP1LC 3A | Microtubule Associated Protein 1 Light Chain 3 Alpha | Protein Coding | 40 | GC20P 034546 | 1.480 463 | https://www.genecards.org/cgi-bin/carddisp.pl?gene=MAP1LC3A |
| TRIM2 | Tripartite Motif Containing 2 | Protein Coding | 40 | GC04P 153152 | 1.480 463 | https://www.genecards.org/cgi-bin/carddisp.pl?gene=TRIM2 |
| BAIAP2L 1 | BAR/IMD Domain Containing Adaptor Protein 2 Like 1 | Protein Coding | 38 | GC07M 098294 | 1.480 463 | https://www.genecards.org/cgi-bin/carddisp.pl?gene=BAIAP2L1 |
| MAL | Mal, T Cell Differentiation Protein | Protein Coding | 36 | GC02P 095025 | 1.480 463 | https://www.genecards.org/cgi-bin/carddisp.pl?gene=MAL |
| YWHAQ | Tyrosine 3-Monooxygenase/Tryptoph | Protein Coding | 44 | GC02M 009583 | 1.478 126 | https://www.genecards.org/cgi- |

| | | | | | | |
|---|---|---|---|---|---|---|
| | an 5-Monooxygenase Activation Protein Theta | | | | | bin/carddisp.pl?gene=YWHAQ |
| NR1D1 | Nuclear Receptor Subfamily 1 Group D Member 1 | Protein Coding | 46 | GC17M040092 | 1.476844 | https://www.genecards.org/cgi-bin/carddisp.pl?gene=NR1D1 |
| CPA6 | Carboxypeptidase A6 | Protein Coding | 43 | GC08M067422 | 1.476844 | https://www.genecards.org/cgi-bin/carddisp.pl?gene=CPA6 |
| GDF2 | Growth Differentiation Factor 2 | Protein Coding | 42 | GC10P047322 | 1.476844 | https://www.genecards.org/cgi-bin/carddisp.pl?gene=GDF2 |
| OPRD1 | Opioid Receptor Delta 1 | Protein Coding | 42 | GC01P028812 | 1.476844 | https://www.genecards.org/cgi-bin/carddisp.pl?gene=OPRD1 |
| CEBPD | CCAAT Enhancer Binding Protein Delta | Protein Coding | 38 | GC08M047759 | 1.476844 | https://www.genecards.org/cgi-bin/carddisp.pl?gene=CEBPD |
| MIR7-1 | MicroRNA 7-1 | RNA Gene | 18 | GC09M093952 | 1.476844 | https://www.genecards.org/cgi-bin/carddisp.pl?gene=MIR7-1 |
| MIR7-3 | MicroRNA 7-3 | RNA Gene | 18 | GC19P004770 | 1.476844 | https://www.genecards.org/cgi-bin/carddisp.pl?gene=MIR7-3 |
| MIR7-2 | MicroRNA 7-2 | RNA Gene | 17 | GC15P088611 | 1.476844 | https://www.genecards.org/cgi-bin/carddisp.pl?gene=MIR7-2 |
| SCG5 | Secretogranin V | Protein Coding | 37 | GC15P032641 | 1.474648 | https://www.genecards.org/cgi-bin/carddisp.pl?gene=SCG5 |
| PYY | Peptide YY | Protein Coding | 42 | GC17M043952 | 1.468681 | https://www.genecards.org/cgi-bin/carddisp.pl?gene=PYY |
| ADGRE2 | Adhesion G Protein-Coupled Receptor E2 | Protein Coding | 39 | GC19M014733 | 1.46716 | https://www.genecards.org/cgi-bin/carddisp.pl?gene=ADGRE2 |
| LRP4 | LDL Receptor Related Protein 4 | Protein Coding | 43 | GC11M089351 | 1.466792 | https://www.genecards.org/cgi-bin/carddisp.pl?gene=LRP4 |
| CD1C | CD1c Molecule | Protein Coding | 38 | GC01P158289 | 1.466792 | https://www.genecards.org/cgi-bin/carddisp.pl?gene=CD1C |

| | | | | | | |
|---|---|---|---|---|---|---|
| CD1E | CD1e Molecule | Protein Coding | 38 | GC01P 158354 | 1.466 792 | https://www.genecards.org/cgi-bin/carddisp.pl?gene=CD1E |
| CD1B | CD1b Molecule | Protein Coding | 37 | GC01M 158297 | 1.466 792 | https://www.genecards.org/cgi-bin/carddisp.pl?gene=CD1B |
| PSMB11 | Proteasome Subunit Beta 11 | Protein Coding | 32 | GC14P 033245 | 1.466 792 | https://www.genecards.org/cgi-bin/carddisp.pl?gene=PSMB11 |
| LDHB | Lactate Dehydrogenase B | Protein Coding | 45 | GC12M 021635 | 1.465 253 | https://www.genecards.org/cgi-bin/carddisp.pl?gene=LDHB |
| LOXL2 | Lysyl Oxidase Like 2 | Protein Coding | 45 | GC08M 023296 | 1.465 253 | https://www.genecards.org/cgi-bin/carddisp.pl?gene=LOXL2 |
| GDF11 | Growth Differentiation Factor 11 | Protein Coding | 41 | GC12P 055743 | 1.465 253 | https://www.genecards.org/cgi-bin/carddisp.pl?gene=GDF11 |
| PIK3C2A | Phosphatidylinositol-4-Phosphate 3-Kinase Catalytic Subunit Type 2 Alpha | Protein Coding | 45 | GC11M 017580 | 1.464 933 | https://www.genecards.org/cgi-bin/carddisp.pl?gene=PIK3C2A |
| OSMR | Oncostatin M Receptor | Protein Coding | 44 | GC05P 038845 | 1.464 27 | https://www.genecards.org/cgi-bin/carddisp.pl?gene=OSMR |
| CKLF | Chemokine Like Factor | Protein Coding | 37 | GC16P 066552 | 1.464 27 | https://www.genecards.org/cgi-bin/carddisp.pl?gene=CKLF |
| STAMBP | STAM Binding Protein | Protein Coding | 43 | GC02P 073828 | 1.463 375 | https://www.genecards.org/cgi-bin/carddisp.pl?gene=STAMBP |
| H2BC21 | H2B Clustered Histone 21 | Protein Coding | 30 | GC01M 152029 | 1.460 906 | https://www.genecards.org/cgi-bin/carddisp.pl?gene=H2BC21 |
| RAPGEF 3 | Rap Guanine Nucleotide Exchange Factor 3 | Protein Coding | 43 | GC12M 047736 | 1.457 924 | https://www.genecards.org/cgi-bin/carddisp.pl?gene=RAPGEF3 |
| LY6G5B | Lymphocyte Antigen 6 Family Member G5B | Protein Coding | 30 | GC06P 083701 | 1.457 831 | https://www.genecards.org/cgi-bin/carddisp.pl?gene=LY6G5B |
| FMR1 | Fragile X Messenger Ribonucleoprotein 1 | Protein Coding | 42 | GC0XP 147943 | 1.456 519 | https://www.genecards.org/cgi- |

| | | | | | | |
|---|---|---|---|---|---|---|
| | | | | | | bin/carddisp.pl?gene=FMR1 |
| SIGLEC5 | Sialic Acid Binding Ig Like Lectin 5 | Protein Coding | 38 | GC19M066230 | 1.453637 | https://www.genecards.org/cgi-bin/carddisp.pl?gene=SIGLEC5 |
| UROD | Uroporphyrinogen Decarboxylase | Protein Coding | 44 | GC01P045353 | 1.450618 | https://www.genecards.org/cgi-bin/carddisp.pl?gene=UROD |
| SAA3P | Serum Amyloid A3, Pseudogene | Pseudo gene | 16 | GC11M018112 | 1.450228 | https://www.genecards.org/cgi-bin/carddisp.pl?gene=SAA3P |
| QDPR | Quinoid Dihydropteridine Reductase | Protein Coding | 45 | GC04M017460 | 1.443045 | https://www.genecards.org/cgi-bin/carddisp.pl?gene=QDPR |
| VSTM1 | V-Set And Transmembrane Domain Containing 1 | Protein Coding | 32 | GC19M054040 | 1.443045 | https://www.genecards.org/cgi-bin/carddisp.pl?gene=VSTM1 |
| MOGS | Mannosyl-Oligosaccharide Glucosidase | Protein Coding | 41 | GC02M074461 | 1.441766 | https://www.genecards.org/cgi-bin/carddisp.pl?gene=MOGS |
| CDKN2B | Cyclin Dependent Kinase Inhibitor 2B | Protein Coding | 45 | GC09M022002 | 1.441158 | https://www.genecards.org/cgi-bin/carddisp.pl?gene=CDKN2B |
| OPRK1 | Opioid Receptor Kappa 1 | Protein Coding | 44 | GC08M053227 | 1.440708 | https://www.genecards.org/cgi-bin/carddisp.pl?gene=OPRK1 |
| NCR2 | Natural Cytotoxicity Triggering Receptor 2 | Protein Coding | 38 | GC06P083892 | 1.440708 | https://www.genecards.org/cgi-bin/carddisp.pl?gene=NCR2 |
| MMD | Monocyte To Macrophage Differentiation Associated | Protein Coding | 33 | GC17M055392 | 1.440708 | https://www.genecards.org/cgi-bin/carddisp.pl?gene=MMD |
| DUSP5 | Dual Specificity Phosphatase 5 | Protein Coding | 40 | GC10P110497 | 1.436743 | https://www.genecards.org/cgi-bin/carddisp.pl?gene=DUSP5 |
| DYRK1A | Dual Specificity Tyrosine Phosphorylation Regulated Kinase 1A | Protein Coding | 49 | GC21P037365 | 1.436542 | https://www.genecards.org/cgi-bin/carddisp.pl?gene=DYRK1A |
| PRKCA | Protein Kinase C Alpha | Protein Coding | 48 | GC17P066302 | 1.436542 | https://www.genecards.org/cgi-bin/carddisp.pl?gene=PRKCA |

| | | | | | | |
|---|---|---|---|---|---|---|
| RARB | Retinoic Acid Receptor Beta | Protein Coding | 48 | GC03P 024689 | 1.436 542 | https://www.genecards .org/cgi-bin/carddisp.pl?gene= RARB |
| EIF4G1 | Eukaryotic Translation Initiation Factor 4 Gamma 1 | Protein Coding | 45 | GC03P 184314 | 1.436 542 | https://www.genecards .org/cgi-bin/carddisp.pl?gene= EIF4G1 |
| PKM | Pyruvate Kinase M1/2 | Protein Coding | 44 | GC15M 072199 | 1.436 542 | https://www.genecards .org/cgi-bin/carddisp.pl?gene= PKM |
| USP5 | Ubiquitin Specific Peptidase 5 | Protein Coding | 44 | GC12P 021073 | 1.436 542 | https://www.genecards .org/cgi-bin/carddisp.pl?gene= USP5 |
| KPNA2 | Karyopherin Subunit Alpha 2 | Protein Coding | 43 | GC17P 068035 | 1.436 542 | https://www.genecards .org/cgi-bin/carddisp.pl?gene= KPNA2 |
| PRMT5 | Protein Arginine Methyltransferase 5 | Protein Coding | 43 | GC14M 022920 | 1.436 542 | https://www.genecards .org/cgi-bin/carddisp.pl?gene= PRMT5 |
| CNP | 2',3'-Cyclic Nucleotide 3' Phosphodiesterase | Protein Coding | 42 | GC17P 041966 | 1.436 542 | https://www.genecards .org/cgi-bin/carddisp.pl?gene= CNP |
| TRIM32 | Tripartite Motif Containing 32 | Protein Coding | 42 | GC09P 116687 | 1.436 542 | https://www.genecards .org/cgi-bin/carddisp.pl?gene= TRIM32 |
| CD63 | CD63 Molecule | Protein Coding | 41 | GC12M 055725 | 1.436 542 | https://www.genecards .org/cgi-bin/carddisp.pl?gene= CD63 |
| GRK5 | G Protein-Coupled Receptor Kinase 5 | Protein Coding | 41 | GC10P 119207 | 1.436 542 | https://www.genecards .org/cgi-bin/carddisp.pl?gene= GRK5 |
| KHDRBS 1 | KH RNA Binding Domain Containing, Signal Transduction Associated 1 | Protein Coding | 41 | GC01P 032013 | 1.436 542 | https://www.genecards .org/cgi-bin/carddisp.pl?gene= KHDRBS1 |
| KLF10 | Kruppel Like Factor 10 | Protein Coding | 41 | GC08M 102648 | 1.436 542 | https://www.genecards .org/cgi-bin/carddisp.pl?gene= KLF10 |
| PCBP1 | Poly(RC) Binding Protein 1 | Protein Coding | 41 | GC02P 070087 | 1.436 542 | https://www.genecards .org/cgi-bin/carddisp.pl?gene= PCBP1 |
| S100A6 | S100 Calcium Binding Protein A6 | Protein Coding | 41 | GC01M 153559 | 1.436 542 | https://www.genecards .org/cgi- |

| | | | | | | |
|---|---|---|---|---|---|---|
| | | | | | | bin/carddisp.pl?gene=S100A6 |
| SPRY1 | Sprouty RTK Signaling Antagonist 1 | Protein Coding | 41 | GC04P123396 | 1.436542 | https://www.genecards.org/cgi-bin/carddisp.pl?gene=SPRY1 |
| STAB1 | Stabilin 1 | Protein Coding | 41 | GC03P052495 | 1.436542 | https://www.genecards.org/cgi-bin/carddisp.pl?gene=STAB1 |
| WNT9A | Wnt Family Member 9A | Protein Coding | 41 | GC01M227920 | 1.436542 | https://www.genecards.org/cgi-bin/carddisp.pl?gene=WNT9A |
| BCL3 | BCL3 Transcription Coactivator | Protein Coding | 40 | GC19P044747 | 1.436542 | https://www.genecards.org/cgi-bin/carddisp.pl?gene=BCL3 |
| CLCF1 | Cardiotrophin Like Cytokine Factor 1 | Protein Coding | 40 | GC11M067364 | 1.436542 | https://www.genecards.org/cgi-bin/carddisp.pl?gene=CLCF1 |
| IRAK2 | Interleukin 1 Receptor Associated Kinase 2 | Protein Coding | 39 | GC03P012457 | 1.436542 | https://www.genecards.org/cgi-bin/carddisp.pl?gene=IRAK2 |
| SERPINB1 | Serpin Family B Member 1 | Protein Coding | 39 | GC06M002835 | 1.436542 | https://www.genecards.org/cgi-bin/carddisp.pl?gene=SERPINB1 |
| CD53 | CD53 Molecule | Protein Coding | 38 | GC01P110871 | 1.436542 | https://www.genecards.org/cgi-bin/carddisp.pl?gene=CD53 |
| MAST3 | Microtubule Associated Serine/Threonine Kinase 3 | Protein Coding | 38 | GC19P066241 | 1.436542 | https://www.genecards.org/cgi-bin/carddisp.pl?gene=MAST3 |
| MT3 | Metallothionein 3 | Protein Coding | 38 | GC16P056589 | 1.436542 | https://www.genecards.org/cgi-bin/carddisp.pl?gene=MT3 |
| OXA1L | OXA1L Mitochondrial Inner Membrane Protein | Protein Coding | 38 | GC14P022766 | 1.436542 | https://www.genecards.org/cgi-bin/carddisp.pl?gene=OXA1L |
| PDLIM2 | PDZ And LIM Domain 2 | Protein Coding | 37 | GC08P022578 | 1.436542 | https://www.genecards.org/cgi-bin/carddisp.pl?gene=PDLIM2 |
| SNAPIN | SNAP Associated Protein | Protein Coding | 37 | GC01P153660 | 1.436542 | https://www.genecards.org/cgi-bin/carddisp.pl?gene=SNAPIN |

| | | | | | | |
|---|---|---|---|---|---|---|
| ORM2 | Orosomucoid 2 | Protein Coding | 36 | GC09P 114329 | 1.436 542 | https://www.genecards.org/cgi-bin/carddisp.pl?gene=ORM2 |
| STXBP6 | Syntaxin Binding Protein 6 | Protein Coding | 36 | GC14M 024812 | 1.436 542 | https://www.genecards.org/cgi-bin/carddisp.pl?gene=STXBP6 |
| KLLN | Killin, P53 Regulated DNA Replication Inhibitor | Protein Coding | 29 | GC10M 087867 | 1.436 542 | https://www.genecards.org/cgi-bin/carddisp.pl?gene=KLLN |
| TUG1 | Taurine Up-Regulated 1 | Protein Coding | 22 | GC22P 030969 | 1.436 542 | https://www.genecards.org/cgi-bin/carddisp.pl?gene=TUG1 |
| MIR92A1 | MicroRNA 92a-1 | RNA Gene | 19 | GC13P 091558 | 1.436 542 | https://www.genecards.org/cgi-bin/carddisp.pl?gene=MIR92A1 |
| MIR421 | MicroRNA 421 | RNA Gene | 16 | GC0X M0742 18 | 1.436 542 | https://www.genecards.org/cgi-bin/carddisp.pl?gene=MIR421 |
| MIR613 | MicroRNA 613 | RNA Gene | 14 | GC12P 021342 | 1.436 542 | https://www.genecards.org/cgi-bin/carddisp.pl?gene=MIR613 |
| MIR4701 | MicroRNA 4701 | RNA Gene | 12 | GC12M 049616 | 1.436 542 | https://www.genecards.org/cgi-bin/carddisp.pl?gene=MIR4701 |
| NR1I3 | Nuclear Receptor Subfamily 1 Group I Member 3 | Protein Coding | 44 | GC01M 161229 | 1.431 934 | https://www.genecards.org/cgi-bin/carddisp.pl?gene=NR1I3 |
| TGFBI | Transforming Growth Factor Beta Induced | Protein Coding | 45 | GC05P 136027 | 1.429 118 | https://www.genecards.org/cgi-bin/carddisp.pl?gene=TGFBI |
| SAT1 | Spermidine/Spermine N1-Acetyltransferase 1 | Protein Coding | 42 | GC0XP 023784 | 1.429 118 | https://www.genecards.org/cgi-bin/carddisp.pl?gene=SAT1 |
| FSTL3 | Follistatin Like 3 | Protein Coding | 39 | GC19P 000676 | 1.429 118 | https://www.genecards.org/cgi-bin/carddisp.pl?gene=FSTL3 |
| IFNLR1 | Interferon Lambda Receptor 1 | Protein Coding | 34 | GC01M 025645 | 1.429 118 | https://www.genecards.org/cgi-bin/carddisp.pl?gene=IFNLR1 |
| MIR331 | MicroRNA 331 | RNA Gene | 20 | GC12P 095308 | 1.429 118 | https://www.genecards.org/cgi- |

| | | | | | | |
|---|---|---|---|---|---|---|
| | | | | | | bin/carddisp.pl?gene=MIR331 |
| FOXD2-AS1 | FOXD2 Adjacent Opposite Strand RNA 1 | RNA Gene | 16 | GC01M047432 | 1.429118 | https://www.genecards.org/cgi-bin/carddisp.pl?gene=FOXD2-AS1 |
| NTRK3 | Neurotrophic Receptor Tyrosine Kinase 3 | Protein Coding | 50 | GC15M087859 | 1.426852 | https://www.genecards.org/cgi-bin/carddisp.pl?gene=NTRK3 |
| NTF4 | Neurotrophin 4 | Protein Coding | 42 | GC19M066077 | 1.426852 | https://www.genecards.org/cgi-bin/carddisp.pl?gene=NTF4 |
| F11R | F11 Receptor | Protein Coding | 41 | GC01M160995 | 1.426852 | https://www.genecards.org/cgi-bin/carddisp.pl?gene=F11R |
| RMRP | RNA Component Of Mitochondrial RNA Processing Endoribonuclease | RNA Gene | 25 | GC09M035655 | 1.426852 | https://www.genecards.org/cgi-bin/carddisp.pl?gene=RMRP |
| PEX5 | Peroxisomal Biogenesis Factor 5 | Protein Coding | 42 | GC12P021103 | 1.42668 | https://www.genecards.org/cgi-bin/carddisp.pl?gene=PEX5 |
| GSDMC | Gasdermin C | Protein Coding | 33 | GC08M129705 | 1.425374 | https://www.genecards.org/cgi-bin/carddisp.pl?gene=GSDMC |
| APCS | Amyloid P Component, Serum | Protein Coding | 42 | GC01P159587 | 1.425147 | https://www.genecards.org/cgi-bin/carddisp.pl?gene=APCS |
| RXRB | Retinoid X Receptor Beta | Protein Coding | 45 | GC06M033193 | 1.421127 | https://www.genecards.org/cgi-bin/carddisp.pl?gene=RXRB |
| AGPAT1 | 1-Acylglycerol-3-Phosphate O-Acyltransferase 1 | Protein Coding | 40 | GC06M032168 | 1.421127 | https://www.genecards.org/cgi-bin/carddisp.pl?gene=AGPAT1 |
| VPS52 | VPS52 Subunit Of GARP Complex | Protein Coding | 34 | GC06M066055 | 1.421127 | https://www.genecards.org/cgi-bin/carddisp.pl?gene=VPS52 |
| HSALNG0049404 | | RNA Gene | 5 | GC06M066657 | 1.421127 | https://www.genecards.org/cgi-bin/carddisp.pl?gene=HSALNG0049404 |
| HSALNG0049429 | | RNA Gene | 5 | GC06P084613 | 1.421127 | https://www.genecards.org/cgi-bin/carddisp.pl?gene=HSALNG0049429 |

| | | | | | | |
|---|---|---|---|---|---|---|
| lnc-HLA-DRB1-8 | | RNA Gene | 5 | GC06M 032619 | 1.421 127 | https://www.genecards .org/cgi-bin/carddisp.pl?gene=l nc-HLA-DRB1-8 |
| HSALNG0049424 | | RNA Gene | 4 | GC06M 066659 | 1.421 127 | https://www.genecards .org/cgi-bin/carddisp.pl?gene= HSALNG0049424 |
| HSALNG0049427 | | RNA Gene | 4 | GC06P 084612 | 1.421 127 | https://www.genecards .org/cgi-bin/carddisp.pl?gene= HSALNG0049427 |
| HSALNG0049428 | | RNA Gene | 4 | GC06M 066660 | 1.421 127 | https://www.genecards .org/cgi-bin/carddisp.pl?gene= HSALNG0049428 |
| RF00017-5040 | | RNA Gene | 4 | GC06M 068718 | 1.421 127 | https://www.genecards .org/cgi-bin/carddisp.pl?gene= RF00017-5040 |
| lnc-HLA-DOB-1 | | RNA Gene | 4 | GC06M 032825 | 1.421 127 | https://www.genecards .org/cgi-bin/carddisp.pl?gene=l nc-HLA-DOB-1 |
| piR-33103-025 | | RNA Gene | 2 | GC06P 086003 | 1.421 127 | https://www.genecards .org/cgi-bin/carddisp.pl?gene= piR-33103-025 |
| piR-35002-057 | | RNA Gene | 2 | GC06P 086099 | 1.421 127 | https://www.genecards .org/cgi-bin/carddisp.pl?gene= piR-35002-057 |
| piR-39858-769 | | RNA Gene | 2 | GC06M 068187 | 1.421 127 | https://www.genecards .org/cgi-bin/carddisp.pl?gene= piR-39858-769 |
| ADK | Adenosine Kinase | Protein Coding | 49 | GC10P 074155 | 1.420 507 | https://www.genecards .org/cgi-bin/carddisp.pl?gene= ADK |
| ITK | IL2 Inducible T Cell Kinase | Protein Coding | 50 | GC05P 157158 | 1.418 892 | https://www.genecards .org/cgi-bin/carddisp.pl?gene=I TK |
| GRHPR | Glyoxylate And Hydroxypyruvate Reductase | Protein Coding | 45 | GC09P 047213 | 1.418 892 | https://www.genecards .org/cgi-bin/carddisp.pl?gene= GRHPR |
| BLZF1 | Basic Leucine Zipper Nuclear Factor 1 | Protein Coding | 38 | GC01P 169367 | 1.418 892 | https://www.genecards .org/cgi-bin/carddisp.pl?gene= BLZF1 |
| SYNJ2BP | Synaptojanin 2 Binding Protein | Protein Coding | 36 | GC14M 070366 | 1.418 892 | https://www.genecards .org/cgi- |

| | | | | | | |
|---|---|---|---|---|---|---|
| | | | | | | bin/carddisp.pl?gene=SYNJ2BP |
| MIR1238 | MicroRNA 1238 | RNA Gene | 15 | GC19P010552 | 1.418892 | https://www.genecards.org/cgi-bin/carddisp.pl?gene=MIR1238 |
| MIR6069 | MicroRNA 6069 | RNA Gene | 9 | GC22M035336 | 1.418892 | https://www.genecards.org/cgi-bin/carddisp.pl?gene=MIR6069 |
| TSSK1B | Testis Specific Serine Kinase 1B | Protein Coding | 37 | GC05M113432 | 1.416734 | https://www.genecards.org/cgi-bin/carddisp.pl?gene=TSSK1B |
| TSIX | TSIX Transcript, XIST Antisense RNA | RNA Gene | 17 | GC0XP073792 | 1.416734 | https://www.genecards.org/cgi-bin/carddisp.pl?gene=TSIX |
| ANG | Angiogenin | Protein Coding | 44 | GC14P032461 | 1.416694 | https://www.genecards.org/cgi-bin/carddisp.pl?gene=ANG |
| CDKN2C | Cyclin Dependent Kinase Inhibitor 2C | Protein Coding | 43 | GC01P050960 | 1.416056 | https://www.genecards.org/cgi-bin/carddisp.pl?gene=CDKN2C |
| CSTA | Cystatin A | Protein Coding | 43 | GC03P122325 | 1.414482 | https://www.genecards.org/cgi-bin/carddisp.pl?gene=CSTA |
| ROM1 | Retinal Outer Segment Membrane Protein 1 | Protein Coding | 42 | GC11P062611 | 1.405944 | https://www.genecards.org/cgi-bin/carddisp.pl?gene=ROM1 |
| CLOCK | Clock Circadian Regulator | Protein Coding | 42 | GC04M055427 | 1.405062 | https://www.genecards.org/cgi-bin/carddisp.pl?gene=CLOCK |
| TRPC5 | Transient Receptor Potential Cation Channel Subfamily C Member 5 | Protein Coding | 41 | GC0XM111774 | 1.401049 | https://www.genecards.org/cgi-bin/carddisp.pl?gene=TRPC5 |
| GSTM3 | Glutathione S-Transferase Mu 3 | Protein Coding | 45 | GC01M109733 | 1.400406 | https://www.genecards.org/cgi-bin/carddisp.pl?gene=GSTM3 |
| MBD4 | Methyl-CpG Binding Domain 4, DNA Glycosylase | Protein Coding | 41 | GC03M129430 | 1.400406 | https://www.genecards.org/cgi-bin/carddisp.pl?gene=MBD4 |
| EPC1 | Enhancer Of Polycomb Homolog 1 | Protein Coding | 38 | GC10M033099 | 1.400406 | https://www.genecards.org/cgi-bin/carddisp.pl?gene=EPC1 |

| | | | | | | |
|---|---|---|---|---|---|---|
| LILRA5 | Leukocyte Immunoglobulin Like Receptor A5 | Protein Coding | 37 | GC19M054307 | 1.400406 | https://www.genecards.org/cgi-bin/carddisp.pl?gene=LILRA5 |
| ENGASE | Endo-Beta-N-Acetylglucosaminidase | Protein Coding | 34 | GC17P079071 | 1.400406 | https://www.genecards.org/cgi-bin/carddisp.pl?gene=ENGASE |
| LEAP2 | Liver Enriched Antimicrobial Peptide 2 | Protein Coding | 32 | GC05P132872 | 1.400406 | https://www.genecards.org/cgi-bin/carddisp.pl?gene=LEAP2 |
| MIR22HG | MIR22 Host Gene | RNA Gene | 26 | GC17M004360 | 1.400406 | https://www.genecards.org/cgi-bin/carddisp.pl?gene=MIR22HG |
| LINC00305 | Long Intergenic Non-Protein Coding RNA 305 | RNA Gene | 20 | GC18M064080 | 1.400406 | https://www.genecards.org/cgi-bin/carddisp.pl?gene=LINC00305 |
| MPZ | Myelin Protein Zero | Protein Coding | 44 | GC01M161304 | 1.399909 | https://www.genecards.org/cgi-bin/carddisp.pl?gene=MPZ |
| LYN | LYN Proto-Oncogene, Src Family Tyrosine Kinase | Protein Coding | 46 | GC08P055879 | 1.399825 | https://www.genecards.org/cgi-bin/carddisp.pl?gene=LYN |
| MSH6 | MutS Homolog 6 | Protein Coding | 48 | GC02P047695 | 1.399124 | https://www.genecards.org/cgi-bin/carddisp.pl?gene=MSH6 |
| ACTG1 | Actin Gamma 1 | Protein Coding | 47 | GC17M081509 | 1.399124 | https://www.genecards.org/cgi-bin/carddisp.pl?gene=ACTG1 |
| MAP3K11 | Mitogen-Activated Protein Kinase Kinase Kinase 11 | Protein Coding | 45 | GC11M089796 | 1.399124 | https://www.genecards.org/cgi-bin/carddisp.pl?gene=MAP3K11 |
| DVL2 | Dishevelled Segment Polarity Protein 2 | Protein Coding | 44 | GC17M007225 | 1.399124 | https://www.genecards.org/cgi-bin/carddisp.pl?gene=DVL2 |
| PTPRS | Protein Tyrosine Phosphatase Receptor Type S | Protein Coding | 44 | GC19M005495 | 1.399124 | https://www.genecards.org/cgi-bin/carddisp.pl?gene=PTPRS |
| NDUFA13 | NADH:Ubiquinone Oxidoreductase Subunit A13 | Protein Coding | 43 | GC19P019515 | 1.399124 | https://www.genecards.org/cgi-bin/carddisp.pl?gene=NDUFA13 |
| CASP5 | Caspase 5 | Protein Coding | 42 | GC11M104995 | 1.399124 | https://www.genecards.org/cgi- |

| | | | | | | |
|---|---|---|---|---|---|---|
| | | | | | | bin/carddisp.pl?gene=CASP5 |
| MAP3K4 | Mitogen-Activated Protein Kinase Kinase Kinase 4 | Protein Coding | 42 | GC06P160991 | 1.399124 | https://www.genecards.org/cgi-bin/carddisp.pl?gene=MAP3K4 |
| PPM1A | Protein Phosphatase, Mg2+/Mn2+ Dependent 1A | Protein Coding | 42 | GC14P060245 | 1.399124 | https://www.genecards.org/cgi-bin/carddisp.pl?gene=PPM1A |
| KMT2C | Lysine Methyltransferase 2C | Protein Coding | 41 | GC07M152134 | 1.399124 | https://www.genecards.org/cgi-bin/carddisp.pl?gene=KMT2C |
| KLK11 | Kallikrein Related Peptidase 11 | Protein Coding | 40 | GC19M066201 | 1.399124 | https://www.genecards.org/cgi-bin/carddisp.pl?gene=KLK11 |
| NLRP2 | NLR Family Pyrin Domain Containing 2 | Protein Coding | 40 | GC19P054953 | 1.399124 | https://www.genecards.org/cgi-bin/carddisp.pl?gene=NLRP2 |
| PPP1R15A | Protein Phosphatase 1 Regulatory Subunit 15A | Protein Coding | 38 | GC19P048872 | 1.399124 | https://www.genecards.org/cgi-bin/carddisp.pl?gene=PPP1R15A |
| TNIP3 | TNFAIP3 Interacting Protein 3 | Protein Coding | 36 | GC04M121131 | 1.399124 | https://www.genecards.org/cgi-bin/carddisp.pl?gene=TNIP3 |
| TNFAIP8L2 | TNF Alpha Induced Protein 8 Like 2 | Protein Coding | 33 | GC01P151156 | 1.399124 | https://www.genecards.org/cgi-bin/carddisp.pl?gene=TNFAIP8L2 |
| MIRLET7C | MicroRNA Let-7c | RNA Gene | 22 | GC21P016551 | 1.399124 | https://www.genecards.org/cgi-bin/carddisp.pl?gene=MIRLET7C |
| TIMP4 | TIMP Metallopeptidase Inhibitor 4 | Protein Coding | 38 | GC03M012153 | 1.398468 | https://www.genecards.org/cgi-bin/carddisp.pl?gene=TIMP4 |
| COL4A5 | Collagen Type IV Alpha 5 Chain | Protein Coding | 43 | GC0XP108439 | 1.397666 | https://www.genecards.org/cgi-bin/carddisp.pl?gene=COL4A5 |
| CEACAM5 | CEA Cell Adhesion Molecule 5 | Protein Coding | 42 | GC19P066761 | 1.393224 | https://www.genecards.org/cgi-bin/carddisp.pl?gene=CEACAM5 |
| MUC16 | Mucin 16, Cell Surface Associated | Protein Coding | 37 | GC19M008848 | 1.393224 | https://www.genecards.org/cgi-bin/carddisp.pl?gene=MUC16 |

| | | | | | | |
|---|---|---|---|---|---|---|
| ACHE | Acetylcholinesterase (Cartwright Blood Group) | Protein Coding | 44 | GC07M 100889 | 1.393 109 | https://www.genecards .org/cgi-bin/carddisp.pl?gene= ACHE |
| ESRRA | Estrogen Related Receptor Alpha | Protein Coding | 46 | GC11P 064305 | 1.389 456 | https://www.genecards .org/cgi-bin/carddisp.pl?gene= ESRRA |
| GRK2 | G Protein-Coupled Receptor Kinase 2 | Protein Coding | 36 | GC11P 067266 | 1.386 07 | https://www.genecards .org/cgi-bin/carddisp.pl?gene= GRK2 |
| MDC1 | Mediator Of DNA Damage Checkpoint 1 | Protein Coding | 40 | GC06M 065881 | 1.384 277 | https://www.genecards .org/cgi-bin/carddisp.pl?gene= MDC1 |
| CACNA1 I | Calcium Voltage-Gated Channel Subunit Alpha1 I | Protein Coding | 44 | GC22P 039570 | 1.382 956 | https://www.genecards .org/cgi-bin/carddisp.pl?gene= CACNA1I |
| STAB2 | Stabilin 2 | Protein Coding | 41 | GC12P 103587 | 1.382 956 | https://www.genecards .org/cgi-bin/carddisp.pl?gene= STAB2 |
| CSMD1 | CUB And Sushi Multiple Domains 1 | Protein Coding | 38 | GC08M 002953 | 1.382 956 | https://www.genecards .org/cgi-bin/carddisp.pl?gene= CSMD1 |
| TOR1A | Torsin Family 1 Member A | Protein Coding | 44 | GC09M 129812 | 1.382 589 | https://www.genecards .org/cgi-bin/carddisp.pl?gene= TOR1A |
| MAN2A1 | Mannosidase Alpha Class 2A Member 1 | Protein Coding | 42 | GC05P 109689 | 1.381 862 | https://www.genecards .org/cgi-bin/carddisp.pl?gene= MAN2A1 |
| CAPN1 | Calpain 1 | Protein Coding | 48 | GC11P 069958 | 1.379 053 | https://www.genecards .org/cgi-bin/carddisp.pl?gene= CAPN1 |
| ABCB7 | ATP Binding Cassette Subfamily B Member 7 | Protein Coding | 43 | GC0X M0750 53 | 1.378 355 | https://www.genecards .org/cgi-bin/carddisp.pl?gene= ABCB7 |
| CFHR1 | Complement Factor H Related 1 | Protein Coding | 42 | GC01P 196822 | 1.374 459 | https://www.genecards .org/cgi-bin/carddisp.pl?gene= CFHR1 |
| RYR1 | Ryanodine Receptor 1 | Protein Coding | 45 | GC19P 066612 | 1.373 652 | https://www.genecards .org/cgi-bin/carddisp.pl?gene= RYR1 |
| CDC25B | Cell Division Cycle 25B | Protein Coding | 44 | GC20P 004259 | 1.373 163 | https://www.genecards .org/cgi- |

| | | | | | | |
|---|---|---|---|---|---|---|
| | | | | | | bin/carddisp.pl?gene=CDC25B |
| TCTA | T Cell Leukemia Translocation Altered | Protein Coding | 33 | GC03P049412 | 1.36799 | https://www.genecards.org/cgi-bin/carddisp.pl?gene=TCTA |
| EPHB2 | EPH Receptor B2 | Protein Coding | 49 | GC01P022710 | 1.362989 | https://www.genecards.org/cgi-bin/carddisp.pl?gene=EPHB2 |
| CA4 | Carbonic Anhydrase 4 | Protein Coding | 45 | GC17P060149 | 1.362989 | https://www.genecards.org/cgi-bin/carddisp.pl?gene=CA4 |
| CSTB | Cystatin B | Protein Coding | 45 | GC21M043772 | 1.362989 | https://www.genecards.org/cgi-bin/carddisp.pl?gene=CSTB |
| KDM4B | Lysine Demethylase 4B | Protein Coding | 45 | GC19P004969 | 1.362989 | https://www.genecards.org/cgi-bin/carddisp.pl?gene=KDM4B |
| TALDO1 | Transaldolase 1 | Protein Coding | 45 | GC11P001784 | 1.362989 | https://www.genecards.org/cgi-bin/carddisp.pl?gene=TALDO1 |
| UNG | Uracil DNA Glycosylase | Protein Coding | 45 | GC12P109097 | 1.362989 | https://www.genecards.org/cgi-bin/carddisp.pl?gene=UNG |
| B4GALNT1 | Beta-1,4-N-Acetyl-Galactosaminyltransferase 1 | Protein Coding | 44 | GC12M057623 | 1.362989 | https://www.genecards.org/cgi-bin/carddisp.pl?gene=B4GALNT1 |
| PARN | Poly(A)-Specific Ribonuclease | Protein Coding | 44 | GC16M014435 | 1.362989 | https://www.genecards.org/cgi-bin/carddisp.pl?gene=PARN |
| PML | PML Nuclear Body Scaffold | Protein Coding | 44 | GC15P073994 | 1.362989 | https://www.genecards.org/cgi-bin/carddisp.pl?gene=PML |
| ROBO1 | Roundabout Guidance Receptor 1 | Protein Coding | 44 | GC03M078597 | 1.362989 | https://www.genecards.org/cgi-bin/carddisp.pl?gene=ROBO1 |
| EYA4 | EYA Transcriptional Coactivator And Phosphatase 4 | Protein Coding | 43 | GC06P133240 | 1.362989 | https://www.genecards.org/cgi-bin/carddisp.pl?gene=EYA4 |
| KREMEN1 | Kringle Containing Transmembrane Protein 1 | Protein Coding | 43 | GC22P029073 | 1.362989 | https://www.genecards.org/cgi-bin/carddisp.pl?gene=KREMEN1 |

| | | | | | | |
|---|---|---|---|---|---|---|
| NONO | Non-POU Domain Containing Octamer Binding | Protein Coding | 43 | GC0XP 071255 | 1.362 989 | https://www.genecards .org/cgi-bin/carddisp.pl?gene= NONO |
| ABCC5 | ATP Binding Cassette Subfamily C Member 5 | Protein Coding | 42 | GC03M 183919 | 1.362 989 | https://www.genecards .org/cgi-bin/carddisp.pl?gene= ABCC5 |
| CDA | Cytidine Deaminase | Protein Coding | 42 | GC01P 020588 | 1.362 989 | https://www.genecards .org/cgi-bin/carddisp.pl?gene= CDA |
| CLIP1 | CAP-Gly Domain Containing Linker Protein 1 | Protein Coding | 42 | GC12M 122271 | 1.362 989 | https://www.genecards .org/cgi-bin/carddisp.pl?gene= CLIP1 |
| CRABP2 | Cellular Retinoic Acid Binding Protein 2 | Protein Coding | 42 | GC01M 156701 | 1.362 989 | https://www.genecards .org/cgi-bin/carddisp.pl?gene= CRABP2 |
| DCK | Deoxycytidine Kinase | Protein Coding | 42 | GC04P 070992 | 1.362 989 | https://www.genecards .org/cgi-bin/carddisp.pl?gene= DCK |
| DDAH1 | Dimethylarginine Dimethylaminohydrolase 1 | Protein Coding | 42 | GC01M 085318 | 1.362 989 | https://www.genecards .org/cgi-bin/carddisp.pl?gene= DDAH1 |
| GADD45 A | Growth Arrest And DNA Damage Inducible Alpha | Protein Coding | 42 | GC01P 067685 | 1.362 989 | https://www.genecards .org/cgi-bin/carddisp.pl?gene= GADD45A |
| GDAP1 | Ganglioside Induced Differentiation Associated Protein 1 | Protein Coding | 42 | GC08P 074315 | 1.362 989 | https://www.genecards .org/cgi-bin/carddisp.pl?gene= GDAP1 |
| MKI67 | Marker Of Proliferation Ki-67 | Protein Coding | 42 | GC10M 128096 | 1.362 989 | https://www.genecards .org/cgi-bin/carddisp.pl?gene= MKI67 |
| NCOR1 | Nuclear Receptor Corepressor 1 | Protein Coding | 42 | GC17M 016029 | 1.362 989 | https://www.genecards .org/cgi-bin/carddisp.pl?gene= NCOR1 |
| NIN | Ninein | Protein Coding | 42 | GC14M 050719 | 1.362 989 | https://www.genecards .org/cgi-bin/carddisp.pl?gene= NIN |
| PDGFA | Platelet Derived Growth Factor Subunit A | Protein Coding | 42 | GC07M 000497 | 1.362 989 | https://www.genecards .org/cgi-bin/carddisp.pl?gene= PDGFA |
| PDGFC | Platelet Derived Growth Factor C | Protein Coding | 42 | GC04M 156760 | 1.362 989 | https://www.genecards .org/cgi- |

| | | | | | | |
|---|---|---|---|---|---|---|
| | | | | | | bin/carddisp.pl?gene=PDGFC |
| REST | RE1 Silencing Transcription Factor | Protein Coding | 42 | GC04P056907 | 1.362989 | https://www.genecards.org/cgi-bin/carddisp.pl?gene=REST |
| SLC8A3 | Solute Carrier Family 8 Member A3 | Protein Coding | 42 | GC14M070044 | 1.362989 | https://www.genecards.org/cgi-bin/carddisp.pl?gene=SLC8A3 |
| ASH2L | ASH2 Like, Histone Lysine Methyltransferase Complex Subunit | Protein Coding | 41 | GC08P038468 | 1.362989 | https://www.genecards.org/cgi-bin/carddisp.pl?gene=ASH2L |
| CAPZA1 | Capping Actin Protein Of Muscle Z-Line Subunit Alpha 1 | Protein Coding | 41 | GC01P112619 | 1.362989 | https://www.genecards.org/cgi-bin/carddisp.pl?gene=CAPZA1 |
| CAV2 | Caveolin 2 | Protein Coding | 41 | GC07P116287 | 1.362989 | https://www.genecards.org/cgi-bin/carddisp.pl?gene=CAV2 |
| CD84 | CD84 Molecule | Protein Coding | 41 | GC01M160541 | 1.362989 | https://www.genecards.org/cgi-bin/carddisp.pl?gene=CD84 |
| CENPJ | Centromere Protein J | Protein Coding | 41 | GC13M024882 | 1.362989 | https://www.genecards.org/cgi-bin/carddisp.pl?gene=CENPJ |
| FOXC1 | Forkhead Box C1 | Protein Coding | 41 | GC06P001610 | 1.362989 | https://www.genecards.org/cgi-bin/carddisp.pl?gene=FOXC1 |
| HGFAC | HGF Activator | Protein Coding | 41 | GC04P003443 | 1.362989 | https://www.genecards.org/cgi-bin/carddisp.pl?gene=HGFAC |
| KAT7 | Lysine Acetyltransferase 7 | Protein Coding | 41 | GC17P049788 | 1.362989 | https://www.genecards.org/cgi-bin/carddisp.pl?gene=KAT7 |
| LPAR3 | Lysophosphatidic Acid Receptor 3 | Protein Coding | 41 | GC01M084811 | 1.362989 | https://www.genecards.org/cgi-bin/carddisp.pl?gene=LPAR3 |
| MTMR3 | Myotubularin Related Protein 3 | Protein Coding | 41 | GC22P036836 | 1.362989 | https://www.genecards.org/cgi-bin/carddisp.pl?gene=MTMR3 |
| NOX4 | NADPH Oxidase 4 | Protein Coding | 41 | GC11M089324 | 1.362989 | https://www.genecards.org/cgi-bin/carddisp.pl?gene=NOX4 |

| | | | | | | |
|---|---|---|---|---|---|---|
| PCSK6 | Proprotein Convertase Subtilisin/Kexin Type 6 | Protein Coding | 41 | GC15M 117314 | 1.362 989 | https://www.genecards .org/cgi-bin/carddisp.pl?gene= PCSK6 |
| PRDX4 | Peroxiredoxin 4 | Protein Coding | 41 | GC0XP 023665 | 1.362 989 | https://www.genecards .org/cgi-bin/carddisp.pl?gene= PRDX4 |
| RPL31 | Ribosomal Protein L31 | Protein Coding | 41 | GC02P 100985 | 1.362 989 | https://www.genecards .org/cgi-bin/carddisp.pl?gene= RPL31 |
| SMG1 | SMG1 Nonsense Mediated MRNA Decay Associated PI3K Related Kinase | Protein Coding | 41 | GC16M 019212 | 1.362 989 | https://www.genecards .org/cgi-bin/carddisp.pl?gene= SMG1 |
| TFF1 | Trefoil Factor 1 | Protein Coding | 41 | GC21M 042362 | 1.362 989 | https://www.genecards .org/cgi-bin/carddisp.pl?gene= TFF1 |
| THOP1 | Thimet Oligopeptidase 1 | Protein Coding | 41 | GC19P 002785 | 1.362 989 | https://www.genecards .org/cgi-bin/carddisp.pl?gene= THOP1 |
| TTPA | Alpha Tocopherol Transfer Protein | Protein Coding | 41 | GC08M 063048 | 1.362 989 | https://www.genecards .org/cgi-bin/carddisp.pl?gene= TTPA |
| ASCC1 | Activating Signal Cointegrator 1 Complex Subunit 1 | Protein Coding | 40 | GC10M 072096 | 1.362 989 | https://www.genecards .org/cgi-bin/carddisp.pl?gene= ASCC1 |
| BRD1 | Bromodomain Containing 1 | Protein Coding | 40 | GC22M 049773 | 1.362 989 | https://www.genecards .org/cgi-bin/carddisp.pl?gene= BRD1 |
| CLDN5 | Claudin 5 | Protein Coding | 40 | GC22M 019523 | 1.362 989 | https://www.genecards .org/cgi-bin/carddisp.pl?gene= CLDN5 |
| DHRS3 | Dehydrogenase/Reductase 3 | Protein Coding | 40 | GC01M 012567 | 1.362 989 | https://www.genecards .org/cgi-bin/carddisp.pl?gene= DHRS3 |
| FGL1 | Fibrinogen Like 1 | Protein Coding | 40 | GC08M 017864 | 1.362 989 | https://www.genecards .org/cgi-bin/carddisp.pl?gene= FGL1 |
| MEOX2 | Mesenchyme Homeobox 2 | Protein Coding | 40 | GC07M 015617 | 1.362 989 | https://www.genecards .org/cgi-bin/carddisp.pl?gene= MEOX2 |
| MINK1 | Misshapen Like Kinase 1 | Protein Coding | 40 | GC17P 004833 | 1.362 989 | https://www.genecards .org/cgi- |

| | | | | | | |
|---|---|---|---|---|---|---|
| | | | | | | bin/carddisp.pl?gene=MINK1 |
| TPST1 | Tyrosylprotein Sulfotransferase 1 | Protein Coding | 40 | GC07P070512 | 1.362989 | https://www.genecards.org/cgi-bin/carddisp.pl?gene=TPST1 |
| TRAIP | TRAF Interacting Protein | Protein Coding | 40 | GC03M051209 | 1.362989 | https://www.genecards.org/cgi-bin/carddisp.pl?gene=TRAIP |
| TRIM63 | Tripartite Motif Containing 63 | Protein Coding | 40 | GC01M026062 | 1.362989 | https://www.genecards.org/cgi-bin/carddisp.pl?gene=TRIM63 |
| UNC5C | Unc-5 Netrin Receptor C | Protein Coding | 40 | GC04M095162 | 1.362989 | https://www.genecards.org/cgi-bin/carddisp.pl?gene=UNC5C |
| VNN2 | Vanin 2 | Protein Coding | 40 | GC06M132743 | 1.362989 | https://www.genecards.org/cgi-bin/carddisp.pl?gene=VNN2 |
| BHLHE41 | Basic Helix-Loop-Helix Family Member E41 | Protein Coding | 39 | GC12M026120 | 1.362989 | https://www.genecards.org/cgi-bin/carddisp.pl?gene=BHLHE41 |
| MBD2 | Methyl-CpG Binding Domain Protein 2 | Protein Coding | 39 | GC18M054151 | 1.362989 | https://www.genecards.org/cgi-bin/carddisp.pl?gene=MBD2 |
| SEC14L2 | SEC14 Like Lipid Binding 2 | Protein Coding | 39 | GC22P030396 | 1.362989 | https://www.genecards.org/cgi-bin/carddisp.pl?gene=SEC14L2 |
| SV2B | Synaptic Vesicle Glycoprotein 2B | Protein Coding | 39 | GC15P091099 | 1.362989 | https://www.genecards.org/cgi-bin/carddisp.pl?gene=SV2B |
| BICD1 | BICD Cargo Adaptor 1 | Protein Coding | 38 | GC12P032107 | 1.362989 | https://www.genecards.org/cgi-bin/carddisp.pl?gene=BICD1 |
| BOK | BCL2 Family Apoptosis Regulator BOK | Protein Coding | 38 | GC02P241558 | 1.362989 | https://www.genecards.org/cgi-bin/carddisp.pl?gene=BOK |
| ELL | Elongation Factor For RNA Polymerase II | Protein Coding | 38 | GC19M018443 | 1.362989 | https://www.genecards.org/cgi-bin/carddisp.pl?gene=ELL |
| FCRL2 | Fc Receptor Like 2 | Protein Coding | 38 | GC01M157745 | 1.362989 | https://www.genecards.org/cgi-bin/carddisp.pl?gene=FCRL2 |

| | | | | | | |
|---|---|---|---|---|---|---|
| HSPB6 | Heat Shock Protein Family B (Small) Member 6 | Protein Coding | 38 | GC19M 065727 | 1.362 989 | https://www.genecards.org/cgi-bin/carddisp.pl?gene=HSPB6 |
| KCNK5 | Potassium Two Pore Domain Channel Subfamily K Member 5 | Protein Coding | 38 | GC06M 066128 | 1.362 989 | https://www.genecards.org/cgi-bin/carddisp.pl?gene=KCNK5 |
| MAPRE3 | Microtubule Associated Protein RP/EB Family Member 3 | Protein Coding | 38 | GC02P 026935 | 1.362 989 | https://www.genecards.org/cgi-bin/carddisp.pl?gene=MAPRE3 |
| MIA3 | MIA SH3 Domain ER Export Factor 3 | Protein Coding | 38 | GC01P 222618 | 1.362 989 | https://www.genecards.org/cgi-bin/carddisp.pl?gene=MIA3 |
| MTDH | Metadherin | Protein Coding | 38 | GC08P 097644 | 1.362 989 | https://www.genecards.org/cgi-bin/carddisp.pl?gene=MTDH |
| OLFM4 | Olfactomedin 4 | Protein Coding | 38 | GC13P 053028 | 1.362 989 | https://www.genecards.org/cgi-bin/carddisp.pl?gene=OLFM4 |
| SLCO1A 2 | Solute Carrier Organic Anion Transporter Family Member 1A2 | Protein Coding | 38 | GC12M 021264 | 1.362 989 | https://www.genecards.org/cgi-bin/carddisp.pl?gene=SLCO1A2 |
| BTG3 | BTG Anti-Proliferation Factor 3 | Protein Coding | 37 | GC21M 017621 | 1.362 989 | https://www.genecards.org/cgi-bin/carddisp.pl?gene=BTG3 |
| CERS6 | Ceramide Synthase 6 | Protein Coding | 37 | GC02P 168455 | 1.362 989 | https://www.genecards.org/cgi-bin/carddisp.pl?gene=CERS6 |
| CYP4F22 | Cytochrome P450 Family 4 Subfamily F Member 22 | Protein Coding | 37 | GC19P 015508 | 1.362 989 | https://www.genecards.org/cgi-bin/carddisp.pl?gene=CYP4F22 |
| EBAG9 | Estrogen Receptor Binding Site Associated Antigen 9 | Protein Coding | 37 | GC08P 109536 | 1.362 989 | https://www.genecards.org/cgi-bin/carddisp.pl?gene=EBAG9 |
| GLCCI1 | Glucocorticoid Induced 1 | Protein Coding | 37 | GC07P 007974 | 1.362 989 | https://www.genecards.org/cgi-bin/carddisp.pl?gene=GLCCI1 |
| KHDRBS 3 | KH RNA Binding Domain Containing, Signal Transduction Associated 3 | Protein Coding | 37 | GC08P 135457 | 1.362 989 | https://www.genecards.org/cgi-bin/carddisp.pl?gene=KHDRBS3 |
| NCR1 | Natural Cytotoxicity Triggering Receptor 1 | Protein Coding | 37 | GC19P 067389 | 1.362 989 | https://www.genecards.org/cgi- |

| | | | | | | |
|---|---|---|---|---|---|---|
| | | | | | | bin/carddisp.pl?gene=NCR1 |
| NLRP6 | NLR Family Pyrin Domain Containing 6 | Protein Coding | 37 | GC11P 000269 | 1.362 989 | https://www.genecards.org/cgi-bin/carddisp.pl?gene=NLRP6 |
| PDZD2 | PDZ Domain Containing 2 | Protein Coding | 37 | GC05P 031639 | 1.362 989 | https://www.genecards.org/cgi-bin/carddisp.pl?gene=PDZD2 |
| PIWIL4 | Piwi Like RNA-Mediated Gene Silencing 4 | Protein Coding | 37 | GC11P 094543 | 1.362 989 | https://www.genecards.org/cgi-bin/carddisp.pl?gene=PIWIL4 |
| SAP30BP | SAP30 Binding Protein | Protein Coding | 37 | GC17P 075667 | 1.362 989 | https://www.genecards.org/cgi-bin/carddisp.pl?gene=SAP30BP |
| TOR2A | Torsin Family 2 Member A | Protein Coding | 37 | GC09M 127800 | 1.362 989 | https://www.genecards.org/cgi-bin/carddisp.pl?gene=TOR2A |
| YTHDF2 | YTH N6-Methyladenosine RNA Binding Protein 2 | Protein Coding | 37 | GC01P 028800 | 1.362 989 | https://www.genecards.org/cgi-bin/carddisp.pl?gene=YTHDF2 |
| ZC3HC1 | Zinc Finger C3HC-Type Containing 1 | Protein Coding | 37 | GC07M 130026 | 1.362 989 | https://www.genecards.org/cgi-bin/carddisp.pl?gene=ZC3HC1 |
| CBLN2 | Cerebellin 2 Precursor | Protein Coding | 36 | GC18M 072537 | 1.362 989 | https://www.genecards.org/cgi-bin/carddisp.pl?gene=CBLN2 |
| CCNG2 | Cyclin G2 | Protein Coding | 36 | GC04P 077158 | 1.362 989 | https://www.genecards.org/cgi-bin/carddisp.pl?gene=CCNG2 |
| CNTNAP 4 | Contactin Associated Protein Family Member 4 | Protein Coding | 36 | GC16P 076278 | 1.362 989 | https://www.genecards.org/cgi-bin/carddisp.pl?gene=CNTNAP4 |
| DERL1 | Derlin 1 | Protein Coding | 36 | GC08M 123013 | 1.362 989 | https://www.genecards.org/cgi-bin/carddisp.pl?gene=DERL1 |
| EMCN | Endomucin | Protein Coding | 36 | GC04M 100395 | 1.362 989 | https://www.genecards.org/cgi-bin/carddisp.pl?gene=EMCN |
| GPR15 | G Protein-Coupled Receptor 15 | Protein Coding | 36 | GC03P 098531 | 1.362 989 | https://www.genecards.org/cgi-bin/carddisp.pl?gene=GPR15 |

| | | | | | | |
|---|---|---|---|---|---|---|
| MRPL17 | Mitochondrial Ribosomal Protein L17 | Protein Coding | 36 | GC11M 006680 | 1.362 989 | https://www.genecards.org/cgi-bin/carddisp.pl?gene=MRPL17 |
| PIWIL2 | Piwi Like RNA-Mediated Gene Silencing 2 | Protein Coding | 36 | GC08P 022275 | 1.362 989 | https://www.genecards.org/cgi-bin/carddisp.pl?gene=PIWIL2 |
| SPAG16 | Sperm Associated Antigen 16 | Protein Coding | 36 | GC02P 213284 | 1.362 989 | https://www.genecards.org/cgi-bin/carddisp.pl?gene=SPAG16 |
| TAS2R38 | Taste 2 Receptor Member 38 | Protein Coding | 36 | GC07M 141972 | 1.362 989 | https://www.genecards.org/cgi-bin/carddisp.pl?gene=TAS2R38 |
| ARAP2 | ArfGAP With RhoGAP Domain, Ankyrin Repeat And PH Domain 2 | Protein Coding | 34 | GC04M 035950 | 1.362 989 | https://www.genecards.org/cgi-bin/carddisp.pl?gene=ARAP2 |
| CASC3 | CASC3 Exon Junction Complex Subunit | Protein Coding | 34 | GC17P 040140 | 1.362 989 | https://www.genecards.org/cgi-bin/carddisp.pl?gene=CASC3 |
| CASP12 | Caspase 12 (Gene/Pseudogene) | Protein Coding | 34 | GC11M 104885 | 1.362 989 | https://www.genecards.org/cgi-bin/carddisp.pl?gene=CASP12 |
| CST2 | Cystatin SA | Protein Coding | 34 | GC20M 023805 | 1.362 989 | https://www.genecards.org/cgi-bin/carddisp.pl?gene=CST2 |
| FOXJ3 | Forkhead Box J3 | Protein Coding | 34 | GC01M 042176 | 1.362 989 | https://www.genecards.org/cgi-bin/carddisp.pl?gene=FOXJ3 |
| GALNT18 | Polypeptide N-Acetylgalactosaminyltransferase 18 | Protein Coding | 34 | GC11M 011293 | 1.362 989 | https://www.genecards.org/cgi-bin/carddisp.pl?gene=GALNT18 |
| IFNL2 | Interferon Lambda 2 | Protein Coding | 34 | GC19P 039268 | 1.362 989 | https://www.genecards.org/cgi-bin/carddisp.pl?gene=IFNL2 |
| ALKBH5 | AlkB Homolog 5, RNA Demethylase | Protein Coding | 33 | GC17P 018183 | 1.362 989 | https://www.genecards.org/cgi-bin/carddisp.pl?gene=ALKBH5 |
| C1orf87 | Chromosome 1 Open Reading Frame 87 | Protein Coding | 33 | GC01M 059987 | 1.362 989 | https://www.genecards.org/cgi-bin/carddisp.pl?gene=C1orf87 |
| EBF4 | EBF Family Member 4 | Protein Coding | 33 | GC20P 004231 | 1.362 989 | https://www.genecards.org/cgi- |

| | | | | | | |
|---|---|---|---|---|---|---|
| | | | | | | bin/carddisp.pl?gene=EBF4 |
| PCNP | PEST Proteolytic Signal Containing Nuclear Protein | Protein Coding | 33 | GC03P101574 | 1.362989 | https://www.genecards.org/cgi-bin/carddisp.pl?gene=PCNP |
| TESPA1 | Thymocyte Expressed, Positive Selection Associated 1 | Protein Coding | 33 | GC12M054949 | 1.362989 | https://www.genecards.org/cgi-bin/carddisp.pl?gene=TESPA1 |
| TTC38 | Tetratricopeptide Repeat Domain 38 | Protein Coding | 33 | GC22P046267 | 1.362989 | https://www.genecards.org/cgi-bin/carddisp.pl?gene=TTC38 |
| NKAPL | NFKB Activating Protein Like | Protein Coding | 32 | GC06P028259 | 1.362989 | https://www.genecards.org/cgi-bin/carddisp.pl?gene=NKAPL |
| RUBCN | Rubicon Autophagy Regulator | Protein Coding | 32 | GC03M198357 | 1.362989 | https://www.genecards.org/cgi-bin/carddisp.pl?gene=RUBCN |
| MTREX | Mtr4 Exosome RNA Helicase | Protein Coding | 30 | GC05P055308 | 1.362989 | https://www.genecards.org/cgi-bin/carddisp.pl?gene=MTREX |
| ENHO | Energy Homeostasis Associated | Protein Coding | 29 | GC09M034516 | 1.362989 | https://www.genecards.org/cgi-bin/carddisp.pl?gene=ENHO |
| IGSF22 | Immunoglobulin Superfamily Member 22 | Protein Coding | 28 | GC11M018705 | 1.362989 | https://www.genecards.org/cgi-bin/carddisp.pl?gene=IGSF22 |
| PALS2 | Protein Associated With LIN7 2, MAGUK P55 Family Member | Protein Coding | 27 | GC07P024574 | 1.362989 | https://www.genecards.org/cgi-bin/carddisp.pl?gene=PALS2 |
| BLTP3A | Bridge-Like Lipid Transfer Protein Family Member 3A | Protein Coding | 26 | GC06P084361 | 1.362989 | https://www.genecards.org/cgi-bin/carddisp.pl?gene=BLTP3A |
| PBOV1 | Prostate And Breast Cancer Overexpressed 1 | Protein Coding | 26 | GC06M138215 | 1.362989 | https://www.genecards.org/cgi-bin/carddisp.pl?gene=PBOV1 |
| FAM215A | Family With Sequence Similarity 215 Member A | RNA Gene | 23 | GC17P043917 | 1.362989 | https://www.genecards.org/cgi-bin/carddisp.pl?gene=FAM215A |
| FER1L4 | Fer-1 Like Family Member 4 (Pseudogene) | Pseudogene | 23 | GC20M035558 | 1.362989 | https://www.genecards.org/cgi-bin/carddisp.pl?gene=FER1L4 |

| | | | | | | |
|---|---|---|---|---|---|---|
| MIR191 | MicroRNA 191 | RNA Gene | 23 | GC03M 051160 | 1.362 989 | https://www.genecards .org/cgi- bin/carddisp.pl?gene= MIR191 |
| HAND2- AS1 | HAND2 Antisense RNA 1 | RNA Gene | 21 | GC04P 173527 | 1.362 989 | https://www.genecards .org/cgi- bin/carddisp.pl?gene= HAND2-AS1 |
| MIR199A 2 | MicroRNA 199a-2 | RNA Gene | 21 | GC01M 172235 | 1.362 989 | https://www.genecards .org/cgi- bin/carddisp.pl?gene= MIR199A2 |
| MIR301A | MicroRNA 301a | RNA Gene | 21 | GC17M 059151 | 1.362 989 | https://www.genecards .org/cgi- bin/carddisp.pl?gene= MIR301A |
| MAPKAP K5-AS1 | MAPKAPK5 Antisense RNA 1 | RNA Gene | 20 | GC12M 111839 | 1.362 989 | https://www.genecards .org/cgi- bin/carddisp.pl?gene= MAPKAPK5-AS1 |
| MIR101- 1 | MicroRNA 101-1 | RNA Gene | 20 | GC01M 065058 | 1.362 989 | https://www.genecards .org/cgi- bin/carddisp.pl?gene= MIR101-1 |
| MIR137 | MicroRNA 137 | RNA Gene | 20 | GC01M 098046 | 1.362 989 | https://www.genecards .org/cgi- bin/carddisp.pl?gene= MIR137 |
| MIR181A 2 | MicroRNA 181a-2 | RNA Gene | 20 | GC09P 124692 | 1.362 989 | https://www.genecards .org/cgi- bin/carddisp.pl?gene= MIR181A2 |
| MIR760 | MicroRNA 760 | RNA Gene | 20 | GC01P 093846 | 1.362 989 | https://www.genecards .org/cgi- bin/carddisp.pl?gene= MIR760 |
| UMAD1 | UBAP1-MVB12- Associated (UMA) Domain Containing 1 | Protein Coding | 20 | GC07P 007640 | 1.362 989 | https://www.genecards .org/cgi- bin/carddisp.pl?gene= UMAD1 |
| MIR128- 1 | MicroRNA 128-1 | RNA Gene | 19 | GC02P 135665 | 1.362 989 | https://www.genecards .org/cgi- bin/carddisp.pl?gene= MIR128-1 |
| MIR129- 2 | MicroRNA 129-2 | RNA Gene | 19 | GC11P 043772 | 1.362 989 | https://www.genecards .org/cgi- bin/carddisp.pl?gene= MIR129-2 |
| MIR29B2 | MicroRNA 29b-2 | RNA Gene | 19 | GC01M 207806 | 1.362 989 | https://www.genecards .org/cgi- bin/carddisp.pl?gene= MIR29B2 |
| CBR3- AS1 | CBR3 Antisense RNA 1 | RNA Gene | 18 | GC21M 036131 | 1.362 989 | https://www.genecards .org/cgi- |

| | | | | | | |
|---|---|---|---|---|---|---|
| | | | | | | bin/carddisp.pl?gene=CBR3-AS1 |
| CYTOR | Cytoskeleton Regulator RNA | RNA Gene | 18 | GC02P088103 | 1.362989 | https://www.genecards.org/cgi-bin/carddisp.pl?gene=CYTOR |
| LINC-PINT | Long Intergenic Non-Protein Coding RNA, P53 Induced Transcript | RNA Gene | 18 | GC07M131004 | 1.362989 | https://www.genecards.org/cgi-bin/carddisp.pl?gene=LINC-PINT |
| MIR218-1 | MicroRNA 218-1 | RNA Gene | 18 | GC04P020849 | 1.362989 | https://www.genecards.org/cgi-bin/carddisp.pl?gene=MIR218-1 |
| MIR31HG | MIR31 Host Gene | RNA Gene | 18 | GC09M021439 | 1.362989 | https://www.genecards.org/cgi-bin/carddisp.pl?gene=MIR31HG |
| MIR543 | MicroRNA 543 | RNA Gene | 18 | GC14P109698 | 1.362989 | https://www.genecards.org/cgi-bin/carddisp.pl?gene=MIR543 |
| GUSBP1 | GUSB Pseudogene 1 | Pseudogene | 17 | GC05P021337 | 1.362989 | https://www.genecards.org/cgi-bin/carddisp.pl?gene=GUSBP1 |
| LINC00452 | Long Intergenic Non-Protein Coding RNA 452 | RNA Gene | 17 | GC13P113884 | 1.362989 | https://www.genecards.org/cgi-bin/carddisp.pl?gene=LINC00452 |
| MIR1915 | MicroRNA 1915 | RNA Gene | 17 | GC10M021496 | 1.362989 | https://www.genecards.org/cgi-bin/carddisp.pl?gene=MIR1915 |
| MIR323A | MicroRNA 323a | RNA Gene | 17 | GC14P109973 | 1.362989 | https://www.genecards.org/cgi-bin/carddisp.pl?gene=MIR323A |
| MIR573 | MicroRNA 573 | RNA Gene | 17 | GC04M024521 | 1.362989 | https://www.genecards.org/cgi-bin/carddisp.pl?gene=MIR573 |
| PICSAR | P38 Inhibited Cutaneous Squamous Cell Carcinoma Associated LincRNA | RNA Gene | 17 | GC21M050753 | 1.362989 | https://www.genecards.org/cgi-bin/carddisp.pl?gene=PICSAR |
| SNHG14 | Small Nucleolar RNA Host Gene 14 | RNA Gene | 17 | GC15P041831 | 1.362989 | https://www.genecards.org/cgi-bin/carddisp.pl?gene=SNHG14 |
| TSPEAR-AS2 | TSPEAR Antisense RNA 2 | RNA Gene | 17 | GC21P045172 | 1.362989 | https://www.genecards.org/cgi-bin/carddisp.pl?gene=TSPEAR-AS2 |

| | | | | | | |
|---|---|---|---|---|---|---|
| MIR1286 | MicroRNA 1286 | RNA Gene | 16 | GC22M020249 | 1.362989 | https://www.genecards.org/cgi-bin/carddisp.pl?gene=MIR1286 |
| MIR363 | MicroRNA 363 | RNA Gene | 16 | GC0XM134308 | 1.362989 | https://www.genecards.org/cgi-bin/carddisp.pl?gene=MIR363 |
| ZNF667-AS1 | ZNF667 Antisense RNA 1 (Head To Head) | RNA Gene | 16 | GC19P056477 | 1.362989 | https://www.genecards.org/cgi-bin/carddisp.pl?gene=ZNF667-AS1 |
| MIR4423 | MicroRNA 4423 | RNA Gene | 15 | GC01P085133 | 1.362989 | https://www.genecards.org/cgi-bin/carddisp.pl?gene=MIR4423 |
| MIR522 | MicroRNA 522 | RNA Gene | 15 | GC19P053751 | 1.362989 | https://www.genecards.org/cgi-bin/carddisp.pl?gene=MIR522 |
| IGKV2D-29 | Immunoglobulin Kappa Variable 2D-29 | Protein Coding | 14 | GC02P091319 | 1.362989 | https://www.genecards.org/cgi-bin/carddisp.pl?gene=IGKV2D-29 |
| MIR5196 | MicroRNA 5196 | RNA Gene | 14 | GC19P066536 | 1.362989 | https://www.genecards.org/cgi-bin/carddisp.pl?gene=MIR5196 |
| MIR523 | MicroRNA 523 | RNA Gene | 14 | GC19P053698 | 1.362989 | https://www.genecards.org/cgi-bin/carddisp.pl?gene=MIR523 |
| LINC01483 | Long Intergenic Non-Protein Coding RNA 1483 | RNA Gene | 13 | GC17P069577 | 1.362989 | https://www.genecards.org/cgi-bin/carddisp.pl?gene=LINC01483 |
| TSPOAP1-AS1 | TSPOAP1, SUPT4H1 And RNF43 Antisense RNA 1 | RNA Gene | 13 | GC17P058326 | 1.362989 | https://www.genecards.org/cgi-bin/carddisp.pl?gene=TSPOAP1-AS1 |
| LOC730100 | Uncharacterized LOC730100 | RNA Gene | 12 | GC02P051033 | 1.362989 | https://www.genecards.org/cgi-bin/carddisp.pl?gene=LOC730100 |
| MIR5571 | MicroRNA 5571 | RNA Gene | 11 | GC22P022886 | 1.362989 | https://www.genecards.org/cgi-bin/carddisp.pl?gene=MIR5571 |
| CLYBL-AS3 | CLYBL Antisense RNA 3 | RNA Gene | 10 | GC13M099741 | 1.362989 | https://www.genecards.org/cgi-bin/carddisp.pl?gene=CLYBL-AS3 |

| | | | | | | |
|---|---|---|---|---|---|---|
| HSPE1P1 | Heat Shock Protein Family E (Hsp10) Member 1 Pseudogene 1 | Pseudo gene | 9 | GC20M 039932 | 1.362 989 | https://www.genecards .org/cgi-bin/carddisp.pl?gene= HSPE1P1 |
| CYP4F23 P | Cytochrome P450 Family 4 Subfamily F Member 23, Pseudogene | Pseudo gene | 8 | GC19P 015822 | 1.362 989 | https://www.genecards .org/cgi-bin/carddisp.pl?gene= CYP4F23P |
| RPL31P3 1 | Ribosomal Protein L31 Pseudogene 31 | Pseudo gene | 8 | GC04P 033972 | 1.362 989 | https://www.genecards .org/cgi-bin/carddisp.pl?gene= RPL31P31 |
| CDR1-AS | CDR1 Antisense RNA | RNA Gene | 7 | GC0XU 902169 | 1.362 989 | https://www.genecards .org/cgi-bin/carddisp.pl?gene= CDR1-AS |
| MRPS35 P1 | Mitochondrial Ribosomal Protein S35 Pseudogene 1 | Pseudo gene | 6 | GC03P 005446 | 1.362 989 | https://www.genecards .org/cgi-bin/carddisp.pl?gene= MRPS35P1 |
| MS | Multiple Sclerosis | Geneti c Locus | 4 | GC00U 990205 | 1.362 989 | https://www.genecards .org/cgi-bin/carddisp.pl?gene= MS |
| NTT | Noncoding Transcript In T Cells | RNA Gene | 3 | GC06U 990159 | 1.362 989 | https://www.genecards .org/cgi-bin/carddisp.pl?gene= NTT |
| LOC1109 73015 | NOS3 5' Regulatory Region | Biologi cal Region | 2 | GC07P 150988 | 1.362 989 | https://www.genecards .org/cgi-bin/carddisp.pl?gene= LOC110973015 |
| SCNN1A | Sodium Channel Epithelial 1 Subunit Alpha | Protein Coding | 46 | GC12M 006346 | 1.361 563 | https://www.genecards .org/cgi-bin/carddisp.pl?gene= SCNN1A |
| NCAPD2 | Non-SMC Condensin I Complex Subunit D2 | Protein Coding | 40 | GC12P 006493 | 1.361 563 | https://www.genecards .org/cgi-bin/carddisp.pl?gene= NCAPD2 |
| CDCA3 | Cell Division Cycle Associated 3 | Protein Coding | 37 | GC12M 006844 | 1.361 563 | https://www.genecards .org/cgi-bin/carddisp.pl?gene= CDCA3 |
| S100A3 | S100 Calcium Binding Protein A3 | Protein Coding | 37 | GC01M 153547 | 1.361 563 | https://www.genecards .org/cgi-bin/carddisp.pl?gene= S100A3 |
| UBAP2L | Ubiquitin Associated Protein 2 Like | Protein Coding | 37 | GC01P 154219 | 1.361 563 | https://www.genecards .org/cgi-bin/carddisp.pl?gene= UBAP2L |
| ZNF384 | Zinc Finger Protein 384 | Protein Coding | 37 | GC12M 006903 | 1.361 563 | https://www.genecards .org/cgi- |

| | | | | | | |
|---|---|---|---|---|---|---|
| | | | | | | bin/carddisp.pl?gene=ZNF384 |
| AQP10 | Aquaporin 10 | Protein Coding | 36 | GC01P 154321 | 1.361 563 | https://www.genecards.org/cgi-bin/carddisp.pl?gene=AQP10 |
| CH25H | Cholesterol 25-Hydroxylase | Protein Coding | 36 | GC10M 089205 | 1.361 563 | https://www.genecards.org/cgi-bin/carddisp.pl?gene=CH25H |
| NOP2 | NOP2 Nucleolar Protein | Protein Coding | 36 | GC12M 006556 | 1.361 563 | https://www.genecards.org/cgi-bin/carddisp.pl?gene=NOP2 |
| UBE2Q1 | Ubiquitin Conjugating Enzyme E2 Q1 | Protein Coding | 36 | GC01M 154548 | 1.361 563 | https://www.genecards.org/cgi-bin/carddisp.pl?gene=UBE2Q1 |
| TOMM7 | Translocase Of Outer Mitochondrial Membrane 7 | Protein Coding | 35 | GC07M 022816 | 1.361 563 | https://www.genecards.org/cgi-bin/carddisp.pl?gene=TOMM7 |
| LONRF2 | LON Peptidase N-Terminal Domain And Ring Finger 2 | Protein Coding | 34 | GC02M 100274 | 1.361 563 | https://www.genecards.org/cgi-bin/carddisp.pl?gene=LONRF2 |
| SPSB2 | SplA/Ryanodine Receptor Domain And SOCS Box Containing 2 | Protein Coding | 34 | GC12M 006918 | 1.361 563 | https://www.genecards.org/cgi-bin/carddisp.pl?gene=SPSB2 |
| TAPBPL | TAP Binding Protein Like | Protein Coding | 34 | GC12P 006451 | 1.361 563 | https://www.genecards.org/cgi-bin/carddisp.pl?gene=TAPBPL |
| PLEKHG 6 | Pleckstrin Homology And RhoGEF Domain Containing G6 | Protein Coding | 33 | GC12P 006310 | 1.361 563 | https://www.genecards.org/cgi-bin/carddisp.pl?gene=PLEKHG6 |
| RMI2 | RecQ Mediated Genome Instability 2 | Protein Coding | 33 | GC16P 012113 | 1.361 563 | https://www.genecards.org/cgi-bin/carddisp.pl?gene=RMI2 |
| SHE | Src Homology 2 Domain Containing E | Protein Coding | 33 | GC01M 154556 | 1.361 563 | https://www.genecards.org/cgi-bin/carddisp.pl?gene=SHE |
| IFFO1 | Intermediate Filament Family Orphan 1 | Protein Coding | 32 | GC12M 006538 | 1.361 563 | https://www.genecards.org/cgi-bin/carddisp.pl?gene=IFFO1 |
| PRM1 | Protamine 1 | Protein Coding | 30 | GC16M 012266 | 1.361 563 | https://www.genecards.org/cgi-bin/carddisp.pl?gene=PRM1 |

| | | | | | | |
|---|---|---|---|---|---|---|
| TDRD10 | Tudor Domain Containing 10 | Protein Coding | 29 | GC01P 154502 | 1.361 563 | https://www.genecards .org/cgi-bin/carddisp.pl?gene= TDRD10 |
| CFAP141 | Cilia And Flagella Associated Protein 141 | Protein Coding | 21 | GC01M 154200 | 1.361 563 | https://www.genecards .org/cgi-bin/carddisp.pl?gene= CFAP141 |
| LOC4004 99 | Putative Uncharacterized Protein LOC400499 | Protein Coding | 21 | GC16M 012281 | 1.361 563 | https://www.genecards .org/cgi-bin/carddisp.pl?gene= LOC400499 |
| SCARNA 12 | Small Cajal Body-Specific RNA 12 | RNA Gene | 21 | GC12M 007743 | 1.361 563 | https://www.genecards .org/cgi-bin/carddisp.pl?gene= SCARNA12 |
| ACTA2-AS1 | ACTA2 Antisense RNA 1 | RNA Gene | 16 | GC10P 088932 | 1.361 563 | https://www.genecards .org/cgi-bin/carddisp.pl?gene= ACTA2-AS1 |
| CD27-AS1 | CD27 Antisense RNA 1 | RNA Gene | 16 | GC12M 006439 | 1.361 563 | https://www.genecards .org/cgi-bin/carddisp.pl?gene= CD27-AS1 |
| IL6R-AS1 | IL6R Antisense RNA 1 | RNA Gene | 12 | GC01M 154544 | 1.361 563 | https://www.genecards .org/cgi-bin/carddisp.pl?gene=I L6R-AS1 |
| SNORA5 8B | Small Nucleolar RNA, H/ACA Box 58B | RNA Gene | 12 | GC01P 154261 | 1.361 563 | https://www.genecards .org/cgi-bin/carddisp.pl?gene= SNORA58B |
| UBE2Q1-AS1 | UBE2Q1 Antisense RNA 1 | RNA Gene | 12 | GC01P 154553 | 1.361 563 | https://www.genecards .org/cgi-bin/carddisp.pl?gene= UBE2Q1-AS1 |
| LINC018 45 | Long Intergenic Non-Protein Coding RNA 1845 | RNA Gene | 10 | GC05M 159448 | 1.361 563 | https://www.genecards .org/cgi-bin/carddisp.pl?gene= LINC01845 |
| ENSG000 00256433 | Novel Transcript | RNA Gene | 9 | GC12P 006393 | 1.361 563 | https://www.genecards .org/cgi-bin/carddisp.pl?gene= ENSG00000256433 |
| PSMD8P 1 | Proteasome 26S Subunit, Non-ATPase, 8 Pseudogene 1 | Pseudo gene | 9 | GC01M 154414 | 1.361 563 | https://www.genecards .org/cgi-bin/carddisp.pl?gene= PSMD8P1 |
| RNU7-57P | RNA, U7 Small Nuclear 57 Pseudogene | Pseudo gene | 9 | GC01P 154382 | 1.361 563 | https://www.genecards .org/cgi-bin/carddisp.pl?gene= RNU7-57P |
| ENSG000 00219410 | Novel Transcript | RNA Gene | 8 | GC12P 021065 | 1.361 563 | https://www.genecards .org/cgi- |

| | | | | | | |
|---|---|---|---|---|---|---|
| | | | | | | bin/carddisp.pl?gene=ENSG00000219410 |
| ENSG00000286116 | FAS Antisense RNA 1 | Uncategorized | 8 | GC10M088990 | 1.361563 | https://www.genecards.org/cgi-bin/carddisp.pl?gene=ENSG00000286116 |
| ENSG00000247853 | Novel Transcript, Antisense To CHD4 | RNA Gene | 7 | GC12P021050 | 1.361563 | https://www.genecards.org/cgi-bin/carddisp.pl?gene=ENSG00000247853 |
| ENSG00000225544 | Pseudogene Similar To Part Of Metallophosphoesterase 1 (MPPE1) | Pseudogene | 6 | GC22M021885 | 1.361563 | https://www.genecards.org/cgi-bin/carddisp.pl?gene=ENSG00000225544 |
| RAB1AP1 | RAB1A Pseudogene 1 | Pseudogene | 6 | GC02M190993 | 1.361563 | https://www.genecards.org/cgi-bin/carddisp.pl?gene=RAB1AP1 |
| MF281465 | | RNA Gene | 5 | GC02M191013 | 1.361563 | https://www.genecards.org/cgi-bin/carddisp.pl?gene=MF281465 |
| MTCYBP42 | MT-CYB Pseudogene 42 | Pseudogene | 5 | GC07M022748 | 1.361563 | https://www.genecards.org/cgi-bin/carddisp.pl?gene=MTCYBP42 |
| lnc-HLA-DQB1-2 | | RNA Gene | 5 | GC06M066017 | 1.361563 | https://www.genecards.org/cgi-bin/carddisp.pl?gene=lnc-HLA-DQB1-2 |
| lnc-PLEKHG6-5 | | RNA Gene | 5 | GC12P021034 | 1.361563 | https://www.genecards.org/cgi-bin/carddisp.pl?gene=lnc-PLEKHG6-5 |
| lnc-PRM1-3 | | RNA Gene | 5 | GC16M012282 | 1.361563 | https://www.genecards.org/cgi-bin/carddisp.pl?gene=lnc-PRM1-3 |
| lnc-PRM1-5 | | RNA Gene | 5 | GC16M012280 | 1.361563 | https://www.genecards.org/cgi-bin/carddisp.pl?gene=lnc-PRM1-5 |
| lnc-RMI2-2 | | RNA Gene | 5 | GC16P012119 | 1.361563 | https://www.genecards.org/cgi-bin/carddisp.pl?gene=lnc-RMI2-2 |
| ENSG00000262636 | Mitochondrial Ribosomal Protein L18 (MRPL18) Pseudogene | Pseudogene | 4 | GC16M012284 | 1.361563 | https://www.genecards.org/cgi-bin/carddisp.pl?gene=ENSG00000262636 |
| HSALNG0049436 | | RNA Gene | 4 | GC06P084614 | 1.361563 | https://www.genecards.org/cgi-bin/carddisp.pl?gene=HSALNG0049436 |

| | | | | | | |
|---|---|---|---|---|---|---|
| HSALNG0075913 | | RNA Gene | 4 | GC10P006045 | 1.361563 | https://www.genecards.org/cgi-bin/carddisp.pl?gene=HSALNG0075913 |
| HSALNG0079553 | | RNA Gene | 4 | GC10M088999 | 1.361563 | https://www.genecards.org/cgi-bin/carddisp.pl?gene=HSALNG0079553 |
| HSALNG0088851 | | RNA Gene | 4 | GC12P021614 | 1.361563 | https://www.genecards.org/cgi-bin/carddisp.pl?gene=HSALNG0088851 |
| HSALNG0088852 | | RNA Gene | 4 | GC12P021615 | 1.361563 | https://www.genecards.org/cgi-bin/carddisp.pl?gene=HSALNG0088852 |
| LOC105371082 | Uncharacterized LOC105371082 | RNA Gene | 4 | GC16P011249 | 1.361563 | https://www.genecards.org/cgi-bin/carddisp.pl?gene=LOC105371082 |
| RF00017-3467 | | RNA Gene | 4 | GC22P038730 | 1.361563 | https://www.genecards.org/cgi-bin/carddisp.pl?gene=RF00017-3467 |
| RF00017-3468 | | RNA Gene | 4 | GC22P038733 | 1.361563 | https://www.genecards.org/cgi-bin/carddisp.pl?gene=RF00017-3468 |
| piR-49732-033 | | RNA Gene | 4 | GC10M006055 | 1.361563 | https://www.genecards.org/cgi-bin/carddisp.pl?gene=piR-49732-033 |
| CDKN2B-AS1 | CDKN2B Antisense RNA 1 | RNA Gene | 22 | GC09P021994 | 1.360434 | https://www.genecards.org/cgi-bin/carddisp.pl?gene=CDKN2B-AS1 |
| RICTOR | RPTOR Independent Companion Of MTOR Complex 2 | Protein Coding | 42 | GC05M038937 | 1.359894 | https://www.genecards.org/cgi-bin/carddisp.pl?gene=RICTOR |
| GALNS | Galactosamine (N-Acetyl)-6-Sulfatase | Protein Coding | 46 | GC16M088813 | 1.358621 | https://www.genecards.org/cgi-bin/carddisp.pl?gene=GALNS |
| C4BPB | Complement Component 4 Binding Protein Beta | Protein Coding | 40 | GC01P207088 | 1.356425 | https://www.genecards.org/cgi-bin/carddisp.pl?gene=C4BPB |
| TTF2 | Transcription Termination Factor 2 | Protein Coding | 39 | GC01P117060 | 1.356425 | https://www.genecards.org/cgi-bin/carddisp.pl?gene=TTF2 |
| TRIP11 | Thyroid Hormone Receptor Interactor 11 | Protein Coding | 41 | GC14M091965 | 1.355733 | https://www.genecards.org/cgi- |

| | | | | | | |
|---|---|---|---|---|---|---|
| | | | | | | bin/carddisp.pl?gene=TRIP11 |
| CCBE1 | Collagen And Calcium Binding EGF Domains 1 | Protein Coding | 41 | GC18M059430 | 1.353179 | https://www.genecards.org/cgi-bin/carddisp.pl?gene=CCBE1 |
| S100A7 | S100 Calcium Binding Protein A7 | Protein Coding | 39 | GC01M153457 | 1.350399 | https://www.genecards.org/cgi-bin/carddisp.pl?gene=S100A7 |
| SEMA5A | Semaphorin 5A | Protein Coding | 40 | GC05M009036 | 1.350374 | https://www.genecards.org/cgi-bin/carddisp.pl?gene=SEMA5A |
| SPZ1 | Spermatogenic Leucine Zipper 1 | Protein Coding | 34 | GC05P080319 | 1.350374 | https://www.genecards.org/cgi-bin/carddisp.pl?gene=SPZ1 |
| ERN1 | Endoplasmic Reticulum To Nucleus Signaling 1 | Protein Coding | 44 | GC17M064039 | 1.345868 | https://www.genecards.org/cgi-bin/carddisp.pl?gene=ERN1 |
| LTC4S | Leukotriene C4 Synthase | Protein Coding | 41 | GC05P179793 | 1.339262 | https://www.genecards.org/cgi-bin/carddisp.pl?gene=LTC4S |
| MIR24-1 | MicroRNA 24-1 | RNA Gene | 20 | GC09P095086 | 1.338933 | https://www.genecards.org/cgi-bin/carddisp.pl?gene=MIR24-1 |
| CNTNAP2 | Contactin Associated Protein 2 | Protein Coding | 43 | GC07P146116 | 1.337749 | https://www.genecards.org/cgi-bin/carddisp.pl?gene=CNTNAP2 |
| UCN3 | Urocortin 3 | Protein Coding | 36 | GC10P005396 | 1.334767 | https://www.genecards.org/cgi-bin/carddisp.pl?gene=UCN3 |
| UCN2 | Urocortin 2 | Protein Coding | 32 | GC03M048561 | 1.334767 | https://www.genecards.org/cgi-bin/carddisp.pl?gene=UCN2 |
| MIRLET7G | MicroRNA Let-7g | RNA Gene | 20 | GC03M052268 | 1.332631 | https://www.genecards.org/cgi-bin/carddisp.pl?gene=MIRLET7G |
| TYRP1 | Tyrosinase Related Protein 1 | Protein Coding | 45 | GC09P012683 | 1.329633 | https://www.genecards.org/cgi-bin/carddisp.pl?gene=TYRP1 |
| IFNAR2 | Interferon Alpha And Beta Receptor Subunit 2 | Protein Coding | 47 | GC21P033229 | 1.329088 | https://www.genecards.org/cgi-bin/carddisp.pl?gene=IFNAR2 |

| | | | | | | |
|---|---|---|---|---|---|---|
| RNASET2 | Ribonuclease T2 | Protein Coding | 43 | GC06M166924 | 1.329088 | https://www.genecards.org/cgi-bin/carddisp.pl?gene=RNASET2 |
| DGCR8 | DGCR8 Microprocessor Complex Subunit | Protein Coding | 40 | GC22P020080 | 1.329088 | https://www.genecards.org/cgi-bin/carddisp.pl?gene=DGCR8 |
| SCARF2 | Scavenger Receptor Class F Member 2 | Protein Coding | 40 | GC22M020424 | 1.329088 | https://www.genecards.org/cgi-bin/carddisp.pl?gene=SCARF2 |
| MED15 | Mediator Complex Subunit 15 | Protein Coding | 39 | GC22P035865 | 1.329088 | https://www.genecards.org/cgi-bin/carddisp.pl?gene=MED15 |
| PLCL1 | Phospholipase C Like 1 (Inactive) | Protein Coding | 38 | GC02P197804 | 1.329088 | https://www.genecards.org/cgi-bin/carddisp.pl?gene=PLCL1 |
| TELO2 | Telomere Maintenance 2 | Protein Coding | 38 | GC16P001493 | 1.329088 | https://www.genecards.org/cgi-bin/carddisp.pl?gene=TELO2 |
| DCTPP1 | DCTP Pyrophosphatase 1 | Protein Coding | 35 | GC16M037170 | 1.329088 | https://www.genecards.org/cgi-bin/carddisp.pl?gene=DCTPP1 |
| RMDN2 | Regulator Of Microtubule Dynamics 2 | Protein Coding | 32 | GC02P037923 | 1.329088 | https://www.genecards.org/cgi-bin/carddisp.pl?gene=RMDN2 |
| SEPTIN5 | Septin 5 | Protein Coding | 29 | GC22P019714 | 1.329088 | https://www.genecards.org/cgi-bin/carddisp.pl?gene=SEPTIN5 |
| PHETA1 | PH Domain Containing Endocytic Trafficking Adaptor 1 | Protein Coding | 27 | GC12M111361 | 1.329088 | https://www.genecards.org/cgi-bin/carddisp.pl?gene=PHETA1 |
| PTGES3 | Prostaglandin E Synthase 3 | Protein Coding | 43 | GC12M056866 | 1.328492 | https://www.genecards.org/cgi-bin/carddisp.pl?gene=PTGES3 |
| P2RX6 | Purinergic Receptor P2X 6 | Protein Coding | 41 | GC22P035894 | 1.325502 | https://www.genecards.org/cgi-bin/carddisp.pl?gene=P2RX6 |
| ITPR3 | Inositol 1,4,5-Trisphosphate Receptor Type 3 | Protein Coding | 44 | GC06P033620 | 1.324167 | https://www.genecards.org/cgi-bin/carddisp.pl?gene=ITPR3 |
| AARS2 | Alanyl-TRNA Synthetase 2, Mitochondrial | Protein Coding | 42 | GC06M066237 | 1.315896 | https://www.genecards.org/cgi- |

| | | | | | | |
|---|---|---|---|---|---|---|
| | | | | | | bin/carddisp.pl?gene=AARS2 |
| POLR2B | RNA Polymerase II Subunit B | Protein Coding | 42 | GC04P056977 | 1.315849 | https://www.genecards.org/cgi-bin/carddisp.pl?gene=POLR2B |
| MAGT1 | Magnesium Transporter 1 | Protein Coding | 39 | GC0XM078008 | 1.315849 | https://www.genecards.org/cgi-bin/carddisp.pl?gene=MAGT1 |
| MT-CO1 | Mitochondrially Encoded Cytochrome C Oxidase I | Protein Coding | 34 | GCMTP005906 | 1.315849 | https://www.genecards.org/cgi-bin/carddisp.pl?gene=MT-CO1 |
| CYTL1 | Cytokine Like 1 | Protein Coding | 32 | GC04M005016 | 1.315397 | https://www.genecards.org/cgi-bin/carddisp.pl?gene=CYTL1 |
| TAF3 | TATA-Box Binding Protein Associated Factor 3 | Protein Coding | 37 | GC10P007860 | 1.312455 | https://www.genecards.org/cgi-bin/carddisp.pl?gene=TAF3 |
| SLC6A4 | Solute Carrier Family 6 Member 4 | Protein Coding | 46 | GC17M030194 | 1.30904 | https://www.genecards.org/cgi-bin/carddisp.pl?gene=SLC6A4 |
| MLANA | Melan-A | Protein Coding | 38 | GC09P006557 | 1.30585 | https://www.genecards.org/cgi-bin/carddisp.pl?gene=MLANA |
| PTS | 6-Pyruvoyltetrahydropterin Synthase | Protein Coding | 44 | GC11P112226 | 1.304664 | https://www.genecards.org/cgi-bin/carddisp.pl?gene=PTS |
| FHL1 | Four And A Half LIM Domains 1 | Protein Coding | 44 | GC0XP136146 | 1.303164 | https://www.genecards.org/cgi-bin/carddisp.pl?gene=FHL1 |
| MIR663A | MicroRNA 663a | RNA Gene | 16 | GC20M026189 | 1.303164 | https://www.genecards.org/cgi-bin/carddisp.pl?gene=MIR663A |
| ZEB1 | Zinc Finger E-Box Binding Homeobox 1 | Protein Coding | 46 | GC10P031318 | 1.302217 | https://www.genecards.org/cgi-bin/carddisp.pl?gene=ZEB1 |
| CCKAR | Cholecystokinin A Receptor | Protein Coding | 44 | GC04M026483 | 1.302217 | https://www.genecards.org/cgi-bin/carddisp.pl?gene=CCKAR |
| NR5A2 | Nuclear Receptor Subfamily 5 Group A Member 2 | Protein Coding | 44 | GC01P199996 | 1.302217 | https://www.genecards.org/cgi-bin/carddisp.pl?gene=NR5A2 |

| | | | | | | |
|---|---|---|---|---|---|---|
| PRDM16 | PR/SET Domain 16 | Protein Coding | 44 | GC01P 003946 | 1.302 217 | https://www.genecards.org/cgi-bin/carddisp.pl?gene=PRDM16 |
| SATB2 | SATB Homeobox 2 | Protein Coding | 43 | GC02M 199269 | 1.302 217 | https://www.genecards.org/cgi-bin/carddisp.pl?gene=SATB2 |
| NDUFS4 | NADH:Ubiquinone Oxidoreductase Subunit S4 | Protein Coding | 42 | GC05P 053560 | 1.302 217 | https://www.genecards.org/cgi-bin/carddisp.pl?gene=NDUFS4 |
| YIPF3 | Yip1 Domain Family Member 3 | Protein Coding | 35 | GC06M 066209 | 1.302 217 | https://www.genecards.org/cgi-bin/carddisp.pl?gene=YIPF3 |
| CCDC15 4 | Coiled-Coil Domain Containing 154 | Protein Coding | 29 | GC16M 001434 | 1.302 217 | https://www.genecards.org/cgi-bin/carddisp.pl?gene=CCDC154 |
| PSMD9 | Proteasome 26S Subunit, Non-ATPase 9 | Protein Coding | 41 | GC12P 126233 | 1.302 004 | https://www.genecards.org/cgi-bin/carddisp.pl?gene=PSMD9 |
| SH3TC2 | SH3 Domain And Tetratricopeptide Repeats 2 | Protein Coding | 37 | GC05M 148923 | 1.302 004 | https://www.genecards.org/cgi-bin/carddisp.pl?gene=SH3TC2 |
| HRG | Histidine Rich Glycoprotein | Protein Coding | 45 | GC03P 186660 | 1.300 443 | https://www.genecards.org/cgi-bin/carddisp.pl?gene=HRG |
| ADCY10 | Adenylate Cyclase 10 | Protein Coding | 43 | GC01M 167809 | 1.299 235 | https://www.genecards.org/cgi-bin/carddisp.pl?gene=ADCY10 |
| ARRB1 | Arrestin Beta 1 | Protein Coding | 42 | GC11M 090410 | 1.290 536 | https://www.genecards.org/cgi-bin/carddisp.pl?gene=ARRB1 |
| CD177 | CD177 Molecule | Protein Coding | 38 | GC19P 043353 | 1.286 208 | https://www.genecards.org/cgi-bin/carddisp.pl?gene=CD177 |
| MAVS | Mitochondrial Antiviral Signaling Protein | Protein Coding | 37 | GC20P 004265 | 1.279 931 | https://www.genecards.org/cgi-bin/carddisp.pl?gene=MAVS |
| ZNF365 | Zinc Finger Protein 365 | Protein Coding | 38 | GC10P 062374 | 1.277 035 | https://www.genecards.org/cgi-bin/carddisp.pl?gene=ZNF365 |
| IRF6 | Interferon Regulatory Factor 6 | Protein Coding | 42 | GC01M 209785 | 1.276 064 | https://www.genecards.org/cgi- |

| | | | | | | |
|---|---|---|---|---|---|---|
| | | | | | | bin/carddisp.pl?gene=IRF6 |
| SNAI1 | Snail Family Transcriptional Repressor 1 | Protein Coding | 42 | GC20P 049982 | 1.275 204 | https://www.genecards.org/cgi-bin/carddisp.pl?gene=SNAI1 |
| PTBP1 | Polypyrimidine Tract Binding Protein 1 | Protein Coding | 41 | GC19P 000797 | 1.275 03 | https://www.genecards.org/cgi-bin/carddisp.pl?gene=PTBP1 |
| CARD16 | Caspase Recruitment Domain Family Member 16 | Protein Coding | 35 | GC11M 105041 | 1.268 728 | https://www.genecards.org/cgi-bin/carddisp.pl?gene=CARD16 |
| VKORC1 | Vitamin K Epoxide Reductase Complex Subunit 1 | Protein Coding | 46 | GC16M 037224 | 1.265 75 | https://www.genecards.org/cgi-bin/carddisp.pl?gene=VKORC1 |
| TSPAN8 | Tetraspanin 8 | Protein Coding | 38 | GC12M 071125 | 1.264 643 | https://www.genecards.org/cgi-bin/carddisp.pl?gene=TSPAN8 |
| RPS27A | Ribosomal Protein S27a | Protein Coding | 41 | GC02P 055231 | 1.264 195 | https://www.genecards.org/cgi-bin/carddisp.pl?gene=RPS27A |
| PAK1 | P21 (RAC1) Activated Kinase 1 | Protein Coding | 46 | GC11M 090480 | 1.263 135 | https://www.genecards.org/cgi-bin/carddisp.pl?gene=PAK1 |
| MIR379 | MicroRNA 379 | RNA Gene | 17 | GC14P 109984 | 1.259 84 | https://www.genecards.org/cgi-bin/carddisp.pl?gene=MIR379 |
| ALOX12 | Arachidonate 12-Lipoxygenase, 12S Type | Protein Coding | 43 | GC17P 006995 | 1.259 021 | https://www.genecards.org/cgi-bin/carddisp.pl?gene=ALOX12 |
| AZU1 | Azurocidin 1 | Protein Coding | 39 | GC19P 000825 | 1.255 604 | https://www.genecards.org/cgi-bin/carddisp.pl?gene=AZU1 |
| DOCK8 | Dedicator Of Cytokinesis 8 | Protein Coding | 43 | GC09P 000214 | 1.251 442 | https://www.genecards.org/cgi-bin/carddisp.pl?gene=DOCK8 |
| HMGCR | 3-Hydroxy-3-Methylglutaryl-CoA Reductase | Protein Coding | 45 | GC05P 075336 | 1.250 626 | https://www.genecards.org/cgi-bin/carddisp.pl?gene=HMGCR |
| ADAMT S1 | ADAM Metallopeptidase With Thrombospondin Type 1 Motif 1 | Protein Coding | 44 | GC21M 026835 | 1.250 154 | https://www.genecards.org/cgi-bin/carddisp.pl?gene=ADAMTS1 |

| | | | | | | |
|---|---|---|---|---|---|---|
| SACS | Sacsin Molecular Chaperone | Protein Coding | 37 | GC13M 023288 | 1.249 107 | https://www.genecards.org/cgi-bin/carddisp.pl?gene=SACS |
| MSX1 | Msh Homeobox 1 | Protein Coding | 44 | GC04P 004861 | 1.248 302 | https://www.genecards.org/cgi-bin/carddisp.pl?gene=MSX1 |
| MIR197 | MicroRNA 197 | RNA Gene | 20 | GC01P 109549 | 1.240 171 | https://www.genecards.org/cgi-bin/carddisp.pl?gene=MIR197 |
| NUP160 | Nucleoporin 160 | Protein Coding | 39 | GC11M 089379 | 1.235 978 | https://www.genecards.org/cgi-bin/carddisp.pl?gene=NUP160 |
| PTPN1 | Protein Tyrosine Phosphatase Non-Receptor Type 1 | Protein Coding | 48 | GC20P 050510 | 1.233 077 | https://www.genecards.org/cgi-bin/carddisp.pl?gene=PTPN1 |
| NARS1 | Asparaginyl-TRNA Synthetase 1 | Protein Coding | 34 | GC18M 057601 | 1.230 197 | https://www.genecards.org/cgi-bin/carddisp.pl?gene=NARS1 |
| SLC9A3 R1 | SLC9A3 Regulator 1 | Protein Coding | 44 | GC17P 074749 | 1.225 507 | https://www.genecards.org/cgi-bin/carddisp.pl?gene=SLC9A3R1 |
| PMP22 | Peripheral Myelin Protein 22 | Protein Coding | 41 | GC17M 015229 | 1.222 336 | https://www.genecards.org/cgi-bin/carddisp.pl?gene=PMP22 |
| CD200R1 L | CD200 Receptor 1 Like | Protein Coding | 29 | GC03M 112815 | 1.219 312 | https://www.genecards.org/cgi-bin/carddisp.pl?gene=CD200R1L |
| LINC019 96 | Long Intergenic Non-Protein Coding RNA 1996 | RNA Gene | 10 | GC17P 004611 | 1.219 312 | https://www.genecards.org/cgi-bin/carddisp.pl?gene=LINC01996 |
| C17orf11 4 | Chromosome 17 Open Reading Frame 114 | Protein Coding | 9 | GC17M 004806 | 1.219 312 | https://www.genecards.org/cgi-bin/carddisp.pl?gene=C17orf114 |
| RGS1 | Regulator Of G Protein Signaling 1 | Protein Coding | 37 | GC01P 192575 | 1.210 462 | https://www.genecards.org/cgi-bin/carddisp.pl?gene=RGS1 |
| UCP2 | Uncoupling Protein 2 | Protein Coding | 44 | GC11M 073974 | 1.210 295 | https://www.genecards.org/cgi-bin/carddisp.pl?gene=UCP2 |
| PEBP1 | Phosphatidylethanolamine Binding Protein 1 | Protein Coding | 43 | GC12P 118135 | 1.209 291 | https://www.genecards.org/cgi- |

| | | | | | | |
|---|---|---|---|---|---|---|
| | | | | | | bin/carddisp.pl?gene=PEBP1 |
| PTPRJ | Protein Tyrosine Phosphatase Receptor Type J | Protein Coding | 46 | GC11P048002 | 1.209265 | https://www.genecards.org/cgi-bin/carddisp.pl?gene=PTPRJ |
| PRKCZ | Protein Kinase C Zeta | Protein Coding | 47 | GC01P003912 | 1.208204 | https://www.genecards.org/cgi-bin/carddisp.pl?gene=PRKCZ |
| CTTN | Cortactin | Protein Coding | 41 | GC11P070398 | 1.207488 | https://www.genecards.org/cgi-bin/carddisp.pl?gene=CTTN |
| LCP2 | Lymphocyte Cytosolic Protein 2 | Protein Coding | 41 | GC05M170246 | 1.207488 | https://www.genecards.org/cgi-bin/carddisp.pl?gene=LCP2 |
| AMT | Aminomethyltransferase | Protein Coding | 44 | GC03M051187 | 1.206704 | https://www.genecards.org/cgi-bin/carddisp.pl?gene=AMT |
| PPIF | Peptidylprolyl Isomerase F | Protein Coding | 43 | GC10P092351 | 1.206704 | https://www.genecards.org/cgi-bin/carddisp.pl?gene=PPIF |
| PEX13 | Peroxisomal Biogenesis Factor 13 | Protein Coding | 40 | GC02P061017 | 1.206704 | https://www.genecards.org/cgi-bin/carddisp.pl?gene=PEX13 |
| REV1 | REV1 DNA Directed Polymerase | Protein Coding | 40 | GC02M099383 | 1.206704 | https://www.genecards.org/cgi-bin/carddisp.pl?gene=REV1 |
| DHX16 | DEAH-Box Helicase 16 | Protein Coding | 39 | GC06M030653 | 1.206704 | https://www.genecards.org/cgi-bin/carddisp.pl?gene=DHX16 |
| GTF2H4 | General Transcription Factor IIH Subunit 4 | Protein Coding | 39 | GC06P083679 | 1.206704 | https://www.genecards.org/cgi-bin/carddisp.pl?gene=GTF2H4 |
| LIMD1 | LIM Domain Containing 1 | Protein Coding | 38 | GC03P045555 | 1.206704 | https://www.genecards.org/cgi-bin/carddisp.pl?gene=LIMD1 |
| PAPOLG | Poly(A) Polymerase Gamma | Protein Coding | 38 | GC02P060756 | 1.206704 | https://www.genecards.org/cgi-bin/carddisp.pl?gene=PAPOLG |
| RASSF5 | Ras Association Domain Family Member 5 | Protein Coding | 38 | GC01P206507 | 1.206704 | https://www.genecards.org/cgi-bin/carddisp.pl?gene=RASSF5 |

| | | | | | | |
|---|---|---|---|---|---|---|
| CARD6 | Caspase Recruitment Domain Family Member 6 | Protein Coding | 37 | GC05P 040841 | 1.206 704 | https://www.genecards.org/cgi-bin/carddisp.pl?gene=CARD6 |
| PSMG1 | Proteasome Assembly Chaperone 1 | Protein Coding | 37 | GC21M 039174 | 1.206 704 | https://www.genecards.org/cgi-bin/carddisp.pl?gene=PSMG1 |
| TCF19 | Transcription Factor 19 | Protein Coding | 37 | GC06P 083684 | 1.206 704 | https://www.genecards.org/cgi-bin/carddisp.pl?gene=TCF19 |
| IKZF4 | IKAROS Family Zinc Finger 4 | Protein Coding | 36 | GC12P 056007 | 1.206 704 | https://www.genecards.org/cgi-bin/carddisp.pl?gene=IKZF4 |
| NRBF2 | Nuclear Receptor Binding Factor 2 | Protein Coding | 36 | GC10P 063133 | 1.206 704 | https://www.genecards.org/cgi-bin/carddisp.pl?gene=NRBF2 |
| SH3BP1 | SH3 Domain Binding Protein 1 | Protein Coding | 36 | GC22P 037634 | 1.206 704 | https://www.genecards.org/cgi-bin/carddisp.pl?gene=SH3BP1 |
| LRRC2 | Leucine Rich Repeat Containing 2 | Protein Coding | 35 | GC03M 046515 | 1.206 704 | https://www.genecards.org/cgi-bin/carddisp.pl?gene=LRRC2 |
| PUS10 | Pseudouridine Synthase 10 | Protein Coding | 35 | GC02M 060940 | 1.206 704 | https://www.genecards.org/cgi-bin/carddisp.pl?gene=PUS10 |
| ZBTB7C | Zinc Finger And BTB Domain Containing 7C | Protein Coding | 33 | GC18M 048026 | 1.206 704 | https://www.genecards.org/cgi-bin/carddisp.pl?gene=ZBTB7C |
| TTC33 | Tetratricopeptide Repeat Domain 33 | Protein Coding | 32 | GC05M 040512 | 1.206 704 | https://www.genecards.org/cgi-bin/carddisp.pl?gene=TTC33 |
| INKA1 | Inka Box Actin Regulator 1 | Protein Coding | 24 | GC03P 049803 | 1.206 704 | https://www.genecards.org/cgi-bin/carddisp.pl?gene=INKA1 |
| AHSA2P | Activator Of HSP90 ATPase Homolog 2, Pseudogene | Pseudo gene | 22 | GC02P 061179 | 1.206 704 | https://www.genecards.org/cgi-bin/carddisp.pl?gene=AHSA2P |
| CCR5AS | CCR5 Antisense RNA | RNA Gene | 12 | GC03M 046365 | 1.206 704 | https://www.genecards.org/cgi-bin/carddisp.pl?gene=CCR5AS |
| IL6ST-DT | IL6ST Divergent Transcript | RNA Gene | 12 | GC05P 055996 | 1.206 704 | https://www.genecards.org/cgi- |

| | | | | | | |
|---|---|---|---|---|---|---|
| | | | | | | bin/carddisp.pl?gene=IL6ST-DT |
| NONOP2 | Non-POU Domain Containing, Octamer-Binding Pseudogene 2 | Pseudo gene | 10 | GC02M 060937 | 1.206 704 | https://www.genecards.org/cgi-bin/carddisp.pl?gene=NONOP2 |
| USP34-DT | USP34 Divergent Transcript | RNA Gene | 10 | GC02P 061473 | 1.206 704 | https://www.genecards.org/cgi-bin/carddisp.pl?gene=USP34-DT |
| ALDH7A1P4 | Aldehyde Dehydrogenase 7 Family Member A1 Pseudogene 4 | Pseudo gene | 9 | GC10M 062740 | 1.206 704 | https://www.genecards.org/cgi-bin/carddisp.pl?gene=ALDH7A1P4 |
| ENSG00000235888 | Novel Transcript | RNA Gene | 9 | GC21M 038988 | 1.206 704 | https://www.genecards.org/cgi-bin/carddisp.pl?gene=ENSG00000235888 |
| RPS12P3 | Ribosomal Protein S12 Pseudogene 3 | Pseudo gene | 9 | GC02M 060938 | 1.206 704 | https://www.genecards.org/cgi-bin/carddisp.pl?gene=RPS12P3 |
| RPS15AP38 | Ribosomal Protein S15a Pseudogene 38 | Pseudo gene | 9 | GC22M 036421 | 1.206 704 | https://www.genecards.org/cgi-bin/carddisp.pl?gene=RPS15AP38 |
| ENSG00000199332 | Y RNA | RNA Gene | 8 | GC06M 065930 | 1.206 704 | https://www.genecards.org/cgi-bin/carddisp.pl?gene=ENSG00000199332 |
| ENSG00000267520 | Novel Transcript | RNA Gene | 8 | GC02P 060925 | 1.206 704 | https://www.genecards.org/cgi-bin/carddisp.pl?gene=ENSG00000267520 |
| ENSG00000272221 | Novel Transcript | RNA Gene | 8 | GC06M 065927 | 1.206 704 | https://www.genecards.org/cgi-bin/carddisp.pl?gene=ENSG00000272221 |
| LOC124901197 | Small Nucleolar RNA U13 | RNA Gene | 8 | GC05M 056001 | 1.206 704 | https://www.genecards.org/cgi-bin/carddisp.pl?gene=LOC124901197 |
| RN7SL51P | RNA, 7SL, Cytoplasmic 51, Pseudogene | Pseudo gene | 8 | GC02M 062262 | 1.206 704 | https://www.genecards.org/cgi-bin/carddisp.pl?gene=RN7SL51P |
| RNA5SP184 | RNA, 5S Ribosomal Pseudogene 184 | Pseudo gene | 8 | GC05M 056146 | 1.206 704 | https://www.genecards.org/cgi-bin/carddisp.pl?gene=RNA5SP184 |
| RNU6-299P | RNA, U6 Small Nuclear 299, Pseudogene | Pseudo gene | 8 | GC05P 056125 | 1.206 704 | https://www.genecards.org/cgi-bin/carddisp.pl?gene=RNU6-299P |

| | | | | | | |
|---|---|---|---|---|---|---|
| RNU6-850P | RNA, U6 Small Nuclear 850, Pseudogene | Pseudo gene | 8 | GC06M 031756 | 1.206 704 | https://www.genecards.org/cgi-bin/carddisp.pl?gene=RNU6-850P |
| RPL23AP 12 | Ribosomal Protein L23a Pseudogene 12 | Pseudo gene | 8 | GC21P 039127 | 1.206 704 | https://www.genecards.org/cgi-bin/carddisp.pl?gene=RPL23AP12 |
| AIMP1P2 | Aminoacyl TRNA Synthetase Complex Interacting Multifunctional Protein 1 Pseudogene 2 | Pseudo gene | 7 | GC01M 172856 | 1.206 704 | https://www.genecards.org/cgi-bin/carddisp.pl?gene=AIMP1P2 |
| HNRNPA 1P41 | Heterogeneous Nuclear Ribonucleoprotein A1 Pseudogene 41 | Pseudo gene | 6 | GC09M 004946 | 1.206 704 | https://www.genecards.org/cgi-bin/carddisp.pl?gene=HNRNPA1P41 |
| ENSG000 00285552 | Novel Transcript | RNA Gene | 5 | GC05P 040463 | 1.206 704 | https://www.genecards.org/cgi-bin/carddisp.pl?gene=ENSG00000285552 |
| ENSG000 00285616 | Novel Transcript | RNA Gene | 5 | GC05M 040474 | 1.206 704 | https://www.genecards.org/cgi-bin/carddisp.pl?gene=ENSG00000285616 |
| MG828730-053 | | RNA Gene | 5 | GC06M 067894 | 1.206 704 | https://www.genecards.org/cgi-bin/carddisp.pl?gene=MG828730-053 |
| NONHSAG034948.2 | | RNA Gene | 5 | GC03P 046242 | 1.206 704 | https://www.genecards.org/cgi-bin/carddisp.pl?gene=NONHSAG034948.2 |
| RF00017-4256 | | RNA Gene | 5 | GC05P 055987 | 1.206 704 | https://www.genecards.org/cgi-bin/carddisp.pl?gene=RF00017-4256 |
| RF00017-5028 | | RNA Gene | 5 | GC06M 068702 | 1.206 704 | https://www.genecards.org/cgi-bin/carddisp.pl?gene=RF00017-5028 |
| lnc-AFF3-1 | | RNA Gene | 5 | GC02M 100109 | 1.206 704 | https://www.genecards.org/cgi-bin/carddisp.pl?gene=lnc-AFF3-1 |
| lnc-IL31RA-5 | | RNA Gene | 5 | GC05P 056122 | 1.206 704 | https://www.genecards.org/cgi-bin/carddisp.pl?gene=lnc-IL31RA-5 |
| lnc-IL6ST-2 | | RNA Gene | 5 | GC05M 056111 | 1.206 704 | https://www.genecards.org/cgi-bin/carddisp.pl?gene=lnc-IL6ST-2 |

| | | | | | |
|---|---|---|---|---|---|
| lnc-RAB5B-2 | RNA Gene | 5 | GC12P 055998 | 1.206 704 | https://www.genecards.org/cgi-bin/carddisp.pl?gene=lnc-RAB5B-2 |
| lnc-ZCCHC24-7 | RNA Gene | 5 | GC10M 079298 | 1.206 704 | https://www.genecards.org/cgi-bin/carddisp.pl?gene=lnc-ZCCHC24-7 |
| HSALNG0015261 | RNA Gene | 4 | GC02M 060924 | 1.206 704 | https://www.genecards.org/cgi-bin/carddisp.pl?gene=HSALNG0015261 |
| HSALNG0017286 | RNA Gene | 4 | GC02P 100109 | 1.206 704 | https://www.genecards.org/cgi-bin/carddisp.pl?gene=HSALNG0017286 |
| HSALNG0069758 | RNA Gene | 4 | GC09M 004978 | 1.206 704 | https://www.genecards.org/cgi-bin/carddisp.pl?gene=HSALNG0069758 |
| HSALNG0079128 | RNA Gene | 4 | GC10P 092600 | 1.206 704 | https://www.genecards.org/cgi-bin/carddisp.pl?gene=HSALNG0079128 |
| HSALNG0091478 | RNA Gene | 4 | GC12P 056006 | 1.206 704 | https://www.genecards.org/cgi-bin/carddisp.pl?gene=HSALNG0091478 |
| HSALNG0133104 | RNA Gene | 4 | GC21M 039035 | 1.206 704 | https://www.genecards.org/cgi-bin/carddisp.pl?gene=HSALNG0133104 |
| MK280144-361 | RNA Gene | 4 | GC02P 100121 | 1.206 704 | https://www.genecards.org/cgi-bin/carddisp.pl?gene=MK280144-361 |
| MN309188 | RNA Gene | 4 | GC10M 079292 | 1.206 704 | https://www.genecards.org/cgi-bin/carddisp.pl?gene=MN309188 |
| RF00001-253 | RNA Gene | 4 | GC05M 056283 | 1.206 704 | https://www.genecards.org/cgi-bin/carddisp.pl?gene=RF00001-253 |
| RF00017-3020 | RNA Gene | 4 | GC02M 099997 | 1.206 704 | https://www.genecards.org/cgi-bin/carddisp.pl?gene=RF00017-3020 |
| RF00017-3680 | RNA Gene | 4 | GC03P 049643 | 1.206 704 | https://www.genecards.org/cgi-bin/carddisp.pl?gene=RF00017-3680 |
| RF00017-4260 | RNA Gene | 4 | GC05P 056157 | 1.206 704 | https://www.genecards.org/cgi- |

| | | | | | | |
|---|---|---|---|---|---|---|
| | | | | | | bin/carddisp.pl?gene=RF00017-4260 |
| lnc-TTC33-6 | | RNA Gene | 4 | GC05M040484 | 1.206704 | https://www.genecards.org/cgi-bin/carddisp.pl?gene=lnc-TTC33-6 |
| piR-43099-059 | | RNA Gene | 4 | GC10M079279 | 1.206704 | https://www.genecards.org/cgi-bin/carddisp.pl?gene=piR-43099-059 |
| piR-58297-114 | | RNA Gene | 4 | GC12M055999 | 1.206704 | https://www.genecards.org/cgi-bin/carddisp.pl?gene=piR-58297-114 |
| piR-58029-003 | | RNA Gene | 3 | GC02M060941 | 1.206704 | https://www.genecards.org/cgi-bin/carddisp.pl?gene=piR-58029-003 |
| RARS1 | Arginyl-TRNA Synthetase 1 | Protein Coding | 35 | GC05P168487 | 1.20346 | https://www.genecards.org/cgi-bin/carddisp.pl?gene=RARS1 |
| C2 | Complement C2 | Protein Coding | 46 | GC06P031897 | 1.202754 | https://www.genecards.org/cgi-bin/carddisp.pl?gene=C2 |
| NR4A1 | Nuclear Receptor Subfamily 4 Group A Member 1 | Protein Coding | 45 | GC12P052022 | 1.199172 | https://www.genecards.org/cgi-bin/carddisp.pl?gene=NR4A1 |
| STAT2 | Signal Transducer And Activator Of Transcription 2 | Protein Coding | 45 | GC12M056341 | 1.196838 | https://www.genecards.org/cgi-bin/carddisp.pl?gene=STAT2 |
| NPHS2 | NPHS2 Stomatin Family Member, Podocin | Protein Coding | 41 | GC01M179519 | 1.196341 | https://www.genecards.org/cgi-bin/carddisp.pl?gene=NPHS2 |
| NOX1 | NADPH Oxidase 1 | Protein Coding | 41 | GC0XM100843 | 1.195215 | https://www.genecards.org/cgi-bin/carddisp.pl?gene=NOX1 |
| CCNA2 | Cyclin A2 | Protein Coding | 44 | GC04M121816 | 1.194973 | https://www.genecards.org/cgi-bin/carddisp.pl?gene=CCNA2 |
| CSF2RA | Colony Stimulating Factor 2 Receptor Subunit Alpha | Protein Coding | 43 | GC0XP001676 | 1.186367 | https://www.genecards.org/cgi-bin/carddisp.pl?gene=CSF2RA |
| TPT1 | Tumor Protein, Translationally-Controlled 1 | Protein Coding | 45 | GC13M045333 | 1.185415 | https://www.genecards.org/cgi-bin/carddisp.pl?gene=TPT1 |

| | | | | | | |
|---|---|---|---|---|---|---|
| LMO2 | LIM Domain Only 2 | Protein Coding | 41 | GC11M 033858 | 1.185 415 | https://www.genecards.org/cgi-bin/carddisp.pl?gene=LMO2 |
| SERPINB 5 | Serpin Family B Member 5 | Protein Coding | 41 | GC18P 063476 | 1.185 415 | https://www.genecards.org/cgi-bin/carddisp.pl?gene=SERPINB5 |
| TRADD | TNFRSF1A Associated Via Death Domain | Protein Coding | 41 | GC16M 067154 | 1.185 415 | https://www.genecards.org/cgi-bin/carddisp.pl?gene=TRADD |
| DDX18 | DEAD-Box Helicase 18 | Protein Coding | 40 | GC02P 122504 | 1.185 415 | https://www.genecards.org/cgi-bin/carddisp.pl?gene=DDX18 |
| RPN2 | Ribophorin II | Protein Coding | 40 | GC20P 037178 | 1.185 415 | https://www.genecards.org/cgi-bin/carddisp.pl?gene=RPN2 |
| NAV2 | Neuron Navigator 2 | Protein Coding | 38 | GC11P 019345 | 1.185 415 | https://www.genecards.org/cgi-bin/carddisp.pl?gene=NAV2 |
| SARM1 | Sterile Alpha And TIR Motif Containing 1 | Protein Coding | 38 | GC17P 028364 | 1.185 415 | https://www.genecards.org/cgi-bin/carddisp.pl?gene=SARM1 |
| SLC30A6 | Solute Carrier Family 30 Member 6 | Protein Coding | 37 | GC02P 032166 | 1.185 415 | https://www.genecards.org/cgi-bin/carddisp.pl?gene=SLC30A6 |
| BTN3A2 | Butyrophilin Subfamily 3 Member A2 | Protein Coding | 36 | GC06P 026365 | 1.185 415 | https://www.genecards.org/cgi-bin/carddisp.pl?gene=BTN3A2 |
| E2F3-IT1 | E2F3 Intronic Transcript 1 | RNA Gene | 12 | GC06P 020403 | 1.185 415 | https://www.genecards.org/cgi-bin/carddisp.pl?gene=E2F3-IT1 |
| SKP1 | S-Phase Kinase Associated Protein 1 | Protein Coding | 41 | GC05M 134148 | 1.182 462 | https://www.genecards.org/cgi-bin/carddisp.pl?gene=SKP1 |
| RPL3 | Ribosomal Protein L3 | Protein Coding | 40 | GC22M 057593 | 1.182 462 | https://www.genecards.org/cgi-bin/carddisp.pl?gene=RPL3 |
| UBR5 | Ubiquitin Protein Ligase E3 Component N-Recognin 5 | Protein Coding | 40 | GC08M 102252 | 1.182 462 | https://www.genecards.org/cgi-bin/carddisp.pl?gene=UBR5 |
| ORMDL3 | ORMDL Sphingolipid Biosynthesis Regulator 3 | Protein Coding | 39 | GC17M 039921 | 1.182 462 | https://www.genecards.org/cgi- |

| | | | | | | |
|---|---|---|---|---|---|---|
| | | | | | | bin/carddisp.pl?gene=ORMDL3 |
| CDH22 | Cadherin 22 | Protein Coding | 38 | GC20M046173 | 1.182462 | https://www.genecards.org/cgi-bin/carddisp.pl?gene=CDH22 |
| ICAM4 | Intercellular Adhesion Molecule 4 (Landsteiner-Wiener Blood Group) | Protein Coding | 36 | GC19P010286 | 1.182462 | https://www.genecards.org/cgi-bin/carddisp.pl?gene=ICAM4 |
| TSPAN33 | Tetraspanin 33 | Protein Coding | 36 | GC07P131929 | 1.182462 | https://www.genecards.org/cgi-bin/carddisp.pl?gene=TSPAN33 |
| ZNF318 | Zinc Finger Protein 318 | Protein Coding | 35 | GC06M066206 | 1.182462 | https://www.genecards.org/cgi-bin/carddisp.pl?gene=ZNF318 |
| UMODL1 | Uromodulin Like 1 | Protein Coding | 34 | GC21P042062 | 1.182462 | https://www.genecards.org/cgi-bin/carddisp.pl?gene=UMODL1 |
| CTIF | Cap Binding Complex Dependent Translation Initiation Factor | Protein Coding | 32 | GC18P048539 | 1.182462 | https://www.genecards.org/cgi-bin/carddisp.pl?gene=CTIF |
| CEP170B | Centrosomal Protein 170B | Protein Coding | 30 | GC14P109737 | 1.182462 | https://www.genecards.org/cgi-bin/carddisp.pl?gene=CEP170B |
| STXBP2 | Syntaxin Binding Protein 2 | Protein Coding | 44 | GC19P007856 | 1.181562 | https://www.genecards.org/cgi-bin/carddisp.pl?gene=STXBP2 |
| THAP1 | THAP Domain Containing 1 | Protein Coding | 39 | GC08M042836 | 1.181562 | https://www.genecards.org/cgi-bin/carddisp.pl?gene=THAP1 |
| CPB2 | Carboxypeptidase B2 | Protein Coding | 44 | GC13M046053 | 1.180554 | https://www.genecards.org/cgi-bin/carddisp.pl?gene=CPB2 |
| DRD2 | Dopamine Receptor D2 | Protein Coding | 48 | GC11M113409 | 1.180007 | https://www.genecards.org/cgi-bin/carddisp.pl?gene=DRD2 |
| PCBD1 | Pterin-4 Alpha-Carbinolamine Dehydratase 1 | Protein Coding | 43 | GC10M070882 | 1.172204 | https://www.genecards.org/cgi-bin/carddisp.pl?gene=PCBD1 |
| MCHR1 | Melanin Concentrating Hormone Receptor 1 | Protein Coding | 40 | GC22P040679 | 1.172204 | https://www.genecards.org/cgi-bin/carddisp.pl?gene=MCHR1 |

| | | | | | | |
|---|---|---|---|---|---|---|
| MAGEA1 | MAGE Family Member A1 | Protein Coding | 37 | GC0XP 153179 | 1.169 714 | https://www.genecards .org/cgi-bin/carddisp.pl?gene= MAGEA1 |
| TRO | Trophinin | Protein Coding | 37 | GC0XP 054920 | 1.169 714 | https://www.genecards .org/cgi-bin/carddisp.pl?gene= TRO |
| GNRH1 | Gonadotropin Releasing Hormone 1 | Protein Coding | 39 | GC08M 025419 | 1.167 608 | https://www.genecards .org/cgi-bin/carddisp.pl?gene= GNRH1 |
| FGF8 | Fibroblast Growth Factor 8 | Protein Coding | 45 | GC10M 101770 | 1.164 202 | https://www.genecards .org/cgi-bin/carddisp.pl?gene= FGF8 |
| TXNIP | Thioredoxin Interacting Protein | Protein Coding | 37 | GC01M 145992 | 1.162 784 | https://www.genecards .org/cgi-bin/carddisp.pl?gene= TXNIP |
| CXCL3 | C-X-C Motif Chemokine Ligand 3 | Protein Coding | 36 | GC04M 074036 | 1.161 772 | https://www.genecards .org/cgi-bin/carddisp.pl?gene= CXCL3 |
| INPP5D | Inositol Polyphosphate-5-Phosphatase D | Protein Coding | 44 | GC02P 233059 | 1.161 755 | https://www.genecards .org/cgi-bin/carddisp.pl?gene=I NPP5D |
| HIP1 | Huntingtin Interacting Protein 1 | Protein Coding | 40 | GC07M 075533 | 1.161 755 | https://www.genecards .org/cgi-bin/carddisp.pl?gene= HIP1 |
| CORD1 | Cone Rod Dystrophy 1 (Autosomal Dominant) | Geneti c Locus | 4 | GC18U 990008 | 1.161 755 | https://www.genecards .org/cgi-bin/carddisp.pl?gene= CORD1 |
| LRG1 | Leucine Rich Alpha-2-Glycoprotein 1 | Protein Coding | 39 | GC19M 005467 | 1.159 959 | https://www.genecards .org/cgi-bin/carddisp.pl?gene= LRG1 |
| FYN | FYN Proto-Oncogene, Src Family Tyrosine Kinase | Protein Coding | 45 | GC06M 111660 | 1.153 29 | https://www.genecards .org/cgi-bin/carddisp.pl?gene= FYN |
| MORC3 | MORC Family CW-Type Zinc Finger 3 | Protein Coding | 34 | GC21P 036320 | 1.153 072 | https://www.genecards .org/cgi-bin/carddisp.pl?gene= MORC3 |
| DPP6 | Dipeptidyl Peptidase Like 6 | Protein Coding | 44 | GC07P 153748 | 1.151 047 | https://www.genecards .org/cgi-bin/carddisp.pl?gene= DPP6 |
| COL4A4 | Collagen Type IV Alpha 4 Chain | Protein Coding | 43 | GC02M 226973 | 1.151 047 | https://www.genecards .org/cgi- |

| | | | | | | |
|---|---|---|---|---|---|---|
| | | | | | | bin/carddisp.pl?gene=COL4A4 |
| SEPSECS | Sep (O-Phosphoserine) TRNA:Sec (Selenocysteine) TRNA Synthase | Protein Coding | 42 | GC04M025121 | 1.151047 | https://www.genecards.org/cgi-bin/carddisp.pl?gene=SEPSECS |
| FST | Follistatin | Protein Coding | 45 | GC05P053480 | 1.146774 | https://www.genecards.org/cgi-bin/carddisp.pl?gene=FST |
| CCNB1 | Cyclin B1 | Protein Coding | 44 | GC05P069167 | 1.142886 | https://www.genecards.org/cgi-bin/carddisp.pl?gene=CCNB1 |
| ABCA3 | ATP Binding Cassette Subfamily A Member 3 | Protein Coding | 48 | GC16M002275 | 1.142593 | https://www.genecards.org/cgi-bin/carddisp.pl?gene=ABCA3 |
| KRT7 | Keratin 7 | Protein Coding | 41 | GC12P052232 | 1.142593 | https://www.genecards.org/cgi-bin/carddisp.pl?gene=KRT7 |
| FPR1 | Formyl Peptide Receptor 1 | Protein Coding | 46 | GC19M051745 | 1.141736 | https://www.genecards.org/cgi-bin/carddisp.pl?gene=FPR1 |
| ATP4A | ATPase H+/K+ Transporting Subunit Alpha | Protein Coding | 40 | GC19M066540 | 1.135156 | https://www.genecards.org/cgi-bin/carddisp.pl?gene=ATP4A |
| IGLL5 | Immunoglobulin Lambda Like Polypeptide 5 | Protein Coding | 29 | GC22P036016 | 1.134276 | https://www.genecards.org/cgi-bin/carddisp.pl?gene=IGLL5 |
| MIR486-1 | MicroRNA 486-1 | RNA Gene | 18 | GC08M041660 | 1.132564 | https://www.genecards.org/cgi-bin/carddisp.pl?gene=MIR486-1 |
| HCP5 | HLA Complex P5 | RNA Gene | 29 | GC06P031400 | 1.131999 | https://www.genecards.org/cgi-bin/carddisp.pl?gene=HCP5 |
| NOTCH3 | Notch Receptor 3 | Protein Coding | 48 | GC19M015159 | 1.131546 | https://www.genecards.org/cgi-bin/carddisp.pl?gene=NOTCH3 |
| TPSB2 | Tryptase Beta 2 | Protein Coding | 39 | GC16M001227 | 1.128264 | https://www.genecards.org/cgi-bin/carddisp.pl?gene=TPSB2 |
| KIF21B | Kinesin Family Member 21B | Protein Coding | 35 | GC01M200938 | 1.128264 | https://www.genecards.org/cgi-bin/carddisp.pl?gene=KIF21B |

| | | | | | | |
|---|---|---|---|---|---|---|
| MEST | Mesoderm Specific Transcript | Protein Coding | 40 | GC07P130486 | 1.125553 | https://www.genecards.org/cgi-bin/carddisp.pl?gene=MEST |
| C1GALT1C1 | C1GALT1 Specific Chaperone 1 | Protein Coding | 36 | GC0XM120625 | 1.125553 | https://www.genecards.org/cgi-bin/carddisp.pl?gene=C1GALT1C1 |
| MME | Membrane Metalloendopeptidase | Protein Coding | 48 | GC03P155024 | 1.1255 | https://www.genecards.org/cgi-bin/carddisp.pl?gene=MME |
| TNNT3 | Troponin T3, Fast Skeletal Type | Protein Coding | 42 | GC11P001920 | 1.123285 | https://www.genecards.org/cgi-bin/carddisp.pl?gene=TNNT3 |
| SLC16A4 | Solute Carrier Family 16 Member 4 | Protein Coding | 38 | GC01M110362 | 1.122542 | https://www.genecards.org/cgi-bin/carddisp.pl?gene=SLC16A4 |
| TP53AIP1 | Tumor Protein P53 Regulated Apoptosis Inducing Protein 1 | Protein Coding | 34 | GC11M128934 | 1.121662 | https://www.genecards.org/cgi-bin/carddisp.pl?gene=TP53AIP1 |
| FOSL2 | FOS Like 2, AP-1 Transcription Factor Subunit | Protein Coding | 40 | GC02P028392 | 1.118083 | https://www.genecards.org/cgi-bin/carddisp.pl?gene=FOSL2 |
| TDRD9 | Tudor Domain Containing 9 | Protein Coding | 39 | GC14P109720 | 1.116959 | https://www.genecards.org/cgi-bin/carddisp.pl?gene=TDRD9 |
| ANXA7 | Annexin A7 | Protein Coding | 41 | GC10M073375 | 1.115158 | https://www.genecards.org/cgi-bin/carddisp.pl?gene=ANXA7 |
| NKX2-5 | NK2 Homeobox 5 | Protein Coding | 43 | GC05M173232 | 1.114008 | https://www.genecards.org/cgi-bin/carddisp.pl?gene=NKX2-5 |
| POFUT2 | Protein O-Fucosyltransferase 2 | Protein Coding | 37 | GC21M045263 | 1.114008 | https://www.genecards.org/cgi-bin/carddisp.pl?gene=POFUT2 |
| ZFAT | Zinc Finger And AT-Hook Domain Containing | Protein Coding | 36 | GC08M134477 | 1.114008 | https://www.genecards.org/cgi-bin/carddisp.pl?gene=ZFAT |
| ARHGAP45 | Rho GTPase Activating Protein 45 | Protein Coding | 29 | GC19P001065 | 1.114008 | https://www.genecards.org/cgi-bin/carddisp.pl?gene=ARHGAP45 |
| CENPE | Centromere Protein E | Protein Coding | 44 | GC04M103105 | 1.11168 | https://www.genecards.org/cgi- |

| | | | | | | |
|---|---|---|---|---|---|---|
| | | | | | | bin/carddisp.pl?gene=CENPE |
| INHBA | Inhibin Subunit Beta A | Protein Coding | 44 | GC07M041668 | 1.11168 | https://www.genecards.org/cgi-bin/carddisp.pl?gene=INHBA |
| EDNRA | Endothelin Receptor Type A | Protein Coding | 47 | GC04P147480 | 1.10989 | https://www.genecards.org/cgi-bin/carddisp.pl?gene=EDNRA |
| NM | Neutrophil Migration | Genetic Locus | 5 | GC07U990060 | 1.108623 | https://www.genecards.org/cgi-bin/carddisp.pl?gene=NM |
| VSIR | V-Set Immunoregulatory Receptor | Protein Coding | 32 | GC10M071748 | 1.099166 | https://www.genecards.org/cgi-bin/carddisp.pl?gene=VSIR |
| USP8 | Ubiquitin Specific Peptidase 8 | Protein Coding | 46 | GC15P050424 | 1.097772 | https://www.genecards.org/cgi-bin/carddisp.pl?gene=USP8 |
| KLF2 | Kruppel Like Factor 2 | Protein Coding | 39 | GC19P066201 | 1.094939 | https://www.genecards.org/cgi-bin/carddisp.pl?gene=KLF2 |
| JUND | JunD Proto-Oncogene, AP-1 Transcription Factor Subunit | Protein Coding | 41 | GC19M018279 | 1.093878 | https://www.genecards.org/cgi-bin/carddisp.pl?gene=JUND |
| HTR1A | 5-Hydroxytryptamine Receptor 1A | Protein Coding | 45 | GC05M063960 | 1.092535 | https://www.genecards.org/cgi-bin/carddisp.pl?gene=HTR1A |
| DCT | Dopachrome Tautomerase | Protein Coding | 43 | GC13M094436 | 1.092535 | https://www.genecards.org/cgi-bin/carddisp.pl?gene=DCT |
| CUL5 | Cullin 5 | Protein Coding | 42 | GC11P108008 | 1.092128 | https://www.genecards.org/cgi-bin/carddisp.pl?gene=CUL5 |
| ETV7 | ETS Variant Transcription Factor 7 | Protein Coding | 34 | GC06M066113 | 1.092128 | https://www.genecards.org/cgi-bin/carddisp.pl?gene=ETV7 |
| FGF14 | Fibroblast Growth Factor 14 | Protein Coding | 42 | GC13M101710 | 1.087138 | https://www.genecards.org/cgi-bin/carddisp.pl?gene=FGF14 |
| NEU1 | Neuraminidase 1 | Protein Coding | 44 | GC06M031857 | 1.083123 | https://www.genecards.org/cgi-bin/carddisp.pl?gene=NEU1 |

| | | | | | | |
|---|---|---|---|---|---|---|
| IGHV4-38-2 | Immunoglobulin Heavy Variable 4-38-2 | Protein Coding | 8 | GC14U901616 | 1.076324 | https://www.genecards.org/cgi-bin/carddisp.pl?gene=IGHV4-38-2 |
| KLK7 | Kallikrein Related Peptidase 7 | Protein Coding | 40 | GC19M066197 | 1.073234 | https://www.genecards.org/cgi-bin/carddisp.pl?gene=KLK7 |
| IVL | Involucrin | Protein Coding | 37 | GC01P152909 | 1.073234 | https://www.genecards.org/cgi-bin/carddisp.pl?gene=IVL |
| NR1I2 | Nuclear Receptor Subfamily 1 Group I Member 2 | Protein Coding | 44 | GC03P119780 | 1.072242 | https://www.genecards.org/cgi-bin/carddisp.pl?gene=NR1I2 |
| PSMA3 | Proteasome 20S Subunit Alpha 3 | Protein Coding | 42 | GC14P058244 | 1.071438 | https://www.genecards.org/cgi-bin/carddisp.pl?gene=PSMA3 |
| LASP1 | LIM And SH3 Protein 1 | Protein Coding | 41 | GC17P038869 | 1.071438 | https://www.genecards.org/cgi-bin/carddisp.pl?gene=LASP1 |
| H1-5 | H1.5 Linker Histone, Cluster Member | Protein Coding | 33 | GC06M066505 | 1.071438 | https://www.genecards.org/cgi-bin/carddisp.pl?gene=H1-5 |
| NUP205 | Nucleoporin 205 | Protein Coding | 37 | GC07P135557 | 1.069558 | https://www.genecards.org/cgi-bin/carddisp.pl?gene=NUP205 |
| AIFM1 | Apoptosis Inducing Factor Mitochondria Associated 1 | Protein Coding | 46 | GC0XM130129 | 1.06615 | https://www.genecards.org/cgi-bin/carddisp.pl?gene=AIFM1 |
| KDM1A | Lysine Demethylase 1A | Protein Coding | 46 | GC01P023019 | 1.06615 | https://www.genecards.org/cgi-bin/carddisp.pl?gene=KDM1A |
| TDO2 | Tryptophan 2,3-Dioxygenase | Protein Coding | 44 | GC04P155854 | 1.065416 | https://www.genecards.org/cgi-bin/carddisp.pl?gene=TDO2 |
| FOXO4 | Forkhead Box O4 | Protein Coding | 40 | GC0XP071095 | 1.065167 | https://www.genecards.org/cgi-bin/carddisp.pl?gene=FOXO4 |
| SMARCB1 | SWI/SNF Related, Matrix Associated, Actin Dependent Regulator Of Chromatin, Subfamily B, Member 1 | Protein Coding | 44 | GC22P023786 | 1.058504 | https://www.genecards.org/cgi-bin/carddisp.pl?gene=SMARCB1 |

| | | | | | | |
|---|---|---|---|---|---|---|
| NRAS | NRAS Proto-Oncogene, GTPase | Protein Coding | 47 | GC01M 114704 | 1.058 404 | https://www.genecards.org/cgi-bin/carddisp.pl?gene=NRAS |
| ARF1 | ADP Ribosylation Factor 1 | Protein Coding | 44 | GC01P 228082 | 1.058 404 | https://www.genecards.org/cgi-bin/carddisp.pl?gene=ARF1 |
| DSC3 | Desmocollin 3 | Protein Coding | 42 | GC18M 030990 | 1.058 404 | https://www.genecards.org/cgi-bin/carddisp.pl?gene=DSC3 |
| NUP107 | Nucleoporin 107 | Protein Coding | 42 | GC12P 068686 | 1.058 404 | https://www.genecards.org/cgi-bin/carddisp.pl?gene=NUP107 |
| DST | Dystonin | Protein Coding | 41 | GC06M 056457 | 1.058 404 | https://www.genecards.org/cgi-bin/carddisp.pl?gene=DST |
| FAF1 | Fas Associated Factor 1 | Protein Coding | 41 | GC01M 050439 | 1.058 404 | https://www.genecards.org/cgi-bin/carddisp.pl?gene=FAF1 |
| ARFGAP 1 | ADP Ribosylation Factor GTPase Activating Protein 1 | Protein Coding | 40 | GC20P 063272 | 1.058 404 | https://www.genecards.org/cgi-bin/carddisp.pl?gene=ARFGAP1 |
| NUP133 | Nucleoporin 133 | Protein Coding | 40 | GC01M 229520 | 1.058 404 | https://www.genecards.org/cgi-bin/carddisp.pl?gene=NUP133 |
| ARFGAP 3 | ADP Ribosylation Factor GTPase Activating Protein 3 | Protein Coding | 39 | GC22M 042796 | 1.058 404 | https://www.genecards.org/cgi-bin/carddisp.pl?gene=ARFGAP3 |
| NUP85 | Nucleoporin 85 | Protein Coding | 39 | GC17P 075205 | 1.058 404 | https://www.genecards.org/cgi-bin/carddisp.pl?gene=NUP85 |
| RPS16 | Ribosomal Protein S16 | Protein Coding | 39 | GC19M 039433 | 1.058 404 | https://www.genecards.org/cgi-bin/carddisp.pl?gene=RPS16 |
| DSC1 | Desmocollin 1 | Protein Coding | 38 | GC18M 031129 | 1.058 404 | https://www.genecards.org/cgi-bin/carddisp.pl?gene=DSC1 |
| PIR | Pirin | Protein Coding | 38 | GC0X M0154 02 | 1.058 404 | https://www.genecards.org/cgi-bin/carddisp.pl?gene=PIR |
| RAB3IL1 | RAB3A Interacting Protein Like 1 | Protein Coding | 37 | GC11M 061897 | 1.058 404 | https://www.genecards.org/cgi- |

| | | | | | | |
|---|---|---|---|---|---|---|
| | | | | | | bin/carddisp.pl?gene=RAB3IL1 |
| AHCTF1 | AT-Hook Containing Transcription Factor 1 | Protein Coding | 36 | GC01M246840 | 1.058404 | https://www.genecards.org/cgi-bin/carddisp.pl?gene=AHCTF1 |
| ANKRD49 | Ankyrin Repeat Domain 49 | Protein Coding | 36 | GC11P094493 | 1.058404 | https://www.genecards.org/cgi-bin/carddisp.pl?gene=ANKRD49 |
| ARFGAP2 | ADP Ribosylation Factor GTPase Activating Protein 2 | Protein Coding | 35 | GC11M089355 | 1.058404 | https://www.genecards.org/cgi-bin/carddisp.pl?gene=ARFGAP2 |
| NUP43 | Nucleoporin 43 | Protein Coding | 33 | GC06M149724 | 1.058404 | https://www.genecards.org/cgi-bin/carddisp.pl?gene=NUP43 |
| MT-TF | Mitochondrially Encoded TRNA-Phe (UUU/C) | RNA Gene | 14 | GCMTP000580 | 1.058404 | https://www.genecards.org/cgi-bin/carddisp.pl?gene=MT-TF |
| MT-TH | Mitochondrially Encoded TRNA-His (CAU/C) | RNA Gene | 14 | GCMTP012140 | 1.058404 | https://www.genecards.org/cgi-bin/carddisp.pl?gene=MT-TH |
| MT-TK | Mitochondrially Encoded TRNA-Lys (AAA/G) | RNA Gene | 14 | GCMTP008297 | 1.058404 | https://www.genecards.org/cgi-bin/carddisp.pl?gene=MT-TK |
| MT-TG | Mitochondrially Encoded TRNA-Gly (GGN) | RNA Gene | 13 | GCMTP009993 | 1.058404 | https://www.genecards.org/cgi-bin/carddisp.pl?gene=MT-TG |
| NPC2 | NPC Intracellular Cholesterol Transporter 2 | Protein Coding | 41 | GC14M074476 | 1.056815 | https://www.genecards.org/cgi-bin/carddisp.pl?gene=NPC2 |
| ISG15 | ISG15 Ubiquitin Like Modifier | Protein Coding | 44 | GC01P001001 | 1.056545 | https://www.genecards.org/cgi-bin/carddisp.pl?gene=ISG15 |
| HUWE1 | HECT, UBA And WWE Domain Containing E3 Ubiquitin Protein Ligase 1 | Protein Coding | 42 | GC0XM053532 | 1.055635 | https://www.genecards.org/cgi-bin/carddisp.pl?gene=HUWE1 |
| PMAIP1 | Phorbol-12-Myristate-13-Acetate-Induced Protein 1 | Protein Coding | 38 | GC18P059899 | 1.055635 | https://www.genecards.org/cgi-bin/carddisp.pl?gene=PMAIP1 |
| SIK2 | Salt Inducible Kinase 2 | Protein Coding | 42 | GC11P111920 | 1.054219 | https://www.genecards.org/cgi-bin/carddisp.pl?gene=SIK2 |

| | | | | | | |
|---|---|---|---|---|---|---|
| SIGLEC15 | Sialic Acid Binding Ig Like Lectin 15 | Protein Coding | 34 | GC18P045825 | 1.052802 | https://www.genecards.org/cgi-bin/carddisp.pl?gene=SIGLEC15 |
| ALYREF | Aly/REF Export Factor | Protein Coding | 37 | GC17M081887 | 1.05225 | https://www.genecards.org/cgi-bin/carddisp.pl?gene=ALYREF |
| SAE1 | SUMO1 Activating Enzyme Subunit 1 | Protein Coding | 44 | GC19P066970 | 1.051125 | https://www.genecards.org/cgi-bin/carddisp.pl?gene=SAE1 |
| UBA2 | Ubiquitin Like Modifier Activating Enzyme 2 | Protein Coding | 43 | GC19P034428 | 1.051125 | https://www.genecards.org/cgi-bin/carddisp.pl?gene=UBA2 |
| KLRG1 | Killer Cell Lectin Like Receptor G1 | Protein Coding | 37 | GC12P008950 | 1.047638 | https://www.genecards.org/cgi-bin/carddisp.pl?gene=KLRG1 |
| NFKBIZ | NFKB Inhibitor Zeta | Protein Coding | 37 | GC03P101827 | 1.045705 | https://www.genecards.org/cgi-bin/carddisp.pl?gene=NFKBIZ |
| TRIM26 | Tripartite Motif Containing 26 | Protein Coding | 36 | GC06M030184 | 1.045705 | https://www.genecards.org/cgi-bin/carddisp.pl?gene=TRIM26 |
| PI4KA | Phosphatidylinositol 4-Kinase Alpha | Protein Coding | 46 | GC22M020707 | 1.043198 | https://www.genecards.org/cgi-bin/carddisp.pl?gene=PI4KA |
| MIR24-2 | MicroRNA 24-2 | RNA Gene | 20 | GC19M014483 | 1.041605 | https://www.genecards.org/cgi-bin/carddisp.pl?gene=MIR24-2 |
| MYO9B | Myosin IXB | Protein Coding | 43 | GC19P066216 | 1.040936 | https://www.genecards.org/cgi-bin/carddisp.pl?gene=MYO9B |
| PDYN | Prodynorphin | Protein Coding | 43 | GC20M001978 | 1.038956 | https://www.genecards.org/cgi-bin/carddisp.pl?gene=PDYN |
| CAPS2 | Calcyphosine 2 | Protein Coding | 32 | GC12M075260 | 1.037439 | https://www.genecards.org/cgi-bin/carddisp.pl?gene=CAPS2 |
| MIR9-2 | MicroRNA 9-2 | RNA Gene | 20 | GC05M088666 | 1.032208 | https://www.genecards.org/cgi-bin/carddisp.pl?gene=MIR9-2 |
| MIR9-3 | MicroRNA 9-3 | RNA Gene | 20 | GC15P089368 | 1.032208 | https://www.genecards.org/cgi- |

| | | | | | | |
|---|---|---|---|---|---|---|
| | | | | | | bin/carddisp.pl?gene=MIR9-3 |
| OAS1 | 2'-5'-Oligoadenylate Synthetase 1 | Protein Coding | 43 | GC12P 112939 | 1.030 091 | https://www.genecards.org/cgi-bin/carddisp.pl?gene=OAS1 |
| CDC42 | Cell Division Cycle 42 | Protein Coding | 49 | GC01P 022052 | 1.029 915 | https://www.genecards.org/cgi-bin/carddisp.pl?gene=CDC42 |
| PRNP | Prion Protein | Protein Coding | 45 | GC20P 004686 | 1.029 915 | https://www.genecards.org/cgi-bin/carddisp.pl?gene=PRNP |
| FGF17 | Fibroblast Growth Factor 17 | Protein Coding | 44 | GC08P 022042 | 1.029 915 | https://www.genecards.org/cgi-bin/carddisp.pl?gene=FGF17 |
| WASF1 | WASP Family Member 1 | Protein Coding | 43 | GC06M 110099 | 1.029 915 | https://www.genecards.org/cgi-bin/carddisp.pl?gene=WASF1 |
| NCK1 | NCK Adaptor Protein 1 | Protein Coding | 42 | GC03P 136862 | 1.029 915 | https://www.genecards.org/cgi-bin/carddisp.pl?gene=NCK1 |
| WASF2 | WASP Family Member 2 | Protein Coding | 41 | GC01M 027404 | 1.029 915 | https://www.genecards.org/cgi-bin/carddisp.pl?gene=WASF2 |
| ACTR3 | Actin Related Protein 3 | Protein Coding | 40 | GC02P 113889 | 1.029 915 | https://www.genecards.org/cgi-bin/carddisp.pl?gene=ACTR3 |
| BAIAP2 | BAR/IMD Domain Containing Adaptor Protein 2 | Protein Coding | 40 | GC17P 081035 | 1.029 915 | https://www.genecards.org/cgi-bin/carddisp.pl?gene=BAIAP2 |
| WASF3 | WASP Family Member 3 | Protein Coding | 40 | GC13P 026557 | 1.029 915 | https://www.genecards.org/cgi-bin/carddisp.pl?gene=WASF3 |
| WASL | WASP Like Actin Nucleation Promoting Factor | Protein Coding | 40 | GC07M 123681 | 1.029 915 | https://www.genecards.org/cgi-bin/carddisp.pl?gene=WASL |
| TRIP10 | Thyroid Hormone Receptor Interactor 10 | Protein Coding | 38 | GC19P 006737 | 1.029 915 | https://www.genecards.org/cgi-bin/carddisp.pl?gene=TRIP10 |
| ARFRP1 | ADP Ribosylation Factor Related Protein 1 | Protein Coding | 37 | GC20M 063698 | 1.029 915 | https://www.genecards.org/cgi-bin/carddisp.pl?gene=ARFRP1 |

| | | | | | | |
|---|---|---|---|---|---|---|
| NCKIPSD | NCK Interacting Protein With SH3 Domain | Protein Coding | 36 | GC03M048667 | 1.029915 | https://www.genecards.org/cgi-bin/carddisp.pl?gene=NCKIPSD |
| PNMA2 | PNMA Family Member 2 | Protein Coding | 36 | GC08M026504 | 1.029915 | https://www.genecards.org/cgi-bin/carddisp.pl?gene=PNMA2 |
| WHAMM | WASP Homolog Associated With Actin, Golgi Membranes And Microtubules | Protein Coding | 33 | GC15P117911 | 1.029915 | https://www.genecards.org/cgi-bin/carddisp.pl?gene=WHAMM |
| WASHC1 | WASH Complex Subunit 1 | Protein Coding | 23 | GC09M000016 | 1.029915 | https://www.genecards.org/cgi-bin/carddisp.pl?gene=WASHC1 |
| WASH3P | WASP Family Homolog 3, Pseudogene | Pseudogene | 20 | GC15P101960 | 1.029915 | https://www.genecards.org/cgi-bin/carddisp.pl?gene=WASH3P |
| WASH6P | WASP Family Homolog 6, Pseudogene | Pseudogene | 18 | GC0XP156054 | 1.029915 | https://www.genecards.org/cgi-bin/carddisp.pl?gene=WASH6P |
| WASF4P | WASP Family Member 4, Pseudogene | Pseudogene | 11 | GC0XP047804 | 1.029915 | https://www.genecards.org/cgi-bin/carddisp.pl?gene=WASF4P |
| ADAM8 | ADAM Metallopeptidase Domain 8 | Protein Coding | 42 | GC10M133262 | 1.029904 | https://www.genecards.org/cgi-bin/carddisp.pl?gene=ADAM8 |
| MIA | MIA SH3 Domain Containing | Protein Coding | 37 | GC19P040771 | 1.027749 | https://www.genecards.org/cgi-bin/carddisp.pl?gene=MIA |
| TRIB3 | Tribbles Pseudokinase 3 | Protein Coding | 41 | GC20P000361 | 1.021446 | https://www.genecards.org/cgi-bin/carddisp.pl?gene=TRIB3 |
| ACO1 | Aconitase 1 | Protein Coding | 43 | GC09P032374 | 1.01676 | https://www.genecards.org/cgi-bin/carddisp.pl?gene=ACO1 |
| UBASH3B | Ubiquitin Associated And SH3 Domain Containing B | Protein Coding | 40 | GC11P122655 | 1.01676 | https://www.genecards.org/cgi-bin/carddisp.pl?gene=UBASH3B |
| HAS3 | Hyaluronan Synthase 3 | Protein Coding | 41 | GC16P069105 | 1.014896 | https://www.genecards.org/cgi-bin/carddisp.pl?gene=HAS3 |
| TRAF5 | TNF Receptor Associated Factor 5 | Protein Coding | 40 | GC01P211326 | 1.014896 | https://www.genecards.org/cgi- |

| | | | | | | |
|---|---|---|---|---|---|---|
| | | | | | | bin/carddisp.pl?gene=TRAF5 |
| TSHR | Thyroid Stimulating Hormone Receptor | Protein Coding | 45 | GC14P080954 | 1.013282 | https://www.genecards.org/cgi-bin/carddisp.pl?gene=TSHR |
| AKT3 | AKT Serine/Threonine Kinase 3 | Protein Coding | 51 | GC01M243488 | 1.012203 | https://www.genecards.org/cgi-bin/carddisp.pl?gene=AKT3 |
| TBK1 | TANK Binding Kinase 1 | Protein Coding | 47 | GC12P064451 | 1.012203 | https://www.genecards.org/cgi-bin/carddisp.pl?gene=TBK1 |
| EXO1 | Exonuclease 1 | Protein Coding | 42 | GC01P241847 | 1.012203 | https://www.genecards.org/cgi-bin/carddisp.pl?gene=EXO1 |
| F10 | Coagulation Factor X | Protein Coding | 48 | GC13P113122 | 1.011449 | https://www.genecards.org/cgi-bin/carddisp.pl?gene=F10 |
| COL17A1 | Collagen Type XVII Alpha 1 Chain | Protein Coding | 43 | GC10M104031 | 1.006446 | https://www.genecards.org/cgi-bin/carddisp.pl?gene=COL17A1 |
| PTPRN | Protein Tyrosine Phosphatase Receptor Type N | Protein Coding | 43 | GC02M219289 | 1.006446 | https://www.genecards.org/cgi-bin/carddisp.pl?gene=PTPRN |
| LIMK1 | LIM Domain Kinase 1 | Protein Coding | 48 | GC07P074082 | 1.004889 | https://www.genecards.org/cgi-bin/carddisp.pl?gene=LIMK1 |
| POLH | DNA Polymerase Eta | Protein Coding | 48 | GC06P043576 | 1.004889 | https://www.genecards.org/cgi-bin/carddisp.pl?gene=POLH |
| ITGA7 | Integrin Subunit Alpha 7 | Protein Coding | 46 | GC12M055684 | 1.004889 | https://www.genecards.org/cgi-bin/carddisp.pl?gene=ITGA7 |
| LEF1 | Lymphoid Enhancer Binding Factor 1 | Protein Coding | 46 | GC04M108047 | 1.004889 | https://www.genecards.org/cgi-bin/carddisp.pl?gene=LEF1 |
| TRPC3 | Transient Receptor Potential Cation Channel Subfamily C Member 3 | Protein Coding | 46 | GC04M121879 | 1.004889 | https://www.genecards.org/cgi-bin/carddisp.pl?gene=TRPC3 |
| ABCD1 | ATP Binding Cassette Subfamily D Member 1 | Protein Coding | 45 | GC0XP153724 | 1.004889 | https://www.genecards.org/cgi-bin/carddisp.pl?gene=ABCD1 |

| | | | | | | |
|---|---|---|---|---|---|---|
| FEN1 | Flap Structure-Specific Endonuclease 1 | Protein Coding | 45 | GC11P 061793 | 1.004 889 | https://www.genecards .org/cgi-bin/carddisp.pl?gene= FEN1 |
| GALT | Galactose-1-Phosphate Uridylyltransferase | Protein Coding | 45 | GC09P 047114 | 1.004 889 | https://www.genecards .org/cgi-bin/carddisp.pl?gene= GALT |
| HADH | Hydroxyacyl-CoA Dehydrogenase | Protein Coding | 45 | GC04P 107989 | 1.004 889 | https://www.genecards .org/cgi-bin/carddisp.pl?gene= HADH |
| HCFC1 | Host Cell Factor C1 | Protein Coding | 45 | GC0X M1539 47 | 1.004 889 | https://www.genecards .org/cgi-bin/carddisp.pl?gene= HCFC1 |
| ACTG2 | Actin Gamma 2, Smooth Muscle | Protein Coding | 44 | GC02P 073892 | 1.004 889 | https://www.genecards .org/cgi-bin/carddisp.pl?gene= ACTG2 |
| COX4I1 | Cytochrome C Oxidase Subunit 4I1 | Protein Coding | 44 | GC16P 085798 | 1.004 889 | https://www.genecards .org/cgi-bin/carddisp.pl?gene= COX4I1 |
| FLNC | Filamin C | Protein Coding | 44 | GC07P 128830 | 1.004 889 | https://www.genecards .org/cgi-bin/carddisp.pl?gene= FLNC |
| GAK | Cyclin G Associated Kinase | Protein Coding | 44 | GC04M 000849 | 1.004 889 | https://www.genecards .org/cgi-bin/carddisp.pl?gene= GAK |
| KCNE1 | Potassium Voltage-Gated Channel Subfamily E Regulatory Subunit 1 | Protein Coding | 44 | GC21M 034446 | 1.004 889 | https://www.genecards .org/cgi-bin/carddisp.pl?gene= KCNE1 |
| KMT2A | Lysine Methyltransferase 2A | Protein Coding | 44 | GC11P 118436 | 1.004 889 | https://www.genecards .org/cgi-bin/carddisp.pl?gene= KMT2A |
| HSPA6 | Heat Shock Protein Family A (Hsp70) Member 6 | Protein Coding | 43 | GC01P 161524 | 1.004 889 | https://www.genecards .org/cgi-bin/carddisp.pl?gene= HSPA6 |
| KIF23 | Kinesin Family Member 23 | Protein Coding | 43 | GC15P 069414 | 1.004 889 | https://www.genecards .org/cgi-bin/carddisp.pl?gene= KIF23 |
| ABCG1 | ATP Binding Cassette Subfamily G Member 1 | Protein Coding | 42 | GC21P 042199 | 1.004 889 | https://www.genecards .org/cgi-bin/carddisp.pl?gene= ABCG1 |
| BEST1 | Bestrophin 1 | Protein Coding | 42 | GC11P 061949 | 1.004 889 | https://www.genecards .org/cgi- |

| | | | | | | |
|---|---|---|---|---|---|---|
| | | | | | | bin/carddisp.pl?gene=BEST1 |
| CDK12 | Cyclin Dependent Kinase 12 | Protein Coding | 42 | GC17P039461 | 1.004889 | https://www.genecards.org/cgi-bin/carddisp.pl?gene=CDK12 |
| CELF2 | CUGBP Elav-Like Family Member 2 | Protein Coding | 42 | GC10P010462 | 1.004889 | https://www.genecards.org/cgi-bin/carddisp.pl?gene=CELF2 |
| CTDP1 | CTD Phosphatase Subunit 1 | Protein Coding | 42 | GC18P079679 | 1.004889 | https://www.genecards.org/cgi-bin/carddisp.pl?gene=CTDP1 |
| CYP2U1 | Cytochrome P450 Family 2 Subfamily U Member 1 | Protein Coding | 42 | GC04P107931 | 1.004889 | https://www.genecards.org/cgi-bin/carddisp.pl?gene=CYP2U1 |
| GART | Phosphoribosylglycinamide Formyltransferase, Phosphoribosylglycinamide Synthetase, Phosphoribosylaminoimidazole Synthetase | Protein Coding | 42 | GC21M033503 | 1.004889 | https://www.genecards.org/cgi-bin/carddisp.pl?gene=GART |
| MAPKAPK5 | MAPK Activated Protein Kinase 5 | Protein Coding | 42 | GC12P111842 | 1.004889 | https://www.genecards.org/cgi-bin/carddisp.pl?gene=MAPKAPK5 |
| TCF7 | Transcription Factor 7 | Protein Coding | 42 | GC05P134114 | 1.004889 | https://www.genecards.org/cgi-bin/carddisp.pl?gene=TCF7 |
| TLE1 | TLE Family Member 1, Transcriptional Corepressor | Protein Coding | 42 | GC09M081583 | 1.004889 | https://www.genecards.org/cgi-bin/carddisp.pl?gene=TLE1 |
| TLE3 | TLE Family Member 3, Transcriptional Corepressor | Protein Coding | 42 | GC15M070047 | 1.004889 | https://www.genecards.org/cgi-bin/carddisp.pl?gene=TLE3 |
| ARHGAP4 | Rho GTPase Activating Protein 4 | Protein Coding | 41 | GC0XM153907 | 1.004889 | https://www.genecards.org/cgi-bin/carddisp.pl?gene=ARHGAP4 |
| BRAP | BRCA1 Associated Protein | Protein Coding | 41 | GC12M111642 | 1.004889 | https://www.genecards.org/cgi-bin/carddisp.pl?gene=BRAP |
| CPA4 | Carboxypeptidase A4 | Protein Coding | 41 | GC07P130293 | 1.004889 | https://www.genecards.org/cgi-bin/carddisp.pl?gene=CPA4 |
| CRB2 | Crumbs Cell Polarity Complex Component 2 | Protein Coding | 41 | GC09P123356 | 1.004889 | https://www.genecards.org/cgi- |

| | | | | | | |
|---|---|---|---|---|---|---|
| | | | | | | bin/carddisp.pl?gene=CRB2 |
| DGKQ | Diacylglycerol Kinase Theta | Protein Coding | 41 | GC04M000958 | 1.004889 | https://www.genecards.org/cgi-bin/carddisp.pl?gene=DGKQ |
| FOXF1 | Forkhead Box F1 | Protein Coding | 41 | GC16P086510 | 1.004889 | https://www.genecards.org/cgi-bin/carddisp.pl?gene=FOXF1 |
| GSTO2 | Glutathione S-Transferase Omega 2 | Protein Coding | 41 | GC10P104268 | 1.004889 | https://www.genecards.org/cgi-bin/carddisp.pl?gene=GSTO2 |
| GTF2IRD1 | GTF2I Repeat Domain Containing 1 | Protein Coding | 41 | GC07P075437 | 1.004889 | https://www.genecards.org/cgi-bin/carddisp.pl?gene=GTF2IRD1 |
| IGSF3 | Immunoglobulin Superfamily Member 3 | Protein Coding | 41 | GC01M116574 | 1.004889 | https://www.genecards.org/cgi-bin/carddisp.pl?gene=IGSF3 |
| IQGAP1 | IQ Motif Containing GTPase Activating Protein 1 | Protein Coding | 41 | GC15P090388 | 1.004889 | https://www.genecards.org/cgi-bin/carddisp.pl?gene=IQGAP1 |
| LARP7 | La Ribonucleoprotein 7, Transcriptional Regulator | Protein Coding | 41 | GC04P112636 | 1.004889 | https://www.genecards.org/cgi-bin/carddisp.pl?gene=LARP7 |
| NEUROD2 | Neuronal Differentiation 2 | Protein Coding | 41 | GC17M039603 | 1.004889 | https://www.genecards.org/cgi-bin/carddisp.pl?gene=NEUROD2 |
| RCAN1 | Regulator Of Calcineurin 1 | Protein Coding | 41 | GC21M034513 | 1.004889 | https://www.genecards.org/cgi-bin/carddisp.pl?gene=RCAN1 |
| SLC4A7 | Solute Carrier Family 4 Member 7 | Protein Coding | 41 | GC03M027372 | 1.004889 | https://www.genecards.org/cgi-bin/carddisp.pl?gene=SLC4A7 |
| SON | SON DNA And RNA Binding Protein | Protein Coding | 41 | GC21P033542 | 1.004889 | https://www.genecards.org/cgi-bin/carddisp.pl?gene=SON |
| VANGL2 | VANGL Planar Cell Polarity Protein 2 | Protein Coding | 41 | GC01P160400 | 1.004889 | https://www.genecards.org/cgi-bin/carddisp.pl?gene=VANGL2 |
| VPS11 | VPS11 Core Subunit Of CORVET And HOPS Complexes | Protein Coding | 41 | GC11P119067 | 1.004889 | https://www.genecards.org/cgi-bin/carddisp.pl?gene=VPS11 |

| | | | | | | |
|---|---|---|---|---|---|---|
| AP4B1 | Adaptor Related Protein Complex 4 Subunit Beta 1 | Protein Coding | 40 | GC01M113894 | 1.004889 | https://www.genecards.org/cgi-bin/carddisp.pl?gene=AP4B1 |
| APEH | Acylaminoacyl-Peptide Hydrolase | Protein Coding | 40 | GC03P049673 | 1.004889 | https://www.genecards.org/cgi-bin/carddisp.pl?gene=APEH |
| ATP6V1F | ATPase H+ Transporting V1 Subunit F | Protein Coding | 40 | GC07P128862 | 1.004889 | https://www.genecards.org/cgi-bin/carddisp.pl?gene=ATP6V1F |
| BACE2 | Beta-Secretase 2 | Protein Coding | 40 | GC21P041168 | 1.004889 | https://www.genecards.org/cgi-bin/carddisp.pl?gene=BACE2 |
| CACNG3 | Calcium Voltage-Gated Channel Auxiliary Subunit Gamma 3 | Protein Coding | 40 | GC16P024309 | 1.004889 | https://www.genecards.org/cgi-bin/carddisp.pl?gene=CACNG3 |
| DNMT3L | DNA Methyltransferase 3 Like | Protein Coding | 40 | GC21M044246 | 1.004889 | https://www.genecards.org/cgi-bin/carddisp.pl?gene=DNMT3L |
| GPC5 | Glypican 5 | Protein Coding | 40 | GC13P091398 | 1.004889 | https://www.genecards.org/cgi-bin/carddisp.pl?gene=GPC5 |
| HS3ST1 | Heparan Sulfate-Glucosamine 3-Sulfotransferase 1 | Protein Coding | 40 | GC04M011394 | 1.004889 | https://www.genecards.org/cgi-bin/carddisp.pl?gene=HS3ST1 |
| ICAM5 | Intercellular Adhesion Molecule 5 | Protein Coding | 40 | GC19P010289 | 1.004889 | https://www.genecards.org/cgi-bin/carddisp.pl?gene=ICAM5 |
| IFT140 | Intraflagellar Transport 140 | Protein Coding | 40 | GC16M007076 | 1.004889 | https://www.genecards.org/cgi-bin/carddisp.pl?gene=IFT140 |
| INCENP | Inner Centromere Protein | Protein Coding | 40 | GC11P062142 | 1.004889 | https://www.genecards.org/cgi-bin/carddisp.pl?gene=INCENP |
| MSI1 | Musashi RNA Binding Protein 1 | Protein Coding | 40 | GC12M120341 | 1.004889 | https://www.genecards.org/cgi-bin/carddisp.pl?gene=MSI1 |
| RENBP | Renin Binding Protein | Protein Coding | 40 | GC0XM153935 | 1.004889 | https://www.genecards.org/cgi-bin/carddisp.pl?gene=RENBP |

| | | | | | | |
|---|---|---|---|---|---|---|
| SETD1A | SET Domain Containing 1A, Histone Lysine Methyltransferase | Protein Coding | 40 | GC16P 041106 | 1.004 889 | https://www.genecards .org/cgi-bin/carddisp.pl?gene= SETD1A |
| SRCAP | Snf2 Related CREBBP Activator Protein | Protein Coding | 40 | GC16P 041094 | 1.004 889 | https://www.genecards .org/cgi-bin/carddisp.pl?gene= SRCAP |
| STX4 | Syntaxin 4 | Protein Coding | 40 | GC16P 041110 | 1.004 889 | https://www.genecards .org/cgi-bin/carddisp.pl?gene= STX4 |
| TRIOBP | TRIO And F-Actin Binding Protein | Protein Coding | 40 | GC22P 037697 | 1.004 889 | https://www.genecards .org/cgi-bin/carddisp.pl?gene= TRIOBP |
| UBE2E3 | Ubiquitin Conjugating Enzyme E2 E3 | Protein Coding | 40 | GC02P 180967 | 1.004 889 | https://www.genecards .org/cgi-bin/carddisp.pl?gene= UBE2E3 |
| UBE4A | Ubiquitination Factor E4A | Protein Coding | 40 | GC11P 118359 | 1.004 889 | https://www.genecards .org/cgi-bin/carddisp.pl?gene= UBE4A |
| VTI1B | Vesicle Transport Through Interaction With T-SNAREs 1B | Protein Coding | 40 | GC14M 067647 | 1.004 889 | https://www.genecards .org/cgi-bin/carddisp.pl?gene= VTI1B |
| XPO5 | Exportin 5 | Protein Coding | 40 | GC06M 043522 | 1.004 889 | https://www.genecards .org/cgi-bin/carddisp.pl?gene= XPO5 |
| ZNF143 | Zinc Finger Protein 143 | Protein Coding | 40 | GC11P 009483 | 1.004 889 | https://www.genecards .org/cgi-bin/carddisp.pl?gene= ZNF143 |
| ABCC10 | ATP Binding Cassette Subfamily C Member 10 | Protein Coding | 39 | GC06P 043427 | 1.004 889 | https://www.genecards .org/cgi-bin/carddisp.pl?gene= ABCC10 |
| CCNT1 | Cyclin T1 | Protein Coding | 39 | GC12M 048688 | 1.004 889 | https://www.genecards .org/cgi-bin/carddisp.pl?gene= CCNT1 |
| KIF3A | Kinesin Family Member 3A | Protein Coding | 39 | GC05M 132689 | 1.004 889 | https://www.genecards .org/cgi-bin/carddisp.pl?gene= KIF3A |
| MVP | Major Vault Protein | Protein Coding | 39 | GC16P 041016 | 1.004 889 | https://www.genecards .org/cgi-bin/carddisp.pl?gene= MVP |
| OLIG2 | Oligodendrocyte Transcription Factor 2 | Protein Coding | 39 | GC21P 033025 | 1.004 889 | https://www.genecards .org/cgi- |

| | | | | | | |
|---|---|---|---|---|---|---|
| | | | | | | bin/carddisp.pl?gene= OLIG2 |
| SAR1A | Secretion Associated Ras Related GTPase 1A | Protein Coding | 39 | GC10M 070147 | 1.004 889 | https://www.genecards .org/cgi-bin/carddisp.pl?gene= SAR1A |
| SLC35B2 | Solute Carrier Family 35 Member B2 | Protein Coding | 39 | GC06M 044254 | 1.004 889 | https://www.genecards .org/cgi-bin/carddisp.pl?gene= SLC35B2 |
| SWAP70 | Switching B Cell Complex Subunit SWAP70 | Protein Coding | 39 | GC11P 009664 | 1.004 889 | https://www.genecards .org/cgi-bin/carddisp.pl?gene= SWAP70 |
| TET3 | Tet Methylcytosine Dioxygenase 3 | Protein Coding | 39 | GC02P 073986 | 1.004 889 | https://www.genecards .org/cgi-bin/carddisp.pl?gene= TET3 |
| USP6NL | USP6 N-Terminal Like | Protein Coding | 39 | GC10M 011453 | 1.004 889 | https://www.genecards .org/cgi-bin/carddisp.pl?gene= USP6NL |
| UTS2 | Urotensin 2 | Protein Coding | 39 | GC01M 007843 | 1.004 889 | https://www.genecards .org/cgi-bin/carddisp.pl?gene= UTS2 |
| BMP2K | BMP2 Inducible Kinase | Protein Coding | 38 | GC04P 078776 | 1.004 889 | https://www.genecards .org/cgi-bin/carddisp.pl?gene= BMP2K |
| CAPN11 | Calpain 11 | Protein Coding | 38 | GC06P 083931 | 1.004 889 | https://www.genecards .org/cgi-bin/carddisp.pl?gene= CAPN11 |
| CDC42E P3 | CDC42 Effector Protein 3 | Protein Coding | 38 | GC02M 037641 | 1.004 889 | https://www.genecards .org/cgi-bin/carddisp.pl?gene= CDC42EP3 |
| CLIC6 | Chloride Intracellular Channel 6 | Protein Coding | 38 | GC21P 034671 | 1.004 889 | https://www.genecards .org/cgi-bin/carddisp.pl?gene= CLIC6 |
| DAP | Death Associated Protein | Protein Coding | 38 | GC05M 010679 | 1.004 889 | https://www.genecards .org/cgi-bin/carddisp.pl?gene= DAP |
| DCTN4 | Dynactin Subunit 4 | Protein Coding | 38 | GC05M 150708 | 1.004 889 | https://www.genecards .org/cgi-bin/carddisp.pl?gene= DCTN4 |
| DDX23 | DEAD-Box Helicase 23 | Protein Coding | 38 | GC12M 048829 | 1.004 889 | https://www.genecards .org/cgi-bin/carddisp.pl?gene= DDX23 |

| | | | | | | |
|---|---|---|---|---|---|---|
| DNASE1L1 | Deoxyribonuclease 1 Like 1 | Protein Coding | 38 | GC0XM154401 | 1.004889 | https://www.genecards.org/cgi-bin/carddisp.pl?gene=DNASE1L1 |
| DNMBP | Dynamin Binding Protein | Protein Coding | 38 | GC10M099875 | 1.004889 | https://www.genecards.org/cgi-bin/carddisp.pl?gene=DNMBP |
| EIF3D | Eukaryotic Translation Initiation Factor 3 Subunit D | Protein Coding | 38 | GC22M036510 | 1.004889 | https://www.genecards.org/cgi-bin/carddisp.pl?gene=EIF3D |
| EXOC2 | Exocyst Complex Component 2 | Protein Coding | 38 | GC06M000485 | 1.004889 | https://www.genecards.org/cgi-bin/carddisp.pl?gene=EXOC2 |
| FIGNL1 | Fidgetin Like 1 | Protein Coding | 38 | GC07M050444 | 1.004889 | https://www.genecards.org/cgi-bin/carddisp.pl?gene=FIGNL1 |
| GTPBP2 | GTP Binding Protein 2 | Protein Coding | 38 | GC06M043605 | 1.004889 | https://www.genecards.org/cgi-bin/carddisp.pl?gene=GTPBP2 |
| LZTS1 | Leucine Zipper Tumor Suppressor 1 | Protein Coding | 38 | GC08M020246 | 1.004889 | https://www.genecards.org/cgi-bin/carddisp.pl?gene=LZTS1 |
| NAB1 | NGFI-A Binding Protein 1 | Protein Coding | 38 | GC02P190646 | 1.004889 | https://www.genecards.org/cgi-bin/carddisp.pl?gene=NAB1 |
| NLRX1 | NLR Family Member X1 | Protein Coding | 38 | GC11P119166 | 1.004889 | https://www.genecards.org/cgi-bin/carddisp.pl?gene=NLRX1 |
| NUP88 | Nucleoporin 88 | Protein Coding | 38 | GC17M006177 | 1.004889 | https://www.genecards.org/cgi-bin/carddisp.pl?gene=NUP88 |
| P2RY10 | P2Y Receptor Family Member 10 | Protein Coding | 38 | GC0XP078945 | 1.004889 | https://www.genecards.org/cgi-bin/carddisp.pl?gene=P2RY10 |
| PARD6G | Par-6 Family Cell Polarity Regulator Gamma | Protein Coding | 38 | GC18M080157 | 1.004889 | https://www.genecards.org/cgi-bin/carddisp.pl?gene=PARD6G |
| PDC | Phosducin | Protein Coding | 38 | GC01M186412 | 1.004889 | https://www.genecards.org/cgi-bin/carddisp.pl?gene=PDC |
| PINX1 | PIN2 (TERF1) Interacting Telomerase Inhibitor 1 | Protein Coding | 38 | GC08M010764 | 1.004889 | https://www.genecards.org/cgi- |

| | | | | | | |
|---|---|---|---|---|---|---|
| | | | | | | bin/carddisp.pl?gene=PINX1 |
| PPIL3 | Peptidylprolyl Isomerase Like 3 | Protein Coding | 38 | GC02M200870 | 1.004889 | https://www.genecards.org/cgi-bin/carddisp.pl?gene=PPIL3 |
| RNF40 | Ring Finger Protein 40 | Protein Coding | 38 | GC16P041101 | 1.004889 | https://www.genecards.org/cgi-bin/carddisp.pl?gene=RNF40 |
| SBF2 | SET Binding Factor 2 | Protein Coding | 38 | GC11M009797 | 1.004889 | https://www.genecards.org/cgi-bin/carddisp.pl?gene=SBF2 |
| SLC36A1 | Solute Carrier Family 36 Member 1 | Protein Coding | 38 | GC05P151344 | 1.004889 | https://www.genecards.org/cgi-bin/carddisp.pl?gene=SLC36A1 |
| SLC9A9 | Solute Carrier Family 9 Member A9 | Protein Coding | 38 | GC03M143265 | 1.004889 | https://www.genecards.org/cgi-bin/carddisp.pl?gene=SLC9A9 |
| USP19 | Ubiquitin Specific Peptidase 19 | Protein Coding | 38 | GC03M051164 | 1.004889 | https://www.genecards.org/cgi-bin/carddisp.pl?gene=USP19 |
| WTAP | WT1 Associated Protein | Protein Coding | 38 | GC06P159725 | 1.004889 | https://www.genecards.org/cgi-bin/carddisp.pl?gene=WTAP |
| ZNF638 | Zinc Finger Protein 638 | Protein Coding | 38 | GC02P071276 | 1.004889 | https://www.genecards.org/cgi-bin/carddisp.pl?gene=ZNF638 |
| ADO | 2-Aminoethanethiol Dioxygenase | Protein Coding | 37 | GC10P062804 | 1.004889 | https://www.genecards.org/cgi-bin/carddisp.pl?gene=ADO |
| AIFM2 | Apoptosis Inducing Factor Mitochondria Associated 2 | Protein Coding | 37 | GC10M070098 | 1.004889 | https://www.genecards.org/cgi-bin/carddisp.pl?gene=AIFM2 |
| ATP10B | ATPase Phospholipid Transporting 10B (Putative) | Protein Coding | 37 | GC05M160499 | 1.004889 | https://www.genecards.org/cgi-bin/carddisp.pl?gene=ATP10B |
| ATP9B | ATPase Phospholipid Transporting 9B (Putative) | Protein Coding | 37 | GC18P079069 | 1.004889 | https://www.genecards.org/cgi-bin/carddisp.pl?gene=ATP9B |
| BOLA3 | BolA Family Member 3 | Protein Coding | 37 | GC02M074136 | 1.004889 | https://www.genecards.org/cgi-bin/carddisp.pl?gene=BOLA3 |

| | | | | | | |
|---|---|---|---|---|---|---|
| CTR9 | CTR9 Homolog, Paf1/RNA Polymerase II Complex Component | Protein Coding | 37 | GC11P 010772 | 1.004 889 | https://www.genecards .org/cgi-bin/carddisp.pl?gene= CTR9 |
| DENND1 A | DENN Domain Containing 1A | Protein Coding | 37 | GC09M 123379 | 1.004 889 | https://www.genecards .org/cgi-bin/carddisp.pl?gene= DENND1A |
| FIS1 | Fission, Mitochondrial 1 | Protein Coding | 37 | GC07M 101239 | 1.004 889 | https://www.genecards .org/cgi-bin/carddisp.pl?gene= FIS1 |
| GATAD1 | GATA Zinc Finger Domain Containing 1 | Protein Coding | 37 | GC07P 092447 | 1.004 889 | https://www.genecards .org/cgi-bin/carddisp.pl?gene= GATAD1 |
| GGA2 | Golgi Associated, Gamma Adaptin Ear Containing, ARF Binding Protein 2 | Protein Coding | 37 | GC16M 023464 | 1.004 889 | https://www.genecards .org/cgi-bin/carddisp.pl?gene= GGA2 |
| GJA10 | Gap Junction Protein Alpha 10 | Protein Coding | 37 | GC06P 089894 | 1.004 889 | https://www.genecards .org/cgi-bin/carddisp.pl?gene= GJA10 |
| GPSM1 | G Protein Signaling Modulator 1 | Protein Coding | 37 | GC09P 136327 | 1.004 889 | https://www.genecards .org/cgi-bin/carddisp.pl?gene= GPSM1 |
| MOB1A | MOB Kinase Activator 1A | Protein Coding | 37 | GC02M 074152 | 1.004 889 | https://www.genecards .org/cgi-bin/carddisp.pl?gene= MOB1A |
| MYO1B | Myosin IB | Protein Coding | 37 | GC02P 191246 | 1.004 889 | https://www.genecards .org/cgi-bin/carddisp.pl?gene= MYO1B |
| NEUROG 2 | Neurogenin 2 | Protein Coding | 37 | GC04M 112513 | 1.004 889 | https://www.genecards .org/cgi-bin/carddisp.pl?gene= NEUROG2 |
| NUDT12 | Nudix Hydrolase 12 | Protein Coding | 37 | GC05M 103548 | 1.004 889 | https://www.genecards .org/cgi-bin/carddisp.pl?gene= NUDT12 |
| OLIG1 | Oligodendrocyte Transcription Factor 1 | Protein Coding | 37 | GC21P 033070 | 1.004 889 | https://www.genecards .org/cgi-bin/carddisp.pl?gene= OLIG1 |
| PDXP | Pyridoxal Phosphatase | Protein Coding | 37 | GC22P 037658 | 1.004 889 | https://www.genecards .org/cgi-bin/carddisp.pl?gene= PDXP |
| POLR2G | RNA Polymerase II Subunit G | Protein Coding | 37 | GC11P 062784 | 1.004 889 | https://www.genecards .org/cgi- |

| | | | | | | |
|---|---|---|---|---|---|---|
| | | | | | | bin/carddisp.pl?gene=POLR2G |
| PPIP5K2 | Diphosphoinositol Pentakisphosphate Kinase 2 | Protein Coding | 37 | GC05P 103120 | 1.004 889 | https://www.genecards.org/cgi-bin/carddisp.pl?gene=PPIP5K2 |
| PSMD5 | Proteasome 26S Subunit, Non-ATPase 5 | Protein Coding | 37 | GC09M 120815 | 1.004 889 | https://www.genecards.org/cgi-bin/carddisp.pl?gene=PSMD5 |
| RAPGEF 6 | Rap Guanine Nucleotide Exchange Factor 6 | Protein Coding | 37 | GC05M 131423 | 1.004 889 | https://www.genecards.org/cgi-bin/carddisp.pl?gene=RAPGEF6 |
| RBFOX1 | RNA Binding Fox-1 Homolog 1 | Protein Coding | 37 | GC16P 011816 | 1.004 889 | https://www.genecards.org/cgi-bin/carddisp.pl?gene=RBFOX1 |
| RSL1D1 | Ribosomal L1 Domain Containing 1 | Protein Coding | 37 | GC16M 011833 | 1.004 889 | https://www.genecards.org/cgi-bin/carddisp.pl?gene=RSL1D1 |
| SESN3 | Sestrin 3 | Protein Coding | 37 | GC11M 096560 | 1.004 889 | https://www.genecards.org/cgi-bin/carddisp.pl?gene=SESN3 |
| SLC26A1 | Solute Carrier Family 26 Member 1 | Protein Coding | 37 | GC04M 001530 | 1.004 889 | https://www.genecards.org/cgi-bin/carddisp.pl?gene=SLC26A1 |
| SMG7 | SMG7 Nonsense Mediated MRNA Decay Factor | Protein Coding | 37 | GC01P 183441 | 1.004 889 | https://www.genecards.org/cgi-bin/carddisp.pl?gene=SMG7 |
| SNAPC4 | Small Nuclear RNA Activating Complex Polypeptide 4 | Protein Coding | 37 | GC09M 136375 | 1.004 889 | https://www.genecards.org/cgi-bin/carddisp.pl?gene=SNAPC4 |
| SSBP3 | Single Stranded DNA Binding Protein 3 | Protein Coding | 37 | GC01M 054225 | 1.004 889 | https://www.genecards.org/cgi-bin/carddisp.pl?gene=SSBP3 |
| TPCN2 | Two Pore Segment Channel 2 | Protein Coding | 37 | GC11P 070233 | 1.004 889 | https://www.genecards.org/cgi-bin/carddisp.pl?gene=TPCN2 |
| ADGRA3 | Adhesion G Protein-Coupled Receptor A3 | Protein Coding | 36 | GC04M 022345 | 1.004 889 | https://www.genecards.org/cgi-bin/carddisp.pl?gene=ADGRA3 |
| AUP1 | AUP1 Lipid Droplet Regulating VLDL Assembly Factor | Protein Coding | 36 | GC02M 074526 | 1.004 889 | https://www.genecards.org/cgi-bin/carddisp.pl?gene=AUP1 |

| | | | | | | |
|---|---|---|---|---|---|---|
| BOLL | Boule Homolog, RNA Binding Protein | Protein Coding | 36 | GC02M 197726 | 1.004 889 | https://www.genecards .org/cgi-bin/carddisp.pl?gene= BOLL |
| CEP68 | Centrosomal Protein 68 | Protein Coding | 36 | GC02P 065056 | 1.004 889 | https://www.genecards .org/cgi-bin/carddisp.pl?gene= CEP68 |
| CNTRL | Centriolin | Protein Coding | 36 | GC09P 121074 | 1.004 889 | https://www.genecards .org/cgi-bin/carddisp.pl?gene= CNTRL |
| CSMD3 | CUB And Sushi Multiple Domains 3 | Protein Coding | 36 | GC08M 112223 | 1.004 889 | https://www.genecards .org/cgi-bin/carddisp.pl?gene= CSMD3 |
| DOK6 | Docking Protein 6 | Protein Coding | 36 | GC18P 069401 | 1.004 889 | https://www.genecards .org/cgi-bin/carddisp.pl?gene= DOK6 |
| FAM136 A | Family With Sequence Similarity 136 Member A | Protein Coding | 36 | GC02M 070296 | 1.004 889 | https://www.genecards .org/cgi-bin/carddisp.pl?gene= FAM136A |
| GPR25 | G Protein-Coupled Receptor 25 | Protein Coding | 36 | GC01P 200872 | 1.004 889 | https://www.genecards .org/cgi-bin/carddisp.pl?gene= GPR25 |
| KCNG2 | Potassium Voltage-Gated Channel Modifier Subfamily G Member 2 | Protein Coding | 36 | GC18P 079797 | 1.004 889 | https://www.genecards .org/cgi-bin/carddisp.pl?gene= KCNG2 |
| KIN | Kin17 DNA And RNA Binding Protein | Protein Coding | 36 | GC10M 007750 | 1.004 889 | https://www.genecards .org/cgi-bin/carddisp.pl?gene= KIN |
| NRIP3 | Nuclear Receptor Interacting Protein 3 | Protein Coding | 36 | GC11M 008966 | 1.004 889 | https://www.genecards .org/cgi-bin/carddisp.pl?gene= NRIP3 |
| RBM22 | RNA Binding Motif Protein 22 | Protein Coding | 36 | GC05M 150690 | 1.004 889 | https://www.genecards .org/cgi-bin/carddisp.pl?gene= RBM22 |
| RBM6 | RNA Binding Motif Protein 6 | Protein Coding | 36 | GC03P 049940 | 1.004 889 | https://www.genecards .org/cgi-bin/carddisp.pl?gene= RBM6 |
| RPS25 | Ribosomal Protein S25 | Protein Coding | 36 | GC11M 119015 | 1.004 889 | https://www.genecards .org/cgi-bin/carddisp.pl?gene= RPS25 |
| RSPH3 | Radial Spoke Head 3 | Protein Coding | 36 | GC06M 158962 | 1.004 889 | https://www.genecards .org/cgi- |

| | | | | | | |
|---|---|---|---|---|---|---|
| | | | | | | bin/carddisp.pl?gene=RSPH3 |
| SEC16A | SEC16 Homolog A, Endoplasmic Reticulum Export Factor | Protein Coding | 36 | GC09M136440 | 1.004889 | https://www.genecards.org/cgi-bin/carddisp.pl?gene=SEC16A |
| SLU7 | SLU7 Homolog, Splicing Factor | Protein Coding | 36 | GC05M160401 | 1.004889 | https://www.genecards.org/cgi-bin/carddisp.pl?gene=SLU7 |
| SMC1B | Structural Maintenance Of Chromosomes 1B | Protein Coding | 36 | GC22M045344 | 1.004889 | https://www.genecards.org/cgi-bin/carddisp.pl?gene=SMC1B |
| SNX20 | Sorting Nexin 20 | Protein Coding | 36 | GC16M050700 | 1.004889 | https://www.genecards.org/cgi-bin/carddisp.pl?gene=SNX20 |
| UPK2 | Uroplakin 2 | Protein Coding | 36 | GC11P118925 | 1.004889 | https://www.genecards.org/cgi-bin/carddisp.pl?gene=UPK2 |
| ZC3H7A | Zinc Finger CCCH-Type Containing 7A | Protein Coding | 36 | GC16M011750 | 1.004889 | https://www.genecards.org/cgi-bin/carddisp.pl?gene=ZC3H7A |
| FBXW2 | F-Box And WD Repeat Domain Containing 2 | Protein Coding | 35 | GC09M120751 | 1.004889 | https://www.genecards.org/cgi-bin/carddisp.pl?gene=FBXW2 |
| FBXW8 | F-Box And WD Repeat Domain Containing 8 | Protein Coding | 35 | GC12P116910 | 1.004889 | https://www.genecards.org/cgi-bin/carddisp.pl?gene=FBXW8 |
| NEURL1 | Neuralized E3 Ubiquitin Protein Ligase 1 | Protein Coding | 35 | GC10P103493 | 1.004889 | https://www.genecards.org/cgi-bin/carddisp.pl?gene=NEURL1 |
| PCGF1 | Polycomb Group Ring Finger 1 | Protein Coding | 35 | GC02M074505 | 1.004889 | https://www.genecards.org/cgi-bin/carddisp.pl?gene=PCGF1 |
| PHLDB1 | Pleckstrin Homology Like Domain Family B Member 1 | Protein Coding | 35 | GC11P118606 | 1.004889 | https://www.genecards.org/cgi-bin/carddisp.pl?gene=PHLDB1 |
| RPAIN | RPA Interacting Protein | Protein Coding | 35 | GC17P005419 | 1.004889 | https://www.genecards.org/cgi-bin/carddisp.pl?gene=RPAIN |
| SAMD4A | Sterile Alpha Motif Domain Containing 4A | Protein Coding | 35 | GC14P054567 | 1.004889 | https://www.genecards.org/cgi-bin/carddisp.pl?gene=SAMD4A |

| | | | | | | |
|---|---|---|---|---|---|---|
| SDF2L1 | Stromal Cell Derived Factor 2 Like 1 | Protein Coding | 35 | GC22P 035927 | 1.004 889 | https://www.genecards.org/cgi-bin/carddisp.pl?gene=SDF2L1 |
| SERBP1 | SERPINE1 MRNA Binding Protein 1 | Protein Coding | 35 | GC01M 067407 | 1.004 889 | https://www.genecards.org/cgi-bin/carddisp.pl?gene=SERBP1 |
| STRBP | Spermatid Perinuclear RNA Binding Protein | Protein Coding | 35 | GC09M 123681 | 1.004 889 | https://www.genecards.org/cgi-bin/carddisp.pl?gene=STRBP |
| SYTL3 | Synaptotagmin Like 3 | Protein Coding | 35 | GC06P 158644 | 1.004 889 | https://www.genecards.org/cgi-bin/carddisp.pl?gene=SYTL3 |
| TMEM30 B | Transmembrane Protein 30B | Protein Coding | 35 | GC14M 061277 | 1.004 889 | https://www.genecards.org/cgi-bin/carddisp.pl?gene=TMEM30B |
| TRMT11 2 | TRNA Methyltransferase Activator Subunit 11-2 | Protein Coding | 35 | GC11M 064316 | 1.004 889 | https://www.genecards.org/cgi-bin/carddisp.pl?gene=TRMT112 |
| TYSND1 | Trypsin Like Peroxisomal Matrix Peptidase 1 | Protein Coding | 35 | GC10M 070137 | 1.004 889 | https://www.genecards.org/cgi-bin/carddisp.pl?gene=TYSND1 |
| ASB8 | Ankyrin Repeat And SOCS Box Containing 8 | Protein Coding | 34 | GC12M 048147 | 1.004 889 | https://www.genecards.org/cgi-bin/carddisp.pl?gene=ASB8 |
| ATG14 | Autophagy Related 14 | Protein Coding | 34 | GC14M 055366 | 1.004 889 | https://www.genecards.org/cgi-bin/carddisp.pl?gene=ATG14 |
| BCL7C | BAF Chromatin Remodeling Complex Subunit BCL7C | Protein Coding | 34 | GC16M 037209 | 1.004 889 | https://www.genecards.org/cgi-bin/carddisp.pl?gene=BCL7C |
| BCL9L | BCL9 Like | Protein Coding | 34 | GC11M 118893 | 1.004 889 | https://www.genecards.org/cgi-bin/carddisp.pl?gene=BCL9L |
| COL26A 1 | Collagen Type XXVI Alpha 1 Chain | Protein Coding | 34 | GC07P 101362 | 1.004 889 | https://www.genecards.org/cgi-bin/carddisp.pl?gene=COL26A1 |
| CPSF7 | Cleavage And Polyadenylation Specific Factor 7 | Protein Coding | 34 | GC11M 061402 | 1.004 889 | https://www.genecards.org/cgi-bin/carddisp.pl?gene=CPSF7 |
| DUSP11 | Dual Specificity Phosphatase 11 | Protein Coding | 34 | GC02M 073788 | 1.004 889 | https://www.genecards.org/cgi- |

| | | | | | | |
|---|---|---|---|---|---|---|
| | | | | | | bin/carddisp.pl?gene=DUSP11 |
| DYDC1 | DPY30 Domain Containing 1 | Protein Coding | 34 | GC10M080336 | 1.004889 | https://www.genecards.org/cgi-bin/carddisp.pl?gene=DYDC1 |
| FAM76B | Family With Sequence Similarity 76 Member B | Protein Coding | 34 | GC11M095768 | 1.004889 | https://www.genecards.org/cgi-bin/carddisp.pl?gene=FAM76B |
| GPR174 | G Protein-Coupled Receptor 174 | Protein Coding | 34 | GC0XP079170 | 1.004889 | https://www.genecards.org/cgi-bin/carddisp.pl?gene=GPR174 |
| HORMAD2 | HORMA Domain Containing 2 | Protein Coding | 34 | GC22P036840 | 1.004889 | https://www.genecards.org/cgi-bin/carddisp.pl?gene=HORMAD2 |
| KCP | Kielin Cysteine Rich BMP Regulator | Protein Coding | 34 | GC07M128862 | 1.004889 | https://www.genecards.org/cgi-bin/carddisp.pl?gene=KCP |
| MAGI3 | Membrane Associated Guanylate Kinase, WW And PDZ Domain Containing 3 | Protein Coding | 34 | GC01P113390 | 1.004889 | https://www.genecards.org/cgi-bin/carddisp.pl?gene=MAGI3 |
| MRPL34 | Mitochondrial Ribosomal Protein L34 | Protein Coding | 34 | GC19P066222 | 1.004889 | https://www.genecards.org/cgi-bin/carddisp.pl?gene=MRPL34 |
| MRPL4 | Mitochondrial Ribosomal Protein L4 | Protein Coding | 34 | GC19P010251 | 1.004889 | https://www.genecards.org/cgi-bin/carddisp.pl?gene=MRPL4 |
| OTUD3 | OTU Deubiquitinase 3 | Protein Coding | 34 | GC01P019881 | 1.004889 | https://www.genecards.org/cgi-bin/carddisp.pl?gene=OTUD3 |
| QRICH1 | Glutamine Rich 1 | Protein Coding | 34 | GC03M051163 | 1.004889 | https://www.genecards.org/cgi-bin/carddisp.pl?gene=QRICH1 |
| SNAPC1 | Small Nuclear RNA Activating Complex Polypeptide 1 | Protein Coding | 34 | GC14P061762 | 1.004889 | https://www.genecards.org/cgi-bin/carddisp.pl?gene=SNAPC1 |
| TCTE1 | T-Complex-Associated-Testis-Expressed 1 | Protein Coding | 34 | GC06M044278 | 1.004889 | https://www.genecards.org/cgi-bin/carddisp.pl?gene=TCTE1 |
| TIFA | TRAF Interacting Protein With Forkhead Associated Domain | Protein Coding | 34 | GC04M112274 | 1.004889 | https://www.genecards.org/cgi-bin/carddisp.pl?gene=TIFA |

| | | | | | | |
|---|---|---|---|---|---|---|
| TRIAP1 | TP53 Regulated Inhibitor Of Apoptosis 1 | Protein Coding | 34 | GC12M 120443 | 1.004 889 | https://www.genecards .org/cgi-bin/carddisp.pl?gene= TRIAP1 |
| TSPAN14 | Tetraspanin 14 | Protein Coding | 34 | GC10P 092360 | 1.004 889 | https://www.genecards .org/cgi-bin/carddisp.pl?gene= TSPAN14 |
| ZNF366 | Zinc Finger Protein 366 | Protein Coding | 34 | GC05M 073593 | 1.004 889 | https://www.genecards .org/cgi-bin/carddisp.pl?gene= ZNF366 |
| C5orf15 | Chromosome 5 Open Reading Frame 15 | Protein Coding | 33 | GC05M 133955 | 1.004 889 | https://www.genecards .org/cgi-bin/carddisp.pl?gene= C5orf15 |
| CCDC69 | Coiled-Coil Domain Containing 69 | Protein Coding | 33 | GC05M 151181 | 1.004 889 | https://www.genecards .org/cgi-bin/carddisp.pl?gene= CCDC69 |
| CNOT6L | CCR4-NOT Transcription Complex Subunit 6 Like | Protein Coding | 33 | GC04M 077713 | 1.004 889 | https://www.genecards .org/cgi-bin/carddisp.pl?gene= CNOT6L |
| DYDC2 | DPY30 Domain Containing 2 | Protein Coding | 33 | GC10P 080344 | 1.004 889 | https://www.genecards .org/cgi-bin/carddisp.pl?gene= DYDC2 |
| FOXR1 | Forkhead Box R1 | Protein Coding | 33 | GC11P 118971 | 1.004 889 | https://www.genecards .org/cgi-bin/carddisp.pl?gene= FOXR1 |
| GPR137 | G Protein-Coupled Receptor 137 | Protein Coding | 33 | GC11P 064270 | 1.004 889 | https://www.genecards .org/cgi-bin/carddisp.pl?gene= GPR137 |
| KANSL2 | KAT8 Regulatory NSL Complex Subunit 2 | Protein Coding | 33 | GC12M 048653 | 1.004 889 | https://www.genecards .org/cgi-bin/carddisp.pl?gene= KANSL2 |
| KIF26A | Kinesin Family Member 26A | Protein Coding | 33 | GC14P 104138 | 1.004 889 | https://www.genecards .org/cgi-bin/carddisp.pl?gene= KIF26A |
| MIER1 | MIER1 Transcriptional Regulator | Protein Coding | 33 | GC01P 066924 | 1.004 889 | https://www.genecards .org/cgi-bin/carddisp.pl?gene= MIER1 |
| NECAB2 | N-Terminal EF-Hand Calcium Binding Protein 2 | Protein Coding | 33 | GC16P 083968 | 1.004 889 | https://www.genecards .org/cgi-bin/carddisp.pl?gene= NECAB2 |
| NHLH1 | Nescient Helix-Loop-Helix 1 | Protein Coding | 33 | GC01P 160367 | 1.004 889 | https://www.genecards .org/cgi- |

| | | | | | | |
|---|---|---|---|---|---|---|
| | | | | | | bin/carddisp.pl?gene=NHLH1 |
| ORAI3 | ORAI Calcium Release-Activated Calcium Modulator 3 | Protein Coding | 33 | GC16P030949 | 1.004889 | https://www.genecards.org/cgi-bin/carddisp.pl?gene=ORAI3 |
| RNF186 | Ring Finger Protein 186 | Protein Coding | 33 | GC01M019814 | 1.004889 | https://www.genecards.org/cgi-bin/carddisp.pl?gene=RNF186 |
| RPAP3 | RNA Polymerase II Associated Protein 3 | Protein Coding | 33 | GC12M047661 | 1.004889 | https://www.genecards.org/cgi-bin/carddisp.pl?gene=RPAP3 |
| SCT | Secretin | Protein Coding | 33 | GC11M000626 | 1.004889 | https://www.genecards.org/cgi-bin/carddisp.pl?gene=SCT |
| SEL1L3 | SEL1L Family Member 3 | Protein Coding | 33 | GC04M025715 | 1.004889 | https://www.genecards.org/cgi-bin/carddisp.pl?gene=SEL1L3 |
| SNN | Stannin | Protein Coding | 33 | GC16P012203 | 1.004889 | https://www.genecards.org/cgi-bin/carddisp.pl?gene=SNN |
| TMEM175 | Transmembrane Protein 175 | Protein Coding | 33 | GC04P000932 | 1.004889 | https://www.genecards.org/cgi-bin/carddisp.pl?gene=TMEM175 |
| TMEM50B | Transmembrane Protein 50B | Protein Coding | 33 | GC21M033432 | 1.004889 | https://www.genecards.org/cgi-bin/carddisp.pl?gene=TMEM50B |
| ZBTB21 | Zinc Finger And BTB Domain Containing 21 | Protein Coding | 33 | GC21M041986 | 1.004889 | https://www.genecards.org/cgi-bin/carddisp.pl?gene=ZBTB21 |
| ZNF641 | Zinc Finger Protein 641 | Protein Coding | 33 | GC12M049432 | 1.004889 | https://www.genecards.org/cgi-bin/carddisp.pl?gene=ZNF641 |
| ZNF689 | Zinc Finger Protein 689 | Protein Coding | 33 | GC16M030602 | 1.004889 | https://www.genecards.org/cgi-bin/carddisp.pl?gene=ZNF689 |
| ZNF764 | Zinc Finger Protein 764 | Protein Coding | 33 | GC16M030553 | 1.004889 | https://www.genecards.org/cgi-bin/carddisp.pl?gene=ZNF764 |
| ADNP2 | ADNP Homeobox 2 | Protein Coding | 32 | GC18P080109 | 1.004889 | https://www.genecards.org/cgi-bin/carddisp.pl?gene=ADNP2 |

| | | | | | | |
|---|---|---|---|---|---|---|
| AP1AR | Adaptor Related Protein Complex 1 Associated Regulatory Protein | Protein Coding | 32 | GC04P 112231 | 1.004 889 | https://www.genecards.org/cgi-bin/carddisp.pl?gene=AP1AR |
| C2CD2 | C2 Calcium Dependent Domain Containing 2 | Protein Coding | 32 | GC21M 041885 | 1.004 889 | https://www.genecards.org/cgi-bin/carddisp.pl?gene=C2CD2 |
| CALHM2 | Calcium Homeostasis Modulator Family Member 2 | Protein Coding | 32 | GC10M 103446 | 1.004 889 | https://www.genecards.org/cgi-bin/carddisp.pl?gene=CALHM2 |
| CAMTA2 | Calmodulin Binding Transcription Activator 2 | Protein Coding | 32 | GC17M 006122 | 1.004 889 | https://www.genecards.org/cgi-bin/carddisp.pl?gene=CAMTA2 |
| CYB561 A3 | Cytochrome B561 Family Member A3 | Protein Coding | 32 | GC11M 061348 | 1.004 889 | https://www.genecards.org/cgi-bin/carddisp.pl?gene=CYB561A3 |
| DNAJC2 8 | DnaJ Heat Shock Protein Family (Hsp40) Member C28 | Protein Coding | 32 | GC21M 033485 | 1.004 889 | https://www.genecards.org/cgi-bin/carddisp.pl?gene=DNAJC28 |
| FAM98B | Family With Sequence Similarity 98 Member B | Protein Coding | 32 | GC15P 038454 | 1.004 889 | https://www.genecards.org/cgi-bin/carddisp.pl?gene=FAM98B |
| FBRS | Fibrosin | Protein Coding | 32 | GC16P 041093 | 1.004 889 | https://www.genecards.org/cgi-bin/carddisp.pl?gene=FBRS |
| GPR31 | G Protein-Coupled Receptor 31 | Protein Coding | 32 | GC06M 167156 | 1.004 889 | https://www.genecards.org/cgi-bin/carddisp.pl?gene=GPR31 |
| IFT46 | Intraflagellar Transport 46 | Protein Coding | 32 | GC11M 118544 | 1.004 889 | https://www.genecards.org/cgi-bin/carddisp.pl?gene=IFT46 |
| INO80B | INO80 Complex Subunit B | Protein Coding | 32 | GC02P 074456 | 1.004 889 | https://www.genecards.org/cgi-bin/carddisp.pl?gene=INO80B |
| KCTD20 | Potassium Channel Tetramerization Domain Containing 20 | Protein Coding | 32 | GC06P 083845 | 1.004 889 | https://www.genecards.org/cgi-bin/carddisp.pl?gene=KCTD20 |
| PRR14 | Proline Rich 14 | Protein Coding | 32 | GC16P 041089 | 1.004 889 | https://www.genecards.org/cgi-bin/carddisp.pl?gene=PRR14 |
| RPP25L | Ribonuclease P/MRP Subunit P25 Like | Protein Coding | 32 | GC09M 034610 | 1.004 889 | https://www.genecards.org/cgi- |

| | | | | | | |
|---|---|---|---|---|---|---|
| | | | | | | bin/carddisp.pl?gene=RPP25L |
| SH2D4B | SH2 Domain Containing 4B | Protein Coding | 32 | GC10P092361 | 1.004889 | https://www.genecards.org/cgi-bin/carddisp.pl?gene=SH2D4B |
| TMEM106C | Transmembrane Protein 106C | Protein Coding | 32 | GC12P047963 | 1.004889 | https://www.genecards.org/cgi-bin/carddisp.pl?gene=TMEM106C |
| TMEM204 | Transmembrane Protein 204 | Protein Coding | 32 | GC16P001528 | 1.004889 | https://www.genecards.org/cgi-bin/carddisp.pl?gene=TMEM204 |
| ZNF785 | Zinc Finger Protein 785 | Protein Coding | 32 | GC16M030573 | 1.004889 | https://www.genecards.org/cgi-bin/carddisp.pl?gene=ZNF785 |
| ABHD8 | Abhydrolase Domain Containing 8 | Protein Coding | 31 | GC19M017292 | 1.004889 | https://www.genecards.org/cgi-bin/carddisp.pl?gene=ABHD8 |
| EMSY | EMSY Transcriptional Repressor, BRCA2 Interacting | Protein Coding | 31 | GC11P077991 | 1.004889 | https://www.genecards.org/cgi-bin/carddisp.pl?gene=EMSY |
| HECTD4 | HECT Domain E3 Ubiquitin Protein Ligase 4 | Protein Coding | 31 | GC12M112160 | 1.004889 | https://www.genecards.org/cgi-bin/carddisp.pl?gene=HECTD4 |
| INO80E | INO80 Complex Subunit E | Protein Coding | 31 | GC16P041024 | 1.004889 | https://www.genecards.org/cgi-bin/carddisp.pl?gene=INO80E |
| MRPL53 | Mitochondrial Ribosomal Protein L53 | Protein Coding | 31 | GC02M074471 | 1.004889 | https://www.genecards.org/cgi-bin/carddisp.pl?gene=MRPL53 |
| OR10AD1 | Olfactory Receptor Family 10 Subfamily AD Member 1 | Protein Coding | 31 | GC12M048201 | 1.004889 | https://www.genecards.org/cgi-bin/carddisp.pl?gene=OR10AD1 |
| PRR12 | Proline Rich 12 | Protein Coding | 31 | GC19P049591 | 1.004889 | https://www.genecards.org/cgi-bin/carddisp.pl?gene=PRR12 |
| ZGRF1 | Zinc Finger GRF-Type Containing 1 | Protein Coding | 31 | GC04M112539 | 1.004889 | https://www.genecards.org/cgi-bin/carddisp.pl?gene=ZGRF1 |
| ZNF688 | Zinc Finger Protein 688 | Protein Coding | 31 | GC16M030569 | 1.004889 | https://www.genecards.org/cgi-bin/carddisp.pl?gene=ZNF688 |

| | | | | | | |
|---|---|---|---|---|---|---|
| BLCAP | BLCAP Apoptosis Inducing Factor | Protein Coding | 30 | GC20M037492 | 1.004889 | https://www.genecards.org/cgi-bin/carddisp.pl?gene=BLCAP |
| CCDC153 | Coiled-Coil Domain Containing 153 | Protein Coding | 30 | GC11M119207 | 1.004889 | https://www.genecards.org/cgi-bin/carddisp.pl?gene=CCDC153 |
| DNAJC4 | DnaJ Heat Shock Protein Family (Hsp40) Member C4 | Protein Coding | 30 | GC11P064230 | 1.004889 | https://www.genecards.org/cgi-bin/carddisp.pl?gene=DNAJC4 |
| IFT22 | Intraflagellar Transport 22 | Protein Coding | 30 | GC07M101986 | 1.004889 | https://www.genecards.org/cgi-bin/carddisp.pl?gene=IFT22 |
| PLAC9 | Placenta Associated 9 | Protein Coding | 30 | GC10P080131 | 1.004889 | https://www.genecards.org/cgi-bin/carddisp.pl?gene=PLAC9 |
| RBFA | Ribosome Binding Factor A | Protein Coding | 30 | GC18P080068 | 1.004889 | https://www.genecards.org/cgi-bin/carddisp.pl?gene=RBFA |
| TMEM109 | Transmembrane Protein 109 | Protein Coding | 30 | GC11P060914 | 1.004889 | https://www.genecards.org/cgi-bin/carddisp.pl?gene=TMEM109 |
| ZGLP1 | Zinc Finger GATA Like Protein 1 | Protein Coding | 30 | GC19M010304 | 1.004889 | https://www.genecards.org/cgi-bin/carddisp.pl?gene=ZGLP1 |
| ANKRD33B | Ankyrin Repeat Domain 33B | Protein Coding | 29 | GC05P010564 | 1.004889 | https://www.genecards.org/cgi-bin/carddisp.pl?gene=ANKRD33B |
| FAM133B | Family With Sequence Similarity 133 Member B | Protein Coding | 29 | GC07M092560 | 1.004889 | https://www.genecards.org/cgi-bin/carddisp.pl?gene=FAM133B |
| SLX1A | SLX1 Homolog A, Structure-Specific Endonuclease Subunit | Protein Coding | 29 | GC16P041045 | 1.004889 | https://www.genecards.org/cgi-bin/carddisp.pl?gene=SLX1A |
| TMEM151B | Transmembrane Protein 151B | Protein Coding | 29 | GC06P044270 | 1.004889 | https://www.genecards.org/cgi-bin/carddisp.pl?gene=TMEM151B |
| ZNF679 | Zinc Finger Protein 679 | Protein Coding | 29 | GC07P064228 | 1.004889 | https://www.genecards.org/cgi-bin/carddisp.pl?gene=ZNF679 |
| C14orf180 | Chromosome 14 Open Reading Frame 180 | Protein Coding | 28 | GC14P104579 | 1.004889 | https://www.genecards.org/cgi- |

| | | | | | | |
|---|---|---|---|---|---|---|
| | | | | | | bin/carddisp.pl?gene=C14orf180 |
| C2orf74 | Chromosome 2 Open Reading Frame 74 | Protein Coding | 28 | GC02P061146 | 1.004889 | https://www.genecards.org/cgi-bin/carddisp.pl?gene=C2orf74 |
| CCDC142 | Coiled-Coil Domain Containing 142 | Protein Coding | 28 | GC02M075091 | 1.004889 | https://www.genecards.org/cgi-bin/carddisp.pl?gene=CCDC142 |
| DENND2B | DENN Domain Containing 2B | Protein Coding | 28 | GC11M008753 | 1.004889 | https://www.genecards.org/cgi-bin/carddisp.pl?gene=DENND2B |
| DNLZ | DNL-Type Zinc Finger | Protein Coding | 28 | GC09M136359 | 1.004889 | https://www.genecards.org/cgi-bin/carddisp.pl?gene=DNLZ |
| PTX4 | Pentraxin 4 | Protein Coding | 28 | GC16M001485 | 1.004889 | https://www.genecards.org/cgi-bin/carddisp.pl?gene=PTX4 |
| SMIM20 | Small Integral Membrane Protein 20 | Protein Coding | 28 | GC04P025867 | 1.004889 | https://www.genecards.org/cgi-bin/carddisp.pl?gene=SMIM20 |
| TMEM179 | Transmembrane Protein 179 | Protein Coding | 28 | GC14M104477 | 1.004889 | https://www.genecards.org/cgi-bin/carddisp.pl?gene=TMEM179 |
| TTC31 | Tetratricopeptide Repeat Domain 31 | Protein Coding | 28 | GC02P074483 | 1.004889 | https://www.genecards.org/cgi-bin/carddisp.pl?gene=TTC31 |
| ABHD16B | Abhydrolase Domain Containing 16B | Protein Coding | 27 | GC20P063861 | 1.004889 | https://www.genecards.org/cgi-bin/carddisp.pl?gene=ABHD16B |
| ENTR1 | Endosome Associated Trafficking Regulator 1 | Protein Coding | 27 | GC09M136403 | 1.004889 | https://www.genecards.org/cgi-bin/carddisp.pl?gene=ENTR1 |
| MACROH2A2 | MacroH2A.2 Histone | Protein Coding | 27 | GC10P070053 | 1.004889 | https://www.genecards.org/cgi-bin/carddisp.pl?gene=MACROH2A2 |
| TMEM235 | Transmembrane Protein 235 | Protein Coding | 27 | GC17P078231 | 1.004889 | https://www.genecards.org/cgi-bin/carddisp.pl?gene=TMEM235 |
| CCDC184 | Coiled-Coil Domain Containing 184 | Protein Coding | 26 | GC12P048183 | 1.004889 | https://www.genecards.org/cgi-bin/carddisp.pl?gene=CCDC184 |

| | | | | | | |
|---|---|---|---|---|---|---|
| CDHR4 | Cadherin Related Family Member 4 | Protein Coding | 26 | GC03M 049790 | 1.004 889 | https://www.genecards .org/cgi-bin/carddisp.pl?gene= CDHR4 |
| CYRIB | CYFIP Related Rac1 Interactor B | Protein Coding | 26 | GC08M 131809 | 1.004 889 | https://www.genecards .org/cgi-bin/carddisp.pl?gene= CYRIB |
| DEFB136 | Defensin Beta 136 | Protein Coding | 26 | GC08M 011973 | 1.004 889 | https://www.genecards .org/cgi-bin/carddisp.pl?gene= DEFB136 |
| FAM205 A | Family With Sequence Similarity 205 Member A | Protein Coding | 26 | GC09M 034723 | 1.004 889 | https://www.genecards .org/cgi-bin/carddisp.pl?gene= FAM205A |
| PRXL2A | Peroxiredoxin Like 2A | Protein Coding | 26 | GC10P 093417 | 1.004 889 | https://www.genecards .org/cgi-bin/carddisp.pl?gene= PRXL2A |
| TMEM11 6 | Transmembrane Protein 116 | Protein Coding | 26 | GC12M 111894 | 1.004 889 | https://www.genecards .org/cgi-bin/carddisp.pl?gene= TMEM116 |
| PHF24 | PHD Finger Protein 24 | Protein Coding | 25 | GC09P 047597 | 1.004 889 | https://www.genecards .org/cgi-bin/carddisp.pl?gene= PHF24 |
| HSPA7 | Heat Shock Protein Family A (Hsp70) Member 7 (Pseudogene) | Pseudo gene | 24 | GC01P 161606 | 1.004 889 | https://www.genecards .org/cgi-bin/carddisp.pl?gene= HSPA7 |
| PRXL2B | Peroxiredoxin Like 2B | Protein Coding | 24 | GC01P 003939 | 1.004 889 | https://www.genecards .org/cgi-bin/carddisp.pl?gene= PRXL2B |
| SANBR | SANT And BTB Domain Regulator Of CSR | Protein Coding | 24 | GC02P 061068 | 1.004 889 | https://www.genecards .org/cgi-bin/carddisp.pl?gene= SANBR |
| CENATA C | Centrosomal AT-AC Splicing Factor | Protein Coding | 23 | GC11P 120147 | 1.004 889 | https://www.genecards .org/cgi-bin/carddisp.pl?gene= CENATAC |
| CLBA1 | Clathrin Binding Box Of Aftiphilin Containing 1 | Protein Coding | 22 | GC14P 109741 | 1.004 889 | https://www.genecards .org/cgi-bin/carddisp.pl?gene= CLBA1 |
| FAM241 A | Family With Sequence Similarity 241 Member A | Protein Coding | 22 | GC04P 112146 | 1.004 889 | https://www.genecards .org/cgi-bin/carddisp.pl?gene= FAM241A |
| LINC026 94 | Long Intergenic Non-Protein Coding RNA 2694 | RNA Gene | 22 | GC15P 042443 | 1.004 889 | https://www.genecards .org/cgi- |

| | | | | | | |
|---|---|---|---|---|---|---|
| | | | | | | bin/carddisp.pl?gene=LINC02694 |
| BNIP5 | BCL2 Interacting Protein 5 | Protein Coding | 21 | GC06M066470 | 1.004889 | https://www.genecards.org/cgi-bin/carddisp.pl?gene=BNIP5 |
| CFAP119 | Cilia And Flagella Associated Protein 119 | Protein Coding | 21 | GC16M037473 | 1.004889 | https://www.genecards.org/cgi-bin/carddisp.pl?gene=CFAP119 |
| CFAP20DC | CFAP20 Domain Containing | Protein Coding | 21 | GC03M058716 | 1.004889 | https://www.genecards.org/cgi-bin/carddisp.pl?gene=CFAP20DC |
| H1-7 | H1.7 Linker Histone | Protein Coding | 21 | GC12P048329 | 1.004889 | https://www.genecards.org/cgi-bin/carddisp.pl?gene=H1-7 |
| SCARNA5 | Small Cajal Body-Specific RNA 5 | RNA Gene | 21 | GC02P233275 | 1.004889 | https://www.genecards.org/cgi-bin/carddisp.pl?gene=SCARNA5 |
| MIR600HG | MIR600 Host Gene | RNA Gene | 20 | GC09M123109 | 1.004889 | https://www.genecards.org/cgi-bin/carddisp.pl?gene=MIR600HG |
| PMS2P3 | PMS1 Homolog 2, Mismatch Repair System Component Pseudogene 3 | Pseudogene | 20 | GC07M075507 | 1.004889 | https://www.genecards.org/cgi-bin/carddisp.pl?gene=PMS2P3 |
| SPATA31D4 | SPATA31 Subfamily D Member 4 | Protein Coding | 20 | GC09P081928 | 1.004889 | https://www.genecards.org/cgi-bin/carddisp.pl?gene=SPATA31D4 |
| STAG3L2 | Stromal Antigen 3-Like 2 (Pseudogene) | Pseudogene | 20 | GC07M074843 | 1.004889 | https://www.genecards.org/cgi-bin/carddisp.pl?gene=STAG3L2 |
| HSP90AB2P | Heat Shock Protein 90 Alpha Family Class B Member 2, Pseudogene | Pseudogene | 19 | GC04P018152 | 1.004889 | https://www.genecards.org/cgi-bin/carddisp.pl?gene=HSP90AB2P |
| C21orf140 | Chromosome 21 Open Reading Frame 140 | Protein Coding | 18 | GC21M034400 | 1.004889 | https://www.genecards.org/cgi-bin/carddisp.pl?gene=C21orf140 |
| CELF2-AS1 | CELF2 Antisense RNA 1 | RNA Gene | 18 | GC10M011277 | 1.004889 | https://www.genecards.org/cgi-bin/carddisp.pl?gene=CELF2-AS1 |
| DGCR11 | DiGeorge Syndrome Critical Region Gene 11 | RNA Gene | 18 | GC22M019046 | 1.004889 | https://www.genecards.org/cgi-bin/carddisp.pl?gene=DGCR11 |

| | | | | | | |
|---|---|---|---|---|---|---|
| HOXA10-AS | HOXA10 Antisense RNA | RNA Gene | 18 | GC07P027735 | 1.004889 | https://www.genecards.org/cgi-bin/carddisp.pl?gene=HOXA10-AS |
| LEF1-AS1 | LEF1 Antisense RNA 1 | RNA Gene | 18 | GC04P108167 | 1.004889 | https://www.genecards.org/cgi-bin/carddisp.pl?gene=LEF1-AS1 |
| LINC00323 | Long Intergenic Non-Protein Coding RNA 323 | RNA Gene | 18 | GC21M041141 | 1.004889 | https://www.genecards.org/cgi-bin/carddisp.pl?gene=LINC00323 |
| PMS2P5 | PMS1 Homolog 2, Mismatch Repair System Component Pseudogene 5 | Pseudogene | 18 | GC07P074890 | 1.004889 | https://www.genecards.org/cgi-bin/carddisp.pl?gene=PMS2P5 |
| DGUOK-AS1 | DGUOK Antisense RNA 1 | RNA Gene | 17 | GC02M073947 | 1.004889 | https://www.genecards.org/cgi-bin/carddisp.pl?gene=DGUOK-AS1 |
| LINC00158 | Long Intergenic Non-Protein Coding RNA 158 | RNA Gene | 17 | GC21M025385 | 1.004889 | https://www.genecards.org/cgi-bin/carddisp.pl?gene=LINC00158 |
| LINC01088 | Long Intergenic Non-Protein Coding RNA 1088 | RNA Gene | 17 | GC04P078971 | 1.004889 | https://www.genecards.org/cgi-bin/carddisp.pl?gene=LINC01088 |
| SBF2-AS1 | SBF2 Antisense RNA 1 | RNA Gene | 17 | GC11P009758 | 1.004889 | https://www.genecards.org/cgi-bin/carddisp.pl?gene=SBF2-AS1 |
| ADAM1A | ADAM Metallopeptidase Domain 1A (Pseudogene) | Pseudogene | 16 | GC12P111899 | 1.004889 | https://www.genecards.org/cgi-bin/carddisp.pl?gene=ADAM1A |
| GATA3-AS1 | GATA3 Antisense RNA 1 | RNA Gene | 16 | GC10M008016 | 1.004889 | https://www.genecards.org/cgi-bin/carddisp.pl?gene=GATA3-AS1 |
| HIF1A-AS1 | HIF1A Antisense RNA 1 | RNA Gene | 16 | GC14M061681 | 1.004889 | https://www.genecards.org/cgi-bin/carddisp.pl?gene=HIF1A-AS1 |
| JAZF1-AS1 | JAZF1 Antisense RNA 1 | RNA Gene | 16 | GC07P028180 | 1.004889 | https://www.genecards.org/cgi-bin/carddisp.pl?gene=JAZF1-AS1 |
| LINC00593 | Long Intergenic Non-Protein Coding RNA 593 | RNA Gene | 16 | GC15P117549 | 1.004889 | https://www.genecards.org/cgi-bin/carddisp.pl?gene=LINC00593 |
| LINC00977 | Long Intergenic Non-Protein Coding RNA 977 | RNA Gene | 16 | GC08M131797 | 1.004889 | https://www.genecards.org/cgi- |

| | | | | | | |
|---|---|---|---|---|---|---|
| | | | | | | bin/carddisp.pl?gene=LINC00977 |
| LINC01082 | Long Intergenic Non-Protein Coding RNA 1082 | RNA Gene | 16 | GC16P086065 | 1.004889 | https://www.genecards.org/cgi-bin/carddisp.pl?gene=LINC01082 |
| MIR181A1HG | MIR181A1 Host Gene | RNA Gene | 16 | GC01M198780 | 1.004889 | https://www.genecards.org/cgi-bin/carddisp.pl?gene=MIR181A1HG |
| PCAT29 | Prostate Cancer Associated Transcript 29 | RNA Gene | 16 | GC15P118926 | 1.004889 | https://www.genecards.org/cgi-bin/carddisp.pl?gene=PCAT29 |
| SNORA72 | Small Nucleolar RNA, H/ACA Box 72 | RNA Gene | 16 | GC08M098042 | 1.004889 | https://www.genecards.org/cgi-bin/carddisp.pl?gene=SNORA72 |
| TMEM9B-AS1 | TMEM9B Antisense RNA 1 | RNA Gene | 16 | GC11P008964 | 1.004889 | https://www.genecards.org/cgi-bin/carddisp.pl?gene=TMEM9B-AS1 |
| CELF2-AS2 | CELF2 Antisense RNA 2 | RNA Gene | 15 | GC10M011071 | 1.004889 | https://www.genecards.org/cgi-bin/carddisp.pl?gene=CELF2-AS2 |
| HTD2 | Hydroxyacyl-Thioester Dehydratase Type 2 | Protein Coding | 15 | GC03P058306 | 1.004889 | https://www.genecards.org/cgi-bin/carddisp.pl?gene=HTD2 |
| SENCR | Smooth Muscle And Endothelial Cell Enriched Migration/Differentiation-Associated LncRNA | RNA Gene | 15 | GC11M128691 | 1.004889 | https://www.genecards.org/cgi-bin/carddisp.pl?gene=SENCR |
| C20orf181 | Chromosome 20 Open Reading Frame 181 | Uncategorized | 14 | GC20M063844 | 1.004889 | https://www.genecards.org/cgi-bin/carddisp.pl?gene=C20orf181 |
| FLNB-AS1 | FLNB Antisense RNA 1 | RNA Gene | 14 | GC03M058150 | 1.004889 | https://www.genecards.org/cgi-bin/carddisp.pl?gene=FLNB-AS1 |
| LINC03033 | Long Intergenic Non-Protein Coding RNA 3033 | RNA Gene | 14 | GC14P061572 | 1.004889 | https://www.genecards.org/cgi-bin/carddisp.pl?gene=LINC03033 |
| MBL1P | Mannose Binding Lectin 1, Pseudogene | Pseudogene | 14 | GC10P092759 | 1.004889 | https://www.genecards.org/cgi-bin/carddisp.pl?gene=MBL1P |
| MIR1208 | MicroRNA 1208 | RNA Gene | 14 | GC08P128150 | 1.004889 | https://www.genecards.org/cgi-bin/carddisp.pl?gene=MIR1208 |

| | | | | | | |
|---|---|---|---|---|---|---|
| MIR3142 HG | MIR3142 Host Gene | RNA Gene | 14 | GC05P 160438 | 1.004 889 | https://www.genecards .org/cgi-bin/carddisp.pl?gene= MIR3142HG |
| MIR4437 | MicroRNA 4437 | RNA Gene | 14 | GC02M 181305 | 1.004 889 | https://www.genecards .org/cgi-bin/carddisp.pl?gene= MIR4437 |
| MIR718 | MicroRNA 718 | RNA Gene | 14 | GC0X M1540 19 | 1.004 889 | https://www.genecards .org/cgi-bin/carddisp.pl?gene= MIR718 |
| NPIPB2 | Nuclear Pore Complex Interacting Protein Family Member B2 | Protein Coding | 14 | GC16M 012293 | 1.004 889 | https://www.genecards .org/cgi-bin/carddisp.pl?gene= NPIPB2 |
| PRKCQ-AS1 | PRKCQ Antisense RNA 1 | RNA Gene | 14 | GC10P 006580 | 1.004 889 | https://www.genecards .org/cgi-bin/carddisp.pl?gene= PRKCQ-AS1 |
| SEMA3B -AS1 | SEMA3B Antisense RNA 1 (Head To Head) | RNA Gene | 14 | GC03M 051236 | 1.004 889 | https://www.genecards .org/cgi-bin/carddisp.pl?gene= SEMA3B-AS1 |
| TPI1P2 | Triosephosphate Isomerase 1 Pseudogene 2 | Pseudo gene | 14 | GC07P 129055 | 1.004 889 | https://www.genecards .org/cgi-bin/carddisp.pl?gene= TPI1P2 |
| BOLA3-DT | BOLA3 Divergent Transcript | RNA Gene | 13 | GC02P 074146 | 1.004 889 | https://www.genecards .org/cgi-bin/carddisp.pl?gene= BOLA3-DT |
| FAM205 BP | Family With Sequence Similarity 205 Member B, Pseudogene | Pseudo gene | 13 | GC09M 034837 | 1.004 889 | https://www.genecards .org/cgi-bin/carddisp.pl?gene= FAM205BP |
| LINC008 24 | Long Intergenic Non-Protein Coding RNA 824 | RNA Gene | 13 | GC08M 128405 | 1.004 889 | https://www.genecards .org/cgi-bin/carddisp.pl?gene= LINC00824 |
| MIR3202 -2 | MicroRNA 3202-2 | RNA Gene | 13 | GC0X M1539 81 | 1.004 889 | https://www.genecards .org/cgi-bin/carddisp.pl?gene= MIR3202-2 |
| MROH3P | Maestro Heat Like Repeat Family Member 3, Pseudogene | Pseudo gene | 13 | GC01P 200920 | 1.004 889 | https://www.genecards .org/cgi-bin/carddisp.pl?gene= MROH3P |
| NEURL1-AS1 | NEURL1 Antisense RNA 1 | RNA Gene | 13 | GC10M 103479 | 1.004 889 | https://www.genecards .org/cgi-bin/carddisp.pl?gene= NEURL1-AS1 |
| P4HA2-AS1 | P4HA2 Antisense RNA 1 | RNA Gene | 13 | GC05P 132186 | 1.004 889 | https://www.genecards .org/cgi- |

| | | | | | | |
|---|---|---|---|---|---|---|
| | | | | | | bin/carddisp.pl?gene=P4HA2-AS1 |
| TGFA-IT1 | TGFA Intronic Transcript 1 | RNA Gene | 13 | GC02M070468 | 1.004889 | https://www.genecards.org/cgi-bin/carddisp.pl?gene=TGFA-IT1 |
| TNFRSF14-AS1 | TNFRSF14 Antisense RNA 1 | RNA Gene | 13 | GC01M006028 | 1.004889 | https://www.genecards.org/cgi-bin/carddisp.pl?gene=TNFRSF14-AS1 |
| WAKMAR2 | Wound And Keratinocyte Migration Associated LncRNA 2 | RNA Gene | 13 | GC06M137820 | 1.004889 | https://www.genecards.org/cgi-bin/carddisp.pl?gene=WAKMAR2 |
| B3GALT9 | Beta-1,3-Galactosyltransferase 9 | Protein Coding | 12 | GC09P120792 | 1.004889 | https://www.genecards.org/cgi-bin/carddisp.pl?gene=B3GALT9 |
| CFAP20DC-AS1 | CFAP20DC Antisense RNA 1 | RNA Gene | 12 | GC03P058825 | 1.004889 | https://www.genecards.org/cgi-bin/carddisp.pl?gene=CFAP20DC-AS1 |
| ENSG00000248751 | Novel Protein | Protein Coding | 12 | GC22M030285 | 1.004889 | https://www.genecards.org/cgi-bin/carddisp.pl?gene=ENSG00000248751 |
| ETS1-AS1 | ETS1 Antisense RNA 1 | RNA Gene | 12 | GC11P128527 | 1.004889 | https://www.genecards.org/cgi-bin/carddisp.pl?gene=ETS1-AS1 |
| FLNC-AS1 | FLNC Antisense RNA 1 | RNA Gene | 12 | GC07M128851 | 1.004889 | https://www.genecards.org/cgi-bin/carddisp.pl?gene=FLNC-AS1 |
| LINC00709 | Long Intergenic Non-Protein Coding RNA 709 | RNA Gene | 12 | GC10P009275 | 1.004889 | https://www.genecards.org/cgi-bin/carddisp.pl?gene=LINC00709 |
| LINC01934 | Long Intergenic Non-Protein Coding RNA 1934 | RNA Gene | 12 | GC02P181086 | 1.004889 | https://www.genecards.org/cgi-bin/carddisp.pl?gene=LINC01934 |
| LINC02098 | Long Intergenic Non-Protein Coding RNA 2098 | RNA Gene | 12 | GC11P128210 | 1.004889 | https://www.genecards.org/cgi-bin/carddisp.pl?gene=LINC02098 |
| LNCRNA-IUR | LncRNA Imatinib Upregulated | RNA Gene | 12 | GC11P095154 | 1.004889 | https://www.genecards.org/cgi-bin/carddisp.pl?gene=LNCRNA-IUR |
| MIR3202-1 | MicroRNA 3202-1 | RNA Gene | 12 | GC0XP153981 | 1.004889 | https://www.genecards.org/cgi-bin/carddisp.pl?gene=MIR3202-1 |

| | | | | | | |
|---|---|---|---|---|---|---|
| SEPT5-GP1BB | SEPT5-GP1BB Readthrough | RNA Gene | 12 | GC22P 019717 | 1.004 889 | https://www.genecards .org/cgi-bin/carddisp.pl?gene= SEPT5-GP1BB |
| SMG1P5 | SMG1 Pseudogene 5 | Pseudo gene | 12 | GC16M 030267 | 1.004 889 | https://www.genecards .org/cgi-bin/carddisp.pl?gene= SMG1P5 |
| TLE1-DT | TLE1 Divergent Transcript | RNA Gene | 12 | GC09P 081690 | 1.004 889 | https://www.genecards .org/cgi-bin/carddisp.pl?gene= TLE1-DT |
| C6orf47-AS1 | C6orf47 Antisense RNA 1 | RNA Gene | 11 | GC06P 083704 | 1.004 889 | https://www.genecards .org/cgi-bin/carddisp.pl?gene= C6orf47-AS1 |
| CD101-AS1 | CD101 Antisense RNA 1 | RNA Gene | 11 | GC01M 117023 | 1.004 889 | https://www.genecards .org/cgi-bin/carddisp.pl?gene= CD101-AS1 |
| CENATA C-DT | CENATAC Divergent Transcript | RNA Gene | 11 | GC11M 118984 | 1.004 889 | https://www.genecards .org/cgi-bin/carddisp.pl?gene= CENATAC-DT |
| LINC013 03 | Long Intergenic Non-Protein Coding RNA 1303 | RNA Gene | 11 | GC14M 061557 | 1.004 889 | https://www.genecards .org/cgi-bin/carddisp.pl?gene= LINC01303 |
| LINC021 53 | Long Intergenic Non-Protein Coding RNA 2153 | RNA Gene | 11 | GC08P 020974 | 1.004 889 | https://www.genecards .org/cgi-bin/carddisp.pl?gene= LINC02153 |
| LINC026 49 | Long Intergenic Non-Protein Coding RNA 2649 | RNA Gene | 11 | GC10P 006357 | 1.004 889 | https://www.genecards .org/cgi-bin/carddisp.pl?gene= LINC02649 |
| LINC027 09 | Long Intergenic Non-Protein Coding RNA 2709 | RNA Gene | 11 | GC11M 009754 | 1.004 889 | https://www.genecards .org/cgi-bin/carddisp.pl?gene= LINC02709 |
| LINC030 04 | Long Intergenic Non-Protein Coding RNA 3004 | RNA Gene | 11 | GC06P 137658 | 1.004 889 | https://www.genecards .org/cgi-bin/carddisp.pl?gene= LINC03004 |
| MIR4328 | MicroRNA 4328 | RNA Gene | 11 | GC0X M0789 01 | 1.004 889 | https://www.genecards .org/cgi-bin/carddisp.pl?gene= MIR4328 |
| RPL13P2 | Ribosomal Protein L13 Pseudogene 2 | Pseudo gene | 11 | GC20P 046099 | 1.004 889 | https://www.genecards .org/cgi-bin/carddisp.pl?gene= RPL13P2 |
| TAGAP-AS1 | TAGAP Antisense RNA 1 | RNA Gene | 11 | GC06P 160653 | 1.004 889 | https://www.genecards .org/cgi- |

| | | | | | | |
|---|---|---|---|---|---|---|
| | | | | | | bin/carddisp.pl?gene=TAGAP-AS1 |
| ADM-DT | ADM Divergent Transcript | RNA Gene | 10 | GC11M010302 | 1.004889 | https://www.genecards.org/cgi-bin/carddisp.pl?gene=ADM-DT |
| CCDC201 | Coiled-Coil Domain Containing 201 | Protein Coding | 10 | GC07M045859 | 1.004889 | https://www.genecards.org/cgi-bin/carddisp.pl?gene=CCDC201 |
| CUTALP | CutA Divalent Cation Tolerance Like, Pseudogene | Pseudogene | 10 | GC09P120826 | 1.004889 | https://www.genecards.org/cgi-bin/carddisp.pl?gene=CUTALP |
| EMSLR | E2F1 MRNA Stabilizing LncRNA | RNA Gene | 10 | GC07P101335 | 1.004889 | https://www.genecards.org/cgi-bin/carddisp.pl?gene=EMSLR |
| ENSG00000167807 | Novel Transcript | Protein Coding | 10 | GC19M010400 | 1.004889 | https://www.genecards.org/cgi-bin/carddisp.pl?gene=ENSG00000167807 |
| ENSG00000228778 | Novel Transcript | RNA Gene | 10 | GC10P099527 | 1.004889 | https://www.genecards.org/cgi-bin/carddisp.pl?gene=ENSG00000228778 |
| ENSG00000249624 | Novel Protein | Protein Coding | 10 | GC21P033246 | 1.004889 | https://www.genecards.org/cgi-bin/carddisp.pl?gene=ENSG00000249624 |
| LINC01343 | Long Intergenic Non-Protein Coding RNA 1343 | RNA Gene | 10 | GC01M038209 | 1.004889 | https://www.genecards.org/cgi-bin/carddisp.pl?gene=LINC01343 |
| LINC01898 | Long Intergenic Non-Protein Coding RNA 1898 | RNA Gene | 10 | GC18M075697 | 1.004889 | https://www.genecards.org/cgi-bin/carddisp.pl?gene=LINC01898 |
| LINC01980 | Long Intergenic Non-Protein Coding RNA 1980 | RNA Gene | 10 | GC03P027798 | 1.004889 | https://www.genecards.org/cgi-bin/carddisp.pl?gene=LINC01980 |
| LINC02528 | Long Intergenic Non-Protein Coding RNA 2528 | RNA Gene | 10 | GC06M137945 | 1.004889 | https://www.genecards.org/cgi-bin/carddisp.pl?gene=LINC02528 |
| LINC02539 | Long Intergenic Non-Protein Coding RNA 2539 | RNA Gene | 10 | GC06M137731 | 1.004889 | https://www.genecards.org/cgi-bin/carddisp.pl?gene=LINC02539 |
| LINC02664 | Long Intergenic Non-Protein Coding RNA 2664 | RNA Gene | 10 | GC10P031190 | 1.004889 | https://www.genecards.org/cgi-bin/carddisp.pl?gene=LINC02664 |

| | | | | | | |
|---|---|---|---|---|---|---|
| LINC02757 | Long Intergenic Non-Protein Coding RNA 2757 | RNA Gene | 10 | GC11M090455 | 1.004889 | https://www.genecards.org/cgi-bin/carddisp.pl?gene=LINC02757 |
| LINC02789 | Long Intergenic Non-Protein Coding RNA 2789 | RNA Gene | 10 | GC01P199149 | 1.004889 | https://www.genecards.org/cgi-bin/carddisp.pl?gene=LINC02789 |
| LNCPRESS1 | LncRNA P53 Regulated And ESC Associated 1 | RNA Gene | 10 | GC07P101304 | 1.004889 | https://www.genecards.org/cgi-bin/carddisp.pl?gene=LNCPRESS1 |
| LOC100996583 | Uncharacterized LOC100996583 | RNA Gene | 10 | GC01P003937 | 1.004889 | https://www.genecards.org/cgi-bin/carddisp.pl?gene=LOC100996583 |
| LOC101929290 | Uncharacterized LOC101929290 | RNA Gene | 10 | GC08M011315 | 1.004889 | https://www.genecards.org/cgi-bin/carddisp.pl?gene=LOC101929290 |
| MIR4308 | MicroRNA 4308 | RNA Gene | 10 | GC14M054878 | 1.004889 | https://www.genecards.org/cgi-bin/carddisp.pl?gene=MIR4308 |
| PCNPP1 | PEST Containing Nuclear Protein Pseudogene 1 | Pseudogene | 10 | GC12M111666 | 1.004889 | https://www.genecards.org/cgi-bin/carddisp.pl?gene=PCNPP1 |
| PLCL2-AS1 | PLCL2 Antisense RNA 1 | RNA Gene | 10 | GC03M017042 | 1.004889 | https://www.genecards.org/cgi-bin/carddisp.pl?gene=PLCL2-AS1 |
| PRDX6-AS1 | PRDX6 Antisense RNA 1 | RNA Gene | 10 | GC01M173418 | 1.004889 | https://www.genecards.org/cgi-bin/carddisp.pl?gene=PRDX6-AS1 |
| RPL7AP14 | Ribosomal Protein L7a Pseudogene 14 | Pseudogene | 10 | GC20M037444 | 1.004889 | https://www.genecards.org/cgi-bin/carddisp.pl?gene=RPL7AP14 |
| SPATA31D5P | SPATA31 Subfamily D Member 5, Pseudogene | Pseudogene | 10 | GC09P081913 | 1.004889 | https://www.genecards.org/cgi-bin/carddisp.pl?gene=SPATA31D5P |
| TDH-AS1 | TDH Antisense RNA 1 | RNA Gene | 10 | GC08M011181 | 1.004889 | https://www.genecards.org/cgi-bin/carddisp.pl?gene=TDH-AS1 |
| TSPAN14-AS1 | TSPAN14 Antisense RNA 1 | RNA Gene | 10 | GC10M082037 | 1.004889 | https://www.genecards.org/cgi-bin/carddisp.pl?gene=TSPAN14-AS1 |
| ZNF232-AS1 | ZNF232 Antisense RNA 1 | RNA Gene | 10 | GC17P005518 | 1.004889 | https://www.genecards.org/cgi- |

| | | | | | | |
|---|---|---|---|---|---|---|
| | | | | | | bin/carddisp.pl?gene=ZNF232-AS1 |
| ZNF747-DT | ZNF747 Divergent Transcript | RNA Gene | 10 | GC16P041647 | 1.004889 | https://www.genecards.org/cgi-bin/carddisp.pl?gene=ZNF747-DT |
| CALM2P2 | Calmodulin 2 Pseudogene 2 | Pseudogene | 9 | GC10P070163 | 1.004889 | https://www.genecards.org/cgi-bin/carddisp.pl?gene=CALM2P2 |
| EBAG9P1 | EBAG9 Pseudogene 1 | Pseudogene | 9 | GC10M099697 | 1.004889 | https://www.genecards.org/cgi-bin/carddisp.pl?gene=EBAG9P1 |
| ENSG00000204929 | Novel Transcript | Uncategorized | 9 | GC02P065451 | 1.004889 | https://www.genecards.org/cgi-bin/carddisp.pl?gene=ENSG00000204929 |
| ENSG00000217702 | Novel Transcript, Antisense To BOLA3 | RNA Gene | 9 | GC02P074130 | 1.004889 | https://www.genecards.org/cgi-bin/carddisp.pl?gene=ENSG00000217702 |
| ENSG00000224015 | Novel Transcript | RNA Gene | 9 | GC05M132062 | 1.004889 | https://www.genecards.org/cgi-bin/carddisp.pl?gene=ENSG00000224015 |
| ENSG00000228005 | Novel Transcript | RNA Gene | 9 | GC07M050181 | 1.004889 | https://www.genecards.org/cgi-bin/carddisp.pl?gene=ENSG00000228005 |
| ENSG00000228509 | Novel Transcript, Antisense To NAB1 | RNA Gene | 9 | GC02M190672 | 1.004889 | https://www.genecards.org/cgi-bin/carddisp.pl?gene=ENSG00000228509 |
| ENSG00000228863 | Novel Transcript, Antisense To CD48 | RNA Gene | 9 | GC01P160671 | 1.004889 | https://www.genecards.org/cgi-bin/carddisp.pl?gene=ENSG00000228863 |
| ENSG00000231128 | Novel Transcript, Antisense To PTPN22 | RNA Gene | 9 | GC01P113812 | 1.004889 | https://www.genecards.org/cgi-bin/carddisp.pl?gene=ENSG00000231128 |
| ENSG00000231355 | Novel Transcript | RNA Gene | 9 | GC21M033482 | 1.004889 | https://www.genecards.org/cgi-bin/carddisp.pl?gene=ENSG00000231355 |
| ENSG00000231467 | Novel Transcript | RNA Gene | 9 | GC22P037080 | 1.004889 | https://www.genecards.org/cgi-bin/carddisp.pl?gene=ENSG00000231467 |
| ENSG00000232638 | Novel Transcript | RNA Gene | 9 | GC10P008051 | 1.004889 | https://www.genecards.org/cgi-bin/carddisp.pl?gene=ENSG00000232638 |

| | | | | | | |
|---|---|---|---|---|---|---|
| ENSG00000234132 | Novel Transcript, Antisense CACNA1S | RNA Gene | 9 | GC01P201032 | 1.004889 | https://www.genecards.org/cgi-bin/carddisp.pl?gene=ENSG00000234132 |
| ENSG00000234255 | Novel Transcript | RNA Gene | 9 | GC02M065442 | 1.004889 | https://www.genecards.org/cgi-bin/carddisp.pl?gene=ENSG00000234255 |
| ENSG00000236935 | Novel Transcript | RNA Gene | 9 | GC11M089710 | 1.004889 | https://www.genecards.org/cgi-bin/carddisp.pl?gene=ENSG00000236935 |
| ENSG00000249141 | Novel Protein | Protein Coding | 9 | GC06M166858 | 1.004889 | https://www.genecards.org/cgi-bin/carddisp.pl?gene=ENSG00000249141 |
| ENSG00000253720 | Novel Transcript | RNA Gene | 9 | GC08P129939 | 1.004889 | https://www.genecards.org/cgi-bin/carddisp.pl?gene=ENSG00000253720 |
| ENSG00000255422 | Novel Transcript | RNA Gene | 9 | GC11P118700 | 1.004889 | https://www.genecards.org/cgi-bin/carddisp.pl?gene=ENSG00000255422 |
| ENSG00000258989 | Novel Protein | Protein Coding | 9 | GC14P061530 | 1.004889 | https://www.genecards.org/cgi-bin/carddisp.pl?gene=ENSG00000258989 |
| ENSG00000260267 | Novel Transcript, Antisense To ARMC5 | RNA Gene | 9 | GC16M031456 | 1.004889 | https://www.genecards.org/cgi-bin/carddisp.pl?gene=ENSG00000260267 |
| ENSG00000269918 | Novel Transcript | RNA Gene | 9 | GC08M011104 | 1.004889 | https://www.genecards.org/cgi-bin/carddisp.pl?gene=ENSG00000269918 |
| ENSG00000269954 | Novel Transcript, Antisense To BLK | RNA Gene | 9 | GC08M011552 | 1.004889 | https://www.genecards.org/cgi-bin/carddisp.pl?gene=ENSG00000269954 |
| LINC02084 | Long Intergenic Non-Protein Coding RNA 2084 | RNA Gene | 9 | GC03P027714 | 1.004889 | https://www.genecards.org/cgi-bin/carddisp.pl?gene=LINC02084 |
| LINC02132 | Long Intergenic Non-Protein Coding RNA 2132 | RNA Gene | 9 | GC16M085935 | 1.004889 | https://www.genecards.org/cgi-bin/carddisp.pl?gene=LINC02132 |
| LINC02356 | Long Intergenic Non-Protein Coding RNA 2356 | RNA Gene | 9 | GC12P111371 | 1.004889 | https://www.genecards.org/cgi-bin/carddisp.pl?gene=LINC02356 |
| LINC02357 | Long Intergenic Non-Protein Coding RNA 2357 | RNA Gene | 9 | GC04P026071 | 1.004889 | https://www.genecards.org/cgi- |

| | | | | | | |
|---|---|---|---|---|---|---|
| | | | | | | bin/carddisp.pl?gene=LINC02357 |
| LINC02676 | Long Intergenic Non-Protein Coding RNA 2676 | RNA Gene | 9 | GC10P008726 | 1.004889 | https://www.genecards.org/cgi-bin/carddisp.pl?gene=LINC02676 |
| LINC02723 | Long Intergenic Non-Protein Coding RNA 2723 | RNA Gene | 9 | GC11M089711 | 1.004889 | https://www.genecards.org/cgi-bin/carddisp.pl?gene=LINC02723 |
| LOC105371083 | Uncharacterized LOC105371083 | RNA Gene | 9 | GC16M012272 | 1.004889 | https://www.genecards.org/cgi-bin/carddisp.pl?gene=LOC105371083 |
| LOC112267968 | Uncharacterized LOC112267968 | Protein Coding | 9 | GC06M159053 | 1.004889 | https://www.genecards.org/cgi-bin/carddisp.pl?gene=LOC112267968 |
| LOC124903255 | Small Nucleolar RNA SNORD116 | RNA Gene | 9 | GC13M039869 | 1.004889 | https://www.genecards.org/cgi-bin/carddisp.pl?gene=LOC124903255 |
| NEFHP1 | Neurofilament Heavy Pseudogene 1 | Pseudogene | 9 | GC01M116739 | 1.004889 | https://www.genecards.org/cgi-bin/carddisp.pl?gene=NEFHP1 |
| PPIAP6 | Peptidylprolyl Isomerase A Pseudogene 6 | Pseudogene | 9 | GC14M068421 | 1.004889 | https://www.genecards.org/cgi-bin/carddisp.pl?gene=PPIAP6 |
| RNU6-474P | RNA, U6 Small Nuclear 474, Pseudogene | Pseudogene | 9 | GC02P203782 | 1.004889 | https://www.genecards.org/cgi-bin/carddisp.pl?gene=RNU6-474P |
| RNU6-933P | RNA, U6 Small Nuclear 933, Pseudogene | Pseudogene | 9 | GC11M060985 | 1.004889 | https://www.genecards.org/cgi-bin/carddisp.pl?gene=RNU6-933P |
| RNU6-959P | RNA, U6 Small Nuclear 959, Pseudogene | Pseudogene | 9 | GC02P191121 | 1.004889 | https://www.genecards.org/cgi-bin/carddisp.pl?gene=RNU6-959P |
| RPL21P97 | Ribosomal Protein L21 Pseudogene 97 | Pseudogene | 9 | GC11P009694 | 1.004889 | https://www.genecards.org/cgi-bin/carddisp.pl?gene=RPL21P97 |
| RPL32P23 | Ribosomal Protein L32 Pseudogene 23 | Pseudogene | 9 | GC10M006072 | 1.004889 | https://www.genecards.org/cgi-bin/carddisp.pl?gene=RPL32P23 |
| RPL7AP70 | Ribosomal Protein L7a Pseudogene 70 | Pseudogene | 9 | GC22P035817 | 1.004889 | https://www.genecards.org/cgi-bin/carddisp.pl?gene=RPL7AP70 |

| | | | | | | |
|---|---|---|---|---|---|---|
| RPL8P5 | Ribosomal Protein L8 Pseudogene 5 | Pseudo gene | 9 | GC22P 035819 | 1.004 889 | https://www.genecards.org/cgi-bin/carddisp.pl?gene=RPL8P5 |
| RPS2P29 | Ribosomal Protein S2 Pseudogene 29 | Pseudo gene | 9 | GC06M 066211 | 1.004 889 | https://www.genecards.org/cgi-bin/carddisp.pl?gene=RPS2P29 |
| RPS5P3 | RPS5 Pseudogene 3 | Pseudo gene | 9 | GC21M 033481 | 1.004 889 | https://www.genecards.org/cgi-bin/carddisp.pl?gene=RPS5P3 |
| RTEL1P1 | Regulator Of Telomere Elongation Helicase 1 Pseudogene 1 | Pseudo gene | 9 | GC04P 112355 | 1.004 889 | https://www.genecards.org/cgi-bin/carddisp.pl?gene=RTEL1P1 |
| ALG1L11 P | ALG1 Like 11, Pseudogene | Pseudo gene | 8 | GC08P 012178 | 1.004 889 | https://www.genecards.org/cgi-bin/carddisp.pl?gene=ALG1L11P |
| DUTP6 | Deoxyuridine Triphosphatase Pseudogene 6 | Pseudo gene | 8 | GC01P 166838 | 1.004 889 | https://www.genecards.org/cgi-bin/carddisp.pl?gene=DUTP6 |
| ENSG000 00205537 | Novel Transcript, Antisense To VDR | RNA Gene | 8 | GC12P 047883 | 1.004 889 | https://www.genecards.org/cgi-bin/carddisp.pl?gene=ENSG00000205537 |
| ENSG000 00222529 | Y RNA | RNA Gene | 8 | GC11P 118970 | 1.004 889 | https://www.genecards.org/cgi-bin/carddisp.pl?gene=ENSG00000222529 |
| ENSG000 00223808 | Novel Transcript | RNA Gene | 8 | GC10P 008943 | 1.004 889 | https://www.genecards.org/cgi-bin/carddisp.pl?gene=ENSG00000223808 |
| ENSG000 00224478 | Novel Transcript | RNA Gene | 8 | GC06P 160537 | 1.004 889 | https://www.genecards.org/cgi-bin/carddisp.pl?gene=ENSG00000224478 |
| ENSG000 00227217 | Novel Transcript | RNA Gene | 8 | GC01P 157527 | 1.004 889 | https://www.genecards.org/cgi-bin/carddisp.pl?gene=ENSG00000227217 |
| ENSG000 00230686 | Novel Transcript | RNA Gene | 8 | GC02P 191017 | 1.004 889 | https://www.genecards.org/cgi-bin/carddisp.pl?gene=ENSG00000230686 |
| ENSG000 00234211 | Novel Transcript, Antisense To FCGR2B | RNA Gene | 8 | GC01M 161671 | 1.004 889 | https://www.genecards.org/cgi-bin/carddisp.pl?gene=ENSG00000234211 |
| ENSG000 00235237 | Novel Transcript | RNA Gene | 8 | GC22P 037166 | 1.004 889 | https://www.genecards.org/cgi- |

| | | | | | | |
|---|---|---|---|---|---|---|
| | | | | | | bin/carddisp.pl?gene=ENSG00000235237 |
| ENSG00000235499 | Novel Transcript (LOC100132318) | RNA Gene | 8 | GC02P073985 | 1.004889 | https://www.genecards.org/cgi-bin/carddisp.pl?gene=ENSG00000235499 |
| ENSG00000236308 | Novel Transcript, Antisense To CHUK | RNA Gene | 8 | GC10P100190 | 1.004889 | https://www.genecards.org/cgi-bin/carddisp.pl?gene=ENSG00000236308 |
| ENSG00000238280 | Novel Transcript | RNA Gene | 8 | GC10M062682 | 1.004889 | https://www.genecards.org/cgi-bin/carddisp.pl?gene=ENSG00000238280 |
| ENSG00000239791 | Novel Transcript | RNA Gene | 8 | GC16P041084 | 1.004889 | https://www.genecards.org/cgi-bin/carddisp.pl?gene=ENSG00000239791 |
| ENSG00000254084 | Novel Transcript | RNA Gene | 8 | GC08M101411 | 1.004889 | https://www.genecards.org/cgi-bin/carddisp.pl?gene=ENSG00000254084 |
| ENSG00000254397 | Novel Transcript | RNA Gene | 8 | GC11M009431 | 1.004889 | https://www.genecards.org/cgi-bin/carddisp.pl?gene=ENSG00000254397 |
| ENSG00000255176 | Novel Transcript | RNA Gene | 8 | GC11P118636 | 1.004889 | https://www.genecards.org/cgi-bin/carddisp.pl?gene=ENSG00000255176 |
| ENSG00000257985 | Novel Transcript, Antisense To A Novel Protein | RNA Gene | 8 | GC12P048011 | 1.004889 | https://www.genecards.org/cgi-bin/carddisp.pl?gene=ENSG00000257985 |
| ENSG00000259598 | Novel Transcript, Antisense To RASGRP1 | RNA Gene | 8 | GC15P038524 | 1.004889 | https://www.genecards.org/cgi-bin/carddisp.pl?gene=ENSG00000259598 |
| ENSG00000259840 | Novel Transcript | RNA Gene | 8 | GC16M007030 | 1.004889 | https://www.genecards.org/cgi-bin/carddisp.pl?gene=ENSG00000259840 |
| ENSG00000260304 | Novel Transcript, Antisense To FUS | RNA Gene | 8 | GC16M031180 | 1.004889 | https://www.genecards.org/cgi-bin/carddisp.pl?gene=ENSG00000260304 |
| ENSG00000260482 | Novel Transcript | RNA Gene | 8 | GC16P023641 | 1.004889 | https://www.genecards.org/cgi-bin/carddisp.pl?gene=ENSG00000260482 |
| ENSG00000262420 | Novel Transcript, Antisense To TXNDC11 | RNA Gene | 8 | GC16P011741 | 1.004889 | https://www.genecards.org/cgi-bin/carddisp.pl?gene=ENSG00000262420 |

| | | | | | | |
|---|---|---|---|---|---|---|
| ENSG00000263220 | Novel Transcript, Antisense To RABEP1 | RNA Gene | 8 | GC17M005364 | 1.004889 | https://www.genecards.org/cgi-bin/carddisp.pl?gene=ENSG00000263220 |
| ENSG00000267303 | Novel Transcript | Protein Coding | 8 | GC19M010315 | 1.004889 | https://www.genecards.org/cgi-bin/carddisp.pl?gene=ENSG00000267303 |
| ENSG00000269514 | Novel Transcript, Antisense To OR10AD1 | RNA Gene | 8 | GC12P048198 | 1.004889 | https://www.genecards.org/cgi-bin/carddisp.pl?gene=ENSG00000269514 |
| ENSG00000269667 | Novel Transcript | RNA Gene | 8 | GC16P085990 | 1.004889 | https://www.genecards.org/cgi-bin/carddisp.pl?gene=ENSG00000269667 |
| ENSG00000270120 | Novel Transcript | RNA Gene | 8 | GC16P050712 | 1.004889 | https://www.genecards.org/cgi-bin/carddisp.pl?gene=ENSG00000270120 |
| ENSG00000271553 | Novel Transcript | RNA Gene | 8 | GC07P128667 | 1.004889 | https://www.genecards.org/cgi-bin/carddisp.pl?gene=ENSG00000271553 |
| ETV7-AS1 | ETV7 And PTX1 Antisense RNA 1 | RNA Gene | 8 | GC06P084401 | 1.004889 | https://www.genecards.org/cgi-bin/carddisp.pl?gene=ETV7-AS1 |
| GAPDHP64 | Glyceraldehyde-3-Phosphate Dehydrogenase Pseudogene 64 | Pseudogene | 8 | GC01M116713 | 1.004889 | https://www.genecards.org/cgi-bin/carddisp.pl?gene=GAPDHP64 |
| GLULP4 | Glutamate-Ammonia Ligase Pseudogene 4 | Pseudogene | 8 | GC09P034917 | 1.004889 | https://www.genecards.org/cgi-bin/carddisp.pl?gene=GLULP4 |
| LINC02498 | Long Intergenic Non-Protein Coding RNA 2498 | RNA Gene | 8 | GC04P018282 | 1.004889 | https://www.genecards.org/cgi-bin/carddisp.pl?gene=LINC02498 |
| LINC02621 | Long Intergenic Non-Protein Coding RNA 2621 | RNA Gene | 8 | GC10P062302 | 1.004889 | https://www.genecards.org/cgi-bin/carddisp.pl?gene=LINC02621 |
| LINC02865 | Long Intergenic Non-Protein Coding RNA 2865 | RNA Gene | 8 | GC06P137946 | 1.004889 | https://www.genecards.org/cgi-bin/carddisp.pl?gene=LINC02865 |
| LINC02943 | Long Intergenic Non-Protein Coding RNA 2943 | RNA Gene | 8 | GC21M039034 | 1.004889 | https://www.genecards.org/cgi-bin/carddisp.pl?gene=LINC02943 |
| LOC101928272 | Uncharacterized LOC101928272 | RNA Gene | 8 | GC10M008849 | 1.004889 | https://www.genecards.org/cgi- |

| | | | | | | |
|---|---|---|---|---|---|---|
| | | | | | | bin/carddisp.pl?gene=LOC101928272 |
| LOC124902827 | Small Nucleolar RNA U13 | RNA Gene | 8 | GC11P009588 | 1.004889 | https://www.genecards.org/cgi-bin/carddisp.pl?gene=LOC124902827 |
| PHB1P9 | PHB1 Pseudogene 9 | Pseudogene | 8 | GC10P100251 | 1.004889 | https://www.genecards.org/cgi-bin/carddisp.pl?gene=PHB1P9 |
| RN7SL38P | RNA, 7SL, Cytoplasmic 38, Pseudogene | Pseudogene | 8 | GC12P024706 | 1.004889 | https://www.genecards.org/cgi-bin/carddisp.pl?gene=RN7SL38P |
| RN7SL688P | RNA, 7SL, Cytoplasmic 688, Pseudogene | Pseudogene | 8 | GC11P118931 | 1.004889 | https://www.genecards.org/cgi-bin/carddisp.pl?gene=RN7SL688P |
| RNU4ATAC4P | RNA, U4atac Small Nuclear 4, Pseudogene | Pseudogene | 8 | GC01M067267 | 1.004889 | https://www.genecards.org/cgi-bin/carddisp.pl?gene=RNU4ATAC4P |
| RNU6-1035P | RNA, U6 Small Nuclear 1035, Pseudogene | Pseudogene | 8 | GC09M081725 | 1.004889 | https://www.genecards.org/cgi-bin/carddisp.pl?gene=RNU6-1035P |
| RNU6-1149P | RNA, U6 Small Nuclear 1149, Pseudogene | Pseudogene | 8 | GC21M042423 | 1.004889 | https://www.genecards.org/cgi-bin/carddisp.pl?gene=RNU6-1149P |
| RNU6-387P | RNA, U6 Small Nuclear 387, Pseudogene | Pseudogene | 8 | GC01P067417 | 1.004889 | https://www.genecards.org/cgi-bin/carddisp.pl?gene=RNU6-387P |
| RNU6-481P | RNA, U6 Small Nuclear 481, Pseudogene | Pseudogene | 8 | GC01P161401 | 1.004889 | https://www.genecards.org/cgi-bin/carddisp.pl?gene=RNU6-481P |
| RNU6-704P | RNA, U6 Small Nuclear 704, Pseudogene | Pseudogene | 8 | GC01M200904 | 1.004889 | https://www.genecards.org/cgi-bin/carddisp.pl?gene=RNU6-704P |
| RNU6-979P | RNA, U6 Small Nuclear 979, Pseudogene | Pseudogene | 8 | GC07M028119 | 1.004889 | https://www.genecards.org/cgi-bin/carddisp.pl?gene=RNU6-979P |
| RNY4P14 | RNY4 Pseudogene 14 | Pseudogene | 8 | GC13M039785 | 1.004889 | https://www.genecards.org/cgi-bin/carddisp.pl?gene=RNY4P14 |
| UQCRC2P1 | Ubiquinol-Cytochrome C Reductase Core Protein 2 Pseudogene 1 | Pseudogene | 8 | GC03M046311 | 1.004889 | https://www.genecards.org/cgi-bin/carddisp.pl?gene=UQCRC2P1 |

| | | | | | | |
|---|---|---|---|---|---|---|
| ENSG00000200998 | Y RNA | RNA Gene | 7 | GC04M078632 | 1.004889 | https://www.genecards.org/cgi-bin/carddisp.pl?gene=ENSG00000200998 |
| ENSG00000226032 | Novel Transcript | RNA Gene | 7 | GC06M159042 | 1.004889 | https://www.genecards.org/cgi-bin/carddisp.pl?gene=ENSG00000226032 |
| ENSG00000232693 | Novel Transcript, Sense Intronic To SPRED2 | RNA Gene | 7 | GC02M065373 | 1.004889 | https://www.genecards.org/cgi-bin/carddisp.pl?gene=ENSG00000232693 |
| ENSG00000245008 | Novel Transcript, Antisense To FLI1 | Uncategorized | 7 | GC11M128616 | 1.004889 | https://www.genecards.org/cgi-bin/carddisp.pl?gene=ENSG00000245008 |
| ENSG00000254024 | Novel Transcript | RNA Gene | 7 | GC08M101461 | 1.004889 | https://www.genecards.org/cgi-bin/carddisp.pl?gene=ENSG00000254024 |
| ENSG00000257955 | Novel Transcript | RNA Gene | 7 | GC12M048021 | 1.004889 | https://www.genecards.org/cgi-bin/carddisp.pl?gene=ENSG00000257955 |
| ENSG00000267287 | Novel Transcript | Uncategorized | 7 | GC18P079577 | 1.004889 | https://www.genecards.org/cgi-bin/carddisp.pl?gene=ENSG00000267287 |
| ENSG00000285163 | Novel Transcript | RNA Gene | 7 | GC16P085924 | 1.004889 | https://www.genecards.org/cgi-bin/carddisp.pl?gene=ENSG00000285163 |
| ENSG00000287564 | Novel Transcript | RNA Gene | 7 | GC10P031057 | 1.004889 | https://www.genecards.org/cgi-bin/carddisp.pl?gene=ENSG00000287564 |
| LDHAP3 | Lactate Dehydrogenase A Pseudogene 3 | Pseudogene | 7 | GC02P041819 | 1.004889 | https://www.genecards.org/cgi-bin/carddisp.pl?gene=LDHAP3 |
| LINC02786 | Long Intergenic Non-Protein Coding RNA 2786 | RNA Gene | 7 | GC01M038132 | 1.004889 | https://www.genecards.org/cgi-bin/carddisp.pl?gene=LINC02786 |
| LOC101927180 | V-Type Proton ATPase Subunit F-Like | Protein Coding | 7 | GC12P048084 | 1.004889 | https://www.genecards.org/cgi-bin/carddisp.pl?gene=LOC101927180 |
| RN7SL563P | RNA, 7SL, Cytoplasmic 563, Pseudogene | Pseudogene | 7 | GC08M101397 | 1.004889 | https://www.genecards.org/cgi-bin/carddisp.pl?gene=RN7SL563P |
| RNU6-376P | RNA, U6 Small Nuclear 376, Pseudogene | Pseudogene | 7 | GC11P118702 | 1.004889 | https://www.genecards.org/cgi- |

| | | | | | | |
|---|---|---|---|---|---|---|
| | | | | | | bin/carddisp.pl?gene=RNU6-376P |
| RNU7-133P | RNA, U7 Small Nuclear 133 Pseudogene | Pseudo gene | 7 | GC06P 014166 | 1.004 889 | https://www.genecards.org/cgi-bin/carddisp.pl?gene=RNU7-133P |
| RPL6P8 | Ribosomal Protein L6 Pseudogene 8 | Pseudo gene | 7 | GC03P 160579 | 1.004 889 | https://www.genecards.org/cgi-bin/carddisp.pl?gene=RPL6P8 |
| SETP16 | SET Pseudogene 16 | Pseudo gene | 7 | GC11M 118833 | 1.004 889 | https://www.genecards.org/cgi-bin/carddisp.pl?gene=SETP16 |
| lnc-LEF1-1 | | RNA Gene | 7 | GC04M 108048 | 1.004 889 | https://www.genecards.org/cgi-bin/carddisp.pl?gene=lnc-LEF1-1 |
| BTF3L4P3 | Basic Transcription Factor 3 Like 4 Pseudogene 3 | Pseudo gene | 6 | GC06M 137543 | 1.004 889 | https://www.genecards.org/cgi-bin/carddisp.pl?gene=BTF3L4P3 |
| CDKN2AIPNLP3 | CDKN2A Interacting Protein N-Terminal Like Pseudogene 3 | Pseudo gene | 6 | GC13M 039817 | 1.004 889 | https://www.genecards.org/cgi-bin/carddisp.pl?gene=CDKN2AIPNLP3 |
| ENSG00000225931 | Novel Transcript | Uncategorized | 6 | GC01P 002566 | 1.004 889 | https://www.genecards.org/cgi-bin/carddisp.pl?gene=ENSG00000225931 |
| ENSG00000242162 | Pseudogene Similar To Part Of Sec61 Alpha 1 Subunit (S. Cerevisiae) (SEC61A1) | Pseudo gene | 6 | GC07M 129066 | 1.004 889 | https://www.genecards.org/cgi-bin/carddisp.pl?gene=ENSG00000242162 |
| ENSG00000245869 | Novel Transcript, Antisense To CXCR5 | RNA Gene | 6 | GC11M 118887 | 1.004 889 | https://www.genecards.org/cgi-bin/carddisp.pl?gene=ENSG00000245869 |
| ENSG00000253067 | | RNA Gene | 6 | GC05P 132080 | 1.004 889 | https://www.genecards.org/cgi-bin/carddisp.pl?gene=ENSG00000253067 |
| ENSG00000273245 | Novel Transcript | RNA Gene | 6 | GC02M 073750 | 1.004 889 | https://www.genecards.org/cgi-bin/carddisp.pl?gene=ENSG00000273245 |
| ENSG00000273300 | Novel Transcript, Antisense To UFD1L | RNA Gene | 6 | GC22P 019454 | 1.004 889 | https://www.genecards.org/cgi-bin/carddisp.pl?gene=ENSG00000273300 |
| ENSG00000273342 | Novel Transcript | RNA Gene | 6 | GC22P 035925 | 1.004 889 | https://www.genecards.org/cgi-bin/carddisp.pl?gene=ENSG00000273342 |

| | | | | | | |
|---|---|---|---|---|---|---|
| ENSG00000284825 | Novel Transcript | RNA Gene | 6 | GC06M167116 | 1.004889 | https://www.genecards.org/cgi-bin/carddisp.pl?gene=ENSG00000284825 |
| ENSG00000284957 | Novel Transcript | RNA Gene | 6 | GC08M011479 | 1.004889 | https://www.genecards.org/cgi-bin/carddisp.pl?gene=ENSG00000284957 |
| ENSG00000285040 | Novel Transcript | RNA Gene | 6 | GC16M085963 | 1.004889 | https://www.genecards.org/cgi-bin/carddisp.pl?gene=ENSG00000285040 |
| ENSG00000288583 | Novel Transcript | RNA Gene | 6 | GC09P047456 | 1.004889 | https://www.genecards.org/cgi-bin/carddisp.pl?gene=ENSG00000288583 |
| ENSG00000288749 | Novel Transcript | RNA Gene | 6 | GC07P101291 | 1.004889 | https://www.genecards.org/cgi-bin/carddisp.pl?gene=ENSG00000288749 |
| ENSG00000288915 | Novel Transcript, Antisense To PRKCQ | RNA Gene | 6 | GC10P006493 | 1.004889 | https://www.genecards.org/cgi-bin/carddisp.pl?gene=ENSG00000288915 |
| ENSG00000289398 | Novel Transcript | RNA Gene | 6 | GC11P128313 | 1.004889 | https://www.genecards.org/cgi-bin/carddisp.pl?gene=ENSG00000289398 |
| ENSG00000289525 | Novel Transcript | RNA Gene | 6 | GC15M090911 | 1.004889 | https://www.genecards.org/cgi-bin/carddisp.pl?gene=ENSG00000289525 |
| H3P5 | H3 Histone Pseudogene 5 | Pseudogene | 6 | GC02P030211 | 1.004889 | https://www.genecards.org/cgi-bin/carddisp.pl?gene=H3P5 |
| HSALNG0036266 | | RNA Gene | 6 | GC04P108027 | 1.004889 | https://www.genecards.org/cgi-bin/carddisp.pl?gene=HSALNG0036266 |
| HSALNG0057943 | | RNA Gene | 6 | GC07M050313 | 1.004889 | https://www.genecards.org/cgi-bin/carddisp.pl?gene=HSALNG0057943 |
| KNOP1P1 | Lysine Rich Nucleolar Protein 1 Pseudogene 1 | Pseudogene | 6 | GC12P024583 | 1.004889 | https://www.genecards.org/cgi-bin/carddisp.pl?gene=KNOP1P1 |
| LOC124900752 | Uncharacterized LOC124900752 | Protein Coding | 6 | GC04P108168 | 1.004889 | https://www.genecards.org/cgi-bin/carddisp.pl?gene=LOC124900752 |
| LOC124901411 | Uncharacterized LOC124901411 | RNA Gene | 6 | GC06P137695 | 1.004889 | https://www.genecards.org/cgi- |

| | | | | | | |
|---|---|---|---|---|---|---|
| | | | | | | bin/carddisp.pl?gene=LOC124901411 |
| LOC124904333 | Octapeptide-Repeat Protein T2-Like | Protein Coding | 6 | GC18P079419 | 1.004889 | https://www.genecards.org/cgi-bin/carddisp.pl?gene=LOC124904333 |
| NONHSAG005220.2 | | RNA Gene | 6 | GC10P011006 | 1.004889 | https://www.genecards.org/cgi-bin/carddisp.pl?gene=NONHSAG005220.2 |
| NPM1P33 | Nucleophosmin 1 Pseudogene 33 | Pseudogene | 6 | GC02M203772 | 1.004889 | https://www.genecards.org/cgi-bin/carddisp.pl?gene=NPM1P33 |
| P2RY10BP | P2Y Receptor Family Member 10B, Pseudogene | Pseudogene | 6 | GC0XP079085 | 1.004889 | https://www.genecards.org/cgi-bin/carddisp.pl?gene=P2RY10BP |
| POLR3GP2 | RNA Polymerase III Subunit G Pseudogene 2 | Pseudogene | 6 | GC18P048478 | 1.004889 | https://www.genecards.org/cgi-bin/carddisp.pl?gene=POLR3GP2 |
| RF00017-7224 | | RNA Gene | 6 | GC09M094967 | 1.004889 | https://www.genecards.org/cgi-bin/carddisp.pl?gene=RF00017-7224 |
| RN7SL335P | RNA, 7SL, Cytoplasmic 335, Pseudogene | Pseudogene | 6 | GC04M122087 | 1.004889 | https://www.genecards.org/cgi-bin/carddisp.pl?gene=RN7SL335P |
| RNA5SP287 | RNA, 5S Ribosomal Pseudogene 287 | Pseudogene | 6 | GC09P081885 | 1.004889 | https://www.genecards.org/cgi-bin/carddisp.pl?gene=RNA5SP287 |
| RNU1-61P | RNA, U1 Small Nuclear 61, Pseudogene | Pseudogene | 6 | GC06M032549 | 1.004889 | https://www.genecards.org/cgi-bin/carddisp.pl?gene=RNU1-61P |
| RNU6-420P | RNA, U6 Small Nuclear 420, Pseudogene | Pseudogene | 6 | GC04P022121 | 1.004889 | https://www.genecards.org/cgi-bin/carddisp.pl?gene=RNU6-420P |
| RPS2P9 | Ribosomal Protein S2 Pseudogene 9 | Pseudogene | 6 | GC01P198950 | 1.004889 | https://www.genecards.org/cgi-bin/carddisp.pl?gene=RPS2P9 |
| TREHP1 | Trehalase Pseudogene 1 | Pseudogene | 6 | GC11M118688 | 1.004889 | https://www.genecards.org/cgi-bin/carddisp.pl?gene=TREHP1 |
| VDAC1P9 | Voltage Dependent Anion Channel 1 Pseudogene 9 | Pseudogene | 6 | GC01M157724 | 1.004889 | https://www.genecards.org/cgi-bin/carddisp.pl?gene=VDAC1P9 |

| | | | | | |
|---|---|---|---|---|---|
| hsa-miR-5096-032 | RNA Gene | 6 | GC05M 054039 | 1.004 889 | https://www.genecards.org/cgi-bin/carddisp.pl?gene=hsa-miR-5096-032 |
| lnc-ALPK1-2 | RNA Gene | 6 | GC04P 112286 | 1.004 889 | https://www.genecards.org/cgi-bin/carddisp.pl?gene=lnc-ALPK1-2 |
| lnc-ANXA6-3 | RNA Gene | 6 | GC05M 151030 | 1.004 889 | https://www.genecards.org/cgi-bin/carddisp.pl?gene=lnc-ANXA6-3 |
| lnc-CD247-1 | RNA Gene | 6 | GC01M 167430 | 1.004 889 | https://www.genecards.org/cgi-bin/carddisp.pl?gene=lnc-CD247-1 |
| lnc-FAM76B-1 | RNA Gene | 6 | GC11M 095571 | 1.004 889 | https://www.genecards.org/cgi-bin/carddisp.pl?gene=lnc-FAM76B-1 |
| lnc-IL10-5 | RNA Gene | 6 | GC01M 206769 | 1.004 889 | https://www.genecards.org/cgi-bin/carddisp.pl?gene=lnc-IL10-5 |
| lnc-KCNE1-1 | RNA Gene | 6 | GC21M 034577 | 1.004 889 | https://www.genecards.org/cgi-bin/carddisp.pl?gene=lnc-KCNE1-1 |
| lnc-MAP3K7-3 | RNA Gene | 6 | GC06M 090088 | 1.004 889 | https://www.genecards.org/cgi-bin/carddisp.pl?gene=lnc-MAP3K7-3 |
| lnc-PRDM1-3 | RNA Gene | 6 | GC06P 106132 | 1.004 889 | https://www.genecards.org/cgi-bin/carddisp.pl?gene=lnc-PRDM1-3 |
| lnc-RCAN1-1 | RNA Gene | 6 | GC21M 034514 | 1.004 889 | https://www.genecards.org/cgi-bin/carddisp.pl?gene=lnc-RCAN1-1 |
| lnc-RCSD1-5 | RNA Gene | 6 | GC01P 167418 | 1.004 889 | https://www.genecards.org/cgi-bin/carddisp.pl?gene=lnc-RCSD1-5 |
| lnc-SBF2-3 | RNA Gene | 6 | GC11M 009760 | 1.004 889 | https://www.genecards.org/cgi-bin/carddisp.pl?gene=lnc-SBF2-3 |
| lnc-TMEM50B-3 | RNA Gene | 6 | GC21M 033384 | 1.004 889 | https://www.genecards.org/cgi-bin/carddisp.pl?gene=lnc-TMEM50B-3 |
| lnc-VPS37C-3 | RNA Gene | 6 | GC11M 089558 | 1.004 889 | https://www.genecards.org/cgi- |

| | | | | | | |
|---|---|---|---|---|---|---|
| | | | | | | bin/carddisp.pl?gene=lnc-VPS37C-3 |
| ENSG00000197376 | Biorientation Of Chromosomes In Cell Division 1 Like 2 (BOD1L2) Pseudogene | Pseudo gene | 5 | GC12P048508 | 1.004889 | https://www.genecards.org/cgi-bin/carddisp.pl?gene=ENSG00000197376 |
| ENSG00000220614 | Ribosomal Protein L34 (RPL34) Pseudogene | Pseudo gene | 5 | GC06P083919 | 1.004889 | https://www.genecards.org/cgi-bin/carddisp.pl?gene=ENSG00000220614 |
| ENSG00000224163 | Pseudogene Similar To Part Of Filamin C, Gamma (Actin Binding Protein 280) (FLNC) | Pseudo gene | 5 | GC07M128915 | 1.004889 | https://www.genecards.org/cgi-bin/carddisp.pl?gene=ENSG00000224163 |
| ENSG00000233196 | Novel Pseudogene | Pseudo gene | 5 | GC01M186581 | 1.004889 | https://www.genecards.org/cgi-bin/carddisp.pl?gene=ENSG00000233196 |
| ENSG00000240399 | Ribosomal Protein L37 (RPL37) Pseudogene | Pseudo gene | 5 | GC12M048055 | 1.004889 | https://www.genecards.org/cgi-bin/carddisp.pl?gene=ENSG00000240399 |
| ENSG00000271128 | Chromosome 7 Open Reading Frame 73 (C7orf73) Pseudogene | Pseudo gene | 5 | GC05M134082 | 1.004889 | https://www.genecards.org/cgi-bin/carddisp.pl?gene=ENSG00000271128 |
| ENSG00000271547 | Chromosome 20 Open Reading Frame 27 (C20orf27) Pseudogene | Pseudo gene | 5 | GC12M049419 | 1.004889 | https://www.genecards.org/cgi-bin/carddisp.pl?gene=ENSG00000271547 |
| ENSG00000272980 | Novel Transcript | RNA Gene | 5 | GC06P167000 | 1.004889 | https://www.genecards.org/cgi-bin/carddisp.pl?gene=ENSG00000272980 |
| ENSG00000273112 | Novel Transcript, Readthrough Between FCGR2A And FCGR2C Polymormphic Pseudogene | RNA Gene | 5 | GC01P161704 | 1.004889 | https://www.genecards.org/cgi-bin/carddisp.pl?gene=ENSG00000273112 |
| ENSG00000274425 | Novel Transcript, Antisense To ICAM3 | RNA Gene | 5 | GC19P010333 | 1.004889 | https://www.genecards.org/cgi-bin/carddisp.pl?gene=ENSG00000274425 |
| ENSG00000280216 | TEC | Uncategorized | 5 | GC22P039381 | 1.004889 | https://www.genecards.org/cgi-bin/carddisp.pl?gene=ENSG00000280216 |
| ENSG00000284914 | Novel Transcript | RNA Gene | 5 | GC06M167126 | 1.004889 | https://www.genecards.org/cgi-bin/carddisp.pl?gene=ENSG00000284914 |
| ENSG00000285842 | Novel Transcript | RNA Gene | 5 | GC11P095571 | 1.004889 | https://www.genecards.org/cgi- |

| | | | | | | |
|---|---|---|---|---|---|---|
| | | | | | | bin/carddisp.pl?gene=ENSG00000285842 |
| ENSG00000288064 | Novel Transcript, Antisense To STAT4 | RNA Gene | 5 | GC02P191045 | 1.004889 | https://www.genecards.org/cgi-bin/carddisp.pl?gene=ENSG00000288064 |
| ENSG00000288711 | Novel Protein | Protein Coding | 5 | GC21M034511 | 1.004889 | https://www.genecards.org/cgi-bin/carddisp.pl?gene=ENSG00000288711 |
| HSALNG0001400 | | RNA Gene | 5 | GC01P019765 | 1.004889 | https://www.genecards.org/cgi-bin/carddisp.pl?gene=HSALNG0001400 |
| HSALNG0007924 | | RNA Gene | 5 | GC01M161280 | 1.004889 | https://www.genecards.org/cgi-bin/carddisp.pl?gene=HSALNG0007924 |
| HSALNG0008504 | | RNA Gene | 5 | GC01P173188 | 1.004889 | https://www.genecards.org/cgi-bin/carddisp.pl?gene=HSALNG0008504 |
| HSALNG0021150 | | RNA Gene | 5 | GC02M191076 | 1.004889 | https://www.genecards.org/cgi-bin/carddisp.pl?gene=HSALNG0021150 |
| HSALNG0021699 | | RNA Gene | 5 | GC02P203869 | 1.004889 | https://www.genecards.org/cgi-bin/carddisp.pl?gene=HSALNG0021699 |
| HSALNG0024811 | | RNA Gene | 5 | GC03M027718 | 1.004889 | https://www.genecards.org/cgi-bin/carddisp.pl?gene=HSALNG0024811 |
| HSALNG0035396 | | RNA Gene | 5 | GC04P078693 | 1.004889 | https://www.genecards.org/cgi-bin/carddisp.pl?gene=HSALNG0035396 |
| HSALNG0035397 | | RNA Gene | 5 | GC04M078605 | 1.004889 | https://www.genecards.org/cgi-bin/carddisp.pl?gene=HSALNG0035397 |
| HSALNG0036439 | | RNA Gene | 5 | GC04M112278 | 1.004889 | https://www.genecards.org/cgi-bin/carddisp.pl?gene=HSALNG0036439 |
| HSALNG0045887 | | RNA Gene | 5 | GC05P151057 | 1.004889 | https://www.genecards.org/cgi-bin/carddisp.pl?gene=HSALNG0045887 |
| HSALNG0045890 | | RNA Gene | 5 | GC05P151084 | 1.004889 | https://www.genecards.org/cgi-bin/carddisp.pl?gene=HSALNG0045890 |

| | | | | | |
|---|---|---|---|---|---|
| HSALNG0049408 | RNA Gene | 5 | GC06P 084605 | 1.004 889 | https://www.genecards.org/cgi-bin/carddisp.pl?gene=HSALNG0049408 |
| HSALNG0049409 | RNA Gene | 5 | GC06P 084606 | 1.004 889 | https://www.genecards.org/cgi-bin/carddisp.pl?gene=HSALNG0049409 |
| HSALNG0049410 | RNA Gene | 5 | GC06P 084607 | 1.004 889 | https://www.genecards.org/cgi-bin/carddisp.pl?gene=HSALNG0049410 |
| HSALNG0049413 | RNA Gene | 5 | GC06P 084608 | 1.004 889 | https://www.genecards.org/cgi-bin/carddisp.pl?gene=HSALNG0049413 |
| HSALNG0049415 | RNA Gene | 5 | GC06P 084609 | 1.004 889 | https://www.genecards.org/cgi-bin/carddisp.pl?gene=HSALNG0049415 |
| HSALNG0050281 | RNA Gene | 5 | GC06M 066833 | 1.004 889 | https://www.genecards.org/cgi-bin/carddisp.pl?gene=HSALNG0050281 |
| HSALNG0055144 | RNA Gene | 5 | GC06P 167112 | 1.004 889 | https://www.genecards.org/cgi-bin/carddisp.pl?gene=HSALNG0055144 |
| HSALNG0061260 | RNA Gene | 5 | GC07M 128933 | 1.004 889 | https://www.genecards.org/cgi-bin/carddisp.pl?gene=HSALNG0061260 |
| HSALNG0061261 | RNA Gene | 5 | GC07P 132005 | 1.004 889 | https://www.genecards.org/cgi-bin/carddisp.pl?gene=HSALNG0061261 |
| HSALNG0063445 | RNA Gene | 5 | GC08M 011494 | 1.004 889 | https://www.genecards.org/cgi-bin/carddisp.pl?gene=HSALNG0063445 |
| HSALNG0074141 | RNA Gene | 5 | GC09M 120870 | 1.004 889 | https://www.genecards.org/cgi-bin/carddisp.pl?gene=HSALNG0074141 |
| HSALNG0074148-001 | RNA Gene | 5 | GC09M 120932 | 1.004 889 | https://www.genecards.org/cgi-bin/carddisp.pl?gene=HSALNG0074148-001 |
| HSALNG0074148-002 | RNA Gene | 5 | GC09M 120934 | 1.004 889 | https://www.genecards.org/cgi-bin/carddisp.pl?gene=HSALNG0074148-002 |

| | | | | | |
|---|---|---|---|---|---|
| HSALNG0075250 | RNA Gene | 5 | GC09M 136393 | 1.004 889 | https://www.genecards.org/cgi-bin/carddisp.pl?gene=HSALNG0075250 |
| HSALNG0076038 | RNA Gene | 5 | GC10P 008048 | 1.004 889 | https://www.genecards.org/cgi-bin/carddisp.pl?gene=HSALNG0076038 |
| HSALNG0076041 | RNA Gene | 5 | GC10M 008055 | 1.004 889 | https://www.genecards.org/cgi-bin/carddisp.pl?gene=HSALNG0076041 |
| HSALNG0079213 | RNA Gene | 5 | GC10M 081925 | 1.004 889 | https://www.genecards.org/cgi-bin/carddisp.pl?gene=HSALNG0079213 |
| HSALNG0084559 | RNA Gene | 5 | GC11P 061825 | 1.004 889 | https://www.genecards.org/cgi-bin/carddisp.pl?gene=HSALNG0084559 |
| HSALNG0084560 | RNA Gene | 5 | GC11M 090798 | 1.004 889 | https://www.genecards.org/cgi-bin/carddisp.pl?gene=HSALNG0084560 |
| HSALNG0084561-001 | RNA Gene | 5 | GC11P 061842 | 1.004 889 | https://www.genecards.org/cgi-bin/carddisp.pl?gene=HSALNG0084561-001 |
| HSALNG0087578 | RNA Gene | 5 | GC11P 118845 | 1.004 889 | https://www.genecards.org/cgi-bin/carddisp.pl?gene=HSALNG0087578 |
| HSALNG0094037 | RNA Gene | 5 | GC12P 111432 | 1.004 889 | https://www.genecards.org/cgi-bin/carddisp.pl?gene=HSALNG0094037 |
| HSALNG0102522 | RNA Gene | 5 | GC14M 075477 | 1.004 889 | https://www.genecards.org/cgi-bin/carddisp.pl?gene=HSALNG0102522 |
| HSALNG0122747 | RNA Gene | 5 | GC18M 079391 | 1.004 889 | https://www.genecards.org/cgi-bin/carddisp.pl?gene=HSALNG0122747 |
| HSALNG0122748 | RNA Gene | 5 | GC18P 079392 | 1.004 889 | https://www.genecards.org/cgi-bin/carddisp.pl?gene=HSALNG0122748 |
| HSALNG0122749 | RNA Gene | 5 | GC18P 079394 | 1.004 889 | https://www.genecards.org/cgi-bin/carddisp.pl?gene=HSALNG0122749 |

| | | | | | | |
|---|---|---|---|---|---|---|
| HSALNG0132765 | | RNA Gene | 5 | GC21M 033400 | 1.004 889 | https://www.genecards.org/cgi-bin/carddisp.pl?gene=HSALNG0132765 |
| HSALNG0135279 | | RNA Gene | 5 | GC22M 037128 | 1.004 889 | https://www.genecards.org/cgi-bin/carddisp.pl?gene=HSALNG0135279 |
| HSALNG0135499 | | RNA Gene | 5 | GC22P 039433 | 1.004 889 | https://www.genecards.org/cgi-bin/carddisp.pl?gene=HSALNG0135499 |
| LOC1249 01069 | Short Transmembrane Mitochondrial Protein 1-Like | Protein Coding | 5 | GC05M 134083 | 1.004 889 | https://www.genecards.org/cgi-bin/carddisp.pl?gene=LOC124901069 |
| LOC1249 02436 | Talanin | Protein Coding | 5 | GC10P 062643 | 1.004 889 | https://www.genecards.org/cgi-bin/carddisp.pl?gene=LOC124902436 |
| LOC1249 02802 | Protein GVQW1-Like | Protein Coding | 5 | GC11P 009817 | 1.004 889 | https://www.genecards.org/cgi-bin/carddisp.pl?gene=LOC124902802 |
| LOC1249 03162 | Uncharacterized LOC124903162 | Protein Coding | 5 | GC13M 040107 | 1.004 889 | https://www.genecards.org/cgi-bin/carddisp.pl?gene=LOC124903162 |
| LOC1249 04063 | Uncharacterized LOC124904063 | Protein Coding | 5 | GC17P 078327 | 1.004 889 | https://www.genecards.org/cgi-bin/carddisp.pl?gene=LOC124904063 |
| LOC1249 09485 | Uncharacterized LOC124909485 | Protein Coding | 5 | GC03P 058172 | 1.004 889 | https://www.genecards.org/cgi-bin/carddisp.pl?gene=LOC124909485 |
| LOC6454 81 | Zinc Finger Protein 248 Pseudogene | Pseudo gene | 5 | GC04P 026127 | 1.004 889 | https://www.genecards.org/cgi-bin/carddisp.pl?gene=LOC645481 |
| MK280073-024 | | RNA Gene | 5 | GC01P 173325 | 1.004 889 | https://www.genecards.org/cgi-bin/carddisp.pl?gene=MK280073-024 |
| MK280144-573 | | RNA Gene | 5 | GC06M 067897 | 1.004 889 | https://www.genecards.org/cgi-bin/carddisp.pl?gene=MK280144-573 |
| MK280269-008 | | RNA Gene | 5 | GC10M 070214 | 1.004 889 | https://www.genecards.org/cgi-bin/carddisp.pl?gene=MK280269-008 |
| MN297545 | | RNA Gene | 5 | GC18M 079398 | 1.004 889 | https://www.genecards.org/cgi- |

| | | | | | |
|---|---|---|---|---|---|
| | | | | | bin/carddisp.pl?gene=MN297545 |
| MN298114-116 | | RNA Gene | 5 | GC21P034282 | 1.004889 | https://www.genecards.org/cgi-bin/carddisp.pl?gene=MN298114-116 |
| MN309174-057 | | RNA Gene | 5 | GC10P092764 | 1.004889 | https://www.genecards.org/cgi-bin/carddisp.pl?gene=MN309174-057 |
| NDUFB9P2 | NADH:Ubiquinone Oxidoreductase Subunit B9 Pseudogene 2 | Pseudogene | 5 | GC07M131754 | 1.004889 | https://www.genecards.org/cgi-bin/carddisp.pl?gene=NDUFB9P2 |
| NONHSAG001750.2 | | RNA Gene | 5 | GC01P067182 | 1.004889 | https://www.genecards.org/cgi-bin/carddisp.pl?gene=NONHSAG001750.2 |
| NONHSAG001753.2 | | RNA Gene | 5 | GC01P067308 | 1.004889 | https://www.genecards.org/cgi-bin/carddisp.pl?gene=NONHSAG001753.2 |
| NONHSAG005179.2-001 | | RNA Gene | 5 | GC10P008054 | 1.004889 | https://www.genecards.org/cgi-bin/carddisp.pl?gene=NONHSAG005179.2-001 |
| NONHSAG005179.2-002 | | RNA Gene | 5 | GC10P008064 | 1.004889 | https://www.genecards.org/cgi-bin/carddisp.pl?gene=NONHSAG005179.2-002 |
| NONHSAG031883.2-001 | | RNA Gene | 5 | GC20P046119 | 1.004889 | https://www.genecards.org/cgi-bin/carddisp.pl?gene=NONHSAG031883.2-001 |
| NONHSAG043563.2 | | RNA Gene | 5 | GC06P085607 | 1.004889 | https://www.genecards.org/cgi-bin/carddisp.pl?gene=NONHSAG043563.2 |
| NONHSAG044958.2 | | RNA Gene | 5 | GC06P137874 | 1.004889 | https://www.genecards.org/cgi-bin/carddisp.pl?gene=NONHSAG044958.2 |
| RF00017-1167 | | RNA Gene | 5 | GC12M111251 | 1.004889 | https://www.genecards.org/cgi-bin/carddisp.pl?gene=RF00017-1167 |
| RF00017-4245 | | RNA Gene | 5 | GC05M054016 | 1.004889 | https://www.genecards.org/cgi-bin/carddisp.pl?gene=RF00017-4245 |

| | | | | | |
|---|---|---|---|---|---|
| RF00017-4626 | RNA Gene | 5 | GC05P 150704 | 1.004 889 | https://www.genecards.org/cgi-bin/carddisp.pl?gene=RF00017-4626 |
| RF00017-5297 | RNA Gene | 5 | GC06M 105987 | 1.004 889 | https://www.genecards.org/cgi-bin/carddisp.pl?gene=RF00017-5297 |
| RF00017-6101 | RNA Gene | 5 | GC07P 101334 | 1.004 889 | https://www.genecards.org/cgi-bin/carddisp.pl?gene=RF00017-6101 |
| RF00017-649 | RNA Gene | 5 | GC10P 093424 | 1.004 889 | https://www.genecards.org/cgi-bin/carddisp.pl?gene=RF00017-649 |
| RF00019-050 | RNA Gene | 5 | GC15P 042442 | 1.004 889 | https://www.genecards.org/cgi-bin/carddisp.pl?gene=RF00019-050 |
| RF00026-588 | RNA Gene | 5 | GC02P 191125 | 1.004 889 | https://www.genecards.org/cgi-bin/carddisp.pl?gene=RF00026-588 |
| RF00026-650 | RNA Gene | 5 | GC21M 042431 | 1.004 889 | https://www.genecards.org/cgi-bin/carddisp.pl?gene=RF00026-650 |
| lnc-ADD2-2 | RNA Gene | 5 | GC02M 070456 | 1.004 889 | https://www.genecards.org/cgi-bin/carddisp.pl?gene=lnc-ADD2-2 |
| lnc-ANKRD33B-6 | RNA Gene | 5 | GC05P 010698 | 1.004 889 | https://www.genecards.org/cgi-bin/carddisp.pl?gene=lnc-ANKRD33B-6 |
| lnc-ANKRD33B-7 | RNA Gene | 5 | GC05P 010708 | 1.004 889 | https://www.genecards.org/cgi-bin/carddisp.pl?gene=lnc-ANKRD33B-7 |
| lnc-ASAP1-6 | RNA Gene | 5 | GC08M 129855 | 1.004 889 | https://www.genecards.org/cgi-bin/carddisp.pl?gene=lnc-ASAP1-6 |
| lnc-BAD-1 | RNA Gene | 5 | GC11M 064276 | 1.004 889 | https://www.genecards.org/cgi-bin/carddisp.pl?gene=lnc-BAD-1 |
| lnc-C5-1 | RNA Gene | 5 | GC09M 120924 | 1.004 889 | https://www.genecards.org/cgi-bin/carddisp.pl?gene=lnc-C5-1 |
| lnc-CASTOR1-1 | RNA Gene | 5 | GC22M 057165 | 1.004 889 | https://www.genecards.org/cgi- |

| | | | | | |
|---|---|---|---|---|---|
| | | | | | bin/carddisp.pl?gene=lnc-CASTOR1-1 |
| lnc-CD83-11 | RNA Gene | 5 | GC06P014094 | 1.004889 | https://www.genecards.org/cgi-bin/carddisp.pl?gene=lnc-CD83-11 |
| lnc-CD83-7 | RNA Gene | 5 | GC06P014100 | 1.004889 | https://www.genecards.org/cgi-bin/carddisp.pl?gene=lnc-CD83-7 |
| lnc-CEP76-3 | RNA Gene | 5 | GC18M024982 | 1.004889 | https://www.genecards.org/cgi-bin/carddisp.pl?gene=lnc-CEP76-3 |
| lnc-CHP2-1 | RNA Gene | 5 | GC16P023875 | 1.004889 | https://www.genecards.org/cgi-bin/carddisp.pl?gene=lnc-CHP2-1 |
| lnc-CREB5-4 | RNA Gene | 5 | GC07P028146 | 1.004889 | https://www.genecards.org/cgi-bin/carddisp.pl?gene=lnc-CREB5-4 |
| lnc-CTNND2-10 | RNA Gene | 5 | GC05M010743 | 1.004889 | https://www.genecards.org/cgi-bin/carddisp.pl?gene=lnc-CTNND2-10 |
| lnc-CTNND2-6 | RNA Gene | 5 | GC05M010707 | 1.004889 | https://www.genecards.org/cgi-bin/carddisp.pl?gene=lnc-CTNND2-6 |
| lnc-CYP1B1-7 | RNA Gene | 5 | GC02M037679 | 1.004889 | https://www.genecards.org/cgi-bin/carddisp.pl?gene=lnc-CYP1B1-7 |
| lnc-DYDC1-12 | RNA Gene | 5 | GC10M080453 | 1.004889 | https://www.genecards.org/cgi-bin/carddisp.pl?gene=lnc-DYDC1-12 |
| lnc-ECHDC3-8 | RNA Gene | 5 | GC10P011253 | 1.004889 | https://www.genecards.org/cgi-bin/carddisp.pl?gene=lnc-ECHDC3-8 |
| lnc-FAM109A-1 | RNA Gene | 5 | GC12M111536 | 1.004889 | https://www.genecards.org/cgi-bin/carddisp.pl?gene=lnc-FAM109A-1 |
| lnc-FAM109A-2 | RNA Gene | 5 | GC12M111405 | 1.004889 | https://www.genecards.org/cgi-bin/carddisp.pl?gene=lnc-FAM109A-2 |
| lnc-FAM133B-2 | RNA Gene | 5 | GC07M092611 | 1.004889 | https://www.genecards.org/cgi-bin/carddisp.pl?gene=lnc-FAM133B-2 |

| | | | | | |
|---|---|---|---|---|---|
| lnc-FNDC1-8 | RNA Gene | 5 | GC06P 160581 | 1.004 889 | https://www.genecards .org/cgi- bin/carddisp.pl?gene=l nc-FNDC1-8 |
| lnc-GATA3-19-001 | RNA Gene | 5 | GC10P 008992 | 1.004 889 | https://www.genecards .org/cgi- bin/carddisp.pl?gene=l nc-GATA3-19-001 |
| lnc-GATA3-20 | RNA Gene | 5 | GC10P 009028 | 1.004 889 | https://www.genecards .org/cgi- bin/carddisp.pl?gene=l nc-GATA3-20 |
| lnc-GIN1-2 | RNA Gene | 5 | GC05M 103281 | 1.004 889 | https://www.genecards .org/cgi- bin/carddisp.pl?gene=l nc-GIN1-2 |
| lnc-HLA-DQA1-10 | RNA Gene | 5 | GC06P 085069 | 1.004 889 | https://www.genecards .org/cgi- bin/carddisp.pl?gene=l nc-HLA-DQA1-10 |
| lnc-HLA-DQA1-9 | RNA Gene | 5 | GC06P 085071 | 1.004 889 | https://www.genecards .org/cgi- bin/carddisp.pl?gene=l nc-HLA-DQA1-9 |
| lnc-HLA-DRB1-7 | RNA Gene | 5 | GC06M 032609 | 1.004 889 | https://www.genecards .org/cgi- bin/carddisp.pl?gene=l nc-HLA-DRB1-7 |
| lnc-HLA-DRB5-1-002 | RNA Gene | 5 | GC06M 032459 | 1.004 889 | https://www.genecards .org/cgi- bin/carddisp.pl?gene=l nc-HLA-DRB5-1-002 |
| lnc-IGSF3-3 | RNA Gene | 5 | GC01M 116740 | 1.004 889 | https://www.genecards .org/cgi- bin/carddisp.pl?gene=l nc-IGSF3-3 |
| lnc-IL2RA-4 | RNA Gene | 5 | GC10M 006071 | 1.004 889 | https://www.genecards .org/cgi- bin/carddisp.pl?gene=l nc-IL2RA-4 |
| lnc-IL2RB-1 | RNA Gene | 5 | GC22M 037139 | 1.004 889 | https://www.genecards .org/cgi- bin/carddisp.pl?gene=l nc-IL2RB-1 |
| lnc-IRF5-3 | RNA Gene | 5 | GC07P 132040 | 1.004 889 | https://www.genecards .org/cgi- bin/carddisp.pl?gene=l nc-IRF5-3 |
| lnc-KIN-10 | RNA Gene | 5 | GC10M 008992 | 1.004 889 | https://www.genecards .org/cgi- bin/carddisp.pl?gene=l nc-KIN-10 |
| lnc-MCUR1-13 | RNA Gene | 5 | GC06M 014092 | 1.004 889 | https://www.genecards .org/cgi- |

| | | | | | |
|---|---|---|---|---|---|
| | | | | | bin/carddisp.pl?gene=lnc-MCUR1-13 |
| lnc-OLIG3-1 | RNA Gene | 5 | GC06M137671 | 1.004889 | https://www.genecards.org/cgi-bin/carddisp.pl?gene=lnc-OLIG3-1 |
| lnc-PFKFB3-3 | RNA Gene | 5 | GC10P006343 | 1.004889 | https://www.genecards.org/cgi-bin/carddisp.pl?gene=lnc-PFKFB3-3 |
| lnc-PLA2G1B-1 | RNA Gene | 5 | GC12M120353 | 1.004889 | https://www.genecards.org/cgi-bin/carddisp.pl?gene=lnc-PLA2G1B-1 |
| lnc-PQLC1-16 | RNA Gene | 5 | GC18M079397 | 1.004889 | https://www.genecards.org/cgi-bin/carddisp.pl?gene=lnc-PQLC1-16 |
| lnc-PTPRC-10 | RNA Gene | 5 | GC01P199165 | 1.004889 | https://www.genecards.org/cgi-bin/carddisp.pl?gene=lnc-PTPRC-10 |
| lnc-PTTG1-5 | RNA Gene | 5 | GC05P160447 | 1.004889 | https://www.genecards.org/cgi-bin/carddisp.pl?gene=lnc-PTTG1-5 |
| lnc-PUS10-2 | RNA Gene | 5 | GC02M060834 | 1.004889 | https://www.genecards.org/cgi-bin/carddisp.pl?gene=lnc-PUS10-2 |
| lnc-RAB1A-13 | RNA Gene | 5 | GC02M065341 | 1.004889 | https://www.genecards.org/cgi-bin/carddisp.pl?gene=lnc-RAB1A-13 |
| lnc-RPLP1-6 | RNA Gene | 5 | GC15P117550 | 1.004889 | https://www.genecards.org/cgi-bin/carddisp.pl?gene=lnc-RPLP1-6 |
| lnc-RTKN2-5 | RNA Gene | 5 | GC10M062022 | 1.004889 | https://www.genecards.org/cgi-bin/carddisp.pl?gene=lnc-RTKN2-5 |
| lnc-RTTN-6 | RNA Gene | 5 | GC18M069930 | 1.004889 | https://www.genecards.org/cgi-bin/carddisp.pl?gene=lnc-RTTN-6 |
| lnc-SLC35G5-2 | RNA Gene | 5 | GC08P011494 | 1.004889 | https://www.genecards.org/cgi-bin/carddisp.pl?gene=lnc-SLC35G5-2 |
| lnc-SNN-1 | RNA Gene | 5 | GC16P012273 | 1.004889 | https://www.genecards.org/cgi-bin/carddisp.pl?gene=lnc-SNN-1 |

| | | | | | |
|---|---|---|---|---|---|
| lnc-SWAP70-4 | RNA Gene | 5 | GC11P 009764 | 1.004 889 | https://www.genecards.org/cgi-bin/carddisp.pl?gene=lnc-SWAP70-4 |
| lnc-TLE3-16 | RNA Gene | 5 | GC15M 069721 | 1.004 889 | https://www.genecards.org/cgi-bin/carddisp.pl?gene=lnc-TLE3-16 |
| lnc-TMEM187-3 | RNA Gene | 5 | GC0XP 154020 | 1.004 889 | https://www.genecards.org/cgi-bin/carddisp.pl?gene=lnc-TMEM187-3 |
| lnc-TNFRSF14-1 | RNA Gene | 5 | GC01P 003932 | 1.004 889 | https://www.genecards.org/cgi-bin/carddisp.pl?gene=lnc-TNFRSF14-1 |
| lnc-TRPC3-4 | RNA Gene | 5 | GC04M 122150 | 1.004 889 | https://www.genecards.org/cgi-bin/carddisp.pl?gene=lnc-TRPC3-4 |
| lnc-VWCE-2 | RNA Gene | 5 | GC11M 061138 | 1.004 889 | https://www.genecards.org/cgi-bin/carddisp.pl?gene=lnc-VWCE-2 |
| lnc-WDHD1-3 | RNA Gene | 5 | GC14M 054843 | 1.004 889 | https://www.genecards.org/cgi-bin/carddisp.pl?gene=lnc-WDHD1-3 |
| lnc-YDJC-2 | RNA Gene | 5 | GC22M 021640 | 1.004 889 | https://www.genecards.org/cgi-bin/carddisp.pl?gene=lnc-YDJC-2 |
| lnc-ZEB1-2 | RNA Gene | 5 | GC10P 031133 | 1.004 889 | https://www.genecards.org/cgi-bin/carddisp.pl?gene=lnc-ZEB1-2 |
| lnc-ZFC3H1-13 | RNA Gene | 5 | GC12M 072432 | 1.004 889 | https://www.genecards.org/cgi-bin/carddisp.pl?gene=lnc-ZFC3H1-13 |
| piR-32940 | RNA Gene | 5 | GC11P 061829 | 1.004 889 | https://www.genecards.org/cgi-bin/carddisp.pl?gene=piR-32940 |
| piR-36182-002 | RNA Gene | 5 | GC01P 160440 | 1.004 889 | https://www.genecards.org/cgi-bin/carddisp.pl?gene=piR-36182-002 |
| piR-43107-023 | RNA Gene | 5 | GC01M 167452 | 1.004 889 | https://www.genecards.org/cgi-bin/carddisp.pl?gene=piR-43107-023 |
| piR-44098 | RNA Gene | 5 | GC11M 009755 | 1.004 889 | https://www.genecards.org/cgi- |

| | | | | | | |
|---|---|---|---|---|---|---|
| | | | | | | bin/carddisp.pl?gene=piR-44098 |
| piR-52079-162 | | RNA Gene | 5 | GC04M112286 | 1.004889 | https://www.genecards.org/cgi-bin/carddisp.pl?gene=piR-52079-162 |
| piR-56037-112 | | RNA Gene | 5 | GC09P120932 | 1.004889 | https://www.genecards.org/cgi-bin/carddisp.pl?gene=piR-56037-112 |
| ENSG00000220412 | Protein Tyrosine Phosphatase, Non-Receptor Type 11 (PTPN11) Pseudogene | Pseudogene | 4 | GC06M137706 | 1.004889 | https://www.genecards.org/cgi-bin/carddisp.pl?gene=ENSG00000220412 |
| ENSG00000254478 | Cytochrome C Oxidase Subunit VIIc (COX7C) Pseudogene | Pseudogene | 4 | GC11P118821 | 1.004889 | https://www.genecards.org/cgi-bin/carddisp.pl?gene=ENSG00000254478 |
| ENSG00000259970 | Novel Transcript | RNA Gene | 4 | GC03M049684 | 1.004889 | https://www.genecards.org/cgi-bin/carddisp.pl?gene=ENSG00000259970 |
| ENSG00000260678 | Zinc Finger Protein (ZNF) Pseudogene | Pseudogene | 4 | GC16M037188 | 1.004889 | https://www.genecards.org/cgi-bin/carddisp.pl?gene=ENSG00000260678 |
| ENSG00000275106 | Novel Transcript | Uncategorized | 4 | GC07M128952 | 1.004889 | https://www.genecards.org/cgi-bin/carddisp.pl?gene=ENSG00000275106 |
| ENSG00000279549 | Novel Transcript | Uncategorized | 4 | GC11M061143 | 1.004889 | https://www.genecards.org/cgi-bin/carddisp.pl?gene=ENSG00000279549 |
| ENSG00000288975 | Novel Transcript | RNA Gene | 4 | GC11P128628 | 1.004889 | https://www.genecards.org/cgi-bin/carddisp.pl?gene=ENSG00000288975 |
| HSALNG0001255 | | RNA Gene | 4 | GC01P017846 | 1.004889 | https://www.genecards.org/cgi-bin/carddisp.pl?gene=HSALNG0001255 |
| HSALNG0001405 | | RNA Gene | 4 | GC01P019861 | 1.004889 | https://www.genecards.org/cgi-bin/carddisp.pl?gene=HSALNG0001405 |
| HSALNG0007948-001 | | RNA Gene | 4 | GC01P161702 | 1.004889 | https://www.genecards.org/cgi-bin/carddisp.pl?gene=HSALNG0007948-001 |
| HSALNG0007948-002 | | RNA Gene | 4 | GC01P161703 | 1.004889 | https://www.genecards.org/cgi-bin/carddisp.pl?gene= |

| | | | | | |
|---|---|---|---|---|---|
| | | | | | HSALNG0007948-002 |
| HSALNG0009100 | RNA Gene | 4 | GC01P 186657 | 1.004 889 | https://www.genecards.org/cgi-bin/carddisp.pl?gene=HSALNG0009100 |
| HSALNG0009535 | RNA Gene | 4 | GC01P 199237 | 1.004 889 | https://www.genecards.org/cgi-bin/carddisp.pl?gene=HSALNG0009535 |
| HSALNG0009536 | RNA Gene | 4 | GC01M 199344 | 1.004 889 | https://www.genecards.org/cgi-bin/carddisp.pl?gene=HSALNG0009536 |
| HSALNG0009608 | RNA Gene | 4 | GC01P 200902 | 1.004 889 | https://www.genecards.org/cgi-bin/carddisp.pl?gene=HSALNG0009608 |
| HSALNG0009609 | RNA Gene | 4 | GC01P 200908 | 1.004 889 | https://www.genecards.org/cgi-bin/carddisp.pl?gene=HSALNG0009609 |
| HSALNG0013961 | RNA Gene | 4 | GC02P 030217 | 1.004 889 | https://www.genecards.org/cgi-bin/carddisp.pl?gene=HSALNG0013961 |
| HSALNG0013962 | RNA Gene | 4 | GC02P 030226 | 1.004 889 | https://www.genecards.org/cgi-bin/carddisp.pl?gene=HSALNG0013962 |
| HSALNG0015539 | RNA Gene | 4 | GC02M 065344 | 1.004 889 | https://www.genecards.org/cgi-bin/carddisp.pl?gene=HSALNG0015539 |
| HSALNG0016005 | RNA Gene | 4 | GC02M 073986 | 1.004 889 | https://www.genecards.org/cgi-bin/carddisp.pl?gene=HSALNG0016005 |
| HSALNG0021147 | RNA Gene | 4 | GC02P 191035 | 1.004 889 | https://www.genecards.org/cgi-bin/carddisp.pl?gene=HSALNG0021147 |
| HSALNG0021534 | RNA Gene | 4 | GC02P 201187 | 1.004 889 | https://www.genecards.org/cgi-bin/carddisp.pl?gene=HSALNG0021534 |
| HSALNG0021690 | RNA Gene | 4 | GC02M 203744 | 1.004 889 | https://www.genecards.org/cgi-bin/carddisp.pl?gene=HSALNG0021690 |
| HSALNG0026407 | RNA Gene | 4 | GC03M 058193 | 1.004 889 | https://www.genecards.org/cgi-bin/carddisp.pl?gene=HSALNG0026407 |

| | | | | | |
|---|---|---|---|---|---|
| HSALNG0032205 | RNA Gene | 4 | GC04P 000972 | 1.004 889 | https://www.genecards .org/cgi-bin/carddisp.pl?gene= HSALNG0032205 |
| HSALNG0033465 | RNA Gene | 4 | GC04M 026097 | 1.004 889 | https://www.genecards .org/cgi-bin/carddisp.pl?gene= HSALNG0033465 |
| HSALNG0035363 | RNA Gene | 4 | GC04M 077554 | 1.004 889 | https://www.genecards .org/cgi-bin/carddisp.pl?gene= HSALNG0035363 |
| HSALNG0035365 | RNA Gene | 4 | GC04M 077627 | 1.004 889 | https://www.genecards .org/cgi-bin/carddisp.pl?gene= HSALNG0035365 |
| HSALNG0049303 | RNA Gene | 4 | GC06P 084583 | 1.004 889 | https://www.genecards .org/cgi-bin/carddisp.pl?gene= HSALNG0049303 |
| HSALNG0049304 | RNA Gene | 4 | GC06P 084584 | 1.004 889 | https://www.genecards .org/cgi-bin/carddisp.pl?gene= HSALNG0049304 |
| HSALNG0049423 | RNA Gene | 4 | GC06P 084611 | 1.004 889 | https://www.genecards .org/cgi-bin/carddisp.pl?gene= HSALNG0049423 |
| HSALNG0058921 | RNA Gene | 4 | GC07P 075447 | 1.004 889 | https://www.genecards .org/cgi-bin/carddisp.pl?gene= HSALNG0058921 |
| HSALNG0061263 | RNA Gene | 4 | GC07P 132006 | 1.004 889 | https://www.genecards .org/cgi-bin/carddisp.pl?gene= HSALNG0061263 |
| HSALNG0067380 | RNA Gene | 4 | GC08M 101435 | 1.004 889 | https://www.genecards .org/cgi-bin/carddisp.pl?gene= HSALNG0067380 |
| HSALNG0068535 | RNA Gene | 4 | GC08M 128542 | 1.004 889 | https://www.genecards .org/cgi-bin/carddisp.pl?gene= HSALNG0068535 |
| HSALNG0068536-001 | RNA Gene | 4 | GC08P 128544 | 1.004 889 | https://www.genecards .org/cgi-bin/carddisp.pl?gene= HSALNG0068536-001 |
| HSALNG0072212 | RNA Gene | 4 | GC09P 081829 | 1.004 889 | https://www.genecards .org/cgi-bin/carddisp.pl?gene= HSALNG0072212 |

| | | | | | |
|---|---|---|---|---|---|
| HSALNG0074144 | RNA Gene | 4 | GC09P 120897 | 1.004 889 | https://www.genecards .org/cgi-bin/carddisp.pl?gene= HSALNG0074144 |
| HSALNG0075945 | RNA Gene | 4 | GC10M 006346 | 1.004 889 | https://www.genecards .org/cgi-bin/carddisp.pl?gene= HSALNG0075945 |
| HSALNG0078331 | RNA Gene | 4 | GC10P 062683 | 1.004 889 | https://www.genecards .org/cgi-bin/carddisp.pl?gene= HSALNG0078331 |
| HSALNG0078621 | RNA Gene | 4 | GC10M 070124 | 1.004 889 | https://www.genecards .org/cgi-bin/carddisp.pl?gene= HSALNG0078621 |
| HSALNG0080050 | RNA Gene | 4 | GC10M 099531 | 1.004 889 | https://www.genecards .org/cgi-bin/carddisp.pl?gene= HSALNG0080050 |
| HSALNG0080051 | RNA Gene | 4 | GC10P 099533 | 1.004 889 | https://www.genecards .org/cgi-bin/carddisp.pl?gene= HSALNG0080051 |
| HSALNG0080384 | RNA Gene | 4 | GC10P 103659 | 1.004 889 | https://www.genecards .org/cgi-bin/carddisp.pl?gene= HSALNG0080384 |
| HSALNG0084497 | RNA Gene | 4 | GC11M 090779 | 1.004 889 | https://www.genecards .org/cgi-bin/carddisp.pl?gene= HSALNG0084497 |
| HSALNG0084770 | RNA Gene | 4 | GC11P 064273 | 1.004 889 | https://www.genecards .org/cgi-bin/carddisp.pl?gene= HSALNG0084770 |
| HSALNG0085864 | RNA Gene | 4 | GC11M 076569 | 1.004 889 | https://www.genecards .org/cgi-bin/carddisp.pl?gene= HSALNG0085864 |
| HSALNG0085865 | RNA Gene | 4 | GC11P 077996 | 1.004 889 | https://www.genecards .org/cgi-bin/carddisp.pl?gene= HSALNG0085865 |
| HSALNG0087579 | RNA Gene | 4 | GC11P 118854 | 1.004 889 | https://www.genecards .org/cgi-bin/carddisp.pl?gene= HSALNG0087579 |
| HSALNG0087581 | RNA Gene | 4 | GC11M 118867 | 1.004 889 | https://www.genecards .org/cgi-bin/carddisp.pl?gene= HSALNG0087581 |
| HSALNG0088091-002 | RNA Gene | 4 | GC11P 128262 | 1.004 889 | https://www.genecards .org/cgi- |

| | | | | | |
|---|---|---|---|---|---|
| | | | | | bin/carddisp.pl?gene=HSALNG0088091-002 |
| HSALNG0088092 | RNA Gene | 4 | GC11M128259 | 1.004889 | https://www.genecards.org/cgi-bin/carddisp.pl?gene=HSALNG0088092 |
| HSALNG0092390 | RNA Gene | 4 | GC12P072287 | 1.004889 | https://www.genecards.org/cgi-bin/carddisp.pl?gene=HSALNG0092390 |
| HSALNG0094038 | RNA Gene | 4 | GC12P111446 | 1.004889 | https://www.genecards.org/cgi-bin/carddisp.pl?gene=HSALNG0094038 |
| HSALNG0096509 | RNA Gene | 4 | GC13M039754 | 1.004889 | https://www.genecards.org/cgi-bin/carddisp.pl?gene=HSALNG0096509 |
| HSALNG0101767 | RNA Gene | 4 | GC14M061406 | 1.004889 | https://www.genecards.org/cgi-bin/carddisp.pl?gene=HSALNG0101767 |
| HSALNG0101768 | RNA Gene | 4 | GC14P061442 | 1.004889 | https://www.genecards.org/cgi-bin/carddisp.pl?gene=HSALNG0101768 |
| HSALNG0101769 | RNA Gene | 4 | GC14P061525 | 1.004889 | https://www.genecards.org/cgi-bin/carddisp.pl?gene=HSALNG0101769 |
| HSALNG0102088 | RNA Gene | 4 | GC14M068248 | 1.004889 | https://www.genecards.org/cgi-bin/carddisp.pl?gene=HSALNG0102088 |
| HSALNG0102109 | RNA Gene | 4 | GC14M068741 | 1.004889 | https://www.genecards.org/cgi-bin/carddisp.pl?gene=HSALNG0102109 |
| HSALNG0102110 | RNA Gene | 4 | GC14P068744 | 1.004889 | https://www.genecards.org/cgi-bin/carddisp.pl?gene=HSALNG0102110 |
| HSALNG0106869 | RNA Gene | 4 | GC15P118498 | 1.004889 | https://www.genecards.org/cgi-bin/carddisp.pl?gene=HSALNG0106869 |
| HSALNG0109614 | RNA Gene | 4 | GC16M011691 | 1.004889 | https://www.genecards.org/cgi-bin/carddisp.pl?gene=HSALNG0109614 |
| HSALNG0109615 | RNA Gene | 4 | GC16M011707 | 1.004889 | https://www.genecards.org/cgi- |

| | | | | | |
|---|---|---|---|---|---|
| | | | | | bin/carddisp.pl?gene=HSALNG0109615 |
| HSALNG0113252 | RNA Gene | 4 | GC16M085958 | 1.004889 | https://www.genecards.org/cgi-bin/carddisp.pl?gene=HSALNG0113252 |
| HSALNG0119020 | RNA Gene | 4 | GC17P078232 | 1.004889 | https://www.genecards.org/cgi-bin/carddisp.pl?gene=HSALNG0119020 |
| HSALNG0119021 | RNA Gene | 4 | GC17P078236 | 1.004889 | https://www.genecards.org/cgi-bin/carddisp.pl?gene=HSALNG0119021 |
| HSALNG0123927 | RNA Gene | 4 | GC19M010406 | 1.004889 | https://www.genecards.org/cgi-bin/carddisp.pl?gene=HSALNG0123927 |
| HSALNG0124504 | RNA Gene | 4 | GC19P067670 | 1.004889 | https://www.genecards.org/cgi-bin/carddisp.pl?gene=HSALNG0124504 |
| HSALNG0124505 | RNA Gene | 4 | GC19P067671 | 1.004889 | https://www.genecards.org/cgi-bin/carddisp.pl?gene=HSALNG0124505 |
| HSALNG0131362 | RNA Gene | 4 | GC20M059817 | 1.004889 | https://www.genecards.org/cgi-bin/carddisp.pl?gene=HSALNG0131362 |
| HSALNG0131698 | RNA Gene | 4 | GC20M063850 | 1.004889 | https://www.genecards.org/cgi-bin/carddisp.pl?gene=HSALNG0131698 |
| HSALNG0132884-001 | RNA Gene | 4 | GC21P035319 | 1.004889 | https://www.genecards.org/cgi-bin/carddisp.pl?gene=HSALNG0132884-001 |
| HSALNG0132884-002 | RNA Gene | 4 | GC21P035358 | 1.004889 | https://www.genecards.org/cgi-bin/carddisp.pl?gene=HSALNG0132884-002 |
| HSALNG0132885 | RNA Gene | 4 | GC21M035349 | 1.004889 | https://www.genecards.org/cgi-bin/carddisp.pl?gene=HSALNG0132885 |
| HSALNG0133192 | RNA Gene | 4 | GC21P041149 | 1.004889 | https://www.genecards.org/cgi-bin/carddisp.pl?gene=HSALNG0133192 |
| HSALNG0133311 | RNA Gene | 4 | GC21M042422 | 1.004889 | https://www.genecards.org/cgi- |

| | | | | | | |
|---|---|---|---|---|---|---|
| | | | | | | bin/carddisp.pl?gene=HSALNG0133311 |
| HSALNG0134046 | | RNA Gene | 4 | GC22P035814 | 1.004889 | https://www.genecards.org/cgi-bin/carddisp.pl?gene=HSALNG0134046 |
| HSALNG0134239 | | RNA Gene | 4 | GC22P037082 | 1.004889 | https://www.genecards.org/cgi-bin/carddisp.pl?gene=HSALNG0134239 |
| HSALNG0140819 | | RNA Gene | 4 | GC0XP153988 | 1.004889 | https://www.genecards.org/cgi-bin/carddisp.pl?gene=HSALNG0140819 |
| KR153194-030 | | RNA Gene | 4 | GC11P118807 | 1.004889 | https://www.genecards.org/cgi-bin/carddisp.pl?gene=KR153194-030 |
| KR153226 | | RNA Gene | 4 | GC09M123385 | 1.004889 | https://www.genecards.org/cgi-bin/carddisp.pl?gene=KR153226 |
| LOC105369568 | Uncharacterized LOC105369568 | RNA Gene | 4 | GC11M128598 | 1.004889 | https://www.genecards.org/cgi-bin/carddisp.pl?gene=LOC105369568 |
| LOC105369750 | Uncharacterized LOC105369750 | RNA Gene | 4 | GC12P048012 | 1.004889 | https://www.genecards.org/cgi-bin/carddisp.pl?gene=LOC105369750 |
| LOC105376481 | Uncharacterized LOC105376481 | RNA Gene | 4 | GC10P031055 | 1.004889 | https://www.genecards.org/cgi-bin/carddisp.pl?gene=LOC105376481 |
| LOC105377296 | Uncharacterized LOC105377296 | RNA Gene | 4 | GC04M077580 | 1.004889 | https://www.genecards.org/cgi-bin/carddisp.pl?gene=LOC105377296 |
| LOC107984079 | Uncharacterized LOC107984079 | RNA Gene | 4 | GC03P058536 | 1.004889 | https://www.genecards.org/cgi-bin/carddisp.pl?gene=LOC107984079 |
| LOC107984788 | Uncharacterized LOC107984788 | RNA Gene | 4 | GC15P118770 | 1.004889 | https://www.genecards.org/cgi-bin/carddisp.pl?gene=LOC107984788 |
| LOC124900645 | Uncharacterized LOC124900645 | RNA Gene | 4 | GC04P000968 | 1.004889 | https://www.genecards.org/cgi-bin/carddisp.pl?gene=LOC124900645 |
| LOC124901449 | Uncharacterized LOC124901449 | Protein Coding | 4 | GC06P160592 | 1.004889 | https://www.genecards.org/cgi-bin/carddisp.pl?gene=LOC124901449 |

| | | | | | | |
|---|---|---|---|---|---|---|
| LOC124901993 | Uncharacterized LOC124901993 | Protein Coding | 4 | GC08P101312 | 1.004889 | https://www.genecards.org/cgi-bin/carddisp.pl?gene=LOC124901993 |
| LOC124902375 | Uncharacterized LOC124902375 | RNA Gene | 4 | GC10P011186 | 1.004889 | https://www.genecards.org/cgi-bin/carddisp.pl?gene=LOC124902375 |
| LOC124902435 | Uncharacterized LOC124902435 | RNA Gene | 4 | GC10M061998 | 1.004889 | https://www.genecards.org/cgi-bin/carddisp.pl?gene=LOC124902435 |
| LOC124903019 | Uncharacterized LOC124903019 | RNA Gene | 4 | GC12M111432 | 1.004889 | https://www.genecards.org/cgi-bin/carddisp.pl?gene=LOC124903019 |
| LOC124903774 | Uncharacterized LOC124903774 | RNA Gene | 4 | GC16M050728 | 1.004889 | https://www.genecards.org/cgi-bin/carddisp.pl?gene=LOC124903774 |
| MK280144-020 | | RNA Gene | 4 | GC01P067362 | 1.004889 | https://www.genecards.org/cgi-bin/carddisp.pl?gene=MK280144-020 |
| MK280144-291 | | RNA Gene | 4 | GC18P017623 | 1.004889 | https://www.genecards.org/cgi-bin/carddisp.pl?gene=MK280144-291 |
| MN298230 | | RNA Gene | 4 | GC05P160444 | 1.004889 | https://www.genecards.org/cgi-bin/carddisp.pl?gene=MN298230 |
| MN298231 | | RNA Gene | 4 | GC05M160458 | 1.004889 | https://www.genecards.org/cgi-bin/carddisp.pl?gene=MN298231 |
| MN308685 | | RNA Gene | 4 | GC01P017083 | 1.004889 | https://www.genecards.org/cgi-bin/carddisp.pl?gene=MN308685 |
| MN308836 | | RNA Gene | 4 | GC01P183559 | 1.004889 | https://www.genecards.org/cgi-bin/carddisp.pl?gene=MN308836 |
| MN308841 | | RNA Gene | 4 | GC01P186678 | 1.004889 | https://www.genecards.org/cgi-bin/carddisp.pl?gene=MN308841 |
| MN309368 | | RNA Gene | 4 | GC11M064281 | 1.004889 | https://www.genecards.org/cgi-bin/carddisp.pl?gene=MN309368 |
| NONHSAG019426.2 | | RNA Gene | 4 | GC16P050713 | 1.004889 | https://www.genecards.org/cgi- |

| | | | | | |
|---|---|---|---|---|---|
| | | | | | bin/carddisp.pl?gene=NONHSAG019426.2 |
| NONHSAG031883.2-002 | RNA Gene | 4 | GC20P046128 | 1.004889 | https://www.genecards.org/cgi-bin/carddisp.pl?gene=NONHSAG031883.2-002 |
| OA987689 | RNA Gene | 4 | GC21M041119 | 1.004889 | https://www.genecards.org/cgi-bin/carddisp.pl?gene=OA987689 |
| RF00017-1458 | RNA Gene | 4 | GC14P068292 | 1.004889 | https://www.genecards.org/cgi-bin/carddisp.pl?gene=RF00017-1458 |
| RF00017-3141 | RNA Gene | 4 | GC02P203756 | 1.004889 | https://www.genecards.org/cgi-bin/carddisp.pl?gene=RF00017-3141 |
| RF00017-3469 | RNA Gene | 4 | GC22M037214 | 1.004889 | https://www.genecards.org/cgi-bin/carddisp.pl?gene=RF00017-3469 |
| RF00017-4512 | RNA Gene | 4 | GC05P134091 | 1.004889 | https://www.genecards.org/cgi-bin/carddisp.pl?gene=RF00017-4512 |
| RF00017-4513 | RNA Gene | 4 | GC05P134110 | 1.004889 | https://www.genecards.org/cgi-bin/carddisp.pl?gene=RF00017-4513 |
| RF00017-4900 | RNA Gene | 4 | GC06P014082 | 1.004889 | https://www.genecards.org/cgi-bin/carddisp.pl?gene=RF00017-4900 |
| RF00017-5281 | RNA Gene | 4 | GC06M090261 | 1.004889 | https://www.genecards.org/cgi-bin/carddisp.pl?gene=RF00017-5281 |
| RF00017-5936 | RNA Gene | 4 | GC07M074515 | 1.004889 | https://www.genecards.org/cgi-bin/carddisp.pl?gene=RF00017-5936 |
| RF00017-5942 | RNA Gene | 4 | GC07M074617 | 1.004889 | https://www.genecards.org/cgi-bin/carddisp.pl?gene=RF00017-5942 |
| RF00017-6030 | RNA Gene | 4 | GC07P092606 | 1.004889 | https://www.genecards.org/cgi-bin/carddisp.pl?gene=RF00017-6030 |
| RF00017-621 | RNA Gene | 4 | GC10M070222 | 1.004889 | https://www.genecards.org/cgi- |

| | | | | | |
|---|---|---|---|---|---|
| | | | | | bin/carddisp.pl?gene=RF00017-621 |
| RF00017-6905 | RNA Gene | 4 | GC08M132150 | 1.004889 | https://www.genecards.org/cgi-bin/carddisp.pl?gene=RF00017-6905 |
| RF00017-7230 | RNA Gene | 4 | GC09M094978 | 1.004889 | https://www.genecards.org/cgi-bin/carddisp.pl?gene=RF00017-7230 |
| RF00019-027 | RNA Gene | 4 | GC13M039786 | 1.004889 | https://www.genecards.org/cgi-bin/carddisp.pl?gene=RF00019-027 |
| RF00026-971 | RNA Gene | 4 | GC07M028122 | 1.004889 | https://www.genecards.org/cgi-bin/carddisp.pl?gene=RF00026-971 |
| RF00951-116 | RNA Gene | 4 | GC04M026079 | 1.004889 | https://www.genecards.org/cgi-bin/carddisp.pl?gene=RF00951-116 |
| RF00994-491 | RNA Gene | 4 | GC02P070538 | 1.004889 | https://www.genecards.org/cgi-bin/carddisp.pl?gene=RF00994-491 |
| RF01016-017 | RNA Gene | 4 | GC07M092634 | 1.004889 | https://www.genecards.org/cgi-bin/carddisp.pl?gene=RF01016-017 |
| lnc-AHNAK2-1 | RNA Gene | 4 | GC14M104926 | 1.004889 | https://www.genecards.org/cgi-bin/carddisp.pl?gene=lnc-AHNAK2-1 |
| lnc-AHNAK2-2 | RNA Gene | 4 | GC14M104927 | 1.004889 | https://www.genecards.org/cgi-bin/carddisp.pl?gene=lnc-AHNAK2-2 |
| lnc-AHNAK2-3 | RNA Gene | 4 | GC14M104925 | 1.004889 | https://www.genecards.org/cgi-bin/carddisp.pl?gene=lnc-AHNAK2-3 |
| lnc-AHNAK2-4 | RNA Gene | 4 | GC14M104948 | 1.004889 | https://www.genecards.org/cgi-bin/carddisp.pl?gene=lnc-AHNAK2-4 |
| lnc-ARPC5-2 | RNA Gene | 4 | GC01M183560 | 1.004889 | https://www.genecards.org/cgi-bin/carddisp.pl?gene=lnc-ARPC5-2 |
| lnc-CALHM3-2 | RNA Gene | 4 | GC10M103642 | 1.004889 | https://www.genecards.org/cgi-bin/carddisp.pl?gene=lnc-CALHM3-2 |

| | | | | | |
|---|---|---|---|---|---|
| lnc-FCRL2-1 | RNA Gene | 4 | GC01M 157697 | 1.004 889 | https://www.genecards.org/cgi-bin/carddisp.pl?gene=lnc-FCRL2-1 |
| lnc-FCRL3-2 | RNA Gene | 4 | GC01M 157612 | 1.004 889 | https://www.genecards.org/cgi-bin/carddisp.pl?gene=lnc-FCRL3-2 |
| lnc-GATA3-19-002 | RNA Gene | 4 | GC10P 009002 | 1.004 889 | https://www.genecards.org/cgi-bin/carddisp.pl?gene=lnc-GATA3-19-002 |
| lnc-LONRF2-2 | RNA Gene | 4 | GC02M 100088 | 1.004 889 | https://www.genecards.org/cgi-bin/carddisp.pl?gene=lnc-LONRF2-2 |
| lnc-LONRF2-3 | RNA Gene | 4 | GC02M 100036 | 1.004 889 | https://www.genecards.org/cgi-bin/carddisp.pl?gene=lnc-LONRF2-3 |
| lnc-NUP88-2 | RNA Gene | 4 | GC17M 006180 | 1.004 889 | https://www.genecards.org/cgi-bin/carddisp.pl?gene=lnc-NUP88-2 |
| lnc-PSMD5-5 | RNA Gene | 4 | GC09M 120864 | 1.004 889 | https://www.genecards.org/cgi-bin/carddisp.pl?gene=lnc-PSMD5-5 |
| lnc-SLC37A1-2 | RNA Gene | 4 | GC21P 042439 | 1.004 889 | https://www.genecards.org/cgi-bin/carddisp.pl?gene=lnc-SLC37A1-2 |
| lnc-TMEM106C-4 | RNA Gene | 4 | GC12P 048036 | 1.004 889 | https://www.genecards.org/cgi-bin/carddisp.pl?gene=lnc-TMEM106C-4 |
| lnc-TNPO3-9 | RNA Gene | 4 | GC07M 128953 | 1.004 889 | https://www.genecards.org/cgi-bin/carddisp.pl?gene=lnc-TNPO3-9 |
| lnc-YRDC-3 | RNA Gene | 4 | GC01M 037827 | 1.004 889 | https://www.genecards.org/cgi-bin/carddisp.pl?gene=lnc-YRDC-3 |
| lnc-ZBTB7C-8 | RNA Gene | 4 | GC18M 048521 | 1.004 889 | https://www.genecards.org/cgi-bin/carddisp.pl?gene=lnc-ZBTB7C-8 |
| lnc-ZBTB7C-9 | RNA Gene | 4 | GC18M 048411 | 1.004 889 | https://www.genecards.org/cgi-bin/carddisp.pl?gene=lnc-ZBTB7C-9 |
| lnc-ZNF785-4 | RNA Gene | 4 | GC16M 037197 | 1.004 889 | https://www.genecards.org/cgi- |

| | | | | | |
|---|---|---|---|---|---|
| | | | | | bin/carddisp.pl?gene=lnc-ZNF785-4 |
| lnc-ZPBP2-1 | RNA Gene | 4 | GC17P039887 | 1.004889 | https://www.genecards.org/cgi-bin/carddisp.pl?gene=lnc-ZPBP2-1 |
| piR-30934 | RNA Gene | 4 | GC02P073990 | 1.004889 | https://www.genecards.org/cgi-bin/carddisp.pl?gene=piR-30934 |
| piR-31199-079 | RNA Gene | 4 | GC12P120338 | 1.004889 | https://www.genecards.org/cgi-bin/carddisp.pl?gene=piR-31199-079 |
| piR-31508-004 | RNA Gene | 4 | GC01P116736 | 1.004889 | https://www.genecards.org/cgi-bin/carddisp.pl?gene=piR-31508-004 |
| piR-32214-368 | RNA Gene | 4 | GC02M037712 | 1.004889 | https://www.genecards.org/cgi-bin/carddisp.pl?gene=piR-32214-368 |
| piR-32297-006 | RNA Gene | 4 | GC14P075506 | 1.004889 | https://www.genecards.org/cgi-bin/carddisp.pl?gene=piR-32297-006 |
| piR-32314-017 | RNA Gene | 4 | GC04P026097 | 1.004889 | https://www.genecards.org/cgi-bin/carddisp.pl?gene=piR-32314-017 |
| piR-32461-022 | RNA Gene | 4 | GC10M062677 | 1.004889 | https://www.genecards.org/cgi-bin/carddisp.pl?gene=piR-32461-022 |
| piR-33303-125 | RNA Gene | 4 | GC04M077625 | 1.004889 | https://www.genecards.org/cgi-bin/carddisp.pl?gene=piR-33303-125 |
| piR-34822-252 | RNA Gene | 4 | GC04M122090 | 1.004889 | https://www.genecards.org/cgi-bin/carddisp.pl?gene=piR-34822-252 |
| piR-36455 | RNA Gene | 4 | GC12M111444 | 1.004889 | https://www.genecards.org/cgi-bin/carddisp.pl?gene=piR-36455 |
| piR-37170-024 | RNA Gene | 4 | GC14P068303 | 1.004889 | https://www.genecards.org/cgi-bin/carddisp.pl?gene=piR-37170-024 |
| piR-38259 | RNA Gene | 4 | GC12M111535 | 1.004889 | https://www.genecards.org/cgi-bin/carddisp.pl?gene=piR-38259 |

| | | | | | |
|---|---|---|---|---|---|
| piR-38580-086 | RNA Gene | 4 | GC14M068290 | 1.004889 | https://www.genecards.org/cgi-bin/carddisp.pl?gene=piR-38580-086 |
| piR-39341-038 | RNA Gene | 4 | GC10P031110 | 1.004889 | https://www.genecards.org/cgi-bin/carddisp.pl?gene=piR-39341-038 |
| piR-39701-054 | RNA Gene | 4 | GC06M090275 | 1.004889 | https://www.genecards.org/cgi-bin/carddisp.pl?gene=piR-39701-054 |
| piR-39904-007 | RNA Gene | 4 | GC14M075433 | 1.004889 | https://www.genecards.org/cgi-bin/carddisp.pl?gene=piR-39904-007 |
| piR-40610 | RNA Gene | 4 | GC22P038498 | 1.004889 | https://www.genecards.org/cgi-bin/carddisp.pl?gene=piR-40610 |
| piR-41306-234 | RNA Gene | 4 | GC06P137911 | 1.004889 | https://www.genecards.org/cgi-bin/carddisp.pl?gene=piR-41306-234 |
| piR-48007 | RNA Gene | 4 | GC12M111540 | 1.004889 | https://www.genecards.org/cgi-bin/carddisp.pl?gene=piR-48007 |
| piR-48077 | RNA Gene | 4 | GC19P068867 | 1.004889 | https://www.genecards.org/cgi-bin/carddisp.pl?gene=piR-48077 |
| piR-48156 | RNA Gene | 4 | GC21M035356 | 1.004889 | https://www.genecards.org/cgi-bin/carddisp.pl?gene=piR-48156 |
| piR-48209-054 | RNA Gene | 4 | GC10P006468 | 1.004889 | https://www.genecards.org/cgi-bin/carddisp.pl?gene=piR-48209-054 |
| piR-48222-172 | RNA Gene | 4 | GC02P037723 | 1.004889 | https://www.genecards.org/cgi-bin/carddisp.pl?gene=piR-48222-172 |
| piR-48325-110 | RNA Gene | 4 | GC21M039092 | 1.004889 | https://www.genecards.org/cgi-bin/carddisp.pl?gene=piR-48325-110 |
| piR-49399-010 | RNA Gene | 4 | GC06P086729 | 1.004889 | https://www.genecards.org/cgi-bin/carddisp.pl?gene=piR-49399-010 |
| piR-50346 | RNA Gene | 4 | GC12M111541 | 1.004889 | https://www.genecards.org/cgi- |

| | | | | | |
|---|---|---|---|---|---|
| | | | | | bin/carddisp.pl?gene=piR-50346 |
| piR-50444-644 | RNA Gene | 4 | GC09M094818 | 1.004889 | https://www.genecards.org/cgi-bin/carddisp.pl?gene=piR-50444-644 |
| piR-51327 | RNA Gene | 4 | GC12M111437 | 1.004889 | https://www.genecards.org/cgi-bin/carddisp.pl?gene=piR-51327 |
| piR-51449 | RNA Gene | 4 | GC12P111448 | 1.004889 | https://www.genecards.org/cgi-bin/carddisp.pl?gene=piR-51449 |
| piR-52008-011 | RNA Gene | 4 | GC02M203881 | 1.004889 | https://www.genecards.org/cgi-bin/carddisp.pl?gene=piR-52008-011 |
| piR-52079-091 | RNA Gene | 4 | GC18M069859 | 1.004889 | https://www.genecards.org/cgi-bin/carddisp.pl?gene=piR-52079-091 |
| piR-52916-031 | RNA Gene | 4 | GC06M159081 | 1.004889 | https://www.genecards.org/cgi-bin/carddisp.pl?gene=piR-52916-031 |
| piR-53184 | RNA Gene | 4 | GC22P038660 | 1.004889 | https://www.genecards.org/cgi-bin/carddisp.pl?gene=piR-53184 |
| piR-54014 | RNA Gene | 4 | GC19P010470 | 1.004889 | https://www.genecards.org/cgi-bin/carddisp.pl?gene=piR-54014 |
| piR-55194-146 | RNA Gene | 4 | GC15P120150 | 1.004889 | https://www.genecards.org/cgi-bin/carddisp.pl?gene=piR-55194-146 |
| piR-56480-015 | RNA Gene | 4 | GC12M111439 | 1.004889 | https://www.genecards.org/cgi-bin/carddisp.pl?gene=piR-56480-015 |
| piR-56883-151 | RNA Gene | 4 | GC05P132118 | 1.004889 | https://www.genecards.org/cgi-bin/carddisp.pl?gene=piR-56883-151 |
| piR-57135-050 | RNA Gene | 4 | GC06P106162 | 1.004889 | https://www.genecards.org/cgi-bin/carddisp.pl?gene=piR-57135-050 |
| piR-57461-124 | RNA Gene | 4 | GC02M201198 | 1.004889 | https://www.genecards.org/cgi-bin/carddisp.pl?gene=piR-57461-124 |

| | | | | | | |
|---|---|---|---|---|---|---|
| piR-60051-103 | | RNA Gene | 4 | GC09P 123403 | 1.004 889 | https://www.genecards.org/cgi-bin/carddisp.pl?gene=piR-60051-103 |
| piR-61240-151 | | RNA Gene | 4 | GC02M 191093 | 1.004 889 | https://www.genecards.org/cgi-bin/carddisp.pl?gene=piR-61240-151 |
| ENSG00000289491 | Novel Protein | Protein Coding | 3 | GC16M 037479 | 1.004 889 | https://www.genecards.org/cgi-bin/carddisp.pl?gene=ENSG00000289491 |
| HE856162 | | RNA Gene | 3 | GC03P 017041 | 1.004 889 | https://www.genecards.org/cgi-bin/carddisp.pl?gene=HE856162 |
| HSALNG0001265 | | RNA Gene | 3 | GC01M 017316 | 1.004 889 | https://www.genecards.org/cgi-bin/carddisp.pl?gene=HSALNG0001265 |
| HSALNG0001266 | | RNA Gene | 3 | GC01M 018068 | 1.004 889 | https://www.genecards.org/cgi-bin/carddisp.pl?gene=HSALNG0001266 |
| HSALNG0001267 | | RNA Gene | 3 | GC01P 017351 | 1.004 889 | https://www.genecards.org/cgi-bin/carddisp.pl?gene=HSALNG0001267 |
| HSALNG0008511 | | RNA Gene | 3 | GC01P 173345 | 1.004 889 | https://www.genecards.org/cgi-bin/carddisp.pl?gene=HSALNG0008511 |
| HSALNG0026408 | | RNA Gene | 3 | GC03P 058194 | 1.004 889 | https://www.genecards.org/cgi-bin/carddisp.pl?gene=HSALNG0026408 |
| HSALNG0026409 | | RNA Gene | 3 | GC03P 058202 | 1.004 889 | https://www.genecards.org/cgi-bin/carddisp.pl?gene=HSALNG0026409 |
| HSALNG0086584 | | RNA Gene | 3 | GC11M 095569 | 1.004 889 | https://www.genecards.org/cgi-bin/carddisp.pl?gene=HSALNG0086584 |
| HSALNG0086587 | | RNA Gene | 3 | GC11M 095590 | 1.004 889 | https://www.genecards.org/cgi-bin/carddisp.pl?gene=HSALNG0086587 |
| HSALNG0121440 | | RNA Gene | 3 | GC18P 048493 | 1.004 889 | https://www.genecards.org/cgi-bin/carddisp.pl?gene=HSALNG0121440 |
| HSALNG0134045 | | RNA Gene | 3 | GC22M 019748 | 1.004 889 | https://www.genecards.org/cgi- |

| | | | | | | |
|---|---|---|---|---|---|---|
| | | | | | | bin/carddisp.pl?gene=HSALNG0134045 |
| LOC105374465 | Uncharacterized LOC105374465 | RNA Gene | 3 | GC02M037719 | 1.004889 | https://www.genecards.org/cgi-bin/carddisp.pl?gene=LOC105374465 |
| LOC105374939 | Uncharacterized LOC105374939 | RNA Gene | 3 | GC06P014098 | 1.004889 | https://www.genecards.org/cgi-bin/carddisp.pl?gene=LOC105374939 |
| LOC107986672 | Uncharacterized LOC107986672 | RNA Gene | 3 | GC06M167127 | 1.004889 | https://www.genecards.org/cgi-bin/carddisp.pl?gene=LOC107986672 |
| LOC124902927 | Uncharacterized LOC124902927 | RNA Gene | 3 | GC12M048022 | 1.004889 | https://www.genecards.org/cgi-bin/carddisp.pl?gene=LOC124902927 |
| LOC124903741 | Uncharacterized LOC124903741 | RNA Gene | 3 | GC16M085977 | 1.004889 | https://www.genecards.org/cgi-bin/carddisp.pl?gene=LOC124903741 |
| RF00017-4901 | | RNA Gene | 3 | GC06M014110 | 1.004889 | https://www.genecards.org/cgi-bin/carddisp.pl?gene=RF00017-4901 |
| lnc-PHYKPL-1 | | RNA Gene | 3 | GC05M178190 | 1.004889 | https://www.genecards.org/cgi-bin/carddisp.pl?gene=lnc-PHYKPL-1 |
| piR-31162-423 | | RNA Gene | 3 | GC06P085737 | 1.004889 | https://www.genecards.org/cgi-bin/carddisp.pl?gene=piR-31162-423 |
| piR-31432-579 | | RNA Gene | 3 | GC06P085771 | 1.004889 | https://www.genecards.org/cgi-bin/carddisp.pl?gene=piR-31432-579 |
| piR-31432-580 | | RNA Gene | 3 | GC06M032594 | 1.004889 | https://www.genecards.org/cgi-bin/carddisp.pl?gene=piR-31432-580 |
| piR-43099-508 | | RNA Gene | 3 | GC06P086515 | 1.004889 | https://www.genecards.org/cgi-bin/carddisp.pl?gene=piR-43099-508 |
| piR-43583-375 | | RNA Gene | 3 | GC03M020778 | 1.004889 | https://www.genecards.org/cgi-bin/carddisp.pl?gene=piR-43583-375 |
| piR-43583-471 | | RNA Gene | 3 | GC06P086540 | 1.004889 | https://www.genecards.org/cgi-bin/carddisp.pl?gene=piR-43583-471 |

| | | | | | | |
|---|---|---|---|---|---|---|
| piR-45471-521 | | RNA Gene | 3 | GC06M 032532 | 1.004 889 | https://www.genecards.org/cgi-bin/carddisp.pl?gene=piR-45471-521 |
| piR-46662-570 | | RNA Gene | 3 | GC06M 066009 | 1.004 889 | https://www.genecards.org/cgi-bin/carddisp.pl?gene=piR-46662-570 |
| piR-46756-002 | | RNA Gene | 3 | GC06M 032538 | 1.004 889 | https://www.genecards.org/cgi-bin/carddisp.pl?gene=piR-46756-002 |
| piR-48049-069 | | RNA Gene | 3 | GC06M 066004 | 1.004 889 | https://www.genecards.org/cgi-bin/carddisp.pl?gene=piR-48049-069 |
| piR-50308-545 | | RNA Gene | 3 | GC06P 086752 | 1.004 889 | https://www.genecards.org/cgi-bin/carddisp.pl?gene=piR-50308-545 |
| piR-53431-591 | | RNA Gene | 3 | GC06P 086858 | 1.004 889 | https://www.genecards.org/cgi-bin/carddisp.pl?gene=piR-53431-591 |
| piR-55072-002 | | RNA Gene | 3 | GC21P 044234 | 1.004 889 | https://www.genecards.org/cgi-bin/carddisp.pl?gene=piR-55072-002 |
| piR-55654-437 | | RNA Gene | 3 | GC06P 086927 | 1.004 889 | https://www.genecards.org/cgi-bin/carddisp.pl?gene=piR-55654-437 |
| piR-61028-492 | | RNA Gene | 3 | GC06M 066015 | 1.004 889 | https://www.genecards.org/cgi-bin/carddisp.pl?gene=piR-61028-492 |
| HSALNG0025953 | | RNA Gene | 2 | GC03M 049658 | 1.004 889 | https://www.genecards.org/cgi-bin/carddisp.pl?gene=HSALNG0025953 |
| LOC1053 71505 | Uncharacterized LOC105371505 | RNA Gene | 2 | GC17M 006179 | 1.004 889 | https://www.genecards.org/cgi-bin/carddisp.pl?gene=LOC105371505 |
| LOC1249 03678 | Uncharacterized LOC124903678 | RNA Gene | 2 | GC16P 041517 | 1.004 889 | https://www.genecards.org/cgi-bin/carddisp.pl?gene=LOC124903678 |
| hsa-miR-5095-132 | | RNA Gene | 2 | GC14M 054885 | 1.004 889 | https://www.genecards.org/cgi-bin/carddisp.pl?gene=hsa-miR-5095-132 |
| piR-49732-109 | | RNA Gene | 2 | GC12M 056617 | 1.004 889 | https://www.genecards.org/cgi- |

| | | | | | | |
|---|---|---|---|---|---|---|
| | | | | | | bin/carddisp.pl?gene=piR-49732-109 |
| P2RY12 | Purinergic Receptor P2Y12 | Protein Coding | 46 | GC03M151336 | 1.001303 | https://www.genecards.org/cgi-bin/carddisp.pl?gene=P2RY12 |
| P2RY1 | Purinergic Receptor P2Y1 | Protein Coding | 45 | GC03P152835 | 1.001303 | https://www.genecards.org/cgi-bin/carddisp.pl?gene=P2RY1 |
| P2RY2 | Purinergic Receptor P2Y2 | Protein Coding | 44 | GC11P073202 | 1.001303 | https://www.genecards.org/cgi-bin/carddisp.pl?gene=P2RY2 |
| P2RX1 | Purinergic Receptor P2X 1 | Protein Coding | 42 | GC17M003896 | 1.001303 | https://www.genecards.org/cgi-bin/carddisp.pl?gene=P2RX1 |
| P2RX2 | Purinergic Receptor P2X 2 | Protein Coding | 42 | GC12P132618 | 1.001303 | https://www.genecards.org/cgi-bin/carddisp.pl?gene=P2RX2 |
| P2RY6 | Pyrimidinergic Receptor P2Y6 | Protein Coding | 40 | GC11P073264 | 1.001303 | https://www.genecards.org/cgi-bin/carddisp.pl?gene=P2RY6 |
| P2RY4 | Pyrimidinergic Receptor P2Y4 | Protein Coding | 39 | GC0XM070258 | 1.001303 | https://www.genecards.org/cgi-bin/carddisp.pl?gene=P2RY4 |
| P2RX5 | Purinergic Receptor P2X 5 | Protein Coding | 38 | GC17M003672 | 1.001303 | https://www.genecards.org/cgi-bin/carddisp.pl?gene=P2RX5 |
| NOX5 | NADPH Oxidase 5 | Protein Coding | 37 | GC15P117544 | 1.001303 | https://www.genecards.org/cgi-bin/carddisp.pl?gene=NOX5 |
| HIVEP3 | HIVEP Zinc Finger 3 | Protein Coding | 35 | GC01M041506 | 1.000021 | https://www.genecards.org/cgi-bin/carddisp.pl?gene=HIVEP3 |
| SNHG1 | Small Nucleolar RNA Host Gene 1 | RNA Gene | 21 | GC11M089641 | 1.000021 | https://www.genecards.org/cgi-bin/carddisp.pl?gene=SNHG1 |
| GRIN2D | Glutamate Ionotropic Receptor NMDA Type Subunit 2D | Protein Coding | 47 | GC19P067036 | 0.998033 | https://www.genecards.org/cgi-bin/carddisp.pl?gene=GRIN2D |
| SCN9A | Sodium Voltage-Gated Channel Alpha Subunit 9 | Protein Coding | 47 | GC02M166195 | 0.998033 | https://www.genecards.org/cgi-bin/carddisp.pl?gene=SCN9A |

| | | | | | | |
|---|---|---|---|---|---|---|
| HTR3B | 5-Hydroxytryptamine Receptor 3B | Protein Coding | 44 | GC11P 113956 | 0.998 033 | https://www.genecards .org/cgi-bin/carddisp.pl?gene= HTR3B |
| PPME1 | Protein Phosphatase Methylesterase 1 | Protein Coding | 38 | GC11P 074170 | 0.998 033 | https://www.genecards .org/cgi-bin/carddisp.pl?gene= PPME1 |
| PPP2R5A | Protein Phosphatase 2 Regulatory Subunit B'Alpha | Protein Coding | 38 | GC01P 212285 | 0.998 033 | https://www.genecards .org/cgi-bin/carddisp.pl?gene= PPP2R5A |
| C5orf64 | Chromosome 5 Putative Open Reading Frame 64 | RNA Gene | 28 | GC05P 061640 | 0.998 033 | https://www.genecards .org/cgi-bin/carddisp.pl?gene= C5orf64 |
| SMIM23 | Small Integral Membrane Protein 23 | Protein Coding | 22 | GC05P 171785 | 0.998 033 | https://www.genecards .org/cgi-bin/carddisp.pl?gene= SMIM23 |
| TRIB1 | Tribbles Pseudokinase 1 | Protein Coding | 38 | GC08P 125430 | 0.993 718 | https://www.genecards .org/cgi-bin/carddisp.pl?gene= TRIB1 |
| TRIB2 | Tribbles Pseudokinase 2 | Protein Coding | 37 | GC02P 012717 | 0.993 718 | https://www.genecards .org/cgi-bin/carddisp.pl?gene= TRIB2 |
| STK32B | Serine/Threonine Kinase 32B | Protein Coding | 32 | GC04P 005020 | 0.988 998 | https://www.genecards .org/cgi-bin/carddisp.pl?gene= STK32B |
| PHB1 | Prohibitin 1 | Protein Coding | 36 | GC17M 049406 | 0.986 121 | https://www.genecards .org/cgi-bin/carddisp.pl?gene= PHB1 |
| JAG1 | Jagged Canonical Notch Ligand 1 | Protein Coding | 48 | GC20M 010637 | 0.982 082 | https://www.genecards .org/cgi-bin/carddisp.pl?gene=J AG1 |
| KAT5 | Lysine Acetyltransferase 5 | Protein Coding | 46 | GC11P 065711 | 0.982 082 | https://www.genecards .org/cgi-bin/carddisp.pl?gene= KAT5 |
| INSR | Insulin Receptor | Protein Coding | 51 | GC19M 007112 | 0.980 465 | https://www.genecards .org/cgi-bin/carddisp.pl?gene=I NSR |
| FSHR | Follicle Stimulating Hormone Receptor | Protein Coding | 46 | GC02M 048962 | 0.978 965 | https://www.genecards .org/cgi-bin/carddisp.pl?gene= FSHR |
| CDKN1B | Cyclin Dependent Kinase Inhibitor 1B | Protein Coding | 45 | GC12P 021339 | 0.978 965 | https://www.genecards .org/cgi- |

| | | | | | | |
|---|---|---|---|---|---|---|
| | | | | | | bin/carddisp.pl?gene=CDKN1B |
| ADAMTS3 | ADAM Metallopeptidase With Thrombospondin Type 1 Motif 3 | Protein Coding | 41 | GC04M072280 | 0.978965 | https://www.genecards.org/cgi-bin/carddisp.pl?gene=ADAMTS3 |
| SUCLG2 | Succinate-CoA Ligase GDP-Forming Subunit Beta | Protein Coding | 40 | GC03M067358 | 0.978965 | https://www.genecards.org/cgi-bin/carddisp.pl?gene=SUCLG2 |
| ABL1 | ABL Proto-Oncogene 1, Non-Receptor Tyrosine Kinase | Protein Coding | 50 | GC09P130713 | 0.969663 | https://www.genecards.org/cgi-bin/carddisp.pl?gene=ABL1 |
| ZNF300 | Zinc Finger Protein 300 | Protein Coding | 36 | GC05M150894 | 0.967315 | https://www.genecards.org/cgi-bin/carddisp.pl?gene=ZNF300 |
| SMARCA4 | SWI/SNF Related, Matrix Associated, Actin Dependent Regulator Of Chromatin, Subfamily A, Member 4 | Protein Coding | 48 | GC19P010932 | 0.963885 | https://www.genecards.org/cgi-bin/carddisp.pl?gene=SMARCA4 |
| ARHGEF1 | Rho Guanine Nucleotide Exchange Factor 1 | Protein Coding | 44 | GC19P041883 | 0.963885 | https://www.genecards.org/cgi-bin/carddisp.pl?gene=ARHGEF1 |
| PTPRK | Protein Tyrosine Phosphatase Receptor Type K | Protein Coding | 44 | GC06M127949 | 0.963885 | https://www.genecards.org/cgi-bin/carddisp.pl?gene=PTPRK |
| PARG | Poly(ADP-Ribose) Glycohydrolase | Protein Coding | 40 | GC10M049818 | 0.963885 | https://www.genecards.org/cgi-bin/carddisp.pl?gene=PARG |
| CCN5 | Cellular Communication Network Factor 5 | Protein Coding | 29 | GC20P044715 | 0.963885 | https://www.genecards.org/cgi-bin/carddisp.pl?gene=CCN5 |
| MIR19B1 | MicroRNA 19b-1 | RNA Gene | 18 | GC13P091551 | 0.963885 | https://www.genecards.org/cgi-bin/carddisp.pl?gene=MIR19B1 |
| MIR19B2 | MicroRNA 19b-2 | RNA Gene | 16 | GC0XM134310 | 0.963885 | https://www.genecards.org/cgi-bin/carddisp.pl?gene=MIR19B2 |
| PRKG1 | Protein Kinase CGMP-Dependent 1 | Protein Coding | 48 | GC10P050991 | 0.962127 | https://www.genecards.org/cgi-bin/carddisp.pl?gene=PRKG1 |
| EBP | EBP Cholestenol Delta-Isomerase | Protein Coding | 42 | GC0XP048521 | 0.958073 | https://www.genecards.org/cgi- |

| | | | | | | |
|---|---|---|---|---|---|---|
| | | | | | | bin/carddisp.pl?gene=EBP |
| EFS | Embryonal Fyn-Associated Substrate | Protein Coding | 35 | GC14M023356 | 0.957583 | https://www.genecards.org/cgi-bin/carddisp.pl?gene=EFS |
| PIK3CB | Phosphatidylinositol-4,5-Bisphosphate 3-Kinase Catalytic Subunit Beta | Protein Coding | 46 | GC03M138652 | 0.957089 | https://www.genecards.org/cgi-bin/carddisp.pl?gene=PIK3CB |
| UBE2N | Ubiquitin Conjugating Enzyme E2 N | Protein Coding | 45 | GC12M093406 | 0.956232 | https://www.genecards.org/cgi-bin/carddisp.pl?gene=UBE2N |
| DDB1 | Damage Specific DNA Binding Protein 1 | Protein Coding | 43 | GC11M089571 | 0.956232 | https://www.genecards.org/cgi-bin/carddisp.pl?gene=DDB1 |
| MIR499B | MicroRNA 499b | RNA Gene | 10 | GC20M034990 | 0.952944 | https://www.genecards.org/cgi-bin/carddisp.pl?gene=MIR499B |
| KCNA3 | Potassium Voltage-Gated Channel Subfamily A Member 3 | Protein Coding | 43 | GC01M110654 | 0.949855 | https://www.genecards.org/cgi-bin/carddisp.pl?gene=KCNA3 |
| G6PD | Glucose-6-Phosphate Dehydrogenase | Protein Coding | 48 | GC0XM154551 | 0.947585 | https://www.genecards.org/cgi-bin/carddisp.pl?gene=G6PD |
| CADM1 | Cell Adhesion Molecule 1 | Protein Coding | 42 | GC11M115169 | 0.942037 | https://www.genecards.org/cgi-bin/carddisp.pl?gene=CADM1 |
| SAGE1 | Sarcoma Antigen 1 | Protein Coding | 29 | GC0XP135889 | 0.942037 | https://www.genecards.org/cgi-bin/carddisp.pl?gene=SAGE1 |
| IGHG3 | Immunoglobulin Heavy Constant Gamma 3 (G3m Marker) | Protein Coding | 29 | GC14M112714 | 0.938649 | https://www.genecards.org/cgi-bin/carddisp.pl?gene=IGHG3 |
| PTGIR | Prostaglandin I2 Receptor | Protein Coding | 45 | GC19M065976 | 0.938271 | https://www.genecards.org/cgi-bin/carddisp.pl?gene=PTGIR |
| RYR2 | Ryanodine Receptor 2 | Protein Coding | 46 | GC01P237042 | 0.935749 | https://www.genecards.org/cgi-bin/carddisp.pl?gene=RYR2 |
| HUNK | Hormonally Up-Regulated Neu-Associated Kinase | Protein Coding | 36 | GC21P031873 | 0.935749 | https://www.genecards.org/cgi-bin/carddisp.pl?gene=HUNK |

| | | | | | | |
|---|---|---|---|---|---|---|
| CYP51A1 | Cytochrome P450 Family 51 Subfamily A Member 1 | Protein Coding | 42 | GC07M 092112 | 0.933 388 | https://www.genecards .org/cgi-bin/carddisp.pl?gene= CYP51A1 |
| CNN1 | Calponin 1 | Protein Coding | 39 | GC19P 011539 | 0.933 388 | https://www.genecards .org/cgi-bin/carddisp.pl?gene= CNN1 |
| SRSF6 | Serine And Arginine Rich Splicing Factor 6 | Protein Coding | 38 | GC20P 043457 | 0.929 52 | https://www.genecards .org/cgi-bin/carddisp.pl?gene= SRSF6 |
| SIRPA | Signal Regulatory Protein Alpha | Protein Coding | 42 | GC20P 001894 | 0.928 178 | https://www.genecards .org/cgi-bin/carddisp.pl?gene= SIRPA |
| NR5A1 | Nuclear Receptor Subfamily 5 Group A Member 1 | Protein Coding | 48 | GC09M 124481 | 0.923 375 | https://www.genecards .org/cgi-bin/carddisp.pl?gene= NR5A1 |
| MIR200C | MicroRNA 200c | RNA Gene | 21 | GC12P 021094 | 0.922 258 | https://www.genecards .org/cgi-bin/carddisp.pl?gene= MIR200C |
| CYSLTR 1 | Cysteinyl Leukotriene Receptor 1 | Protein Coding | 41 | GC0X M0782 71 | 0.919 425 | https://www.genecards .org/cgi-bin/carddisp.pl?gene= CYSLTR1 |
| COMMD 1 | Copper Metabolism Domain Containing 1 | Protein Coding | 41 | GC02P 061888 | 0.918 814 | https://www.genecards .org/cgi-bin/carddisp.pl?gene= COMMD1 |
| WNT3A | Wnt Family Member 3A | Protein Coding | 45 | GC01P 229293 | 0.916 329 | https://www.genecards .org/cgi-bin/carddisp.pl?gene= WNT3A |
| GAD1 | Glutamate Decarboxylase 1 | Protein Coding | 48 | GC02P 170813 | 0.916 112 | https://www.genecards .org/cgi-bin/carddisp.pl?gene= GAD1 |
| SLC22A2 | Solute Carrier Family 22 Member 2 | Protein Coding | 44 | GC06M 160190 | 0.909 56 | https://www.genecards .org/cgi-bin/carddisp.pl?gene= SLC22A2 |
| DBP | D-Box Binding PAR BZIP Transcription Factor | Protein Coding | 37 | GC19M 048630 | 0.907 883 | https://www.genecards .org/cgi-bin/carddisp.pl?gene= DBP |
| MAZ | MYC Associated Zinc Finger Protein | Protein Coding | 39 | GC16P 029806 | 0.905 957 | https://www.genecards .org/cgi-bin/carddisp.pl?gene= MAZ |
| ADPRH | ADP-Ribosylarginine Hydrolase | Protein Coding | 34 | GC03P 119579 | 0.900 55 | https://www.genecards .org/cgi- |

| | | | | | | |
|---|---|---|---|---|---|---|
| | | | | | | bin/carddisp.pl?gene=ADPRH |
| TP53BP1 | Tumor Protein P53 Binding Protein 1 | Protein Coding | 42 | GC15M043403 | 0.895364 | https://www.genecards.org/cgi-bin/carddisp.pl?gene=TP53BP1 |
| TRAF4 | TNF Receptor Associated Factor 4 | Protein Coding | 41 | GC17P054910 | 0.894301 | https://www.genecards.org/cgi-bin/carddisp.pl?gene=TRAF4 |
| SMOC2 | SPARC Related Modular Calcium Binding 2 | Protein Coding | 42 | GC06P168441 | 0.893829 | https://www.genecards.org/cgi-bin/carddisp.pl?gene=SMOC2 |
| UBC | Ubiquitin C | Protein Coding | 41 | GC12M124911 | 0.893829 | https://www.genecards.org/cgi-bin/carddisp.pl?gene=UBC |
| ATP8B1 | ATPase Phospholipid Transporting 8B1 | Protein Coding | 40 | GC18M057646 | 0.893829 | https://www.genecards.org/cgi-bin/carddisp.pl?gene=ATP8B1 |
| FMN2 | Formin 2 | Protein Coding | 38 | GC01P240014 | 0.893829 | https://www.genecards.org/cgi-bin/carddisp.pl?gene=FMN2 |
| KCNK12 | Potassium Two Pore Domain Channel Subfamily K Member 12 | Protein Coding | 38 | GC02M047516 | 0.893829 | https://www.genecards.org/cgi-bin/carddisp.pl?gene=KCNK12 |
| ZP4 | Zona Pellucida Glycoprotein 4 | Protein Coding | 32 | GC01M237877 | 0.893829 | https://www.genecards.org/cgi-bin/carddisp.pl?gene=ZP4 |
| SREBF1 | Sterol Regulatory Element Binding Transcription Factor 1 | Protein Coding | 44 | GC17M017810 | 0.891944 | https://www.genecards.org/cgi-bin/carddisp.pl?gene=SREBF1 |
| CXCL14 | C-X-C Motif Chemokine Ligand 14 | Protein Coding | 36 | GC05M135617 | 0.890761 | https://www.genecards.org/cgi-bin/carddisp.pl?gene=CXCL14 |
| ACE2 | Angiotensin Converting Enzyme 2 | Protein Coding | 46 | GC0XM015494 | 0.884451 | https://www.genecards.org/cgi-bin/carddisp.pl?gene=ACE2 |
| EPX | Eosinophil Peroxidase | Protein Coding | 43 | GC17P058192 | 0.883289 | https://www.genecards.org/cgi-bin/carddisp.pl?gene=EPX |
| PNPLA6 | Patatin Like Phospholipase Domain Containing 6 | Protein Coding | 43 | GC19P007534 | 0.883289 | https://www.genecards.org/cgi-bin/carddisp.pl?gene=PNPLA6 |

| | | | | | | |
|---|---|---|---|---|---|---|
| FARSB | Phenylalanyl-TRNA Synthetase Subunit Beta | Protein Coding | 42 | GC02M 222570 | 0.883 289 | https://www.genecards .org/cgi-bin/carddisp.pl?gene= FARSB |
| TOLLIP | Toll Interacting Protein | Protein Coding | 42 | GC11M 001274 | 0.883 289 | https://www.genecards .org/cgi-bin/carddisp.pl?gene= TOLLIP |
| PSIP1 | PC4 And SFRS1 Interacting Protein 1 | Protein Coding | 38 | GC09M 015464 | 0.883 289 | https://www.genecards .org/cgi-bin/carddisp.pl?gene= PSIP1 |
| FOXP2 | Forkhead Box P2 | Protein Coding | 43 | GC07P 114086 | 0.882 679 | https://www.genecards .org/cgi-bin/carddisp.pl?gene= FOXP2 |
| EMD | Emerin | Protein Coding | 42 | GC0XP 154379 | 0.882 679 | https://www.genecards .org/cgi-bin/carddisp.pl?gene= EMD |
| MUC5AC | Mucin 5AC, Oligomeric Mucus/Gel-Forming | Protein Coding | 40 | GC11P 001810 | 0.880 126 | https://www.genecards .org/cgi-bin/carddisp.pl?gene= MUC5AC |
| ILK | Integrin Linked Kinase | Protein Coding | 44 | GC11P 006604 | 0.876 646 | https://www.genecards .org/cgi-bin/carddisp.pl?gene=I LK |
| P4HTM | Prolyl 4-Hydroxylase, Transmembrane | Protein Coding | 36 | GC03P 049627 | 0.876 646 | https://www.genecards .org/cgi-bin/carddisp.pl?gene= P4HTM |
| FUT7 | Fucosyltransferase 7 | Protein Coding | 40 | GC09M 137030 | 0.866 573 | https://www.genecards .org/cgi-bin/carddisp.pl?gene= FUT7 |
| DUSP7 | Dual Specificity Phosphatase 7 | Protein Coding | 38 | GC03M 052048 | 0.865 589 | https://www.genecards .org/cgi-bin/carddisp.pl?gene= DUSP7 |
| CYP24A1 | Cytochrome P450 Family 24 Subfamily A Member 1 | Protein Coding | 45 | GC20M 054153 | 0.855 808 | https://www.genecards .org/cgi-bin/carddisp.pl?gene= CYP24A1 |
| NR3C2 | Nuclear Receptor Subfamily 3 Group C Member 2 | Protein Coding | 45 | GC04M 148078 | 0.855 808 | https://www.genecards .org/cgi-bin/carddisp.pl?gene= NR3C2 |
| SRD5A1 | Steroid 5 Alpha-Reductase 1 | Protein Coding | 42 | GC05P 006633 | 0.855 808 | https://www.genecards .org/cgi-bin/carddisp.pl?gene= SRD5A1 |
| VAV3 | Vav Guanine Nucleotide Exchange Factor 3 | Protein Coding | 42 | GC01M 107571 | 0.855 808 | https://www.genecards .org/cgi- |

| | | | | | | |
|---|---|---|---|---|---|---|
| | | | | | | bin/carddisp.pl?gene=VAV3 |
| TBXT | T-Box Transcription Factor T | Protein Coding | 33 | GC06M166158 | 0.851548 | https://www.genecards.org/cgi-bin/carddisp.pl?gene=TBXT |
| PADI1 | Peptidyl Arginine Deiminase 1 | Protein Coding | 37 | GC01P017205 | 0.850721 | https://www.genecards.org/cgi-bin/carddisp.pl?gene=PADI1 |
| PRKRA | Protein Activator Of Interferon Induced Protein Kinase EIF2AK2 | Protein Coding | 40 | GC02M178431 | 0.848315 | https://www.genecards.org/cgi-bin/carddisp.pl?gene=PRKRA |
| CA1 | Carbonic Anhydrase 1 | Protein Coding | 46 | GC08M085327 | 0.846249 | https://www.genecards.org/cgi-bin/carddisp.pl?gene=CA1 |
| SERPING1 | Serpin Family G Member 1 | Protein Coding | 45 | GC11P057597 | 0.846249 | https://www.genecards.org/cgi-bin/carddisp.pl?gene=SERPING1 |
| COTL1 | Coactosin Like F-Actin Binding Protein 1 | Protein Coding | 40 | GC16M084566 | 0.842726 | https://www.genecards.org/cgi-bin/carddisp.pl?gene=COTL1 |
| CORT | Cortistatin | Protein Coding | 35 | GC01P010508 | 0.837322 | https://www.genecards.org/cgi-bin/carddisp.pl?gene=CORT |
| GCGR | Glucagon Receptor | Protein Coding | 45 | GC17P081804 | 0.836011 | https://www.genecards.org/cgi-bin/carddisp.pl?gene=GCGR |
| TXNRD2 | Thioredoxin Reductase 2 | Protein Coding | 45 | GC22M019863 | 0.835236 | https://www.genecards.org/cgi-bin/carddisp.pl?gene=TXNRD2 |
| CDR1 | Cerebellar Degeneration Related Protein 1 | Protein Coding | 32 | GC0XM140782 | 0.835019 | https://www.genecards.org/cgi-bin/carddisp.pl?gene=CDR1 |
| CARD11 | Caspase Recruitment Domain Family Member 11 | Protein Coding | 45 | GC07M002906 | 0.828873 | https://www.genecards.org/cgi-bin/carddisp.pl?gene=CARD11 |
| CPE | Carboxypeptidase E | Protein Coding | 44 | GC04P165361 | 0.828873 | https://www.genecards.org/cgi-bin/carddisp.pl?gene=CPE |
| PDE5A | Phosphodiesterase 5A | Protein Coding | 44 | GC04M119494 | 0.828873 | https://www.genecards.org/cgi-bin/carddisp.pl?gene=PDE5A |

| | | | | | | |
|---|---|---|---|---|---|---|
| APOA2 | Apolipoprotein A2 | Protein Coding | 43 | GC01M 161222 | 0.828 873 | https://www.genecards .org/cgi-bin/carddisp.pl?gene= APOA2 |
| IL31RA | Interleukin 31 Receptor A | Protein Coding | 42 | GC05P 055840 | 0.828 873 | https://www.genecards .org/cgi-bin/carddisp.pl?gene=I L31RA |
| INSL3 | Insulin Like 3 | Protein Coding | 39 | GC19M 017816 | 0.828 873 | https://www.genecards .org/cgi-bin/carddisp.pl?gene=I NSL3 |
| PHF11 | PHD Finger Protein 11 | Protein Coding | 35 | GC13P 049495 | 0.828 873 | https://www.genecards .org/cgi-bin/carddisp.pl?gene= PHF11 |
| TMEM79 | Transmembrane Protein 79 | Protein Coding | 34 | GC01P 156252 | 0.828 873 | https://www.genecards .org/cgi-bin/carddisp.pl?gene= TMEM79 |
| SLC1A1 | Solute Carrier Family 1 Member 1 | Protein Coding | 47 | GC09P 004490 | 0.828 172 | https://www.genecards .org/cgi-bin/carddisp.pl?gene= SLC1A1 |
| ARG2 | Arginase 2 | Protein Coding | 43 | GC14P 067619 | 0.828 172 | https://www.genecards .org/cgi-bin/carddisp.pl?gene= ARG2 |
| SH3BP2 | SH3 Domain Binding Protein 2 | Protein Coding | 40 | GC04P 002794 | 0.828 172 | https://www.genecards .org/cgi-bin/carddisp.pl?gene= SH3BP2 |
| MAPK11 | Mitogen-Activated Protein Kinase 11 | Protein Coding | 45 | GC22M 050263 | 0.817 537 | https://www.genecards .org/cgi-bin/carddisp.pl?gene= MAPK11 |
| MAPK12 | Mitogen-Activated Protein Kinase 12 | Protein Coding | 45 | GC22M 057729 | 0.817 537 | https://www.genecards .org/cgi-bin/carddisp.pl?gene= MAPK12 |
| HNRNPD | Heterogeneous Nuclear Ribonucleoprotein D | Protein Coding | 41 | GC04M 082352 | 0.817 537 | https://www.genecards .org/cgi-bin/carddisp.pl?gene= HNRNPD |
| SPPL2A | Signal Peptide Peptidase Like 2A | Protein Coding | 40 | GC15M 050702 | 0.800 444 | https://www.genecards .org/cgi-bin/carddisp.pl?gene= SPPL2A |
| PSMC6 | Proteasome 26S Subunit, ATPase 6 | Protein Coding | 39 | GC14P 052707 | 0.800 444 | https://www.genecards .org/cgi-bin/carddisp.pl?gene= PSMC6 |
| MAGI1 | Membrane Associated Guanylate Kinase, WW | Protein Coding | 38 | GC03M 065330 | 0.800 444 | https://www.genecards .org/cgi- |

| | | | | | | |
|---|---|---|---|---|---|---|
| | And PDZ Domain Containing 1 | | | | | bin/carddisp.pl?gene=MAGI1 |
| ZNF395 | Zinc Finger Protein 395 | Protein Coding | 32 | GC08M028345 | 0.800444 | https://www.genecards.org/cgi-bin/carddisp.pl?gene=ZNF395 |
| NCSTN | Nicastrin | Protein Coding | 45 | GC01P160343 | 0.7991 | https://www.genecards.org/cgi-bin/carddisp.pl?gene=NCSTN |
| UGT2B17 | UDP Glucuronosyltransferase Family 2 Member B17 | Protein Coding | 41 | GC04M068537 | 0.7991 | https://www.genecards.org/cgi-bin/carddisp.pl?gene=UGT2B17 |
| TRIM5 | Tripartite Motif Containing 5 | Protein Coding | 40 | GC11M006531 | 0.7991 | https://www.genecards.org/cgi-bin/carddisp.pl?gene=TRIM5 |
| FIP1L1 | Factor Interacting With PAPOLA And CPSF1 | Protein Coding | 39 | GC04P053427 | 0.7991 | https://www.genecards.org/cgi-bin/carddisp.pl?gene=FIP1L1 |
| SBDS | SBDS Ribosome Maturation Factor | Protein Coding | 41 | GC07M066987 | 0.792955 | https://www.genecards.org/cgi-bin/carddisp.pl?gene=SBDS |
| PTGDR2 | Prostaglandin D2 Receptor 2 | Protein Coding | 40 | GC11M060850 | 0.792955 | https://www.genecards.org/cgi-bin/carddisp.pl?gene=PTGDR2 |
| KRT9 | Keratin 9 | Protein Coding | 41 | GC17M041565 | 0.792552 | https://www.genecards.org/cgi-bin/carddisp.pl?gene=KRT9 |
| SLC9A3 | Solute Carrier Family 9 Member A3 | Protein Coding | 46 | GC05M000472 | 0.789809 | https://www.genecards.org/cgi-bin/carddisp.pl?gene=SLC9A3 |
| HMGCS2 | 3-Hydroxy-3-Methylglutaryl-CoA Synthase 2 | Protein Coding | 45 | GC01M119747 | 0.789809 | https://www.genecards.org/cgi-bin/carddisp.pl?gene=HMGCS2 |
| PRCP | Prolylcarboxypeptidase | Protein Coding | 45 | GC11M090534 | 0.789809 | https://www.genecards.org/cgi-bin/carddisp.pl?gene=PRCP |
| ABCA7 | ATP Binding Cassette Subfamily A Member 7 | Protein Coding | 44 | GC19P002825 | 0.789809 | https://www.genecards.org/cgi-bin/carddisp.pl?gene=ABCA7 |
| EFEMP2 | EGF Containing Fibulin Extracellular Matrix Protein 2 | Protein Coding | 44 | GC11M089827 | 0.789809 | https://www.genecards.org/cgi-bin/carddisp.pl?gene=EFEMP2 |

| | | | | | | |
|---|---|---|---|---|---|---|
| NPPA | Natriuretic Peptide A | Protein Coding | 44 | GC01M 011846 | 0.789 809 | https://www.genecards.org/cgi-bin/carddisp.pl?gene=NPPA |
| S1PR3 | Sphingosine-1-Phosphate Receptor 3 | Protein Coding | 43 | GC09P 089316 | 0.789 809 | https://www.genecards.org/cgi-bin/carddisp.pl?gene=S1PR3 |
| PALB2 | Partner And Localizer Of BRCA2 | Protein Coding | 42 | GC16M 023603 | 0.789 809 | https://www.genecards.org/cgi-bin/carddisp.pl?gene=PALB2 |
| PLSCR1 | Phospholipid Scramblase 1 | Protein Coding | 42 | GC03M 146515 | 0.789 809 | https://www.genecards.org/cgi-bin/carddisp.pl?gene=PLSCR1 |
| S1PR2 | Sphingosine-1-Phosphate Receptor 2 | Protein Coding | 42 | GC19M 010223 | 0.789 809 | https://www.genecards.org/cgi-bin/carddisp.pl?gene=S1PR2 |
| NDUFAB 1 | NADH:Ubiquinone Oxidoreductase Subunit AB1 | Protein Coding | 41 | GC16M 023582 | 0.789 809 | https://www.genecards.org/cgi-bin/carddisp.pl?gene=NDUFAB1 |
| BNIP3 | BCL2 Interacting Protein 3 | Protein Coding | 40 | GC10M 131966 | 0.789 809 | https://www.genecards.org/cgi-bin/carddisp.pl?gene=BNIP3 |
| CLEC1B | C-Type Lectin Domain Family 1 Member B | Protein Coding | 38 | GC12M 021103 | 0.789 809 | https://www.genecards.org/cgi-bin/carddisp.pl?gene=CLEC1B |
| KIR2DL4 | Killer Cell Immunoglobulin Like Receptor, Two Ig Domains And Long Cytoplasmic Tail 4 | Protein Coding | 38 | GC19P 067383 | 0.789 809 | https://www.genecards.org/cgi-bin/carddisp.pl?gene=KIR2DL4 |
| SEZ6L | Seizure Related 6 Homolog Like | Protein Coding | 38 | GC22P 026169 | 0.789 809 | https://www.genecards.org/cgi-bin/carddisp.pl?gene=SEZ6L |
| ABLIM1 | Actin Binding LIM Protein 1 | Protein Coding | 37 | GC10M 114487 | 0.789 809 | https://www.genecards.org/cgi-bin/carddisp.pl?gene=ABLIM1 |
| FBXO38 | F-Box Protein 38 | Protein Coding | 37 | GC05P 148383 | 0.789 809 | https://www.genecards.org/cgi-bin/carddisp.pl?gene=FBXO38 |
| PSRC1 | Proline And Serine Rich Coiled-Coil 1 | Protein Coding | 37 | GC01M 109279 | 0.789 809 | https://www.genecards.org/cgi-bin/carddisp.pl?gene=PSRC1 |

| | | | | | | |
|---|---|---|---|---|---|---|
| DCTN5 | Dynactin Subunit 5 | Protein Coding | 36 | GC16P 024282 | 0.789 809 | https://www.genecards .org/cgi- bin/carddisp.pl?gene= DCTN5 |
| LAIR2 | Leukocyte Associated Immunoglobulin Like Receptor 2 | Protein Coding | 36 | GC19P 067370 | 0.789 809 | https://www.genecards .org/cgi- bin/carddisp.pl?gene= LAIR2 |
| ZFAND6 | Zinc Finger AN1-Type Containing 6 | Protein Coding | 36 | GC15P 117829 | 0.789 809 | https://www.genecards .org/cgi- bin/carddisp.pl?gene= ZFAND6 |
| ZDHHC1 | Zinc Finger DHHC-Type Containing 1 | Protein Coding | 35 | GC16M 067394 | 0.789 809 | https://www.genecards .org/cgi- bin/carddisp.pl?gene= ZDHHC1 |
| ZGPAT | Zinc Finger CCCH-Type And G-Patch Domain Containing | Protein Coding | 35 | GC20P 063707 | 0.789 809 | https://www.genecards .org/cgi- bin/carddisp.pl?gene= ZGPAT |
| KLHDC1 | Kelch Domain Containing 1 | Protein Coding | 34 | GC14P 049693 | 0.789 809 | https://www.genecards .org/cgi- bin/carddisp.pl?gene= KLHDC1 |
| NDST3 | N-Deacetylase And N-Sulfotransferase 3 | Protein Coding | 34 | GC04P 118033 | 0.789 809 | https://www.genecards .org/cgi- bin/carddisp.pl?gene= NDST3 |
| TRAFD1 | TRAF-Type Zinc Finger Domain Containing 1 | Protein Coding | 34 | GC12P 112125 | 0.789 809 | https://www.genecards .org/cgi- bin/carddisp.pl?gene= TRAFD1 |
| FAM135 A | Family With Sequence Similarity 135 Member A | Protein Coding | 33 | GC06P 070412 | 0.789 809 | https://www.genecards .org/cgi- bin/carddisp.pl?gene= FAM135A |
| HIGD1C | HIG1 Hypoxia Inducible Domain Family Member 1C | Protein Coding | 28 | GC12P 050932 | 0.789 809 | https://www.genecards .org/cgi- bin/carddisp.pl?gene= HIGD1C |
| SLC35F4 | Solute Carrier Family 35 Member F4 | Protein Coding | 27 | GC14M 057563 | 0.789 809 | https://www.genecards .org/cgi- bin/carddisp.pl?gene= SLC35F4 |
| C3AR1 | Complement C3a Receptor 1 | Protein Coding | 41 | GC12M 008058 | 0.783 075 | https://www.genecards .org/cgi- bin/carddisp.pl?gene= C3AR1 |
| SLC3A2 | Solute Carrier Family 3 Member 2 | Protein Coding | 40 | GC11P 062856 | 0.782 247 | https://www.genecards .org/cgi- bin/carddisp.pl?gene= SLC3A2 |
| ITIH4 | Inter-Alpha-Trypsin Inhibitor Heavy Chain 4 | Protein Coding | 42 | GC03M 052812 | 0.781 451 | https://www.genecards .org/cgi- |

| | | | | | | |
|---|---|---|---|---|---|---|
| | | | | | | bin/carddisp.pl?gene=ITIH4 |
| FABP5 | Fatty Acid Binding Protein 5 | Protein Coding | 41 | GC08P081282 | 0.77223 | https://www.genecards.org/cgi-bin/carddisp.pl?gene=FABP5 |
| TNFSF12-TNFSF13 | TNFSF12-TNFSF13 Readthrough | Protein Coding | 23 | GC17P011168 | 0.769389 | https://www.genecards.org/cgi-bin/carddisp.pl?gene=TNFSF12-TNFSF13 |
| SLC26A4 | Solute Carrier Family 26 Member 4 | Protein Coding | 42 | GC07P107660 | 0.766114 | https://www.genecards.org/cgi-bin/carddisp.pl?gene=SLC26A4 |
| COL4A3 | Collagen Type IV Alpha 3 Chain | Protein Coding | 44 | GC02P227164 | 0.766052 | https://www.genecards.org/cgi-bin/carddisp.pl?gene=COL4A3 |
| LMNB2 | Lamin B2 | Protein Coding | 42 | GC19M005364 | 0.766052 | https://www.genecards.org/cgi-bin/carddisp.pl?gene=LMNB2 |
| NAA80 | N-Alpha-Acetyltransferase 80, NatH Catalytic Subunit | Protein Coding | 25 | GC03M051240 | 0.764308 | https://www.genecards.org/cgi-bin/carddisp.pl?gene=NAA80 |
| PTGES2 | Prostaglandin E Synthase 2 | Protein Coding | 41 | GC09M128120 | 0.761813 | https://www.genecards.org/cgi-bin/carddisp.pl?gene=PTGES2 |
| SPR | Sepiapterin Reductase | Protein Coding | 49 | GC02P072850 | 0.761076 | https://www.genecards.org/cgi-bin/carddisp.pl?gene=SPR |
| TSC2 | TSC Complex Subunit 2 | Protein Coding | 48 | GC16P011696 | 0.761076 | https://www.genecards.org/cgi-bin/carddisp.pl?gene=TSC2 |
| ATP1A3 | ATPase Na+/K+ Transporting Subunit Alpha 3 | Protein Coding | 46 | GC19M041966 | 0.761076 | https://www.genecards.org/cgi-bin/carddisp.pl?gene=ATP1A3 |
| LIG4 | DNA Ligase 4 | Protein Coding | 46 | GC13M108207 | 0.761076 | https://www.genecards.org/cgi-bin/carddisp.pl?gene=LIG4 |
| NFS1 | NFS1 Cysteine Desulfurase | Protein Coding | 45 | GC20M035668 | 0.761076 | https://www.genecards.org/cgi-bin/carddisp.pl?gene=NFS1 |
| AGXT | Alanine--Glyoxylate And Serine--Pyruvate Aminotransferase | Protein Coding | 44 | GC02P240868 | 0.761076 | https://www.genecards.org/cgi-bin/carddisp.pl?gene=AGXT |

| | | | | | | |
|---|---|---|---|---|---|---|
| SLC7A9 | Solute Carrier Family 7 Member 9 | Protein Coding | 44 | GC19M 032830 | 0.761 076 | https://www.genecards .org/cgi-bin/carddisp.pl?gene= SLC7A9 |
| TAF1 | TATA-Box Binding Protein Associated Factor 1 | Protein Coding | 44 | GC0XP 071366 | 0.761 076 | https://www.genecards .org/cgi-bin/carddisp.pl?gene= TAF1 |
| CHRNA1 | Cholinergic Receptor Nicotinic Alpha 1 Subunit | Protein Coding | 43 | GC02M 174747 | 0.761 076 | https://www.genecards .org/cgi-bin/carddisp.pl?gene= CHRNA1 |
| CHRNE | Cholinergic Receptor Nicotinic Epsilon Subunit | Protein Coding | 43 | GC17M 004897 | 0.761 076 | https://www.genecards .org/cgi-bin/carddisp.pl?gene= CHRNE |
| C7 | Complement C7 | Protein Coding | 42 | GC05P 040909 | 0.761 076 | https://www.genecards .org/cgi-bin/carddisp.pl?gene= C7 |
| PNKD | PNKD Metallo-Beta-Lactamase Domain Containing | Protein Coding | 42 | GC02P 218270 | 0.761 076 | https://www.genecards .org/cgi-bin/carddisp.pl?gene= PNKD |
| SGCE | Sarcoglycan Epsilon | Protein Coding | 42 | GC07M 094524 | 0.761 076 | https://www.genecards .org/cgi-bin/carddisp.pl?gene= SGCE |
| RAPSN | Receptor Associated Protein Of The Synapse | Protein Coding | 41 | GC11M 089370 | 0.761 076 | https://www.genecards .org/cgi-bin/carddisp.pl?gene= RAPSN |
| AKAP12 | A-Kinase Anchoring Protein 12 | Protein Coding | 40 | GC06P 151239 | 0.761 076 | https://www.genecards .org/cgi-bin/carddisp.pl?gene= AKAP12 |
| NHEJ1 | Non-Homologous End Joining Factor 1 | Protein Coding | 40 | GC02M 219119 | 0.761 076 | https://www.genecards .org/cgi-bin/carddisp.pl?gene= NHEJ1 |
| CALB2 | Calbindin 2 | Protein Coding | 39 | GC16P 071358 | 0.761 076 | https://www.genecards .org/cgi-bin/carddisp.pl?gene= CALB2 |
| PTMA | Prothymosin Alpha | Protein Coding | 39 | GC02P 231707 | 0.761 076 | https://www.genecards .org/cgi-bin/carddisp.pl?gene= PTMA |
| PRRT2 | Proline Rich Transmembrane Protein 2 | Protein Coding | 38 | GC16P 029811 | 0.761 076 | https://www.genecards .org/cgi-bin/carddisp.pl?gene= PRRT2 |
| HOGA1 | 4-Hydroxy-2-Oxoglutarate Aldolase 1 | Protein Coding | 37 | GC10P 097586 | 0.761 076 | https://www.genecards .org/cgi- |

| | | | | | | |
|---|---|---|---|---|---|---|
| | | | | | | bin/carddisp.pl?gene=HOGA1 |
| NLRP4 | NLR Family Pyrin Domain Containing 4 | Protein Coding | 36 | GC19P055836 | 0.761076 | https://www.genecards.org/cgi-bin/carddisp.pl?gene=NLRP4 |
| LRRC56 | Leucine Rich Repeat Containing 56 | Protein Coding | 34 | GC11P000518 | 0.761076 | https://www.genecards.org/cgi-bin/carddisp.pl?gene=LRRC56 |
| SUCO | SUN Domain Containing Ossification Factor | Protein Coding | 33 | GC01P172532 | 0.761076 | https://www.genecards.org/cgi-bin/carddisp.pl?gene=SUCO |
| TAF1L | TATA-Box Binding Protein Associated Factor 1 Like | Protein Coding | 33 | GC09M032619 | 0.761076 | https://www.genecards.org/cgi-bin/carddisp.pl?gene=TAF1L |
| UBTD1 | Ubiquitin Domain Containing 1 | Protein Coding | 33 | GC10P097498 | 0.761076 | https://www.genecards.org/cgi-bin/carddisp.pl?gene=UBTD1 |
| TERC | Telomerase RNA Component | RNA Gene | 29 | GC03M169765 | 0.761076 | https://www.genecards.org/cgi-bin/carddisp.pl?gene=TERC |
| EVPLL | Envoplakin Like | Protein Coding | 27 | GC17P054656 | 0.761076 | https://www.genecards.org/cgi-bin/carddisp.pl?gene=EVPLL |
| MIR449C | MicroRNA 449c | RNA Gene | 18 | GC05M055173 | 0.761076 | https://www.genecards.org/cgi-bin/carddisp.pl?gene=MIR449C |
| MYAS1 | Myasthenia Gravis With Thymus Hyperplasia | Genetic Locus | 2 | GC06U990322 | 0.761076 | https://www.genecards.org/cgi-bin/carddisp.pl?gene=MYAS1 |
| THM | Thymoma | Genetic Locus | 2 | GC00U990370 | 0.761076 | https://www.genecards.org/cgi-bin/carddisp.pl?gene=THM |
| CSF2RB | Colony Stimulating Factor 2 Receptor Subunit Beta | Protein Coding | 46 | GC22P036913 | 0.738352 | https://www.genecards.org/cgi-bin/carddisp.pl?gene=CSF2RB |
| CLIC1 | Chloride Intracellular Channel 1 | Protein Coding | 40 | GC06M065968 | 0.738352 | https://www.genecards.org/cgi-bin/carddisp.pl?gene=CLIC1 |
| EGFL8 | EGF Like Domain Multiple 8 | Protein Coding | 34 | GC06P083721 | 0.738352 | https://www.genecards.org/cgi-bin/carddisp.pl?gene=EGFL8 |

| | | | | | | |
|---|---|---|---|---|---|---|
| ATF1 | Activating Transcription Factor 1 | Protein Coding | 43 | GC12P050763 | 0.737481 | https://www.genecards.org/cgi-bin/carddisp.pl?gene=ATF1 |
| PDK1 | Pyruvate Dehydrogenase Kinase 1 | Protein Coding | 45 | GC02P172555 | 0.736053 | https://www.genecards.org/cgi-bin/carddisp.pl?gene=PDK1 |
| RAB7A | RAB7A, Member RAS Oncogene Family | Protein Coding | 45 | GC03P134197 | 0.734205 | https://www.genecards.org/cgi-bin/carddisp.pl?gene=RAB7A |
| IBD5 | Inflammatory Bowel Disease 5 | Genetic Locus | 5 | GC05U990104 | 0.732442 | https://www.genecards.org/cgi-bin/carddisp.pl?gene=IBD5 |
| KIR3DL2 | Killer Cell Immunoglobulin Like Receptor, Three Ig Domains And Long Cytoplasmic Tail 2 | Protein Coding | 39 | GC19P067386 | 0.731972 | https://www.genecards.org/cgi-bin/carddisp.pl?gene=KIR3DL2 |
| CD5L | CD5 Molecule Like | Protein Coding | 38 | GC01M157800 | 0.731972 | https://www.genecards.org/cgi-bin/carddisp.pl?gene=CD5L |
| DEFA5 | Defensin Alpha 5 | Protein Coding | 37 | GC08M007057 | 0.731972 | https://www.genecards.org/cgi-bin/carddisp.pl?gene=DEFA5 |
| DEFA6 | Defensin Alpha 6 | Protein Coding | 36 | GC08M006924 | 0.731972 | https://www.genecards.org/cgi-bin/carddisp.pl?gene=DEFA6 |
| E2F1 | E2F Transcription Factor 1 | Protein Coding | 42 | GC20M033675 | 0.72554 | https://www.genecards.org/cgi-bin/carddisp.pl?gene=E2F1 |
| CORIN | Corin, Serine Peptidase | Protein Coding | 43 | GC04M047596 | 0.716256 | https://www.genecards.org/cgi-bin/carddisp.pl?gene=CORIN |
| UBTF | Upstream Binding Transcription Factor | Protein Coding | 41 | GC17M044205 | 0.716256 | https://www.genecards.org/cgi-bin/carddisp.pl?gene=UBTF |
| LMNB1 | Lamin B1 | Protein Coding | 45 | GC05P126776 | 0.709613 | https://www.genecards.org/cgi-bin/carddisp.pl?gene=LMNB1 |
| DPYD | Dihydropyrimidine Dehydrogenase | Protein Coding | 50 | GC01M097015 | 0.705716 | https://www.genecards.org/cgi-bin/carddisp.pl?gene=DPYD |

| | | | | | | |
|---|---|---|---|---|---|---|
| SNCA | Synuclein Alpha | Protein Coding | 48 | GC04M 089724 | 0.705 716 | https://www.genecards .org/cgi-bin/carddisp.pl?gene= SNCA |
| DUSP6 | Dual Specificity Phosphatase 6 | Protein Coding | 47 | GC12M 089347 | 0.705 716 | https://www.genecards .org/cgi-bin/carddisp.pl?gene= DUSP6 |
| ITGA3 | Integrin Subunit Alpha 3 | Protein Coding | 46 | GC17P 050055 | 0.705 716 | https://www.genecards .org/cgi-bin/carddisp.pl?gene=I TGA3 |
| ABCC4 | ATP Binding Cassette Subfamily C Member 4 | Protein Coding | 45 | GC13M 095019 | 0.705 716 | https://www.genecards .org/cgi-bin/carddisp.pl?gene= ABCC4 |
| GNRHR | Gonadotropin Releasing Hormone Receptor | Protein Coding | 45 | GC04M 067737 | 0.705 716 | https://www.genecards .org/cgi-bin/carddisp.pl?gene= GNRHR |
| LHCGR | Luteinizing Hormone/Choriogonadotr opin Receptor | Protein Coding | 45 | GC02M 048686 | 0.705 716 | https://www.genecards .org/cgi-bin/carddisp.pl?gene= LHCGR |
| MOG | Myelin Oligodendrocyte Glycoprotein | Protein Coding | 45 | GC06P 083635 | 0.705 716 | https://www.genecards .org/cgi-bin/carddisp.pl?gene= MOG |
| NR0B1 | Nuclear Receptor Subfamily 0 Group B Member 1 | Protein Coding | 45 | GC0X M0303 04 | 0.705 716 | https://www.genecards .org/cgi-bin/carddisp.pl?gene= NR0B1 |
| ADRA2C | Adrenoceptor Alpha 2C | Protein Coding | 44 | GC04P 003766 | 0.705 716 | https://www.genecards .org/cgi-bin/carddisp.pl?gene= ADRA2C |
| KCNA4 | Potassium Voltage-Gated Channel Subfamily A Member 4 | Protein Coding | 44 | GC11M 030009 | 0.705 716 | https://www.genecards .org/cgi-bin/carddisp.pl?gene= KCNA4 |
| NEFL | Neurofilament Light Chain | Protein Coding | 44 | GC08M 024950 | 0.705 716 | https://www.genecards .org/cgi-bin/carddisp.pl?gene= NEFL |
| FLRT3 | Fibronectin Leucine Rich Transmembrane Protein 3 | Protein Coding | 43 | GC20M 014322 | 0.705 716 | https://www.genecards .org/cgi-bin/carddisp.pl?gene= FLRT3 |
| GMPS | Guanine Monophosphate Synthase | Protein Coding | 43 | GC03P 155870 | 0.705 716 | https://www.genecards .org/cgi-bin/carddisp.pl?gene= GMPS |
| RNASEH 1 | Ribonuclease H1 | Protein Coding | 43 | GC02M 003522 | 0.705 716 | https://www.genecards .org/cgi- |

| | | | | | | |
|---|---|---|---|---|---|---|
| | | | | | | bin/carddisp.pl?gene=RNASEH1 |
| FSHB | Follicle Stimulating Hormone Subunit Beta | Protein Coding | 42 | GC11P030210 | 0.705716 | https://www.genecards.org/cgi-bin/carddisp.pl?gene=FSHB |
| HTT | Huntingtin | Protein Coding | 42 | GC04P003041 | 0.705716 | https://www.genecards.org/cgi-bin/carddisp.pl?gene=HTT |
| IL17RD | Interleukin 17 Receptor D | Protein Coding | 42 | GC03M057089 | 0.705716 | https://www.genecards.org/cgi-bin/carddisp.pl?gene=IL17RD |
| LGI1 | Leucine Rich Glioma Inactivated 1 | Protein Coding | 42 | GC10P093757 | 0.705716 | https://www.genecards.org/cgi-bin/carddisp.pl?gene=LGI1 |
| POLR3A | RNA Polymerase III Subunit A | Protein Coding | 42 | GC10M078577 | 0.705716 | https://www.genecards.org/cgi-bin/carddisp.pl?gene=POLR3A |
| SPRY4 | Sprouty RTK Signaling Antagonist 4 | Protein Coding | 42 | GC05M142310 | 0.705716 | https://www.genecards.org/cgi-bin/carddisp.pl?gene=SPRY4 |
| TGM3 | Transglutaminase 3 | Protein Coding | 42 | GC20P002296 | 0.705716 | https://www.genecards.org/cgi-bin/carddisp.pl?gene=TGM3 |
| CTSE | Cathepsin E | Protein Coding | 41 | GC01M206009 | 0.705716 | https://www.genecards.org/cgi-bin/carddisp.pl?gene=CTSE |
| CYP4V2 | Cytochrome P450 Family 4 Subfamily V Member 2 | Protein Coding | 41 | GC04P186191 | 0.705716 | https://www.genecards.org/cgi-bin/carddisp.pl?gene=CYP4V2 |
| FCER1A | Fc Epsilon Receptor Ia | Protein Coding | 41 | GC01P159259 | 0.705716 | https://www.genecards.org/cgi-bin/carddisp.pl?gene=FCER1A |
| HS6ST1 | Heparan Sulfate 6-O-Sulfotransferase 1 | Protein Coding | 41 | GC02M128236 | 0.705716 | https://www.genecards.org/cgi-bin/carddisp.pl?gene=HS6ST1 |
| IGBP1 | Immunoglobulin Binding Protein 1 | Protein Coding | 41 | GC0XP070133 | 0.705716 | https://www.genecards.org/cgi-bin/carddisp.pl?gene=IGBP1 |
| IGHMBP2 | Immunoglobulin Mu DNA Binding Protein 2 | Protein Coding | 41 | GC11P068903 | 0.705716 | https://www.genecards.org/cgi-bin/carddisp.pl?gene=IGHMBP2 |

| | | | | | | |
|---|---|---|---|---|---|---|
| KISS1 | KiSS-1 Metastasis Suppressor | Protein Coding | 41 | GC01M 204190 | 0.705 716 | https://www.genecards .org/cgi- bin/carddisp.pl?gene= KISS1 |
| PROKR2 | Prokineticin Receptor 2 | Protein Coding | 41 | GC20M 005409 | 0.705 716 | https://www.genecards .org/cgi- bin/carddisp.pl?gene= PROKR2 |
| RCC1 | Regulator Of Chromosome Condensation 1 | Protein Coding | 41 | GC01P 028505 | 0.705 716 | https://www.genecards .org/cgi- bin/carddisp.pl?gene= RCC1 |
| MS4A2 | Membrane Spanning 4- Domains A2 | Protein Coding | 40 | GC11P 060088 | 0.705 716 | https://www.genecards .org/cgi- bin/carddisp.pl?gene= MS4A2 |
| PACSIN2 | Protein Kinase C And Casein Kinase Substrate In Neurons 2 | Protein Coding | 40 | GC22M 042835 | 0.705 716 | https://www.genecards .org/cgi- bin/carddisp.pl?gene= PACSIN2 |
| SFTPA2 | Surfactant Protein A2 | Protein Coding | 40 | GC10M 081851 | 0.705 716 | https://www.genecards .org/cgi- bin/carddisp.pl?gene= SFTPA2 |
| FEZF1 | FEZ Family Zinc Finger 1 | Protein Coding | 39 | GC07M 122301 | 0.705 716 | https://www.genecards .org/cgi- bin/carddisp.pl?gene= FEZF1 |
| MADCA M1 | Mucosal Vascular Addressin Cell Adhesion Molecule 1 | Protein Coding | 39 | GC19P 002778 | 0.705 716 | https://www.genecards .org/cgi- bin/carddisp.pl?gene= MADCAM1 |
| CSAD | Cysteine Sulfinic Acid Decarboxylase | Protein Coding | 38 | GC12M 053160 | 0.705 716 | https://www.genecards .org/cgi- bin/carddisp.pl?gene= CSAD |
| LHB | Luteinizing Hormone Subunit Beta | Protein Coding | 38 | GC19M 049015 | 0.705 716 | https://www.genecards .org/cgi- bin/carddisp.pl?gene= LHB |
| NUDT15 | Nudix Hydrolase 15 | Protein Coding | 38 | GC13P 048037 | 0.705 716 | https://www.genecards .org/cgi- bin/carddisp.pl?gene= NUDT15 |
| WDR11 | WD Repeat Domain 11 | Protein Coding | 38 | GC10P 120851 | 0.705 716 | https://www.genecards .org/cgi- bin/carddisp.pl?gene= WDR11 |
| CRLF2 | Cytokine Receptor Like Factor 2 | Protein Coding | 37 | GC0X M0011 90 | 0.705 716 | https://www.genecards .org/cgi- bin/carddisp.pl?gene= CRLF2 |
| NSMF | NMDA Receptor Synaptonuclear Signaling | Protein Coding | 37 | GC09M 137447 | 0.705 716 | https://www.genecards .org/cgi- |

| | | | | | | |
|---|---|---|---|---|---|---|
| | And Neuronal Migration Factor | | | | | bin/carddisp.pl?gene=NSMF |
| SNRPC | Small Nuclear Ribonucleoprotein Polypeptide C | Protein Coding | 37 | GC06P083816 | 0.705716 | https://www.genecards.org/cgi-bin/carddisp.pl?gene=SNRPC |
| ANOS1 | Anosmin 1 | Protein Coding | 36 | GC0XM008528 | 0.705716 | https://www.genecards.org/cgi-bin/carddisp.pl?gene=ANOS1 |
| CSN3 | Casein Kappa | Protein Coding | 36 | GC04P070242 | 0.705716 | https://www.genecards.org/cgi-bin/carddisp.pl?gene=CSN3 |
| UGT1A8 | UDP Glucuronosyltransferase Family 1 Member A8 | Protein Coding | 36 | GC02P233618 | 0.705716 | https://www.genecards.org/cgi-bin/carddisp.pl?gene=UGT1A8 |
| NDNF | Neuron Derived Neurotrophic Factor | Protein Coding | 35 | GC04M121035 | 0.705716 | https://www.genecards.org/cgi-bin/carddisp.pl?gene=NDNF |
| ADM2 | Adrenomedullin 2 | Protein Coding | 34 | GC22P050481 | 0.705716 | https://www.genecards.org/cgi-bin/carddisp.pl?gene=ADM2 |
| CNOT11 | CCR4-NOT Transcription Complex Subunit 11 | Protein Coding | 30 | GC02P101252 | 0.705716 | https://www.genecards.org/cgi-bin/carddisp.pl?gene=CNOT11 |
| DEFB103A | Defensin Beta 103A | Protein Coding | 26 | GC08P007881 | 0.705716 | https://www.genecards.org/cgi-bin/carddisp.pl?gene=DEFB103A |
| LINC01194 | Long Intergenic Non-Protein Coding RNA 1194 | RNA Gene | 17 | GC05P012578 | 0.705716 | https://www.genecards.org/cgi-bin/carddisp.pl?gene=LINC01194 |
| ZFAT-AS1 | ZFAT Antisense RNA 1 | RNA Gene | 16 | GC08P134643 | 0.705716 | https://www.genecards.org/cgi-bin/carddisp.pl?gene=ZFAT-AS1 |
| MIR3681 | MicroRNA 3681 | RNA Gene | 12 | GC02P012199 | 0.705716 | https://www.genecards.org/cgi-bin/carddisp.pl?gene=MIR3681 |
| MT-TR | Mitochondrially Encoded TRNA-Arg (CGN) | RNA Gene | 12 | GCMTP010407 | 0.705716 | https://www.genecards.org/cgi-bin/carddisp.pl?gene=MT-TR |
| LOC110806263 | TERT 5' Regulatory Region | Biological Region | 3 | GC05P001294 | 0.705716 | https://www.genecards.org/cgi-bin/carddisp.pl?gene=LOC110806263 |

| | | | | | | |
|---|---|---|---|---|---|---|
| TENM3 | Teneurin Transmembrane Protein 3 | Protein Coding | 37 | GC04P 181448 | 0.700 934 | https://www.genecards .org/cgi-bin/carddisp.pl?gene= TENM3 |
| IL27RA | Interleukin 27 Receptor Subunit Alpha | Protein Coding | 39 | GC19P 014031 | 0.700 063 | https://www.genecards .org/cgi-bin/carddisp.pl?gene=I L27RA |
| ZBTB7A | Zinc Finger And BTB Domain Containing 7A | Protein Coding | 37 | GC19M 005443 | 0.700 063 | https://www.genecards .org/cgi-bin/carddisp.pl?gene= ZBTB7A |
| FASN | Fatty Acid Synthase | Protein Coding | 47 | GC17M 082078 | 0.698 669 | https://www.genecards .org/cgi-bin/carddisp.pl?gene= FASN |
| WDR5 | WD Repeat Domain 5 | Protein Coding | 42 | GC09P 134135 | 0.688 472 | https://www.genecards .org/cgi-bin/carddisp.pl?gene= WDR5 |
| SOAT1 | Sterol O-Acyltransferase 1 | Protein Coding | 43 | GC01P 179262 | 0.683 136 | https://www.genecards .org/cgi-bin/carddisp.pl?gene= SOAT1 |
| PSEN1 | Presenilin 1 | Protein Coding | 50 | GC14P 073136 | 0.678 235 | https://www.genecards .org/cgi-bin/carddisp.pl?gene= PSEN1 |
| EPHX1 | Epoxide Hydrolase 1 | Protein Coding | 44 | GC01P 225810 | 0.672 335 | https://www.genecards .org/cgi-bin/carddisp.pl?gene= EPHX1 |
| IDO2 | Indoleamine 2,3-Dioxygenase 2 | Protein Coding | 36 | GC08P 039965 | 0.672 335 | https://www.genecards .org/cgi-bin/carddisp.pl?gene=I DO2 |
| PRKAB1 | Protein Kinase AMP-Activated Non-Catalytic Subunit Beta 1 | Protein Coding | 45 | GC12P 119632 | 0.671 612 | https://www.genecards .org/cgi-bin/carddisp.pl?gene= PRKAB1 |
| PPARGC 1A | PPARG Coactivator 1 Alpha | Protein Coding | 44 | GC04M 023755 | 0.671 612 | https://www.genecards .org/cgi-bin/carddisp.pl?gene= PPARGC1A |
| TUBB | Tubulin Beta Class I | Protein Coding | 48 | GC06P 083675 | 0.664 799 | https://www.genecards .org/cgi-bin/carddisp.pl?gene= TUBB |
| FOXP1 | Forkhead Box P1 | Protein Coding | 44 | GC03M 070926 | 0.664 799 | https://www.genecards .org/cgi-bin/carddisp.pl?gene= FOXP1 |
| PRICKLE 2 | Prickle Planar Cell Polarity Protein 2 | Protein Coding | 38 | GC03M 064079 | 0.664 799 | https://www.genecards .org/cgi- |

| | | | | | | |
|---|---|---|---|---|---|---|
| | | | | | | bin/carddisp.pl?gene=PRICKLE2 |
| MRPL23 | Mitochondrial Ribosomal Protein L23 | Protein Coding | 36 | GC11P 001948 | 0.664 799 | https://www.genecards.org/cgi-bin/carddisp.pl?gene=MRPL23 |
| VARS1 | Valyl-TRNA Synthetase 1 | Protein Coding | 35 | GC06M 065973 | 0.664 799 | https://www.genecards.org/cgi-bin/carddisp.pl?gene=VARS1 |
| VWA7 | Von Willebrand Factor A Domain Containing 7 | Protein Coding | 31 | GC06M 065969 | 0.664 799 | https://www.genecards.org/cgi-bin/carddisp.pl?gene=VWA7 |
| SNHG32 | Small Nucleolar RNA Host Gene 32 | RNA Gene | 22 | GC06P 087379 | 0.664 799 | https://www.genecards.org/cgi-bin/carddisp.pl?gene=SNHG32 |
| LINC002 43 | Long Intergenic Non-Protein Coding RNA 243 | RNA Gene | 18 | GC06M 065884 | 0.664 799 | https://www.genecards.org/cgi-bin/carddisp.pl?gene=LINC00243 |
| SNORD4 8 | Small Nucleolar RNA, C/D Box 48 | RNA Gene | 17 | GC06P 083715 | 0.664 799 | https://www.genecards.org/cgi-bin/carddisp.pl?gene=SNORD48 |
| FARSA | Phenylalanyl-TRNA Synthetase Subunit Alpha | Protein Coding | 41 | GC19M 012922 | 0.659 761 | https://www.genecards.org/cgi-bin/carddisp.pl?gene=FARSA |
| CALCB | Calcitonin Related Polypeptide Beta | Protein Coding | 37 | GC11P 014904 | 0.659 761 | https://www.genecards.org/cgi-bin/carddisp.pl?gene=CALCB |
| TINAGL 1 | Tubulointerstitial Nephritis Antigen Like 1 | Protein Coding | 37 | GC01P 031576 | 0.659 761 | https://www.genecards.org/cgi-bin/carddisp.pl?gene=TINAGL1 |
| TRAT1 | T Cell Receptor Associated Transmembrane Adaptor 1 | Protein Coding | 36 | GC03P 108823 | 0.659 749 | https://www.genecards.org/cgi-bin/carddisp.pl?gene=TRAT1 |
| SETD6 | SET Domain Containing 6, Protein Lysine Methyltransferase | Protein Coding | 35 | GC16P 058514 | 0.659 749 | https://www.genecards.org/cgi-bin/carddisp.pl?gene=SETD6 |
| UHRF1 | Ubiquitin Like With PHD And Ring Finger Domains 1 | Protein Coding | 38 | GC19P 004960 | 0.648 814 | https://www.genecards.org/cgi-bin/carddisp.pl?gene=UHRF1 |
| OPRL1 | Opioid Related Nociceptin Receptor 1 | Protein Coding | 45 | GC20P 064080 | 0.641 638 | https://www.genecards.org/cgi-bin/carddisp.pl?gene=OPRL1 |

| | | | | | | |
|---|---|---|---|---|---|---|
| PDPK1 | 3-Phosphoinositide Dependent Protein Kinase 1 | Protein Coding | 47 | GC16P 002537 | 0.636 199 | https://www.genecards .org/cgi-bin/carddisp.pl?gene= PDPK1 |
| FUCA1 | Alpha-L-Fucosidase 1 | Protein Coding | 46 | GC01M 023845 | 0.636 06 | https://www.genecards .org/cgi-bin/carddisp.pl?gene= FUCA1 |
| TNNI2 | Troponin I2, Fast Skeletal Type | Protein Coding | 43 | GC11P 001865 | 0.636 06 | https://www.genecards .org/cgi-bin/carddisp.pl?gene= TNNI2 |
| SMAD1 | SMAD Family Member 1 | Protein Coding | 41 | GC04P 145481 | 0.636 06 | https://www.genecards .org/cgi-bin/carddisp.pl?gene= SMAD1 |
| NOG | Noggin | Protein Coding | 45 | GC17P 056593 | 0.635 476 | https://www.genecards .org/cgi-bin/carddisp.pl?gene= NOG |
| HSD3B2 | Hydroxy-Delta-5-Steroid Dehydrogenase, 3 Beta- And Steroid Delta-Isomerase 2 | Protein Coding | 45 | GC01P 119414 | 0.632 033 | https://www.genecards .org/cgi-bin/carddisp.pl?gene= HSD3B2 |
| PPP2R1A | Protein Phosphatase 2 Scaffold Subunit Aalpha | Protein Coding | 45 | GC19P 067249 | 0.632 033 | https://www.genecards .org/cgi-bin/carddisp.pl?gene= PPP2R1A |
| MAP3K1 2 | Mitogen-Activated Protein Kinase Kinase Kinase 12 | Protein Coding | 44 | GC12M 053479 | 0.632 033 | https://www.genecards .org/cgi-bin/carddisp.pl?gene= MAP3K12 |
| CYP4F3 | Cytochrome P450 Family 4 Subfamily F Member 3 | Protein Coding | 42 | GC19P 015640 | 0.632 033 | https://www.genecards .org/cgi-bin/carddisp.pl?gene= CYP4F3 |
| TCP1 | T-Complex 1 | Protein Coding | 42 | GC06M 159778 | 0.632 033 | https://www.genecards .org/cgi-bin/carddisp.pl?gene= TCP1 |
| DNAJB1 | DnaJ Heat Shock Protein Family (Hsp40) Member B1 | Protein Coding | 41 | GC19M 014514 | 0.632 033 | https://www.genecards .org/cgi-bin/carddisp.pl?gene= DNAJB1 |
| CRHBP | Corticotropin Releasing Hormone Binding Protein | Protein Coding | 40 | GC05P 076952 | 0.632 033 | https://www.genecards .org/cgi-bin/carddisp.pl?gene= CRHBP |
| DNAJA1 | DnaJ Heat Shock Protein Family (Hsp40) Member A1 | Protein Coding | 40 | GC09P 033025 | 0.632 033 | https://www.genecards .org/cgi-bin/carddisp.pl?gene= DNAJA1 |

| | | | | | | |
|---|---|---|---|---|---|---|
| IGF2BP3 | Insulin Like Growth Factor 2 MRNA Binding Protein 3 | Protein Coding | 40 | GC07M 023316 | 0.632 033 | https://www.genecards .org/cgi-bin/carddisp.pl?gene=I GF2BP3 |
| RPL9 | Ribosomal Protein L9 | Protein Coding | 40 | GC04M 039452 | 0.632 033 | https://www.genecards .org/cgi-bin/carddisp.pl?gene= RPL9 |
| TRPC1 | Transient Receptor Potential Cation Channel Subfamily C Member 1 | Protein Coding | 40 | GC03P 142724 | 0.632 033 | https://www.genecards .org/cgi-bin/carddisp.pl?gene= TRPC1 |
| TIMM50 | Translocase Of Inner Mitochondrial Membrane 50 | Protein Coding | 38 | GC19P 039480 | 0.632 033 | https://www.genecards .org/cgi-bin/carddisp.pl?gene= TIMM50 |
| RBM39 | RNA Binding Motif Protein 39 | Protein Coding | 37 | GC20M 035703 | 0.632 033 | https://www.genecards .org/cgi-bin/carddisp.pl?gene= RBM39 |
| ZC3H12 A | Zinc Finger CCCH-Type Containing 12A | Protein Coding | 36 | GC01P 037474 | 0.632 033 | https://www.genecards .org/cgi-bin/carddisp.pl?gene= ZC3H12A |
| H2BC12L | H2B Clustered Histone 12 Like | Protein Coding | 23 | GC21P 043569 | 0.632 033 | https://www.genecards .org/cgi-bin/carddisp.pl?gene= H2BC12L |
| MIR3926 -1 | MicroRNA 3926-1 | RNA Gene | 12 | GC08M 012727 | 0.632 033 | https://www.genecards .org/cgi-bin/carddisp.pl?gene= MIR3926-1 |
| MRPS17 P7 | Mitochondrial Ribosomal Protein S17 Pseudogene 7 | Pseudo gene | 6 | GC18M 057763 | 0.632 033 | https://www.genecards .org/cgi-bin/carddisp.pl?gene= MRPS17P7 |
| DAB2 | DAB Adaptor Protein 2 | Protein Coding | 41 | GC05M 039371 | 0.622 343 | https://www.genecards .org/cgi-bin/carddisp.pl?gene= DAB2 |
| MUC2 | Mucin 2, Oligomeric Mucus/Gel-Forming | Protein Coding | 39 | GC11P 001074 | 0.622 343 | https://www.genecards .org/cgi-bin/carddisp.pl?gene= MUC2 |
| MUC17 | Mucin 17, Cell Surface Associated | Protein Coding | 36 | GC07P 101020 | 0.622 343 | https://www.genecards .org/cgi-bin/carddisp.pl?gene= MUC17 |
| MUC3A | Mucin 3A, Cell Surface Associated | Protein Coding | 35 | GC07P 100949 | 0.622 343 | https://www.genecards .org/cgi-bin/carddisp.pl?gene= MUC3A |
| MUC3B | Mucin 3B, Cell Surface Associated | Protein Coding | 19 | GC07U 903146 | 0.622 343 | https://www.genecards .org/cgi- |

| | | | | | | |
|---|---|---|---|---|---|---|
| | | | | | | bin/carddisp.pl?gene=MUC3B |
| TRAF3 | TNF Receptor Associated Factor 3 | Protein Coding | 45 | GC14P109708 | 0.606795 | https://www.genecards.org/cgi-bin/carddisp.pl?gene=TRAF3 |
| PPIB | Peptidylprolyl Isomerase B | Protein Coding | 48 | GC15M064155 | 0.598059 | https://www.genecards.org/cgi-bin/carddisp.pl?gene=PPIB |
| ACTB | Actin Beta | Protein Coding | 47 | GC07M005527 | 0.598059 | https://www.genecards.org/cgi-bin/carddisp.pl?gene=ACTB |
| ABCC6 | ATP Binding Cassette Subfamily C Member 6 | Protein Coding | 45 | GC16M016148 | 0.598059 | https://www.genecards.org/cgi-bin/carddisp.pl?gene=ABCC6 |
| CEBPA | CCAAT Enhancer Binding Protein Alpha | Protein Coding | 45 | GC19M033299 | 0.598059 | https://www.genecards.org/cgi-bin/carddisp.pl?gene=CEBPA |
| FBLN5 | Fibulin 5 | Protein Coding | 45 | GC14M091869 | 0.598059 | https://www.genecards.org/cgi-bin/carddisp.pl?gene=FBLN5 |
| SCUBE3 | Signal Peptide, CUB Domain And EGF Like Domain Containing 3 | Protein Coding | 39 | GC06P083821 | 0.595897 | https://www.genecards.org/cgi-bin/carddisp.pl?gene=SCUBE3 |
| CUX1 | Cut Like Homeobox 1 | Protein Coding | 43 | GC07P101815 | 0.594615 | https://www.genecards.org/cgi-bin/carddisp.pl?gene=CUX1 |
| KPNB1 | Karyopherin Subunit Beta 1 | Protein Coding | 42 | GC17P047649 | 0.594615 | https://www.genecards.org/cgi-bin/carddisp.pl?gene=KPNB1 |
| SOX4 | SRY-Box Transcription Factor 4 | Protein Coding | 42 | GC06P021593 | 0.594615 | https://www.genecards.org/cgi-bin/carddisp.pl?gene=SOX4 |
| MIP | Major Intrinsic Protein Of Lens Fiber | Protein Coding | 40 | GC12M056449 | 0.594615 | https://www.genecards.org/cgi-bin/carddisp.pl?gene=MIP |
| BDKRB2 | Bradykinin Receptor B2 | Protein Coding | 43 | GC14P096205 | 0.581992 | https://www.genecards.org/cgi-bin/carddisp.pl?gene=BDKRB2 |
| ITGBL1 | Integrin Subunit Beta Like 1 | Protein Coding | 36 | GC13P101454 | 0.576676 | https://www.genecards.org/cgi-bin/carddisp.pl?gene=ITGBL1 |

| | | | | | | |
|---|---|---|---|---|---|---|
| ITPR1 | Inositol 1,4,5-Trisphosphate Receptor Type 1 | Protein Coding | 47 | GC03P004486 | 0.561536 | https://www.genecards.org/cgi-bin/carddisp.pl?gene=ITPR1 |
| ATR | ATR Serine/Threonine Kinase | Protein Coding | 50 | GC03M142449 | 0.55848 | https://www.genecards.org/cgi-bin/carddisp.pl?gene=ATR |
| SLC9A1 | Solute Carrier Family 9 Member A1 | Protein Coding | 49 | GC01M027135 | 0.55848 | https://www.genecards.org/cgi-bin/carddisp.pl?gene=SLC9A1 |
| PHGDH | Phosphoglycerate Dehydrogenase | Protein Coding | 48 | GC01P119660 | 0.55848 | https://www.genecards.org/cgi-bin/carddisp.pl?gene=PHGDH |
| SCNN1B | Sodium Channel Epithelial 1 Subunit Beta | Protein Coding | 48 | GC16P023278 | 0.55848 | https://www.genecards.org/cgi-bin/carddisp.pl?gene=SCNN1B |
| ESRRB | Estrogen Related Receptor Beta | Protein Coding | 47 | GC14P076310 | 0.55848 | https://www.genecards.org/cgi-bin/carddisp.pl?gene=ESRRB |
| RARA | Retinoic Acid Receptor Alpha | Protein Coding | 47 | GC17P040309 | 0.55848 | https://www.genecards.org/cgi-bin/carddisp.pl?gene=RARA |
| CHAT | Choline O-Acetyltransferase | Protein Coding | 46 | GC10P049609 | 0.55848 | https://www.genecards.org/cgi-bin/carddisp.pl?gene=CHAT |
| GLUL | Glutamate-Ammonia Ligase | Protein Coding | 46 | GC01M182378 | 0.55848 | https://www.genecards.org/cgi-bin/carddisp.pl?gene=GLUL |
| KAT2B | Lysine Acetyltransferase 2B | Protein Coding | 46 | GC03P020043 | 0.55848 | https://www.genecards.org/cgi-bin/carddisp.pl?gene=KAT2B |
| SDHA | Succinate Dehydrogenase Complex Flavoprotein Subunit A | Protein Coding | 46 | GC05P000208 | 0.55848 | https://www.genecards.org/cgi-bin/carddisp.pl?gene=SDHA |
| DRD5 | Dopamine Receptor D5 | Protein Coding | 45 | GC04P009783 | 0.55848 | https://www.genecards.org/cgi-bin/carddisp.pl?gene=DRD5 |
| MYCN | MYCN Proto-Oncogene, BHLH Transcription Factor | Protein Coding | 45 | GC02P015949 | 0.55848 | https://www.genecards.org/cgi-bin/carddisp.pl?gene=MYCN |
| CBLB | Cbl Proto-Oncogene B | Protein Coding | 44 | GC03M105655 | 0.55848 | https://www.genecards.org/cgi- |

| | | | | | | |
|---|---|---|---|---|---|---|
| | | | | | | bin/carddisp.pl?gene=CBLB |
| DOT1L | DOT1 Like Histone Lysine Methyltransferase | Protein Coding | 44 | GC19P002935 | 0.55848 | https://www.genecards.org/cgi-bin/carddisp.pl?gene=DOT1L |
| EIF4EBP1 | Eukaryotic Translation Initiation Factor 4E Binding Protein 1 | Protein Coding | 44 | GC08P038469 | 0.55848 | https://www.genecards.org/cgi-bin/carddisp.pl?gene=EIF4EBP1 |
| F2RL3 | F2R Like Thrombin Or Trypsin Receptor 3 | Protein Coding | 44 | GC19P016888 | 0.55848 | https://www.genecards.org/cgi-bin/carddisp.pl?gene=F2RL3 |
| GJA8 | Gap Junction Protein Alpha 8 | Protein Coding | 44 | GC01P147902 | 0.55848 | https://www.genecards.org/cgi-bin/carddisp.pl?gene=GJA8 |
| KLF4 | Kruppel Like Factor 4 | Protein Coding | 44 | GC09M107484 | 0.55848 | https://www.genecards.org/cgi-bin/carddisp.pl?gene=KLF4 |
| PSMA7 | Proteasome 20S Subunit Alpha 7 | Protein Coding | 44 | GC20M062136 | 0.55848 | https://www.genecards.org/cgi-bin/carddisp.pl?gene=PSMA7 |
| CCT5 | Chaperonin Containing TCP1 Subunit 5 | Protein Coding | 43 | GC05P010236 | 0.55848 | https://www.genecards.org/cgi-bin/carddisp.pl?gene=CCT5 |
| COL4A6 | Collagen Type IV Alpha 6 Chain | Protein Coding | 43 | GC0XM108155 | 0.55848 | https://www.genecards.org/cgi-bin/carddisp.pl?gene=COL4A6 |
| RUVBL2 | RuvB Like AAA ATPase 2 | Protein Coding | 43 | GC19P048993 | 0.55848 | https://www.genecards.org/cgi-bin/carddisp.pl?gene=RUVBL2 |
| ACP2 | Acid Phosphatase 2, Lysosomal | Protein Coding | 42 | GC11M089360 | 0.55848 | https://www.genecards.org/cgi-bin/carddisp.pl?gene=ACP2 |
| ATP6AP1 | ATPase H+ Transporting Accessory Protein 1 | Protein Coding | 42 | GC0XP154428 | 0.55848 | https://www.genecards.org/cgi-bin/carddisp.pl?gene=ATP6AP1 |
| ATP6V0D1 | ATPase H+ Transporting V0 Subunit D1 | Protein Coding | 42 | GC16M067438 | 0.55848 | https://www.genecards.org/cgi-bin/carddisp.pl?gene=ATP6V0D1 |
| ERLIN1 | ER Lipid Raft Associated 1 | Protein Coding | 42 | GC10M100150 | 0.55848 | https://www.genecards.org/cgi-bin/carddisp.pl?gene=ERLIN1 |

| | | | | | | |
|---|---|---|---|---|---|---|
| KDM6B | Lysine Demethylase 6B | Protein Coding | 42 | GC17P 007834 | 0.558 48 | https://www.genecards .org/cgi-bin/carddisp.pl?gene= KDM6B |
| LAMA5 | Laminin Subunit Alpha 5 | Protein Coding | 42 | GC20M 062307 | 0.558 48 | https://www.genecards .org/cgi-bin/carddisp.pl?gene= LAMA5 |
| LGR5 | Leucine Rich Repeat Containing G Protein-Coupled Receptor 5 | Protein Coding | 42 | GC12P 071439 | 0.558 48 | https://www.genecards .org/cgi-bin/carddisp.pl?gene= LGR5 |
| NMT1 | N-Myristoyltransferase 1 | Protein Coding | 42 | GC17P 055763 | 0.558 48 | https://www.genecards .org/cgi-bin/carddisp.pl?gene= NMT1 |
| PSMA1 | Proteasome 20S Subunit Alpha 1 | Protein Coding | 42 | GC11M 014505 | 0.558 48 | https://www.genecards .org/cgi-bin/carddisp.pl?gene= PSMA1 |
| PSMA5 | Proteasome 20S Subunit Alpha 5 | Protein Coding | 42 | GC01M 109399 | 0.558 48 | https://www.genecards .org/cgi-bin/carddisp.pl?gene= PSMA5 |
| RPS20 | Ribosomal Protein S20 | Protein Coding | 42 | GC08M 056067 | 0.558 48 | https://www.genecards .org/cgi-bin/carddisp.pl?gene= RPS20 |
| TREM2 | Triggering Receptor Expressed On Myeloid Cells 2 | Protein Coding | 42 | GC06M 066157 | 0.558 48 | https://www.genecards .org/cgi-bin/carddisp.pl?gene= TREM2 |
| UBE2D2 | Ubiquitin Conjugating Enzyme E2 D2 | Protein Coding | 42 | GC05P 139526 | 0.558 48 | https://www.genecards .org/cgi-bin/carddisp.pl?gene= UBE2D2 |
| EXOSC2 | Exosome Component 2 | Protein Coding | 41 | GC09P 130693 | 0.558 48 | https://www.genecards .org/cgi-bin/carddisp.pl?gene= EXOSC2 |
| GALE | UDP-Galactose-4-Epimerase | Protein Coding | 41 | GC01M 023795 | 0.558 48 | https://www.genecards .org/cgi-bin/carddisp.pl?gene= GALE |
| GNB2 | G Protein Subunit Beta 2 | Protein Coding | 41 | GC07P 100673 | 0.558 48 | https://www.genecards .org/cgi-bin/carddisp.pl?gene= GNB2 |
| GRPR | Gastrin Releasing Peptide Receptor | Protein Coding | 41 | GC0XP 016141 | 0.558 48 | https://www.genecards .org/cgi-bin/carddisp.pl?gene= GRPR |
| HAX1 | HCLS1 Associated Protein X-1 | Protein Coding | 41 | GC01P 154273 | 0.558 48 | https://www.genecards .org/cgi- |

| | | | | | | |
|---|---|---|---|---|---|---|
| | | | | | | bin/carddisp.pl?gene=HAX1 |
| HNRNPU | Heterogeneous Nuclear Ribonucleoprotein U | Protein Coding | 41 | GC01M244844 | 0.55848 | https://www.genecards.org/cgi-bin/carddisp.pl?gene=HNRNPU |
| INHA | Inhibin Subunit Alpha | Protein Coding | 41 | GC02P219569 | 0.55848 | https://www.genecards.org/cgi-bin/carddisp.pl?gene=INHA |
| KLF6 | Kruppel Like Factor 6 | Protein Coding | 41 | GC10M003779 | 0.55848 | https://www.genecards.org/cgi-bin/carddisp.pl?gene=KLF6 |
| MATR3 | Matrin 3 | Protein Coding | 41 | GC05P139274 | 0.55848 | https://www.genecards.org/cgi-bin/carddisp.pl?gene=MATR3 |
| PSMA2 | Proteasome 20S Subunit Alpha 2 | Protein Coding | 41 | GC07M042916 | 0.55848 | https://www.genecards.org/cgi-bin/carddisp.pl?gene=PSMA2 |
| PSMB6 | Proteasome 20S Subunit Beta 6 | Protein Coding | 41 | GC17P004796 | 0.55848 | https://www.genecards.org/cgi-bin/carddisp.pl?gene=PSMB6 |
| RBCK1 | RANBP2-Type And C3HC4-Type Zinc Finger Containing 1 | Protein Coding | 41 | GC20P000407 | 0.55848 | https://www.genecards.org/cgi-bin/carddisp.pl?gene=RBCK1 |
| BNIP3L | BCL2 Interacting Protein 3 Like | Protein Coding | 40 | GC08P026383 | 0.55848 | https://www.genecards.org/cgi-bin/carddisp.pl?gene=BNIP3L |
| CDO1 | Cysteine Dioxygenase Type 1 | Protein Coding | 40 | GC05M115804 | 0.55848 | https://www.genecards.org/cgi-bin/carddisp.pl?gene=CDO1 |
| DEF6 | DEF6 Guanine Nucleotide Exchange Factor | Protein Coding | 40 | GC06P083820 | 0.55848 | https://www.genecards.org/cgi-bin/carddisp.pl?gene=DEF6 |
| FHL3 | Four And A Half LIM Domains 3 | Protein Coding | 40 | GC01M038118 | 0.55848 | https://www.genecards.org/cgi-bin/carddisp.pl?gene=FHL3 |
| KPNA1 | Karyopherin Subunit Alpha 1 | Protein Coding | 40 | GC03M122421 | 0.55848 | https://www.genecards.org/cgi-bin/carddisp.pl?gene=KPNA1 |
| MTCH2 | Mitochondrial Carrier 2 | Protein Coding | 40 | GC11M047604 | 0.55848 | https://www.genecards.org/cgi-bin/carddisp.pl?gene=MTCH2 |

| | | | | | | |
|---|---|---|---|---|---|---|
| MUC4 | Mucin 4, Cell Surface Associated | Protein Coding | 40 | GC03M 195746 | 0.558 48 | https://www.genecards .org/cgi- bin/carddisp.pl?gene= MUC4 |
| NADSYN 1 | NAD Synthetase 1 | Protein Coding | 40 | GC11P 071454 | 0.558 48 | https://www.genecards .org/cgi- bin/carddisp.pl?gene= NADSYN1 |
| RANBP9 | RAN Binding Protein 9 | Protein Coding | 40 | GC06M 013621 | 0.558 48 | https://www.genecards .org/cgi- bin/carddisp.pl?gene= RANBP9 |
| RNF31 | Ring Finger Protein 31 | Protein Coding | 40 | GC14P 024146 | 0.558 48 | https://www.genecards .org/cgi- bin/carddisp.pl?gene= RNF31 |
| SLC9A2 | Solute Carrier Family 9 Member A2 | Protein Coding | 40 | GC02P 102620 | 0.558 48 | https://www.genecards .org/cgi- bin/carddisp.pl?gene= SLC9A2 |
| UCK2 | Uridine-Cytidine Kinase 2 | Protein Coding | 40 | GC01P 165796 | 0.558 48 | https://www.genecards .org/cgi- bin/carddisp.pl?gene= UCK2 |
| UNC13B | Unc-13 Homolog B | Protein Coding | 40 | GC09P 035161 | 0.558 48 | https://www.genecards .org/cgi- bin/carddisp.pl?gene= UNC13B |
| CD93 | CD93 Molecule | Protein Coding | 39 | GC20M 023079 | 0.558 48 | https://www.genecards .org/cgi- bin/carddisp.pl?gene= CD93 |
| DDX17 | DEAD-Box Helicase 17 | Protein Coding | 39 | GC22M 038483 | 0.558 48 | https://www.genecards .org/cgi- bin/carddisp.pl?gene= DDX17 |
| GP5 | Glycoprotein V Platelet | Protein Coding | 39 | GC03M 194395 | 0.558 48 | https://www.genecards .org/cgi- bin/carddisp.pl?gene= GP5 |
| GTF2F1 | General Transcription Factor IIF Subunit 1 | Protein Coding | 39 | GC19M 006417 | 0.558 48 | https://www.genecards .org/cgi- bin/carddisp.pl?gene= GTF2F1 |
| LCN1 | Lipocalin 1 | Protein Coding | 39 | GC09P 135521 | 0.558 48 | https://www.genecards .org/cgi- bin/carddisp.pl?gene= LCN1 |
| MOV10 | Mov10 RISC Complex RNA Helicase | Protein Coding | 39 | GC01P 112673 | 0.558 48 | https://www.genecards .org/cgi- bin/carddisp.pl?gene= MOV10 |
| SHARPI N | SHANK Associated RH Domain Interactor | Protein Coding | 39 | GC08M 144098 | 0.558 48 | https://www.genecards .org/cgi- |

| | | | | | | |
|---|---|---|---|---|---|---|
| | | | | | | bin/carddisp.pl?gene=SHARPIN |
| UBR2 | Ubiquitin Protein Ligase E3 Component N-Recognin 2 | Protein Coding | 39 | GC06P083911 | 0.55848 | https://www.genecards.org/cgi-bin/carddisp.pl?gene=UBR2 |
| ARID3A | AT-Rich Interaction Domain 3A | Protein Coding | 38 | GC19P002812 | 0.55848 | https://www.genecards.org/cgi-bin/carddisp.pl?gene=ARID3A |
| BAG2 | BAG Cochaperone 2 | Protein Coding | 38 | GC06P057172 | 0.55848 | https://www.genecards.org/cgi-bin/carddisp.pl?gene=BAG2 |
| HNRNPF | Heterogeneous Nuclear Ribonucleoprotein F | Protein Coding | 38 | GC10M043385 | 0.55848 | https://www.genecards.org/cgi-bin/carddisp.pl?gene=HNRNPF |
| HNRNPH1 | Heterogeneous Nuclear Ribonucleoprotein H1 | Protein Coding | 38 | GC05M179614 | 0.55848 | https://www.genecards.org/cgi-bin/carddisp.pl?gene=HNRNPH1 |
| HNRNPR | Heterogeneous Nuclear Ribonucleoprotein R | Protein Coding | 38 | GC01M023303 | 0.55848 | https://www.genecards.org/cgi-bin/carddisp.pl?gene=HNRNPR |
| HSPA14 | Heat Shock Protein Family A (Hsp70) Member 14 | Protein Coding | 38 | GC10P014858 | 0.55848 | https://www.genecards.org/cgi-bin/carddisp.pl?gene=HSPA14 |
| IPO7 | Importin 7 | Protein Coding | 38 | GC11P009384 | 0.55848 | https://www.genecards.org/cgi-bin/carddisp.pl?gene=IPO7 |
| RPS11 | Ribosomal Protein S11 | Protein Coding | 38 | GC19P049496 | 0.55848 | https://www.genecards.org/cgi-bin/carddisp.pl?gene=RPS11 |
| RPS8 | Ribosomal Protein S8 | Protein Coding | 38 | GC01P044775 | 0.55848 | https://www.genecards.org/cgi-bin/carddisp.pl?gene=RPS8 |
| ALDOC | Aldolase, Fructose-Bisphosphate C | Protein Coding | 37 | GC17M035139 | 0.55848 | https://www.genecards.org/cgi-bin/carddisp.pl?gene=ALDOC |
| MAEA | Macrophage Erythroblast Attacher, E3 Ubiquitin Ligase | Protein Coding | 37 | GC04P001289 | 0.55848 | https://www.genecards.org/cgi-bin/carddisp.pl?gene=MAEA |
| PCDH18 | Protocadherin 18 | Protein Coding | 37 | GC04M137518 | 0.55848 | https://www.genecards.org/cgi-bin/carddisp.pl?gene=PCDH18 |

| | | | | | | |
|---|---|---|---|---|---|---|
| SH3RF1 | SH3 Domain Containing Ring Finger 1 | Protein Coding | 37 | GC04M 169094 | 0.558 48 | https://www.genecards .org/cgi-bin/carddisp.pl?gene= SH3RF1 |
| SLA | Src Like Adaptor | Protein Coding | 37 | GC08M 133040 | 0.558 48 | https://www.genecards .org/cgi-bin/carddisp.pl?gene= SLA |
| STOM | Stomatin | Protein Coding | 37 | GC09M 121338 | 0.558 48 | https://www.genecards .org/cgi-bin/carddisp.pl?gene= STOM |
| ARID5A | AT-Rich Interaction Domain 5A | Protein Coding | 36 | GC02P 096536 | 0.558 48 | https://www.genecards .org/cgi-bin/carddisp.pl?gene= ARID5A |
| ATP5F1A | ATP Synthase F1 Subunit Alpha | Protein Coding | 36 | GC18M 046081 | 0.558 48 | https://www.genecards .org/cgi-bin/carddisp.pl?gene= ATP5F1A |
| HS3ST3A 1 | Heparan Sulfate-Glucosamine 3-Sulfotransferase 3A1 | Protein Coding | 36 | GC17M 013494 | 0.558 48 | https://www.genecards .org/cgi-bin/carddisp.pl?gene= HS3ST3A1 |
| LAS1L | LAS1 Like Ribosome Biogenesis Factor | Protein Coding | 36 | GC0X M0654 40 | 0.558 48 | https://www.genecards .org/cgi-bin/carddisp.pl?gene= LAS1L |
| MYCL | MYCL Proto-Oncogene, BHLH Transcription Factor | Protein Coding | 36 | GC01M 039895 | 0.558 48 | https://www.genecards .org/cgi-bin/carddisp.pl?gene= MYCL |
| NASP | Nuclear Autoantigenic Sperm Protein | Protein Coding | 36 | GC01P 045583 | 0.558 48 | https://www.genecards .org/cgi-bin/carddisp.pl?gene= NASP |
| RNF25 | Ring Finger Protein 25 | Protein Coding | 36 | GC02M 218663 | 0.558 48 | https://www.genecards .org/cgi-bin/carddisp.pl?gene= RNF25 |
| SGTA | Small Glutamine Rich Tetratricopeptide Repeat Co-Chaperone Alpha | Protein Coding | 36 | GC19M 002754 | 0.558 48 | https://www.genecards .org/cgi-bin/carddisp.pl?gene= SGTA |
| LRTM1 | Leucine Rich Repeats And Transmembrane Domains 1 | Protein Coding | 35 | GC03M 054928 | 0.558 48 | https://www.genecards .org/cgi-bin/carddisp.pl?gene= LRTM1 |
| TBCC | Tubulin Folding Cofactor C | Protein Coding | 35 | GC06M 042744 | 0.558 48 | https://www.genecards .org/cgi-bin/carddisp.pl?gene= TBCC |
| CCNDBP 1 | Cyclin D1 Binding Protein 1 | Protein Coding | 34 | GC15P 043185 | 0.558 48 | https://www.genecards .org/cgi- |

| | | | | | | |
|---|---|---|---|---|---|---|
| | | | | | | bin/carddisp.pl?gene=CCNDBP1 |
| SUGP1 | SURP And G-Patch Domain Containing 1 | Protein Coding | 34 | GC19M019276 | 0.55848 | https://www.genecards.org/cgi-bin/carddisp.pl?gene=SUGP1 |
| UBL4A | Ubiquitin Like 4A | Protein Coding | 34 | GC0XM154483 | 0.55848 | https://www.genecards.org/cgi-bin/carddisp.pl?gene=UBL4A |
| PCID2 | PCI Domain Containing 2 | Protein Coding | 33 | GC13M113167 | 0.55848 | https://www.genecards.org/cgi-bin/carddisp.pl?gene=PCID2 |
| GRWD1 | Glutamate Rich WD Repeat Containing 1 | Protein Coding | 32 | GC19P048445 | 0.55848 | https://www.genecards.org/cgi-bin/carddisp.pl?gene=GRWD1 |
| SPART | Spartin | Protein Coding | 32 | GC13M036687 | 0.55848 | https://www.genecards.org/cgi-bin/carddisp.pl?gene=SPART |
| M1AP | Meiosis 1 Associated Protein | Protein Coding | 31 | GC02M074557 | 0.55848 | https://www.genecards.org/cgi-bin/carddisp.pl?gene=M1AP |
| CLECL1 | C-Type Lectin Like 1 | Pseudo gene | 30 | GC12M021099 | 0.55848 | https://www.genecards.org/cgi-bin/carddisp.pl?gene=CLECL1 |
| KRBOX4 | KRAB Box Domain Containing 4 | Protein Coding | 30 | GC0XP046587 | 0.55848 | https://www.genecards.org/cgi-bin/carddisp.pl?gene=KRBOX4 |
| CLEC2A | C-Type Lectin Domain Family 2 Member A | Protein Coding | 29 | GC12M021101 | 0.55848 | https://www.genecards.org/cgi-bin/carddisp.pl?gene=CLEC2A |
| MTARC1 | Mitochondrial Amidoxime Reducing Component 1 | Protein Coding | 26 | GC01P220788 | 0.55848 | https://www.genecards.org/cgi-bin/carddisp.pl?gene=MTARC1 |
| AARD | Alanine And Arginine Rich Domain Containing Protein | Protein Coding | 25 | GC08P116938 | 0.55848 | https://www.genecards.org/cgi-bin/carddisp.pl?gene=AARD |
| MIR595 | MicroRNA 595 | RNA Gene | 17 | GC07M158532 | 0.55848 | https://www.genecards.org/cgi-bin/carddisp.pl?gene=MIR595 |
| MIR1908 | MicroRNA 1908 | RNA Gene | 16 | GC11M061815 | 0.55848 | https://www.genecards.org/cgi-bin/carddisp.pl?gene=MIR1908 |

| | | | | | | |
|---|---|---|---|---|---|---|
| NR2F2-AS1 | NR2F2 Antisense RNA 1 | RNA Gene | 16 | GC15M117279 | 0.55848 | https://www.genecards.org/cgi-bin/carddisp.pl?gene=NR2F2-AS1 |
| TRBV19 | T Cell Receptor Beta Variable 19 | Protein Coding | 16 | GC07P148316 | 0.55848 | https://www.genecards.org/cgi-bin/carddisp.pl?gene=TRBV19 |
| MIR3614 | MicroRNA 3614 | RNA Gene | 14 | GC17M056891 | 0.55848 | https://www.genecards.org/cgi-bin/carddisp.pl?gene=MIR3614 |
| MIR1246 | MicroRNA 1246 | RNA Gene | 13 | GC02M176600 | 0.55848 | https://www.genecards.org/cgi-bin/carddisp.pl?gene=MIR1246 |
| MIR1972-2 | MicroRNA 1972-2 | RNA Gene | 13 | GC16P070030 | 0.55848 | https://www.genecards.org/cgi-bin/carddisp.pl?gene=MIR1972-2 |
| MIR5100 | MicroRNA 5100 | RNA Gene | 13 | GC10P043180 | 0.55848 | https://www.genecards.org/cgi-bin/carddisp.pl?gene=MIR5100 |
| MIR1972-1 | MicroRNA 1972-1 | RNA Gene | 12 | GC16M015697 | 0.55848 | https://www.genecards.org/cgi-bin/carddisp.pl?gene=MIR1972-1 |
| MIR371B | MicroRNA 371b | RNA Gene | 12 | GC19M053787 | 0.55848 | https://www.genecards.org/cgi-bin/carddisp.pl?gene=MIR371B |
| MIR4728 | MicroRNA 4728 | RNA Gene | 12 | GC17P039726 | 0.55848 | https://www.genecards.org/cgi-bin/carddisp.pl?gene=MIR4728 |
| UBA52P6 | Ubiquitin A-52 Residue Ribosomal Protein Fusion Product 1 Pseudogene 6 | Pseudo gene | 10 | GC09P022032 | 0.55848 | https://www.genecards.org/cgi-bin/carddisp.pl?gene=UBA52P6 |
| NPM1P7 | Nucleophosmin 1 Pseudogene 7 | Pseudo gene | 9 | GC12P021195 | 0.55848 | https://www.genecards.org/cgi-bin/carddisp.pl?gene=NPM1P7 |
| RNU2-3P | RNA, U2 Small Nuclear 3, Pseudogene | Pseudo gene | 9 | GC15P095745 | 0.55848 | https://www.genecards.org/cgi-bin/carddisp.pl?gene=RNU2-3P |
| AAVS1 | Adeno-Associated Virus Integration Site 1 | Genetic Locus | 6 | GC19U990001 | 0.55848 | https://www.genecards.org/cgi-bin/carddisp.pl?gene=AAVS1 |
| RPS14P10 | Ribosomal Protein S14 Pseudogene 10 | Pseudo gene | 6 | GC07P131665 | 0.55848 | https://www.genecards.org/cgi- |

| | | | | | | |
|---|---|---|---|---|---|---|
| | | | | | | bin/carddisp.pl?gene=RPS14P10 |
| RPL39P10 | Ribosomal Protein L39 Pseudogene 10 | Pseudo gene | 5 | GC01M239053 | 0.55848 | https://www.genecards.org/cgi-bin/carddisp.pl?gene=RPL39P10 |
| IBD15 | Inflammatory Bowel Disease-15 | Genetic Locus | 2 | GC10U901075 | 0.55848 | https://www.genecards.org/cgi-bin/carddisp.pl?gene=IBD15 |
| IBD18 | Inflammatory Bowel Disease-18 | Genetic Locus | 2 | GC05U901053 | 0.55848 | https://www.genecards.org/cgi-bin/carddisp.pl?gene=IBD18 |
| POMGNT2 | Protein O-Linked Mannose N-Acetylglucosaminyltransferase 2 (Beta 1,4-) | Protein Coding | 36 | GC03M043121 | 0.553841 | https://www.genecards.org/cgi-bin/carddisp.pl?gene=POMGNT2 |
| DCC | DCC Netrin 1 Receptor | Protein Coding | 45 | GC18P052340 | 0.548455 | https://www.genecards.org/cgi-bin/carddisp.pl?gene=DCC |
| THSD7A | Thrombospondin Type 1 Domain Containing 7A | Protein Coding | 36 | GC07M011371 | 0.548455 | https://www.genecards.org/cgi-bin/carddisp.pl?gene=THSD7A |
| BCAT1 | Branched Chain Amino Acid Transaminase 1 | Protein Coding | 44 | GC12M024732 | 0.545865 | https://www.genecards.org/cgi-bin/carddisp.pl?gene=BCAT1 |
| FGF11 | Fibroblast Growth Factor 11 | Protein Coding | 38 | GC17P011161 | 0.53919 | https://www.genecards.org/cgi-bin/carddisp.pl?gene=FGF11 |
| BDKRB1 | Bradykinin Receptor B1 | Protein Coding | 41 | GC14P096296 | 0.539168 | https://www.genecards.org/cgi-bin/carddisp.pl?gene=BDKRB1 |
| SLC29A3 | Solute Carrier Family 29 Member 3 | Protein Coding | 43 | GC10P071320 | 0.538765 | https://www.genecards.org/cgi-bin/carddisp.pl?gene=SLC29A3 |
| EEF2 | Eukaryotic Translation Elongation Factor 2 | Protein Coding | 47 | GC19M003976 | 0.537908 | https://www.genecards.org/cgi-bin/carddisp.pl?gene=EEF2 |
| PLA1A | Phospholipase A1 Member A | Protein Coding | 38 | GC03P119597 | 0.518574 | https://www.genecards.org/cgi-bin/carddisp.pl?gene=PLA1A |
| MLXIPL | MLX Interacting Protein Like | Protein Coding | 41 | GC07M073593 | 0.512319 | https://www.genecards.org/cgi-bin/carddisp.pl?gene=MLXIPL |

| | | | | | | |
|---|---|---|---|---|---|---|
| TFAP2A | Transcription Factor AP-2 Alpha | Protein Coding | 45 | GC06M 010393 | 0.511 037 | https://www.genecards .org/cgi-bin/carddisp.pl?gene= TFAP2A |
| SLC25A1 | Solute Carrier Family 25 Member 1 | Protein Coding | 45 | GC22M 019355 | 0.501 772 | https://www.genecards .org/cgi-bin/carddisp.pl?gene= SLC25A1 |
| DLAT | Dihydrolipoamide S-Acetyltransferase | Protein Coding | 44 | GC11P 112026 | 0.501 772 | https://www.genecards .org/cgi-bin/carddisp.pl?gene= DLAT |
| PROX1 | Prospero Homeobox 1 | Protein Coding | 42 | GC01P 213983 | 0.501 772 | https://www.genecards .org/cgi-bin/carddisp.pl?gene= PROX1 |
| CHD3 | Chromodomain Helicase DNA Binding Protein 3 | Protein Coding | 41 | GC17P 011184 | 0.501 772 | https://www.genecards .org/cgi-bin/carddisp.pl?gene= CHD3 |
| RPS9 | Ribosomal Protein S9 | Protein Coding | 41 | GC19P 069272 | 0.501 772 | https://www.genecards .org/cgi-bin/carddisp.pl?gene= RPS9 |
| SLC25A6 | Solute Carrier Family 25 Member 6 | Protein Coding | 40 | GC0X M0013 86 | 0.501 772 | https://www.genecards .org/cgi-bin/carddisp.pl?gene= SLC25A6 |
| HYDIN | HYDIN Axonemal Central Pair Apparatus Protein | Protein Coding | 37 | GC16M 070920 | 0.501 772 | https://www.genecards .org/cgi-bin/carddisp.pl?gene= HYDIN |
| MARS1 | Methionyl-TRNA Synthetase 1 | Protein Coding | 36 | GC12P 057476 | 0.501 772 | https://www.genecards .org/cgi-bin/carddisp.pl?gene= MARS1 |
| MZB1 | Marginal Zone B And B1 Cell Specific Protein | Protein Coding | 33 | GC05M 139387 | 0.501 772 | https://www.genecards .org/cgi-bin/carddisp.pl?gene= MZB1 |
| VPS13C | Vacuolar Protein Sorting 13 Homolog C | Protein Coding | 33 | GC15M 061852 | 0.501 772 | https://www.genecards .org/cgi-bin/carddisp.pl?gene= VPS13C |
| CAMK2 G | Calcium/Calmodulin Dependent Protein Kinase II Gamma | Protein Coding | 46 | GC10M 073812 | 0.495 198 | https://www.genecards .org/cgi-bin/carddisp.pl?gene= CAMK2G |
| CD3E | CD3 Epsilon Subunit Of T-Cell Receptor Complex | Protein Coding | 45 | GC11P 118304 | 0.495 198 | https://www.genecards .org/cgi-bin/carddisp.pl?gene= CD3E |
| RABEP2 | Rabaptin, RAB GTPase Binding Effector Protein 2 | Protein Coding | 33 | GC16M 036985 | 0.491 658 | https://www.genecards .org/cgi- |

| | | | | | | |
|---|---|---|---|---|---|---|
| | | | | | | bin/carddisp.pl?gene=RABEP2 |
| CEACAM3 | CEA Cell Adhesion Molecule 3 | Protein Coding | 41 | GC19P041796 | 0.488322 | https://www.genecards.org/cgi-bin/carddisp.pl?gene=CEACAM3 |
| CSNK2A1 | Casein Kinase 2 Alpha 1 | Protein Coding | 48 | GC20M000472 | 0.474901 | https://www.genecards.org/cgi-bin/carddisp.pl?gene=CSNK2A1 |
| ACACA | Acetyl-CoA Carboxylase Alpha | Protein Coding | 46 | GC17M037084 | 0.474901 | https://www.genecards.org/cgi-bin/carddisp.pl?gene=ACACA |
| JUP | Junction Plakoglobin | Protein Coding | 46 | GC17M041754 | 0.474901 | https://www.genecards.org/cgi-bin/carddisp.pl?gene=JUP |
| NR1H4 | Nuclear Receptor Subfamily 1 Group H Member 4 | Protein Coding | 46 | GC12P100473 | 0.474901 | https://www.genecards.org/cgi-bin/carddisp.pl?gene=NR1H4 |
| USP9X | Ubiquitin Specific Peptidase 9 X-Linked | Protein Coding | 46 | GC0XP041085 | 0.474901 | https://www.genecards.org/cgi-bin/carddisp.pl?gene=USP9X |
| AKR1B1 | Aldo-Keto Reductase Family 1 Member B | Protein Coding | 45 | GC07M134442 | 0.474901 | https://www.genecards.org/cgi-bin/carddisp.pl?gene=AKR1B1 |
| ETV6 | ETS Variant Transcription Factor 6 | Protein Coding | 45 | GC12P011649 | 0.474901 | https://www.genecards.org/cgi-bin/carddisp.pl?gene=ETV6 |
| PPARD | Peroxisome Proliferator Activated Receptor Delta | Protein Coding | 45 | GC06P083822 | 0.474901 | https://www.genecards.org/cgi-bin/carddisp.pl?gene=PPARD |
| CNTN2 | Contactin 2 | Protein Coding | 44 | GC01P205043 | 0.474901 | https://www.genecards.org/cgi-bin/carddisp.pl?gene=CNTN2 |
| CS | Citrate Synthase | Protein Coding | 43 | GC12M056271 | 0.474901 | https://www.genecards.org/cgi-bin/carddisp.pl?gene=CS |
| BCL10 | BCL10 Immune Signaling Adaptor | Protein Coding | 42 | GC01M085265 | 0.474901 | https://www.genecards.org/cgi-bin/carddisp.pl?gene=BCL10 |
| MTTP | Microsomal Triglyceride Transfer Protein | Protein Coding | 42 | GC04P099563 | 0.474901 | https://www.genecards.org/cgi-bin/carddisp.pl?gene=MTTP |

| | | | | | | |
|---|---|---|---|---|---|---|
| MEGF10 | Multiple EGF Like Domains 10 | Protein Coding | 41 | GC05P 127230 | 0.474 901 | https://www.genecards.org/cgi-bin/carddisp.pl?gene=MEGF10 |
| COLQ | Collagen Like Tail Subunit Of Asymmetric Acetylcholinesterase | Protein Coding | 40 | GC03M 020745 | 0.474 901 | https://www.genecards.org/cgi-bin/carddisp.pl?gene=COLQ |
| PPIG | Peptidylprolyl Isomerase G | Protein Coding | 40 | GC02P 169584 | 0.474 901 | https://www.genecards.org/cgi-bin/carddisp.pl?gene=PPIG |
| SORBS1 | Sorbin And SH3 Domain Containing 1 | Protein Coding | 40 | GC10M 095311 | 0.474 901 | https://www.genecards.org/cgi-bin/carddisp.pl?gene=SORBS1 |
| GNPTG | N-Acetylglucosamine-1-Phosphate Transferase Subunit Gamma | Protein Coding | 39 | GC16P 001351 | 0.474 901 | https://www.genecards.org/cgi-bin/carddisp.pl?gene=GNPTG |
| RC3H1 | Ring Finger And CCCH-Type Domains 1 | Protein Coding | 35 | GC01M 173931 | 0.474 901 | https://www.genecards.org/cgi-bin/carddisp.pl?gene=RC3H1 |
| SNTG1 | Syntrophin Gamma 1 | Protein Coding | 33 | GC08P 049909 | 0.474 901 | https://www.genecards.org/cgi-bin/carddisp.pl?gene=SNTG1 |
| LOC1019 29710 | Uncharacterized LOC101929710 | RNA Gene | 13 | GC05P 095962 | 0.474 901 | https://www.genecards.org/cgi-bin/carddisp.pl?gene=LOC101929710 |
| PPP3CA | Protein Phosphatase 3 Catalytic Subunit Alpha | Protein Coding | 50 | GC04M 101024 | 0.473 039 | https://www.genecards.org/cgi-bin/carddisp.pl?gene=PPP3CA |
| RHBDF2 | Rhomboid 5 Homolog 2 | Protein Coding | 42 | GC17M 076470 | 0.468 146 | https://www.genecards.org/cgi-bin/carddisp.pl?gene=RHBDF2 |
| SRGN | Serglycin | Protein Coding | 38 | GC10P 069088 | 0.468 146 | https://www.genecards.org/cgi-bin/carddisp.pl?gene=SRGN |
| RNU11 | RNA, U11 Small Nuclear | RNA Gene | 17 | GC01P 028797 | 0.468 146 | https://www.genecards.org/cgi-bin/carddisp.pl?gene=RNU11 |
| CLTC | Clathrin Heavy Chain | Protein Coding | 45 | GC17P 059619 | 0.467 47 | https://www.genecards.org/cgi-bin/carddisp.pl?gene=CLTC |

| | | | | | | |
|---|---|---|---|---|---|---|
| KCNA2 | Potassium Voltage-Gated Channel Subfamily A Member 2 | Protein Coding | 45 | GC01M 110519 | 0.466 486 | https://www.genecards .org/cgi-bin/carddisp.pl?gene= KCNA2 |
| CGB5 | Chorionic Gonadotropin Subunit Beta 5 | Protein Coding | 33 | GC19P 049043 | 0.466 486 | https://www.genecards .org/cgi-bin/carddisp.pl?gene= CGB5 |
| AQP9 | Aquaporin 9 | Protein Coding | 41 | GC15P 058138 | 0.458 626 | https://www.genecards .org/cgi-bin/carddisp.pl?gene= AQP9 |
| RPL4 | Ribosomal Protein L4 | Protein Coding | 40 | GC15M 066498 | 0.452 445 | https://www.genecards .org/cgi-bin/carddisp.pl?gene= RPL4 |
| YWHAG | Tyrosine 3-Monooxygenase/Tryptophan 5-Monooxygenase Activation Protein Gamma | Protein Coding | 47 | GC07M 076839 | 0.438 759 | https://www.genecards .org/cgi-bin/carddisp.pl?gene= YWHAG |
| CNTFR | Ciliary Neurotrophic Factor Receptor | Protein Coding | 42 | GC09M 034551 | 0.438 759 | https://www.genecards .org/cgi-bin/carddisp.pl?gene= CNTFR |
| HMGN2 | High Mobility Group Nucleosomal Binding Domain 2 | Protein Coding | 39 | GC01P 026473 | 0.438 759 | https://www.genecards .org/cgi-bin/carddisp.pl?gene= HMGN2 |
| TSPAN32 | Tetraspanin 32 | Protein Coding | 35 | GC11P 002302 | 0.438 759 | https://www.genecards .org/cgi-bin/carddisp.pl?gene= TSPAN32 |
| PPP2CA | Protein Phosphatase 2 Catalytic Subunit Alpha | Protein Coding | 48 | GC05M 134194 | 0.429 069 | https://www.genecards .org/cgi-bin/carddisp.pl?gene= PPP2CA |
| DUSP2 | Dual Specificity Phosphatase 2 | Protein Coding | 40 | GC02M 098251 | 0.429 069 | https://www.genecards .org/cgi-bin/carddisp.pl?gene= DUSP2 |
| PTPN18 | Protein Tyrosine Phosphatase Non-Receptor Type 18 | Protein Coding | 38 | GC02P 130356 | 0.429 069 | https://www.genecards .org/cgi-bin/carddisp.pl?gene= PTPN18 |
| RIDA | Reactive Intermediate Imine Deaminase A Homolog | Protein Coding | 29 | GC08M 098103 | 0.429 069 | https://www.genecards .org/cgi-bin/carddisp.pl?gene= RIDA |
| CTSV | Cathepsin V | Protein Coding | 40 | GC09M 097029 | 0.427 418 | https://www.genecards .org/cgi-bin/carddisp.pl?gene= CTSV |
| ADORA1 | Adenosine A1 Receptor | Protein Coding | 45 | GC01P 203090 | 0.425 08 | https://www.genecards .org/cgi- |

| | | | | | | |
|---|---|---|---|---|---|---|
| | | | | | | bin/carddisp.pl?gene=ADORA1 |
| KLK3 | Kallikrein Related Peptidase 3 | Protein Coding | 45 | GC19P050854 | 0.408265 | https://www.genecards.org/cgi-bin/carddisp.pl?gene=KLK3 |
| IGHV3-33 | Immunoglobulin Heavy Variable 3-33 | Protein Coding | 16 | GC14M112850 | 0.408265 | https://www.genecards.org/cgi-bin/carddisp.pl?gene=IGHV3-33 |
| AP2A1 | Adaptor Related Protein Complex 2 Subunit Alpha 1 | Protein Coding | 38 | GC19P067127 | 0.402623 | https://www.genecards.org/cgi-bin/carddisp.pl?gene=AP2A1 |
| NR4A3 | Nuclear Receptor Subfamily 4 Group A Member 3 | Protein Coding | 43 | GC09P099821 | 0.401341 | https://www.genecards.org/cgi-bin/carddisp.pl?gene=NR4A3 |
| ITPR2 | Inositol 1,4,5-Trisphosphate Receptor Type 2 | Protein Coding | 45 | GC12M026336 | 0.397066 | https://www.genecards.org/cgi-bin/carddisp.pl?gene=ITPR2 |
| NPR2 | Natriuretic Peptide Receptor 2 | Protein Coding | 48 | GC09P047173 | 0.387662 | https://www.genecards.org/cgi-bin/carddisp.pl?gene=NPR2 |
| MASP1 | MBL Associated Serine Protease 1 | Protein Coding | 47 | GC03M187216 | 0.380906 | https://www.genecards.org/cgi-bin/carddisp.pl?gene=MASP1 |
| SOS1 | SOS Ras/Rac Guanine Nucleotide Exchange Factor 1 | Protein Coding | 46 | GC02M039031 | 0.380906 | https://www.genecards.org/cgi-bin/carddisp.pl?gene=SOS1 |
| FZD5 | Frizzled Class Receptor 5 | Protein Coding | 45 | GC02M207762 | 0.380906 | https://www.genecards.org/cgi-bin/carddisp.pl?gene=FZD5 |
| EHMT1 | Euchromatic Histone Lysine Methyltransferase 1 | Protein Coding | 44 | GC09P137618 | 0.380906 | https://www.genecards.org/cgi-bin/carddisp.pl?gene=EHMT1 |
| RALBP1 | RalA Binding Protein 1 | Protein Coding | 43 | GC18P009465 | 0.380906 | https://www.genecards.org/cgi-bin/carddisp.pl?gene=RALBP1 |
| HDAC10 | Histone Deacetylase 10 | Protein Coding | 42 | GC22M050245 | 0.380906 | https://www.genecards.org/cgi-bin/carddisp.pl?gene=HDAC10 |
| NRIP1 | Nuclear Receptor Interacting Protein 1 | Protein Coding | 42 | GC21M014961 | 0.380906 | https://www.genecards.org/cgi-bin/carddisp.pl?gene=NRIP1 |

| | | | | | | |
|---|---|---|---|---|---|---|
| SETD7 | SET Domain Containing 7, Histone Lysine Methyltransferase | Protein Coding | 42 | GC04M 139495 | 0.380 906 | https://www.genecards.org/cgi-bin/carddisp.pl?gene=SETD7 |
| SP100 | SP100 Nuclear Antigen | Protein Coding | 40 | GC02P 230415 | 0.380 906 | https://www.genecards.org/cgi-bin/carddisp.pl?gene=SP100 |
| ENAM | Enamelin | Protein Coding | 39 | GC04P 070628 | 0.380 906 | https://www.genecards.org/cgi-bin/carddisp.pl?gene=ENAM |
| WWTR1 | WW Domain Containing Transcription Regulator 1 | Protein Coding | 39 | GC03M 149517 | 0.380 906 | https://www.genecards.org/cgi-bin/carddisp.pl?gene=WWTR1 |
| MRGPR X2 | MAS Related GPR Family Member X2 | Protein Coding | 36 | GC11M 019077 | 0.380 906 | https://www.genecards.org/cgi-bin/carddisp.pl?gene=MRGPRX2 |
| SAMD9 | Sterile Alpha Motif Domain Containing 9 | Protein Coding | 36 | GC07M 093099 | 0.380 906 | https://www.genecards.org/cgi-bin/carddisp.pl?gene=SAMD9 |
| JPH4 | Junctophilin 4 | Protein Coding | 34 | GC14M 023568 | 0.380 906 | https://www.genecards.org/cgi-bin/carddisp.pl?gene=JPH4 |
| OTUD1 | OTU Deubiquitinase 1 | Protein Coding | 32 | GC10P 023439 | 0.380 906 | https://www.genecards.org/cgi-bin/carddisp.pl?gene=OTUD1 |
| MIRLET 7I | MicroRNA Let-7i | RNA Gene | 21 | GC12P 062606 | 0.380 906 | https://www.genecards.org/cgi-bin/carddisp.pl?gene=MIRLET7I |
| MOXD2P | Monooxygenase DBH Like 2, Pseudogene | Pseudo gene | 14 | GC07M 142254 | 0.380 906 | https://www.genecards.org/cgi-bin/carddisp.pl?gene=MOXD2P |
| LOC1107 40340 | CYP2D6 Promoter | Biologi cal Region | 2 | GC22P 043272 | 0.380 906 | https://www.genecards.org/cgi-bin/carddisp.pl?gene=LOC110740340 |
| HCST | Hematopoietic Cell Signal Transducer | Protein Coding | 35 | GC19P 066557 | 0.376 471 | https://www.genecards.org/cgi-bin/carddisp.pl?gene=HCST |
| ABCA4 | ATP Binding Cassette Subfamily A Member 4 | Protein Coding | 45 | GC01M 093992 | 0.365 205 | https://www.genecards.org/cgi-bin/carddisp.pl?gene=ABCA4 |
| CD37 | CD37 Molecule | Protein Coding | 41 | GC19P 049335 | 0.365 205 | https://www.genecards.org/cgi- |

| | | | | | | |
|---|---|---|---|---|---|---|
| | | | | | | bin/carddisp.pl?gene=CD37 |
| TPPP3 | Tubulin Polymerization Promoting Protein Family Member 3 | Protein Coding | 36 | GC16M067389 | 0.365205 | https://www.genecards.org/cgi-bin/carddisp.pl?gene=TPPP3 |
| KIR2DS5 | Killer Cell Immunoglobulin Like Receptor, Two Ig Domains And Short Cytoplasmic Tail 5 | Protein Coding | 23 | GC19Mr00079 | 0.355146 | https://www.genecards.org/cgi-bin/carddisp.pl?gene=KIR2DS5 |
| USP4 | Ubiquitin Specific Peptidase 4 | Protein Coding | 42 | GC03M049277 | 0.322824 | https://www.genecards.org/cgi-bin/carddisp.pl?gene=USP4 |
| PRKD1 | Protein Kinase D1 | Protein Coding | 48 | GC14M029576 | 0.307566 | https://www.genecards.org/cgi-bin/carddisp.pl?gene=PRKD1 |
| RORA | RAR Related Orphan Receptor A | Protein Coding | 46 | GC15M060488 | 0.307566 | https://www.genecards.org/cgi-bin/carddisp.pl?gene=RORA |
| RPS2 | Ribosomal Protein S2 | Protein Coding | 41 | GC16M007099 | 0.307566 | https://www.genecards.org/cgi-bin/carddisp.pl?gene=RPS2 |
| SOX13 | SRY-Box Transcription Factor 13 | Protein Coding | 40 | GC01P204074 | 0.307566 | https://www.genecards.org/cgi-bin/carddisp.pl?gene=SOX13 |
| CAPNS1 | Calpain Small Subunit 1 | Protein Coding | 39 | GC19P066563 | 0.307566 | https://www.genecards.org/cgi-bin/carddisp.pl?gene=CAPNS1 |
| ATP6V1G2 | ATPase H+ Transporting V1 Subunit G2 | Protein Coding | 37 | GC06M065955 | 0.307566 | https://www.genecards.org/cgi-bin/carddisp.pl?gene=ATP6V1G2 |
| SLC25A16 | Solute Carrier Family 25 Member 16 | Protein Coding | 37 | GC10M068477 | 0.307566 | https://www.genecards.org/cgi-bin/carddisp.pl?gene=SLC25A16 |
| PLD5 | Phospholipase D Family Member 5 | Protein Coding | 35 | GC01M242082 | 0.307566 | https://www.genecards.org/cgi-bin/carddisp.pl?gene=PLD5 |
| ZNF330 | Zinc Finger Protein 330 | Protein Coding | 35 | GC04P141220 | 0.307566 | https://www.genecards.org/cgi-bin/carddisp.pl?gene=ZNF330 |
| TMEM258 | Transmembrane Protein 258 | Protein Coding | 30 | GC11M061768 | 0.307566 | https://www.genecards.org/cgi- |

| | | | | | | |
|---|---|---|---|---|---|---|
| | | | | | | bin/carddisp.pl?gene=TMEM258 |
| CRLF1 | Cytokine Receptor Like Factor 1 | Protein Coding | 42 | GC19M018572 | 0.30223 | https://www.genecards.org/cgi-bin/carddisp.pl?gene=CRLF1 |
| ADAM33 | ADAM Metallopeptidase Domain 33 | Protein Coding | 37 | GC20M003669 | 0.30223 | https://www.genecards.org/cgi-bin/carddisp.pl?gene=ADAM33 |
| HMGA1 | High Mobility Group AT-Hook 1 | Protein Coding | 44 | GC06P083808 | 0.279838 | https://www.genecards.org/cgi-bin/carddisp.pl?gene=HMGA1 |
| CD7 | CD7 Molecule | Protein Coding | 38 | GC17M082314 | 0.279838 | https://www.genecards.org/cgi-bin/carddisp.pl?gene=CD7 |
| VEGFB | Vascular Endothelial Growth Factor B | Protein Coding | 42 | GC11P064234 | 0.278854 | https://www.genecards.org/cgi-bin/carddisp.pl?gene=VEGFB |
| OMD | Osteomodulin | Protein Coding | 39 | GC09M094205 | 0.278854 | https://www.genecards.org/cgi-bin/carddisp.pl?gene=OMD |
| DPP9 | Dipeptidyl Peptidase 9 | Protein Coding | 41 | GC19M004675 | 0.264812 | https://www.genecards.org/cgi-bin/carddisp.pl?gene=DPP9 |
| NCL | Nucleolin | Protein Coding | 41 | GC02M231453 | 0.264812 | https://www.genecards.org/cgi-bin/carddisp.pl?gene=NCL |
| MAP2K2 | Mitogen-Activated Protein Kinase Kinase 2 | Protein Coding | 50 | GC19M004090 | 0.251126 | https://www.genecards.org/cgi-bin/carddisp.pl?gene=MAP2K2 |
| ADAM9 | ADAM Metallopeptidase Domain 9 | Protein Coding | 47 | GC08P038996 | 0.251126 | https://www.genecards.org/cgi-bin/carddisp.pl?gene=ADAM9 |
| CHRM2 | Cholinergic Receptor Muscarinic 2 | Protein Coding | 46 | GC07P136868 | 0.251126 | https://www.genecards.org/cgi-bin/carddisp.pl?gene=CHRM2 |
| PRKCI | Protein Kinase C Iota | Protein Coding | 46 | GC03P170222 | 0.251126 | https://www.genecards.org/cgi-bin/carddisp.pl?gene=PRKCI |
| ADORA2B | Adenosine A2b Receptor | Protein Coding | 45 | GC17P015851 | 0.251126 | https://www.genecards.org/cgi-bin/carddisp.pl?gene=ADORA2B |

| | | | | | | |
|---|---|---|---|---|---|---|
| APAF1 | Apoptotic Peptidase Activating Factor 1 | Protein Coding | 45 | GC12P 098645 | 0.251 126 | https://www.genecards.org/cgi-bin/carddisp.pl?gene=APAF1 |
| ATF6 | Activating Transcription Factor 6 | Protein Coding | 45 | GC01P 161766 | 0.251 126 | https://www.genecards.org/cgi-bin/carddisp.pl?gene=ATF6 |
| CD3D | CD3 Delta Subunit Of T-Cell Receptor Complex | Protein Coding | 45 | GC11M 118338 | 0.251 126 | https://www.genecards.org/cgi-bin/carddisp.pl?gene=CD3D |
| FH | Fumarate Hydratase | Protein Coding | 45 | GC01M 241499 | 0.251 126 | https://www.genecards.org/cgi-bin/carddisp.pl?gene=FH |
| PTPRD | Protein Tyrosine Phosphatase Receptor Type D | Protein Coding | 45 | GC09M 008307 | 0.251 126 | https://www.genecards.org/cgi-bin/carddisp.pl?gene=PTPRD |
| CTSZ | Cathepsin Z | Protein Coding | 44 | GC20M 058995 | 0.251 126 | https://www.genecards.org/cgi-bin/carddisp.pl?gene=CTSZ |
| KMO | Kynurenine 3-Monooxygenase | Protein Coding | 44 | GC01P 241532 | 0.251 126 | https://www.genecards.org/cgi-bin/carddisp.pl?gene=KMO |
| COL7A1 | Collagen Type VII Alpha 1 Chain | Protein Coding | 43 | GC03M 048564 | 0.251 126 | https://www.genecards.org/cgi-bin/carddisp.pl?gene=COL7A1 |
| PIAS1 | Protein Inhibitor Of Activated STAT 1 | Protein Coding | 43 | GC15P 068054 | 0.251 126 | https://www.genecards.org/cgi-bin/carddisp.pl?gene=PIAS1 |
| TNFRSF21 | TNF Receptor Superfamily Member 21 | Protein Coding | 43 | GC06M 047231 | 0.251 126 | https://www.genecards.org/cgi-bin/carddisp.pl?gene=TNFRSF21 |
| CYP3A7 | Cytochrome P450 Family 3 Subfamily A Member 7 | Protein Coding | 42 | GC07M 099705 | 0.251 126 | https://www.genecards.org/cgi-bin/carddisp.pl?gene=CYP3A7 |
| GRK6 | G Protein-Coupled Receptor Kinase 6 | Protein Coding | 42 | GC05P 177403 | 0.251 126 | https://www.genecards.org/cgi-bin/carddisp.pl?gene=GRK6 |
| HBA1 | Hemoglobin Subunit Alpha 1 | Protein Coding | 42 | GC16P 011598 | 0.251 126 | https://www.genecards.org/cgi-bin/carddisp.pl?gene=HBA1 |
| ITGA11 | Integrin Subunit Alpha 11 | Protein Coding | 42 | GC15M 068296 | 0.251 126 | https://www.genecards.org/cgi- |

| | | | | | | |
|---|---|---|---|---|---|---|
| | | | | | | bin/carddisp.pl?gene=ITGA11 |
| PSMD2 | Proteasome 26S Subunit Ubiquitin Receptor, Non-ATPase 2 | Protein Coding | 42 | GC03P184298 | 0.251126 | https://www.genecards.org/cgi-bin/carddisp.pl?gene=PSMD2 |
| DBT | Dihydrolipoamide Branched Chain Transacylase E2 | Protein Coding | 41 | GC01M100186 | 0.251126 | https://www.genecards.org/cgi-bin/carddisp.pl?gene=DBT |
| DPP10 | Dipeptidyl Peptidase Like 10 | Protein Coding | 41 | GC02P114442 | 0.251126 | https://www.genecards.org/cgi-bin/carddisp.pl?gene=DPP10 |
| ENDOG | Endonuclease G | Protein Coding | 41 | GC09P128818 | 0.251126 | https://www.genecards.org/cgi-bin/carddisp.pl?gene=ENDOG |
| RGS2 | Regulator Of G Protein Signaling 2 | Protein Coding | 41 | GC01P192809 | 0.251126 | https://www.genecards.org/cgi-bin/carddisp.pl?gene=RGS2 |
| SDCCAG8 | SHH Signaling And Ciliogenesis Regulator SDCCAG8 | Protein Coding | 41 | GC01P243255 | 0.251126 | https://www.genecards.org/cgi-bin/carddisp.pl?gene=SDCCAG8 |
| GREM2 | Gremlin 2, DAN Family BMP Antagonist | Protein Coding | 40 | GC01M240489 | 0.251126 | https://www.genecards.org/cgi-bin/carddisp.pl?gene=GREM2 |
| METTL3 | Methyltransferase 3, N6-Adenosine-Methyltransferase Complex Catalytic Subunit | Protein Coding | 40 | GC14M021498 | 0.251126 | https://www.genecards.org/cgi-bin/carddisp.pl?gene=METTL3 |
| RGS7 | Regulator Of G Protein Signaling 7 | Protein Coding | 40 | GC01M240767 | 0.251126 | https://www.genecards.org/cgi-bin/carddisp.pl?gene=RGS7 |
| WDFY3 | WD Repeat And FYVE Domain Containing 3 | Protein Coding | 40 | GC04M084669 | 0.251126 | https://www.genecards.org/cgi-bin/carddisp.pl?gene=WDFY3 |
| ZBTB17 | Zinc Finger And BTB Domain Containing 17 | Protein Coding | 40 | GC01M015943 | 0.251126 | https://www.genecards.org/cgi-bin/carddisp.pl?gene=ZBTB17 |
| ECE2 | Endothelin Converting Enzyme 2 | Protein Coding | 39 | GC03P184276 | 0.251126 | https://www.genecards.org/cgi-bin/carddisp.pl?gene=ECE2 |
| TNNI3K | TNNI3 Interacting Kinase | Protein Coding | 39 | GC01P074235 | 0.251126 | https://www.genecards.org/cgi- |

| | | | | | | |
|---|---|---|---|---|---|---|
| | | | | | | bin/carddisp.pl?gene=TNNI3K |
| ZNF423 | Zinc Finger Protein 423 | Protein Coding | 39 | GC16M049487 | 0.251126 | https://www.genecards.org/cgi-bin/carddisp.pl?gene=ZNF423 |
| AGMAT | Agmatinase | Protein Coding | 38 | GC01M015571 | 0.251126 | https://www.genecards.org/cgi-bin/carddisp.pl?gene=AGMAT |
| CD207 | CD207 Molecule | Protein Coding | 38 | GC02M070830 | 0.251126 | https://www.genecards.org/cgi-bin/carddisp.pl?gene=CD207 |
| CEP170 | Centrosomal Protein 170 | Protein Coding | 38 | GC01M243124 | 0.251126 | https://www.genecards.org/cgi-bin/carddisp.pl?gene=CEP170 |
| OPN3 | Opsin 3 | Protein Coding | 38 | GC01M241590 | 0.251126 | https://www.genecards.org/cgi-bin/carddisp.pl?gene=OPN3 |
| PDPR | Pyruvate Dehydrogenase Phosphatase Regulatory Subunit | Protein Coding | 38 | GC16P070113 | 0.251126 | https://www.genecards.org/cgi-bin/carddisp.pl?gene=PDPR |
| TNFRSF19 | TNF Receptor Superfamily Member 19 | Protein Coding | 38 | GC13P023570 | 0.251126 | https://www.genecards.org/cgi-bin/carddisp.pl?gene=TNFRSF19 |
| ATRN | Attractin | Protein Coding | 37 | GC20P003471 | 0.251126 | https://www.genecards.org/cgi-bin/carddisp.pl?gene=ATRN |
| CHML | CHM Like Rab Escort Protein | Protein Coding | 37 | GC01M241628 | 0.251126 | https://www.genecards.org/cgi-bin/carddisp.pl?gene=CHML |
| KLHL12 | Kelch Like Family Member 12 | Protein Coding | 37 | GC01M202891 | 0.251126 | https://www.genecards.org/cgi-bin/carddisp.pl?gene=KLHL12 |
| PLA2G2E | Phospholipase A2 Group IIE | Protein Coding | 37 | GC01M019920 | 0.251126 | https://www.genecards.org/cgi-bin/carddisp.pl?gene=PLA2G2E |
| SRRM1 | Serine And Arginine Repetitive Matrix 1 | Protein Coding | 37 | GC01P024631 | 0.251126 | https://www.genecards.org/cgi-bin/carddisp.pl?gene=SRRM1 |
| NRAP | Nebulin Related Anchoring Protein | Protein Coding | 36 | GC10M113588 | 0.251126 | https://www.genecards.org/cgi-bin/carddisp.pl?gene=NRAP |

| | | | | | | |
|---|---|---|---|---|---|---|
| RTCB | RNA 2',3'-Cyclic Phosphate And 5'-OH Ligase | Protein Coding | 35 | GC22M 032387 | 0.251 126 | https://www.genecards.org/cgi-bin/carddisp.pl?gene=RTCB |
| CCDC91 | Coiled-Coil Domain Containing 91 | Protein Coding | 34 | GC12P 028133 | 0.251 126 | https://www.genecards.org/cgi-bin/carddisp.pl?gene=CCDC91 |
| METTL7 B | Methyltransferase Like 7B | Protein Coding | 34 | GC12P 055681 | 0.251 126 | https://www.genecards.org/cgi-bin/carddisp.pl?gene=METTL7B |
| WDR64 | WD Repeat Domain 64 | Protein Coding | 30 | GC01P 241652 | 0.251 126 | https://www.genecards.org/cgi-bin/carddisp.pl?gene=WDR64 |
| MCCD1 | Mitochondrial Coiled-Coil Domain 1 | Protein Coding | 29 | GC06P 031528 | 0.251 126 | https://www.genecards.org/cgi-bin/carddisp.pl?gene=MCCD1 |
| OVOS2 | Alpha-2-Macroglobulin Like 1 Pseudogene | Pseudo gene | 23 | GC12M 031111 | 0.251 126 | https://www.genecards.org/cgi-bin/carddisp.pl?gene=OVOS2 |
| BCAR1 | BCAR1 Scaffold Protein, Cas Family Member | Protein Coding | 42 | GC16M 075228 | 0.241 437 | https://www.genecards.org/cgi-bin/carddisp.pl?gene=BCAR1 |
| DPP8 | Dipeptidyl Peptidase 8 | Protein Coding | 38 | GC15M 065442 | 0.241 437 | https://www.genecards.org/cgi-bin/carddisp.pl?gene=DPP8 |
| C1QTNF 3 | C1q And TNF Related 3 | Protein Coding | 36 | GC05M 034017 | 0.241 437 | https://www.genecards.org/cgi-bin/carddisp.pl?gene=C1QTNF3 |
| CFL1 | Cofilin 1 | Protein Coding | 44 | GC11M 065823 | 0.214 991 | https://www.genecards.org/cgi-bin/carddisp.pl?gene=CFL1 |
| CDH2 | Cadherin 2 | Protein Coding | 50 | GC18M 027950 | 0.213 709 | https://www.genecards.org/cgi-bin/carddisp.pl?gene=CDH2 |
| BMPR1A | Bone Morphogenetic Protein Receptor Type 1A | Protein Coding | 48 | GC10P 092414 | 0.213 709 | https://www.genecards.org/cgi-bin/carddisp.pl?gene=BMPR1A |
| XPO1 | Exportin 1 | Protein Coding | 46 | GC02M 061445 | 0.213 709 | https://www.genecards.org/cgi-bin/carddisp.pl?gene=XPO1 |

| | | | | | | |
|---|---|---|---|---|---|---|
| GALNT2 | Polypeptide N-Acetylgalactosaminyltransferase 2 | Protein Coding | 45 | GC01P 230057 | 0.213 709 | https://www.genecards.org/cgi-bin/carddisp.pl?gene=GALNT2 |
| IGFBP7 | Insulin Like Growth Factor Binding Protein 7 | Protein Coding | 44 | GC04M 057030 | 0.213 709 | https://www.genecards.org/cgi-bin/carddisp.pl?gene=IGFBP7 |
| CHIA | Chitinase Acidic | Protein Coding | 42 | GC01P 111291 | 0.213 709 | https://www.genecards.org/cgi-bin/carddisp.pl?gene=CHIA |
| SOAT2 | Sterol O-Acyltransferase 2 | Protein Coding | 42 | GC12P 053103 | 0.213 709 | https://www.genecards.org/cgi-bin/carddisp.pl?gene=SOAT2 |
| DUT | Deoxyuridine Triphosphatase | Protein Coding | 41 | GC15P 048331 | 0.213 709 | https://www.genecards.org/cgi-bin/carddisp.pl?gene=DUT |
| BCL2A1 | BCL2 Related Protein A1 | Protein Coding | 39 | GC15M 090460 | 0.213 709 | https://www.genecards.org/cgi-bin/carddisp.pl?gene=BCL2A1 |
| HSPB2 | Heat Shock Protein Family B (Small) Member 2 | Protein Coding | 38 | GC11P 111917 | 0.213 709 | https://www.genecards.org/cgi-bin/carddisp.pl?gene=HSPB2 |
| DNAH10 | Dynein Axonemal Heavy Chain 10 | Protein Coding | 37 | GC12P 123762 | 0.213 709 | https://www.genecards.org/cgi-bin/carddisp.pl?gene=DNAH10 |
| SLC5A12 | Solute Carrier Family 5 Member 12 | Protein Coding | 37 | GC11M 026648 | 0.213 709 | https://www.genecards.org/cgi-bin/carddisp.pl?gene=SLC5A12 |
| HOXD3 | Homeobox D3 | Protein Coding | 36 | GC02P 176136 | 0.213 709 | https://www.genecards.org/cgi-bin/carddisp.pl?gene=HOXD3 |
| GPHA2 | Glycoprotein Hormone Subunit Alpha 2 | Protein Coding | 34 | GC11M 089739 | 0.213 709 | https://www.genecards.org/cgi-bin/carddisp.pl?gene=GPHA2 |
| RNASE7 | Ribonuclease A Family Member 7 | Protein Coding | 34 | GC14P 021042 | 0.213 709 | https://www.genecards.org/cgi-bin/carddisp.pl?gene=RNASE7 |
| CDK5 | Cyclin Dependent Kinase 5 | Protein Coding | 50 | GC07M 151053 | 0.177 573 | https://www.genecards.org/cgi-bin/carddisp.pl?gene=CDK5 |
| ATP2A2 | ATPase Sarcoplasmic/Endoplasmi | Protein Coding | 49 | GC12P 110280 | 0.177 573 | https://www.genecards.org/cgi- |

| | | | | | | |
|---|---|---|---|---|---|---|
| | c Reticulum Ca2+ Transporting 2 | | | | | bin/carddisp.pl?gene=ATP2A2 |
| CAD | Carbamoyl-Phosphate Synthetase 2, Aspartate Transcarbamylase, And Dihydroorotase | Protein Coding | 48 | GC02P 027217 | 0.177 573 | https://www.genecards.org/cgi-bin/carddisp.pl?gene=CAD |
| DLD | Dihydrolipoamide Dehydrogenase | Protein Coding | 48 | GC07P 107890 | 0.177 573 | https://www.genecards.org/cgi-bin/carddisp.pl?gene=DLD |
| DNM2 | Dynamin 2 | Protein Coding | 48 | GC19P 010718 | 0.177 573 | https://www.genecards.org/cgi-bin/carddisp.pl?gene=DNM2 |
| F12 | Coagulation Factor XII | Protein Coding | 48 | GC05M 177402 | 0.177 573 | https://www.genecards.org/cgi-bin/carddisp.pl?gene=F12 |
| LAMB1 | Laminin Subunit Beta 1 | Protein Coding | 48 | GC07M 107923 | 0.177 573 | https://www.genecards.org/cgi-bin/carddisp.pl?gene=LAMB1 |
| AHCY | Adenosylhomocysteinase | Protein Coding | 47 | GC20M 034827 | 0.177 573 | https://www.genecards.org/cgi-bin/carddisp.pl?gene=AHCY |
| USP7 | Ubiquitin Specific Peptidase 7 | Protein Coding | 47 | GC16M 008892 | 0.177 573 | https://www.genecards.org/cgi-bin/carddisp.pl?gene=USP7 |
| YWHAE | Tyrosine 3-Monooxygenase/Tryptophan 5-Monooxygenase Activation Protein Epsilon | Protein Coding | 47 | GC17M 003080 | 0.177 573 | https://www.genecards.org/cgi-bin/carddisp.pl?gene=YWHAE |
| ABCB11 | ATP Binding Cassette Subfamily B Member 11 | Protein Coding | 46 | GC02M 168922 | 0.177 573 | https://www.genecards.org/cgi-bin/carddisp.pl?gene=ABCB11 |
| FGR | FGR Proto-Oncogene, Src Family Tyrosine Kinase | Protein Coding | 46 | GC01M 027919 | 0.177 573 | https://www.genecards.org/cgi-bin/carddisp.pl?gene=FGR |
| HCK | HCK Proto-Oncogene, Src Family Tyrosine Kinase | Protein Coding | 46 | GC20P 032052 | 0.177 573 | https://www.genecards.org/cgi-bin/carddisp.pl?gene=HCK |
| MAP4K4 | Mitogen-Activated Protein Kinase Kinase Kinase Kinase 4 | Protein Coding | 46 | GC02P 101696 | 0.177 573 | https://www.genecards.org/cgi-bin/carddisp.pl?gene=MAP4K4 |
| OAT | Ornithine Aminotransferase | Protein Coding | 46 | GC10M 124397 | 0.177 573 | https://www.genecards.org/cgi-bin/carddisp.pl?gene=OAT |

| | | | | | | |
|---|---|---|---|---|---|---|
| PROS1 | Protein S | Protein Coding | 46 | GC03M 093873 | 0.177 573 | https://www.genecards .org/cgi- bin/carddisp.pl?gene= PROS1 |
| RANBP2 | RAN Binding Protein 2 | Protein Coding | 46 | GC02P 108719 | 0.177 573 | https://www.genecards .org/cgi- bin/carddisp.pl?gene= RANBP2 |
| RHO | Rhodopsin | Protein Coding | 46 | GC03P 134215 | 0.177 573 | https://www.genecards .org/cgi- bin/carddisp.pl?gene= RHO |
| VLDLR | Very Low Density Lipoprotein Receptor | Protein Coding | 46 | GC09P 002611 | 0.177 573 | https://www.genecards .org/cgi- bin/carddisp.pl?gene= VLDLR |
| ACLY | ATP Citrate Lyase | Protein Coding | 45 | GC17M 041866 | 0.177 573 | https://www.genecards .org/cgi- bin/carddisp.pl?gene= ACLY |
| CACNA1 E | Calcium Voltage-Gated Channel Subunit Alpha1 E | Protein Coding | 45 | GC01P 181317 | 0.177 573 | https://www.genecards .org/cgi- bin/carddisp.pl?gene= CACNA1E |
| EIF2AK2 | Eukaryotic Translation Initiation Factor 2 Alpha Kinase 2 | Protein Coding | 45 | GC02M 037099 | 0.177 573 | https://www.genecards .org/cgi- bin/carddisp.pl?gene= EIF2AK2 |
| GANAB | Glucosidase II Alpha Subunit | Protein Coding | 45 | GC11M 089627 | 0.177 573 | https://www.genecards .org/cgi- bin/carddisp.pl?gene= GANAB |
| GRM3 | Glutamate Metabotropic Receptor 3 | Protein Coding | 45 | GC07P 086643 | 0.177 573 | https://www.genecards .org/cgi- bin/carddisp.pl?gene= GRM3 |
| HSPA9 | Heat Shock Protein Family A (Hsp70) Member 9 | Protein Coding | 45 | GC05M 138554 | 0.177 573 | https://www.genecards .org/cgi- bin/carddisp.pl?gene= HSPA9 |
| MYH10 | Myosin Heavy Chain 10 | Protein Coding | 45 | GC17M 008474 | 0.177 573 | https://www.genecards .org/cgi- bin/carddisp.pl?gene= MYH10 |
| PSAT1 | Phosphoserine Aminotransferase 1 | Protein Coding | 45 | GC09P 078297 | 0.177 573 | https://www.genecards .org/cgi- bin/carddisp.pl?gene= PSAT1 |
| SLC25A1 2 | Solute Carrier Family 25 Member 12 | Protein Coding | 45 | GC02M 171783 | 0.177 573 | https://www.genecards .org/cgi- bin/carddisp.pl?gene= SLC25A12 |
| SLC25A1 3 | Solute Carrier Family 25 Member 13 | Protein Coding | 45 | GC07M 096120 | 0.177 573 | https://www.genecards .org/cgi- |

| | | | | | | |
|---|---|---|---|---|---|---|
| | | | | | | bin/carddisp.pl?gene=SLC25A13 |
| SLC26A3 | Solute Carrier Family 26 Member 3 | Protein Coding | 45 | GC07M107765 | 0.177573 | https://www.genecards.org/cgi-bin/carddisp.pl?gene=SLC26A3 |
| TUFM | Tu Translation Elongation Factor, Mitochondrial | Protein Coding | 45 | GC16M036978 | 0.177573 | https://www.genecards.org/cgi-bin/carddisp.pl?gene=TUFM |
| YWHAB | Tyrosine 3-Monooxygenase/Tryptophan 5-Monooxygenase Activation Protein Beta | Protein Coding | 45 | GC20P044885 | 0.177573 | https://www.genecards.org/cgi-bin/carddisp.pl?gene=YWHAB |
| YWHAZ | Tyrosine 3-Monooxygenase/Tryptophan 5-Monooxygenase Activation Protein Zeta | Protein Coding | 45 | GC08M100917 | 0.177573 | https://www.genecards.org/cgi-bin/carddisp.pl?gene=YWHAZ |
| ALPI | Alkaline Phosphatase, Intestinal | Protein Coding | 44 | GC02P232456 | 0.177573 | https://www.genecards.org/cgi-bin/carddisp.pl?gene=ALPI |
| ARAF | A-Raf Proto-Oncogene, Serine/Threonine Kinase | Protein Coding | 44 | GC0XP047572 | 0.177573 | https://www.genecards.org/cgi-bin/carddisp.pl?gene=ARAF |
| CDK9 | Cyclin Dependent Kinase 9 | Protein Coding | 44 | GC09P128023 | 0.177573 | https://www.genecards.org/cgi-bin/carddisp.pl?gene=CDK9 |
| CNTN1 | Contactin 1 | Protein Coding | 44 | GC12P040692 | 0.177573 | https://www.genecards.org/cgi-bin/carddisp.pl?gene=CNTN1 |
| CTPS1 | CTP Synthase 1 | Protein Coding | 44 | GC01P040979 | 0.177573 | https://www.genecards.org/cgi-bin/carddisp.pl?gene=CTPS1 |
| DDOST | Dolichyl-Diphosphooligosaccharide--Protein Glycosyltransferase Non-Catalytic Subunit | Protein Coding | 44 | GC01M020651 | 0.177573 | https://www.genecards.org/cgi-bin/carddisp.pl?gene=DDOST |
| DDX3X | DEAD-Box Helicase 3 X-Linked | Protein Coding | 44 | GC0XP041333 | 0.177573 | https://www.genecards.org/cgi-bin/carddisp.pl?gene=DDX3X |
| EIF2B4 | Eukaryotic Translation Initiation Factor 2B Subunit Delta | Protein Coding | 44 | GC02M027364 | 0.177573 | https://www.genecards.org/cgi-bin/carddisp.pl?gene=EIF2B4 |
| EIF4A1 | Eukaryotic Translation Initiation Factor 4A1 | Protein Coding | 44 | GC17P007572 | 0.177573 | https://www.genecards.org/cgi- |

| | | | | | | |
|---|---|---|---|---|---|---|
| | | | | | | bin/carddisp.pl?gene=EIF4A1 |
| GFRA1 | GDNF Family Receptor Alpha 1 | Protein Coding | 44 | GC10M116056 | 0.177573 | https://www.genecards.org/cgi-bin/carddisp.pl?gene=GFRA1 |
| GNAI1 | G Protein Subunit Alpha I1 | Protein Coding | 44 | GC07P079769 | 0.177573 | https://www.genecards.org/cgi-bin/carddisp.pl?gene=GNAI1 |
| HELLS | Helicase, Lymphoid Specific | Protein Coding | 44 | GC10P094501 | 0.177573 | https://www.genecards.org/cgi-bin/carddisp.pl?gene=HELLS |
| KLK2 | Kallikrein Related Peptidase 2 | Protein Coding | 44 | GC19P050861 | 0.177573 | https://www.genecards.org/cgi-bin/carddisp.pl?gene=KLK2 |
| MCM3 | Minichromosome Maintenance Complex Component 3 | Protein Coding | 44 | GC06M052264 | 0.177573 | https://www.genecards.org/cgi-bin/carddisp.pl?gene=MCM3 |
| MCM5 | Minichromosome Maintenance Complex Component 5 | Protein Coding | 44 | GC22P035400 | 0.177573 | https://www.genecards.org/cgi-bin/carddisp.pl?gene=MCM5 |
| MIB1 | MIB E3 Ubiquitin Protein Ligase 1 | Protein Coding | 44 | GC18P021704 | 0.177573 | https://www.genecards.org/cgi-bin/carddisp.pl?gene=MIB1 |
| MYL3 | Myosin Light Chain 3 | Protein Coding | 44 | GC03M046836 | 0.177573 | https://www.genecards.org/cgi-bin/carddisp.pl?gene=MYL3 |
| PDHB | Pyruvate Dehydrogenase E1 Subunit Beta | Protein Coding | 44 | GC03M058428 | 0.177573 | https://www.genecards.org/cgi-bin/carddisp.pl?gene=PDHB |
| PGRMC1 | Progesterone Receptor Membrane Component 1 | Protein Coding | 44 | GC0XP119236 | 0.177573 | https://www.genecards.org/cgi-bin/carddisp.pl?gene=PGRMC1 |
| PRKD2 | Protein Kinase D2 | Protein Coding | 44 | GC19M046674 | 0.177573 | https://www.genecards.org/cgi-bin/carddisp.pl?gene=PRKD2 |
| PTGFR | Prostaglandin F Receptor | Protein Coding | 44 | GC01P078303 | 0.177573 | https://www.genecards.org/cgi-bin/carddisp.pl?gene=PTGFR |
| PXN | Paxillin | Protein Coding | 44 | GC12M120210 | 0.177573 | https://www.genecards.org/cgi-bin/carddisp.pl?gene=PXN |

| | | | | | | |
|---|---|---|---|---|---|---|
| SKI | SKI Proto-Oncogene | Protein Coding | 44 | GC01P 002228 | 0.177 573 | https://www.genecards.org/cgi-bin/carddisp.pl?gene=SKI |
| SLC27A4 | Solute Carrier Family 27 Member 4 | Protein Coding | 44 | GC09P 128340 | 0.177 573 | https://www.genecards.org/cgi-bin/carddisp.pl?gene=SLC27A4 |
| SMC1A | Structural Maintenance Of Chromosomes 1A | Protein Coding | 44 | GC0X M0533 74 | 0.177 573 | https://www.genecards.org/cgi-bin/carddisp.pl?gene=SMC1A |
| SMC3 | Structural Maintenance Of Chromosomes 3 | Protein Coding | 44 | GC10P 110567 | 0.177 573 | https://www.genecards.org/cgi-bin/carddisp.pl?gene=SMC3 |
| TRPM7 | Transient Receptor Potential Cation Channel Subfamily M Member 7 | Protein Coding | 44 | GC15M 050552 | 0.177 573 | https://www.genecards.org/cgi-bin/carddisp.pl?gene=TRPM7 |
| TUBB4A | Tubulin Beta 4A Class IVa | Protein Coding | 44 | GC19M 006496 | 0.177 573 | https://www.genecards.org/cgi-bin/carddisp.pl?gene=TUBB4A |
| ABCD3 | ATP Binding Cassette Subfamily D Member 3 | Protein Coding | 43 | GC01P 094385 | 0.177 573 | https://www.genecards.org/cgi-bin/carddisp.pl?gene=ABCD3 |
| ALDH18 A1 | Aldehyde Dehydrogenase 18 Family Member A1 | Protein Coding | 43 | GC10M 095605 | 0.177 573 | https://www.genecards.org/cgi-bin/carddisp.pl?gene=ALDH18A1 |
| CD151 | CD151 Molecule (Raph Blood Group) | Protein Coding | 43 | GC11P 001795 | 0.177 573 | https://www.genecards.org/cgi-bin/carddisp.pl?gene=CD151 |
| CHD2 | Chromodomain Helicase DNA Binding Protein 2 | Protein Coding | 43 | GC15P 092900 | 0.177 573 | https://www.genecards.org/cgi-bin/carddisp.pl?gene=CHD2 |
| CKB | Creatine Kinase B | Protein Coding | 43 | GC14M 103519 | 0.177 573 | https://www.genecards.org/cgi-bin/carddisp.pl?gene=CKB |
| DOCK2 | Dedicator Of Cytokinesis 2 | Protein Coding | 43 | GC05P 169637 | 0.177 573 | https://www.genecards.org/cgi-bin/carddisp.pl?gene=DOCK2 |
| DYNC1H 1 | Dynein Cytoplasmic 1 Heavy Chain 1 | Protein Coding | 43 | GC14P 109704 | 0.177 573 | https://www.genecards.org/cgi-bin/carddisp.pl?gene=DYNC1H1 |
| EMX2 | Empty Spiracles Homeobox 2 | Protein Coding | 43 | GC10P 117542 | 0.177 573 | https://www.genecards.org/cgi- |

| | | | | | | |
|---|---|---|---|---|---|---|
| | | | | | | bin/carddisp.pl?gene=EMX2 |
| HIF1AN | Hypoxia Inducible Factor 1 Subunit Alpha Inhibitor | Protein Coding | 43 | GC10P 100529 | 0.177 573 | https://www.genecards.org/cgi-bin/carddisp.pl?gene=HIF1AN |
| KLK4 | Kallikrein Related Peptidase 4 | Protein Coding | 43 | GC19M 066193 | 0.177 573 | https://www.genecards.org/cgi-bin/carddisp.pl?gene=KLK4 |
| MCM7 | Minichromosome Maintenance Complex Component 7 | Protein Coding | 43 | GC07M 100092 | 0.177 573 | https://www.genecards.org/cgi-bin/carddisp.pl?gene=MCM7 |
| NDRG1 | N-Myc Downstream Regulated 1 | Protein Coding | 43 | GC08M 133237 | 0.177 573 | https://www.genecards.org/cgi-bin/carddisp.pl?gene=NDRG1 |
| RPL10 | Ribosomal Protein L10 | Protein Coding | 43 | GC0XP 154389 | 0.177 573 | https://www.genecards.org/cgi-bin/carddisp.pl?gene=RPL10 |
| RUVBL1 | RuvB Like AAA ATPase 1 | Protein Coding | 43 | GC03M 128064 | 0.177 573 | https://www.genecards.org/cgi-bin/carddisp.pl?gene=RUVBL1 |
| SLC25A3 | Solute Carrier Family 25 Member 3 | Protein Coding | 43 | GC12P 098593 | 0.177 573 | https://www.genecards.org/cgi-bin/carddisp.pl?gene=SLC25A3 |
| SMURF1 | SMAD Specific E3 Ubiquitin Protein Ligase 1 | Protein Coding | 43 | GC07M 099027 | 0.177 573 | https://www.genecards.org/cgi-bin/carddisp.pl?gene=SMURF1 |
| SYT2 | Synaptotagmin 2 | Protein Coding | 43 | GC01M 202559 | 0.177 573 | https://www.genecards.org/cgi-bin/carddisp.pl?gene=SYT2 |
| ZBTB16 | Zinc Finger And BTB Domain Containing 16 | Protein Coding | 43 | GC11P 114059 | 0.177 573 | https://www.genecards.org/cgi-bin/carddisp.pl?gene=ZBTB16 |
| CRBN | Cereblon | Protein Coding | 42 | GC03M 003144 | 0.177 573 | https://www.genecards.org/cgi-bin/carddisp.pl?gene=CRBN |
| DLG1 | Discs Large MAGUK Scaffold Protein 1 | Protein Coding | 42 | GC03M 197042 | 0.177 573 | https://www.genecards.org/cgi-bin/carddisp.pl?gene=DLG1 |
| DPM1 | Dolichyl-Phosphate Mannosyltransferase Subunit 1, Catalytic | Protein Coding | 42 | GC20M 050934 | 0.177 573 | https://www.genecards.org/cgi-bin/carddisp.pl?gene=DPM1 |

| | | | | | | |
|---|---|---|---|---|---|---|
| EEA1 | Early Endosome Antigen 1 | Protein Coding | 42 | GC12M092770 | 0.177573 | https://www.genecards.org/cgi-bin/carddisp.pl?gene=EEA1 |
| EIF4A3 | Eukaryotic Translation Initiation Factor 4A3 | Protein Coding | 42 | GC17M080135 | 0.177573 | https://www.genecards.org/cgi-bin/carddisp.pl?gene=EIF4A3 |
| FUT8 | Fucosyltransferase 8 | Protein Coding | 42 | GC14P065411 | 0.177573 | https://www.genecards.org/cgi-bin/carddisp.pl?gene=FUT8 |
| HBA2 | Hemoglobin Subunit Alpha 2 | Protein Coding | 42 | GC16P011600 | 0.177573 | https://www.genecards.org/cgi-bin/carddisp.pl?gene=HBA2 |
| HOXA1 | Homeobox A1 | Protein Coding | 42 | GC07M027092 | 0.177573 | https://www.genecards.org/cgi-bin/carddisp.pl?gene=HOXA1 |
| LRPPRC | Leucine Rich Pentatricopeptide Repeat Containing | Protein Coding | 42 | GC02M043886 | 0.177573 | https://www.genecards.org/cgi-bin/carddisp.pl?gene=LRPPRC |
| MCM6 | Minichromosome Maintenance Complex Component 6 | Protein Coding | 42 | GC02M135839 | 0.177573 | https://www.genecards.org/cgi-bin/carddisp.pl?gene=MCM6 |
| NCOR2 | Nuclear Receptor Corepressor 2 | Protein Coding | 42 | GC12M124324 | 0.177573 | https://www.genecards.org/cgi-bin/carddisp.pl?gene=NCOR2 |
| NOP56 | NOP56 Ribonucleoprotein | Protein Coding | 42 | GC20P004223 | 0.177573 | https://www.genecards.org/cgi-bin/carddisp.pl?gene=NOP56 |
| PAPPA | Pappalysin 1 | Protein Coding | 42 | GC09P118958 | 0.177573 | https://www.genecards.org/cgi-bin/carddisp.pl?gene=PAPPA |
| PDE7A | Phosphodiesterase 7A | Protein Coding | 42 | GC08M065736 | 0.177573 | https://www.genecards.org/cgi-bin/carddisp.pl?gene=PDE7A |
| PPP2R2A | Protein Phosphatase 2 Regulatory Subunit Balpha | Protein Coding | 42 | GC08P026292 | 0.177573 | https://www.genecards.org/cgi-bin/carddisp.pl?gene=PPP2R2A |
| PSMC3 | Proteasome 26S Subunit, ATPase 3 | Protein Coding | 42 | GC11M089365 | 0.177573 | https://www.genecards.org/cgi-bin/carddisp.pl?gene=PSMC3 |
| RARS2 | Arginyl-TRNA Synthetase 2, Mitochondrial | Protein Coding | 42 | GC06M087514 | 0.177573 | https://www.genecards.org/cgi- |

| | | | | | | |
|---|---|---|---|---|---|---|
| | | | | | | bin/carddisp.pl?gene=RARS2 |
| ROBO2 | Roundabout Guidance Receptor 2 | Protein Coding | 42 | GC03P075955 | 0.177573 | https://www.genecards.org/cgi-bin/carddisp.pl?gene=ROBO2 |
| RPN1 | Ribophorin I | Protein Coding | 42 | GC03M128619 | 0.177573 | https://www.genecards.org/cgi-bin/carddisp.pl?gene=RPN1 |
| RPS3 | Ribosomal Protein S3 | Protein Coding | 42 | GC11P077882 | 0.177573 | https://www.genecards.org/cgi-bin/carddisp.pl?gene=RPS3 |
| SLC1A5 | Solute Carrier Family 1 Member 5 | Protein Coding | 42 | GC19M065991 | 0.177573 | https://www.genecards.org/cgi-bin/carddisp.pl?gene=SLC1A5 |
| SLC25A11 | Solute Carrier Family 25 Member 11 | Protein Coding | 42 | GC17M004937 | 0.177573 | https://www.genecards.org/cgi-bin/carddisp.pl?gene=SLC25A11 |
| SLC25A5 | Solute Carrier Family 25 Member 5 | Protein Coding | 42 | GC0XP119468 | 0.177573 | https://www.genecards.org/cgi-bin/carddisp.pl?gene=SLC25A5 |
| SLIT1 | Slit Guidance Ligand 1 | Protein Coding | 42 | GC10M096998 | 0.177573 | https://www.genecards.org/cgi-bin/carddisp.pl?gene=SLIT1 |
| SNRNP200 | Small Nuclear Ribonucleoprotein U5 Subunit 200 | Protein Coding | 42 | GC02M098261 | 0.177573 | https://www.genecards.org/cgi-bin/carddisp.pl?gene=SNRNP200 |
| ST6GAL1 | ST6 Beta-Galactoside Alpha-2,6-Sialyltransferase 1 | Protein Coding | 42 | GC03P186930 | 0.177573 | https://www.genecards.org/cgi-bin/carddisp.pl?gene=ST6GAL1 |
| TSPAN7 | Tetraspanin 7 | Protein Coding | 42 | GC0XP038561 | 0.177573 | https://www.genecards.org/cgi-bin/carddisp.pl?gene=TSPAN7 |
| TUBB4B | Tubulin Beta 4B Class IVb | Protein Coding | 42 | GC09P137241 | 0.177573 | https://www.genecards.org/cgi-bin/carddisp.pl?gene=TUBB4B |
| VDAC2 | Voltage Dependent Anion Channel 2 | Protein Coding | 42 | GC10P075210 | 0.177573 | https://www.genecards.org/cgi-bin/carddisp.pl?gene=VDAC2 |
| ADCYAP1 | Adenylate Cyclase Activating Polypeptide 1 | Protein Coding | 41 | GC18P000895 | 0.177573 | https://www.genecards.org/cgi-bin/carddisp.pl?gene=ADCYAP1 |

| | | | | | | |
|---|---|---|---|---|---|---|
| ATXN10 | Ataxin 10 | Protein Coding | 41 | GC22P 045673 | 0.177 573 | https://www.genecards .org/cgi-bin/carddisp.pl?gene= ATXN10 |
| CACNB1 | Calcium Voltage-Gated Channel Auxiliary Subunit Beta 1 | Protein Coding | 41 | GC17M 039173 | 0.177 573 | https://www.genecards .org/cgi-bin/carddisp.pl?gene= CACNB1 |
| CADPS | Calcium Dependent Secretion Activator | Protein Coding | 41 | GC03M 062398 | 0.177 573 | https://www.genecards .org/cgi-bin/carddisp.pl?gene= CADPS |
| CCT7 | Chaperonin Containing TCP1 Subunit 7 | Protein Coding | 41 | GC02P 073233 | 0.177 573 | https://www.genecards .org/cgi-bin/carddisp.pl?gene= CCT7 |
| CD82 | CD82 Molecule | Protein Coding | 41 | GC11P 044586 | 0.177 573 | https://www.genecards .org/cgi-bin/carddisp.pl?gene= CD82 |
| CHKA | Choline Kinase Alpha | Protein Coding | 41 | GC11M 068052 | 0.177 573 | https://www.genecards .org/cgi-bin/carddisp.pl?gene= CHKA |
| CYFIP2 | Cytoplasmic FMR1 Interacting Protein 2 | Protein Coding | 41 | GC05P 157267 | 0.177 573 | https://www.genecards .org/cgi-bin/carddisp.pl?gene= CYFIP2 |
| DUSP10 | Dual Specificity Phosphatase 10 | Protein Coding | 41 | GC01M 221701 | 0.177 573 | https://www.genecards .org/cgi-bin/carddisp.pl?gene= DUSP10 |
| EFTUD2 | Elongation Factor Tu GTP Binding Domain Containing 2 | Protein Coding | 41 | GC17M 044952 | 0.177 573 | https://www.genecards .org/cgi-bin/carddisp.pl?gene= EFTUD2 |
| EIF3A | Eukaryotic Translation Initiation Factor 3 Subunit A | Protein Coding | 41 | GC10M 119034 | 0.177 573 | https://www.genecards .org/cgi-bin/carddisp.pl?gene= EIF3A |
| FUT6 | Fucosyltransferase 6 | Protein Coding | 41 | GC19M 005830 | 0.177 573 | https://www.genecards .org/cgi-bin/carddisp.pl?gene= FUT6 |
| GPM6A | Glycoprotein M6A | Protein Coding | 41 | GC04M 175632 | 0.177 573 | https://www.genecards .org/cgi-bin/carddisp.pl?gene= GPM6A |
| GPSM2 | G Protein Signaling Modulator 2 | Protein Coding | 41 | GC01P 108875 | 0.177 573 | https://www.genecards .org/cgi-bin/carddisp.pl?gene= GPSM2 |
| KLK15 | Kallikrein Related Peptidase 15 | Protein Coding | 41 | GC19M 050825 | 0.177 573 | https://www.genecards .org/cgi- |

| | | | | | | |
|---|---|---|---|---|---|---|
| | | | | | | bin/carddisp.pl?gene=KLK15 |
| LRP1B | LDL Receptor Related Protein 1B | Protein Coding | 41 | GC02M140231 | 0.177573 | https://www.genecards.org/cgi-bin/carddisp.pl?gene=LRP1B |
| MLYCD | Malonyl-CoA Decarboxylase | Protein Coding | 41 | GC16P083899 | 0.177573 | https://www.genecards.org/cgi-bin/carddisp.pl?gene=MLYCD |
| MSRB3 | Methionine Sulfoxide Reductase B3 | Protein Coding | 41 | GC12P065279 | 0.177573 | https://www.genecards.org/cgi-bin/carddisp.pl?gene=MSRB3 |
| NRG3 | Neuregulin 3 | Protein Coding | 41 | GC10P092362 | 0.177573 | https://www.genecards.org/cgi-bin/carddisp.pl?gene=NRG3 |
| NRXN3 | Neurexin 3 | Protein Coding | 41 | GC14P078170 | 0.177573 | https://www.genecards.org/cgi-bin/carddisp.pl?gene=NRXN3 |
| PAICS | Phosphoribosylaminoimidazole Carboxylase And Phosphoribosylaminoimidazolesuccinocarboxamide Synthase | Protein Coding | 41 | GC04P056410 | 0.177573 | https://www.genecards.org/cgi-bin/carddisp.pl?gene=PAICS |
| PHB2 | Prohibitin 2 | Protein Coding | 41 | GC12M006965 | 0.177573 | https://www.genecards.org/cgi-bin/carddisp.pl?gene=PHB2 |
| PLXND1 | Plexin D1 | Protein Coding | 41 | GC03M129555 | 0.177573 | https://www.genecards.org/cgi-bin/carddisp.pl?gene=PLXND1 |
| PRPF8 | Pre-MRNA Processing Factor 8 | Protein Coding | 41 | GC17M001650 | 0.177573 | https://www.genecards.org/cgi-bin/carddisp.pl?gene=PRPF8 |
| PUF60 | Poly(U) Binding Splicing Factor 60 | Protein Coding | 41 | GC08M143816 | 0.177573 | https://www.genecards.org/cgi-bin/carddisp.pl?gene=PUF60 |
| RBX1 | Ring-Box 1 | Protein Coding | 41 | GC22P040951 | 0.177573 | https://www.genecards.org/cgi-bin/carddisp.pl?gene=RBX1 |
| RPL7A | Ribosomal Protein L7a | Protein Coding | 41 | GC09P133348 | 0.177573 | https://www.genecards.org/cgi-bin/carddisp.pl?gene=RPL7A |
| RPS15A | Ribosomal Protein S15a | Protein Coding | 41 | GC16M018781 | 0.177573 | https://www.genecards.org/cgi- |

| | | | | | | |
|---|---|---|---|---|---|---|
| | | | | | | bin/carddisp.pl?gene=RPS15A |
| RPS27 | Ribosomal Protein S27 | Protein Coding | 41 | GC01P153991 | 0.177573 | https://www.genecards.org/cgi-bin/carddisp.pl?gene=RPS27 |
| SBF1 | SET Binding Factor 1 | Protein Coding | 41 | GC22M057734 | 0.177573 | https://www.genecards.org/cgi-bin/carddisp.pl?gene=SBF1 |
| SEPHS1 | Selenophosphate Synthetase 1 | Protein Coding | 41 | GC10M013317 | 0.177573 | https://www.genecards.org/cgi-bin/carddisp.pl?gene=SEPHS1 |
| SMYD2 | SET And MYND Domain Containing 2 | Protein Coding | 41 | GC01P214281 | 0.177573 | https://www.genecards.org/cgi-bin/carddisp.pl?gene=SMYD2 |
| TRIM28 | Tripartite Motif Containing 28 | Protein Coding | 41 | GC19P058544 | 0.177573 | https://www.genecards.org/cgi-bin/carddisp.pl?gene=TRIM28 |
| TUBA1B | Tubulin Alpha 1b | Protein Coding | 41 | GC12M049127 | 0.177573 | https://www.genecards.org/cgi-bin/carddisp.pl?gene=TUBA1B |
| TUBA1C | Tubulin Alpha 1c | Protein Coding | 41 | GC12P049188 | 0.177573 | https://www.genecards.org/cgi-bin/carddisp.pl?gene=TUBA1C |
| UBE2T | Ubiquitin Conjugating Enzyme E2 T | Protein Coding | 41 | GC01M202332 | 0.177573 | https://www.genecards.org/cgi-bin/carddisp.pl?gene=UBE2T |
| VAV2 | Vav Guanine Nucleotide Exchange Factor 2 | Protein Coding | 41 | GC09M133761 | 0.177573 | https://www.genecards.org/cgi-bin/carddisp.pl?gene=VAV2 |
| ADAM28 | ADAM Metallopeptidase Domain 28 | Protein Coding | 40 | GC08P024294 | 0.177573 | https://www.genecards.org/cgi-bin/carddisp.pl?gene=ADAM28 |
| ARF4 | ADP Ribosylation Factor 4 | Protein Coding | 40 | GC03M057572 | 0.177573 | https://www.genecards.org/cgi-bin/carddisp.pl?gene=ARF4 |
| ARR3 | Arrestin 3 | Protein Coding | 40 | GC0XP070269 | 0.177573 | https://www.genecards.org/cgi-bin/carddisp.pl?gene=ARR3 |
| ATAD3A | ATPase Family AAA Domain Containing 3A | Protein Coding | 40 | GC01P003886 | 0.177573 | https://www.genecards.org/cgi-bin/carddisp.pl?gene=ATAD3A |

| | | | | | | |
|---|---|---|---|---|---|---|
| AUTS2 | Activator Of Transcription And Developmental Regulator AUTS2 | Protein Coding | 40 | GC07P 069598 | 0.177 573 | https://www.genecards .org/cgi-bin/carddisp.pl?gene= AUTS2 |
| CCT4 | Chaperonin Containing TCP1 Subunit 4 | Protein Coding | 40 | GC02M 061868 | 0.177 573 | https://www.genecards .org/cgi-bin/carddisp.pl?gene= CCT4 |
| CD300A | CD300a Molecule | Protein Coding | 40 | GC17P 074466 | 0.177 573 | https://www.genecards .org/cgi-bin/carddisp.pl?gene= CD300A |
| CDC37 | Cell Division Cycle 37, HSP90 Cochaperone | Protein Coding | 40 | GC19M 010391 | 0.177 573 | https://www.genecards .org/cgi-bin/carddisp.pl?gene= CDC37 |
| CFHR3 | Complement Factor H Related 3 | Protein Coding | 40 | GC01P 196774 | 0.177 573 | https://www.genecards .org/cgi-bin/carddisp.pl?gene= CFHR3 |
| CNOT1 | CCR4-NOT Transcription Complex Subunit 1 | Protein Coding | 40 | GC16M 058519 | 0.177 573 | https://www.genecards .org/cgi-bin/carddisp.pl?gene= CNOT1 |
| COIL | Coilin | Protein Coding | 40 | GC17M 056938 | 0.177 573 | https://www.genecards .org/cgi-bin/carddisp.pl?gene= COIL |
| ERP44 | Endoplasmic Reticulum Protein 44 | Protein Coding | 40 | GC09M 099979 | 0.177 573 | https://www.genecards .org/cgi-bin/carddisp.pl?gene= ERP44 |
| FANCI | FA Complementation Group I | Protein Coding | 40 | GC15P 089243 | 0.177 573 | https://www.genecards .org/cgi-bin/carddisp.pl?gene= FANCI |
| FOSB | FosB Proto-Oncogene, AP-1 Transcription Factor Subunit | Protein Coding | 40 | GC19P 045467 | 0.177 573 | https://www.genecards .org/cgi-bin/carddisp.pl?gene= FOSB |
| HSD17B1 2 | Hydroxysteroid 17-Beta Dehydrogenase 12 | Protein Coding | 40 | GC11P 043652 | 0.177 573 | https://www.genecards .org/cgi-bin/carddisp.pl?gene= HSD17B12 |
| KTN1 | Kinectin 1 | Protein Coding | 40 | GC14P 055559 | 0.177 573 | https://www.genecards .org/cgi-bin/carddisp.pl?gene= KTN1 |
| LPO | Lactoperoxidase | Protein Coding | 40 | GC17P 058218 | 0.177 573 | https://www.genecards .org/cgi-bin/carddisp.pl?gene= LPO |
| LRRC32 | Leucine Rich Repeat Containing 32 | Protein Coding | 40 | GC11M 076657 | 0.177 573 | https://www.genecards .org/cgi- |

| | | | | | | |
|---|---|---|---|---|---|---|
| | | | | | | bin/carddisp.pl?gene=LRRC32 |
| MTHFD1L | Methylenetetrahydrofolate Dehydrogenase (NADP+ Dependent) 1 Like | Protein Coding | 40 | GC06P150865 | 0.177573 | https://www.genecards.org/cgi-bin/carddisp.pl?gene=MTHFD1L |
| NCAPD3 | Non-SMC Condensin II Complex Subunit D3 | Protein Coding | 40 | GC11M134150 | 0.177573 | https://www.genecards.org/cgi-bin/carddisp.pl?gene=NCAPD3 |
| NMBR | Neuromedin B Receptor | Protein Coding | 40 | GC06M142059 | 0.177573 | https://www.genecards.org/cgi-bin/carddisp.pl?gene=NMBR |
| NSMAF | Neutral Sphingomyelinase Activation Associated Factor | Protein Coding | 40 | GC08M058569 | 0.177573 | https://www.genecards.org/cgi-bin/carddisp.pl?gene=NSMAF |
| PARD6A | Par-6 Family Cell Polarity Regulator Alpha | Protein Coding | 40 | GC16P067661 | 0.177573 | https://www.genecards.org/cgi-bin/carddisp.pl?gene=PARD6A |
| PCBP2 | Poly(RC) Binding Protein 2 | Protein Coding | 40 | GC12P053452 | 0.177573 | https://www.genecards.org/cgi-bin/carddisp.pl?gene=PCBP2 |
| PDSS2 | Decaprenyl Diphosphate Synthase Subunit 2 | Protein Coding | 40 | GC06M107152 | 0.177573 | https://www.genecards.org/cgi-bin/carddisp.pl?gene=PDSS2 |
| PRMT3 | Protein Arginine Methyltransferase 3 | Protein Coding | 40 | GC11P020409 | 0.177573 | https://www.genecards.org/cgi-bin/carddisp.pl?gene=PRMT3 |
| RPLP2 | Ribosomal Protein Lateral Stalk Subunit P2 | Protein Coding | 40 | GC11P001790 | 0.177573 | https://www.genecards.org/cgi-bin/carddisp.pl?gene=RPLP2 |
| RPS3A | Ribosomal Protein S3A | Protein Coding | 40 | GC04P151099 | 0.177573 | https://www.genecards.org/cgi-bin/carddisp.pl?gene=RPS3A |
| SOX11 | SRY-Box Transcription Factor 11 | Protein Coding | 40 | GC02P005703 | 0.177573 | https://www.genecards.org/cgi-bin/carddisp.pl?gene=SOX11 |
| SRSF1 | Serine And Arginine Rich Splicing Factor 1 | Protein Coding | 40 | GC17M058000 | 0.177573 | https://www.genecards.org/cgi-bin/carddisp.pl?gene=SRSF1 |
| SRSF2 | Serine And Arginine Rich Splicing Factor 2 | Protein Coding | 40 | GC17M076734 | 0.177573 | https://www.genecards.org/cgi-bin/carddisp.pl?gene=SRSF2 |

| | | | | | | |
|---|---|---|---|---|---|---|
| TLN1 | Talin 1 | Protein Coding | 40 | GC09M 035696 | 0.177 573 | https://www.genecards .org/cgi-bin/carddisp.pl?gene= TLN1 |
| TRIP13 | Thyroid Hormone Receptor Interactor 13 | Protein Coding | 40 | GC05P 000892 | 0.177 573 | https://www.genecards .org/cgi-bin/carddisp.pl?gene= TRIP13 |
| TUBB6 | Tubulin Beta 6 Class V | Protein Coding | 40 | GC18P 012307 | 0.177 573 | https://www.genecards .org/cgi-bin/carddisp.pl?gene= TUBB6 |
| USP22 | Ubiquitin Specific Peptidase 22 | Protein Coding | 40 | GC17M 020999 | 0.177 573 | https://www.genecards .org/cgi-bin/carddisp.pl?gene= USP22 |
| XPOT | Exportin For TRNA | Protein Coding | 40 | GC12P 064404 | 0.177 573 | https://www.genecards .org/cgi-bin/carddisp.pl?gene= XPOT |
| ALDH1L 2 | Aldehyde Dehydrogenase 1 Family Member L2 | Protein Coding | 39 | GC12M 105019 | 0.177 573 | https://www.genecards .org/cgi-bin/carddisp.pl?gene= ALDH1L2 |
| CCT6A | Chaperonin Containing TCP1 Subunit 6A | Protein Coding | 39 | GC07P 056051 | 0.177 573 | https://www.genecards .org/cgi-bin/carddisp.pl?gene= CCT6A |
| CCT8 | Chaperonin Containing TCP1 Subunit 8 | Protein Coding | 39 | GC21M 029055 | 0.177 573 | https://www.genecards .org/cgi-bin/carddisp.pl?gene= CCT8 |
| CSE1L | Chromosome Segregation 1 Like | Protein Coding | 39 | GC20P 049046 | 0.177 573 | https://www.genecards .org/cgi-bin/carddisp.pl?gene= CSE1L |
| CST6 | Cystatin E/M | Protein Coding | 39 | GC11P 070022 | 0.177 573 | https://www.genecards .org/cgi-bin/carddisp.pl?gene= CST6 |
| DNER | Delta/Notch Like EGF Repeat Containing | Protein Coding | 39 | GC02M 229357 | 0.177 573 | https://www.genecards .org/cgi-bin/carddisp.pl?gene= DNER |
| IPO5 | Importin 5 | Protein Coding | 39 | GC13P 097953 | 0.177 573 | https://www.genecards .org/cgi-bin/carddisp.pl?gene=I PO5 |
| IRS4 | Insulin Receptor Substrate 4 | Protein Coding | 39 | GC0X M1087 20 | 0.177 573 | https://www.genecards .org/cgi-bin/carddisp.pl?gene=I RS4 |
| MYBBP1 A | MYB Binding Protein 1a | Protein Coding | 39 | GC17M 004538 | 0.177 573 | https://www.genecards .org/cgi- |

| | | | | | | |
|---|---|---|---|---|---|---|
| | | | | | | bin/carddisp.pl?gene=MYBBP1A |
| NXF1 | Nuclear RNA Export Factor 1 | Protein Coding | 39 | GC11M089638 | 0.177573 | https://www.genecards.org/cgi-bin/carddisp.pl?gene=NXF1 |
| RAB11FIP2 | RAB11 Family Interacting Protein 2 | Protein Coding | 39 | GC10M118004 | 0.177573 | https://www.genecards.org/cgi-bin/carddisp.pl?gene=RAB11FIP2 |
| RAB5C | RAB5C, Member RAS Oncogene Family | Protein Coding | 39 | GC17M042124 | 0.177573 | https://www.genecards.org/cgi-bin/carddisp.pl?gene=RAB5C |
| RCN2 | Reticulocalbin 2 | Protein Coding | 39 | GC15P076931 | 0.177573 | https://www.genecards.org/cgi-bin/carddisp.pl?gene=RCN2 |
| SMARCD3 | SWI/SNF Related, Matrix Associated, Actin Dependent Regulator Of Chromatin, Subfamily D, Member 3 | Protein Coding | 39 | GC07M151238 | 0.177573 | https://www.genecards.org/cgi-bin/carddisp.pl?gene=SMARCD3 |
| UBE3C | Ubiquitin Protein Ligase E3C | Protein Coding | 39 | GC07P157138 | 0.177573 | https://www.genecards.org/cgi-bin/carddisp.pl?gene=UBE3C |
| UPF1 | UPF1 RNA Helicase And ATPase | Protein Coding | 39 | GC19P018831 | 0.177573 | https://www.genecards.org/cgi-bin/carddisp.pl?gene=UPF1 |
| ABCB9 | ATP Binding Cassette Subfamily B Member 9 | Protein Coding | 38 | GC12M122920 | 0.177573 | https://www.genecards.org/cgi-bin/carddisp.pl?gene=ABCB9 |
| ABCF2 | ATP Binding Cassette Subfamily F Member 2 | Protein Coding | 38 | GC07M151211 | 0.177573 | https://www.genecards.org/cgi-bin/carddisp.pl?gene=ABCF2 |
| AGR2 | Anterior Gradient 2, Protein Disulphide Isomerase Family Member | Protein Coding | 38 | GC07M017058 | 0.177573 | https://www.genecards.org/cgi-bin/carddisp.pl?gene=AGR2 |
| ARAP3 | ArfGAP With RhoGAP Domain, Ankyrin Repeat And PH Domain 3 | Protein Coding | 38 | GC05M141653 | 0.177573 | https://www.genecards.org/cgi-bin/carddisp.pl?gene=ARAP3 |
| ARHGAP24 | Rho GTPase Activating Protein 24 | Protein Coding | 38 | GC04P085475 | 0.177573 | https://www.genecards.org/cgi-bin/carddisp.pl?gene=ARHGAP24 |
| CCDC22 | Coiled-Coil Domain Containing 22 | Protein Coding | 38 | GC0XP050747 | 0.177573 | https://www.genecards.org/cgi- |

| | | | | | | |
|---|---|---|---|---|---|---|
| | | | | | | bin/carddisp.pl?gene=CCDC22 |
| CDK18 | Cyclin Dependent Kinase 18 | Protein Coding | 38 | GC01P205504 | 0.177573 | https://www.genecards.org/cgi-bin/carddisp.pl?gene=CDK18 |
| COL19A1 | Collagen Type XIX Alpha 1 Chain | Protein Coding | 38 | GC06P069866 | 0.177573 | https://www.genecards.org/cgi-bin/carddisp.pl?gene=COL19A1 |
| DDX21 | DExD-Box Helicase 21 | Protein Coding | 38 | GC10P068956 | 0.177573 | https://www.genecards.org/cgi-bin/carddisp.pl?gene=DDX21 |
| DNAL4 | Dynein Axonemal Light Chain 4 | Protein Coding | 38 | GC22M038778 | 0.177573 | https://www.genecards.org/cgi-bin/carddisp.pl?gene=DNAL4 |
| DOK5 | Docking Protein 5 | Protein Coding | 38 | GC20P054476 | 0.177573 | https://www.genecards.org/cgi-bin/carddisp.pl?gene=DOK5 |
| DUSP19 | Dual Specificity Phosphatase 19 | Protein Coding | 38 | GC02P183078 | 0.177573 | https://www.genecards.org/cgi-bin/carddisp.pl?gene=DUSP19 |
| EEF1G | Eukaryotic Translation Elongation Factor 1 Gamma | Protein Coding | 38 | GC11M089615 | 0.177573 | https://www.genecards.org/cgi-bin/carddisp.pl?gene=EEF1G |
| ELL2 | Elongation Factor For RNA Polymerase II 2 | Protein Coding | 38 | GC05M095885 | 0.177573 | https://www.genecards.org/cgi-bin/carddisp.pl?gene=ELL2 |
| EPB41L2 | Erythrocyte Membrane Protein Band 4.1 Like 2 | Protein Coding | 38 | GC06M130820 | 0.177573 | https://www.genecards.org/cgi-bin/carddisp.pl?gene=EPB41L2 |
| LRRC4C | Leucine Rich Repeat Containing 4C | Protein Coding | 38 | GC11M040118 | 0.177573 | https://www.genecards.org/cgi-bin/carddisp.pl?gene=LRRC4C |
| MARCKS | Myristoylated Alanine Rich Protein Kinase C Substrate | Protein Coding | 38 | GC06P113857 | 0.177573 | https://www.genecards.org/cgi-bin/carddisp.pl?gene=MARCKS |
| MDN1 | Midasin AAA ATPase 1 | Protein Coding | 38 | GC06M089642 | 0.177573 | https://www.genecards.org/cgi-bin/carddisp.pl?gene=MDN1 |
| MGAT3 | Beta-1,4-Mannosyl-Glycoprotein 4-Beta-N-Acetylglucosaminyltransferase | Protein Coding | 38 | GC22P039447 | 0.177573 | https://www.genecards.org/cgi-bin/carddisp.pl?gene=MGAT3 |

| | | | | | | |
|---|---|---|---|---|---|---|
| MTSS1 | MTSS I-BAR Domain Containing 1 | Protein Coding | 38 | GC08M 124550 | 0.177 573 | https://www.genecards.org/cgi-bin/carddisp.pl?gene=MTSS1 |
| PARVB | Parvin Beta | Protein Coding | 38 | GC22P 043999 | 0.177 573 | https://www.genecards.org/cgi-bin/carddisp.pl?gene=PARVB |
| PGRMC2 | Progesterone Receptor Membrane Component 2 | Protein Coding | 38 | GC04M 128269 | 0.177 573 | https://www.genecards.org/cgi-bin/carddisp.pl?gene=PGRMC2 |
| PIGF | Phosphatidylinositol Glycan Anchor Biosynthesis Class F | Protein Coding | 38 | GC02M 046580 | 0.177 573 | https://www.genecards.org/cgi-bin/carddisp.pl?gene=PIGF |
| PIK3AP1 | Phosphoinositide-3-Kinase Adaptor Protein 1 | Protein Coding | 38 | GC10M 096593 | 0.177 573 | https://www.genecards.org/cgi-bin/carddisp.pl?gene=PIK3AP1 |
| PLCH1 | Phospholipase C Eta 1 | Protein Coding | 38 | GC03M 155381 | 0.177 573 | https://www.genecards.org/cgi-bin/carddisp.pl?gene=PLCH1 |
| POU6F2 | POU Class 6 Homeobox 2 | Protein Coding | 38 | GC07P 038977 | 0.177 573 | https://www.genecards.org/cgi-bin/carddisp.pl?gene=POU6F2 |
| PTPRR | Protein Tyrosine Phosphatase Receptor Type R | Protein Coding | 38 | GC12M 070638 | 0.177 573 | https://www.genecards.org/cgi-bin/carddisp.pl?gene=PTPRR |
| RNF213 | Ring Finger Protein 213 | Protein Coding | 38 | GC17P 080260 | 0.177 573 | https://www.genecards.org/cgi-bin/carddisp.pl?gene=RNF213 |
| RPL23 | Ribosomal Protein L23 | Protein Coding | 38 | GC17M 038847 | 0.177 573 | https://www.genecards.org/cgi-bin/carddisp.pl?gene=RPL23 |
| SF3B3 | Splicing Factor 3b Subunit 3 | Protein Coding | 38 | GC16P 070523 | 0.177 573 | https://www.genecards.org/cgi-bin/carddisp.pl?gene=SF3B3 |
| SFXN1 | Sideroflexin 1 | Protein Coding | 38 | GC05P 175477 | 0.177 573 | https://www.genecards.org/cgi-bin/carddisp.pl?gene=SFXN1 |
| SSR1 | Signal Sequence Receptor Subunit 1 | Protein Coding | 38 | GC06M 007268 | 0.177 573 | https://www.genecards.org/cgi-bin/carddisp.pl?gene=SSR1 |

| | | | | | | |
|---|---|---|---|---|---|---|
| TIMM44 | Translocase Of Inner Mitochondrial Membrane 44 | Protein Coding | 38 | GC19M 007926 | 0.177 573 | https://www.genecards.org/cgi-bin/carddisp.pl?gene=TIMM44 |
| TNRC6A | Trinucleotide Repeat Containing Adaptor 6A | Protein Coding | 38 | GC16P 024611 | 0.177 573 | https://www.genecards.org/cgi-bin/carddisp.pl?gene=TNRC6A |
| USP30 | Ubiquitin Specific Peptidase 30 | Protein Coding | 38 | GC12P 109027 | 0.177 573 | https://www.genecards.org/cgi-bin/carddisp.pl?gene=USP30 |
| ABI3 | ABI Family Member 3 | Protein Coding | 37 | GC17P 049210 | 0.177 573 | https://www.genecards.org/cgi-bin/carddisp.pl?gene=ABI3 |
| ADAM2 | ADAM Metallopeptidase Domain 2 | Protein Coding | 37 | GC08M 039743 | 0.177 573 | https://www.genecards.org/cgi-bin/carddisp.pl?gene=ADAM2 |
| ANO2 | Anoctamin 2 | Protein Coding | 37 | GC12M 005532 | 0.177 573 | https://www.genecards.org/cgi-bin/carddisp.pl?gene=ANO2 |
| ATP11C | ATPase Phospholipid Transporting 11C | Protein Coding | 37 | GC0X M1397 26 | 0.177 573 | https://www.genecards.org/cgi-bin/carddisp.pl?gene=ATP11C |
| BAG4 | BAG Cochaperone 4 | Protein Coding | 37 | GC08P 038176 | 0.177 573 | https://www.genecards.org/cgi-bin/carddisp.pl?gene=BAG4 |
| CAND1 | Cullin Associated And Neddylation Dissociated 1 | Protein Coding | 37 | GC12P 067270 | 0.177 573 | https://www.genecards.org/cgi-bin/carddisp.pl?gene=CAND1 |
| CD248 | CD248 Molecule | Protein Coding | 37 | GC11M 066314 | 0.177 573 | https://www.genecards.org/cgi-bin/carddisp.pl?gene=CD248 |
| CD300LB | CD300 Molecule Like Family Member B | Protein Coding | 37 | GC17M 074520 | 0.177 573 | https://www.genecards.org/cgi-bin/carddisp.pl?gene=CD300LB |
| COPG1 | COPI Coat Complex Subunit Gamma 1 | Protein Coding | 37 | GC03P 129249 | 0.177 573 | https://www.genecards.org/cgi-bin/carddisp.pl?gene=COPG1 |
| CRISP3 | Cysteine Rich Secretory Protein 3 | Protein Coding | 37 | GC06M 049727 | 0.177 573 | https://www.genecards.org/cgi-bin/carddisp.pl?gene=CRISP3 |
| CST7 | Cystatin F | Protein Coding | 37 | GC20P 024949 | 0.177 573 | https://www.genecards.org/cgi- |

| | | | | | | |
|---|---|---|---|---|---|---|
| | | | | | | bin/carddisp.pl?gene=CST7 |
| CTSW | Cathepsin W | Protein Coding | 37 | GC11P065879 | 0.177573 | https://www.genecards.org/cgi-bin/carddisp.pl?gene=CTSW |
| DAND5 | DAN Domain BMP Antagonist Family Member 5 | Protein Coding | 37 | GC19P012965 | 0.177573 | https://www.genecards.org/cgi-bin/carddisp.pl?gene=DAND5 |
| DEPTOR | DEP Domain Containing MTOR Interacting Protein | Protein Coding | 37 | GC08P119873 | 0.177573 | https://www.genecards.org/cgi-bin/carddisp.pl?gene=DEPTOR |
| EXOSC1 | Exosome Component 1 | Protein Coding | 37 | GC10M097435 | 0.177573 | https://www.genecards.org/cgi-bin/carddisp.pl?gene=EXOSC1 |
| FMNL2 | Formin Like 2 | Protein Coding | 37 | GC02P152335 | 0.177573 | https://www.genecards.org/cgi-bin/carddisp.pl?gene=FMNL2 |
| GPR158 | G Protein-Coupled Receptor 158 | Protein Coding | 37 | GC10P025174 | 0.177573 | https://www.genecards.org/cgi-bin/carddisp.pl?gene=GPR158 |
| GRK3 | G Protein-Coupled Receptor Kinase 3 | Protein Coding | 37 | GC22P036163 | 0.177573 | https://www.genecards.org/cgi-bin/carddisp.pl?gene=GRK3 |
| HNRNPUL1 | Heterogeneous Nuclear Ribonucleoprotein U Like 1 | Protein Coding | 37 | GC19P041262 | 0.177573 | https://www.genecards.org/cgi-bin/carddisp.pl?gene=HNRNPUL1 |
| IFI35 | Interferon Induced Protein 35 | Protein Coding | 37 | GC17P043006 | 0.177573 | https://www.genecards.org/cgi-bin/carddisp.pl?gene=IFI35 |
| IFT74 | Intraflagellar Transport 74 | Protein Coding | 37 | GC09P026947 | 0.177573 | https://www.genecards.org/cgi-bin/carddisp.pl?gene=IFT74 |
| INTS6 | Integrator Complex Subunit 6 | Protein Coding | 37 | GC13M051334 | 0.177573 | https://www.genecards.org/cgi-bin/carddisp.pl?gene=INTS6 |
| IPMK | Inositol Polyphosphate Multikinase | Protein Coding | 37 | GC10M058191 | 0.177573 | https://www.genecards.org/cgi-bin/carddisp.pl?gene=IPMK |
| ITGA10 | Integrin Subunit Alpha 10 | Protein Coding | 37 | GC01M145891 | 0.177573 | https://www.genecards.org/cgi-bin/carddisp.pl?gene=ITGA10 |

| | | | | | | |
|---|---|---|---|---|---|---|
| LARS1 | Leucyl-TRNA Synthetase 1 | Protein Coding | 37 | GC05M 146114 | 0.177 573 | https://www.genecards.org/cgi-bin/carddisp.pl?gene=LARS1 |
| LRRTM4 | Leucine Rich Repeat Transmembrane Neuronal 4 | Protein Coding | 37 | GC02M 076747 | 0.177 573 | https://www.genecards.org/cgi-bin/carddisp.pl?gene=LRRTM4 |
| MPRIP | Myosin Phosphatase Rho Interacting Protein | Protein Coding | 37 | GC17P 017042 | 0.177 573 | https://www.genecards.org/cgi-bin/carddisp.pl?gene=MPRIP |
| OXER1 | Oxoeicosanoid Receptor 1 | Protein Coding | 37 | GC02M 042762 | 0.177 573 | https://www.genecards.org/cgi-bin/carddisp.pl?gene=OXER1 |
| PPP1R12 B | Protein Phosphatase 1 Regulatory Subunit 12B | Protein Coding | 37 | GC01P 202348 | 0.177 573 | https://www.genecards.org/cgi-bin/carddisp.pl?gene=PPP1R12B |
| RALGAP A2 | Ral GTPase Activating Protein Catalytic Subunit Alpha 2 | Protein Coding | 37 | GC20M 020374 | 0.177 573 | https://www.genecards.org/cgi-bin/carddisp.pl?gene=RALGAPA2 |
| RBM14 | RNA Binding Motif Protein 14 | Protein Coding | 37 | GC11P 070099 | 0.177 573 | https://www.genecards.org/cgi-bin/carddisp.pl?gene=RBM14 |
| RNPS1 | RNA Binding Protein With Serine Rich Domain 1 | Protein Coding | 37 | GC16M 002253 | 0.177 573 | https://www.genecards.org/cgi-bin/carddisp.pl?gene=RNPS1 |
| SCUBE1 | Signal Peptide, CUB Domain And EGF Like Domain Containing 1 | Protein Coding | 37 | GC22M 043197 | 0.177 573 | https://www.genecards.org/cgi-bin/carddisp.pl?gene=SCUBE1 |
| SMYD1 | SET And MYND Domain Containing 1 | Protein Coding | 37 | GC02P 088075 | 0.177 573 | https://www.genecards.org/cgi-bin/carddisp.pl?gene=SMYD1 |
| SRPRB | SRP Receptor Subunit Beta | Protein Coding | 37 | GC03P 133784 | 0.177 573 | https://www.genecards.org/cgi-bin/carddisp.pl?gene=SRPRB |
| STAC | SH3 And Cysteine Rich Domain | Protein Coding | 37 | GC03P 036421 | 0.177 573 | https://www.genecards.org/cgi-bin/carddisp.pl?gene=STAC |
| TCHH | Trichohyalin | Protein Coding | 37 | GC01M 152106 | 0.177 573 | https://www.genecards.org/cgi-bin/carddisp.pl?gene=TCHH |

| | | | | | | |
|---|---|---|---|---|---|---|
| ASCL2 | Achaete-Scute Family BHLH Transcription Factor 2 | Protein Coding | 36 | GC11M 003304 | 0.177 573 | https://www.genecards .org/cgi-bin/carddisp.pl?gene= ASCL2 |
| ATAD3B | ATPase Family AAA Domain Containing 3B | Protein Coding | 36 | GC01P 003884 | 0.177 573 | https://www.genecards .org/cgi-bin/carddisp.pl?gene= ATAD3B |
| CCDC40 | Coiled-Coil Domain Containing 40 | Protein Coding | 36 | GC17P 080037 | 0.177 573 | https://www.genecards .org/cgi-bin/carddisp.pl?gene= CCDC40 |
| EAPP | E2F Associated Phosphoprotein | Protein Coding | 36 | GC14M 034516 | 0.177 573 | https://www.genecards .org/cgi-bin/carddisp.pl?gene= EAPP |
| EYS | Eyes Shut Homolog | Protein Coding | 36 | GC06M 063719 | 0.177 573 | https://www.genecards .org/cgi-bin/carddisp.pl?gene= EYS |
| IPO4 | Importin 4 | Protein Coding | 36 | GC14M 024181 | 0.177 573 | https://www.genecards .org/cgi-bin/carddisp.pl?gene=I PO4 |
| JAKMIP1 | Janus Kinase And Microtubule Interacting Protein 1 | Protein Coding | 36 | GC04M 006026 | 0.177 573 | https://www.genecards .org/cgi-bin/carddisp.pl?gene=J AKMIP1 |
| LAYN | Layilin | Protein Coding | 36 | GC11P 111717 | 0.177 573 | https://www.genecards .org/cgi-bin/carddisp.pl?gene= LAYN |
| LINGO2 | Leucine Rich Repeat And Ig Domain Containing 2 | Protein Coding | 36 | GC09M 028340 | 0.177 573 | https://www.genecards .org/cgi-bin/carddisp.pl?gene= LINGO2 |
| MMS19 | MMS19 Homolog, Cytosolic Iron-Sulfur Assembly Component | Protein Coding | 36 | GC10M 097458 | 0.177 573 | https://www.genecards .org/cgi-bin/carddisp.pl?gene= MMS19 |
| MRGPR X1 | MAS Related GPR Family Member X1 | Protein Coding | 36 | GC11M 018955 | 0.177 573 | https://www.genecards .org/cgi-bin/carddisp.pl?gene= MRGPRX1 |
| NAV1 | Neuron Navigator 1 | Protein Coding | 36 | GC01P 201563 | 0.177 573 | https://www.genecards .org/cgi-bin/carddisp.pl?gene= NAV1 |
| RAB3C | RAB3C, Member RAS Oncogene Family | Protein Coding | 36 | GC05P 058582 | 0.177 573 | https://www.genecards .org/cgi-bin/carddisp.pl?gene= RAB3C |
| SNX6 | Sorting Nexin 6 | Protein Coding | 36 | GC14M 034561 | 0.177 573 | https://www.genecards .org/cgi- |

| | | | | | | |
|---|---|---|---|---|---|---|
| | | | | | | bin/carddisp.pl?gene=SNX6 |
| SRRM2 | Serine/Arginine Repetitive Matrix 2 | Protein Coding | 36 | GC16P011722 | 0.177573 | https://www.genecards.org/cgi-bin/carddisp.pl?gene=SRRM2 |
| TMEM132D | Transmembrane Protein 132D | Protein Coding | 36 | GC12M129071 | 0.177573 | https://www.genecards.org/cgi-bin/carddisp.pl?gene=TMEM132D |
| TPTE | Transmembrane Phosphatase With Tensin Homology | Protein Coding | 36 | GC21P010521 | 0.177573 | https://www.genecards.org/cgi-bin/carddisp.pl?gene=TPTE |
| TSPAN4 | Tetraspanin 4 | Protein Coding | 36 | GC11P001793 | 0.177573 | https://www.genecards.org/cgi-bin/carddisp.pl?gene=TSPAN4 |
| TTC26 | Tetratricopeptide Repeat Domain 26 | Protein Coding | 36 | GC07P139135 | 0.177573 | https://www.genecards.org/cgi-bin/carddisp.pl?gene=TTC26 |
| TXNRD3 | Thioredoxin Reductase 3 | Protein Coding | 36 | GC03M126577 | 0.177573 | https://www.genecards.org/cgi-bin/carddisp.pl?gene=TXNRD3 |
| COQ8A | Coenzyme Q8A | Protein Coding | 35 | GC01P226939 | 0.177573 | https://www.genecards.org/cgi-bin/carddisp.pl?gene=COQ8A |
| CPNE8 | Copine 8 | Protein Coding | 35 | GC12M038646 | 0.177573 | https://www.genecards.org/cgi-bin/carddisp.pl?gene=CPNE8 |
| IARS1 | Isoleucyl-TRNA Synthetase 1 | Protein Coding | 35 | GC09M094184 | 0.177573 | https://www.genecards.org/cgi-bin/carddisp.pl?gene=IARS1 |
| MEF2B | Myocyte Enhancer Factor 2B | Protein Coding | 35 | GC19M019150 | 0.177573 | https://www.genecards.org/cgi-bin/carddisp.pl?gene=MEF2B |
| NCOA5 | Nuclear Receptor Coactivator 5 | Protein Coding | 35 | GC20M046060 | 0.177573 | https://www.genecards.org/cgi-bin/carddisp.pl?gene=NCOA5 |
| RACK1 | Receptor For Activated C Kinase 1 | Protein Coding | 35 | GC05M181795 | 0.177573 | https://www.genecards.org/cgi-bin/carddisp.pl?gene=RACK1 |
| TRIM6 | Tripartite Motif Containing 6 | Protein Coding | 35 | GC11P005596 | 0.177573 | https://www.genecards.org/cgi-bin/carddisp.pl?gene=TRIM6 |

| | | | | | | |
|---|---|---|---|---|---|---|
| WDR6 | WD Repeat Domain 6 | Protein Coding | 35 | GC03P 049007 | 0.177 573 | https://www.genecards.org/cgi-bin/carddisp.pl?gene=WDR6 |
| CD300C | CD300c Molecule | Protein Coding | 34 | GC17M 074544 | 0.177 573 | https://www.genecards.org/cgi-bin/carddisp.pl?gene=CD300C |
| CD300E | CD300e Molecule | Protein Coding | 34 | GC17M 074609 | 0.177 573 | https://www.genecards.org/cgi-bin/carddisp.pl?gene=CD300E |
| CNTNAP 5 | Contactin Associated Protein Family Member 5 | Protein Coding | 34 | GC02P 124025 | 0.177 573 | https://www.genecards.org/cgi-bin/carddisp.pl?gene=CNTNAP5 |
| FAIM2 | Fas Apoptotic Inhibitory Molecule 2 | Protein Coding | 34 | GC12M 049866 | 0.177 573 | https://www.genecards.org/cgi-bin/carddisp.pl?gene=FAIM2 |
| FOXQ1 | Forkhead Box Q1 | Protein Coding | 34 | GC06P 001312 | 0.177 573 | https://www.genecards.org/cgi-bin/carddisp.pl?gene=FOXQ1 |
| FRY | FRY Microtubule Binding Protein | Protein Coding | 34 | GC13P 031883 | 0.177 573 | https://www.genecards.org/cgi-bin/carddisp.pl?gene=FRY |
| NTPCR | Nucleoside-Triphosphatase, Cancer-Related | Protein Coding | 34 | GC01P 232950 | 0.177 573 | https://www.genecards.org/cgi-bin/carddisp.pl?gene=NTPCR |
| OTOR | Otoraplin | Protein Coding | 34 | GC20P 016748 | 0.177 573 | https://www.genecards.org/cgi-bin/carddisp.pl?gene=OTOR |
| PDCD11 | Programmed Cell Death 11 | Protein Coding | 34 | GC10P 103396 | 0.177 573 | https://www.genecards.org/cgi-bin/carddisp.pl?gene=PDCD11 |
| PGAM5 | PGAM Family Member 5, Mitochondrial Serine/Threonine Protein Phosphatase | Protein Coding | 34 | GC12P 132710 | 0.177 573 | https://www.genecards.org/cgi-bin/carddisp.pl?gene=PGAM5 |
| SGCZ | Sarcoglycan Zeta | Protein Coding | 34 | GC08M 014089 | 0.177 573 | https://www.genecards.org/cgi-bin/carddisp.pl?gene=SGCZ |
| SHISA6 | Shisa Family Member 6 | Protein Coding | 34 | GC17P 011241 | 0.177 573 | https://www.genecards.org/cgi-bin/carddisp.pl?gene=SHISA6 |
| SYT9 | Synaptotagmin 9 | Protein Coding | 34 | GC11P 007238 | 0.177 573 | https://www.genecards.org/cgi- |

| | | | | | | |
|---|---|---|---|---|---|---|
| | | | | | | bin/carddisp.pl?gene=SYT9 |
| TBC1D14 | TBC1 Domain Family Member 14 | Protein Coding | 34 | GC04P006910 | 0.177573 | https://www.genecards.org/cgi-bin/carddisp.pl?gene=TBC1D14 |
| ANKFN1 | Ankyrin Repeat And Fibronectin Type III Domain Containing 1 | Protein Coding | 33 | GC17P055882 | 0.177573 | https://www.genecards.org/cgi-bin/carddisp.pl?gene=ANKFN1 |
| ARMC3 | Armadillo Repeat Containing 3 | Protein Coding | 33 | GC10P022928 | 0.177573 | https://www.genecards.org/cgi-bin/carddisp.pl?gene=ARMC3 |
| ATP5F1B | ATP Synthase F1 Subunit Beta | Protein Coding | 33 | GC12M056646 | 0.177573 | https://www.genecards.org/cgi-bin/carddisp.pl?gene=ATP5F1B |
| ATP5PO | ATP Synthase Peripheral Stalk Subunit OSCP | Protein Coding | 33 | GC21M033904 | 0.177573 | https://www.genecards.org/cgi-bin/carddisp.pl?gene=ATP5PO |
| BEND7 | BEN Domain Containing 7 | Protein Coding | 33 | GC10M013392 | 0.177573 | https://www.genecards.org/cgi-bin/carddisp.pl?gene=BEND7 |
| C15orf48 | Chromosome 15 Open Reading Frame 48 | Protein Coding | 33 | GC15P045430 | 0.177573 | https://www.genecards.org/cgi-bin/carddisp.pl?gene=C15orf48 |
| CEP131 | Centrosomal Protein 131 | Protein Coding | 33 | GC17M081189 | 0.177573 | https://www.genecards.org/cgi-bin/carddisp.pl?gene=CEP131 |
| KMT5B | Lysine Methyltransferase 5B | Protein Coding | 33 | GC11M090188 | 0.177573 | https://www.genecards.org/cgi-bin/carddisp.pl?gene=KMT5B |
| NELFE | Negative Elongation Factor Complex Member E | Protein Coding | 33 | GC06M031952 | 0.177573 | https://www.genecards.org/cgi-bin/carddisp.pl?gene=NELFE |
| NPBWR1 | Neuropeptides B And W Receptor 1 | Protein Coding | 33 | GC08P052938 | 0.177573 | https://www.genecards.org/cgi-bin/carddisp.pl?gene=NPBWR1 |
| PTPA | Protein Phosphatase 2 Phosphatase Activator | Protein Coding | 33 | GC09P129111 | 0.177573 | https://www.genecards.org/cgi-bin/carddisp.pl?gene=PTPA |
| SLC38A10 | Solute Carrier Family 38 Member 10 | Protein Coding | 33 | GC17M081244 | 0.177573 | https://www.genecards.org/cgi-bin/carddisp.pl?gene=SLC38A10 |

| | | | | | | |
|---|---|---|---|---|---|---|
| SNX31 | Sorting Nexin 31 | Protein Coding | 33 | GC08M 100572 | 0.177 573 | https://www.genecards .org/cgi-bin/carddisp.pl?gene= SNX31 |
| SYT8 | Synaptotagmin 8 | Protein Coding | 33 | GC11P 001858 | 0.177 573 | https://www.genecards .org/cgi-bin/carddisp.pl?gene= SYT8 |
| CCT8L2 | Chaperonin Containing TCP1 Subunit 8 Like 2 | Protein Coding | 32 | GC22M 016593 | 0.177 573 | https://www.genecards .org/cgi-bin/carddisp.pl?gene= CCT8L2 |
| DNASE2 B | Deoxyribonuclease 2 Beta | Protein Coding | 32 | GC01P 084398 | 0.177 573 | https://www.genecards .org/cgi-bin/carddisp.pl?gene= DNASE2B |
| EFCAB6 | EF-Hand Calcium Binding Domain 6 | Protein Coding | 32 | GC22M 057620 | 0.177 573 | https://www.genecards .org/cgi-bin/carddisp.pl?gene= EFCAB6 |
| MON2 | MON2 Homolog, Regulator Of Endosome-To-Golgi Trafficking | Protein Coding | 32 | GC12P 062466 | 0.177 573 | https://www.genecards .org/cgi-bin/carddisp.pl?gene= MON2 |
| TMEM18 2 | Transmembrane Protein 182 | Protein Coding | 32 | GC02P 102744 | 0.177 573 | https://www.genecards .org/cgi-bin/carddisp.pl?gene= TMEM182 |
| TSPAN17 | Tetraspanin 17 | Protein Coding | 32 | GC05P 176647 | 0.177 573 | https://www.genecards .org/cgi-bin/carddisp.pl?gene= TSPAN17 |
| ZNF385D | Zinc Finger Protein 385D | Protein Coding | 32 | GC03M 021412 | 0.177 573 | https://www.genecards .org/cgi-bin/carddisp.pl?gene= ZNF385D |
| ZNF804B | Zinc Finger Protein 804B | Protein Coding | 32 | GC07P 088759 | 0.177 573 | https://www.genecards .org/cgi-bin/carddisp.pl?gene= ZNF804B |
| CD300L D | CD300 Molecule Like Family Member D | Protein Coding | 31 | GC17M 074579 | 0.177 573 | https://www.genecards .org/cgi-bin/carddisp.pl?gene= CD300LD |
| CSRNP1 | Cysteine And Serine Rich Nuclear Protein 1 | Protein Coding | 31 | GC03M 039159 | 0.177 573 | https://www.genecards .org/cgi-bin/carddisp.pl?gene= CSRNP1 |
| PROX2 | Prospero Homeobox 2 | Protein Coding | 31 | GC14M 074852 | 0.177 573 | https://www.genecards .org/cgi-bin/carddisp.pl?gene= PROX2 |
| PYCR3 | Pyrroline-5-Carboxylate Reductase 3 | Protein Coding | 31 | GC08M 143604 | 0.177 573 | https://www.genecards .org/cgi- |

| | | | | | | |
|---|---|---|---|---|---|---|
| | | | | | | bin/carddisp.pl?gene=PYCR3 |
| ATP5F1C | ATP Synthase F1 Subunit Gamma | Protein Coding | 30 | GC10P007789 | 0.177573 | https://www.genecards.org/cgi-bin/carddisp.pl?gene=ATP5F1C |
| C4orf17 | Chromosome 4 Open Reading Frame 17 | Protein Coding | 30 | GC04P099511 | 0.177573 | https://www.genecards.org/cgi-bin/carddisp.pl?gene=C4orf17 |
| RELL1 | RELT Like 1 | Protein Coding | 30 | GC04M037592 | 0.177573 | https://www.genecards.org/cgi-bin/carddisp.pl?gene=RELL1 |
| TTLL2 | Tubulin Tyrosine Ligase Like 2 | Protein Coding | 30 | GC06P167325 | 0.177573 | https://www.genecards.org/cgi-bin/carddisp.pl?gene=TTLL2 |
| GCN1 | GCN1 Activator Of EIF2AK4 | Protein Coding | 29 | GC12M120128 | 0.177573 | https://www.genecards.org/cgi-bin/carddisp.pl?gene=GCN1 |
| SEPTIN6 | Septin 6 | Protein Coding | 29 | GC0XM119616 | 0.177573 | https://www.genecards.org/cgi-bin/carddisp.pl?gene=SEPTIN6 |
| TMEM255B | Transmembrane Protein 255B | Protein Coding | 29 | GC13P113759 | 0.177573 | https://www.genecards.org/cgi-bin/carddisp.pl?gene=TMEM255B |
| TOPAZ1 | Testis And Ovary Specific TOPAZ 1 | Protein Coding | 29 | GC03P044283 | 0.177573 | https://www.genecards.org/cgi-bin/carddisp.pl?gene=TOPAZ1 |
| EMC9 | ER Membrane Protein Complex Subunit 9 | Protein Coding | 28 | GC14M024138 | 0.177573 | https://www.genecards.org/cgi-bin/carddisp.pl?gene=EMC9 |
| MTARC2 | Mitochondrial Amidoxime Reducing Component 2 | Protein Coding | 28 | GC01P220748 | 0.177573 | https://www.genecards.org/cgi-bin/carddisp.pl?gene=MTARC2 |
| REC114 | REC114 Meiotic Recombination Protein | Protein Coding | 28 | GC15P073443 | 0.177573 | https://www.genecards.org/cgi-bin/carddisp.pl?gene=REC114 |
| TENT2 | Terminal Nucleotidyltransferase 2 | Protein Coding | 28 | GC05P079613 | 0.177573 | https://www.genecards.org/cgi-bin/carddisp.pl?gene=TENT2 |
| TRIM6-TRIM34 | TRIM6-TRIM34 Readthrough | Protein Coding | 26 | GC11P005597 | 0.177573 | https://www.genecards.org/cgi-bin/carddisp.pl?gene=TRIM6-TRIM34 |

| | | | | | | |
|---|---|---|---|---|---|---|
| BAGE2 | BAGE Family Member 2, Pseudogene | Pseudo gene | 21 | GC21P 010413 | 0.177 573 | https://www.genecards .org/cgi- bin/carddisp.pl?gene= BAGE2 |
| CFAP92 | Cilia And Flagella Associated Protein 92 (Putative) | Protein Coding | 21 | GC03M 130351 | 0.177 573 | https://www.genecards .org/cgi- bin/carddisp.pl?gene= CFAP92 |
| BORCS8-MEF2B | BORCS8-MEF2B Readthrough | Protein Coding | 20 | GC19M 019146 | 0.177 573 | https://www.genecards .org/cgi- bin/carddisp.pl?gene= BORCS8-MEF2B |
| NPSR1-AS1 | NPSR1 Antisense RNA 1 | RNA Gene | 20 | GC07M 034387 | 0.177 573 | https://www.genecards .org/cgi- bin/carddisp.pl?gene= NPSR1-AS1 |
| CLRN1-AS1 | CLRN1 Antisense RNA 1 | RNA Gene | 18 | GC03P 150852 | 0.177 573 | https://www.genecards .org/cgi- bin/carddisp.pl?gene= CLRN1-AS1 |
| LINC004 69 | Long Intergenic Non-Protein Coding RNA 469 | RNA Gene | 18 | GC17M 073750 | 0.177 573 | https://www.genecards .org/cgi- bin/carddisp.pl?gene= LINC00469 |
| MIR496 | MicroRNA 496 | RNA Gene | 16 | GC14P 109999 | 0.177 573 | https://www.genecards .org/cgi- bin/carddisp.pl?gene= MIR496 |
| ANKRD3 4C-AS1 | ANKRD34C Antisense RNA 1 | RNA Gene | 14 | GC15M 090452 | 0.177 573 | https://www.genecards .org/cgi- bin/carddisp.pl?gene= ANKRD34C-AS1 |
| GAS5-AS1 | GAS5 Antisense RNA 1 | RNA Gene | 14 | GC01P 173863 | 0.177 573 | https://www.genecards .org/cgi- bin/carddisp.pl?gene= GAS5-AS1 |
| KLKP1 | Kallikrein Pseudogene 1 | Pseudo gene | 14 | GC19M 066191 | 0.177 573 | https://www.genecards .org/cgi- bin/carddisp.pl?gene= KLKP1 |
| PRICKLE 2-AS3 | PRICKLE2 Antisense RNA 3 | RNA Gene | 14 | GC03P 064187 | 0.177 573 | https://www.genecards .org/cgi- bin/carddisp.pl?gene= PRICKLE2-AS3 |
| C13orf42 | Chromosome 13 Open Reading Frame 42 | Protein Coding | 13 | GC13M 051166 | 0.177 573 | https://www.genecards .org/cgi- bin/carddisp.pl?gene= C13orf42 |
| LACTB2-AS1 | LACTB2 Antisense RNA 1 | RNA Gene | 13 | GC08P 070608 | 0.177 573 | https://www.genecards .org/cgi- bin/carddisp.pl?gene= LACTB2-AS1 |
| LINC003 31 | Long Intergenic Non-Protein Coding RNA 331 | RNA Gene | 13 | GC13M 078787 | 0.177 573 | https://www.genecards .org/cgi- |

| | | | | | | |
|---|---|---|---|---|---|---|
| | | | | | | bin/carddisp.pl?gene=LINC00331 |
| SYT9-AS1 | SYT9 Antisense RNA 1 | RNA Gene | 13 | GC11M007384 | 0.177573 | https://www.genecards.org/cgi-bin/carddisp.pl?gene=SYT9-AS1 |
| ZNF815P | Zinc Finger Protein 815, Pseudogene | Pseudogene | 13 | GC07P005823 | 0.177573 | https://www.genecards.org/cgi-bin/carddisp.pl?gene=ZNF815P |
| FILNC1 | FOXO Induced Long Non-Coding RNA 1 | RNA Gene | 12 | GC06P139677 | 0.177573 | https://www.genecards.org/cgi-bin/carddisp.pl?gene=FILNC1 |
| HRES1 | HTLV-1 Related Endogenous Sequence | Pseudogene | 12 | GC01U990083 | 0.177573 | https://www.genecards.org/cgi-bin/carddisp.pl?gene=HRES1 |
| LINC00836 | Long Intergenic Non-Protein Coding RNA 836 | RNA Gene | 12 | GC10P025651 | 0.177573 | https://www.genecards.org/cgi-bin/carddisp.pl?gene=LINC00836 |
| LINC01342 | Long Intergenic Non-Protein Coding RNA 1342 | RNA Gene | 12 | GC01P003863 | 0.177573 | https://www.genecards.org/cgi-bin/carddisp.pl?gene=LINC01342 |
| LINC02367 | Long Intergenic Non-Protein Coding RNA 2367 | RNA Gene | 12 | GC12P021743 | 0.177573 | https://www.genecards.org/cgi-bin/carddisp.pl?gene=LINC02367 |
| NIPAL4-DT | NIPAL4 Divergent Transcript | RNA Gene | 12 | GC05M157364 | 0.177573 | https://www.genecards.org/cgi-bin/carddisp.pl?gene=NIPAL4-DT |
| ADAM3B | ADAM Metallopeptidase Domain 3B (Pseudogene) | Pseudogene | 11 | GC16M049517 | 0.177573 | https://www.genecards.org/cgi-bin/carddisp.pl?gene=ADAM3B |
| IATPR | ITGB1 Adjacent Tumor Promoting LncRNA | RNA Gene | 11 | GC10M033153 | 0.177573 | https://www.genecards.org/cgi-bin/carddisp.pl?gene=IATPR |
| DNMT3AP1 | DNA Methyltransferase 3A Pseudogene 1 | Pseudogene | 9 | GC02M066819 | 0.177573 | https://www.genecards.org/cgi-bin/carddisp.pl?gene=DNMT3AP1 |
| ERVK-18 | Endogenous Retrovirus Group K Member 18 | Protein Coding | 9 | GC01U903332 | 0.177573 | https://www.genecards.org/cgi-bin/carddisp.pl?gene=ERVK-18 |
| ERVK-24 | Endogenous Retrovirus Group K Member 24 | Protein Coding | 9 | GC22U900840 | 0.177573 | https://www.genecards.org/cgi-bin/carddisp.pl?gene=ERVK-24 |

| | | | | | | |
|---|---|---|---|---|---|---|
| ISCA2P1 | Iron-Sulfur Cluster Assembly 2 Pseudogene 1 | Pseudo gene | 9 | GC22M 026644 | 0.177 573 | https://www.genecards.org/cgi-bin/carddisp.pl?gene=ISCA2P1 |
| LRRTM4 -AS1 | LRRTM4 Antisense RNA 1 | RNA Gene | 9 | GC02P 076986 | 0.177 573 | https://www.genecards.org/cgi-bin/carddisp.pl?gene=LRRTM4-AS1 |
| LOC1027 24560 | Cystathionine Beta-Synthase Like | Protein Coding | 8 | GC21M 006444 | 0.177 573 | https://www.genecards.org/cgi-bin/carddisp.pl?gene=LOC102724560 |
| FRAXA | Fragile Site, Folic Acid Type, Rare, Fra(X)(Q27.3) A | Biologi cal Region | 7 | GC0XP 147950 | 0.177 573 | https://www.genecards.org/cgi-bin/carddisp.pl?gene=FRAXA |
| ERVK-11 | Endogenous Retrovirus Group K Member 11 | Protein Coding | 6 | GC03U 901891 | 0.177 573 | https://www.genecards.org/cgi-bin/carddisp.pl?gene=ERVK-11 |
| PPIAP33 | Peptidylprolyl Isomerase A Pseudogene 33 | Pseudo gene | 5 | GC09P 007499 | 0.177 573 | https://www.genecards.org/cgi-bin/carddisp.pl?gene=PPIAP33 |
| ERVK-1 | Endogenous Retrovirus Group K Member 1 | Uncate gorized | 3 | GC01U 903451 | 0.177 573 | https://www.genecards.org/cgi-bin/carddisp.pl?gene=ERVK-1 |
| LOC1019 27484 | Uncharacterized LOC101927484 | RNA Gene | 3 | GC12M 055023 | 0.177 573 | https://www.genecards.org/cgi-bin/carddisp.pl?gene=LOC101927484 |
| LOC1066 27981 | GBA Recombination Region | Biologi cal Region | 3 | GC01P 155233 | 0.177 573 | https://www.genecards.org/cgi-bin/carddisp.pl?gene=LOC106627981 |
| HSERVP RODH | Human-Specific Endogenous Retroviral Insert PRODH Enhancer | Biologi cal Region | 2 | GC22P 037156 | 0.177 573 | https://www.genecards.org/cgi-bin/carddisp.pl?gene=HSERVPRODH |

**Table S5. List of 244 common genes (data obtained from venny)**

| Common genes |
|---|
| IL6ST |
| TYK2 |
| BLK |
| MIF |
| MMP2 |
| ABCB1 |
| PTGS2 |
| ALOX5 |

| |
|---|
| PRKCQ |
| PTGS1 |
| ADORA2A |
| HSD11B1 |
| STS |
| CTSD |
| NR3C1 |
| PTK2 |
| ABCG2 |
| MECP2 |
| PRKCH |
| MAPK14 |
| EGFR |
| AKT1 |
| ESR1 |
| AR |
| JAK1 |
| JAK2 |
| JAK3 |
| KDR |
| MMP1 |
| MMP3 |
| MMP9 |
| MMP13 |
| NOS3 |
| PDE2A |
| PIK3CA |
| PIK3CB |
| MAPK8 |
| STAT3 |
| BTK |
| TLR4 |
| TNFRSF1A |
| TYMS |
| DDR1 |
| PARP1 |
| PTGES |
| CTSK |
| NOS2 |
| P2RX7 |
| PPARG |
| CASP1 |
| ESR2 |
| FLT1 |

| |
|---|
| BRAF |
| ADA |
| CNR2 |
| CYP1A2 |
| DHODH |
| MTOR |
| IGF1R |
| MDM2 |
| ROS1 |
| TSPO |
| CD38 |
| CSF1R |
| HTR2A |
| CXCR2 |
| PRKCA |
| REN |
| ADAMTS5 |
| CCR1 |
| ERBB2 |
| ALOX15 |
| HSP90AA1 |
| CXCR1 |
| MAP3K5 |
| DDR2 |
| PDE3A |
| PFKFB3 |
| PPIA |
| PRKCB |
| RPS6KA3 |
| TACR1 |
| PER2 |
| CDK2 |
| CFTR |
| CNR1 |
| CPB1 |
| CRHR1 |
| ADRA1A |
| ADRA2B |
| CYP3A5 |
| F2R |
| FLT4 |
| BRD4 |
| GSK3B |
| HDAC1 |

| |
|---|
| INSR |
| KCNMA1 |
| MET |
| NOS1 |
| NOX4 |
| PAK1 |
| PDE7A |
| PDGFRA |
| ENPP2 |
| PGR |
| RORC |
| SLC6A2 |
| BRS3 |
| CA1 |
| SPHK1 |
| MBD2 |
| HDAC9 |
| HDAC6 |
| CDK4 |
| PIM2 |
| CHEK2 |
| CHRM3 |
| ADORA3 |
| GPBAR1 |
| CTSS |
| CYP1A1 |
| CYP2B6 |
| CYP2C19 |
| CYP3A4 |
| NQO1 |
| AGTR1 |
| DYRK1A |
| S1PR3 |
| ELANE |
| EPHA3 |
| AKT2 |
| F10 |
| FGFR1 |
| FLT3 |
| FPR1 |
| BRD1 |
| ALOX5AP |
| QPCT |
| GCK |

| |
|---|
| HCRTR1 |
| LCK |
| MAPT |
| MTNR1B |
| P2RX3 |
| P2RY1 |
| PDPK1 |
| PIM1 |
| PLK1 |
| ATR |
| EGLN1 |
| PPARA |
| PRCP |
| PRKCZ |
| MAPK9 |
| MAPK10 |
| PSEN1 |
| SPHK2 |
| PTGER4 |
| RET |
| SCD |
| SMO |
| SRC |
| AURKA |
| TACR2 |
| TACR3 |
| TGFBR1 |
| TRPV1 |
| CA2 |
| CASR |
| HDAC4 |
| CDK1 |
| SYK |
| MAP3K8 |
| LRRK2 |
| PRKCD |
| CYP2C9 |
| OPRM1 |
| TGFBR2 |
| CCR8 |
| PDGFRB |
| ALPL |
| MAPKAPK2 |
| RIPK2 |

| |
|---|
| MAP2K6 |
| HTR3A |
| MC4R |
| MAP2K3 |
| HSP90AB1 |
| BAD |
| PRKDC |
| FAAH |
| MTNR1A |
| HDAC2 |
| GRM5 |
| PLA2G7 |
| EPHA2 |
| AOC3 |
| CAPN2 |
| PTGER1 |
| METAP2 |
| DRD4 |
| LTA4H |
| CETP |
| CPT1A |
| TOP1 |
| DRD1 |
| DRD3 |
| OPRD1 |
| LDHB |
| PKM |
| ITK |
| LYN |
| ACHE |
| CAPN1 |
| CDC25B |
| ERN1 |
| CCKAR |
| PTPN1 |
| FYN |
| BACE2 |
| SCN9A |
| ABL1 |
| PTGIR |
| HUNK |
| ILK |
| NR3C2 |
| GCGR |

| |
|---|
| PDE5A |
| GNRHR |
| ADRA2C |
| MAP3K12 |
| CSNK2A1 |
| NR1H4 |
| AKR1B1 |
| CTSV |
| ADORA1 |
| HDAC10 |
| CHRM2 |
| ADORA2B |
| ECE2 |
| TNNI3K |
| FGR |
| HCK |

**Table S6. Result of PPI network analysis in String Database**

| | Identifier | Node degree |
|---|---|---|
| 1 | 9606.ENSP00000362680 | 54 |
| 2 | 9606.ENSP00000335153 | 43 |
| 3 | 9606.ENSP00000264657 | 41 |
| 4 | 9606.ENSP00000263967 | 40 |
| 5 | 9606.ENSP00000451828 | 34 |
| 6 | 9606.ENSP00000337825 | 32 |
| 7 | 9606.ENSP00000346671 | 29 |
| 8 | 9606.ENSP00000428924 | 27 |
| 9 | 9606.ENSP00000275493 | 26 |
| 10 | 9606.ENSP00000378974 | 24 |
| 11 | 9606.ENSP00000371067 | 23 |
| 12 | 9606.ENSP00000444986 | 22 |
| 12 | 9606.ENSP00000343204 | 22 |
| 12 | 9606.ENSP00000341189 | 22 |
| 12 | 9606.ENSP00000229795 | 22 |
| 16 | 9606.ENSP00000405330 | 20 |
| 17 | 9606.ENSP00000363117 | 19 |
| 17 | 9606.ENSP00000367830 | 19 |
| 17 | 9606.ENSP00000362649 | 19 |
| 20 | 9606.ENSP00000289153 | 18 |

**Table S7.** Results of KEGG analysis in ShinyGO 0.76 platform.

| Enrichment FDR | nGenes | Pathway Genes | Fold Enrichment | Pathway | URL | Genes |
|---|---|---|---|---|---|---|
| 1.11E-13 | 58 | 530 | 3.120662 | Pathways in cancer | http://www.genome.jp/kegg-bin/show_pathway?hsa05200 | BAD NOS2 FLT4 MAPK9 PIK3CB PTGS2 FGFR1 HSP90AA1 GSK3B MMP2 ESR1 HSP90AB1 JAK2 ABL1 MMP9 PIM2 AKT2 JAK3 MET TGFBR1 MAPK8 MAPK10 PDGFRB HDAC1 PIK3CA FLT3 CDK2 SMO PPARG IL6ST PDGFRA CDK4 MDM2 EGLN1 PIM1 ESR2 IGF1R ERBB2 AKT1 AGTR1 EGFR PRKCA BRAF PTGER1 JAK1 TGFBR2 RET PRKCB STAT3 AR PTK2 PTGER4 NQO1 F2R CSF1R HDAC2 MMP1 MTOR |
| 1.31E-13 | 35 | 240 | 4.797242 | Calcium signaling pathway | http://www.genome.jp/kegg-bin/show_pathway?hsa04020 | CD38 NOS2 FLT4 SPHK2 TACR2 FGFR1 P2RX7 NOS1 HTR2A FLT1 MET P2RX3 PDGFRB TACR1 ADRA1A KDR ADORA2A CHRM3 PDGFRA ERBB2 AGTR1 EGFR PRKCA PTGER1 CCKAR NOS3 RET PRKCB GRM5 TACR3 ADORA2B SPHK1 CHRM2 F2R DRD1 |
| 1.31E-13 | 42 | 350 | 3.983165 | Neuroactive ligand-receptor interaction | http://www.genome.jp/kegg-bin/show_pathway?hsa04080 | TACR2 P2RX7 TSPO BRS3 HTR2A GNRHR P2RX3 OPRM1 NR3C1 TACR1 OPRD1 CNR1 ADRA1A HCRTR1 ADORA2A CHRM3 MTNR1B AGTR1 DRD3 PTGIR PTGER1 CCKAR ADORA1 MC4R MTNR1A GRM5 TACR3 P2RY1 ADORA2B FPR1 PTGER4 CHRM2 F2R ADRA2C DRD1 CNR2 TRPV1 S1PR3 ADRA2B DRD4 ADORA3 GCGR |
| 3.09E-12 | 19 | 100 | 8.494709 | Progesterone-mediated oocyte maturation | http://www.genome.jp/kegg-bin/show_pathway?hsa04914 | MAPK9 PIK3CB HSP90AA1 PGR AURKA HSP90AB1 CDC25B AKT2 MAPK8 MAPK10 MAPK14 PIK3CA CDK2 IGF1R AKT1 BRAF PLK1 CDK1 RPS6KA3 |
| 1.00E-11 | 22 | 108 | 6.663082 | Insulin resistance | http://www.genome.jp/kegg-bin/show_pathway?hsa04931 | MAPK9 PIK3CB PRKCQ TNFRSF1A PRKCZ GSK3B AKT2 MAPK8 MAPK10 CPT1A PIK3CA PDPK1 AKT1 PRKCD NOS3 PRKCB STAT3 INSR RPS6KA3 PPARA PTPN1 MTOR |

| | | | | | | |
|---|---|---|---|---|---|---|
| 2.15E-11 | 23 | 119 | 6.082942 | Sphingolipid signaling pathway | http://www.genome.jp/kegg-bin/show_pathway?hsa04071 | FYN MAPK9 PIK3CB SPHK2 TNFRSF1A PRKCZ AKT2 MAPK8 MAPK10 MAPK14 OPRD1 CTSD PIK3CA PDPK1 AKT1 PRKCA ADORA1 NOS3 PRKCB SPHK1 MAP3K5 S1PR3 ADORA3 |
| 8.78E-11 | 22 | 95 | 5.987118 | Endocrine resistance | http://www.genome.jp/kegg-bin/show_pathway?hsa01522 | BAD MAPK9 PIK3CB MMP2 ESR1 MMP9 AKT2 MAPK8 MAPK10 MAPK14 PIK3CA CDK4 MDM2 ESR2 IGF1R ERBB2 AKT1 EGFR BRAF PTK2 SRC MTOR |
| 1.69E-10 | 21 | 79 | 6.066667 | EGFR tyrosine kinase inhibitor resistance | http://www.genome.jp/kegg-bin/show_pathway?hsa01521 | BAD PIK3CB GSK3B JAK2 AKT2 MET PDGFRB PIK3CA KDR PDGFRA IGF1R ERBB2 AKT1 EGFR PRKCA BRAF JAK1 PRKCB STAT3 SRC MTOR |
| 1.69E-10 | 30 | 210 | 4.20398 | Rap1 signaling pathway | http://www.genome.jp/kegg-bin/show_pathway?hsa04015 | MAP2K3 FLT4 PIK3CB PRKCZ FGFR1 FLT1 AKT2 MET MAP2K6 MAPK14 PDGFRB CNR1 PIK3CA KDR ADORA2A PDGFRA IGF1R AKT1 EPHA2 EGFR PRKCA BRAF PRKCB P2RY1 ADORA2B FPR1 INSR F2R CSF1R SRC |
| 2.25E-10 | 34 | 214 | 3.711886 | Lipid and atherosclerosis | http://www.genome.jp/kegg-bin/show_pathway?hsa05417 | BAD MAP2K3 MAPK9 PIK3CB TNFRSF1A HSP90AA1 GSK3B HSP90AB1 JAK2 MMP9 AKT2 MAPK8 MAP2K6 MAPK10 MAPK14 PIK3CA PPARG TLR4 CASP1 CYP2C9 CYP1A1 PDPK1 AKT1 MMP3 PRKCA NOS3 STAT3 PTK2 ERN1 MMP1 SRC CYP2B6 MAP3K5 LYN |
| 4.32E-10 | 25 | 203 | 4.790249 | Diabetic cardiomyopathy | http://www.genome.jp/kegg-bin/show_pathway?hsa05415 | MAPK9 PIK3CB PRKCZ GSK3B MMP2 MMP9 AKT2 TGFBR1 MAPK8 MAPK10 MAPK14 CTSD PIK3CA AKT1 PARP1 REN AGTR1 PRKCA TGFBR2 PRKCD NOS3 PRKCB INSR PPARA MTOR |
| 5.63E-10 | 36 | 294 | 3.414141 | MAPK signaling pathway | http://www.genome.jp/kegg-bin/show_pathway?hsa04010 | MAP2K3 FLT4 MAPK9 TNFRSF1A FGFR1 CDC25B FLT1 AKT2 MET TGFBR1 MAPK8 MAP3K8 MAP2K6 MAPK10 MAPK14 PDGFRB FLT3 KDR PDGFRA MAP3K12 IGF1R ERBB2 AKT1 EPHA2 EGFR PAK1 PRKCA BRAF MAPKAPK2 TGFBR2 PRKCB INSR RPS6KA3 CSF1R MAP3K5 MAPT |
| 1.86E-09 | 21 | 129 | 5.328829 | Relaxin signaling pathway | http://www.genome.jp/kegg-bin/show_pathway?hsa04926 | NOS2 MAPK9 PIK3CB PRKCZ MMP2 NOS1 MMP9 AKT2 TGFBR1 MAPK8 MAPK10 MAPK14 PIK3CA MMP13 AKT1 EGFR PRKCA TGFBR2 NOS3 MMP1 SRC |
| 2.03E-09 | 22 | 97 | 5.03794 | Prostate cancer | http://www.genome.jp/kegg-bin/show_pathway?hsa05215 | BAD PIK3CB FGFR1 HSP90AA1 GSK3B HSP90AB1 MMP9 AKT2 PDGFRB PIK3CA CDK2 PDGFRA MDM2 IGF1R PDPK1 ERBB2 AKT1 EGFR MMP3 BRAF AR MTOR |
| 2.20E-09 | 19 | 84 | 5.848816 | ErbB signaling pathway | http://www.genome.jp/kegg-bin/show_pathway?hsa04012 | BAD MAPK9 PIK3CB GSK3B ABL1 AKT2 MAPK8 MAPK10 PIK3CA ERBB2 AKT1 EGFR PAK1 PRKCA BRAF PRKCB PTK2 SRC MTOR |
| 2.43E-09 | 28 | 200 | 3.953216 | Focal adhesion | http://www.genome.jp/kegg-bin/show_pathway?hsa04510 | BAD FYN FLT4 MAPK9 PIK3CB GSK3B FLT1 AKT2 MET MAPK8 MAPK10 PDGFRB PIK3CA KDR PDGFRA IGF1R PDPK1 ERBB2 AKT1 EGFR PAK1 PRKCA BRAF CAPN2 ILK PRKCB PTK2 SRC |
| 3.39E-09 | 18 | 68 | 6.035714 | Fc epsilon RI signaling pathway | http://www.genome.jp/kegg-bin/show_pathway?hsa04664 | BTK FYN MAP2K3 MAPK9 PIK3CB AKT2 MAPK8 MAP2K6 MAPK10 MAPK14 PIK3CA ALOX5AP PDPK1 AKT1 PRKCA SYK LYN ALOX5 |
| 4.09E-09 | 16 | 59 | 6.828283 | VEGF signaling pathway | http://www.genome.jp/kegg-bin/show_pathway?hsa04370 | BAD PIK3CB SPHK2 PTGS2 AKT2 MAPK14 PIK3CA KDR AKT1 PRKCA MAPKAPK2 NOS3 PRKCB PTK2 SPHK1 SRC |
| 5.27E-09 | 22 | 100 | 4.748404 | AGE-RAGE signaling pathway in diabetic complications | http://www.genome.jp/kegg-bin/show_pathway?hsa04933 | MAPK9 PIK3CB PRKCZ NOX4 MMP2 JAK2 AKT2 TGFBR1 MAPK8 MAPK10 MAPK14 PIK3CA CDK4 PIM1 AKT1 AGTR1 PRKCA TGFBR2 PRKCD NOS3 PRKCB STAT3 |
| 8.04E-09 | 37 | 354 | 3.007696 | PI3K-Akt signaling pathway | http://www.genome.jp/kegg-bin/show_pathway?hsa04151 | BAD FLT4 PIK3CB FGFR1 HSP90AA1 GSK3B HSP90AB1 JAK2 FLT1 AKT2 JAK3 MET PDGFRB PIK3CA FLT3 CDK2 KDR PDGFRA CDK4 MDM2 TLR4 IGF1R PDPK1 ERBB2 AKT1 EPHA2 EGFR PRKCA JAK1 NOS3 SYK PTK2 INSR CHRM2 F2R CSF1R MTOR |
| 1.59E-08 | 24 | 197 | 4.134557 | Chemical carcinogenesis | http://www.genome.jp/kegg-bin/show_pathway?hsa05207 | BAD PIK3CB HSP90AA1 PGR ESR1 HSP90AB1 JAK2 AKT2 PIK3CA ESR2 CYP1A1 CYP1A2 AKT1 EGFR PRKCA CYP3A4 PRKCB STAT3 AR RPS6KA3 PPARA SRC CYP2B6 MTOR |
| 2.70E-08 | 19 | 148 | 5.025039 | Phospholipase D signaling pathway | http://www.genome.jp/kegg-bin/show_pathway?hsa04072 | FYN PIK3CB SPHK2 AKT2 PDGFRB PIK3CA PDGFRA AKT1 AGTR1 EGFR PRKCA CXCR1 SYK GRM5 INSR SPHK1 CXCR2 F2R MTOR |
| 7.39E-08 | 16 | 70 | 5.668763 | Central carbon metabolism in cancer | http://www.genome.jp/kegg-bin/show_pathway?hsa05230 | PIK3CB PKM FGFR1 AKT2 MET GCK LDHB PDGFRB PIK3CA FLT3 PDGFRA ERBB2 AKT1 EGFR RET MTOR |
| 8.76E-08 | 17 | 98 | 5.233151 | Inflammatory mediator regulation of TRP channels | http://www.genome.jp/kegg-bin/show_pathway?hsa04750 | PRKCH MAP2K3 MAPK9 PIK3CB PRKCQ HTR2A MAPK8 MAP2K6 MAPK10 MAPK14 PIK3CA PRKCA PRKCD PRKCB PTGER4 TRPV1 SRC |
| 8.76E-08 | 17 | 76 | 5.233151 | Pancreatic cancer | http://www.genome.jp/kegg-bin/show_pathway?hsa05212 | BAD MAPK9 PIK3CB AKT2 TGFBR1 MAPK8 MAPK10 PIK3CA CDK4 ERBB2 AKT1 EGFR BRAF JAK1 TGFBR2 STAT3 MTOR |
| 1.36E-07 | 26 | 202 | 3.462569 | Proteoglycans in cancer | http://www.genome.jp/kegg-bin/show_pathway?hsa05205 | PIK3CB FGFR1 MMP2 ESR1 MMP9 AKT2 MET MAPK14 PIK3CA KDR SMO MDM2 TLR4 IGF1R PDPK1 ERBB2 AKT1 EGFR PAK1 PRKCA BRAF PRKCB STAT3 PTK2 SRC MTOR |

| | | | | | | |
|---|---|---|---|---|---|---|
| 1.44E-07 | 25 | 161 | 3.556397 | Hepatitis B | http://www.genome.jp/kegg-bin/show_pathway?hsa05161 | BAD MAP2K3 MAPK9 PIK3CB JAK2 MMP9 AKT2 TYK2 JAK3 TGFBR1 MAPK8 MAP2K6 MAPK10 MAPK14 PIK3CA CDK2 TLR4 AKT1 PRKCA BRAF JAK1 TGFBR2 PRKCB STAT3 SRC |
| 2.00E-07 | 26 | 194 | 3.390432 | Kaposi sarcoma-associated herpesvirus infection | http://www.genome.jp/kegg-bin/show_pathway?hsa05167 | MAPK9 PIK3CB TNFRSF1A PTGS2 GSK3B JAK2 HCK AKT2 TYK2 MAPK8 MAP2K6 MAPK10 MAPK14 PIK3CA IL6ST CDK4 AKT1 JAK1 MAPKAPK2 CCR1 SYK STAT3 CCR8 SRC MTOR LYN |
| 2.14E-07 | 19 | 109 | 4.404664 | HIF-1 signaling pathway | http://www.genome.jp/kegg-bin/show_pathway?hsa04066 | NOS2 PIK3CB FLT1 AKT2 LDHB PIK3CA EGLN1 TLR4 IGF1R ERBB2 AKT1 EGFR PRKCA NOS3 PRKCB STAT3 PFKFB3 INSR MTOR |
| 2.32E-07 | 16 | 72 | 5.180077 | Non-small cell lung cancer | http://www.genome.jp/kegg-bin/show_pathway?hsa05223 | BAD PIK3CB AKT2 JAK3 MET PIK3CA CDK4 PDPK1 ERBB2 AKT1 EGFR PRKCA BRAF RET PRKCB STAT3 |
| 2.37E-07 | 25 | 231 | 3.451797 | Ras signaling pathway | http://www.genome.jp/kegg-bin/show_pathway?hsa04014 | BAD FLT4 MAPK9 PIK3CB FGFR1 ABL1 FLT1 AKT2 MET MAPK8 MAPK10 PDGFRB PIK3CA FLT3 KDR PDGFRA IGF1R AKT1 EPHA2 EGFR PAK1 PRKCA PRKCB INSR CSF1R |
| 3.67E-07 | 19 | 119 | 4.247354 | Neurotrophin signaling pathway | http://www.genome.jp/kegg-bin/show_pathway?hsa04722 | BAD MAPK9 PIK3CB PSEN1 GSK3B ABL1 RIPK2 AKT2 MAPK8 MAPK10 MAPK14 PIK3CA PDPK1 AKT1 BRAF MAPKAPK2 PRKCD RPS6KA3 MAP3K5 |
| 7.68E-07 | 17 | 138 | 4.496088 | Estrogen signaling pathway | http://www.genome.jp/kegg-bin/show_pathway?hsa04915 | PIK3CB HSP90AA1 PGR MMP2 ESR1 HSP90AB1 MMP9 AKT2 OPRM1 CTSD PIK3CA ESR2 AKT1 EGFR PRKCD NOS3 SRC |
| 7.79E-07 | 21 | 138 | 3.720126 | Fluid shear stress and atherosclerosis | http://www.genome.jp/kegg-bin/show_pathway?hsa05418 | MAPK9 PIK3CB TNFRSF1A PRKCZ HSP90AA1 MMP2 HSP90AB1 MMP9 AKT2 MAPK8 MAP2K6 MAPK10 MAPK14 PIK3CA KDR AKT1 NOS3 PTK2 NQO1 SRC MAP3K5 |
| 8.50E-07 | 15 | 70 | 5.029762 | Prolactin signaling pathway | http://www.genome.jp/kegg-bin/show_pathway?hsa04917 | MAPK9 PIK3CB GSK3B ESR1 JAK2 AKT2 GCK MAPK8 MAPK10 MAPK14 PIK3CA ESR2 AKT1 STAT3 SRC |
| 1.06E-06 | 19 | 131 | 3.964198 | FoxO signaling pathway | http://www.genome.jp/kegg-bin/show_pathway?hsa04068 | MAPK9 PIK3CB AKT2 TGFBR1 MAPK8 MAPK10 MAPK14 PIK3CA CDK2 MDM2 IGF1R PDPK1 AKT1 EGFR BRAF TGFBR2 PLK1 STAT3 INSR |
| 1.06E-06 | 17 | 119 | 4.372907 | Growth hormone synthesis, secretion and action | http://www.genome.jp/kegg-bin/show_pathway?hsa04935 | MAP2K3 MAPK9 PIK3CB GSK3B JAK2 AKT2 MAPK8 MAP2K6 MAPK10 MAPK14 PIK3CA AKT1 PRKCA PRKCB STAT3 PTK2 MTOR |
| 1.13E-06 | 24 | 191 | 3.2657 | Chemokine signaling pathway | http://www.genome.jp/kegg-bin/show_pathway?hsa04062 | FGR BAD PIK3CB PRKCZ GSK3B JAK2 HCK AKT2 JAK3 ITK PIK3CA AKT1 PAK1 BRAF CXCR1 CCR1 PRKCD PRKCB STAT3 PTK2 CCR8 CXCR2 SRC LYN |
| 1.13E-06 | 11 | 46 | 6.885185 | Type II diabetes mellitus | http://www.genome.jp/kegg-bin/show_pathway?hsa04930 | MAPK9 PIK3CB PKM PRKCZ GCK MAPK8 MAPK10 PIK3CA PRKCD INSR MTOR |
| 1.52E-06 | 21 | 126 | 3.552553 | Osteoclast differentiation | http://www.genome.jp/kegg-bin/show_pathway?hsa04380 | BTK FYN MAPK9 PIK3CB TNFRSF1A AKT2 TYK2 TGFBR1 MAPK8 MAP2K6 MAPK10 MAPK14 PIK3CA PPARG AKT1 CTSK JAK1 TGFBR2 SYK CSF1R LCK |
| 1.94E-06 | 16 | 124 | 4.418301 | Platelet activation | http://www.genome.jp/kegg-bin/show_pathway?hsa04611 | BTK FYN PIK3CB PRKCZ PTGS1 AKT2 MAPK14 PIK3CA AKT1 PTGIR NOS3 SYK P2RY1 F2R SRC LYN |
| 2.15E-06 | 27 | 383 | 2.897143 | Alzheimer disease | http://www.genome.jp/kegg-bin/show_pathway?hsa05010 | BAD NOS2 CAPN1 MAPK9 PIK3CB TNFRSF1A PTGS2 PSEN1 GSK3B NOX4 NOS1 CSNK2A1 AKT2 MAPK8 MAPK10 PIK3CA CHRM3 AKT1 BRAF CAPN2 GRM5 INSR ERN1 BACE2 MAP3K5 MTOR MAPT |
| 3.40E-06 | 24 | 224 | 3.06576 | Human cytomegalovirus infection | http://www.genome.jp/kegg-bin/show_pathway?hsa05163 | PIK3CB TNFRSF1A PTGS2 GSK3B AKT2 MAP2K6 MAPK14 PIK3CA PDGFRA CDK4 MDM2 AKT1 EGFR PRKCA PTGER1 JAK1 CCR1 PRKCB STAT3 PTK2 PTGER4 CXCR2 SRC MTOR |
| 3.73E-06 | 18 | 159 | 3.797753 | Necroptosis | http://www.genome.jp/kegg-bin/show_pathway?hsa04217 | CAPN1 MAPK9 TNFRSF1A HSP90AA1 HSP90AB1 JAK2 TYK2 JAK3 MAPK8 MAPK10 TLR4 CASP1 PARP1 ALOX15 JAK1 CAPN2 STAT3 PPIA |
| 4.10E-06 | 16 | 137 | 4.17284 | Insulin signaling pathway | http://www.genome.jp/kegg-bin/show_pathway?hsa04910 | BAD MAPK9 PIK3CB PRKCZ GSK3B AKT2 GCK MAPK8 MAPK10 PIK3CA PDPK1 AKT1 BRAF INSR PTPN1 MTOR |
| 4.40E-06 | 22 | 203 | 3.202412 | Viral carcinogenesis | http://www.genome.jp/kegg-bin/show_pathway?hsa05203 | BAD HDAC9 PIK3CB PKM HDAC4 HDAC10 JAK3 HDAC1 PIK3CA CDK2 IL6ST CDK4 MDM2 JAK1 MAPKAPK2 SYK STAT3 CDK1 CCR8 HDAC2 SRC LYN |
| 4.40E-06 | 14 | 75 | 4.694444 | Glioma | http://www.genome.jp/kegg-bin/show_pathway?hsa05214 | PIK3CB AKT2 PDGFRB PIK3CA PDGFRA CDK4 MDM2 IGF1R AKT1 EGFR PRKCA BRAF PRKCB MTOR |
| 5.76E-06 | 21 | 161 | 3.258953 | MicroRNAs in cancer | http://www.genome.jp/kegg-bin/show_pathway?hsa05206 | PIK3CB HDAC4 PTGS2 ABCB1 ABL1 MMP9 CDC25B MET PDGFRB HDAC1 PIK3CA PDGFRA MDM2 PIM1 ERBB2 EGFR PRKCA PRKCB STAT3 HDAC2 MTOR |
| 6.04E-06 | 23 | 246 | 3.041471 | Shigellosis | http://www.genome.jp/kegg-bin/show_pathway?hsa05131 | CAPN1 MAPK9 PIK3CB PRKCQ TNFRSF1A GSK3B RIPK2 AKT2 MAPK8 MAPK10 MAPK14 PIK3CA MDM2 TLR4 CASP1 AKT1 EGFR CAPN2 PRKCD ILK PTK2 SRC MTOR |
| 6.46E-06 | 14 | 72 | 4.532567 | Melanoma | http://www.genome.jp/kegg-bin/show_pathway?hsa05218 | BAD PIK3CB FGFR1 AKT2 MET PDGFRB PIK3CA PDGFRA CDK4 MDM2 IGF1R AKT1 EGFR BRAF |
| 6.52E-06 | 16 | 89 | 4.005926 | PD-L1 expression and PD-1 checkpoint pathway in cancer | http://www.genome.jp/kegg-bin/show_pathway?hsa05235 | MAP2K3 PIK3CB PRKCQ JAK2 CSNK2A1 AKT2 MAP2K6 MAPK14 PIK3CA TLR4 AKT1 EGFR JAK1 STAT3 LCK MTOR |

| | | | | | | |
|---|---|---|---|---|---|---|
| 7.62E-06 | 16 | 166 | 3.953216 | CGMP-PKG signaling pathway | http://www.genome.jp/kegg-bin/show_pathway?hsa04022 | BAD AKT2 OPRD1 ADRA1A PDE5A AKT1 AGTR1 KCNMA1 ADORA1 NOS3 INSR PDE3A ADRA2C PDE2A ADRA2B ADORA3 |
| 7.62E-06 | 14 | 98 | 4.455744 | Choline metabolism in cancer | http://www.genome.jp/kegg-bin/show_pathway?hsa05231 | MAPK9 PIK3CB AKT2 MAPK8 MAPK10 PDGFRB PIK3CA PDGFRA PDPK1 AKT1 EGFR PRKCA PRKCB MTOR |
| 7.65E-06 | 19 | 190 | 3.430556 | Neutrophil extracellular trap formation | http://www.genome.jp/kegg-bin/show_pathway?hsa04613 | HDAC9 PIK3CB HDAC6 HDAC10 AKT2 MAPK14 HDAC1 PIK3CA TLR4 CASP1 AKT1 PRKCA SYK PRKCB FPR1 HDAC2 SRC MTOR ELANE |
| 8.22E-06 | 18 | 112 | 3.557895 | Toxoplasmosis | http://www.genome.jp/kegg-bin/show_pathway?hsa05145 | BAD NOS2 MAP2K3 MAPK9 TNFRSF1A JAK2 AKT2 TYK2 MAPK8 MAP2K6 MAPK10 MAPK14 TLR4 PDPK1 AKT1 JAK1 STAT3 ALOX5 |
| 8.38E-06 | 12 | 69 | 5.121212 | Adipocytokine signaling pathway | http://www.genome.jp/kegg-bin/show_pathway?hsa04920 | MAPK9 PRKCQ TNFRSF1A JAK2 AKT2 MAPK8 MAPK10 CPT1A AKT1 STAT3 PPARA MTOR |
| 8.97E-06 | 13 | 93 | 4.694444 | GnRH signaling pathway | http://www.genome.jp/kegg-bin/show_pathway?hsa04912 | MAP2K3 MAPK9 MMP2 MAPK8 MAP2K6 GNRHR MAPK10 MAPK14 EGFR PRKCA PRKCD PRKCB SRC |
| 9.31E-06 | 15 | 141 | 4.082126 | Autophagy | http://www.genome.jp/kegg-bin/show_pathway?hsa04140 | BAD MAPK9 PIK3CB PRKCQ AKT2 MAPK8 MAPK10 CTSD PIK3CA IGF1R PDPK1 AKT1 PRKCD ERN1 MTOR |
| 9.31E-06 | 19 | 135 | 3.365828 | Apoptosis | http://www.genome.jp/kegg-bin/show_pathway?hsa04210 | BAD CAPN1 MAPK9 PIK3CB TNFRSF1A AKT2 MAPK8 MAPK10 CTSD PIK3CA CTSV PDPK1 AKT1 CTSK PARP1 CAPN2 CTSS ERN1 MAP3K5 |
| 9.31E-06 | 17 | 103 | 3.669221 | T cell receptor signaling pathway | http://www.genome.jp/kegg-bin/show_pathway?hsa04660 | FYN MAPK9 PIK3CB PRKCQ GSK3B AKT2 MAPK8 MAP3K8 MAPK10 MAPK14 ITK PIK3CA CDK4 PDPK1 AKT1 PAK1 LCK |
| 9.31E-06 | 15 | 121 | 4.082126 | Thyroid hormone signaling pathway | http://www.genome.jp/kegg-bin/show_pathway?hsa04919 | BAD PIK3CB GSK3B ESR1 AKT2 HDAC1 PIK3CA MDM2 PDPK1 AKT1 PRKCA PRKCB HDAC2 SRC MTOR |
| 9.86E-06 | 12 | 71 | 5.007407 | Adherens junction | http://www.genome.jp/kegg-bin/show_pathway?hsa04520 | FYN FGFR1 CSNK2A1 MET TGFBR1 IGF1R ERBB2 EGFR TGFBR2 INSR PTPN1 SRC |
| 1.19E-05 | 19 | 156 | 3.303498 | Cellular senescence | http://www.genome.jp/kegg-bin/show_pathway?hsa04218 | CAPN1 MAP2K3 PIK3CB AKT2 TGFBR1 MAP2K6 MAPK14 PIK3CA CDK2 CDK4 MDM2 AKT1 MAPKAPK2 CAPN2 TGFBR2 CDK1 ATR CHEK2 MTOR |
| 1.32E-05 | 18 | 108 | 3.414141 | Th17 cell differentiation | http://www.genome.jp/kegg-bin/show_pathway?hsa04659 | MAPK9 PRKCQ HSP90AA1 HSP90AB1 JAK2 TYK2 JAK3 TGFBR1 MAPK8 MAPK10 MAPK14 IL6ST RORC JAK1 TGFBR2 STAT3 LCK MTOR |
| 1.76E-05 | 14 | 120 | 4.107639 | AMPK signaling pathway | http://www.genome.jp/kegg-bin/show_pathway?hsa04152 | CFTR PIK3CB SCD AKT2 CPT1A ADRA1A PIK3CA PPARG IGF1R PDPK1 AKT1 PFKFB3 INSR MTOR |
| 2.08E-05 | 16 | 104 | 3.619813 | C-type lectin receptor signaling pathway | http://www.genome.jp/kegg-bin/show_pathway?hsa04625 | MAPK9 PIK3CB PTGS2 AKT2 MAPK8 MAPK10 MAPK14 PIK3CA MDM2 CASP1 AKT1 PAK1 MAPKAPK2 PRKCD SYK SRC |
| 2.64E-05 | 27 | 475 | 2.497537 | Pathways of neurodegeneration | http://www.genome.jp/kegg-bin/show_pathway?hsa05022 | BAD NOS2 CAPN1 MAP2K3 MAPK9 TNFRSF1A PTGS2 PSEN1 GSK3B NOX4 NOS1 CSNK2A1 MAPK8 MAP2K6 MAPK10 MAPK14 CHRM3 PRKCA BRAF CAPN2 PRKCB GRM5 ERN1 LRRK2 MAP3K5 MTOR MAPT |
| 2.81E-05 | 13 | 97 | 4.208812 | Fc gamma R-mediated phagocytosis | http://www.genome.jp/kegg-bin/show_pathway?hsa04666 | PIK3CB SPHK2 HCK AKT2 PIK3CA AKT1 PAK1 PRKCA PRKCD SYK PRKCB SPHK1 LYN |
| 2.91E-05 | 17 | 167 | 3.360234 | Hepatocellular carcinoma | http://www.genome.jp/kegg-bin/show_pathway?hsa05225 | BAD PIK3CB GSK3B AKT2 MET TGFBR1 PIK3CA CDK4 IGF1R AKT1 EGFR PRKCA BRAF TGFBR2 PRKCB NQO1 MTOR |
| 3.18E-05 | 16 | 181 | 3.49354 | Axon guidance | http://www.genome.jp/kegg-bin/show_pathway?hsa04360 | FYN EPHA3 PIK3CB PRKCZ GSK3B ABL1 MET PIK3CA SMO PDPK1 EPHA2 PAK1 PRKCA ILK PTK2 SRC |
| 3.29E-05 | 13 | 67 | 4.137476 | Acute myeloid leukemia | http://www.genome.jp/kegg-bin/show_pathway?hsa05221 | BAD PIK3CB PIM2 AKT2 PIK3CA FLT3 PER2 PIM1 AKT1 BRAF STAT3 CSF1R MTOR |
| 3.39E-05 | 14 | 86 | 3.866013 | Colorectal cancer | http://www.genome.jp/kegg-bin/show_pathway?hsa05210 | BAD MAPK9 PIK3CB GSK3B AKT2 TGFBR1 MAPK8 MAPK10 PIK3CA AKT1 EGFR BRAF TGFBR2 MTOR |
| 3.48E-05 | 11 | 58 | 4.803618 | Endometrial cancer | http://www.genome.jp/kegg-bin/show_pathway?hsa05213 | BAD PIK3CB GSK3B AKT2 PIK3CA PDPK1 ERBB2 AKT1 EGFR BRAF ILK |
| 5.50E-05 | 11 | 113 | 4.590123 | Cholinergic synapse | http://www.genome.jp/kegg-bin/show_pathway?hsa04725 | FYN PIK3CB ACHE JAK2 AKT2 PIK3CA CHRM3 AKT1 PRKCA PRKCB CHRM2 |
| 5.55E-05 | 13 | 76 | 3.937276 | Chronic myeloid leukemia | http://www.genome.jp/kegg-bin/show_pathway?hsa05220 | BAD PIK3CB ABL1 AKT2 TGFBR1 HDAC1 PIK3CA CDK4 MDM2 AKT1 BRAF TGFBR2 HDAC2 |
| 6.21E-05 | 16 | 147 | 3.301587 | Breast cancer | http://www.genome.jp/kegg-bin/show_pathway?hsa05224 | FLT4 PIK3CB FGFR1 PGR GSK3B ESR1 AKT2 PIK3CA CDK4 ESR2 IGF1R ERBB2 AKT1 EGFR BRAF MTOR |
| 6.35E-05 | 14 | 154 | 3.651235 | MTOR signaling pathway | http://www.genome.jp/kegg-bin/show_pathway?hsa04150 | PIK3CB TNFRSF1A GSK3B AKT2 PIK3CA IGF1R PDPK1 AKT1 PRKCA BRAF PRKCB INSR RPS6KA3 MTOR |
| 6.55E-05 | 11 | 70 | 4.490338 | Epithelial cell signaling in Helicobacter pylori infection | http://www.genome.jp/kegg-bin/show_pathway?hsa05120 | MAPK9 MET MAPK8 MAPK10 MAPK14 EGFR PAK1 CXCR1 CXCR2 SRC LYN |
| 6.64E-05 | 22 | 202 | 2.648148 | Epstein-Barr virus infection | http://www.genome.jp/kegg-bin/show_pathway?hsa05169 | BTK MAP2K3 MAPK9 PIK3CB AKT2 TYK2 JAK3 MAPK8 MAP2K6 MAPK10 MAPK14 HDAC1 PIK3CA CDK2 CDK4 MDM2 AKT1 JAK1 SYK STAT3 HDAC2 LYN |

| | | | | | | |
|---|---|---|---|---|---|---|
| 7.62E-05 | 20 | 210 | 2.781893 | Human immunodeficiency virus 1 infection | http://www.genome.jp/kegg-bin/show_pathway?hsa05170 | BAD MAP2K3 MAPK9 PIK3CB TNFRSF1A AKT2 MAPK8 MAP2K6 MAPK10 MAPK14 PIK3CA TLR4 AKT1 PAK1 PRKCA PRKCB PTK2 CDK1 ATR MTOR |
| 7.63E-05 | 15 | 155 | 3.393574 | Non-alcoholic fatty liver disease | http://www.genome.jp/kegg-bin/show_pathway?hsa04932 | MAPK9 PIK3CB TNFRSF1A GSK3B AKT2 MAPK8 MAPK10 MAPK14 PIK3CA PPARG AKT1 INSR ERN1 PPARA MAP3K5 |
| 8.51E-05 | 17 | 137 | 3.069444 | Yersinia infection | http://www.genome.jp/kegg-bin/show_pathway?hsa05135 | MAP2K3 MAPK9 PIK3CB GSK3B AKT2 MAPK8 MAP2K6 MAPK10 MAPK14 PIK3CA TLR4 CASP1 AKT1 PTK2 RPS6KA3 LCK SRC |
| 9.77E-05 | 10 | 148 | 4.694444 | Retrograde endocannabinoid signaling | http://www.genome.jp/kegg-bin/show_pathway?hsa04723 | MAPK9 PTGS2 MAPK8 MAPK10 MAPK14 FAAH CNR1 PRKCA PRKCB GRM5 |
| 0.000101 | 12 | 132 | 3.953216 | Dopaminergic synapse | http://www.genome.jp/kegg-bin/show_pathway?hsa04728 | MAPK9 GSK3B AKT2 MAPK8 MAPK10 MAPK14 AKT1 DRD3 PRKCA PRKCB DRD1 DRD4 |
| 0.000125 | 18 | 219 | 2.864407 | CAMP signaling pathway | http://www.genome.jp/kegg-bin/show_pathway?hsa04024 | CFTR BAD MAPK9 PIK3CB AKT2 MAPK8 MAPK10 PIK3CA ADORA2A AKT1 PAK1 BRAF ADORA1 PDE3A CHRM2 F2R DRD1 PPARA |
| 0.000137 | 19 | 179 | 2.744444 | Tuberculosis | http://www.genome.jp/kegg-bin/show_pathway?hsa05152 | BAD NOS2 MAPK9 SPHK2 TNFRSF1A JAK2 RIPK2 AKT2 MAPK8 MAPK10 MAPK14 CTSD TLR4 AKT1 JAK1 CTSS SYK SPHK1 SRC |
| 0.000137 | 24 | 331 | 2.397163 | Human papillomavirus infection | http://www.genome.jp/kegg-bin/show_pathway?hsa05165 | BAD PIK3CB TNFRSF1A PKM PRKCZ PTGS2 PSEN1 GSK3B AKT2 TYK2 PDGFRB HDAC1 PIK3CA CDK2 CDK4 MDM2 AKT1 EGFR JAK1 PTK2 PTGER4 ATR HDAC2 MTOR |
| 0.000153 | 44 | 1527 | 1.807926 | Metabolic pathways | http://www.genome.jp/kegg-bin/show_pathway?hsa01100 | CD38 NOS2 PIK3CB SPHK2 PKM PTGS2 AKR1B1 NOS1 PTGS1 SCD STS DHODH CA2 CYP3A5 GCK LTA4H LDHB HSD11B1 PIK3CA AOC3 CA1 ENPP2 CYP2C9 PDE5A CYP1A1 CYP1A2 PLA2G7 PTGES CYP3A4 ALOX15 ALPL NOS3 CYP2C19 PFKFB3 PDE3A SPHK1 TYMS NQO1 PDE2A ADA CYP2B6 PDE7A ALOX5 MIF |
| 0.000157 | 16 | 141 | 3.034792 | Alcoholic liver disease | http://www.genome.jp/kegg-bin/show_pathway?hsa04936 | MAP2K3 MAPK9 TNFRSF1A GSK3B NOX4 SCD AKT2 MAPK8 MAP2K6 MAPK10 CPT1A MAPK14 TLR4 AKT1 PPARA MAP3K5 |
| 0.00016 | 11 | 112 | 4.050109 | Serotonergic synapse | http://www.genome.jp/kegg-bin/show_pathway?hsa04726 | PTGS2 PTGS1 HTR2A CYP2C9 PRKCA BRAF ALOX15 CYP2C19 PRKCB HTR3A ALOX5 |
| 0.000179 | 13 | 143 | 3.487302 | Signaling pathways regulating pluripotency of stem cells | http://www.genome.jp/kegg-bin/show_pathway?hsa04550 | PIK3CB FGFR1 GSK3B JAK2 AKT2 JAK3 MAPK14 PIK3CA IL6ST IGF1R AKT1 JAK1 STAT3 |
| 0.000199 | 8 | 56 | 5.365079 | Regulation of lipolysis in adipocytes | http://www.genome.jp/kegg-bin/show_pathway?hsa04923 | PIK3CB PTGS2 PTGS1 AKT2 PIK3CA AKT1 ADORA1 INSR |
| 0.0002 | 17 | 139 | 2.850198 | Measles | http://www.genome.jp/kegg-bin/show_pathway?hsa05162 | BAD MAPK9 PIK3CB GSK3B CSNK2A1 AKT2 TYK2 JAK3 MAPK8 MAPK10 PIK3CA CDK2 CDK4 TLR4 AKT1 JAK1 STAT3 |
| 0.000206 | 20 | 232 | 2.572298 | Coronavirus disease | http://www.genome.jp/kegg-bin/show_pathway?hsa05171 | MAPK9 PIK3CB TNFRSF1A TYK2 MAPK8 MAPK10 MAPK14 PIK3CA IL6ST TLR4 CASP1 AGTR1 EGFR MMP3 PRKCA JAK1 SYK PRKCB STAT3 MMP1 |
| 0.000215 | 9 | 62 | 4.694444 | Longevity regulating pathway | http://www.genome.jp/kegg-bin/show_pathway?hsa04213 | PIK3CB AKT2 HDAC1 PIK3CA IGF1R AKT1 INSR HDAC2 MTOR |
| 0.000239 | 16 | 112 | 2.916936 | TNF signaling pathway | http://www.genome.jp/kegg-bin/show_pathway?hsa04668 | MAP2K3 MAPK9 PIK3CB TNFRSF1A PTGS2 MMP9 AKT2 MAPK8 MAP3K8 MAP2K6 MAPK10 MAPK14 PIK3CA AKT1 MMP3 MAP3K5 |
| 0.000292 | 12 | 126 | 3.520833 | Cell cycle | http://www.genome.jp/kegg-bin/show_pathway?hsa04110 | GSK3B ABL1 CDC25B HDAC1 CDK4 MDM2 PLK1 CDK1 ATR CHEK2 HDAC2 PRKDC |
| 0.000343 | 7 | 37 | 5.714976 | Aldosterone-regulated sodium reabsorption | http://www.genome.jp/kegg-bin/show_pathway?hsa04960 | PIK3CB PIK3CA PDPK1 NR3C2 PRKCA PRKCB INSR |
| 0.000387 | 12 | 134 | 3.414141 | Vascular smooth muscle contraction | http://www.genome.jp/kegg-bin/show_pathway?hsa04270 | PRKCH PRKCQ ADRA1A ADORA2A AGTR1 PRKCA KCNMA1 BRAF PTGIR PRKCD PRKCB ADORA2B |
| 0.000401 | 8 | 61 | 4.845878 | Arachidonic acid metabolism | http://www.genome.jp/kegg-bin/show_pathway?hsa00590 | PTGS2 PTGS1 LTA4H CYP2C9 ALOX15 CYP2C19 CYP2B6 ALOX5 |
| 0.000505 | 8 | 91 | 4.694444 | Morphine addiction | http://www.genome.jp/kegg-bin/show_pathway?hsa05032 | OPRM1 PRKCA ADORA1 PRKCB PDE3A DRD1 PDE2A PDE7A |
| 0.000539 | 14 | 148 | 2.953808 | Gastric cancer | http://www.genome.jp/kegg-bin/show_pathway?hsa05226 | PIK3CB GSK3B ABCB1 AKT2 MET TGFBR1 PIK3CA CDK2 ERBB2 AKT1 EGFR BRAF TGFBR2 MTOR |
| 0.000665 | 12 | 142 | 3.219048 | Spinocerebellar ataxia | http://www.genome.jp/kegg-bin/show_pathway?hsa05017 | MAPK9 PIK3CB AKT2 MAPK8 MAPK10 PIK3CA AKT1 PRKCA PRKCB ERN1 MAP3K5 MTOR |
| 0.000696 | 9 | 101 | 4.02381 | Pancreatic secretion | http://www.genome.jp/kegg-bin/show_pathway?hsa04972 | CFTR CD38 CA2 CHRM3 CPB1 PRKCA KCNMA1 CCKAR PRKCB |

| | | | | | | |
|---|---|---|---|---|---|---|
| 0.00076 5 | 8 | 41 | 4.418301 | Bladder cancer | http://www.genome.jp/kegg-bin/show_pathway?hsa05219 | MMP2 MMP9 MDM2 ERBB2 EGFR BRAF MMP1 SRC |
| 0.00090 2 | 5 | 47 | 7.222222 | Carbohydrate digestion and absorption | http://www.genome.jp/kegg-bin/show_pathway?hsa04973 | PIK3CB AKT2 PIK3CA AKT1 PRKCB |
| 0.00090 3 | 13 | 101 | 2.941098 | Chagas disease | http://www.genome.jp/kegg-bin/show_pathway?hsa05142 | NOS2 MAPK9 PIK3CB TNFRSF1A AKT2 TGFBR1 MAPK8 MAPK10 MAPK14 PIK3CA TLR4 AKT1 TGFBR2 |
| 0.00096 3 | 11 | 138 | 3.27866 | Apelin signaling pathway | http://www.genome.jp/kegg-bin/show_pathway?hsa04371 | NOS2 SPHK2 HDAC4 NOS1 AKT2 TGFBR1 AKT1 AGTR1 NOS3 SPHK1 MTOR |
| 0.00096 3 | 9 | 88 | 3.840909 | Gap junction | http://www.genome.jp/kegg-bin/show_pathway?hsa04540 | HTR2A PDGFRB PDGFRA EGFR PRKCA PRKCB GRM5 DRD1 SRC |
| 0.00109 5 | 15 | 192 | 2.632399 | Transcriptional misregulation in cancer | http://www.genome.jp/kegg-bin/show_pathway?hsa05202 | MMP9 FLT1 MET HDAC1 FLT3 PPARG PER2 MDM2 IGF1R MMP3 TGFBR2 PTK2 CSF1R HDAC2 ELANE |
| 0.00116 6 | 7 | 74 | 4.694444 | Metabolism of xenobiotics by cytochrome P450 | http://www.genome.jp/kegg-bin/show_pathway?hsa00980 | CYP3A5 HSD11B1 CYP2C9 CYP1A1 CYP1A2 CYP3A4 CYP2B6 |
| 0.00119 1 | 15 | 157 | 2.608025 | Hepatitis C | http://www.genome.jp/kegg-bin/show_pathway?hsa05160 | BAD PIK3CB TNFRSF1A GSK3B AKT2 TYK2 PIK3CA CDK2 CDK4 AKT1 EGFR BRAF JAK1 STAT3 PPARA |
| 0.00144 3 | 7 | 75 | 4.532567 | PPAR signaling pathway | http://www.genome.jp/kegg-bin/show_pathway?hsa03320 | SCD CPT1A PPARG PDPK1 ILK PPARA MMP1 |
| 0.00171 8 | 18 | 222 | 2.283784 | Human T-cell leukemia virus 1 infection | http://www.genome.jp/kegg-bin/show_pathway?hsa05166 | MAPK9 PIK3CB TNFRSF1A TSPO AKT2 JAK3 TGFBR1 MAPK8 MAPK10 PIK3CA CDK2 CDK4 AKT1 JAK1 TGFBR2 ATR LCK CHEK2 |
| 0.00174 | 6 | 68 | 5.121212 | Retinol metabolism | http://www.genome.jp/kegg-bin/show_pathway?hsa00830 | CYP3A5 CYP2C9 CYP1A1 CYP1A2 CYP3A4 CYP2B6 |
| 0.00185 7 | 13 | 103 | 2.712346 | Toll-like receptor signaling pathway | http://www.genome.jp/kegg-bin/show_pathway?hsa04620 | MAP2K3 MAPK9 PIK3CB AKT2 MAPK8 MAP3K8 MAP2K6 MAPK10 MAPK14 PIK3CA TLR4 AKT1 CTSK |
| 0.00206 1 | 9 | 68 | 3.44898 | Renal cell carcinoma | http://www.genome.jp/kegg-bin/show_pathway?hsa05211 | BAD PIK3CB AKT2 MET PIK3CA EGLN1 AKT1 PAK1 BRAF |
| 0.00218 | 17 | 249 | 2.296563 | Salmonella infection | http://www.genome.jp/kegg-bin/show_pathway?hsa05132 | MAP2K3 MAPK9 PIK3CB TNFRSF1A HSP90AA1 HSP90AB1 RIPK2 AKT2 MAPK8 MAP2K6 MAPK10 MAPK14 PIK3CA TLR4 CASP1 AKT1 PAK1 |
| 0.00239 5 | 5 | 29 | 5.868056 | Linoleic acid metabolism | http://www.genome.jp/kegg-bin/show_pathway?hsa00591 | CYP2C9 CYP1A2 CYP3A4 ALOX15 CYP2C19 |
| 0.00269 | 9 | 73 | 3.313725 | Platinum drug resistance | http://www.genome.jp/kegg-bin/show_pathway?hsa01524 | BAD PIK3CB AKT2 PIK3CA MDM2 PDPK1 ERBB2 AKT1 MAP3K5 |
| 0.00269 | 9 | 129 | 3.313725 | Oocyte meiosis | http://www.genome.jp/kegg-bin/show_pathway?hsa04114 | PGR AURKA MAPK14 CDK2 IGF1R PLK1 AR CDK1 RPS6KA3 |
| 0.00276 4 | 12 | 93 | 2.714859 | IL-17 signaling pathway | http://www.genome.jp/kegg-bin/show_pathway?hsa04657 | MAPK9 PTGS2 HSP90AA1 GSK3B HSP90AB1 MMP9 MAPK8 MAPK10 MAPK14 MMP13 MMP3 MMP1 |
| 0.00402 8 | 8 | 107 | 3.414141 | Glucagon signaling pathway | http://www.genome.jp/kegg-bin/show_pathway?hsa04922 | PKM AKT2 GCK CPT1A LDHB AKT1 PPARA GCGR |
| 0.00512 2 | 6 | 69 | 4.17284 | Drug metabolism | http://www.genome.jp/kegg-bin/show_pathway?hsa00982 | CYP3A5 CYP2C9 CYP1A2 CYP3A4 CYP2C19 CYP2B6 |
| 0.00745 8 | 6 | 86 | 3.885057 | Insulin secretion | http://www.genome.jp/kegg-bin/show_pathway?hsa04911 | GCK CHRM3 PRKCA KCNMA1 CCKAR PRKCB |
| 0.00770 3 | 15 | 162 | 2.150127 | JAK-STAT signaling pathway | http://www.genome.jp/kegg-bin/show_pathway?hsa04630 | PIK3CB JAK2 AKT2 TYK2 JAK3 PDGFRB PIK3CA IL6ST PDGFRA PIM1 AKT1 EGFR JAK1 STAT3 MTOR |
| 0.00803 8 | 7 | 64 | 3.37037 | GnRH secretion | http://www.genome.jp/kegg-bin/show_pathway?hsa04929 | PIK3CB AKT2 PIK3CA ESR2 AKT1 PRKCA PRKCB |
| 0.00834 3 | 5 | 51 | 4.470899 | Ovarian steroidogenesis | http://www.genome.jp/kegg-bin/show_pathway?hsa04913 | PTGS2 IGF1R CYP1A1 INSR ALOX5 |
| 0.00904 8 | 9 | 81 | 2.770492 | B cell receptor signaling pathway | http://www.genome.jp/kegg-bin/show_pathway?hsa04662 | BTK PIK3CB GSK3B AKT2 PIK3CA AKT1 SYK PRKCB LYN |
| 0.00994 6 | 6 | 61 | 3.634409 | Steroid hormone biosynthesis | http://www.genome.jp/kegg-bin/show_pathway?hsa00140 | STS CYP3A5 HSD11B1 CYP1A1 CYP1A2 CYP3A4 |
| 0.00994 6 | 9 | 169 | 2.725806 | Protein processing in endoplasmic reticulum | http://www.genome.jp/kegg-bin/show_pathway?hsa04141 | CAPN1 MAPK9 HSP90AA1 HSP90AB1 MAPK8 MAPK10 CAPN2 ERN1 MAP3K5 |
| 0.00994 6 | 6 | 69 | 3.634409 | Renin secretion | http://www.genome.jp/kegg-bin/show_pathway?hsa04924 | REN AGTR1 KCNMA1 ADORA1 PTGER4 PDE3A |
| 0.01151 1 | 11 | 104 | 2.374202 | NF-kappa B signaling pathway | http://www.genome.jp/kegg-bin/show_pathway?hsa04064 | BTK PRKCQ TNFRSF1A PTGS2 CSNK2A1 TLR4 PARP1 SYK PRKCB LCK LYN |
| 0.01153 7 | 6 | 60 | 3.520833 | Long-term depression | http://www.genome.jp/kegg-bin/show_pathway?hsa04730 | NOS1 IGF1R PRKCA BRAF PRKCB LYN |
| 0.01191 1 | 5 | 85 | 4.082126 | Taste transduction | http://www.genome.jp/kegg-bin/show_pathway?hsa04742 | P2RX3 CHRM3 HTR3A SCN9A P2RY1 |

| | | | | | | |
|---|---|---|---|---|---|---|
| 0.012475 | 14 | 180 | 2.08642 | NOD-like receptor signaling pathway | http://www.genome.jp/kegg-bin/show_pathway?hsa04621 | CASR MAPK9 HSP90AA1 P2RX7 HSP90AB1 RIPK2 TYK2 MAPK8 MAPK10 MAPK14 TLR4 CASP1 JAK1 PRKCD |
| 0.014137 | 9 | 169 | 2.560606 | Tight junction | http://www.genome.jp/kegg-bin/show_pathway?hsa04530 | CFTR MAPK9 PRKCZ MAPK8 MAPK10 CDK4 ERBB2 SRC MAP3K5 |
| 0.014137 | 9 | 114 | 2.560606 | Leukocyte transendothelial migration | http://www.genome.jp/kegg-bin/show_pathway?hsa04670 | PIK3CB MMP2 MMP9 MAPK14 ITK PIK3CA PRKCA PRKCB PTK2 |
| 0.014137 | 9 | 232 | 2.560606 | Thermogenesis | http://www.genome.jp/kegg-bin/show_pathway?hsa04714 | MAP2K3 FGFR1 CPT1A MAPK14 CNR1 PPARG RPS6KA3 MAP3K5 MTOR |
| 0.017075 | 10 | 92 | 2.347222 | Th1 and Th2 cell differentiation | http://www.genome.jp/kegg-bin/show_pathway?hsa04658 | MAPK9 PRKCQ JAK2 TYK2 JAK3 MAPK8 MAPK10 MAPK14 JAK1 LCK |
| 0.017087 | 6 | 97 | 3.219048 | Circadian entrainment | http://www.genome.jp/kegg-bin/show_pathway?hsa04713 | NOS1 PER2 MTNR1B PRKCA PRKCB MTNR1A |
| 0.017087 | 6 | 92 | 3.219048 | Salivary secretion | http://www.genome.jp/kegg-bin/show_pathway?hsa04970 | NOS1 ADRA1A CHRM3 PRKCA KCNMA1 PRKCB |
| 0.017699 | 3 | 22 | 6.259259 | Arginine biosynthesis | http://www.genome.jp/kegg-bin/show_pathway?hsa00220 | NOS2 NOS1 NOS3 |
| 0.021818 | 9 | 92 | 2.380282 | Small cell lung cancer | http://www.genome.jp/kegg-bin/show_pathway?hsa05222 | NOS2 PIK3CB PTGS2 AKT2 PIK3CA CDK2 CDK4 AKT1 PTK2 |
| 0.023152 | 13 | 171 | 1.984643 | Influenza A | http://www.genome.jp/kegg-bin/show_pathway?hsa05164 | PIK3CB TNFRSF1A JAK2 AKT2 TYK2 PIK3CA CDK4 TLR4 CASP1 AKT1 PRKCA JAK1 PRKCB |
| 0.023379 | 12 | 197 | 2.048485 | Pathogenic Escherichia coli infection | http://www.genome.jp/kegg-bin/show_pathway?hsa05130 | FYN MAPK9 TNFRSF1A ABL1 MAPK8 MAPK10 MAPK14 TLR4 CASP1 PAK1 F2R SRC |
| 0.027571 | 13 | 217 | 1.93739 | Regulation of actin cytoskeleton | http://www.genome.jp/kegg-bin/show_pathway?hsa04810 | PIK3CB FGFR1 PDGFRB PIK3CA CHRM3 PDGFRA EGFR PAK1 BRAF PTK2 CHRM2 F2R SRC |
| 0.029623 | 5 | 76 | 3.237548 | Gastric acid secretion | http://www.genome.jp/kegg-bin/show_pathway?hsa04971 | CFTR CA2 CHRM3 PRKCA PRKCB |
| 0.038659 | 6 | 77 | 2.68254 | Bacterial invasion of epithelial cells | http://www.genome.jp/kegg-bin/show_pathway?hsa05100 | PIK3CB MET PIK3CA ILK PTK2 SRC |
| 0.038992 | 8 | 166 | 2.276094 | Wnt signaling pathway | http://www.genome.jp/kegg-bin/show_pathway?hsa04310 | MAPK9 PSEN1 GSK3B CSNK2A1 MAPK8 MAPK10 PRKCA PRKCB |
| 0.039819 | 10 | 252 | 2.041063 | Endocytosis | http://www.genome.jp/kegg-bin/show_pathway?hsa04144 | PRKCZ TGFBR1 PDGFRA MDM2 IGF1R EGFR CXCR1 TGFBR2 CXCR2 SRC |
| 0.054546 | 5 | 69 | 2.761438 | Amphetamine addiction | http://www.genome.jp/kegg-bin/show_pathway?hsa05031 | HDAC1 PRKCA PRKCB DRD1 HDAC2 |
| 0.060364 | 5 | 89 | 2.68254 | Bile secretion | http://www.genome.jp/kegg-bin/show_pathway?hsa04976 | CFTR ABCB1 CA2 ABCG2 CYP3A4 |
| 0.060364 | 8 | 187 | 2.08642 | Alcoholism | http://www.genome.jp/kegg-bin/show_pathway?hsa05034 | HDAC9 HDAC10 HDAC1 ADORA2A BRAF ADORA2B DRD1 HDAC2 |
| 0.060553 | 2 | 17 | 6.259259 | Nitrogen metabolism | http://www.genome.jp/kegg-bin/show_pathway?hsa00910 | CA2 CA1 |
| 0.060553 | 12 | 363 | 1.774278 | Amyotrophic lateral sclerosis | http://www.genome.jp/kegg-bin/show_pathway?hsa05014 | BAD NOS2 MAP2K3 TNFRSF1A NOS1 HDAC6 MAP2K6 MAPK14 CASP1 ERN1 MAP3K5 MTOR |
| 0.065727 | 5 | 67 | 2.608025 | Long-term potentiation | http://www.genome.jp/kegg-bin/show_pathway?hsa04720 | PRKCA BRAF PRKCB GRM5 RPS6KA3 |
| 0.068235 | 3 | 23 | 3.755556 | Renin-angiotensin system | http://www.genome.jp/kegg-bin/show_pathway?hsa04614 | PRCP REN AGTR1 |
| 0.072421 | 7 | 106 | 2.120072 | Parathyroid hormone synthesis, secretion and action | http://www.genome.jp/kegg-bin/show_pathway?hsa04928 | CASR FGFR1 MMP13 EGFR PRKCA BRAF PRKCB |
| 0.073738 | 10 | 306 | 1.823085 | Huntington disease | http://www.genome.jp/kegg-bin/show_pathway?hsa05016 | MAPK9 MAPK8 MAPK10 HDAC1 PPARG GRM5 ERN1 HDAC2 MAP3K5 MTOR |
| 0.07793 | 5 | 72 | 2.47076 | Mitophagy | http://www.genome.jp/kegg-bin/show_pathway?hsa04137 | MAPK9 CSNK2A1 MAPK8 MAPK10 SRC |
| 0.083679 | 1 | 5 | 18.77778 | Caffeine metabolism | http://www.genome.jp/kegg-bin/show_pathway?hsa00232 | CYP1A2 |
| 0.083679 | 11 | 272 | 1.721296 | Prion disease | http://www.genome.jp/kegg-bin/show_pathway?hsa05020 | BAD FYN MAPK9 PIK3CB GSK3B CSNK2A1 MAPK8 MAPK10 MAPK14 PIK3CA PRKCD |
| 0.092655 | 7 | 76 | 1.991582 | Pertussis | http://www.genome.jp/kegg-bin/show_pathway?hsa05133 | NOS2 MAPK9 MAPK8 MAPK10 MAPK14 TLR4 CASP1 |
| 0.092655 | 7 | 102 | 1.991582 | Amoebiasis | http://www.genome.jp/kegg-bin/show_pathway?hsa05146 | NOS2 PIK3CB PIK3CA TLR4 PRKCA PRKCB PTK2 |
| 0.104928 | 9 | 130 | 1.760417 | Natural killer cell mediated cytotoxicity | http://www.genome.jp/kegg-bin/show_pathway?hsa04650 | FYN PIK3CB PIK3CA PAK1 PRKCA BRAF SYK PRKCB LCK |
| 0.117481 | 3 | 53 | 2.964912 | Endocrine and other factor-regulated calcium reabsorption | http://www.genome.jp/kegg-bin/show_pathway?hsa04961 | ESR1 PRKCA PRKCB |

| | | | | | | |
|---|---|---|---|---|---|---|
| 0.117481 | 7 | 76 | 1.877778 | Leishmaniasis | http://www.genome.jp/kegg-bin/show_pathway?hsa05140 | NOS2 PTGS2 JAK2 MAPK14 TLR4 JAK1 PRKCB |
| 0.119546 | 2 | 33 | 4.17284 | Fructose and mannose metabolism | http://www.genome.jp/kegg-bin/show_pathway?hsa00051 | AKR1B1 PFKFB3 |
| 0.119546 | 9 | 266 | 1.707071 | Parkinson disease | http://www.genome.jp/kegg-bin/show_pathway?hsa05012 | MAPK9 MAPK8 MAPK10 ADORA2A ERN1 DRD1 LRRK2 MAP3K5 MAPT |
| 0.119659 | 4 | 97 | 2.422939 | Phosphatidylinositol signaling system | http://www.genome.jp/kegg-bin/show_pathway?hsa04070 | PIK3CB PIK3CA PRKCA PRKCB |
| 0.128312 | 6 | 150 | 1.942529 | Adrenergic signaling in cardiomyocytes | http://www.genome.jp/kegg-bin/show_pathway?hsa04261 | AKT2 MAPK14 ADRA1A AKT1 AGTR1 PRKCA |
| 0.144782 | 3 | 67 | 2.68254 | Glycolysis / Gluconeogenesis | http://www.genome.jp/kegg-bin/show_pathway?hsa00010 | PKM GCK LDHB |
| 0.152988 | 6 | 154 | 1.846995 | Oxytocin signaling pathway | http://www.genome.jp/kegg-bin/show_pathway?hsa04921 | PTGS2 EGFR PRKCA NOS3 PRKCB SRC |
| 0.163828 | 4 | 126 | 2.146032 | Purine metabolism | http://www.genome.jp/kegg-bin/show_pathway?hsa00230 | PDE5A PDE3A PDE2A PDE7A |
| 0.163828 | 2 | 49 | 3.414141 | Sphingolipid metabolism | http://www.genome.jp/kegg-bin/show_pathway?hsa00600 | SPHK2 SPHK1 |
| 0.163828 | 12 | 495 | 1.472767 | Herpes simplex virus 1 infection | http://www.genome.jp/kegg-bin/show_pathway?hsa05168 | BAD PIK3CB TNFRSF1A JAK2 AKT2 TYK2 PIK3CA AKT1 JAK1 SYK SRC MTOR |
| 0.17372 | 3 | 89 | 2.449275 | GABAergic synapse | http://www.genome.jp/kegg-bin/show_pathway?hsa04727 | PRKCA PRKCB SRC |
| 0.187876 | 2 | 47 | 3.12963 | Pyruvate metabolism | http://www.genome.jp/kegg-bin/show_pathway?hsa00620 | PKM LDHB |
| 0.189065 | 3 | 51 | 2.347222 | Arginine and proline metabolism | http://www.genome.jp/kegg-bin/show_pathway?hsa00330 | NOS2 NOS1 NOS3 |

**Table S8. Molecular docking results of cannabinoids based on their docking scores**

| **Cannabinoids** | **PIK3CA (PDB ID: 4L23) (Kcal/mol)** | **Akt1 (AlphaFold-predicted protein) (Kcal/mol)** | **MAPK9 (AlphaFold-predicted protein) (Kcal/mol)** | **PRKCD (AlphaFold-predicted protein) (Kcal/mol)** | **BRAF (PDB ID: 6P3D) (Kcal/mol)** | **IGF1R (PDB ID: 5FXS) (Kcal/mol)** | **NOS3 (PDB ID: 6NH5) (Kcal/mol)** |
|---|---|---|---|---|---|---|---|
| $\Delta^9$-tetrahydrocannabinol | -6.600 | -2.975 | -5.769 | **-5.123** | -4.126 | **-8.569** | -7.966 |
| Cannabinol | -6.797 | -4.304 | -5.742 | -4.754 | -4.259 | -7.663 | **-8.888** |
| Cannabidiol | -5.393 | -4.090 | -4.761 | -3.018 | -4.662 | -5.455 | -6.703 |
| $\Delta^8$-tetrahydrocannabinol | -5.616 | -3.034 | **-6.751** | -3.274 | -5.464 | -4.788 | -6.380 |
| Cannabigerol | **-8.307** | -0.183 | -4.407 | -4.636 | **-9.484** | **-8.190** | **-8.080** |
| Cannabichromene | **-7.314** | **-4.599** | **-7.663** | -4.399 | **-7.522** | -7.230 | **-8.152** |
| $\Delta^9$-tetrahydrocannabivarin | **-7.334** | -3.396 | -5.550 | **-5.121** | -4.205 | **-8.402** | -7.744 |
| Cannabivarin | -4.819 | -4.353 | -5.486 | -3.939 | -4.455 | -8.029 | -8.015 |
| Cannabidivarin | -5.412 | -3.540 | -4.802 | -2.344 | -3.706 | -5.832 | -5.544 |

| | | | | | | | |
|---|---|---|---|---|---|---|---|
| Cannadinodiol | -5.927 | -4.175 | **-7.664** | -3.310 | **-8.076** | -7.700 | -8.068 |
| Cannabielsoin | -4.945 | **-4.466** | -6.453 | -2.998 | -4.916 | -7.160 | -7.534 |
| Cannabicyclol | -5.741 | -4.011 | -6.167 | -3.156 | -5.040 | -6.793 | -7.546 |
| Cannabitriol | -6.423 | **-6.035** | -4.605 | **-7.308** | -4.899 | -7.639 | -7.943 |

**Table S9. Molecular docking results of cannabinoids based on their interactions**

| **Protein name** | **Compound Name** | **H-bond forming residues** | **Hydrophobic interaction forming residues** | **Polar interaction forming residues** |
|---|---|---|---|---|
| PIK3CA (PDB ID: 7JIU) | Cannabigerol | Val 851 | Met 772, Pro 778, Trp 780, Ile 800, Tyr 836, Ile 848, Val 850, Val 851, Met 922, Phe 930, Ile 932 | Ser 774, Asn 853, Ser 854, Hie 855, Phr 856, Gln 859 |
| | Δ9-Tetrahydrocannabivarin | - | Leu 807, Leu 814, Val Tyr 836, Cys 838, Ile 848, 850, Val 851, Met 922, Phe 930, Ile 932, Phe 934 | Ser 854, Phr 856, Gln 859 |
| | Cannabichromene | Tyr 836 | Met 772, Pro 778, Trp 780, Ile 800, Tyr 836, Ile 848, Val 851, val 850, Met 922, Phe 930, Ile 932 | Ser 774, Ser 854, Thr 856, Ser 919 |
| Akt1 | Cannabitriol | Lys 276, Asn 279, Asp 292 | Val 164, Ala177, Leu 156, Met 227, Ala 230, Tyr 229, Met 281, Phe 438, Phe 442 | Thr 211, Asn 279 |

| | | | | |
|---|---|---|---|---|
| | Cannabichromene | Asn 279 | Leu 156, Phe 161, Val 164, Ala 177, Leu 181, Met 227, Tyr 229, Ala 230, Met 281, Phe 438 | Thr 160, Thr 211, Asn 279, Phr 291 |
| | Cannabielsoin | Lys 158, Glu 278 | Leu 156, Val 164, Ala 177, Met 227, Tyr 229, Ala 230, Met 281, Phe 442, Phe 438 | Asn 231, Asn 279, Thr 291 |
| MAPK9 | Cannabinodiol | Ile 32, Ser 155 | Ile 32, Ile 39, Val 40, Ala 53, Ile 86, Met 109, Leu 110, Met 111, Ala 113, Val 158, Leu 168 | Ser 34, Asn 114, Gln 117, Ser 155, Asn 156 |
| | Cannabichromene | Glu 109, Met 111 | Ile 32, Val 40, Ala 53, Ile 86, Leu 77, Leu 106, Met 108, Leu 110, Met 111, Ala 113, Val 158, Leu 168, Phe 170 | Ser 34, Asn 114, Ser 155 |
| | $\Delta^8$-Tetrahydrocannabinol | Lys 55 | Ile 32, Ala 36, Ile 39, Val 40, Ala 53, Ile 86, Met 108, Leu 110, Met 111, Val 158, Leu 168 | Ser 34, Gln 37, Asn 114, Ser 155, Asn 156 |
| PRKCD | Cannabitriol | Val 174, Leu 279, Gln 284 | Val 174, Cys 175, Cys 264, Met 266, Leu 279, Cys 280, Ile 282, Leu 286 | Ser 173, Hie 231, Asn 283, Gln 284 |
| | $\Delta^9$-tetrahydrocannabinol | Val 174, Gln 284 | Val 174, Cys 175, Cys 264, Met 266, Leu 279, | Ser 173, Hie 231, Asn 283, Gln 284 |

| | | | | |
|---|---|---|---|---|
| | | | Cys 280, Ile 282, Leu 286, | |
| | Δ⁹-Tetrahydrocannabivarin | Val 174, Gln 284 | Val 174, Cys 175, Cys 264, Met 266, Leu 279, Cys 280, ile 282, Leu 286 | Ser 173, Hie 231, Asn 283, Gln 284 |
| BRAF | Cannabigerol | Cys 532 | Ile 463, Val 471, Ala 481, Val 482, Leu 514, Trp 531, Cys 532, Phe 583, Phe 595, Leu 597, Ala 598 | Thr 529, Gln 530, Ser 535, Ser 536, His 539, Asn 580, Thr 599 |
| | Cannabinodiol | Cys 532 | Ile 463, Val 471, Ala 481, Val 482, Leu 514, Trp 531, Cys 532, Phe 583, Phe 595, Leu 597 | Thr 529, Gln 530, Ser 536, Asn 580 |
| | Cannabichromene | - | Ile 463, Val 471, Ala 481, Val 482, Leu 505, Ile 513, Leu 514, Ile 527, Val 528, Trp 531, Cys 532, Leu 567, Phe 583, Ile 592, Phe 595, Leu 597 | Thr 508, Thr 529, Gln 530, Hie 574 |
| IGF1R | Δ⁹-tetrahydrocannabinol | Met 1082 | Leu 1005, Phe 1010, val 1013, Ala 1031, Met 1142, Met 1156, Ile 1160, Leu 1081, Met 1082 | Gln 1007, Ser 1009, Thr 1083 |

| | | | | |
|---|---|---|---|---|
| | $\Delta^9$-Tetrahydrocannabivarin | Met 1082 | Leu 1005, Phe 1010, Val 1013, Ala 1031, Val 1063, Met 1079, Leu 1081, Met 1082, Met 1142, Met 1156, Ile 1160 | Thr 1083, Thr 1157 |
| | Cannabigerol | Met 1082 | Leu 1005, Phe 1010, Val 1013, Ala 1031, Met 1079, Leu 1081, Met 1082, Met 1142, Met 1156, Ile 1160 | Gln 1007, Thr 1083 |
| NOS3 | Cannabinol | - | Al 174, Ala 177, Trp 178, Ala 181, Pro 182, Cys 184, Leu 193, Ala 227, Ile 228, Pro 334, Val 336, Met 339, Phe 353, Trp 356, Trp 447, Phe 473, Tyr 475 | Ser 226, ser 254 |
| | Cannabichromene | Ser 354 | Ala 177, Trp 178, Ala 181, Cys 184, Leu 193, Ala 227, Pro 334, Val 336, , Met 339, Phe 353, Trp 356, Tyr 357, Met 358, Phe 473 | Gln 189, Ser 226, Ser 354 |
| | Cannabigerol | Cys 184, Pro 334 | Ala 174, Trp 178, Ala 181, Cys 184, Leu 193, Ala 227, Ile 228, Pro | Ser 226, Ser 354 |

| | | | | |
|---|---|---|---|---|
| | | | 334, Ala 335, Val 336, Met 339, Phe 353, Trp 447, Phe 473 | |

**Table S10. Results of Induced Fit docking**

| Protein | Cannabinoids | Glidescore) (Kcal/mol) | IFDScore |
|---|---|---|---|
| PIK3CA | Cannabigerol | -7.702 | -2125.814 |
| Akt1 | Cannabitriol | -7.109 | -1019.941 |
| MAPK9 | Cannabinodiol | -8.825 | -865.667 |
| PRKCD | Cannabitriol | -7.728 | -1400.602 |
| BRAF | Cannabigerol | -9.950 | -595.181 |
| IGF1R | $\Delta^9$-tetrahydrocannabinol | -10.544 | -451.272 |
| NOS3 | Cannabinol | -10.054 | -842.164 |

**Table S11. Binding free energies results of cannabinoids the based on MM-GBSA calculations**

| Protein | Cannabinoids | MM-GBSA $\Delta G_{bind}$ |
|---|---|---|
| PIK3CA | Cannabigerol | -2.445 |
| | $\Delta^9$-Tetrahydrocannabivarin | -9.269 |
| | Cannabichromene | -37.318 |
| Akt1 | Cannabitriol | -33.851 |
| | Cannabichromene | -24.115 |
| | Cannabielsoin | -33.147 |
| MAPK9 | Cannabinodiol | -51.893 |
| | Cannabichromene | -38.202 |
| | $\Delta^8$-Tetrahydrocannabinol | -38.375 |

| | | |
|---|---|---|
| PRKCD | Cannabitriol | -44.818 |
| | $\Delta^9$-tetrahydrocannabinol | -43.322 |
| | $\Delta^9$-Tetrahydrocannabivarin | -43.582 |
| BRAF | Cannabigerol | -49.429 |
| | Cannabinodiol | -25.703 |
| | Cannabichromene | -38.882 |
| IGF1R | $\Delta^9$-tetrahydrocannabinol | -36.616 |
| | $\Delta^9$-Tetrahydrocannabivarin | -42.273 |
| | Cannabigerol | -35.050 |
| NOS3 | Cannabinol | -57.754 |
| | Cannabichromene | -44.761 |
| | Cannabigerol | -54.273 |

**Table S12. Physico-chemical properties and ADME/T profile of cannabinoids**

| Phytocompound | mol_MW | SASA | FOSA | FISA | PISA | volume | donorHB | glob | QPpolrz | QPlogPC16 | QPlogPoct | QPlogPo/w | CIQPlogS | QPPCaco | QPlogBB | QPPMDCK | QPlogKp | QPlogKhsa | PercentHumanOralAbsorption | PSA | Jm | CYP1A2inhibitor | CYP2C19inhibitor | CYP2C9inhibitor | CYP2D6inhibitor | CYP3A4inhibitor |
|---|---|---|---|---|---|---|---|---|---|---|---|---|---|---|---|---|---|---|---|---|---|---|---|---|---|---|

| | | | | | | | | | | | | | | | | | | | | | | | | | | |
|---|---|---|---|---|---|---|---|---|---|---|---|---|---|---|---|---|---|---|---|---|---|---|---|---|---|---|
| Cannabidiol | 314.467 | 649.009 | 495.853 | 64.232 | 88.923 | 1159.843 | 2 | 0.822585 | 36.617 | 10.522 | 14.971 | 5.336 | -5.269 | 2436.615 | -0.491 | 1295.451 | -1.718 | 1.059 | 100 | 39.085 | 0.005 | No | Yes | Yes | Yes | Yes |
| $\Delta^8$-Tetrahydrocannabinol | 314.467 | 630.761 | 513.941 | 30.372 | 86.448 | 1131.795 | 1 | 0.832681 | 36.756 | 9.778 | 13.516 | 5.645 | -5.482 | 5103.682 | -0.03 | 2880.636 | -1.295 | 1.208 | 100 | 25.657 | 0.006 | No | Yes | Yes | Yes | No |
| Cannabigerol | 316.483 | 688.202 | 541.523 | 72.256 | 74.423 | 1225.024 | 2 | 0.804537 | 36.519 | 11.109 | 14.693 | 5.681 | -5.21 | 2045.034 | -0.844 | 1071.965 | -1.533 | 1.07 | 100 | 41.961 | 0.006 | Yes | Yes | No | Yes | No |
| Cannabichromene | 314.467 | 677.722 | 515.294 | 46.749 | 115.679 | 1202.37 | 1 | 0.806874 | 37.933 | 10.774 | 13.856 | 6.019 | -5.501 | 3569.289 | -0.397 | 1957.156 | -1.205 | 1.279 | 100 | 28.131 | 0.003 | No | No | Yes | Yes | Yes |
| $\Delta^9$-Tetrahydrocannabivarin | 286.413 | 585.92 | 462.868 | 37.809 | 85.242 | 1030.863 | 1 | 0.842288 | 33.989 | 8.875 | 12.727 | 4.942 | -4.897 | 4338.636 | 0.027 | 2416.87 | -1.628 | 1.008 | 100 | 26.006 | 0.008 | No | Yes | Yes | Yes | No |
| Cannabivarin | 282.382 | 576.607 | 389.412 | 38.481 | 148.715 | 1001.259 | 1 | 0.839426 | 33.41 | 9.033 | 12.552 | 4.869 | -5.073 | 4275.455 | 0.019 | 2378.851 | -1.417 | 0.924 | 100 | 26.446 | 0.02 | Yes | Yes | No | Yes | No |
| Cannabidivarin | 286.413 | 569.112 | 410.05 | 64.194 | 94.878 | 1030.272 | 2 | 0.866818 | 32.773 | 9.422 | 14.043 | 4.518 | -4.689 | 2438.625 | -0.307 | 1296.606 | -1.888 | 0.787 | 100 | 39.048 | 0.04 | No | Yes | Yes | No | Yes |
| Cannabinodiol | 310.435 | 646.4 | 412.0 | 68.146 | 166.2 | 1142.6 | 2 | 0.8176 | 36.026 | 10.931 | 14.796 | 5.288 | -5.444 | 2237.03 | -0.598 | 1181.147 | -1.422 | 0.967 | 100 | 41.108 | 0.015 | Yes | Yes | No | Yes | No |

| | | | | | | | | | | | | | | | | | | | | | | | | | | |
|---|---|---|---|---|---|---|---|---|---|---|---|---|---|---|---|---|---|---|---|---|---|---|---|---|---|---|
| | | 74 | 52 | | 76 | 43 | | 26 | | | | | | | | | | | | | | | | | | |
| Cannabielsoin | 330.466 | 627.231 | 470.639 | 77.497 | 79.095 | 1137.631 | 2 | 0.840243 | 35.635 | 10.347 | 15.362 | 4.803 | -5.262 | 1823.886 | -0.59 | 947.235 | -1.997 | 0.872 | 100 | 49.051 | 0.011 | No | No | Yes | Yes | No |
| Cannabicyclol | 314.467 | 620.257 | 525.795 | 31.46 | 63.003 | 1114.781 | 1 | 0.838274 | 35.851 | 9.465 | 13.222 | 5.501 | -5.112 | 4983.862 | -0.036 | 2807.607 | -1.397 | 1.161 | 100 | 26.972 | 0.008 | No | Yes | Yes | Yes | No |
| Cannabitriol | 346.466 | 657.725 | 491.709 | 100.521 | 65.495 | 1178.113 | 3 | 0.820185 | 37.125 | 11.013 | 17.984 | 4 | -4.98 | 1103.217 | -0.855 | 550.125 | -2.469 | 0.581 | 100 | 64.595 | 0.006 | No | No | No | Yes | No |
| Cannabinol | 310.435 | 643.191 | 455.883 | 38.49 | 148.819 | 1122.929 | 1 | 0.812319 | 36.996 | 10.182 | 13.554 | 5.602 | -5.657 | 4274.672 | -0.127 | 2378.38 | -1.225 | 1.181 | 100 | 26.451 | 0.004 | Yes | Yes | No | Yes | No |
| $\Delta^9$-tetrahydrocannabinol | 314.467 | 652.152 | 529.203 | 37.781 | 85.168 | 1152.505 | 1 | 0.815163 | 37.572 | 10.013 | 13.732 | 5.724 | -5.482 | 4341.323 | -0.117 | 2418.488 | -1.436 | 1.265 | 100 | 25.996 | 0.002 | No | Yes | Yes | Yes | No |

mol_MW - Molecular Weight (130/725), SASA - Total SASA (300/1000), FOSA - Hydrophobic SASA (0/750), FISA - Hydrophilic SASA (7/330), PISA – Carbon Pi SASA (0/450), donorHB – No. of donor hydrogen bonds (0/6), glob – Globularity (0.75/0.95), QP polrz – Predicted polarizability (13/70), QP log PC 16 – Predicted hexadecane/gas partition coefficient (4/18), QP logP oct – Predicted octanol/gas partition coefficient (8/35), QP logP o/w – Predicted octanol/water partition coefficient (-2/6.5), CI QP log S – Conformation independent log S for aqueous solubility (-6.5/0.5), QP P Caco – Apparent Caco-2 Permeability (<25 poor, >500 great), QP log BB – Predicted brain/blood barrier permeability (-3.0/1.2), QP P MDCK – Apparent MDCK (Madin-Darby canine kidney) Permeability (<25 poor, >500 great), QP log Kp – Predicted skin permeability, QP log K hsa – Predicted Serum Protein Binding (-1.5/1.5), PSA – vdW Polar SA (7/200), Jm – Predicted maximum transdermal transport rate
